# Massively Parallel Sequencing of Human Urinary Exosome/Microvesicle RNA Reveals a Predominance of Non-Coding RNA

**DOI:** 10.1371/journal.pone.0096094

**Published:** 2014-05-09

**Authors:** Kevin C. Miranda, Daniel T. Bond, Joshua Z. Levin, Xian Adiconis, Andrey Sivachenko, Carsten Russ, Dennis Brown, Chad Nusbaum, Leileata M. Russo

**Affiliations:** 1 Program in Membrane Biology, Division of Nephrology & Center for Systems Biology, Massachusetts General Hospital and Harvard Medical School, Boston, Massachusetts, United States of America; 2 Broad Institute of Massachusetts Institute of Technology and Harvard University, Cambridge, Massachusetts, United States of America; Emory University, United States of America

## Abstract

Intact RNA from exosomes/microvesicles (collectively referred to as microvesicles) has sparked much interest as potential biomarkers for the non-invasive analysis of disease. Here we use the Illumina Genome Analyzer to determine the comprehensive array of nucleic acid reads present in urinary microvesicles. Extraneous nucleic acids were digested using RNase and DNase treatment and the microvesicle inner nucleic acid cargo was analyzed with and without DNase digestion to examine both DNA and RNA sequences contained in microvesicles. Results revealed that a substantial proportion (∼87%) of reads aligned to ribosomal RNA. Of the non-ribosomal RNA sequences, ∼60% aligned to non-coding RNA and repeat sequences including LINE, SINE, satellite repeats, and RNA repeats (tRNA, snRNA, scRNA and srpRNA). The remaining ∼40% of non-ribosomal RNA reads aligned to protein coding genes and splice sites encompassing approximately 13,500 of the known 21,892 protein coding genes of the human genome. Analysis of protein coding genes specific to the renal and genitourinary tract revealed that complete segments of the renal nephron and collecting duct as well as genes indicative of the bladder and prostate could be identified. This study reveals that the entire genitourinary system may be mapped using microvesicle transcript analysis and that the majority of non-ribosomal RNA sequences contained in microvesicles is potentially functional non-coding RNA, which play an emerging role in cell regulation.

## Introduction

Exosomes (derived from multi-vesicular bodies) and shedding microvesicles (derived from plasma membrane budding) are unique forms of vesicles released by all cells into various biofluids [1], [2]. Previous microvesicle research has focused on proteomics via mass spectrometry and specific protein analysis [3]–[5]. However, it has now been shown that microvesicles contain nucleic acids including mRNA, miRNA, rRNA and DNA [6]–[9], encapsulated from the parent cell cytoplasm during the biogenesis of the microvesicle. Analysis of these microvesicles may allow for the non-invasive examination of the transcriptional profile of the parent cell. Studies have confirmed that microvesicle RNA analysis has the potential to be used to diagnose various cancers including glioblastoma multiforme [7], potentially circumventing the need for biopsy, enabling longitudinal monitoring previously not possible due to the need for repeat biopsy.

Microvesicles are extremely stable and highly protective of their nucleic acid cargo. Our previous studies have demonstrated that both urinary and serum microvesicles carry high quality RNA including characteristic 18S and 28S rRNA profiles similar to that observed in well handled tissue [8]. Such high integrity RNA could be obtained in urine that had been stored for over 5 months at 4°C and −80°C *(Russo, unpublished observation)* without prior treatment with stabilization buffers indicating the exceptional stability of microvesicles that could not be achieved from urinary cells stored over similar periods of time [8]. The finding that microvesicles contain high integrity RNA increases their potential use as a source of reliable RNA-based biomarkers beyond that offered by small RNA such as microRNA (miRNA) or degraded RNA.

We have previously shown that isolation of microvesicles using established differential centrifugation techniques can lead to the co-isolation of genomic DNA (gDNA) [8]. This DNA is believed to be outside of the microvesicle as DNase digestion of the microvesicle pellet results in the removal of this material without disruption to the RNA cargo which is believed to be protected within the microvesicles [8]. In order to ensure that non-coding RNA species identified using next-generation sequencing techniques are truly RNA derived, DNA digestion of the microvesicle pellet was carried out prior to RNA isolation. Further DNase digestion of the inner microvesicle cargo may further delineate DNA derived non-coding material packaged within microvesicles.

Various studies have been carried out to examine the array for genes present in microvesicles using sequencing [10]–[12]. Many of these studies have been focused on small RNA species such as non-coding microRNA (miRNA). However, there have been no studies that have optimally extracted large RNA species to comprehensively address the array of RNAs in microvesicles. Here we assess the array of nucleic acids contained within urinary microvesicles using massively parallel sequencing.

## Materials and Methods

### Exosome and RNA isolation

For massively parallel sequencing 3300 ml of urine from a healthy male subject was obtained under the approved IRB guidelines of the Massachusetts General Hospital where written informed consent was waived by the IRB committee. Briefly, the urine was initially centrifuged at 300×g for 10 minutes to pellet whole cell contaminants. The supernatant was carefully removed and centrifuged at 17,000×g for 20 minutes to pull down cell fragments and apoptotic cells. The supernatant was then removed and filtered through a 0.8 µm filter to separate residual debris from the microvesicle containing supernatant. Finally, the filtrate underwent ultracentrifugation at 118,000×g for 70 minutes, the supernatant removed and the microvesicle pellet washed in PBS. The microvesicle pellet was treated with RNase and DNase to remove extraneous nucleic acids as previously described [8]. Following RNase and DNase treatment the pellet was washed and RNA extracted using the RNeasy Micro Kit (Qiagen, CA) according to the manufacturer's instructions and eluted in 16 µl of nuclease free water. Isolated RNA was analyzed on a RNA Pico 6000 chip (Agilent, CA) using an Agilent Bioanalyzer to check for integrity. The total amount of RNA obtained was ∼154 ng determined using the Quant-iT kit (Invitrogen, CA) according to the manufacturer's instructions. Approximately, 60 ng of RNA was used for downstream deep sequencing. For analysis of whether non-coding material was RNA or DNA derived, we performed a pilot DNase treatment experiment on a 20 ng aliquot of the extracted RNA, following the rigorous protocol of the TURBO DNA-free kit (Applied Biosystems/Ambion, TX). There was no DNA detected by qPCR after the treatment (data not shown). Also the Bioanalyzer Pico 6000 assay (Agilent, CA) indicated the RNA was intact after DNase treatment. The same DNase treatment method was then applied to ∼70 ng of the extracted RNA and a cDNA library was constructed.

### Massively parallel sequencing

DNase-treated RNA or non-DNase treated RNA was ethanol precipitated and re-suspended in 8 µl H_2_O. We fragmented the RNA by heating at 98°C for 30 minutes in a total volume of 10 µl with 0.2 mM sodium citrate, pH 6.4 (Applied Biosystems/Ambion, TX). The fragmented RNA was then mixed with 3 µg random hexamers, followed by incubation at 70°C for 10 min, and chilling on ice. We synthesized the cDNA and used it to construct an Illumina paired-end library as previously described [13] with the following modifications. First, no cDNA shearing was needed since the input RNA was already fragmented. Second, paired-end adaptors, instead of single read adaptors, were ligated to the cDNA fragments. Third, final enrichment PCR reaction contained 2M Betaine (Sigma, MO) and enrichment primers for paired-end sequencing were used. Fourth, 11 PCR cycles were performed. Fifth, the final PCR product was size selected on a 10% Criterion TBE gel (Bio-Rad, CA) for an insert size range of 150 to 350 bp. Each of these resulting libraries was loaded in a single lane of a flow cell to generate 76 base, paired end reads on an Illumina GAII sequencer (Illumina, CA).

### Data Analysis

Data analysis utilized an Amazon Elastic Cloud Compute (EC2) extra large instance running CentOS. Version 1.7 of Casavas (Illumina, CA) eland_rna workflow was used for aligning reads to UCSC human genome build 19 as supplied in the Illumina iGenomes dataset. The “abundant sequences” files supplied by iGenomes was augmented with the 531 ncRNA sequences identified in the “curated from literature” set of rnaDB 2.0 [14]. During alignment the flag “KEEP_INTERMEDIARY” was set to true to allow interrogation of the *extended_contam.txt, *extended_splice and *eland_extended.txt files. These 3 files were combined (extended_combined.txt) to provide the pertinent information that allowed for the identification of repeats.

For repeat analysis the extended_combined.txt file was interrogated for reads that: 1) passed filter; 2) aligned to genome; 3) did not align to any sequences in the contamination dataset; and 4) were assigned an RM tag by eland. From this set of reads the genomic location of the best alignment was selected for further analysis. This co-ordinate was then evaluated to see if it overlapped with any known repeat regions as supplied in the UCSC build 19 rmsk table [15]. If so, then the read was deemed to be transcribed from a repeat region. Casavas default readBases method was used to count the number of reads aligning to genes as defined in UCSC build 19 refFlat table [15]. The datasets have been deposited in the NIH Short Read Archive (Study accession # SRP039357, +DNase sample accession #SRS565563 and -DNase sample accession # SRS565564).

## Results and Discussion

**Figure 1:**
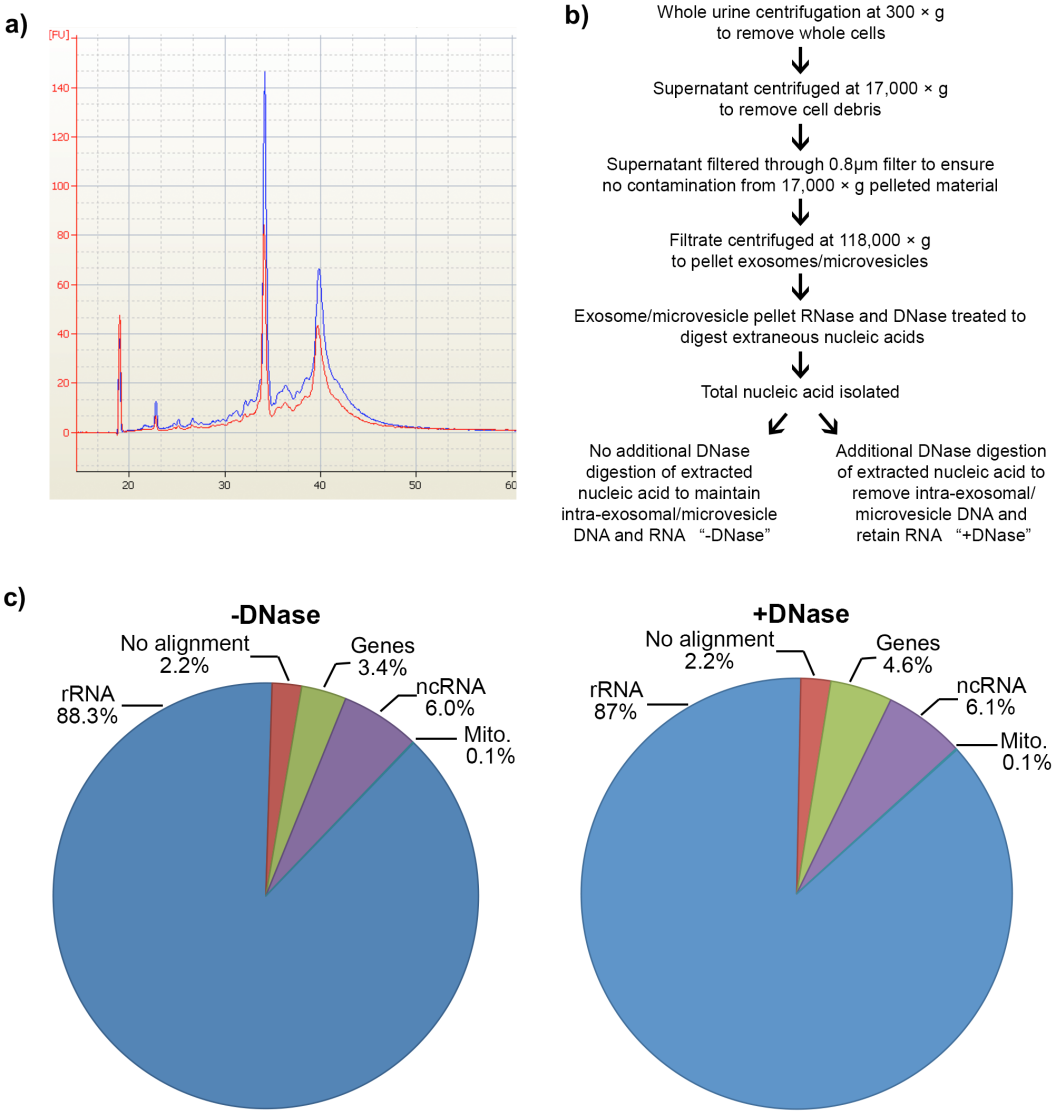
Urinary microvesicle RNA integrity and alignment to the genome. a) RNA isolated from urinary microvesicles was shown to be of high integrity with prominent 18S and 28S rRNA peaks when analyzed using the Agilent Bioanalyzer. Red trace 1.7 ng RNA with DNase, Blue trace 2.2 ng RNA without DNase. b) Flow chart outlining sample processing. An initial RNase and DNase digestion was carried out to remove extraneous nucleic acids co-isolating with the microvesicle pellet. To determine the proportion of potential DNA inside the microvesicles the extracted RNA was divided into two groups; No DNase digestion (-DNase), which yields RNA+DNA and DNase digested (+DNase) which yields RNA. c) Both the -DNase and the +DNase samples showed a similar trend in read distribution with ∼88% of reads mapping to rRNA, ∼4% mapping to genes and ∼6% mapping to ncRNA. A smaller proportion of reads (<0.1%) mapped to mitochondrial genome. Approximately 2% of reads failed to hit the human genome. (mito – mitochondrial).

Urinary microvesicles were isolated from a healthy male subject via differential centrifugation. Due to the potential for co-isolation of extraneous DNA during microvesicle isolation [8], both RNase and DNase digestion of the microvesicle pellet was carried out to remove any extraneous nucleic acids not contained within microvesicles [8]. The extracted nucleic acid material was assessed for size distribution and quality using the Agilent Bioanalyzer (see Fig. 1a), which demonstrated that it was of high integrity with prominent 18S and 28S rRNA peaks. In order to determine both the RNA and DNA distribution *within* microvesicles the isolated nucleic acids were divided and treated in parallel with and without DNase digestion (see Fig. 1b flow-chart for sample preparation). From the two RNA samples (+DNase and -DNase), we constructed cDNA libraries, which were sequenced using an Illumina Genome Analyzer (see Methods).

The Illumina 76 paired-end sequencing run yielded 17,383,693 passing filter (PF) reads for the +DNase RNA sample and 18,875,496 PF reads for the -DNase RNA sample. The PF reads were aligned back to the human genome using Casava 1.7's eland_rna workflow (Illumina, San Diego) which only supports analysis of single reads.

Reads could be divided into 5 major bins shown in Fig. 1c. The majority of the sequence reads aligned to ribosomal RNA, corroborating the data presented in Fig. 1a and demonstrating that the microvesicle nucleic acid payload is dominated by ribosomal RNA consistent with a typical eukaryotic cell RNA profile. Approximately 2% of PF reads either failed the aligner QC step; aligned to contaminants or failed to align to the human genome (Fig. 1c). This failure to align to the human genome suggests the possibility of nucleic acids from a different origin including potentially viral, bacterial or other species as previously reported [16]. A small percentage (<0.01%) aligned to mitochondrial DNA species (Fig. 1c). The other two categories of aligned reads shown in Fig. 1c were coding RNA (aligning to protein coding genes) and non-coding RNA both of which are discussed in detail below.

The ncRNA found in microvesicles (Fig. 1c) could be divided into two groups, known ncRNA identified from a literature search as defined in RNAdb [14] or known repeats as listed in the repeat masked table produced by UCSC [15]. A total of 690 different repeat sequences encompassing all known repeat classes including Short interspersed nuclear elements (SINE) (which include ALUs), Long interspersed nuclear elements (LINE), Long terminal repeat elements (LTR) (which include retrotransposons), DNA repeat elements, Simple repeats (micro-satellites), Low complexity repeats, Satellite repeats, RNA repeats (including RNA, transfer RNA (tRNA), rRNA, small nuclear RNA (snRNA), small cytoplasmic RNA (scRNA), signal recognition particle RNA (srpRNA)), Other repeats (including Rolling Circle (RC)) were identified in microvesicles representing ∼50% of all known repeats. A breakdown of the representation of different classes is summarized in Table 1 while the exact repeats are listed in Table S1 and 2. A total 201 of the 531 known human ncRNAs identified from literature were identified in microvesicles. The top 10 species that received the most aligned reads are shown in Table 2 and 3 while the full list are listed in Table S3 and S4. It should be noted, the number and type of ncRNA identified in both the +DNase and the -DNase prepared samples were qualitatively similar suggesting these repeat species are transcribed and exist at the RNA level.

**Table 1:** Alignment of microvesicle non-coding RNA to repeat regions.

		+DNase treated		-DNase treated	
Repeat Class	# known	Hits	% hits	Hits	% hits
SINE	49	36	73%	40	82%
LINE	146	102	70%	114	78%
LTR	504	200	40%	264	52%
DNA	204	81	39%	104	51%
Simple_repeat	311	84	27%	105	34%
Low_complexity	10	10	10%	10	10%
Satellite	24	8	33%	14	58%
RNA	84	16	19%	27	32%
RC	10	6	60%	8	80%
Unknown	29	2	7%	4	14%

Repeat class was defined by the UCSC repeatMasker dataset [12] and the number of known repeats listed under ‘# known’. All repeat classes were found in microvesicles and the number of hits and percentage hits are shown for each sample, with DNase treatment (+DNase) (RNA sample) and without DNase treatment (-DNase) (RNA+DNA sample). SINE-Short interspersed nuclear elements (which include ALUs), LINE-Long interspersed nuclear elements, LTR-Long terminal repeat elements (which include retrotransposons), DNA repeat elements, Simple repeats (micro-satellites), Low complexity repeats, Satellite repeats, RNA repeats (which includes RNA, rRNA – ribosomal RNA, scRNA – small cytoplasmic RNA, snRNA – small nuclear RNA, srpRNA – signal recognition particle RNA, tRNA – transfer RNA), Other repeats (including Rolling Circle (RC)) were detected within the non-ORF reads of microvesicles and presented as hits and % hits detected.

**Table 2:** Top 10 expressed ncRNA in microvesicles (+DNase).

No. Reads aligning	RNAdb ID	RNAdb definition	Accession ID
855908	LIT1626	Homo sapiens non-coding chimeric transcript hc9(5)h-2-1/4 (UM 9(5))	AY072609
69064	LIT1868	Human 7S L gene, complete	M20910
2722	LIT3330	Homo sapiens ret finger protein-like 3 antisense (RFPL3S) on chromosome 22.	NR_001450
2505	LIT3569	Homo sapiens mRNA sequence	AY927568
2484	LIT3497	Homo sapiens mRNA sequence	AY927481
1668	LIT1867	Homo sapiens 7S RNA	V00477
900	LIT2062	Homo sapiens ribosomal protein, large, P0 pseudogene 2 (RPLP0P2), misc RNA	XR_000076
621	LIT1834	Homo sapiens non-small cell lung carcinoma noncoding RNA, partial sequence	AY166681
520	LIT2056	Homo sapiens EMX2OS mRNA, complete sequence.	AY117413
505	LIT2059	Homo sapiens general transcription factor II, i, pseudogene 1 (GTF2IP1), misc RNA	XR_000139

The most abundantly expressed ncRNA as defined by the RNAdb [14] are listed by number of reads present in the microvesicle population, the RNAdb ID, ncRNA name and accession ID number as identified in the DNase treated sample.

**Table 3:** Top 10 expressed ncRNA in microvesicles (-DNase).

No. Reads aligning	RNAdb ID	RNAdb definition	Accession ID
914237	LIT1626	Homo sapiens non-coding chimeric transcript hc9(5)h-2-1/4 (UM 9(5))	AY072609
52467	LIT1868	Human 7S L gene, complete	M20910
2972	LIT3330	Homo sapiens ret finger protein-like 3 antisense (RFPL3S) on chromosome 22.	NR_001450
2073	LIT3569	Homo sapiens mRNA sequence	AY927568
1620	LIT3497	Homo sapiens mRNA sequence	AY927481
1608	LIT2062	Homo sapiens ribosomal protein, large, P0 pseudogene 2 (RPLP0P2), misc RNA	XR_000076
1393	LIT1867	Homo sapiens 7S RNA	V00477
1033	LIT1834	Homo sapiens non-small cell lung carcinoma noncoding RNA, partial sequence	AY166681
890	LIT2059	Homo sapiens general transcription factor II, i, pseudogene 1 (GTF2IP1), misc RNA	XR_000139
843	LIT2056	Homo sapiens EMX2OS mRNA, complete sequence.	AY117413

The most abundantly expressed ncRNA as defined by the RNAdb [14] are listed by number of reads present in the microvesicle population, the RNAdb ID, ncRNA name and accession ID number as identified in the sample without DNase treatment.

**Figure 2:**
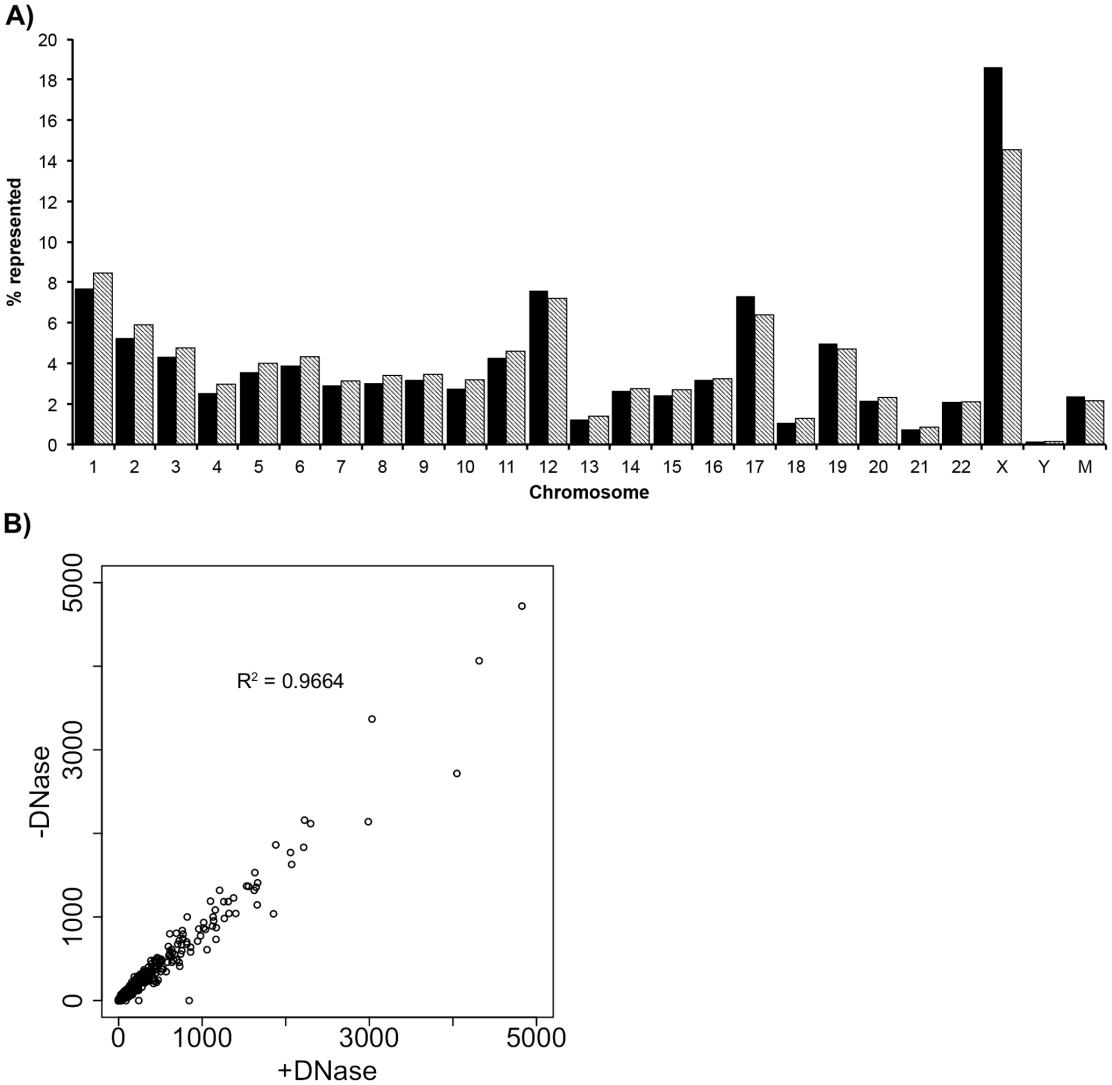
Chromosomal alignment of genes. a) Reads aligning to coding genes were mapped back to the human chromosomes and represented as the % reads aligning to each chromosome. All chromosomes of the human genome are represented within the urinary microvesicles including the mitochondrial chromosome (M). Solid bars – +DNase sample, lined bars – -DNase sample. b) Correlation of reads aligning to coding genes in the +DNase and -DNase samples suggests little if any DNA was present (R^2^ = 0.9664).

The final alignment grouping was protein coding genes and splice sites (Fig. 1c). The distribution of protein coding reads across the chromosomes is shown in Fig. 2a and demonstrates that all chromosomes of the human genome were represented in the urinary microvesicle population. The seemingly low expression coming from the Y chromosome is believed to be an artifact of analysis as both the X and Y chromosomes share common genes. The data was first mapped to the X chromosome so all common genes were attributed to the X chromosome potentially inflating the number of hits exclusively attributable to the X chromosome. Analysis of the correlation between reads in the +DNase versus -DNase sample revealed that the two samples were very similar (R^2^ = 0.9664)(Fig. 2b) and suggested that sequences of DNA origin were not prevalent. Interestingly, the distribution of reads run on two separate sequencing lanes is very similar with little variability suggesting that the exoRNA could be reproducibly sequenced.

**Figure 3:**
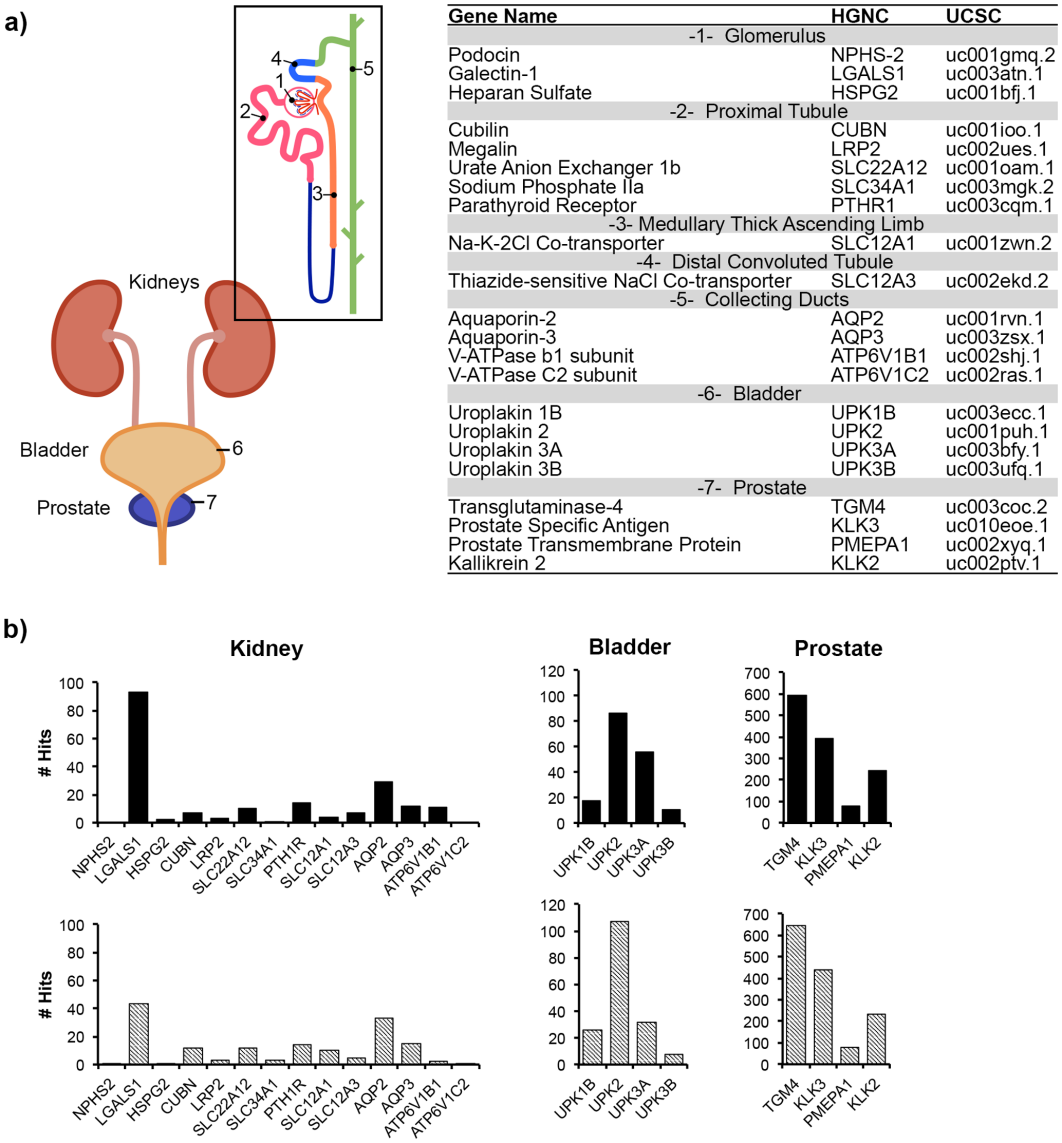
Mapping of coding genes to the genitourinary epithelium. a) Cartoon of the genitourinary system, highlighting specialized regions including the glomerulus (1), proximal tubules (2), medullary thick ascending limb (3), distal convoluted tubule (4), collecting duct (5), bladder (6) and prostate (7). b) The number of deep sequencing reads normalized to gene length were graphed to produce a transcriptional profile for each of the sub-regions of the genitourinary tract (solid bars – +DNase, lined bars – -DNase).

Protein coding reads were also interrogated to determine whether select genes indicative of the genitourinary (GU) tract could be detected. Figure 3a demonstrates that markers indicative of segments of the renal nephron, the urinary bladder and the prostate essentially encompassing the entire male GU tract were found. This analysis included podocin expressed by glomerular podocytes [17], megalin expressed in the proximal tubule [18] and aquaporin 2 expressed by the collecting ducts [19]. In addition, transcripts from the bladder and prostate including the abundantly expressed uroplakins of the bladder [20] and reads aligning to the prostate specific transglutaminase-4 [21] were also detected. Many of the genes identified encode critical proteins and receptors implicated in various genetic and acquired renal diseases as well as diseases of the bladder and prostate such as prostate specific antigen (PSA)(KLK3) whose over expression is implicated in prostatic hyperplasia and prostate cancer [22]. In addition, ncRNA markers of the prostate were also identified such as prostate cancer antigen (PCA3) whose upregulation is also implicated in prostate cancer [23]. These data are consistent with the hypothesis that all regions of the urogenital tract release microvesicles. One may further speculate that such markers may be used in future targeted evaluations as non-invasive biomarkers. An expression profile could also be built based on the normalized read count method [24] (see Fig. 3b), this demonstrated that some of the most abundant reads were prostate related. It is anticipated that in addition to the analysis of transcript mutations [7], the analysis of changes in transcript expression levels may also be employed to give insights into biomarker discovery using deep sequencing.

**Figure 4:**
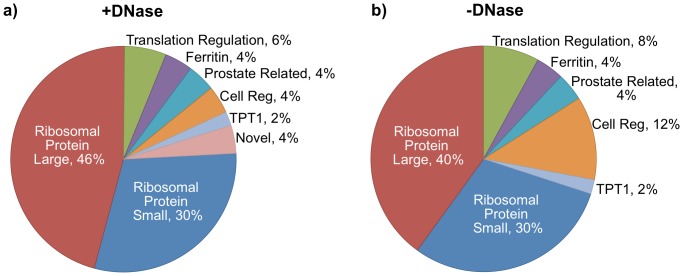
Analysis of the top 50 genes found in urinary exosomes. The top 50 most highly expressed genes were determined and grouped in terms of function or name. a) In the +DNase sample 76% of genes were related to ribosomal proteins and a further 6% related to translation regulation. Other genes related to ferritin, prostate specific genes, cell regulation and novel genes were also featured. A similar distribution was also seen for the -DNase sample (b). In both instances the TPT1 gene was the most abundantly expressed gene.

The 50 most highly expressed genes (normalized to transcript length) were also analyzed to determine which were the most common transcripts detected in microvesicles (see Table S4 and S6 for full list). A striking feature was the abundance of transcripts related to translation. Featured highly was the ribosome complex which made up 76% of the 50 most highly expressed genes in the +DNase sample. This included transcripts encoding 23 proteins of the 60S large ribosome unit and 15 proteins of the small 40S ribosome unit (Figure 4a). A similar trend was found in the -DNase sample with 70% of hits being associated with the ribosome complex including GNB2L1 which is part of the small ribosomal complex and is involved in translation repression (Figure 4b). In addition, genes related to translation elongation and initiation (EEF2 [25] and PABPC1 [26]), RPPH1 (ribosonuclease P RNA component 1) were also abundant in both samples. Additionally EEF1A1 in the -DNase sample was also abundantly expressed. Two genes related to the prostate MSMB [27] involved in spermatogenesis and the androgen regulated tumor suppressor protein NKX3-1 [28], expression of which is lost in prostate cancer, were also highly expressed consistent with the male origin of the sample. Interestingly, the two genes that make up the 24 subunit dodecahedron protein structure of ferritin encoded by ferritin light chain (FTL [29]) and ferritin heavy chain (FTH1) were both abundantly expressed within microvesicles highlighting the important role of iron metabolism in cellular function.

Proteins of unknown function, transmembrane protein 183a (TMEM183a) and C10orf116 reported to be expressed exclusively in adipose tissue, were also among the 50 most highly expressed genes. Other highly expressed proteins included ALDOB, GAPDH (+DNase sample) and additionally ACTB, ACTG1, HSPA8, UBB (-DNase sample). The most highly expressed transcript was TPT1 (tumor protein, translationally-controlled, also known as translationally controlled tumor protein (TCTP)). Its expression has been found both in mammals and higher plants [30] and double knockout in mouse models is embryonically lethal [31]. TPT1 secretion at the protein level occurs via the exosomal pathway [32], however it is not known whether its release via microvesicles at the mRNA level is also a mode of regulation.

When these top 50 mRNA transcripts were compared to the urinary exosome protein database [4] it was noted that 8 of these top 50 transcripts had also previously been reported at the protein level in urinary exosomes. This included the 2 subunits of ferritin (FTL and FTH1), GNB2L1 (RACK1) and RPS11 components of the 40S ribosome subunit that is involved in translation repression. Elongation factors EEF2 and EEF1A1, heatshock protein HSPA8 and GAPDH. Novel protein C10orf116 was also detected at the protein level suggesting it may play an important role in cellular function.

Although RNase digestion was carried out to limit extravesicular RNA contamination, there is still a possibility that the RNA analyzed may in part be derived from free RNA protected by protein/lipids rather than being fully packaged in a lipid bilayer vesicle. This continues to be a caveat in studies examining RNA from isolated exosome/microvesicle pellets.

Using deep sequencing we were able to comprehensively assess the nucleic acid profile within urinary microvesicles. We demonstrate that a transcriptional profile of the urogenital system can be constructed non-invasively, which has immediate and obvious applications for the discovery of new biomarkers allowing for routine organ function analysis at the transcriptional level without the need for biopsy. We also demonstrate that microvesicles are surprisingly rich in non-coding RNA which a growing body of data indicates plays an important role in cellular regulation [33]–[35] and which can now be analyzed in humans on a larger scale using non-invasive microvesicle RNA analysis.

## Supporting Information

Table S1 **Known repeats found in microvesicles (including exosomes) (+DNase).** Listing of all 545 human repeats for which a known loci overlapped with the alignment loci of a microvesicle derived read.
(ZIP)

Table S2 **Known repeats found in microvesicles (including exosomes) (-DNase).** Listing of all 690 human repeats for which a known loci overlapped with the alignment loci of a microvesicle derived read.
(ZIP)

Table S3 **Known ncRNA found in microvesicles (including exosomes) (+DNase).** Listing of the 196 ncRNA transcripts (rnaDB set  =  "curated from literature") and the number of microvesicle derived sequencing transcripts that aligned against them.
(ZIP)

Table S4 **Known ncRNA found in microvesicles (including exosomes) (-DNase).** Listing of the 201 ncRNA transcripts (rnaDB set  =  "curated from literature") and the number of microvesicle derived sequencing transcripts that aligned against them.
(ZIP)

Table S5 **Genes found within microvesicles (including exosomes) (+DNase).** Raw and normalized counts (as per Casava's 1.7 read counts method) of genes identified in microvesicles.
(ZIP)

Table S6 **Genes found within microvesicles (including exosomes) (-DNase).** Raw and normalized counts (as per Casava's 1.7 read counts method) of genes identified in microvesicles.
(ZIP)
