# Supplementary material for: Massively Parallel Sequencing of Human Urinary Exosome/Microvesicle RNA Reveals a Predominance of Non-Coding RNA
Source: PLoS One. 2014 May 9;9(5):e96094. doi: 10.1371/journal.pone.0096094 (PMC4015934; doi:10.1371/journal.pone.0096094)
Supplement: Table S1 — Known repeats found in microvesicles (including exosomes) (+DNase). Listing of all 545 human repeats for which a known loci overlapped with the alignment loci of a microvesicle derived read. (ZIP) [file pone.0096094.s001.zip › PLOS Russo Supp Table S1 Final 040814.pdf]

Supp. Table S1: Known repeats found in microvesicles (including exosomes) (+DNase)

Listing of all 545 human repeats for which a known loci overlapped with the alignment loci of a microvesicle derived read

count	Name of Repeat	Class of Repeat	Family of repeat
1	Arthur1A	DNA	hAT-Tip100
2	Arthur1B	DNA	hAT-Tip100
3	Charlie1	DNA	hAT-Charlie
4	Charlie10	DNA	hAT-Charlie
5	Charlie16a	DNA	hAT-Charlie
6	Charlie17a	DNA	hAT-Charlie
7	Charlie19a	DNA	hAT-Charlie
8	Charlie1b	DNA	hAT-Charlie
9	Charlie23a	DNA	hAT-Charlie
10	Charlie25	DNA	hAT-Charlie
11	Charlie2b	DNA	hAT-Charlie
12	Charlie4a	DNA	hAT-Charlie
13	Charlie4z	DNA	hAT-Charlie
14	Charlie5	DNA	hAT-Charlie
15	Charlie6	DNA	hAT-Charlie
16	Charlie7	DNA	hAT-Charlie
17	Charlie8	DNA	hAT-Charlie
18	Cheshire	DNA	hAT-Charlie
19	HSMAR1	DNA	TcMar-Mariner
20	HSMAR2	DNA	TcMar-Mariner
21	Kanga2_a	DNA	TcMar-Tc2
22	MARNA	DNA	TcMar-Mariner
23	MER102a	DNA	hAT-Charlie
24	MER102b	DNA	hAT-Charlie
25	MER102c	DNA	hAT-Charlie
26	MER103C	DNA	hAT-Charlie
27	MER112	DNA	hAT-Charlie
28	MER113A	DNA	hAT-Charlie
29	MER115	DNA	hAT-Tip100
30	MER117	DNA	hAT-Charlie
31	MER119	DNA	hAT-Charlie
32	MER126	DNA	DNA
33	MER135	DNA	DNA
34	MER1A	DNA	hAT-Charlie
35	MER1B	DNA	hAT-Charlie
36	MER2	DNA	TcMar-Tigger

37	MER20	DNA	hAT-Charlie
38	MER2B	DNA	TcMar-Tigger
39	MER3	DNA	hAT-Charlie
40	MER30	DNA	hAT-Charlie
41	MER33	DNA	hAT-Charlie
42	MER44A	DNA	TcMar-Tigger
43	MER44B	DNA	TcMar-Tigger
44	MER45A	DNA	hAT-Tip100
45	MER45C	DNA	hAT-Tip100
46	MER47B	DNA	TcMar-Tigger
47	MER53	DNA	hAT
48	MER58A	DNA	hAT-Charlie
49	MER58B	DNA	hAT-Charlie
50	MER5A	DNA	hAT-Charlie
51	MER5A1	DNA	hAT-Charlie
52	MER5B	DNA	hAT-Charlie
53	MER5C1	DNA	hAT-Charlie
54	MER63A	DNA	hAT-Blackjack
55	MER6A	DNA	TcMar-Tigger
56	MER8	DNA	TcMar-Tigger
57	MER82	DNA	TcMar-Tigger
58	MER91A	DNA	hAT-Tip100
59	MER91B	DNA	hAT-Tip100
60	MER91C	DNA	hAT-Tip100
61	MER96B	DNA	hAT
62	MamRep1161	DNA	TcMar
63	MamRep1894	DNA	hAT
64	MamRep38	DNA	hAT
65	MamRep434	DNA	TcMar-Tigger
66	Tigger1	DNA	TcMar-Tigger
67	Tigger10	DNA	TcMar-Tigger
68	Tigger13a	DNA	TcMar-Tigger
69	Tigger14a	DNA	TcMar-Tigger
70	Tigger15a	DNA	TcMar-Tigger
71	Tigger16b	DNA	TcMar-Tigger
72	Tigger2	DNA	TcMar-Tigger
73	Tigger2a	DNA	TcMar-Tigger
74	Tigger3a	DNA	TcMar-Tigger
75	Tigger3b	DNA	TcMar-Tigger
76	Tigger3c	DNA	TcMar-Tigger
77	Tigger4	DNA	TcMar-Tigger

78	Tigger4a	DNA	TcMar-Tigger
79	Tigger4b	DNA	TcMar-Tigger
80	Tigger7	DNA	TcMar-Tigger
81	Zaphod3	DNA	hAT-Tip100
82	HAL1	LINE	L1
83	HAL1-3A_ME	LINE	L1
84	L1HS	LINE	L1
85	L1M1	LINE	L1
86	L1M2	LINE	L1
87	L1M3	LINE	L1
88	L1M3a	LINE	L1
89	L1M3c	LINE	L1
90	L1M3f	LINE	L1
91	L1M4	LINE	L1
92	L1M4b	LINE	L1
93	L1M4c	LINE	L1
94	L1M5	LINE	L1
95	L1M6	LINE	L1
96	L1M7	LINE	L1
97	L1MA1	LINE	L1
98	L1MA10	LINE	L1
99	L1MA2	LINE	L1
100	L1MA3	LINE	L1
101	L1MA4	LINE	L1
102	L1MA4A	LINE	L1
103	L1MA5	LINE	L1
104	L1MA6	LINE	L1
105	L1MA7	LINE	L1
106	L1MA8	LINE	L1
107	L1MA9	LINE	L1
108	L1MB1	LINE	L1
109	L1MB2	LINE	L1
110	L1MB3	LINE	L1
111	L1MB4	LINE	L1
112	L1MB5	LINE	L1
113	L1MB7	LINE	L1
114	L1MB8	LINE	L1
115	L1MC	LINE	L1
116	L1MC1	LINE	L1
117	L1MC2	LINE	L1
118	L1MC3	LINE	L1

119	L1MC4	LINE	L1
120	L1MC4a	LINE	L1
121	L1MC5	LINE	L1
122	L1MCa	LINE	L1
123	L1MCb	LINE	L1
124	L1MCc	LINE	L1
125	L1MD	LINE	L1
126	L1MD1	LINE	L1
127	L1MD2	LINE	L1
128	L1MD3	LINE	L1
129	L1MDa	LINE	L1
130	L1MDb	LINE	L1
131	L1ME1	LINE	L1
132	L1ME2	LINE	L1
133	L1ME2z	LINE	L1
134	L1ME3	LINE	L1
135	L1ME3A	LINE	L1
136	L1ME3B	LINE	L1
137	L1ME3C	LINE	L1
138	L1ME3D	LINE	L1
139	L1ME3E	LINE	L1
140	L1ME3F	LINE	L1
141	L1ME4a	LINE	L1
142	L1ME5	LINE	L1
143	L1MEc	LINE	L1
144	L1MEe	LINE	L1
145	L1MEf	LINE	L1
146	L1MEg	LINE	L1
147	L1MEg1	LINE	L1
148	L1MEg2	LINE	L1
149	L1P1	LINE	L1
150	L1P2	LINE	L1
151	L1P3	LINE	L1
152	L1PA10	LINE	L1
153	L1PA11	LINE	L1
154	L1PA12	LINE	L1
155	L1PA13	LINE	L1
156	L1PA14	LINE	L1
157	L1PA15	LINE	L1
158	L1PA16	LINE	L1
159	L1PA2	LINE	L1

160	L1PA3	LINE	L1
161	L1PA4	LINE	L1
162	L1PA5	LINE	L1
163	L1PA6	LINE	L1
164	L1PA7	LINE	L1
165	L1PA8	LINE	L1
166	L1PA8A	LINE	L1
167	L1PB1	LINE	L1
168	L1PB2	LINE	L1
169	L1PB3	LINE	L1
170	L1PB4	LINE	L1
171	L1PREC2	LINE	L1
172	L2	LINE	L2
173	L2a	LINE	L2
174	L2b	LINE	L2
175	L2c	LINE	L2
176	L3	LINE	CR1
177	L3b	LINE	CR1
178	L4	LINE	RTE
179	Plat_L3	LINE	CR1
180	X3_LINE	LINE	RTE-BovB
181	X5A_LINE	LINE	CR1
182	X6B_LINE	LINE	CR1
183	X9_LINE	LINE	L1?
184	ERV3-16A3_I-int	LTR	ERVL
185	ERVL-B4-int	LTR	ERVL
186	HERV15-int	LTR	ERV1
187	HERV16-int	LTR	ERVL
188	HERV17-int	LTR	ERV1
189	HERV3-int	LTR	ERV1
190	HERV9-int	LTR	ERV1
191	HERVE-int	LTR	ERV1
192	HERVE_a-int	LTR	ERV1
193	HERVH-int	LTR	ERV1
194	HERVI-int	LTR	ERV1
195	HERVIP10F-int	LTR	ERV1
196	HERVIP10FH-int	LTR	ERV1
197	HERVK-int	LTR	ERVK
198	HERVK14-int	LTR	ERVK
199	HERVK22-int	LTR	ERVK
200	HERVK3-int	LTR	ERVK

201	HERVL-int	LTR	ERVL
202	HERVL40-int	LTR	ERVL
203	HERVL66-int	LTR	ERVL
204	HERVL74-int	LTR	ERVL
205	HERVP71A-int	LTR	ERV1
206	HERVS71-int	LTR	ERV1
207	HUERS-P1-int	LTR	ERV1
208	HUERS-P3-int	LTR	ERV1
209	Harlequin-int	LTR	ERV1
210	LOR1-int	LTR	ERV1
211	LTR1	LTR	ERV1
212	LTR10C	LTR	ERV1
213	LTR12	LTR	ERV1
214	LTR12C	LTR	ERV1
215	LTR12D	LTR	ERV1
216	LTR12E	LTR	ERV1
217	LTR12_	LTR	ERV1
218	LTR13	LTR	ERVK
219	LTR14B	LTR	ERVK
220	LTR16A1	LTR	ERVL
221	LTR16C	LTR	ERVL
222	LTR19-int	LTR	ERV1
223	LTR19A	LTR	ERV1
224	LTR19B	LTR	ERV1
225	LTR19C	LTR	ERV1
226	LTR1C	LTR	ERV1
227	LTR1D	LTR	ERV1
228	LTR2	LTR	ERV1
229	LTR21A	LTR	ERV1
230	LTR23	LTR	ERV1
231	LTR24B	LTR	ERV1
232	LTR25-int	LTR	ERV1
233	LTR2B	LTR	ERV1
234	LTR2C	LTR	ERV1
235	LTR3	LTR	ERVK
236	LTR30	LTR	ERV1
237	LTR32	LTR	ERVL
238	LTR33	LTR	ERVL
239	LTR35	LTR	ERV1
240	LTR37B	LTR	ERV1
241	LTR38B	LTR	ERV1

242	LTR39	LTR	ERV1
243	LTR3A	LTR	ERVK
244	LTR3B_	LTR	ERVK
245	LTR40a	LTR	ERVL
246	LTR40c	LTR	ERVL
247	LTR41	LTR	ERVL
248	LTR41B	LTR	ERVL
249	LTR46	LTR	ERV1
250	LTR46-int	LTR	ERV1
251	LTR47A	LTR	ERVL
252	LTR47B	LTR	ERVL
253	LTR48	LTR	ERV1
254	LTR5	LTR	ERVK
255	LTR50	LTR	ERVL
256	LTR52	LTR	ERVL
257	LTR53	LTR	ERVL
258	LTR55	LTR	ERV
259	LTR5A	LTR	ERVK
260	LTR5B	LTR	ERVK
261	LTR5_Hs	LTR	ERVK
262	LTR66	LTR	ERVL
263	LTR6A	LTR	ERV1
264	LTR6B	LTR	ERV1
265	LTR7	LTR	ERV1
266	LTR77	LTR	ERV1
267	LTR78	LTR	ERV1
268	LTR79	LTR	ERVL
269	LTR7B	LTR	ERV1
270	LTR7C	LTR	ERV1
271	LTR7Y	LTR	ERV1
272	LTR8	LTR	ERV1
273	LTR85b	LTR	Gypsy?
274	LTR87	LTR	ERVL?
275	LTR88c	LTR	Gypsy?
276	LTR8A	LTR	ERV1
277	LTR9	LTR	ERV1
278	LTR9B	LTR	ERV1
279	MER101-int	LTR	ERV1
280	MER11A	LTR	ERVK
281	MER11C	LTR	ERVK
282	MER11D	LTR	ERVK

283	MER21-int	LTR	ERVL
284	MER21A	LTR	ERVL
285	MER21B	LTR	ERVL
286	MER21C	LTR	ERVL
287	MER31A	LTR	ERV1
288	MER34A1	LTR	ERV1
289	MER34B-int	LTR	ERV1
290	MER34C	LTR	ERV1
291	MER34C_	LTR	ERV1
292	MER4-int	LTR	ERV1
293	MER41-int	LTR	ERV1
294	MER41A	LTR	ERV1
295	MER41B	LTR	ERV1
296	MER41D	LTR	ERV1
297	MER49	LTR	ERV1
298	MER4A	LTR	ERV1
299	MER4A1	LTR	ERV1
300	MER4A1_	LTR	ERV1
301	MER4D1	LTR	ERV1
302	MER50-int	LTR	ERV1
303	MER51-int	LTR	ERV1
304	MER51A	LTR	ERV1
305	MER51B	LTR	ERV1
306	MER51C	LTR	ERV1
307	MER52-int	LTR	ERV1
308	MER52A	LTR	ERV1
309	MER52C	LTR	ERV1
310	MER52D	LTR	ERV1
311	MER57-int	LTR	ERV1
312	MER57A-int	LTR	ERV1
313	MER57A1	LTR	ERV1
314	MER57B2	LTR	ERV1
315	MER57E1	LTR	ERV1
316	MER61-int	LTR	ERV1
317	MER65A	LTR	ERV1
318	MER65D	LTR	ERV1
319	MER66B	LTR	ERV1
320	MER67C	LTR	ERV1
321	MER68	LTR	ERVL
322	MER68-int	LTR	ERVL
323	MER74A	LTR	ERVL

324	MER77	LTR	ERVL
325	MER83	LTR	ERV1
326	MER89-int	LTR	ERV1
327	MER90	LTR	ERV1
328	MER90a	LTR	ERV1
329	MLT1A	LTR	ERVL-MaLR
330	MLT1A0	LTR	ERVL-MaLR
331	MLT1A1	LTR	ERVL-MaLR
332	MLT1B	LTR	ERVL-MaLR
333	MLT1C	LTR	ERVL-MaLR
334	MLT1D	LTR	ERVL-MaLR
335	MLT1E	LTR	ERVL-MaLR
336	MLT1E1A	LTR	ERVL-MaLR
337	MLT1E2	LTR	ERVL-MaLR
338	MLT1E3	LTR	ERVL-MaLR
339	MLT1F	LTR	ERVL-MaLR
340	MLT1F-int	LTR	ERVL-MaLR
341	MLT1F1	LTR	ERVL-MaLR
342	MLT1F2	LTR	ERVL-MaLR
343	MLT1G	LTR	ERVL-MaLR
344	MLT1G1	LTR	ERVL-MaLR
345	MLT1G3	LTR	ERVL-MaLR
346	MLT1H	LTR	ERVL-MaLR
347	MLT1H2	LTR	ERVL-MaLR
348	MLT1I	LTR	ERVL-MaLR
349	MLT1J	LTR	ERVL-MaLR
350	MLT1J1	LTR	ERVL-MaLR
351	MLT1K	LTR	ERVL-MaLR
352	MLT1L	LTR	ERVL-MaLR
353	MLT1N2	LTR	ERVL-MaLR
354	MLT2A1	LTR	ERVL
355	MLT2A2	LTR	ERVL
356	MLT2B1	LTR	ERVL
357	MLT2B2	LTR	ERVL
358	MLT2C2	LTR	ERVL
359	MLT2D	LTR	ERVL
360	MLT2F	LTR	ERVL
361	MSTA	LTR	ERVL-MaLR
362	MSTA-int	LTR	ERVL-MaLR
363	MSTB	LTR	ERVL-MaLR
364	MSTB-int	LTR	ERVL-MaLR

365	MSTB1	LTR	ERVL-MaLR
366	MSTD	LTR	ERVL-MaLR
367	MamGyp-int	LTR	Gypsy
368	MamGypLTR1b	LTR	Gypsy
369	MamGypLTR1c	LTR	Gypsy
370	MamGypLTR2b	LTR	Gypsy
371	MamGypLTR3	LTR	Gypsy
372	MamRep1527	LTR	LTR
373	PABL_A	LTR	ERV1
374	PRIMA41-int	LTR	ERV1
375	PRIMA4_LTR	LTR	ERV1
376	THE1A	LTR	ERVL-MaLR
377	THE1A-int	LTR	ERVL-MaLR
378	THE1B	LTR	ERVL-MaLR
379	THE1B-int	LTR	ERVL-MaLR
380	THE1C	LTR	ERVL-MaLR
381	THE1C-int	LTR	ERVL-MaLR
382	THE1D	LTR	ERVL-MaLR
383	THE1D-int	LTR	ERVL-MaLR
384	A-rich	Low_complexity	Low_complexity
385	AT_rich	Low_complexity	Low_complexity
386	C-rich	Low_complexity	Low_complexity
387	CT-rich	Low_complexity	Low_complexity
388	G-rich	Low_complexity	Low_complexity
389	GA-rich	Low_complexity	Low_complexity
390	GC_rich	Low_complexity	Low_complexity
391	T-rich	Low_complexity	Low_complexity
392	polypurine	Low_complexity	Low_complexity
393	polypyrimidine	Low_complexity	Low_complexity
394	SVA_A	Other	Other
395	SVA_B	Other	Other
396	SVA_C	Other	Other
397	SVA_D	Other	Other
398	SVA_E	Other	Other
399	SVA_F	Other	Other
400	7SK	RNA	RNA
401	AluJb	SINE	Alu
402	AluJo	SINE	Alu
403	AluJr	SINE	Alu
404	AluJr4	SINE	Alu
405	AluSc	SINE	Alu

406	AluSc5	SINE	Alu
407	AluSc8	SINE	Alu
408	AluSg	SINE	Alu
409	AluSg4	SINE	Alu
410	AluSg7	SINE	Alu
411	AluSp	SINE	Alu
412	AluSq	SINE	Alu
413	AluSq10	SINE	Alu
414	AluSq2	SINE	Alu
415	AluSq4	SINE	Alu
416	AluSx	SINE	Alu
417	AluSx1	SINE	Alu
418	AluSx3	SINE	Alu
419	AluSx4	SINE	Alu
420	AluSz	SINE	Alu
421	AluSz6	SINE	Alu
422	AluY	SINE	Alu
423	AluYa5	SINE	Alu
424	AluYa8	SINE	Alu
425	AluYc	SINE	Alu
426	AluYg6	SINE	Alu
427	AluYk4	SINE	Alu
428	FAM	SINE	Alu
429	FLAM_A	SINE	Alu
430	FLAM_C	SINE	Alu
431	FRAM	SINE	Alu
432	MIR	SINE	MIR
433	MIR3	SINE	MIR
434	MIRb	SINE	MIR
435	MIRc	SINE	MIR
436	MamSINE1	SINE	tRNA
437	ALR/Alpha	Satellite	centr
438	BSR/Beta	Satellite	Satellite
439	GSATII	Satellite	centr
440	LSAU	Satellite	Satellite
441	MSR1	Satellite	Satellite
442	REP522	Satellite	telo
443	SST1	Satellite	centr
444	TAR1	Satellite	telo
445	(A)n	Simple_repeat	Simple_repeat
446	(AATAG)n	Simple_repeat	Simple_repeat

447	(AGGGGG)n	Simple_repeat	Simple_repeat
448	(ATAGG)n	Simple_repeat	Simple_repeat
449	(ATG)n	Simple_repeat	Simple_repeat
450	(ATTG)n	Simple_repeat	Simple_repeat
451	(C)n	Simple_repeat	Simple_repeat
452	(CA)n	Simple_repeat	Simple_repeat
453	(CAA)n	Simple_repeat	Simple_repeat
454	(CAAA)n	Simple_repeat	Simple_repeat
455	(CAAAA)n	Simple_repeat	Simple_repeat
456	(CAAAAA)n	Simple_repeat	Simple_repeat
457	(CAAT)n	Simple_repeat	Simple_repeat
458	(CACCC)n	Simple_repeat	Simple_repeat
459	(CAG)n	Simple_repeat	Simple_repeat
460	(CAGAGA)n	Simple_repeat	Simple_repeat
461	(CAGCC)n	Simple_repeat	Simple_repeat
462	(CAGGC)n	Simple_repeat	Simple_repeat
463	(CAGGG)n	Simple_repeat	Simple_repeat
464	(CAT)n	Simple_repeat	Simple_repeat
465	(CATATA)n	Simple_repeat	Simple_repeat
466	(CCA)n	Simple_repeat	Simple_repeat
467	(CCCA)n	Simple_repeat	Simple_repeat
468	(CCCCAA)n	Simple_repeat	Simple_repeat
469	(CCCCAG)n	Simple_repeat	Simple_repeat
470	(CCCCCG)n	Simple_repeat	Simple_repeat
471	(CCCCCT)n	Simple_repeat	Simple_repeat
472	(CCCCG)n	Simple_repeat	Simple_repeat
473	(CCCG)n	Simple_repeat	Simple_repeat
474	(CCG)n	Simple_repeat	Simple_repeat
475	(CCGCG)n	Simple_repeat	Simple_repeat
476	(CG)n	Simple_repeat	Simple_repeat
477	(CGG)n	Simple_repeat	Simple_repeat
478	(CGGA)n	Simple_repeat	Simple_repeat
479	(CGGG)n	Simple_repeat	Simple_repeat
480	(CGGGG)n	Simple_repeat	Simple_repeat
481	(CGGGGG)n	Simple_repeat	Simple_repeat
482	(CTG)n	Simple_repeat	Simple_repeat
483	(CTGGGG)n	Simple_repeat	Simple_repeat
484	(G)n	Simple_repeat	Simple_repeat
485	(GA)n	Simple_repeat	Simple_repeat
486	(GAA)n	Simple_repeat	Simple_repeat
487	(GAAA)n	Simple_repeat	Simple_repeat

488	(GAAAA)n	Simple_repeat	Simple_repeat
489	(GCTG)n	Simple_repeat	Simple_repeat
490	(GGA)n	Simple_repeat	Simple_repeat
491	(GGAAA)n	Simple_repeat	Simple_repeat
492	(GGGA)n	Simple_repeat	Simple_repeat
493	(GGGGA)n	Simple_repeat	Simple_repeat
494	(GGGTG)n	Simple_repeat	Simple_repeat
495	(T)n	Simple_repeat	Simple_repeat
496	(TA)n	Simple_repeat	Simple_repeat
497	(TAAA)n	Simple_repeat	Simple_repeat
498	(TAAAA)n	Simple_repeat	Simple_repeat
499	(TAAAAA)n	Simple_repeat	Simple_repeat
500	(TAGA)n	Simple_repeat	Simple_repeat
501	(TAGG)n	Simple_repeat	Simple_repeat
502	(TATATG)n	Simple_repeat	Simple_repeat
503	(TATG)n	Simple_repeat	Simple_repeat
504	(TC)n	Simple_repeat	Simple_repeat
505	(TCC)n	Simple_repeat	Simple_repeat
506	(TCCC)n	Simple_repeat	Simple_repeat
507	(TCCCC)n	Simple_repeat	Simple_repeat
508	(TCTCCC)n	Simple_repeat	Simple_repeat
509	(TCTCTG)n	Simple_repeat	Simple_repeat
510	(TG)n	Simple_repeat	Simple_repeat
511	(TGG)n	Simple_repeat	Simple_repeat
512	(TGGA)n	Simple_repeat	Simple_repeat
513	(TGGGGG)n	Simple_repeat	Simple_repeat
514	(TTA)n	Simple_repeat	Simple_repeat
515	(TTAA)n	Simple_repeat	Simple_repeat
516	(TTAGGG)n	Simple_repeat	Simple_repeat
517	(TTC)n	Simple_repeat	Simple_repeat
518	(TTCC)n	Simple_repeat	Simple_repeat
519	(TTCGGG)n	Simple_repeat	Simple_repeat
520	(TTG)n	Simple_repeat	Simple_repeat
521	(TTTA)n	Simple_repeat	Simple_repeat
522	(TTTC)n	Simple_repeat	Simple_repeat
523	(TTTG)n	Simple_repeat	Simple_repeat
524	(TTTTA)n	Simple_repeat	Simple_repeat
525	(TTTTC)n	Simple_repeat	Simple_repeat
526	(TTTTG)n	Simple_repeat	Simple_repeat
527	(TTTTTA)n	Simple_repeat	Simple_repeat
528	(TTTTTG)n	Simple_repeat	Simple_repeat

529	MamRep605	Unknown	Unknown
530	UCON28a	Unknown	Unknown
531	5S	rRNA	rRNA
532	LSU-rRNA_Hsa	rRNA	rRNA
533	SSU-rRNA_Hsa	rRNA	rRNA
534	U1	snRNA	snRNA
535	U13_	snRNA	snRNA
536	U2	snRNA	snRNA
537	U3	snRNA	snRNA
538	U4	snRNA	snRNA
539	U6	snRNA	snRNA
540	7SLRNA	srpRNA	srpRNA
541	tRNA-Gly-GGY	tRNA	tRNA
542	tRNA-Leu-CTY	tRNA	tRNA
543	tRNA-Met-i	tRNA	tRNA
544	tRNA-Pro-CCA	tRNA	tRNA
545	tRNA-Ser-TCG	tRNA	tRNA
