# Supplementary material for: Massively Parallel Sequencing of Human Urinary Exosome/Microvesicle RNA Reveals a Predominance of Non-Coding RNA
Source: PLoS One. 2014 May 9;9(5):e96094. doi: 10.1371/journal.pone.0096094 (PMC4015934; doi:10.1371/journal.pone.0096094)
Supplement: Table S2 — Known repeats found in microvesicles (including exosomes) (-DNase). Listing of all 690 human repeats for which a known loci overlapped with the alignment loci of a microvesicle derived read. (ZIP) [file pone.0096094.s002.zip › PLOS Russo Supp Table S2 Final 040814.pdf]

Supp. Table S2: Known repeats found in microvesicles (including exosomes) (-DNase)
Listing of all 690 human repeats for which a known loci overlapped with the alignment loci of a microvesicle derived read

count	Name of Repeat	Class of Repeat	Family of repeat
1	Arthur1	DNA	hAT-Tip100
2	Arthur1A	DNA	hAT-Tip100
3	Arthur1B	DNA	hAT-Tip100
4	Charlie10	DNA	hAT-Charlie
5	Charlie10a	DNA	hAT-Charlie
6	Charlie14a	DNA	hAT-Charlie
7	Charlie15a	DNA	hAT-Charlie
8	Charlie16a	DNA	hAT-Charlie
9	Charlie17a	DNA	hAT-Charlie
10	Charlie18a	DNA	hAT-Charlie
11	Charlie19a	DNA	hAT-Charlie
12	Charlie1a	DNA	hAT-Charlie
13	Charlie21a	DNA	hAT-Charlie
14	Charlie23a	DNA	hAT-Charlie
15	Charlie24	DNA	hAT-Charlie
16	Charlie25	DNA	hAT-Charlie
17	Charlie2a	DNA	hAT-Charlie
18	Charlie3	DNA	hAT-Charlie
19	Charlie4a	DNA	hAT-Charlie
20	Charlie4z	DNA	hAT-Charlie
21	Charlie6	DNA	hAT-Charlie
22	Charlie8	DNA	hAT-Charlie
23	FordPrefect	DNA	hAT-Tip100
24	HSMAR1	DNA	TcMar-Mariner
25	HSMAR2	DNA	TcMar-Mariner
26	Kanga1a	DNA	TcMar-Tc2
27	Kanga1c	DNA	TcMar-Tc2
28	Kanga2_a	DNA	TcMar-Tc2
29	Looper	DNA	PiggyBac
30	MADE1	DNA	TcMar-Mariner
31	MARNA	DNA	TcMar-Mariner
32	MER102a	DNA	hAT-Charlie
33	MER102b	DNA	hAT-Charlie
34	MER102c	DNA	hAT-Charlie
35	MER103C	DNA	hAT-Charlie

36	MER105	DNA	DNA
37	MER112	DNA	hAT-Charlie
38	MER113	DNA	hAT-Charlie
39	MER113A	DNA	hAT-Charlie
40	MER115	DNA	hAT-Tip100
41	MER117	DNA	hAT-Charlie
42	MER119	DNA	hAT-Charlie
43	MER1A	DNA	hAT-Charlie
44	MER1B	DNA	hAT-Charlie
45	MER2	DNA	TcMar-Tigger
46	MER20	DNA	hAT-Charlie
47	MER20B	DNA	hAT-Charlie
48	MER2B	DNA	TcMar-Tigger
49	MER3	DNA	hAT-Charlie
50	MER30	DNA	hAT-Charlie
51	MER30B	DNA	hAT-Charlie
52	MER33	DNA	hAT-Charlie
53	MER44A	DNA	TcMar-Tigger
54	MER44C	DNA	TcMar-Tigger
55	MER45A	DNA	hAT-Tip100
56	MER46C	DNA	TcMar-Tigger
57	MER47A	DNA	TcMar-Tigger
58	MER47C	DNA	TcMar-Tigger
59	MER53	DNA	hAT
60	MER58A	DNA	hAT-Charlie
61	MER58B	DNA	hAT-Charlie
62	MER58C	DNA	hAT-Charlie
63	MER5A	DNA	hAT-Charlie
64	MER5A1	DNA	hAT-Charlie
65	MER5B	DNA	hAT-Charlie
66	MER5C	DNA	hAT-Charlie
67	MER63A	DNA	hAT-Blackjack
68	MER63B	DNA	hAT-Blackjack
69	MER8	DNA	TcMar-Tigger
70	MER82	DNA	TcMar-Tigger
71	MER91B	DNA	hAT-Tip100
72	MER94	DNA	hAT-Blackjack
73	MER96	DNA	hAT
74	MER96B	DNA	hAT
75	MER97a	DNA	hAT-Tip100
76	MamRep1161	DNA	TcMar

77	MamRep4096	DNA	hAT?
78	MamRep434	DNA	TcMar-Tigger
79	ORSL	DNA	hAT-Tip100
80	Ricksha_c	DNA	MuDR
81	Tigger1	DNA	TcMar-Tigger
82	Tigger10	DNA	TcMar-Tigger
83	Tigger12	DNA	TcMar-Tigger
84	Tigger12c	DNA	TcMar-Tigger
85	Tigger13a	DNA	TcMar-Tigger
86	Tigger14a	DNA	TcMar-Tigger
87	Tigger15a	DNA	TcMar-Tigger
88	Tigger16b	DNA	TcMar-Tigger
89	Tigger2	DNA	TcMar-Tigger
90	Tigger2a	DNA	TcMar-Tigger
91	Tigger2b_Pri	DNA	TcMar-Tigger
92	Tigger3	DNA	TcMar-Tigger
93	Tigger3a	DNA	TcMar-Tigger
94	Tigger3b	DNA	TcMar-Tigger
95	Tigger3c	DNA	TcMar-Tigger
96	Tigger4	DNA	TcMar-Tigger
97	Tigger4a	DNA	TcMar-Tigger
98	Tigger4b	DNA	TcMar-Tigger
99	Tigger5	DNA	TcMar-Tigger
100	Tigger7	DNA	TcMar-Tigger
101	Tigger8	DNA	TcMar-Tigger
102	Tigger9a	DNA	TcMar-Tigger
103	Zaphod	DNA	hAT-Tip100
104	Zaphod3	DNA	hAT-Tip100
105	CR1_Mam	LINE	CR1
106	HAL1	LINE	L1
107	HAL1-2a_MD	LINE	L1
108	HAL1-3A_ME	LINE	L1
109	HAL1b	LINE	L1
110	L1HS	LINE	L1
111	L1M1	LINE	L1
112	L1M2	LINE	L1
113	L1M2a	LINE	L1
114	L1M2a1	LINE	L1
115	L1M3	LINE	L1
116	L1M3a	LINE	L1
117	L1M3d	LINE	L1

118	L1M3f	LINE	L1
119	L1M4	LINE	L1
120	L1M4b	LINE	L1
121	L1M4c	LINE	L1
122	L1M5	LINE	L1
123	L1M6	LINE	L1
124	L1M7	LINE	L1
125	L1MA1	LINE	L1
126	L1MA10	LINE	L1
127	L1MA2	LINE	L1
128	L1MA3	LINE	L1
129	L1MA4	LINE	L1
130	L1MA4A	LINE	L1
131	L1MA5	LINE	L1
132	L1MA5A	LINE	L1
133	L1MA6	LINE	L1
134	L1MA7	LINE	L1
135	L1MA8	LINE	L1
136	L1MA9	LINE	L1
137	L1MB1	LINE	L1
138	L1MB2	LINE	L1
139	L1MB3	LINE	L1
140	L1MB4	LINE	L1
141	L1MB5	LINE	L1
142	L1MB7	LINE	L1
143	L1MB8	LINE	L1
144	L1MC	LINE	L1
145	L1MC1	LINE	L1
146	L1MC2	LINE	L1
147	L1MC3	LINE	L1
148	L1MC4	LINE	L1
149	L1MC4a	LINE	L1
150	L1MC5	LINE	L1
151	L1MCa	LINE	L1
152	L1MCb	LINE	L1
153	L1MCc	LINE	L1
154	L1MD	LINE	L1
155	L1MD1	LINE	L1
156	L1MD2	LINE	L1
157	L1MD3	LINE	L1
158	L1MDa	LINE	L1

159	L1ME1	LINE	L1
160	L1ME2	LINE	L1
161	L1ME2z	LINE	L1
162	L1ME3	LINE	L1
163	L1ME3A	LINE	L1
164	L1ME3B	LINE	L1
165	L1ME3C	LINE	L1
166	L1ME3D	LINE	L1
167	L1ME3E	LINE	L1
168	L1ME3F	LINE	L1
169	L1ME4a	LINE	L1
170	L1ME5	LINE	L1
171	L1MEa	LINE	L1
172	L1MEc	LINE	L1
173	L1MEd	LINE	L1
174	L1MEe	LINE	L1
175	L1MEf	LINE	L1
176	L1MEg	LINE	L1
177	L1MEg2	LINE	L1
178	L1P1	LINE	L1
179	L1P2	LINE	L1
180	L1P3	LINE	L1
181	L1P4	LINE	L1
182	L1P4a	LINE	L1
183	L1P4b	LINE	L1
184	L1PA10	LINE	L1
185	L1PA11	LINE	L1
186	L1PA12	LINE	L1
187	L1PA13	LINE	L1
188	L1PA14	LINE	L1
189	L1PA15	LINE	L1
190	L1PA15-16	LINE	L1
191	L1PA16	LINE	L1
192	L1PA17	LINE	L1
193	L1PA2	LINE	L1
194	L1PA3	LINE	L1
195	L1PA4	LINE	L1
196	L1PA5	LINE	L1
197	L1PA6	LINE	L1
198	L1PA7	LINE	L1
199	L1PA8	LINE	L1

200	L1PA8A	LINE	L1
201	L1PB	LINE	L1
202	L1PB1	LINE	L1
203	L1PB2	LINE	L1
204	L1PB3	LINE	L1
205	L1PB4	LINE	L1
206	L1PBa	LINE	L1
207	L1PREC2	LINE	L1
208	L2	LINE	L2
209	L2a	LINE	L2
210	L2b	LINE	L2
211	L2c	LINE	L2
212	L3	LINE	CR1
213	L3b	LINE	CR1
214	L4	LINE	RTE
215	Plat_L3	LINE	CR1
216	X5A_LINE	LINE	CR1
217	X6A_LINE	LINE	CR1
218	X9_LINE	LINE	L1?
219	ERV3-16A3_I-int	LTR	ERVL
220	ERVL-B4-int	LTR	ERVL
221	ERVL-E-int	LTR	ERVL
222	ERVL-int	LTR	ERVL
223	HERV16-int	LTR	ERVL
224	HERV17-int	LTR	ERV1
225	HERV3-int	LTR	ERV1
226	HERV30-int	LTR	ERV1
227	HERV35I-int	LTR	ERV1
228	HERV4_I-int	LTR	ERV1
229	HERV9-int	LTR	ERV1
230	HERVE-int	LTR	ERV1
231	HERVE_a-int	LTR	ERV1
232	HERVFH21-int	LTR	ERV1
233	HERVH-int	LTR	ERV1
234	HERVI-int	LTR	ERV1
235	HERVIP10F-int	LTR	ERV1
236	HERVIP10FH-int	LTR	ERV1
237	HERVK-int	LTR	ERVK
238	HERVK11-int	LTR	ERVK
239	HERVK14-int	LTR	ERVK
240	HERVK3-int	LTR	ERVK

241	HERVK9-int	LTR	ERVK
242	HERVL-int	LTR	ERVL
243	HERVL18-int	LTR	ERVL
244	HERVL40-int	LTR	ERVL
245	HUERS-P1-int	LTR	ERV1
246	HUERS-P2-int	LTR	ERV1
247	HUERS-P3-int	LTR	ERV1
248	HUERS-P3b-int	LTR	ERV1
249	Harlequin-int	LTR	ERV1
250	LOR1-int	LTR	ERV1
251	LOR1a	LTR	ERV1
252	LTR10A	LTR	ERV1
253	LTR10C	LTR	ERV1
254	LTR10F	LTR	ERV1
255	LTR12	LTR	ERV1
256	LTR12B	LTR	ERV1
257	LTR12C	LTR	ERV1
258	LTR12D	LTR	ERV1
259	LTR12E	LTR	ERV1
260	LTR12F	LTR	ERV1
261	LTR12_	LTR	ERV1
262	LTR13	LTR	ERVK
263	LTR13A	LTR	ERVK
264	LTR14	LTR	ERVK
265	LTR14B	LTR	ERVK
266	LTR15	LTR	ERV1
267	LTR16A	LTR	ERVL
268	LTR16A1	LTR	ERVL
269	LTR16B1	LTR	ERVL
270	LTR16B2	LTR	ERVL
271	LTR16C	LTR	ERVL
272	LTR16E1	LTR	ERVL
273	LTR16E2	LTR	ERVL
274	LTR17	LTR	ERV1
275	LTR18B	LTR	ERVL
276	LTR19-int	LTR	ERV1
277	LTR19A	LTR	ERV1
278	LTR1B	LTR	ERV1
279	LTR1C	LTR	ERV1
280	LTR1D	LTR	ERV1
281	LTR2	LTR	ERV1

282	LTR21A	LTR	ERV1
283	LTR22C	LTR	ERVK
284	LTR23	LTR	ERV1
285	LTR24	LTR	ERV1
286	LTR25	LTR	ERV1
287	LTR26B	LTR	ERV1
288	LTR26E	LTR	ERV1
289	LTR27	LTR	ERV1
290	LTR2B	LTR	ERV1
291	LTR2C	LTR	ERV1
292	LTR3	LTR	ERVK
293	LTR30	LTR	ERV1
294	LTR32	LTR	ERVL
295	LTR33	LTR	ERVL
296	LTR33A_	LTR	ERVL
297	LTR35B	LTR	ERV1
298	LTR37A	LTR	ERV1
299	LTR39	LTR	ERV1
300	LTR39-int	LTR	ERV1
301	LTR3A	LTR	ERVK
302	LTR3B	LTR	ERVK
303	LTR3B_	LTR	ERVK
304	LTR4	LTR	ERV1
305	LTR41	LTR	ERVL
306	LTR41B	LTR	ERVL
307	LTR45B	LTR	ERV1
308	LTR46	LTR	ERV1
309	LTR46-int	LTR	ERV1
310	LTR47B	LTR	ERVL
311	LTR48B	LTR	ERV1
312	LTR49-int	LTR	ERV1
313	LTR5	LTR	ERVK
314	LTR50	LTR	ERVL
315	LTR52	LTR	ERVL
316	LTR53	LTR	ERVL
317	LTR54B	LTR	ERV1
318	LTR55	LTR	ERV
319	LTR56	LTR	ERV1
320	LTR57	LTR	ERVL
321	LTR5A	LTR	ERVK
322	LTR5B	LTR	ERVK

323	LTR5_Hs	LTR	ERVK
324	LTR61	LTR	ERV1
325	LTR65	LTR	ERV1
326	LTR66	LTR	ERVL
327	LTR67B	LTR	ERVL
328	LTR6A	LTR	ERV1
329	LTR6B	LTR	ERV1
330	LTR7	LTR	ERV1
331	LTR73	LTR	ERV1
332	LTR78	LTR	ERV1
333	LTR79	LTR	ERVL
334	LTR7B	LTR	ERV1
335	LTR7C	LTR	ERV1
336	LTR8	LTR	ERV1
337	LTR80B	LTR	ERVL
338	LTR81A	LTR	Gypsy
339	LTR82A	LTR	ERVL
340	LTR84b	LTR	ERVL
341	LTR85a	LTR	Gypsy?
342	LTR85b	LTR	Gypsy?
343	LTR86A2	LTR	ERVL
344	LTR87	LTR	ERVL?
345	LTR89	LTR	ERVL?
346	LTR8A	LTR	ERV1
347	LTR9	LTR	ERV1
348	LTR90B	LTR	LTR
349	LTR9B	LTR	ERV1
350	MER101-int	LTR	ERV1
351	MER11A	LTR	ERVK
352	MER11B	LTR	ERVK
353	MER11C	LTR	ERVK
354	MER11D	LTR	ERVK
355	MER21-int	LTR	ERVL
356	MER21A	LTR	ERVL
357	MER21B	LTR	ERVL
358	MER21C	LTR	ERVL
359	MER31-int	LTR	ERV1
360	MER31A	LTR	ERV1
361	MER31B	LTR	ERV1
362	MER34B-int	LTR	ERV1
363	MER4-int	LTR	ERV1

364	MER41-int	LTR	ERV1
365	MER41A	LTR	ERV1
366	MER41B	LTR	ERV1
367	MER41G	LTR	ERV1
368	MER49	LTR	ERV1
369	MER4A	LTR	ERV1
370	MER4A1	LTR	ERV1
371	MER4B	LTR	ERV1
372	MER4C	LTR	ERV1
373	MER4D	LTR	ERV1
374	MER4D0	LTR	ERV1
375	MER4D1	LTR	ERV1
376	MER4E	LTR	ERV1
377	MER4E1	LTR	ERV1
378	MER50	LTR	ERV1
379	MER50-int	LTR	ERV1
380	MER51-int	LTR	ERV1
381	MER51A	LTR	ERV1
382	MER51B	LTR	ERV1
383	MER51C	LTR	ERV1
384	MER51E	LTR	ERV1
385	MER52-int	LTR	ERV1
386	MER52A	LTR	ERV1
387	MER57-int	LTR	ERV1
388	MER57A-int	LTR	ERV1
389	MER57A1	LTR	ERV1
390	MER57B1	LTR	ERV1
391	MER57B2	LTR	ERV1
392	MER57F	LTR	ERV1
393	MER61-int	LTR	ERV1
394	MER61A	LTR	ERV1
395	MER61B	LTR	ERV1
396	MER65-int	LTR	ERV1
397	MER65A	LTR	ERV1
398	MER65D	LTR	ERV1
399	MER66-int	LTR	ERV1
400	MER66B	LTR	ERV1
401	MER66C	LTR	ERV1
402	MER67A	LTR	ERV1
403	MER67D	LTR	ERV1
404	MER68	LTR	ERVL

405	MER77	LTR	ERVL
406	MER77B	LTR	ERVL
407	MER83C	LTR	ERV1
408	MER87	LTR	ERV1
409	MER87B	LTR	ERV1
410	MER9a2	LTR	ERVK
411	MER9a3	LTR	ERVK
412	MLT1-int	LTR	ERVL-MaLR
413	MLT1A	LTR	ERVL-MaLR
414	MLT1A0	LTR	ERVL-MaLR
415	MLT1A0-int	LTR	ERVL-MaLR
416	MLT1A1	LTR	ERVL-MaLR
417	MLT1B	LTR	ERVL-MaLR
418	MLT1B-int	LTR	ERVL-MaLR
419	MLT1C	LTR	ERVL-MaLR
420	MLT1D	LTR	ERVL-MaLR
421	MLT1E1	LTR	ERVL-MaLR
422	MLT1E1A	LTR	ERVL-MaLR
423	MLT1E2	LTR	ERVL-MaLR
424	MLT1E3	LTR	ERVL-MaLR
425	MLT1E3-int	LTR	ERVL-MaLR
426	MLT1F	LTR	ERVL-MaLR
427	MLT1F1	LTR	ERVL-MaLR
428	MLT1F2	LTR	ERVL-MaLR
429	MLT1F2-int	LTR	ERVL-MaLR
430	MLT1G	LTR	ERVL-MaLR
431	MLT1G1	LTR	ERVL-MaLR
432	MLT1G3	LTR	ERVL-MaLR
433	MLT1G3-int	LTR	ERVL-MaLR
434	MLT1H	LTR	ERVL-MaLR
435	MLT1H1	LTR	ERVL-MaLR
436	MLT1H1-int	LTR	ERVL-MaLR
437	MLT1H2	LTR	ERVL-MaLR
438	MLT1I	LTR	ERVL-MaLR
439	MLT1J	LTR	ERVL-MaLR
440	MLT1J-int	LTR	ERVL-MaLR
441	MLT1J1	LTR	ERVL-MaLR
442	MLT1J2	LTR	ERVL-MaLR
443	MLT1K	LTR	ERVL-MaLR
444	MLT1L	LTR	ERVL-MaLR
445	MLT1M	LTR	ERVL-MaLR

446	MLT1N2	LTR	ERVL-MaLR
447	MLT2A1	LTR	ERVL
448	MLT2A2	LTR	ERVL
449	MLT2B2	LTR	ERVL
450	MLT2B3	LTR	ERVL
451	MLT2B4	LTR	ERVL
452	MLT2C1	LTR	ERVL
453	MLT2C2	LTR	ERVL
454	MLT2D	LTR	ERVL
455	MST-int	LTR	ERVL-MaLR
456	MSTA	LTR	ERVL-MaLR
457	MSTA-int	LTR	ERVL-MaLR
458	MSTB	LTR	ERVL-MaLR
459	MSTB-int	LTR	ERVL-MaLR
460	MSTB1	LTR	ERVL-MaLR
461	MSTC	LTR	ERVL-MaLR
462	MSTD	LTR	ERVL-MaLR
463	MamGyp-int	LTR	Gypsy
464	MamGypLTR1a	LTR	Gypsy
465	MamGypLTR1c	LTR	Gypsy
466	MamGypLTR2b	LTR	Gypsy
467	MamGypLTR3	LTR	Gypsy
468	MamRep1527	LTR	LTR
469	PABL_A	LTR	ERV1
470	PABL_B	LTR	ERV1
471	PRIMA4-int	LTR	ERV1
472	PRIMA41-int	LTR	ERV1
473	PRIMAX-int	LTR	ERV1
474	THE1-int	LTR	ERVL-MaLR
475	THE1A	LTR	ERVL-MaLR
476	THE1A-int	LTR	ERVL-MaLR
477	THE1B	LTR	ERVL-MaLR
478	THE1B-int	LTR	ERVL-MaLR
479	THE1C	LTR	ERVL-MaLR
480	THE1C-int	LTR	ERVL-MaLR
481	THE1D	LTR	ERVL-MaLR
482	THE1D-int	LTR	ERVL-MaLR
483	A-rich	Low_complexity	Low_complexity
484	AT_rich	Low_complexity	Low_complexity
485	C-rich	Low_complexity	Low_complexity
486	CT-rich	Low_complexity	Low_complexity

487	G-rich	Low_complexity	Low_complexity
488	GA-rich	Low_complexity	Low_complexity
489	GC_rich	Low_complexity	Low_complexity
490	T-rich	Low_complexity	Low_complexity
491	polypurine	Low_complexity	Low_complexity
492	polypyrimidine	Low_complexity	Low_complexity
493	SVA_A	Other	Other
494	SVA_B	Other	Other
495	SVA_C	Other	Other
496	SVA_D	Other	Other
497	SVA_E	Other	Other
498	SVA_F	Other	Other
499	Helitron2Na_Mam	RC	Helitron?
500	Helitron3Na_Mam	RC	Helitron
501	7SK	RNA	RNA
502	AluJb	SINE	Alu
503	AluJo	SINE	Alu
504	AluJr	SINE	Alu
505	AluJr4	SINE	Alu
506	AluSc	SINE	Alu
507	AluSc5	SINE	Alu
508	AluSc8	SINE	Alu
509	AluSg	SINE	Alu
510	AluSg4	SINE	Alu
511	AluSg7	SINE	Alu
512	AluSp	SINE	Alu
513	AluSq	SINE	Alu
514	AluSq10	SINE	Alu
515	AluSq2	SINE	Alu
516	AluSq4	SINE	Alu
517	AluSx	SINE	Alu
518	AluSx1	SINE	Alu
519	AluSx3	SINE	Alu
520	AluSx4	SINE	Alu
521	AluSz	SINE	Alu
522	AluSz6	SINE	Alu
523	AluY	SINE	Alu
524	AluYa5	SINE	Alu
525	AluYa8	SINE	Alu
526	AluYb8	SINE	Alu
527	AluYc	SINE	Alu

528	AluYc3	SINE	Alu
529	AluYd8	SINE	Alu
530	AluYf4	SINE	Alu
531	AluYk11	SINE	Alu
532	AluYk4	SINE	Alu
533	FAM	SINE	Alu
534	FLAM_A	SINE	Alu
535	FLAM_C	SINE	Alu
536	FRAM	SINE	Alu
537	MIR	SINE	MIR
538	MIR3	SINE	MIR
539	MIRb	SINE	MIR
540	MIRc	SINE	MIR
541	MamSINE1	SINE	tRNA
542	(CATTC)n	Satellite	Satellite
543	(GAATG)n	Satellite	Satellite
544	ACRO1	Satellite	acro
545	ALR/Alpha	Satellite	centr
546	BSR/Beta	Satellite	Satellite
547	CER	Satellite	Satellite
548	GSATII	Satellite	centr
549	HSAT5	Satellite	Satellite
550	HSATI	Satellite	Satellite
551	HSATII	Satellite	Satellite
552	MSR1	Satellite	Satellite
553	SAR	Satellite	Satellite
554	SATR1	Satellite	Satellite
555	TAR1	Satellite	telo
556	(A)n	Simple_repeat	Simple_repeat
557	(AGGGGG)n	Simple_repeat	Simple_repeat
558	(ATAGG)n	Simple_repeat	Simple_repeat
559	(ATG)n	Simple_repeat	Simple_repeat
560	(ATTG)n	Simple_repeat	Simple_repeat
561	(C)n	Simple_repeat	Simple_repeat
562	(CA)n	Simple_repeat	Simple_repeat
563	(CAA)n	Simple_repeat	Simple_repeat
564	(CAAA)n	Simple_repeat	Simple_repeat
565	(CAAAA)n	Simple_repeat	Simple_repeat
566	(CAAAAA)n	Simple_repeat	Simple_repeat
567	(CACCAT)n	Simple_repeat	Simple_repeat
568	(CACCC)n	Simple_repeat	Simple_repeat

569	(CACG)n	Simple_repeat	Simple_repeat
570	(CACTC)n	Simple_repeat	Simple_repeat
571	(CAG)n	Simple_repeat	Simple_repeat
572	(CAGA)n	Simple_repeat	Simple_repeat
573	(CAGAGA)n	Simple_repeat	Simple_repeat
574	(CAGG)n	Simple_repeat	Simple_repeat
575	(CAGGG)n	Simple_repeat	Simple_repeat
576	(CAT)n	Simple_repeat	Simple_repeat
577	(CATATA)n	Simple_repeat	Simple_repeat
578	(CATTA)n	Simple_repeat	Simple_repeat
579	(CATTC)n	Simple_repeat	Simple_repeat
580	(CCA)n	Simple_repeat	Simple_repeat
581	(CCAA)n	Simple_repeat	Simple_repeat
582	(CCCA)n	Simple_repeat	Simple_repeat
583	(CCCCAG)n	Simple_repeat	Simple_repeat
584	(CCCCCA)n	Simple_repeat	Simple_repeat
585	(CCCCCG)n	Simple_repeat	Simple_repeat
586	(CCCCCT)n	Simple_repeat	Simple_repeat
587	(CCCCG)n	Simple_repeat	Simple_repeat
588	(CCCG)n	Simple_repeat	Simple_repeat
589	(CCCTAA)n	Simple_repeat	Simple_repeat
590	(CCG)n	Simple_repeat	Simple_repeat
591	(CCGCG)n	Simple_repeat	Simple_repeat
592	(CCGGG)n	Simple_repeat	Simple_repeat
593	(CCTG)n	Simple_repeat	Simple_repeat
594	(CG)n	Simple_repeat	Simple_repeat
595	(CGA)n	Simple_repeat	Simple_repeat
596	(CGAG)n	Simple_repeat	Simple_repeat
597	(CGCGG)n	Simple_repeat	Simple_repeat
598	(CGG)n	Simple_repeat	Simple_repeat
599	(CGGA)n	Simple_repeat	Simple_repeat
600	(CGGG)n	Simple_repeat	Simple_repeat
601	(CGGGG)n	Simple_repeat	Simple_repeat
602	(CGGGGG)n	Simple_repeat	Simple_repeat
603	(CTCG)n	Simple_repeat	Simple_repeat
604	(CTG)n	Simple_repeat	Simple_repeat
605	(CTGGGG)n	Simple_repeat	Simple_repeat
606	(G)n	Simple_repeat	Simple_repeat
607	(GA)n	Simple_repeat	Simple_repeat
608	(GAA)n	Simple_repeat	Simple_repeat
609	(GAAA)n	Simple_repeat	Simple_repeat

610	(GAAAA)n	Simple_repeat	Simple_repeat
611	(GAATG)n	Simple_repeat	Simple_repeat
612	(GAGTG)n	Simple_repeat	Simple_repeat
613	(GGA)n	Simple_repeat	Simple_repeat
614	(GGAA)n	Simple_repeat	Simple_repeat
615	(GGAAA)n	Simple_repeat	Simple_repeat
616	(GGCTG)n	Simple_repeat	Simple_repeat
617	(GGGA)n	Simple_repeat	Simple_repeat
618	(GGGAA)n	Simple_repeat	Simple_repeat
619	(GGGAGA)n	Simple_repeat	Simple_repeat
620	(GGGGA)n	Simple_repeat	Simple_repeat
621	(GGGTG)n	Simple_repeat	Simple_repeat
622	(T)n	Simple_repeat	Simple_repeat
623	(TA)n	Simple_repeat	Simple_repeat
624	(TAA)n	Simple_repeat	Simple_repeat
625	(TAAA)n	Simple_repeat	Simple_repeat
626	(TAAAA)n	Simple_repeat	Simple_repeat
627	(TAATG)n	Simple_repeat	Simple_repeat
628	(TAGA)n	Simple_repeat	Simple_repeat
629	(TAGG)n	Simple_repeat	Simple_repeat
630	(TC)n	Simple_repeat	Simple_repeat
631	(TCC)n	Simple_repeat	Simple_repeat
632	(TCCA)n	Simple_repeat	Simple_repeat
633	(TCCC)n	Simple_repeat	Simple_repeat
634	(TCCCC)n	Simple_repeat	Simple_repeat
635	(TCCCG)n	Simple_repeat	Simple_repeat
636	(TCTCTG)n	Simple_repeat	Simple_repeat
637	(TCTG)n	Simple_repeat	Simple_repeat
638	(TG)n	Simple_repeat	Simple_repeat
639	(TGAA)n	Simple_repeat	Simple_repeat
640	(TGAG)n	Simple_repeat	Simple_repeat
641	(TGG)n	Simple_repeat	Simple_repeat
642	(TGGA)n	Simple_repeat	Simple_repeat
643	(TGGGGG)n	Simple_repeat	Simple_repeat
644	(TTA)n	Simple_repeat	Simple_repeat
645	(TTATA)n	Simple_repeat	Simple_repeat
646	(TTATG)n	Simple_repeat	Simple_repeat
647	(TTC)n	Simple_repeat	Simple_repeat
648	(TTCA)n	Simple_repeat	Simple_repeat
649	(TTCC)n	Simple_repeat	Simple_repeat
650	(TTG)n	Simple_repeat	Simple_repeat

651	(TTGG)n	Simple_repeat	Simple_repeat
652	(TTTA)n	Simple_repeat	Simple_repeat
653	(TTTAA)n	Simple_repeat	Simple_repeat
654	(TTTC)n	Simple_repeat	Simple_repeat
655	(TTTG)n	Simple_repeat	Simple_repeat
656	(TTTTA)n	Simple_repeat	Simple_repeat
657	(TTTTC)n	Simple_repeat	Simple_repeat
658	(TTTTG)n	Simple_repeat	Simple_repeat
659	(TTTTTA)n	Simple_repeat	Simple_repeat
660	(TTTTTG)n	Simple_repeat	Simple_repeat
661	MamRep605	Unknown	Unknown
662	UCON26	Unknown	Unknown
663	UCON28a	Unknown	Unknown
664	UCON6	Unknown	Unknown
665	5S	rRNA	rRNA
666	LSU-rRNA_Hsa	rRNA	rRNA
667	SSU-rRNA_Hsa	rRNA	rRNA
668	HY3	scRNA	scRNA
669	HY4	scRNA	scRNA
670	U1	snRNA	snRNA
671	U14	snRNA	snRNA
672	U2	snRNA	snRNA
673	U3	snRNA	snRNA
674	U4	snRNA	snRNA
675	U5	snRNA	snRNA
676	U6	snRNA	snRNA
677	U8	snRNA	snRNA
678	7SLRNA	srpRNA	srpRNA
679	tRNA-Arg-CGG	tRNA	tRNA
680	tRNA-Asn-AAC	tRNA	tRNA
681	tRNA-Gln-CAG	tRNA	tRNA
682	tRNA-Glu-GAG_	tRNA	tRNA
683	tRNA-Gly-GGA	tRNA	tRNA
684	tRNA-Leu-CTY	tRNA	tRNA
685	tRNA-Leu-TTG	tRNA	tRNA
686	tRNA-Lys-AAA	tRNA	tRNA
687	tRNA-Lys-AAG	tRNA	tRNA
688	tRNA-Pro-CCG	tRNA	tRNA
689	tRNA-SeC(e)-TGA	tRNA	tRNA
690	tRNA-Thr-ACY	tRNA	tRNA
