# Supplementary material for: Massively Parallel Sequencing of Human Urinary Exosome/Microvesicle RNA Reveals a Predominance of Non-Coding RNA
Source: PLoS One. 2014 May 9;9(5):e96094. doi: 10.1371/journal.pone.0096094 (PMC4015934; doi:10.1371/journal.pone.0096094)
Supplement: Table S3 — Known ncRNA found in microvesicles (including exosomes) (+DNase). Listing of the 196 ncRNA transcripts (rnaDB set = "curated from literature") and the number of microvesicle derived sequencing transcripts that aligned against them. (ZIP) [file pone.0096094.s003.zip › PLOS Russo Supp Table S3 Final 040814.pdf]

Supp. Table S3: Known ncRNA found in microvesicles (including exosomes) (+DNase)

Listing of the 196 ncRNA transcripts (rnaDB set = "curated from literature") and the number of microvesicle derived sequencing transcripts that aligned against them

count	No. Reads aligning	rnaDB ID	rnaDB definition	Accession ID	Pubmed ID
1	855908	LIT1626	Homo sapiens non-coding chimeric transcript hc9(5)h-2-1/4 (UM 9(5))	AY072609	12515386
2	69064	LIT1868	Human 7S L gene, complete	M20910	6802847, 6181418, 3403542, 6084597
3	2722	LIT3330	Homo sapiens ret finger protein-like 3 antisense (RFPL3S) on chromosome 22.	NR_001450	10508838
4	2505	LIT3569	Homo sapiens mRNA sequence	AY927568	15790800
5	2484	LIT3497	Homo sapiens mRNA sequence	AY927481	15790800
6	1668	LIT1867	Homo sapiens 7S RNA	V00477	6802847, 6181418, 3403542, 6084597
7	900	LIT2062	Homo sapiens ribosomal protein, large, P0 pseudogene 2 (RPLP0P2), misc RNA	XR_000076	8486366
8	621	LIT1834	Homo sapiens non-small cell lung carcinoma noncoding RNA, partial sequence	AY166681	15254752
9	520	LIT2056	Homo sapiens EMX2OS mRNA, complete sequence.	AY117413	12573261
10	505	LIT2059	Homo sapiens general transcription factor II, i, pseudogene 1 (GTF2IP1), misc RNA	XR_000139	9466987
11	471	LIT3583	Homo sapiens mRNA sequence	AY927585	15790800
12	466	LIT3571	Homo sapiens mRNA sequence	AY927570	15790800
13	363	LIT3612	Homo sapiens mRNA sequence	AY927624	15790800

14	331	LIT1869	Homo sapiens RNA, 7SK, nuclear (RN7SK) on chromosome 6	NR_001445	11713533, 11713532, 1646389, 9018369, 6434539
15	327	LIT3420	Homo sapiens candidate tumor suppressor (DLEU2) RNA, antisense sequence	AY455757	11264177, 15188451
16	315	LIT3355	Homo sapiens, clone IMAGE:4391558, mRNA	BC017743	16607615
17	277	LIT3418	Psoriasis-susceptibility Related RNA Gene Induced by Stress (PRINS) RNA	AK022045	15855153
18	277	LIT2089	Homo sapiens clone BAC 33J7 diphosphoinositol polyphosphate phosphohydrolase type 2 (NUDT4) pseudogene, mRNA sequence.	AF311215	11376937
19	267	LIT3537	Homo sapiens mRNA sequence	AY927529	15790800
20	264	LIT2045	Homo sapiens RET finger protein-like 1 antisense transcript (RFPL1S), partial.	AJ010230	10508838
21	256	LIT3319	Homo sapiens non-coding RNA in rhabdomyosarcoma (RMS) (NCRMS), misc RNA	XR_000219	12082533, 15872005
22	210	LIT2197	Human SNRPN mRNA, 3 prime UTR, partial sequence incorporating IC-SNURF-SNRPN transcript noncoding exons 10a, 11 and 12	U81001	9070929
23	204	LIT2019	Human Ro RNA (scRNA) hY3 from small cytoplasmic ribonucleoprotein particles.	K01563	7505620, 6201849
24	190	LIT2112	Human aldolase pseudogene mRNA, complete cds.	M21191	3674018
25	165	LIT2014	Human gene hY1 encoding a cytoplasmic Ro RNA.	V00584	7505620, 6187471
26	158	LIT1721	Human BC200 scRNA	U01305	9605471, 9131672, 9858834; 9422992; 7684772; 9847409
27	154	LIT2102	Human destrin-2 pseudogene mRNA, complete cds.	U72518	9074930
28	151	LIT3500	Homo sapiens mRNA sequence	AY927484	15790800

29	145	LIT2232	Homo sapiens TTTY15 from cDNA DKFZp434I143 (from clone DKFZp434I143).	AL080135	10507722, 12815422
30	137	LIT2121	Homo sapiens noncoding RNA GA3824 implicated in autism	CB338058	12690205
31	133	LIT3356	Homo sapiens cDNA clone IMAGE:5294683	BC043430	16607615
32	132	LIT2101	Human RPL13-2 pseudogene mRNA, complete cds.	U72513	9074930
33	125	LIT3623	Homo sapiens FMR1P binding RNA, partial sequence	AF040097	10973830
34	113	LIT1956	Homo sapiens partial BCMS gene for B-cell neoplasia associated transcript, exon 32	AJ412006	11406609
35	106	LIT1548	Human NTT gene	U54776	9027504
36	105	LIT1751	Homo sapiens 1 DISC2 gene, complete sequence	AF222981	10814723
37	101	LIT3364	Homo sapiens oncomir-1 transcript (C13orf25 isoform 1) mRNA	AB176708	15944707
38	92	LIT2090	Homo sapiens clone BAC 608B23 diphosphoinositol polyphosphate phosphohydrolase type 2 (NUDT4) pseudogene, mRNA sequence.	AF311216	11376937
39	91	LIT1821	Homo sapiens MESTIT1 antisense RNA, partial sequence	AF482998	12095916, 11821432
40	90	LIT2191	Homo sapiens HERC2P3/D15F37 pseudogene, S4 allele, mRNA sequence.	AF041081	9949213
41	85	LIT1094	Homo sapiens U50HG RNA	AB017710	10792466
42	83	LIT2065	Homo sapiens protocadherin-beta (PCDHpsi-5) pseudogene, mRNA sequence.	AF329369	11322959
43	81	LIT1267	Homo sapiens growth arrest specific transcript 5 gene, exons 1 through 12 and alternatively spliced mRNA sequences	AF141346	9819378
44	77	LIT2021	Human hy4 Ro RNA (associated with erythrocyte Ro RNPs).	X57566	8863726, 1698620

45	75	LIT1750	Human unknown protein mRNA within the p53 intron 1, complete cds	U58658	8975713
46	74	LIT1553	Homo sapiens non-coding RNA DD3 gene, exons 2, 3, and 4	AF103908	10606244
47	60	LIT2013	Homo sapiens metastasis associated in lung adenocarcinoma transcript 1 short isoform, transcribed non-coding RNA, complete sequence.	BK001411	12970751, 16878148
48	58	LIT3492	Homo sapiens mRNA sequence	AY927476	15790800
49	54	LIT2100	Homo sapiens beta-glucuronidase-like protein (5G4) pseudogene mRNA, partial sequence.	U21555	7552146
50	50	LIT1319	Homo sapiens mRNA containing U19H snoRNA; mRNA 2	AJ224167	9630250, 8657112
51	47	LIT3354	Human U1 small nuclear RNA (snRNA) gene HU1-1	J00318	6180116, 12389039, 16115885
52	47	LIT1011	Homo sapiens Jpx noncoding transcript	AV714079	12045143
53	46	LIT3532	Homo sapiens mRNA sequence	AY927522	15790800
54	44	LIT3426	Homo sapiens taurine upregulated gene 1 (TUG1), predicted sequence based on ESTs	AF279660	15797018
55	44	LIT2225	Homo sapiens IC-SNURF-SNRPN transcript noncoding exons 36-40, 42-44, 46-48, and 50-61	AF400487	11726556
56	40	LIT3490	Homo sapiens mRNA sequence	AY927473	15790800
57	37	LIT3414	Homo sapiens Pax6 opposite strand (Pax6OS) RNA, variant isoform	BX648962	15703187
58	37	LIT1973	Homo sapiens partial BCMS gene for B-cell neoplasia associated transcript, exon 18	AJ411992	11406609
59	34	LIT3551	Homo sapiens mRNA sequence	AY927545	15790800
60	34	LIT2070	Homo sapiens HERC2P7 pseudogene, partial mRNA sequence.	AF071178	9949213

61	34	LIT2063	Homo sapiens C2H2 zinc finger protein pseudogene, mRNA sequence.	AF033199	9628832
62	34	LIT1758	H.sapiens MRP RNA gene encoding the RNA component of RNase MRP (RMRP)	X51867	11207361, 1690392
63	34	LIT1316	Homo sapiens RNA transcript from U17 small nucleolar RNA host gene, variant U17HG-A	AJ006834	9671460
64	34	LIT1275	Homo sapiens DLEU1 noncoding transcript (BCMS)	AF279660	11264177
65	33	LIT3372	Homo sapiens U87HG mRNA, complete sequence	AY264285	16226852
66	32	LIT2060	Homo sapiens Shwachman-Bodian-Diamond syndrome pseudogene (SBDSP), mRNA (cDNA clone IMAGE:4329436)	BC010183	12496757
67	31	LIT3609	Homo sapiens mRNA sequence	AY927621	15790800
68	31	LIT1584	Homo sapiens RNA for differentiation or sex determination (CMPD)	D43770	8789441
69	30	LIT3470	Homo sapiens mRNA sequence	AY927437	15998900
70	28	LIT2044	Homo sapiens topoisomerase (DNA) I pseudogene 2 (TOP1P2), misc RNA	M55630	2170234
71	27	LIT3549	Homo sapiens mRNA sequence	AY927543	15790800
72	27	LIT3477	Homo sapiens mRNA sequence	AY927449	15998900
73	27	LIT3337	Homo sapiens human accelarated region forward mRNA, complete sequence	DQ860409	16915236
74	26	LIT1998	Homo sapiens partial BCMS gene for B-cell neoplasia associated transcript, exon 4a	AJ411977	11406609
75	25	LIT1596	Homo sapiens X (inactive)-specific transcript (XIST) complete exon	M97168	11734999, 2034278, 10723727, 2034279, 1423610, 1985261, 1423611, 11290427

76	24	LIT3316	Homo sapiens myosin XVB, pseudogene, mRNA (cDNA clone MGC:34148 IMAGE:5205535), complete cds.	BC027875	11964073
77	22	LIT1826	Human 2G7 gene, complete sequence	U62668	8968746
78	21	LIT1318	Homo sapiens mRNA containing U19H snoRNA; mRNA 1	AJ224166	9630250, 8657112
79	20	LIT3580	Homo sapiens mRNA sequence	AY927581	15790800
80	20	LIT2116	Homo sapiens SZ-1 mRNA (PSZA11q14), complete sequence	AF525782	13130513
81	20	LIT2113	Human cytochrome b5 pseudogene mRNA.	M64714	1712589
82	19	LIT1912	Homo sapiens mRNA for B-cell neoplasia associated transcript, (BCMS gene), splice variant K, non coding transcript	AJ412033	11406609
83	19	LIT1320	Homo sapiens mRNA containing U19H snoRNA; mRNA 3	AJ224168	9630250, 8657112
84	18	LIT2233	Homo sapiens CYorf14 from PRO2834 mRNA, complete cds.	AF119903	12815422
85	18	LIT1752	Homo sapiens ncR-uPAR transcript	AF222981	12084570
86	18	LIT1007	Homo sapiens Ftx noncoding RNA	AK057701	12045143
87	17	LIT2214	Homo sapiens IC-SNURF-SNRPN transcript noncoding exons 106-144 and 146	AF400501	11726556
88	16	LIT3598	Homo sapiens mRNA sequence	AY927608	15790800
89	15	LIT3550	Homo sapiens mRNA sequence	AY927544	15790800
90	15	LIT3514	Homo sapiens mRNA sequence	AY927500	15790800
91	15	LIT3336	Homo sapiens urothelial cancer associated 1 (UCA1) mRNA, complete sequence	DQ343132	16914600
92	14	LIT3496	Homo sapiens mRNA sequence	AY927480	15790800

93	14	LIT3447	Homo sapiens SLC4A9 noncoding transcript (SLC4A9N) from NCI_CGAP_Kid11 cDNA clone IMAGE:2384256	AI830440	11305939
94	14	LIT1251	Homo sapiens steroid receptor RNA activator isoform 3 mRNA, complete cds	AF293026	12444263, 10199399, 14517287, 15147866, 16848684
95	14	LIT1000	Homo sapiens COPG2IT1 noncoding transcript		10995575
96	12	LIT3533	Homo sapiens mRNA sequence	AY927524	15790800
97	12	LIT3374	Homo sapiens noncoding repressor of NFAT (NRON) RNA, exon 3	AY264286	16141075
98	11	LIT3471	Homo sapiens mRNA sequence	AY927440	15998900
99	11	LIT2190	Homo sapiens HERC2P2/D15F37 pseudogene, S3 allele, mRNA sequence	AF041080	9949213
100	11	LIT1321	Homo sapiens mRNA containing U19H snoRNA; mRNA 4	AJ224169	9630250, 8657112
101	9	LIT3624	Homo sapiens FMR1P binding RNA, partial sequence	AF040098	10973830
102	9	LIT3526	Homo sapiens mRNA sequence	AY927516	15790800
103	9	LIT1997	Homo sapiens partial BCMS gene for B-cell neoplasia associated transcript, exon 5	AJ411978	11406609
104	8	LIT3575	Homo sapiens mRNA sequence	AY927575	15790800
105	8	LIT3524	Homo sapiens mRNA sequence	AY927514	15790800
106	8	LIT2203	Homo sapiens IC-SNURF-SNRPN transcript noncoding exons 12, 13d, 14, 15, 16, 17, 18, 19 and 20	AF319524	9070929, 11159938
107	7	LIT3563	Homo sapiens mRNA sequence	AY927560	15790800
108	7	LIT3495	Homo sapiens mRNA sequence	AY927479	15790800
109	7	LIT2032	Homo sapiens vault-associated RNA 1, complete sequence	AF045143	9535882, 16150923

110	7	LIT1704	Human small cellular 7 SK mRNA	X04236	6196783, 9018369, 11713533, 11713532, 6434539, 9018369
111	7	LIT1625	Homo sapiens KVLQT1 gene	AJ006345	10024302
112	6	LIT3574	Homo sapiens mRNA sequence	AY927573	15790800
113	6	LIT3570	Homo sapiens mRNA sequence	AY927569	15790800
114	6	LIT2209	Homo sapiens IC-SNURF-SNRPN transcript noncoding exons 22-37, 40, 42-44, and 46-58	AF400486	11726556
115	6	LIT2077	Homo sapiens TRIpartite motif protein pseudogene mRNA sequence.	AF230412	11331580
116	6	LIT2054	Homo sapiens olfactory receptor, family 7, subfamily E, member 24 pseudogene(OR7E24P), misc RNA	XR_000082	12727898
117	6	LIT1674	Homo sapiens IPW mRNA sequence	U12897	7849716, 9063754, 11726556
118	6	LIT1386	Homo sapiens spliced UHG RNA	L36587	7985025
119	6	LIT1227	H.sapiens predicted non coding cDNA (DGCR5)	X91348	8659529
120	5	LIT3587	Homo sapiens mRNA sequence	AY927593	15790800
121	5	LIT3566	Homo sapiens mRNA sequence	AY927564	15790800
122	5	LIT3530	Homo sapiens mRNA sequence	AY927520	15790800
123	5	LIT3320	Homo sapiens SCA8 mRNA, repeat region.	AF126749	10888605, 10192387
124	5	LIT2050	Homo sapiens COX11 homolog, cytochrome c oxidase assembly protein (yeast) pseudogene (COX11P), misc RNA	NR_000031	12631698
125	5	LIT1702	Homo sapiens hypoxia inducible factor (aHIF) antisense RNA sequence	U85044	9923855, 14744852, 12459261
126	5	LIT1110	Homo sapiens PAR5 gene, complete sequence.	AF019618	7987392
127	4	LIT3614	Homo sapiens mRNA sequence	AY927628	15790800

128	4	LIT3611	Homo sapiens mRNA sequence	AY927623	15790800
129	4	LIT3610	Homo sapiens mRNA sequence	AY927622	15790800
130	4	LIT3601	Homo sapiens mRNA sequence	AY927613	15790800
131	4	LIT3357	Homo sapiens heat shock RNA-1 (HSR-1)	BC043430	16554823
132	4	LIT2224	Homo sapiens IC-SNURF-SNRPN transcript noncoding exons 33, 34, 34.2, 35, and 36	AY421730	11726556
133	4	LIT2117	Homo sapiens telomerase RNA component (TERC) on chromosome 3	NR_001566	7544491, 10591218, 10721988, 11574891, 12514136, 12525685
134	4	LIT2088	Homo sapiens olfactory receptor-like (PJCG9) pseudogene mRNA, partial sequence.	AF359419	11705801
135	4	LIT2057	Homo sapiens proliferating cell nuclear antigen (PCNA) antisense	AL121924	10488129
136	4	LIT1662	Homo sapiens UM 9(5)h non-coding transcript mRNA sequence	BK000166	12515386
137	4	LIT1581	Homo sapiens homeo box A11, antisense (Hoxa11as), misc RNA	XR_000158	12050232
138	4	LIT1554	Homo sapiens non-coding RNA DD3, transcript III	AF103908	10606244
139	3	LIT3593	Homo sapiens mRNA sequence	AY927602	15790800
140	3	LIT3548	Homo sapiens mRNA sequence	AY927542	15790800
141	3	LIT3534	Homo sapiens mRNA sequence	AY927525	15790800
142	3	LIT3516	Homo sapiens mRNA sequence	AY927503	15790800
143	3	LIT3363	Homo sapiens oncomir-1 transcript (C13orf25 isoform 2) mRNA	AB176707	15944707
144	3	LIT2111	H.sapiens mRNA for cAMP dependent proteinkinase PRKX, pseudogene.	Y10483	9154127
145	3	LIT2024	Human Ro RNA (scRNA) hY5 from small cytoplasmic ribonucleoprotein particles.	K01564	8863726, 7529149, 6201849

146	3	LIT2006	Homo sapiens ST7OT1 mRNA, non-coding transcript	AF400039	12213198, 12690205
147	3	LIT1610	Homo sapiens H19 gene, complete sequence	AF125183	1688465, 10666466, 12140686, 11813134, 12490325, 12419837, 11794466, 1811930, 11726548
148	3	LIT1561	Homo sapiens non-coding RNA DD3, transcript I	AF103908	10606244
149	3	LIT1245	Homo sapiens steroid receptor RNA activator 1 (SRA1), misc RNA	XR_000132	12444263, 10199399, 14517287, 15147866, 16848684
150	2	LIT3552	Homo sapiens mRNA sequence	AY927546	15790800
151	2	LIT3498	Homo sapiens mRNA sequence	AY927482	15790800
152	2	LIT2213	Homo sapiens IC-SNURF-SNRPN transcript noncoding exons 44-48 and 50-61	AF400488	11726556
153	2	LIT2099	H.sapiens mRNA for olfactory receptor expressed pseudogene, poly A site.	X87825	8647456
154	2	LIT2058	Homo sapiens hair and skin epidermal-type 12-lipoxygenase-related protein (ALOX12E) mRNA, complete pseudogene sequence.	AF020774	9691181
155	2	LIT2038	Human beta globin intergenic transcript 2 from Human beta globin region on chromosome 11	U01317	9334315, 10882078
156	2	LIT1980	Homo sapiens ST7 overlapping transcript 4, mRNA sequence	BM413625	12213198, 12690205
157	2	LIT1905	Homo sapiens mRNA for B-cell neoplasia associated transcript, (BCMS gene), splice variant R, non coding transcript	AJ412040	11406609
158	2	LIT1899	Homo sapiens clone IMAGE:1409652 ST7OT2 mRNA, non-coding transcript	AF400045	12213198, 12690205

159	2	LIT1837	Homo sapiens Tmevpg, mRNA sequence	BF510189	12719555, 11735227
160	2	LIT1568	Homo sapiens clone J HOXA 11 antisense RNA	AF071167	12050232
161	1	LIT3604	Homo sapiens mRNA sequence	AY927616	15790800
162	1	LIT3586	Homo sapiens mRNA sequence	AY927592	15790800
163	1	LIT3582	Homo sapiens mRNA sequence	AY927584	15790800
164	1	LIT3578	Homo sapiens mRNA sequence	AY927579	15790800
165	1	LIT3572	Homo sapiens mRNA sequence	AY927571	15790800
166	1	LIT3567	Homo sapiens mRNA sequence	AY927566	15790800
167	1	LIT3543	Homo sapiens mRNA sequence	AY927537	15790800
168	1	LIT3542	Homo sapiens mRNA sequence	AY927536	15790800
169	1	LIT3528	Homo sapiens mRNA sequence	AY927518	15790800
170	1	LIT3523	Homo sapiens mRNA sequence	AY927513	15790800
171	1	LIT3518	Homo sapiens mRNA sequence	AY927508	15790800
172	1	LIT3489	Homo sapiens mRNA sequence	AY927471	15790800
173	1	LIT3467	Homo sapiens mRNA sequence	AY927433	15998900
174	1	LIT3465	Homo sapiens mRNA sequence	AY927429	15998900
175	1	LIT2239	Homo sapiens testis transcript Y 8 (TTY8) mRNA, complete cds, alternatively spliced.	AF332234	12815422
176	1	LIT2238	Homo sapiens testis transcript Y 10 (TTY10) mRNA, complete cds.	AF332239	12815422
177	1	LIT2234	Homo sapiens testis transcript Y 14 (TTY14) mRNA, complete cds.	AF332243	12815422
178	1	LIT2222	Homo sapiens IC-SNURF-SNRPN transcript noncoding exons 61-66a and 67-8	AF400490	11726556
179	1	LIT2212	Homo sapiens IC-SNURF-SNRPN transcript noncoding exons 86-88	AF400496	11726556
180	1	LIT2210	Homo sapiens IC-SNURF-SNRPN transcript noncoding exons 102-104a, 105-107	AF400499	11726556

181	1	LIT2098	Homo sapiens syndecan 4 pseudogene mRNA, partial sequence.	AF520746	12652906
182	1	LIT2067	Homo sapiens serotonin-7 receptor pseudogene, complete sequence.	U86813	9473718
183	1	LIT2048	Homo sapiens cytochrome P450 4Z2 pseudogene (CYP4Z2P), misc RNA	XR_000161	9501312
184	1	LIT2037	Human beta globin intergenic transcript 1 from Human beta globin region on chromosome 11	U01317	9334315, 10882078
185	1	LIT1994	Homo sapiens partial BCMS gene for B-cell neoplasia associated transcript, exon 8	AJ411981	11406609
186	1	LIT1904	Homo sapiens mRNA for B-cell neoplasia associated transcript, (BCMS gene), splice variant S, non coding transcript	AJ412041	11406609
187	1	LIT1897	Homo sapiens mRNA for B-cell neoplasia associated transcript, (BCMS gene), splice variant W, non coding transcript	AJ412045	11406609
188	1	LIT1624	Homo sapiens KVLQT1 gene	AJ006345	10024302
189	1	LIT1570	Homo sapiens clone H HOXA 11 antisense RNA	AF071166	12050232
190	1	LIT1562	Homo sapiens PCGEM1 gene, non-coding mRNA.	AF223389	11050243, 16569192
191	1	LIT1556	Homo sapiens non-coding RNA DD3, transcript (major) II	AF103908	10606244
192	1	LIT1552	Homo sapiens non-coding RNA DD3 sequence	AF103907	10606244
193	1	LIT1354	Homo sapiens PWCR1 mRNA, complete sequence	AF241255	11007541
194	1	LIT1315	Homo sapiens RNA transcript from U17 small nucleolar RNA host gene, variant U17HG-AB.	AJ006835	9671460
195	1	LIT1271	Homo sapiens testis transcript Y 4 (TTY4) mRNA, partial sequence.	AF332231	11687796, 12815422

196	1	LIT1250	Homo sapiens steroid receptor RNA activator isoform 2 mRNA, complete cds	AF293025	12444263, 10199399, 14517287, 15147866, 16848684
