# Supplementary material for: Massively Parallel Sequencing of Human Urinary Exosome/Microvesicle RNA Reveals a Predominance of Non-Coding RNA
Source: PLoS One. 2014 May 9;9(5):e96094. doi: 10.1371/journal.pone.0096094 (PMC4015934; doi:10.1371/journal.pone.0096094)
Supplement: Table S4 — Known ncRNA found in microvesicles (including exosomes) (-DNase). Listing of the 201 ncRNA transcripts (rnaDB set = "curated from literature") and the number of microvesicle derived sequencing transcripts that aligned against them. (ZIP) [file pone.0096094.s004.zip › PLOS Russo Supp Table S4 Final 040814.pdf]

Supp. Table S4: Known ncRNA found in microvesicles (including exosomes) (-DNase)

Listing of the 201 ncRNA transcripts (rnaDB set = "curated from literature") and the number of microvesicle derived sequencing transcripts that aligned against them

count	No. Reads aligning	rnaDB ID	rnaDB definition	Accession ID	Pubmed ID
1	914237	LIT1626	Homo sapiens non-coding chimeric transcript hc9(5)h-2-1/4 (UM 9(5))	AY072609	12515386
2	52467	LIT1868	Human 7S L gene, complete	M20910	6802847, 6181418, 3403542, 6084597
3	2972	LIT3330	Homo sapiens ret finger protein-like 3 antisense (RFPL3S) on chromosome 22.	NR_001450	10508838
4	2073	LIT3569	Homo sapiens mRNA sequence	AY927568	15790800
5	1620	LIT3497	Homo sapiens mRNA sequence	AY927481	15790800
6	1608	LIT2062	Homo sapiens ribosomal protein, large, P0 pseudogene 2 (RPLP0P2), misc RNA	XR_000076	8486366
7	1393	LIT1867	Homo sapiens 7S RNA	V00477	6802847, 6181418, 3403542, 6084597
8	1033	LIT1834	Homo sapiens non-small cell lung carcinoma noncoding RNA, partial sequence	AY166681	15254752
9	890	LIT2059	Homo sapiens general transcription factor II, i, pseudogene 1 (GTF2IP1), misc RNA	XR_000139	9466987
10	843	LIT2056	Homo sapiens EMX2OS mRNA, complete sequence.	AY117413	12573261

11	765	LIT3583	Homo sapiens mRNA sequence	AY927585	15790800
12	697	LIT3571	Homo sapiens mRNA sequence	AY927570	15790800
13	570	LIT3355	Homo sapiens, clone IMAGE:4391558, mRNA	BC017743	16607615
14	545	LIT3612	Homo sapiens mRNA sequence	AY927624	15790800
15	492	LIT3420	Homo sapiens candidate tumor suppressor (DLEU2) RNA, antisense sequence	AY455757	11264177, 15188451
16	454	LIT3418	Psoriasis-susceptibility Related RNA Gene Induced by Stress (PRINS) RNA	AK022045	15855153
17	448	LIT3319	Homo sapiens non-coding RNA in rhabdomyosarcoma (RMS) (NCRMS), misc RNA	XR_000219	12082533, 15872005
18	413	LIT2089	Homo sapiens clone BAC 33J7 diphosphoinositol polyphosphate phosphohydrolase type 2 (NUDT4) pseudogene, mRNA sequence.	AF311215	11376937
19	391	LIT2045	Homo sapiens RET finger protein-like 1 antisense transcript (RFPL1S), partial.	AJ010230	10508838
20	362	LIT3537	Homo sapiens mRNA sequence	AY927529	15790800
21	359	LIT2197	Human SNRPN mRNA, 3 prime UTR, partial sequence incorporating IC-SNURF-SNRPN transcript noncoding exons 10a, 11 and 12	U81001	9070929
22	306	LIT2019	Human Ro RNA (scRNA) hY3 from small cytoplasmic ribonucleoprotein particles.	K01563	7505620, 6201849
23	286	LIT1869	Homo sapiens RNA, 7SK, nuclear (RN7SK) on chromosome 6	NR_001445	11713533, 11713532, 1646389, 9018369, 6434539
24	283	LIT2014	Human gene hY1 encoding a cytoplasmic Ro RNA.	V00584	7505620,

					6187471
25	282	LIT3500	Homo sapiens mRNA sequence	AY927484	15790800
26	274	LIT2112	Human aldolase pseudogene mRNA, complete cds.	M21191	3674018
27	235	LIT3356	Homo sapiens cDNA clone IMAGE:5294683	BC043430	16607615
28	230	LIT2102	Human destrin-2 pseudogene mRNA, complete cds.	U72518	9074930
29	213	LIT1751	Homo sapiens 1 DISC2 gene, complete sequence	AF222981	10814723
30	206	LIT2232	Homo sapiens TTTY15 from cDNA DKFZp434I143 (from clone DKFZp434I143).	AL080135	10507722, 12815422
31	203	LIT2121	Homo sapiens noncoding RNA GA3824 implicated in autism	CB338058	12690205
32	194	LIT3623	Homo sapiens FMR1P binding RNA, partial sequence	AF040097	10973830
33	188	LIT3364	Homo sapiens oncomir-1 transcript (C13orf25 isoform 1) mRNA	AB176708	15944707
34	182	LIT1956	Homo sapiens partial BCMS gene for B-cell neoplasia associated transcript, exon 32	AJ412006	11406609
35	179	LIT2090	Homo sapiens clone BAC 608B23 diphosphoinositol polyphosphate phosphohydrolase type 2 (NUDT4) pseudogene, mRNA sequence.	AF311216	11376937
36	177	LIT1548	Human NTT gene	U54776	9027504
37	155	LIT2101	Human RPL13-2 pseudogene mRNA, complete cds.	U72513	9074930
38	139	LIT1821	Homo sapiens MESTIT1 antisense RNA, partial sequence	AF482998	12095916, 11821432
39	138	LIT1721	Human BC200 scRNA	U01305	9605471, 9131672, 9858834;

					9422992; 7684772; 9847409
40	138	LIT1553	Homo sapiens non-coding RNA DD3 gene, exons 2, 3, and 4	AF103908	10606244
41	134	LIT2191	Homo sapiens HERC2P3/D15F37 pseudogene, S4 allele, mRNA sequence.	AF041081	9949213
42	131	LIT1267	Homo sapiens growth arrest specific transcript 5 gene, exons 1 through 12 and alternatively spliced mRNA sequences	AF141346	9819378
43	123	LIT2065	Homo sapiens protocadherin-beta (PCDHpsi-5) pseudogene, mRNA sequence.	AF329369	11322959
44	119	LIT1094	Homo sapiens U50HG RNA	AB017710	10792466
45	107	LIT1750	Human unknown protein mRNA within the p53 intron 1, complete cds	U58658	8975713
46	100	LIT3414	Homo sapiens Pax6 opposite strand (Pax6OS) RNA, variant isoform	BX648962	15703187
47	94	LIT1319	Homo sapiens mRNA containing U19H snoRNA; mRNA 2	AJ224167	9630250, 8657112
48	86	LIT3426	Homo sapiens taurine upregulated gene 1 (TUG1), predicted sequence based on ESTs	AF279660	15797018
49	84	LIT2100	Homo sapiens beta-glucuronidase-like protein (5G4) pseudogene mRNA, partial sequence.	U21555	7552146
50	84	LIT2021	Human hy4 Ro RNA (associated with erythrocyte Ro RNPs).	X57566	8863726, 1698620
51	81	LIT2013	Homo sapiens metastasis associated in lung adenocarcinoma transcript 1 short isoform, transcribed non-coding RNA, complete sequence.	BK001411	12970751, 16878148

52	79	LIT3490	Homo sapiens mRNA sequence	AY927473	15790800
53	79	LIT2225	Homo sapiens IC-SNURF-SNRPN transcript noncoding exons 36-40, 42-44, 46-48, and 50-61	AF400487	11726556
54	77	LIT1011	Homo sapiens Jpx noncoding transcript	AV714079	12045143
55	73	LIT3492	Homo sapiens mRNA sequence	AY927476	15790800
56	73	LIT2063	Homo sapiens C2H2 zinc finger protein pseudogene, mRNA sequence.	AF033199	9628832
57	69	LIT3532	Homo sapiens mRNA sequence	AY927522	15790800
58	65	LIT2070	Homo sapiens HERC2P7 pseudogene, partial mRNA sequence.	AF071178	9949213
59	62	LIT1584	Homo sapiens RNA for differentiation or sex determination (CMPD)	D43770	8789441
60	59	LIT1275	Homo sapiens DLEU1 noncoding transcript (BCMS)	AF279660	11264177
61	56	LIT3551	Homo sapiens mRNA sequence	AY927545	15790800
62	56	LIT1316	Homo sapiens RNA transcript from U17 small nucleolar RNA host gene, variant U17HG-A	AJ006834	9671460
63	52	LIT1973	Homo sapiens partial BCMS gene for B-cell neoplasia associated transcript, exon 18	AJ411992	11406609
64	51	LIT2060	Homo sapiens Shwachman-Bodian-Diamond syndrome pseudogene (SBDSP), mRNA (cDNA clone IMAGE:4329436)	BC010183	12496757
65	50	LIT3354	Human U1 small nuclear RNA (snRNA) gene HU1-1	J00318	6180116, 12389039, 16115885
66	50	LIT1007	Homo sapiens Ftx noncoding RNA	AK057701	12045143
67	46	LIT3477	Homo sapiens mRNA sequence	AY927449	15998900
68	43	LIT2044	Homo sapiens topoisomerase (DNA) I pseudogene	M55630	2170234

			2 (TOP1P2), misc RNA		
69	41	LIT2214	Homo sapiens IC-SNURF-SNRPN transcript noncoding exons 106-144 and 146	AF400501	11726556
70	39	LIT2116	Homo sapiens SZ-1 mRNA (PSZA11q14), complete sequence	AF525782	13130513
71	39	LIT1826	Human 2G7 gene, complete sequence	U62668	8968746
72	37	LIT3549	Homo sapiens mRNA sequence	AY927543	15790800
73	37	LIT3470	Homo sapiens mRNA sequence	AY927437	15998900
74	37	LIT1912	Homo sapiens mRNA for B-cell neoplasia associated transcript, (BCMS gene), splice variant K,<br>non coding transcript	AJ412033	11406609
75	36	LIT3337	Homo sapiens human accelarated region forward mRNA, complete sequence	DQ860409	16915236
76	36	LIT1596	Homo sapiens X (inactive)-specific transcript (XIST) complete exon	M97168	11734999, 2034278, 10723727, 2034279, 1423610, 1985261, 1423611, 11290427
77	34	LIT3374	Homo sapiens noncoding repressor of NFAT (NRON) RNA, exon 3	AY264286	16141075
78	33	LIT1998	Homo sapiens partial BCMS gene for B-cell neoplasia associated transcript, exon 4a	AJ411977	11406609
79	33	LIT1318	Homo sapiens mRNA containing U19H snoRNA; mRNA 1	AJ224166	9630250, 8657112

80	29	LIT2233	Homo sapiens CYorf14 from PRO2834 mRNA, complete cds.	AF119903	12815422
81	28	LIT3316	Homo sapiens myosin XVB, pseudogene, mRNA (cDNA clone MGC:34148 IMAGE:5205535), complete cds.	BC027875	11964073
82	27	LIT3580	Homo sapiens mRNA sequence	AY927581	15790800
83	26	LIT1320	Homo sapiens mRNA containing U19H snoRNA; mRNA 3	AJ224168	9630250, 8657112
84	26	LIT1000	Homo sapiens COPG2IT1 noncoding transcript		10995575
85	25	LIT3447	Homo sapiens SLC4A9 noncoding transcript (SLC4A9N) from NCI_CGAP_Kid11 cDNA clone IMAGE:2384256	AI830440	11305939
86	25	LIT3372	Homo sapiens U87HG mRNA, complete sequence	AY264285	16226852
87	25	LIT2113	Human cytochrome b5 pseudogene mRNA.	M64714	1712589
88	23	LIT3624	Homo sapiens FMR1P binding RNA, partial sequence	AF040098	10973830
89	23	LIT3570	Homo sapiens mRNA sequence	AY927569	15790800
90	23	LIT3471	Homo sapiens mRNA sequence	AY927440	15998900
91	23	LIT1752	Homo sapiens ncR-uPAR transcript	AF222981	12084570
92	21	LIT3598	Homo sapiens mRNA sequence	AY927608	15790800
93	21	LIT3575	Homo sapiens mRNA sequence	AY927575	15790800
94	21	LIT1251	Homo sapiens steroid receptor RNA activator isoform 3 mRNA, complete cds	AF293026	12444263, 10199399, 14517287, 15147866, 16848684
95	19	LIT3336	Homo sapiens urothelial cancer associated 1 (UCA1) mRNA, complete sequence	DQ343132	16914600

96	18	LIT3524	Homo sapiens mRNA sequence	AY927514	15790800
97	17	LIT3550	Homo sapiens mRNA sequence	AY927544	15790800
98	17	LIT3496	Homo sapiens mRNA sequence	AY927480	15790800
99	17	LIT1997	Homo sapiens partial BCMS gene for B-cell neoplasia associated transcript, exon 5	AJ411978	11406609
100	17	LIT1321	Homo sapiens mRNA containing U19H snoRNA; mRNA 4	AJ224169	9630250, 8657112
101	16	LIT3533	Homo sapiens mRNA sequence	AY927524	15790800
102	15	LIT3495	Homo sapiens mRNA sequence	AY927479	15790800
103	15	LIT2190	Homo sapiens HERC2P2/D15F37 pseudogene, S3 allele, mRNA sequence	AF041080	9949213
104	15	LIT1758	H.sapiens MRP RNA gene encoding the RNA component of RNase MRP (RMRP)	X51867	11207361, 1690392
105	14	LIT3609	Homo sapiens mRNA sequence	AY927621	15790800
106	14	LIT1110	Homo sapiens PAR5 gene, complete sequence.	AF019618	7987392
107	13	LIT3548	Homo sapiens mRNA sequence	AY927542	15790800
108	12	LIT3514	Homo sapiens mRNA sequence	AY927500	15790800
109	11	LIT3526	Homo sapiens mRNA sequence	AY927516	15790800
110	11	LIT2203	Homo sapiens IC-SNURF-SNRPN transcript noncoding exons 12, 13d, 14, 15, 16, 17, 18, 19 and 20	AF319524	9070929, 11159938
111	11	LIT2050	Homo sapiens COX11 homolog, cytochrome c oxidase assembly protein (yeast) pseudogene (COX11P), misc RNA	NR_000031	12631698
112	11	LIT1702	Homo sapiens hypoxia inducible factor (aHIF) antisense RNA sequence	U85044	9923855, 14744852, 12459261
113	10	LIT3563	Homo sapiens mRNA sequence	AY927560	15790800

114	10	LIT2054	Homo sapiens olfactory receptor, family 7, subfamily E, member 24 pseudogene(OR7E24P), misc RNA	XR_000082	12727898
115	9	LIT3593	Homo sapiens mRNA sequence	AY927602	15790800
116	9	LIT3574	Homo sapiens mRNA sequence	AY927573	15790800
117	9	LIT3363	Homo sapiens oncomir-1 transcript (C13orf25 isoform 2) mRNA	AB176707	15944707
118	9	LIT2209	Homo sapiens IC-SNURF-SNRPN transcript noncoding exons 22-37, 40, 42-44, and 46-58	AF400486	11726556
119	8	LIT3566	Homo sapiens mRNA sequence	AY927564	15790800
120	8	LIT2024	Human Ro RNA (scRNA) hY5 from small cytoplasmic ribonucleoprotein particles.	K01564	8863726, 7529149, 6201849
121	8	LIT1625	Homo sapiens KVLQT1 gene	AJ006345	10024302
122	7	LIT3610	Homo sapiens mRNA sequence	AY927622	15790800
123	7	LIT3587	Homo sapiens mRNA sequence	AY927593	15790800
124	7	LIT2057	Homo sapiens proliferating cell nuclear antigen (PCNA) antisense	AL121924	10488129
125	7	LIT1581	Homo sapiens homeo box A11, antisense (Hoxa11as), misc RNA	XR_000158	12050232
126	7	LIT1570	Homo sapiens clone H HOXA 11 antisense RNA	AF071166	12050232
127	7	LIT1354	Homo sapiens PWCR1 mRNA, complete sequence	AF241255	11007541
128	6	LIT3611	Homo sapiens mRNA sequence	AY927623	15790800
129	6	LIT3542	Homo sapiens mRNA sequence	AY927536	15790800
130	6	LIT3320	Homo sapiens SCA8 mRNA, repeat region.	AF126749	10888605, 10192387
131	6	LIT2099	H.sapiens mRNA for olfactory receptor expressed pseudogene, poly A site.	X87825	8647456

132	6	LIT1227	H.sapiens predicted non coding cDNA (DGCR5)	X91348	8659529
133	5	LIT2077	Homo sapiens TRIpartite motif protein pseudogene mRNA sequence.	AF230412	11331580
134	4	LIT3614	Homo sapiens mRNA sequence	AY927628	15790800
135	4	LIT3534	Homo sapiens mRNA sequence	AY927525	15790800
136	4	LIT2234	Homo sapiens testis transcript Y 14 (TTY14) mRNA, complete cds.	AF332243	12815422
137	4	LIT2111	H.sapiens mRNA for cAMP dependent proteinkinase PRKX, pseudogene.	Y10483	9154127
138	4	LIT2048	Homo sapiens cytochrome P450 4Z2 pseudogene (CYP4Z2P), misc RNA	XR_000161	9501312
139	4	LIT2037	Human beta globin intergenic transcript 1 from Human beta globin region on chromosome 11	U01317	9334315, 10882078
140	4	LIT2002	Homo sapiens partial BCMS gene for B-cell neoplasia associated transcript, exon 4	AJ411976	11406609
141	4	LIT1994	Homo sapiens partial BCMS gene for B-cell neoplasia associated transcript, exon 8	AJ411981	11406609
142	4	LIT1674	Homo sapiens IPW mRNA sequence	U12897	7849716, 9063754, 11726556
143	4	LIT1554	Homo sapiens non-coding RNA DD3, transcript III	AF103908	10606244
144	4	LIT1386	Homo sapiens spliced UHG RNA	L36587	7985025
145	3	LIT3601	Homo sapiens mRNA sequence	AY927613	15790800
146	3	LIT3582	Homo sapiens mRNA sequence	AY927584	15790800
147	3	LIT3518	Homo sapiens mRNA sequence	AY927508	15790800
148	3	LIT3516	Homo sapiens mRNA sequence	AY927503	15790800
149	3	LIT2245	Homo sapiens testis transcript Y 9 (TTY9) mRNA, complete cds.	AF332238	12815422

150	3	LIT2226	Homo sapiens IC-SNURF-SNRPN transcript noncoding exons 138-145	AF400500	11726556
151	3	LIT2088	Homo sapiens olfactory receptor-like (PJCG9) pseudogene mRNA, partial sequence.	AF359419	11705801
152	3	LIT2032	Homo sapiens vault-associated RNA 1, complete sequence	AF045143	9535882, 16150923
153	3	LIT1704	Human small cellular 7 SK mRNA	X04236	6196783, 9018369, 11713533, 11713532, 6434539, 9018369
154	3	LIT1662	Homo sapiens UM 9(5)h non-coding transcript mRNA sequence	BK000166	12515386
155	2	LIT3607	Homo sapiens mRNA sequence	AY927619	15790800
156	2	LIT3604	Homo sapiens mRNA sequence	AY927616	15790800
157	2	LIT3567	Homo sapiens mRNA sequence	AY927566	15790800
158	2	LIT3530	Homo sapiens mRNA sequence	AY927520	15790800
159	2	LIT3489	Homo sapiens mRNA sequence	AY927471	15790800
160	2	LIT3466	Homo sapiens mRNA sequence	AY927430	15998900
161	2	LIT3357	Homo sapiens heat shock RNA-1 (HSR-1)	BC043430	16554823
162	2	LIT3321	Homo sapiens maternally expressed gene 3 (MEG3) mRNA, complete sequence.	AY314975	14602737
163	2	LIT2239	Homo sapiens testis transcript Y 8 (TTY8) mRNA, complete cds, alternatively spliced.	AF332234	12815422
164	2	LIT2224	Homo sapiens IC-SNURF-SNRPN transcript noncoding exons 33, 34, 34.2, 35, and 36	AY421730	11726556
165	2	LIT2213	Homo sapiens IC-SNURF-SNRPN transcript	AF400488	11726556

			noncoding exons 44-48 and 50-61		
166	2	LIT2064	Homo sapiens cytidine monophosphate-N-acetylneuraminic acid hydroxylase(CMP-N-acetylneuraminate monooxygenase) (CMAH), misc RNA	XR_000114	12192086
167	2	LIT2058	Homo sapiens hair and skin epidermal-type 12-lipoxygenase-related protein (ALOX12E) mRNA, complete pseudogene sequence.	AF020774	9691181
168	2	LIT1980	Homo sapiens ST7 overlapping transcript 4, mRNA sequence	BM413625	12213198, 12690205
169	2	LIT1900	Homo sapiens clone IMAGE:1628386 ST7OT3 mRNA, non-coding transcript	AF400044	12213198, 12690205
170	2	LIT1610	Homo sapiens H19 gene, complete sequence	AF125183	1688465, 10666466, 12140686, 11813134, 12490325, 12419837, 11794466, 1811930, 11726548
171	2	LIT1568	Homo sapiens clone J HOXA 11 antisense RNA	AF071167	12050232
172	2	LIT1556	Homo sapiens non-coding RNA DD3, transcript (major) II	AF103908	10606244
173	2	LIT1315	Homo sapiens RNA transcript from U17 small nucleolar RNA host gene, variant U17HG-AB.	AJ006835	9671460
174	2	LIT1245	Homo sapiens steroid receptor RNA activator 1 (SRA1), misc RNA	XR_000132	12444263, 10199399, 14517287,

					15147866, 16848684
175	1	LIT3606	Homo sapiens mRNA sequence	AY927618	15790800
176	1	LIT3602	Homo sapiens mRNA sequence	AY927614	15790800
177	1	LIT3590	Homo sapiens mRNA sequence	AY927597	15790800
178	1	LIT3586	Homo sapiens mRNA sequence	AY927592	15790800
179	1	LIT3528	Homo sapiens mRNA sequence	AY927518	15790800
180	1	LIT3522	Homo sapiens mRNA sequence	AY927512	15790800
181	1	LIT3519	Homo sapiens mRNA sequence	AY927509	15790800
182	1	LIT3508	Homo sapiens mRNA sequence	AY927493	15790800
183	1	LIT3498	Homo sapiens mRNA sequence	AY927482	15790800
184	1	LIT3494	Homo sapiens mRNA sequence	AY927478	15790800
185	1	LIT2244	Homo sapiens testis transcript Y 8 (TTY8) mRNA, complete cds, alternatively spliced.	AF332235	12815422
186	1	LIT2085	Homo sapiens olfactory receptor-like (PJCG6) pseudogene mRNA, partial sequence.	AF359416	11705801
187	1	LIT2079	Homo sapiens olfactory-like receptor (OR5E1P) pseudogene, mRNA sequence.	AF309699	11705801
188	1	LIT2047	Homo sapiens mannose-binding lectin (protein A) 1, pseudogene 1 (MBL1P1), misc RNA	AF019382	9501312
189	1	LIT2046	Homo sapiens D15F37 (pseudogene) (MN7), misc RNA	XR_000150	9730612
190	1	LIT2038	Human beta globin intergenic transcript 2 from Human beta globin region on chromosome 11	U01317	9334315, 10882078
191	1	LIT2008	Homo sapiens ST7 overlapping transcript 4, mRNA sequence	BM413626	12213198, 12690205
192	1	LIT1905	Homo sapiens mRNA for B-cell neoplasia associated transcript, (BCMS gene), splice variant	AJ412040	11406609

			R, non coding transcript		
193	1	LIT1899	Homo sapiens clone IMAGE:1409652 ST7OT2 mRNA, non-coding transcript	AF400045	12213198, 12690205
194	1	LIT1897	Homo sapiens mRNA for B-cell neoplasia associated transcript, (BCMS gene), splice variant W, non coding transcript	AJ412045	11406609
195	1	LIT1757	Homo sapiens RNase MRP RNA component, complete sequence	AF458223	11207361, 1690392
196	1	LIT1660	Homo sapiens LIT1 transcript	AA622687	10393948, 10369866, 11813134
197	1	LIT1624	Homo sapiens KVLQT1 gene	AJ006345	10024302
198	1	LIT1562	Homo sapiens PCGEM1 gene, non-coding mRNA.	AF223389	11050243, 16569192
199	1	LIT1561	Homo sapiens non-coding RNA DD3, transcript I	AF103908	10606244
200	1	LIT1552	Homo sapiens non-coding RNA DD3 sequence	AF103907	10606244
201	1	LIT1550	Homo sapiens partial mRNA for TTY2 gene, clone TTY2L2A	AJ297964	11381023, 9381176, 11097430, 12815422
