# Supplementary material for: Massively Parallel Sequencing of Human Urinary Exosome/Microvesicle RNA Reveals a Predominance of Non-Coding RNA
Source: PLoS One. 2014 May 9;9(5):e96094. doi: 10.1371/journal.pone.0096094 (PMC4015934; doi:10.1371/journal.pone.0096094)
Supplement: Table S5 — Genes found within microvesicles (including exosomes) (+DNase). Raw and normalized counts (as per Casava's 1.7 read counts method) of genes identified in microvesicles. (ZIP) [file pone.0096094.s005.zip › PLOS Russo Supp Table S5 Final 040814.pdf]

Supp. Table S5: Genes found within microvesicles (including exosomes) (+DNase)

Raw and normalized counts (as per Casava's 1.7 read counts method) of genes identified in microvesicles

chr	start	end	hgnc	normal	raw
chr1.fa	14362	29370	WASH7P	0	0
chr1.fa	69091	70008	OR4F5	0	0
chr1.fa	661139	665731	LOC100133331	0	0
chr1.fa	700245	714068	LOC100288069	0	0
chr1.fa	761586	762902	NCRNA00115	0	0
chr1.fa	763064	789740	LOC643837	4.381549084	304
chr1.fa	803451	812182	FAM41C	0	0
chr1.fa	852953	854817	FLJ39609	3.407698539	76
chr1.fa	861121	879582	SAMD11	7.771011554	760
chr1.fa	879962	894679	NOC2L	13.96260165	1520
chr1.fa	895967	901099	KLHL17	0	0
chr1.fa	901877	910484	PLEKHN1	8.325877302	912
chr1.fa	910579	917473	C1orf170	1.111955406	152
chr1.fa	934342	935552	HES4	24.37739838	1140
chr1.fa	948847	949919	ISG15	35.52919915	1064
chr1.fa	955503	991492	AGRN	6.927926965	2280
chr1.fa	1017198	1051736	C1orf159	1.606553171	152
chr1.fa	1102484	1102578	MIR200B	0	0
chr1.fa	1103243	1103332	MIR200A	0	0
chr1.fa	1104385	1104467	MIR429	0	0
chr1.fa	1109286	1133313	TTLL10	0	0
chr1.fa	1138888	1142089	TNFRSF18	0	0
chr1.fa	1146706	1149548	TNFRSF4	0	0
chr1.fa	1152288	1167447	SDF4	8.359013573	760
chr1.fa	1167629	1170420	B3GALT6	3.026742616	380
chr1.fa	1177833	1182102	FAM132A	0	0
chr1.fa	1189292	1209234	UBE2J2	11.09664778	1216
chr1.fa	1210638	1227409	SCNN1D	0.976074456	152
chr1.fa	1227764	1243269	ACAP3	4.40756884	745
chr1.fa	1243994	1246964	PUSL1	5.894253219	304
chr1.fa	1247058	1260046	CPSF3L	23.17581936	2128
chr1.fa	1260143	1264276	GLTPD1	10.78997048	1064
chr1.fa	1266726	1269844	TAS1R3	0	0
chr1.fa	1270658	1284492	DVL1	15.02896688	1976
chr1.fa	1288071	1293915	MXRA8	1.487129161	152
chr1.fa	1309110	1310818	AURKAIP1	58.9423098	3040
chr1.fa	1321091	1334718	CCNL2	6.516725855	1064
chr1.fa	1334910	1337275	LOC148413	19.05024242	1368

chr1.fa	1337427	1342693	MRPL20	153.9159778	4028
chr1.fa	1353800	1356650	LOC441869	8.375470513	760
chr1.fa	1361508	1363167	TMEM88B	0	0
chr1.fa	1370903	1378262	VWA1	1.451101805	304
chr1.fa	1385069	1405538	ATAD3C	4.379769955	760
chr1.fa	1407164	1431582	ATAD3B	0.694749738	76
chr1.fa	1447523	1470067	ATAD3A	4.816990821	608
chr1.fa	1470158	1475740	C1orf70	0	0
chr1.fa	1477053	1510262	SSU72	105.0682216	6156
chr1.fa	1550795	1565990	MIB2	6.620804881	1064
chr1.fa	1567560	1568499	MMP23B	0	0
chr1.fa	1568159	1633247	MMP23A	0	0
chr1.fa	1571100	1654073	CDK11B	10.95142641	944
chr1.fa	1592939	1624243	SLC35E2B	4.786523243	1292
chr1.fa	1634170	1655791	CDK11A	2.642228437	228
chr1.fa	1656277	1677438	SLC35E2	2.084693996	684
chr1.fa	1682671	1711508	NADK	17.39943342	3268
chr1.fa	1716729	1822495	GNB1	146.7298548	20638
chr1.fa	1846266	1848733	CALML6	0	0
chr1.fa	1849029	1850740	TMEM52	1.764228448	76
chr1.fa	1884752	1935276	KIAA1751	0	0
chr1.fa	1950768	1962192	GABRD	0	0
chr1.fa	1981909	2106752	PRKCZ	13.83494917	1368
chr1.fa	2115899	2139172	C1orf86	10.96877291	946
chr1.fa	2120482	2125024	LOC100128003	1.960822164	380
chr1.fa	2160134	2241652	SKI	15.10413507	3876
chr1.fa	2252696	2322993	MORN1	5.14991027	380
chr1.fa	2281853	2284100	LOC100129534	1.503808492	152
chr1.fa	2323214	2336240	RER1	17.87090251	1976
chr1.fa	2336886	2344010	PEX10	23.45736647	1444
chr1.fa	2407754	2436964	PLCH2	3.843585058	836
chr1.fa	2439975	2458035	PANK4	5.117885954	608
chr1.fa	2460184	2461684	HES5	2.588409795	152
chr1.fa	2481359	2484284	LOC115110	0	0
chr1.fa	2487805	2495267	TNFRSF14	17.83309603	1368
chr1.fa	2517899	2522908	C1orf93	22.68678138	2584
chr1.fa	2522996	2564481	MMEL1	0.688745179	76
chr1.fa	2938046	2939467	ACTRT2	0	0
chr1.fa	2976181	2984289	FLJ42875	1.756889542	304
chr1.fa	2985742	3355185	PRDM16	6.006560715	2356
chr1.fa	3044539	3044599	MIR4251	0	0
chr1.fa	3371147	3397677	ARHGEF16	6.248966993	816
chr1.fa	3404506	3528059	MEGF6	0.227283685	76

chr1.fa	3477260	3477354	MIR551A	0	0
chr1.fa	3541556	3546694	TPRG1L	39.69502888	4332
chr1.fa	3547331	3566671	WDR8	3.979243618	304
chr1.fa	3569129	3650467	TP73	1.084156521	152
chr1.fa	3652548	3663335	KIAA0495	4.048852026	684
chr1.fa	3668965	3688209	CCDC27	0	0
chr1.fa	3689352	3692546	LOC388588	6.191145312	152
chr1.fa	3696784	3713068	LRRC47	29.99922252	3572
chr1.fa	3728645	3773797	KIAA0562	11.74291627	3410
chr1.fa	3773845	3801993	DFFB	1.674604842	228
chr1.fa	3805697	3816857	C1orf174	8.116162512	608
chr1.fa	3816968	3832011	LOC100133612	0	0
chr1.fa	4472111	4484744	LOC284661	0	0
chr1.fa	4715105	4843851	AJAP1	0.509497967	76
chr1.fa	5922870	6052357	NPHP4	2.100483763	456
chr1.fa	6052532	6161253	KCNAB2	1.81604557	456
chr1.fa	6161847	6240194	CHD5	0.524842952	228
chr1.fa	6245080	6259679	RPL22	165.3693409	15504
chr1.fa	6266189	6281252	RNF207	1.312329771	228
chr1.fa	6281360	6296044	ICMT	9.692915278	2052
chr1.fa	6304252	6305638	HES3	0	0
chr1.fa	6307406	6321035	GPR153	1.656146382	304
chr1.fa	6324332	6453826	ACOT7	12.7345581	1368
chr1.fa	6475294	6479979	HES2	0.793491378	152
chr1.fa	6484848	6521004	ESPN	2.172983255	345
chr1.fa	6489894	6489956	MIR4252	0	0
chr1.fa	6521214	6526151	TNFRSF25	0	0
chr1.fa	6526256	6580069	PLEKHG5	1.830278599	456
chr1.fa	6581407	6614658	NOL9	4.532552628	1368
chr1.fa	6615338	6639817	TAS1R1	0	0
chr1.fa	6640063	6649340	ZBTB48	7.229044489	760
chr1.fa	6650784	6662929	KLHL21	13.93724907	2812
chr1.fa	6673756	6684093	PHF13	5.52641837	912
chr1.fa	6684925	6694227	THAP3	7.618228881	532
chr1.fa	6695647	6761966	DNAJC11	18.0381406	1520
chr1.fa	6845384	7829766	CAMTA1	1.354361685	532
chr1.fa	7831329	7841492	VAMP3	49.7933631	4937
chr1.fa	7844763	7905237	PER3	4.632183832	1292
chr1.fa	7907672	7913551	UTS2	0	0
chr1.fa	7975931	8003225	TNFRSF9	0	0
chr1.fa	8021714	8045342	PARK7	254.0026392	10976
chr1.fa	8071779	8086393	ERRFI1	16.74515886	2356
chr1.fa	8384390	8404227	SLC45A1	0	0

chr1.fa	8412464	8877699	RERE	68.56139124	25653
chr1.fa	8921063	8938780	ENO1	213.2843889	17378
chr1.fa	9005922	9035148	CA6	0	0
chr1.fa	9063359	9086404	SLC2A7	0	0
chr1.fa	9097005	9148510	SLC2A5	6.604570332	836
chr1.fa	9164476	9189229	GPR157	1.466002008	76
chr1.fa	9211727	9211836	MIR34A	0	0
chr1.fa	9294863	9331394	H6PD	3.52311951	1444
chr1.fa	9352941	9429590	SPSB1	3.253359128	456
chr1.fa	9599528	9642831	SLC25A33	8.097926443	532
chr1.fa	9648932	9674935	TMEM201	0.438777603	76
chr1.fa	9711790	9789078	PIK3CD	0.635816101	152
chr1.fa	9712668	9714644	C1orf200	0	0
chr1.fa	9789173	9884550	CLSTN1	7.956930498	1824
chr1.fa	9908334	9970316	CTNNBIP1	28.78096418	3876
chr1.fa	9989776	10002826	LZIC	26.42762176	1672
chr1.fa	10003486	10045556	NMNAT1	4.917289199	836
chr1.fa	10057255	10076078	RBP7	15.4824223	456
chr1.fa	10093041	10241296	UBE4B	21.84947896	5776
chr1.fa	10270764	10441661	KIF1B	6.754017139	4864
chr1.fa	10459085	10480201	PGD	49.66771214	4326
chr1.fa	10502322	10502872	APITD1	17.1132161	424
chr1.fa	10509776	10510379	CORT	0	0
chr1.fa	10520603	10532613	DFFA	10.9589877	1672
chr1.fa	10535003	10690815	PEX14	5.05739558	438
chr1.fa	10696666	10856707	CASZ1	31.14164551	11932
chr1.fa	11006530	11042094	C1orf127	0	0
chr1.fa	11072679	11085549	TARDBP	20.84160258	3952
chr1.fa	11086580	11107285	MASP2	0	0
chr1.fa	11114649	11120091	SRM	23.89881277	1368
chr1.fa	11126676	11159938	EXOSC10	21.5814977	2724
chr1.fa	11166588	11322608	MTOR	14.90086962	5846
chr1.fa	11249398	11256038	ANGPTL7	0	0
chr1.fa	11333255	11348491	UBIAD1	3.237791753	532
chr1.fa	11539295	11597640	PTCHD2	0	0
chr1.fa	11708448	11714739	FBXO2	7.630682782	456
chr1.fa	11714914	11723384	FBXO44	2.036435131	304
chr1.fa	11724150	11734409	FBXO6	2.182323681	152
chr1.fa	11734537	11751678	MAD2L2	3.533571891	211
chr1.fa	11751781	11780336	C1orf187	0	0
chr1.fa	11796142	11810828	AGTRAP	9.243240512	532
chr1.fa	11845787	11866160	MTHFR	10.40100848	3344
chr1.fa	11866207	11903201	CLCN6	2.40426998	608

chr1.fa	11905767	11907840	NPPA	0	0
chr1.fa	11917521	11918992	NPPB	0	0
chr1.fa	11980123	11986480	KIAA2013	4.333735001	456
chr1.fa	11994746	12035594	PLOD1	3.375896614	456
chr1.fa	12040238	12073572	MFN2	44.81113571	9424
chr1.fa	12079512	12092106	MIIP	2.12872743	152
chr1.fa	12123434	12204264	TNFRSF8	0	0
chr1.fa	12227060	12269277	TNFRSF1B	0.459904756	76
chr1.fa	12290113	12572098	VPS13D	9.723382857	7139
chr1.fa	12627939	12677820	DHRS3	16.84567963	1368
chr1.fa	12704566	12727097	AADACL4	0	0
chr1.fa	12776118	12788726	AADACL3	0	0
chr1.fa	12806163	12821102	C1orf158	0	0
chr1.fa	12834984	12838048	PRAMEF12	0	0
chr1.fa	12851546	12856777	PRAMEF1	0	0
chr1.fa	12884468	12891264	PRAMEF11	0	0
chr1.fa	12907236	12907260	LOC649330	0	0
chr1.fa	12908238	12908578	HNRNPCL1	0	0
chr1.fa	12916941	12921764	PRAMEF2	0	0
chr1.fa	12939033	12946025	PRAMEF4	0	0
chr1.fa	12952727	12958094	PRAMEF10	0	0
chr1.fa	12976450	13611550	PRAMEF8	0	0
chr1.fa	12998302	13007406	PRAMEF6	0	0
chr1.fa	13035543	13038381	PRAMEF22	0	0
chr1.fa	13108514	13369057	PRAMEF5	0	0
chr1.fa	13182960	13184326	LOC440563	0	0
chr1.fa	13328196	13331692	PRAMEF3	0	0
chr1.fa	13449560	13449703	PRAMEF13	0	0
chr1.fa	13495254	13498259	PRAMEF16	0	0
chr1.fa	13516066	13737001	PRAMEF20	0	0
chr1.fa	13521963	13742807	PRAMEF21	0	0
chr1.fa	13668269	13673511	PRAMEF14	0	0
chr1.fa	13716088	13719064	PRAMEF17	0	0
chr1.fa	13801445	13840242	LRRC38	0	0
chr1.fa	13910252	13944452	PDPN	0	0
chr1.fa	14026735	14151574	PRDM2	20.9881583	9044
chr1.fa	14925213	15444544	KAZ	3.428380909	912
chr1.fa	15438311	15478960	C1orf126	0.47925278	76
chr1.fa	15479028	15546974	TMEM51	2.265720336	228
chr1.fa	15573768	15724622	FHAD1	0.664059769	152
chr1.fa	15736391	15756839	EFHD2	20.96124898	2280
chr1.fa	15764938	15773153	CTRC	0	0
chr1.fa	15783223	15798586	CELA2A	0	0

chr1.fa	15802596	15817895	CELA2B	0	0
chr1.fa	15818769	15851285	CASP9	7.656702538	817
chr1.fa	15853352	15898228	DNAJC16	7.293760293	1976
chr1.fa	15899152	15911605	AGMAT	23.08975402	2280
chr1.fa	15943953	15986363	DDI2	41.94251315	2812
chr1.fa	15987553	15988217	RSC1A1	33.04086534	988
chr1.fa	16010827	16061264	PLEKHM2	15.99147548	2964
chr1.fa	16062809	16067884	SLC25A34	0.550640317	76
chr1.fa	16068917	16074477	TMEM82	1.111955406	76
chr1.fa	16085255	16113084	FBLIM1	9.588836252	1444
chr1.fa	16133657	16134194	UQCRHL	3.141496414	76
chr1.fa	16160710	16163401	FLJ37453	1.255620045	152
chr1.fa	16174359	16266950	SPEN	31.48190386	17252
chr1.fa	16268364	16302627	ZBTB17	6.143776012	760
chr1.fa	16330731	16333184	C1orf64	0	0
chr1.fa	16340523	16345285	HSPB7	0.584443762	76
chr1.fa	16348486	16360545	CLCNKA	1.999518212	228
chr1.fa	16370247	16383803	CLCNKB	2.312867246	304
chr1.fa	16384264	16400127	FAM131C	0	0
chr1.fa	16450832	16482582	EPHA2	6.82206881	1216
chr1.fa	16524599	16539104	ARHGEF19	1.115958446	152
chr1.fa	16558182	16563659	C1orf89	7.584203046	532
chr1.fa	16576559	16678948	FBXO42	14.63177641	1958
chr1.fa	16693583	16724640	C1orf144	49.18423393	8037
chr1.fa	16725138	16763919	SPATA21	0	0
chr1.fa	16767167	16786584	NECAP2	18.68040605	1748
chr1.fa	16793931	16819196	CROCCP3	0.314905771	76
chr1.fa	16875409	17185516	MIR3675	0	0
chr1.fa	16890412	16939982	NBPF1	7.011546011	1368
chr1.fa	16944751	16957401	CROCCP2	4.636854045	368
chr1.fa	16972069	16976915	MST1P2	0	0
chr1.fa	17017713	17046652	ESPNP	0	0
chr1.fa	17081401	17090975	MST1P9	1.537834327	304
chr1.fa	17248445	17299474	CROCC	2.285290751	684
chr1.fa	17300999	17308081	MFAP2	0	0
chr1.fa	17312453	17338423	ATP13A2	0.845975673	152
chr1.fa	17345225	17380665	SDHB	45.76007845	2356
chr1.fa	17393256	17445948	PADI2	1.937026318	380
chr1.fa	17531621	17572501	PADI1	0.439444777	76
chr1.fa	17575593	17610727	PADI3	27.03853006	3876
chr1.fa	17634690	17690495	PADI4	0	0
chr1.fa	17698741	17728195	PADI6	0	0
chr1.fa	17733251	17766220	RCC2	25.7706785	4787

chr1.fa	17866330	18024370	ARHGEF10L	17.21129056	3478
chr1.fa	18081808	18153558	ACTL8	0	0
chr1.fa	18434240	18704977	IGSF21	0.869326737	76
chr1.fa	18807424	18812480	KLHDC7A	16.04284782	3648
chr1.fa	18957500	19075360	PAX7	0	0
chr1.fa	19166093	19186155	TAS1R2	0	0
chr1.fa	19197924	19229293	ALDH4A1	48.57443759	5609
chr1.fa	19230774	19282826	IFFO2	6.372616435	1672
chr1.fa	19401000	19536746	UBR4	10.96143401	7828
chr1.fa	19544584	19578046	KIAA0090	1.595211226	304
chr1.fa	19578075	19586622	MRTO4	4.461609873	456
chr1.fa	19592476	19600568	AKR7L	0	0
chr1.fa	19609057	19615280	AKR7A3	19.06714414	1064
chr1.fa	19630459	19638640	AKR7A2	31.02377823	1900
chr1.fa	19638740	19655794	PQLC2	0.89668084	76
chr1.fa	19665267	19812066	CAPZB	184.636637	14197
chr1.fa	19923471	19956314	C1orf151	30.32858371	5440
chr1.fa	19969726	19984945	NBL1	110.5141344	10868
chr1.fa	19991780	20006055	HTR6	0	0
chr1.fa	20008706	20126410	TMCO4	19.89110309	2660
chr1.fa	20140522	20141771	RNF186	6.760688871	380
chr1.fa	20208888	20239437	OTUD3	6.227172667	1824
chr1.fa	20246800	20250110	PLA2G2E	0	0
chr1.fa	20301924	20306932	PLA2G2A	33.76964091	1520
chr1.fa	20396701	20418394	PLA2G5	1.784688427	152
chr1.fa	20438434	20446008	PLA2G2D	0	0
chr1.fa	20465823	20476879	PLA2G2F	1.864749217	228
chr1.fa	20490484	20501687	PLA2G2C	0	0
chr1.fa	20512578	20519942	UBXN10	2.843492365	380
chr1.fa	20617412	20681387	VWA5B1	1.510702615	304
chr1.fa	20686294	20755287	LOC339505	0	0
chr1.fa	20808884	20812728	CAMK2N1	34.5079793	3648
chr1.fa	20825941	20834674	MUL1	2.750532894	304
chr1.fa	20878932	20881513	FAM43B	0	0
chr1.fa	20915444	20945400	CDA	1.762449319	76
chr1.fa	20959948	20978004	PINK1	31.13475138	3724
chr1.fa	20978260	20988037	DDOST	15.10502463	1444
chr1.fa	20990507	21044317	KIF17	0	0
chr1.fa	21046225	21059133	SH2D5	0	0
chr1.fa	21069171	21113181	HP1BP3	41.81241437	7372
chr1.fa	21132785	21503381	EIF4G3	19.65025355	6156
chr1.fa	21543740	21672034	ECE1	3.308067334	836
chr1.fa	21766631	21811393	NBPF3	3.141496414	532

chr1.fa	21835858	21904905	ALPL	9.114920858	1064
chr1.fa	21922708	21995856	RAP1GAP	45.46162962	7351
chr1.fa	22004792	22109688	USP48	18.84408588	4396
chr1.fa	22138758	22151714	LDLRAD2	0	0
chr1.fa	22154356	22263750	HSPG2	2.141403722	1216
chr1.fa	22303418	22315847	CELA3B	0	0
chr1.fa	22328149	22339035	CELA3A	0	0
chr1.fa	22351707	22357715	HSPC157	11.29613258	608
chr1.fa	22379120	22419436	CDC42	54.10686052	7676
chr1.fa	22443798	22469519	WNT4	0	0
chr1.fa	22778344	22857650	ZBTB40	12.78148262	5168
chr1.fa	22890004	22930087	EPHA8	0	0
chr1.fa	22963118	22966175	C1QA	1.539391065	76
chr1.fa	22970118	22974603	C1QC	0	0
chr1.fa	22979682	22988029	C1QB	0	0
chr1.fa	23037331	23241823	EPHB2	0	0
chr1.fa	23189686	23189719	MIR4253	0	0
chr1.fa	23345941	23410184	KDM1A	44.06301211	6156
chr1.fa	23370798	23370865	MIR3115	0	0
chr1.fa	23410516	23495351	LUZP1	11.74981039	4484
chr1.fa	23518388	23521222	HTR1D	0	0
chr1.fa	23636276	23670853	HNRNPR	41.93050403	5168
chr1.fa	23685941	23694879	ZNF436	19.19323988	3724
chr1.fa	23695464	23698330	C1orf213	0.71365298	76
chr1.fa	23707555	23751261	TCEA3	78.636152	5608
chr1.fa	23755056	23810750	ASAP3	17.50417962	3268
chr1.fa	23832920	23857712	E2F2	0	0
chr1.fa	23884409	23886322	ID3	94.48173893	5472
chr1.fa	23953824	23967056	MDS2	0	0
chr1.fa	24018269	24022915	RPL11	2297.994397	65512
chr1.fa	24069856	24088549	TCEB3	27.62897838	6141
chr1.fa	24104876	24114722	C1orf128	44.64278566	3256
chr1.fa	24117646	24122029	LYPLA2	21.73250125	1587
chr1.fa	24122089	24127294	GALE	13.64502718	1140
chr1.fa	24128367	24151949	HMGCL	48.35071216	3496
chr1.fa	24171572	24194859	FUCA1	2.395151946	228
chr1.fa	24200460	24239817	CNR2	0	0
chr1.fa	24286301	24289949	PNRC2	47.61059464	5168
chr1.fa	24292937	24306953	SRSF10	9.776534325	2191
chr1.fa	24382531	24438665	MYOM3	3.496877362	912
chr1.fa	24446261	24469611	IL22RA1	1.208028354	152
chr1.fa	24480647	24513751	IL28RA	3.715487795	760
chr1.fa	24526730	24538180	LOC284632	0	0

chr1.fa	24645812	24690970	GRHL3	158.9491328	24458
chr1.fa	24683489	24741587	C1orf201	5.547100741	760
chr1.fa	24742245	24799473	NIPAL3	29.28045455	7144
chr1.fa	24829387	24862427	RCAN3	122.3609073	9348
chr1.fa	24882567	24935818	C1orf130	0	0
chr1.fa	24969594	24999772	SRRM1	44.6817041	7904
chr1.fa	25071760	25170815	CLIC4	16.77206818	3344
chr1.fa	25226002	25291501	RUNX3	0	0
chr1.fa	25548767	25559013	SYF2	25.6808325	2052
chr1.fa	25568740	25573985	C1orf63	3.461072398	228
chr1.fa	25598981	25656936	RHD	0	0
chr1.fa	25664811	25688739	TMEM50A	10.13947657	988
chr1.fa	25688853	25747363	RHCE	0	0
chr1.fa	25757388	25826698	TMEM57	3.433495904	608
chr1.fa	25870076	25895377	LDLRAP1	13.24494563	1748
chr1.fa	25943959	26111258	MAN1C1	0.527956427	76
chr1.fa	26126667	26144713	SEPN1	8.583406174	1672
chr1.fa	26146397	26159433	FAM54B	32.99794386	2699
chr1.fa	26146521	26150097	LOC646471	0.550640317	76
chr1.fa	26160497	26185848	C1orf135	0	0
chr1.fa	26187975	26197744	PAQR7	23.48227427	3192
chr1.fa	26210677	26233368	STMN1	4.77985151	760
chr1.fa	26232853	26232879	MIR3917	0	0
chr1.fa	26286258	26324648	PAFAH2	12.27153987	1976
chr1.fa	26348271	26362954	EXTL1	0	0
chr1.fa	26364514	26372604	SLC30A2	8.201338296	912
chr1.fa	26377798	26394121	TRIM63	0.958060778	76
chr1.fa	26437656	26452026	PDIK1L	8.474879326	1748
chr1.fa	26485511	26489119	GRRP1	0	0
chr1.fa	26496388	26497364	ZNF593	31.25128431	912
chr1.fa	26503981	26516375	CNKSR1	11.32993603	1292
chr1.fa	26517119	26529033	CATSPER4	0	0
chr1.fa	26560693	26605299	CCDC21	3.006727419	532
chr1.fa	26606213	26608013	SH3BGRL3	72.76080202	3648
chr1.fa	26608773	26644756	UBXN11	1.65147617	152
chr1.fa	26644411	26647014	CD52	3.52111799	76
chr1.fa	26648350	26680621	AIM1L	14.82325513	3496
chr1.fa	26688125	26699266	ZNF683	0	0
chr1.fa	26737269	26756219	LIN28A	0	0
chr1.fa	26758802	26797795	DHDDS	16.39444812	2432
chr1.fa	26798902	26803133	HMGN2	29.26444239	2591
chr1.fa	26856249	26901520	RPS6KA1	14.48855656	2204
chr1.fa	26881033	26881084	MIR1976	0	0

chr1.fa	27022522	27108601	ARID1A	45.08445435	17404
chr1.fa	27114486	27124887	PIGV	1.42930748	152
chr1.fa	27153201	27182211	ZDHHC18	5.887359095	831
chr1.fa	27189633	27190947	SFN	82.2593475	4864
chr1.fa	27205873	27216869	GPN2	14.55104845	988
chr1.fa	27216979	27226962	GPATCH3	4.76339457	456
chr1.fa	27237975	27240567	NR0B2	0	0
chr1.fa	27248224	27272887	NUDC	54.76825159	3268
chr1.fa	27276047	27286901	C1orf172	4.687114429	380
chr1.fa	27320195	27327377	TRNP1	1.734428043	152
chr1.fa	27331511	27339333	FAM46B	0.711873851	76
chr1.fa	27425300	27481451	SLC9A1	8.132841843	1672
chr1.fa	27561007	27635110	WDTC1	25.17533758	5436
chr1.fa	27648636	27662891	TMEM222	5.46681756	456
chr1.fa	27650365	27653016	LOC644961	0	0
chr1.fa	27668483	27680423	SYTL1	373.9021219	33054
chr1.fa	27681670	27693337	MAP3K6	4.295706126	836
chr1.fa	27695601	27701315	FCN3	0	0
chr1.fa	27705596	27709805	CD164L2	0	0
chr1.fa	27719152	27722317	GPR3	0	0
chr1.fa	27732126	27816669	WASF2	43.14498173	8284
chr1.fa	27860756	27930143	AHDC1	3.902741086	1140
chr1.fa	27938801	27961727	FGR	1.218925517	152
chr1.fa	27992572	27998724	IFI6	7.861302333	304
chr1.fa	28052490	28089423	FAM76A	3.908968036	608
chr1.fa	28099694	28150963	STX12	26.8977565	3724
chr1.fa	28157252	28178183	PPP1R8	13.63034937	1748
chr1.fa	28160912	28161077	SCARNA1	0	0
chr1.fa	28199055	28213193	C1orf38	0	0
chr1.fa	28218049	28241236	RPA2	21.61841462	1672
chr1.fa	28261504	28285663	SMPDL3B	0.902463008	76
chr1.fa	28286504	28294604	XKR8	0	0
chr1.fa	28300819	28415131	EYA3	13.96326882	1284
chr1.fa	28421446	28423069	LOC653566	0	0
chr1.fa	28473677	28520437	PTAFR	0.373617017	76
chr1.fa	28526790	28559542	DNAJC8	36.08184099	2862
chr1.fa	28562611	28564611	ATPIF1	142.9498736	3876
chr1.fa	28586006	28609002	SESN2	6.789822103	1064
chr1.fa	28655513	28662478	MED18	8.138846402	684
chr1.fa	28696093	28826881	PHACTR4	22.65008685	6612
chr1.fa	28832732	28832839	SNHG3	0	0
chr1.fa	28843237	28865708	RCC1	22.52754936	2812
chr1.fa	28879529	28905049	TRNAU1AP	8.421283076	760

chr1.fa	28905058	28908366	SNHG12	4.624177753	152
chr1.fa	28905255	28905334	SNORD99	0	0
chr1.fa	28906276	28906405	SNORA61	0	0
chr1.fa	28906893	28907024	SNORA44	0	0
chr1.fa	28907432	28907565	SNORA16A	0	0
chr1.fa	28918712	28921088	RAB42	0	0
chr1.fa	28929609	28969604	TAF12	8.159528773	532
chr1.fa	28975112	28975246	RNU11	0	0
chr1.fa	28995244	29041387	GMEB1	6.030134169	532
chr1.fa	29063133	29096287	YTHDF2	22.89983203	3154
chr1.fa	29138654	29190208	OPRD1	0	0
chr1.fa	29213603	29445936	EPB41	49.3172238	12434
chr1.fa	29446559	29450421	TMEM200B	0	0
chr1.fa	29474250	29508637	SRSF4	24.04981631	2736
chr1.fa	29519385	29557454	MECR	2.777664605	304
chr1.fa	29563028	29653325	PTPRU	2.996719821	760
chr1.fa	31184124	31196432	MATN1	0	0
chr1.fa	31191805	31199593	LOC100129196	0	0
chr1.fa	31205315	31230683	LAPTM5	0	0
chr1.fa	31342313	31381480	SDC3	2.310420944	532
chr1.fa	31404353	31538564	PUM1	50.51591173	12232
chr1.fa	31441010	31441084	SNORD85	0	0
chr1.fa	31460912	31461481	PRO0611	0	0
chr1.fa	31652592	31712734	NKAIN1	0	0
chr1.fa	31732415	31769644	SNRNP40	22.89649617	1672
chr1.fa	31769842	31837780	ZCCHC17	17.93561832	1292
chr1.fa	31838100	31845923	FABP3	10.78507788	532
chr1.fa	31882412	31907527	SERINC2	16.19207224	2584
chr1.fa	31971839	31974167	LOC149086	0	0
chr1.fa	31984036	31989846	LOC284551	0	0
chr1.fa	32042086	32053287	TINAGL1	3.777757298	380
chr1.fa	32083301	32092919	HCRTR1	0	0
chr1.fa	32095463	32110838	PEF1	20.5840737	2052
chr1.fa	32117848	32169768	COL16A1	0	0
chr1.fa	32192718	32229648	BAI2	0	0
chr1.fa	32224261	32224336	MIR4254	0	0
chr1.fa	32256025	32281580	SPOCD1	0	0
chr1.fa	32372022	32403988	PTP4A2	80.01319758	14136
chr1.fa	32479491	32509472	KHDRBS1	39.02807803	4712
chr1.fa	32538503	32568467	TMEM39B	2.789006551	228
chr1.fa	32573644	32642168	KPNA6	22.69767854	7524
chr1.fa	32645345	32663886	TXLNA	27.48820482	5928
chr1.fa	32666202	32670991	CCDC28B	0	0

chr1.fa	32671236	32674288	IQCC	2.469875349	228
chr1.fa	32674695	32681797	DCDC2B	0	0
chr1.fa	32681798	32687926	C1orf91	0	0
chr1.fa	32687971	32697205	EIF3I	97.3152237	6380
chr1.fa	32697261	32707311	MTMR9LP	0	0
chr1.fa	32712818	32714461	FAM167B	0	0
chr1.fa	32716840	32751766	LCK	2.27061294	228
chr1.fa	32757708	32799224	HDAC1	53.97587217	5075
chr1.fa	32799440	32801834	MARCKSL1	110.4187286	7676
chr1.fa	32826871	32827844	LOC100128071	2.418058227	76
chr1.fa	32827862	32829924	TSSK3	0	0
chr1.fa	32830705	32860062	BSDC1	39.62275178	5092
chr1.fa	32930658	32953459	ZBTB8B	0.420541535	76
chr1.fa	33004772	33071542	ZBTB8A	2.074464006	684
chr1.fa	33087307	33116185	ZBTB8OS	18.22672824	586
chr1.fa	33116749	33151812	RBBP4	15.58027437	4104
chr1.fa	33160466	33168361	SYNC	1.267851554	76
chr1.fa	33207512	33240571	KIAA1522	345.1347236	87001
chr1.fa	33240840	33283633	YARS	24.77258733	3344
chr1.fa	33290914	33324476	S100PBP	4.779629119	948
chr1.fa	33327869	33338082	FNDC5	0.531514684	76
chr1.fa	33352098	33360195	HPCA	0	0
chr1.fa	33360248	33366953	TMEM54	76.92707654	3255
chr1.fa	33402050	33430286	RNF19B	21.42738068	2508
chr1.fa	33473541	33502512	AK2	16.40089746	4256
chr1.fa	33546714	33585995	ADC	0	0
chr1.fa	33611003	33647671	TRIM62	9.680683769	1672
chr1.fa	33722174	33766320	ZNF362	16.32483971	2280
chr1.fa	33789224	33841194	PHC2	25.98395155	4864
chr1.fa	33798006	33798093	MIR3605	0	0
chr1.fa	33938232	33961995	ZSCAN20	1.602772523	304
chr1.fa	33979609	34631443	CSMD2	0	0
chr1.fa	34326076	34330392	HMGB4	0	0
chr1.fa	34632624	34684731	C1orf94	0	0
chr1.fa	35220721	35224112	GJB5	0	0
chr1.fa	35225342	35229325	GJB4	0	0
chr1.fa	35246790	35251965	GJB3	0	0
chr1.fa	35258599	35261348	GJA4	0	0
chr1.fa	35315963	35325417	C1orf212	4.243221831	1216
chr1.fa	35331037	35370984	DLGAP3	0	0
chr1.fa	35447127	35450948	LOC100130633	5.481717763	228
chr1.fa	35451767	35497569	ZMYM6	6.242962434	1444
chr1.fa	35544972	35581455	ZMYM1	4.458496398	836

chr1.fa	35649201	35658743	SFPQ	57.12114923	7893
chr1.fa	35734568	35887545	ZMYM4	21.87972414	6881
chr1.fa	35899091	36023037	KIAA0319L	8.781111845	1900
chr1.fa	36023393	36032380	NCDN	5.908041466	912
chr1.fa	36038971	36060927	TFAP2E	0	0
chr1.fa	36065143	36107445	PSMB2	18.83785893	4104
chr1.fa	36179477	36184790	C1orf216	1.728201093	228
chr1.fa	36197713	36235551	CLSPN	0	0
chr1.fa	36273828	36321188	EIF2C4	15.27470903	3268
chr1.fa	36348810	36389899	EIF2C1	23.05394905	7752
chr1.fa	36396772	36522063	EIF2C3	6.178913803	988
chr1.fa	36549676	36553876	TEKT2	0	0
chr1.fa	36554453	36559533	ADPRHL2	10.99457028	836
chr1.fa	36560844	36565850	COL8A2	0	0
chr1.fa	36602173	36615067	TRAPPC3	42.85698528	2432
chr1.fa	36621803	36646441	MAP7D1	27.89251181	4169
chr1.fa	36690017	36770957	THRAP3	72.09273922	14364
chr1.fa	36771994	36786948	C1orf113	0	0
chr1.fa	36787632	36789755	FAM176B	0	0
chr1.fa	36805225	36851485	STK40	20.65479407	3572
chr1.fa	36859031	36863493	LSM10	25.84962733	988
chr1.fa	36883507	36916086	OSCP1	3.045423467	228
chr1.fa	36921362	36930040	MRPS15	55.21748158	2396
chr1.fa	36931644	36948915	CSF3R	0	0
chr1.fa	37261128	37499844	GRIK3	0	0
chr1.fa	37627164	37627235	MIR4255	0	0
chr1.fa	37940119	37949978	ZC3H12A	16.17984073	1976
chr1.fa	37958176	37980364	MEAF6	66.58277779	6428
chr1.fa	38002142	38019903	SNIP1	9.57593757	1064
chr1.fa	38022520	38032412	DNALI1	18.18091568	2128
chr1.fa	38032459	38061586	GNL2	22.95654176	2432
chr1.fa	38076951	38100491	RSPO1	0	0
chr1.fa	38147242	38156192	C1orf109	3.588502488	380
chr1.fa	38158159	38175391	CDCA8	0	0
chr1.fa	38181646	38230824	EPHA10	0.254415397	76
chr1.fa	38259774	38267278	MANEAL	1.08793717	152
chr1.fa	38268614	38273472	YRDC	8.199114385	532
chr1.fa	38273866	38275126	C1orf122	51.2173332	1824
chr1.fa	38275239	38325292	MTF1	6.996423418	2508
chr1.fa	38326369	38412729	INPP5B	2.656239075	532
chr1.fa	38422652	38455761	SF3A3	16.60883312	2118
chr1.fa	38462442	38471234	FHL3	0	0
chr1.fa	38478384	38490497	UTP11L	3.297837345	304

chr1.fa	38509523	38512450	POU3F1	0	0
chr1.fa	38554903	38555001	MIR3659	0	0
chr1.fa	39305015	39325340	RRAGC	11.80296186	760
chr1.fa	39338963	39339050	MYCBP	47.7635997	189
chr1.fa	39339267	39339581	GJA9-MYCBP	0	0
chr1.fa	39339739	39340449	GJA9	0	0
chr1.fa	39351479	39407456	RHBDL2	4.600381908	380
chr1.fa	39456916	39471737	AKIRIN1	27.28093633	3344
chr1.fa	39491967	39500308	NDUFS5	201.2814975	5168
chr1.fa	39547118	39952789	MACF1	8.938119948	9688
chr1.fa	39875176	39882154	KIAA0754	7.991845898	2508
chr1.fa	39957318	39995541	BMP8A	0	0
chr1.fa	39987952	40025370	PPIEL	0	0
chr1.fa	40026485	40042521	PABPC4	62.24726366	8968
chr1.fa	40033046	40033182	SNORA55	0	0
chr1.fa	40089103	40105348	HEYL	0.410756327	76
chr1.fa	40124793	40137710	NT5C1A	1.526714773	76
chr1.fa	40144645	40157089	HPCAL4	0	0
chr1.fa	40204517	40229586	PPIE	7.888211654	1649
chr1.fa	40223903	40254533	BMP8B	0	0
chr1.fa	40235197	40237020	OXCT2	0.926703636	76
chr1.fa	40306706	40349177	TRIT1	14.29663305	1368
chr1.fa	40361096	40367687	MYCL1	48.63804144	10032
chr1.fa	40420784	40435628	MFSD2A	0	0
chr1.fa	40506255	40538321	CAP1	64.60816737	8056
chr1.fa	40538382	40563142	PPT1	6.749791709	760
chr1.fa	40627041	40706593	RLF	11.62838486	3268
chr1.fa	40713573	40717365	TMCO2	0	0
chr1.fa	40723733	40759856	ZMPSTE24	9.110028254	1292
chr1.fa	40766163	40782939	COL9A2	4.705572889	599
chr1.fa	40839378	40888998	SMAP2	18.16267961	2660
chr1.fa	40916337	40929390	ZNF643	1.664374852	152
chr1.fa	40943302	40962015	ZNF642	2.348449819	228
chr1.fa	40974433	40982214	DEM1	3.836023761	380
chr1.fa	40997233	41013841	ZNF684	1.611890557	152
chr1.fa	41086352	41131324	RIMS3	1.402842941	456
chr1.fa	41154752	41157933	LOC100130557	0	0
chr1.fa	41161278	41237275	NFYC	10.88092843	1140
chr1.fa	41220027	41220118	MIR30E	0	0
chr1.fa	41222956	41223044	MIR30C1	0	0
chr1.fa	41249684	41306124	KCNQ4	0	0
chr1.fa	41326728	41328018	CITED4	5.236865182	304
chr1.fa	41445007	41478235	CTPS	7.925128573	1140

chr1.fa	41481269	41487427	SLFNL1	0	0
chr1.fa	41492871	41707815	SCMH1	29.17237248	4986
chr1.fa	41944446	41950344	EDN2	0	0
chr1.fa	41975684	42384496	HIVEP3	1.143090158	456
chr1.fa	42619092	42621495	GUCA2B	0	0
chr1.fa	42628362	42630395	GUCA2A	0	0
chr1.fa	42642210	42801548	FOXJ3	16.42936352	4256
chr1.fa	42846468	42889900	RIMKLA	0.959617516	456
chr1.fa	42896001	42921938	ZMYND12	6.669286137	532
chr1.fa	42922173	42926086	PPCS	44.92544472	2109
chr1.fa	43000560	43120335	CCDC30	1.091050645	152
chr1.fa	43124048	43142429	PPIH	12.67629163	456
chr1.fa	43148066	43168020	YBX1	190.7730741	13262
chr1.fa	43198764	43205925	CLDN19	2.364684367	304
chr1.fa	43212006	43232755	LEPRE1	0	0
chr1.fa	43232916	43241415	C1orf50	11.63349985	532
chr1.fa	43272723	43283059	CCDC23	9.089568275	300
chr1.fa	43282776	43310660	ERMAP	4.673103791	722
chr1.fa	43312280	43318146	ZNF691	6.438666586	456
chr1.fa	43391046	43424719	SLC2A1	5.726125561	912
chr1.fa	43424848	43449029	FLJ32224	0	0
chr1.fa	43613594	43622067	FAM183A	0	0
chr1.fa	43629845	43638241	EBNA1BP2	30.61902646	1900
chr1.fa	43638015	43720029	WDR65	0.826182867	152
chr1.fa	43735665	43739673	TMEM125	26.49767495	2128
chr1.fa	43747557	43751288	C1orf210	8.936785602	608
chr1.fa	43766664	43788779	TIE1	0	0
chr1.fa	43803475	43820135	MPL	0	0
chr1.fa	43824626	43828873	CDC20	1.009655509	76
chr1.fa	43829072	43833699	ELOVL1	23.93372817	1596
chr1.fa	43849588	43855483	MED8	12.15945476	988
chr1.fa	43855556	43872565	C1orf84	2.845493885	152
chr1.fa	43888797	43918304	KIAA0467	3.34209317	1444
chr1.fa	43917099	43919660	HYI	23.55766485	661
chr1.fa	43996547	44089343	PTPRF	21.63798504	7524
chr1.fa	44115797	44171189	KDM4A	34.44949045	6774
chr1.fa	44165409	44173012	LOC100132774	0	0
chr1.fa	44173218	44396831	ST3GAL3	3.513779085	380
chr1.fa	44398992	44402912	ARTN	0	0
chr1.fa	44412478	44433694	IPO13	13.82827744	2504
chr1.fa	44435653	44439043	DPH2	8.799347914	988
chr1.fa	44440602	44443972	ATP6V0B	18.19159045	836
chr1.fa	44444866	44456840	B4GALT2	18.26809298	1900

chr1.fa	44457280	44462154	CCDC24	10.48396035	668
chr1.fa	44462199	44497134	SLC6A9	0	0
chr1.fa	44584522	44600809	KLF17	0	0
chr1.fa	44679125	44686351	DMAP1	8.613651361	684
chr1.fa	44686742	44820939	ERI3	26.18165722	2052
chr1.fa	44870960	45117396	RNF220	16.28414215	2204
chr1.fa	45119501	45140099	TMEM53	5.281788181	380
chr1.fa	45140394	45191263	C1orf228	0	0
chr1.fa	45205490	45233438	KIF2C	0	0
chr1.fa	45241246	45244412	RPS8	2056.975617	65208
chr1.fa	45241537	45241610	SNORD55	0	0
chr1.fa	45242164	45242261	SNORD46	0	0
chr1.fa	45243514	45243584	SNORD38A	0	0
chr1.fa	45244062	45244130	SNORD38B	0	0
chr1.fa	45249257	45253426	BEST4	0	0
chr1.fa	45266036	45271585	PLK3	2.227913852	228
chr1.fa	45271668	45272957	TCTEX1D4	0	0
chr1.fa	45274154	45279801	BTBD19	0	0
chr1.fa	45285516	45308616	PTCH2	0	0
chr1.fa	45316194	45452361	EIF2B3	10.99545984	1064
chr1.fa	45468220	45477027	HECTD3	17.65407121	2880
chr1.fa	45477805	45481341	UROD	25.50336442	1672
chr1.fa	45482076	45672250	ZSWIM5	0.579328767	152
chr1.fa	45769582	45771291	LOC400752	0.988305965	76
chr1.fa	45792545	45794346	HPDL	1.875868771	152
chr1.fa	45794914	45806142	MUTYH	6.447562229	532
chr1.fa	45805342	45809554	TOE1	5.042717768	456
chr1.fa	45809651	45956840	TESK2	26.65712935	3572
chr1.fa	45959598	45965751	CCDC163P	0	0
chr1.fa	45965856	45976706	MMACHC	4.19985557	532
chr1.fa	45976740	45987609	PRDX1	263.1658189	14212
chr1.fa	46016498	46035721	AKR1A1	83.68287281	5776
chr1.fa	46049660	46084578	NASP	10.78507788	1596
chr1.fa	46085716	46089731	CCDC17	0	0
chr1.fa	46092976	46152302	GPBP1L1	47.02459414	7904
chr1.fa	46111452	46112357	RPS15AP10	0	0
chr1.fa	46153847	46159997	TMEM69	18.64171	1140
chr1.fa	46160109	46216485	IPP	11.66685852	1748
chr1.fa	46269285	46501796	MAST2	3.240238055	836
chr1.fa	46505812	46598708	PIK3R3	17.96230525	4788
chr1.fa	46640749	46651634	TSPAN1	15.56337265	1140
chr1.fa	46654353	46664121	POMGNT1	15.8664917	1952
chr1.fa	46669006	46686928	C1orf190	0	0

chr1.fa	46713367	46744071	RAD54L	0	0
chr1.fa	46744146	46769038	LRRC41	34.10923209	4408
chr1.fa	46769380	46782447	UQCRH	234.905917	5556
chr1.fa	46806390	46830690	NSUN4	8.763320559	1748
chr1.fa	46859939	46879520	FAAH	6.45401157	608
chr1.fa	46972668	46979886	DMBX1	0	0
chr1.fa	47011316	47016887	KNCN	0	0
chr1.fa	47023090	47069966	MKNK1	4.662651411	608
chr1.fa	47073387	47082563	MOBKL2C	5.662299321	760
chr1.fa	47098411	47134099	ATPAF1	20.70416489	3844
chr1.fa	47137500	47139251	C1orf223	0	0
chr1.fa	47140831	47184736	KIAA0494	40.44448683	10488
chr1.fa	47264670	47285021	CYP4B1	3.132823162	304
chr1.fa	47323906	47366147	CYP4Z2P	0	0
chr1.fa	47394846	47407156	CYP4A11	5.960748152	684
chr1.fa	47489240	47516423	CYP4X1	4.30260025	456
chr1.fa	47533160	47583992	CYP4Z1	0	0
chr1.fa	47603107	47614526	CYP4A22	0	0
chr1.fa	47649261	47655771	PDZK1IP1	139.9024486	5624
chr1.fa	47681963	47695443	TAL1	0	0
chr1.fa	47715811	47779819	STIL	1.347912344	304
chr1.fa	47799469	47844511	CMPK1	62.29997034	8208
chr1.fa	47881744	47883724	FOXE3	0	0
chr1.fa	47897807	47900313	MGC12982	0	0
chr1.fa	47901689	47906363	FOXD2	0	0
chr1.fa	48226200	48462562	LOC388630	0.240404759	76
chr1.fa	48567387	48648100	SKINTL	0	0
chr1.fa	48688357	48714316	SLC5A9	0	0
chr1.fa	48761044	48937876	SPATA6	3.03986369	684
chr1.fa	48998527	50489626	AGBL4	0	0
chr1.fa	49193540	49242547	BEND5	1.272744158	76
chr1.fa	50513686	50667540	ELAVL4	0	0
chr1.fa	50883227	50889141	DMRTA2	0	0
chr1.fa	50906935	51425936	FAF1	26.17387353	3040
chr1.fa	51434367	51440306	CDKN2C	2.731629652	304
chr1.fa	51567906	51613754	C1orf185	0	0
chr1.fa	51701945	51739119	RNF11	31.34024074	4332
chr1.fa	51752930	51810785	TTC39A	17.70433159	2280
chr1.fa	51819935	51984995	EPS15	44.89564432	10538
chr1.fa	52082546	52254865	OSBPL9	34.80375944	6623
chr1.fa	52254892	52344609	NRD1	27.44550573	4892
chr1.fa	52302016	52302040	MIR761	0	0
chr1.fa	52373628	52456436	RAB3B	20.39682041	11780

chr1.fa	52485804	52521047	TXNDC12	9.374006468	674
chr1.fa	52497777	52499472	KTI12	0.996534435	76
chr1.fa	52521857	52554090	BTF3L4	11.53208952	1216
chr1.fa	52608046	52812358	ZFYVE9	32.96725389	7286
chr1.fa	52816265	52831877	CC2D1B	7.7723459	1976
chr1.fa	52838501	52870143	ORC1	0	0
chr1.fa	52870219	52883992	PRPF38A	16.83989746	2099
chr1.fa	52888948	53018762	ZCCHC11	10.11212247	2660
chr1.fa	53068043	53074723	GPX7	0	0
chr1.fa	53099066	53122737	FAM159A	0	0
chr1.fa	53152014	53164038	C1orf163	7.995181764	760
chr1.fa	53192131	53293013	ZYG11B	17.35806868	6355
chr1.fa	53308183	53360247	ZYG11A	2.770103309	532
chr1.fa	53361582	53387446	ECHDC2	30.5334059	2183
chr1.fa	53392901	53517289	SCP2	90.69953381	15090
chr1.fa	53527724	53551174	PODN	0	0
chr1.fa	53552855	53608289	SLC1A7	0	0
chr1.fa	53662101	53679771	CPT2	14.10359759	1900
chr1.fa	53679870	53686289	C1orf123	47.18471572	2092
chr1.fa	53692564	53704207	MAGOH	14.53214521	445
chr1.fa	53711212	53793726	LRP8	0.374506581	76
chr1.fa	53904043	53905693	FLJ40434	0	0
chr1.fa	53925072	53933158	DMRTB1	0	0
chr1.fa	53971906	54199877	GLIS1	0	0
chr1.fa	54231134	54304225	TMEM48	1.401286203	304
chr1.fa	54317392	54355487	YIPF1	15.05209556	1288
chr1.fa	54359861	54376759	DIO1	8.252933027	619
chr1.fa	54387234	54411288	HSPB11	19.31577737	522
chr1.fa	54412037	54433839	LRRC42	18.73578143	1444
chr1.fa	54474506	54483803	LDLRAD1	0	0
chr1.fa	54497349	54519111	TMEM59	17.93806462	1368
chr1.fa	54519274	54565416	C1orf83	4.125354558	760
chr1.fa	54604668	54619443	CDCP2	0	0
chr1.fa	54638027	54665746	CYB5RL	1.435089648	228
chr1.fa	54665840	54684056	MRPL37	44.44819346	2964
chr1.fa	54692195	54871979	SSBP3	18.74356511	1748
chr1.fa	55013807	55100417	ACOT11	12.87266296	1520
chr1.fa	55076085	55089200	FAM151A	5.559777032	304
chr1.fa	55117517	55181283	C1orf175-TTC4	0	0
chr1.fa	55175619	55175939	C1orf175	0	0
chr1.fa	55181495	55181692	TTC4	17.07251853	152
chr1.fa	55222571	55230226	PARS2	5.666969534	608
chr1.fa	55246752	55266941	TTC22	3.300061255	456

chr1.fa	55271736	55307937	C1orf177	0	0
chr1.fa	55315300	55352921	DHCR24	20.50601443	3952
chr1.fa	55446465	55457966	TMEM61	1.540725411	76
chr1.fa	55464617	55474465	BSND	0	0
chr1.fa	55505149	55530526	PCSK9	0	0
chr1.fa	55532032	55681039	USP24	14.69560265	7138
chr1.fa	56960419	57045257	PPAP2B	4.598380388	684
chr1.fa	57110990	57181008	PRKAA2	1.44576442	608
chr1.fa	57184477	57285369	C1orf168	3.907856081	608
chr1.fa	57320443	57383894	C8A	0	0
chr1.fa	57394883	57431688	C8B	0	0
chr1.fa	57463579	58716211	DAB1	7.154543476	830
chr1.fa	58946391	59012446	OMA1	22.13436193	1900
chr1.fa	59041095	59043166	TACSTD2	21.20877025	1976
chr1.fa	59125590	59165747	MYSM1	12.13743804	1413
chr1.fa	59246463	59249785	JUN	23.3968761	3496
chr1.fa	59250823	59365384	LOC100131060	1.505142838	76
chr1.fa	59597608	59612479	LOC729467	0	0
chr1.fa	59762625	60228402	FGGY	28.35864351	2660
chr1.fa	60280533	60342050	HOOK1	19.91156307	5244
chr1.fa	60358980	60392423	CYP2J2	0.910246696	76
chr1.fa	60456066	60539442	C1orf87	0	0
chr1.fa	61542946	61928460	NFIA	13.49024299	6080
chr1.fa	62146719	62191095	TM2D1	1.352137774	76
chr1.fa	62208149	62629591	INADL	64.9964622	24854
chr1.fa	62660474	62678001	L1TD1	0	0
chr1.fa	62701837	62785083	KANK4	0	0
chr1.fa	62901975	62917475	USP1	6.844530309	1292
chr1.fa	62920397	63153969	DOCK7	10.0663099	3192
chr1.fa	63063187	63071180	ANGPTL3	3.179970071	304
chr1.fa	63249803	63330050	ATG4C	3.741285161	304
chr1.fa	63788730	63790797	FOXD3	0	0
chr1.fa	63833261	63904233	ALG6	1.002761386	152
chr1.fa	63906462	63988835	ITGB3BP	1.922793289	76
chr1.fa	63989013	64038364	EFCAB7	1.431753781	152
chr1.fa	64014651	64016307	DLEU2L	0	0
chr1.fa	64058947	64125916	PGM1	18.65082803	2812
chr1.fa	64239690	64644707	ROR1	0	0
chr1.fa	64669490	64710027	UBE2U	0	0
chr1.fa	64936476	65158741	CACHD1	1.602327741	380
chr1.fa	65210778	65298905	RAVER2	3.883615453	760
chr1.fa	65298915	65432187	JAK1	29.15235728	6612
chr1.fa	65523438	65523525	MIR3671	0	0

chr1.fa	65524117	65524191	MIR101-1	0	0
chr1.fa	65613232	65697828	AK4	18.01034172	5852
chr1.fa	65730430	65881552	DNAJC6	1.175781647	304
chr1.fa	65886131	65901690	LEPROT	3.116366222	698
chr1.fa	65991372	66103176	LEPR	0.502603844	152
chr1.fa	66258193	66840262	PDE4B	7.781241544	1824
chr1.fa	66999825	67210768	SGIP1	0	0
chr1.fa	67094123	67094200	MIR3117	0	0
chr1.fa	67218140	67244730	TCTEX1D1	0	0
chr1.fa	67263424	67266939	INSL5	0	0
chr1.fa	67278572	67390570	WDR78	2.991382435	532
chr1.fa	67390578	67454302	MIER1	7.279749655	1894
chr1.fa	67465015	67520080	SLC35D1	3.401249197	988
chr1.fa	67557859	67594220	C1orf141	0	0
chr1.fa	67632169	67725650	IL23R	0	0
chr1.fa	67773047	67862583	IL12RB2	0	0
chr1.fa	67873493	67896123	SERBP1	24.65205136	7448
chr1.fa	68150860	68154021	GADD45A	12.24752163	760
chr1.fa	68167149	68299155	GNG12	20.70060663	4104
chr1.fa	68511645	68516460	DIRAS3	0	0
chr1.fa	68564142	68698229	WLS	6.135992324	836
chr1.fa	68649201	68649293	MIR1262	0	0
chr1.fa	68894507	68915642	RPE65	0	0
chr1.fa	68939835	68962799	DEPDC1	0	0
chr1.fa	70225858	70589171	LRRC7	0	0
chr1.fa	70385005	70386000	PIN1P1	0	0
chr1.fa	70610485	70671361	LRRC40	4.017494884	532
chr1.fa	70671365	70717701	SRSF11	22.39834014	2812
chr1.fa	70724685	70820417	ANKRD13C	7.389388458	1883
chr1.fa	70820493	70833705	HHLA3	5.937397089	228
chr1.fa	70876901	70905534	CTH	3.159287701	304
chr1.fa	71318036	71513491	PTGER3	0.657610427	304
chr1.fa	71528974	71546972	ZRANB2	13.3081047	1882
chr1.fa	71533314	71533399	MIR186	0	0
chr1.fa	71868625	72748277	NEGR1	0	0
chr1.fa	74491702	74663871	LRRIQ3	0	0
chr1.fa	74670075	74674148	FPGT	2.074241615	380
chr1.fa	74701071	74701175	TNNI3K	0	0
chr1.fa	74930319	74930609	FPGT-TNNI3K	0	0
chr1.fa	75033795	75139422	C1orf173	0	0
chr1.fa	75171172	75198835	CRYZ	43.69562204	4020
chr1.fa	75198840	75232360	TYW3	9.535462393	1520
chr1.fa	75594119	75627218	LHX8	0	0

chr1.fa	75667816	76076799	SLC44A5	0.400081555	76
chr1.fa	76190043	76229355	ACADM	30.3439287	3568
chr1.fa	76251886	76260764	RABGGTB	31.63424175	2128
chr1.fa	76252757	76252834	SNORD45C	0	0
chr1.fa	76253574	76253657	SNORD45A	0	0
chr1.fa	76255162	76255232	SNORD45B	0	0
chr1.fa	76262630	76378923	MSH4	0	0
chr1.fa	76384558	76398116	ASB17	0	0
chr1.fa	76540389	77096669	ST6GALNAC3	0	0
chr1.fa	77333186	77529737	ST6GALNAC5	0	0
chr1.fa	77554667	77685132	PIGK	0.365388547	76
chr1.fa	77747742	78025654	AK5	2.383365218	456
chr1.fa	78030190	78148343	ZZZ3	17.98476674	3496
chr1.fa	78161674	78225537	USP33	17.64450839	3629
chr1.fa	78245309	78344081	FAM73A	2.890194493	684
chr1.fa	78354200	78409578	NEXN	0	0
chr1.fa	78413591	78444777	FUBP1	30.91413943	4009
chr1.fa	78470636	78482995	DNAJB4	1.511147397	152
chr1.fa	78511589	78603112	GIPC2	2.993606345	380
chr1.fa	78695283	78835147	MGC27382	1.41907749	76
chr1.fa	78956728	79006386	PTGFR	0	0
chr1.fa	79086088	79111830	IFI44L	0.575548118	152
chr1.fa	79115477	79129763	IFI44	3.916974115	304
chr1.fa	79355449	79472495	ELTD1	0	0
chr1.fa	82266082	82458107	LPHN2	4.146926493	1064
chr1.fa	84335057	84464833	TTLL7	6.486480668	1064
chr1.fa	84543745	84704181	PRKACB	13.26095779	3344
chr1.fa	84764049	84816481	SAMD13	7.499472044	608
chr1.fa	84830641	84863576	UOX	0	0
chr1.fa	84864215	84880691	DNASE2B	7.539724829	456
chr1.fa	84944920	84964005	RPF1	12.10763764	1060
chr1.fa	84964034	84972262	GNG5	55.09027388	1900
chr1.fa	84971985	85031877	SPATA1	0	0
chr1.fa	85018808	85040163	CTBS	6.887007006	912
chr1.fa	85093913	85100703	C1orf180	0	0
chr1.fa	85109390	85156240	SSX2IP	3.040975646	836
chr1.fa	85279086	85358896	LPAR3	12.7670272	988
chr1.fa	85391266	85462796	MCOLN2	0	0
chr1.fa	85483765	85514169	MCOLN3	0	0
chr1.fa	85527993	85598821	WDR63	1.124186916	152
chr1.fa	85623356	85666728	SYDE2	5.74035859	1216
chr1.fa	85715637	85725355	C1orf52	1.495802413	228
chr1.fa	85731460	85742587	BCL10	10.34585549	1444

chr1.fa	85784168	86044046	DDAH1		44.87518434	9044
chr1.fa	86046444	86049648	CYR61		2.211234521	228
chr1.fa	86115106	86174116	ZNHIT6		16.33217862	4551
chr1.fa	86194916	86622121	COL24A1		0	0
chr1.fa	86812507	86862025	ODF2L		2.353342422	684
chr1.fa	86889769	86922240	CLCA2		0	0
chr1.fa	86934526	86965974	CLCA1		0	0
chr1.fa	87012759	87046432	CLCA4		0	0
chr1.fa	87099959	87121059	CLCA3P		0	0
chr1.fa	87170257	87213867	SH3GLB1		13.52560317	3876
chr1.fa	87328128	87380107		15-Sep	14.69715939	1216
chr1.fa	87380335	87575681	HS2ST1		2.09381203	684
chr1.fa	87595448	87634886	LOC339524		0	0
chr1.fa	87794151	87814607	LMO4		1.25050505	304
chr1.fa	89149922	89301938	PKN2		10.20508194	2801
chr1.fa	89318321	89357301	GTF2B		30.82384865	2280
chr1.fa	89401456	89435150	CCBL2		19.94358739	1503
chr1.fa	89445139	89449749	RBMXL1		3.052984764	633
chr1.fa	89472360	89488549	GBP3		24.46301894	3344
chr1.fa	89517987	89531043	GBP1		7.203914297	988
chr1.fa	89573310	89591799	GBP2		9.769862593	1140
chr1.fa	89597434	89641723	GBP7		0	0
chr1.fa	89646831	89664633	GBP4		6.876109843	1900
chr1.fa	89724634	89738544	GBP5		0	0
chr1.fa	89829436	89853719	GBP6		0.347374869	76
chr1.fa	89873238	89890493	LOC400759		0	0
chr1.fa	89990397	90063420	LRRC8B		1.677273535	608
chr1.fa	90090408	90098453	FLJ27354		0	0
chr1.fa	90098644	90185094	LRRC8C		0.703645381	228
chr1.fa	90286573	90401989	LRRC8D		1.668377892	304
chr1.fa	90458824	90460525	GEMIN8P4		1.986174747	152
chr1.fa	90460678	90494094	ZNF326		5.588465482	684
chr1.fa	91177579	91182794	BARHL2		0	0
chr1.fa	91380857	91487671	ZNF644		8.704831704	2280
chr1.fa	91726323	91870426	HFM1		0	0
chr1.fa	91966404	91991321	CDC7		0.494820156	76
chr1.fa	92100568	92109335	HSP90B3P		0	0
chr1.fa	92145900	92371559	TGFBR3		4.723141785	1444
chr1.fa	92414928	92479985	BRDT		0	0
chr1.fa	92495533	92529093	EPHX4		0	0
chr1.fa	92545862	92613401	BTBD8		3.504216268	228
chr1.fa	92632609	92650280	KIAA1107		2.596860656	532
chr1.fa	92683573	92711367	C1orf146		0	0

chr1.fa	92711955	92764521	GLMN	4.253007039	380
chr1.fa	92764567	92853732	RPAP2	7.686058161	1060
chr1.fa	92940318	92952433	GFI1	0.580885504	76
chr1.fa	92974253	93257961	EVI5	10.63429673	3540
chr1.fa	93297594	93307481	RPL5	1100.005444	50996
chr1.fa	93302846	93302940	SNORD21	0	0
chr1.fa	93306276	93306408	SNORA66	0	0
chr1.fa	93307721	93427079	FAM69A	1.300543043	152
chr1.fa	93544792	93604638	MTF2	6.141552101	1140
chr1.fa	93615299	93645919	TMED5	2.596638265	684
chr1.fa	93646247	93744268	CCDC18	0.780815086	152
chr1.fa	93775666	93811368	LOC100131564	0.838191985	152
chr1.fa	93811478	93828148	DR1	14.22524552	2052
chr1.fa	93913688	94020218	FNBP1L	26.30641862	6526
chr1.fa	94027349	94147385	BCAR3	6.81584186	932
chr1.fa	94057525	94065587	LOC100129046	0	0
chr1.fa	94312388	94312467	MIR760	0	0
chr1.fa	94335014	94344762	DNTTIP2	17.38608995	2128
chr1.fa	94352590	94375012	GCLM	10.44682104	1444
chr1.fa	94458394	94586705	ABCA4	0	0
chr1.fa	94634463	94703307	ARHGAP29	9.269260269	3800
chr1.fa	94883933	94984219	ABCD3	30.20471188	5157
chr1.fa	94994732	95007413	F3	2.825256297	304
chr1.fa	95285901	95360802	SLC44A3	6.568987759	760
chr1.fa	95362507	95392735	CNN3	63.81089535	5928
chr1.fa	95393584	95428826	LOC729970	12.42765841	532
chr1.fa	95448279	95538507	ALG14	4.796975624	228
chr1.fa	95558073	95663161	TMEM56	0.059823201	18
chr1.fa	95583479	95710509	TMEM56-RWDD3	0	0
chr1.fa	95699711	95712781	RWDD3	4.245668133	168
chr1.fa	95940293	95944912	FLJ31662	0	0
chr1.fa	97187175	97280605	PTBP2	1.454437672	228
chr1.fa	97543300	98386615	DPYD	1.504698056	380
chr1.fa	98511626	98511727	MIR137	0	0
chr1.fa	99127236	99226056	SNX7	9.800774953	836
chr1.fa	99355801	99470449	LPPR5	0	0
chr1.fa	99469832	99614408	LOC100129620	0	0
chr1.fa	99729848	99775138	LPPR4	0	0
chr1.fa	100111431	100160097	PALMD	13.59343245	1566
chr1.fa	100174259	100231349	FRRS1	3.786875332	456
chr1.fa	100315640	100389579	AGL	7.199244084	2660
chr1.fa	100435540	100489006	SLC35A3	2.359569373	228
chr1.fa	100503789	100548929	HIAT1	7.691395547	912

chr1.fa	100549102	100598511	SASS6	1.734872825	304
chr1.fa	100598706	100616054	CCDC76	3.969680801	518
chr1.fa	100617941	100643771	LRRC39	1.46844831	76
chr1.fa	100652478	100715409	DBT	7.501473563	3648
chr1.fa	100731714	100758325	RTCD1	12.75168221	1504
chr1.fa	100746797	100746864	MIR553	0	0
chr1.fa	100818023	100985833	CDC14A	3.166626607	608
chr1.fa	101003728	101007583	GPR88	0	0
chr1.fa	101185196	101204601	VCAM1	1.055468072	152
chr1.fa	101337941	101360418	EXTL2	1.185566854	152
chr1.fa	101361632	101447311	SLC30A7	1.925461982	684
chr1.fa	101455180	101491362	DPH5	12.15945476	988
chr1.fa	101702305	101707076	S1PR1	0	0
chr1.fa	102268127	102462790	OLFM3	0	0
chr1.fa	102337567	102360299	HEJ1	0	0
chr1.fa	103342023	103574052	COL11A1	0	0
chr1.fa	104068578	104097859	RNPC3	5.605811986	456
chr1.fa	104097322	104122149	AMY2B	4.77985151	452
chr1.fa	104112026	104114008	LOC648740	0	0
chr1.fa	104159999	104168400	AMY2A	0	0
chr1.fa	104198141	104292440	AMY1A	0	0
chr1.fa	104198325	104292688	AMY1B	0	0
chr1.fa	104238890	104238905	AMY1C	0	0
chr1.fa	104615645	104619693	LOC100129138	0	0
chr1.fa	107599267	107601916	PRMT6	19.13408385	2280
chr1.fa	107682629	108024475	NTNG1	0	0
chr1.fa	108113782	108507545	VAV3	5.18082263	1111
chr1.fa	108677447	108742974	SLC25A24	15.43950082	2584
chr1.fa	108765963	108786703	NBPF4	0	0
chr1.fa	108992904	109013260	NBPF6	0	0
chr1.fa	109102971	109181949	FAM102B	6.963287147	1672
chr1.fa	109190910	109204148	C1orf59	4.968661538	456
chr1.fa	109234932	109244422	PRPF38B	11.24987524	1900
chr1.fa	109255556	109285367	FNDC7	0	0
chr1.fa	109289285	109352148	STXBP3	18.11642226	2039
chr1.fa	109358525	109399716	AKNAD1	0	0
chr1.fa	109399839	109401146	LOC642864	0	0
chr1.fa	109419603	109466836	GPSM2	0.731444266	76
chr1.fa	109472130	109506111	CLCC1	2.88352276	532
chr1.fa	109512838	109584850	WDR47	10.4403717	2052
chr1.fa	109606998	109618624	TAF13	46.86780843	1216
chr1.fa	109633403	109639554	TMEM167B	20.89675556	2584
chr1.fa	109642815	109643234	SCARNA2	0	0

chr1.fa	109648573	109656479	C1orf194	4.624177753	152
chr1.fa	109656585	109749403	KIAA1324	6.489594143	2052
chr1.fa	109756515	109780804	SARS	17.42167253	4940
chr1.fa	109792641	109818378	CELSR2	1.603662087	760
chr1.fa	109822176	109825790	PSRC1	0	0
chr1.fa	109834987	109849663	MYBPHL	0	0
chr1.fa	109852192	109940563	SORT1	6.984191908	2204
chr1.fa	109941653	109969108	PSMA5	22.26779658	3869
chr1.fa	110009100	110024764	SYPL2	0	0
chr1.fa	110026561	110035420	ATXN7L2	0.70964994	76
chr1.fa	110036658	110043063	CYB561D1	5.243981697	1216
chr1.fa	110049446	110052336	AMIGO1	0	0
chr1.fa	110082494	110088455	GPR61	0	0
chr1.fa	110091186	110138452	GNAI3	17.85711426	3798
chr1.fa	110141515	110141589	MIR197	0	0
chr1.fa	110145889	110155705	GNAT2	1.238273541	76
chr1.fa	110162459	110174677	AMPD2	4.435145334	912
chr1.fa	110198698	110208123	GSTM4	7.935136172	684
chr1.fa	110210644	110226619	GSTM2	8.375470513	760
chr1.fa	110230418	110236367	GSTM1	290.9662608	15504
chr1.fa	110254864	110260890	GSTM5	27.99014149	1976
chr1.fa	110276554	110283660	GSTM3	60.10541515	11154
chr1.fa	110292702	110306564	EPS8L3	1.987509094	200
chr1.fa	110453233	110472355	CSF1	0	0
chr1.fa	110527308	110566364	AHCYL1	60.87822416	11010
chr1.fa	110577241	110597263	FAM40A	13.98973336	2052
chr1.fa	110602997	110613322	ALX3	0	0
chr1.fa	110655062	110656569	UBL4B	0	0
chr1.fa	110693132	110744823	SLC6A17	0	0
chr1.fa	110753336	110776674	KCNC4	0.819733526	152
chr1.fa	110828999	110881793	LOC440600	0	0
chr1.fa	110881945	110889303	RBM15	6.896569822	1064
chr1.fa	110905505	110933636	SLC16A4	2.673363188	304
chr1.fa	110943877	110950546	HBXIP	115.470342	4403
chr1.fa	110993788	110999976	PROK1	0	0
chr1.fa	111023388	111033891	CYMP	0	0
chr1.fa	111059839	111061797	KCNA10	0	0
chr1.fa	111145776	111148345	KCNA2	0	0
chr1.fa	111214310	111217655	KCNA3	0	0
chr1.fa	111413821	111442558	CD53	0	0
chr1.fa	111489812	111506566	C1orf103	7.095165058	1064
chr1.fa	111659954	111682832	DRAM2	2.697159034	228
chr1.fa	111682249	111727724	CEPT1	4.330176744	456

chr1.fa	111729801	111743281	DENND2D	43.04068031	3952
chr1.fa	111770281	111786062	CHI3L2	1.049241122	76
chr1.fa	111823146	111828730	LOC149620	0	0
chr1.fa	111833484	111863185	CHIA	0	0
chr1.fa	111889195	111895639	C1orf88	15.84981236	1672
chr1.fa	111927141	111932473	LOC441897	0	0
chr1.fa	111956937	111970399	OVGP1	4.523212203	456
chr1.fa	111982512	111991742	WDR77	26.00641305	2713
chr1.fa	111991831	112004525	ATP5F1	63.73439282	5769
chr1.fa	112016604	112021134	C1orf162	0	0
chr1.fa	112025970	112106597	ADORA3	0	0
chr1.fa	112141629	112150940	LOC100129269	0	0
chr1.fa	112162405	112256101	RAP1A	31.70184864	2717
chr1.fa	112264686	112282046	C1orf183	0.565318129	152
chr1.fa	112298190	112310199	DDX20	4.952204598	760
chr1.fa	112318454	112531777	KCND3	1.882762894	228
chr1.fa	112938800	113003786	CTTNBP2NL	14.8964218	3952
chr1.fa	113004392	113004455	MIR4256	26.4089409	76
chr1.fa	113010040	113063910	WNT2B	0	0
chr1.fa	113066141	113162040	ST7L	1.081932611	228
chr1.fa	113162075	113214241	CAPZA1	47.31903993	5830
chr1.fa	113217048	113243368	MOV10	12.42432254	1976
chr1.fa	113243749	113250025	RHOC	76.1151267	5548
chr1.fa	113252616	113257950	PPM1J	1.972164109	152
chr1.fa	113263189	113269856	FAM19A3	0	0
chr1.fa	113454470	113498975	SLC16A1	0	0
chr1.fa	113465972	113467295	AKR7A2P1	0	0
chr1.fa	113615831	113667342	LRIG2	2.104931584	380
chr1.fa	113933475	114228545	MAGI3	6.67395635	2167
chr1.fa	114239824	114301777	PHTF1	2.084026823	304
chr1.fa	114304454	114355070	RSBN1	6.408866181	1900
chr1.fa	114356433	114414375	PTPN22	0	0
chr1.fa	114419436	114430169	BCL2L15	0	0
chr1.fa	114437677	114447741	AP4B1	6.123760815	684
chr1.fa	114447915	114456708	DCLRE1B	0.892455409	152
chr1.fa	114471996	114520422	HIPK1	19.72653369	7372
chr1.fa	114522030	114524875	OLFML3	0.931818631	76
chr1.fa	114631914	114696472	SYT6	1.547174753	304
chr1.fa	114935399	115053781	TRIM33	15.40391825	5776
chr1.fa	115110181	115124265	BCAS2	28.35864351	1659
chr1.fa	115127196	115212732	DENND2C	1.275857633	228
chr1.fa	115215720	115238239	AMPD1	0	0
chr1.fa	115247078	115259515	NRAS	12.33847958	2475

chr1.fa	115259537	115300624	CSDE1	197.8408851	37924
chr1.fa	115312105	115323308	SIKE1	6.756908223	1672
chr1.fa	115397455	115537990	SYCP1	0	0
chr1.fa	115572415	115576941	TSHB	0	0
chr1.fa	115590633	115632121	TSPAN2	0.526177298	76
chr1.fa	115828537	115880857	NGF	0	0
chr1.fa	116184574	116240845	VANGL1	2.69849338	1064
chr1.fa	116242626	116311426	CASQ2	0	0
chr1.fa	116378999	116383747	NHLH2	0	0
chr1.fa	116519119	116612675	SLC22A15	1.432420955	304
chr1.fa	116654376	116677861	C1orf161	0	0
chr1.fa	116915795	116947335	ATP1A1	53.18771818	9368
chr1.fa	116935611	116961244	ATP1A1OS	0.750569899	76
chr1.fa	117057156	117113715	CD58	0	0
chr1.fa	117117031	117210314	IGSF3	4.660649891	1520
chr1.fa	117214371	117214449	MIR320B1	0	0
chr1.fa	117297086	117311851	CD2	0	0
chr1.fa	117452689	117532972	PTGFRN	3.018069364	836
chr1.fa	117544382	117579167	CD101	0	0
chr1.fa	117602949	117645491	TTF2	7.660483186	1672
chr1.fa	117637265	117637350	MIR942	0	0
chr1.fa	117653677	117664411	TRIM45	0.94405014	152
chr1.fa	117686209	117753549	VTCN1	1.297651959	152
chr1.fa	117910085	118068320	MAN1A2	26.35022966	6384
chr1.fa	118148604	118171011	FAM46C	11.52386105	2964
chr1.fa	118406107	118472302	GDAP2	4.703793761	2044
chr1.fa	118472372	118503049	WDR3	9.634648815	1661
chr1.fa	118496288	118727848	SPAG17	0.923145378	294
chr1.fa	119425666	119532179	TBX15	0	0
chr1.fa	119573839	119683295	WARS2	7.750329183	988
chr1.fa	119911402	119936751	HAO2	46.77284744	3567
chr1.fa	119957554	119965662	HSD3B2	0	0
chr1.fa	120049826	120057681	HSD3B1	0	0
chr1.fa	120106503	120115199	HSD3BP4	0.194369805	76
chr1.fa	120140325	120141914	LOC644242	0	0
chr1.fa	120162000	120190390	ZNF697	7.319557659	1672
chr1.fa	120254419	120286849	PHGDH	66.9490559	6066
chr1.fa	120290619	120311555	HMGCS2	56.88741621	6308
chr1.fa	120336641	120354203	REG4	0	0
chr1.fa	120377388	120387779	NBPF7	0	0
chr1.fa	120436156	120439113	ADAM30	0	0
chr1.fa	120454176	120612317	NOTCH2	2.715617494	1444
chr1.fa	120839005	120855681	FAM72B	0	0

chr1.fa	120906034	120914842	HIST2H2BA	6.563872765	152
chr1.fa	120926908	120935944	FCGR1B	0	0
chr1.fa	121260910	121313686	EMBP1	0	0
chr1.fa	143687130	143714180	LOC100130000	0	0
chr1.fa	143767144	143767881	PPIAL4G	0	0
chr1.fa	143896452	143913143	FAM72D	0	0
chr1.fa	143915748	144094477	SRGAP2P2	9.817009502	456
chr1.fa	144300512	149616786	LOC728855	0	0
chr1.fa	144341670	144521969	LOC728875	0	0
chr1.fa	144610815	144612727	C1orf152	0	0
chr1.fa	144614959	144828810	NBPF9	1.73175935	228
chr1.fa	144676437	144680025	LOC653513	3.885394581	152
chr1.fa	144851424	145076186	PDE4DIP	8.254044982	4783
chr1.fa	145096407	145116922	SEC22B	66.82718558	5472
chr1.fa	145209111	145285912	NOTCH2NL	4.42802882	988
chr1.fa	145293371	145368684	NBPF10	2.29107292	1102
chr1.fa	145413191	145417545	HFE2	0	0
chr1.fa	145438462	145442628	TXNIP	268.5105437	35340
chr1.fa	145456236	145470387	POLR3GL	21.76185687	1140
chr1.fa	145470508	145475647	ANKRD34A	0.93582167	152
chr1.fa	145477085	145499091	LIX1L	14.47676983	912
chr1.fa	145507638	145509819	RBM8A	31.73187144	1658
chr1.fa	145511445	145516076	GNRHR2	2.919105333	608
chr1.fa	145516165	145523732	PEX11B	17.5419861	1520
chr1.fa	145524990	145543868	ITGA10	0	0
chr1.fa	145549209	145568526	ANKRD35	4.020608359	608
chr1.fa	145575988	145586492	PIAS3	12.4625738	1596
chr1.fa	145586547	145589435	NUDT17	1.629904235	76
chr1.fa	145592605	145610884	POLR3C	9.10713717	758
chr1.fa	145611036	145688776	RNF115	16.14492533	1216
chr1.fa	145695798	145715565	CD160	0	0
chr1.fa	145727726	145764073	PDZK1	5.615152412	532
chr1.fa	145764595	145827103	GPR89A	0.831742644	76
chr1.fa	145883868	147465755	GPR89C	0	0
chr1.fa	145924388	147484331	PDZK1P1	0	0
chr1.fa	146032542	147624469	NBPF11	0	0
chr1.fa	146057587	147607526	NBPF24	0	0
chr1.fa	146490895	146514599	LOC728989	0	0
chr1.fa	146626685	146644129	PRKAB2	9.661780527	2356
chr1.fa	146649430	146651528	PDIA3P	2.415611925	228
chr1.fa	146655884	146697230	FMO5	6.913916327	988
chr1.fa	146714291	146767443	CHD1L	15.16106719	2052
chr1.fa	147013182	147098015	BCL9	8.348561192	2356

chr1.fa	147119168	147142634	ACP6	22.43592424	1824
chr1.fa	147228332	147245484	GJA5	0	0
chr1.fa	147374946	147381395	GJA8	0	0
chr1.fa	147400506	147416212	GPR89B	0	0
chr1.fa	147906022	147931980	FLJ39739	0	0
chr1.fa	147954635	149553787	PPIAL4B	0	0
chr1.fa	148003642	148025848	NBPF14	2.748976156	456
chr1.fa	148558188	148577251	NBPF15	0	0
chr1.fa	148577357	148758311	NBPF16	0	0
chr1.fa	148644011	148644795	PPIAL4E	0	0
chr1.fa	148928286	148953054	LOC645166	2.17387282	152
chr1.fa	149279476	149291742	LOC388692	0.551085099	304
chr1.fa	149369294	149378297	FCGR1C	0	0
chr1.fa	149397114	149783928	HIST2H2BF	7.02355513	1292
chr1.fa	149755559	149764074	FCGR1A	0	0
chr1.fa	149784780	149785236	HIST2H3D	3.698363682	76
chr1.fa	149821759	149822340	HIST2H2BC	0	0
chr1.fa	149856010	149858232	HIST2H2BE	120.8895679	12084
chr1.fa	149858525	149858961	HIST2H2AC	58.01516138	1140
chr1.fa	149859019	149859466	HIST2H2AB	15.0907916	304
chr1.fa	149871155	149872348	BOLA1	1.972164109	76
chr1.fa	149874872	149889434	SV2A	0	0
chr1.fa	149895211	149899702	SF3B4	20.73151899	1444
chr1.fa	149900543	149908791	MTMR11	1.182453379	152
chr1.fa	149912232	149982686	OTUD7B	27.67745963	7980
chr1.fa	150039342	150117505	VPS45	16.19963354	2052
chr1.fa	150122170	150131825	PLEKHO1	0	0
chr1.fa	150190717	150208504	ANP32E	5.708334275	912
chr1.fa	150230218	150237480	CA14	0	0
chr1.fa	150237799	150241532	APH1A	16.98667557	1672
chr1.fa	150245183	150253335	C1orf54	0	0
chr1.fa	150255229	150259501	C1orf51	0	0
chr1.fa	150266269	150280819	MRPS21	73.7987012	3116
chr1.fa	150293928	150325704	PRPF3	16.46939392	1824
chr1.fa	150336990	150449041	RPRD2	12.49059508	4391
chr1.fa	150459920	150479749	TARS2	6.410200527	684
chr1.fa	150480487	150486265	ECM1	0	0
chr1.fa	150521898	150533412	ADAMTSL4	0	0
chr1.fa	150524405	150524490	MIR4257	0	0
chr1.fa	150547027	150552214	MCL1	50.47766046	9272
chr1.fa	150594599	150602098	ENSA	45.66934289	8512
chr1.fa	150618701	150669672	GOLPH3L	22.38633103	3192
chr1.fa	150670535	150693364	HORMAD1	0	0

chr1.fa	150702672	150738433	CTSS	2.469208176	456
chr1.fa	150768684	150780917	CTSK	1.867640301	152
chr1.fa	150782181	150849244	ARNT	17.95229765	3945
chr1.fa	150898815	150937220	SETDB1	11.78472579	2356
chr1.fa	150937649	150947440	LASS2	45.12626387	5083
chr1.fa	150954499	150968114	ANXA9	47.20940113	3872
chr1.fa	150969301	150980854	FAM63A	21.51366842	2254
chr1.fa	150980973	151008189	PRUNE	5.677421914	760
chr1.fa	151009029	151020076	BNIPL	12.55797958	1362
chr1.fa	151020259	151023446	C1orf56	4.217424466	304
chr1.fa	151023872	151032125	CDC42SE1	41.6720856	5168
chr1.fa	151032151	151040973	MLLT11	2.349561774	228
chr1.fa	151043080	151091007	GABPB2	11.25054241	988
chr1.fa	151104163	151119140	SEMA6C	0	0
chr1.fa	151129105	151132223	TNFAIP8L2	0	0
chr1.fa	151132226	151138424	LYSMD1	1.348801908	152
chr1.fa	151138517	151141614	SCNM1	23.6388376	912
chr1.fa	151142463	151148547	TMOD4	1.278526326	76
chr1.fa	151148934	151162640	VPS72	25.5313857	1520
chr1.fa	151171021	151222007	PIP5K1A	19.41185031	3323
chr1.fa	151227197	151239954	PSMD4	58.36298103	3493
chr1.fa	151254791	151264272	ZNF687	16.13714164	3192
chr1.fa	151264382	151300133	PI4KB	35.52319459	6688
chr1.fa	151313116	151319769	RFX5	8.876072837	1444
chr1.fa	151336780	151345164	SELENBP1	35.68687443	2736
chr1.fa	151372041	151374412	PSMB4	157.1404261	6536
chr1.fa	151375200	151431941	POGZ	16.33484731	4847
chr1.fa	151483862	151511167	CGN	69.91308423	16036
chr1.fa	151512781	151556059	TUFT1	15.21199474	2128
chr1.fa	151518272	151518367	MIR554	0	0
chr1.fa	151584662	151671559	SNX27	17.89180727	5700
chr1.fa	151672534	151688882	CELF3	0	0
chr1.fa	151694013	151702082	C1orf230	0	0
chr1.fa	151732123	151736040	MRPL9	36.36627918	1856
chr1.fa	151735445	151743806	OAZ3	23.24453821	971
chr1.fa	151744041	151763010	TDRKH	6.215830722	912
chr1.fa	151772765	151777882	LINGO4	0	0
chr1.fa	151778547	151804348	RORC	16.30215582	2356
chr1.fa	151810339	151813033	C2CD4D	0	0
chr1.fa	151814217	151816641	LOC100132111	0	0
chr1.fa	151819577	151826173	THEM5	1.474897651	76
chr1.fa	151846060	151882113	THEM4	7.661595142	760
chr1.fa	151955386	151966714	S100A10	121.7564483	5776

chr1.fa	152004982	152009511	S100A11	614.6079984	16416
chr1.fa	152056620	152061540	TCHHL1	0	0
chr1.fa	152078793	152086556	TCHH	0	0
chr1.fa	152126071	152131704	RPTN	0	0
chr1.fa	152184558	152196669	HRNR	0	0
chr1.fa	152274651	152297679	FLG	0.530402729	304
chr1.fa	152321213	152332482	FLG2	0	0
chr1.fa	152381719	152386750	CRNN	0.883559766	76
chr1.fa	152483320	152484653	LCE5A	0	0
chr1.fa	152486978	152488481	CRCT1	0	0
chr1.fa	152538130	152539248	LCE3E	0	0
chr1.fa	152551860	152552980	LCE3D	0	0
chr1.fa	152573138	152573562	LCE3C	0	0
chr1.fa	152586287	152586574	LCE3B	0	0
chr1.fa	152595310	152595579	LCE3A	0	0
chr1.fa	152635872	152637135	LCE2D	0	0
chr1.fa	152647771	152649049	LCE2C	0	0
chr1.fa	152658599	152659876	LCE2B	0	0
chr1.fa	152670840	152671918	LCE2A	0	0
chr1.fa	152681523	152681910	LCE4A	0	0
chr1.fa	152691998	152692905	C1orf68	0	0
chr1.fa	152730506	152734529	KPRP	0	0
chr1.fa	152748848	152749445	LCE1F	0	0
chr1.fa	152758753	152760901	LCE1E	0	0
chr1.fa	152769227	152770657	LCE1D	0	0
chr1.fa	152777311	152779107	LCE1C	0	0
chr1.fa	152784447	152785585	LCE1B	0	0
chr1.fa	152799949	152800573	LCE1A	0	0
chr1.fa	152815330	152816459	LCE6A	0	0
chr1.fa	152850798	152857523	SMCP	0	0
chr1.fa	152881039	152884362	IVL	6.333253213	608
chr1.fa	152943128	152945069	SPRR4	0	0
chr1.fa	152956564	152958290	SPRR1A	28.38466327	836
chr1.fa	152974223	152976332	SPRR3	34.28336431	1520
chr1.fa	153003679	153005376	SPRR1B	2.730517696	76
chr1.fa	153012201	153013594	SPRR2D	2.47832621	76
chr1.fa	153028596	153029988	SPRR2A	0	0
chr1.fa	153042718	153044084	SPRR2B	0	0
chr1.fa	153065611	153067001	SPRR2E	0	0
chr1.fa	153084613	153085989	SPRR2F	0	0
chr1.fa	153112594	153113969	SPRR2C	0	0
chr1.fa	153122058	153123427	SPRR2G	0	0
chr1.fa	153175919	153177596	LELP1	0	0

chr1.fa	153190060	153191793	PRR9	0	0
chr1.fa	153232179	153234600	LOR	0	0
chr1.fa	153270338	153283194	PGLYRP3	0	0
chr1.fa	153302597	153321022	PGLYRP4	0	0
chr1.fa	153330330	153333503	S100A9	35.15091192	912
chr1.fa	153346184	153348075	S100A12	0	0
chr1.fa	153362508	153363664	S100A8	25.85340798	608
chr1.fa	153389000	153395701	S100A7A	0	0
chr1.fa	153409471	153412503	S100A7L2	0	0
chr1.fa	153430220	153433137	S100A7	0	0
chr1.fa	153507076	153508717	S100A6	534.5200774	16416
chr1.fa	153509623	153514241	S100A5	0	0
chr1.fa	153516095	153518282	S100A4	38.95780245	988
chr1.fa	153519809	153521734	S100A3	0	0
chr1.fa	153533585	153538306	S100A2	5.270891018	228
chr1.fa	153579367	153585514	S100A16	37.9815056	1824
chr1.fa	153586732	153588790	S100A14	14.59841775	684
chr1.fa	153591276	153606524	S100A13	10.31383118	608
chr1.fa	153600873	153604509	S100A1	0	0
chr1.fa	153606569	153617979	C1orf77	36.53685314	2052
chr1.fa	153631145	153634326	SNAPIN	26.06378995	1213
chr1.fa	153634514	153643479	ILF2	70.24911715	5092
chr1.fa	153651164	153666468	NPR1	0	0
chr1.fa	153700567	153746555	INTS3	16.45849675	3317
chr1.fa	153747768	153752633	SLC27A3	0	0
chr1.fa	153777203	153895451	GATAD2B	17.18215733	5776
chr1.fa	153901977	153919154	DENND4B	5.645842381	1444
chr1.fa	153920148	153931132	CRTC2	7.357586534	886
chr1.fa	153931588	153940188	SLC39A1	2.785670684	304
chr1.fa	153940397	153946744	CREB3L4	27.50733045	2052
chr1.fa	153946833	153950451	JTB	70.05474735	4681
chr1.fa	153954128	153958806	RAB13	105.0813427	5448
chr1.fa	153963239	153964631	RPS27	67.41429804	1064
chr1.fa	153965168	154127592	NUP210L	0	0
chr1.fa	154127780	154164609	TPM3	20.38125304	9029
chr1.fa	154166141	154166219	MIR190B	0	0
chr1.fa	154171848	154178809	C1orf189	0	0
chr1.fa	154179183	154193273	C1orf43	136.6802242	11665
chr1.fa	154192655	154243329	UBAP2L	33.96979289	6808
chr1.fa	154245039	154248355	HAX1	47.30769799	2508
chr1.fa	154293592	154297801	AQP10	0	0
chr1.fa	154298036	154323780	ATP8B2	3.485980199	988
chr1.fa	154377669	154440188	IL6R	1.619007072	304

chr1.fa	154451954	154474526	SHE	0	0
chr1.fa	154474695	154520623	TDRD10	0	0
chr1.fa	154521051	154531120	UBE2Q1	17.65051295	2558
chr1.fa	154540257	154552353	CHRNB2	0	0
chr1.fa	154554534	154600456	ADAR	63.62875705	20128
chr1.fa	154679915	154842754	KCNN3	0	0
chr1.fa	154897208	154909484	PMVK	70.26112627	4104
chr1.fa	154916559	154928567	PBXIP1	5.817305905	836
chr1.fa	154929502	154934258	PYGO2	23.38597894	3344
chr1.fa	154934774	154946959	SHC1	29.00602395	4848
chr1.fa	154947118	154951725	CKS1B	4.142478671	152
chr1.fa	154948169	154948259	MIR4258	0	0
chr1.fa	154955770	154965587	FLAD1	8.725069293	1064
chr1.fa	154966062	154966791	LENEP	0	0
chr1.fa	154975112	154990999	ZBTB7B	41.60003089	6764
chr1.fa	154991003	155006257	DCST2	0.698752777	76
chr1.fa	155006282	155023406	DCST1	0	0
chr1.fa	155023762	155035252	ADAM15	2.780778081	371
chr1.fa	155036213	155042029	EFNA4	5.378528301	304
chr1.fa	155051348	155060014	EFNA3	1.896995923	152
chr1.fa	155100349	155107386	EFNA1	9.713597649	684
chr1.fa	155108288	155111334	SLC50A1	10.06808903	608
chr1.fa	155112367	155112996	DPM3	30.7304444	532
chr1.fa	155141884	155145804	KRTCAP2	33.33842461	820
chr1.fa	155146360	155157445	TRIM46	0	0
chr1.fa	155158300	155162700	MUC1	10.09477596	532
chr1.fa	155164968	155165063	MIR92B	0	0
chr1.fa	155165379	155177690	THBS3	4.126911296	584
chr1.fa	155178490	155183614	MTX1	6.213829203	456
chr1.fa	155183616	155197325	GBAP1	0	0
chr1.fa	155204239	155214653	GBA	0.654274561	76
chr1.fa	155216996	155225274	FAM189B	3.708816063	532
chr1.fa	155225770	155232195	SCAMP3	5.311588586	380
chr1.fa	155232659	155243281	CLK2	11.07262955	1064
chr1.fa	155247374	155259083	HCN3	0	0
chr1.fa	155259639	155271225	PKLR	4.534776539	532
chr1.fa	155278539	155290250	FDPS	77.46615252	5016
chr1.fa	155290458	155291478	C1orf104	0	0
chr1.fa	155291487	155300909	RUSC1	6.475805896	1140
chr1.fa	155305052	155491409	ASH1L	30.90324227	15744
chr1.fa	155316141	155316173	MIR555	0	0
chr1.fa	155402971	155404053	POU5F1P4	0	0
chr1.fa	155531772	155533735	LOC645676	3.203098744	228

chr1.fa	155580007	155584758	MSTO1	3.4635187	380
chr1.fa	155629233	155658764	YY1AP1	19.99184625	2660
chr1.fa	155658882	155708800	DAP3	66.07528134	6587
chr1.fa	155715609	155721369	MSTO2P	0	0
chr1.fa	155719510	155826972	GON4L	17.52352764	5548
chr1.fa	155829260	155854990	SYT11	0	0
chr1.fa	155867601	155881177	RIT1	12.27198465	1890
chr1.fa	155882836	155904188	KIAA0907	9.696028754	1284
chr1.fa	155889700	155889833	SNORA42	0	0
chr1.fa	155895749	155895877	SCARNA4	0	0
chr1.fa	155911480	155912625	RXFP4	0	0
chr1.fa	155916630	155948336	ARHGEF2	11.03660219	2128
chr1.fa	155978839	155990758	SSR2	75.18553198	3800
chr1.fa	156005092	156023516	UBQLN4	15.90407579	2508
chr1.fa	156024517	156028301	ROBLD3	57.08356514	1748
chr1.fa	156030966	156040295	RAB25	81.00372745	3952
chr1.fa	156041804	156051789	MEX3A	0.827961996	228
chr1.fa	156084461	156109878	LMNA	55.02644764	8284
chr1.fa	156119735	156147542	SEMA4A	5.384310469	836
chr1.fa	156163730	156182587	SLC25A44	8.84182461	1444
chr1.fa	156195348	156209868	PMF1	16.73137061	483
chr1.fa	156211951	156212086	BGLAP	0	0
chr1.fa	156213207	156217843	PAQR6	0	0
chr1.fa	156219015	156252620	SMG5	25.92857617	5320
chr1.fa	156252704	156262234	TMEM79	20.10393136	2128
chr1.fa	156262484	156265449	C1orf85	24.52617801	1748
chr1.fa	156268415	156269428	VHLL	0	0
chr1.fa	156278752	156308206	CCT3	75.36277768	8072
chr1.fa	156307105	156316785	C1orf182	0	0
chr1.fa	156339003	156355011	RHBG	0.883559766	76
chr1.fa	156374055	156399184	C1orf61	0	0
chr1.fa	156390133	156390221	MIR9-1	0	0
chr1.fa	156433519	156470529	MEF2D	11.79717969	3116
chr1.fa	156495197	156542396	IQGAP3	0	0
chr1.fa	156549519	156556562	TTC24	0	0
chr1.fa	156561558	156564091	APOA1BP	61.8171593	3116
chr1.fa	156564100	156571279	GPATCH4	14.09670347	1366
chr1.fa	156589086	156595517	HAPLN2	0	0
chr1.fa	156611740	156629320	BCAN	0	0
chr1.fa	156638556	156647189	NES	0.608239607	152
chr1.fa	156669400	156675608	CRABP2	40.6570927	2128
chr1.fa	156692413	156697705	ISG20L2	14.00930378	1292
chr1.fa	156698263	156706752	C1orf66	8.938787122	912

chr1.fa	156707094	156710923	MRPL24	45.58172081	1900
chr1.fa	156711899	156722240	HDGF	77.32026397	9728
chr1.fa	156737274	156770609	PRCC	24.54374691	2343
chr1.fa	156776035	156786640	SH2D2A	0	0
chr1.fa	156785542	156851642	NTRK1	0	0
chr1.fa	156810665	156828712	INSRR	0	0
chr1.fa	156863523	156886226	PEAR1	0	0
chr1.fa	156890424	156902880	C1orf92	0	0
chr1.fa	156904632	157015162	ARHGEF11	19.30465781	5980
chr1.fa	156905923	156906036	MIR765	0	0
chr1.fa	157061835	157069600	ETV3L	0	0
chr1.fa	157094459	157108177	ETV3	20.47554686	2508
chr1.fa	157098154	157098463	CYCSP52	0	0
chr1.fa	157483167	157522310	FCRL5	0	0
chr1.fa	157543539	157567870	FCRL4	0	0
chr1.fa	157647978	157670647	FCRL3	0	0
chr1.fa	157715523	157746922	FCRL2	0	0
chr1.fa	157764194	157789940	FCRL1	0	0
chr1.fa	157800704	157811634	CD5L	0	0
chr1.fa	157963063	158065844	KIRREL	0.467910835	76
chr1.fa	158101834	158110430	LOC646268	0	0
chr1.fa	158149737	158156216	CD1D	0	0
chr1.fa	158223927	158228058	CD1A	0	0
chr1.fa	158259563	158264564	CD1C	0	0
chr1.fa	158297740	158301321	CD1B	0	0
chr1.fa	158323486	158327343	CD1E	0	0
chr1.fa	158368312	158369256	OR10T2	0	0
chr1.fa	158389718	158390656	OR10K2	0	0
chr1.fa	158435352	158436293	OR10K1	0	0
chr1.fa	158449668	158450675	OR10R2	0	0
chr1.fa	158516918	158517895	OR6Y1	0	0
chr1.fa	158532441	158533394	OR6P1	0	0
chr1.fa	158548709	158549689	OR10X1	0	0
chr1.fa	158576229	158577170	OR10Z1	0	0
chr1.fa	158580496	158656506	SPTA1	0	0
chr1.fa	158669468	158670442	OR6K2	0	0
chr1.fa	158686958	158687905	OR6K3	0	0
chr1.fa	158724606	158725637	OR6K6	0	0
chr1.fa	158735534	158736472	OR6N1	0	0
chr1.fa	158746472	158747425	OR6N2	0	0
chr1.fa	158801168	158819270	MNDA	0	0
chr1.fa	158901342	158946843	PYHIN1	0	0
chr1.fa	158979682	159024945	IFI16	2.472766433	304

chr1.fa	159032275	159046647	AIM2	0	0
chr1.fa	159141377	159172932	CADM3	0	0
chr1.fa	159173803	159176290	DARC	0	0
chr1.fa	159259504	159278014	FCER1A	0	0
chr1.fa	159283460	159284449	OR10J3	0	0
chr1.fa	159409512	159410600	OR10J1	0	0
chr1.fa	159504868	159505797	OR10J5	0	0
chr1.fa	159557616	159558661	APCS	0	0
chr1.fa	159682079	159684379	CRP	0	0
chr1.fa	159750759	159752333	DUSP23	19.73920999	608
chr1.fa	159772173	159786047	FCRL6	0	0
chr1.fa	159796479	159807282	SLAMF8	0	0
chr1.fa	159804264	159825137	C1orf204	0	0
chr1.fa	159824106	159832447	VSIG8	0	0
chr1.fa	159842154	159869906	CCDC19	0	0
chr1.fa	159887903	159895284	TAGLN2	94.33629517	5769
chr1.fa	159896829	159915386	IGSF9	2.960692465	532
chr1.fa	159921282	159924044	SLAMF9	2.924220328	152
chr1.fa	159997462	160001783	PIGM	1.564298866	304
chr1.fa	160007257	160040051	KCNJ10	0.637150448	152
chr1.fa	160051360	160059212	KCNJ9	0	0
chr1.fa	160061130	160068408	IGSF8	0.743898167	76
chr1.fa	160085520	160113374	ATP1A2	0	0
chr1.fa	160121352	160156767	ATP1A4	0	0
chr1.fa	160160285	160171676	CASQ1	0.863322178	76
chr1.fa	160175125	160185162	PEA15	71.8785766	7980
chr1.fa	160185505	160232350	DCAF8	25.94725702	5472
chr1.fa	160246599	160254941	PEX19	36.91558515	6004
chr1.fa	160258377	160313354	COPA	40.77584954	9956
chr1.fa	160287055	160288260	SUMO1P3	0	0
chr1.fa	160314512	160328742	NCSTN	1.860079004	228
chr1.fa	160336861	160342638	NHLH1	0	0
chr1.fa	160370364	160398468	VANGL2	1.263181342	304
chr1.fa	160454820	160493052	SLAMF6	0	0
chr1.fa	160510884	160549306	CD84	0	0
chr1.fa	160579891	160617081	SLAMF1	0	0
chr1.fa	160648536	160681585	CD48	0	0
chr1.fa	160709077	160724601	SLAMF7	0	0
chr1.fa	160765928	160798045	LY9	0	0
chr1.fa	160799950	160832692	CD244	0	0
chr1.fa	160846330	160854960	ITLN1	0	0
chr1.fa	160914816	160924589	ITLN2	8.818251156	456
chr1.fa	160965001	160991133	F11R	7.896440124	1715

chr1.fa	161007422	161008774	TSTD1	201.8977432	5320
chr1.fa	161009041	161015757	USF1	19.4670033	1573
chr1.fa	161016732	161039760	ARHGAP30	0.445671727	76
chr1.fa	161040781	161059385	PVRL4	17.37474801	2736
chr1.fa	161068151	161070138	KLHDC9	6.177579456	380
chr1.fa	161070346	161087861	PFDN2	55.71986303	1596
chr1.fa	161087867	161095235	NIT1	13.05658038	1520
chr1.fa	161090985	161102478	DEDD	12.86576884	1061
chr1.fa	161123534	161128646	UFC1	133.7913641	6750
chr1.fa	161129254	161135516	USP21	9.333976073	988
chr1.fa	161136181	161141010	PPOX	3.824014643	304
chr1.fa	161141100	161147758	B4GALT3	9.883949217	1064
chr1.fa	161159538	161168845	ADAMTS4	0	0
chr1.fa	161169105	161184184	NDUFS2	44.71795385	4104
chr1.fa	161185087	161189038	FCER1G	0	0
chr1.fa	161192083	161193418	APOA2	0	0
chr1.fa	161195833	161200407	TOMM40L	7.888878827	912
chr1.fa	161200603	161208000	NR1I3	2.98626744	152
chr1.fa	161228517	161255240	PCP4L1	0	0
chr1.fa	161274525	161279762	MPZ	0	0
chr1.fa	161284166	161334520	SDHC	33.33130809	4252
chr1.fa	161334536	161337664	C1orf192	2.226801897	76
chr1.fa	161475205	161489360	FCGR2A	0	0
chr1.fa	161494036	161496687	HSPA6	0	0
chr1.fa	161511551	161520413	FCGR3A	0	0
chr1.fa	161551129	161570032	FCGR2C	0	0
chr1.fa	161575849	161578341	HSPA7	0	0
chr1.fa	161592988	161601158	FCGR3B	0	0
chr1.fa	161632905	161648444	FCGR2B	0	0
chr1.fa	161653495	161655042	RPL31P11	2.183658027	152
chr1.fa	161676762	161684142	FCRLA	0	0
chr1.fa	161692457	161697933	FCRLB	1.911896126	152
chr1.fa	161719581	161726952	DUSP12	9.472525717	532
chr1.fa	161736084	161928852	ATF6	16.30393495	1824
chr1.fa	161952982	161993644	OLFML2B	0	0
chr1.fa	162039581	162339813	NOS1AP	1.718415885	532
chr1.fa	162312336	162312430	MIR556	0	0
chr1.fa	162343515	162346644	C1orf111	0	0
chr1.fa	162348696	162356608	C1orf226	3.577605325	684
chr1.fa	162365056	162381928	SH2D1B	0	0
chr1.fa	162466964	162499419	UHMK1	21.86415677	8512
chr1.fa	162531296	162569633	UAP1	28.32684159	2964
chr1.fa	162602228	162750247	DDR2	0.521062303	76

chr1.fa	162760496	162782608	HSD17B7	1.11262258	76
chr1.fa	162824087	162838605	C1orf110	0	0
chr1.fa	163038396	163046592	RGS4	2.124502	380
chr1.fa	163112089	163172963	RGS5	0.570433124	152
chr1.fa	163291723	163325553	NUF2	0	0
chr1.fa	164528802	164821045	PBX1	12.64537927	3800
chr1.fa	165171104	165325952	LMX1A	0	0
chr1.fa	165370159	165414592	RXRG	0	0
chr1.fa	165446079	165551341	LOC400794	0	0
chr1.fa	165513478	165533185	LRRC52	0	0
chr1.fa	165600450	165624855	MGST3	139.6925114	4152
chr1.fa	165631449	165667900	ALDH9A1	64.22654428	7220
chr1.fa	165667987	165679199	LOC440700	0	0
chr1.fa	165693528	165738135	TMCO1	2.21101213	442
chr1.fa	165796732	165880855	UCK2	7.914453801	1731
chr1.fa	166039256	166135958	FAM78B	0	0
chr1.fa	166123980	166124035	MIR921	0	0
chr1.fa	166573153	166594473	FMO9P	49.63791174	3040
chr1.fa	166808724	166823709	POGK	56.60231084	9804
chr1.fa	166825749	166845654	TADA1	12.03513815	1216
chr1.fa	166882441	166944561	ILDR2	0	0
chr1.fa	166958519	166991447	MAEL	0	0
chr1.fa	167022082	167059868	GPA33	0	0
chr1.fa	167064087	167098402	DUSP27	0	0
chr1.fa	167190066	167396582	POU2F1	4.974888489	3192
chr1.fa	167399877	167487847	CD247	0	0
chr1.fa	167510251	167523056	CREG1	7.511925944	684
chr1.fa	167599474	167675486	RCSD1	0	0
chr1.fa	167691187	167761156	MPZL1	1.686836352	380
chr1.fa	167778625	167883453	ADCY10	0	0
chr1.fa	167887310	167906278	BRP44	187.6502585	6109
chr1.fa	167905797	168045083	DCAF6	33.00461559	5267
chr1.fa	168053997	168105624	GPR161	0	0
chr1.fa	168148171	168171351	TIPRL	10.50130686	1520
chr1.fa	168195255	168212088	SFT2D2	1.974610411	76
chr1.fa	168214819	168216668	ANKRD36BP1	2.740747686	228
chr1.fa	168250278	168283664	TBX19	0	0
chr1.fa	168344762	168344859	MIR557	0	0
chr1.fa	168510003	168513235	XCL2	0	0
chr1.fa	168545711	168551315	XCL1	0	0
chr1.fa	168664695	168698442	DPT	0	0
chr1.fa	168756179	168762126	MGC4473	0	0
chr1.fa	169075947	169101768	ATP1B1	43.76945588	3952

chr1.fa	169101773	169337186	NME7	8.351674667	608
chr1.fa	169337194	169365780	BLZF1	8.027650862	836
chr1.fa	169364114	169396670	C1orf114	0	0
chr1.fa	169433149	169455208	SLC19A2	4.183621021	684
chr1.fa	169481192	169555769	F5	0	0
chr1.fa	169558088	169599377	SELP	0	0
chr1.fa	169659806	169680843	SELL	0	0
chr1.fa	169691781	169703220	SELE	0	0
chr1.fa	169761673	169764031	C1orf156	0	0
chr1.fa	169764550	169821077	C1orf112	1.654812036	228
chr1.fa	169821804	169863076	SCYL3	12.23217664	1748
chr1.fa	169890470	170043879	KIFAP3	7.331344386	988
chr1.fa	170115188	170136923	METTL11B	0	0
chr1.fa	170240546	170253349	LOC284688	0	0
chr1.fa	170501263	170522974	GORAB	2.396708683	380
chr1.fa	170633313	170708541	PRRX1	0	0
chr1.fa	170904612	171033906	C1orf129	0	0
chr1.fa	171060018	171086959	FMO3	0	0
chr1.fa	171070869	171070947	MIR1295	0	0
chr1.fa	171106879	171130702	FMO6P	0	0
chr1.fa	171154388	171181822	FMO2	0.326247716	76
chr1.fa	171217663	171255113	FMO1	7.950036374	760
chr1.fa	171283486	171311223	FMO4	3.160622047	304
chr1.fa	171308035	171310463	TOP1P1	0	0
chr1.fa	171454666	171562650	PRRC2C	36.07205578	16843
chr1.fa	171604557	171621773	MYOC	0	0
chr1.fa	171669296	171711379	VAMP4	2.962026812	380
chr1.fa	171750761	171766856	METTL13	14.91154439	1976
chr1.fa	171810621	172381857	DNM3	0.221056735	76
chr1.fa	172107938	172108047	MIR214	0	0
chr1.fa	172113675	172113784	MIR199A2	0	0
chr1.fa	172389828	172437969	C1orf105	0	0
chr1.fa	172410597	172413230	PIGC	1.144424504	76
chr1.fa	172502260	172580973	C1orf9	4.782075421	1216
chr1.fa	172628185	172636012	FASLG	0	0
chr1.fa	173010360	173020103	TNFSF18	0	0
chr1.fa	173152870	173176471	TNFSF4	0.483922993	76
chr1.fa	173331510	173332878	LOC646870	0	0
chr1.fa	173446486	173457946	PRDX6	229.7419961	17252
chr1.fa	173469604	173572233	SLC9A11	0	0
chr1.fa	173577475	173639001	ANKRD45	0	0
chr1.fa	173604661	173606272	LOC730159	0	0
chr1.fa	173684080	173755840	KLHL20	13.66192891	2128

chr1.fa	173768688	173793777	CENPL	0.606460479	76
chr1.fa	173793797	173827682	DARS2	10.60138285	1596
chr1.fa	173832386	173833038	LOC100506046	0	0
chr1.fa	173833080	173837125	GAS5	9.896625509	263
chr1.fa	173833313	173833355	SNORD81	0	0
chr1.fa	173833507	173833583	SNORD47	0	0
chr1.fa	173833971	173834041	SNORD80	0	0
chr1.fa	173834488	173834568	SNORD79	0	0
chr1.fa	173834771	173834824	SNORD78	0	0
chr1.fa	173835106	173835166	SNORD44	0	0
chr1.fa	173835449	173835509	SNORD77	0	0
chr1.fa	173835773	173835853	SNORD76	0	0
chr1.fa	173836017	173836076	SNORD75	0	0
chr1.fa	173836812	173836883	SNORD74	0	0
chr1.fa	173837493	173855774	ZBTB37	5.878908234	608
chr1.fa	173872942	173886516	SERPINC1	0	0
chr1.fa	173900352	173962210	RC3H1	7.839063225	3876
chr1.fa	174128634	174927327	RABGAP1L	12.04670248	1793
chr1.fa	174417212	174418683	GPR52	0	0
chr1.fa	174968571	174981163	CACYBP	9.687355501	1444
chr1.fa	174982094	174992591	MRPS14	18.88878649	1900
chr1.fa	175036994	175117202	TNN	0	0
chr1.fa	175126123	175162229	KIAA0040	9.471636153	2052
chr1.fa	175291935	175712752	TNR	0	0
chr1.fa	175913967	176176370	RFWD2	15.84158389	1976
chr1.fa	175937533	175937676	SCARNA3	0	0
chr1.fa	176432307	176811970	PAPPA2	0.244185407	76
chr1.fa	176830203	177134024	ASTN1	0	0
chr1.fa	176998499	176998581	MIR488	0	0
chr1.fa	177140633	177251558	FAM5B	0	0
chr1.fa	177898242	177939050	SEC16B	1.680164619	304
chr1.fa	177975275	178007142	LOC730102	2.273281633	304
chr1.fa	178060643	178062863	LOC100302401	0.815285704	76
chr1.fa	178063129	178448648	RASAL2	8.78422532	4256
chr1.fa	178482212	178492635	C1orf49	0	0
chr1.fa	178511931	178518024	C1orf220	0	0
chr1.fa	178694300	178889237	RALGPS2	9.207435548	2415
chr1.fa	178818670	178840215	ANGPTL1	0	0
chr1.fa	178995074	179045702	FAM20B	9.380455809	2508
chr1.fa	179051112	179065129	TOR3A	1.592987315	152
chr1.fa	179068462	179198819	ABL2	7.396282582	4104
chr1.fa	179263017	179324453	SOAT1	7.354028276	1122
chr1.fa	179334855	179523870	C1orf125	0.466576489	76

chr1.fa	179519674	179545084	NPHS2	0.91313778	76
chr1.fa	179560748	179660407	TDRD5	3.5458034	684
chr1.fa	179712298	179785333	FAM163A	0	0
chr1.fa	179809102	179846941	TOR1AIP2	6.881447229	4560
chr1.fa	179851420	179889211	TOR1AIP1	19.86152508	3400
chr1.fa	179923908	180084015	CEP350	19.21792529	11628
chr1.fa	180123968	180167143	QSOX1	6.678404171	988
chr1.fa	180167170	180169859	FLJ23867	6.28321522	760
chr1.fa	180199433	180244188	LHX4	0	0
chr1.fa	180238798	180243247	LOC100527964	0.724105361	76
chr1.fa	180257352	180472022	ACBD6	19.59687969	1424
chr1.fa	180407449	180407525	MIR3121	0	0
chr1.fa	180601146	180859415	XPR1	6.151337309	2350
chr1.fa	180882313	180915239	KIAA1614	0.406753288	76
chr1.fa	180942176	180992046	STX6	14.53503629	2964
chr1.fa	181002561	181031074	MR1	1.290313054	456
chr1.fa	181057638	181059979	IER5	2.164977176	228
chr1.fa	181452716	181770715	CACNA1E	0	0
chr1.fa	182023705	182030847	ZNF648	0	0
chr1.fa	182350839	182361341	GLUL	65.55822207	14592
chr1.fa	182367252	182369751	TEDDM1	0	0
chr1.fa	182376756	182383948	NCRNA00272	0	0
chr1.fa	182419256	182529732	RGSL1	0	0
chr1.fa	182542769	182558394	RNASEL	11.15891729	2128
chr1.fa	182567758	182573548	RGS16	2.779888516	304
chr1.fa	182584275	182585764	LOC284648	0	0
chr1.fa	182615792	182642067	RGS8	1.85318488	76
chr1.fa	182761394	182798588	NPL	3.883170671	271
chr1.fa	182808439	182857117	DHX9	36.49726753	7669
chr1.fa	182869000	182922553	C1orf14	0	0
chr1.fa	182992595	183114727	LAMC1	4.499193966	1596
chr1.fa	183155174	183214262	LAMC2	0.601123093	152
chr1.fa	183217379	183387737	NMNAT2	0	0
chr1.fa	183441506	183523328	SMG7	21.9755747	6004
chr1.fa	183524697	183560056	NCF2	0	0
chr1.fa	183595332	183604985	ARPC5	40.07976545	3572
chr1.fa	183605208	183897666	RGL1	3.849367226	885
chr1.fa	183615411	183622448	APOBEC4	0	0
chr1.fa	183904966	184006863	GLT25D2	0	0
chr1.fa	184020811	184043344	TSEN15	11.40354748	1064
chr1.fa	184356150	184598155	C1orf21	12.95339092	6004
chr1.fa	184659625	184724041	EDEM3	7.645582984	2280
chr1.fa	184760166	184943682	FAM129A	23.18315827	7220

chr1.fa	185014551	185071740	RNF2	8.567394016	1368
chr1.fa	185087218	185126116	C1orf25	4.218758812	836
chr1.fa	185126192	185260913	C1orf26	3.855816568	684
chr1.fa	185265522	185286461	IVNS1ABP	27.38434819	5168
chr1.fa	185703683	186160085	HMCN1	0	0
chr1.fa	186029867	186446655	MIR548F1	0	0
chr1.fa	186265405	186283688	PRG4	0	0
chr1.fa	186280786	186344457	TPR	17.85333362	7254
chr1.fa	186344890	186390503	C1orf27	4.338182823	760
chr1.fa	186369704	186370587	OCLM	0	0
chr1.fa	186412715	186430239	PDC	0	0
chr1.fa	186640944	186649559	PTGS2	1.128634738	228
chr1.fa	186798032	186958113	PLA2G4A	5.772382906	760
chr1.fa	190066797	190446759	FAM5C	2.344224388	304
chr1.fa	190594020	190770788	LOC440704	0	0
chr1.fa	192127592	192154945	RGS18	0	0
chr1.fa	192286122	192336414	RGS21	0	0
chr1.fa	192544857	192549159	RGS1	0	0
chr1.fa	192605268	192629440	RGS13	0	0
chr1.fa	192778169	192781407	RGS2	7.511925944	456
chr1.fa	192981496	193028523	UCHL5	4.226097718	1064
chr1.fa	193028552	193060906	TROVE2	22.80286952	2508
chr1.fa	193065600	193075244	GLRX2	14.20056011	825
chr1.fa	193091088	193223942	CDC73	14.45408594	3860
chr1.fa	193105633	193105713	MIR1278	0	0
chr1.fa	193147860	193155743	B3GALT2	0	0
chr1.fa	196194913	196577499	KCNT2	0	0
chr1.fa	196621008	196716634	CFH	0.386293308	76
chr1.fa	196743930	196763203	CFHR3	0	0
chr1.fa	196788861	196801319	CFHR1	0	0
chr1.fa	196857212	196887763	CFHR4	0	0
chr1.fa	196912934	196928356	CFHR2	0	0
chr1.fa	196946667	196978803	CFHR5	0	0
chr1.fa	197008321	197036397	F13B	0	0
chr1.fa	197053257	197115824	ASPM	0	0
chr1.fa	197122814	197169672	ZBTB41	4.786968025	1824
chr1.fa	197237334	197447585	CRB1	0	0
chr1.fa	197473879	197744623	DENND1B	4.792750193	2356
chr1.fa	197871682	197876497	C1orf53	0	0
chr1.fa	197881635	197899273	LHX9	0	0
chr1.fa	198126108	198291548	NEK7	12.68029467	2356
chr1.fa	198492352	198510075	ATP6V1G3	14.95735695	456
chr1.fa	198608137	198726545	PTPRC	0.317129682	76

chr1.fa	198828002	198828111	MIR181B1	0	0
chr1.fa	198828173	198828282	MIR181A1	0	0
chr1.fa	199996770	200146550	NR5A2	0	0
chr1.fa	200182656	200183643	FAM58B	0	0
chr1.fa	200375420	200379166	ZNF281	7.680498384	1216
chr1.fa	200520625	200589862	KIF14	0	0
chr1.fa	200613165	200639126	DDX59	9.470746588	988
chr1.fa	200708686	200829831	CAMSAP1L1	9.656887923	3192
chr1.fa	200842166	200843251	GPR25	0	0
chr1.fa	200860639	200884864	C1orf106	11.27811891	2204
chr1.fa	200938520	200992828	KIF21B	1.878982246	836
chr1.fa	201008640	201081694	CACNA1S	0	0
chr1.fa	201103900	201123632	TMEM9	12.08050593	836
chr1.fa	201159953	201198080	IGFN1	0	0
chr1.fa	201252580	201302121	PKP1	27.30584414	6688
chr1.fa	201328142	201346805	TNNT2	1.443318118	76
chr1.fa	201349966	201368669	LAD1	45.63754097	5826
chr1.fa	201372895	201390874	TNNI1	0.825515694	228
chr1.fa	201434622	201438299	PHLDA3	21.18297288	1444
chr1.fa	201452658	201476387	CSRP1	49.4542167	5168
chr1.fa	201489032	201489720	RPS10P7	4.906169645	152
chr1.fa	201617450	201796102	NAV1	0.12743009	76
chr1.fa	201777739	201777830	MIR1231	0	0
chr1.fa	201798288	201853422	IPO9	7.550621992	3876
chr1.fa	201857797	201861715	SHISA4	4.07020157	304
chr1.fa	201865584	201915716	LMOD1	0.426101312	76
chr1.fa	201924619	201939789	TIMM17A	19.35691972	1444
chr1.fa	201951766	201975275	RNPEP	35.72267939	3876
chr1.fa	201979690	201986315	ELF3	153.2105533	21584
chr1.fa	202092029	202098634	GPR37L1	0	0
chr1.fa	202101977	202113866	ARL8A	18.01879258	1900
chr1.fa	202116141	202130716	PTPN7	0	0
chr1.fa	202137179	202158577	PTPRVP	0	0
chr1.fa	202163118	202288889	LGR6	0	0
chr1.fa	202300785	202311094	UBE2T	0	0
chr1.fa	202317830	202557697	PPP1R12B	9.411812952	4982
chr1.fa	202559725	202679551	SYT2	0.217276086	76
chr1.fa	202696532	202777549	KDM5B	34.42302591	9880
chr1.fa	202830882	202844369	LOC148709	0.7281084	76
chr1.fa	202848086	202858263	RABIF	6.262532849	684
chr1.fa	202860230	202896371	KLHL12	24.3044541	3648
chr1.fa	202909961	202927700	ADIPOR1	50.70516654	5244
chr1.fa	202931001	202936404	CYB5R1	3.086121035	228

chr1.fa	202955580	202976393	LOC401980	0.902463008	76
chr1.fa	202976534	202976535	TMEM183A	845.0861089	76
chr1.fa	203020311	203047864	PPFIA4	0	0
chr1.fa	203052257	203055377	MYOG	0	0
chr1.fa	203096836	203136533	ADORA1	0	0
chr1.fa	203136939	203144942	MYBPH	0	0
chr1.fa	203148059	203155922	CHI3L1	0	0
chr1.fa	203185207	203198860	CHIT1	0	0
chr1.fa	203267886	203274453	LOC730227	0	0
chr1.fa	203274664	203278729	BTG2	51.74640158	6308
chr1.fa	203309752	203320289	FMOD	13.0187739	1748
chr1.fa	203444883	203460479	PRELP	0	0
chr1.fa	203463271	203478077	OPTC	0	0
chr1.fa	203595928	203713209	ATP2B4	0.189699592	76
chr1.fa	203698709	203698833	SNORA77	0	0
chr1.fa	203734284	203745480	LAX1	0	0
chr1.fa	203764751	203823256	ZC3H11A	30.72888766	6602
chr1.fa	203766651	203769590	ZBED6	15.52200791	2052
chr1.fa	203830740	203840280	SNRPE	16.0432926	1116
chr1.fa	204001575	204010392	C1orf157	0	0
chr1.fa	204042246	204096871	SOX13	9.129376278	1672
chr1.fa	204100190	204121307	ETNK2	18.77959247	2204
chr1.fa	204123944	204135465	REN	0	0
chr1.fa	204159469	204165619	KISS1	0	0
chr1.fa	204167288	204183220	GOLT1A	9.570600184	380
chr1.fa	204187981	204329044	PLEKHA6	28.31060704	9419
chr1.fa	204337558	204338847	LOC127841	0	0
chr1.fa	204372492	204380944	PPP1R15B	11.53475821	2736
chr1.fa	204391758	204459474	PIK3C2B	33.99892612	11628
chr1.fa	204485511	204527248	MDM4	4.530328717	2052
chr1.fa	204586303	204654481	LRRN2	0	0
chr1.fa	204797782	204991950	NFASC	0	0
chr1.fa	205012340	205047171	CNTN2	0	0
chr1.fa	205052257	205053588	TMEM81	1.26896351	76
chr1.fa	205055270	205091150	RBBP5	10.68856015	2120
chr1.fa	205111631	205180727	DSTYK	6.411090092	2280
chr1.fa	205197038	205242471	TMCC2	0.422098272	76
chr1.fa	205271191	205290883	NUAK2	7.454549045	1140
chr1.fa	205305648	205326039	KLHDC8A	0	0
chr1.fa	205350506	205391214	LEMD1	0	0
chr1.fa	205417430	205417526	MIR135B	0	0
chr1.fa	205473684	205501921	CDK18	4.122241083	575
chr1.fa	205523401	205525763	LOC284578	0.715209717	76

chr1.fa	205538112	205572046	MFSD4	2.480772512	456
chr1.fa	205585235	205602000	ELK4	214.3118357	35492
chr1.fa	205626981	205649630	SLC45A3	89.95585804	13680
chr1.fa	205681947	205719372	NUCKS1	52.98311838	15400
chr1.fa	205737114	205744610	RAB7L1	22.48106963	3344
chr1.fa	205758221	205782161	SLC41A1	7.312218753	1596
chr1.fa	205797150	205819276	PM20D1	2.322652453	228
chr1.fa	205882177	205912588	SLC26A9	0.704534946	152
chr1.fa	206138911	206155074	FAM72A	0	0
chr1.fa	206224283	206231482	AVPR1B	0	0
chr1.fa	206238872	206288647	C1orf186	2.030208181	152
chr1.fa	206317459	206332104	CTSE	0.758575978	76
chr1.fa	206516200	206637783	SRGAP2	12.8617658	3455
chr1.fa	206643586	206670223	IKBKE	3.892066314	608
chr1.fa	206680879	206762616	RASSF5	10.99634941	2052
chr1.fa	206764974	206785904	LGTN	24.1799151	2280
chr1.fa	206808881	206822542	DYRK3	3.737726903	380
chr1.fa	206858289	206907626	MAPKAPK2	24.47614002	3950
chr1.fa	206940948	206945839	IL10	0	0
chr1.fa	206972215	207016326	IL19	0.858429574	76
chr1.fa	207039154	207042568	IL20	0	0
chr1.fa	207070788	207076630	IL24	0	0
chr1.fa	207077485	207095378	FAIM3	0.757686414	76
chr1.fa	207101867	207119811	PIGR	8.302526238	1596
chr1.fa	207131312	207143970	FCAMR	1.87787029	228
chr1.fa	207191866	207206101	C1orf116	156.0536009	38608
chr1.fa	207217194	207224422	YOD1	4.046628115	1140
chr1.fa	207226620	207254368	PFKFB2	5.545766394	2280
chr1.fa	207262212	207273337	C4BPB	0	0
chr1.fa	207277607	207318317	C4BPA	0	0
chr1.fa	207494817	207534311	CD55	6.838080968	896
chr1.fa	207627645	207663240	CR2	0	0
chr1.fa	207669473	207815110	CR1	0	0
chr1.fa	207818458	207897036	CR1L	0	0
chr1.fa	207925383	207968861	CD46	9.398914269	1434
chr1.fa	207975197	207975284	MIR29C	0	0
chr1.fa	207975788	207975868	MIR29B2	0	0
chr1.fa	207991724	207995941	LOC148696	0	0
chr1.fa	208059883	208084683	CD34	0	0
chr1.fa	208195588	208417665	PLXNA2	1.329231493	684
chr1.fa	209602168	209605892	LOC642587	0	0
chr1.fa	209605478	209605548	MIR205	0	0
chr1.fa	209757045	209787284	CAMK1G	0	0

chr1.fa	209788218	209825820	LAMB3	0.408754807	76
chr1.fa	209796789	209796849	MIR4260	0	0
chr1.fa	209848670	209849735	G0S2	7.020441654	304
chr1.fa	209859550	209908295	HSD11B1	0	0
chr1.fa	209929394	209955661	TRAF3IP3	0	0
chr1.fa	209955669	209957890	C1orf74	2.175207166	152
chr1.fa	209961262	209979479	IRF6	58.4161325	5700
chr1.fa	210001312	210030910	C1orf107	5.178376328	1976
chr1.fa	210111538	210337633	SYT14	0.314905771	76
chr1.fa	210404804	210407466	C1orf133	2.112715272	76
chr1.fa	210406195	210416440	SERTAD4	0	0
chr1.fa	210501596	210849638	HHAT	0.426546094	76
chr1.fa	210851657	211307457	KCNH1	0	0
chr1.fa	211432708	211489725	RCOR3	6.931485222	1444
chr1.fa	211499957	211548286	TRAF5	2.051780116	380
chr1.fa	211556097	211605877	C1orf97	13.37860267	456
chr1.fa	211649864	211666259	RD3	0	0
chr1.fa	211748381	211752099	SLC30A1	4.187846452	380
chr1.fa	211836122	211848967	NEK2	0	0
chr1.fa	211916799	212004114	LPGAT1	2.823477168	988
chr1.fa	212113741	212208918	INTS7	5.273559711	1064
chr1.fa	212209003	212278187	DTL	0	0
chr1.fa	212250955	212251027	MIR3122	0	0
chr1.fa	212458879	212535205	PPP2R5A	15.24513101	2341
chr1.fa	212526160	212526292	SNORA16B	0	0
chr1.fa	212537816	212588243	TMEM206	0	0
chr1.fa	212606229	212619721	NENF	24.93404325	1064
chr1.fa	212738697	212794116	ATF3	1.358142333	152
chr1.fa	212797789	212800120	FAM71A	0	0
chr1.fa	212859759	212873327	BATF3	0	0
chr1.fa	212899495	212965139	NSL1	1.150651455	684
chr1.fa	212965170	212990167	TATDN3	7.126077418	836
chr1.fa	213003485	213020991	C1orf227	0	0
chr1.fa	213029946	213031480	NCRNA00292	1.794251244	76
chr1.fa	213031597	213070197	FLVCR1	1.477788735	228
chr1.fa	213123887	213164927	VASH2	0	0
chr1.fa	213165524	213189168	ANGEL2	2.908430561	608
chr1.fa	213224588	213446808	RPS6KC1	13.30854948	2508
chr1.fa	214161860	214209762	PROX1	0.550417926	76
chr1.fa	214454565	214510477	SMYD2	23.04416384	1746
chr1.fa	214522039	214725024	PTPN14	6.789377321	4104
chr1.fa	214776532	214837914	CENPF	0	0
chr1.fa	215178885	215410436	KCNK2	0	0

chr1.fa	215740735	215795149	KCTD3	39.47708562	6978
chr1.fa	215796236	216596738	USH2A	0	0
chr1.fa	216676588	217311097	ESRRG	1.667488328	456
chr1.fa	217603834	217804409	GPATCH2	11.57656774	1216
chr1.fa	217804695	218040484	SPATA17	2.737189429	152
chr1.fa	218458629	218511325	RRP15	3.262477163	1140
chr1.fa	218518676	218617961	TGFB2	0.28377102	76
chr1.fa	219347192	219386207	LYPLAL1	13.39995221	1140
chr1.fa	220087606	220101993	SLC30A10	0	0
chr1.fa	220141942	220220000	EPRS	31.98183901	7212
chr1.fa	220230824	220263191	BPNT1	9.614856009	1064
chr1.fa	220267455	220321383	IARS2	15.69702969	2505
chr1.fa	220291195	220291304	MIR215	0	0
chr1.fa	220291499	220291583	MIR194-1	0	0
chr1.fa	220321610	220445843	RAB3GAP2	10.94208598	3606
chr1.fa	220373884	220373887	MIR664	0	0
chr1.fa	220373962	220374018	SNORA36B	0	0
chr1.fa	220439521	220441057	AURKAPS1	0	0
chr1.fa	220701568	220837799	MARK1	9.096240007	2158
chr1.fa	220863628	220872499	C1orf115	50.61420858	6764
chr1.fa	220921676	220957596	MOSC2	5.372523742	380
chr1.fa	220960039	220987741	MOSC1	8.288960382	836
chr1.fa	221052743	221058400	HLX	0.741896647	76
chr1.fa	221503270	221509638	LOC400804	0	0
chr1.fa	221874766	221915461	DUSP10	2.610648903	380
chr1.fa	222695602	222721444	HHIPL2	0	0
chr1.fa	222731665	222763255	TAF1A	5.396986761	456
chr1.fa	222791444	222841351	MIA3	4.785633678	1748
chr1.fa	222841355	222885864	AIDA	6.184918362	836
chr1.fa	222885906	222906106	BROX	21.11447643	1596
chr1.fa	222910558	222924002	FAM177B	0	0
chr1.fa	223101783	223179335	DISP1	1.42530444	304
chr1.fa	223282748	223316624	TLR5	0.792157032	152
chr1.fa	223394161	223537544	SUSD4	1.575640811	228
chr1.fa	223566715	223568812	C1orf65	0	0
chr1.fa	223714972	223853436	CAPN8	0	0
chr1.fa	223889295	223963720	CAPN2	31.01065716	5197
chr1.fa	223967595	224033674	TP53BP2	16.94064062	3648
chr1.fa	224301791	224349749	FBXO28	13.33768271	3268
chr1.fa	224370928	224381142	DEGS1	4.132471073	380
chr1.fa	224415036	224517872	NVL	9.305732406	1216
chr1.fa	224444706	224444843	MIR320B2	0	0
chr1.fa	224544595	224563695	CNIH4	8.031876292	225

chr1.fa	224572845	224622001	WDR26	23.11933203	7144
chr1.fa	224804179	224928249	CNIH3	0.712541024	76
chr1.fa	225117356	225586996	DNAH14	1.806705144	1140
chr1.fa	225589204	225616519	LBR	4.32928718	760
chr1.fa	225674534	225840845	ENAH	10.77173441	6380
chr1.fa	225965515	225978168	SRP9	35.47271181	2584
chr1.fa	225997797	226033232	EPHX1	4.504753743	380
chr1.fa	226033263	226070420	TMEM63A	7.164995857	1425
chr1.fa	226073982	226076836	LEFTY1	0	0
chr1.fa	226107580	226111965	PYCR2	36.93826904	2812
chr1.fa	226124298	226129083	LEFTY2	0	0
chr1.fa	226170403	226187066	C1orf55	7.588650867	1368
chr1.fa	226250421	226250427	H3F3A	241.4531104	76
chr1.fa	226332380	226374423	ACBD3	31.29064753	5016
chr1.fa	226411383	226413513	MIXL1	0	0
chr1.fa	226418861	226497198	LIN9	0.50794123	76
chr1.fa	226548392	226595801	PARP1	30.11353154	5396
chr1.fa	226736501	226796915	C1orf95	0.219277606	76
chr1.fa	226819391	226926876	ITPKB	13.440205	3724
chr1.fa	227058273	227083804	PSEN2	5.148575923	532
chr1.fa	227127938	227175246	ADCK3	15.02896688	1976
chr1.fa	227177566	227505826	CDC42BPA	10.13525114	4788
chr1.fa	227751220	227850164	ZNF678	0.974072936	380
chr1.fa	227884733	227885408	ZNF847P	0	0
chr1.fa	227916240	227922055	LOC100130093	0	0
chr1.fa	227918890	227922517	JMJD4	0	0
chr1.fa	227922697	227968932	SNAP47	14.60753578	1292
chr1.fa	228003418	228034171	PRSS38	0	0
chr1.fa	228109165	228135676	WNT9A	0	0
chr1.fa	228194752	228248961	WNT3A	0	0
chr1.fa	228270361	228286913	ARF1	250.3663226	23788
chr1.fa	228284964	228285042	MIR3620	0	0
chr1.fa	228288428	228291022	C1orf35	13.10217055	760
chr1.fa	228294380	228297013	MRPL55	16.84567963	684
chr1.fa	228327929	228336655	GUK1	124.0697603	6717
chr1.fa	228337553	228347527	GJC2	0	0
chr1.fa	228353429	228369958	C1orf69	2.993828736	1064
chr1.fa	228395861	228566575	OBSCN	1.051242641	1216
chr1.fa	228581377	228594517	TRIM11	17.99944456	2178
chr1.fa	228595636	228604583	TRIM17	1.951036956	228
chr1.fa	228612546	228613026	HIST3H3	0	0
chr1.fa	228645065	228645560	HIST3H2A	17.03804791	380
chr1.fa	228645808	228646259	HIST3H2BB	0	0

chr1.fa	228675068	228683889	RNF187	59.34216896	8360
chr1.fa	228780657	228788159	DUSP5P	0	0
chr1.fa	228870869	228882411	RHOU	64.13425198	12464
chr1.fa	229406879	229440128	RAB4A	90.73178052	5928
chr1.fa	229440519	229441250	SPHAR	0	0
chr1.fa	229456752	229478688	C1orf96	0	0
chr1.fa	229566993	229569843	ACTA1	0	0
chr1.fa	229577044	229644088	NUP133	14.18610468	2660
chr1.fa	229652329	229694442	ABCB10	1.314553682	228
chr1.fa	229728866	229761794	TAF5L	4.146036929	1140
chr1.fa	229761981	229795946	URB2	4.215645337	1064
chr1.fa	230202956	230417875	GALNT2	4.927519188	988
chr1.fa	230457392	230513391	PGBD5	0	0
chr1.fa	230778202	230829731	COG2	18.27209602	2432
chr1.fa	230838272	230850336	AGT	1.309661078	152
chr1.fa	230883130	230937518	CAPN9	2.862173216	304
chr1.fa	230972865	231005335	C1orf198	19.22504181	3572
chr1.fa	231041987	231114618	TTC13	0.514390571	76
chr1.fa	231114823	231136479	ARV1	8.18777244	532
chr1.fa	231154704	231175995	FAM89A	2.412720841	152
chr1.fa	231298674	231357314	TRIM67	1.388387521	532
chr1.fa	231359509	231376918	C1orf131	4.731147864	304
chr1.fa	231376925	231413719	GNPAT	18.33014009	2204
chr1.fa	231468482	231473578	EXOC8	7.626902133	1748
chr1.fa	231473682	231489989	C1orf124	0.603569395	76
chr1.fa	231499497	231560790	EGLN1	7.382716726	2356
chr1.fa	231611510	231612271	SNRPD2P2	0	0
chr1.fa	231700274	231702269	TSNAX	5.92738949	532
chr1.fa	231727038	231747836	LOC100287814	0	0
chr1.fa	231752390	231753805	TSNAX-DISC1	0	0
chr1.fa	231762561	232095249	DISC1	0	0
chr1.fa	231950372	231954074	DISC2	0	0
chr1.fa	232533712	232651243	SIPA1L2	15.63053476	4560
chr1.fa	232940638	232946092	KIAA1383	0.92959472	228
chr1.fa	233086370	233114219	NTPCR	22.48573984	912
chr1.fa	233119882	233431459	PCNXL2	7.304212674	2239
chr1.fa	233463514	233520894	KIAA1804	5.819085033	1520
chr1.fa	233749750	233808258	KCNK1	0.782149433	76
chr1.fa	234040679	234460262	SLC35F3	0.584666153	76
chr1.fa	234509429	234519791	C1orf31	18.45734779	532
chr1.fa	234527059	234614849	TARBP1	9.225004443	2128
chr1.fa	234740015	234745271	IRF2BP2	21.74784623	4560
chr1.fa	234765057	234770526	NCRNA00184	0	0

chr1.fa	235272658	235292256	TOMM20	64.93686139	9884
chr1.fa	235291118	235291252	SNORA14B	0	0
chr1.fa	235294498	235324571	RBM34	15.44172473	1438
chr1.fa	235330210	235491532	ARID4B	15.87939038	4332
chr1.fa	235491753	235507844	GGPS1	14.23191725	1900
chr1.fa	235530728	235606226	TBCE	9.994477585	684
chr1.fa	235610533	235667781	B3GALNT2	11.31459104	2204
chr1.fa	235710985	235814054	GNG4	22.27268918	5320
chr1.fa	235824345	236030220	LYST	3.511777565	2128
chr1.fa	236016300	236016360	MIR1537	0	0
chr1.fa	236139132	236228481	NID1	0	0
chr1.fa	236305832	236372209	GPR137B	9.082896542	834
chr1.fa	236378422	236445339	ERO1LB	0.668952373	152
chr1.fa	236557680	236648008	EDARADD	6.722659997	912
chr1.fa	236681565	236712304	LGALS8	21.36555596	2128
chr1.fa	236686369	236687808	LOC100287902	0	0
chr1.fa	236712997	236767841	HEATR1	8.242703037	2888
chr1.fa	236849770	236927558	ACTN2	0	0
chr1.fa	236958581	237067281	MTR	11.18693856	5311
chr1.fa	237205702	237997288	RYR2	0	0
chr1.fa	238025475	238091619	LOC100130331	0	0
chr1.fa	238041164	238054222	ZP4	0	0
chr1.fa	238643684	238649317	LOC339535	3.311847983	228
chr1.fa	239792373	240072717	CHRM3	0.615800904	76
chr1.fa	240170824	240176560	RPS7P5	0	0
chr1.fa	240255185	240638489	FMN2	0	0
chr1.fa	240652873	240775462	GREM2	0	0
chr1.fa	240938817	241520478	RGS7	0	0
chr1.fa	241295572	241295646	MIR3123	0	0
chr1.fa	241660857	241683085	FH	34.40100919	2888
chr1.fa	241695680	241758944	KMO	2.433403212	380
chr1.fa	241761048	241803701	OPN3	0	0
chr1.fa	241792167	241799232	CHML	1.674382451	532
chr1.fa	241815580	241965434	WDR64	0	0
chr1.fa	242011493	242053241	EXO1	0	0
chr1.fa	242158792	242162385	MAP1LC3C	0	0
chr1.fa	242251689	242687998	PLD5	0.491261899	76
chr1.fa	243219616	243265046	LOC731275	0	0
chr1.fa	243287730	243418708	CEP170	4.685780083	1520
chr1.fa	243419307	243663393	SDCCAG8	14.37891775	1672
chr1.fa	243651535	244006553	AKT3	38.88508057	6688
chr1.fa	244080704	244210619	LOC339529	0	0
chr1.fa	244214561	244220778	ZNF238	9.254804848	1824

chr1.fa	244515937	244552393	C1orf100	0	0
chr1.fa	244571794	244615436	ADSS	21.77542273	2724
chr1.fa	244624673	244803662	C1orf101	0	0
chr1.fa	244816352	244872334	PPPDE1	7.847736477	1444
chr1.fa	244998639	244999058	FAM36A	32.19377771	608
chr1.fa	245003940	245010243	NCRNA00201	0	0
chr1.fa	245013602	245027827	HNRNPU	58.01783007	17860
chr1.fa	245133171	245288530	EFCAB2	0.683407793	152
chr1.fa	245318287	245866428	KIF26B	0.695861693	228
chr1.fa	245912642	246670644	SMYD3	8.682370205	684
chr1.fa	246703863	246729565	TFB2M	9.463407683	760
chr1.fa	246729639	246831884	CNST	6.075279559	1520
chr1.fa	246887378	246931440	SCCPDH	71.64128532	7973
chr1.fa	246952919	246954788	LOC149134	3.010063285	152
chr1.fa	247002402	247094726	AHCTF1	6.6452679	2579
chr1.fa	247148625	247171355	ZNF695	0	0
chr1.fa	247199700	247242115	ZNF670	0.693193	76
chr1.fa	247263264	247267674	ZNF669	0.85353697	76
chr1.fa	247273462	247275719	C1orf229	0	0
chr1.fa	247319203	247335318	ZNF124	39.37678725	2964
chr1.fa	247365269	247365362	MIR3916	0	0
chr1.fa	247419374	247420447	VN1R5	0	0
chr1.fa	247463622	247495045	ZNF496	4.872810982	532
chr1.fa	247579458	247612406	NLRP3	0	0
chr1.fa	247614331	247615284	OR2B11	0	0
chr1.fa	247654370	247655711	OR2W5	0	0
chr1.fa	247687981	247691724	LOC148824	0.99542248	76
chr1.fa	247693434	247697141	OR2C3	0	0
chr1.fa	247712451	247739848	C1orf150	0.646268482	76
chr1.fa	247751662	247752615	OR2G2	0	0
chr1.fa	247768888	247769817	OR2G3	0	0
chr1.fa	247835420	247836343	OR13G1	0	0
chr1.fa	247875131	247876057	OR6F1	0	0
chr1.fa	247920764	247921708	OR1C1	0	0
chr1.fa	247978102	247979031	OR14A16	0	0
chr1.fa	248004230	248005198	OR11L1	0	0
chr1.fa	248020501	248043438	TRIM58	1.311217815	304
chr1.fa	248058889	248059833	OR2W3	0	0
chr1.fa	248084320	248085258	OR2T8	0	0
chr1.fa	248100493	248264224	OR2L13	0	0
chr1.fa	248112160	248113098	OR2L8	0	0
chr1.fa	248128634	248129641	OR2AK2	0	0
chr1.fa	248153569	248154493	OR2L1P	1.827165124	76

chr1.fa	248201474	248202607	OR2L2	0	0
chr1.fa	248223984	248224922	OR2L3	0	0
chr1.fa	248285438	248286082	OR2M1P	0	0
chr1.fa	248308450	248309388	OR2M5	0	0
chr1.fa	248343288	248344331	OR2M2	0	0
chr1.fa	248366370	248367308	OR2M3	0	0
chr1.fa	248402231	248403166	OR2M4	0	0
chr1.fa	248436154	248437116	OR2T33	0	0
chr1.fa	248457918	248458880	OR2T12	0	0
chr1.fa	248486932	248487870	OR2M7	0	0
chr1.fa	248512077	248513015	OR14C36	0	0
chr1.fa	248524883	248525929	OR2T4	0	0
chr1.fa	248550910	248551836	OR2T6	0	0
chr1.fa	248569296	248570405	OR2T1	0	0
chr1.fa	248616099	248617073	OR2T2	0	0
chr1.fa	248636652	248637608	OR2T3	0	0
chr1.fa	248651890	248652837	OR2T5	0	0
chr1.fa	248684948	248685898	OR2G6	0	0
chr1.fa	248721845	248722774	OR2T29	0	0
chr1.fa	248737102	248738058	OR2T34	0	0
chr1.fa	248756131	248757069	OR2T10	0	0
chr1.fa	248789479	248790429	OR2T11	0	0
chr1.fa	248801588	248802559	OR2T35	0	0
chr1.fa	248813232	248814185	OR2T27	0	0
chr1.fa	248844670	248845605	OR14I1	0	0
chr1.fa	248902717	248903151	LOC646627	0	0
chr1.fa	249104651	249120154	SH3BP5L	13.27919386	1900
chr1.fa	249120576	249120642	MIR3124	0	0
chr1.fa	249132530	249143714	ZNF672	14.39181644	1900
chr1.fa	249144203	249153315	ZNF692	3.546248182	380
chr1.fa	249200442	249213345	PGBD2	4.991345429	608
chr10.fa	92828	95504	TUBB8	0	0
chr10.fa	180424	300577	ZMYND11	45.42093205	9348
chr10.fa	320130	735608	DIP2C	6.207602252	2204
chr10.fa	695888	711109	C10orf108	0	0
chr10.fa	855484	931702	LARP4B	35.36173866	8968
chr10.fa	1034349	1063708	GTPBP4	11.34483623	1284
chr10.fa	1064847	1071799	IDI2	2.670027322	152
chr10.fa	1068577	1090141	IDI2-AS1	0	0
chr10.fa	1085964	1095061	IDI1	46.60805565	4114
chr10.fa	1102776	1178237	WDR37	5.864897596	1216
chr10.fa	1205708	1210612	NCRNA00200	0	0
chr10.fa	1228073	1779718	ADARB2	0	0

chr10.fa	1568825	1599179	NCRNA00168	0	0
chr10.fa	3109752	3178996	PFKP	11.62971921	1368
chr10.fa	3179920	3215003	PITRM1	22.9854526	3572
chr10.fa	3818188	3827473	KLF6	37.34190885	7828
chr10.fa	4692377	4720262	LOC100216001	0	0
chr10.fa	4698348	4704606	LOC338588	0	0
chr10.fa	4868402	4890251	AKR1E2	0	0
chr10.fa	4913859	4958465	tAKR	0	0
chr10.fa	5005454	5020158	AKR1C1	3.677014138	228
chr10.fa	5031965	5060207	AKR1C2	12.63670602	1216
chr10.fa	5136568	5149878	AKR1C3	33.14071894	1824
chr10.fa	5196655	5227150	AKR1CL1	0	0
chr10.fa	5238798	5260912	AKR1C4	0	0
chr10.fa	5406976	5416169	UCN3	0	0
chr10.fa	5435061	5446793	TUBAL3	0	0
chr10.fa	5454518	5500426	NET1	27.58405538	4557
chr10.fa	5540658	5541533	CALML5	0	0
chr10.fa	5566924	5568225	CALML3	2.596193483	152
chr10.fa	5680820	5708558	ASB13	13.24205454	1672
chr10.fa	5726801	5805703	C10orf18	12.03736206	4669
chr10.fa	5807186	5855512	GDI2	100.2049734	10868
chr10.fa	5903689	5931860	ANKRD16	3.844252231	456
chr10.fa	5932214	5979556	FBXO18	21.36088575	3648
chr10.fa	5994334	6020142	IL15RA	1.649029868	152
chr10.fa	6052657	6104333	IL2RA	0	0
chr10.fa	6130949	6159422	RBM17	9.387572324	1672
chr10.fa	6186843	6277507	PFKFB3	26.34689379	5548
chr10.fa	6194159	6194240	MIR3155	0	0
chr10.fa	6469105	6622238	PRKCQ	1.037009612	152
chr10.fa	6622387	6627323	LOC439949	0	0
chr10.fa	7200586	7453448	SFMBT2	4.401564281	1596
chr10.fa	7601232	7708961	ITIH5	0	0
chr10.fa	7745236	7791483	ITIH2	0	0
chr10.fa	7797367	7829944	KIN	7.747438099	664
chr10.fa	7830093	7849762	ATP5C1	128.0009675	6596
chr10.fa	7860673	8056714	TAF3	4.240553138	532
chr10.fa	8092413	8095447	FLJ45983	1.499138279	152
chr10.fa	8096667	8117164	GATA3	149.1975062	20596
chr10.fa	10826402	10836877	SFTA1P	0	0
chr10.fa	10976903	10994126	LOC254312	0	0
chr10.fa	11047259	11378672	CELF2	1.00365095	380
chr10.fa	11502509	11653679	USP6NL	8.386145285	1824
chr10.fa	11784356	11806065	ECHDC3	14.30619587	1064

chr10.fa	11865397	11914276	C10orf47	5.578235492	836
chr10.fa	11962021	12085023	UPF2	16.32862036	3876
chr10.fa	12110934	12165224	DHTKD1	21.14338727	4940
chr10.fa	12171640	12211957	SEC61A2	0.981411842	152
chr10.fa	12209573	12237960	NUDT5	15.6968073	679
chr10.fa	12238144	12292589	CDC123	28.36509286	1741
chr10.fa	12391583	12871733	CAMK1D	0.628699587	76
chr10.fa	12875133	12877545	LOC283070	3.502214748	380
chr10.fa	12938625	13043704	CCDC3	0.617357642	76
chr10.fa	13142082	13180276	OPTN	23.02437104	3724
chr10.fa	13203554	13253104	MCM10	0	0
chr10.fa	13263767	13276328	UCMA	0	0
chr10.fa	13319796	13342130	PHYH	27.04964961	2356
chr10.fa	13359438	13390298	SEPHS1	23.33060356	3420
chr10.fa	13480484	13544976	BEND7	3.8275729	532
chr10.fa	13628939	13672868	PRPF18	7.852184299	602
chr10.fa	13685706	14372866	FRMD4A	4.660872282	1426
chr10.fa	14478575	14478660	MIR1265	0	0
chr10.fa	14560559	14816896	FAM107B	30.36505585	5168
chr10.fa	14861251	14879983	CDNF	1.270742639	76
chr10.fa	14880261	14913740	HSPA14	7.487018143	608
chr10.fa	14920782	14946304	SUV39H2	6.072388475	912
chr10.fa	14948871	14996094	DCLRE1C	6.584555135	1216
chr10.fa	15001438	15014850	MEIG1	0	0
chr10.fa	15085895	15115851	OLAH	0	0
chr10.fa	15117474	15130775	ACBD7	0	0
chr10.fa	15137384	15139181	C10orf111	0	0
chr10.fa	15139319	15146256	RPP38	5.818195469	304
chr10.fa	15147771	15210695	NMT2	2.364239585	532
chr10.fa	15196721	15197346	LOC100192204	0	0
chr10.fa	15253644	15413058	FAM171A1	4.279026795	760
chr10.fa	15559088	15761770	ITGA8	0	0
chr10.fa	15820175	15902519	FAM188A	19.21458942	2052
chr10.fa	16478967	16555736	PTER	8.730184287	1520
chr10.fa	16555742	16564004	C1QL3	0	0
chr10.fa	16632617	16859453	RSU1	10.358087	1748
chr10.fa	16865965	17171816	CUBN	7.36514783	3952
chr10.fa	17184982	17244070	TRDMT1	0.440779123	152
chr10.fa	17270258	17279592	VIM	7.912897064	760
chr10.fa	17362676	17496254	ST8SIA6	0	0
chr10.fa	17428935	17450285	LOC100128098	3.034526304	76
chr10.fa	17631958	17659373	PTPLA	1.293204138	76
chr10.fa	17686124	17757907	STAM	12.53663004	1672

chr10.fa	17794251	18089854	FAM23A	0	0
chr10.fa	18240768	18332221	SLC39A12	0	0
chr10.fa	18429606	18830688	CACNB2	0.281991891	76
chr10.fa	18834264	18940550	NSUN6	6.270316537	684
chr10.fa	18948313	18966940	ARL5B	4.238329227	684
chr10.fa	20105372	20569115	PLXDC2	1.303211736	152
chr10.fa	21068903	21463116	NEBL	7.832836274	3496
chr10.fa	21414692	21435488	C10orf113	0	0
chr10.fa	21783421	21786213	C10orf114	0.849756322	76
chr10.fa	21785491	21785570	MIR1915	0	0
chr10.fa	21802409	21814611	C10orf140	1.281195019	380
chr10.fa	21823101	22032559	MLLT10	10.28269643	2728
chr10.fa	22045477	22292650	DNAJC1	4.04151312	380
chr10.fa	22497743	22498912	EBLN1	0	0
chr10.fa	22605299	22609237	COMMD3	33.05309685	1406
chr10.fa	22610006	22620414	BMI1	10.28002773	1647
chr10.fa	22634399	22706539	SPAG6	0	0
chr10.fa	22724354	22726858	LOC100499489	4.723141785	532
chr10.fa	22823766	23003503	PIP4K2A	7.937137692	1368
chr10.fa	23216954	23326514	ARMC3	0	0
chr10.fa	23384427	23410942	MSRB2	10.26601709	836
chr10.fa	23481460	23483181	PTF1A	0	0
chr10.fa	23605520	23633772	C10orf67	0	0
chr10.fa	23728198	23731310	OTUD1	5.972312488	836
chr10.fa	23983675	24836772	KIAA1217	38.664691	14592
chr10.fa	24536054	24544975	PRINS	0	0
chr10.fa	24564614	24564710	MIR603	0	0
chr10.fa	24872538	25012597	ARHGAP21	11.94951758	3851
chr10.fa	25137554	25241533	PRTFDC1	0.612020256	53
chr10.fa	25270917	25305030	ENKUR	0	0
chr10.fa	25305508	25315593	THNSL1	0.89668084	152
chr10.fa	25447001	25448832	LOC100128811	0	0
chr10.fa	25464290	25891157	GPR158	0	0
chr10.fa	26223002	26501465	MYO3A	0.292221881	76
chr10.fa	26505236	26593491	GAD2	0	0
chr10.fa	26727266	26856732	APBB1IP	1.281862193	152
chr10.fa	26878794	26883251	NCRNA00264	0	0
chr10.fa	26932037	26942383	LOC731789	0	0
chr10.fa	26986595	27035524	PDSS1	0	0
chr10.fa	27035727	27150016	ABI1	16.56568925	2660
chr10.fa	27220135	27230930	NCRNA00202	0	0
chr10.fa	27293045	27389427	ANKRD26	2.978706143	908
chr10.fa	27399383	27443321	YME1L1	43.23015751	7828

chr10.fa	27443753	27475848	MASTL	6.059712183	988
chr10.fa	27484143	27531068	ACBD5	23.65017954	4324
chr10.fa	27534782	27541235	LOC387646	0.523730996	152
chr10.fa	27687117	27703297	PTCHD3	0	0
chr10.fa	27793249	27829099	RAB18	37.99774015	4767
chr10.fa	27961803	28034778	MKX	0.464352578	76
chr10.fa	28101097	28287977	ARMC4	0	0
chr10.fa	28339923	28571067	MPP7	27.92053308	6536
chr10.fa	28808846	28821283	LOC220906	3.143942716	760
chr10.fa	28821427	28909923	WAC	44.76132011	7904
chr10.fa	28966424	28971868	BAMBI	13.72531036	1064
chr10.fa	29577990	29600158	LYZL1	0	0
chr10.fa	29698501	29711299	LOC387647	10.86113563	988
chr10.fa	29746277	30024730	SVIL	15.40369586	5920
chr10.fa	29833933	29834026	MIR604	0	0
chr10.fa	29891193	29891275	MIR938	0	0
chr10.fa	30301729	30348488	KIAA1462	4.543449791	1900
chr10.fa	30598730	30638267	MTPAP	2.70672185	684
chr10.fa	30653256	30663377	LOC729668	0	0
chr10.fa	30722866	30750761	MAP3K8	2.183658027	304
chr10.fa	30900708	30918647	LYZL2	0	0
chr10.fa	31133565	31320866	ZNF438	1.32255976	228
chr10.fa	31605457	31608024	LOC220930	0	0
chr10.fa	31608101	31818742	ZEB1	0.793713769	228
chr10.fa	32095225	32217770	ARHGAP12	53.16703581	9974
chr10.fa	32297938	32345371	KIF5B	51.19264779	13556
chr10.fa	32557859	32636113	EPC1	23.20873324	3040
chr10.fa	32735041	32863492	CCDC7	0	0
chr10.fa	32871962	33171792	C10orf68	0	0
chr10.fa	33189246	33247293	ITGB1	7.857521684	1444
chr10.fa	33466419	33623833	NRP1	2.001519732	532
chr10.fa	34398488	35104253	PARD3	19.01554941	5244
chr10.fa	35297479	35379570	CUL2	14.73763457	3108
chr10.fa	35415801	35501886	CREM	3.212439169	532
chr10.fa	35535953	35860847	CCNY	19.77501495	3796
chr10.fa	35894338	35897863	GJD4	0	0
chr10.fa	35927177	35930362	FZD8	0.530402729	76
chr10.fa	37414785	37521495	ANKRD30A	0	0
chr10.fa	37890366	37891859	MTRNR2L7	0	0
chr10.fa	38117899	38146486	ZNF248	3.461294789	760
chr10.fa	38238795	38265453	ZNF25	3.619192457	608
chr10.fa	38299578	38348995	ZNF33A	11.43379266	3147
chr10.fa	38383264	38412278	ZNF37A	2.164087612	684

chr10.fa	38464599	38503273	LOC100129055	0	0
chr10.fa	38645308	38667433	HSD17B7P2	3.619192457	228
chr10.fa	38671998	38691780	SEPT7L	0	0
chr10.fa	38717074	38741081	LOC399744	0.643599789	152
chr10.fa	42827314	42863493	LOC441666	0	0
chr10.fa	42970939	42990785	LOC84856	2.198780621	228
chr10.fa	43008961	43048280	ZNF37BP	1.364369284	532
chr10.fa	43084555	43133992	ZNF33B	11.26877848	3019
chr10.fa	43277954	43330385	BMS1	14.49078047	5244
chr10.fa	43572517	43625797	RET	0	0
chr10.fa	43633934	43680749	CSGALNACT2	4.085768946	684
chr10.fa	43689984	43762367	RASGEF1A	1.567412341	228
chr10.fa	43867092	43871783	FXYD4	35.85366774	1243
chr10.fa	43881065	43904696	HNRNPF	60.48214564	8507
chr10.fa	43932574	43978007	ZNF487P	0	0
chr10.fa	44051793	44070065	ZNF239	2.450749716	228
chr10.fa	44101855	44113352	ZNF485	0.822402219	76
chr10.fa	44139307	44144326	ZNF32	23.78672766	1368
chr10.fa	44282860	44285865	HNRNPA3P1	0	0
chr10.fa	44340754	44346070	C10orf136	0	0
chr10.fa	44404752	44465355	LOC283033	0	0
chr10.fa	44865605	44880545	CXCL12	0.370948324	76
chr10.fa	45306472	45455137	LOC220980	0	0
chr10.fa	45406764	45430642	TMEM72	10.9042795	532
chr10.fa	45455219	45490172	RASSF4	3.387016168	380
chr10.fa	45471709	45474330	C10orf10	4.102448277	380
chr10.fa	45493146	45496470	C10orf25	0	0
chr10.fa	45498728	45500777	ZNF22	13.19157177	1216
chr10.fa	45594924	45650044	LOC100133308	0	0
chr10.fa	45650110	45681489	LOC338579	0	0
chr10.fa	45659462	45659536	MIR3156-1	0	0
chr10.fa	45798102	45811056	OR13A1	0	0
chr10.fa	45869629	45941563	ALOX5	43.71141181	5016
chr10.fa	45952817	46090354	8-Mar	7.26218076	898
chr10.fa	46111039	46168251	ANUBL1	4.081098733	679
chr10.fa	46222648	46288412	FAM21C	4.002594681	836
chr10.fa	46321050	46342921	AGAP4	0	0
chr10.fa	46897641	46939145	FAM35B	0	0
chr10.fa	46955444	46970601	SYT15	0	0
chr10.fa	46993546	47000568	GPRIN2	7.417187343	608
chr10.fa	47083534	47088320	PPYR1	0	0
chr10.fa	47096454	47151400	LOC643650	0.587557237	76
chr10.fa	47133295	47133836	LOC728643	0	0

chr10.fa	47157984	48271368	ANXA8	0	0
chr10.fa	47191844	48237508	AGAP9	0	0
chr10.fa	47242687	48186660	LOC642826	0	0
chr10.fa	47379720	47421238	FAM35B2	0	0
chr10.fa	47658234	47701446	ANTXRL	0	0
chr10.fa	47746920	47763040	ANXA8L2	0	0
chr10.fa	47894023	51837912	FAM21B	0.776589656	152
chr10.fa	48155943	48158691	CTSLL2	0	0
chr10.fa	48355089	48373866	ZNF488	0	0
chr10.fa	48381487	48390991	RBP3	0	0
chr10.fa	48413092	48416853	GDF2	0	0
chr10.fa	48425789	48439166	GDF10	0	0
chr10.fa	48844036	49365413	FRMPD2P1	0	0
chr10.fa	49218159	49239658	LOC399753	0	0
chr10.fa	49382999	49482941	FRMPD2	0.474582567	76
chr10.fa	49609687	49643183	MAPK8	19.14742732	1282
chr10.fa	49654079	49813138	ARHGAP22	0	0
chr10.fa	49893518	50191001	WDFY4	0	0
chr10.fa	50117529	50122280	LRRC18	0	0
chr10.fa	50193557	50193632	MIR4294	0	0
chr10.fa	50222333	50323559	C10orf72	0.210159572	76
chr10.fa	50339201	50342065	FAM170B	0	0
chr10.fa	50363891	50396407	C10orf128	4.412906226	76
chr10.fa	50507187	50535537	C10orf71	0.319798375	76
chr10.fa	50574161	50599907	DRGX	0	0
chr10.fa	50664491	50747147	ERCC6	9.448285089	2660
chr10.fa	50723151	50725167	PGBD3	1.675939189	152
chr10.fa	50817141	50873150	CHAT	0	0
chr10.fa	50818347	50820766	SLC18A3	0	0
chr10.fa	50887684	50916956	C10orf53	0	0
chr10.fa	50942687	50970425	OGDHL	11.23186156	1900
chr10.fa	51026325	51371331	PARG	4.785633678	836
chr10.fa	51224688	51246535	AGAP8	0	0
chr10.fa	51253908	51352122	LOC728407	5.073630129	240
chr10.fa	51464162	51486327	AGAP7	0.698307995	76
chr10.fa	51549553	51562592	MSMB	2224.848859	56924
chr10.fa	51565108	51590734	NCOA4	94.40456923	17328
chr10.fa	51592088	51623336	TIMM23	47.12489252	2475
chr10.fa	51748078	51770259	AGAP6	0	0
chr10.fa	51827684	51829471	FAM21A	13.59387724	228
chr10.fa	51947000	52008370	ASAH2	0	0
chr10.fa	52065345	52383737	SGMS1	6.305009546	1064
chr10.fa	52499708	52514569	ASAH2B	2.674252753	76

chr10.fa	52559169	52645435	A1CF	1.241609407	532
chr10.fa	52750945	54055274	PRKG1	0	0
chr10.fa	53059333	53059415	MIR605	0	0
chr10.fa	53455246	53459355	CSTF2T	6.168461422	1140
chr10.fa	54074041	54077417	DKK1	0	0
chr10.fa	54525140	54531460	MBL2	0	0
chr10.fa	55562533	56561051	PCDH15	0	0
chr10.fa	57358750	57360487	MTRNR2L5	0	0
chr10.fa	58117199	58121034	ZWINT	2.082692476	152
chr10.fa	59064239	59064319	MIR3924	0	0
chr10.fa	59951278	60027694	IPMK	1.653477689	456
chr10.fa	60028895	60049019	CISD1	11.40354748	1064
chr10.fa	60094739	60130509	UBE2D1	2.919327724	348
chr10.fa	60145176	60155897	TFAM	12.22239144	1064
chr10.fa	60272904	60588845	BICC1	12.56331696	1762
chr10.fa	60474775	60477293	LOC728640	0	0
chr10.fa	60936348	61007534	PHYHIPL	2.388480213	228
chr10.fa	61011335	61122661	FAM13C	18.37150484	1672
chr10.fa	61410522	61469649	SLC16A9	2.967364198	532
chr10.fa	61548506	61666414	CCDC6	27.56025953	7220
chr10.fa	61717975	61720671	C10orf40	0	0
chr10.fa	61788159	62149488	ANK3	7.635130603	5396
chr10.fa	62538089	62554610	CDK1	0	0
chr10.fa	62629198	62761198	RHOBTB1	3.527122549	760
chr10.fa	63166401	63213208	TMEM26	0	0
chr10.fa	63422719	63526091	C10orf107	1.349913863	76
chr10.fa	63661013	63856707	ARID5B	27.45662529	9804
chr10.fa	63952953	64028466	RTKN2	0	0
chr10.fa	64133916	64431771	ZNF365	0	0
chr10.fa	64564516	64568239	ADO	6.807835781	1140
chr10.fa	64571756	64578927	EGR2	0	0
chr10.fa	64893007	64914786	NRBF2	5.841324141	498
chr10.fa	64926988	65140242	JMJD1C	17.52174851	6916
chr10.fa	65132717	65132808	MIR1296	0	0
chr10.fa	65224989	65226322	LOC84989	1.951704129	76
chr10.fa	65281123	65384883	REEP3	3.896736527	912
chr10.fa	66585285	66586634	ANXA2P3	0	0
chr10.fa	67679725	69455949	CTNNA3	0	0
chr10.fa	68685792	68860867	LRRTM3	0	0
chr10.fa	69556427	69597937	DNAJC12	3.318297324	228
chr10.fa	69644427	69678147	SIRT1	4.702459414	912
chr10.fa	69681656	69835103	HERC4	15.68101753	3185
chr10.fa	69869250	69971773	MYPN	0	0

chr10.fa	69990382	69991855	ATOH7	0	0
chr10.fa	70042417	70092684	PBLD	15.37411784	1973
chr10.fa	70091768	70102953	HNRNPH3	35.03682529	3800
chr10.fa	70103275	70167051	RUFY2	2.152300885	589
chr10.fa	70173821	70231879	DNA2	2.296410305	456
chr10.fa	70242097	70287584	SLC25A16	46.52465899	5320
chr10.fa	70320117	70454239	TET1	2.82169804	1216
chr10.fa	70480971	70551309	CCAR1	31.98094945	5548
chr10.fa	70514929	70514995	SNORD98	0	0
chr10.fa	70587294	70655209	STOX1	0.940714274	152
chr10.fa	70661034	70706603	DDX50	16.64864113	1900
chr10.fa	70715892	70744279	DDX21	22.36765017	4177
chr10.fa	70748477	70776739	KIAA1279	18.59523026	2128
chr10.fa	70847828	70864567	SRGN	1.347912344	76
chr10.fa	70883908	70932616	VPS26A	18.14688984	2204
chr10.fa	70939993	70968849	SUPV3L1	9.564373233	1064
chr10.fa	70980059	71027315	HKDC1	0.458125627	76
chr10.fa	71029756	71161637	HK1	45.10847258	8588
chr10.fa	71163958	71176674	TACR2	0	0
chr10.fa	71211226	71267423	TSPAN15	8.953242542	684
chr10.fa	71331791	71333210	NEUROG3	0	0
chr10.fa	71390003	71393355	C10orf35	12.56643044	608
chr10.fa	71561644	71718904	COL13A1	0	0
chr10.fa	71812357	71872022	H2AFY2	6.256973072	608
chr10.fa	71872041	71892690	AIFM2	4.710910275	684
chr10.fa	71897733	71906496	TYSND1	4.001037944	684
chr10.fa	71909961	71930285	SAR1A	39.62631004	5472
chr10.fa	71962586	71993190	PPA1	88.32439707	5171
chr10.fa	72014713	72043450	NPFFR1	0	0
chr10.fa	72058729	72142382	LRRC20	1.044570909	152
chr10.fa	72163861	72188374	EIF4EBP2	52.0675343	17632
chr10.fa	72191692	72201465	NODAL	0	0
chr10.fa	72238564	72328206	KIAA1274	0	0
chr10.fa	72357104	72362531	PRF1	0	0
chr10.fa	72432559	72522195	ADAMTS14	0	0
chr10.fa	72530995	72545157	C10orf27	0	0
chr10.fa	72575704	72640932	SGPL1	24.02401895	6232
chr10.fa	72643267	72648541	PCBD1	115.9262437	5244
chr10.fa	72972298	73060749	UNC5B	1.014548113	228
chr10.fa	73079010	73123147	SLC29A3	5.218851505	532
chr10.fa	73156691	73575704	CDH23	0.138994426	76
chr10.fa	73471458	73497581	C10orf105	0	0
chr10.fa	73507314	73533337	C10orf54	0.708315594	152

chr10.fa	73576055	73611082	PSAP	32.80157254	4180
chr10.fa	73724120	73773322	CHST3	0.969625114	304
chr10.fa	73818792	73848790	SPOCK2	0.554420966	152
chr10.fa	73856274	73976183	ASCC1	10.25511993	1216
chr10.fa	73975806	73995618	ANAPC16	37.03033895	5320
chr10.fa	74033677	74035797	DDIT4	17.36474041	1368
chr10.fa	74092588	74114907	DNAJB12	7.927574875	1140
chr10.fa	74119698	74336541	MIR1256	0	0
chr10.fa	74127085	74385949	CBARA1	17.70477637	2128
chr10.fa	74451889	74647452	CCDC109A	9.170073846	1216
chr10.fa	74653339	74692787	OIT3	0	0
chr10.fa	74694938	74714510	PLA2G12B	1.547841926	76
chr10.fa	74766980	74856732	P4HA1	3.369224882	456
chr10.fa	74870210	74891581	NUDT13	1.606553171	152
chr10.fa	74894282	74927853	ECD	12.30000592	1292
chr10.fa	74927877	75001939	FAM149B1	6.475805896	912
chr10.fa	75002582	75007025	DNAJC9	7.057136183	456
chr10.fa	75008601	75012451	MRPS16	30.80049759	3648
chr10.fa	75013516	75118617	TTC18	1.825163604	304
chr10.fa	75135189	75173841	ANXA7	46.778852	4636
chr10.fa	75183337	75193319	ZMYND17	0.697640822	76
chr10.fa	75196563	75255782	PPP3CB	12.35271261	1748
chr10.fa	75257296	75335433	USP54	12.06671768	3344
chr10.fa	75391412	75401515	MYOZ1	0	0
chr10.fa	75404644	75415832	SYNPO2L	0	0
chr10.fa	75434033	75457554	AGAP5	0	0
chr10.fa	75458909	75490272	BMS1P4	3.449285671	304
chr10.fa	75504132	75531919	SEC24C	54.47358341	11096
chr10.fa	75532049	75535976	FUT11	2.467429047	228
chr10.fa	75541808	75543406	CHCHD1	44.05656277	1672
chr10.fa	75545605	75561551	KIAA0913	21.1451664	5548
chr10.fa	75561669	75571589	NDST2	3.806668139	684
chr10.fa	75572259	75634338	CAMK2G	25.27052096	4435
chr10.fa	75669727	75682535	C10orf55	0	0
chr10.fa	75672043	75677258	PLAU	4.401564281	380
chr10.fa	75757872	75879914	VCL	11.70733369	2888
chr10.fa	75880015	75910826	AP3M1	17.6965479	4180
chr10.fa	75910965	76469060	ADK	11.42511941	1140
chr10.fa	76586379	76792639	MYST4	11.66307787	4484
chr10.fa	76797594	76818272	DUPD1	0	0
chr10.fa	76854190	76868970	DUSP13	0	0
chr10.fa	76871393	76941881	SAMD8	3.375896614	1064
chr10.fa	76969912	76991207	VDAC2	10.97188639	1040

chr10.fa	76993729	76995770	COMTD1	8.667470003	380
chr10.fa	77157603	77161285	ZNF503	12.01957077	1596
chr10.fa	77161514	77168740	NCRNA00245	3.998814033	456
chr10.fa	77542519	78317126	C10orf11	1.894772013	76
chr10.fa	78629359	79397577	KCNMA1	0.574658554	380
chr10.fa	79550549	79686348	DLG5	19.88710005	6688
chr10.fa	79686570	79689583	LOC100128292	0	0
chr10.fa	79734907	79789298	POLR3A	7.896884906	2356
chr10.fa	79793518	79816571	RPS24	302.0673564	40232
chr10.fa	80703083	80827205	LOC283050	0.694749738	76
chr10.fa	80828792	81076285	ZMIZ1	29.52463995	10030
chr10.fa	81107220	81115089	PPIF	8.450861089	836
chr10.fa	81142083	81205383	ZCCHC24	2.742082032	608
chr10.fa	81272357	81276192	EIF5AL1	0.881113464	152
chr10.fa	81315608	81320163	SFTPA2	0.767916404	76
chr10.fa	81370695	81375199	SFTPA1	0	0
chr10.fa	81442731	81448650	LOC650623	0.856428054	228
chr10.fa	81585658	81587358	LOC642361	0.993643351	76
chr10.fa	81664654	81691557	LOC100288974	0	0
chr10.fa	81679934	81682875	MBL1P	0	0
chr10.fa	81697496	81708861	SFTPD	0	0
chr10.fa	81805989	81838949	LOC219347	0	0
chr10.fa	81838426	81852307	C10orf57	11.11955406	988
chr10.fa	81892258	81904784	PLAC9	0	0
chr10.fa	81914880	81965328	ANXA11	118.7388237	14576
chr10.fa	82031576	82049434	MAT1A	0	0
chr10.fa	82095862	82116500	DYDC1	0	0
chr10.fa	82116558	82127829	DYDC2	0	0
chr10.fa	82168242	82192753	C10orf58	29.27111412	2356
chr10.fa	82214038	82282391	TSPAN14	7.573750665	1900
chr10.fa	82297658	82406316	SH2D4B	0	0
chr10.fa	83635070	84746935	NRG3	0	0
chr10.fa	85899185	85913311	GHITM	95.93061683	10564
chr10.fa	85933554	85945050	C10orf99	6.123760815	228
chr10.fa	85954412	85979376	CDHR1	0	0
chr10.fa	85980249	85985284	LRIT2	0	0
chr10.fa	85991276	86001217	LRIT1	0	0
chr10.fa	86004809	86018944	RGR	0	0
chr10.fa	86088410	86278276	FAM190B	7.70651814	2660
chr10.fa	87359312	88126250	GRID1	0	0
chr10.fa	88024451	88024545	MIR346	0	0
chr10.fa	88195013	88281541	WAPAL	24.40586444	6916
chr10.fa	88414314	88426216	OPN4	0	0

chr10.fa	88428206	88495824	LDB3	0.25908561	76
chr10.fa	88516396	88684945	BMPR1A	6.543857567	1064
chr10.fa	88695298	88717425	MMRN2	9.706703525	1824
chr10.fa	88718288	88723017	SNCG	317.028494	11775
chr10.fa	88728188	88730497	C10orf116	1058.501931	23941
chr10.fa	88730667	88769960	AGAP11	2.264163599	304
chr10.fa	88780046	88784487	FAM25A	0	0
chr10.fa	88809959	88854776	GLUD1	73.62056594	11507
chr10.fa	88854953	88951222	FAM35A	10.42702824	1596
chr10.fa	88985205	88994733	FAM22A	0	0
chr10.fa	88998424	89102167	LOC728190	5.015363665	152
chr10.fa	89102316	89103331	LOC439994	0	0
chr10.fa	89117477	89130452	FAM22D	0	0
chr10.fa	89264223	89313218	MINPP1	1.023443756	152
chr10.fa	89419476	89507462	PAPSS2	3.50466105	608
chr10.fa	89512875	89577917	ATAD1	5.570674195	760
chr10.fa	89578070	89605369	CFLP1	0	0
chr10.fa	89618918	89623194	KILLIN	1.580755806	304
chr10.fa	89623195	89728532	PTEN	17.36785389	4332
chr10.fa	90033621	90343082	RNLS	3.300283646	532
chr10.fa	90346519	90366733	LIPJ	0	0
chr10.fa	90424146	90438572	LIPF	0	0
chr10.fa	90484301	90512513	LIPK	0	0
chr10.fa	90521163	90537999	LIPN	0	0
chr10.fa	90562487	90577653	LIPM	0	0
chr10.fa	90579659	90611732	ANKRD22	1.382382961	228
chr10.fa	90640026	90683244	STAMBPL1	1.69261852	152
chr10.fa	90694831	90751147	ACTA2	0.91313778	76
chr10.fa	90750288	90775542	FAS	0.901573444	109
chr10.fa	90751181	90752732	FAS-AS1	0	0
chr10.fa	90965694	90967071	CH25H	0	0
chr10.fa	90973326	91011660	LIPA	2.544376361	304
chr10.fa	91061706	91069033	IFIT2	2.418058227	380
chr10.fa	91087602	91100725	IFIT3	8.174206584	988
chr10.fa	91137813	91144962	IFIT1B	0	0
chr10.fa	91152322	91163744	IFIT1	3.634759833	304
chr10.fa	91174325	91180759	IFIT5	3.351878377	608
chr10.fa	91190051	91295313	SLC16A12	2.559721346	532
chr10.fa	91342749	91405215	PANK1	8.068793212	1292
chr10.fa	91352504	91352584	MIR107	0	0
chr10.fa	91451057	91457685	FLJ37201	0	0
chr10.fa	91461367	91534700	KIF20B	0.267758862	76
chr10.fa	92500576	92617671	HTR7	0	0

chr10.fa	92631709	92668312	RPP30	2.581960454	496
chr10.fa	92671857	92681032	ANKRD1	0	0
chr10.fa	92911761	92912837	NUDT9P1	0	0
chr10.fa	92980369	93044021	PCGF5	5.928723836	1900
chr10.fa	93066719	93371217	LOC100188947	3.304286686	152
chr10.fa	93170096	93274520	HECTD2	0.845975673	222
chr10.fa	93388199	93392858	PPP1R3C	46.01782972	5320
chr10.fa	93558151	93625232	TNKS2	28.28636641	7980
chr10.fa	93666345	93669258	FGFBP3	1.31833433	152
chr10.fa	93683736	93790080	BTAF1	10.0863251	3192
chr10.fa	93808397	94050875	CPEB3	9.038418326	2432
chr10.fa	94050920	94113721	5-Mar	11.62371465	2052
chr10.fa	94211441	94333852	IDE	14.11760823	3800
chr10.fa	94352825	94415152	KIF11	1.331455404	304
chr10.fa	94449681	94455408	HHEX	0	0
chr10.fa	94594470	94819251	EXOC6	7.603773461	1276
chr10.fa	94821021	94828454	CYP26C1	0	0
chr10.fa	94833232	94837641	CYP26A1	0	0
chr10.fa	95066186	95242074	MYOF	32.87273768	10115
chr10.fa	95256369	95288849	CEP55	0	0
chr10.fa	95326422	95349829	GPR120	0	0
chr10.fa	95351593	95360993	RBP4	23.65151389	988
chr10.fa	95372345	95425429	PDE6C	0	0
chr10.fa	95427640	95462329	FRA10AC1	4.852351003	674
chr10.fa	95517566	95557916	LGI1	0	0
chr10.fa	95653730	95662491	TMEM20	0	0
chr10.fa	95717897	95721672	PIPSL	0.895124102	152
chr10.fa	95753746	96088148	PLCE1	74.15297019	27225
chr10.fa	96039047	96046827	LOC100128054	0	0
chr10.fa	96092989	96122683	NOC3L	5.90270408	912
chr10.fa	96162186	96296089	TBC1D12	5.585796789	1444
chr10.fa	96305574	96361856	HELLS	0	0
chr10.fa	96443251	96495947	CYP2C18	0	0
chr10.fa	96522463	96612671	CYP2C19	0	0
chr10.fa	96698415	96749148	CYP2C9	0	0
chr10.fa	96796529	96829254	CYP2C8	0	0
chr10.fa	96953957	96988686	C10orf129	0	0
chr10.fa	96997330	97050781	PDLIM1	41.93406229	2736
chr10.fa	97071530	97321171	SORBS1	2.289293791	759
chr10.fa	97365686	97416567	ALDH18A1	13.66593195	2128
chr10.fa	97423153	97453900	TCTN3	4.319501972	532
chr10.fa	97471536	97637023	ENTPD1	0.905131701	532
chr10.fa	97667722	97698415	C10orf131	0	0

chr10.fa	97759848	97792441	CC2D2B	0	0
chr10.fa	97803159	97820625	CCNJ	0.816842442	152
chr10.fa	97824072	97824156	MIR3157	0	0
chr10.fa	97889472	97923517	ZNF518A	4.890157487	1824
chr10.fa	97951455	98031333	BLNK	20.72640399	1699
chr10.fa	98064085	98098321	DNTT	0	0
chr10.fa	98102975	98119092	OPALIN	0	0
chr10.fa	98124363	98273683	TLL2	0.499267978	152
chr10.fa	98277867	98346809	TM9SF3	10.73570706	2964
chr10.fa	98353069	98480279	PIK3AP1	2.459200577	532
chr10.fa	98592017	98724198	LCOR	9.35799431	3344
chr10.fa	98741041	98745585	C10orf12	10.41257282	2128
chr10.fa	98757795	98945683	SLIT1	0.213050656	76
chr10.fa	98981930	99052413	ARHGAP19	3.403250717	836
chr10.fa	99079022	99081672	FRAT1	2.550158529	304
chr10.fa	99092254	99094458	FRAT2	8.431735456	836
chr10.fa	99116458	99161127	RRP12	6.491818054	1292
chr10.fa	99186027	99193198	PGAM1	12.77458849	988
chr10.fa	99195666	99205768	EXOSC1	15.95411378	825
chr10.fa	99205930	99217127	ZDHHC16	16.22698764	1444
chr10.fa	99218081	99258366	MMS19	10.50419794	1741
chr10.fa	99258768	99330960	UBTD1	5.42767673	380
chr10.fa	99332256	99343641	ANKRD2	0	0
chr10.fa	99344102	99372555	HOGA1	9.533683264	1064
chr10.fa	99349450	99350691	C10orf62	0	0
chr10.fa	99374310	99393913	MORN4	5.909598203	684
chr10.fa	99400443	99436187	PI4K2A	8.077244073	1520
chr10.fa	99437181	99447015	AVPI1	3.642543521	228
chr10.fa	99473465	99477909	MARVELD1	0	0
chr10.fa	99496878	99520664	ZFYVE27	2.75809419	380
chr10.fa	99526508	99531756	SFRP5	0	0
chr10.fa	99609996	99627782	GOLGA7B	0	0
chr10.fa	99624758	99790585	CRTAC1	0	0
chr10.fa	99894381	100004654	C10orf28	15.54291267	2356
chr10.fa	100007443	100028007	LOXL4	0.462128667	76
chr10.fa	100143322	100174978	PYROXD2	4.248781608	380
chr10.fa	100155051	100155064	MIR1287	0	0
chr10.fa	100175956	100206704	HPS1	14.94045523	2788
chr10.fa	100216834	100995632	HPSE2	0	0
chr10.fa	101088856	101154087	CNNM1	0.283548629	76
chr10.fa	101156627	101190530	GOT1	20.66035384	1976
chr10.fa	101292690	101296280	NKX2-3	3.225560243	304
chr10.fa	101370275	101380221	SLC25A28	11.95618931	836

chr10.fa	101419263	101468504	ENTPD7	2.790340897	760
chr10.fa	101471003	101492423	COX15	10.17772784	2280
chr10.fa	101496003	101515894	CUTC	4.893048571	255
chr10.fa	101542463	101611662	ABCC2	2.007746682	456
chr10.fa	101635334	101769676	DNMBP	17.15991822	4409
chr10.fa	101686966	101718755	NCRNA00093	0	0
chr10.fa	101802065	101841642	CPN1	0	0
chr10.fa	101909847	101945814	ERLIN1	1.921236551	304
chr10.fa	101948124	101989344	CHUK	10.07186968	1596
chr10.fa	101992055	102027437	CWF19L1	3.209548085	380
chr10.fa	101996913	101997059	SNORA12	0	0
chr10.fa	102033713	102046439	BLOC1S2	12.08495375	1064
chr10.fa	102047903	102090243	PKD2L1	1.107952367	152
chr10.fa	102106772	102124588	SCD	24.41898551	6004
chr10.fa	102133333	102148111	NCRNA00263	35.92461049	1596
chr10.fa	102222812	102243399	WNT8B	0.846865238	76
chr10.fa	102246403	102279595	SEC31B	0.366500502	76
chr10.fa	102283497	102289636	NDUFB8	113.3351652	3496
chr10.fa	102295641	102313681	HIF1AN	11.98176429	3724
chr10.fa	102505468	102589698	PAX2	3.212439169	608
chr10.fa	102672326	102724891	FAM178A	8.241813473	2735
chr10.fa	102732286	102745372	SEMA4G	6.81384034	1122
chr10.fa	102734742	102734841	MIR608	0	0
chr10.fa	102737579	102747272	MRPL43	18.55631182	1824
chr10.fa	102747293	102754158	C10orf2	5.601141774	912
chr10.fa	102756864	102767439	LZTS2	15.05721055	1824
chr10.fa	102767594	102790914	PDZD7	0.396300907	76
chr10.fa	102790996	102800998	SFXN3	7.657814493	1064
chr10.fa	102820999	102825351	KAZALD1	0.672288239	76
chr10.fa	102849078	102890903	TLX1NB	0	0
chr10.fa	102891061	102897546	TLX1	0	0
chr10.fa	102986733	102988717	LBX1	0	0
chr10.fa	102989351	102998616	FLJ41350	0	0
chr10.fa	103113825	103317070	BTRC	8.007635664	2204
chr10.fa	103338639	103348027	POLL	2.941566832	380
chr10.fa	103348089	103369410	DPCD	14.80746537	532
chr10.fa	103370421	103454743	FBXW4	61.12730217	6080
chr10.fa	103529887	103535759	FGF8	0	0
chr10.fa	103541082	103543170	NPM3	11.56322427	456
chr10.fa	103544200	103578222	MGEA5	40.20741793	9405
chr10.fa	103585731	103603677	KCNIP2	0	0
chr10.fa	103605356	103815932	C10orf76	9.365333216	1727
chr10.fa	103825147	103827793	HPS6	5.746807932	684

chr10.fa	103867325	103880210	LDB1	16.35597447	1960
chr10.fa	103892787	103910090	PPRC1	11.08196997	2660
chr10.fa	103911933	103923627	NOLC1	26.14340595	4627
chr10.fa	103986143	103989344	ELOVL3	0	0
chr10.fa	103989946	104001231	PITX3	0	0
chr10.fa	104005255	104142656	GBF1	27.50888719	7966
chr10.fa	104154229	104162281	NFKB2	8.582071827	1292
chr10.fa	104162376	104178901	PSD	0.404084595	76
chr10.fa	104179571	104182893	FBXL15	2.862173216	304
chr10.fa	104183002	104192423	CUEDC2	15.34053679	825
chr10.fa	104196269	104196341	MIR146B	0	0
chr10.fa	104209594	104211300	C10orf95	8.053893009	532
chr10.fa	104221170	104236802	TMEM180	0.59645288	76
chr10.fa	104238986	104262512	ACTR1A	38.02242556	4912
chr10.fa	104263719	104393214	SUFU	26.87329348	6460
chr10.fa	104404252	104418076	TRIM8	57.37267354	7079
chr10.fa	104433484	104474190	ARL3	9.598399069	1672
chr10.fa	104474298	104498946	SFXN2	12.37828759	1426
chr10.fa	104503727	104576021	C10orf26	14.99471866	2964
chr10.fa	104590288	104597290	CYP17A1	11.74981039	988
chr10.fa	104629210	104629350	AS3MT	0	0
chr10.fa	104678114	104838240	CNNM2	2.34266765	456
chr10.fa	104845940	104953056	NT5C2	30.88411663	7445
chr10.fa	105005644	105007773	LOC729020	0	0
chr10.fa	105036920	105050108	INA	2.092477684	304
chr10.fa	105062553	105110891	PCGF6	0.757908805	76
chr10.fa	105127724	105148813	TAF5	1.555848005	228
chr10.fa	105148823	105156223	USMG5	124.3066068	2247
chr10.fa	105154152	105154158	MIR1307	0	0
chr10.fa	105156412	105206019	PDCD11	6.544079958	1888
chr10.fa	105206543	105212162	CALHM2	1.831168163	152
chr10.fa	105213144	105218648	CALHM1	0	0
chr10.fa	105232561	105238997	CALHM3	0	0
chr10.fa	105253735	105352309	NEURL	0	0
chr10.fa	105353784	105615164	SH3PXD2A	5.53664836	2800
chr10.fa	105637318	105678045	OBFC1	10.43147606	3040
chr10.fa	105727470	105787342	SLK	74.34600565	18741
chr10.fa	105791046	105845638	COL17A1	0	0
chr10.fa	105807863	105807944	MIR936	0	0
chr10.fa	105881816	105886143	C10orf78	1.105506065	76
chr10.fa	105889646	105992120	C10orf79	0.945162096	228
chr10.fa	105978547	105978641	MIR609	0	0
chr10.fa	106013952	106027222	GSTO1	42.57677251	2508

chr10.fa	106028631	106059176	GSTO2	14.87262595	1064
chr10.fa	106071899	106093663	ITPRIP	0.774588136	152
chr10.fa	106113522	106214848	CCDC147	1.020330281	152
chr10.fa	106400859	107024993	SORCS3	0	0
chr10.fa	108333421	108924292	SORCS1	0	0
chr10.fa	111624524	111683311	XPNPEP1	29.7105589	3392
chr10.fa	111765726	111895323	ADD3	45.28549588	9424
chr10.fa	111967363	112047123	MXI1	7.051576406	1216
chr10.fa	112052798	112064707	SMNDC1	4.998017161	456
chr10.fa	112257625	112271302	DUSP5	4.679997915	532
chr10.fa	112327449	112364392	SMC3	11.91993957	2204
chr10.fa	112404155	112599227	RBM20	0.467243662	152
chr10.fa	112628648	112630662	LOC282997	0.838859159	76
chr10.fa	112631553	112658487	PDCD4	103.3231188	11248
chr10.fa	112659765	112679124	BBIP1	4.28614331	228
chr10.fa	112679301	112773425	SHOC2	23.98020791	4256
chr10.fa	112696361	112697013	RPL13AP6	5.176597199	152
chr10.fa	112836790	112840662	ADRA2A	0	0
chr10.fa	113909622	113943525	GPAM	2.652903209	760
chr10.fa	114043493	114063070	TECTB	0	0
chr10.fa	114067936	114116353	GUCY2GP	0	0
chr10.fa	114133916	114188138	ACSL5	4.062862664	684
chr10.fa	114190058	114206672	ZDHHC6	15.36233111	1499
chr10.fa	114206756	114578504	VTI1A	8.081469503	1596
chr10.fa	114393929	114394013	MIR4295	0	0
chr10.fa	114610673	114615127	LOC143188	0	0
chr10.fa	114710009	114927436	TCF7L2	15.93654489	3100
chr10.fa	115310590	115349360	HABP2	0	0
chr10.fa	115349425	115423805	NRAP	0	0
chr10.fa	115438935	115490664	CASP7	15.29138836	1950
chr10.fa	115511213	115542192	C10orf81	1.994625608	380
chr10.fa	115594484	115613859	DCLRE1A	6.430882898	1292
chr10.fa	115614391	115672265	NHLRC2	10.31227444	2964
chr10.fa	115803806	115806667	ADRB1	2.362238066	304
chr10.fa	115881974	115934364	C10orf118	15.26114317	4256
chr10.fa	115939029	115992063	TDRD1	0	0
chr10.fa	115999018	116049751	VWA2	0	0
chr10.fa	116054583	116164515	AFAP1L2	18.27899015	3268
chr10.fa	116190869	116444414	ABLIM1	22.53644501	8414
chr10.fa	116581503	116659586	FAM160B1	9.075112855	2508
chr10.fa	116697952	116737439	TRUB1	4.953538945	760
chr10.fa	116853124	117708496	ATRNL1	0	0
chr10.fa	117816442	118032796	GFRA1	0	0

chr10.fa	118083940	118139541	C10orf96	0	0
chr10.fa	118187424	118237468	PNLIPRP3	0	0
chr10.fa	118305428	118327367	PNLIP	0	0
chr10.fa	118350490	118368686	PNLIPRP1	0	0
chr10.fa	118380465	118404654	PNLIPRP2	0	0
chr10.fa	118423207	118429481	C10orf82	3.400804415	152
chr10.fa	118430703	118502085	HSPA12A	2.36312763	608
chr10.fa	118644306	118765088	KIAA1598	12.28421616	2178
chr10.fa	118888032	118897812	VAX1	0	0
chr10.fa	118927189	118927285	MIR3663	0	0
chr10.fa	118957000	118969810	KCNK18	0	0
chr10.fa	119000584	119038941	SLC18A2	0.436553693	76
chr10.fa	119042606	119134937	PDZD8	4.845456879	836
chr10.fa	119243804	119304579	EMX2OS	0.232176289	76
chr10.fa	119301956	119309057	EMX2	25.67949816	3344
chr10.fa	119764427	119806114	RAB11FIP2	21.60796224	5889
chr10.fa	119806332	119969665	CASC2	0.648270002	152
chr10.fa	120068572	120101839	C10orf84	15.88739646	1976
chr10.fa	120352916	120355160	PRLHR	0	0
chr10.fa	120440494	120514758	C10orf46	18.05526472	5472
chr10.fa	120789228	120793854	NANOS1	2.191664106	456
chr10.fa	120794541	120840334	EIF3A	98.12873028	23408
chr10.fa	120819525	120819650	SNORA19	13.41396285	76
chr10.fa	120863611	120863628	FAM45A	0	0
chr10.fa	120897224	120897376	FAM45B	0	0
chr10.fa	120900425	120925204	SFXN4	8.251598681	515
chr10.fa	120927215	120938345	PRDX3	99.85937767	7144
chr10.fa	120967197	121215131	GRK5	1.321447805	152
chr10.fa	121259339	121302222	RGS10	38.17632019	1672
chr10.fa	121332978	121356541	TIAL1	14.33443954	2508
chr10.fa	121410882	121437329	BAG3	23.02681734	2660
chr10.fa	121485609	121588659	INPP5F	3.813784653	912
chr10.fa	121588972	121632394	MCMBP	11.49450543	2192
chr10.fa	121652223	121701245	SEC23IP	23.9005919	4560
chr10.fa	122216466	122349367	PPAPDC1A	3.333642309	228
chr10.fa	122521324	122610686	LOC283089	0	0
chr10.fa	122610692	122669038	WDR11	21.08689994	4484
chr10.fa	123237844	123357972	FGFR2	2.879964503	684
chr10.fa	123502625	123687546	ATE1	32.18866272	3384
chr10.fa	123716603	123734743	NSMCE4A	24.05937913	1520
chr10.fa	123748689	124014057	TACC2	38.6497908	17546
chr10.fa	124030821	124097676	BTBD16	0	0
chr10.fa	124134094	124191871	PLEKHA1	6.415760304	1216

chr10.fa	124176481	124176583	MIR3941	0	0
chr10.fa	124214179	124216868	ARMS2	0	0
chr10.fa	124221041	124274424	HTRA1	1.594544053	152
chr10.fa	124320181	124403252	DMBT1	0	0
chr10.fa	124457225	124459338	C10orf120	0	0
chr10.fa	124516210	124557161	FLJ46361	0	0
chr10.fa	124591674	124605691	CUZD1	0	0
chr10.fa	124608610	124639148	FAM24B	2.215237561	76
chr10.fa	124639158	124658230	LOC399815	0	0
chr10.fa	124670217	124672627	FAM24A	0	0
chr10.fa	124690419	124713919	C10orf88	1.095053684	152
chr10.fa	124739556	124749907	PSTK	2.972256802	228
chr10.fa	124753197	124768311	IKZF5	16.31038429	1216
chr10.fa	124768429	124817806	ACADSB	19.91445416	5320
chr10.fa	124895567	124897247	HMX3	0	0
chr10.fa	124907638	124910188	HMX2	1.038121568	76
chr10.fa	124913760	124924886	BUB3	22.01382596	2736
chr10.fa	125425871	125456913	GPR26	0	0
chr10.fa	125505152	125651500	CPXM2	0	0
chr10.fa	125767182	125851930	CHST15	0	0
chr10.fa	126085872	126107545	OAT	23.42978998	2204
chr10.fa	126135998	126138550	NKX1-2	0	0
chr10.fa	126150341	126302710	LHPP	7.02155361	532
chr10.fa	126307863	126432930	FAM53B	6.772475599	1748
chr10.fa	126447406	126480439	METTL10	2.621546066	304
chr10.fa	126490354	126525239	FAM175B	4.519209163	608
chr10.fa	126630692	126676005	ZRANB1	18.46802256	4104
chr10.fa	126676418	126849624	CTBP2	14.85861531	3648
chr10.fa	126721352	126721439	MIR4296	0	0
chr10.fa	127262940	127267014	LOC100169752	0	0
chr10.fa	127344263	127371713	C10orf122	0	0
chr10.fa	127371812	127398246	LOC283038	0	0
chr10.fa	127393859	127408062	FLJ37035	0.965844466	76
chr10.fa	127408084	127452712	C10orf137	11.81163511	2432
chr10.fa	127455027	127464390	MMP21	0	0
chr10.fa	127477147	127511837	UROS	23.71734165	1444
chr10.fa	127512104	127542264	BCCIP	27.16351384	2052
chr10.fa	127525125	127569884	DHX32	24.87288571	3192
chr10.fa	127585108	127698161	FANK1	2.608202601	152
chr10.fa	127702902	128077127	ADAM12	0	0
chr10.fa	128113574	128210010	C10orf90	0	0
chr10.fa	128594023	129250780	DOCK1	27.53957716	8360
chr10.fa	128933690	128994422	FAM196A	0	0

chr10.fa	129347613	129350935	NPS	0	0
chr10.fa	129535538	129539450	FOXI2	0	0
chr10.fa	129676114	129691211	CLRN3	8.872292188	456
chr10.fa	129705325	129884164	PTPRE	1.533386506	380
chr10.fa	129894925	129924468	MKI67	0.135213777	76
chr10.fa	131265454	131565783	MGMT	12.02490816	684
chr10.fa	131633547	131762091	EBF3	0	0
chr10.fa	131641563	131641638	MIR4297	0	0
chr10.fa	131862162	131909081	LOC387723	1.265627644	152
chr10.fa	131934639	131978646	GLRX3	17.90248204	1288
chr10.fa	132760851	132760931	MIR378C	0	0
chr10.fa	132890655	133109984	TCERG1L	1.9374711	228
chr10.fa	133747960	133770053	PPP2R2D	31.8575224	2812
chr10.fa	133781204	133795435	BNIP3	53.98854846	3724
chr10.fa	133918313	133998313	JAKMIP3	0.521062303	152
chr10.fa	134000414	134019280	DPYSL4	1.864749217	228
chr10.fa	134020996	134121477	STK32C	2.392928035	228
chr10.fa	134145614	134195010	LRRC27	1.974165629	836
chr10.fa	134210702	134231358	PWWP2B	14.25460114	1480
chr10.fa	134258714	134261825	C10orf91	0	0
chr10.fa	134351353	134596984	INPP5A	25.34257567	3348
chr10.fa	134598320	134599537	NKX6-2	0	0
chr10.fa	134742689	134756089	C10orf93	0	0
chr10.fa	134901409	134945179	GPR123	0	0
chr10.fa	134973971	135039916	KNDC1	0.249522793	76
chr10.fa	135043778	135045062	UTF1	0	0
chr10.fa	135051408	135055433	VENTX	0	0
chr10.fa	135061015	135061124	MIR202	0	0
chr10.fa	135075920	135090407	ADAM8	2.549936138	377
chr10.fa	135093138	135122644	TUBGCP2	20.70750075	2812
chr10.fa	135122894	135126666	ZNF511	16.86413809	684
chr10.fa	135138928	135150475	CALY	0	0
chr10.fa	135160844	135166187	PRAP1	4.210530342	142
chr10.fa	135168658	135171529	C10orf125	22.00737662	760
chr10.fa	135175987	135186908	ECHS1	174.114203	10405
chr10.fa	135185060	135185167	MIR3944	0	0
chr10.fa	135192741	135205198	PAOX	2.73340878	228
chr10.fa	135207621	135234169	MTG1	14.70627742	1064
chr10.fa	135234175	135238121	SPRN	2.66758102	380
chr10.fa	135267432	135281953	LOC619207	0.344706176	76
chr10.fa	135340867	135352620	CYP2E1	1.01388094	76
chr10.fa	135367404	135379138	SYCE1	0.972516199	76
chr10.fa	135380223	135383462	SPRNP1	0	0

chr10.fa	135438603	135440299	FRG2B	0	0
chr10.fa	135480368	135480370	DUX4L2	0	0
chr10.fa	135480432	135498492	DUX2	0	0
chr11.fa	126987	131920	LOC100133161	0	0
chr11.fa	194501	194573	SCGB1C1	0	0
chr11.fa	196761	200258	ODF3	0	0
chr11.fa	202924	207422	BET1L	30.62125038	3876
chr11.fa	208530	215029	RIC8A	32.15241297	3785
chr11.fa	215111	236362	SIRT3	9.586167559	1216
chr11.fa	236808	252984	PSMD13	57.84347546	4791
chr11.fa	278570	285304	NLRP6	0	0
chr11.fa	289138	295688	ATHL1	1.551622574	228
chr11.fa	298203	299526	IFITM5	0	0
chr11.fa	308107	309410	IFITM2	37.04479437	1216
chr11.fa	313991	315272	IFITM1	7.071813994	228
chr11.fa	319673	320914	IFITM3	60.72477431	1824
chr11.fa	369795	382117	B4GALNT4	0.489260379	76
chr11.fa	394217	404908	PKP3	18.65260716	2356
chr11.fa	405716	417397	SIGIRR	8.235808914	684
chr11.fa	417930	442011	ANO9	0.587779628	76
chr11.fa	450280	491387	PTDSS2	4.147593666	456
chr11.fa	494512	507273	RNH1	64.56324438	6384
chr11.fa	532242	535550	HRAS	27.01873725	1368
chr11.fa	537522	554849	LRRC56	1.873200078	228
chr11.fa	554917	560779	C11orf35	2.496562279	228
chr11.fa	560971	564025	RASSF7	20.07079509	1444
chr11.fa	568089	568198	MIR210	0	0
chr11.fa	573808	575885	LOC143666	0	0
chr11.fa	576483	612222	PHRF1	16.8314466	4180
chr11.fa	612555	615999	IRF7	4.285476137	380
chr11.fa	616565	625067	CDHR5	1.937693491	304
chr11.fa	626313	627173	SCT	0	0
chr11.fa	637305	640705	DRD4	0	0
chr11.fa	644225	695615	DEAF1	15.00339191	1748
chr11.fa	695741	704129	TMEM80	5.733242076	380
chr11.fa	706120	727727	EPS8L2	31.74788359	4484
chr11.fa	747432	765024	TALDO1	161.8106388	9044
chr11.fa	767223	777487	PDDC1	12.39919235	2432
chr11.fa	787110	790126	CEND1	0	0
chr11.fa	790475	798269	SLC25A22	5.75058858	836
chr11.fa	799179	805250	LRDD	0.561982262	76
chr11.fa	809936	812876	RPLP2	1157.020068	25649
chr11.fa	811681	811814	SNORA52	0	0

chr11.fa	818901	825571	PNPLA2	18.10641467	1976
chr11.fa	827585	831991	EFCAB4A	10.21531193	836
chr11.fa	832952	838835	CD151	5.469708645	380
chr11.fa	839721	842529	POLR2L	150.4947134	5928
chr11.fa	842824	867116	TSPAN4	0.957615996	76
chr11.fa	867859	915058	CHID1	3.012287196	517
chr11.fa	925841	1012240	AP2A2	24.16145664	5015
chr11.fa	1012824	1036706	MUC6	0	0
chr11.fa	1074875	1104417	MUC2	0	0
chr11.fa	1244295	1283406	MUC5B	0	0
chr11.fa	1295598	1330892	TOLLIP	48.95027851	8056
chr11.fa	1330938	1331937	LOC255512	3.380344436	152
chr11.fa	1411129	1483328	BRSK2	0.965177293	152
chr11.fa	1490685	1785501	MOB2	14.70561025	1140
chr11.fa	1575281	1593150	DUSP8	0.377842447	76
chr11.fa	1593971	1620414	LOC338651	0	0
chr11.fa	1605572	1606513	KRTAP5-1	0	0
chr11.fa	1618407	1618477	KRTAP5-2	0	0
chr11.fa	1628795	1629693	KRTAP5-3	0	0
chr11.fa	1642188	1643368	KRTAP5-4	0	0
chr11.fa	1651033	1652159	KRTAP5-5	0	0
chr11.fa	1686829	1689086	FAM99A	0	0
chr11.fa	1704500	1706859	FAM99B	0	0
chr11.fa	1718425	1718985	KRTAP5-6	0	0
chr11.fa	1756030	1769349	LOC402778	1.560740609	76
chr11.fa	1773985	1785222	CTSD	26.3093097	2508
chr11.fa	1855674	1858750	SYT8	0	0
chr11.fa	1860233	1862910	TNNI2	0	0
chr11.fa	1874200	1913493	LSP1	0	0
chr11.fa	1880694	1880766	MIR4298	0	0
chr11.fa	1940799	1959936	TNNT3	0	0
chr11.fa	1968502	1977839	MRPL23	43.27930594	1444
chr11.fa	2004439	2011150	LOC100133545	0	0
chr11.fa	2016406	2019065	H19	0	0
chr11.fa	2150342	2156763	INS-IGF2	0	0
chr11.fa	2155364	2155439	MIR483	0	0
chr11.fa	2159459	2162341	IGF2	1.572304945	76
chr11.fa	2161758	2169896	IGF2AS	0	0
chr11.fa	2181009	2182397	INS	0	0
chr11.fa	2185159	2193035	TH	0	0
chr11.fa	2289728	2292182	ASCL2	2.742304424	228
chr11.fa	2317507	2323143	C11orf21	0	0
chr11.fa	2323243	2339430	TSPAN32	0	0

chr11.fa	2398547	2418649	CD81	14.67758897	988
chr11.fa	2423523	2425106	TSSC4	13.0561356	836
chr11.fa	2425746	2444275	TRPM5	0.430326742	76
chr11.fa	2466221	2870340	KCNQ1	1.497581541	228
chr11.fa	2661768	2721228	KCNQ1OT1	0.028466058	76
chr11.fa	2891263	2893336	KCNQ1DN	0	0
chr11.fa	2904448	2906995	CDKN1C	14.88752616	1292
chr11.fa	2909327	2925175	SLC22A18AS	1.168887523	76
chr11.fa	2921021	2946476	SLC22A18	7.763227866	532
chr11.fa	2949503	2950650	PHLDA2	29.39431878	1216
chr11.fa	2965660	3013607	NAP1L4	62.89308736	7121
chr11.fa	2985001	2985123	SNORA54	0	0
chr11.fa	3022152	3078681	CARS	15.19931845	1900
chr11.fa	3108346	3186582	OSBPL5	5.652736504	988
chr11.fa	3239174	3240043	MRGPRG	0	0
chr11.fa	3242800	3244361	C11orf36	0	0
chr11.fa	3249041	3253616	MRGPRE	0	0
chr11.fa	3380011	3400448	ZNF195	5.622268926	608
chr11.fa	3402191	3430378	LOC650368	0	0
chr11.fa	3647714	3658789	TRPC2	0	0
chr11.fa	3659736	3663546	ART5	0	0
chr11.fa	3666361	3685646	ART1	0	0
chr11.fa	3686817	3692614	CHRNA10	0	0
chr11.fa	3696240	3819022	NUP98	28.17316935	9804
chr11.fa	3819049	3847582	PGAP2	6.631034871	831
chr11.fa	3848208	3862213	RHOG	16.65820394	988
chr11.fa	3876933	4114440	STIM1	4.604162556	836
chr11.fa	4115924	4160106	RRM1	13.24160976	1900
chr11.fa	4388493	4389616	OR52B4	0	0
chr11.fa	4406127	4414926	TRIM21	11.3904264	988
chr11.fa	4470525	4471591	OR52K2	0	0
chr11.fa	4510109	4511138	OR52K1	0	0
chr11.fa	4566421	4567374	OR52M1	0	0
chr11.fa	4592653	4599050	C11orf40	0	0
chr11.fa	4608021	4609135	OR52I2	0	0
chr11.fa	4615269	4616243	OR52I1	0	0
chr11.fa	4619902	4629437	TRIM68	16.35575207	2427
chr11.fa	4660945	4662068	OR51D1	0	0
chr11.fa	4665156	4676716	OR51E1	1.633462492	228
chr11.fa	4701401	4719076	OR51E2	1.820715783	228
chr11.fa	4790209	4791147	OR51F1	0	0
chr11.fa	4824663	4825610	OR52R1	0	0
chr11.fa	4842616	4843644	OR51F2	0	0

chr11.fa	4869467	4870438	OR51S1	0	0
chr11.fa	4903049	4904113	OR51T1	0	0
chr11.fa	4928600	4929538	OR51A7	0	0
chr11.fa	4935949	4936893	OR51G2	0	0
chr11.fa	4944604	4945569	OR51G1	0	0
chr11.fa	4967389	4968330	OR51A4	0	0
chr11.fa	4976002	4976943	OR51A2	0	0
chr11.fa	5009424	5013659	MMP26	0	0
chr11.fa	5020213	5021160	OR51L1	0	0
chr11.fa	5067756	5068691	OR52J3	0	0
chr11.fa	5079880	5080857	OR52E2	0	0
chr11.fa	5141850	5142847	OR52A4	0	0
chr11.fa	5152922	5153872	OR52A5	0	0
chr11.fa	5172661	5173599	OR52A1	0	0
chr11.fa	5220965	5221930	OR51V1	0	0
chr11.fa	5246696	5248301	HBB	0	0
chr11.fa	5254059	5255858	HBD	0	0
chr11.fa	5263185	5264822	HBBP1	0	0
chr11.fa	5269502	5271087	HBG1	0	0
chr11.fa	5274421	5276011	HBG2	0	0
chr11.fa	5289580	5291373	HBE1	0	0
chr11.fa	5322244	5323176	OR51B4	0	0
chr11.fa	5344528	5345582	OR51B2	0	0
chr11.fa	5363816	5364754	OR51B5	0	0
chr11.fa	5372738	5373676	OR51B6	0	0
chr11.fa	5410607	5411664	OR51M1	0	0
chr11.fa	5443341	5444436	OR51Q1	0	0
chr11.fa	5461772	5462783	OR51I1	0	0
chr11.fa	5474638	5475707	OR51I2	0	0
chr11.fa	5509915	5510978	OR52D1	0	0
chr11.fa	5528530	5531153	UBQLN3	0	0
chr11.fa	5535623	5537956	UBQLNL	0	0
chr11.fa	5565791	5566753	OR52H1	0	0
chr11.fa	5602107	5603114	OR52B6	0	0
chr11.fa	5617331	5634188	TRIM6	11.71400543	1192
chr11.fa	5641174	5646290	TRIM34	0	0
chr11.fa	5684425	5706339	TRIM5	7.117626557	988
chr11.fa	5686626	5687610	TRIM78P	0	0
chr11.fa	5710817	5732093	TRIM22	21.19587157	2946
chr11.fa	5757678	5758768	OR56B1	0	0
chr11.fa	5775923	5776959	OR52N4	0	0
chr11.fa	5798864	5799897	OR52N5	0	0
chr11.fa	5809084	5810046	OR52N1	0	0

chr11.fa	5841566	5842531	OR52N2	0	0
chr11.fa	5862186	5863127	OR52E6	0	0
chr11.fa	5877979	5878932	OR52E8	0	0
chr11.fa	5905523	5906461	OR52E4	0	0
chr11.fa	5968577	5969524	OR56A3	0	0
chr11.fa	5988783	5989724	OR56A5	0	0
chr11.fa	6007122	6008215	OR52L1	0	0
chr11.fa	6023281	6024378	OR56A4	0	0
chr11.fa	6047901	6048971	OR56A1	0	0
chr11.fa	6129009	6129968	OR56B4	0	0
chr11.fa	6190585	6191556	OR52B2	0	0
chr11.fa	6220454	6221416	OR52W1	0	0
chr11.fa	6226798	6232365	C11orf42	0	0
chr11.fa	6232564	6255941	FAM160A2	3.872051117	608
chr11.fa	6260330	6265707	CNGA4	0	0
chr11.fa	6280966	6293356	CCKBR	0	0
chr11.fa	6340176	6341740	PRKCDBP	1.629904235	76
chr11.fa	6411644	6416228	SMPD1	3.418595702	380
chr11.fa	6416355	6440644	APBB1	6.239181786	760
chr11.fa	6452268	6462254	HPX	0	0
chr11.fa	6469843	6495205	TRIM3	3.339202086	456
chr11.fa	6496926	6502595	ARFIP2	32.10015107	3724
chr11.fa	6502677	6505911	FXC1	25.57252805	3268
chr11.fa	6518526	6593254	DNHD1	0.454789761	304
chr11.fa	6621152	6624811	RRP8	11.1062106	836
chr11.fa	6624964	6632072	ILK	34.26601781	3040
chr11.fa	6632100	6633445	TAF10	110.3786982	3648
chr11.fa	6633997	6640692	TPP1	0.956726432	152
chr11.fa	6642558	6677074	DCHS1	0	0
chr11.fa	6701616	6704632	MRPL17	12.85842993	1368
chr11.fa	6734381	6743110	GVINP1	0.193702632	76
chr11.fa	6789238	6790188	OR2AG2	0	0
chr11.fa	6806248	6807245	OR2AG1	0	0
chr11.fa	6815756	6817139	OR6A2	0	0
chr11.fa	6866914	6867867	OR10A5	0	0
chr11.fa	6890986	6891897	OR10A2	0	0
chr11.fa	6897856	6898850	OR10A4	0	0
chr11.fa	6912805	6913731	OR2D2	0	0
chr11.fa	6942233	6943225	OR2D3	0	0
chr11.fa	6947654	6979278	ZNF215	0.924034943	152
chr11.fa	7020549	7041586	ZNF214	1.26696199	152
chr11.fa	7041700	7092757	NLRP14	0	0
chr11.fa	7110165	7112379	RBMXL2	0	0

chr11.fa	7273181	7490276	SYT9	1.708185895	304
chr11.fa	7506600	7532606	OLFML1	0	0
chr11.fa	7535001	7674991	PPFIBP2	153.9938147	24658
chr11.fa	7686326	7694821	CYB5R2	7.523045498	456
chr11.fa	7712444	7727941	OVCH2	0.997201609	76
chr11.fa	7817521	7818489	OR5P2	0	0
chr11.fa	7846584	7847519	OR5P3	0	0
chr11.fa	7870598	7871118	OR5E1P	0	0
chr11.fa	7872298	7927502	LOC283299	0	0
chr11.fa	7949265	7950209	OR10A6	0	0
chr11.fa	7960123	7961067	OR10A3	0	0
chr11.fa	7981156	7985059	NLRP10	0	0
chr11.fa	8008867	8017719	EIF3F	83.36574313	4712
chr11.fa	8060180	8127596	TUB	2.841046064	836
chr11.fa	8127655	8190590	RIC3	0.294001009	76
chr11.fa	8245857	8285406	LMO1	0	0
chr11.fa	8413418	8615503	STK33	3.746177764	456
chr11.fa	8633584	8680383	TRIM66	3.29272235	1444
chr11.fa	8703995	8711419	RPL27A	111.3049571	24419
chr11.fa	8705774	8705903	SNORA3	13.00142739	76
chr11.fa	8706986	8707116	SNORA45	0	0
chr11.fa	8714899	8932498	ST5	5.029151913	1064
chr11.fa	8932701	8941622	C11orf17	6.792490796	456
chr11.fa	8941627	8954553	C11orf16	0	0
chr11.fa	8959119	8964580	ASCL3	0	0
chr11.fa	8968840	8985989	TMEM9B	8.677477601	684
chr11.fa	9002123	9025596	NRIP3	0	0
chr11.fa	9041047	9113150	SCUBE2	23.33994398	4940
chr11.fa	9115910	9117737	FLJ46111	0	0
chr11.fa	9160375	9286871	DENND5A	15.32808289	3420
chr11.fa	9302201	9336296	TMEM41B	1.691951347	304
chr11.fa	9406169	9469674	IPO7	30.0147899	8357
chr11.fa	9450313	9450501	SNORA23	8.942790161	76
chr11.fa	9481103	9482245	LOC644656	0	0
chr11.fa	9482512	9550071	ZNF143	9.176300797	1216
chr11.fa	9595228	9611313	WEE1	34.66387545	6308
chr11.fa	9685628	9774507	SWAP70	23.71200426	5168
chr11.fa	9776317	9781080	LOC440028	0	0
chr11.fa	9779840	9832866	LOC283104	2.095146377	228
chr11.fa	9800214	10315754	SBF2	13.94503275	4484
chr11.fa	10326642	10328923	ADM	6.998647328	456
chr11.fa	10471868	10529126	AMPD3	5.973424444	1520
chr11.fa	10529434	10530723	MTRNR2L8	0	0

chr11.fa	10533225	10562774	RNF141	46.35141634	8512
chr11.fa	10562787	10621479	LOC100129827	2.784558729	76
chr11.fa	10579413	10590365	LYVE1	0	0
chr11.fa	10594638	10715535	MRVI1	0	0
chr11.fa	10772811	10801290	CTR9	22.75016283	4408
chr11.fa	10818593	10830582	EIF4G2	248.885198	48783
chr11.fa	10823014	10823155	SNORD97	0	0
chr11.fa	10874251	10879620	ZBED5	8.632776994	1064
chr11.fa	10879764	10900823	LOC729013	27.33431019	1368
chr11.fa	11292421	11643561	GALNTL4	2.016197543	228
chr11.fa	11373321	11374904	CSNK2A1P	0	0
chr11.fa	11678198	11678269	MIR4299	0	0
chr11.fa	11862970	11980872	USP47	32.83960141	11484
chr11.fa	11984543	12030917	DKK3	0	0
chr11.fa	12132138	12285331	MICAL2	11.2534335	1976
chr11.fa	12308447	12380691	MICALCL	4.442039458	608
chr11.fa	12399026	12556903	PARVA	17.97698306	7048
chr11.fa	12695969	12966284	TEAD1	20.82203216	8816
chr11.fa	13030970	13033653	RASSF10	1.259400693	152
chr11.fa	13299325	13408812	ARNTL	1.179562295	152
chr11.fa	13409556	13484838	BTBD10	16.18028551	1824
chr11.fa	13513601	13517567	PTH	0	0
chr11.fa	13690206	13753893	FAR1	13.41084938	3181
chr11.fa	13983914	14289655	SPON1	0.628032414	152
chr11.fa	14299466	14386052	RRAS2	10.44081648	1292
chr11.fa	14479049	14521441	COPB1	37.37838099	5928
chr11.fa	14526422	14665180	PSMA1	45.19186924	4180
chr11.fa	14665269	14893604	PDE3B	0.832632208	228
chr11.fa	14899556	14913751	CYP2R1	4.178506027	304
chr11.fa	14988215	14993832	CALCA	0	0
chr11.fa	15095146	15100177	CALCB	0	0
chr11.fa	15133970	15268754	INSC	0	0
chr11.fa	15987995	16497935	SOX6	0.902685399	380
chr11.fa	16760148	16779901	C11orf58	44.69615952	7999
chr11.fa	16809207	17035963	PLEKHA7	23.52030315	5085
chr11.fa	17095939	17099220	RPS13	959.5187742	22824
chr11.fa	17108124	17191354	PIK3C2A	14.00952617	5221
chr11.fa	17298286	17353070	NUCB2	2.096925506	152
chr11.fa	17373317	17398871	DKFZp686O24166	0.530180338	152
chr11.fa	17406796	17410878	KCNJ11	0.613354602	76
chr11.fa	17414432	17498449	ABCC8	0	0
chr11.fa	17515442	17565963	USH1C	3.563594687	532
chr11.fa	17741110	17743678	MYOD1	0	0

chr11.fa	17757495	17804602	KCNC1	0	0
chr11.fa	17809597	18034637	SERGEF	15.85959757	1064
chr11.fa	18042084	18062335	TPH1	0	0
chr11.fa	18101890	18127638	SAAL1	7.509924424	517
chr11.fa	18134019	18137679	SAA3P	0	0
chr11.fa	18142502	18160027	MRGPRX3	0	0
chr11.fa	18194384	18195827	MRGPRX4	0	0
chr11.fa	18230685	18235111	LOC494141	0	0
chr11.fa	18258264	18258384	SAA4	0	0
chr11.fa	18259780	18267062	SAA2	0	0
chr11.fa	18287808	18291523	SAA1	0	0
chr11.fa	18300217	18343721	HPS5	6.234289182	1368
chr11.fa	18343816	18388590	GTF2H1	18.75290554	2964
chr11.fa	18415936	18429765	LDHA	53.62471665	6156
chr11.fa	18433853	18472793	LDHC	0	0
chr11.fa	18477374	18501147	LDHAL6A	0	0
chr11.fa	18501858	18548489	TSG101	26.44341152	1824
chr11.fa	18553244	18610281	UEVLD	16.48785238	1571
chr11.fa	18627948	18656020	SPTY2D1	7.958709626	2052
chr11.fa	18686477	18688026	LOC100132715	0	0
chr11.fa	18720351	18726332	TMEM86A	2.524138773	380
chr11.fa	18727364	18747777	IGSF22	0	0
chr11.fa	18749475	18813389	PTPN5	0	0
chr11.fa	18955360	18956549	MRGPRX1	0	0
chr11.fa	19076003	19082228	MRGPRX2	0	0
chr11.fa	19138692	19197967	ZDHHC13	8.332771425	912
chr11.fa	19203578	19223589	CSRP3	0	0
chr11.fa	19245610	19262507	E2F8	0.908467567	152
chr11.fa	19372271	20143147	NAV2	9.303953277	4484
chr11.fa	19532440	19545625	FLJ13439	0	0
chr11.fa	19732480	19736146	LOC100126784	0	0
chr11.fa	20177760	20181870	DBX1	0	0
chr11.fa	20385231	20405329	HTATIP2	14.38114166	1444
chr11.fa	20409076	20530879	PRMT3	4.329064789	532
chr11.fa	20620946	20676610	SLC6A5	0	0
chr11.fa	20691117	21597229	NELL1	1.041012652	152
chr11.fa	22214722	22304913	ANO5	0.253748224	76
chr11.fa	22359667	22401046	SLC17A6	0	0
chr11.fa	22644079	22647387	FANCF	8.172427456	1216
chr11.fa	22688160	22834547	GAS2	1.331900186	152
chr11.fa	22843598	22851382	SVIP	28.83567238	1801
chr11.fa	22868468	22881972	LOC100500938	2.112715272	76
chr11.fa	24518556	25104186	LUZP2	0.642932616	152

chr11.fa	26353678	26684836	ANO3	0	0
chr11.fa	26580579	26593815	MUC15	2.029541008	304
chr11.fa	26688566	26743574	SLC5A12	4.324839358	1216
chr11.fa	27015628	27018632	FIBIN	1.124854089	152
chr11.fa	27062509	27149354	BBOX1	25.98884415	2204
chr11.fa	27360061	27384795	CCDC34	3.445727414	456
chr11.fa	27387508	27494334	LGR4	8.053003445	1900
chr11.fa	27515965	27528326	LIN7C	19.14520341	4177
chr11.fa	27528399	27719718	BDNF-AS1	1.220704645	152
chr11.fa	27676442	27743605	BDNF	0	0
chr11.fa	28042163	28129746	KIF18A	0	0
chr11.fa	28078362	28078457	MIR610	0	0
chr11.fa	28129798	28355054	METT5D1	1.148205153	228
chr11.fa	30031765	30038488	KCNA4	0	0
chr11.fa	30252563	30256824	FSHB	0	0
chr11.fa	30344649	30359165	C11orf46	8.594080946	684
chr11.fa	30406040	30607930	MPPED2	5.393206113	1748
chr11.fa	30886486	30953499	DCDC5	0	0
chr11.fa	31284171	31391321	DCDC1	0	0
chr11.fa	31391377	31454382	DNAJC24	3.579606845	456
chr11.fa	31455007	31531169	IMMP1L	2.691376866	76
chr11.fa	31531297	31805329	ELP4	6.301228898	444
chr11.fa	31806340	31839509	PAX6	0	0
chr11.fa	31838114	31908587	DKFZp686K1684	0	0
chr11.fa	32112477	32127272	RCN1	1.343464522	152
chr11.fa	32409322	32457081	WT1	0	0
chr11.fa	32457285	32461620	WT1-AS	0	0
chr11.fa	32605391	32623368	EIF3M	77.10409984	3630
chr11.fa	32623626	32816187	CCDC73	0	0
chr11.fa	32851489	32876105	PRRG4	2.57906937	228
chr11.fa	32914792	33001814	QSER1	4.376211698	1824
chr11.fa	33037410	33055128	DEPDC7	0.782816606	76
chr11.fa	33060963	33095109	TCP11L1	3.370336837	456
chr11.fa	33097696	33101000	NCRNA00294	3.068329749	456
chr11.fa	33106130	33183037	CSTF3	8.501343865	1216
chr11.fa	33183203	33213142	LOC338739	0	0
chr11.fa	33279168	33375939	HIPK3	58.44148508	12464
chr11.fa	33563877	33695646	C11orf41	0.290887534	152
chr11.fa	33719654	33722286	C11orf91	0	0
chr11.fa	33724556	33758025	CD59	6.783595153	2432
chr11.fa	33762490	33796071	FBXO3	13.91834582	1748
chr11.fa	33880123	33913836	LMO2	0.699642342	76
chr11.fa	34073230	34124157	CAPRIN1	50.29996999	12580

chr11.fa	34127111	34168458	NAT10	11.29301911	2052
chr11.fa	34172534	34379555	ABTB2	4.136696503	912
chr11.fa	34460472	34493607	CAT	81.56926797	8436
chr11.fa	34500342	34535330	ELF5	17.83999015	2052
chr11.fa	34642588	34684834	EHF	148.4960848	36391
chr11.fa	34903843	34916657	APIP	24.09073627	1184
chr11.fa	34937677	35017675	PDHX	11.3755262	1444
chr11.fa	35160417	35253949	CD44	2.358902199	608
chr11.fa	35272752	35441532	SLC1A2	0	0
chr11.fa	35453376	35547176	PAMR1	0	0
chr11.fa	35639735	35642421	FJX1	0.628921978	76
chr11.fa	35684353	35830930	TRIM44	24.94138216	4864
chr11.fa	35965612	36252841	LDLRAD3	2.84638345	380
chr11.fa	36293842	36310999	COMMD9	8.524694928	1140
chr11.fa	36317725	36486754	PRR5L	3.208658521	608
chr11.fa	36510723	36531822	TRAF6	10.50619946	1216
chr11.fa	36589563	36601310	RAG1	0.513723398	152
chr11.fa	36613493	36619829	RAG2	0	0
chr11.fa	36616093	36680817	C11orf74	4.122463474	152
chr11.fa	40135753	40315664	LRRC4C	0.416983277	76
chr11.fa	43283054	43290919	LOC399881	0.898015186	76
chr11.fa	43333505	43366080	API5	25.15398803	4332
chr11.fa	43380435	43516483	TTC17	2.988046568	608
chr11.fa	43581206	43581303	MIR670	0	0
chr11.fa	43602944	43603033	MIR129-2	0	0
chr11.fa	43702143	43878169	HSD17B12	5.732352512	656
chr11.fa	43902357	43941825	ALKBH3	11.84944159	836
chr11.fa	43918853	43921424	LOC729799	0	0
chr11.fa	43964106	43965433	C11orf96	0	0
chr11.fa	44069531	44081527	ACCSL	0	0
chr11.fa	44087729	44105569	ACCS	3.696584553	375
chr11.fa	44117099	44266980	EXT2	3.510220827	608
chr11.fa	44282278	44331716	ALX4	0	0
chr11.fa	44587141	44641315	CD82	1.99907343	152
chr11.fa	44785976	44953898	TSPAN18	2.03398883	380
chr11.fa	44953978	44972608	TP53I11	7.98206069	1281
chr11.fa	44995453	44999578	LOC221122	0	0
chr11.fa	45115564	45246903	PRDM11	0	0
chr11.fa	45261853	45307884	SYT13	1.310995424	304
chr11.fa	45670427	45687172	CHST1	0	0
chr11.fa	45792983	45793909	DKFZp779M0652	0	0
chr11.fa	45825623	45834567	SLC35C1	8.16308703	1444
chr11.fa	45868669	45904799	CRY2	17.41255449	3420

chr11.fa	45907202	45928016	MAPK8IP1	4.433143815	608
chr11.fa	45928086	45928833	C11orf94	0	0
chr11.fa	45931220	45939674	PEX16	17.15902866	1368
chr11.fa	45943196	45950647	GYLTL1B	1.337237572	152
chr11.fa	45950870	46142985	PHF21A	7.309105278	2412
chr11.fa	46299228	46342972	CREB3L1	12.65583165	1520
chr11.fa	46354455	46402104	DGKZ	2.785448293	679
chr11.fa	46402618	46405375	MDK	9.843029258	532
chr11.fa	46406640	46408107	CHRM4	0	0
chr11.fa	46417964	46612914	AMBRA1	9.758075865	2204
chr11.fa	46473355	46473439	MIR3160-1	0	0
chr11.fa	46624856	46638777	HARBI1	2.221019729	152
chr11.fa	46638826	46696368	ATG13	21.65510915	4746
chr11.fa	46698632	46722120	ARHGAP1	33.35866219	5016
chr11.fa	46722317	46727466	ZNF408	3.328527314	380
chr11.fa	46740743	46761056	F2	0	0
chr11.fa	46765084	46867843	CKAP5	18.16846178	5472
chr11.fa	46783939	46784049	SNORD67	0	0
chr11.fa	46878268	46940173	LRP4	0.821735045	304
chr11.fa	46958251	47185851	C11orf49	8.725736466	760
chr11.fa	47185932	47198419	ARFGAP2	30.19114602	3641
chr11.fa	47199073	47208010	PACSIN3	12.67228859	1216
chr11.fa	47236493	47260769	DDB2	5.487499931	456
chr11.fa	47260853	47270448	ACP2	2.935562273	304
chr11.fa	47270458	47290401	NR1H3	4.663763366	380
chr11.fa	47290927	47351582	MADD	24.5264004	6916
chr11.fa	47352957	47374253	MYBPC3	0	0
chr11.fa	47376409	47400127	SPI1	1.200467057	76
chr11.fa	47430046	47438051	SLC39A13	4.828999939	532
chr11.fa	47440320	47448024	PSMC3	53.40210318	3794
chr11.fa	47459315	47470730	RAPSN	0	0
chr11.fa	47487489	47574792	CELF1	20.7312966	5472
chr11.fa	47586982	47593748	PTPMT1	21.03441564	1292
chr11.fa	47595014	47600561	KBTBD4	6.837191403	380
chr11.fa	47600568	47606115	NDUFS3	44.33810988	1900
chr11.fa	47608230	47610746	FAM180B	0	0
chr11.fa	47611216	47615961	C1QTNF4	0	0
chr11.fa	47638858	47664206	MTCH2	37.79291796	4466
chr11.fa	47681143	47736302	AGBL2	0.996756826	152
chr11.fa	47738069	47788993	FNBP4	14.38625666	2660
chr11.fa	47799670	47870057	NUP160	9.41959664	2280
chr11.fa	48002110	48192394	PTPRJ	3.272039979	1332
chr11.fa	48238362	48239291	OR4B1	0	0

chr11.fa	48266656	48267567	OR4X2	0	0
chr11.fa	48285413	48286330	OR4X1	0	0
chr11.fa	48327775	48328704	OR4S1	0	0
chr11.fa	48346493	48347482	OR4C3	0	0
chr11.fa	48366900	48373999	OR4C45	0	0
chr11.fa	48510345	48511274	OR4A47	0	0
chr11.fa	49168187	49230222	FOLH1	17.9409557	2204
chr11.fa	49580080	49831969	LOC440040	0	0
chr11.fa	49973943	49974971	OR4C13	0	0
chr11.fa	50003009	50004071	OR4C12	0	0
chr11.fa	50238999	50257633	LOC441601	0	0
chr11.fa	50368318	50379802	LOC646813	0	0
chr11.fa	51411378	51412448	OR4A5	0	0
chr11.fa	51515282	51516211	OR4C46	0	0
chr11.fa	55029658	55038595	TRIM48	0	0
chr11.fa	55110677	55111663	OR4A16	0	0
chr11.fa	55135360	55136394	OR4A15	0	0
chr11.fa	55321783	55322895	OR4C15	0	0
chr11.fa	55339604	55340536	OR4C16	0	0
chr11.fa	55370917	55371849	OR4C11	0	0
chr11.fa	55405834	55406772	OR4P4	0	0
chr11.fa	55418380	55419315	OR4S2	0	0
chr11.fa	55432643	55433572	OR4C6	0	0
chr11.fa	55540914	55541858	OR5D13	0	0
chr11.fa	55563032	55563976	OR5D14	0	0
chr11.fa	55578943	55579878	OR5L1	0	0
chr11.fa	55587106	55588047	OR5D18	0	0
chr11.fa	55594695	55595630	OR5L2	0	0
chr11.fa	55606228	55607214	OR5D16	0	0
chr11.fa	55650773	55659284	SPRYD5	0	0
chr11.fa	55681126	55682058	OR5W2	0	0
chr11.fa	55702932	55703876	OR5I1	0	0
chr11.fa	55735034	55735939	OR10AG1	0	0
chr11.fa	55746179	55753881	OR7E5P	0	0
chr11.fa	55761157	55762101	OR5F1	0	0
chr11.fa	55797895	55798869	OR5AS1	0	0
chr11.fa	55860784	55861716	OR8I2	0	0
chr11.fa	55872519	55873457	OR8H2	0	0
chr11.fa	55889849	55890787	OR8H3	0	0
chr11.fa	55904247	55905194	OR8J3	0	0
chr11.fa	55926870	55927793	OR8K5	0	0
chr11.fa	55944094	55945032	OR5J2	0	0
chr11.fa	55999582	56000661	OR5T2	0	0

chr11.fa	56019676	56020698	OR5T3	0	0
chr11.fa	56043115	56044095	OR5T1	0	0
chr11.fa	56057603	56058538	OR8H1	0	0
chr11.fa	56085783	56086721	OR8K3	0	0
chr11.fa	56113515	56114474	OR8K1	0	0
chr11.fa	56127723	56128673	OR8J1	0	0
chr11.fa	56143898	56144029	OR8U1	0	0
chr11.fa	56184734	56185708	OR5R1	0	0
chr11.fa	56229945	56230877	OR5M9	0	0
chr11.fa	56237050	56237973	OR5M3	0	0
chr11.fa	56257911	56258846	OR5M8	0	0
chr11.fa	56309816	56310733	OR5M11	0	0
chr11.fa	56344250	56345197	OR5M10	0	0
chr11.fa	56380031	56380978	OR5M1	0	0
chr11.fa	56408965	56409915	OR5AP2	0	0
chr11.fa	56431162	56432094	OR5AR1	0	0
chr11.fa	56508508	56508560	OR8U8	0	0
chr11.fa	56510304	56511287	OR9G4	0	0
chr11.fa	56756389	56757318	OR5AK2	0	0
chr11.fa	56805009	56805935	OR5AK4P	0	0
chr11.fa	56949221	56959188	LRRC55	0	0
chr11.fa	57001052	57004927	APLNR	0	0
chr11.fa	57067103	57092413	TNKS1BP1	28.46005385	7448
chr11.fa	57093459	57103351	SSRP1	44.65968738	5661
chr11.fa	57105949	57137549	P2RX3	0	0
chr11.fa	57144242	57148623	PRG3	0	0
chr11.fa	57154834	57158130	PRG2	0	0
chr11.fa	57174427	57195053	SLC43A3	0	0
chr11.fa	57228339	57244384	RTN4RL2	0	0
chr11.fa	57252004	57283192	SLC43A1	1.921458942	228
chr11.fa	57295936	57298232	TIMM10	42.63036877	1292
chr11.fa	57310114	57317747	SMTNL1	0	0
chr11.fa	57319129	57335453	UBE2L6	64.35286241	4028
chr11.fa	57365027	57382326	SERPING1	0.852425015	76
chr11.fa	57408671	57408759	MIR130A	0	0
chr11.fa	57412560	57417417	YPEL4	0	0
chr11.fa	57425216	57429337	CLP1	3.065661056	228
chr11.fa	57435474	57468659	ZDHHC5	12.6022354	2584
chr11.fa	57471187	57479673	MED19	6.698196978	456
chr11.fa	57505308	57508445	TMX2	10.51976532	684
chr11.fa	57508722	57510883	C11orf31	65.30803211	3800
chr11.fa	57510986	57519253	BTBD18	0	0
chr11.fa	57529234	57583473	CTNND1	85.19023956	1425

chr11.fa	57791353	57949038	OR9Q1	0	0
chr11.fa	57798425	57799378	OR6Q1	0	0
chr11.fa	57885972	57886916	OR9I1	0	0
chr11.fa	57957906	57958990	OR9Q2	0	0
chr11.fa	57970676	57971653	OR1S2	0	0
chr11.fa	57982217	57983194	OR1S1	0	0
chr11.fa	57995354	57996390	OR10Q1	0	0
chr11.fa	58034264	58035732	OR10W1	0	0
chr11.fa	58125598	58126542	OR5B17	0	0
chr11.fa	58169938	58170882	OR5B3	0	0
chr11.fa	58189738	58190786	OR5B2	0	0
chr11.fa	58206593	58207646	OR5B12	0	0
chr11.fa	58274649	58275578	OR5B21	0	0
chr11.fa	58294344	58345639	LPXN	4.148705622	380
chr11.fa	58385042	58389023	ZFP91	17.40254689	3116
chr11.fa	58390146	58390238	CNTF	0	0
chr11.fa	58476230	58499447	GLYAT	16.38288379	1824
chr11.fa	58601540	58611997	GLYATL2	0	0
chr11.fa	58701116	58825925	LOC283194	0.649381957	76
chr11.fa	58710722	58724516	GLYATL1	21.32508079	1983
chr11.fa	58874658	58894888	FAM111B	0	0
chr11.fa	58910318	58922511	FAM111A	14.13362039	2490
chr11.fa	58939812	58975982	DTX4	38.12583741	9801
chr11.fa	58976061	58980494	MPEG1	0	0
chr11.fa	59131932	59132867	OR5AN1	0	0
chr11.fa	59189452	59190426	OR5A2	0	0
chr11.fa	59210642	59211589	OR5A1	0	0
chr11.fa	59224434	59225378	OR4D6	0	0
chr11.fa	59244903	59245838	OR4D10	0	0
chr11.fa	59271049	59271984	OR4D11	0	0
chr11.fa	59282386	59283330	OR4D9	0	0
chr11.fa	59341871	59383617	OSBP	70.66810195	16152
chr11.fa	59362550	59362631	MIR3162	0	0
chr11.fa	59404192	59436511	PATL1	16.38154944	3040
chr11.fa	59480389	59481318	OR10V1	0	0
chr11.fa	59522532	59573355	STX3	8.328990777	2425
chr11.fa	59573608	59578345	MRPL16	24.30867953	1292
chr11.fa	59596746	59612974	GIF	0	0
chr11.fa	59620281	59634041	TCN1	0	0
chr11.fa	59807748	59815516	PLAC1L	0	0
chr11.fa	59824101	59838588	MS4A3	0	0
chr11.fa	59856137	59865940	MS4A2	0	0
chr11.fa	59939080	59950674	MS4A6A	0	0

chr11.fa	60048139	60076445	MS4A4A	0	0
chr11.fa	60102355	60108441	MS4A6E	0	0
chr11.fa	60145958	60163426	MS4A7	0	0
chr11.fa	60163487	60185228	MS4A14	0	0
chr11.fa	60197062	60215265	MS4A5	0	0
chr11.fa	60223282	60238225	MS4A1	0	0
chr11.fa	60260251	60274901	MS4A12	0	0
chr11.fa	60282886	60310191	MS4A13	0	0
chr11.fa	60383224	60454621	C11orf64	0	0
chr11.fa	60467047	60483285	MS4A8B	0	0
chr11.fa	60524340	60544204	MS4A15	0	0
chr11.fa	60552821	60568778	MS4A10	0	0
chr11.fa	60609429	60618397	CCDC86	9.131155407	760
chr11.fa	60618562	60623444	GPR44	0	0
chr11.fa	60635015	60643164	ZP1	0	0
chr11.fa	60658020	60674061	PRPF19	50.32309866	5295
chr11.fa	60681371	60690915	TMEM109	4.954428509	532
chr11.fa	60691913	60704554	TMEM132A	0.981856624	152
chr11.fa	60704632	60719257	SLC15A3	0.82885156	76
chr11.fa	60739115	60787848	CD6	0	0
chr11.fa	60869930	60895323	CD5	0	0
chr11.fa	60897728	60928916	VPS37C	19.46344504	2356
chr11.fa	60970984	60980350	PGA3	0	0
chr11.fa	60989821	60999179	PGA4	0	0
chr11.fa	61008644	61018929	PGA5	0	0
chr11.fa	61025758	61062788	VWCE	0	0
chr11.fa	61066919	61100653	DDB1	69.17007563	13452
chr11.fa	61100667	61116219	DAK	12.02690968	2280
chr11.fa	61116232	61129472	CYBASC3	9.337979113	1292
chr11.fa	61129756	61136975	TMEM138	9.757186301	684
chr11.fa	61159832	61166335	TMEM216	10.2435556	608
chr11.fa	61170120	61197464	CPSF7	18.84453066	3192
chr11.fa	61197597	61214239	SDHAF2	20.79779153	1140
chr11.fa	61248585	61258400	C11orf66	1.098167159	76
chr11.fa	61276272	61278490	LRRC10B	0	0
chr11.fa	61282785	61348298	SYT7	17.52819785	3572
chr11.fa	61382508	61406921	RPLP0P2	0.479475171	76
chr11.fa	61447905	61514474	DAGLA	0.293556227	76
chr11.fa	61520121	61555989	C11orf9	0.282436673	76
chr11.fa	61521501	61525136	DKFZP434K028	0	0
chr11.fa	61556602	61560085	C11orf10	149.3040316	2719
chr11.fa	61559967	61560027	MIR611	0	0
chr11.fa	61560150	61564710	FEN1	2.254600782	228

chr11.fa	61567097	61584529	FADS1	4.206304912	836
chr11.fa	61582633	61582712	MIR1908	0	0
chr11.fa	61595713	61634825	FADS2	15.56515178	2204
chr11.fa	61640998	61659006	FADS3	0	0
chr11.fa	61664773	61684997	RAB3IL1	0.739895127	76
chr11.fa	61717356	61731756	BEST1	0	0
chr11.fa	61731936	61735132	FTH1	1630.613662	76988
chr11.fa	61891445	61920635	INCENP	2.853722355	532
chr11.fa	61957710	61961009	SCGB1D1	0	0
chr11.fa	61976140	61981411	SCGB2A1	0	0
chr11.fa	62009724	62012280	SCGB1D2	0	0
chr11.fa	62037630	62040628	SCGB2A2	0	0
chr11.fa	62063754	62066536	SCGB1D4	0	0
chr11.fa	62104774	62160887	ASRGL1	30.04436791	3268
chr11.fa	62186507	62190678	SCGB1A1	11.21785092	228
chr11.fa	62201016	62314332	AHNAK	30.44645099	26372
chr11.fa	62327073	62341460	EEF1G	506.6119982	35036
chr11.fa	62342517	62359109	TUT1	11.36351708	1444
chr11.fa	62360675	62369303	MTA2	39.19464895	5393
chr11.fa	62369691	62380212	EML3	5.232639752	760
chr11.fa	62380238	62382592	ROM1	0	0
chr11.fa	62382768	62389448	B3GAT3	3.516225386	228
chr11.fa	62392298	62414104	GANAB	12.92514725	2280
chr11.fa	62414320	62420774	INTS5	10.29025772	1520
chr11.fa	62430289	62439125	C11orf48	19.65314464	1064
chr11.fa	62432779	62434923	METTL12	0	0
chr11.fa	62432894	62433042	SNORA57	0	0
chr11.fa	62439242	62441162	C11orf83	4.607943205	380
chr11.fa	62443972	62446527	UBXN1	52.19029418	3025
chr11.fa	62453876	62457371	LRRN4CL	0	0
chr11.fa	62457747	62477046	BSCL2	11.93217108	1292
chr11.fa	62475201	62476618	GNG3	0	0
chr11.fa	62482220	62494821	HNRNPUL2	70.14103509	9424
chr11.fa	62495952	62506108	TTC9C	7.419633645	380
chr11.fa	62518435	62521656	ZBTB3	5.683204083	760
chr11.fa	62529011	62534187	POLR2G	65.01692218	2447
chr11.fa	62538875	62554813	TAF6L	6.710428487	608
chr11.fa	62554874	62557786	TMEM179B	12.36272021	532
chr11.fa	62557873	62559486	TMEM223	9.930428953	380
chr11.fa	62559598	62572964	NXF1	7.534609834	1405
chr11.fa	62574369	62599560	STX5	31.09005077	2508
chr11.fa	62600383	62607628	WDR74	18.54608183	1140
chr11.fa	62619460	62623360	SNHG1	0	0

chr11.fa	62620382	62620507	SNORD22	0	0
chr11.fa	62620798	62620865	SNORD31	0	0
chr11.fa	62621135	62621204	SNORD30	0	0
chr11.fa	62621376	62621440	SNORD29	0	0
chr11.fa	62622093	62622167	SNORD28	0	0
chr11.fa	62622484	62622555	SNORD27	0	0
chr11.fa	62622764	62622838	SNORD26	0	0
chr11.fa	62623037	62623103	SNORD25	0	0
chr11.fa	62623484	62656355	SLC3A2	8.031209119	1048
chr11.fa	62676151	62689012	CHRM1	1.796252764	228
chr11.fa	62744069	62752469	SLC22A6	3.848255271	380
chr11.fa	62760296	62783317	SLC22A8	5.306695982	456
chr11.fa	62847412	62911693	SLC22A24	0.805500496	76
chr11.fa	62931296	62997124	SLC22A25	0	0
chr11.fa	63057430	63079246	SLC22A10	0	0
chr11.fa	63137261	63177712	SLC22A9	0	0
chr11.fa	63228876	63258680	HRASLS5	0	0
chr11.fa	63273524	63284246	LGALS12	0	0
chr11.fa	63304273	63313930	RARRES3	37.55940733	1292
chr11.fa	63320242	63330855	HRASLS2	0	0
chr11.fa	63341944	63381941	PLA2G16	18.42154283	912
chr11.fa	63396437	63439083	ATL3	25.29498398	2566
chr11.fa	63448922	63527363	RTN3	2.717396622	608
chr11.fa	63527364	63536113	C11orf95	0.908467567	228
chr11.fa	63580923	63595190	C11orf84	5.171259813	456
chr11.fa	63606400	63678492	MARK2	38.55238351	8978
chr11.fa	63678693	63684316	RCOR2	0	0
chr11.fa	63706442	63724446	NAA40	7.092941147	1064
chr11.fa	63742079	63744015	COX8A	333.3673443	7600
chr11.fa	63753325	63765892	OTUB1	25.67015773	2888
chr11.fa	63766030	63933585	MACROD1	22.62829252	1279
chr11.fa	63871362	63886645	FLRT1	1.041012652	152
chr11.fa	63953587	63972020	STIP1	54.47113711	5391
chr11.fa	63974152	63991270	FERMT3	0	0
chr11.fa	63991364	63993726	TRPT1	13.99729466	608
chr11.fa	63993738	63997488	NUDT22	24.18858835	1216
chr11.fa	63997753	64001753	DNAJC4	38.14407348	2204
chr11.fa	64002266	64006259	VEGFB	64.92707619	3264
chr11.fa	64008413	64011607	FKBP2	21.6688974	912
chr11.fa	64011951	64014413	PPP1R14B	33.0319697	1466
chr11.fa	64018995	64036924	PLCB3	14.67603224	3780
chr11.fa	64037300	64052152	BAD	51.74239854	2550
chr11.fa	64052177	64056972	GPR137	4.659760326	532

chr11.fa	64058793	64067503	KCNK4	0	0
chr11.fa	64067863	64072239	C11orf20	0	0
chr11.fa	64073044	64084164	ESRRA	14.76476628	1444
chr11.fa	64084211	64085033	TRMT112	154.8008719	3724
chr11.fa	64085569	64089283	PRDX5	189.1329399	7518
chr11.fa	64107690	64125006	CCDC88B	3.778869253	836
chr11.fa	64126625	64139687	RPS6KA4	8.081691894	1140
chr11.fa	64136074	64136175	MIR1237	0	0
chr11.fa	64323098	64338999	SLC22A11	1.345688433	152
chr11.fa	64358282	64369820	SLC22A12	10.54378356	1520
chr11.fa	64373646	64490660	NRXN2	0.22839564	76
chr11.fa	64494383	64512928	RASGRP2	0	0
chr11.fa	64513861	64528187	PYGM	0	0
chr11.fa	64532076	64546316	SF1	48.09985502	8208
chr11.fa	64556609	64570713	MAP4K2	4.003706637	532
chr11.fa	64570986	64578766	MEN1	7.20658299	1140
chr11.fa	64591662	64612041	CDC42BPG	29.42990135	6536
chr11.fa	64620208	64646191	EHD1	12.71165182	1976
chr11.fa	64658609	64658718	MIR192	0	0
chr11.fa	64658827	64658911	MIR194-2	0	0
chr11.fa	64662004	64684722	ATG2A	12.46479772	3572
chr11.fa	64692143	64701942	PPP2R5B	7.234604266	892
chr11.fa	64701951	64703360	GPHA2	0	0
chr11.fa	64705929	64727609	C11orf85	0	0
chr11.fa	64755417	64764517	BATF2	0.78971073	76
chr11.fa	64787891	64789657	ARL2	34.90650412	857
chr11.fa	64794790	64794879	ARL2-SNX15	0	0
chr11.fa	64808376	64812294	SAC3D1	2.165421959	152
chr11.fa	64812301	64826009	NAALADL1	0	0
chr11.fa	64844927	64851615	CDCA5	0	0
chr11.fa	64851694	64855874	ZFPL1	19.51125913	1216
chr11.fa	64863683	64879180	C11orf2	51.17663563	5776
chr11.fa	64879341	64883707	TM7SF2	23.44491257	1672
chr11.fa	64883875	64885170	ZNHIT2	2.608202601	152
chr11.fa	64888099	64889654	FAU	473.3696465	11856
chr11.fa	64889673	64894750	MRPL49	46.1030055	4256
chr11.fa	64894842	64902003	SYVN1	2.855056702	380
chr11.fa	64937707	64940688	SPDYC	0	0
chr11.fa	64948686	64979477	CAPN1	126.1284346	19266
chr11.fa	64981311	65010228	SLC22A20	0.584443762	76
chr11.fa	65029432	65065088	POLA2	2.047109903	228
chr11.fa	65082331	65089900	CDC42EP2	8.531811443	760
chr11.fa	65101346	65120451	DPF2	14.73385392	1596

chr11.fa	65122282	65125082	TIGD3	0.85976392	76
chr11.fa	65142663	65150142	SLC25A45	1.4786783	152
chr11.fa	65154041	65180995	FRMD8	7.209474074	1216
chr11.fa	65190269	65194003	NEAT1	10.86046846	1824
chr11.fa	65211929	65212028	MIR612	0	0
chr11.fa	65265233	65273939	MALAT1	0	0
chr11.fa	65292548	65306029	SCYL1	35.21184708	3952
chr11.fa	65306183	65325699	LTBP3	4.443818587	909
chr11.fa	65337943	65339239	SSSCA1	7.752997876	228
chr11.fa	65339820	65341669	FAM89B	27.82179144	1824
chr11.fa	65343509	65360116	EHBP1L1	5.54687835	1292
chr11.fa	65360326	65363467	KCNK7	0	0
chr11.fa	65365226	65381720	MAP3K11	12.29911636	1976
chr11.fa	65383783	65404910	PCNXL3	2.574176766	760
chr11.fa	65405578	65418391	SIPA1	5.016698012	836
chr11.fa	65421067	65430443	RELA	26.19855894	3037
chr11.fa	65479489	65484453	KAT5	17.86178448	1216
chr11.fa	65485144	65488409	RNASEH2C	11.06907129	988
chr11.fa	65543378	65547822	DKFZp761E198	13.30854948	2660
chr11.fa	65554529	65564685	OVOL1	24.84708834	3344
chr11.fa	65601410	65621172	SNX32	0.991197049	76
chr11.fa	65622285	65625804	CFL1	341.5304314	19304
chr11.fa	65627872	65633911	MUS81	7.756333742	836
chr11.fa	65633915	65640340	EFEMP2	7.60577498	684
chr11.fa	65647284	65651210	CTSW	1.30610282	76
chr11.fa	65651213	65656010	FIBP	22.17772819	1444
chr11.fa	65657875	65659106	CCDC85B	9.603291673	532
chr11.fa	65659692	65667997	FOSL1	0	0
chr11.fa	65684283	65686531	C11orf68	14.10293042	988
chr11.fa	65686728	65689048	DRAP1	40.36242452	1824
chr11.fa	65713115	65727434	TSGA10IP	0	0
chr11.fa	65729160	65747607	SART1	12.56153784	2034
chr11.fa	65764017	65769549	EIF1AD	7.225486231	912
chr11.fa	65769579	65771617	BANF1	69.23234513	3496
chr11.fa	65779462	65780976	CST6	0	0
chr11.fa	65784223	65793988	CATSPER1	0	0
chr11.fa	65808236	65816651	GAL3ST3	0.509053185	76
chr11.fa	65819816	65836382	SF3B2	67.66248649	8805
chr11.fa	65837824	66012213	PACS1	29.62738463	5979
chr11.fa	66024765	66035331	KLC2	12.35137826	1815
chr11.fa	66036056	66044963	RAB1B	123.6289812	10868
chr11.fa	66045696	66051683	CNIH2	0	0
chr11.fa	66052051	66056638	YIF1A	13.56963661	684

chr11.fa	66059373	66064135	TMEM151A	0	0
chr11.fa	66081958	66084515	CD248	0	0
chr11.fa	66099542	66104000	RIN1	0	0
chr11.fa	66104804	66112582	BRMS1	29.34583752	1788
chr11.fa	66112843	66115161	B3GNT1	0.816397659	76
chr11.fa	66129992	66139291	SLC29A2	1.344131695	152
chr11.fa	66188475	66194177	NPAS4	0	0
chr11.fa	66202550	66206310	MRPL11	11.02726177	836
chr11.fa	66234336	66244808	PELI3	2.46387079	304
chr11.fa	66247880	66277130	DPP3	14.4674294	1748
chr11.fa	66278119	66301084	BBS1	15.76530375	2389
chr11.fa	66306735	66313671	ZDHHC24	0	0
chr11.fa	66314391	66330797	ACTN3	0	0
chr11.fa	66330935	66336047	CTSF	2.517689431	228
chr11.fa	66357640	66360554	CCDC87	0	0
chr11.fa	66360690	66373490	CCS	21.29083256	988
chr11.fa	66391685	66397397	RBM14	6.891454827	1528
chr11.fa	66406088	66435858	RBM4	13.29876427	1849
chr11.fa	66432470	66445275	RBM4B	4.907503991	380
chr11.fa	66452720	66488870	SPTBN2	26.46520585	9362
chr11.fa	66512207	66610882	C11orf80	5.885579967	587
chr11.fa	66610988	66614003	RCE1	3.711929538	228
chr11.fa	66615997	66725847	PC	10.36186765	1976
chr11.fa	66624876	66627946	LRFN4	0	0
chr11.fa	66742754	66744479	C11orf86	2.8526104	152
chr11.fa	66790190	66818334	SYT12	0	0
chr11.fa	66824289	66839488	RHOD	11.9341726	608
chr11.fa	66886740	67025550	KDM2A	32.69082178	10844
chr11.fa	67033905	67054029	ADRBK1	48.46235248	7505
chr11.fa	67056762	67069955	ANKRD13D	8.16508855	836
chr11.fa	67070919	67080078	SSH3	33.79921893	4374
chr11.fa	67085310	67159158	LOC100130987	3.976797316	152
chr11.fa	67119019	67121052	POLD4	65.62049158	2812
chr11.fa	67131639	67141648	CLCF1	0.856650445	76
chr11.fa	67159423	67165651	RAD9A	4.471395081	380
chr11.fa	67165884	67169376	PPP1CA	92.83648972	5168
chr11.fa	67171412	67177560	TBC1D10C	0	0
chr11.fa	67183149	67193078	CARNS1	0	0
chr11.fa	67195935	67202879	RPS6KB2	23.87257062	1900
chr11.fa	67202981	67205153	PTPRCAP	0	0
chr11.fa	67205518	67211263	CORO1B	63.27315371	5548
chr11.fa	67218772	67220200	GPR152	0	0
chr11.fa	67222818	67229245	CABP4	1.261402213	228

chr11.fa	67231819	67236731	TMEM134	74.55638761	3091
chr11.fa	67250505	67258579	AIP	43.86486166	2432
chr11.fa	67259239	67272843	PITPNM1	4.39934037	836
chr11.fa	67273968	67276102	CDK2AP2	40.47584397	2508
chr11.fa	67286418	67290899	CABP2	0	0
chr11.fa	67351066	67354124	GSTP1	135.1292688	5821
chr11.fa	67374323	67380012	NDUFV1	51.81400847	3800
chr11.fa	67380995	67383135	DOC2GP	0	0
chr11.fa	67395409	67397401	NUDT8	6.267647844	228
chr11.fa	67398774	67407031	TBX10	0	0
chr11.fa	67410026	67418130	ACY3	23.40243588	1368
chr11.fa	67429633	67448685	ALDH3B2	14.45675463	1748
chr11.fa	67559238	67572807	LOC645332	0	0
chr11.fa	67758575	67771593	UNC93B1	0	0
chr11.fa	67776048	67796743	ALDH3B1	18.45912692	2432
chr11.fa	67798084	67804114	NDUFS8	73.49424781	2657
chr11.fa	67806462	67818366	TCIRG1	1.667488328	228
chr11.fa	67820326	67888858	CHKA	8.709057135	1064
chr11.fa	67923507	67980784	SUV420H1	20.45619883	5358
chr11.fa	68028803	68039469	C11orf24	4.020385968	380
chr11.fa	68080108	68216743	LRP5	3.955225381	912
chr11.fa	68228186	68382801	PPP6R3	26.50568102	6231
chr11.fa	68451983	68458643	GAL	0	0
chr11.fa	68474908	68518988	MTL5	0	0
chr11.fa	68522088	68609399	CPT1A	7.703627056	1900
chr11.fa	68658746	68671303	MRPL21	54.60968675	1741
chr11.fa	68671319	68708069	IGHMBP2	4.702014632	836
chr11.fa	68747490	68748455	MRGPRD	0	0
chr11.fa	68771862	68780850	MRGPRF	0	0
chr11.fa	68816350	68858072	TPCN2	0.67251063	152
chr11.fa	69061622	69064754	MYEOV	0	0
chr11.fa	69455873	69469242	CCND1	70.93274734	13680
chr11.fa	69480332	69490165	ORAOV1	4.681999435	532
chr11.fa	69513006	69519106	FGF19	0	0
chr11.fa	69587797	69590171	FGF4	0	0
chr11.fa	69624736	69634192	FGF3	0	0
chr11.fa	69924408	70035652	ANO1	0.412535456	76
chr11.fa	70049269	70053508	FADD	19.15476622	1596
chr11.fa	70116823	70230502	PPFIA1	23.33127073	5469
chr11.fa	70130061	70130176	MIR548K	0	0
chr11.fa	70244612	70282690	CTTN	107.5785721	11600
chr11.fa	70313961	70935808	SHANK2	19.08515781	8968
chr11.fa	70718375	70718473	MIR3664	0	0

chr11.fa	71145457	71159477	DHCR7	3.171074428	380
chr11.fa	71164217	71212581	NADSYN1	12.51972831	1368
chr11.fa	71238313	71239210	KRTAP5-7	0	0
chr11.fa	71249071	71250253	KRTAP5-8	1.428640306	76
chr11.fa	71259466	71260653	KRTAP5-9	0	0
chr11.fa	71276609	71277666	KRTAP5-10	0	0
chr11.fa	71292901	71293921	KRTAP5-11	0	0
chr11.fa	71498557	71512280	FAM86C	3.855371785	380
chr11.fa	71544246	71548608	DEFB108B	0	0
chr11.fa	71576555	71639493	LOC100133315	0	0
chr11.fa	71639768	71708643	RNF121	2.46387079	304
chr11.fa	71709958	71713574	IL18BP	0	0
chr11.fa	71713911	71780957	NUMA1	158.9571388	50834
chr11.fa	71783274	71783348	MIR3165	0	0
chr11.fa	71791382	71821828	LRTOMT	9.296169589	2052
chr11.fa	71808338	71814433	C11orf59	81.713155	4332
chr11.fa	71822203	71823822	C11orf51	36.674958	564
chr11.fa	71846771	71850934	FOLR3	2.000185385	76
chr11.fa	71900602	71907367	FOLR1	10.81710219	608
chr11.fa	71927819	71932994	FOLR2	0	0
chr11.fa	71935882	71950120	INPPL1	26.91465822	5624
chr11.fa	71950189	71955220	PHOX2A	0	0
chr11.fa	72003470	72145568	CLPB	8.138846402	1140
chr11.fa	72287185	72385494	PDE2A	0	0
chr11.fa	72326107	72326174	MIR139	0	0
chr11.fa	72396114	72463434	ARAP1	15.85804083	4026
chr11.fa	72465774	72504750	STARD10	55.01599526	4918
chr11.fa	72525451	72540680	ATG16L2	0	0
chr11.fa	72547790	72853143	FCHSD2	6.775811465	1368
chr11.fa	72929344	72947395	P2RY2	2.365573932	304
chr11.fa	72975570	73008850	P2RY6	0	0
chr11.fa	73019663	73080425	ARHGEF17	3.677681311	1292
chr11.fa	73087405	73108519	RELT	0	0
chr11.fa	73117028	73309228	FAM168A	5.739024244	456
chr11.fa	73357223	73373864	PLEKHB1	13.28030581	1727
chr11.fa	73386683	73472201	RAB6A	44.42751109	7020
chr11.fa	73498917	73575656	MRPL48	17.24665075	760
chr11.fa	73583713	73587890	CHCHD8	60.06738628	2204
chr11.fa	73588033	73638779	PAAF1	16.80920749	1198
chr11.fa	73661364	73681332	DNAJB13	0	0
chr11.fa	73685716	73693889	UCP2	115.0055447	8512
chr11.fa	73711326	73720282	UCP3	0	0
chr11.fa	73745480	73882064	C2CD3	8.340555113	2356

chr11.fa	73882368	73965748	PPME1	23.7260149	2617
chr11.fa	73977702	74022699	P4HA3	0	0
chr11.fa	74041361	74109502	PGM2L1	1.78935864	684
chr11.fa	74165886	74178600	KCNE3	2.752756804	380
chr11.fa	74202923	74204755	LIPT2	3.52111799	152
chr11.fa	74303629	74353765	POLD3	4.927519188	760
chr11.fa	74407474	74442186	CHRDL2	0	0
chr11.fa	74459913	74551954	RNF169	10.16393959	2888
chr11.fa	74553459	74660232	XRRA1	14.45519789	2273
chr11.fa	74660292	74690076	SPCS2	3.744843418	456
chr11.fa	74699950	74718743	NEU3	9.840805347	1216
chr11.fa	74799796	74800758	OR2AT4	0	0
chr11.fa	74862032	74917445	SLCO2B1	0.759465543	152
chr11.fa	74951950	74954748	LOC441617	0	0
chr11.fa	74976482	75062873	ARRB1	39.46329738	3911
chr11.fa	75046136	75046230	MIR326	0	0
chr11.fa	75110562	75116733	RPS3	1312.318874	49627
chr11.fa	75111435	75111582	SNORD15A	0	0
chr11.fa	75115465	75115610	SNORD15B	0	0
chr11.fa	75133438	75141674	KLHL35	0	0
chr11.fa	75145685	75236599	GDPD5	0	0
chr11.fa	75273170	75283846	SERPINH1	3.067440184	304
chr11.fa	75297963	75379479	MAP6	3.624307452	912
chr11.fa	75428934	75442331	MOGAT2	0	0
chr11.fa	75479778	75512579	DGAT2	4.135806939	456
chr11.fa	75526212	75855282	UVRAG	9.160733421	2128
chr11.fa	75897370	75917574	WNT11	0	0
chr11.fa	76061004	76091880	PRKRIR	6.88789657	988
chr11.fa	76156069	76262589	C11orf30	5.827091112	1444
chr11.fa	76368568	76381791	LRRC32	0	0
chr11.fa	76391210	76432833	GUCY2E	0	0
chr11.fa	76494285	76509198	TSKU	1.870976167	228
chr11.fa	76571917	76734850	ACER3	2.311532899	456
chr11.fa	76745435	76753005	B3GNT6	0	0
chr11.fa	76777992	76837198	CAPN5	80.46598582	15884
chr11.fa	76813886	76814377	OMP	3.435275033	76
chr11.fa	76839310	76926286	MYO7A	1.358587116	456
chr11.fa	76927603	76998463	GDPD4	0	0
chr11.fa	77033060	77185108	PAK1	16.30704843	2551
chr11.fa	77300680	77321401	AQP11	0.785040517	76
chr11.fa	77327196	77348851	CLNS1A	50.47121112	3116
chr11.fa	77377274	77531880	RSF1	21.06955343	4864
chr11.fa	77532208	77583398	C11orf67	30.18158321	684

chr11.fa	77589766	77705717	INTS4	8.587631604	1216
chr11.fa	77726761	77734320	KCTD14	9.103356522	684
chr11.fa	77774907	77779395	THRSP	0	0
chr11.fa	77779398	77791265	NDUFC2	13.00142739	1444
chr11.fa	77811988	77850699	ALG8	4.848347963	380
chr11.fa	77882298	77899664	KCTD21	8.505791686	1292
chr11.fa	77899858	77925757	USP35	1.176226429	228
chr11.fa	77926336	78128868	GAB2	7.318000921	2052
chr11.fa	78147016	78285909	NARS2	14.07001654	1588
chr11.fa	78364328	79151695	ODZ4	0.249522793	152
chr11.fa	79113066	79113153	MIR708	0	0
chr11.fa	81601783	81601878	MIR4300	0	0
chr11.fa	82443046	82444906	FAM181B	2.724513137	228
chr11.fa	82535409	82611557	PRCP	11.49250391	1140
chr11.fa	82612737	82645699	C11orf82	0.474360176	76
chr11.fa	82692478	82782884	RAB30	6.330139738	456
chr11.fa	82752506	82752640	SNORA70E	0	0
chr11.fa	82868137	82896835	PCF11	23.72868359	6308
chr11.fa	82905291	82960013	ANKRD42	5.937397089	684
chr11.fa	82972502	82997377	CCDC90B	18.03925256	1280
chr11.fa	83166056	85338314	DLG2	0.382735051	152
chr11.fa	85339622	85347583	TMEM126B	21.5067743	1292
chr11.fa	85358963	85367597	TMEM126A	18.59611983	684
chr11.fa	85368608	85376182	CREBZF	2.883077978	982
chr11.fa	85394893	85397320	CCDC89	0	0
chr11.fa	85405265	85522178	SYTL2	70.04140388	22040
chr11.fa	85566144	85631051	CCDC83	0	0
chr11.fa	85668485	85780108	PICALM	39.77553445	6927
chr11.fa	85955815	85989780	EED	12.70164422	1140
chr11.fa	86013253	86056985	C11orf73	0	0
chr11.fa	86085778	86134151	CCDC81	0	0
chr11.fa	86152150	86383678	ME3	10.83911891	1140
chr11.fa	86511491	86522273	PRSS23	0	0
chr11.fa	86656721	86666433	FZD4	2.289293791	760
chr11.fa	86748886	87039876	TMEM135	3.328527314	1359
chr11.fa	87846415	87908635	RAB38	0	0
chr11.fa	88026760	88070941	CTSC	0.446116509	152
chr11.fa	88237744	88796816	GRM5	0.210604354	76
chr11.fa	88911040	89028927	TYR	0	0
chr11.fa	89057522	89322779	NOX4	11.6846498	2574
chr11.fa	89392465	89431886	FOLH1B	3.424822652	304
chr11.fa	89443467	89451040	TRIM77	0	0
chr11.fa	89530823	89541743	TRIM49	0	0

chr11.fa	89575165	89735676	TRIM53	0	0
chr11.fa	89603606	89609185	TRIM64B	0	0
chr11.fa	89644579	89666229	TRIM49L1	0	0
chr11.fa	89701672	89707240	TRIM64	0	0
chr11.fa	89764274	89775193	TRIM49L2	0	0
chr11.fa	89819118	89820299	UBTFL1	0	0
chr11.fa	89867818	89925779	NAALAD2	0	0
chr11.fa	89933597	89956532	CHORDC1	3.922311501	597
chr11.fa	92085262	92629635	FAT3	0.532404249	456
chr11.fa	92702789	92715948	MTNR1B	0	0
chr11.fa	92880850	92931095	SLC36A4	2.742749206	304
chr11.fa	93063883	93171636	CCDC67	1.901888527	228
chr11.fa	93211639	93276546	C11orf75	72.80750415	3192
chr11.fa	93394816	93463522	KIAA1731	5.673196484	2052
chr11.fa	93454680	93455032	SCARNA9	0	0
chr11.fa	93463681	93463812	SNORA25	0	0
chr11.fa	93464145	93464265	SNORA32	0	0
chr11.fa	93464669	93464739	SNORD6	0	0
chr11.fa	93465170	93465299	SNORA1	0	0
chr11.fa	93465527	93465665	SNORA8	0	0
chr11.fa	93466394	93466466	SNORD5	0	0
chr11.fa	93466632	93466763	SNORA18	0	0
chr11.fa	93466840	93466930	MIR1304	0	0
chr11.fa	93468276	93468402	SNORA40	0	0
chr11.fa	93469096	93474703	TAF1D	16.75983667	988
chr11.fa	93474793	93496247	C11orf54	35.35929236	3800
chr11.fa	93517405	93546496	MED17	8.21646089	1292
chr11.fa	93553735	93583668	C11orf90	0	0
chr11.fa	93754378	93847374	HEPHL1	0	0
chr11.fa	93862094	93915137	PANX1	1.831835337	228
chr11.fa	94038803	94040858	FOLR4	0	0
chr11.fa	94110477	94134585	GPR83	0	0
chr11.fa	94150469	94227040	MRE11A	2.577957414	608
chr11.fa	94227153	94232744	ANKRD49	5.323375313	456
chr11.fa	94245695	94265289	LOC643037	0	0
chr11.fa	94277017	94283064	FUT4	1.117737575	304
chr11.fa	94300474	94354587	PIWIL4	0	0
chr11.fa	94501508	94609918	AMOTL1	27.79710603	11248
chr11.fa	94695787	94706776	CWC15	21.46251847	1520
chr11.fa	94706845	94732676	KDM4D	0	0
chr11.fa	94758422	94760760	KDM4DL	0.722548623	76
chr11.fa	94800056	94804388	SRSF8	21.84391918	4256
chr11.fa	94822974	94865815	ENDOD1	2.521025298	532

chr11.fa	94906133	94964246	SESN3	13.60988939	1216
chr11.fa	95502106	95522954	FAM76B	1.71107698	304
chr11.fa	95523642	95565854	CEP57	10.70412753	1520
chr11.fa	95566044	95657371	MTMR2	18.42443391	4028
chr11.fa	95711440	96076344	MAML2	21.8434744	5244
chr11.fa	96085929	96123083	CCDC82	4.952871772	608
chr11.fa	96123158	96126727	JRKL	7.101614399	1140
chr11.fa	98891871	100227473	CNTN5	0	0
chr11.fa	100558407	100861656	ARHGAP42	4.268129632	912
chr11.fa	100862811	100864666	TMEM133	2.731852043	228
chr11.fa	100900355	101000544	PGR	0	0
chr11.fa	101322295	101454659	TRPC6	0	0
chr11.fa	101390551	101390636	MIR3920	0	0
chr11.fa	101761405	101787253	ANGPTL5	0	0
chr11.fa	101785746	101871793	KIAA1377	0	0
chr11.fa	101918169	101955291	C11orf70	0.769028359	76
chr11.fa	101981192	102104154	YAP1	27.91208222	7144
chr11.fa	102188194	102208465	BIRC3	1.266517208	152
chr11.fa	102217966	102249401	BIRC2	9.857039896	1659
chr11.fa	102267056	102323775	TMEM123	10.35764222	1672
chr11.fa	102391239	102401478	MMP7	1.510480224	76
chr11.fa	102447566	102496063	MMP20	0	0
chr11.fa	102562415	102576468	MMP27	0	0
chr11.fa	102582526	102595685	MMP8	0	0
chr11.fa	102641233	102651359	MMP10	0	0
chr11.fa	102660641	102668966	MMP1	0	0
chr11.fa	102706528	102714342	MMP3	0	0
chr11.fa	102733464	102745764	MMP12	0	0
chr11.fa	102813721	102826463	MMP13	0	0
chr11.fa	102921413	102962944	DCUN1D5	1.592987315	912
chr11.fa	102980160	103350591	DYNC2H1	2.714283147	1672
chr11.fa	103777914	104035027	PDGFD	0	0
chr11.fa	103907308	103909922	DDI1	0	0
chr11.fa	104756445	104769397	CASP12	0	0
chr11.fa	104772276	104788902	LOC643733	2.730517696	76
chr11.fa	104813594	104839325	CASP4	20.26294098	1292
chr11.fa	104864967	104893895	CASP5	0	0
chr11.fa	104896237	104905201	CASP1	5.079412297	304
chr11.fa	104912053	104916051	CARD16	0	0
chr11.fa	104963196	104971506	CARD17	0	0
chr11.fa	105008448	105010461	CARD18	0	0
chr11.fa	105480800	105852819	GRIA4	0	0
chr11.fa	105878629	105892954	KIAA1826	3.308289725	608

chr11.fa	105921825	105948291	KBTBD3	2.083137259	380
chr11.fa	105948466	105969419	AASDHPPT	11.95129671	1444
chr11.fa	106557910	106889171	GUCY1A2	1.716414365	228
chr11.fa	107197072	107328572	CWF19L2	8.247150859	1216
chr11.fa	107373453	107436461	ALKBH8	4.155822136	760
chr11.fa	107461817	107537505	ELMOD1	0	0
chr11.fa	107462471	107463949	LOC643923	0	0
chr11.fa	107578101	107582787	SLN	0	0
chr11.fa	107661717	107729914	SLC35F2	2.665801892	380
chr11.fa	107799277	107834208	RAB39	0	0
chr11.fa	107879408	107978488	CUL5	10.5851483	3040
chr11.fa	107992258	108018891	ACAT1	49.63679978	4763
chr11.fa	108028119	108093365	NPAT	6.736225853	1798
chr11.fa	108093559	108239826	ATM	3.471079997	2052
chr11.fa	108253727	108338258	C11orf65	0	0
chr11.fa	108342833	108369159	KDELC2	3.13638142	608
chr11.fa	108376158	108464374	EXPH5	10.61850696	4864
chr11.fa	108535816	108811648	DDX10	11.12311232	1596
chr11.fa	109292846	109299893	C11orf87	0	0
chr11.fa	109964087	110042566	ZC3H12C	4.605941685	1824
chr11.fa	110100166	110167437	RDX	16.18651246	3268
chr11.fa	110300594	110335605	FDX1	11.55143754	1672
chr11.fa	110447766	110583451	ARHGAP20	0	0
chr11.fa	111126707	111156972	C11orf53	0	0
chr11.fa	111164114	111170539	C11orf92	2.431846474	587
chr11.fa	111169976	111179353	C11orf93	2.63466714	152
chr11.fa	111222981	111250157	POU2AF1	1.115291273	152
chr11.fa	111338256	111383064	BTG4	0	0
chr11.fa	111383663	111383746	MIR34B	0	0
chr11.fa	111384164	111384240	MIR34C	0	0
chr11.fa	111385510	111407756	C11orf88	0	0
chr11.fa	111411233	111431788	LAYN	0	0
chr11.fa	111473170	111597632	SIK2	11.31147757	2888
chr11.fa	111597632	111637169	PPP2R1B	11.79117513	3116
chr11.fa	111652919	111742305	ALG9	1.267851554	380
chr11.fa	111744780	111749947	FDXACB1	2.387145867	304
chr11.fa	111750154	111754797	C11orf1	28.27836033	988
chr11.fa	111779350	111782473	CRYAB	85.60922436	2660
chr11.fa	111784165	111784817	HSPB2	2.588409795	76
chr11.fa	111789402	111789600	HSPB2-C11ORF52	0	0
chr11.fa	111807927	111893307	DIXDC1	3.177968552	912
chr11.fa	111895538	111935002	DLAT	8.176875277	1520
chr11.fa	111938523	111944895	PIH1D2	1.359699071	76

chr11.fa	111944968	111955538	C11orf57	12.39652365	1898
chr11.fa	111955875	111957522	TIMM8B	65.16837051	2356
chr11.fa	111957571	111966518	SDHD	24.76435886	1520
chr11.fa	112013976	112034840	IL18	3.904075432	201
chr11.fa	112038095	112043279	TEX12	0	0
chr11.fa	112046208	112089649	BCO2	0	0
chr11.fa	112097088	112104695	PTS	16.28636606	684
chr11.fa	112118876	112131583	C11orf34	0	0
chr11.fa	112831995	113149158	NCAM1	0	0
chr11.fa	113140254	113144623	LOC100288346	0	0
chr11.fa	113185251	113237114	TTC12	15.33208593	1596
chr11.fa	113258513	113271140	ANKK1	0	0
chr11.fa	113280317	113346001	DRD2	0	0
chr11.fa	113320745	113320810	MIR4301	0	0
chr11.fa	113558268	113577068	TMPRSS5	0	0
chr11.fa	113603909	113644425	ZW10	9.376897552	1216
chr11.fa	113650518	113651207	CLDN25	0	0
chr11.fa	113668597	113746256	USP28	5.473266902	1140
chr11.fa	113775589	113817283	HTR3B	0	0
chr11.fa	113845797	113861034	HTR3A	0	0
chr11.fa	113930431	114121397	ZBTB16	13.24249933	1444
chr11.fa	114166535	114183238	NNMT	0	0
chr11.fa	114262170	114271139	C11orf71	13.06169538	380
chr11.fa	114271384	114279635	RBM7	7.053355534	608
chr11.fa	114310108	114321000	REXO2	64.11001135	3344
chr11.fa	114392437	114430580	FAM55A	0	0
chr11.fa	114441313	114466484	FAM55D	0	0
chr11.fa	114549200	114577652	FAM55B	0	0
chr11.fa	115044345	115375241	CADM1	1.96215651	380
chr11.fa	115626051	115630918	LOC283143	0.753460983	76
chr11.fa	116618886	116643714	BUD13	8.427954808	836
chr11.fa	116649276	116658739	ZNF259	14.15897297	1132
chr11.fa	116660086	116663136	APOA5	0	0
chr11.fa	116691418	116694011	APOA4	0	0
chr11.fa	116700624	116703787	APOC3	0	0
chr11.fa	116706469	116708338	APOA1	1.884319632	76
chr11.fa	116714118	116968993	SIK3	10.86113563	2964
chr11.fa	117015000	117047592	PAFAH1B2	28.49652598	8056
chr11.fa	117049939	117068161	SIDT2	4.744046546	836
chr11.fa	117070040	117075508	TAGLN	0	0
chr11.fa	117075788	117102811	PCSK7	5.336273996	836
chr11.fa	117103404	117156404	RNF214	7.41852169	912
chr11.fa	117156415	117186972	BACE1	4.635297308	1216

chr11.fa	117198571	117283982	CEP164	5.705887973	1444
chr11.fa	117298489	117667976	DSCAML1	0.245074972	76
chr11.fa	117690790	117698807	FXYD2	159.8956292	4810
chr11.fa	117707693	117748145	FXYD6	3.582497929	380
chr11.fa	117771360	117800115	TMPRSS13	1.495357631	228
chr11.fa	117857106	117872199	IL10RA	0	0
chr11.fa	117947727	117990556	TMPRSS4	0.956726432	152
chr11.fa	118004092	118023630	SCN4B	0	0
chr11.fa	118033519	118047337	SCN2B	0	0
chr11.fa	118064442	118095809	AMICA1	0	0
chr11.fa	118100336	118123011	MPZL3	10.31649987	456
chr11.fa	118124131	118135251	MPZL2	3.183083547	456
chr11.fa	118175295	118186890	CD3E	0	0
chr11.fa	118209789	118213459	CD3D	0	0
chr11.fa	118215059	118224497	CD3G	0	0
chr11.fa	118230302	118269926	UBE4A	5.245538435	1444
chr11.fa	118272104	118280562	ATP5L	102.0663868	6384
chr11.fa	118307205	118397539	MLL	12.93559963	9652
chr11.fa	118398210	118401740	TTC36	2.489223373	76
chr11.fa	118401803	118417313	TMEM25	2.807687401	380
chr11.fa	118415243	118436791	IFT46	18.02857779	1520
chr11.fa	118443102	118473747	ARCN1	67.77523876	12236
chr11.fa	118477213	118528741	PHLDB1	3.49443106	912
chr11.fa	118528942	118550381	TREH	0.912025824	76
chr11.fa	118618473	118661972	DDX6	67.03156299	18766
chr11.fa	118754541	118766850	CXCR5	0	0
chr11.fa	118766972	118781613	BCL9L	15.08879008	5168
chr11.fa	118827008	118829269	UPK2	86.86106375	3648
chr11.fa	118842417	118851995	FOXR1	0	0
chr11.fa	118868852	118886421	CCDC84	2.894197532	152
chr11.fa	118873671	118874303	RPL23AP64	0	0
chr11.fa	118886456	118889057	RPS25	1165.902145	24011
chr11.fa	118889241	118894385	TRAPPC4	50.14207232	2647
chr11.fa	118889681	118889722	MIR3656	0	0
chr11.fa	118895061	118901616	SLC37A4	6.704646319	836
chr11.fa	118914896	118927925	HYOU1	6.964399102	1444
chr11.fa	118938493	118952688	VPS11	18.88456106	2736
chr11.fa	118955587	118964259	HMBS	9.501214166	684
chr11.fa	118964585	118966177	H2AFX	6.365944702	456
chr11.fa	118967213	118972785	DPAGT1	1.578087113	152
chr11.fa	118978093	118987827	C2CD2L	3.032524785	456
chr11.fa	118992288	119005765	HINFP	10.28803381	1064
chr11.fa	119019750	119033374	ABCG4	0.859096747	152

chr11.fa	119039440	119054725	NLRX1	9.028633119	1520
chr11.fa	119056166	119060932	PDZD3	5.173261333	532
chr11.fa	119060963	119066584	CCDC153	0	0
chr11.fa	119076990	119178859	CBL	5.720788175	2888
chr11.fa	119179234	119187840	MCAM	0	0
chr11.fa	119205237	119208022	RNF26	1.820048609	228
chr11.fa	119225925	119252436	USP2	5.402546538	988
chr11.fa	119252488	119369944	LOC100499227	1.461998968	76
chr11.fa	119288655	119294246	THY1	2.820586084	304
chr11.fa	119508808	119599435	PVRL1	2.764988314	760
chr11.fa	119981994	120008863	TRIM29	1.119739094	152
chr11.fa	120081747	120100650	OAF	5.446357581	456
chr11.fa	120110951	120190653	POU2F3	2.850164098	380
chr11.fa	120195838	120204388	TMEM136	2.470320131	456
chr11.fa	120207618	120360645	ARHGEF12	34.63807809	15309
chr11.fa	120531028	120856969	GRIK4	0	0
chr11.fa	120894803	120960354	TBCEL	7.950703548	1444
chr11.fa	120973375	121061515	TECTA	0	0
chr11.fa	121163388	121184119	SC5DL	4.723141785	1520
chr11.fa	121322912	121504471	SORL1	14.15452515	6984
chr11.fa	121959811	122073770	LOC399959	0	0
chr11.fa	121970465	121970552	MIR125B1	0	0
chr11.fa	121986062	121986923	BLID	0	0
chr11.fa	122017230	122017301	MIRLET7A2	0	0
chr11.fa	122022937	122023016	MIR100	0	0
chr11.fa	122526398	122685187	UBASH3B	10.52843857	3268
chr11.fa	122709255	122743347	CRTAM	0	0
chr11.fa	122753473	122830430	C11orf63	5.143016146	836
chr11.fa	122848357	122852379	BSX	0	0
chr11.fa	122888274	122890319	LOC341056	0	0
chr11.fa	122928200	122932844	HSPA8	691.308236	67455
chr11.fa	122943033	123066007	ASAM	0	0
chr11.fa	123396528	123493518	GRAMD1B	1.224262903	152
chr11.fa	123499895	123525315	SCN3B	0	0
chr11.fa	123594997	123612363	ZNF202	2.084026823	380
chr11.fa	123624288	123625226	OR6X1	0	0
chr11.fa	123676116	123677057	OR6M1	0	0
chr11.fa	123753633	123756340	TMEM225	0	0
chr11.fa	123777139	123778083	OR8D4	0	0
chr11.fa	123810324	123811280	OR4D5	0	0
chr11.fa	123813574	123814545	OR6T1	0	0
chr11.fa	123847403	123848398	OR10S1	0	0
chr11.fa	123886282	123887217	OR10G4	0	0

chr11.fa	123893720	123894655	OR10G9	0	0
chr11.fa	123900330	123901265	OR10G8	0	0
chr11.fa	123908773	123909708	OR10G7	0	0
chr11.fa	123986111	124017618	VWA5A	9.081784587	1444
chr11.fa	124095398	124096312	OR8G2	0	0
chr11.fa	124120423	124121283	OR8G1	0	0
chr11.fa	124134723	124135763	OR8G5	0	0
chr11.fa	124179736	124180662	OR8D1	0	0
chr11.fa	124189158	124190093	OR8D2	0	0
chr11.fa	124252298	124253239	OR8B2	0	0
chr11.fa	124266306	124267247	OR8B3	0	0
chr11.fa	124293838	124294767	OR8B4	0	0
chr11.fa	124310046	124310981	OR8B8	0	0
chr11.fa	124412618	124413550	OR8B12	0	0
chr11.fa	124439965	124440945	OR8A1	0	0
chr11.fa	124481453	124490251	PANX3	0	0
chr11.fa	124492742	124505692	TBRG1	10.4079026	2355
chr11.fa	124505823	124543717	SIAE	3.119479697	380
chr11.fa	124543740	124564687	SPA17	3.607628121	152
chr11.fa	124609829	124617102	NRGN	0	0
chr11.fa	124617370	124622109	VSIG2	22.61606101	1140
chr11.fa	124623019	124632223	ESAM	0.904242137	76
chr11.fa	124636394	124670299	C11orf61	2.27884141	228
chr11.fa	124735305	124751370	ROBO3	1.113512144	228
chr11.fa	124754114	124767831	ROBO4	0	0
chr11.fa	124792284	124806308	HEPACAM	0	0
chr11.fa	124824017	124911385	CCDC15	0.868214781	152
chr11.fa	124933013	124960412	SLC37A2	0.402750248	76
chr11.fa	124966399	124973205	TMEM218	6.429548551	608
chr11.fa	125034559	125303285	PKNOX2	2.718730969	456
chr11.fa	125315641	125366206	FEZ1	0	0
chr11.fa	125439298	125454575	EI24	35.852111	3645
chr11.fa	125462739	125491280	STT3A	7.235938612	912
chr11.fa	125495036	125546150	CHEK1	1.567412341	228
chr11.fa	125542229	125550793	ACRV1	0	0
chr11.fa	125616188	125619743	PATE1	0	0
chr11.fa	125646028	125648714	PATE2	0	0
chr11.fa	125658006	125661495	PATE3	0	0
chr11.fa	125703211	125709967	PATE4	0	0
chr11.fa	125753509	125770541	HYLS1	2.013751241	228
chr11.fa	125763380	125773116	PUS3	4.582813012	380
chr11.fa	125774272	125793005	DDX25	0.9275932	76
chr11.fa	125826713	125933187	CDON	0.420096753	152

chr11.fa	126071989	126081587	RPUSD4	6.929706093	760
chr11.fa	126081619	126132813	FAM118B	10.02071973	912
chr11.fa	126132880	126138877	SRPR	25.6932864	3496
chr11.fa	126138935	126148027	FOXRED1	6.160010561	608
chr11.fa	126152982	126164828	TIRAP	5.03893712	532
chr11.fa	126173647	126215644	DCPS	10.28492034	684
chr11.fa	126211612	126225482	FLJ39051	0	0
chr11.fa	126226096	126284533	ST3GAL4	15.51511379	1216
chr11.fa	126293396	126870766	KIRREL3	0	0
chr11.fa	126858354	126858438	MIR3167	0	0
chr11.fa	128328656	128457453	ETS1	2.745195508	684
chr11.fa	128562389	128683162	FLI1	0.394299387	76
chr11.fa	128707915	128737268	KCNJ1	5.959636197	760
chr11.fa	128761313	128787951	KCNJ5	0.583109415	76
chr11.fa	128769460	128775592	C11orf45	0	0
chr11.fa	128804627	128813294	TP53AIP1	0	0
chr11.fa	128834955	129062093	ARHGAP32	23.94662685	11248
chr11.fa	129245881	129322174	BARX2	0	0
chr11.fa	129685741	129729898	TMEM45B	2.297522261	228
chr11.fa	129733670	129765490	NFRKB	7.920903143	1799
chr11.fa	129769601	129830977	PRDM10	5.738801853	1667
chr11.fa	129872519	129875381	NCRNA00167	0	0
chr11.fa	129939716	130014706	APLP2	19.95226064	3420
chr11.fa	130029682	130080257	ST14	6.658166583	988
chr11.fa	130096574	130184607	ZBTB44	9.410033823	3952
chr11.fa	130274818	130298539	ADAMTS8	0	0
chr11.fa	130318869	130346539	ADAMTS15	0	0
chr11.fa	130745766	130786382	SNX19	8.06434539	2204
chr11.fa	131240371	132206716	NTM	0	0
chr11.fa	132284875	133402403	OPCML	0	0
chr11.fa	133710517	133715392	SPATA19	0	0
chr11.fa	133766330	133771635	LOC283174	0.318464028	76
chr11.fa	133785185	133826880	IGSF9B	0	0
chr11.fa	133902167	133911236	LOC100128239	0	0
chr11.fa	133938820	134021649	JAM3	0.922255814	152
chr11.fa	134022337	134094426	NCAPD3	35.55077108	8960
chr11.fa	134094561	134117686	VPS26B	30.86432383	5092
chr11.fa	134118173	134123260	THYN1	33.43560951	1520
chr11.fa	134123434	134135746	ACAD8	24.57310253	2432
chr11.fa	134146275	134189458	GLB1L3	0	0
chr11.fa	134201768	134246218	GLB1L2	0	0
chr11.fa	134248398	134281812	B3GAT1	0.462351058	76
chr11.fa	134306376	134375555	LOC283177	0.617580033	76

chr12.fa	87984	91263	LOC100288778	2.768546571	152
chr12.fa	147946	149412	FAM138D	0	0
chr12.fa	176049	287625	IQSEC3	0	0
chr12.fa	246577	258332	LOC574538	0	0
chr12.fa	299250	323371	SLC6A12	2.215682343	380
chr12.fa	329789	372039	SLC6A13	4.929298317	684
chr12.fa	389223	498515	KDM5A	17.32693393	8360
chr12.fa	498621	551806	CCDC77	3.522674728	380
chr12.fa	569543	671058	B4GALNT3	2.949795302	456
chr12.fa	673462	772755	NINJ2	1.591430578	76
chr12.fa	862089	1020618	WNK1	28.23543885	15167
chr12.fa	1021255	1058863	RAD52	0.632257844	76
chr12.fa	1100404	1605099	ERC1	15.68991318	6612
chr12.fa	1609657	1613590	LOC100292680	0	0
chr12.fa	1675159	1703331	FBXL14	7.205915816	684
chr12.fa	1726222	1756378	WNT5B	0	0
chr12.fa	1769481	1769546	MIR3649	0	0
chr12.fa	1800247	1897845	ADIPOR2	12.78815435	2280
chr12.fa	1901123	2027870	CACNA2D4	0	0
chr12.fa	1929433	1945918	LRTM2	0	0
chr12.fa	2038368	2045742	LOC100271702	0	0
chr12.fa	2055214	2113677	DCP1B	6.463351996	608
chr12.fa	2162416	2807115	CACNA1C	0.11920162	76
chr12.fa	2870366	2880835	LOC283440	0	0
chr12.fa	2904108	2914587	FKBP4	86.74497561	14592
chr12.fa	2921787	2934237	ITFG2	14.12316801	1500
chr12.fa	2934514	2944221	NRIP2	0	0
chr12.fa	2966849	2986303	FOXM1	0.466131706	76
chr12.fa	2986365	2998626	C12orf32	12.54641524	1064
chr12.fa	3000033	3050306	TULP3	10.13591831	912
chr12.fa	3068478	3149842	TEAD4	0	0
chr12.fa	3186521	3395730	TSPAN9	5.049834283	988
chr12.fa	3600425	3703138	PRMT8	0	0
chr12.fa	3724494	3862366	EFCAB4B	23.53097792	4007
chr12.fa	3918027	3982608	PARP11	5.643840861	1140
chr12.fa	4382902	4414522	CCND2	4.66465293	1368
chr12.fa	4430359	4469190	C12orf5	3.082340387	1140
chr12.fa	4477393	4488894	FGF23	0	0
chr12.fa	4543308	4554780	FGF6	0	0
chr12.fa	4596901	4647637	C12orf4	1.327674755	228
chr12.fa	4647950	4669213	RAD51AP1	0	0
chr12.fa	4699244	4723041	DYRK4	5.496395574	456
chr12.fa	4724676	4754343	AKAP3	1.119294312	152

chr12.fa	4758264	4796720	NDUFA9	21.00906306	1520
chr12.fa	4829752	4881892	GALNT8	0	0
chr12.fa	4918342	4960278	KCNA6	0	0
chr12.fa	5019073	5027422	KCNA1	0	0
chr12.fa	5153085	5155949	KCNA5	0	0
chr12.fa	5541280	5604465	NTF3	0	0
chr12.fa	5671817	6055398	ANO2	0	0
chr12.fa	6058040	6233836	VWF	4.400897108	1748
chr12.fa	6309555	6347427	CD9	32.9521313	1824
chr12.fa	6419602	6437672	PLEKHG6	17.94028853	2888
chr12.fa	6437923	6451283	TNFRSF1A	4.5556813	456
chr12.fa	6456009	6486523	SCNN1A	16.23744002	2508
chr12.fa	6493357	6500732	LTBR	11.2409796	1064
chr12.fa	6548167	6560433	LOC678655	7.146537398	608
chr12.fa	6554051	6560884	CD27	0	0
chr12.fa	6561177	6571403	TAPBPL	0.975184891	76
chr12.fa	6571489	6579843	VAMP1	0	0
chr12.fa	6601316	6602471	MRPL51	162.2765481	4940
chr12.fa	6603298	6641132	NCAPD2	7.033562728	1520
chr12.fa	6619388	6619717	SCARNA10	0	0
chr12.fa	6643657	6647536	GAPDH	768.7092279	45281
chr12.fa	6648694	6665249	IFFO1	0.602235048	76
chr12.fa	6666037	6677480	NOP2	11.76737928	1444
chr12.fa	6679248	6716551	CHD4	59.57345569	17404
chr12.fa	6690639	6690775	SCARNA11	0	0
chr12.fa	6728001	6745297	LPAR5	0.578883985	76
chr12.fa	6747242	6756580	ACRBP	0	0
chr12.fa	6759704	6772308	ING4	28.01393734	1824
chr12.fa	6775643	6798669	ZNF384	8.854278511	1368
chr12.fa	6802957	6809812	C12orf53	0	0
chr12.fa	6833150	6841041	COPS7A	39.59628724	4104
chr12.fa	6857158	6862636	MLF2	71.28545959	6164
chr12.fa	6875541	6880118	PTMS	229.7204242	11879
chr12.fa	6881670	6887621	LAG3	0	0
chr12.fa	6898638	6929976	CD4	0	0
chr12.fa	6930963	6936583	GPR162	0	0
chr12.fa	6937572	6949018	LEPREL2	1.281862193	152
chr12.fa	6949375	6956557	GNB3	0	0
chr12.fa	6957972	6960456	CDCA3	0	0
chr12.fa	6961285	6975795	USP5	22.78285432	3268
chr12.fa	6976584	6980099	TPI1	86.1118282	7144
chr12.fa	6980111	6982521	SPSB2	9.272151353	532
chr12.fa	6993145	6993768	RPL13P5	0	0

chr12.fa	6993846	6994902	DSTNP2	0	0
chr12.fa	7013897	7023406	LRRC23	7.267295755	532
chr12.fa	7023614	7032859	ENO2	1.395059253	152
chr12.fa	7033626	7051484	ATN1	37.4829048	7448
chr12.fa	7053203	7055165	C12orf57	155.632837	3800
chr12.fa	7055740	7070479	PTPN6	12.58355455	1444
chr12.fa	7072862	7072929	MIR200C	0	0
chr12.fa	7073260	7073354	MIR141	0	0
chr12.fa	7074515	7079891	PHB2	104.8907535	6688
chr12.fa	7076500	7076769	SCARNA12	0	0
chr12.fa	7079944	7085165	EMG1	25.04234771	1206
chr12.fa	7085347	7125842	LPCAT3	5.886469531	608
chr12.fa	7167980	7178335	C1S	0	0
chr12.fa	7187515	7245043	C1R	0	0
chr12.fa	7247146	7261869	C1RL	2.62643867	381
chr12.fa	7261076	7274447	LOC283314	0.331362711	76
chr12.fa	7276280	7281466	RBP5	62.93200579	2660
chr12.fa	7282967	7311530	CLSTN3	2.53392398	456
chr12.fa	7341759	7371169	PEX5	8.585852476	1064
chr12.fa	7456928	7480969	ACSM4	0	0
chr12.fa	7507557	7596749	CD163L1	0	0
chr12.fa	7623410	7656414	CD163	0	0
chr12.fa	7801996	7818502	APOBEC1	0	0
chr12.fa	7842381	7848360	GDF3	0	0
chr12.fa	7864089	7870152	DPPA3	0	0
chr12.fa	7882011	7902069	CLEC4C	0	0
chr12.fa	7917812	7926717	NANOGNB	0	0
chr12.fa	7941995	7948655	NANOG	0	0
chr12.fa	7966397	8025495	SLC2A14	0	0
chr12.fa	8071824	8088892	SLC2A3	0	0
chr12.fa	8185359	8208118	FOXJ2	6.166237511	1520
chr12.fa	8210919	8218955	C3AR1	0	0
chr12.fa	8234807	8250373	NECAP1	21.51011017	2508
chr12.fa	8276228	8291203	CLEC4A	0	0
chr12.fa	8286365	8287448	POU5F1P3	0	0
chr12.fa	8325150	8332642	ZNF705A	0	0
chr12.fa	8332805	8353596	FAM66C	0	0
chr12.fa	8373856	8380214	FAM90A1	0	0
chr12.fa	8383645	8395542	LOC653113	0	0
chr12.fa	8509560	8543348	LOC389634	0	0
chr12.fa	8608591	8630926	CLEC6A	0	0
chr12.fa	8666136	8674960	CLEC4D	0	0
chr12.fa	8685901	8693558	CLEC4E	0	0

chr12.fa	8754762	8765442	AICDA	0	0
chr12.fa	8798540	8815433	MFAP5	0	0
chr12.fa	8850518	8929787	RIMKLB	1.739543038	456
chr12.fa	8975150	9029377	A2ML1	2.280398148	532
chr12.fa	9067316	9092963	PHC1	2.071795313	380
chr12.fa	9094061	9102252	M6PR	13.70062495	836
chr12.fa	9142221	9163340	KLRG1	1.890546582	152
chr12.fa	9208185	9217666	C12orf33	0	0
chr12.fa	9217773	9220651	LOC144571	0	0
chr12.fa	9220779	9268558	A2M	0	0
chr12.fa	9301436	9360966	PZP	0	0
chr12.fa	9392599	9395645	LOC100499405	0	0
chr12.fa	9436253	9466684	LOC642846	0	0
chr12.fa	9570287	9600768	DDX12	0	0
chr12.fa	9747870	9760497	KLRB1	0	0
chr12.fa	9800643	9811008	LOC374443	4.529661544	532
chr12.fa	9822308	9852151	CLEC2D	0.310902732	76
chr12.fa	9875091	9885860	CLECL1	0	0
chr12.fa	9905082	9913497	CD69	0	0
chr12.fa	9980077	9997603	KLRF1	0	0
chr12.fa	10004968	10022458	CLEC2B	0	0
chr12.fa	10034088	10048432	KLRF2	0	0
chr12.fa	10065826	10084980	CLEC2A	0	0
chr12.fa	10124008	10138194	CLEC12A	0	0
chr12.fa	10145662	10151899	CLEC1B	0	0
chr12.fa	10163231	10171399	CLEC12B	0	0
chr12.fa	10183276	10218565	CLEC9A	0	0
chr12.fa	10223080	10251605	CLEC1A	0	0
chr12.fa	10269376	10282868	CLEC7A	0	0
chr12.fa	10310899	10324790	OLR1	0.671843457	76
chr12.fa	10331557	10344403	C12orf59	9.616412746	1206
chr12.fa	10365489	10375724	GABARAPL1	132.1719122	11096
chr12.fa	10457050	10469850	KLRD1	0	0
chr12.fa	10524952	10562745	KLRK1	1.929465021	152
chr12.fa	10559983	10562356	KLRC4	0	0
chr12.fa	10564914	10573194	KLRC3	0	0
chr12.fa	10583206	10588592	KLRC2	0	0
chr12.fa	10598638	10607215	KLRC1	0	0
chr12.fa	10741077	10752434	KLRAP1	0.765470102	76
chr12.fa	10756364	10766208	MAGOHB	2.785448293	380
chr12.fa	10771538	10826891	STYK1	1.693063302	228
chr12.fa	10851676	10875953	CSDA	33.79810697	3000
chr12.fa	10954131	10955226	TAS2R7	0	0

chr12.fa	10958650	10959579	TAS2R8	0	0
chr12.fa	10961693	10962767	TAS2R9	0	0
chr12.fa	10977945	10978868	TAS2R10	0	0
chr12.fa	10998448	11002075	PRR4	2.878407765	73
chr12.fa	11033560	11187484	PRH1	0	0
chr12.fa	11060525	11062161	TAS2R13	0	0
chr12.fa	11081834	11087444	PRH2	0	0
chr12.fa	11090853	11091806	TAS2R14	0	0
chr12.fa	11138512	11139511	TAS2R50	0	0
chr12.fa	11148561	11150474	TAS2R20	0	0
chr12.fa	11174271	11175170	TAS2R19	0	0
chr12.fa	11182986	11184006	TAS2R31	0	0
chr12.fa	11213964	11214893	TAS2R46	0	0
chr12.fa	11243886	11244912	TAS2R43	0	0
chr12.fa	11285884	11286843	TAS2R30	0	0
chr12.fa	11323780	11325723	LOC100129361	16.38132705	1292
chr12.fa	11338599	11339543	TAS2R42	0	0
chr12.fa	11418857	11422641	PRB3	1.549176272	76
chr12.fa	11460015	11463366	PRB4	0	0
chr12.fa	11504757	11508524	PRB1	0	0
chr12.fa	11544474	11548498	PRB2	0	0
chr12.fa	11700964	11717335	LOC338817	0	0
chr12.fa	11802788	12048325	ETV6	19.22570898	5168
chr12.fa	12223878	12252627	BCL2L14	3.030300874	304
chr12.fa	12268961	12419811	LRP6	3.68768891	1672
chr12.fa	12482218	12503169	MANSC1	0.735892088	76
chr12.fa	12508342	12510001	LOH12CR2	2.197891056	152
chr12.fa	12510020	12619838	LOH12CR1	8.106377304	760
chr12.fa	12626216	12715448	DUSP16	71.22719313	20216
chr12.fa	12764831	12798041	CREBL2	38.33110438	6460
chr12.fa	12813995	12849121	GPR19	0	0
chr12.fa	12870302	12875305	CDKN1B	17.51107374	1900
chr12.fa	12878851	12944399	APOLD1	0.350265953	76
chr12.fa	12917583	12917677	MIR613	0	0
chr12.fa	12966280	12982915	DDX47	26.04555388	2128
chr12.fa	13028411	13029070	RPL13AP20	2.560833301	76
chr12.fa	13043956	13066600	GPRC5A	1.778461477	228
chr12.fa	13068763	13068852	MIR614	0	0
chr12.fa	13093709	13103318	GPRC5D	0	0
chr12.fa	13127799	13153243	HEBP1	34.97878122	1900
chr12.fa	13153376	13157764	HTR7P1	0.770140315	152
chr12.fa	13197315	13236383	KIAA1467	3.228896109	684
chr12.fa	13237042	13256619	GSG1	0	0

chr12.fa	13349602	13369708	EMP1	0.60267983	76
chr12.fa	13523605	13529679	C12orf36	1.055690463	152
chr12.fa	13714410	14133022	GRIN2B	0.284438193	76
chr12.fa	14518611	14651697	ATF7IP	22.08632546	4629
chr12.fa	14656597	14720791	PLBD1	4.374210178	380
chr12.fa	14765568	14849519	GUCY2C	1.759780626	304
chr12.fa	14923654	14924065	HIST4H4	127.1732279	2356
chr12.fa	14927270	14930936	H2AFJ	49.77868529	8208
chr12.fa	14939412	14956401	WBP11	24.01890395	2888
chr12.fa	14956506	14976791	C12orf60	0	0
chr12.fa	14957584	14967116	C12orf69	0.803276586	76
chr12.fa	14982245	14996413	ART4	0	0
chr12.fa	15034115	15038853	MGP	1.14753798	76
chr12.fa	15066976	15091463	ERP27	58.31983716	4028
chr12.fa	15094950	15114562	ARHGDIB	43.088272	2356
chr12.fa	15125956	15134799	PDE6H	0	0
chr12.fa	15260716	15374411	RERG	0	0
chr12.fa	15475487	15750335	PTPRO	0	0
chr12.fa	15773075	15942510	EPS8	14.07780023	2584
chr12.fa	16035288	16056410	STRAP	46.13591938	3952
chr12.fa	16064186	16190315	DERA	11.7644882	912
chr12.fa	16341419	16430619	SLC15A5	0	0
chr12.fa	16500076	16517344	MGST1	44.47821626	2280
chr12.fa	16701307	16761024	LMO3	0.870883474	152
chr12.fa	17141681	17143562	LOC728622	0	0
chr12.fa	18233803	18243114	RERGL	0	0
chr12.fa	18414474	18801352	PIK3C2G	1.395726426	304
chr12.fa	18836116	18890918	PLCZ1	0	0
chr12.fa	18891045	18892122	CAPZA3	0	0
chr12.fa	19282626	19529333	PLEKHA5	12.20615689	5016
chr12.fa	19592608	19675173	AEBP2	7.266183799	1666
chr12.fa	20522197	20833881	PDE3A	0.819733526	152
chr12.fa	20848289	20906320	SLCO1C1	0	0
chr12.fa	20963638	21069658	SLCO1B3	0	0
chr12.fa	21168630	21243040	LST-3TM12	0	0
chr12.fa	21284128	21392730	SLCO1B1	0	0
chr12.fa	21417534	21548371	SLCO1A2	0	0
chr12.fa	21525802	21532914	IAPP	0	0
chr12.fa	21590538	21624182	PYROXD1	5.288015131	608
chr12.fa	21624362	21654603	RECQL	4.584592141	456
chr12.fa	21654699	21671337	GOLT1B	2.582850018	380
chr12.fa	21679256	21684210	C12orf39	2.899090136	76
chr12.fa	21689123	21757781	GYS2	0	0

chr12.fa	21788275	21810789	LDHB	229.229607	16492
chr12.fa	21917889	21927747	KCNJ8	1.419744663	152
chr12.fa	21950324	22089628	ABCC9	0	0
chr12.fa	22199159	22218602	CMAS	42.71554455	3344
chr12.fa	22346325	22487648	ST8SIA1	0.173909826	76
chr12.fa	22601518	22697452	KIAA0528	14.19033012	2812
chr12.fa	22778076	22843608	ETNK1	4.164050606	1368
chr12.fa	23685231	24715380	SOX5	0.716988846	152
chr12.fa	24365355	24365429	MIR920	0	0
chr12.fa	24719898	24737102	C12orf67	0	0
chr12.fa	24962958	25102393	BCAT1	0.330473147	152
chr12.fa	25070636	25071266	DAD1P1	0	0
chr12.fa	25146365	25150373	C12orf77	0	0
chr12.fa	25205241	25261222	LRMP	1.22381812	152
chr12.fa	25261270	25348094	CASC1	0	0
chr12.fa	25348150	25357949	LYRM5	4.467392041	228
chr12.fa	25358180	25403854	KRAS	9.665338784	2356
chr12.fa	25629016	25801488	IFLTD1	0	0
chr12.fa	26026953	26027012	MIR4302	0	0
chr12.fa	26111964	26232825	RASSF8	8.369465954	2660
chr12.fa	26272959	26278003	BHLHE41	13.41396285	2280
chr12.fa	26348269	26387708	SSPN	2.145406761	456
chr12.fa	26488285	26986131	ITPR2	4.437814027	2508
chr12.fa	27058112	27091254	C12orf11	9.086899582	1216
chr12.fa	27091305	27119581	FGFR1OP2	6.979744087	988
chr12.fa	27124506	27167339	TM7SF3	8.951018631	1748
chr12.fa	27175483	27182682	MED21	12.53418373	988
chr12.fa	27233990	27235455	C12orf71	0	0
chr12.fa	27397078	27478890	STK38L	7.287978125	1670
chr12.fa	27485988	27573466	ARNTL2	0.875776078	76
chr12.fa	27619743	27655118	C12orf70	0	0
chr12.fa	27677045	27848497	PPFIBP1	9.601734935	2660
chr12.fa	27849428	27850566	REP15	0	0
chr12.fa	27863706	27909237	MRPS35	21.8757211	1866
chr12.fa	27915599	27924209	LOC100287284	0	0
chr12.fa	27933187	27955973	KLHDC5	5.225523237	1520
chr12.fa	28111017	28124916	PTHLH	0	0
chr12.fa	28410133	28703099	CCDC91	10.85757737	1140
chr12.fa	29376598	29487006	FAR2	2.420282138	228
chr12.fa	29493579	29534143	ERGIC2	12.26464574	980
chr12.fa	29580489	29650619	OVCH1	0	0
chr12.fa	29653746	29937692	TMTC1	0.183917424	76
chr12.fa	30781915	30848929	IPO8	13.06992385	3344

chr12.fa	30862486	30907448	CAPRIN2	4.145369755	836
chr12.fa	31079838	31149537	TSPAN11	0	0
chr12.fa	31226779	31257725	DDX11	0	0
chr12.fa	31433520	31479121	FAM60A	11.38353228	1596
chr12.fa	31477250	31478879	FLJ13224	0	0
chr12.fa	31535157	31743952	DENND5B	1.259845476	532
chr12.fa	31800094	31822016	C12orf72	1.419744663	152
chr12.fa	31824071	31882108	AMN1	4.005040983	380
chr12.fa	31944119	31945175	H3F3C	0	0
chr12.fa	32112353	32146043	C12orf35	4.881706626	1368
chr12.fa	32260185	32531141	BICD1	8.744862099	1368
chr12.fa	32655041	32798984	FGD4	9.552586506	3563
chr12.fa	32832137	32898584	DNM1L	20.65746276	4284
chr12.fa	32899477	32908874	YARS2	6.256973072	608
chr12.fa	32943680	33049780	PKP2	8.376582468	1672
chr12.fa	33528348	33592754	SYT10	0	0
chr12.fa	34175216	34181236	ALG10	1.081043046	152
chr12.fa	38710557	38723528	ALG10B	0.503271017	228
chr12.fa	39046002	39299420	CPNE8	0	0
chr12.fa	39687030	39837192	KIF21A	14.48344156	4325
chr12.fa	39945022	40013843	ABCD2	0	0
chr12.fa	40019972	40115720	C12orf40	0	0
chr12.fa	40148823	40499661	SLC2A13	0.482588646	152
chr12.fa	40618813	40763086	LRRK2	5.497062748	2280
chr12.fa	41086358	41464094	CNTN1	0	0
chr12.fa	41582250	41968392	PDZRN4	0	0
chr12.fa	42475648	42538673	GXYLT1	1.354361685	456
chr12.fa	42550907	42632151	YAF2	0.184806989	76
chr12.fa	42705888	42719932	ZCRB1	19.24817048	1596
chr12.fa	42719947	42842422	PPHLN1	10.90539145	2037
chr12.fa	42852140	42983572	PRICKLE1	1.056802418	228
chr12.fa	43748012	43945724	ADAMTS20	0	0
chr12.fa	44122412	44152596	PUS7L	3.459515661	684
chr12.fa	44152747	44183346	IRAK4	6.895680258	1346
chr12.fa	44187526	44200178	TWF1	16.65197699	2280
chr12.fa	44229962	44783541	TMEM117	0.626698067	76
chr12.fa	44902058	45307711	NELL2	0	0
chr12.fa	45408539	45444882	DBX2	0	0
chr12.fa	45456401	45459194	RACGAP1P	0	0
chr12.fa	45566817	45609769	PLEKHA9	0	0
chr12.fa	45580874	45581252	RNY5	0	0
chr12.fa	45609790	45834187	ANO6	3.346985774	1064
chr12.fa	46119503	46121704	LOC400027	12.28110268	1216

chr12.fa	46123620	46301819	ARID2	8.983932511	3420
chr12.fa	46312914	46384401	SRSF2IP	18.91324951	6460
chr12.fa	46576841	46663208	SLC38A1	9.960896531	3800
chr12.fa	46751971	46766645	SLC38A2	22.4941907	4940
chr12.fa	47158544	47219780	SLC38A4	1.272966549	228
chr12.fa	47469490	47473734	AMIGO2	0.42788044	76
chr12.fa	47602203	47610047	LOC100233209	0	0
chr12.fa	47610052	47630443	FAM113B	0	0
chr12.fa	48055715	48099844	RPAP3	8.251376289	1368
chr12.fa	48103518	48119355	ENDOU	0	0
chr12.fa	48128453	48152889	RAPGEF3	1.071702621	304
chr12.fa	48166967	48176506	SLC48A1	3.93854605	532
chr12.fa	48176537	48213763	HDAC7	9.129598669	1748
chr12.fa	48235320	48298814	VDR	2.831705638	608
chr12.fa	48357330	48362661	TMEM106C	23.31748248	1665
chr12.fa	48366748	48398285	COL2A1	0	0
chr12.fa	48436757	48499641	SENP1	5.006912804	1064
chr12.fa	48499656	48540187	PFKM	11.31614778	1900
chr12.fa	48541572	48551377	ASB8	19.40495619	2280
chr12.fa	48577366	48579709	C12orf68	0	0
chr12.fa	48596122	48597075	OR10AD1	0	0
chr12.fa	48722763	48724062	H1FNT	0	0
chr12.fa	48733793	48745029	ZNF641	2.525917901	532
chr12.fa	48866448	48866843	ANP32D	0	0
chr12.fa	48876286	48890297	C12orf54	0	0
chr12.fa	48919415	48921886	OR8S1	0	0
chr12.fa	48961467	48963829	LALBA	0	0
chr12.fa	49046995	49076035	C12orf41	11.33393907	1216
chr12.fa	49048165	49048226	SNORA34	0	0
chr12.fa	49048302	49048313	MIR1291	0	0
chr12.fa	49050431	49050565	SNORA2A	0	0
chr12.fa	49061240	49061376	SNORA2B	0	0
chr12.fa	49082245	49086229	LOC144438	11.02748416	1976
chr12.fa	49086752	49110781	CCNT1	9.872607272	1140
chr12.fa	49121218	49159568	LOC255411	0	0
chr12.fa	49159975	49182820	ADCY6	14.82236557	4027
chr12.fa	49212512	49222724	CACNB3	2.505902704	304
chr12.fa	49223539	49245957	DDX23	28.45404929	4180
chr12.fa	49250916	49259653	RND1	0	0
chr12.fa	49297893	49315359	CCDC65	0	0
chr12.fa	49315742	49319330	FKBP11	1.214255304	73
chr12.fa	49329992	49351252	ARF3	80.75754053	12844
chr12.fa	49359123	49365641	WNT10B	0	0

chr12.fa	49372236	49376396	WNT1	0	0
chr12.fa	49388933	49393088	DDN	0.451453895	76
chr12.fa	49396055	49412592	PRKAG1	34.57847728	2707
chr12.fa	49412758	49449107	MLL2	16.97222015	14820
chr12.fa	49458468	49463775	RHEBL1	0	0
chr12.fa	49483206	49488602	DHH	0	0
chr12.fa	49490923	49504680	LMBR1L	4.222094678	445
chr12.fa	49521567	49525304	TUBA1B	51.15884434	4028
chr12.fa	49578583	49582861	TUBA1A	12.18147148	912
chr12.fa	49658865	49667113	TUBA1C	35.91482528	2508
chr12.fa	49688909	49692481	PRPH	0	0
chr12.fa	49716971	49725514	TROAP	0	0
chr12.fa	49726200	49730971	C1QL4	0	0
chr12.fa	49741041	49745685	DNAJC22	1.850293796	152
chr12.fa	49760688	49921207	SPATS2	7.124075898	1216
chr12.fa	49782957	49786116	LOC100335030	0	0
chr12.fa	49932940	49952077	KCNH3	0	0
chr12.fa	49952086	49961911	MCRS1	18.45534627	1634
chr12.fa	49976658	49999433	FAM186B	0	0
chr12.fa	50017403	50035807	PRPF40B	3.577382934	388
chr12.fa	50031724	50101197	FMNL3	0	0
chr12.fa	50135293	50158717	TMBIM6	36.65205172	5531
chr12.fa	50184929	50222208	NCKAP5L	5.518857074	1216
chr12.fa	50222326	50234937	LOC100286844	0	0
chr12.fa	50229826	50236912	BCDIN3D	0.636705666	76
chr12.fa	50260680	50297720	FAIM2	0	0
chr12.fa	50302666	50305646	LOC283332	0	0
chr12.fa	50344524	50352664	AQP2	29.11988819	5472
chr12.fa	50355279	50359461	AQP5	0	0
chr12.fa	50366620	50370922	AQP6	2.547267445	304
chr12.fa	50382945	50419307	RACGAP1	1.466002008	228
chr12.fa	50451487	50477394	ACCN2	1.342130176	228
chr12.fa	50478983	50494494	SMARCD1	11.82964879	1824
chr12.fa	50497801	50505095	GPD1	8.227580444	1064
chr12.fa	50505900	50514234	C12orf62	64.6822236	1748
chr12.fa	50523581	50561097	LASS5	3.479530858	304
chr12.fa	50569563	50677353	LIMA1	31.89933192	5366
chr12.fa	50627925	50627995	MIR1293	0	0
chr12.fa	50721095	50790405	FAM186A	0	0
chr12.fa	50794592	50873788	LARP4	11.04683218	3262
chr12.fa	50898768	51142450	DIP2B	8.717063214	3416
chr12.fa	51157789	51214943	ATF1	6.072388475	684
chr12.fa	51236701	51281663	TMPRSS12	0	0

chr12.fa	51318534	51326300	METTL7A	9.472970499	1444
chr12.fa	51347782	51364289	HIGD1C	0	0
chr12.fa	51373566	51422058	SLC11A2	1.940806966	456
chr12.fa	51442084	51454207	LETMD1	22.19729861	2128
chr12.fa	51454990	51477333	CSRNP2	7.563965457	1520
chr12.fa	51487539	51566926	TFCP2	23.46626212	3920
chr12.fa	51580719	51611477	POU6F1	2.550825703	608
chr12.fa	51632508	51640501	DAZAP2	90.50116097	11248
chr12.fa	51639133	51664202	SMAGP	15.75574094	836
chr12.fa	51674822	51717938	BIN2	0	0
chr12.fa	51722227	51740463	CELA1	0	0
chr12.fa	51745833	51785200	GALNT6	0	0
chr12.fa	51818594	51902980	SLC4A8	0	0
chr12.fa	51985020	52202299	SCN8A	0.46902279	152
chr12.fa	52211676	52225701	FIGNL2	0.360051161	76
chr12.fa	52281793	52285505	ANKRD33	0	0
chr12.fa	52301202	52317145	ACVRL1	0	0
chr12.fa	52345451	52390863	ACVR1B	6.328805392	1368
chr12.fa	52400748	52409671	GRASP	0.874886514	76
chr12.fa	52445191	52453287	NR4A1	16.45849675	1976
chr12.fa	52463758	52471279	C12orf44	32.93300567	2128
chr12.fa	52562780	52585784	KRT80	5.146352012	893
chr12.fa	52604714	52617597	LOC283404	0	0
chr12.fa	52626954	52642709	KRT7	362.960036	27778
chr12.fa	52679697	52685299	KRT81	0	0
chr12.fa	52695649	52702947	KRT86	0	0
chr12.fa	52708085	52715182	KRT83	0	0
chr12.fa	52753790	52761309	KRT85	0	0
chr12.fa	52771596	52779417	KRT84	0	0
chr12.fa	52787735	52800176	KRT82	0	0
chr12.fa	52817854	52828110	KRT75	0	0
chr12.fa	52840435	52845910	KRT6B	0	0
chr12.fa	52862300	52867569	KRT6C	0	0
chr12.fa	52880958	52887181	KRT6A	0	0
chr12.fa	52908359	52914243	KRT5	0.733890568	76
chr12.fa	52937693	52946931	KRT71	0	0
chr12.fa	52959603	52967609	KRT74	0	0
chr12.fa	52979373	52995322	KRT72	0	0
chr12.fa	53001354	53012343	KRT73	0	0
chr12.fa	53038342	53045959	KRT2	0	0
chr12.fa	53068520	53074191	KRT1	0	0
chr12.fa	53083411	53097247	KRT77	0	0
chr12.fa	53161939	53171129	KRT76	0	0

chr12.fa	53183469	53189892	KRT3	0	0
chr12.fa	53200333	53208335	KRT4	2.62443715	304
chr12.fa	53215231	53228077	KRT79	0.818176788	76
chr12.fa	53232746	53242778	KRT78	0	0
chr12.fa	53290971	53298868	KRT8	330.5405313	26694
chr12.fa	53342655	53346685	KRT18	356.7528785	24624
chr12.fa	53400062	53435993	EIF4B	144.0793979	26096
chr12.fa	53436973	53447358	LOC283335	3.747512111	456
chr12.fa	53440810	53458099	TENC1	2.036657523	456
chr12.fa	53458163	53473204	SPRYD3	34.70346106	4477
chr12.fa	53491436	53496128	IGFBP6	0	0
chr12.fa	53497274	53518323	SOAT2	0	0
chr12.fa	53551447	53574430	CSAD	2.467429047	299
chr12.fa	53574535	53584654	ZNF740	12.70809356	2432
chr12.fa	53585107	53601000	ITGB7	1.219815081	152
chr12.fa	53604353	53626036	RARG	17.03204335	2584
chr12.fa	53645370	53648190	MFSD5	3.813562262	380
chr12.fa	53662083	53687427	ESPL1	0.765692493	228
chr12.fa	53689235	53693234	PFDN5	422.9962875	13067
chr12.fa	53693470	53700965	C12orf10	20.86628799	1140
chr12.fa	53701240	53715412	AAAS	11.04082762	912
chr12.fa	53720360	53730004	SP7	0	0
chr12.fa	53773979	53810226	SP1	19.76033714	6840
chr12.fa	53817639	53825318	AMHR2	0	0
chr12.fa	53835433	53840427	PRR13	62.23236345	3344
chr12.fa	53845886	53874275	PCBP2	238.4477174	26805
chr12.fa	53874947	53893444	MAP3K12	0	0
chr12.fa	53894705	53900215	TARBP2	7.651587543	684
chr12.fa	53900474	53901422	NPFF	0	0
chr12.fa	53905843	54020199	ATF7	11.55788689	3493
chr12.fa	54058944	54070512	ATP5G2	233.3983279	13507
chr12.fa	54104902	54121307	CALCOCO1	22.75016283	3116
chr12.fa	54332576	54340328	HOXC13	0	0
chr12.fa	54348714	54350350	HOXC12	1.99084496	76
chr12.fa	54356096	54362515	HOTAIR	0	0
chr12.fa	54366910	54370203	HOXC11	0	0
chr12.fa	54378946	54384062	HOXC10	10.3531944	912
chr12.fa	54385522	54385631	MIR196A2	0	0
chr12.fa	54393877	54397120	HOXC9	0	0
chr12.fa	54402890	54406545	HOXC8	0	0
chr12.fa	54422194	54424607	HOXC6	1.005430079	76
chr12.fa	54426832	54429144	HOXC5	1.048573948	76
chr12.fa	54427734	54427829	MIR615	0	0

chr12.fa	54446944	54449814	HOXC4	0	0
chr12.fa	54452038	54516018	FLJ12825	0	0
chr12.fa	54472623	54475607	LOC100240735	0	0
chr12.fa	54495993	54496483	LOC100240734	0	0
chr12.fa	54519855	54526626	LOC400043	0	0
chr12.fa	54575252	54582757	SMUG1	12.91847552	912
chr12.fa	54624731	54673915	CBX5	17.37608236	9272
chr12.fa	54674488	54677018	HNRNPA1	20.02631687	181
chr12.fa	54685896	54694791	NFE2	0	0
chr12.fa	54718911	54745633	COPZ1	107.6081501	9179
chr12.fa	54731000	54731098	MIR148B	0	0
chr12.fa	54756229	54758270	GPR84	0	0
chr12.fa	54762920	54785083	ZNF385A	15.19420346	1596
chr12.fa	54789045	54813050	ITGA5	0	0
chr12.fa	54849736	54867386	GTSF1	0	0
chr12.fa	54891495	54936899	NCKAP1L	0	0
chr12.fa	54943177	54973023	PDE1B	0	0
chr12.fa	54973024	54982443	PPP1R1A	4.607943205	380
chr12.fa	55001971	55004246	GLYCAM1	0	0
chr12.fa	55024623	55028663	LACRT	0	0
chr12.fa	55038375	55042149	DCD	0	0
chr12.fa	55248299	55252174	MUCL1	0	0
chr12.fa	55343865	55378456	KIAA0748	0	0
chr12.fa	55413729	55423801	NEUROD4	0.427213267	76
chr12.fa	55523553	55524560	OR9K2	0	0
chr12.fa	55614809	55615759	OR10A7	0	0
chr12.fa	55641072	55642010	OR6C74	0	0
chr12.fa	55688072	55689016	OR6C6	0	0
chr12.fa	55714384	55715322	OR6C1	0	0
chr12.fa	55725485	55726420	OR6C3	0	0
chr12.fa	55758895	55759833	OR6C75	0	0
chr12.fa	55794313	55795251	OR6C65	0	0
chr12.fa	55820038	55820976	OR6C76	0	0
chr12.fa	55845998	55846936	OR6C2	0	0
chr12.fa	55862984	55863922	OR6C70	0	0
chr12.fa	55886147	55887100	OR6C68	0	0
chr12.fa	55945011	55945940	OR6C4	0	0
chr12.fa	56030676	56031617	OR10P1	0	0
chr12.fa	56075330	56078353	METTL7B	0	0
chr12.fa	56078395	56106089	ITGA7	0.386515699	76
chr12.fa	56110076	56113491	BLOC1S1	33.69135925	962
chr12.fa	56114151	56115278	RDH5	0	0
chr12.fa	56119230	56122910	CD63	56.50534833	2584

chr12.fa	56137064	56146665	GDF11	2.176986295	380
chr12.fa	56151054	56211540	SARNP	59.87813147	2504
chr12.fa	56211806	56214743	ORMDL2	6.047035892	304
chr12.fa	56214960	56223391	DNAJC14	9.313738485	1292
chr12.fa	56223421	56229213	LOC440104	10.18173088	1216
chr12.fa	56230031	56236735	MMP19	0	0
chr12.fa	56295197	56321697	WIBG	25.16710911	1444
chr12.fa	56324946	56347807	DGKA	16.73826473	2200
chr12.fa	56347889	56359826	SILV	0.795048116	76
chr12.fa	56360556	56366568	CDK2	10.31939095	1064
chr12.fa	56367862	56388488	RAB5B	109.8047068	16264
chr12.fa	56391043	56399309	SUOX	19.22215072	2128
chr12.fa	56414689	56432219	IKZF4	1.860301395	456
chr12.fa	56435686	56438007	RPS26	12.39118627	380
chr12.fa	56473892	56497128	ERBB3	26.97336947	7213
chr12.fa	56498103	56507694	PA2G4	41.25999492	4872
chr12.fa	56510374	56511616	RPL41	116.5649509	3061
chr12.fa	56512030	56516278	ZC3H10	2.390704124	228
chr12.fa	56521986	56538460	ESYT1	4.248114435	817
chr12.fa	56546204	56551771	MYL6B	25.06080617	1199
chr12.fa	56552045	56555366	MYL6	603.1499651	21941
chr12.fa	56555636	56583351	SMARCC2	30.54697175	7420
chr12.fa	56598286	56615735	RNF41	29.95363235	4636
chr12.fa	56618125	56623638	OBFC2B	14.47676983	912
chr12.fa	56623820	56631590	SLC39A5	8.169091589	760
chr12.fa	56631630	56652143	ANKRD52	19.81059752	7676
chr12.fa	56660642	56664750	COQ10A	6.55475473	532
chr12.fa	56665483	56694175	CS	65.31247993	8740
chr12.fa	56704213	56710006	CNPY2	11.45113917	760
chr12.fa	56710129	56727837	PAN2	8.075020162	1976
chr12.fa	56732663	56734194	IL23A	0	0
chr12.fa	56735382	56754037	STAT2	21.16584877	4319
chr12.fa	56754355	56756583	APOF	1.000759866	76
chr12.fa	56810157	56843200	TIMELESS	2.631553665	608
chr12.fa	56843286	56848435	MIP	0	0
chr12.fa	56862301	56864735	SPRYD4	11.41466703	1064
chr12.fa	56864768	56882181	GLS2	0	0
chr12.fa	56915609	56989379	RBMS2	6.645045509	2356
chr12.fa	56989981	57030163	BAZ2A	37.89966568	14201
chr12.fa	57031959	57039852	ATP5B	263.2009567	21812
chr12.fa	57037464	57037538	SNORD59B	0	0
chr12.fa	57038811	57038885	SNORD59A	0	0
chr12.fa	57057125	57082078	PTGES3	68.01742265	5860

chr12.fa	57106211	57119326	NACA	94.83778706	29557
chr12.fa	57125364	57146146	PRIM1	2.312200072	152
chr12.fa	57157108	57181574	HSD17B6	3.353435115	228
chr12.fa	57316938	57328189	SDR9C7	0	0
chr12.fa	57345216	57351418	RDH16	0.98096706	76
chr12.fa	57388355	57390469	GPR182	0	0
chr12.fa	57392617	57400297	ZBTB39	5.419003478	1520
chr12.fa	57403781	57410344	TAC3	5.309364675	228
chr12.fa	57422301	57443893	MYO1A	0	0
chr12.fa	57449426	57472574	TMEM194A	0.300895133	76
chr12.fa	57482677	57489186	NAB2	5.125669642	608
chr12.fa	57489260	57505196	STAT6	51.94966703	9797
chr12.fa	57522282	57607125	LRP1	1.248725921	836
chr12.fa	57588287	57588359	MIR1228	0	0
chr12.fa	57610578	57620232	NXPH4	1.873867251	152
chr12.fa	57623356	57628685	SHMT2	8.980819036	1140
chr12.fa	57628719	57634475	NDUFA4L2	0	0
chr12.fa	57637242	57644969	STAC3	2.057339893	152
chr12.fa	57647548	57704246	R3HDM2	48.0429229	8585
chr12.fa	57828543	57844609	INHBC	0	0
chr12.fa	57849096	57851791	INHBE	0	0
chr12.fa	57853918	57866037	GLI1	0	0
chr12.fa	57866048	57873633	ARHGAP9	0	0
chr12.fa	57881851	57910370	MARS	19.17833968	2356
chr12.fa	57910434	57914300	DDIT3	11.95885801	456
chr12.fa	57916659	57923931	MBD6	12.21816601	2280
chr12.fa	57924094	57940978	DCTN2	64.94286595	5014
chr12.fa	57943847	57978554	KIF5A	0	0
chr12.fa	57984942	57997211	PIP4K2C	13.65169892	1976
chr12.fa	57998604	58003583	DTX3	6.664171142	608
chr12.fa	58003963	58011026	ARHGEF25	0	0
chr12.fa	58013693	58019550	SLC26A10	0	0
chr12.fa	58019935	58026985	B4GALNT1	0	0
chr12.fa	58087738	58115340	OS9	13.59699071	1748
chr12.fa	58118994	58135940	AGAP2	0.395856125	76
chr12.fa	58120748	58122100	LOC100130776	2.665801892	76
chr12.fa	58138784	58142002	TSPAN31	8.864508501	684
chr12.fa	58142027	58146164	CDK4	34.54200514	2204
chr12.fa	58148900	58153689	9-Mar	2.746085072	304
chr12.fa	58156117	58160976	CYP27B1	2.718508578	304
chr12.fa	58162351	58165914	METTL1	7.759002435	456
chr12.fa	58166383	58176324	FAM119B	12.4977116	1596
chr12.fa	58176528	58196639	TSFM	5.394985241	532

chr12.fa	58191371	58209852	AVIL	0.607350043	76
chr12.fa	58213710	58240747	CTDSP2	43.1756717	9746
chr12.fa	58218392	58218475	MIR26A2	0	0
chr12.fa	58335445	58351052	XRCC6BP1	5.463926476	285
chr12.fa	59265937	59314262	LRIG3	6.527400627	1216
chr12.fa	60083126	60175408	SLC16A7	0.954724912	152
chr12.fa	62102041	62586551	FAM19A2	0	0
chr12.fa	62654187	62799898	USP15	7.683167077	1593
chr12.fa	62860597	62991363	MON2	8.956578408	4180
chr12.fa	62995531	62997214	C12orf61	0	0
chr12.fa	62997466	62997549	MIRLET7I	0	0
chr12.fa	63037763	63328665	PPM1H	28.35130461	7904
chr12.fa	63540216	63546590	AVPR1A	0	0
chr12.fa	63952693	64062354	DPY19L2	0.416538495	76
chr12.fa	64173637	64202887	TMEM5	4.659315544	304
chr12.fa	64238541	64541613	SRGAP1	3.988584043	1520
chr12.fa	64586419	64616076	C12orf66	1.950147392	228
chr12.fa	64660763	64784345	C12orf56	0	0
chr12.fa	64798153	64842463	XPOT	21.7262743	3856
chr12.fa	64845840	64895899	TBK1	12.04536814	1672
chr12.fa	65004293	65089192	RASSF3	50.56617211	3040
chr12.fa	65107222	65153226	GNS	3.625641798	836
chr12.fa	65218352	65274798	TBC1D30	7.327786129	2578
chr12.fa	65277554	65371302	FLJ41278	0	0
chr12.fa	65444404	65515346	WIF1	0	0
chr12.fa	65563351	65642141	LEMD3	4.931744619	1064
chr12.fa	65672423	65860687	MSRB3	0	0
chr12.fa	66151801	66220754	RPSAP52	0	0
chr12.fa	66218240	66360071	HMGA2	0	0
chr12.fa	66516849	66524533	LLPH	16.62907071	910
chr12.fa	66530717	66563807	TMBIM4	5.35161898	437
chr12.fa	66582978	66648394	IRAK3	0	0
chr12.fa	66696356	66731958	HELB	1.50558762	228
chr12.fa	66741211	67072925	GRIP1	5.010471062	1140
chr12.fa	67663061	67708388	CAND1	24.03091307	6384
chr12.fa	68042512	68056444	DYRK2	10.1537096	2812
chr12.fa	68548550	68553521	IFNG	0	0
chr12.fa	68595129	68619571	IL26	0	0
chr12.fa	68642025	68647281	IL22	0	0
chr12.fa	68688346	68726161	MDM1	3.014066325	608
chr12.fa	69004652	69054374	RAP1B	10.86091324	1029
chr12.fa	69021014	69021155	SNORA70G	0	0
chr12.fa	69080731	69136473	NUP107	9.525010012	1338

chr12.fa	69139936	69159853	SLC35E3	2.160529355	228
chr12.fa	69201971	69239212	MDM2	22.18773579	7347
chr12.fa	69244956	69357020	CPM	8.292518639	2508
chr12.fa	69633317	69668138	CPSF6	9.541689343	2812
chr12.fa	69742134	69748013	LYZ	0	0
chr12.fa	69753532	69784576	YEATS4	6.101744098	380
chr12.fa	69864129	69973562	FRS2	8.735076891	2720
chr12.fa	69978502	69978603	MIR3913-1	0	0
chr12.fa	69979208	69995357	CCT2	42.21716614	4332
chr12.fa	70002345	70004942	LRRC10	0	0
chr12.fa	70047389	70093196	BEST3	0	0
chr12.fa	70132631	70216984	RAB3IP	6.743787149	3040
chr12.fa	70636774	70748773	CNOT2	15.19353628	2812
chr12.fa	70760062	70824978	KCNMB4	3.108804925	228
chr12.fa	70910632	71031219	PTPRB	0	0
chr12.fa	71031862	71314584	PTPRR	0	0
chr12.fa	71518877	71551779	TSPAN8	34.40167637	1748
chr12.fa	71833813	71978621	LGR5	0	0
chr12.fa	72003379	72057676	ZFC3H1	9.780759756	3116
chr12.fa	72057750	72074428	THAP2	0.354491384	76
chr12.fa	72079878	72097839	TMEM19	2.177208686	456
chr12.fa	72148658	72181150	RAB21	16.07620648	1899
chr12.fa	72233487	72320629	TBC1D15	7.176337802	1900
chr12.fa	72242074	72244763	MRS2P2	0	0
chr12.fa	72332626	72426221	TPH2	0	0
chr12.fa	72647287	72665942	LOC283392	0	0
chr12.fa	72666529	73059422	TRHDE	0.703200599	152
chr12.fa	74931551	74935232	ATXN7L3B	61.05191159	10108
chr12.fa	75433896	75603511	KCNC2	0	0
chr12.fa	75669759	75723836	CAPS2	1.583202108	152
chr12.fa	75728463	75764169	GLIPR1L1	0	0
chr12.fa	75784889	75817820	GLIPR1L2	0	0
chr12.fa	75874513	75895702	GLIPR1	1.278526326	152
chr12.fa	75891419	75905418	KRR1	18.56454029	1748
chr12.fa	76419227	76425556	PHLDA1	1.715080019	456
chr12.fa	76438672	76478738	NAP1L1	33.25658469	7664
chr12.fa	76738266	76742222	BBS10	1.886988325	304
chr12.fa	76745578	76953589	OSBPL8	7.450990788	2426
chr12.fa	77157854	77247474	ZDHHC17	1.771344963	380
chr12.fa	77252496	77272799	CSRP2	24.38651641	988
chr12.fa	77415026	77459360	E2F7	0	0
chr12.fa	78225069	78606790	NAV3	0	0
chr12.fa	79257773	79845788	SYT1	0.634481755	152

chr12.fa	79813037	79813101	MIR1252	0	0
chr12.fa	79985745	80084790	PAWR	7.796808919	684
chr12.fa	80167343	80329235	PPP1R12A	16.776516	4484
chr12.fa	80603233	80772870	C12orf64	0.62736524	228
chr12.fa	80838126	81073968	PTPRQ	0.223947819	76
chr12.fa	81101408	81103256	MYF6	0	0
chr12.fa	81110708	81113447	MYF5	0	0
chr12.fa	81191171	81331694	LIN7A	6.870550066	380
chr12.fa	81226312	81226408	MIR617	0	0
chr12.fa	81329515	81329612	MIR618	0	0
chr12.fa	81471809	81649582	ACSS3	9.473415281	1292
chr12.fa	81653356	82153109	PPFIA2	0	0
chr12.fa	82746083	82752199	CCDC59	6.271428493	456
chr12.fa	82752276	82873016	C12orf26	6.874108323	608
chr12.fa	83080934	83528067	TMTC2	10.12279724	2204
chr12.fa	85253267	85306606	SLC6A15	0	0
chr12.fa	85408094	85430055	TSPAN19	0	0
chr12.fa	85430099	85638883	LRRIQ1	0.620915899	152
chr12.fa	85674036	85695561	ALX1	0	0
chr12.fa	86198331	86230318	RASSF9	0.941159056	76
chr12.fa	86268073	86276770	NTS	0	0
chr12.fa	86373037	87232681	MGAT4C	0	0
chr12.fa	88176663	88178488	MKRN9P	0	0
chr12.fa	88373816	88423176	C12orf50	0	0
chr12.fa	88429268	88443937	C12orf29	6.837191403	760
chr12.fa	88444131	88535993	CEP290	4.47539812	1520
chr12.fa	88536073	88593664	TMTC3	1.407957936	456
chr12.fa	88886570	88974250	KITLG	3.726162567	912
chr12.fa	89741837	89746296	DUSP6	6.592783605	836
chr12.fa	89813498	89919344	POC1B	4.856798824	684
chr12.fa	89917451	89918583	GALNT4	4.47539812	228
chr12.fa	89981826	90049844	ATP2B1	4.144480191	1292
chr12.fa	90102732	90105729	LOC338758	1.691284173	228
chr12.fa	91345992	91348953	C12orf12	0	0
chr12.fa	91357456	91398803	EPYC	0	0
chr12.fa	91444271	91452131	KERA	0	0
chr12.fa	91497232	91505542	LUM	0.798828764	76
chr12.fa	91539035	91576806	DCN	0	0
chr12.fa	92534054	92539673	BTG1	21.6688974	4560
chr12.fa	92813870	92821924	CLLU1OS	0	0
chr12.fa	92815307	92824778	CLLU1	0	0
chr12.fa	93096619	93102325	C12orf74	1.819381436	152
chr12.fa	93130265	93165868	PLEKHG7	0	0

chr12.fa	93166285	93323107	EEA1	25.31255287	9109
chr12.fa	93771701	93772054	NUDT4	0	0
chr12.fa	93802088	93836026	UBE2N	41.72456989	4788
chr12.fa	93861270	93896432	MRPL42	14.89864571	1368
chr12.fa	93963598	93969978	SOCS2	39.00405979	3876
chr12.fa	94071151	94244531	CRADD	18.49515428	988
chr12.fa	94542499	94701451	PLXNC1	0	0
chr12.fa	94702056	94853764	CCDC41	3.668563277	532
chr12.fa	94853779	94856344	LOC144486	0	0
chr12.fa	94960900	95044324	TMCC3	4.035953343	1064
chr12.fa	95228174	95228229	MIR492	0	0
chr12.fa	95228290	95228804	KRT19P2	0	0
chr12.fa	95365110	95397511	NDUFA12	59.25476927	1596
chr12.fa	95414058	95467404	NR2C1	1.131748213	228
chr12.fa	95470525	95611240	FGD6	2.911544036	1216
chr12.fa	95611522	95696566	VEZT	6.668841355	1368
chr12.fa	95702196	95702289	MIR331	0	0
chr12.fa	95703699	95703760	MIR3685	0	0
chr12.fa	95867822	95909613	METAP2	18.32947292	2888
chr12.fa	95910888	95945263	USP44	0.47725126	76
chr12.fa	96051583	96184536	NTN4	5.143016146	836
chr12.fa	96252709	96260238	SNRPF	14.91955047	532
chr12.fa	96260826	96336428	CCDC38	0	0
chr12.fa	96337071	96362370	AMDHD1	0.791267467	76
chr12.fa	96367142	96390071	HAL	0	0
chr12.fa	96394611	96429365	LTA4H	38.29218594	3547
chr12.fa	96588207	96661606	ELK3	1.57141538	152
chr12.fa	96672039	96794366	CDK17	6.662836796	1412
chr12.fa	97301001	97347469	NEDD1	4.949313514	912
chr12.fa	97858799	97927544	RMST	0	0
chr12.fa	97885687	97885756	MIR1251	0	0
chr12.fa	97957590	97957689	MIR135A2	0	0
chr12.fa	98389161	98389226	MIR4303	0	0
chr12.fa	98847619	98850923	LOC121456	0.511499487	76
chr12.fa	98906751	98910004	LOC100128191	0	0
chr12.fa	98909676	98944157	TMPO	4.671769445	1368
chr12.fa	98987403	98995778	SLC25A3	193.32857	17708
chr12.fa	98993413	98993662	SNORA53	0	0
chr12.fa	99007182	99038589	IKBIP	0	0
chr12.fa	99039078	99129070	APAF1	5.539984226	1748
chr12.fa	99129212	100378432	ANKS1B	0.68896757	195
chr12.fa	100041535	100043826	FAM71C	0	0
chr12.fa	100430863	100536642	UHRF1BP1L	10.56935853	2584

chr12.fa	100550175	100567121	GOLGA2B	1.129969084	228
chr12.fa	100583662	100583727	MIR1827	0	0
chr12.fa	100594574	100618201	ACTR6	3.85225831	304
chr12.fa	100646042	100660857	DEPDC4	0	0
chr12.fa	100661549	100733914	SCYL2	18.21694303	3116
chr12.fa	100750857	100815837	SLC17A8	0	0
chr12.fa	100867679	100957643	NR1H4	0.788598774	76
chr12.fa	100967489	101018685	GAS2L3	0.72610688	76
chr12.fa	101188374	101522419	ANO4	0	0
chr12.fa	101549994	101604016	SLC5A8	0.514390571	76
chr12.fa	101673905	101780397	UTP20	3.939435614	1596
chr12.fa	101786903	101801572	ARL1	42.12264993	6080
chr12.fa	101871335	101880775	SPIC	0	0
chr12.fa	101988747	102079658	MYBPC1	13.55518119	2432
chr12.fa	102091417	102122846	CHPT1	13.6396898	912
chr12.fa	102122426	102133250	SYCP3	0	0
chr12.fa	102139275	102224645	GNPTAB	7.205026252	1824
chr12.fa	102271105	102317401	DRAM1	1.431086608	228
chr12.fa	102406717	102455899	CCDC53	20.21735081	830
chr12.fa	102467973	102512361	NUP37	2.8052411	152
chr12.fa	102513956	102591298	C12orf48	0.640486314	76
chr12.fa	102591300	102591614	PMCH	0	0
chr12.fa	102789645	102874378	IGF1	0	0
chr12.fa	103203061	103218177	LOC283432	0	0
chr12.fa	103232104	103311381	PAH	9.431605758	1137
chr12.fa	103351452	103354294	ASCL1	0	0
chr12.fa	103695733	103889749	C12orf42	0	0
chr12.fa	103981069	104160502	STAB2	0.205266968	76
chr12.fa	104166081	104234975	NT5DC3	3.983024266	1292
chr12.fa	104237527	104323989	GNN	0	0
chr12.fa	104324189	104341703	HSP90B1	28.30882791	3372
chr12.fa	104343981	104350993	C12orf73	0.998313564	76
chr12.fa	104359593	104382656	TDG	5.731240556	836
chr12.fa	104382765	104443915	GLT8D2	0	0
chr12.fa	104458236	104500304	HCFC2	4.715358097	1216
chr12.fa	104510858	104532040	NFYB	4.854130132	760
chr12.fa	104609559	104744062	TXNRD1	15.5720459	2964
chr12.fa	104697549	104698982	EID3	0	0
chr12.fa	104850692	105155792	CHST11	0.293778618	76
chr12.fa	104985411	104985494	MIR3922	0	0
chr12.fa	105197275	105322472	SLC41A2	1.907670695	304
chr12.fa	105380098	105388505	C12orf45	13.58653833	380
chr12.fa	105413562	105478341	ALDH1L2	0.89668084	304

chr12.fa	105501492	105562906	KIAA1033	6.695528285	1748
chr12.fa	105567075	105630008	APPL2	28.98667593	4210
chr12.fa	105724414	105765296	C12orf75	14.84771815	912
chr12.fa	106457125	106533811	NUAK1	0	0
chr12.fa	106631659	106641713	CKAP4	2.783446774	380
chr12.fa	106696581	106740792	TCP11L2	9.906410716	988
chr12.fa	106751436	106903976	POLR3B	8.806909211	1748
chr12.fa	106977033	107156581	RFX4	0	0
chr12.fa	107168399	107283094	RIC8B	3.048981725	684
chr12.fa	107349544	107367813	C12orf23	16.05396738	2432
chr12.fa	107371069	107380929	MTERFD3	7.660927969	608
chr12.fa	107385143	107487598	CRY1	5.708334275	836
chr12.fa	107712197	108053419	BTBD11	5.432791725	1444
chr12.fa	108079590	108106257	PWP1	26.45163999	2204
chr12.fa	108126643	108154914	PRDM4	17.7614861	3344
chr12.fa	108168162	108170421	ASCL4	0	0
chr12.fa	108296927	108297548	LOC728739	0	0
chr12.fa	108523511	108644313	WSCD2	6.563872765	1368
chr12.fa	108681821	108733094	CMKLR1	0	0
chr12.fa	108909051	108913380	FICD	1.023666147	76
chr12.fa	108915991	108955165	SART3	18.80917048	3696
chr12.fa	108956294	108963160	ISCU	83.57212205	4408
chr12.fa	108983622	108991894	TMEM119	0	0
chr12.fa	109015680	109027670	SELPLG	0	0
chr12.fa	109038887	109125295	CORO1C	9.751848915	1672
chr12.fa	109176466	109251359	SSH1	11.21762853	4636
chr12.fa	109273857	109294710	DAO	6.353935584	456
chr12.fa	109304658	109458845	SVOP	0	0
chr12.fa	109490380	109525831	USP30	0.900683879	152
chr12.fa	109525993	109531293	ALKBH2	5.648066292	304
chr12.fa	109535399	109548798	UNG	12.85842993	1368
chr12.fa	109577202	109706030	ACACB	5.482162545	2280
chr12.fa	109715783	109747025	FOXN4	0	0
chr12.fa	109826524	109886176	MYO1H	0	0
chr12.fa	109886460	109915155	KCTD10	17.88091011	3192
chr12.fa	109915439	109974507	UBE3B	14.42962292	3494
chr12.fa	109991521	110011358	MMAB	20.90631838	3890
chr12.fa	110011500	110035071	MVK	12.22995273	1140
chr12.fa	110152187	110208312	C12orf34	0.538186417	76
chr12.fa	110171130	110211289	MGC14436	0	0
chr12.fa	110220892	110271212	TRPV4	0.521729477	76
chr12.fa	110288748	110318293	GLTP	50.12294669	5310
chr12.fa	110338079	110355874	TCHP	11.23386308	1672

chr12.fa	110367607	110434194	GIT2	10.01671669	2888
chr12.fa	110437235	110477235	ANKRD13A	12.97474047	2280
chr12.fa	110478983	110505500	C12orf76	0.868437172	76
chr12.fa	110562140	110656600	IFT81	5.113882914	760
chr12.fa	110719032	110788897	ATP2A2	10.11612551	1976
chr12.fa	110810705	110841535	ANAPC7	13.91478757	2280
chr12.fa	110872706	110888158	ARPC3	110.893756	4408
chr12.fa	110890291	110906089	GPN3	13.39639396	912
chr12.fa	110906232	110928192	C12orf24	2.659574941	152
chr12.fa	110929330	110939916	VPS29	40.76161651	2018
chr12.fa	110940005	110969891	RAD9B	0.89668084	76
chr12.fa	110972237	111021064	PPTC7	7.215256242	1216
chr12.fa	111051832	111085698	TCTN1	5.463704085	526
chr12.fa	111086491	111127617	HVCN1	0.988305965	76
chr12.fa	111157615	111180757	PPP1CC	49.69084081	5548
chr12.fa	111284811	111345338	CCDC63	0	0
chr12.fa	111348624	111358404	MYL2	0	0
chr12.fa	111374406	111375250	LOC100131138	0	0
chr12.fa	111471828	111788358	CUX2	13.06925667	4022
chr12.fa	111798455	111806925	FAM109A	0.519060784	76
chr12.fa	111843752	111889427	SH2B3	0.938045581	228
chr12.fa	111890018	112037480	ATXN2	25.81004172	5457
chr12.fa	112079950	112123790	BRAP	14.27372677	2584
chr12.fa	112123857	112194911	ACAD10	5.761485743	1064
chr12.fa	112204346	112247784	ALDH2	48.59245126	5244
chr12.fa	112277573	112280706	C12orf47	2.547934618	228
chr12.fa	112303054	112331228	MAPKAPK5	29.957413	2356
chr12.fa	112336867	112339706	ADAM1	0	0
chr12.fa	112369087	112451023	TMEM116	6.32635909	456
chr12.fa	112451152	112461024	ERP29	63.15172818	4180
chr12.fa	112464493	112546635	NAA25	7.842399091	2052
chr12.fa	112563349	112591408	TRAFD1	32.96347324	4712
chr12.fa	112597992	112819896	C12orf51	11.35150796	7828
chr12.fa	112842994	112847443	RPL6	348.5046157	17708
chr12.fa	112856536	112947717	PTPN11	31.7427686	8968
chr12.fa	113229549	113336684	RPH3A	0	0
chr12.fa	113344739	113357712	OAS1	37.60455272	2812
chr12.fa	113376249	113411054	OAS3	6.631034871	1976
chr12.fa	113416274	113449528	OAS2	8.320539916	1748
chr12.fa	113495662	113535833	DTX1	0.513945789	76
chr12.fa	113536624	113574044	RASAL1	1.318111939	228
chr12.fa	113587663	113597081	CCDC42B	0	0
chr12.fa	113594978	113623284	DDX54	13.40084178	2338

chr12.fa	113623555	113630119	C12orf52	6.665505489	532
chr12.fa	113633246	113658880	IQCD	1.252061788	76
chr12.fa	113659260	113736389	TPCN1	6.303008026	1516
chr12.fa	113736571	113772925	SLC24A6	1.703738074	228
chr12.fa	113796371	113827458	PLBD2	1.193572933	152
chr12.fa	113830251	113841692	SDS	0	0
chr12.fa	113860216	113876081	SDSL	12.0985196	760
chr12.fa	113900694	113909877	LHX5	0	0
chr12.fa	114254543	114404176	RBM19	2.69426795	684
chr12.fa	114791735	114846247	TBX5	0	0
chr12.fa	115108059	115121969	TBX3	101.7196791	21877
chr12.fa	116396381	116714991	MED13L	21.98780621	9272
chr12.fa	116586365	116586459	MIR620	0	0
chr12.fa	116971227	116974318	NCRNA00173	0	0
chr12.fa	116997186	117014425	MAP1LC3B2	0	0
chr12.fa	117153596	117175843	C12orf49	10.35430635	1292
chr12.fa	117176096	117291436	RNFT2	0	0
chr12.fa	117299027	117319232	HRK	0	0
chr12.fa	117348761	117468953	FBXW8	3.266480202	684
chr12.fa	117476728	117537251	TESC	33.10758266	1517
chr12.fa	117581585	117628300	FBXO21	25.68172207	4864
chr12.fa	117648777	117799607	NOS1	0	0
chr12.fa	117890817	118406028	KSR2	13.79225008	10548
chr12.fa	118454508	118470039	RFC5	6.163568818	684
chr12.fa	118470492	118498951	WSB2	58.69478852	7448
chr12.fa	118501398	118541810	VSIG10	6.41887378	1444
chr12.fa	118573870	118583390	PEBP1	452.2996483	30324
chr12.fa	118587606	118810750	TAOK3	30.07149962	5928
chr12.fa	118814358	118855840	SUDS3	23.40554935	4976
chr12.fa	119419396	119600857	SRRM4	0.201708711	76
chr12.fa	119616595	119632551	HSPB8	52.28747908	4707
chr12.fa	119721630	119741185	LOC144742	0	0
chr12.fa	119772517	119978852	CCDC60	0	0
chr12.fa	120031264	120079363	TMEM233	0	0
chr12.fa	120105761	120119429	PRKAB1	14.07891218	1520
chr12.fa	120123598	120315092	CIT	1.184454899	456
chr12.fa	120151454	120151529	MIR1178	0	0
chr12.fa	120427648	120532299	CCDC64	27.42904879	3800
chr12.fa	120532899	120554643	RAB35	18.93904687	2508
chr12.fa	120565014	120632513	GCN1L1	20.47399012	7980
chr12.fa	120634503	120639014	RPLP0	155.8214246	8436
chr12.fa	120648250	120703563	PXN	23.81119068	4332
chr12.fa	120740124	120751045	SIRT4	4.190515145	228

chr12.fa	120759914	120765592	PLA2G1B	0	0
chr12.fa	120779133	120806958	MSI1	4.01060076	532
chr12.fa	120875904	120878529	COX6A1	43.41718841	1064
chr12.fa	120881764	120884215	TRIAP1	18.58900331	988
chr12.fa	120884241	120901556	GATC	8.338998375	1444
chr12.fa	120901753	120907558	SRSF9	192.5946794	5785
chr12.fa	120907660	120936298	DYNLL1	97.9063392	4332
chr12.fa	120941082	120966964	COQ5	16.80097902	1140
chr12.fa	120972132	121015397	RNF10	79.43520316	13741
chr12.fa	121016848	121019201	POP5	23.21095716	836
chr12.fa	121078422	121105127	CABP1	0	0
chr12.fa	121124949	121139667	MLEC	12.77814675	3648
chr12.fa	121147831	121161443	UNC119B	28.45649559	6156
chr12.fa	121163571	121177811	ACADS	9.698475055	836
chr12.fa	121201035	121342151	SPPL3	3.476639774	532
chr12.fa	121407641	121410095	NCRNA00262	0	0
chr12.fa	121416549	121440314	HNF1A	2.088029862	304
chr12.fa	121440848	121454300	C12orf43	10.38988893	897
chr12.fa	121458095	121476780	OASL	0	0
chr12.fa	121570622	121624354	P2RX7	0	0
chr12.fa	121647664	121671909	P2RX4	5.833985236	532
chr12.fa	121675495	121736111	CAMKK2	17.71389441	4987
chr12.fa	121746048	121792012	ANAPC5	27.83958273	3420
chr12.fa	121837902	121862155	RNF34	11.40354748	1064
chr12.fa	121866900	122018920	KDM2B	3.946107347	988
chr12.fa	122064455	122079946	ORAI1	3.375896614	228
chr12.fa	122089293	122107560	MORN3	0	0
chr12.fa	122150658	122219974	TMEM120B	1.262291777	304
chr12.fa	122231058	122231594	RHOF	0	0
chr12.fa	122233173	122241390	LOC338799	3.333642309	228
chr12.fa	122242638	122270562	SETD1B	12.37472933	4560
chr12.fa	122277433	122326517	HPD	90.51383726	7904
chr12.fa	122326646	122355771	PSMD9	18.03435996	1900
chr12.fa	122356463	122441832	WDR66	0	0
chr12.fa	122459861	122499950	BCL7A	9.721603728	1596
chr12.fa	122516760	122628967	MLXIP	12.57777239	3036
chr12.fa	122652266	122688018	LRRC43	0.809058754	76
chr12.fa	122656577	122658746	IL31	0	0
chr12.fa	122688228	122692084	B3GNT4	0	0
chr12.fa	122692209	122712068	DIABLO	15.11658897	1596
chr12.fa	122716094	122751068	VPS33A	12.9863048	1520
chr12.fa	122755981	122907116	CLIP1	23.28301187	6156
chr12.fa	122957435	122985518	ZCCHC8	3.540910796	456

chr12.fa	122989190	123011560	RSRC2	21.58861422	2356
chr12.fa	123011809	123110947	KNTC1	0.7281084	228
chr12.fa	123185840	123187904	GPR109A	0	0
chr12.fa	123199303	123201439	GPR109B	0	0
chr12.fa	123212153	123215129	GPR81	3.406364192	456
chr12.fa	123237371	123255953	DENR	24.49126261	3371
chr12.fa	123259073	123311927	CCDC62	1.056580027	152
chr12.fa	123320039	123347507	HIP1R	30.61658016	6158
chr12.fa	123349875	123380712	VPS37B	30.74156395	3800
chr12.fa	123413539	123459752	ABCB9	0.456124108	76
chr12.fa	123460358	123464588	OGFOD2	2.872847988	228
chr12.fa	123464880	123467460	ARL6IP4	112.9355285	6815
chr12.fa	123468027	123594975	PITPNM2	0.50193667	152
chr12.fa	123495214	123495275	MIR4304	0	0
chr12.fa	123640946	123706441	MPHOSPH9	4.200300352	682
chr12.fa	123717844	123742506	C12orf65	5.67453083	532
chr12.fa	123745540	123756687	CDK2AP1	63.91853263	4636
chr12.fa	123773656	123834988	SBNO1	15.84736606	7825
chr12.fa	123868704	123893900	SETD8	25.189793	3116
chr12.fa	123899936	123921264	RILPL2	14.13384278	912
chr12.fa	123942651	123950937	SNRNP35	21.37378443	988
chr12.fa	123955909	124018265	RILPL1	7.08537985	836
chr12.fa	124020956	124021081	MIR3908	0	0
chr12.fa	124069076	124082688	TMED2	26.23503108	2508
chr12.fa	124086672	124105482	DDX55	5.801516138	684
chr12.fa	124105570	124118323	EIF2B1	20.78822872	1748
chr12.fa	124118381	124145334	GTF2H3	10.10745225	684
chr12.fa	124155660	124192948	TCTN2	2.90398274	380
chr12.fa	124196865	124246301	ATP6V0A2	2.583961974	760
chr12.fa	124247042	124420267	DNAH10	0.123649441	76
chr12.fa	124420955	124457163	CCDC92	14.75987367	1190
chr12.fa	124457670	124499969	ZNF664	39.04475736	8968
chr12.fa	124773710	124800568	FAM101A	3.147500974	304
chr12.fa	124808961	125052010	NCOR2	17.48260768	6976
chr12.fa	125262174	125348519	SCARB1	1.232713764	152
chr12.fa	125396192	125399587	UBC	184.4538315	21432
chr12.fa	125431370	125473667	DHX37	7.427639724	1520
chr12.fa	125478194	125510349	BRI3BP	0	0
chr12.fa	125549925	125627871	AACS	13.09171817	1955
chr12.fa	125811162	126143589	TMEM132B	0	0
chr12.fa	126443234	126467920	LOC400084	0	0
chr12.fa	126927027	126957331	LOC100128554	0	0
chr12.fa	127215247	127256808	LOC387895	0	0

chr12.fa	127354040	127359236	LOC440117	0	0
chr12.fa	128366162	128383184	FLJ37505	0	0
chr12.fa	128751948	129192460	TMEM132C	0	0
chr12.fa	128778637	128778723	MIR3612	0	0
chr12.fa	129277739	129308541	SLC15A4	1.212031393	152
chr12.fa	129338081	129469509	GLT1D1	1.24227658	152
chr12.fa	129556271	130388212	TMEM132D	0.292666663	76
chr12.fa	130517999	130526887	LOC100190940	0.479030389	76
chr12.fa	130636138	130646223	FLJ31485	0	0
chr12.fa	130647004	130650285	FZD10	0	0
chr12.fa	130822433	130856877	PIWIL1	0	0
chr12.fa	130880681	131002410	RIMBP2	0	0
chr12.fa	131274147	131323811	STX2	1.467558745	228
chr12.fa	131356617	131360826	RAN	107.5200832	5178
chr12.fa	131438452	131626008	GPR133	0	0
chr12.fa	131649556	131697476	LOC116437	0	0
chr12.fa	132195635	132284282	SFSWAP	8.33366099	1216
chr12.fa	132312941	132336316	MMP17	0	0
chr12.fa	132379279	132407707	ULK1	9.64176533	2264
chr12.fa	132413745	132428406	PUS1	4.059082016	380
chr12.fa	132434508	132565005	EP400	9.461406163	5218
chr12.fa	132515769	132515905	SNORA49	0	0
chr12.fa	132568828	132610885	EP400NL	0	0
chr12.fa	132621140	132628880	DDX51	2.508126615	532
chr12.fa	132628993	132636986	NOC4L	0	0
chr12.fa	132680917	132905905	GALNT9	0.578883985	76
chr12.fa	132851977	132857486	LOC100130238	0.87199543	152
chr12.fa	133067157	133161773	FBRSL1	6.910802851	1413
chr12.fa	133179736	133187037	LOC645277	0	0
chr12.fa	133195403	133198972	P2RX2	0	0
chr12.fa	133200348	133263945	POLE	2.561500474	903
chr12.fa	133264192	133281577	PXMP2	30.02390793	1292
chr12.fa	133287393	133299323	PGAM5	12.46590967	1824
chr12.fa	133302254	133338451	ANKLE2	4.928631144	988
chr12.fa	133345495	133405426	GOLGA3	32.08258217	13829
chr12.fa	133416938	133464204	CHFR	5.569784631	836
chr12.fa	133498019	133532868	ZNF605	3.765525789	1064
chr12.fa	133563056	133589153	ZNF26	4.420245132	608
chr12.fa	133613878	133639885	ZNF84	3.362553149	1064
chr12.fa	133657037	133684258	ZNF140	8.271613878	1140
chr12.fa	133707214	133736049	ZNF10	2.303304429	456
chr12.fa	133757995	133783697	ZNF268	4.608610378	1216
chr12.fa	133794898	133812422	LOC647589	0	0

chr13.fa	19408543	19446109	LOC284232	0	0
chr13.fa	19582399	19586774	LOC348021	0	0
chr13.fa	19620293	19692423	PHF2P1	0	0
chr13.fa	19747918	19755936	TUBA3C	0	0
chr13.fa	19836940	19919113	LOC100101938	0	0
chr13.fa	19919189	19920889	LOC100287114	0	0
chr13.fa	19997019	20110903	TPTE2	0	0
chr13.fa	20207788	20247599	MPHOSPH8	21.3844592	4104
chr13.fa	20248896	20357083	PSPC1	20.48266337	2204
chr13.fa	20397624	20437776	ZMYM5	5.843325661	912
chr13.fa	20532810	20665968	ZMYM2	20.5117966	9559
chr13.fa	20712395	20735183	GJA3	0	0
chr13.fa	20761604	20767114	GJB2	0	0
chr13.fa	20796101	20806534	GJB6	3.369669664	380
chr13.fa	20977806	21100012	CRYL1	72.06605229	4864
chr13.fa	21141208	21265576	IFT88	7.375600211	1048
chr13.fa	21277482	21297237	IL17D	0.908245176	76
chr13.fa	21303073	21348057	N6AMT2	5.814859603	228
chr13.fa	21351468	21476913	XPO4	3.248688916	1444
chr13.fa	21547176	21635722	LATS2	3.354769461	836
chr13.fa	21714653	21723224	SAP18	56.55938936	5852
chr13.fa	21727734	21746820	SKA3	0	0
chr13.fa	21750372	21753220	MRP63	18.80294353	2052
chr13.fa	21946710	22033508	ZDHHC20	7.912897064	1900
chr13.fa	22066828	22178355	EFHA1	22.04362637	1916
chr13.fa	22245215	22278640	FGF9	1.492466547	304
chr13.fa	23471169	23472320	BASP1P1	2.934227927	152
chr13.fa	23755060	23899304	SGCG	0	0
chr13.fa	23902965	24007841	SACS	0.216164131	152
chr13.fa	24144723	24250232	TNFRSF19	1.573639291	304
chr13.fa	24304328	24463587	MIPEP	10.43748062	1140
chr13.fa	24463028	24466242	PCOTH	0	0
chr13.fa	24465428	24471125	C1QTNF9B	0	0
chr13.fa	24734861	24881212	SPATA13	11.79139752	4484
chr13.fa	24736555	24736643	MIR2276	0	0
chr13.fa	24883716	24896669	C1QTNF9	0	0
chr13.fa	24995069	25086948	PARP4	31.83172503	7828
chr13.fa	25154346	25171812	LOC374491	0	0
chr13.fa	25254549	25285923	ATP12A	1.357919942	228
chr13.fa	25338301	25454058	RNF17	0	0
chr13.fa	25456412	25497085	CENPJ	0.980299886	228
chr13.fa	25502893	25542607	TPTE2P1	0.260864738	76
chr13.fa	25670276	25672704	PABPC3	0.695861693	76

chr13.fa	25742672	25745857	FAM123A	0	0
chr13.fa	25820341	25861704	MTMR6	10.3732096	2432
chr13.fa	25875666	25916561	NUPL1	10.71680382	2089
chr13.fa	25946209	26595420	ATP8A2	0	0
chr13.fa	26618735	26625198	SHISA2	0	0
chr13.fa	26786905	26796508	RNF6	18.27676623	3192
chr13.fa	26828756	26978569	CDK8	12.25730684	975
chr13.fa	27131840	27263082	WASF3	5.94384643	1292
chr13.fa	27329339	27334922	GPR12	0	0
chr13.fa	27640287	27746033	USP12	8.632999385	1748
chr13.fa	27825692	27825722	RPL21	0	0
chr13.fa	27829201	27829272	SNORD102	0	0
chr13.fa	27829538	27829663	SNORA27	0	0
chr13.fa	27844464	27847827	RASL11A	5.476825159	380
chr13.fa	27998681	28009775	GTF3A	90.0241321	5396
chr13.fa	28009847	28024739	MTIF3	40.76206129	2128
chr13.fa	28120050	28194720	LNX2	6.332808431	1368
chr13.fa	28196003	28241548	POLR1D	23.48383101	2660
chr13.fa	28366780	28368089	GSX1	0	0
chr13.fa	28494168	28500451	PDX1	0	0
chr13.fa	28519343	28519710	ATP5EP2	4.592820611	76
chr13.fa	28536278	28543317	CDX2	0.808613972	76
chr13.fa	28552243	28562774	PRHOXNB	0	0
chr13.fa	28577411	28674729	FLT3	0	0
chr13.fa	28710980	28713311	LOC100288730	1.02433332	76
chr13.fa	28712643	28869475	PAN3	12.44544969	2849
chr13.fa	28874483	29069265	FLT1	0	0
chr13.fa	29233141	29253093	POMP	36.38028982	2390
chr13.fa	29274218	29293150	SLC46A3	0.505494928	76
chr13.fa	29598748	30080084	MTUS2	5.155025265	1668
chr13.fa	30083551	30169825	SLC7A1	2.992271999	988
chr13.fa	30338545	30424820	UBL3	30.33436588	5928
chr13.fa	30510668	30524625	LOC440131	0	0
chr13.fa	30776767	30881584	KATNAL1	0.889341934	304
chr13.fa	30914407	30948036	LOC100188949	0	0
chr13.fa	31032879	31040081	HMGB1	13.32011381	2052
chr13.fa	31191830	31233686	USPL1	3.896291744	684
chr13.fa	31309669	31338556	ALOX5AP	0	0
chr13.fa	31480312	31499709	C13orf33	0	0
chr13.fa	31506834	31549153	C13orf26	0	0
chr13.fa	31710763	31736117	HSPH1	49.15398874	8105
chr13.fa	31774112	31906411	B3GALTL	0.348486824	66
chr13.fa	32313679	32377009	RXFP2	0	0

chr13.fa	32420920	32533721	EEF1DP3	2.939342922	76
chr13.fa	32605437	32870776	FRY	8.200893514	3952
chr13.fa	32877908	32886091	ZAR1L	0	0
chr13.fa	32889617	32973809	BRCA2	0.148334851	76
chr13.fa	32974860	33002315	N4BP2L1	0	0
chr13.fa	33006930	33112936	N4BP2L2	13.47156214	2964
chr13.fa	33078643	33083532	CG030	0	0
chr13.fa	33160564	33352158	PDS5B	7.438759278	2508
chr13.fa	33590571	33640282	KL	5.064512094	1140
chr13.fa	33677272	33859901	STARD13	3.189088106	912
chr13.fa	34392206	34540695	RFC3	1.769788225	228
chr13.fa	35516424	36246873	NBEA	3.487759328	1744
chr13.fa	36047926	36050832	MAB21L1	0	0
chr13.fa	36168361	36515382	MIR548F5	0	0
chr13.fa	36342790	36705514	DCLK1	0.4937082	304
chr13.fa	36788616	36788752	SOHLH2	0	0
chr13.fa	36801179	36869994	C13orf38	3.577160543	152
chr13.fa	36875775	36944317	SPG20	10.65164323	2584
chr13.fa	37005967	37017019	CCNA1	0	0
chr13.fa	37248049	37271975	C13orf36	0	0
chr13.fa	37393339	37403740	RFXAP	0.598232009	76
chr13.fa	37418968	37494409	SMAD9	17.22819229	4332
chr13.fa	37523908	37573504	ALG5	4.253896603	228
chr13.fa	37574678	37583450	EXOSC8	4.568135201	228
chr13.fa	37583752	37633850	FAM48A	20.80223935	2548
chr13.fa	37677397	37679801	CSNK1A1L	0	0
chr13.fa	38136719	38172981	POSTN	0	0
chr13.fa	38210773	38443939	TRPC4	0	0
chr13.fa	38923942	38937143	UFM1	32.54204214	3806
chr13.fa	39261173	39461267	FREM2	2.928223367	2128
chr13.fa	39540062	39564996	STOML3	0	0
chr13.fa	39584002	39612252	C13orf23	12.5799963	2946
chr13.fa	39612455	39624246	NHLRC3	1.905002002	304
chr13.fa	39917029	40177356	LHFP	0	0
chr13.fa	40229764	40365802	COG6	5.02870713	1584
chr13.fa	40238171	40238272	MIR4305	0	0
chr13.fa	40768646	40794639	FLJ42392	0	0
chr13.fa	40921271	41055143	LOC646982	0	0
chr13.fa	41129801	41240734	FOXO1	4.418466003	1140
chr13.fa	41301964	41302011	MIR320D1	0	0
chr13.fa	41303432	41345347	MRPS31	5.04916711	304
chr13.fa	41363547	41386596	SLC25A15	4.149372795	684
chr13.fa	41371127	41495886	SUGT1P3	2.152523276	380

chr13.fa	41506055	41593508	ELF1	34.80687292	5852
chr13.fa	41635697	41658139	WBP4	9.418039902	988
chr13.fa	41701709	41706936	KBTBD6	6.142664057	1444
chr13.fa	41765711	41768702	KBTBD7	6.778702549	912
chr13.fa	41790516	41837713	MTRF1	3.88717371	380
chr13.fa	41885341	41951166	NAA16	1.840286198	380
chr13.fa	42005476	42017723	OR7E37P	0	0
chr13.fa	42031542	42045013	C13orf15	3.050760853	152
chr13.fa	42140961	42535221	KIAA0564	7.523045498	2508
chr13.fa	42622889	42803888	DGKH	3.580941191	684
chr13.fa	42846289	42897402	AKAP11	8.68348216	3873
chr13.fa	43136872	43182149	TNFSF11	0	0
chr13.fa	43355686	43365685	C13orf30	0	0
chr13.fa	43462122	43566377	EPSTI1	8.595860074	608
chr13.fa	43597362	43683306	DNAJC15	1.826497951	228
chr13.fa	43787666	44361106	ENOX1	3.256694995	456
chr13.fa	44410489	44449063	CCDC122	3.470635215	304
chr13.fa	44453420	44468068	C13orf31	6.022795264	1216
chr13.fa	44596471	44604599	NCRNA00284	0	0
chr13.fa	44947978	44971850	SERP2	4.311718284	152
chr13.fa	45007655	45150701	TSC22D1	52.0550804	12078
chr13.fa	45513384	45563613	NUFIP1	1.454215281	228
chr13.fa	45563687	45602404	KIAA1704	10.92785295	684
chr13.fa	45694631	45858239	GTF2F2	11.38731293	744
chr13.fa	45766988	45775175	KCTD4	0	0
chr13.fa	45911304	45915297	TPT1	4825.859555	179892
chr13.fa	45911615	45911744	SNORA31	0	0
chr13.fa	45915480	45965618	LOC100190939	1.107507585	76
chr13.fa	45967454	45992516	SLC25A30	19.81393339	3268
chr13.fa	46039071	46110760	COG3	20.19488931	4070
chr13.fa	46115432	46189874	FAM194B	0	0
chr13.fa	46276446	46288693	SPERT	0	0
chr13.fa	46354416	46425846	SIAH3	0	0
chr13.fa	46536314	46626894	ZC3H13	24.52573323	7068
chr13.fa	46627322	46679211	CPB2	0	0
chr13.fa	46700058	46756459	LCP1	118.1781758	20140
chr13.fa	46916137	46961635	C13orf18	0	0
chr13.fa	47127296	47327175	LRCH1	4.776960426	1736
chr13.fa	47345391	47371367	ESD	70.89649759	3800
chr13.fa	47407513	47471169	HTR2A	0	0
chr13.fa	48516791	48575462	SUCLA2	20.30697442	1976
chr13.fa	48611703	48621282	NUDT15	26.74853209	2432
chr13.fa	48650092	48669240	MED4	19.80837361	1801

chr13.fa	48807274	48836232	ITM2B	27.63476054	2356
chr13.fa	48877883	49056026	RB1	6.482477629	1391
chr13.fa	48985182	49018840	LPAR6	0.553753792	76
chr13.fa	49063099	49107316	RCBTB2	3.726384958	532
chr13.fa	49280951	49283498	CYSLTR2	0	0
chr13.fa	49550048	49783915	FNDC3A	21.44094654	6151
chr13.fa	49794474	49796513	MLNR	0	0
chr13.fa	49822047	49867622	CDADC1	0.993865742	152
chr13.fa	49882786	50018221	CAB39L	55.49191217	9956
chr13.fa	50018429	50069139	SETDB2	5.162141779	1444
chr13.fa	50069801	50103117	PHF11	8.816027245	754
chr13.fa	50106082	50159719	RCBTB1	8.381697463	1520
chr13.fa	50202435	50208008	ARL11	0	0
chr13.fa	50234810	50265623	EBPL	5.184603278	228
chr13.fa	50273443	50367057	KPNA3	8.783335756	1767
chr13.fa	50464545	50467516	CTAGE10P	0	0
chr13.fa	50486842	50510625	C13orf1	4.222761852	608
chr13.fa	50556688	50699677	DLEU2	0	0
chr13.fa	50570551	50570637	MIR3613	0	0
chr13.fa	50571143	50592603	TRIM13	5.358513104	1444
chr13.fa	50592959	50595058	KCNRG	0	0
chr13.fa	50623109	50623197	MIR16-1	0	0
chr13.fa	50623255	50623337	MIR15A	0	0
chr13.fa	50656414	50679433	DLEU1	0	0
chr13.fa	50746154	50747751	ST13P4	0	0
chr13.fa	51286759	51417885	DLEU7	0	0
chr13.fa	51483892	51544596	RNASEH2B	8.93834234	836
chr13.fa	51568647	51640293	GUCY1B2	0	0
chr13.fa	51796507	51855616	FAM124A	0.803276586	76
chr13.fa	51915168	51935500	SERPINE3	0	0
chr13.fa	51935701	52027275	INTS6	6.984191908	2736
chr13.fa	52158484	52336171	WDFY2	3.993476647	684
chr13.fa	52342131	52378293	DHRS12	7.562186328	912
chr13.fa	52387483	52419286	NCRNA00282	0	0
chr13.fa	52436117	52440372	CCDC70	0	0
chr13.fa	52506806	52585630	ATP7B	2.290628137	684
chr13.fa	52586523	52598826	ALG11	3.407698539	152
chr13.fa	52603781	52607736	UTP14C	3.417928528	608
chr13.fa	52638900	52703214	NEK5	0	0
chr13.fa	52706779	52733996	NEK3	5.253099731	608
chr13.fa	52741847	52768602	THSD1P1	2.419392573	380
chr13.fa	52793481	52864119	TPTE2P2	0	0
chr13.fa	52951303	52980629	THSD1	0	0

chr13.fa	52986737	53024763	VPS36	14.31998412	2848
chr13.fa	53029495	53050763	CKAP2	3.166848998	532
chr13.fa	53063128	53161225	TPTE2P3	0.646935655	76
chr13.fa	53191605	53217919	HNRNPA1L2	2.858614959	304
chr13.fa	53226831	53262433	SUGT1	19.49969479	1566
chr13.fa	53277400	53313947	LECT1	0	0
chr13.fa	53384185	53384275	MIR759	0	0
chr13.fa	53418109	53422774	PCDH8	0	0
chr13.fa	53602972	53626188	OLFM4	0.60045592	76
chr13.fa	54886107	54886183	MIR1297	0	0
chr13.fa	58205789	58303065	PCDH17	0.422098272	152
chr13.fa	60239723	60738119	DIAPH3	0	0
chr13.fa	60970591	61148013	TDRD3	7.543060696	1064
chr13.fa	61773932	61774014	MIR3169	0	0
chr13.fa	61983819	61989655	PCDH20	0	0
chr13.fa	64311568	64316701	OR7E156P	0	0
chr13.fa	66876966	67804468	PCDH9	0	0
chr13.fa	70274725	70682625	KLHL1	0	0
chr13.fa	70689273	70713885	ATXN8OS	0	0
chr13.fa	72012098	72441330	DACH1	4.839229929	1140
chr13.fa	73282495	73301938	MZT1	3.503771486	380
chr13.fa	73302042	73329539	C13orf34	6.815174686	608
chr13.fa	73330329	73356229	DIS3	12.90957988	3952
chr13.fa	73356345	73590591	PIBF1	9.020182257	1216
chr13.fa	73633142	73651676	KLF5	51.96656875	7828
chr13.fa	74260149	74708066	KLF12	0.46479736	228
chr13.fa	75126980	75131257	LOC338864	0	0
chr13.fa	75811889	75814517	CTAGE11P	0	0
chr13.fa	75858809	76056250	TBC1D4	8.234474567	2356
chr13.fa	76099350	76111991	COMMD6	40.75872543	3156
chr13.fa	76123927	76180068	UCHL3	20.04944554	760
chr13.fa	76194570	76434006	LMO7	25.11106655	9609
chr13.fa	77454304	77460540	KCTD12	2.167868261	608
chr13.fa	77502585	77503224	BTF3P11	0	0
chr13.fa	77566059	77576652	CLN5	3.62186115	456
chr13.fa	77579389	77601331	FBXL3	23.81185786	3800
chr13.fa	77618792	77901177	MYCBP2	13.70751908	9261
chr13.fa	78109809	78219398	SCEL	1.571860163	228
chr13.fa	78272147	78272251	MIR3665	0	0
chr13.fa	78272470	78338377	SLAIN1	7.002650368	836
chr13.fa	78469616	78549664	EDNRB	0.394744169	76
chr13.fa	79173230	79177695	POU4F1	0	0
chr13.fa	79188421	79233314	RNF219	3.826016163	608

chr13.fa	79894100	79979923	RBM26	16.84100941	2736
chr13.fa	80055259	80130212	NDFIP2	2.904649913	608
chr13.fa	80910112	80915086	SPRY2	6.408199008	608
chr13.fa	84451343	84456528	SLITRK1	0	0
chr13.fa	86366922	86373483	SLITRK6	0.404306986	76
chr13.fa	88096242	88323218	LOC642345	0	0
chr13.fa	88324870	88331870	SLITRK5	0	0
chr13.fa	90883436	90883531	MIR622	0	0
chr13.fa	91543208	91578851	LOC144776	0	0
chr13.fa	92000074	92006829	MIR17HG	0	0
chr13.fa	92050935	93519487	GPC5	0.586890064	76
chr13.fa	93879078	95060273	GPC6	0.237958457	76
chr13.fa	95091841	95131936	DCT	0	0
chr13.fa	95226308	95248511	TGDS	1.777349522	152
chr13.fa	95254104	95281946	GPR180	0.861987831	152
chr13.fa	95361879	95364389	SOX21	1.346133215	152
chr13.fa	95672083	95953687	ABCC4	32.38748034	9105
chr13.fa	96085853	96230455	CLDN10	2.547267445	152
chr13.fa	96232011	96296957	DZIP1	1.139531901	304
chr13.fa	96329393	96447243	DNAJC3	12.39429974	3116
chr13.fa	96453836	96705736	UGGT2	3.429048083	760
chr13.fa	96743093	97491812	HS6ST3	0.216608913	76
chr13.fa	97637973	97646604	OXGR1	0	0
chr13.fa	97874574	98046374	MBNL2	28.38510805	6004
chr13.fa	98086475	98120252	RAP2A	8.920106271	1748
chr13.fa	98605929	98676550	IPO5	25.62567952	6916
chr13.fa	98795434	99102023	FARP1	33.86237799	8740
chr13.fa	98828039	98829521	RNF113B	0	0
chr13.fa	98860778	98860854	MIR3170	0	0
chr13.fa	99102455	99229396	STK24	38.24392708	8490
chr13.fa	99336055	99404929	SLC15A1	2.70249642	380
chr13.fa	99445741	99738660	DOCK9	14.51190762	5251
chr13.fa	99848628	99853009	LOC100289373	0	0
chr13.fa	99853012	100038753	UBAC2	6.810726865	912
chr13.fa	99906967	99910682	GPR18	0	0
chr13.fa	99946789	99959749	GPR183	0	0
chr13.fa	100003674	100004281	FKSG29	0	0
chr13.fa	100008385	100008482	MIR623	0	0
chr13.fa	100153728	100215276	TM9SF2	6.367279049	684
chr13.fa	100258919	100549388	CLYBL	2.05044577	532
chr13.fa	100295313	100295403	MIR4306	0	0
chr13.fa	100615275	100624178	ZIC5	0	0
chr13.fa	100634319	100639019	ZIC2	0	0

chr13.fa	100741269	101182417	PCCA	30.95016678	3187
chr13.fa	101182692	101241046	A2LD1	7.71474661	912
chr13.fa	101256090	101327103	TMTC4	3.151726404	532
chr13.fa	101706130	102068813	NALCN	0	0
chr13.fa	102104966	102368794	ITGBL1	0	0
chr13.fa	102373205	103054124	FGF14	0	0
chr13.fa	102944670	103046869	LOC283480	0.793491378	76
chr13.fa	103046928	103048055	LOC283481	0	0
chr13.fa	103249286	103331523	TPP2	11.12422428	2356
chr13.fa	103338097	103346854	C13orf39	0	0
chr13.fa	103381717	103411422	C13orf40	0	0
chr13.fa	103418463	103426149	C13orf27	4.409125578	228
chr13.fa	103436631	103451398	KDELC1	0.804388541	76
chr13.fa	103451405	103493888	BIVM	4.792972584	836
chr13.fa	103498174	103528345	ERCC5	21.04464563	3876
chr13.fa	103532449	103548383	LOC121952	0	0
chr13.fa	103696348	103719196	SLC10A2	0	0
chr13.fa	106118216	106143383	DAOA	0	0
chr13.fa	107028911	107030142	LOC728192	0	0
chr13.fa	107142096	107187337	EFNB2	4.696010073	912
chr13.fa	107195662	107220514	ARGLU1	13.51359406	1064
chr13.fa	107820879	108519460	FAM155A	0	0
chr13.fa	108859792	108870716	LIG4	2.827702599	532
chr13.fa	108870763	108886603	ABHD13	0.31868642	76
chr13.fa	108921977	108959365	TNFSF13B	0	0
chr13.fa	109248500	109860355	MYO16	0	0
chr13.fa	110406184	110438914	IRS2	9.660890963	3040
chr13.fa	110801310	110959496	COL4A1	0.776367265	228
chr13.fa	110959631	111165373	COL4A2	1.619229463	456
chr13.fa	111175413	111214071	RAB20	16.80097902	1140
chr13.fa	111268008	111292342	CARKD	22.39789536	2680
chr13.fa	111293757	111358480	CARS2	17.1630317	1437
chr13.fa	111365083	111373421	ING1	3.291388003	608
chr13.fa	111516334	111522655	C13orf29	0	0
chr13.fa	111530887	111567416	ANKRD10	13.54850945	1520
chr13.fa	111767624	111958081	ARHGEF7	11.35773491	4484
chr13.fa	111973015	111996594	C13orf16	0	0
chr13.fa	112721913	112726020	SOX1	0.4114235	76
chr13.fa	113030651	113089009	C13orf28	0	0
chr13.fa	113139328	113242481	TUBGCP3	11.29679976	1976
chr13.fa	113301358	113338811	C13orf35	0	0
chr13.fa	113344643	113541482	ATP11A	1.717748712	684
chr13.fa	113621798	113622756	LOC100289410	7.049797277	304

chr13.fa	113622953	113752862	MCF2L	10.48262601	2584
chr13.fa	113760105	113774995	F7	0	0
chr13.fa	113777113	113803843	F10	0	0
chr13.fa	113812968	113826694	PROZ	1.138197554	76
chr13.fa	113831925	113863029	PCID2	21.83435636	1672
chr13.fa	113863086	113919392	CUL4A	24.80438925	4332
chr13.fa	113951469	113977741	LAMP1	15.28716293	1746
chr13.fa	113978505	114018463	GRTP1	7.212587549	456
chr13.fa	114076260	114107839	ADPRHL1	1.555625614	152
chr13.fa	114110134	114145023	DCUN1D2	0.557979223	76
chr13.fa	114145308	114204544	TMCO3	6.024574392	836
chr13.fa	114239003	114295788	TFDP1	23.21318107	2812
chr13.fa	114303122	114312513	ATP4B	0	0
chr13.fa	114321597	114438637	GRK1	0.805500496	76
chr13.fa	114451484	114454062	LOC100130386	0	0
chr13.fa	114462216	114514899	FAM70B	0	0
chr13.fa	114523522	114567046	GAS6	1.15999188	152
chr13.fa	114586610	114626485	FLJ44054	0	0
chr13.fa	114747194	114898095	RASA3	0.804833323	152
chr13.fa	115000362	115038150	CDC16	31.41407458	3239
chr13.fa	115047078	115071283	UPF3A	3.582497929	380
chr13.fa	115079965	115092803	ZNF828	10.18173088	1748
chr14.fa	19377594	19378574	OR11H12	0	0
chr14.fa	19553365	19584942	POTEG	1.711521762	152
chr14.fa	19983954	20020272	POTEM	0.253525833	76
chr14.fa	20181063	20182491	OR11H2	0	0
chr14.fa	20215587	20216528	OR4Q3	0	0
chr14.fa	20248482	20249423	OR4M1	0	0
chr14.fa	20295608	20296531	OR4N2	0	0
chr14.fa	20344427	20345371	OR4K2	0	0
chr14.fa	20388766	20389737	OR4K5	0	0
chr14.fa	20403767	20404842	OR4K1	0	0
chr14.fa	20443678	20444724	OR4K15	0	0
chr14.fa	20482420	20483352	OR4K14	0	0
chr14.fa	20502003	20502917	OR4K13	0	0
chr14.fa	20528204	20529142	OR4L1	0	0
chr14.fa	20585566	20586597	OR4K17	0	0
chr14.fa	20611895	20612821	OR4N5	0	0
chr14.fa	20665495	20666532	OR11G2	0	0
chr14.fa	20691869	20692861	OR11H6	0	0
chr14.fa	20710951	20711925	OR11H4	0	0
chr14.fa	20757301	20774153	TTC5	8.156415298	684
chr14.fa	20779529	20801457	CCNB1IP1	16.13958794	1368

chr14.fa	20794600	20794698	SNORD126	0	0
chr14.fa	20811230	20811570	RPPH1	1853.7373	28424
chr14.fa	20811773	20826063	PARP2	6.30589911	524
chr14.fa	20833826	20881579	TEP1	3.917863679	1884
chr14.fa	20896970	20903801	KLHL33	0	0
chr14.fa	20915207	20923267	OSGEP	9.251468982	676
chr14.fa	20923290	20925926	APEX1	63.37856709	4252
chr14.fa	20926012	20929637	TMEM55B	19.72186348	1520
chr14.fa	20937538	20946165	PNP	16.75516646	1824
chr14.fa	20978631	20979281	RNASE10	0	0
chr14.fa	21024252	21029090	RNASE9	0	0
chr14.fa	21051052	21055784	RNASE11	0	0
chr14.fa	21058240	21058982	RNASE12	0	0
chr14.fa	21108855	21109850	OR6S1	0	0
chr14.fa	21152336	21162345	ANG	4.079319604	124
chr14.fa	21167514	21168758	RNASE4	10.86046846	608
chr14.fa	21214099	21216539	EDDM3A	0	0
chr14.fa	21236586	21239107	EDDM3B	0	0
chr14.fa	21249210	21250626	RNASE6	0	0
chr14.fa	21269515	21271036	RNASE1	0	0
chr14.fa	21359562	21360507	RNASE3	0	0
chr14.fa	21387500	21388335	ECRP	0	0
chr14.fa	21423630	21424594	RNASE2	0	0
chr14.fa	21457965	21465194	METT11D1	9.388684279	754
chr14.fa	21467414	21470034	SLC39A2	4.767842392	304
chr14.fa	21484922	21493935	NDRG2	137.9551923	13641
chr14.fa	21498345	21500332	TPPP2	0	0
chr14.fa	21500979	21502944	RNASE13	0	0
chr14.fa	21510385	21512392	RNASE7	0	0
chr14.fa	21526052	21526516	RNASE8	0	0
chr14.fa	21538527	21558036	ARHGEF40	4.645082515	1140
chr14.fa	21558205	21572863	ZNF219	5.965418365	912
chr14.fa	21567174	21571883	C14orf176	1.167330786	76
chr14.fa	21623096	21624184	OR5AU1	0	0
chr14.fa	21677296	21737638	HNRNPC	67.66137453	9891
chr14.fa	21756136	21819460	RPGRIP1	0	0
chr14.fa	21819631	21852425	SUPT16H	24.53707517	5168
chr14.fa	21853353	21905457	CHD8	26.77277271	10092
chr14.fa	21860310	21860412	SNORD9	0	0
chr14.fa	21865452	21865560	SNORD8	0	0
chr14.fa	21927179	21945132	RAB2B	8.004299798	1038
chr14.fa	21945335	21967319	TOX4	21.03107978	3952
chr14.fa	21967450	21979457	METTL3	26.24481629	1900

chr14.fa	21989232	22005337	SALL2	3.44083481	760
chr14.fa	22037934	22038875	OR10G3	0	0
chr14.fa	22102066	22102998	OR10G2	0	0
chr14.fa	22133297	22134238	OR4E2	0	0
chr14.fa	23033807	23058143	DAD1	55.77323689	1748
chr14.fa	23067147	23081265	ABHD4	23.57745766	2660
chr14.fa	23235731	23240998	OXA1L	49.16155004	3800
chr14.fa	23242432	23289014	SLC7A7	20.83515323	2508
chr14.fa	23299092	23304246	MRPL52	14.2904061	836
chr14.fa	23305793	23316803	MMP14	2.388702604	380
chr14.fa	23340960	23347291	LRP10	12.48125466	1824
chr14.fa	23352432	23356889	REM2	0	0
chr14.fa	23369854	23388396	RBM23	28.50764554	3338
chr14.fa	23389733	23398661	PRMT5	23.26544297	2584
chr14.fa	23415437	23426351	HAUS4	11.35039601	836
chr14.fa	23440410	23451848	JUB	3.563594687	760
chr14.fa	23456110	23479360	C14orf93	4.80453692	474
chr14.fa	23495060	23504429	PSMB5	61.60877886	4028
chr14.fa	23511376	23513269	PSMB11	0	0
chr14.fa	23516270	23526747	CDH24	0	0
chr14.fa	23527774	23564787	ACIN1	30.29945048	6958
chr14.fa	23564824	23569665	C14orf119	19.13808689	2413
chr14.fa	23586515	23588474	CEBPE	0	0
chr14.fa	23594504	23652849	SLC7A8	26.36646421	5168
chr14.fa	23742844	23755309	HOMEZ	8.132397061	1368
chr14.fa	23765112	23772057	PPP1R3E	1.872532905	380
chr14.fa	23776104	23780968	BCL2L2	51.31985548	6916
chr14.fa	23789397	23791029	PABPN1	12.37917715	909
chr14.fa	23815527	23822080	SLC22A17	0	0
chr14.fa	23825611	23834842	EFS	13.00965586	1824
chr14.fa	23842018	23845612	IL25	0	0
chr14.fa	23846017	23848981	CMTM5	0	0
chr14.fa	23851199	23877486	MYH6	0	0
chr14.fa	23857805	23857875	MIR208A	0	0
chr14.fa	23881947	23904870	MYH7	0	0
chr14.fa	23887196	23887272	MIR208B	0	0
chr14.fa	23938898	23947402	NGDN	8.332771425	595
chr14.fa	23990065	24020858	ZFHX2	1.288756316	532
chr14.fa	24025198	24028776	THTPA	14.16342079	1140
chr14.fa	24028788	24037243	AP1G2	13.67037977	1739
chr14.fa	24037280	24048009	JPH4	0	0
chr14.fa	24105573	24114848	DHRS2	636.3522863	48272
chr14.fa	24391457	24403777	C14orf165	0	0

chr14.fa	24407940	24424243	C14orf167	14.3104213	2128
chr14.fa	24424299	24438488	DHRS4	19.0980565	912
chr14.fa	24439083	24475617	DHRS4L2	2.598862176	228
chr14.fa	24505710	24520580	DHRS4L1	0	0
chr14.fa	24521206	24538937	LRRC16B	0	0
chr14.fa	24540756	24547296	CPNE6	0.840860678	76
chr14.fa	24549316	24553832	NRL	0	0
chr14.fa	24563483	24573339	PCK2	19.02488983	1900
chr14.fa	24583906	24594451	DCAF11	40.36064539	8116
chr14.fa	24600675	24602058	FITM1	0	0
chr14.fa	24605378	24608173	PSME1	122.1358475	5448
chr14.fa	24608177	24610797	FAM158A	24.37739838	912
chr14.fa	24612574	24615855	PSME2	101.1087708	3769
chr14.fa	24616659	24629870	RNF31	14.7342987	2356
chr14.fa	24630422	24635774	IRF9	32.23202898	2432
chr14.fa	24641234	24649424	REC8	1.474897651	152
chr14.fa	24649464	24658124	IPO4	11.39131597	1824
chr14.fa	24658353	24664882	TM9SF1	2.432736038	304
chr14.fa	24674926	24677454	TSSK4	0	0
chr14.fa	24678787	24683036	CHMP4A	61.07548505	3724
chr14.fa	24683468	24685276	MDP1	9.458070297	111
chr14.fa	24686057	24686429	NEDD8	159.9069711	2682
chr14.fa	24701648	24708447	GMPR2	35.1164413	3496
chr14.fa	24708851	24711880	TINF2	22.84023122	1900
chr14.fa	24718320	24732416	TGM1	0.60868439	76
chr14.fa	24734744	24740803	RABGGTA	8.685261289	760
chr14.fa	24759804	24769039	DHRS1	15.67701449	1216
chr14.fa	24769098	24774374	C14orf21	15.68791166	1444
chr14.fa	24774393	24778036	CIDEB	24.46301894	1672
chr14.fa	24779357	24781259	LTB4R2	0	0
chr14.fa	24782313	24787242	LTB4R	0.391853085	76
chr14.fa	24787555	24804277	ADCY4	0	0
chr14.fa	24805227	24809242	RIPK3	7.038677723	608
chr14.fa	24836145	24848810	NFATC4	0.32091033	76
chr14.fa	24867992	24888494	NYNRIN	2.581293281	912
chr14.fa	24895740	24898731	CBLN3	1.442206162	152
chr14.fa	24899141	24910547	KHNYN	23.05906405	5624
chr14.fa	24910880	24912007	SDR39U1	10.93430229	177
chr14.fa	24974712	24977471	CMA1	0	0
chr14.fa	25042724	25045466	CTSG	0	0
chr14.fa	25075693	25078864	GZMH	0	0
chr14.fa	25100161	25103432	GZMB	0	0
chr14.fa	25281306	25519095	STXBP6	8.636557642	760

chr14.fa	26915089	27066960	NOVA1	0.78770921	152
chr14.fa	27377848	27377931	MIR4307	0	0
chr14.fa	29236287	29238871	FOXG1	0	0
chr14.fa	29241910	29264000	C14orf23	0	0
chr14.fa	30045687	30396899	PRKD1	13.7293134	2270
chr14.fa	31028329	31089046	G2E3	1.817157525	456
chr14.fa	31091521	31205018	SCFD1	32.6779231	3168
chr14.fa	31343741	31359822	COCH	0	0
chr14.fa	31363005	31495607	STRN3	14.41160924	2736
chr14.fa	31483852	31483948	MIR624	0	0
chr14.fa	31494683	31562638	AP4S1	0.732333831	76
chr14.fa	31569324	31676689	HECTD1	50.51635651	20748
chr14.fa	31760994	31858181	HEATR5A	8.135288145	2508
chr14.fa	31915243	31926680	C14orf126	9.459849425	1140
chr14.fa	31952150	31957151	GPR33	0	0
chr14.fa	32030591	32330429	NUBPL	6.079727381	836
chr14.fa	32544625	32545905	C14orf128	1.319446285	76
chr14.fa	32546495	32628934	ARHGAP5	17.59869583	7600
chr14.fa	32798479	33302268	AKAP6	0	0
chr14.fa	33408459	34273382	NPAS3	0	0
chr14.fa	34393421	34420284	EGLN3	4.346411293	532
chr14.fa	34902144	34931468	C14orf147	13.33990662	1520
chr14.fa	34985135	35008943	EAPP	30.09218199	1824
chr14.fa	35030618	35099366	SNX6	20.28807117	2714
chr14.fa	35179588	35184029	CFL2	3.847810489	608
chr14.fa	35221937	35344853	BAZ1A	12.36160825	3344
chr14.fa	35409128	35409702	C14orf19	0	0
chr14.fa	35452104	35498773	SRP54	31.86197022	3268
chr14.fa	35514113	35552589	FAM177A1	13.59387724	2052
chr14.fa	35554678	35591519	PPP2R3C	11.0588413	912
chr14.fa	35591776	35743270	KIAA0391	7.123408725	836
chr14.fa	35761574	35786682	PSMA6	66.28121548	3040
chr14.fa	35870716	35873960	NFKBIA	49.61567263	3496
chr14.fa	36003248	36006260	INSM2	0	0
chr14.fa	36007558	36278432	RALGAPA1	3.43883329	1216
chr14.fa	36295597	36341169	BRMS1L	3.270483242	380
chr14.fa	36767764	36789882	MBIP	6.15356122	456
chr14.fa	36942494	36982990	SFTA3	0	0
chr14.fa	36985604	36989430	NKX2-1	0	0
chr14.fa	37049216	37051786	NKX2-8	0	0
chr14.fa	37126773	37147011	PAX9	3.264923465	456
chr14.fa	37147126	37344209	SLC25A21	1.216034433	76
chr14.fa	37641231	37642998	LOC100129794	1.217813561	76

chr14.fa	37667118	38020464	MIPOL1	2.824366733	811
chr14.fa	38059191	38064489	FOXA1	109.7795766	15352
chr14.fa	38677204	38682268	SSTR1	0	0
chr14.fa	38723319	38725574	CLEC14A	0	0
chr14.fa	39501123	39572437	SEC23A	8.36057031	1444
chr14.fa	39583488	39606177	SIP1	4.974666098	304
chr14.fa	39617015	39639634	TRAPPC6B	6.052595669	912
chr14.fa	39644387	39652422	PNN	33.27837901	5396
chr14.fa	39703125	39722575	MIA2	0.741007083	76
chr14.fa	39734476	39820397	CTAGE5	6.666172662	1217
chr14.fa	39865577	39901704	FBXO33	7.221483192	1672
chr14.fa	42076764	42373752	LRFN5	0	0
chr14.fa	44973354	44976499	FSCB	0	0
chr14.fa	45366507	45376460	C14orf28	1.163327746	152
chr14.fa	45393527	45431179	KLHL28	3.870049597	1140
chr14.fa	45431416	45543634	FAM179B	10.28936816	2888
chr14.fa	45553302	45584801	PRPF39	4.719583527	608
chr14.fa	45580078	45580176	SNORD127	0	0
chr14.fa	45584805	45603732	FKBP3	35.21184708	2128
chr14.fa	45605136	45670093	FANCM	1.185566854	380
chr14.fa	45672393	45722605	C14orf106	4.953316554	1064
chr14.fa	47120220	47121028	RPL10L	0	0
chr14.fa	47308828	48144157	MDGA2	0	0
chr14.fa	48230198	48230307	MIR548Y	0	0
chr14.fa	50044042	50053094	RPS29	697.7586893	25916
chr14.fa	50065415	50081390	PPIL5	1.937248709	152
chr14.fa	50085407	50087349	RPL36AL	314.0742509	7372
chr14.fa	50087489	50090199	MGAT2	2.493893586	304
chr14.fa	50091892	50101948	C14orf104	3.422598741	456
chr14.fa	50110270	50155098	POLE2	0.868881955	76
chr14.fa	50159823	50219870	KLHDC1	2.512352045	304
chr14.fa	50234787	50249856	KLHDC2	33.33397678	2707
chr14.fa	50250532	50319539	SDCCAG1	14.96447347	2773
chr14.fa	50359736	50363772	ARF6	54.49404339	9652
chr14.fa	50448430	50474238	C14orf182	0	0
chr14.fa	50550369	50559361	C14orf183	0	0
chr14.fa	50575350	50583297	C14orf138	2.842158019	228
chr14.fa	50583846	50698099	SOS2	13.99729466	3344
chr14.fa	50709152	50778947	L2HGDH	2.495672714	684
chr14.fa	50779047	50792946	ATP5S	6.289886952	532
chr14.fa	50796720	50862617	CDKL1	10.05207687	532
chr14.fa	50885243	50999376	MAP4K5	15.9710155	3162
chr14.fa	50999800	51099784	ATL1	1.735317607	228

chr14.fa	51100360	51135023	SAV1	12.82551605	1748
chr14.fa	51186481	51297839	NIN	56.2516001	27664
chr14.fa	51338878	51371688	ABHD12B	0	0
chr14.fa	51371935	51411248	PYGL	25.53672308	3268
chr14.fa	51441981	51562422	TRIM9	0.244630189	76
chr14.fa	51706886	51724372	TMX1	1.647695521	304
chr14.fa	51955855	52197444	FRMD6	0.980077495	228
chr14.fa	52116236	52118462	C14orf82	0	0
chr14.fa	52327050	52436518	GNG2	0.43811043	76
chr14.fa	52456228	52471420	C14orf166	104.3185412	4930
chr14.fa	52471520	52535946	NID2	0	0
chr14.fa	52734431	52743442	PTGDR	0	0
chr14.fa	52781016	52795322	PTGER2	0	0
chr14.fa	52897308	53019301	TXNDC16	5.09853793	1064
chr14.fa	53019866	53104431	GPR137C	0	0
chr14.fa	53108605	53162419	ERO1L	4.085101772	608
chr14.fa	53173896	53194716	PSMC6	22.53221958	1614
chr14.fa	53196883	53241705	STYX	2.929780105	608
chr14.fa	53241911	53258386	GNPNAT1	14.62977489	2584
chr14.fa	53323989	53417815	FERMT2	4.291480696	664
chr14.fa	53503458	53620046	DDHD1	1.952816085	1140
chr14.fa	54416455	54423554	BMP4	0.815285704	76
chr14.fa	54863673	54886934	CDKN3	0	0
chr14.fa	54893647	54908148	CNIH	18.9259258	1211
chr14.fa	54941209	54955744	GMFB	11.02526025	2045
chr14.fa	54976587	55005334	CGRRF1	7.665153399	456
chr14.fa	55034637	55260033	SAMD4A	1.754220849	532
chr14.fa	55308724	55369542	GCH1	4.044848987	532
chr14.fa	55344831	55344911	MIR4308	0	0
chr14.fa	55405656	55493819	WDHD1	0.560425525	152
chr14.fa	55493844	55516206	SOCS4	4.097778064	1292
chr14.fa	55518362	55536912	MAPK1IP1L	11.44446743	3329
chr14.fa	55595935	55612148	LGALS3	52.77429316	4841
chr14.fa	55614834	55658396	DLGAP5	0	0
chr14.fa	55738021	55820329	FBXO34	22.10300479	3572
chr14.fa	55833110	55878576	ATG14	4.633518179	988
chr14.fa	55880930	55907263	TBPL2	0	0
chr14.fa	56042875	56046810	C14orf33	1.117737575	76
chr14.fa	56046925	56151302	KTN1	17.88913858	3952
chr14.fa	56232963	56234435	RPL13AP3	2.294853568	152
chr14.fa	56247853	56263392	C14orf34	0	0
chr14.fa	56585093	56768031	PELI2	2.670472104	684
chr14.fa	57046511	57116232	C14orf101	3.171741601	608

chr14.fa	57267425	57277184	OTX2	0	0
chr14.fa	57279901	57397550	OTX2OS1	0	0
chr14.fa	57669194	57735605	EXOC5	7.954706587	3040
chr14.fa	57735618	57756797	MUDENG	14.25371157	1976
chr14.fa	57857271	57879466	NAA30	7.121407205	1433
chr14.fa	57936595	57960576	C14orf105	8.074130598	912
chr14.fa	58030640	58063615	SLC35F4	0.950054699	76
chr14.fa	58470808	58618847	C14orf37	0	0
chr14.fa	58666833	58702353	ACTR10	12.78214979	1403
chr14.fa	58711593	58738726	PSMA3	50.85972834	2120
chr14.fa	58732083	58764855	FLJ31306	3.888508057	456
chr14.fa	58765222	58840451	ARID4A	11.44869287	2888
chr14.fa	58862644	58875369	TOMM20L	0	0
chr14.fa	58875420	58894232	TIMM9	16.44137264	760
chr14.fa	58894710	59015549	KIAA0586	4.232324668	1064
chr14.fa	59104757	59115038	DACT1	0.437665648	76
chr14.fa	59655399	59836471	DAAM1	17.07652157	3268
chr14.fa	59930240	59932059	GPR135	0	0
chr14.fa	59939406	59951073	C14orf149	0	0
chr14.fa	59951161	59972081	JKAMP	1.590763405	152
chr14.fa	59977364	60043549	C14orf38	0.687633223	73
chr14.fa	60062694	60337557	RTN1	0.922033423	152
chr14.fa	60558629	60601532	C14orf135	7.30754854	1292
chr14.fa	60611500	60632211	DHRS7	54.79671765	3420
chr14.fa	60712470	60765805	PPM1A	10.21753584	5244
chr14.fa	60902674	60952764	C14orf39	0	0
chr14.fa	60975938	60978525	SIX6	0	0
chr14.fa	61111417	61116155	SIX1	3.145054672	380
chr14.fa	61176256	61190852	SIX4	4.579032364	1292
chr14.fa	61201459	61435398	MNAT1	10.9990181	684
chr14.fa	61438167	61447782	TRMT5	3.200430051	760
chr14.fa	61447832	61550451	SLC38A6	0.747901206	76
chr14.fa	61744089	61748530	TMEM30B	4.56591129	912
chr14.fa	61788515	62017698	PRKCH	30.2440751	4768
chr14.fa	62162119	62214977	HIF1A	41.64006128	7600
chr14.fa	62229075	62263146	SNAPC1	4.454493358	532
chr14.fa	62462541	62568427	SYT16	2.155191969	304
chr14.fa	62584075	62600903	FLJ43390	0	0
chr14.fa	63173945	63568584	KCNH5	0	0
chr14.fa	63671145	63758559	RHOJ	0	0
chr14.fa	63779642	63784563	GPHB5	0	0
chr14.fa	63841355	64010079	PPP2R5E	15.56604134	2356
chr14.fa	64063757	64108591	WDR89	6.823625548	988

chr14.fa	64150935	64194756	SGPP1	0	0
chr14.fa	64319683	64693167	SYNE2	7.210363638	7220
chr14.fa	64693751	64805268	ESR2	0.496599285	76
chr14.fa	64812191	64814329	TEX21P	0	0
chr14.fa	64854759	64926725	MTHFD1	20.83937866	3231
chr14.fa	64932217	64941221	AKAP5	0	0
chr14.fa	64953555	64970554	ZBTB25	6.430660507	456
chr14.fa	64971292	65000408	ZBTB1	8.647677196	2204
chr14.fa	65007186	65009954	HSPA2	6.103968008	760
chr14.fa	65016620	65056096	C14orf50	1.202913359	76
chr14.fa	65171193	65211060	PLEKHG3	7.298430506	1444
chr14.fa	65213001	65289866	SPTB	0.16256788	76
chr14.fa	65381140	65401913	CHURC1	15.99681287	2432
chr14.fa	65405872	65409531	GPX2	10.20219085	456
chr14.fa	65412532	65438875	RAB15	33.89306796	5168
chr14.fa	65453507	65529370	FNTB	7.794362617	988
chr14.fa	65472892	65569227	MAX	32.7177311	4237
chr14.fa	65877311	65879335	LOC645431	0	0
chr14.fa	65879535	66209962	FUT8	0.998091173	152
chr14.fa	66953109	66965271	NCRNA00238	0	0
chr14.fa	66974125	67648525	GPHN	5.060953837	979
chr14.fa	67656110	67695267	FAM71D	0	0
chr14.fa	67708021	67802536	MPP5	31.5506227	7600
chr14.fa	67804581	67826720	ATP6V1D	52.38377442	4254
chr14.fa	67827034	67853233	EIF2S1	19.47856764	3648
chr14.fa	67853700	67878828	PLEK2	4.630627095	304
chr14.fa	67936983	67982021	TMEM229B	4.154710181	760
chr14.fa	68000008	68056022	PLEKHH1	17.95496634	5092
chr14.fa	68056256	68067017	PIGH	9.818343848	532
chr14.fa	68086579	68118436	ARG2	141.5314633	10488
chr14.fa	68120154	68141602	VTI1B	88.82277548	3986
chr14.fa	68143519	68162510	RDH11	22.39500428	2584
chr14.fa	68168603	68201168	RDH12	0.900016706	76
chr14.fa	68213237	68283306	ZFYVE26	7.686502943	3344
chr14.fa	68286496	69062738	RAD51L1	1.754220849	304
chr14.fa	69254375	69259785	ZFP36L1	29.64228484	4028
chr14.fa	69261464	69263190	C14orf181	0	0
chr14.fa	69340840	69446083	ACTN1	34.32962166	5852
chr14.fa	69517637	69619914	DCAF5	18.63859652	5016
chr14.fa	69658194	69710737	EXD2	3.13237838	760
chr14.fa	69726681	69821190	GALNTL1	0.784595735	152
chr14.fa	69846840	69865021	ERH	101.2837925	3648
chr14.fa	69865407	69929107	SLC39A9	10.014048	2432

chr14.fa	69951471	69995215	UPF0639	0	0
chr14.fa	70036531	70037918	C14orf162	0	0
chr14.fa	70078310	70181861	KIAA0247	5.014474101	1216
chr14.fa	70233000	70234430	LOC100289511	0	0
chr14.fa	70233834	70238722	SRSF5	96.88734326	6609
chr14.fa	70242552	70264006	SLC10A1	0	0
chr14.fa	70346143	70499083	SMOC1	0	0
chr14.fa	70510934	70655787	SLC8A3	0	0
chr14.fa	70712470	70714518	ADAM21P1	0	0
chr14.fa	70791798	70826444	COX16	29.48327521	2271
chr14.fa	70833213	70883807	SYNJ2BP	19.63090553	6232
chr14.fa	70924217	70926622	ADAM21	0	0
chr14.fa	70989078	71001732	ADAM20	0	0
chr14.fa	71050957	71067384	MED6	9.431828149	570
chr14.fa	71108504	71142077	TTC9	8.423284595	1976
chr14.fa	71194854	71275888	MAP3K9	7.243499909	1824
chr14.fa	71374122	71582099	PCNX	2.602865216	1512
chr14.fa	71865054	71865124	SNORD56B	0	0
chr14.fa	71954578	71956420	LOC145474	0	0
chr14.fa	71996042	72206120	SIPA1L1	12.10274504	3268
chr14.fa	72399786	73033238	RGS6	0	0
chr14.fa	73136660	73360809	DPF3	2.232806456	228
chr14.fa	73393040	73426357	DCAF4	4.668878361	532
chr14.fa	73436159	73493920	ZFYVE1	10.02739146	2052
chr14.fa	73525221	73588076	RBM25	26.316871	5092
chr14.fa	73603143	73690399	PSEN1	6.67395635	1824
chr14.fa	73704205	73741347	PAPLN	0.289775579	76
chr14.fa	73741918	73925286	NUMB	24.81461924	4066
chr14.fa	73945192	74025651	HEATR4	3.96523298	608
chr14.fa	73957644	73960105	C14orf169	6.330139738	684
chr14.fa	74003928	74010498	ACOT1	11.59813967	836
chr14.fa	74035772	74042359	ACOT2	12.43477492	988
chr14.fa	74058410	74062470	ACOT4	5.178153937	380
chr14.fa	74083548	74086592	ACOT6	1.683500485	76
chr14.fa	74111578	74168649	DNAL1	3.509776045	1064
chr14.fa	74178486	74181128	PNMA1	12.15033673	1444
chr14.fa	74181825	74253896	C14orf43	18.3623868	6764
chr14.fa	74318534	74352168	PTGR2	11.97798364	1444
chr14.fa	74353586	74398803	ZNF410	28.71269012	2812
chr14.fa	74400705	74413308	FAM161B	4.730035908	456
chr14.fa	74416643	74429813	COQ6	16.10645167	1216
chr14.fa	74433181	74486026	ENTPD5	23.10776769	2128
chr14.fa	74486059	74532796	C14orf45	5.378973083	684

chr14.fa	74526872	74551196	ALDH6A1	78.96573558	6750
chr14.fa	74551656	74667117	LIN52	7.520154414	608
chr14.fa	74706175	74729441	VSX2	0	0
chr14.fa	74751980	74769767	ABCD4	2.000630167	284
chr14.fa	74815166	74826711	VRTN	0	0
chr14.fa	74872596	74892805	TMEM90A	0	0
chr14.fa	74946643	74960084	NPC2	67.83306044	2736
chr14.fa	74960450	74962271	ISCA2	23.89503212	1140
chr14.fa	74964886	75079034	LTBP2	0.395188951	152
chr14.fa	75127955	75179807	KIAA0317	12.10585851	2964
chr14.fa	75179850	75203390	FCF1	11.94707128	1292
chr14.fa	75230069	75304013	YLPM1	25.75666786	9424
chr14.fa	75321835	75330537	PROX2	2.850164098	228
chr14.fa	75348594	75370450	DLST	34.74371385	4440
chr14.fa	75372346	75389145	RPS6KL1	0	0
chr14.fa	75408538	75422291	PGF	0	0
chr14.fa	75469612	75476294	EIF2B2	18.64571304	1292
chr14.fa	75480467	75518235	MLH3	5.137233978	1824
chr14.fa	75519928	75530736	ACYP1	2.46387079	76
chr14.fa	75536299	75544799	FAM164C	1.082822175	76
chr14.fa	75548818	75593778	NEK9	14.63622423	3648
chr14.fa	75598171	75643349	TMED10	6.933709133	1292
chr14.fa	75745481	75748937	FOS	10.18173088	988
chr14.fa	75894509	75939404	JDP2	3.163735523	608
chr14.fa	75988784	76013334	BATF	10.78841374	456
chr14.fa	76044940	76114512	FLVCR2	0.885561286	152
chr14.fa	76117233	76127538	C14orf1	12.07272224	836
chr14.fa	76127551	76421425	TTLL5	9.237903126	1976
chr14.fa	76424442	76448092	TGFB3	1.592987315	228
chr14.fa	76452096	76550092	C14orf179	18.18914415	759
chr14.fa	76618259	76669134	C14orf118	11.40688334	1290
chr14.fa	76837690	76968180	ESRRB	1.121518223	152
chr14.fa	77228235	77249363	VASH1	0.280212762	76
chr14.fa	77253586	77279283	ANGEL1	2.466761874	456
chr14.fa	77292725	77336645	C14orf166B	0	0
chr14.fa	77490886	77495034	C14orf4	50.10626735	9348
chr14.fa	77564578	77583630	KIAA1737	23.71222665	4636
chr14.fa	77597613	77608134	ZDHHC22	0	0
chr14.fa	77648102	77725838	TMEM63C	0	0
chr14.fa	77731834	77737655	NGB	0	0
chr14.fa	77741299	77787225	POMT2	1.040345478	228
chr14.fa	77787230	77797940	GSTZ1	26.24259238	2032
chr14.fa	77808114	77843396	TMED8	19.01021202	836

chr14.fa	77843762	77857587	SAMD15	1.669267456	152
chr14.fa	77860424	77889379	C14orf148	0.914694517	76
chr14.fa	77893018	77923983	VIPAR	10.17194567	1358
chr14.fa	77924373	77935815	AHSA1	80.37858612	5060
chr14.fa	77940738	77965210	ISM2	0	0
chr14.fa	77973270	78083110	SPTLC2	4.6728814	1520
chr14.fa	78138749	78174356	ALKBH1	4.576808453	532
chr14.fa	78174425	78183941	C14orf156	83.97086926	1499
chr14.fa	78183944	78227497	SNW1	34.84690331	3325
chr14.fa	78227173	78236085	C14orf178	0	0
chr14.fa	78266426	78400297	ADCK1	4.539446751	456
chr14.fa	78870093	80330760	NRXN3	0	0
chr14.fa	80663869	80678525	DIO2	0.260197565	76
chr14.fa	80962821	81405884	C14orf145	1.137752772	228
chr14.fa	81421869	81612646	TSHR	0	0
chr14.fa	81646394	81687575	GTF2A1	19.5019187	1596
chr14.fa	81669039	81669178	SNORA79	0	0
chr14.fa	81736909	81864927	STON2	11.58057078	1520
chr14.fa	81939239	82000205	SEL1L	2.193888017	649
chr14.fa	85996488	86094270	FLRT2	0	0
chr14.fa	88399358	88459907	GALC	2.388924995	456
chr14.fa	88471468	88481155	GPR65	0	0
chr14.fa	88646452	88793256	KCNK10	0.826627649	304
chr14.fa	88851988	88904804	SPATA7	7.556626551	684
chr14.fa	88932122	89021123	PTPN21	7.883096659	2203
chr14.fa	89029253	89079853	ZC3H14	20.75331332	4679
chr14.fa	89081174	89259096	EML5	23.80518612	7676
chr14.fa	89290978	89344335	TTC8	26.61020483	2636
chr14.fa	89622516	90085494	FOXN3	15.66522777	5700
chr14.fa	89883698	89886137	LOC400236	6.277433052	304
chr14.fa	90042560	90043820	PRO1768	0	0
chr14.fa	90263469	90421089	C14orf143	2.431846474	76
chr14.fa	90422246	90511108	TDP1	3.964788197	668
chr14.fa	90528108	90652195	KCNK13	0.671398674	76
chr14.fa	90722894	90738966	PSMC1	5.146129621	367
chr14.fa	90744398	90798279	C14orf102	8.433069803	1444
chr14.fa	90863327	90874619	CALM1	95.26655706	19204
chr14.fa	90921574	90925249	LOC400238	0	0
chr14.fa	91006932	91282761	TTC7B	10.33584789	1596
chr14.fa	91337167	91526993	RPS6KA5	3.918753244	760
chr14.fa	91580357	91691703	C14orf159	7.220593628	1138
chr14.fa	91592770	91592896	SNORA11B	0	0
chr14.fa	91698876	91720224	GPR68	0	0

chr14.fa	91737667	91884133	CCDC88C	27.98902954	9424
chr14.fa	91923956	91976644	SMEK1	31.69339778	5548
chr14.fa	92038788	92041383	C14orf184	0	0
chr14.fa	92047118	92198413	CATSPERB	0.468800399	76
chr14.fa	92246267	92333880	TC2N	21.35021098	5092
chr14.fa	92335755	92414046	FBLN5	3.851591137	456
chr14.fa	92434243	92506403	TRIP11	15.4475069	5618
chr14.fa	92524896	92572965	ATXN3	4.058414843	1292
chr14.fa	92582468	92588153	NDUFB1	149.2515473	2812
chr14.fa	92588298	92630543	CPSF2	16.99512643	3876
chr14.fa	92788925	92967825	SLC24A4	0	0
chr14.fa	92980125	93155334	RIN3	1.318111939	228
chr14.fa	93170152	93215047	LGMN	1.472451349	144
chr14.fa	93260650	93306304	GOLGA5	41.46281559	5390
chr14.fa	93389445	93401638	CHGA	0	0
chr14.fa	93403259	93582263	ITPK1	18.77714617	4209
chr14.fa	93533797	93538478	ITPK1-AS1	0	0
chr14.fa	93648541	93651249	MOAP1	30.06927571	3268
chr14.fa	93651296	93653425	C14orf109	13.71085494	836
chr14.fa	93669237	93673400	C14orf142	10.59204242	532
chr14.fa	93673460	93695561	UBR7	12.08917918	1976
chr14.fa	93703896	93799385	BTBD7	13.83650591	5928
chr14.fa	93799565	94173689	KIAA1409	0	0
chr14.fa	93813537	93814700	COX8C	0	0
chr14.fa	94184644	94254766	PRIMA1	0.926036463	152
chr14.fa	94371076	94392718	C14orf86	0	0
chr14.fa	94385258	94395944	FAM181A	0	0
chr14.fa	94400513	94423767	ASB2	0	0
chr14.fa	94463616	94478041	C14orf48	0	0
chr14.fa	94492724	94515276	OTUB2	1.745547597	304
chr14.fa	94517268	94547558	DDX24	35.54632326	4712
chr14.fa	94547639	94569060	IFI27L1	2.363794803	76
chr14.fa	94577079	94583033	IFI27	0	0
chr14.fa	94594118	94595957	IFI27L2	0	0
chr14.fa	94640649	94746072	PPP4R4	0	0
chr14.fa	94749650	94759596	SERPINA10	0	0
chr14.fa	94770585	94789688	SERPINA6	2.265720336	152
chr14.fa	94843084	94857029	SERPINA1	7.334680252	1216
chr14.fa	94908801	94919122	SERPINA11	0	0
chr14.fa	94929058	94942670	SERPINA9	0	0
chr14.fa	94953620	94984181	SERPINA12	0	0
chr14.fa	95027783	95036243	SERPINA4	0	0
chr14.fa	95047731	95059457	SERPINA5	2.193220844	228

chr14.fa	95078714	95090390	SERPINA3	7.440983189	532
chr14.fa	95107062	95113331	SERPINA13	0	0
chr14.fa	95234560	95236499	GSC	0	0
chr14.fa	95552565	95623759	DICER1	20.28718161	9500
chr14.fa	95604256	95604323	MIR3173	0	0
chr14.fa	95624025	95646270	FLJ45244	0	0
chr14.fa	95648276	95786245	CLMN	18.2849947	10483
chr14.fa	95873604	95876427	C14orf139	2.39403999	304
chr14.fa	95883831	95942173	C14orf49	3.096573416	456
chr14.fa	95999249	96001209	SNHG10	0	0
chr14.fa	95999692	95999966	SCARNA13	0	0
chr14.fa	96001323	96011055	GLRX5	47.63083223	2508
chr14.fa	96117515	96139789	TCL6	2.555495915	380
chr14.fa	96152754	96158980	TCL1B	0	0
chr14.fa	96176304	96180533	TCL1A	0	0
chr14.fa	96505662	96560135	C14orf132	1.128189955	380
chr14.fa	96671135	96710666	BDKRB2	0	0
chr14.fa	96722559	96731100	BDKRB1	0	0
chr14.fa	96747595	96829678	ATG2B	6.548082998	3268
chr14.fa	96846022	96853563	C14orf129	13.08304492	1216
chr14.fa	96858448	96955112	AK7	1.276524807	152
chr14.fa	96968720	97033448	PAPOLA	38.50034399	7806
chr14.fa	97263684	97347951	VRK1	7.939806385	608
chr14.fa	98098984	98152995	LOC100129345	0	0
chr14.fa	98391947	98444461	C14orf64	0	0
chr14.fa	99177950	99184103	C14orf177	0	0
chr14.fa	99635625	99737822	BCL11B	0	0
chr14.fa	99864083	99947226	SETD3	54.78226223	8129
chr14.fa	99947739	99977602	CCNK	26.43963087	2801
chr14.fa	99977853	100070727	CCDC85C	19.01866288	4636
chr14.fa	100111480	100143011	HHIPL1	0	0
chr14.fa	100150755	100193638	CYP46A1	0	0
chr14.fa	100259745	100408395	EML1	8.226468488	1672
chr14.fa	100531751	100610573	EVL	16.29904235	1350
chr14.fa	100612753	100626012	DEGS2	2.406049109	152
chr14.fa	100705102	100744804	YY1	32.60364447	3800
chr14.fa	100757453	100772860	SLC25A29	7.844623002	836
chr14.fa	100774196	100774293	MIR345	0	0
chr14.fa	100789679	100796715	SLC25A47	0	0
chr14.fa	100800125	100842680	WARS	21.63976417	2888
chr14.fa	100842755	100996640	WDR25	2.305750731	228
chr14.fa	101003484	101036131	BEGAIN	0	0
chr14.fa	101123605	101139081	C14orf70	0	0

chr14.fa	101193202	101201467	DLK1	0	0
chr14.fa	101292445	101327360	MEG3	0	0
chr14.fa	101318727	101318824	MIR770	0	0
chr14.fa	101335397	101335485	MIR493	0	0
chr14.fa	101340830	101340922	MIR337	0	0
chr14.fa	101341370	101341441	MIR665	0	0
chr14.fa	101346992	101351184	RTL1	0	0
chr14.fa	101361107	101373305	MEG8	0	0
chr14.fa	101391158	101391227	SNORD113-1	0	0
chr14.fa	101393679	101393749	SNORD113-2	0	0
chr14.fa	101402828	101402901	SNORD113-4	0	0
chr14.fa	101404524	101404600	SNORD113-5	0	0
chr14.fa	101405893	101405966	SNORD113-6	0	0
chr14.fa	101407463	101407538	SNORD113-7	0	0
chr14.fa	101411986	101412056	SNORD113-9	0	0
chr14.fa	101416170	101416240	SNORD114-1	0	0
chr14.fa	101418193	101418269	SNORD114-2	0	0
chr14.fa	101419686	101419759	SNORD114-3	0	0
chr14.fa	101420681	101420784	SNORD114-4	0	0
chr14.fa	101421707	101421775	SNORD114-5	0	0
chr14.fa	101423503	101423573	SNORD114-6	0	0
chr14.fa	101429391	101429466	SNORD114-7	0	0
chr14.fa	101431118	101431188	SNORD114-8	0	0
chr14.fa	101432366	101432436	SNORD114-9	0	0
chr14.fa	101433389	101433459	SNORD114-10	0	0
chr14.fa	101434448	101434521	SNORD114-11	0	0
chr14.fa	101435285	101435358	SNORD114-12	0	0
chr14.fa	101436216	101436288	SNORD114-13	0	0
chr14.fa	101438440	101438513	SNORD114-14	0	0
chr14.fa	101439007	101439077	SNORD114-15	0	0
chr14.fa	101439932	101440000	SNORD114-16	0	0
chr14.fa	101441143	101441216	SNORD114-17	0	0
chr14.fa	101442162	101442232	SNORD114-18	0	0
chr14.fa	101442814	101442887	SNORD114-19	0	0
chr14.fa	101447341	101447411	SNORD114-20	0	0
chr14.fa	101448312	101448382	SNORD114-21	0	0
chr14.fa	101449263	101449333	SNORD114-22	0	0
chr14.fa	101450213	101450283	SNORD114-23	0	0
chr14.fa	101451114	101451184	SNORD114-24	0	0
chr14.fa	101452394	101452464	SNORD114-25	0	0
chr14.fa	101453383	101453453	SNORD114-26	0	0
chr14.fa	101454498	101454566	SNORD114-27	0	0
chr14.fa	101455467	101455537	SNORD114-28	0	0

chr14.fa	101456428	101456496	SNORD114-29	0	0
chr14.fa	101458256	101458326	SNORD114-30	0	0
chr14.fa	101459573	101459646	SNORD114-31	0	0
chr14.fa	101488403	101488469	MIR379	0	0
chr14.fa	101489662	101489757	MIR411	0	0
chr14.fa	101490131	101490193	MIR299	0	0
chr14.fa	101491354	101491414	MIR380	0	0
chr14.fa	101491901	101491988	MIR1197	0	0
chr14.fa	101492069	101492154	MIR323	0	0
chr14.fa	101492357	101492444	MIR758	0	0
chr14.fa	101493122	101493201	MIR329-1	0	0
chr14.fa	101493437	101493520	MIR329-2	0	0
chr14.fa	101495971	101496051	MIR494	0	0
chr14.fa	101496389	101496466	MIR1193	0	0
chr14.fa	101498324	101498401	MIR543	0	0
chr14.fa	101500092	101500173	MIR495	0	0
chr14.fa	101506027	101506092	MIR376C	0	0
chr14.fa	101506406	101506485	MIR376A2	0	0
chr14.fa	101506556	101506636	MIR654	0	0
chr14.fa	101506773	101506872	MIR376B	0	0
chr14.fa	101507119	101507186	MIR376A1	0	0
chr14.fa	101507700	101507781	MIR300	0	0
chr14.fa	101509314	101509398	MIR1185-1	0	0
chr14.fa	101510535	101510619	MIR1185-2	0	0
chr14.fa	101512257	101512331	MIR381	0	0
chr14.fa	101512792	101512875	MIR487B	0	0
chr14.fa	101513658	101513735	MIR539	0	0
chr14.fa	101514238	101514316	MIR889	0	0
chr14.fa	101515887	101515983	MIR655	0	0
chr14.fa	101518783	101518862	MIR487A	0	0
chr14.fa	101520643	101520718	MIR382	0	0
chr14.fa	101521024	101521096	MIR134	0	0
chr14.fa	101521595	101521660	MIR668	0	0
chr14.fa	101521756	101521828	MIR485	0	0
chr14.fa	101522556	101522637	MIR323B	0	0
chr14.fa	101526092	101526175	MIR154	0	0
chr14.fa	101526910	101527011	MIR496	0	0
chr14.fa	101528387	101528455	MIR377	0	0
chr14.fa	101530832	101530915	MIR541	0	0
chr14.fa	101531637	101531715	MIR409	0	0
chr14.fa	101531784	101531874	MIR412	0	0
chr14.fa	101531935	101532004	MIR369	0	0
chr14.fa	101532249	101532328	MIR410	0	0

chr14.fa	101533061	101533138	MIR656	0	0
chr14.fa	102018560	102022013	DIO3OS	0	0
chr14.fa	102026624	102026759	MIR1247	0	0
chr14.fa	102027688	102029789	DIO3	0	0
chr14.fa	102196774	102198862	NCRNA00239	0	0
chr14.fa	102228135	102394328	PPP2R5C	20.78155698	4705
chr14.fa	102430865	102517135	DYNC1H1	28.75894746	18544
chr14.fa	102547075	102606086	HSP90AA1	307.0022145	56240
chr14.fa	102606212	102690010	WDR20	12.42009711	1824
chr14.fa	102695178	102771531	RAGE	0.87399695	76
chr14.fa	102786096	102809044	ZNF839	5.09231098	760
chr14.fa	102814619	102829253	CINP	21.12715272	1064
chr14.fa	102829300	102968818	TECPR2	5.218851505	2128
chr14.fa	102973198	102976128	ANKRD9	17.67097293	1292
chr14.fa	103005981	103006063	MIR4309	0	0
chr14.fa	103059233	103196913	RCOR1	17.45592075	4332
chr14.fa	103243816	103377837	TRAF3	7.607554109	2660
chr14.fa	103388993	103397179	AMN	3.256694995	228
chr14.fa	103398716	103523742	CDC42BPB	41.61737739	12540
chr14.fa	103566481	103576894	C14orf73	0.655386517	76
chr14.fa	103592664	103603776	TNFAIP2	28.33818353	5320
chr14.fa	103653558	103655365	LOC100131366	0	0
chr14.fa	103800493	103811361	EIF5	48.39385603	13224
chr14.fa	103804186	103804311	SNORA28	0	0
chr14.fa	103851701	103970166	MARK3	27.47441657	4361
chr14.fa	103985996	103989170	CKB	670.8720524	43168
chr14.fa	103995509	104003410	TRMT61A	2.086028343	304
chr14.fa	104022881	104029151	BAG5	11.90259306	2812
chr14.fa	104029299	104057236	C14orf153	21.89707065	1216
chr14.fa	104095525	104167888	KLC1	16.91528803	2402
chr14.fa	104163954	104181823	XRCC3	3.016957409	358
chr14.fa	104182081	104200005	ZFYVE21	32.31831672	2190
chr14.fa	104200088	104313927	PPP1R13B	15.67812645	3496
chr14.fa	104378625	104387903	C14orf2	117.3046237	4858
chr14.fa	104394817	104519004	TDRD9	2.483441205	532
chr14.fa	104552048	104578925	ASPG	0	0
chr14.fa	104583742	104583851	MIR203	0	0
chr14.fa	104605060	104647235	KIF26A	1.000537475	304
chr14.fa	105046056	105056183	C14orf180	0	0
chr14.fa	105060420	105071097	TMEM179	0	0
chr14.fa	105155943	105185947	INF2	16.81854791	3562
chr14.fa	105190534	105213647	ADSSL1	4.066865704	380
chr14.fa	105219470	105225996	SIVA1	26.30619622	912

chr14.fa	105235687	105262080	AKT1	102.8743336	13216
chr14.fa	105266933	105271048	ZBTB42	10.22665387	1672
chr14.fa	105287538	105290055	MGC23270	1.342574958	152
chr14.fa	105331654	105363087	KIAA0284	11.11799733	3340
chr14.fa	105391187	105399573	PLD4	0	0
chr14.fa	105403591	105444694	AHNAK2	0.092292299	76
chr14.fa	105452616	105461855	C14orf79	0.894234538	76
chr14.fa	105475910	105487425	CDCA4	1.37793514	152
chr14.fa	105515737	105531754	GPR132	0	0
chr14.fa	105608076	105635161	JAG2	0	0
chr14.fa	105639276	105647660	NUDT14	26.66113239	1037
chr14.fa	105675623	105767329	BRF1	9.060657434	1824
chr14.fa	105714879	105717430	BTBD6	37.42708464	3344
chr14.fa	105781081	105864484	PACS2	8.007858055	2280
chr14.fa	105864920	105880196	LOC647310	0	0
chr14.fa	105886186	105937057	MTA1	12.79527086	1634
chr14.fa	105941131	105946500	CRIP2	37.74755018	2047
chr14.fa	105953257	105955124	CRIP1	3.536018193	76
chr14.fa	105956192	105965585	C14orf80	2.483218814	228
chr14.fa	105992953	105996539	TMEM121	0	0
chr14.fa	106383838	106398502	KIAA0125	0	0
chr14.fa	106435819	106438358	ADAM6	0	0
chr14.fa	106744269	106744966	NCRNA00226	0	0
chr14.fa	106938455	106951529	NCRNA00221	0	0
chr15.fa	20613650	20711433	HERC2P3	0	0
chr15.fa	20737094	20747114	GOLGA6L6	0	0
chr15.fa	20767674	20781026	GOLGA8C	0	0
chr15.fa	20874797	20961480	BCL8	37.01099092	912
chr15.fa	21040701	22083137	POTEB	0	0
chr15.fa	21122021	22145802	NF1P1	0	0
chr15.fa	21932514	21940739	LOC646214	0	0
chr15.fa	22014420	22016878	CXADRP2	0	0
chr15.fa	22278032	22371088	LOC727924	0	0
chr15.fa	22368478	22369561	OR4M2	0	0
chr15.fa	22382473	22383423	OR4N4	0	0
chr15.fa	22413462	22414395	OR4N3P	0	0
chr15.fa	22546565	22570831	REREP3	0	0
chr15.fa	22702285	22715728	GOLGA8DP	0	0
chr15.fa	22736246	22746002	GOLGA6L1	0	0
chr15.fa	22833395	22873891	TUBGCP5	10.10233726	1748
chr15.fa	22892684	23003603	CYFIP1	32.73218652	7524
chr15.fa	23004684	23034427	NIPA2	13.99217966	2204
chr15.fa	23043279	23086843	NIPA1	3.334754264	988

chr15.fa	23187729	23208357	WHAMML1	2.355343942	380
chr15.fa	23255242	23262743	GOLGA8IP	0	0
chr15.fa	23282265	23378259	HERC2P2	0	0
chr15.fa	23390722	23393943	HERC2P7	0.958060778	76
chr15.fa	23435070	23448423	GOLGA8E	0	0
chr15.fa	23810454	23813166	MKRN3	0.622917419	76
chr15.fa	23888696	23892993	MAGEL2	0	0
chr15.fa	23930554	23932450	NDN	2.672918406	228
chr15.fa	24409926	24415053	PWRN2	0	0
chr15.fa	24803304	24832926	PWRN1	1.175336865	76
chr15.fa	24920541	24928593	C15orf2	0	0
chr15.fa	25068794	25165271	SNRPN	7.0791529	212
chr15.fa	25200070	25213976	SNURF	0.444782163	2
chr15.fa	25227216	25228937	PAR-SN	0	0
chr15.fa	25230007	25233379	PAR5	0	0
chr15.fa	25296623	25296719	SNORD116-1	0	0
chr15.fa	25299356	25299452	SNORD116-2	0	0
chr15.fa	25304684	25304781	SNORD116-4	0	0
chr15.fa	25315578	25315674	SNORD116-8	0	0
chr15.fa	25319260	25319363	SNORD116-10	0	0
chr15.fa	25321075	25321168	SNORD116-11	0	0
chr15.fa	25322197	25322290	SNORD116-12	0	0
chr15.fa	25324204	25324297	SNORD116-13	0	0
chr15.fa	25325288	25325381	SNORD116-14	0	0
chr15.fa	25326433	25326526	SNORD116-15	0	0
chr15.fa	25327914	25328007	SNORD116-16	0	0
chr15.fa	25330531	25330624	SNORD116-18	0	0
chr15.fa	25332808	25332901	SNORD116-20	0	0
chr15.fa	25333950	25334043	SNORD116-21	0	0
chr15.fa	25335069	25335162	SNORD116-22	0	0
chr15.fa	25336932	25337025	SNORD116-23	0	0
chr15.fa	25339183	25339276	SNORD116-24	0	0
chr15.fa	25342809	25342902	SNORD116-25	0	0
chr15.fa	25344645	25344742	SNORD116-26	0	0
chr15.fa	25346721	25346814	SNORD116-27	0	0
chr15.fa	25349788	25349880	SNORD116-28	0	0
chr15.fa	25351667	25351751	SNORD116-29	0	0
chr15.fa	25361692	25367623	IPW	0.375840927	76
chr15.fa	25380789	25383200	PAR1	0	0
chr15.fa	25415870	25415951	SNORD115-1	0	0
chr15.fa	25417782	25417863	SNORD115-2	0	0
chr15.fa	25420074	25420155	SNORD115-3	0	0
chr15.fa	25421979	25422060	SNORD115-4	0	0

chr15.fa	25423885	25423887	SNORD115-5	0	0
chr15.fa	25425644	25425725	SNORD115-6	0	0
chr15.fa	25427532	25427613	SNORD115-7	0	0
chr15.fa	25429453	25429534	SNORD115-8	0	0
chr15.fa	25432683	25432763	SNORD115-10	0	0
chr15.fa	25438468	25438549	SNORD115-13	0	0
chr15.fa	25440068	25440148	SNORD115-14	0	0
chr15.fa	25444595	25444676	SNORD115-16	0	0
chr15.fa	25451434	25451490	SNORD115-20	0	0
chr15.fa	25455065	25455146	SNORD115-22	0	0
chr15.fa	25456839	25457180	PAR4	0	0
chr15.fa	25458806	25458876	SNORD115-24	0	0
chr15.fa	25460688	25460769	SNORD115-25	0	0
chr15.fa	25463764	25463845	SNORD115-26	0	0
chr15.fa	25465650	25465725	HBII-52-27	0	0
chr15.fa	25467501	25467574	HBII-52-28	0	0
chr15.fa	25470350	25470431	SNORD115-30	0	0
chr15.fa	25472256	25472337	SNORD115-31	0	0
chr15.fa	25474114	25474195	SNORD115-32	0	0
chr15.fa	25475985	25476066	SNORD115-33	0	0
chr15.fa	25477534	25477557	SNORD115-34	0	0
chr15.fa	25477560	25477615	SNORD115-21	0	0
chr15.fa	25479394	25479475	SNORD115-35	0	0
chr15.fa	25483133	25483214	SNORD115-37	0	0
chr15.fa	25484985	25485066	SNORD115-38	0	0
chr15.fa	25486893	25486974	SNORD115-39	0	0
chr15.fa	25488761	25488842	SNORD115-40	0	0
chr15.fa	25490625	25490706	SNORD115-41	0	0
chr15.fa	25496006	25496087	SNORD115-44	0	0
chr15.fa	25509674	25509726	HBII-52-45	0	0
chr15.fa	25513664	25513696	HBII-52-46	0	0
chr15.fa	25514930	25515005	SNORD115-48	0	0
chr15.fa	25582396	25684128	UBE3A	18.38773938	4297
chr15.fa	25923860	26108349	ATP10A	0	0
chr15.fa	26788694	27018935	GABRB3	0.818843961	228
chr15.fa	27111866	27194357	GABRA5	0	0
chr15.fa	27216429	27778373	GABRG3	2.530143332	228
chr15.fa	28000023	28344458	OCA2	0	0
chr15.fa	28356188	28567295	HERC2	5.842658488	4028
chr15.fa	28764757	28771064	GOLGA8G	0	0
chr15.fa	28899588	28930410	HERC2P9	0	0
chr15.fa	28982729	29003508	WHAMML2	0.322244677	76
chr15.fa	29033389	29034538	LOC100289656	0	0

chr15.fa	29034980	29101720	LOC646278	0.804833323	76
chr15.fa	29213840	29410516	APBA2	0.929372329	152
chr15.fa	29412455	29862927	FAM189A1	3.590726399	760
chr15.fa	29560358	29562017	NDNL2	3.054541502	228
chr15.fa	29992357	30114706	TJP1	55.12274298	17757
chr15.fa	30395935	32713929	FAM7A3	0	0
chr15.fa	30427990	30439395	LOC653075	0.973850545	152
chr15.fa	30488239	30506743	DKFZP434L187	0	0
chr15.fa	30653443	30685864	CHRFAM7A	0	0
chr15.fa	30918879	30931013	ARHGAP11B	0	0
chr15.fa	31196055	31235310	FAN1	7.809707602	1824
chr15.fa	31231144	31283807	MTMR10	18.13087769	3482
chr15.fa	31293551	31393924	TRPM1	0.312237078	76
chr15.fa	31357235	31357344	MIR211	0	0
chr15.fa	31619083	31670102	KLF13	17.15035541	5244
chr15.fa	31775329	31947542	OTUD7A	0	0
chr15.fa	32322691	32462384	CHRNA7	0	0
chr15.fa	32812049	32825942	LOC100288615	6.471580466	456
chr15.fa	32907691	32931868	ARHGAP11A	0.287329277	76
chr15.fa	32933870	32989298	SCG5	0	0
chr15.fa	33010205	33026870	GREM1	0	0
chr15.fa	33057747	33360085	FMN1	3.693693469	2052
chr15.fa	33603177	34158303	RYR3	0	0
chr15.fa	34158428	34331303	AVEN	2.207898655	152
chr15.fa	34261089	34357287	CHRM5	0	0
chr15.fa	34376224	34394053	C15orf24	6.420430517	304
chr15.fa	34394274	34396591	PGBD4	2.916659031	304
chr15.fa	34432875	34502297	C15orf29	3.701032375	456
chr15.fa	34517245	34522196	TMEM85	26.47254475	988
chr15.fa	34522353	34630265	SLC12A6	4.636631654	1660
chr15.fa	34633917	34635362	NOP10	107.4128907	2584
chr15.fa	34638066	34649931	C15orf55	0	0
chr15.fa	34651089	34659395	LPCAT4	0.885783677	76
chr15.fa	34671270	34729667	GOLGA8A	0.777034438	228
chr15.fa	34817484	34875771	GOLGA8B	0	0
chr15.fa	35044679	35046689	GJD2	0	0
chr15.fa	35080297	35087927	ACTC1	0	0
chr15.fa	35148552	35261995	AQR	11.31214474	2584
chr15.fa	35270542	35280497	ZNF770	13.96060013	3420
chr15.fa	35529527	35530264	LOC723972	0	0
chr15.fa	35663170	35838404	ATPBD4	1.250949832	228
chr15.fa	35664457	35664565	MIR3942	0	0
chr15.fa	36871812	37102449	C15orf41	3.08300756	351

chr15.fa	37091301	37110707	CSNK1A1P1	0	0
chr15.fa	37156644	37178734	LOC145845	0	0
chr15.fa	37183232	37393500	MEIS2	4.030393566	836
chr15.fa	38227457	38243623	TMCO5A	0	0
chr15.fa	38545052	38649450	SPRED1	0.698975169	228
chr15.fa	38746328	38777063	FAM98B	15.3652222	1064
chr15.fa	38780302	38857007	RASGRP1	1.681721357	380
chr15.fa	38988799	38992239	C15orf53	0	0
chr15.fa	39542885	39547048	C15orf54	0	0
chr15.fa	39873280	39889668	THBS1	4.670657489	1216
chr15.fa	39892232	40075039	FSIP1	1.201579012	152
chr15.fa	40092931	40213093	GPR176	2.400711723	304
chr15.fa	40226347	40327797	EIF2AK4	20.11683004	4986
chr15.fa	40327891	40331389	SRP14	212.614547	10765
chr15.fa	40380092	40401075	BMF	5.318260318	1140
chr15.fa	40453210	40513337	BUB1B	0	0
chr15.fa	40509629	40569688	PAK6	4.684223345	836
chr15.fa	40542868	40545110	C15orf56	0.795270507	76
chr15.fa	40573645	40574787	LOC100131244	0	0
chr15.fa	40580098	40600174	PLCB2	1.806927536	380
chr15.fa	40623653	40633168	C15orf52	13.96060013	3344
chr15.fa	40643234	40648634	PHGR1	0	0
chr15.fa	40650434	40663256	DISP2	0.671843457	152
chr15.fa	40674922	40686489	C15orf23	3.325413839	304
chr15.fa	40697686	40713512	IVD	35.1440178	7372
chr15.fa	40733411	40760441	BAHD1	14.93734176	3040
chr15.fa	40763160	40765357	CHST14	0.769028359	76
chr15.fa	40824083	40824749	MRPL42P5	0	0
chr15.fa	40845298	40857252	C15orf57	12.70075465	988
chr15.fa	40861537	40866661	RPUSD2	5.514409252	456
chr15.fa	40886447	40954881	CASC5	0.40519655	140
chr15.fa	40987327	41024356	RAD51	0.698307995	76
chr15.fa	41028086	41047458	FAM82A2	4.505198525	456
chr15.fa	41056285	41059911	GCHFR	7.111621998	228
chr15.fa	41060067	41072195	DNAJC17	14.8913068	608
chr15.fa	41062159	41064648	C15orf62	0	0
chr15.fa	41099274	41106767	ZFYVE19	11.72112194	1140
chr15.fa	41107643	41120907	PPP1R14D	1.936136754	76
chr15.fa	41136246	41149853	SPINT1	16.77429209	1900
chr15.fa	41164412	41166487	RHOV	27.75640847	2128
chr15.fa	41186628	41196173	VPS18	15.62163911	2736
chr15.fa	41221531	41231258	DLL4	0	0
chr15.fa	41245636	41248717	CHAC1	3.558257301	228

chr15.fa	41271079	41408340	INO80	15.74528856	4408
chr15.fa	41474931	41522895	EXD1	0	0
chr15.fa	41523437	41574083	CHP	132.9936473	19304
chr15.fa	41576201	41591795	LOC729082	22.3096061	1900
chr15.fa	41601466	41624819	OIP5	0	0
chr15.fa	41624926	41673248	NUSAP1	0.698307995	76
chr15.fa	41679551	41694642	NDUFAF1	18.56053725	1216
chr15.fa	41709302	41775761	RTF1	18.51405752	4180
chr15.fa	41786122	41795747	ITPKA	0	0
chr15.fa	41795840	41806085	LTK	0	0
chr15.fa	41809375	41836464	RPAP1	5.42767673	1140
chr15.fa	41851220	41871536	TYRO3	3.830686375	684
chr15.fa	41952610	42062141	MGA	16.84278854	9120
chr15.fa	41983783	41983876	MIR626	0	0
chr15.fa	42066632	42120053	MAPKBP1	5.595137214	1824
chr15.fa	42128979	42129785	JMJD7	18.87055042	577
chr15.fa	42131011	42131120	PLA2G4B	0	0
chr15.fa	42140347	42186275	SPTBN5	1.29787435	684
chr15.fa	42158693	42158749	MIR4310	0	0
chr15.fa	42191639	42264755	EHD4	48.49104093	6308
chr15.fa	42275952	42302445	PLA2G4E	0	0
chr15.fa	42359881	42386752	PLA2G4D	0.943160576	152
chr15.fa	42433332	42448839	PLA2G4F	3.872051117	608
chr15.fa	42450899	42500502	VPS39	16.7391543	3637
chr15.fa	42491768	42491864	MIR627	0	0
chr15.fa	42502726	42565755	TMEM87A	4.940862653	902
chr15.fa	42566366	42645864	GANC	2.031542528	380
chr15.fa	42646538	42704515	CAPN3	3.335421437	608
chr15.fa	42705021	42749730	ZFP106	17.76993696	8358
chr15.fa	42787835	42825256	SNAP23	38.14474066	3957
chr15.fa	42834720	42841002	LRRC57	4.89905313	532
chr15.fa	42841011	42862190	HAUS2	4.285476137	760
chr15.fa	42867857	43013196	STARD9	0	0
chr15.fa	43015760	43029417	CDAN1	3.930539971	836
chr15.fa	43036542	43213007	TTBK2	6.911025242	1748
chr15.fa	43235098	43398286	UBR1	5.673863657	1976
chr15.fa	43425722	43477341	TMEM62	3.052984764	380
chr15.fa	43477466	43488229	CCNDBP1	23.00813649	2812
chr15.fa	43489426	43513323	EPB42	0	0
chr15.fa	43524793	43559055	TGM5	0	0
chr15.fa	43568479	43594453	TGM7	0	0
chr15.fa	43619974	43622820	LCMT2	5.936729915	760
chr15.fa	43622872	43646096	ADAL	0.852202624	152

chr15.fa	43650370	43662258	ZSCAN29	11.15335751	2812
chr15.fa	43663313	43698240	TUBGCP4	9.913082449	1368
chr15.fa	43699412	43802707	TP53BP1	13.02989345	3724
chr15.fa	43809806	43823818	MAP1A	0.65805521	304
chr15.fa	43825660	43882451	PPIP5K1	8.57228662	2280
chr15.fa	43885252	43891604	CKMT1B	0	0
chr15.fa	43891761	43910998	STRC	0	0
chr15.fa	43922772	43941039	CATSPER2	0.633814582	76
chr15.fa	43985084	43991420	CKMT1A	0.950054699	76
chr15.fa	44028146	44038496	CATSPER2P1	1.033673746	76
chr15.fa	44038590	44064797	PDIA3	9.429826629	1292
chr15.fa	44064805	44069293	ELL3	0	0
chr15.fa	44069503	44088287	SERF2	198.4317782	24341
chr15.fa	44088353	44092255	SERINC4	0	0
chr15.fa	44092619	44092692	C15orf63	0	0
chr15.fa	44096733	44116951	MFAP1	23.75915117	2204
chr15.fa	44119112	44160617	WDR76	0	0
chr15.fa	44165730	44487429	FRMD5	0	0
chr15.fa	44168024	44170369	LOC728758	0	0
chr15.fa	44580909	44707959	CASC4	20.31942832	3641
chr15.fa	44719579	44819429	CTDSPL2	5.201505001	1140
chr15.fa	44826703	44829098	LOC645212	7.316666575	456
chr15.fa	44829266	44854893	EIF3J	20.00607928	2204
chr15.fa	44855002	44955876	SPG11	23.10776769	7980
chr15.fa	44957930	44969086	PATL2	0	0
chr15.fa	45003685	45010357	B2M	160.2476743	7112
chr15.fa	45028560	45060025	TRIM69	0	0
chr15.fa	45248903	45271421	C15orf43	0	0
chr15.fa	45315302	45367287	SORD	213.4969948	27821
chr15.fa	45384852	45406359	DUOX2	0	0
chr15.fa	45406523	45410301	DUOXA2	1.434867257	76
chr15.fa	45409686	45422057	DUOXA1	0	0
chr15.fa	45422192	45457774	DUOX1	0.595340925	152
chr15.fa	45459412	45493373	SHF	2.028206661	228
chr15.fa	45544428	45568132	SLC28A2	0	0
chr15.fa	45653322	45670897	GATM	73.51426301	8274
chr15.fa	45670981	45672321	LOC145663	0	0
chr15.fa	45694519	45713616	SPATA5L1	6.553642775	760
chr15.fa	45722763	45725647	C15orf48	0	0
chr15.fa	45774680	45815002	SLC30A4	26.9402332	5106
chr15.fa	45803334	45848928	HMGN2P46	25.9477018	2169
chr15.fa	45879417	45901909	PLDN	27.44750725	4864
chr15.fa	45927256	45983479	SQRDL	69.96267744	5260

chr15.fa	47476403	48066420	SEMA6D	0.517281655	152
chr15.fa	48413169	48431372	SLC24A5	0	0
chr15.fa	48431629	48470558	MYEF2	7.144313487	1586
chr15.fa	48483867	48495951	CTXN2	0	0
chr15.fa	48498498	48596275	SLC12A1	4.572805414	988
chr15.fa	48623621	48635570	DUT	11.76493298	1021
chr15.fa	48700503	48937985	FBN1	0.134991386	71
chr15.fa	49030135	49103343	CEP152	0.299783178	76
chr15.fa	49115934	49255641	SHC4	0	0
chr15.fa	49170290	49172380	EID1	75.98102488	7144
chr15.fa	49280835	49338760	SECISBP2L	28.7131349	9265
chr15.fa	49417471	49447854	COPS2	17.16658996	3171
chr15.fa	49447976	49622002	GALK2	10.98233877	1520
chr15.fa	49448495	49450822	LOC100306975	0	0
chr15.fa	49623977	49913118	C15orf33	0	0
chr15.fa	49715375	49779523	FGF7	0	0
chr15.fa	49913226	49937333	DTWD1	2.458533404	304
chr15.fa	50150435	50411419	ATP8B4	1.488685898	380
chr15.fa	50474393	50528589	SLC27A2	7.775681767	836
chr15.fa	50534146	50558162	HDC	0	0
chr15.fa	50569389	50638592	GABPB1	4.071980699	520
chr15.fa	50641135	50646370	FLJ10038	0.645601309	152
chr15.fa	50647077	50650503	LOC100129387	0	0
chr15.fa	50716579	50793277	USP8	22.28002809	5443
chr15.fa	50821994	50838902	USP50	0	0
chr15.fa	50849352	50979012	TRPM7	4.384440168	2051
chr15.fa	50999737	51057910	SPPL2A	5.906707119	532
chr15.fa	51200946	51298097	AP4E1	4.300153948	1292
chr15.fa	51236326	51238762	LOC100132724	0	0
chr15.fa	51348799	51397473	TNFAIP8L3	0.74856838	76
chr15.fa	51500254	51630795	CYP19A1	0	0
chr15.fa	51633713	51700209	GLDN	0	0
chr15.fa	51739921	51914967	DMXL2	8.932560171	4256
chr15.fa	51973550	52013223	SCG3	0	0
chr15.fa	52015261	52043650	LYSMD2	1.802924496	152
chr15.fa	52043758	52108558	TMOD2	0.736114479	304
chr15.fa	52121825	52204331	TMOD3	18.11152966	3800
chr15.fa	52230222	52263958	LEO1	19.67138071	1900
chr15.fa	52311411	52358462	MAPK6	14.00152009	2660
chr15.fa	52401822	52404972	BCL2L10	0	0
chr15.fa	52413123	52483565	GNB5	11.02837372	1572
chr15.fa	52484515	52587995	MYO5C	32.47065461	10184
chr15.fa	52569314	52569397	MIR1266	0	0

chr15.fa	52599480	52821247	MYO5A	8.433514585	4636
chr15.fa	52839432	52861213	ARPP19	24.88445004	5776
chr15.fa	52873518	52970820	KIAA1370	17.63516796	3344
chr15.fa	53049353	53082209	ONECUT1	0	0
chr15.fa	53805938	54051859	WDR72	6.243629607	2052
chr15.fa	54305101	54920806	UNC13C	2.283956405	836
chr15.fa	55473512	55489231	RSL24D1	53.83887926	3566
chr15.fa	55495800	55582001	RAB27A	65.5150782	8664
chr15.fa	55611133	55647176	PIGB	1.843177282	152
chr15.fa	55647438	55700574	CCPG1	1.067254799	304
chr15.fa	55665138	55665232	MIR628	0	0
chr15.fa	55700723	55710910	FLJ27352	0	0
chr15.fa	55709954	55800432	DYX1C1	0	0
chr15.fa	55838221	55881050	PYGO1	2.682703614	152
chr15.fa	55903739	56035177	PRTG	1.411738584	760
chr15.fa	56119122	56285835	NEDD4	3.999925988	1368
chr15.fa	56382731	56535483	RFX7	8.926555612	2888
chr15.fa	56657644	56738072	TEX9	2.390704124	152
chr15.fa	56720929	56757335	MNS1	3.341870779	304
chr15.fa	56922374	57025787	ZNF280D	3.634759833	988
chr15.fa	57178368	57210697	LOC145783	0.560203134	76
chr15.fa	57210833	57580714	TCF12	15.10635898	3342
chr15.fa	57592563	57599967	LOC283663	0	0
chr15.fa	57668705	57842921	CGNL1	51.98369286	16872
chr15.fa	57884102	57977562	GCOM1	31.01243629	3344
chr15.fa	57998901	58001556	GRINL1A	26.35022966	1064
chr15.fa	58245627	58357906	ALDH1A2	3.497322145	532
chr15.fa	58430408	58478110	AQP9	0.559535961	76
chr15.fa	58724175	58861073	LIPC	1.053688943	76
chr15.fa	58888510	59042177	ADAM10	11.19027443	1976
chr15.fa	58983302	58985324	HSP90AB4P	0	0
chr15.fa	59063393	59149734	FAM63B	10.15526634	2280
chr15.fa	59171244	59225852	SLTM	36.68096256	6840
chr15.fa	59279865	59389253	RNF111	18.3150175	4560
chr15.fa	59397320	59417244	CCNB2	1.117070401	76
chr15.fa	59428563	59665071	MYO1E	26.16809136	5548
chr15.fa	59463382	59463461	MIR2116	0	0
chr15.fa	59499042	59500709	LDHAL6B	0	0
chr15.fa	59730372	59815751	FAM81A	3.411479187	532
chr15.fa	59903982	59912210	GCNT3	0	0
chr15.fa	59930261	59949737	GTF2A2	21.36755748	1520
chr15.fa	59955062	59981642	BNIP2	8.729517114	988
chr15.fa	60296421	60298142	FOXB1	0	0

chr15.fa	60639350	60690185	ANXA2	123.3338683	10049
chr15.fa	60711808	60771344	NARG2	8.63188743	2736
chr15.fa	60780483	61521502	RORA	2.197891056	1140
chr15.fa	62144590	62352664	VPS13C	6.307678239	4839
chr15.fa	62359176	62363116	C2CD4A	0.490594725	76
chr15.fa	62455737	62457482	C2CD4B	0	0
chr15.fa	62929371	62937380	MGC15885	0	0
chr15.fa	62939510	63136829	TLN2	5.803740049	3040
chr15.fa	63116156	63116240	MIR190	0	0
chr15.fa	63334838	63364113	TPM1	34.80286988	5241
chr15.fa	63413999	63434260	LACTB	8.570952273	760
chr15.fa	63445539	63449741	RPS27L	99.78865731	4864
chr15.fa	63481728	63559973	RAB8B	7.253285117	1590
chr15.fa	63569749	63601325	APH1B	2.418503009	456
chr15.fa	63615730	63674075	CA12	5.527530326	988
chr15.fa	63796810	63883663	USP3	21.110251	1926
chr15.fa	63879909	63893026	LOC100130855	2.378917397	152
chr15.fa	63889552	63894620	FBXL22	0	0
chr15.fa	63900817	64126147	HERC1	16.67377132	11394
chr15.fa	64199235	64338521	DAPK2	8.360792701	988
chr15.fa	64364761	64386207	FAM96A	49.52894011	2432
chr15.fa	64388177	64436433	SNX1	25.07148094	9268
chr15.fa	64443916	64449680	SNX22	6.044367199	842
chr15.fa	64452303	64455354	PPIB	66.63036948	1534
chr15.fa	64457716	64648442	CSNK1G1	6.418651388	2356
chr15.fa	64657211	64673702	KIAA0101	1.13730799	76
chr15.fa	64680003	64747502	TRIP4	14.87685138	1368
chr15.fa	64791619	64978266	ZNF609	20.00674646	7752
chr15.fa	64979773	64995462	OAZ2	59.98532397	5168
chr15.fa	65032095	65067770	RBPMS2	3.390574425	304
chr15.fa	65054586	65054714	MIR1272	0	0
chr15.fa	65107833	65117838	PIF1	0	0
chr15.fa	65134082	65160201	PLEKHO2	3.179525289	532
chr15.fa	65204101	65251041	ANKDD1A	1.629237062	228
chr15.fa	65255363	65282251	SPG21	55.22259657	4636
chr15.fa	65293850	65321977	MTFMT	6.770474079	836
chr15.fa	65337708	65345674	OSTBETA	1.920569378	76
chr15.fa	65345735	65360388	RASL12	0	0
chr15.fa	65369154	65372276	KBTBD13	0	0
chr15.fa	65385342	65398697	LOC390595	0	0
chr15.fa	65409717	65426174	PDCD7	5.335606823	684
chr15.fa	65442784	65477563	CLPX	33.78898894	3572
chr15.fa	65488337	65503840	CILP	0	0

chr15.fa	65550437	65579018	PARP16	5.336051605	608
chr15.fa	65619465	65670378	IGDCC3	1.509368269	304
chr15.fa	65673825	65715410	IGDCC4	0	0
chr15.fa	65737998	65810035	DPP8	16.123131	3116
chr15.fa	65822827	65870693	PTPLAD1	4.214978164	608
chr15.fa	65871118	65903469	C15orf44	21.82990854	2508
chr15.fa	65914270	65948598	SLC24A1	3.809336832	988
chr15.fa	65952957	66084631	DENND4A	8.279842348	2732
chr15.fa	66161796	66181792	RAB11A	110.1825493	11856
chr15.fa	66187634	66546075	MEGF11	0	0
chr15.fa	66332571	66332670	MIR4311	0	0
chr15.fa	66585633	66626236	DIS3L	11.88702569	2052
chr15.fa	66629008	66649054	TIPIN	2.64089409	152
chr15.fa	66639544	66639680	SCARNA14	0	0
chr15.fa	66679211	66783882	MAP2K1	32.15752796	3427
chr15.fa	66782666	66790146	SNAPC5	14.85216597	380
chr15.fa	66791653	66797184	RPL4	762.4660431	49096
chr15.fa	66793590	66793656	SNORD18C	0	0
chr15.fa	66794360	66794429	SNORD18B	0	0
chr15.fa	66795149	66795248	SNORD16	0	0
chr15.fa	66795583	66795652	SNORD18A	0	0
chr15.fa	66797431	66841822	ZWILCH	1.118627139	152
chr15.fa	66840537	66857835	LCTL	0	0
chr15.fa	66994674	67074337	SMAD6	6.95995128	988
chr15.fa	67358195	67487533	SMAD3	12.75991068	3800
chr15.fa	67493367	67547074	AAGAB	34.45682935	4408
chr15.fa	67547138	67794142	IQCH	2.844159539	608
chr15.fa	67813522	67819641	C15orf61	3.909412818	228
chr15.fa	67835021	68099452	MAP2K5	6.312348452	671
chr15.fa	68117941	68126174	SKOR1	0	0
chr15.fa	68346572	68480404	PIAS1	22.15126365	2271
chr15.fa	68483043	68498448	CALML4	3.070776051	640
chr15.fa	68499330	68522080	CLN6	3.769306437	380
chr15.fa	68570141	68583640	FEM1B	26.41917089	3040
chr15.fa	68594042	68724492	ITGA11	0	0
chr15.fa	68871573	69020144	CORO2B	0	0
chr15.fa	69070875	69113261	ANP32A	95.26922575	10564
chr15.fa	69094189	69094264	MIR4312	0	0
chr15.fa	69096160	69099440	C15orf28	0	0
chr15.fa	69116303	69489862	MIR548H4	0	0
chr15.fa	69237938	69239150	SPESP1	0	0
chr15.fa	69267068	69349501	NOX5	0	0
chr15.fa	69373190	69388163	NCRNA00277	1.338127136	152

chr15.fa	69452973	69564544	GLCE	2.010860157	456
chr15.fa	69591294	69699976	PAQR5	4.242109876	1064
chr15.fa	69706688	69740764	KIF23	0.934264933	152
chr15.fa	69745159	69747884	RPLP1	2987.511495	68780
chr15.fa	69854059	69863779	LOC145837	14.86951248	1140
chr15.fa	70127573	70135306	C15orf50	0	0
chr15.fa	70340543	70390256	TLE3	26.94890645	6460
chr15.fa	70371711	70371807	MIR629	0	0
chr15.fa	70946893	71055850	UACA	9.680238987	3161
chr15.fa	71123889	71146498	LARP6	20.93767552	2204
chr15.fa	71173681	71184772	THAP10	2.476991864	228
chr15.fa	71184928	71342436	LRRC49	4.597490824	608
chr15.fa	71402583	71407839	CT62	0	0
chr15.fa	71433788	72075722	THSD4	0.924034943	380
chr15.fa	72102894	72110597	NR2E3	0	0
chr15.fa	72118361	72410422	MYO9A	13.04635039	5092
chr15.fa	72406599	72433311	SENP8	2.209455393	228
chr15.fa	72452147	72490136	GRAMD2	2.053781636	304
chr15.fa	72491370	72523684	PKM2	207.6276494	26524
chr15.fa	72533522	72563628	PARP6	12.16123389	1436
chr15.fa	72577068	72612525	CELF6	0.472803439	76
chr15.fa	72635778	72668453	HEXA	8.40771722	896
chr15.fa	72668521	72671129	C15orf34	0	0
chr15.fa	72690668	72700708	TMEM202	0	0
chr15.fa	72766667	72878896	ARIH1	24.017792	5671
chr15.fa	72879558	72879654	MIR630	0	0
chr15.fa	72947038	72959738	GOLGA6B	0	0
chr15.fa	72968123	72978490	HIGD2B	0	0
chr15.fa	72978526	73030817	BBS4	18.31234881	2052
chr15.fa	73043708	73076126	ADPGK	6.097296276	760
chr15.fa	73344825	73597547	NEO1	5.484386456	1748
chr15.fa	73612200	73661605	HCN4	0.233733026	76
chr15.fa	73735499	73852343	C15orf60	0	0
chr15.fa	73852354	73925753	NPTN	2.76632266	304
chr15.fa	73976622	74006859	CD276	5.466595169	836
chr15.fa	74032141	74043816	C15orf59	3.772642303	228
chr15.fa	74165968	74181556	TBC1D21	0	0
chr15.fa	74218789	74244478	LOXL1	0	0
chr15.fa	74275560	74284635	STOML1	1.702181336	152
chr15.fa	74287014	74340155	PML	7.187234965	2280
chr15.fa	74362198	74374891	GOLGA6A	0	0
chr15.fa	74418714	74421619	LOC283731	0	0
chr15.fa	74421715	74429143	ISLR2	0	0

chr15.fa	74466087	74469212	ISLR	0	0
chr15.fa	74471808	74502046	STRA6	0	0
chr15.fa	74528667	74628482	CCDC33	0	0
chr15.fa	74630103	74660081	CYP11A1	0	0
chr15.fa	74701630	74726299	SEMA7A	1.470894612	228
chr15.fa	74738318	74753529	UBL7	22.34741259	1444
chr15.fa	74833548	74890472	ARID3B	1.992624088	380
chr15.fa	74900713	74922542	CLK3	11.0588413	1368
chr15.fa	74922899	74988386	EDC3	14.95357631	2736
chr15.fa	75011883	75017877	CYP1A1	9.721158946	1140
chr15.fa	75041184	75048941	CYP1A2	0	0
chr15.fa	75074425	75095539	CSK	17.50284527	1748
chr15.fa	75105194	75118099	LMAN1L	7.775904158	607
chr15.fa	75118951	75124136	CPLX3	0	0
chr15.fa	75128459	75135552	ULK3	14.11160367	1672
chr15.fa	75137197	75165670	SCAMP2	14.40115686	836
chr15.fa	75182410	75190565	MPI	22.90472464	1824
chr15.fa	75192328	75199462	C15orf17	33.80344436	5396
chr15.fa	75212617	75230495	COX5A	96.7067617	3344
chr15.fa	75247443	75249775	RPP25	2.173428038	228
chr15.fa	75287876	75313836	SCAMP5	15.75574094	2508
chr15.fa	75315927	75343067	PPCDC	6.093515628	608
chr15.fa	75494221	75504510	C15orf39	9.544580427	1900
chr15.fa	75550899	75565796	GOLGA6C	0	0
chr15.fa	75575182	75588148	GOLGA6D	0	0
chr15.fa	75628374	75632614	COMMD4	21.92664866	912
chr15.fa	75639331	75647588	NEIL1	1.786689947	152
chr15.fa	75645952	75646026	MIR631	0	0
chr15.fa	75648133	75660941	MAN2C1	14.02420398	2052
chr15.fa	75661720	75748124	SIN3A	22.72592221	7144
chr15.fa	75759462	75871625	PTPN9	20.60142021	3648
chr15.fa	75890424	75918719	SNUPN	13.04812952	1140
chr15.fa	75931426	75932664	IMP3	51.83735953	2888
chr15.fa	75941348	75950968	SNX33	17.25198813	2508
chr15.fa	75966663	76005189	CSPG4	0	0
chr15.fa	76016319	76020027	ODF3L1	0	0
chr15.fa	76025203	76032502	DNM1P35	0	0
chr15.fa	76054556	76054656	MIR4313	0	0
chr15.fa	76135622	76193388	UBE2Q2	22.88537661	3648
chr15.fa	76196200	76227608	FBXO22	4.385552123	760
chr15.fa	76225024	76225025	FBXO22OS	0	0
chr15.fa	76234328	76304785	NRG4	6.974629092	684
chr15.fa	76352299	76497304	C15orf27	0	0

chr15.fa	76508629	76603810	ETFA	43.68983988	2660
chr15.fa	76551630	76552493	TYRO3P	0	0
chr15.fa	76629147	76634816	ISL2	0	0
chr15.fa	76640527	77176217	SCAPER	6.377953821	1444
chr15.fa	77223962	77242601	RCN2	7.637576905	760
chr15.fa	77287465	77329671	PSTPIP1	0	0
chr15.fa	77336360	77363570	TSPAN3	2.193220844	380
chr15.fa	77400471	77577333	PEAK1	3.066105838	1596
chr15.fa	77516250	77517746	C15orf5	0	0
chr15.fa	77713243	77777945	HMG20A	7.3958378	1292
chr15.fa	77905369	77924709	LINGO1	0	0
chr15.fa	78206559	78219188	LOC645752	0	0
chr15.fa	78285575	78286567	LOC91450	0	0
chr15.fa	78287327	78369994	TBC1D2B	9.553698462	2584
chr15.fa	78384927	78396393	SH2D7	0	0
chr15.fa	78396991	78423878	CIB2	17.48082855	1216
chr15.fa	78441719	78462884	IDH3A	18.39218721	2204
chr15.fa	78463187	78527049	ACSBG1	0	0
chr15.fa	78556487	78574538	DNAJA4	61.65214512	9348
chr15.fa	78575578	78591940	WDR61	54.8129522	2933
chr15.fa	78632666	78640572	CRABP1	4.378658	152
chr15.fa	78730518	78793798	IREB2	14.60019688	4180
chr15.fa	78799906	78829715	AGPHD1	3.873607854	228
chr15.fa	78832747	78841563	PSMA4	67.72720229	3487
chr15.fa	78857862	78887611	CHRNA5	0	0
chr15.fa	78885395	78913637	CHRNA3	0	0
chr15.fa	78916636	78933587	CHRNB4	0	0
chr15.fa	79044379	79045734	LOC646938	0	0
chr15.fa	79051545	79103773	ADAMTS7	0	0
chr15.fa	79165172	79190074	MORF4L1	67.66782387	5909
chr15.fa	79214092	79237420	CTSH	37.87386832	2529
chr15.fa	79252289	79383215	RASGRF1	0	0
chr15.fa	79502130	79502213	MIR184	0	0
chr15.fa	79575146	79590581	ANKRD34C	0	0
chr15.fa	79603491	79615189	TMED3	28.00726561	1748
chr15.fa	79724858	79764642	KIAA1024	0.752126637	228
chr15.fa	80189133	80189627	MTHFS	29.88380155	516
chr15.fa	80191182	80207598	ST20	0	0
chr15.fa	80215113	80217196	C15orf37	3.307622552	228
chr15.fa	80253232	80263643	BCL2A1	1.792249724	76
chr15.fa	80352021	80430710	ZFAND6	29.21040136	2204
chr15.fa	80445341	80478682	FAH	16.06063911	1045
chr15.fa	80555410	80634147	LOC283688	0	0

chr15.fa	80696692	80890277	ARNT2	4.896829219	1444
chr15.fa	80987652	81047962	FAM108C1	12.89112142	1368
chr15.fa	81071712	81243999	KIAA1199	0	0
chr15.fa	81134319	81134414	MIR549	0	0
chr15.fa	81268095	81282205	MESDC2	1.991734524	380
chr15.fa	81293295	81296345	MESDC1	9.41759512	1292
chr15.fa	81426644	81441516	C15orf26	0	0
chr15.fa	81489219	81605006	IL16	0.205044577	76
chr15.fa	81605105	81616524	STARD5	2.775218304	152
chr15.fa	81624760	81666418	TMC3	0	0
chr15.fa	82334128	82338361	MEX3B	0	0
chr15.fa	82422561	82555104	EFTUD1	8.255824111	1368
chr15.fa	82555152	82577267	FAM154B	0	0
chr15.fa	82633123	83018198	GOLGA6L10	0	0
chr15.fa	82647543	83084257	UBE2Q2P2	0	0
chr15.fa	82707817	83084729	UBE2Q2P3	0	0
chr15.fa	82722185	83108111	GOLGA6L9	0	0
chr15.fa	82753447	83145927	LOC440297	0	0
chr15.fa	82763613	83142538	LOC727849	0	0
chr15.fa	82769979	83146651	LOC80154	0	0
chr15.fa	83211951	83316728	CPEB1	0	0
chr15.fa	83328033	83378635	AP3B2	0.456124108	76
chr15.fa	83379223	83382745	LOC338963	0	0
chr15.fa	83394650	83408532	LOC283693	0	0
chr15.fa	83424697	83424823	SCARNA15	0	0
chr15.fa	83428024	83474806	FSD2	0	0
chr15.fa	83477973	83503613	WHAMM	10.313164	1976
chr15.fa	83517736	83621473	HOMER2	116.2046774	10184
chr15.fa	83654995	83659423	FAM103A1	2.267944247	98
chr15.fa	83657715	83680393	C15orf40	3.861153954	304
chr15.fa	83685181	83736106	BTBD1	29.79640185	4408
chr15.fa	83776324	83806111	TM6SF1	0	0
chr15.fa	83806816	83876321	HDGFRP3	14.56305757	1292
chr15.fa	83924655	83953468	BNC1	0	0
chr15.fa	84116091	84287493	SH3GL3	0	0
chr15.fa	84322838	84708593	ADAMTSL3	0	0
chr15.fa	84748939	84795353	LOC648809	0	0
chr15.fa	84860600	84878025	LOC440300	0	0
chr15.fa	84867600	84898920	LOC388152	0.694304956	76
chr15.fa	85045806	85047737	DNM1P41	0	0
chr15.fa	85050250	85060078	GOLGA6L5	0.658277601	142
chr15.fa	85070427	85114026	UBE2Q2P1	2.358457417	228
chr15.fa	85144249	85166947	ZSCAN2	6.521396068	1292

chr15.fa	85174691	85185694	SCAND2	2.623992368	532
chr15.fa	85186012	85197521	WDR73	14.68114723	1216
chr15.fa	85198360	85201802	NMB	1.641023789	76
chr15.fa	85212775	85259674	SEC11A	86.76943863	5396
chr15.fa	85291818	85349663	ZNF592	10.53911334	3876
chr15.fa	85359911	85416713	ALPK3	1.393280124	684
chr15.fa	85427913	85489027	SLC28A1	0.991864223	152
chr15.fa	85525205	85682372	PDE8A	18.77002965	3306
chr15.fa	85923871	86292586	AKAP13	18.14355398	10947
chr15.fa	86302559	86338189	KLHL25	3.67323349	608
chr15.fa	86313727	86313809	MIR1276	0	0
chr15.fa	86685242	87572283	AGBL1	0.967845986	152
chr15.fa	88120160	88122917	NCRNA00052	0	0
chr15.fa	88419988	88799661	NTRK3	0	0
chr15.fa	89002709	89010633	MRPL46	6.760688871	304
chr15.fa	89010684	89021861	MRPS11	15.76441419	1064
chr15.fa	89055714	89089912	DET1	0.721436668	76
chr15.fa	89151338	89151428	MIR1179	0	0
chr15.fa	89155056	89155165	MIR7-2	0	0
chr15.fa	89164527	89175512	AEN	5.424118473	760
chr15.fa	89182039	89198879	ISG20	8.839823091	380
chr15.fa	89346674	89418585	ACAN	0	0
chr15.fa	89420519	89438770	HAPLN3	0	0
chr15.fa	89441914	89456663	MFGE8	0	0
chr15.fa	89631381	89745591	ABHD2	7.402509532	3040
chr15.fa	89753098	89764922	RLBP1	0	0
chr15.fa	89787194	89860362	FANCI	2.348449819	456
chr15.fa	89859536	89878026	POLG	9.655108795	1744
chr15.fa	89911248	89911337	MIR9-3	0	0
chr15.fa	89921273	89941718	LOC254559	0.402527857	76
chr15.fa	90014638	90039799	RHCG	3.49020563	304
chr15.fa	90048161	90067265	LOC283761	0	0
chr15.fa	90118818	90171200	C15orf42	0.255527352	76
chr15.fa	90171254	90198682	KIF7	2.251042525	456
chr15.fa	90207600	90222648	PLIN1	0	0
chr15.fa	90226287	90233958	PEX11A	16.02750284	836
chr15.fa	90234028	90286869	WDR93	0.701643861	76
chr15.fa	90293098	90294540	MESP1	37.81804816	1976
chr15.fa	90319589	90321982	MESP2	1.049241122	76
chr15.fa	90328126	90358072	ANPEP	56.95657983	9348
chr15.fa	90420538	90437617	AP3S2	9.088011537	255
chr15.fa	90443832	90445035	C15orf38	28.85168454	1562
chr15.fa	90544752	90625432	ZNF710	11.67197351	2356

chr15.fa	90627212	90645708	IDH2	67.37248852	5244
chr15.fa	90728152	90772892	SEMA4B	5.573787671	988
chr15.fa	90773477	90777279	CIB1	202.0411854	8876
chr15.fa	90777487	90785312	C15orf58	6.255193944	380
chr15.fa	90792764	90802312	TTLL13	0	0
chr15.fa	90808895	90815443	NGRN	130.3022704	7904
chr15.fa	90889763	90892679	GABARAPL3	0	0
chr15.fa	90895477	90904715	ZNF774	0	0
chr15.fa	90931473	91045475	IQGAP1	33.7474018	10941
chr15.fa	91073198	91188577	CRTC3	8.1713155	1900
chr15.fa	91260579	91358686	BLM	0.373172234	76
chr15.fa	91411885	91426687	FURIN	9.704257224	1824
chr15.fa	91427665	91439006	FES	3.614077462	456
chr15.fa	91447420	91465815	MAN2A2	1.617672725	456
chr15.fa	91473410	91497323	UNC45A	20.46420491	3724
chr15.fa	91474155	91475774	HDDC3	6.469578946	304
chr15.fa	91498106	91506355	RCCD1	3.66500502	456
chr15.fa	91509268	91537804	PRC1	0.542856629	76
chr15.fa	91541774	91565833	VPS33B	8.325877302	988
chr15.fa	91643182	91844539	SV2B	1.444652464	760
chr15.fa	92396938	92715665	SLCO3A1	0	0
chr15.fa	92937140	93011958	ST8SIA2	0	0
chr15.fa	93014907	93044347	C15orf32	0	0
chr15.fa	93111048	93115493	LOC100144604	0	0
chr15.fa	93160679	93199031	FAM174B	13.50069537	1596
chr15.fa	93338714	93340338	ASB9P1	0	0
chr15.fa	93426073	93441977	LOC100507217	13.28875667	1216
chr15.fa	93443551	93571237	CHD2	46.51042596	19912
chr15.fa	93447629	93447705	MIR3175	0	0
chr15.fa	93586636	93632443	RGMA	0.434996955	76
chr15.fa	94841430	95027181	MCTP2	3.461739572	1339
chr15.fa	95822519	95870329	LOC400456	0	0
chr15.fa	95976322	96051076	LOC145820	0	0
chr15.fa	96869157	96883492	NR2F2	10.23510473	2812
chr15.fa	96876490	96876536	MIR1469	35.96108263	76
chr15.fa	97326679	97328845	SPATA8	0	0
chr15.fa	98285846	98417659	LOC91948	0	0
chr15.fa	98503933	98517068	ARRDC4	10.38944415	1900
chr15.fa	98980391	99057611	FAM169B	0	0
chr15.fa	99192761	99507759	IGF1R	11.42623137	5776
chr15.fa	99511459	99551024	PGPEP1L	0	0
chr15.fa	99645286	99675800	SYNM	0.91936473	304
chr15.fa	99676528	99789815	TTC23	5.769936604	988

chr15.fa	99791652	99926498	LRRC28	15.1532835	988
chr15.fa	100106133	100256629	MEF2A	8.568061189	2280
chr15.fa	100267612	100273626	LYSMD4	1.758668671	228
chr15.fa	100330361	100347132	C15orf51	0	0
chr15.fa	100511643	100882183	ADAMTS17	0.266869298	76
chr15.fa	100884662	100890923	FLJ42289	0	0
chr15.fa	100940600	101084925	LASS3	0	0
chr15.fa	101109429	101142445	LINS	6.057933055	684
chr15.fa	101142755	101191904	ASB7	6.129987765	1368
chr15.fa	101420009	101456830	ALDH1A3	101.5491051	15959
chr15.fa	101459460	101610317	LRRK1	2.87929733	988
chr15.fa	101715928	101792137	CHSY1	2.971812019	608
chr15.fa	101811214	101817700	SELS	2.710057717	228
chr15.fa	101821715	101835460	SNRPA1	14.11204845	672
chr15.fa	101844133	102030187	PCSK6	0.252636268	76
chr15.fa	102182049	102192594	TM2D3	4.804981703	304
chr15.fa	102193955	102264645	TARSL2	7.183454317	1064
chr15.fa	102345923	102346861	OR4F6	0	0
chr15.fa	102358390	102359328	OR4F15	0	0
chr15.fa	102382322	102390527	GPCRLTM7	0	0
chr15.fa	102462345	102463262	OR4F4	0	0
chr15.fa	102495088	102496558	FAM138E	0	0
chr15.fa	102501016	102516760	WASH3P	0.67451215	76
chr15.fa	102516809	102519296	DDX11L9	0	0
chr16.fa	96979	103632	POLR3K	18.46068366	684
chr16.fa	103829	107669	SNRNP25	29.57023012	1444
chr16.fa	108058	122629	RHBDF1	2.259493386	304
chr16.fa	127018	135803	MPG	13.78202009	836
chr16.fa	135844	188672	NPRL3	8.868956322	1064
chr16.fa	202854	204504	HBZ	0	0
chr16.fa	215973	216767	HBM	0	0
chr16.fa	222846	223709	HBA2	0	0
chr16.fa	226679	227520	HBA1	0	0
chr16.fa	230333	231178	HBQ1	0	0
chr16.fa	238970	279449	LUC7L	6.141996883	575
chr16.fa	284801	316119	ITFG3	4.007932067	532
chr16.fa	318310	325914	RGS11	0	0
chr16.fa	330606	333003	ARHGDIG	0	0
chr16.fa	333118	337209	PDIA2	0	0
chr16.fa	337440	402676	AXIN1	6.9270374	1140
chr16.fa	417384	420569	MRPL28	23.72178947	1216
chr16.fa	420776	431950	TMEM8A	18.02968974	2964
chr16.fa	432241	442960	LOC100134368	0	0

chr16.fa	447192	450754	NME4	224.9087707	10184
chr16.fa	451858	462487	DECR2	17.0831933	1216
chr16.fa	475668	572481	RAB11FIP3	21.96401036	4393
chr16.fa	576847	577407	NCRNA00235	3.012731978	76
chr16.fa	577856	604636	SOLH	4.987787171	1064
chr16.fa	593277	593366	MIR3176	0	0
chr16.fa	610422	615528	C16orf11	3.813562262	380
chr16.fa	617032	619495	NHLRC4	1.653700081	152
chr16.fa	620004	634109	PIGQ	3.470635215	456
chr16.fa	639357	679273	RAB40C	8.067681256	1064
chr16.fa	681012	684116	WFIKKN1	0	0
chr16.fa	684429	686347	C16orf13	22.13324998	836
chr16.fa	691849	698474	FAM195A	29.71723063	1216
chr16.fa	699363	717829	WDR90	1.525158036	380
chr16.fa	718133	724171	RHOT2	13.39350287	1499
chr16.fa	726075	728267	RHBDL1	1.084156521	76
chr16.fa	730115	731603	STUB1	31.44387498	1322
chr16.fa	731667	734439	JMJD8	12.29222224	760
chr16.fa	734702	740400	WDR24	1.60210535	228
chr16.fa	742500	755825	FBXL16	0.477028869	76
chr16.fa	765173	767480	METRN	10.8344487	532
chr16.fa	771158	772581	FAM173A	15.7690844	602
chr16.fa	772591	776473	CCDC78	0	0
chr16.fa	776958	779715	HAGHL	0	0
chr16.fa	779769	790997	NARFL	14.54949171	1368
chr16.fa	810765	818865	MSLN	0	0
chr16.fa	819428	832926	MSLNL	0	0
chr16.fa	834974	838383	RPUSD1	9.069108295	836
chr16.fa	838622	848040	CHTF18	0.553086619	76
chr16.fa	848075	850733	GNG13	0	0
chr16.fa	855443	863861	PRR25	0	0
chr16.fa	903635	1031318	LMF1	0.361607898	76
chr16.fa	1031808	1036979	SOX8	0.554420966	76
chr16.fa	1114082	1128731	LOC146336	0	0
chr16.fa	1122756	1131454	SSTR5	0	0
chr16.fa	1138226	1146244	C1QTNF8	0	0
chr16.fa	1203241	1271650	CACNA1H	1.272966549	456
chr16.fa	1271773	1275254	TPSG1	0	0
chr16.fa	1278336	1280185	TPSB2	0	0
chr16.fa	1290678	1292555	TPSAB1	0	0
chr16.fa	1306273	1308494	TPSD1	0	0
chr16.fa	1359180	1375390	UBE2I	35.23341901	2584
chr16.fa	1383606	1399240	BAIAP3	1.40061903	304

chr16.fa	1399443	1401873	C16orf42	36.9542812	1665
chr16.fa	1401900	1413205	GNPTG	9.235901606	456
chr16.fa	1413353	1464705	UNKL	2.873515161	760
chr16.fa	1469944	1479345	C16orf91	0	0
chr16.fa	1484389	1494490	CCDC154	1.542726931	152
chr16.fa	1494935	1525085	CLCN7	3.200430051	608
chr16.fa	1535940	1538468	PTX4	0	0
chr16.fa	1543352	1560427	TELO2	6.155340348	912
chr16.fa	1560461	1662109	IFT140	10.00871061	2356
chr16.fa	1584231	1605243	TMEM204	0	0
chr16.fa	1664641	1727909	CRAMP1L	10.79352874	3724
chr16.fa	1728278	1752073	HN1L	85.35770004	14136
chr16.fa	1756221	1820318	MAPK8IP3	11.963973	3032
chr16.fa	1784986	1785067	MIR3177	0	0
chr16.fa	1820321	1821710	NME3	33.33664548	1520
chr16.fa	1821896	1823140	MRPS34	57.16095724	2578
chr16.fa	1823229	1826239	EME2	0	0
chr16.fa	1826713	1832581	SPSB3	17.514632	1216
chr16.fa	1832933	1839192	NUBP2	23.23675452	1444
chr16.fa	1840414	1844909	IGFALS	0	0
chr16.fa	1859104	1877195	HAGH	32.54359888	2356
chr16.fa	1877225	1890203	FAHD1	14.48522069	1672
chr16.fa	1883984	1922179	C16orf73	0.990085094	76
chr16.fa	1928286	1934232	NCRNA00254	0	0
chr16.fa	1961465	1968231	HS3ST6	3.183083547	152
chr16.fa	1988234	1993294	SEPX1	43.27441334	2660
chr16.fa	1994580	2004679	RPL3L	0	0
chr16.fa	2009517	2011976	NDUFB10	87.29717266	2736
chr16.fa	2012062	2014827	RPS2	358.8475801	15200
chr16.fa	2012335	2012467	SNORA10	0	0
chr16.fa	2012974	2013107	SNORA64	0	0
chr16.fa	2014997	2015505	SNHG9	50.77766603	532
chr16.fa	2015185	2015311	SNORA78	0	0
chr16.fa	2016875	2018976	RNF151	0	0
chr16.fa	2022064	2028751	TBL3	10.42502672	1216
chr16.fa	2028918	2031184	NOXO1	0	0
chr16.fa	2034150	2037750	GFER	7.717637694	836
chr16.fa	2039946	2044276	SYNGR3	0	0
chr16.fa	2047768	2059763	ZNF598	4.758057184	684
chr16.fa	2069521	2070756	NPW	0	0
chr16.fa	2076888	2089027	SLC9A3R2	33.33308722	3260
chr16.fa	2089816	2097867	NTHL1	3.168183344	152
chr16.fa	2097990	2138710	TSC2	11.71022478	2964

chr16.fa	2138714	2185899	PKD1	1.435089648	912
chr16.fa	2140196	2140285	MIR1225	0	0
chr16.fa	2185978	2186130	MIR3180-5	0	0
chr16.fa	2198651	2204141	RAB26	2.081580521	152
chr16.fa	2205024	2205106	SNORD60	0	0
chr16.fa	2205799	2227183	TRAF7	27.85603967	3407
chr16.fa	2228131	2246465	CASKIN1	1.067921972	228
chr16.fa	2255178	2259253	MLST8	19.65314464	1672
chr16.fa	2259419	2261069	C16orf79	0	0
chr16.fa	2261603	2264822	PGP	4.850571874	684
chr16.fa	2273567	2285743	E4F1	9.853259248	1140
chr16.fa	2286468	2288712	DNASE1L2	0	0
chr16.fa	2289873	2301602	DCI	46.6365217	2204
chr16.fa	2303100	2318413	RNPS1	29.62805181	3268
chr16.fa	2320714	2320773	MIR3677	0	0
chr16.fa	2321748	2321841	MIR940	0	0
chr16.fa	2325879	2390747	ABCA3	1.276080024	380
chr16.fa	2390923	2476700	ABCA17P	0	0
chr16.fa	2479395	2508859	CCNF	1.18267577	228
chr16.fa	2510115	2514964	C16orf59	0	0
chr16.fa	2521500	2524146	NTN3	0	0
chr16.fa	2525147	2555734	TBC1D24	6.407087052	1900
chr16.fa	2563727	2570224	ATP6V0C	42.56320666	2356
chr16.fa	2570363	2579732	AMDHD2	6.398191409	456
chr16.fa	2580036	2581409	CEMP1	7.50080639	228
chr16.fa	2581923	2582006	MIR3178	0	0
chr16.fa	2587970	2653189	PDPK1	18.17602307	5914
chr16.fa	2653385	2680495	LOC652276	0.622695028	76
chr16.fa	2688983	2696130	FLJ42627	0	0
chr16.fa	2732495	2759031	KCTD5	19.21436703	2128
chr16.fa	2762423	2770552	PRSS27	2.312200072	152
chr16.fa	2787077	2799660	LOC100128788	1.13730799	76
chr16.fa	2802330	2821413	SRRM2	41.99166158	17360
chr16.fa	2821415	2827297	TCEB2	80.24048126	3572
chr16.fa	2833954	2836708	PRSS33	0	0
chr16.fa	2848486	2855133	PRSS41	0	0
chr16.fa	2867164	2871718	PRSS21	0	0
chr16.fa	2880173	2882285	ZG16B	114.3112397	4256
chr16.fa	2889574	2892752	PRSS30P	0	0
chr16.fa	2902728	2908171	PRSS22	1.219370299	76
chr16.fa	2933196	2949383	FLYWCH2	29.73902496	1368
chr16.fa	2961980	3001209	FLYWCH1	6.016123531	1368
chr16.fa	3014217	3018381	KREMEN2	1.645694002	152

chr16.fa	3019342	3023485	PAQR4	1.444652464	152
chr16.fa	3024001	3030540	PKMYT1	0	0
chr16.fa	3039055	3044510	LOC283875	0	0
chr16.fa	3062457	3064506	CLDN9	0.824403738	76
chr16.fa	3064713	3068188	CLDN6	0	0
chr16.fa	3070313	3072383	TNFRSF12A	4.913286159	228
chr16.fa	3072626	3074287	HCFC1R1	29.82664704	1140
chr16.fa	3074288	3077756	THOC6	7.26017924	380
chr16.fa	3077868	3085542	CCDC64B	39.19776243	3192
chr16.fa	3096682	3110724	MMP25	0	0
chr16.fa	3115313	3119668	IL32	0	0
chr16.fa	3138895	3142861	ZSCAN10	0	0
chr16.fa	3160461	3165599	MGC3771	0	0
chr16.fa	3162563	3170518	ZNF205	5.433236507	451
chr16.fa	3185057	3192805	ZNF213	5.563112899	836
chr16.fa	3254247	3255185	OR1F1	0	0
chr16.fa	3265562	3266546	OR1F2P	0	0
chr16.fa	3272325	3285457	ZNF200	3.696362162	608
chr16.fa	3292028	3306627	MEFV	0	0
chr16.fa	3313768	3317566	FLJ39639	0	0
chr16.fa	3333487	3341459	ZNF263	13.89188128	2052
chr16.fa	3348808	3355432	TIGD7	0.987638792	152
chr16.fa	3355440	3368576	ZNF75A	7.185455837	684
chr16.fa	3405889	3406924	OR2C1	0	0
chr16.fa	3421053	3422283	MTRNR2L4	0	0
chr16.fa	3432085	3451025	ZNF434	6.141996883	836
chr16.fa	3451190	3459364	ZNF174	6.453566788	760
chr16.fa	3486110	3493490	ZNF597	4.766508045	380
chr16.fa	3493668	3536963	NAT15	14.14362799	1824
chr16.fa	3543484	3545421	C16orf90	0	0
chr16.fa	3550963	3586585	CLUAP1	13.5200434	1056
chr16.fa	3589036	3627392	NLRC3	0	0
chr16.fa	3631184	3661585	SLX4	2.086250734	684
chr16.fa	3702940	3708037	DNASE1	0.556200094	76
chr16.fa	3708097	3767598	TRAP1	18.13332399	1824
chr16.fa	3775056	3930121	CREBBP	26.19144243	12008
chr16.fa	4012650	4166186	ADCY9	1.093719338	380
chr16.fa	4239375	4292081	SRL	0	0
chr16.fa	4307187	4323001	TFAP4	7.897996861	760
chr16.fa	4382225	4389598	GLIS2	3.649437644	608
chr16.fa	4390252	4401300	PAM16	6.615245104	152
chr16.fa	4404543	4466639	CORO7	4.328842397	684
chr16.fa	4421849	4433529	VASN	3.011620023	380

chr16.fa	4475858	4506775	DNAJA3		21.20521199	2584
chr16.fa	4511695	4524896	NMRAL1		16.46272218	912
chr16.fa	4526341	4560348	HMOX2		22.60249516	2052
chr16.fa	4560677	4588816	C16orf5		8.196223301	1064
chr16.fa	4606491	4650318	LOC342346		0	0
chr16.fa	4658884	4664927	FAM100A		14.35400995	912
chr16.fa	4674826	4740975	MGRN1		10.27958295	2992
chr16.fa	4743694	4745860	NUDT16L1		22.12368716	1343
chr16.fa	4746513	4784163	ANKS3		5.714338834	684
chr16.fa	4784289	4799397	C16orf71		1.249170704	152
chr16.fa	4800815	4817166	ZNF500		3.638095699	532
chr16.fa	4827672	4838397		12-Sep	0	0
chr16.fa	4838400	4846282	LOC440335		20.84293692	836
chr16.fa	4846969	4852674	ROGDI		26.50990646	1738
chr16.fa	4853204	4897303	GLYR1		38.01264035	6379
chr16.fa	4897912	4932363	UBN1		14.14674146	4311
chr16.fa	4932508	4987136	PPL		118.3649843	33201
chr16.fa	5008318	5069156	SEC14L5		0.261754303	76
chr16.fa	5074845	5083942	NAGPA		3.060546061	304
chr16.fa	5094123	5116111	C16orf89		2.232806456	152
chr16.fa	5121810	5137380	ALG1		1.07770718	143
chr16.fa	5134301	5147789	FAM86A		7.962045493	498
chr16.fa	6069132	7763340	RBFOX1		0	0
chr16.fa	8619502	8622226	TMEM114		0	0
chr16.fa	8715527	8740079	C16orf68		4.306158507	304
chr16.fa	8768444	8878432	ABAT		46.52221269	12405
chr16.fa	8889037	8891505	TMEM186		9.337979113	608
chr16.fa	8891670	8943194	PMM2		16.00682047	1672
chr16.fa	8946802	8962863	CARHSP1		13.28230733	1824
chr16.fa	8985951	9057341	USP7		65.45569979	15929
chr16.fa	9185537	9213555	C16orf72		17.84043493	3192
chr16.fa	9328774	9328803	MIR548X		0	0
chr16.fa	9847265	10276611	GRIN2A		0	0
chr16.fa	10522725	10577495	ATF7IP2		1.879427028	304
chr16.fa	10622279	10674539	EMP2		4.567245637	1064
chr16.fa	10721361	10788802	TEKT5		2.100928545	152
chr16.fa	10837698	10863208	NUBP1		13.91723387	684
chr16.fa	10860533	10912621	FAM18A		0	0
chr16.fa	10971055	11018840	CIITA		1.089493907	228
chr16.fa	11022748	11036257	DEXI		13.51225971	912
chr16.fa	11038345	11276046	CLEC16A		6.622361619	2052
chr16.fa	11348274	11350039	SOCS1		0	0
chr16.fa	11361714	11363160	TNP2		0	0

chr16.fa	11367144	11367452	PRM3	0	0
chr16.fa	11369493	11370337	PRM2	0	0
chr16.fa	11374693	11375192	PRM1	0	0
chr16.fa	11439311	11445617	C16orf75	1.180229468	76
chr16.fa	11641578	11681322	LITAF	108.0184617	14212
chr16.fa	11762289	11772942	SNN	15.74684529	2280
chr16.fa	11773016	11836648	TXNDC11	15.91274904	2128
chr16.fa	11844442	11891114	ZC3H7A	15.84002716	2718
chr16.fa	11913692	11922689	BCAR4	0	0
chr16.fa	11928055	11945442	RSL1D1	24.0277996	5548
chr16.fa	11961985	12010519	GSPT1	27.39991556	9578
chr16.fa	12058964	12061925	TNFRSF17	0	0
chr16.fa	12070602	12147141	RUNDC2A	9.033748113	903
chr16.fa	12155385	12668146	SNX29	13.07948666	4104
chr16.fa	12753656	12897744	CPPED1	8.254044982	2280
chr16.fa	12995477	13334273	SHISA9	1.707518722	760
chr16.fa	14014014	14046205	ERCC4	3.999925988	1216
chr16.fa	14165196	14360630	MKL2	14.0635672	5460
chr16.fa	14397824	14397906	MIR193B	0	0
chr16.fa	14403142	14403228	MIR365-1	0	0
chr16.fa	14529558	14724124	PARN	16.95754234	2347
chr16.fa	14726668	14763093	BFAR	9.44228053	1292
chr16.fa	14766405	14788526	PLA2G10	0	0
chr16.fa	14916289	14918559	ABCC6P2	0	0
chr16.fa	14927643	14990014	NOMO1	0.397857644	76
chr16.fa	15031300	15045931	NPIP	6.11464278	303
chr16.fa	15068833	15131552	PDXDC1	84.92559417	15317
chr16.fa	15131715	15149833	NTAN1	66.91747636	3343
chr16.fa	15153879	15188158	RRN3	14.43829617	2432
chr16.fa	15248707	15248859	MIR3180-4	0	0
chr16.fa	15489611	15503543	MPV17L	2.928000976	304
chr16.fa	15528325	15682116	C16orf45	1.443318118	152
chr16.fa	15688226	15737023	KIAA0430	21.12715272	7372
chr16.fa	15737124	15820208	NDE1	3.922756283	608
chr16.fa	15796992	15950887	MYH11	0	0
chr16.fa	15959577	15982447	C16orf63	19.53082954	1976
chr16.fa	16043434	16236930	ABCC1	6.438221804	1900
chr16.fa	16243422	16317328	ABCC6	0.908912349	228
chr16.fa	16326389	16388667	NOMO3	0.783483779	152
chr16.fa	16411466	16444465	PKD1P1	2.95335356	836
chr16.fa	17196181	17564738	XYLT1	3.982801875	1672
chr16.fa	18511182	18573434	NOMO2	0	0
chr16.fa	18582570	18609607	ABCC6P1	0	0

chr16.fa	18794277	18801656	RPS15A	1017.060465	24833
chr16.fa	18802991	18812857	ARL6IP1	22.39633862	2280
chr16.fa	18816175	18937726	SMG1	11.78339144	8512
chr16.fa	18995256	19075262	TMC7	1.800255803	380
chr16.fa	19078917	19091417	COQ7	11.05083522	1368
chr16.fa	19125254	19132952	ITPRIPL2	11.85477898	4104
chr16.fa	19179638	19278554	SYT17	16.79564163	1444
chr16.fa	19297105	19315716	LOC728276	0	0
chr16.fa	19422057	19510434	TMC5	8.322986218	2052
chr16.fa	19513015	19533450	GDE1	28.77384766	3800
chr16.fa	19535179	19564728	CP110	3.932763882	988
chr16.fa	19566737	19712485	C16orf62	15.19153476	2660
chr16.fa	19717674	19726359	C16orf88	12.05537574	1050
chr16.fa	19727778	19868859	IQCK	2.662688416	304
chr16.fa	19870293	19896151	GPRC5B	3.564484251	456
chr16.fa	20043043	20085100	GPR139	0	0
chr16.fa	20321811	20338835	GP2	1.390611431	152
chr16.fa	20344373	20364037	UMOD	7.195241044	749
chr16.fa	20370492	20416033	PDILT	0	0
chr16.fa	20420856	20452281	ACSM5	0.698752777	76
chr16.fa	20462859	20498991	ACSM2A	12.5450809	1672
chr16.fa	20548083	20587695	ACSM2B	6.231620489	684
chr16.fa	20634559	20702578	ACSM1	0	0
chr16.fa	20744989	20753199	THUMPD1	25.27785986	5016
chr16.fa	20775312	20803657	ACSM3	27.26003157	2302
chr16.fa	20791515	20817766	ERI2	5.835986755	988
chr16.fa	20817796	20860990	LOC81691	8.613873752	1064
chr16.fa	20869396	20911556	DCUN1D3	4.722697002	608
chr16.fa	20911562	20936328	LYRM1	21.08311929	1824
chr16.fa	20944476	21170762	DNAH3	0	0
chr16.fa	21169912	21191937	TMEM159	40.76984498	3496
chr16.fa	21208773	21222868	ZP2	0	0
chr16.fa	21245016	21263750	ANKS4B	1.163327746	152
chr16.fa	21269839	21314404	CRYM	62.84838675	4256
chr16.fa	21312170	21329912	NCRNA00169	0	0
chr16.fa	21413455	21436658	NPIPL3	0	0
chr16.fa	21443345	21445776	LOC100190986	0	0
chr16.fa	21458004	21513552	LOC100271836	0.282881455	63
chr16.fa	21529230	21531765	SLC7A5P2	0.666506071	76
chr16.fa	21610856	21668792	METTL9	67.18367849	9652
chr16.fa	21652606	21663972	IGSF6	0	0
chr16.fa	21689835	21772050	OTOA	0	0
chr16.fa	21807951	21830495	RRN3P1	0	0

chr16.fa	21964609	21994668	UQCRC2	67.46122256	5078
chr16.fa	21995186	22012431	PDZD9	0	0
chr16.fa	22019456	22095972	C16orf52	1.14153342	228
chr16.fa	22103863	22168287	VWA3A	0	0
chr16.fa	22217592	22300066	EEF2K	9.085342844	3028
chr16.fa	22308741	22345343	POLR3E	18.81584222	2204
chr16.fa	22357257	22385938	CDR2	21.89351239	2660
chr16.fa	22430867	22449036	RRN3P3	1.684167659	152
chr16.fa	22466023	22503541	LOC641298	0	0
chr16.fa	22524844	22547841	LOC100132247	0	0
chr16.fa	22557019	22588186	LOC653786	0	0
chr16.fa	22825860	22927659	HS3ST2	0	0
chr16.fa	23072728	23160591	USP31	6.951500419	3344
chr16.fa	23194040	23228200	SCNN1G	25.11840546	3952
chr16.fa	23313591	23392620	SCNN1B	10.4130176	1216
chr16.fa	23399814	23464512	COG7	10.9340799	1444
chr16.fa	23474863	23521815	GGA2	14.4127212	3871
chr16.fa	23533334	23568696	EARS2	4.692451815	836
chr16.fa	23568862	23585710	UBFD1	19.53750127	4332
chr16.fa	23592335	23607639	NDUFAB1	48.14588997	1444
chr16.fa	23614483	23652678	PALB2	4.981560221	909
chr16.fa	23652687	23685068	DCTN5	12.69830835	4484
chr16.fa	23690201	23701625	PLK1	0	0
chr16.fa	23701689	23724821	ERN2	0.95027709	152
chr16.fa	23765948	23770256	CHP2	0.714320153	76
chr16.fa	23847300	24231932	PRKCB	0	0
chr16.fa	24266874	24373737	CACNG3	0	0
chr16.fa	24550908	24584183	RBBP6	10.7746255	3312
chr16.fa	24741049	24837547	TNRC6A	17.5097394	6631
chr16.fa	24857552	24922944	SLC5A11	0.719212757	76
chr16.fa	24930712	25026675	ARHGAP17	14.02242485	2193
chr16.fa	25123047	25189551	LCMT1	19.73920999	1216
chr16.fa	25228285	25240253	AQP8	0	0
chr16.fa	25247322	25268855	ZKSCAN2	2.134731989	684
chr16.fa	25703347	26149009	HS3ST4	4.221427505	608
chr16.fa	27078219	27080487	C16orf82	0	0
chr16.fa	27214807	27233089	JMJD5	2.530143332	304
chr16.fa	27236315	27280113	NSMCE1	52.51943298	2508
chr16.fa	27279526	27301789	FLJ21408	0	0
chr16.fa	27325251	27376099	IL4R	0.875776078	152
chr16.fa	27413483	27463363	IL21R	0	0
chr16.fa	27458991	27464714	LOC283888	0	0
chr16.fa	27471934	27561251	GTF3C1	16.64730678	5320

chr16.fa	27561468	27791692	KIAA0556	10.71836055	3192
chr16.fa	27798850	28074830	GSG1L	5.050279065	1140
chr16.fa	28109316	28223190	XPO6	22.55090043	4484
chr16.fa	28303840	28335170	SBK1	2.031542528	456
chr16.fa	28390900	28747053	EIF3CL	0	0
chr16.fa	28415059	28722784	EIF3C	0	0
chr16.fa	28488600	28503623	CLN3	7.02155361	604
chr16.fa	28505993	28510282	APOB48R	6.33881299	1064
chr16.fa	28510683	28518155	IL27	0	0
chr16.fa	28548662	28550495	NUPR1	116.7553177	4788
chr16.fa	28565249	28603111	CCDC101	4.405344929	228
chr16.fa	28603264	28608391	SULT1A2	0	0
chr16.fa	28616916	28634866	SULT1A1	1.680831792	152
chr16.fa	28834414	28848558	ATXN2L	24.53262735	4332
chr16.fa	28853732	28857729	TUFM	31.82838916	2964
chr16.fa	28875078	28885534	SH2B1	5.663856059	1292
chr16.fa	28889809	28915741	ATP2A1	0	0
chr16.fa	28915831	28936532	RABEP2	27.69992113	2729
chr16.fa	28943260	28950668	CD19	0	0
chr16.fa	28962318	28977767	NFATC2IP	9.200096642	1596
chr16.fa	28986096	28995869	SPNS1	1.527604337	152
chr16.fa	28996147	29002104	LAT	0	0
chr16.fa	29086163	29128038	RRN3P2	0	0
chr16.fa	29302541	29385597	RUNDC2C	0	0
chr16.fa	29460666	30200575	LOC606724	0	0
chr16.fa	29476281	29476288	SLX1B-SULT1A4	0	0
chr16.fa	29538929	29577540	LOC440354	0.436331302	76
chr16.fa	29624424	29625038	SLC7A5P1	0	0
chr16.fa	29674300	29681823	SPN	0	0
chr16.fa	29690441	29709314	QPRT	44.59274767	3116
chr16.fa	29753786	29757340	C16orf54	0.651161086	76
chr16.fa	29789561	29791840	ZG16	0	0
chr16.fa	29802058	29816706	KIF22	61.97461219	5841
chr16.fa	29817855	29822504	MAZ	227.9848842	28489
chr16.fa	29823409	29827202	PRRT2	1.316777592	152
chr16.fa	29827528	29833816	C16orf53	15.49776728	2584
chr16.fa	29841795	29859360	MVP	30.87966881	3899
chr16.fa	29869678	29874578	CDIPT	18.0381406	1520
chr16.fa	29875004	29879374	LOC440356	0	0
chr16.fa	29882480	29910580	SEZ6L2	1.427528351	228
chr16.fa	29912147	29917377	ASPHD1	1.14353494	76
chr16.fa	29917661	29937545	KCTD13	0.986749228	76
chr16.fa	29973351	29984373	TMEM219	130.2735819	5852

chr16.fa	29985222	30003581	TAOK2		11.92638891	3496
chr16.fa	30004311	30007412	HIRIP3		3.559814038	380
chr16.fa	30007531	30016834	INO80E		13.04434887	532
chr16.fa	30017113	30022401	DOC2A		0	0
chr16.fa	30034655	30035747	C16orf92		0	0
chr16.fa	30036024	30042186	FAM57B		0	0
chr16.fa	30064491	30081735	ALDOA		110.9162175	13900
chr16.fa	30087384	30096695	PPP4C		56.02387164	3353
chr16.fa	30097115	30103205	TBX6		0	0
chr16.fa	30103635	30107537	YPEL3		57.41559502	2432
chr16.fa	30116131	30124878	GDPD3		4.273689409	211
chr16.fa	30125426	30134630	MAPK3		25.09038418	1672
chr16.fa	30194731	30199897	CORO1A		0	0
chr16.fa	30234350	30256932	LOC613037		0	0
chr16.fa	30346296	30346695	LOC595101		0	0
chr16.fa	30362087	30366682	CD2BP2		13.89944258	2280
chr16.fa	30368422	30381522	TBC1D10B		19.53372063	2888
chr16.fa	30386123	30389310	MYLPF		0	0
chr16.fa	30389456	30394171		1-Sep	1.063029369	76
chr16.fa	30407057	30410715	ZNF48		5.370522222	532
chr16.fa	30418735	30429916	ZNF771		1.161548618	76
chr16.fa	30435019	30441373	DCTPP1		42.25430545	2128
chr16.fa	30454952	30457224	SEPHS2		45.35888494	4636
chr16.fa	30483983	30534506	ITGAL		0	0
chr16.fa	30535322	30537910	ZNF768		18.98241314	1976
chr16.fa	30542779	30546194	ZNF747		10.69278558	1140
chr16.fa	30565085	30569642	ZNF764		2.418058227	304
chr16.fa	30581019	30583728	ZNF688		4.6255121	380
chr16.fa	30591994	30597092	ZNF785		3.623640279	532
chr16.fa	30614700	30621682	ZNF689		3.120591653	380
chr16.fa	30662241	30667734	PRR14		10.37409916	988
chr16.fa	30675778	30682131	FBRS		27.64254423	3494
chr16.fa	30710462	30751450	SRCAP		26.78722813	12616
chr16.fa	30721858	30721986	SNORA30		0	0
chr16.fa	30759620	30772497	PHKG2		8.654126538	760
chr16.fa	30768744	30773565	C16orf93		1.080598264	76
chr16.fa	30773630	30786538	RNF40		31.95025948	6080
chr16.fa	30789770	30798523	ZNF629		8.885413262	2432
chr16.fa	30899116	30905399	BCL7C		54.52161988	1900
chr16.fa	30907928	30914881	CTF1		4.062862664	304
chr16.fa	30930640	30934590	NCRNA00095		2.13895742	380
chr16.fa	30935896	30960104	FBXL19		6.678848954	1140
chr16.fa	30960405	30966259	ORAI3		6.179803367	608

chr16.fa	30968615	30995981	SETD1A	8.91365693	2584
chr16.fa	30996519	31000473	HSD3B7	2.843047583	304
chr16.fa	31000577	31021829	STX1B	0.372060279	76
chr16.fa	31044903	31051485	STX4	23.16981481	1444
chr16.fa	31072164	31085641	ZNF668	3.006727419	532
chr16.fa	31085743	31094744	ZNF646	6.905465465	1900
chr16.fa	31094834	31100130	PRSS53	0	0
chr16.fa	31102175	31106276	VKORC1	20.32276418	912
chr16.fa	31119662	31124112	BCKDK	22.45393791	2195
chr16.fa	31128985	31142714	MYST1	9.271484179	760
chr16.fa	31142754	31147151	PRSS8	1.742434122	152
chr16.fa	31150248	31161415	PRSS36	1.191126631	152
chr16.fa	31191431	31206192	FUS	15.75729767	3627
chr16.fa	31212807	31214097	PYCARD	8.745974054	304
chr16.fa	31225342	31236510	TRIM72	0	0
chr16.fa	31227283	31228395	PYDC1	0	0
chr16.fa	31271288	31344213	ITGAM	0	0
chr16.fa	31366509	31394318	ITGAX	0	0
chr16.fa	31404633	31437826	ITGAD	0	0
chr16.fa	31439055	31439721	COX6A2	0	0
chr16.fa	31446885	31454348	ZNF843	0	0
chr16.fa	31470317	31478488	ARMC5	1.085046086	228
chr16.fa	31483476	31489281	TGFB1I1	0	0
chr16.fa	31494439	31500659	SLC5A2	0	0
chr16.fa	31500813	31519706	C16orf58	6.652384415	650
chr16.fa	31539203	31540124	AHSP	0	0
chr16.fa	31579088	31580845	CSDAP1	0	0
chr16.fa	31711934	31718745	KIAA0664L3	0.951611437	76
chr16.fa	31724550	31772886	ZNF720	6.702200017	836
chr16.fa	31885079	31928627	ZNF267	7.245056647	1064
chr16.fa	32162609	32163874	HERC2P4	0	0
chr16.fa	32300868	33298702	LOC390705	0	0
chr16.fa	32684841	33261514	TP53TG3B	0	0
chr16.fa	32888797	32896463	SLC6A10P	0	0
chr16.fa	34403802	34404762	UBE2MP1	1.758668671	76
chr16.fa	34597787	34626084	LOC283914	0	0
chr16.fa	34711785	34714967	LOC146481	0	0
chr16.fa	34739459	34740840	LOC100130700	0	0
chr16.fa	34980923	34990995	FLJ26245	0	0
chr16.fa	46503249	46603009	ANKRD26P1	0	0
chr16.fa	46614468	46655311	SHCBP1	0.522841432	76
chr16.fa	46693589	46723144	VPS35	48.36405562	7144
chr16.fa	46723558	46732306	ORC6	1.038121568	76

chr16.fa	46736194	46782221	MYLK3	0	0
chr16.fa	46835959	46865074	C16orf87	1.222038992	76
chr16.fa	46918308	46965201	GPT2	31.70829798	6004
chr16.fa	46989274	47007625	DNAJA2	32.71661914	4484
chr16.fa	47115442	47177908	NETO2	0	0
chr16.fa	47189298	47495015	ITFG1	1.51581761	152
chr16.fa	47495210	47735434	PHKB	27.55469976	7055
chr16.fa	48116884	48180681	ABCC12	0	0
chr16.fa	48200822	48269088	ABCC11	0	0
chr16.fa	48278211	48387407	LONP2	51.77175416	10108
chr16.fa	48394446	48419229	SIAH1	9.472525717	1064
chr16.fa	48572637	48644120	N4BP1	14.7467526	4712
chr16.fa	49311829	49315742	CBLN1	0	0
chr16.fa	49407808	49433319	C16orf78	0	0
chr16.fa	49524521	49860918	ZNF423	0.350710735	76
chr16.fa	50059189	50070999	TMEM188	0	0
chr16.fa	50099881	50139375	HEATR3	12.961397	1520
chr16.fa	50186829	50269219	PAPD5	2.500342927	912
chr16.fa	50321823	50352043	ADCY7	0	0
chr16.fa	50352929	50402845	BRD7	22.66231836	2356
chr16.fa	50582241	50668641	NKD1	1.307214776	152
chr16.fa	50700211	50715264	SNX20	0	0
chr16.fa	50731050	50766987	NOD2	0.753683375	152
chr16.fa	50775961	50835846	CYLD	10.94141881	4332
chr16.fa	51169886	51185183	SALL1	1.576752766	380
chr16.fa	52471918	52581714	TOX3	5.493949272	836
chr16.fa	52585999	52640887	LOC643714	0	0
chr16.fa	53088945	53361414	CHD9	17.54754588	9044
chr16.fa	53468351	53525191	RBL2	38.16631259	7752
chr16.fa	53525561	53537170	AKTIP	12.76969589	1064
chr16.fa	53633818	53737771	RPGRIP1L	2.187216285	608
chr16.fa	53737875	54148379	FTO	13.38905505	2584
chr16.fa	54317212	54320378	IRX3	10.98456268	1140
chr16.fa	54952778	54963079	CRNDE	14.03954896	827
chr16.fa	54965111	54968395	IRX5	0	0
chr16.fa	55358471	55364672	IRX6	0.725662098	76
chr16.fa	55513081	55540586	MMP2	0	0
chr16.fa	55542913	55620582	LPCAT2	0.313793816	76
chr16.fa	55600584	55601592	CAPNS2	5.025371264	228
chr16.fa	55689542	55740104	SLC6A2	0.316907291	76
chr16.fa	55758837	55784123	CES1P2	0	0
chr16.fa	55794511	55808826	CES1P1	0	0
chr16.fa	55836764	55867075	CES1	0	0

chr16.fa	55880066	55989943	CES5A	0	0
chr16.fa	56126899	56225006	LOC283856	0	0
chr16.fa	56225251	56391356	GNAO1	0.213273047	76
chr16.fa	56226529	56228437	DKFZP434H168	0	0
chr16.fa	56279432	56279535	MIR3935	0	0
chr16.fa	56395364	56459444	AMFR	8.464871727	1368
chr16.fa	56463048	56485261	NUDT21	13.42330328	2660
chr16.fa	56485424	56511407	OGFOD1	9.514112849	1292
chr16.fa	56518259	56554008	BBS2	12.61313257	1596
chr16.fa	56598961	56602869	MT4	0	0
chr16.fa	56623267	56625000	MT3	0	0
chr16.fa	56642478	56643409	MT2A	584.2676279	11008
chr16.fa	56651373	56652730	MT1L	43.64291536	836
chr16.fa	56659585	56661024	MT1E	454.1437152	10333
chr16.fa	56666534	56667898	MT1M	23.0477221	456
chr16.fa	56669651	56670998	MT1JP	0	0
chr16.fa	56672578	56673999	MT1A	12.8043889	228
chr16.fa	56677599	56678853	MT1DP	0	0
chr16.fa	56685811	56687116	MT1B	0	0
chr16.fa	56691855	56693215	MT1F	210.6412709	4177
chr16.fa	56700653	56701977	MT1G	639.6552387	11390
chr16.fa	56703726	56705041	MT1H	80.66569301	1440
chr16.fa	56709649	56711653	MT1IP	0	0
chr16.fa	56716382	56718108	MT1X	418.3594334	7600
chr16.fa	56764017	56878677	NUP93	13.9837288	1721
chr16.fa	56892430	56892513	MIR138-2	0	0
chr16.fa	56899119	56949762	SLC12A3	7.627124524	1900
chr16.fa	56965748	56977793	HERPUD1	66.79916431	6536
chr16.fa	56995835	57017756	CETP	0	0
chr16.fa	57023410	57117436	NLRC5	0.247743665	76
chr16.fa	57126455	57181878	CPNE2	4.531218281	456
chr16.fa	57186378	57219976	FAM192A	23.144907	2964
chr16.fa	57220241	57272947	RSPRY1	16.33796079	1520
chr16.fa	57279038	57287545	ARL2BP	13.68238889	1292
chr16.fa	57290009	57318584	PLLP	4.516095688	304
chr16.fa	57392695	57400102	CCL22	0	0
chr16.fa	57406414	57418956	CX3CL1	1.032784182	152
chr16.fa	57438679	57449974	CCL17	0	0
chr16.fa	57462087	57481336	CIAPIN1	16.31438733	1520
chr16.fa	57481370	57495187	COQ9	31.63980153	2356
chr16.fa	57496551	57505869	POLR2C	40.51787588	3192
chr16.fa	57505922	57520385	DOK4	11.90259306	1444
chr16.fa	57546090	57570477	CCDC102A	2.783224382	304

chr16.fa	57576601	57611100	GPR114	0.449452375	76
chr16.fa	57653910	57698944	GPR56	6.275209141	1274
chr16.fa	57702157	57723290	GPR97	0	0
chr16.fa	57728713	57765367	CCDC135	0	0
chr16.fa	57769660	57791162	KATNB1	7.026446214	836
chr16.fa	57792129	57836439	KIFC3	11.3955414	1900
chr16.fa	57916244	58005020	CNGB1	0	0
chr16.fa	58010339	58022017	TEPP	1.042569389	76
chr16.fa	58028573	58033762	ZNF319	9.21188337	1748
chr16.fa	58035277	58055527	C16orf57	20.69304533	2128
chr16.fa	58059282	58080804	MMP15	2.285513143	456
chr16.fa	58147497	58163296	C16orf80	24.87466483	1444
chr16.fa	58191812	58231782	CSNK2A2	30.46135119	2271
chr16.fa	58283840	58317734	CCDC113	5.627606312	1140
chr16.fa	58318494	58328951	PRSS54	0	0
chr16.fa	58426298	58440048	GINS3	2.120721351	228
chr16.fa	58497549	58547523	NDRG4	0.430771524	76
chr16.fa	58549383	58553854	SETD6	4.925517669	532
chr16.fa	58554432	58663750	CNOT1	53.61671057	19608
chr16.fa	58582403	58582537	SNORA46	0	0
chr16.fa	58593700	58593835	SNORA50	0	0
chr16.fa	58700298	58718674	SLC38A7	3.047647378	380
chr16.fa	58741035	58768246	GOT2	77.35984959	8512
chr16.fa	59788045	59789095	LOC644649	0	0
chr16.fa	61687235	62070036	CDH8	0	0
chr16.fa	64980683	65155919	CDH11	0	0
chr16.fa	65318402	65610203	LOC283867	0	0
chr16.fa	66400525	66438689	CDH5	0	0
chr16.fa	66460816	66527432	BEAN1	0	0
chr16.fa	66541906	66584315	TK2	10.15415438	2335
chr16.fa	66586466	66600154	CKLF	6.05081654	228
chr16.fa	66600294	66613038	CMTM1	0	0
chr16.fa	66613351	66622177	CMTM2	0	0
chr16.fa	66637935	66647795	CMTM3	1.303211736	152
chr16.fa	66648653	66730610	CMTM4	11.03593502	4028
chr16.fa	66754799	66785525	DYNC1LI2	47.48383172	9258
chr16.fa	66788879	66835523	CCDC79	0	0
chr16.fa	66836781	66864879	NAE1	22.716137	1952
chr16.fa	66878282	66888049	CA7	0	0
chr16.fa	66914383	66925002	PDP2	6.491373272	2052
chr16.fa	66942025	66952766	CDH16	10.14214526	1292
chr16.fa	66955582	66959439	RRAD	1.145091678	76
chr16.fa	66965958	66968320	FAM96B	55.77434884	1738

chr16.fa	66968347	66978994	CES2	17.02047902	3180
chr16.fa	66995132	67009052	CES3	0	0
chr16.fa	67022492	67043659	CES4A	1.943475659	228
chr16.fa	67063050	67134958	CBFB	5.993439641	836
chr16.fa	67143915	67182004	C16orf70	7.736985718	836
chr16.fa	67182443	67184902	B3GNT9	0.745454905	76
chr16.fa	67188089	67193812	TRADD	20.52847593	1368
chr16.fa	67193891	67198077	FBXL8	0	0
chr16.fa	67198344	67203848	HSF4	0.988973139	90
chr16.fa	67204405	67209504	NOL3	16.37198662	1292
chr16.fa	67209644	67217883	KIAA0895L	1.55495844	228
chr16.fa	67218282	67224107	EXOC3L	0.676958451	76
chr16.fa	67226068	67232821	E2F4	13.57653073	1282
chr16.fa	67233028	67237927	ELMO3	14.80257276	1672
chr16.fa	67236224	67236298	MIR328	0	0
chr16.fa	67241042	67260901	LRRC29	4.349524768	292
chr16.fa	67261016	67263182	TMEM208	12.77214219	456
chr16.fa	67263292	67281425	FHOD1	1.754220849	304
chr16.fa	67282855	67306094	SLC9A5	0	0
chr16.fa	67311413	67323392	PLEKHG4	0.24885562	76
chr16.fa	67323404	67360661	KCTD19	0	0
chr16.fa	67360747	67419109	LRRC36	0	0
chr16.fa	67423712	67427421	TPPP3	0	0
chr16.fa	67428322	67450339	ZDHHC1	0.841305461	76
chr16.fa	67465036	67471454	HSD11B2	19.75766844	1672
chr16.fa	67471917	67515089	ATP6V0D1	54.65238584	4104
chr16.fa	67516474	67517716	AGRP	0	0
chr16.fa	67562717	67580691	FAM65A	21.40024897	4311
chr16.fa	67596310	67673088	CTCF	34.30960646	6080
chr16.fa	67679030	67691414	RLTPR	0	0
chr16.fa	67691473	67694718	ACD	2.536592673	228
chr16.fa	67694851	67696681	PARD6A	2.676476663	152
chr16.fa	67696850	67700628	C16orf48	1.098834333	76
chr16.fa	67700717	67702661	C16orf86	1.331900186	76
chr16.fa	67708436	67753273	GFOD2	6.943271949	2128
chr16.fa	67757005	67840555	RANBP10	8.602087025	2052
chr16.fa	67841010	67861971	TSNAXIP1	0	0
chr16.fa	67862060	67881361	CENPT	6.486703059	684
chr16.fa	67876213	67878098	THAP11	14.33866497	1216
chr16.fa	67880819	67905219	NUTF2	99.19754181	3952
chr16.fa	67906999	67918406	EDC4	13.22960064	2812
chr16.fa	67918781	67920271	NRN1L	0	0
chr16.fa	67927175	67963473	PSKH1	23.55121551	3572

chr16.fa	67963582	67965778	CTRL	0	0
chr16.fa	67968407	67970753	PSMB10	37.9815056	1672
chr16.fa	67973787	67977115	LCAT	0	0
chr16.fa	67977377	68002597	SLC12A4	2.086250734	456
chr16.fa	68009566	68014452	DPEP3	0	0
chr16.fa	68021293	68033364	DPEP2	0	0
chr16.fa	68055177	68057770	DDX28	2.658462986	304
chr16.fa	68059318	68113184	DUS2L	6.30990215	532
chr16.fa	68119269	68262449	NFATC3	24.42788115	6384
chr16.fa	68263163	68270136	ESRP2	49.35236159	7341
chr16.fa	68279247	68294961	PLA2G15	1.241387016	152
chr16.fa	68298423	68335722	SLC7A6	3.953891034	988
chr16.fa	68336284	68344868	SLC7A6OS	8.067681256	304
chr16.fa	68344877	68391169	PRMT7	12.34203784	1368
chr16.fa	68392230	68482409	SMPD3	0.64159827	152
chr16.fa	68573661	68601039	ZFP90	6.554087557	1292
chr16.fa	68678151	68732957	CDH3	0.395188951	76
chr16.fa	68771195	68869444	CDH1	17.20017101	3724
chr16.fa	68877509	69119085	TMCO7	1.753998458	380
chr16.fa	69139467	69151570	HAS3	6.337256253	1292
chr16.fa	69151912	69166493	CHTF8	38.51502181	4560
chr16.fa	69166499	69202937	CIRH1A	22.49574744	2280
chr16.fa	69221050	69342955	SNTB2	12.97340612	5700
chr16.fa	69345287	69358946	VPS4A	47.38064226	4636
chr16.fa	69363900	69364498	PDF	14.10826781	380
chr16.fa	69364716	69373414	COG8	13.89944258	1140
chr16.fa	69373527	69377013	NIP7	11.48738891	1140
chr16.fa	69377149	69385712	TMED6	0	0
chr16.fa	69389464	69419874	TERF2	8.547823601	1140
chr16.fa	69458498	69500167	CYB5B	18.77292074	3618
chr16.fa	69598997	69738553	NFAT5	8.714616912	5548
chr16.fa	69743304	69760533	NQO1	29.35451078	3420
chr16.fa	69775774	69788829	NOB1	21.6688974	1672
chr16.fa	69796274	69975643	WWP2	27.39101992	6077
chr16.fa	69966984	69967083	MIR140	0	0
chr16.fa	69984626	69997889	CLEC18A	0	0
chr16.fa	70010202	70099851	PDXDC2P	4.04151312	760
chr16.fa	70147529	70195184	PDPR	5.493726881	1976
chr16.fa	70207928	70220798	CLEC18C	0	0
chr16.fa	70253484	70259936	LOC729513	0	0
chr16.fa	70284134	70285833	EXOSC6	9.942215681	760
chr16.fa	70286297	70323412	AARS	35.38041951	5320
chr16.fa	70333072	70367731	DDX19B	12.10585851	988

chr16.fa	70380824	70407281	DDX19A	8.742193406	1140
chr16.fa	70413338	70472991	ST3GAL2	2.27884141	456
chr16.fa	70488498	70514177	FUK	9.027743554	1586
chr16.fa	70514472	70557457	COG4	17.96786502	2280
chr16.fa	70557691	70611571	SF3B3	14.78033365	6460
chr16.fa	70563402	70563502	MIR3647	0	0
chr16.fa	70571908	70572001	SNORD111	0	0
chr16.fa	70613798	70694585	IL34	0	0
chr16.fa	70695107	70719954	MTSS1L	12.56509609	2812
chr16.fa	70721342	70835061	VAC14	9.476528756	1292
chr16.fa	70788963	70807154	LOC100130894	0.78148226	76
chr16.fa	70841289	71264625	HYDIN	0.409644372	304
chr16.fa	71316203	71323509	FTSJD1	8.408829175	1596
chr16.fa	71392616	71424342	CALB2	0	0
chr16.fa	71481503	71496117	ZNF23	4.696454855	684
chr16.fa	71507976	71523254	ZNF19	3.697029336	456
chr16.fa	71560023	71572493	CHST4	0	0
chr16.fa	71600754	71610998	TAT	0	0
chr16.fa	71660070	71675868	MARVELD3	2.506125095	380
chr16.fa	71678852	71748704	PHLPP2	6.61635706	2356
chr16.fa	71732470	71732604	SNORA70D	0	0
chr16.fa	71762905	71842976	AP1G1	38.48477662	11847
chr16.fa	71792305	71792390	SNORD71	0	0
chr16.fa	71879899	71919174	ATXN1L	16.91239695	6080
chr16.fa	71893588	71917412	ZNF821	5.665635187	415
chr16.fa	71929446	71962906	KIAA0174	48.92803941	5168
chr16.fa	71963441	72033877	PKD1L3	0	0
chr16.fa	72042643	72058955	DHODH	0.818176788	76
chr16.fa	72088508	72094955	HP	0	0
chr16.fa	72097125	72111145	HPR	0	0
chr16.fa	72118756	72128215	TXNL4B	1.23604963	152
chr16.fa	72127769	72146811	DHX38	16.50875714	3192
chr16.fa	72152996	72206349	PMFBP1	0	0
chr16.fa	72816786	73092534	ZFHX3	28.85257411	20976
chr16.fa	73126248	73127672	HTA	0	0
chr16.fa	74330681	74340186	PSMD7	65.08475146	4864
chr16.fa	74366304	74402153	LOC283922	0.729442747	76
chr16.fa	74442529	74455368	CLEC18B	0	0
chr16.fa	74481326	74641042	GLG1	1.811152966	684
chr16.fa	74655297	74700779	RFWD3	6.484923931	1444
chr16.fa	74705753	74734789	MLKL	2.664912327	304
chr16.fa	74746856	74808729	FA2H	0.696306476	76
chr16.fa	74907471	75019017	WDR59	16.61105704	2736

chr16.fa	75032915	75144892	ZNRF1	6.566541458	1368
chr16.fa	75145758	75150670	LDHD	21.20877025	1976
chr16.fa	75182421	75206132	ZFP1	2.056227938	304
chr16.fa	75237994	75241072	CTRB2	0	0
chr16.fa	75252884	75258822	CTRB1	0	0
chr16.fa	75262928	75301951	BCAR1	8.46331499	2052
chr16.fa	75327608	75467387	CFDP1	47.57323294	2736
chr16.fa	75480923	75498584	TMEM170A	14.43229161	684
chr16.fa	75507022	75528926	CHST6	0	0
chr16.fa	75562428	75569068	CHST5	0	0
chr16.fa	75572015	75590170	TMEM231	0	0
chr16.fa	75600249	75611779	GABARAPL2	119.474271	5383
chr16.fa	75632247	75657221	ADAT1	7.070034866	1127
chr16.fa	75661622	75681585	KARS	53.94985241	5320
chr16.fa	75681635	75691341	TERF2IP	35.69688202	3496
chr16.fa	76311176	76593135	CNTNAP4	0	0
chr16.fa	77224836	77233543	MON1B	22.87536901	3344
chr16.fa	77240338	77246976	SYCE1L	2.071350531	76
chr16.fa	77316025	77469011	ADAMTS18	0	0
chr16.fa	77756411	77776154	NUDT7	3.092792768	152
chr16.fa	77822483	78014001	VAT1L	0.448785202	76
chr16.fa	78056443	78065998	CLEC3A	2.620434111	228
chr16.fa	78133551	79246564	WWOX	6.514501944	684
chr16.fa	79627745	79634622	MAF	19.16744252	5928
chr16.fa	80574854	80584541	DYNLRB2	0	0
chr16.fa	80637676	80838175	CDYL2	47.64239656	4788
chr16.fa	81009699	81031034	C16orf61	65.85289026	1676
chr16.fa	81040103	81066709	CENPN	16.34952512	3781
chr16.fa	81069458	81080951	ATMIN	23.61926718	5168
chr16.fa	81087102	81110872	C16orf46	0	0
chr16.fa	81115552	81115928	GCSH	0	0
chr16.fa	81134484	81253975	PKD1L2	0	0
chr16.fa	81272296	81324747	BCMO1	0.696306476	76
chr16.fa	81348571	81413803	GAN	21.20165374	4332
chr16.fa	81478775	81745367	CMIP	17.13256412	3469
chr16.fa	81812930	81991899	PLCG2	7.970718745	1520
chr16.fa	82031251	82045093	SDR42E1	3.045423467	380
chr16.fa	82068858	82132139	HSD17B2	1.178672731	76
chr16.fa	82181767	82203829	MPHOSPH6	7.67560578	380
chr16.fa	82660578	83830199	CDH13	0.441668687	76
chr16.fa	83541951	83542013	MIR3182	0	0
chr16.fa	83841593	83846594	HSBP1	61.67216032	5244
chr16.fa	83932730	83949787	MLYCD	12.32024351	1216

chr16.fa	83982672	83999937	OSGIN1	3.327192967	380
chr16.fa	84002237	84036379	NECAB2	0	0
chr16.fa	84043389	84075762	SLC38A8	0	0
chr16.fa	84087369	84150517	MBTPS1	17.47504639	3415
chr16.fa	84155744	84178800	HSDL1	5.990326166	988
chr16.fa	84178865	84211460	LRRC50	0	0
chr16.fa	84211525	84220662	TAF1C	3.270038459	532
chr16.fa	84224723	84230772	ADAD2	0	0
chr16.fa	84254741	84273356	KCNG4	0	0
chr16.fa	84328401	84363450	WFDC1	0	0
chr16.fa	84402133	84497793	ATP2C2	2.496562279	380
chr16.fa	84509966	84538288	KIAA1609	5.039381902	1140
chr16.fa	84599204	84651669	COTL1	77.79417937	6384
chr16.fa	84682131	84695916	KLHL36	23.37730568	2280
chr16.fa	84733555	84813527	USP10	24.41831834	3709
chr16.fa	84853587	84943116	CRISPLD2	5.127226379	1058
chr16.fa	85008067	85045141	ZDHHC7	14.45920093	2128
chr16.fa	85061410	85127828	KIAA0513	5.746585541	1900
chr16.fa	85131965	85146114	FAM92B	0	0
chr16.fa	85170756	85183049	LOC400548	0	0
chr16.fa	85645029	85709812	KIAA0182	12.16279063	4104
chr16.fa	85711280	85722588	GINS2	0	0
chr16.fa	85741124	85784689	C16orf74	0	0
chr16.fa	85775227	85775306	MIR1910	0	0
chr16.fa	85812231	85833148	COX4NB	6.888118961	608
chr16.fa	85833196	85840607	COX4I1	305.9612018	11020
chr16.fa	85932774	85956211	IRF8	5.716562745	684
chr16.fa	86365456	86379285	LOC732275	0	0
chr16.fa	86508131	86542466	LOC400550	0	0
chr16.fa	86544133	86548070	FOXF1	0	0
chr16.fa	86563782	86588841	MTHFSD	4.625067318	684
chr16.fa	86588926	86590905	FLJ30679	0	0
chr16.fa	86600857	86602537	FOXC2	0	0
chr16.fa	86612115	86615304	FOXL1	0	0
chr16.fa	87336404	87351026	LOC100506581	2.921551635	152
chr16.fa	87362942	87425708	FBXO31	4.058637234	684
chr16.fa	87425801	87438380	MAP1LC3B	21.42271047	2204
chr16.fa	87439852	87525460	ZCCHC14	4.891269442	1520
chr16.fa	87636499	87731748	JPH3	0	0
chr16.fa	87731762	87739290	LOC100129637	1.220259863	380
chr16.fa	87741418	87799598	KLHDC4	7.005986234	608
chr16.fa	87863629	87903100	SLC7A5	1.116180837	228
chr16.fa	87921625	87970112	CA5A	0	0

chr16.fa	87985038	88110924	BANP	5.04916711	608
chr16.fa	88493879	88507165	ZNF469	0	0
chr16.fa	88520014	88601574	ZFPM1	4.693563771	684
chr16.fa	88636789	88698372	ZC3H18	14.96536303	2508
chr16.fa	88705001	88706882	IL17C	0	0
chr16.fa	88709697	88717457	CYBA	4.913286159	152
chr16.fa	88718348	88729495	MVD	24.48169979	1976
chr16.fa	88729781	88753594	MGC23284	1.196241626	152
chr16.fa	88744090	88752882	SNAI3	0	0
chr16.fa	88762903	88772829	RNF166	0.623362201	76
chr16.fa	88772891	88781745	CTU2	4.019496403	304
chr16.fa	88781787	88851372	FAM38A	3.253581519	1140
chr16.fa	88870186	88875666	CDT1	0.631813062	76
chr16.fa	88875877	88878342	APRT	96.34181794	3496
chr16.fa	88880142	88923374	GALNS	1.430641826	152
chr16.fa	88923506	88927520	TRAPPC2L	37.61900814	1064
chr16.fa	88929748	88933068	PABPN1L	0	0
chr16.fa	88941263	89043504	CBFA2T3	1.48490525	304
chr16.fa	89160254	89222169	ACSF3	4.662651411	760
chr16.fa	89225628	89230083	C16orf81	0	0
chr16.fa	89238163	89261900	CDH15	0.591115494	76
chr16.fa	89284111	89295363	ZNF778	2.892196012	380
chr16.fa	89334035	89556969	ANKRD11	15.80444458	6612
chr16.fa	89574805	89624174	SPG7	4.184732977	760
chr16.fa	89627090	89629848	RPL13	861.6884927	50448
chr16.fa	89627838	89627909	SNORD68	0	0
chr16.fa	89642176	89663654	CPNE7	0	0
chr16.fa	89679716	89704839	DPEP1	10.14281244	836
chr16.fa	89710844	89724129	CHMP1A	24.41209139	2584
chr16.fa	89724210	89737675	C16orf55	2.315313547	228
chr16.fa	89753076	89762764	CDK10	11.33082559	912
chr16.fa	89762773	89768121	SPATA2L	2.900424482	304
chr16.fa	89773541	89787392	C16orf7	9.426045981	988
chr16.fa	89778264	89784573	LOC100128881	0	0
chr16.fa	89787395	89807332	ZNF276	3.302952339	532
chr16.fa	89803959	89883065	FANCA	0	0
chr16.fa	89894907	89937727	SPIRE2	5.731240556	836
chr16.fa	89939994	89977792	TCF25	20.94946225	2128
chr16.fa	89984287	89987385	MC1R	0	0
chr16.fa	89988417	90002505	TUBB3	0.842639807	76
chr16.fa	90015151	90034467	DEF8	5.849997394	988
chr16.fa	90036183	90039240	CENPBD1	5.296465992	684
chr16.fa	90044013	90067195	AFG3L1P	1.818936654	304

chr16.fa	90071279	90085937	DBNDD1	7.367816523	760
chr16.fa	90089008	90111379	GAS8	7.232602746	735
chr16.fa	90095316	90096309	C16orf3	0	0
chr16.fa	90106169	90114191	LOC100130015	4.450935101	289
chr16.fa	90122974	90142338	PRDM7	0	0
chr17.fa	6011	31420	DOC2B	3.150392058	152
chr17.fa	62180	202633	RPH3AL	5.523527286	684
chr17.fa	260118	264457	C17orf97	3.676346965	304
chr17.fa	289771	295731	FAM101B	0.93382015	152
chr17.fa	411908	618096	VPS53	6.221390499	3820
chr17.fa	635847	646075	FAM57A	1.625234022	152
chr17.fa	647661	655501	GEMIN4	11.29502063	1900
chr17.fa	655900	658576	DBIL5P	0.63136828	76
chr17.fa	662549	685512	GLOD4	42.70197869	3414
chr17.fa	685572	695741	RNMTL1	6.791823623	532
chr17.fa	702585	883010	NXN	12.37806519	1672
chr17.fa	900357	905390	TIMM22	7.071813994	532
chr17.fa	906759	1090616	ABR	16.83033464	4484
chr17.fa	925716	925799	MIR3183	0	0
chr17.fa	1173858	1174565	BHLHA9	0	0
chr17.fa	1182957	1204281	TUSC5	0	0
chr17.fa	1247834	1303556	YWHAE	304.3888969	25458
chr17.fa	1325458	1359544	CRK	23.97398096	2584
chr17.fa	1367480	1396001	MYO1C	74.84905428	17148
chr17.fa	1397871	1420182	INPP5K	21.55325404	3268
chr17.fa	1420213	1421282	LOC100306951	15.15839849	304
chr17.fa	1421391	1466110	PITPNA	53.55510824	8508
chr17.fa	1477696	1532130	SLC43A2	2.815915871	380
chr17.fa	1537152	1549041	SCARF1	0	0
chr17.fa	1549445	1553392	RILP	5.662299321	456
chr17.fa	1553923	1588176	PRPF8	54.67862799	17936
chr17.fa	1606084	1613662	TLCD2	0.859319138	228
chr17.fa	1614798	1619566	C17orf91	3.758631665	228
chr17.fa	1619817	1641893	WDR81	7.015993833	2280
chr17.fa	1646130	1658559	SERPINF2	13.75310925	1444
chr17.fa	1665259	1680868	SERPINF1	0	0
chr17.fa	1682829	1733175	SMYD4	3.11992448	608
chr17.fa	1733273	1802848	RPA1	19.4496568	3800
chr17.fa	1837971	1928178	RTN4RL1	0	0
chr17.fa	1933431	1945169	DPH1	12.27376378	755
chr17.fa	1945277	1945525	OVCA2	9.288608293	104
chr17.fa	1953202	1953302	MIR132	0	0
chr17.fa	1953565	1953674	MIR212	0	0

chr17.fa	1958393	1962981	HIC1	0	0
chr17.fa	1963133	2207069	SMG6	12.00934078	3496
chr17.fa	2207248	2228553	SRR	5.079412297	304
chr17.fa	2225982	2240678	TSR1	18.55831334	3116
chr17.fa	2232419	2232504	SNORD91B	0	0
chr17.fa	2233573	2233664	SNORD91A	0	0
chr17.fa	2240806	2284348	SGSM2	26.04533149	5700
chr17.fa	2287354	2304258	MNT	6.981300824	1520
chr17.fa	2310275	2318730	LOC284009	0	0
chr17.fa	2319348	2415200	METT10D	8.813580943	2280
chr17.fa	2496923	2588909	PAFAH1B1	32.21379291	8132
chr17.fa	2592680	2614927	KIAA0664	37.20291443	8739
chr17.fa	2651372	2651476	MIR1253	0	0
chr17.fa	2699732	2941035	RAP1GAP2	21.81523073	6536
chr17.fa	2965963	2966901	OR1D5	0	0
chr17.fa	2995352	2996290	OR1D2	0	0
chr17.fa	3029904	3030845	OR1G1	0	0
chr17.fa	3100813	3101742	OR1A2	0	0
chr17.fa	3118915	3119844	OR1A1	0	0
chr17.fa	3143970	3144559	OR1D4	0	0
chr17.fa	3181184	3182268	OR3A2	0	0
chr17.fa	3194929	3195876	OR3A1	0	0
chr17.fa	3213539	3214740	OR3A4	0	0
chr17.fa	3300760	3301704	OR1E1	0	0
chr17.fa	3323862	3324827	OR3A3	0	0
chr17.fa	3336164	3337135	OR1E2	0	0
chr17.fa	3343306	3375142	SPATA22	0	0
chr17.fa	3377404	3402700	ASPA	6.838080968	456
chr17.fa	3416486	3461289	TRPV3	0.494152983	76
chr17.fa	3468740	3500336	TRPV1	2.871291251	608
chr17.fa	3511556	3539616	SHPK	5.640282604	912
chr17.fa	3539762	3566188	CTNS	2.16986978	374
chr17.fa	3566398	3571973	TAX1BP3	57.68513301	3040
chr17.fa	3572090	3572962	TMEM93	23.59369221	836
chr17.fa	3576522	3599583	P2RX5	0	0
chr17.fa	3617919	3704537	ITGAE	4.815211692	836
chr17.fa	3627197	3629992	GSG2	0	0
chr17.fa	3714460	3749540	C17orf85	14.6893757	2508
chr17.fa	3763617	3796337	CAMKK1	1.512481744	304
chr17.fa	3799885	3819960	P2RX1	0	0
chr17.fa	3827169	3867736	ATP2A3	1.73998782	380
chr17.fa	3907739	4046253	ZZEF1	12.60779518	6460
chr17.fa	4046462	4060991	CYB5D2	9.625085999	760

chr17.fa	4066665	4167274	ANKFY1	18.98174596	7220
chr17.fa	4172512	4269969	UBE2G1	18.47625103	3496
chr17.fa	4337219	4391499	SPNS3	0	0
chr17.fa	4402129	4442190	SPNS2	7.65714732	1140
chr17.fa	4442734	4458681	MYBBP1A	14.69715939	2660
chr17.fa	4460222	4463876	GGT6	10.92496187	1292
chr17.fa	4487276	4511614	SMTNL2	3.51689256	380
chr17.fa	4534214	4544960	ALOX15	2.518801387	304
chr17.fa	4574679	4607632	PELP1	8.280731912	1368
chr17.fa	4613789	4624795	ARRB2	1.761559755	152
chr17.fa	4634723	4636827	MED11	22.2099749	760
chr17.fa	4636897	4643223	CXCL16	3.628532882	380
chr17.fa	4643319	4649410	ZMYND15	0.700976688	76
chr17.fa	4675187	4686506	TM4SF5	2.387145867	76
chr17.fa	4688580	4689729	VMO1	0	0
chr17.fa	4692254	4693884	GLTPD2	4.149372795	228
chr17.fa	4699457	4701790	PSMB6	93.99114421	3491
chr17.fa	4710421	4726727	PLD2	8.656128057	1351
chr17.fa	4736635	4801063	MINK1	25.27430161	5396
chr17.fa	4801357	4806369	CHRNE	0	0
chr17.fa	4802948	4806227	C17orf107	6.879890491	684
chr17.fa	4835592	4838325	GP1BA	0.675846496	76
chr17.fa	4840426	4843462	SLC25A11	22.38633103	1824
chr17.fa	4843630	4848517	RNF167	13.85385241	1064
chr17.fa	4848947	4851825	PFN1	299.4976274	10868
chr17.fa	4854384	4860426	ENO3	0	0
chr17.fa	4862521	4871132	SPAG7	34.6278481	1596
chr17.fa	4871287	4890960	CAMTA2	14.75075564	3268
chr17.fa	4891425	4900905	INCA1	4.353305416	304
chr17.fa	4901243	4931694	KIF1C	90.09129421	32072
chr17.fa	4935897	4938727	GPR172B	0	0
chr17.fa	4981754	4999669	ZFP3	4.737152423	1064
chr17.fa	5009031	5026397	ZNF232	3.253359128	304
chr17.fa	5031687	5078324	USP6	0	0
chr17.fa	5082831	5095178	ZNF594	2.772549611	608
chr17.fa	5095379	5138931	LOC100130950	0	0
chr17.fa	5113901	5124645	C17orf87	0	0
chr17.fa	5185558	5289132	RABEP1	37.23249244	9044
chr17.fa	5289346	5322960	NUP88	19.04623938	2052
chr17.fa	5323060	5336098	RPAIN	10.10567313	608
chr17.fa	5336341	5342471	C1QBP	66.06527374	2736
chr17.fa	5344232	5372380	DHX33	6.017013096	1520
chr17.fa	5377613	5389494	DERL2	6.014789185	304

chr17.fa	5390247	5394130	MIS12	2.027317097	228
chr17.fa	5402747	5404319	LOC728392	0	0
chr17.fa	5404719	5487832	NLRP1	0.277099287	76
chr17.fa	5973934	6027747	WSCD1	0	0
chr17.fa	6327059	6338519	AIPL1	0	0
chr17.fa	6347735	6354385	FAM64A	0	0
chr17.fa	6354583	6459877	PITPNM3	0.236401719	76
chr17.fa	6481645	6544221	KIAA0753	8.343890979	1748
chr17.fa	6544248	6546632	TXNDC17	26.7954566	988
chr17.fa	6547862	6554954	MED31	19.7912495	380
chr17.fa	6555059	6556617	C17orf100	1.084156521	76
chr17.fa	6588039	6616740	SLC13A5	0	0
chr17.fa	6659156	6678964	XAF1	2.798346976	456
chr17.fa	6679552	6690965	FBXO39	0	0
chr17.fa	6703300	6735060	TEKT1	0	0
chr17.fa	6756895	6803668	ALOX12P2	0	0
chr17.fa	6899384	6914055	ALOX12	0	0
chr17.fa	6915798	6917851	RNASEK	160.4307021	5396
chr17.fa	6918073	6920839	C17orf49	45.99114279	1824
chr17.fa	6920934	6921020	MIR195	0	0
chr17.fa	6921230	6921341	MIR497	0	0
chr17.fa	6926369	6932961	BCL6B	0.479475171	76
chr17.fa	6939394	6943440	SLC16A13	0.966956421	76
chr17.fa	6944949	6947242	SLC16A11	0	0
chr17.fa	6977856	6983600	CLEC10A	0	0
chr17.fa	7004641	7018128	ASGR2	1.09616564	76
chr17.fa	7076751	7082883	ASGR1	1.124631698	76
chr17.fa	7093210	7123369	DLG4	3.673455881	760
chr17.fa	7123441	7128585	ACADVL	122.5561666	11435
chr17.fa	7126616	7126698	MIR324	0	0
chr17.fa	7128661	7137863	DVL2	10.08721467	1368
chr17.fa	7138347	7142825	PHF23	32.02120223	2933
chr17.fa	7143738	7145753	GABARAP	429.6406658	17252
chr17.fa	7146906	7155259	CTDNEP1	30.06771897	2508
chr17.fa	7155372	7163221	C17orf81	31.82082787	1976
chr17.fa	7163260	7166512	CLDN7	9.268148313	836
chr17.fa	7185054	7191367	SLC2A4	0	0
chr17.fa	7191571	7197876	YBX2	0	0
chr17.fa	7210318	7215782	EIF5A	132.0155713	10792
chr17.fa	7215978	7218658	GPS2	24.83396727	1292
chr17.fa	7218951	7232638	NEURL4	10.1012253	2356
chr17.fa	7239848	7254793	ACAP1	1.347912344	152
chr17.fa	7255208	7258262	KCTD11	4.97933631	684

chr17.fa	7258497	7260538	TMEM95	0	0
chr17.fa	7284365	7293054	TNK1	3.021405231	380
chr17.fa	7293093	7297843	PLSCR3	7.916455321	608
chr17.fa	7306295	7307418	C17orf61	116.518916	2342
chr17.fa	7311502	7323179	NLGN2	0.3640542	76
chr17.fa	7323679	7324951	SPEM1	0	0
chr17.fa	7328935	7330886	C17orf74	0	0
chr17.fa	7338762	7340998	TMEM102	6.853203561	608
chr17.fa	7342689	7348251	FGF11	0.622472637	76
chr17.fa	7348406	7360932	CHRNB1	2.774106348	304
chr17.fa	7362685	7387568	ZBTB4	43.41585406	11932
chr17.fa	7384721	7386383	AMAC1L3	0	0
chr17.fa	7387698	7417935	POLR2A	58.44615529	17708
chr17.fa	7456904	7461207	TNFSF12	12.72655202	590
chr17.fa	7461609	7463210	TNFSF13	7.589762823	358
chr17.fa	7465319	7475287	SENP3	18.88300432	2110
chr17.fa	7476057	7482324	EIF4A1	150.4758102	12477
chr17.fa	7478031	7478165	SNORA48	0	0
chr17.fa	7480129	7480276	SNORD10	0	0
chr17.fa	7481273	7481409	SNORA67	0	0
chr17.fa	7482805	7485429	CD68	2.731852043	228
chr17.fa	7486965	7491497	MPDU1	11.70777847	836
chr17.fa	7491528	7493488	SOX15	0	0
chr17.fa	7494548	7518215	FXR2	20.91387968	2809
chr17.fa	7517382	7536700	SHBG	0	0
chr17.fa	7529556	7531194	SAT2	71.03571441	3076
chr17.fa	7554254	7561089	ATP1B2	0	0
chr17.fa	7571720	7590757	TP53	27.15973319	3102
chr17.fa	7589389	7606820	WRAP53	2.427176261	228
chr17.fa	7608520	7614693	EFNB3	0	0
chr17.fa	7623039	7737058	DNAH2	0.125206179	76
chr17.fa	7657638	7658286	RPL29P2	0	0
chr17.fa	7743235	7758118	KDM6B	10.08454597	3040
chr17.fa	7758384	7759417	TMEM88	1.933912843	76
chr17.fa	7760003	7761063	LSMD1	45.57771777	1824
chr17.fa	7761173	7765600	CYB5D1	7.435423412	1216
chr17.fa	7788123	7816075	CHD3	108.463911	37086
chr17.fa	7809441	7809578	SCARNA21	0	0
chr17.fa	7816640	7819265	LOC284023	1.338794309	152
chr17.fa	7826027	7832753	KCNAB3	1.05102025	76
chr17.fa	7833663	7835317	TRAPPC1	80.10771379	2734
chr17.fa	7835473	7852896	CNTROB	4.930187881	836
chr17.fa	7905988	7923658	GUCY2D	0.932930586	152

chr17.fa	7942358	7952451	ALOX15B	63.17819272	7676
chr17.fa	7975954	7991021	ALOX12B	0.675624105	76
chr17.fa	7991374	7991465	MIR4314	0	0
chr17.fa	7999218	8022234	ALOXE3	0	0
chr17.fa	8023908	8027410	HES7	0	0
chr17.fa	8043788	8055753	PER1	6.819622508	1444
chr17.fa	8062465	8066293	VAMP2	62.72362535	6078
chr17.fa	8076297	8079714	TMEM107	2.856613439	228
chr17.fa	8090493	8090577	MIR3676	0	0
chr17.fa	8091651	8093564	C17orf59	12.36272021	1064
chr17.fa	8108049	8113883	AURKB	0	0
chr17.fa	8123948	8127361	C17orf44	0	0
chr17.fa	8128139	8151413	C17orf68	6.213829203	1976
chr17.fa	8152596	8173809	PFAS	3.431939167	831
chr17.fa	8191082	8198170	SLC25A35	4.877925977	456
chr17.fa	8193131	8193409	RANGRF	13.52382404	90
chr17.fa	8213556	8225834	ARHGEF15	1.592320142	304
chr17.fa	8243188	8249363	ODF4	0	0
chr17.fa	8261731	8263859	LOC100128288	0	0
chr17.fa	8271973	8274858	KRBA2	2.581738063	228
chr17.fa	8280834	8286565	RPL26	1321.354623	34580
chr17.fa	8294023	8301144	RNF222	0	0
chr17.fa	8339179	8371481	NDEL1	18.88945366	2047
chr17.fa	8377530	8534036	MYH10	19.45165832	6664
chr17.fa	8633246	8648154	CCDC42	0	0
chr17.fa	8656424	8661877	SPDYE4	0	0
chr17.fa	8700428	8702667	MFSD6L	0.754572939	76
chr17.fa	8706055	8770994	PIK3R6	0	0
chr17.fa	8782233	8869024	PIK3R5	0.371837888	76
chr17.fa	8924859	9147317	NTN1	0	0
chr17.fa	9153788	9479275	STX8	19.20658335	836
chr17.fa	9479944	9546776	WDR16	0	0
chr17.fa	9548950	9633003	USP43	12.04892639	2204
chr17.fa	9674755	9694601	DHRS7C	0	0
chr17.fa	9729381	9793022	GLP2R	0	0
chr17.fa	9801027	9808684	RCVRN	0	0
chr17.fa	9813926	10101868	GAS7	0.778591176	304
chr17.fa	10204183	10276322	MYH13	0	0
chr17.fa	10293642	10325267	MYH8	0.839748723	228
chr17.fa	10346608	10372876	MYH4	0.281102327	76
chr17.fa	10395627	10421859	MYH1	0	0
chr17.fa	10424465	10453017	MYH2	0	0
chr17.fa	10531843	10559465	MYH3	0.561759871	152

chr17.fa	10583649	10600885	SCO1	16.38132705	1292
chr17.fa	10600927	10614875	C17orf48	7.793917835	532
chr17.fa	10616639	10633646	TMEM220	1.639911834	228
chr17.fa	10698230	10707416	LOC100289255	15.46552058	1064
chr17.fa	10725792	10741418	PIRT	0	0
chr17.fa	11144740	11467380	SHISA6	0.455901717	152
chr17.fa	11501748	11873065	DNAH9	0	0
chr17.fa	11880762	11900689	ZNF18	6.719101739	836
chr17.fa	11924135	12047051	MAP2K4	15.80444458	2660
chr17.fa	11985216	11985313	MIR744	0	0
chr17.fa	12453285	12540504	FLJ34690	0	0
chr17.fa	12569207	12670651	MYOCD	0	0
chr17.fa	12692829	12894928	ARHGAP44	5.601808947	1064
chr17.fa	12894961	12921381	ELAC2	12.54641524	2128
chr17.fa	13399006	13505244	HS3ST3A1	0	0
chr17.fa	13927815	13928915	CDRT15P	0	0
chr17.fa	13972719	14111996	COX10	3.941214743	532
chr17.fa	14139173	14140150	CDRT15	0	0
chr17.fa	14204506	14249492	HS3ST3B1	1.663485288	152
chr17.fa	14207057	14209062	MGC12916	0	0
chr17.fa	14934292	14935274	CDRT7	0	0
chr17.fa	15133096	15168644	PMP22	0.850201104	76
chr17.fa	15207129	15244958	TEKT3	0	0
chr17.fa	15339338	15370925	CDRT4	2.716284667	304
chr17.fa	15405578	15466945	FAM18B2	0.650493913	152
chr17.fa	15491977	15523018	CDRT1	1.835171203	228
chr17.fa	15531280	15586193	TRIM16	16.89594001	2204
chr17.fa	15602891	15624100	ZNF286A	1.52404608	380
chr17.fa	15635591	15648098	TBC1D26	0	0
chr17.fa	15668017	15669003	CDRT15L1	0	0
chr17.fa	15690164	15693019	MEIS3P1	5.918049064	760
chr17.fa	15848231	15879210	ADORA2B	0	0
chr17.fa	15879875	15897092	ZSWIM7	16.46405653	1357
chr17.fa	15902782	15932096	TTC19	9.440946183	1108
chr17.fa	15933408	16118874	NCOR1	32.49311611	14808
chr17.fa	16120509	16229573	PIGL	2.934227927	152
chr17.fa	16185328	16185402	MIR1288	0	0
chr17.fa	16245848	16256812	CENPV	2.90398274	152
chr17.fa	16284367	16286054	UBB	751.961178	32832
chr17.fa	16318856	16340317	TRPV2	0	0
chr17.fa	16342301	16373962	NCRNA00188	306.9477287	31883
chr17.fa	16342823	16342841	SNORD49B	0	0
chr17.fa	16343350	16343420	SNORD49A	0	0

chr17.fa	16344540	16344612	SNORD65	0	0
chr17.fa	16345341	16395480	C17orf76	0	0
chr17.fa	16453631	16472520	ZNF287	1.975499975	380
chr17.fa	16524048	16557167	ZNF624	1.194684889	228
chr17.fa	16593639	16678311	CCDC144A	0	0
chr17.fa	16690206	16707819	LOC162632	0.433217826	76
chr17.fa	16692057	16693815	FAM106C	0	0
chr17.fa	16733797	16736147	KRT16P2	0	0
chr17.fa	16842398	16875402	TNFRSF13B	0	0
chr17.fa	16946107	17088872	MPRIP	26.86951283	4712
chr17.fa	17104309	17109646	PLD6	0.655831299	76
chr17.fa	17115527	17140502	FLCN	16.68489087	3040
chr17.fa	17149938	17184617	COPS3	23.80518612	2128
chr17.fa	17206680	17250977	NT5M	2.12872743	152
chr17.fa	17380300	17396534	MED9	6.845864656	684
chr17.fa	17397753	17399709	RASD1	9.031301812	679
chr17.fa	17408877	17494994	PEMT	4.190515145	228
chr17.fa	17584787	17714662	RAI1	23.48383101	7980
chr17.fa	17680000	17682843	SMCR5	0	0
chr17.fa	17714766	17740325	SREBF1	9.662002918	2128
chr17.fa	17717150	17717245	MIR33B	0	0
chr17.fa	17746822	17875784	TOM1L2	67.36448244	17593
chr17.fa	17876127	17920189	LRRC48	2.614429552	304
chr17.fa	17921334	17942480	ATPAF2	16.33551449	1140
chr17.fa	17942611	17971718	C17orf39	5.591356566	1064
chr17.fa	17991283	18011292	DRG2	8.952130587	760
chr17.fa	18012020	18083116	MYO15A	0.142552683	76
chr17.fa	18086867	18113267	ALKBH5	57.60262592	8892
chr17.fa	18128936	18148152	LLGL1	7.264404671	1368
chr17.fa	18148189	18162055	FLII	42.175579	7821
chr17.fa	18163848	18169095	SMCR7	0.638929577	76
chr17.fa	18177235	18218321	TOP3A	5.338275516	988
chr17.fa	18218594	18231186	SMCR8	13.51982101	4940
chr17.fa	18231371	18266856	SHMT1	27.50599611	2888
chr17.fa	18281079	18292960	EVPLL	0	0
chr17.fa	18325495	18328647	LOC339240	0	0
chr17.fa	18380099	18398259	LGALS9C	0	0
chr17.fa	18414576	18424566	LOC220594	0	0
chr17.fa	18427880	18430160	FAM106A	0	0
chr17.fa	18441115	18528930	CCDC144B	0.38873961	152
chr17.fa	18538842	18547740	TBC1D28	0	0
chr17.fa	18561742	18585572	ZNF286B	0.968290768	228
chr17.fa	18569236	18576494	FOXO3B	0.943160576	304

chr17.fa	18625402	18639431	TRIM16L	5.116551607	456
chr17.fa	18647326	18682662	FBXW10	0	0
chr17.fa	18684582	18710026	FAM18B1	4.723808958	380
chr17.fa	18761492	18834580	PRPSAP2	37.33590429	3173
chr17.fa	18855478	18923989	SLC5A10	11.26610979	1000
chr17.fa	18874532	18908060	FAM83G	21.89506913	2736
chr17.fa	18924004	18950336	GRAP	0	0
chr17.fa	19030782	19062148	GRAPL	0	0
chr17.fa	19140690	19240028	EPN2	12.23506773	2660
chr17.fa	19246483	19266046	B9D1	9.106469997	380
chr17.fa	19247819	19247887	MIR1180	0	0
chr17.fa	19281034	19286754	MAPK7	2.700717291	380
chr17.fa	19286858	19290532	MFAP4	0	0
chr17.fa	19314491	19320589	RNF112	0	0
chr17.fa	19437167	19482346	SLC47A1	4.062640273	599
chr17.fa	19552064	19580908	ALDH3A2	49.90589299	8588
chr17.fa	19581628	19620043	SLC47A2	0	0
chr17.fa	19641298	19651746	ALDH3A1	0	0
chr17.fa	19674143	19771239	ULK2	3.513779085	1444
chr17.fa	19808750	19881129	AKAP10	12.70586965	1666
chr17.fa	19990335	20218067	SPECC1	32.93033697	6536
chr17.fa	20224487	20305504	CCDC144C	0.402083075	76
chr17.fa	20353176	20370848	LGALS9B	0	0
chr17.fa	20404827	20407811	KRT16P3	0	0
chr17.fa	20483037	20484224	CDRT15L2	0	0
chr17.fa	20766708	20799453	CCDC144NL	0	0
chr17.fa	20902906	20946352	USP22	77.03582578	18082
chr17.fa	21030258	21094836	DHRS7B	0	0
chr17.fa	21101263	21117908	TMEM11	14.01797703	1064
chr17.fa	21142184	21156578	C17orf103	7.064475089	1520
chr17.fa	21187968	21218551	MAP2K3	16.37487771	1748
chr17.fa	21279699	21280225	KCNJ12	0	0
chr17.fa	21308448	21308639	KCNJ18	0	0
chr17.fa	21431571	21454941	C17orf51	1.281639802	456
chr17.fa	21825370	21826499	FAM27L	0	0
chr17.fa	21904062	21913070	FLJ36000	0	0
chr17.fa	22022437	22023991	MTRNR2L1	0	0
chr17.fa	25621106	25640645	WSB1	11.93617412	1520
chr17.fa	25745031	25758649	LOC440419	0	0
chr17.fa	25799036	25950718	KSR1	2.634444749	532
chr17.fa	25958174	25976586	LGALS9	3.832687895	304
chr17.fa	26083792	26127555	NOS2	0.401860684	76
chr17.fa	26205340	26220409	C17orf108	15.97724245	1064

chr17.fa	26369688	26523404	NLK	17.67564314	2812
chr17.fa	26553589	26555085	PYY2	0	0
chr17.fa	26574470	26575315	PPY2	0	0
chr17.fa	26603012	26634408	FLJ40504	2.600196523	228
chr17.fa	26646121	26655352	TMEM97	109.4135209	10868
chr17.fa	26655712	26662495	IFT20	27.48242265	760
chr17.fa	26662548	26673658	TNFAIP1	14.63822575	2259
chr17.fa	26674036	26684603	POLDIP2	52.32795426	5513
chr17.fa	26684687	26689089	TMEM199	15.16373588	988
chr17.fa	26691290	26692265	SEBOX	0	0
chr17.fa	26694299	26697373	VTN	0	0
chr17.fa	26698987	26728065	SARM1	2.669360149	380
chr17.fa	26721661	26733228	SLC46A1	0	0
chr17.fa	26800664	26824798	SLC13A2	4.472951818	532
chr17.fa	26850959	26865175	FOXN1	0	0
chr17.fa	26873725	26879646	UNC119	14.65468269	912
chr17.fa	26880406	26898887	PIGS	4.118015652	532
chr17.fa	26900133	26903951	ALDOC	14.10315281	1047
chr17.fa	26904583	26926056	SPAG5	1.331455404	228
chr17.fa	26934982	26941211	SGK494	0.894456929	152
chr17.fa	26941458	26972173	KIAA0100	26.15652703	8740
chr17.fa	26975607	26988933	SDF2	14.15029972	684
chr17.fa	26989302	27029249	SUPT6H	38.24970925	10108
chr17.fa	27030534	27038872	PROCA1	0	0
chr17.fa	27041299	27045286	RAB34	6.177579456	365
chr17.fa	27047000	27051365	RPL23A	121.35481	5244
chr17.fa	27047568	27047634	SNORD42B	0	0
chr17.fa	27049600	27049671	SNORD4A	0	0
chr17.fa	27050447	27050509	SNORD42A	0	0
chr17.fa	27050699	27050772	SNORD4B	0	0
chr17.fa	27051375	27053949	TLCD1	0	0
chr17.fa	27055832	27069784	NEK8	2.365573932	304
chr17.fa	27071023	27077976	TRAF4	16.59882553	2166
chr17.fa	27082996	27169841	C17orf63	8.859615897	1672
chr17.fa	27182043	27188072	ERAL1	24.05470892	1974
chr17.fa	27188387	27188458	MIR451	0	0
chr17.fa	27188551	27188636	MIR144	0	0
chr17.fa	27206357	27224715	FLOT2	40.45627355	4788
chr17.fa	27224799	27230089	DHRS13	3.519338862	304
chr17.fa	27232271	27278508	PHF12	17.55844304	3800
chr17.fa	27281947	27333081	SEZ6	0	0
chr17.fa	27369918	27384236	PIPOX	14.71539546	1596
chr17.fa	27400528	27507407	MYO18A	12.70609204	3536

chr17.fa	27401933	27402627	TIAF1	0	0
chr17.fa	27573875	27581502	CRYBA1	0	0
chr17.fa	27582854	27621166	NUFIP2	15.51422422	7590
chr17.fa	27717943	27878921	TAOK1	15.49065077	8415
chr17.fa	27887689	27894042	ABHD15	1.007653989	152
chr17.fa	27895739	27900175	TP53I13	1.163327746	76
chr17.fa	27900487	27916610	GIT1	10.33873898	1748
chr17.fa	27920527	27941773	ANKRD13B	0	0
chr17.fa	27941780	27948441	CORO6	0	0
chr17.fa	27952965	28257018	SSH2	13.24850389	5442
chr17.fa	28256874	28435470	EFCAB5	0.267981253	76
chr17.fa	28443834	28513486	CCDC55	19.60488577	2203
chr17.fa	28444097	28444190	MIR423	88.95643252	76
chr17.fa	28523378	28562954	SLC6A4	0.372949843	76
chr17.fa	28575223	28619074	BLMH	6.293667601	684
chr17.fa	28643366	28661065	TMIGD1	0	0
chr17.fa	28705942	28796675	CPD	1.651920952	684
chr17.fa	28804426	28853832	GOSR1	10.36609308	2428
chr17.fa	28886584	28890509	TBC1D29	0	0
chr17.fa	28903483	28964484	LRRC37BP1	2.573731984	532
chr17.fa	28951336	28953825	SH3GL1P2	0	0
chr17.fa	29058724	29085353	SUZ12P	0	0
chr17.fa	29109702	29151778	CRLF3	4.02283227	532
chr17.fa	29159023	29222295	ATAD5	0.541522283	152
chr17.fa	29226001	29233286	C17orf42	6.311236496	380
chr17.fa	29248754	29286211	ADAP2	0	0
chr17.fa	29297956	29326927	RNF135	6.858318556	684
chr17.fa	29302353	29303017	DPRXP4	0	0
chr17.fa	29421945	29704695	NF1	14.55527388	8132
chr17.fa	29621668	29624380	OMG	0	0
chr17.fa	29630788	29641130	EVI2B	0	0
chr17.fa	29643428	29648767	EVI2A	0	0
chr17.fa	29718642	29865232	RAB11FIP4	27.80600168	10784
chr17.fa	29887015	29887102	MIR193A	0	0
chr17.fa	29902430	29902540	MIR365-2	0	0
chr17.fa	30178884	30186326	C17orf79	97.40595926	4104
chr17.fa	30190190	30228729	UTP6	17.05895267	1584
chr17.fa	30264044	30328057	SUZ12	11.70333065	2356
chr17.fa	30348155	30380519	LRRC37B	1.675049624	228
chr17.fa	30367355	30369851	SH3GL1P1	0	0
chr17.fa	30469473	30552745	RHOT1	11.83898921	1761
chr17.fa	30477387	30478590	ARGFXP2	0	0
chr17.fa	30593195	30651680	RHBDL3	0	0

chr17.fa	30658390	30669189	C17orf75	11.51919084	836
chr17.fa	30677128	30677156	MIR632	0	0
chr17.fa	30677222	30697468	ZNF207	40.22343009	4084
chr17.fa	30771502	30808042	PSMD11	40.04729636	2847
chr17.fa	30814105	30818271	CDK5R1	0.438555212	76
chr17.fa	30819628	31203902	MYO1D	22.17906254	5168
chr17.fa	31254928	31268667	TMEM98	13.98773184	1129
chr17.fa	31318882	31324892	SPACA3	0	0
chr17.fa	31340106	32483825	ACCN1	0	0
chr17.fa	31856806	31860755	AA06	0	0
chr17.fa	32582296	32584220	CCL2	0	0
chr17.fa	32597240	32599256	CCL7	0	0
chr17.fa	32612687	32615199	CCL11	0	0
chr17.fa	32646066	32648421	CCL8	0	0
chr17.fa	32683471	32685629	CCL13	0	0
chr17.fa	32687399	32690252	CCL1	0	0
chr17.fa	32901142	32906388	C17orf102	0	0
chr17.fa	32907768	32966337	TMEM132E	0.386960481	76
chr17.fa	33254878	33288528	CCT6B	3.562037949	304
chr17.fa	33288549	33290205	ZNF830	9.180081445	684
chr17.fa	33307517	33332088	LIG3	23.40243588	4104
chr17.fa	33336131	33416294	RFFL	23.4566993	4527
chr17.fa	33426811	33446888	RAD51L3	1.308771513	152
chr17.fa	33448631	33457751	FNDC8	0	0
chr17.fa	33458368	33469322	NLE1	5.909598203	684
chr17.fa	33474836	33516364	UNC45B	0	0
chr17.fa	33519539	33521412	AMAC1	0	0
chr17.fa	33570086	33594761	SLFN5	21.0953508	4408
chr17.fa	33677329	33700720	SLFN11	0.643155007	152
chr17.fa	33738081	33759543	SLFN12	4.24299944	456
chr17.fa	33762115	33775856	SLFN13	0.1994848	76
chr17.fa	33801942	33814758	SLFN12L	0	0
chr17.fa	33875144	33885110	SLFN14	0	0
chr17.fa	33900676	33900772	SNORD7	0	0
chr17.fa	33901814	33905656	PEX12	5.669638227	684
chr17.fa	33914282	34053436	AP2B1	39.59272899	10260
chr17.fa	34058679	34070540	RASL10B	0.529068382	76
chr17.fa	34071537	34079897	GAS2L2	0	0
chr17.fa	34087916	34092098	C17orf50	0	0
chr17.fa	34092876	34122640	MMP28	0.593561796	76
chr17.fa	34136488	34174237	TAF15	125.6462907	12164
chr17.fa	34181960	34195895	C17orf66	0	0
chr17.fa	34198496	34207377	CCL5	0	0

chr17.fa	34245085	34257780	RDM1	0	0
chr17.fa	34261523	34270703	LYZL6	0	0
chr17.fa	34303535	34308523	CCL16	0	0
chr17.fa	34324618	34324756	CCL15	12.15945476	76
chr17.fa	34340097	34345005	CCL23	0	0
chr17.fa	34391643	34398841	CCL18	0	0
chr17.fa	34415603	34417506	CCL3	0	0
chr17.fa	34431220	34433014	CCL4	0	0
chr17.fa	34493061	34503988	TBC1D3B	0	0
chr17.fa	34581085	34592027	TBC1D3C	0	0
chr17.fa	34745936	34757050	TBC1D3H	0	0
chr17.fa	34797175	34808103	TBC1D3G	0	0
chr17.fa	34842473	34851598	ZNHIT3	23.61504175	944
chr17.fa	34851663	34891305	MYO19	4.690895078	988
chr17.fa	34891403	34895150	PIGW	2.998054167	304
chr17.fa	34900737	34946276	GGNBP2	31.48657407	4028
chr17.fa	34948226	34957233	DHRS11	10.57669744	760
chr17.fa	34958025	34965407	MRM1	5.532422929	456
chr17.fa	35294772	35301915	LHX1	1.477788735	228
chr17.fa	35306175	35414171	AATF	26.84060199	2584
chr17.fa	35391042	35391110	MIR2909	0	0
chr17.fa	35441927	35766902	ACACA	26.85105437	13740
chr17.fa	35732985	35749662	C17orf78	0	0
chr17.fa	35766977	35837226	TADA2A	5.326266397	456
chr17.fa	35849951	35873588	DUSP14	10.08721467	684
chr17.fa	35874900	35969486	SYNRG	24.02579808	8902
chr17.fa	35972363	36003493	DDX52	11.16203076	1976
chr17.fa	36046434	36105096	HNF1B	14.89931288	1900
chr17.fa	36202573	36244363	LOC284100	0	0
chr17.fa	36337528	36337710	TBC1D3	0	0
chr17.fa	36351796	36413256	LOC440434	1.022998974	76
chr17.fa	36452998	36479101	MRPL45	24.69119219	1883
chr17.fa	36481493	36499693	GPR179	0.211716309	76
chr17.fa	36508007	36561846	SOCS7	9.957115883	3572
chr17.fa	36584720	36668628	ARHGAP23	32.42862269	9258
chr17.fa	36686259	36762183	SRCIN1	0.479475171	152
chr17.fa	36827959	36831187	C17orf96	1.570303425	228
chr17.fa	36861873	36886056	MLLT6	33.07689269	11155
chr17.fa	36886510	36891858	CISD3	72.30089727	3030
chr17.fa	36892343	36904558	PCGF2	43.95937787	1779
chr17.fa	36909002	36920478	PSMB3	69.489874	2356
chr17.fa	36921944	36956158	PIP4K2B	13.82382961	3563
chr17.fa	36957446	36981589	CWC25	9.43004902	988

chr17.fa	36991341	36997642	C17orf98	0	0
chr17.fa	37006321	37010053	RPL23	2214.506339	57456
chr17.fa	37009116	37009248	SNORA21	0	0
chr17.fa	37026112	37078023	LASP1	42.77870362	7904
chr17.fa	37092685	37123655	FBXO47	0	0
chr17.fa	37186159	37209458	FLJ43826	0	0
chr17.fa	37213272	37237704	LOC100131347	0	0
chr17.fa	37219556	37307902	PLXDC1	0	0
chr17.fa	37313147	37322414	ARL5C	0	0
chr17.fa	37329709	37353901	CACNB1	2.500342927	456
chr17.fa	37356536	37360980	RPL19	1265.320077	41648
chr17.fa	37366789	37382040	STAC2	1.031449835	152
chr17.fa	37408897	37557909	FBXL20	3.093459941	1444
chr17.fa	37560538	37607527	MED1	11.28612498	4135
chr17.fa	37617739	37690800	CDK12	13.65992739	5092
chr17.fa	37760021	37764175	NEUROD2	0	0
chr17.fa	37783179	37792877	PPP1R1B	17.33093697	1596
chr17.fa	37793333	37820454	STARD3	5.344057684	681
chr17.fa	37821599	37822807	TCAP	0	0
chr17.fa	37824507	37826728	PNMT	2.921551635	152
chr17.fa	37827375	37844310	PGAP3	0.628921978	76
chr17.fa	37844393	37884915	ERBB2	31.30488056	7217
chr17.fa	37885409	37886788	C17orf37	103.1138488	3496
chr17.fa	37894187	37903538	GRB7	13.26785191	1368
chr17.fa	37921199	38020441	IKZF3	4.856798824	532
chr17.fa	38024455	38034149	ZPBP2	0	0
chr17.fa	38060848	38074903	GSDMB	1.063029369	76
chr17.fa	38077296	38083884	ORMDL3	13.43286609	1292
chr17.fa	38097727	38100986	LOC728129	0	0
chr17.fa	38119226	38134019	GSDMA	0	0
chr17.fa	38137060	38154212	PSMD3	36.99964898	3572
chr17.fa	38171614	38174066	CSF3	0	0
chr17.fa	38175350	38210889	MED24	10.38455154	1745
chr17.fa	38183795	38183898	SNORD124	0	0
chr17.fa	38218446	38250120	THRA	10.50419794	1596
chr17.fa	38249037	38256973	NR1D1	3.37100401	380
chr17.fa	38278790	38293045	MSL1	11.50384585	1976
chr17.fa	38296507	38328431	CASC3	36.33269813	6829
chr17.fa	38334242	38351906	RAPGEFL1	6.348820589	1064
chr17.fa	38375574	38438439	WIPF2	21.10802709	5244
chr17.fa	38444146	38459413	CDC6	1.107285194	152
chr17.fa	38465423	38513895	RARA	5.425897602	1064
chr17.fa	38516905	38520945	GJD3	2.509460961	456

chr17.fa	38544773	38574202	TOP2A	0.293778618	76
chr17.fa	38599676	38613982	IGFBP4	28.72380967	2888
chr17.fa	38632080	38657854	TNS4	0.414981758	76
chr17.fa	38710022	38721736	CCR7	0	0
chr17.fa	38783976	38804103	SMARCE1	39.02185108	4248
chr17.fa	38811872	38821416	KRT222	0	0
chr17.fa	38854243	38860002	KRT24	0	0
chr17.fa	38904273	38911584	KRT25	0	0
chr17.fa	38922490	38928411	KRT26	0	0
chr17.fa	38933060	38938786	KRT27	0	0
chr17.fa	38948448	38956211	KRT28	0	0
chr17.fa	38974369	38978863	KRT10	6.53207084	608
chr17.fa	38975374	38992526	TMEM99	6.330139738	608
chr17.fa	39017430	39023462	KRT12	0	0
chr17.fa	39032141	39041495	KRT20	14.98204236	1216
chr17.fa	39078952	39093836	KRT23	34.45149197	3326
chr17.fa	39114669	39123144	KRT39	0	0
chr17.fa	39133968	39143387	KRT40	0	0
chr17.fa	39149682	39150385	KRTAP3-3	0	0
chr17.fa	39155445	39156138	KRTAP3-2	0	0
chr17.fa	39164774	39165366	KRTAP3-1	0	0
chr17.fa	39182279	39183454	KRTAP1-5	0	0
chr17.fa	39190137	39191107	KRTAP1-3	0	0
chr17.fa	39196811	39197713	KRTAP1-1	0	0
chr17.fa	39202796	39203568	KRTAP2-1	0	0
chr17.fa	39210751	39211463	KRTAP2-2	0	0
chr17.fa	39215493	39216344	LOC730755	0	0
chr17.fa	39221368	39222131	KRTAP2-4	0	0
chr17.fa	39240459	39241396	KRTAP4-7	0	0
chr17.fa	39253234	39254375	KRTAP4-8	0	0
chr17.fa	39261641	39262740	KRTAP4-9	0	0
chr17.fa	39273434	39274606	KRTAP4-11	0	0
chr17.fa	39279345	39280419	KRTAP4-12	0	0
chr17.fa	39305176	39306054	KRTAP4-5	0	0
chr17.fa	39315906	39316983	KRTAP4-4	0	0
chr17.fa	39323483	39324424	KRTAP4-3	0	0
chr17.fa	39333698	39334460	KRTAP4-2	0	0
chr17.fa	39340352	39341147	KRTAP4-1	0	0
chr17.fa	39346139	39346891	KRTAP9-1	0	0
chr17.fa	39382900	39383904	KRTAP9-2	0	0
chr17.fa	39388715	39389706	KRTAP9-3	0	0
chr17.fa	39394270	39395256	KRTAP9-8	0	0
chr17.fa	39405939	39406905	KRTAP9-4	0	0

chr17.fa	39411636	39412616	KRTAP9-9	0	0
chr17.fa	39463952	39465505	KRTAP16-1	0	0
chr17.fa	39471169	39471947	KRTAP17-1	0	0
chr17.fa	39502371	39507056	KRT33A	0	0
chr17.fa	39519746	39526047	KRT33B	0	0
chr17.fa	39533921	39538636	KRT34	0	0
chr17.fa	39549977	39553844	KRT31	0	0
chr17.fa	39576809	39580822	KRT37	0	0
chr17.fa	39592621	39597596	KRT38	0	0
chr17.fa	39615765	39623638	KRT32	0	0
chr17.fa	39632941	39637392	KRT35	0	0
chr17.fa	39642388	39646116	KRT36	0	0
chr17.fa	39657233	39661865	KRT13	14.78277996	1140
chr17.fa	39669997	39675270	KRT15	0	0
chr17.fa	39679869	39684641	KRT19	203.372196	13452
chr17.fa	39722094	39728310	KRT9	0	0
chr17.fa	39738531	39743147	KRT14	0	0
chr17.fa	39766031	39769079	KRT16	0	0
chr17.fa	39775692	39780882	KRT17	0	0
chr17.fa	39782579	39796451	KRT42P	0	0
chr17.fa	39845127	39847898	EIF1	322.4837472	19228
chr17.fa	39868613	39872221	GAST	0	0
chr17.fa	39878891	39890898	HAP1	0	0
chr17.fa	39910859	39942964	JUP	82.30760636	11884
chr17.fa	39958205	39968451	LEPREL4	0.649604348	76
chr17.fa	39968962	39979469	FKBP10	0	0
chr17.fa	39981334	39992523	NT5C3L	22.03495312	1511
chr17.fa	39994043	40004599	KLHL10	0	0
chr17.fa	40009799	40021629	KLHL11	6.539854528	684
chr17.fa	40023179	40075272	ACLY	77.21618495	15114
chr17.fa	40086888	40117668	TTC25	0.811505056	76
chr17.fa	40118759	40129754	CNP	22.14192323	4788
chr17.fa	40133873	40169715	DNAJC7	30.27832333	2369
chr17.fa	40172087	40177656	NKIRAS2	13.57564117	1520
chr17.fa	40253422	40264751	DHX58	7.750106792	912
chr17.fa	40265129	40273382	KAT2A	3.258696514	456
chr17.fa	40274756	40275371	HSPB9	0	0
chr17.fa	40276997	40307022	RAB5C	73.44420982	5852
chr17.fa	40308910	40333296	KCNH4	0	0
chr17.fa	40336078	40337470	HCRT	0	0
chr17.fa	40341105	40346550	GHDC	7.600882377	836
chr17.fa	40351195	40428424	STAT5B	13.27185495	3040
chr17.fa	40439565	40463960	STAT5A	2.751422458	532

chr17.fa	40465343	40540405	STAT3	67.84418	14689
chr17.fa	40554467	40575338	PTRF	21.39468919	3496
chr17.fa	40610862	40674597	ATP6V0A1	7.717637694	1444
chr17.fa	40687951	40696466	NAGLU	3.645212214	456
chr17.fa	40703984	40707232	HSD17B1	9.848589035	988
chr17.fa	40714092	40718295	COASY	24.32424691	2508
chr17.fa	40719078	40725221	MLX	20.64701038	1660
chr17.fa	40725329	40729747	PSMC3IP	2.913990338	76
chr17.fa	40731526	40761357	FAM134C	9.08445328	1520
chr17.fa	40761446	40767256	TUBG1	14.51568827	1216
chr17.fa	40811266	40819024	TUBG2	6.507830212	532
chr17.fa	40819933	40829048	PLEKHH3	5.521525766	760
chr17.fa	40831420	40833845	CCR10	0	0
chr17.fa	40834632	40852011	CNTNAP1	0.309790776	76
chr17.fa	40852293	40897071	EZH1	14.81013406	3094
chr17.fa	40905947	40913211	LOC100190938	0	0
chr17.fa	40913276	40915060	RAMP2	2.340888522	76
chr17.fa	40925454	40931617	VPS25	44.51869144	2204
chr17.fa	40932649	40949084	WNK4	13.34991422	2508
chr17.fa	40949652	40950704	CCDC56	118.9629939	4028
chr17.fa	40950854	40963605	CNTD1	0.681628664	76
chr17.fa	40963673	40976310	BECN1	51.91319489	3142
chr17.fa	40985423	40995777	PSME3	36.71676752	5303
chr17.fa	40996609	41002724	AOC2	1.268518728	152
chr17.fa	41003201	41010140	AOC3	0.419874361	76
chr17.fa	41019162	41021234	LOC90586	0	0
chr17.fa	41026691	41050751	LOC388387	0.916473646	76
chr17.fa	41052815	41065386	G6PC	8.188884396	1140
chr17.fa	41102543	41132545	AARSD1	3.007394592	456
chr17.fa	41132582	41145707	RUNDC1	14.57039647	2508
chr17.fa	41150446	41154971	RPL27	1038.248553	22876
chr17.fa	41158742	41166475	IFI35	28.41757715	1596
chr17.fa	41166622	41174459	VAT1	68.63633703	8512
chr17.fa	41177258	41184058	RND2	1.221149427	228
chr17.fa	41196312	41277500	BRCA1	1.595656008	528
chr17.fa	41277600	41297125	NBR2	3.711929538	228
chr17.fa	41322498	41363707	NBR1	82.54089461	18784
chr17.fa	41363894	41371589	TMEM106A	0.60468135	76
chr17.fa	41447213	41466266	LOC100130581	0.384291788	76
chr17.fa	41476353	41478504	ARL4D	4.265460939	304
chr17.fa	41522174	41522253	MIR2117	0	0
chr17.fa	41561334	41601680	DHX8	25.35258327	4788
chr17.fa	41605211	41623762	ETV4	1.369039496	152

chr17.fa	41717758	41739262	MEOX1	0	0
chr17.fa	41831099	41836156	SOST	0	0
chr17.fa	41843489	41856368	DUSP3	29.52263843	5472
chr17.fa	41857803	41862054	C17orf105	0	0
chr17.fa	41878167	41910547	MPP3	1.192460978	152
chr17.fa	41924516	41940997	CD300LG	0	0
chr17.fa	41952727	41985113	MPP2	0.766137275	152
chr17.fa	41994576	41995355	C17orf88	0	0
chr17.fa	42018172	42019833	PPY	0	0
chr17.fa	42030107	42081837	PYY	0	0
chr17.fa	42082032	42086436	NAGS	1.631460972	152
chr17.fa	42088556	42092345	TMEM101	6.589447739	456
chr17.fa	42112003	42144987	LSM12	1.360811026	152
chr17.fa	42148098	42153712	G6PC3	2.861506043	228
chr17.fa	42154121	42201014	HDAC5	10.15882459	2432
chr17.fa	42219274	42239844	C17orf53	0	0
chr17.fa	42248074	42256451	ASB16	0	0
chr17.fa	42253352	42264082	C17orf65	4.127356078	304
chr17.fa	42264354	42269099	TMUB2	3.981467529	456
chr17.fa	42269173	42275529	ATXN7L3	20.33143743	3304
chr17.fa	42282401	42298250	UBTF	30.11909131	6536
chr17.fa	42325758	42345502	SLC4A1	1.023666147	228
chr17.fa	42385927	42396038	RUNDC3A	1.389277085	152
chr17.fa	42396993	42402217	SLC25A39	81.63554051	5910
chr17.fa	42422491	42430470	GRN	5.866009551	608
chr17.fa	42431101	42441235	FAM171A2	0	0
chr17.fa	42449550	42466873	ITGA2B	0	0
chr17.fa	42472645	42580957	GPATCH8	12.00911839	3800
chr17.fa	42634812	42638630	FZD2	0	0
chr17.fa	42733762	42753165	C17orf104	0.361163116	76
chr17.fa	42754805	42767165	CCDC43	13.43909304	1292
chr17.fa	42785976	42829636	DBF4B	1.637687923	380
chr17.fa	42836568	42859214	ADAM11	0.383847006	76
chr17.fa	42875816	42908179	GJC1	0	0
chr17.fa	42925279	42926756	HIGD1B	0	0
chr17.fa	42927655	42976993	EFTUD2	23.09709292	4173
chr17.fa	42977154	42980629	CCDC103	1.345688433	76
chr17.fa	42982993	42992914	GFAP	0	0
chr17.fa	43003448	43025082	KIF18B	0	0
chr17.fa	43037061	43045644	C1QL1	0	0
chr17.fa	43100706	43138473	DCAKD	31.77301379	3116
chr17.fa	43138680	43186382	NMT1	24.91647436	5472
chr17.fa	43189008	43209891	PLCD3	24.92025501	3876

chr17.fa	43209967	43221543	ACBD4	4.547008048	532
chr17.fa	43224684	43229468	HEXIM1	34.26268194	7372
chr17.fa	43238264	43247406	HEXIM2	7.290424427	436
chr17.fa	43299292	43324683	FMNL1	2.560166128	456
chr17.fa	43325303	43345997	LOC100133991	0	0
chr17.fa	43331760	43339479	C17orf46	0	0
chr17.fa	43340488	43394414	MAP3K14	12.73122223	2508
chr17.fa	43471268	43503012	ARHGAP27	15.28093598	2508
chr17.fa	43506718	43510282	SH3D20	1.227376378	76
chr17.fa	43513266	43568146	PLEKHM1	12.93671159	3040
chr17.fa	43583249	43597889	LRRC37A4	0.467021271	152
chr17.fa	43677491	43679748	LOC644172	0	0
chr17.fa	43697712	43715329	MGC57346	5.13145181	608
chr17.fa	43716341	43723595	C17orf69	1.794251244	76
chr17.fa	43861646	43913194	CRHR1	0	0
chr17.fa	43920722	43972879	LOC100128977	0	0
chr17.fa	43922256	43924438	IMP5	0	0
chr17.fa	43971748	44105699	MAPT	3.968124064	1216
chr17.fa	43973149	43976164	LOC100130148	0	0
chr17.fa	44076616	44077060	STH	0	0
chr17.fa	44107282	44302740	KIAA1267	29.52908777	7437
chr17.fa	44270939	44274089	LOC644246	6.475805896	152
chr17.fa	44372497	44410022	LRRC37A	0.351155517	76
chr17.fa	44376500	44657088	ARL17A	0.344039003	152
chr17.fa	44450179	44500463	NSFP1	0	0
chr17.fa	44590076	44627885	LRRC37A2	1.053688943	228
chr17.fa	44668038	44834833	NSF	31.8001455	4856
chr17.fa	44841687	44896082	WNT3	0	0
chr17.fa	44928968	44954437	WNT9B	1.154432103	76
chr17.fa	45000486	45018733	GOSR2	25.31233048	1672
chr17.fa	45055522	45056614	RPRML	0	0
chr17.fa	45195311	45266665	CDC27	6.929483702	1748
chr17.fa	45286428	45301045	MYL4	0	0
chr17.fa	45331208	45390077	ITGB3	0.345373349	76
chr17.fa	45401327	45518677	C17orf57	1.079041526	228
chr17.fa	45500843	45504058	LOC100272146	1.05102025	152
chr17.fa	45527593	45569986	MRPL45P2	0	0
chr17.fa	45608444	45700642	NPEPPS	23.04371906	4496
chr17.fa	45727275	45761004	KPNB1	57.53257273	10868
chr17.fa	45772630	45789429	TBKBP1	2.044218819	380
chr17.fa	45810610	45823485	TBX21	0	0
chr17.fa	45884733	45899147	OSBPL7	4.623065798	760
chr17.fa	45900638	45908907	MRPL10	13.54850945	1216

chr17.fa	45908993	45915046	LRRC46	0	0
chr17.fa	45915080	45918699	SCRN2	23.13801288	1596
chr17.fa	45922280	45933240	SP6	4.009266414	684
chr17.fa	45973516	46006323	SP2	8.689931502	1216
chr17.fa	46018889	46026674	PNPO	32.80980101	5112
chr17.fa	46029334	46035110	PRR15L	40.30037741	2508
chr17.fa	46048429	46059147	CDK5RAP3	52.04462802	4409
chr17.fa	46103533	46115152	COPZ2	10.73770858	436
chr17.fa	46114527	46114613	MIR152	0	0
chr17.fa	46125686	46138907	NFE2L1	21.15317248	4636
chr17.fa	46147414	46178883	CBX1	15.78998916	1900
chr17.fa	46184920	46200105	SNX11	10.55468072	1140
chr17.fa	46210802	46507594	SKAP1	9.651995319	684
chr17.fa	46233789	46233873	MIR1203	0	0
chr17.fa	46606807	46608272	HOXB1	0	0
chr17.fa	46620019	46622393	HOXB2	11.60547858	836
chr17.fa	46626232	46651810	HOXB3	6.073055648	988
chr17.fa	46652869	46655743	HOXB4	4.988231954	456
chr17.fa	46657200	46657309	MIR10A	0	0
chr17.fa	46667823	46683774	LOC404266	0	0
chr17.fa	46668619	46671103	HOXB5	1.933912843	152
chr17.fa	46673099	46682334	HOXB6	11.19316551	760
chr17.fa	46684595	46688383	HOXB7	9.898627029	608
chr17.fa	46689708	46692301	HOXB8	8.344335761	684
chr17.fa	46698519	46703835	HOXB9	4.381993866	532
chr17.fa	46709852	46709921	MIR196A1	0	0
chr17.fa	46799082	46799882	PRAC	194.1712098	3344
chr17.fa	46800542	46802119	NCRNA00253	20.44552406	456
chr17.fa	46802127	46806111	HOXB13	159.2191155	21736
chr17.fa	46839593	46894469	TTLL6	0	0
chr17.fa	46908372	46942229	CALCOCO2	36.3807346	5369
chr17.fa	46970148	46973232	ATP5G1	71.54343325	1853
chr17.fa	46985731	47006422	UBE2Z	44.87651869	6308
chr17.fa	47007459	47022154	SNF8	41.72812815	2276
chr17.fa	47035918	47045955	GIP	0	0
chr17.fa	47074774	47133507	IGF2BP1	0.192813067	76
chr17.fa	47209822	47247351	B4GALNT2	0.801052675	76
chr17.fa	47283596	47286762	GNGT2	0	0
chr17.fa	47287589	47300587	ABI3	0.854426534	76
chr17.fa	47300732	47308128	PHOSPHO1	0	0
chr17.fa	47325605	47336027	FLJ40194	0	0
chr17.fa	47366568	47439835	ZNF652	16.67799675	8664
chr17.fa	47481420	47492242	PHB	65.5724551	5384

chr17.fa	47572655	47592382	NGFR	0	0
chr17.fa	47653298	47661171	NXPH3	0.30445339	76
chr17.fa	47676246	47755525	SPOP	18.28833057	2583
chr17.fa	47778690	47785282	SLC35B1	11.40087878	608
chr17.fa	47787687	47841518	FAM117A	10.73815336	1140
chr17.fa	47865981	47906458	MYST2	26.88708173	4408
chr17.fa	47915671	47925379	TAC4	0	0
chr17.fa	48046562	48052323	DLX4	0	0
chr17.fa	48067369	48072588	DLX3	1.948590654	228
chr17.fa	48133340	48167849	ITGA3	3.362108367	760
chr17.fa	48172101	48188733	PDK2	13.6746052	1900
chr17.fa	48190035	48207167	SAMD14	0	0
chr17.fa	48211101	48227877	PPP1R9B	12.1085272	2204
chr17.fa	48243366	48253293	SGCA	0	0
chr17.fa	48248789	48249837	HILS1	0	0
chr17.fa	48261457	48279000	COL1A1	0.85553849	228
chr17.fa	48348767	48358846	TMEM92	0	0
chr17.fa	48423393	48438512	XYLT2	2.369132189	380
chr17.fa	48445228	48450562	MRPL27	50.48878001	1596
chr17.fa	48450581	48458593	EME1	0	0
chr17.fa	48458821	48474914	LRRC59	57.73227992	6978
chr17.fa	48503519	48552200	ACSF2	21.13649315	2128
chr17.fa	48541853	48546227	CHAD	0	0
chr17.fa	48556190	48563336	RSAD1	14.2032288	1596
chr17.fa	48585745	48608862	MYCBPAP	0	0
chr17.fa	48610048	48621111	EPN3	31.41896718	5548
chr17.fa	48624562	48633213	SPATA20	30.4711364	3572
chr17.fa	48638449	48704431	CACNA1G	0	0
chr17.fa	48712218	48769063	ABCC3	1.308771513	304
chr17.fa	48770551	48785270	ANKRD40	15.40569737	2888
chr17.fa	48796926	48830072	LUC7L3	21.84125049	1976
chr17.fa	48834759	48844918	C17orf73	0	0
chr17.fa	48912605	48919709	WFIKKN2	0	0
chr17.fa	48939587	48941413	TOB1	69.38312628	5700
chr17.fa	49039535	49198226	SPAG9	105.3837945	39203
chr17.fa	49230897	49230919	NME1-NME2	0	0
chr17.fa	49239089	49239450	NME1	48.1027461	783
chr17.fa	49242796	49244187	NME2	18.24918974	526
chr17.fa	49254786	49337427	MBTD1	10.5575718	2555
chr17.fa	49337897	49375292	UTP18	18.83941567	1596
chr17.fa	49707674	50237377	CA10	0	0
chr17.fa	51900239	51902573	KIF2B	0	0
chr17.fa	52978052	53037988	TOM1L1	92.02876531	6650

chr17.fa	53029259	53046064	COX11	3.407698539	304
chr17.fa	53046126	53241449	STXBP4	4.472729427	1216
chr17.fa	53342321	53402426	HLF	0.301784697	76
chr17.fa	53469974	53499341	MMD	0	0
chr17.fa	53796988	53809482	TMEM100	0	0
chr17.fa	53828340	53854748	PCTP	17.71367202	1824
chr17.fa	54230836	54560007	ANKFN1	9.753628044	1064
chr17.fa	54671060	54672951	NOG	0	0
chr17.fa	54869274	54893250	C17orf67	2.081580521	76
chr17.fa	54911460	54946036	DGKE	1.08793717	380
chr17.fa	54961463	54962274	MTVR2	0	0
chr17.fa	54965270	54991409	TRIM25	22.02605747	5689
chr17.fa	55015561	55038411	COIL	5.77727551	684
chr17.fa	55055468	55084129	SCPEP1	1.7379863	152
chr17.fa	55122839	55124156	RNF126P1	0	0
chr17.fa	55162553	55198710	AKAP1	8.945014072	1596
chr17.fa	55333931	55757299	MSI2	24.89267851	3424
chr17.fa	55916287	55927433	MRPS23	36.25041343	2432
chr17.fa	55940337	55980750	CUEDC1	12.33136307	1216
chr17.fa	56048910	56065615	VEZF1	12.76836154	2660
chr17.fa	56078280	56084707	SRSF1	21.56526315	5472
chr17.fa	56160780	56167618	DYNLL2	225.4302778	15428
chr17.fa	56232515	56233447	OR4D1	0	0
chr17.fa	56234320	56236480	MSX2P1	0	0
chr17.fa	56247017	56247940	OR4D2	0	0
chr17.fa	56270089	56282535	EPX	0	0
chr17.fa	56282797	56296966	MKS1	1.994625608	228
chr17.fa	56315787	56345879	LPO	0	0
chr17.fa	56347217	56358296	MPO	0	0
chr17.fa	56378594	56406152	BZRAP1	2.856613439	988
chr17.fa	56408593	56408679	MIR142	0	0
chr17.fa	56422539	56429563	SUPT4H1	38.26505423	2562
chr17.fa	56431038	56494931	RNF43	7.417854517	1520
chr17.fa	56497529	56565759	HSF5	0	0
chr17.fa	56566893	56595251	MTMR4	31.89555127	8360
chr17.fa	56597611	56618179	4-Sep	0.728775573	76
chr17.fa	56618948	56621683	C17orf47	0	0
chr17.fa	56634039	56769416	TEX14	0	0
chr17.fa	56769963	56811692	RAD51C	11.8443266	760
chr17.fa	56833232	57062534	PPM1E	1.35124821	380
chr17.fa	57075561	57184266	TRIM37	11.9764269	2432
chr17.fa	57187308	57232800	SKA2	5.907819075	836
chr17.fa	57215119	57215233	MIR454	0	0

chr17.fa	57228497	57228582	MIR301A	0	0
chr17.fa	57232860	57284070	PRR11	0.261087129	76
chr17.fa	57287371	57292611	C17orf71	6.210048554	912
chr17.fa	57297828	57353330	GDPD1	0	0
chr17.fa	57409053	57479095	YPEL2	13.89099172	3268
chr17.fa	57642886	57685713	DHX40	14.62732859	2356
chr17.fa	57697050	57774317	CLTC	83.17804505	32072
chr17.fa	57774667	57784856	PTRH2	6.353935584	228
chr17.fa	57784863	57917952	TMEM49	10.10678508	988
chr17.fa	57918627	57918698	MIR21	0	0
chr17.fa	57936841	57970306	TUBD1	3.898960437	456
chr17.fa	57970443	58027786	RPS6KB1	7.874423407	1883
chr17.fa	58029723	58042117	RNFT1	0.838414376	76
chr17.fa	58053385	58096336	DHX40P1	0	0
chr17.fa	58120552	58156292	HEATR6	11.16759054	1976
chr17.fa	58160927	58165828	LOC645638	40.82544275	1140
chr17.fa	58179121	58180280	LOC653653	0	0
chr17.fa	58227302	58236906	CA4	0	0
chr17.fa	58254691	58469586	USP32	12.73077745	4022
chr17.fa	58308877	58309006	SCARNA20	0	0
chr17.fa	58499865	58508787	C17orf64	0	0
chr17.fa	58520520	58603580	APPBP2	15.15617458	4408
chr17.fa	58677544	58743640	PPM1D	4.599492343	988
chr17.fa	58755172	59470199	BCAS3	12.6447121	2052
chr17.fa	59477257	59486827	TBX2	51.50532965	7828
chr17.fa	59489112	59490641	C17orf82	0	0
chr17.fa	59533807	59561664	TBX4	0	0
chr17.fa	59667794	59668563	NACA2	0	0
chr17.fa	59756547	59940920	BRIP1	0.620915899	228
chr17.fa	59942728	60005377	INTS2	2.468541002	684
chr17.fa	60019966	60142643	MED13	17.6042556	8284
chr17.fa	60342067	60353016	TBC1D3P2	0	0
chr17.fa	60447579	60493839	EFCAB3	0	0
chr17.fa	60501246	60527454	METTL2A	4.025723354	456
chr17.fa	60556386	60692841	TLK2	9.56370606	2339
chr17.fa	60704762	60770952	MRC2	0.282436673	76
chr17.fa	60778675	60885705	10-Mar	0	0
chr17.fa	60798858	61268734	MIR548W	0	0
chr17.fa	61086898	61505067	TANC2	30.3588289	16036
chr17.fa	61509665	61523722	CYB561	12.46079468	1900
chr17.fa	61554422	61575741	ACE	3.886284146	912
chr17.fa	61600695	61626338	KCNH6	0.472136266	76
chr17.fa	61627822	61671642	DCAF7	36.96829184	10564

chr17.fa	61678231	61685725	TACO1	10.35497353	684
chr17.fa	61699801	61773248	MAP3K3	8.443077402	1672
chr17.fa	61773671	61777519	LIMD2	1.206026834	152
chr17.fa	61780192	61819330	STRADA	8.678589557	912
chr17.fa	61822610	61851088	CCDC47	2.966029851	456
chr17.fa	61851567	61896677	DDX42	47.40777397	8512
chr17.fa	61896793	61905031	FTSJ3	12.23351099	1900
chr17.fa	61905044	61909387	PSMC5	78.27565606	5262
chr17.fa	61909441	61920351	SMARCD2	41.25866058	5139
chr17.fa	61934376	61941739	TCAM1P	0	0
chr17.fa	61949372	61951089	CSH2	0	0
chr17.fa	61957572	61959223	GH2	0	0
chr17.fa	61972273	61973987	CSH1	0	0
chr17.fa	61986965	61988618	CSHL1	0	0
chr17.fa	61994563	61996198	GH1	0	0
chr17.fa	62006098	62009704	CD79B	0	0
chr17.fa	62015914	62050278	SCN4A	0	0
chr17.fa	62075711	62081644	C17orf72	0	0
chr17.fa	62082467	62097994	ICAM2	0	0
chr17.fa	62120390	62207502	ERN1	4.220093159	760
chr17.fa	62223438	62223517	SNORD104	0	0
chr17.fa	62223699	62223831	SNORA76	0	0
chr17.fa	62224795	62340653	TEX2	10.29381598	2356
chr17.fa	62396777	62407083	PECAM1	0	0
chr17.fa	62461569	62464760	C17orf60	0	0
chr17.fa	62473902	62493184	POLG2	4.241442703	304
chr17.fa	62494374	62502484	DDX5	93.77231139	15850
chr17.fa	62503158	62534062	CCDC45	9.411812952	1138
chr17.fa	62540735	62658386	SMURF2	6.995089071	1216
chr17.fa	62745780	62778117	LOC146880	2.721622053	608
chr17.fa	62780959	62833302	PLEKHM1P	0.583109415	76
chr17.fa	62850488	62914903	LRRC37A3	0	0
chr17.fa	62962668	62971703	AMZ2P1	1.473118523	228
chr17.fa	63005407	63052920	GNA13	10.97322073	3114
chr17.fa	63133456	63223821	RGS9	0	0
chr17.fa	63524683	63557740	AXIN2	3.594507047	684
chr17.fa	63631658	64188212	CCDC46	2.52147008	456
chr17.fa	64208147	64225556	APOH	1.407290762	76
chr17.fa	64298926	64806862	PRKCA	4.61528211	1820
chr17.fa	64783190	64783286	MIR634	0	0
chr17.fa	64873391	64881395	CACNG5	0	0
chr17.fa	64960980	65029518	CACNG4	17.73946938	2736
chr17.fa	65040652	65052911	CACNG1	0	0

chr17.fa	65066554	65241319	HELZ	6.890342872	4285
chr17.fa	65336619	65362721	PSMD12	25.39483757	2052
chr17.fa	65373924	65689647	PITPNC1	8.819585502	836
chr17.fa	65714061	65740266	NOL11	21.35110054	2356
chr17.fa	65736786	65736917	SNORA38B	0	0
chr17.fa	65821780	65980494	BPTF	30.6230295	15549
chr17.fa	65987217	65989765	C17orf58	8.645453286	608
chr17.fa	66031848	66042970	KPNA2	8.536259265	760
chr17.fa	66097696	66132070	LOC100499466	6.795159489	3420
chr17.fa	66194801	66196443	LOC440461	0	0
chr17.fa	66244145	66253305	AMZ2	42.68107393	3800
chr17.fa	66255323	66417000	ARSG	0.610241127	76
chr17.fa	66263167	66287405	SLC16A6	0	0
chr17.fa	66417422	66453653	WIPI1	26.35401031	2280
chr17.fa	66420592	66420689	MIR635	0	0
chr17.fa	66508110	66528910	PRKAR1A	66.42999511	11330
chr17.fa	66531257	66597095	FAM20A	1.159547098	228
chr17.fa	66863431	66951533	ABCA8	0	0
chr17.fa	66970773	67057136	ABCA9	0	0
chr17.fa	67074847	67138015	ABCA6	0	0
chr17.fa	67144148	67225001	ABCA10	0	0
chr17.fa	67240576	67323323	ABCA5	0.557534441	228
chr17.fa	67410838	67538470	MAP2K6	2.712948801	228
chr17.fa	68071426	68131746	KCNJ16	5.999221809	1140
chr17.fa	68163102	68165543	FLJ36644	0	0
chr17.fa	68165676	68176183	KCNJ2	0	0
chr17.fa	70117161	70122560	SOX9	20.62232497	3648
chr17.fa	70399463	70588943	LOC100499467	6.73978411	684
chr17.fa	70594180	70636611	FLJ26484	0	0
chr17.fa	70642085	71088853	SLC39A11	1.183120553	148
chr17.fa	71161160	71168062	SSTR2	0	0
chr17.fa	71189173	71203395	COG1	8.328990777	1064
chr17.fa	71203492	71228533	FAM104A	6.271428493	760
chr17.fa	71228776	71245095	C17orf80	8.614096143	1444
chr17.fa	71248769	71258019	CPSF4L	0	0
chr17.fa	71279763	71308143	CDC42EP4	21.80166487	3040
chr17.fa	71330523	71640227	SDK2	0.157675277	76
chr17.fa	71745409	71824676	C17orf54	0	0
chr17.fa	72199795	72206019	RPL38	1658.838204	26629
chr17.fa	72206135	72209460	MGC16275	0.680071927	76
chr17.fa	72209696	72258157	TTYH2	0	0
chr17.fa	72270386	72311023	DNAI2	0	0
chr17.fa	72322351	72351959	KIF19	0	0

chr17.fa	72352555	72357958	BTBD17	0	0
chr17.fa	72363645	72368739	GPR142	0	0
chr17.fa	72427667	72443568	GPRC5C	8.332771425	912
chr17.fa	72462522	72480933	CD300A	0	0
chr17.fa	72517313	72527613	CD300LB	0	0
chr17.fa	72537247	72542282	CD300C	0	0
chr17.fa	72576111	72584988	CD300LD	0	0
chr17.fa	72581057	72590348	C17orf77	0	0
chr17.fa	72606022	72619897	CD300E	0	0
chr17.fa	72667256	72743474	RAB37	0.46902279	76
chr17.fa	72690452	72709108	CD300LF	0	0
chr17.fa	72744752	72744762	MIR3615	0	0
chr17.fa	72744839	72765499	SLC9A3R1	68.94345911	5977
chr17.fa	72766686	72772470	NAT9	15.32430224	1292
chr17.fa	72772622	72835922	TMEM104	0.711873851	152
chr17.fa	72838168	72856007	GRIN2C	0	0
chr17.fa	72858619	72869156	FDXR	1.795140808	152
chr17.fa	72873473	72889705	FADS6	0	0
chr17.fa	72912176	72919351	USH1G	0.474582567	76
chr17.fa	72920370	72930006	OTOP2	0	0
chr17.fa	72931897	72945511	OTOP3	0	0
chr17.fa	72946839	72968900	C17orf28	30.8300756	4633
chr17.fa	72983727	73001892	CDR2L	10.52465792	1672
chr17.fa	73008780	73017356	ICT1	9.614188836	380
chr17.fa	73034955	73043074	ATP5H	176.3038656	4931
chr17.fa	73043279	73061981	KCTD2	14.62554946	2432
chr17.fa	73084055	73102248	SLC16A5	8.780222281	760
chr17.fa	73106082	73126322	ARMC7	8.094590577	760
chr17.fa	73126361	73127877	NT5C	20.58896631	836
chr17.fa	73131344	73150774	HN1	24.04981631	1833
chr17.fa	73163825	73179098	SUMO2	53.88713813	2583
chr17.fa	73201597	73231854	NUP85	6.582331224	684
chr17.fa	73232694	73258444	GGA3	10.64541628	1976
chr17.fa	73257749	73262309	MRPS7	48.02379727	2736
chr17.fa	73262458	73267303	MIF4GD	5.352953327	304
chr17.fa	73267380	73269976	LOC100287042	0.872662603	76
chr17.fa	73273434	73285530	SLC25A19	0	0
chr17.fa	73314157	73401790	GRB2	44.50512558	6612
chr17.fa	73402150	73402243	MIR3678	0	0
chr17.fa	73452664	73496341	KIAA0195	3.526455376	836
chr17.fa	73496534	73511627	CASKIN2	4.790971064	1064
chr17.fa	73512609	73520820	TSEN54	8.707722788	760
chr17.fa	73521783	73571290	LLGL2	22.30804937	3559

chr17.fa	73584139	73622927	MYO15B	1.258066347	532
chr17.fa	73622950	73663269	RECQL5	5.062955357	1368
chr17.fa	73629514	73637486	LOC643008	3.301840384	760
chr17.fa	73642646	73644057	LOC100130933	10.20641629	380
chr17.fa	73663399	73704139	SAP30BP	5.897366694	668
chr17.fa	73717516	73753899	ITGB4	2.780778081	760
chr17.fa	73754018	73761280	GALK1	11.17670857	684
chr17.fa	73772515	73775860	H3F3B	104.3470073	12692
chr17.fa	73780681	73821886	UNK	5.009136715	912
chr17.fa	73823308	73840798	UNC13D	16.15137467	3189
chr17.fa	73841780	73851501	WBP2	93.02908039	8132
chr17.fa	73870245	73874656	TRIM47	2.240590144	228
chr17.fa	73885041	73893054	TRIM65	6.941048038	1064
chr17.fa	73894724	73901181	MRPL38	17.16992582	1216
chr17.fa	73906618	73937119	FBF1	1.750662592	304
chr17.fa	73937589	73975300	ACOX1	64.28103009	22268
chr17.fa	73975516	73996667	C17orf106	23.86634367	836
chr17.fa	73996987	74002080	CDK3	4.306158507	304
chr17.fa	74002927	74023507	EVPL	332.5871964	98509
chr17.fa	74035191	74068607	SRP68	50.14629775	5671
chr17.fa	74070892	74073573	GALR2	0	0
chr17.fa	74075263	74076505	ZACN	0	0
chr17.fa	74077086	74099868	EXOC7	29.61626508	5512
chr17.fa	74132415	74137380	FOXJ1	0	0
chr17.fa	74138534	74236390	RNF157	0.691191481	152
chr17.fa	74261286	74267379	FAM100B	32.86184052	2348
chr17.fa	74270130	74303761	QRICH2	1.261624604	304
chr17.fa	74306868	74350230	PRPSAP1	10.03072733	1064
chr17.fa	74380690	74383941	SPHK1	0	0
chr17.fa	74385613	74449288	UBE2O	11.04683218	2660
chr17.fa	74449433	74466199	AANAT	0	0
chr17.fa	74466975	74497509	RHBDF2	0.480364736	76
chr17.fa	74523440	74533782	CYGB	0	0
chr17.fa	74536121	74541458	PRCD	0	0
chr17.fa	74554874	74554951	SNORD1C	0	0
chr17.fa	74557190	74557275	SNORD1B	0	0
chr17.fa	74557715	74557788	SNORD1A	0	0
chr17.fa	74561461	74582145	ST6GALNAC2	0	0
chr17.fa	74620845	74639894	ST6GALNAC1	0	0
chr17.fa	74671809	74707056	MXRA7	5.639615431	988
chr17.fa	74708914	74722881	JMJD6	1.183342944	304
chr17.fa	74722950	74729962	C17orf95	18.45512388	912
chr17.fa	74730199	74733493	SRSF2	31.55440335	4180

chr17.fa	74732547	74732630	MIR636	0	0
chr17.fa	74733783	74775338	MFSD11	5.346948768	603
chr17.fa	74864798	74946471	MGAT5B	0	0
chr17.fa	75084725	75091068	C17orf86	1.558516698	152
chr17.fa	75085389	75085575	SCARNA16	0	0
chr17.fa	75137005	75213181	SEC14L1	23.84165826	6070
chr17.fa	75277492	75496678	9-Sep	27.76263542	8208
chr17.fa	75393066	75393136	MIR4316	0	0
chr17.fa	75875083	75880169	FLJ45079	0	0
chr17.fa	76000318	76104916	TNRC6C	4.147593666	1824
chr17.fa	76108999	76128439	TMC6	10.45816299	1368
chr17.fa	76126859	76139049	TMC8	0	0
chr17.fa	76142434	76162364	C17orf99	0	0
chr17.fa	76164671	76169009	SYNGR2	16.96154538	1292
chr17.fa	76170160	76183285	TK1	1.059693502	76
chr17.fa	76183398	76203782	AFMID	25.2147008	2432
chr17.fa	76210277	76221716	BIRC5	0	0
chr17.fa	76219545	76220144	EPR1	2.817027827	76
chr17.fa	76227391	76237068	LOC283999	0	0
chr17.fa	76352859	76356158	SOCS3	1.854519227	228
chr17.fa	76374735	76420639	PGS1	3.222446768	304
chr17.fa	76419778	76573476	DNAH17	0.124094223	76
chr17.fa	76670130	76778376	CYTH1	26.03398954	3876
chr17.fa	76792965	76836969	USP36	7.750106792	1824
chr17.fa	76849059	76921472	TIMP2	0.462351058	76
chr17.fa	76967335	76976061	LGALS3BP	6.730666076	684
chr17.fa	76987798	77005899	CANT1	14.08891978	2204
chr17.fa	77020251	77045870	C1QTNF1	0	0
chr17.fa	77071019	77084681	ENGASE	12.04581292	2432
chr17.fa	77085427	77478563	RBFOX3	0.630923498	76
chr17.fa	77704882	77716021	ENPP7	0	0
chr17.fa	77751977	77761449	CBX2	0.338256835	76
chr17.fa	77768176	77770915	CBX8	2.186549111	152
chr17.fa	77806955	77813213	CBX4	11.36729773	1354
chr17.fa	77913819	78009647	TBC1D16	32.6728081	4788
chr17.fa	78010435	78074412	CCDC40	2.755203106	532
chr17.fa	78075355	78093679	GAA	2.354009596	380
chr17.fa	78109013	78120982	EIF4A3	47.46648522	3701
chr17.fa	78152281	78182851	CARD14	2.901091656	527
chr17.fa	78183079	78194199	SGSH	1.221149427	152
chr17.fa	78194200	78227308	SLC26A11	1.660816595	228
chr17.fa	78234667	78370086	RNF213	16.82210617	15350
chr17.fa	78325631	78388966	LOC100294362	0.53885359	76

chr17.fa	78388969	78411884	FLJ35220	4.532552628	684
chr17.fa	78440633	78450404	NPTX1	0	0
chr17.fa	78518625	78940173	RPTOR	7.149206091	2204
chr17.fa	78965641	78973933	CHMP6	9.141607788	684
chr17.fa	79002933	79008519	FLJ90757	10.32228204	2052
chr17.fa	79008947	79091095	BAIAP2	17.98276522	3849
chr17.fa	79091233	79139872	AATK	0	0
chr17.fa	79099076	79099173	MIR657	0	0
chr17.fa	79099677	79099755	MIR3065	0	0
chr17.fa	79106996	79107108	MIR1250	0	0
chr17.fa	79139307	79156964	LOC388428	0	0
chr17.fa	79163393	79196751	AZI1	1.868085083	304
chr17.fa	79202077	79212891	C17orf56	0.739005563	76
chr17.fa	79213111	79215098	C17orf89	75.45418041	1900
chr17.fa	79218799	79269096	SLC38A10	2.407605846	532
chr17.fa	79276624	79283048	C17orf55	0	0
chr17.fa	79285072	79304474	TMEM105	0.509275576	76
chr17.fa	79373540	79433358	BAHCC1	1.660816595	760
chr17.fa	79418130	79418214	MIR3186	0	0
chr17.fa	79476997	79479892	ACTG1	707.3833305	66956
chr17.fa	79495417	79504156	FSCN2	0.970292288	76
chr17.fa	79506911	79519429	C17orf70	5.26933428	912
chr17.fa	79523913	79604138	NPLOC4	23.5690068	4643
chr17.fa	79609349	79615779	TSPAN10	2.095591159	152
chr17.fa	79617489	79623607	PDE6G	0	0
chr17.fa	79632066	79633618	C17orf90	10.9042795	304
chr17.fa	79633761	79640936	CCDC137	9.621527741	912
chr17.fa	79648224	79650954	ARL16	7.001538413	380
chr17.fa	79650962	79669151	HGS	20.27116945	2709
chr17.fa	79670400	79674556	MRPL12	38.29952485	1748
chr17.fa	79679371	79688042	SLC25A10	20.43395972	1732
chr17.fa	79762010	79771889	GCGR	2.472321651	228
chr17.fa	79780293	79791167	FAM195B	38.60108715	2128
chr17.fa	79791368	79792926	DYSFIP1	0	0
chr17.fa	79801034	79818544	P4HB	60.31646429	6992
chr17.fa	79825597	79829282	ARHGDIA	100.5594648	9880
chr17.fa	79845711	79849462	THOC4	26.19233199	1292
chr17.fa	79849599	79858363	ANAPC11	45.73917369	2793
chr17.fa	79860072	79860776	NPB	2.761652448	76
chr17.fa	79860782	79869340	PCYT2	26.42962327	4232
chr17.fa	79869815	79876058	SIRT7	8.787783578	684
chr17.fa	79876146	79885588	MAFG	3.234900669	759
chr17.fa	79885705	79888629	LOC92659	2.67580949	228

chr17.fa	79890267	79894968	PYCR1	28.70268252	2660
chr17.fa	79897521	79905109	MYADML2	0	0
chr17.fa	79910383	79919057	NOTUM	0	0
chr17.fa	79935426	79975282	ASPSCR1	9.17074102	760
chr17.fa	79976579	79980773	STRA13	92.33788891	3031
chr17.fa	79981280	79989027	LRRC45	4.552123043	532
chr17.fa	79989532	79992080	RAC3	9.567041926	456
chr17.fa	79993757	79995573	DCXR	321.8321413	11910
chr17.fa	80005778	80009650	RFNG	2.773883957	228
chr17.fa	80009763	80015346	GPS1	36.460573	3056
chr17.fa	80015751	80023680	DUS1L	39.72972189	3330
chr17.fa	80036214	80056106	FASN	69.54124634	26448
chr17.fa	80059346	80170689	CCDC57	5.639615431	760
chr17.fa	80186293	80197369	SLC16A3	2.263718817	228
chr17.fa	80202240	80231573	CSNK1D	47.93639757	4408
chr17.fa	80272746	80275480	CD7	0	0
chr17.fa	80278900	80291921	SECTM1	0	0
chr17.fa	80317123	80321652	TEX19	0	0
chr17.fa	80332201	80333370	UTS2R	0	0
chr17.fa	80347086	80373503	C17orf101	3.548249702	684
chr17.fa	80376252	80400462	HEXDC	4.351526288	380
chr17.fa	80400517	80408707	C17orf62	1.744213251	228
chr17.fa	80416060	80446143	NARF	16.2205383	1520
chr17.fa	80477594	80562483	FOXK2	14.45408594	3420
chr17.fa	80572438	80606411	WDR45L	63.3732297	7372
chr17.fa	80614943	80656598	RAB40B	14.41316598	1140
chr17.fa	80674582	80685893	FN3KRP	25.91723422	2128
chr17.fa	80693452	80709073	FN3K	33.02507557	2128
chr17.fa	80709940	80901062	TBCD	12.03269184	2398
chr17.fa	80787310	80797931	ZNF750	16.41980071	2356
chr17.fa	80901667	81009686	B3GNTL1	0	0
chr17.fa	81037567	81052591	METRNL	8.882299787	532
chr17.fa	81174666	81188573	FLJ43681	0	0
chr18.fa	109065	122222	ROCK1P1	0	0
chr18.fa	158483	213739	USP14	35.89503248	6708
chr18.fa	214520	268059	THOC1	3.75551819	358
chr18.fa	319355	500729	COLEC12	0.539298372	76
chr18.fa	580369	581524	CETN1	0	0
chr18.fa	596998	650293	CLUL1	1.752441721	152
chr18.fa	649620	658340	C18orf56	2.518801387	76
chr18.fa	657604	673499	TYMS	1.670157021	76
chr18.fa	670324	712662	ENOSF1	9.760077385	1748
chr18.fa	721592	812327	YES1	11.54432103	2432

chr18.fa	904944	912173	ADCYAP1	0	0
chr18.fa	1268312	1359630	C18orf2	0	0
chr18.fa	2537524	2571489	METTL4	1.840063807	304
chr18.fa	2571510	2616634	NDC80	0	0
chr18.fa	2652722	2653231	LOC645158	0	0
chr18.fa	2655886	2805015	SMCHD1	11.49917564	4484
chr18.fa	2847028	2914090	EMILIN2	2.951129649	532
chr18.fa	2916992	3011945	LPIN2	7.59754651	2128
chr18.fa	2943213	2946621	LOC727896	0	0
chr18.fa	3066805	3220106	MYOM1	0.867102826	228
chr18.fa	3247528	3256234	MYL12A	145.7755747	8056
chr18.fa	3262111	3278282	MYL12B	282.6519478	14972
chr18.fa	3412072	3458406	TGIF1	11.93728607	1292
chr18.fa	3498837	3880135	DLGAP1	0.495264938	76
chr18.fa	3594112	3597377	FLJ35776	8.510461899	380
chr18.fa	4264602	4296000	LOC284215	0	0
chr18.fa	5143672	5197255	LOC642597	0	0
chr18.fa	5236723	5238028	C18orf18	5.176597199	304
chr18.fa	5238099	5246505	LOC339290	5.524861633	988
chr18.fa	5289018	5296039	ZFP161	5.668526271	912
chr18.fa	5392388	5543986	EPB41L3	10.26423797	2052
chr18.fa	5890184	5892103	TMEM200C	0	0
chr18.fa	5954705	6414910	L3MBTL4	4.240775529	684
chr18.fa	6374360	6374424	MIR4317	0	0
chr18.fa	6834432	6915712	ARHGAP28	2.750310502	684
chr18.fa	6925473	6929868	LOC400643	0	0
chr18.fa	6941886	7117813	LAMA1	0.532849031	228
chr18.fa	7231137	7232042	LRRC30	0	0
chr18.fa	7567314	8406859	PTPRM	5.432791725	1368
chr18.fa	8360818	8367032	LOC100192426	2.743861161	76
chr18.fa	8609443	8639380	RAB12	11.85811485	1140
chr18.fa	8717369	8832775	KIAA0802	11.92994717	3268
chr18.fa	9102628	9134343	NDUFV2	101.7021102	4253
chr18.fa	9136758	9285206	ANKRD12	15.57249069	7372
chr18.fa	9334765	9402418	TWSG1	0.449897157	76
chr18.fa	9475530	9538106	RALBP1	124.6083915	24396
chr18.fa	9546792	9614600	PPP4R1	31.85618805	5608
chr18.fa	9708228	9862553	RAB31	1.69261852	304
chr18.fa	9885723	9888156	TXNDC2	0	0
chr18.fa	9913955	9960018	VAPA	19.33290148	6080
chr18.fa	10454625	10488698	APCDD1	3.298504518	380
chr18.fa	10525873	10552766	NAPG	13.78046335	2302
chr18.fa	10670244	11148761	FAM38B	0	0

chr18.fa	11689136	11883144	GNAL	0.349821171	76
chr18.fa	11851389	11854448	CHMP1B	27.61719165	3800
chr18.fa	11883472	11908641	MPPE1	1.205582052	152
chr18.fa	11981455	12030876	IMPA2	42.81762206	2888
chr18.fa	12254318	12277594	CIDEA	0	0
chr18.fa	12308257	12326568	TUBB6	3.724828221	304
chr18.fa	12328943	12377275	AFG3L2	22.12124086	3192
chr18.fa	12407895	12432236	SLMO1	0	0
chr18.fa	12446511	12657912	SPIRE1	9.332641727	2319
chr18.fa	12658738	12725739	PSMG2	18.95795012	988
chr18.fa	12672631	12702703	CEP76	3.534683846	456
chr18.fa	12785480	12884334	PTPN2	10.72747859	1368
chr18.fa	12947983	12987536	SEH1L	10.60583067	1672
chr18.fa	12991361	13125051	CEP192	8.476213673	3040
chr18.fa	13218786	13652753	C18orf1	4.206972085	1900
chr18.fa	13663346	13726591	C18orf19	3.46974565	684
chr18.fa	13726704	13764554	RNMT	9.536574348	2660
chr18.fa	13825543	13826861	MC5R	0	0
chr18.fa	13882043	13915535	MC2R	0	0
chr18.fa	14075989	14132489	ZNF519	0.190366766	76
chr18.fa	14337422	14342523	LOC284233	0	0
chr18.fa	14477954	14498705	CXADRP3	0	0
chr18.fa	14511737	14543599	POTEC	0	0
chr18.fa	14748239	14852737	ANKRD30B	0	0
chr18.fa	14830165	14830241	MIR3156-2	0	0
chr18.fa	15313555	15325918	LOC644669	0	0
chr18.fa	18529703	18691812	ROCK1	18.25497191	5457
chr18.fa	18822203	19102791	GREB1L	0	0
chr18.fa	19109262	19180693	ESCO1	5.635167609	1140
chr18.fa	19192260	19210208	SNRPD1	25.35258327	1824
chr18.fa	19230858	19284766	ABHD3	0	0
chr18.fa	19263471	19263558	MIR320C1	0	0
chr18.fa	19321545	19450912	MIB1	21.61307724	9044
chr18.fa	19405659	19405746	MIR133A1	0	0
chr18.fa	19408965	19409049	MIR1-2	0	0
chr18.fa	19749416	19782227	GATA6	1.935024798	304
chr18.fa	19993564	19997878	CTAGE1	0	0
chr18.fa	20513295	20606449	RBBP8	13.78757987	2204
chr18.fa	20714528	20840434	CABLES1	2.589744142	608
chr18.fa	20875979	21017925	C18orf45	1.129746693	152
chr18.fa	21032787	21063099	RIOK3	14.66802616	2660
chr18.fa	21083462	21110576	C18orf8	12.58199782	1140
chr18.fa	21111463	21166581	NPC1	5.805741568	1216

chr18.fa	21179978	21242849	ANKRD29	3.717934097	380
chr18.fa	21269562	21535029	LAMA3	1.105506065	532
chr18.fa	21572737	21715574	TTC39C	3.132600771	836
chr18.fa	21718955	21741564	CABYR	2.140291766	228
chr18.fa	21742011	21977790	OSBPL1A	11.33860928	2432
chr18.fa	21901650	21901699	MIR320C2	0	0
chr18.fa	22006609	22033494	IMPACT	11.69354545	1976
chr18.fa	22040593	22059921	HRH4	0	0
chr18.fa	22641888	22932214	ZNF521	0	0
chr18.fa	23596217	23670611	SS18	25.578755	3952
chr18.fa	23713816	23773319	PSMA8	0	0
chr18.fa	23806409	23971650	TAF4B	0.711429069	152
chr18.fa	24034875	24220308	KCTD1	4.122463474	760
chr18.fa	24267585	24283602	LOC728606	3.903408259	456
chr18.fa	24432008	24445716	AQP4	0	0
chr18.fa	24445272	24515910	C18orf16	0	0
chr18.fa	24495595	24765289	CHST9	1.505142838	152
chr18.fa	25530930	25757445	CDH2	0.386960481	76
chr18.fa	27878876	27878926	MIR302F	0	0
chr18.fa	28570052	28622781	DSC3	0.484367775	152
chr18.fa	28645942	28682388	DSC2	4.189180798	988
chr18.fa	28709214	28742819	DSC1	0	0
chr18.fa	28898052	28937393	DSG1	0	0
chr18.fa	28956740	28993880	DSG4	0	0
chr18.fa	29027732	29058665	DSG3	0	0
chr18.fa	29078027	29128814	DSG2	13.15532202	3344
chr18.fa	29171730	29178986	TTR	0	0
chr18.fa	29202209	29264686	B4GALT6	1.755555196	380
chr18.fa	29339659	29340843	MCART2	0	0
chr18.fa	29409136	29523091	TRAPPC8	14.04266244	3949
chr18.fa	29598445	29653154	RNF125	1.435312039	380
chr18.fa	29671818	29711524	RNF138	1.845178802	304
chr18.fa	29769987	29800366	MEP1B	0	0
chr18.fa	29847477	30050447	FAM59A	18.89701496	2584
chr18.fa	30091626	30094597	WBP11P1	0.568653995	76
chr18.fa	30252634	30352974	KLHL14	1.983283663	380
chr18.fa	30517366	31020685	C18orf34	0	0
chr18.fa	31158541	31327399	ASXL3	0.663614987	228
chr18.fa	31431064	31803515	NOL4	0.365610938	76
chr18.fa	32073254	32471808	DTNA	0.315128162	152
chr18.fa	32556892	32722378	MAPRE2	4.542337836	760
chr18.fa	32820994	32838397	ZNF397	9.594173638	1368
chr18.fa	32831023	32870196	ZSCAN30	4.04151312	760

chr18.fa	32870236	32890730	ZNF271	10.86402671	2508
chr18.fa	32912178	32924426	ZNF24	20.35389893	5776
chr18.fa	32946661	32957301	ZNF396	1.212476175	152
chr18.fa	33048291	33077955	INO80C	20.1951117	988
chr18.fa	33234533	33291798	GALNT1	12.77103023	2204
chr18.fa	33484781	33484889	MIR187	0	0
chr18.fa	33552588	33559241	C18orf21	13.0138813	608
chr18.fa	33569792	33647373	RPRD1A	22.97388826	4408
chr18.fa	33688494	33709357	SLC39A6	11.94462498	1976
chr18.fa	33709887	33754607	ELP2	36.59556438	4104
chr18.fa	33767480	33848685	MOCOS	3.099019718	380
chr18.fa	33877702	34360018	FHOD3	5.130117463	1140
chr18.fa	34376034	34409079	C18orf10	28.08443531	2508
chr18.fa	34414283	34805288	KIAA1328	1.416186406	304
chr18.fa	34823008	35146000	CELF4	0	0
chr18.fa	35237098	35237178	MIR4318	0	0
chr18.fa	36786888	37331959	LOC647946	0	0
chr18.fa	39060236	39100561	KC6	0	0
chr18.fa	39535199	39661446	PIK3C3	11.02170199	1520
chr18.fa	40323192	40695657	RIT2	0	0
chr18.fa	40847857	40857615	SYT4	0	0
chr18.fa	42260138	42648475	SETBP1	5.391649375	2660
chr18.fa	42550047	42550131	MIR4319	0	0
chr18.fa	43194766	43263060	SLC14A2	0	0
chr18.fa	43304092	43332485	SLC14A1	6.004559195	1198
chr18.fa	43405545	43422521	SIGLEC15	2.246149921	152
chr18.fa	43427574	43547305	KIAA1632	6.788265366	3876
chr18.fa	43563502	43652250	PSTPIP2	1.676828753	228
chr18.fa	43664110	43684199	ATP5A1	213.7698686	18696
chr18.fa	43684298	43708299	HAUS1	2.901536438	152
chr18.fa	43753988	43846955	C18orf25	8.350340321	2052
chr18.fa	43914187	44040783	RNF165	0	0
chr18.fa	44056935	44236996	LOXHD1	0	0
chr18.fa	44259081	44337039	ST8SIA5	1.288756316	152
chr18.fa	44392060	44497466	PIAS2	22.64652859	2502
chr18.fa	44526787	44628614	KATNAL2	0	0
chr18.fa	44542731	44544371	TCEB3CL	0	0
chr18.fa	44550299	44556449	TCEB3C	0	0
chr18.fa	44558943	44561988	TCEB3B	0	0
chr18.fa	44633781	44676871	HDHD2	30.84986841	3031
chr18.fa	44681413	44702745	IER3IP1	10.27113209	684
chr18.fa	45359466	45457512	SMAD2	10.24244364	4940
chr18.fa	45553639	45663680	ZBTB7C	10.94097403	2052

chr18.fa	46065427	46389586	KIAA0427	22.95009242	6004
chr18.fa	46446223	46477081	SMAD7	9.009952268	1292
chr18.fa	46570172	46987079	DYM	14.79212038	1748
chr18.fa	47013413	47013644	C18orf32	17.25443443	180
chr18.fa	47013743	47013792	MIR1539	0	0
chr18.fa	47014851	47018827	RPL17	32.43195856	560
chr18.fa	47015605	47015694	SNORD58C	0	0
chr18.fa	47017653	47017717	SNORD58A	0	0
chr18.fa	47018034	47018099	SNORD58B	25.60877779	76
chr18.fa	47088427	47119278	LIPG	0.408087634	76
chr18.fa	47309874	47340251	ACAA2	33.18608672	2886
chr18.fa	47340393	47340813	SCARNA17	4.0146038	76
chr18.fa	47349156	47721451	MYO5B	36.21794433	15504
chr18.fa	47652869	47652933	MIR4320	0	0
chr18.fa	47753563	47792865	CCDC11	2.779888516	228
chr18.fa	47795216	47808144	MBD1	18.58944809	2725
chr18.fa	47808713	47814692	CXXC1	18.2127176	2429
chr18.fa	47901392	47920538	SKA1	0	0
chr18.fa	48086484	48258196	MAPK4	5.353175718	1140
chr18.fa	48321490	48351754	MRO	1.584314063	380
chr18.fa	48405432	48476162	ME2	6.216497896	1292
chr18.fa	48494387	48514490	ELAC1	7.569080452	760
chr18.fa	48556583	48611411	SMAD4	15.12437266	5965
chr18.fa	48700920	48724051	MEX3C	7.752997876	1444
chr18.fa	49866542	51062273	DCC	0	0
chr18.fa	51680575	51751158	MBD2	28.63752193	3876
chr18.fa	51748654	51748782	SNORA37	0	0
chr18.fa	51795849	51824604	POLI	6.41687226	1748
chr18.fa	51851062	51880943	STARD6	0	0
chr18.fa	51885171	51908404	C18orf54	0	0
chr18.fa	52258390	52266724	C18orf26	0	0
chr18.fa	52495840	52557211	RAB27B	87.35721825	5244
chr18.fa	52568740	52626739	CCDC68	1.206694007	228
chr18.fa	52889562	53255860	TCF4	2.637113442	988
chr18.fa	54270053	54305920	TXNL1	39.03119151	2508
chr18.fa	54318616	54697036	WDR7	9.748290658	3192
chr18.fa	54814293	54817639	BOD1P	0	0
chr18.fa	55019721	55036161	ST8SIA3	0	0
chr18.fa	55102917	55158530	ONECUT2	0.104746199	76
chr18.fa	55212073	55253969	FECH	9.289275466	3040
chr18.fa	55267894	55289177	NARS	43.04223705	5545
chr18.fa	55313659	55470327	ATP8B1	30.74601177	8208
chr18.fa	55711619	56068772	NEDD4L	81.06221631	35047

chr18.fa	56118306	56118390	MIR122	0	0
chr18.fa	56148482	56296189	ALPK2	0.926036463	304
chr18.fa	56338618	56417370	MALT1	12.09896439	2736
chr18.fa	56530061	56653709	ZNF532	39.4826454	11476
chr18.fa	56702971	56720446	LOC390858	8.836932007	211
chr18.fa	56807125	56826063	SEC11C	90.77892743	3188
chr18.fa	56887400	56898002	GRP	0	0
chr18.fa	56934267	56940625	RAX	0	0
chr18.fa	56962636	56985881	CPLX4	0	0
chr18.fa	56995056	57026508	LMAN1	7.34246394	1596
chr18.fa	57098171	57364644	CCBE1	0	0
chr18.fa	57567192	57571538	PMAIP1	0	0
chr18.fa	58038564	58040001	MC4R	0	0
chr18.fa	59157775	59222365	CDH20	0.592005058	76
chr18.fa	59482304	59560304	RNF152	17.7912865	1444
chr18.fa	59711458	59854289	PIGN	0.693415391	152
chr18.fa	59854524	59974355	KIAA1468	20.70994705	5079
chr18.fa	59992548	60053504	TNFRSF11A	1.081265437	152
chr18.fa	60190658	60245818	ZCCHC2	16.1607151	4028
chr18.fa	60382672	60647666	PHLPP1	13.46644715	3876
chr18.fa	60790579	60986613	BCL2	3.063659536	912
chr18.fa	60994971	61034506	KDSR	1.625901195	380
chr18.fa	61056425	61089752	VPS4B	24.9778543	3794
chr18.fa	61144144	61172318	SERPINB5	1.925684373	228
chr18.fa	61223393	61234244	SERPINB12	0	0
chr18.fa	61254534	61266433	SERPINB13	0.531514684	76
chr18.fa	61304493	61311502	SERPINB4	0	0
chr18.fa	61322431	61329197	SERPINB3	0.951166655	76
chr18.fa	61370194	61391122	SERPINB11	26.13406553	2033
chr18.fa	61420277	61472603	SERPINB7	0	0
chr18.fa	61554939	61571124	SERPINB2	0	0
chr18.fa	61582745	61602476	SERPINB10	0	0
chr18.fa	61616588	61627645	HMSD	0	0
chr18.fa	61637263	61656608	SERPINB8	3.139050113	532
chr18.fa	61747243	61816260	C18orf20	0	0
chr18.fa	61771325	62090827	LOC284294	0	0
chr18.fa	61880318	61927290	LOC400654	0	0
chr18.fa	63417488	63548174	CDH7	0	0
chr18.fa	64171321	64271216	CDH19	0	0
chr18.fa	65173819	65183782	DSEL	2.415611925	988
chr18.fa	65183968	65566856	LOC643542	0	0
chr18.fa	66340925	66382353	TMX3	0	0
chr18.fa	66382491	66722426	CCDC102B	0	0

chr18.fa	67068284	67516322	DOK6	0.189921983	76
chr18.fa	67530193	67624232	CD226	0	0
chr18.fa	67671043	67872962	RTTN	1.429752262	456
chr18.fa	67956137	67997434	SOCS6	7.806149345	2052
chr18.fa	70203915	70211723	CBLN2	0	0
chr18.fa	70414787	70534810	NETO1	0	0
chr18.fa	70821293	70931733	LOC400655	0	0
chr18.fa	71740588	71815100	FBXO15	0	0
chr18.fa	71815746	71826204	C18orf55	11.01814373	760
chr18.fa	71920527	71959221	CYB5A	191.7053375	7172
chr18.fa	71983110	72026422	DKFZP781G0119	0	0
chr18.fa	72102963	72124503	FAM69C	0	0
chr18.fa	72163500	72190689	CNDP2	98.88463756	23864
chr18.fa	72201692	72252261	CNDP1	0.769028359	76
chr18.fa	72259010	72265071	LOC400657	0.972960981	152
chr18.fa	72342919	72777628	ZNF407	5.236865182	2128
chr18.fa	72909278	72921281	ZADH2	9.227450745	2280
chr18.fa	72922731	73001901	TSHZ1	6.80561187	1520
chr18.fa	73121827	73139589	C18orf62	0	0
chr18.fa	74069637	74207146	ZNF516	6.07350043	2356
chr18.fa	74240612	74271784	LOC284276	0	0
chr18.fa	74536116	74682682	ZNF236	6.24207287	2280
chr18.fa	74690789	74844774	MBP	4.164272997	1292
chr18.fa	74962008	74982096	GALR1	0	0
chr18.fa	76740275	76758193	SALL3	3.894512616	836
chr18.fa	76829397	77138282	ATP9B	7.339795247	1438
chr18.fa	77155772	77289323	NFATC1	1.732426523	380
chr18.fa	77439801	77514507	CTDP1	11.71845325	1976
chr18.fa	77623668	77659816	KCNG2	0	0
chr18.fa	77662420	77711653	PQLC1	7.972497873	912
chr18.fa	77724582	77730822	HSBP1L1	29.29624431	988
chr18.fa	77732867	77748532	TXNL4A	46.30604856	2888
chr18.fa	77794346	77810652	RBFA	2.329991359	595
chr18.fa	77866915	77898228	ADNP2	10.667433	2584
chr18.fa	77905807	77936315	LOC100130522	0.904464528	152
chr18.fa	77915117	78005397	PARD6G	1.32255976	228
chr19.fa	60951	70966	WASH5P	0	0
chr19.fa	110679	111596	OR4F17	0	0
chr19.fa	197016	202209	FLJ45445	0	0
chr19.fa	281044	291435	PPAP2C	4.424470562	304
chr19.fa	305575	344791	MIER2	4.895494873	608
chr19.fa	362057	376009	THEG	0	0
chr19.fa	405443	409170	C2CD4C	0	0

chr19.fa	416583	460996	SHC2	9.407587521	1055
chr19.fa	463346	474983	ODF3L2	0	0
chr19.fa	496490	505343	MADCAM1	0	0
chr19.fa	507497	519654	C19orf20	3.034526304	152
chr19.fa	531733	542087	CDC34	11.69665892	760
chr19.fa	544027	549919	GZMM	0	0
chr19.fa	571325	583493	BSG	14.95735695	1520
chr19.fa	589893	617159	HCN2	0	0
chr19.fa	617223	633568	POLRMT	3.558257301	608
chr19.fa	639926	643604	FGF22	0	0
chr19.fa	647526	663233	RNF126	15.3652222	1140
chr19.fa	676389	683392	FSTL3	2.019311018	228
chr19.fa	685546	695461	PRSSL1	0	0
chr19.fa	708953	748330	PALM	4.650642292	601
chr19.fa	751146	764318	C19orf21	4.7427122	608
chr19.fa	797392	812327	PTBP1	52.97133165	7815
chr19.fa	812518	821952	LPPR3	0	0
chr19.fa	827831	832017	AZU1	0	0
chr19.fa	840985	848175	PRTN3	0	0
chr19.fa	852291	856246	ELANE	0	0
chr19.fa	859665	863610	CFD	1.45821832	76
chr19.fa	867962	893218	MED16	15.02896688	1976
chr19.fa	896503	913225	C19orf22	21.4892054	1748
chr19.fa	917342	921015	KISS1R	0	0
chr19.fa	926037	972803	ARID3A	1.804481234	228
chr19.fa	984328	994569	WDR18	11.98710167	836
chr19.fa	1000437	1009649	GRIN3B	0	0
chr19.fa	1009724	1021141	C19orf6	3.850034399	456
chr19.fa	1026298	1039064	CNN2	17.19060819	1900
chr19.fa	1040102	1065570	ABCA7	0.743675776	228
chr19.fa	1067174	1086577	HMHA1	2.005522771	380
chr19.fa	1086628	1095391	POLR2E	39.42393416	4992
chr19.fa	1103936	1106787	GPX4	141.4071467	7751
chr19.fa	1107633	1174282	SBNO2	12.89957228	2964
chr19.fa	1205798	1228434	STK11	20.12105547	2964
chr19.fa	1229947	1237990	C19orf26	0	0
chr19.fa	1241749	1244824	ATP5D	62.77788878	1976
chr19.fa	1248552	1259142	MIDN	23.17137154	3952
chr19.fa	1267470	1270259	C19orf23	0	0
chr19.fa	1269267	1274809	CIRBP	269.2991425	19617
chr19.fa	1275520	1279243	C19orf24	13.21914826	532
chr19.fa	1286168	1299944	EFNA2	2.63266562	76
chr19.fa	1354976	1378430	MUM1	10.50731142	1991

chr19.fa	1383883	1395588	NDUFS7	46.3173905	1637
chr19.fa	1397088	1401552	GAMT	34.40790332	2115
chr19.fa	1407584	1435682	DAZAP1	25.47756706	2604
chr19.fa	1438363	1440492	RPS15	732.0896453	17480
chr19.fa	1450148	1473199	APC2	0.167238093	76
chr19.fa	1473244	1479228	C19orf25	10.69256319	1064
chr19.fa	1481427	1490407	PCSK4	0	0
chr19.fa	1491165	1497924	REEP6	7.386052592	456
chr19.fa	1505017	1513188	ADAMTSL5	0.591560276	76
chr19.fa	1524078	1535455	PLK5P	0.876665642	76
chr19.fa	1554668	1568057	MEX3D	13.63991219	1748
chr19.fa	1576678	1592652	MBD3	18.49337515	2128
chr19.fa	1597154	1605483	UQCR11	103.815715	6232
chr19.fa	1609292	1652326	TCF3	7.957152889	1672
chr19.fa	1753662	1775444	ONECUT3	0	0
chr19.fa	1782074	1812270	ATP8B3	0	0
chr19.fa	1815245	1848452	REXO1	6.423543992	1292
chr19.fa	1816158	1816223	MIR1909	0	0
chr19.fa	1822088	1824542	LOC100288123	0	0
chr19.fa	1852398	1863564	KLF16	7.025334258	912
chr19.fa	1876975	1885518	FAM108A1	6.894568302	532
chr19.fa	1905373	1926012	SCAMP4	5.826201548	656
chr19.fa	1911889	1913446	ADAT3	2.169647389	152
chr19.fa	1941161	1981336	CSNK1G2	18.87722215	2465
chr19.fa	1952526	1954548	C19orf34	0	0
chr19.fa	1985447	2015702	BTBD2	37.44465353	4408
chr19.fa	2037470	2051243	MKNK2	50.39493098	8577
chr19.fa	2071035	2096269	MOBKL2A	15.22222473	2280
chr19.fa	2096868	2099583	IZUMO4	7.449656441	340
chr19.fa	2100993	2151556	AP3D1	49.60544264	11184
chr19.fa	2164148	2232577	DOT1L	6.136881888	2052
chr19.fa	2233155	2236328	PLEKHJ1	30.60390387	1672
chr19.fa	2234061	2234148	MIR1227	0	0
chr19.fa	2236816	2248678	SF3A2	5.997887463	445
chr19.fa	2249113	2252072	AMH	0	0
chr19.fa	2250638	2250650	MIR4321	0	0
chr19.fa	2252250	2256422	JSRP1	0	0
chr19.fa	2269520	2273487	OAZ1	345.1135965	17784
chr19.fa	2274631	2282181	C19orf35	0	0
chr19.fa	2289774	2308156	LINGO3	0	0
chr19.fa	2321520	2328614	LSM7	42.25430545	988
chr19.fa	2328629	2355100	SPPL2B	2.703608375	456
chr19.fa	2389784	2425491	TMPRSS9	0	0

chr19.fa	2425622	2427875	TIMM13	34.85869004	2812
chr19.fa	2428164	2456958	LMNB2	9.480976578	1976
chr19.fa	2476123	2478257	GADD45B	23.35506658	1444
chr19.fa	2511218	2702746	GNG7	1.927463502	368
chr19.fa	2714565	2721390	DIRAS1	1.492244156	228
chr19.fa	2732523	2740074	SLC39A3	1.284753277	228
chr19.fa	2754712	2783354	SGTA	25.55340241	2660
chr19.fa	2785506	2813599	THOP1	21.87283002	2508
chr19.fa	2819872	2836733	ZNF554	2.285290751	380
chr19.fa	2841433	2860472	ZNF555	0.791045076	304
chr19.fa	2867333	2878501	ZNF556	0	0
chr19.fa	2900896	2918474	ZNF57	1.687725916	152
chr19.fa	2933216	2944969	ZNF77	1.657035947	152
chr19.fa	2977536	2995182	TLE6	2.513908783	228
chr19.fa	2997636	3047633	TLE2	30.27476507	3971
chr19.fa	3052908	3062964	AES	295.7227612	25797
chr19.fa	3094408	3121454	GNA11	25.36837303	1824
chr19.fa	3136191	3163766	GNA15	13.47689953	1292
chr19.fa	3178736	3180330	S1PR4	0	0
chr19.fa	3185875	3209573	NCLN	1.358364725	228
chr19.fa	3224701	3297073	CELF5	0.321355112	76
chr19.fa	3359616	3463603	NFIC	40.67799746	4560
chr19.fa	3474405	3480540	C19orf77	37.2284894	1520
chr19.fa	3490819	3500938	DOHH	6.563872765	608
chr19.fa	3506295	3536755	FZR1	11.68865284	1900
chr19.fa	3538263	3557571	C19orf28	4.79830997	608
chr19.fa	3539155	3544028	C19orf71	2.518801387	76
chr19.fa	3572943	3579081	HMG20B	64.54122766	4597
chr19.fa	3585569	3593539	GIPC3	0	0
chr19.fa	3594504	3606831	TBXA2R	0	0
chr19.fa	3610627	3626813	C19orf29	5.130117463	836
chr19.fa	3630179	3700477	PIP5K1C	10.29248163	2352
chr19.fa	3728374	3750682	TJP3	20.06100988	2579
chr19.fa	3750771	3761673	APBA3	1.566522777	152
chr19.fa	3762665	3767563	MRPL54	65.11099361	1824
chr19.fa	3769089	3772219	RAX2	0	0
chr19.fa	3777967	3801810	MATK	0	0
chr19.fa	3804022	3869027	ZFR2	0	0
chr19.fa	3880618	3928080	ATCAY	0	0
chr19.fa	3933101	3942414	ITGB1BP3	0	0
chr19.fa	3958452	3969826	DAPK3	15.25580579	1444
chr19.fa	3961412	3961510	MIR637	0	0
chr19.fa	3976054	3985461	EEF2	1130.884668	160588

chr19.fa	3982505	3982570	SNORD37	0	0
chr19.fa	4007749	4038067	PIAS4	11.67642133	912
chr19.fa	4045216	4066816	ZBTB7A	19.40984879	3876
chr19.fa	4090320	4124126	MAP2K2	53.11455151	4139
chr19.fa	4153629	4173048	CREB3L3	0	0
chr19.fa	4174106	4182596	SIRT6	2.963361158	218
chr19.fa	4183351	4224811	ANKRD24	0.419874361	76
chr19.fa	4229540	4237524	EBI3	0	0
chr19.fa	4247111	4269085	CCDC94	10.88871212	684
chr19.fa	4278598	4290720	SHD	0	0
chr19.fa	4292225	4302428	TMIGD2	0	0
chr19.fa	4304591	4323843	FSD1	0	0
chr19.fa	4324040	4338847	STAP2	55.54217255	3494
chr19.fa	4343524	4360083	MPND	11.22029722	836
chr19.fa	4360364	4400415	SH3GL1	33.84925692	3484
chr19.fa	4402660	4443394	CHAF1A	21.16807268	3182
chr19.fa	4445003	4457791	UBXN6	65.30024842	5852
chr19.fa	4472255	4502191	HDGFRP2	12.61313257	1292
chr19.fa	4502223	4517716	PLIN4	6.683741557	1900
chr19.fa	4522544	4535208	PLIN5	4.846791226	532
chr19.fa	4537227	4540036	LRG1	0.949609917	76
chr19.fa	4542600	4559771	SEMA6B	0	0
chr19.fa	4639527	4655580	TNFAIP8L1	0	0
chr19.fa	4657557	4670415	C19orf10	17.68965378	836
chr19.fa	4675244	4723855	DPP9	7.522378325	1444
chr19.fa	4769117	4772568	C19orf30	0	0
chr19.fa	4770682	4770791	MIR7-3	0	0
chr19.fa	4791728	4795571	FEM1A	12.31134787	2128
chr19.fa	4815938	4831737	TICAM1	8.754202524	1064
chr19.fa	4838346	4867780	PLIN3	24.55797993	2584
chr19.fa	4890449	4902879	ARRDC5	0	0
chr19.fa	4909510	4962165	UHRF1	0	0
chr19.fa	4969124	5153608	KDM4B	19.84573531	5000
chr19.fa	5205519	5340814	PTPRS	1.61033382	532
chr19.fa	5455426	5456867	ZNRF4	0	0
chr19.fa	5558178	5568005	PLAC2	10.52643705	1748
chr19.fa	5587010	5622938	SAFB2	18.33814617	2736
chr19.fa	5623164	5668489	SAFB	32.00407812	4300
chr19.fa	5678433	5680911	C19orf70	29.52864299	1195
chr19.fa	5681035	5688533	HSD11B1L	1.960822164	152
chr19.fa	5690272	5691678	RPL36	724.7591905	19684
chr19.fa	5691845	5720176	LONP1	21.18831027	2964
chr19.fa	5720688	5778742	TMEM146	0	0

chr19.fa	5782971	5784776	PRR22	0	0
chr19.fa	5785153	5791249	DUS3L	4.803869747	456
chr19.fa	5823818	5828335	NRTN	0	0
chr19.fa	5830637	5839742	FUT6	3.771530348	532
chr19.fa	5842899	5851485	FUT3	3.262921945	380
chr19.fa	5865837	5870551	FUT5	0	0
chr19.fa	5891287	5904024	NDUFA11	26.46298194	3496
chr19.fa	5904852	5910263	VMAC	2.024203622	152
chr19.fa	5914193	5916151	CAPS	65.9365093	4560
chr19.fa	5916223	5978320	RANBP3	13.70818625	2052
chr19.fa	5993175	6110664	RFX2	0.410978718	76
chr19.fa	6135710	6193112	ACSBG2	0.62313981	76
chr19.fa	6210392	6279959	MLLT1	26.26238518	5320
chr19.fa	6306510	6333640	ACER1	0	0
chr19.fa	6361463	6368915	CLPP	29.36874381	1520
chr19.fa	6372444	6375261	ALKBH7	39.3654453	2356
chr19.fa	6375305	6375860	PSPN	0	0
chr19.fa	6379580	6393291	GTF2F1	20.3997115	2340
chr19.fa	6413119	6424822	KHSRP	42.79716208	6212
chr19.fa	6416421	6416500	MIR3940	0	0
chr19.fa	6426048	6433790	SLC25A41	0	0
chr19.fa	6440075	6459781	SLC25A23	35.65974271	5548
chr19.fa	6464260	6467217	CRB3	20.10259701	988
chr19.fa	6467231	6481798	DENND1C	11.54320907	1444
chr19.fa	6494330	6502330	TUBB4	0	0
chr19.fa	6531010	6535939	TNFSF9	0	0
chr19.fa	6585850	6591163	CD70	0	0
chr19.fa	6663148	6670599	TNFSF14	0	0
chr19.fa	6677846	6720662	C3	1.656813556	380
chr19.fa	6729925	6737633	GPR108	16.90350131	1809
chr19.fa	6739707	6751529	TRIP10	21.79677227	1971
chr19.fa	6752173	6767523	SH2D3A	12.19570451	1292
chr19.fa	6772722	6857371	VAV1	0.585333326	76
chr19.fa	6887582	6940464	EMR1	0	0
chr19.fa	6952511	6990857	EMR4P	0	0
chr19.fa	7004964	7006426	FLJ25758	0	0
chr19.fa	7030594	7033022	MBD3L5	0	0
chr19.fa	7037755	7040184	MBD3L4	0	0
chr19.fa	7049351	7051746	MBD3L2	0	0
chr19.fa	7056216	7058645	MBD3L3	0	0
chr19.fa	7069471	7087978	ZNF557	2.816583045	760
chr19.fa	7112266	7294011	INSR	5.410552617	2204
chr19.fa	7459999	7537371	ARHGEF18	14.95735695	3952

chr19.fa	7537723	7538247	LOC100128573	0	0
chr19.fa	7541761	7553905	PEX11G	1.591430578	76
chr19.fa	7562445	7573336	C19orf45	0	0
chr19.fa	7581004	7585911	ZNF358	22.50108482	1976
chr19.fa	7587496	7598895	MCOLN1	1.611223384	152
chr19.fa	7599038	7626653	PNPLA6	4.033729433	912
chr19.fa	7660788	7683196	KIAA1543	15.34676374	2888
chr19.fa	7684411	7694439	XAB2	19.73453977	2356
chr19.fa	7694671	7696506	LOC100131801	50.54815843	966
chr19.fa	7696511	7698570	PCP2	0	0
chr19.fa	7701991	7712759	STXBP2	49.50269796	4207
chr19.fa	7733972	7735340	RETN	0	0
chr19.fa	7741943	7744719	C19orf59	0	0
chr19.fa	7745707	7747748	TRAPPC5	43.74566004	1672
chr19.fa	7753643	7767032	FCER2	0	0
chr19.fa	7793843	7797057	CLEC4G	0	0
chr19.fa	7804881	7812464	CD209	0	0
chr19.fa	7828035	7834491	CLEC4M	0	0
chr19.fa	7852370	7855898	CLEC4GP1	0	0
chr19.fa	7895161	7929862	EVI5L	9.176745579	1587
chr19.fa	7933605	7939326	FLJ22184	2.619322156	532
chr19.fa	7943517	7945117	LOC388499	0	0
chr19.fa	7953390	7966908	LRRC8E	1.876980726	304
chr19.fa	7968765	7979363	MAP2K7	9.523453274	1444
chr19.fa	7981032	7983980	LOC645781	0	0
chr19.fa	7985194	7988136	SNAPC2	11.01414069	836
chr19.fa	7989381	7991051	CTXN1	2.676476663	152
chr19.fa	7991603	8008708	TIMM44	7.60577498	684
chr19.fa	8023457	8070529	ELAVL1	12.5548661	3420
chr19.fa	8117934	8127547	CCL25	0	0
chr19.fa	8130287	8212385	FBN3	0.188587637	76
chr19.fa	8274217	8327304	LASS4	6.514946727	532
chr19.fa	8367011	8373240	CD320	0	0
chr19.fa	8376234	8386280	NDUFA7	74.57373412	1794
chr19.fa	8386384	8387280	RPS28	114.513838	1967
chr19.fa	8387468	8408146	KANK3	1.212920957	152
chr19.fa	8429011	8439257	ANGPTL4	0	0
chr19.fa	8455205	8469317	RAB11B	59.17671	4332
chr19.fa	8478187	8503899	2-Mar	21.49832344	1596
chr19.fa	8509803	8553996	HNRNPM	64.74582745	7028
chr19.fa	8554940	8567538	PRAM1	0	0
chr19.fa	8575462	8579048	ZNF414	6.508942167	456
chr19.fa	8585674	8642331	MYO1F	0.404974159	76

chr19.fa	8645126	8675588	ADAMTS10	0	0
chr19.fa	8807751	8809172	ACTL9	0	0
chr19.fa	8841391	8842335	OR2Z1	0	0
chr19.fa	8920382	8933565	ZNF558	5.607813506	760
chr19.fa	8953269	8954016	MBD3L1	0	0
chr19.fa	8959520	9092018	MUC16	0	0
chr19.fa	9203921	9204862	OR1M1	0	0
chr19.fa	9212945	9213982	OR7G2	0	0
chr19.fa	9225504	9226439	OR7G1	0	0
chr19.fa	9236688	9237626	OR7G3	0	0
chr19.fa	9251056	9274091	ZNF317	10.78396592	1976
chr19.fa	9296270	9299493	OR7D2	0	0
chr19.fa	9324526	9325547	OR7D4	0	0
chr19.fa	9361720	9362739	OR7E24	0	0
chr19.fa	9405986	9415795	ZNF699	2.421394093	228
chr19.fa	9434928	9454521	ZNF559	7.82772128	988
chr19.fa	9466333	9493293	ZNF177	0.586000499	76
chr19.fa	9523271	9546234	ZNF266	4.693563771	760
chr19.fa	9577031	9609279	ZNF560	0	0
chr19.fa	9638681	9649303	ZNF426	11.73735649	1216
chr19.fa	9676292	9695209	ZNF121	14.66313355	1140
chr19.fa	9718002	9731916	ZNF561	11.1684801	2280
chr19.fa	9759338	9785776	ZNF562	14.90287114	3876
chr19.fa	9800814	9811493	ZNF812	2.52147008	228
chr19.fa	9868151	9879410	ZNF846	2.377138268	228
chr19.fa	9920943	9929731	FBXL12	2.737856602	228
chr19.fa	9938568	9940797	UBL5	299.3924365	6987
chr19.fa	9945999	9960358	PIN1	52.55301403	2356
chr19.fa	9964394	10047070	OLFM2	0.908245176	76
chr19.fa	10070237	10121147	COL5A3	0	0
chr19.fa	10123925	10132954	RDH8	0	0
chr19.fa	10152032	10184813	C3P1	0	0
chr19.fa	10196806	10203928	C19orf66	9.935543948	751
chr19.fa	10204025	10213425	ANGPTL6	0	0
chr19.fa	10218327	10218411	SNORD105	0	0
chr19.fa	10220425	10220516	SNORD105B	18.37150484	76
chr19.fa	10221761	10221975	PPAN	15.72260467	152
chr19.fa	10222197	10222416	P2RY11	15.3652222	152
chr19.fa	10226155	10230599	EIF3G	111.0854571	5050
chr19.fa	10244022	10305755	DNMT1	3.335866219	810
chr19.fa	10332109	10341948	S1PR2	0	0
chr19.fa	10341089	10341161	MIR4322	0	0
chr19.fa	10362640	10370721	MRPL4	15.34187113	912

chr19.fa	10381517	10397291	ICAM1	0.520617521	76
chr19.fa	10397650	10399198	ICAM4	0	0
chr19.fa	10400655	10407454	ICAM5	0	0
chr19.fa	10415479	10420233	ZGLP1	0	0
chr19.fa	10420891	10426691	FDX1L	17.77549674	673
chr19.fa	10426889	10444314	RAVER1	13.64569436	2204
chr19.fa	10444452	10450345	ICAM3	5.697214721	456
chr19.fa	10461204	10491248	TYK2	12.7318894	2432
chr19.fa	10501809	10514271	CDC37	53.15013408	3800
chr19.fa	10531333	10580307	PDE4A	3.66700654	912
chr19.fa	10596796	10614054	KEAP1	23.15313547	2812
chr19.fa	10623418	10628668	S1PR5	0	0
chr19.fa	10654647	10663760	ATG4D	5.410330226	380
chr19.fa	10662798	10662880	MIR1238	0	0
chr19.fa	10664095	10676702	KRI1	6.960618454	836
chr19.fa	10677138	10679655	CDKN2D	4.75094067	304
chr19.fa	10683347	10697991	AP1M2	79.15143214	6232
chr19.fa	10713121	10755235	SLC44A2	26.65446066	4256
chr19.fa	10762538	10764548	LOC147727	7.564187848	684
chr19.fa	10764937	10803095	ILF3	29.6282742	9956
chr19.fa	10812112	10824043	QTRT1	13.93702667	836
chr19.fa	10828729	10942586	DNM2	63.49109697	10863
chr19.fa	10829080	10829179	MIR638	0	0
chr19.fa	10928102	10928172	MIR199A1	0	0
chr19.fa	10943114	10946983	TMED1	8.718619951	532
chr19.fa	10959106	10980360	C19orf38	0	0
chr19.fa	10982253	11033443	CARM1	10.83800696	1444
chr19.fa	11033449	11039357	YIPF2	6.765136693	456
chr19.fa	11039424	11040916	C19orf52	7.264404671	456
chr19.fa	11071598	11172958	SMARCA4	37.82738858	9993
chr19.fa	11200038	11244505	LDLR	8.63811438	2052
chr19.fa	11257831	11266484	SPC24	0	0
chr19.fa	11274943	11308243	KANK2	4.867918379	1140
chr19.fa	11309971	11373157	DOCK6	7.674271433	2204
chr19.fa	11350295	11352619	LOC55908	0	0
chr19.fa	11406824	11437672	TSPAN16	0	0
chr19.fa	11432722	11450344	RAB3D	55.77212493	10716
chr19.fa	11453452	11456981	TMEM205	18.45156562	760
chr19.fa	11457181	11465620	CCDC159	25.43998296	1216
chr19.fa	11466062	11476374	LPPR2	6.197817045	760
chr19.fa	11485383	11487627	C19orf39	3.050760853	228
chr19.fa	11487881	11495018	EPOR	0	0
chr19.fa	11504732	11530018	RGL3	4.652421421	532

chr19.fa	11531272	11545980	CCDC151	0	0
chr19.fa	11546269	11561782	PRKCSH	50.3644634	5168
chr19.fa	11562143	11591803	ELAVL3	0	0
chr19.fa	11594242	11616738	ZNF653	2.263718817	228
chr19.fa	11616744	11639987	ECSIT	17.7815013	1444
chr19.fa	11649579	11661138	CNN1	0	0
chr19.fa	11663858	11670051	ELOF1	43.12518892	1976
chr19.fa	11685475	11689801	ACP5	6.536518662	532
chr19.fa	11708235	11729974	ZNF627	7.671825132	988
chr19.fa	11784813	11797384	ZNF833P	0	0
chr19.fa	11832080	11849760	ZNF823	11.99710927	1292
chr19.fa	11877815	11894893	ZNF441	1.520043041	304
chr19.fa	11909391	11919306	ZNF491	1.197353582	152
chr19.fa	11925107	11946016	ZNF440	22.59537864	4248
chr19.fa	11976844	11980306	ZNF439	16.53544407	1896
chr19.fa	11998670	12025365	ZNF69	6.112641261	456
chr19.fa	12035900	12061578	ZNF700	5.309364675	684
chr19.fa	12075869	12091198	ZNF763	1.933023279	228
chr19.fa	12125532	12146525	ZNF433	4.369317574	456
chr19.fa	12154620	12167127	ZNF878	0	0
chr19.fa	12175546	12188626	ZNF844	8.945903636	1140
chr19.fa	12203078	12225494	ZNF788	0.44834042	76
chr19.fa	12242803	12251140	ZNF20	0.72188145	76
chr19.fa	12255709	12267529	ZNF625	2.019311018	152
chr19.fa	12273872	12300064	ZNF136	7.280416828	988
chr19.fa	12382625	12405714	ZNF44	8.469097158	1064
chr19.fa	12428304	12444534	ZNF563	5.42144978	676
chr19.fa	12460185	12476475	ZNF442	2.393150426	304
chr19.fa	12500828	12512088	ZNF799	1.959265426	228
chr19.fa	12540520	12551926	ZNF443	1.903445265	228
chr19.fa	12571998	12595632	ZNF709	2.76009571	608
chr19.fa	12636184	12662356	ZNF564	13.75978098	1824
chr19.fa	12686920	12721623	ZNF490	4.679108351	1283
chr19.fa	12721732	12740676	ZNF791	5.41944826	608
chr19.fa	12757322	12777591	MAN2B1	13.10617359	1900
chr19.fa	12777618	12786533	WDR83	8.747753183	509
chr19.fa	12778881	12780465	C19orf56	101.4895043	3482
chr19.fa	12786647	12792677	DHPS	19.44943441	1004
chr19.fa	12799730	12807455	FBXW9	2.919105333	228
chr19.fa	12810008	12834810	TNPO2	16.52143343	4028
chr19.fa	12817263	12817332	SNORD41	0	0
chr19.fa	12841454	12845529	C19orf43	176.4466406	7220
chr19.fa	12848306	12859137	ASNA1	38.29307551	2204

chr19.fa	12863407	12869271	BEST2	0	0
chr19.fa	12873817	12886434	HOOK2	35.56544889	4174
chr19.fa	12902310	12904125	JUNB	59.56544961	4864
chr19.fa	12907634	12912694	PRDX2	131.5007359	7752
chr19.fa	12917428	12924462	RNASEH2A	2.960025292	152
chr19.fa	12936296	12946230	RTBDN	0	0
chr19.fa	12949259	12985766	MAST1	0	0
chr19.fa	12986025	12992335	DNASE2	2.525250728	228
chr19.fa	12995237	12998017	KLF1	0	0
chr19.fa	13001974	13010783	GCDH	20.3472272	1667
chr19.fa	13009894	13030086	SYCE2	0	0
chr19.fa	13033284	13044558	FARSA	31.01243629	2584
chr19.fa	13049414	13055304	CALR	45.99114279	3952
chr19.fa	13056654	13064455	RAD23A	52.78852619	4256
chr19.fa	13064972	13068050	GADD45GIP1	36.39674676	1216
chr19.fa	13080432	13085567	DAND5	0	0
chr19.fa	13106584	13209610	NFIX	80.93189513	20292
chr19.fa	13209844	13213974	LYL1	0	0
chr19.fa	13215714	13227563	TRMT1	5.5181899	532
chr19.fa	13229109	13251959	NACC1	27.47330462	5548
chr19.fa	13255224	13260987	STX10	11.17893248	377
chr19.fa	13261282	13265718	IER2	26.43473827	2432
chr19.fa	13317256	13617274	CACNA1A	0	0
chr19.fa	13858753	13874106	CCDC130	10.35653027	760
chr19.fa	13875337	13885096	MRI1	2.587520231	380
chr19.fa	13885257	13889586	C19orf53	76.08755021	3110
chr19.fa	13906274	13943044	ZSWIM4	3.505773006	684
chr19.fa	13945330	13947100	LOC284454	0	0
chr19.fa	13947104	13947173	MIR24-2	0	0
chr19.fa	13947254	13947331	MIR27A	0	0
chr19.fa	13947401	13947473	MIR23A	0	0
chr19.fa	13985513	13985622	MIR181C	0	0
chr19.fa	13985689	13985825	MIR181D	0	0
chr19.fa	13987950	13991571	NANOS3	0	0
chr19.fa	13993168	14016909	C19orf57	0	0
chr19.fa	14016956	14041693	CC2D1A	12.90357532	2112
chr19.fa	14042000	14064204	PODNL1	0	0
chr19.fa	14063319	14072256	DCAF15	5.988102255	608
chr19.fa	14072342	14117134	RFX1	8.109045997	1596
chr19.fa	14139017	14141783	RLN3	0	0
chr19.fa	14142262	14164026	IL27RA	0	0
chr19.fa	14164179	14169971	PALM3	64.10956657	6515
chr19.fa	14183821	14185874	LOC113230	20.80201696	1216

chr19.fa	14199229	14201232	SAMD1	9.108026735	532
chr19.fa	14202507	14228559	PRKACA	23.61259545	2888
chr19.fa	14230321	14247440	ASF1B	0	0
chr19.fa	14258549	14316997	LPHN1	0.430326742	152
chr19.fa	14492213	14519535	CD97	1.045682864	152
chr19.fa	14519610	14530195	DDX39	18.65772216	1292
chr19.fa	14544166	14582679	PKN1	21.55836903	3040
chr19.fa	14583278	14586174	PTGER1	1.189347503	76
chr19.fa	14588571	14606944	GIPC1	56.90631945	4936
chr19.fa	14599758	14600690	LOC100130932	0	0
chr19.fa	14625582	14629201	DNAJB1	52.22654392	5244
chr19.fa	14640355	14640381	MIR639	0	0
chr19.fa	14640453	14676792	TECR	13.76711989	676
chr19.fa	14676890	14682889	NDUFB7	86.67558959	2128
chr19.fa	14693896	14721951	CLEC17A	0	0
chr19.fa	14730051	14785730	EMR3	0	0
chr19.fa	14800870	14831772	ZNF333	2.243481228	380
chr19.fa	14843205	14889353	EMR2	0	0
chr19.fa	14909986	14910948	OR7C1	0	0
chr19.fa	14937139	14939276	OR7A5	0	0
chr19.fa	14951760	14952689	OR7A10	0	0
chr19.fa	14991238	14992167	OR7A17	0	0
chr19.fa	15052301	15053260	OR7C2	0	0
chr19.fa	15060991	15083730	SLC1A6	0	0
chr19.fa	15121539	15134083	CCDC105	0	0
chr19.fa	15160291	15169103	CASP14	0	0
chr19.fa	15197877	15198944	OR1I1	0	0
chr19.fa	15218214	15225784	SYDE1	0.520172739	76
chr19.fa	15225790	15236577	ILVBL	3.685464999	380
chr19.fa	15270444	15311792	NOTCH3	3.141274023	1140
chr19.fa	15337730	15343858	EPHX3	0	0
chr19.fa	15348301	15391262	BRD4	19.4372029	6514
chr19.fa	15464335	15490603	AKAP8	13.51604036	2146
chr19.fa	15490859	15529833	AKAP8L	17.5893554	1672
chr19.fa	15532318	15560762	WIZ	5.677199523	1064
chr19.fa	15560359	15560419	MIR1470	0	0
chr19.fa	15562438	15575382	RASAL3	0.513723398	76
chr19.fa	15579457	15590315	PGLYRP2	0	0
chr19.fa	15619336	15663128	CYP4F22	1.29565044	152
chr19.fa	15726029	15740447	CYP4F8	0	0
chr19.fa	15751707	15771570	CYP4F3	0	0
chr19.fa	15783886	15807984	CYP4F12	0.979855104	76
chr19.fa	15838834	15839862	OR10H2	0	0

chr19.fa	15852203	15853153	OR10H3	0	0
chr19.fa	15870144	15890798	CYP4F24P	0	0
chr19.fa	15904859	15905806	OR10H5	0	0
chr19.fa	15917817	15918936	OR10H1	0	0
chr19.fa	15939757	15946230	UCA1	0	0
chr19.fa	15988834	16008884	CYP4F2	0	0
chr19.fa	16023180	16045676	CYP4F11	0.568431604	76
chr19.fa	16059818	16060768	OR10H4	0	0
chr19.fa	16126444	16138272	LOC126536	0	0
chr19.fa	16144523	16152918	FLJ25328	0	0
chr19.fa	16178317	16213813	TPM4	102.3755104	13824
chr19.fa	16222490	16244445	RAB8A	42.55764688	4166
chr19.fa	16244838	16269377	HSH2D	4.291703087	456
chr19.fa	16272179	16284286	CIB3	0	0
chr19.fa	16296235	16302857	FAM32A	61.79069477	4026
chr19.fa	16308665	16346156	AP1M1	11.31014322	1216
chr19.fa	16435651	16438339	KLF2	0	0
chr19.fa	16472408	16582762	EPS15L1	21.26103215	2652
chr19.fa	16589875	16607003	CALR3	0	0
chr19.fa	16607205	16632163	C19orf44	1.691284173	228
chr19.fa	16628700	16653263	CHERP	15.77575613	2658
chr19.fa	16660648	16683193	SLC35E1	7.632906692	1748
chr19.fa	16685718	16739015	MED26	4.777405208	684
chr19.fa	16756959	16770968	C19orf42	30.46735575	1881
chr19.fa	16771938	16799816	TMEM38A	2.080246174	152
chr19.fa	16830787	16928774	NWD1	15.25469383	5244
chr19.fa	16940218	16991164	SIN3B	9.227005963	2128
chr19.fa	16999826	17002830	F2RL3	0	0
chr19.fa	17003762	17137625	CPAMD8	1.667488328	449
chr19.fa	17160571	17186343	HAUS8	2.321762889	152
chr19.fa	17186591	17324104	MYO9B	12.8043889	4389
chr19.fa	17326155	17330638	USE1	19.69295264	758
chr19.fa	17337055	17340028	OCEL1	29.06784867	1426
chr19.fa	17342694	17356151	NR2F6	50.59263665	4104
chr19.fa	17360849	17375544	USHBP1	0	0
chr19.fa	17378232	17390162	C19orf62	40.04885309	2613
chr19.fa	17392454	17398454	ANKLE1	0	0
chr19.fa	17402940	17414282	ABHD8	3.234678277	304
chr19.fa	17416477	17417652	MRPL34	34.92095954	1520
chr19.fa	17420337	17434031	DDA1	8.824478106	1588
chr19.fa	17434107	17445638	ANO8	0.829073951	152
chr19.fa	17445791	17453539	GTPBP3	4.931299837	608
chr19.fa	17462264	17488137	PLVAP	0	0

chr19.fa	17513755	17516384	BST2	3.599844433	152
chr19.fa	17530912	17536140	FAM125A	5.611149372	301
chr19.fa	17546318	17559376	TMEM221	0	0
chr19.fa	17566234	17571725	NXNL1	0	0
chr19.fa	17581300	17616977	SLC27A1	3.812005525	608
chr19.fa	17622432	17632097	PGLS	50.67247505	2356
chr19.fa	17634110	17664648	FAM129C	0.61491134	76
chr19.fa	17666511	17693965	GLT25D1	0.940714274	152
chr19.fa	17712137	17799008	UNC13A	0	0
chr19.fa	17830303	17845324	MAP1S	5.159250695	760
chr19.fa	17858527	17899377	FCHO1	0	0
chr19.fa	17905919	17924385	B3GNT3	3.728386478	456
chr19.fa	17927322	17932320	INSL3	0	0
chr19.fa	17935593	17958841	JAK3	0.311125123	76
chr19.fa	17970727	17970730	RPL18AP3	0	0
chr19.fa	17973397	17973529	SNORA68	0	0
chr19.fa	17974119	17974124	RPL18A	0	0
chr19.fa	17982782	18005983	SLC5A5	0	0
chr19.fa	18043824	18054794	CCDC124	24.970293	1216
chr19.fa	18062111	18109930	KCNN1	0	0
chr19.fa	18111944	18124911	ARRDC2	4.021497923	532
chr19.fa	18170371	18197697	IL12RB1	0	0
chr19.fa	18208603	18262499	MAST3	3.441724374	912
chr19.fa	18264016	18281343	PIK3R2	19.74299063	3496
chr19.fa	18284579	18288927	IFI30	2.823032386	131
chr19.fa	18304040	18307550	MPV17L2	6.222947237	380
chr19.fa	18307611	18314874	RAB3A	5.630052614	380
chr19.fa	18318771	18359010	PDE4C	0	0
chr19.fa	18360760	18366229	LOC729966	0	0
chr19.fa	18367906	18385319	KIAA1683	0	0
chr19.fa	18390563	18392432	JUND	58.74927434	4940
chr19.fa	18392887	18392971	MIR3188	0	0
chr19.fa	18417717	18434001	LSM4	69.00328231	3562
chr19.fa	18451408	18480763	PGPEP1	21.47319325	6840
chr19.fa	18496968	18499986	GDF15	14.08469435	760
chr19.fa	18497372	18497444	MIR3189	0	0
chr19.fa	18501954	18508415	LRRC25	0.70342299	76
chr19.fa	18530221	18545197	SSBP4	3.270038459	226
chr19.fa	18545373	18549111	ISYNA1	2.258603822	228
chr19.fa	18553473	18632937	ELL	7.134973061	1292
chr19.fa	18642568	18654383	FKBP8	109.1050645	8664
chr19.fa	18668572	18680197	C19orf50	43.37471171	3105
chr19.fa	18682614	18688270	UBA52	192.406759	24320

chr19.fa	18699495	18703147	C19orf60	23.0477221	912
chr19.fa	18704035	18717660	CRLF1	0	0
chr19.fa	18723682	18731849	TMEM59L	0	0
chr19.fa	18747838	18781302	KLHL26	5.955188375	836
chr19.fa	18794425	18893143	CRTC1	3.867603295	1216
chr19.fa	18893583	18902114	COMP	0	0
chr19.fa	18942744	18979039	UPF1	36.36472244	8740
chr19.fa	18988718	18989738	LASS1	0	0
chr19.fa	19010323	19030199	COPE	84.66161596	4317
chr19.fa	19030484	19039442	DDX49	11.04683218	912
chr19.fa	19040010	19052041	HOMER3	2.468541002	228
chr19.fa	19101697	19144380	SUGP2	13.7766827	3116
chr19.fa	19144387	19168987	ARMC6	8.831372229	988
chr19.fa	19174803	19223704	SLC25A42	11.18338031	1575
chr19.fa	19230430	19249267	TMEM161A	0	0
chr19.fa	19281014	19281098	MEF2B	0	0
chr19.fa	19287713	19293381	LOC729991	8.08702928	456
chr19.fa	19303769	19310043	RFXANK	17.66741467	684
chr19.fa	19312224	19314238	NR2C2AP	7.715636175	374
chr19.fa	19322782	19363061	NCAN	0	0
chr19.fa	19366452	19373596	HAPLN4	0	0
chr19.fa	19375174	19384074	TM6SF2	1.139754292	76
chr19.fa	19387322	19431307	SUGP1	13.03256215	1216
chr19.fa	19431630	19469563	MAU2	10.02672429	2188
chr19.fa	19496642	19619741	GATAD2A	18.47513908	4712
chr19.fa	19625028	19626469	TSSK6	3.516225386	228
chr19.fa	19627019	19639013	NDUFA13	202.6959047	4940
chr19.fa	19639670	19648393	YJEFN3	0	0
chr19.fa	19649074	19657468	CILP2	0	0
chr19.fa	19672522	19729439	PBX4	0	0
chr19.fa	19734466	19739039	LPAR2	0	0
chr19.fa	19740285	19754455	GMIP	10.06675469	1592
chr19.fa	19756010	19774503	ATP13A1	2.62443715	456
chr19.fa	19779663	19791138	ZNF101	4.662651411	304
chr19.fa	19821281	19843921	ZNF14	6.845197482	912
chr19.fa	19867181	19887222	LOC284440	2.138512638	228
chr19.fa	19903520	19932560	ZNF506	3.050760853	456
chr19.fa	19976714	20004293	ZNF253	6.531403667	684
chr19.fa	20011722	20046382	ZNF93	1.209807482	152
chr19.fa	20115227	20150277	ZNF682	3.082340387	456
chr19.fa	20188803	20231977	ZNF90	1.351025819	228
chr19.fa	20278083	20308984	ZNF486	29.98343275	2052
chr19.fa	20578626	20607771	ZNF826P	0	0

chr19.fa	20720798	20748626	ZNF737	6.848533349	2584
chr19.fa	20802745	20844402	ZNF626	12.36583368	3800
chr19.fa	21106059	21133503	ZNF85	0.481699082	76
chr19.fa	21203426	21242852	ZNF430	4.744046546	836
chr19.fa	21264953	21307883	ZNF714	1.730425004	684
chr19.fa	21324840	21368805	ZNF431	3.515780604	608
chr19.fa	21473963	21512212	ZNF708	2.111158535	380
chr19.fa	21541735	21571384	ZNF738	0.288441232	152
chr19.fa	21579921	21610296	ZNF493	1.882762894	532
chr19.fa	21666517	21686040	LOC400680	0	0
chr19.fa	21688437	21721079	ZNF429	5.727015126	608
chr19.fa	21906843	21950430	ZNF100	3.781093164	760
chr19.fa	21933547	21936240	LOC641367	0	0
chr19.fa	21987752	22018970	ZNF43	2.267721856	532
chr19.fa	22148897	22193745	ZNF208	1.487796334	608
chr19.fa	22235266	22273903	ZNF257	0.480142345	76
chr19.fa	22361903	22379753	ZNF676	3.456402186	456
chr19.fa	22573899	22605148	ZNF98	0	0
chr19.fa	22779059	22786352	LOC440518	0	0
chr19.fa	22817126	22850472	ZNF492	0	0
chr19.fa	22939007	22952784	ZNF99	0	0
chr19.fa	23540498	23578269	ZNF91	33.51522552	8132
chr19.fa	23835708	23870017	ZNF675	6.886117441	684
chr19.fa	23921997	23941693	ZNF681	0.780370304	228
chr19.fa	23945816	24010919	RPSAP58	0	0
chr19.fa	24097684	24106517	ZNF726	5.434570854	152
chr19.fa	24269976	24312654	ZNF254	6.360384925	1140
chr19.fa	24344995	24346249	LOC100101266	0	0
chr19.fa	28281401	28284848	LOC148189	5.768602258	532
chr19.fa	29456038	29460055	LOC148145	0	0
chr19.fa	29698167	29704136	UQCRFS1	30.3788441	1672
chr19.fa	30017491	30055226	VSTM2B	0	0
chr19.fa	30097170	30108162	POP4	9.019070302	1052
chr19.fa	30156327	30166383	PLEKHF1	14.57884733	1140
chr19.fa	30189793	30206452	C19orf12	9.09268175	1976
chr19.fa	30302901	30315219	CCNE1	1.702181336	152
chr19.fa	30433425	30506613	C19orf2	40.1184615	4104
chr19.fa	30863328	31048965	ZNF536	0	0
chr19.fa	31640783	31641311	DKFZp566F0947	0	0
chr19.fa	31765851	31840190	TSHZ3	2.064011626	456
chr19.fa	32836514	32878573	ZNF507	17.300247	6004
chr19.fa	32896655	32976799	DPY19L3	2.730962478	760
chr19.fa	33072094	33078358	PDCD5	27.65166227	751

chr19.fa	33087907	33166102	ANKRD27	22.65853771	4537
chr19.fa	33166313	33169206	RGS9BP	0.583998979	76
chr19.fa	33182867	33204702	NUDT19	12.5324046	1672
chr19.fa	33210679	33281714	TDRD12	1.045238082	76
chr19.fa	33321419	33360683	SLC7A9	0.952723392	76
chr19.fa	33369904	33462869	CCDC123	14.12895018	1655
chr19.fa	33463148	33467960	C19orf40	4.455605314	228
chr19.fa	33469498	33555824	RHPN2	23.93328339	3800
chr19.fa	33571786	33621318	GPATCH1	18.60101243	2812
chr19.fa	33622998	33666703	WDR88	0	0
chr19.fa	33685599	33699569	LRP3	8.707278006	1368
chr19.fa	33699774	33716756	SLC7A10	0	0
chr19.fa	33790840	33793430	CEBPA	27.39769165	3192
chr19.fa	33793763	33795963	LOC80054	6.911247634	684
chr19.fa	33864609	33873592	CEBPG	15.38167914	2584
chr19.fa	33877855	34012799	PEPD	60.08984778	5431
chr19.fa	34112861	34264414	CHST8	0	0
chr19.fa	34287751	34306666	KCTD15	3.769751219	836
chr19.fa	34663352	34720420	LSM14A	40.66354204	6897
chr19.fa	34745456	34846471	KIAA0355	10.87492388	3268
chr19.fa	34855645	34893318	GPI	40.45694073	8139
chr19.fa	34895303	34917072	PDCD2L	2.921551635	152
chr19.fa	34919268	34960798	UBA2	35.51585568	4256
chr19.fa	34972880	34992085	WTIP	3.834244633	380
chr19.fa	35067638	35068596	LOC643719	0	0
chr19.fa	35084346	35085490	SCGBL	0	0
chr19.fa	35168567	35177302	ZNF302	5.799292227	684
chr19.fa	35225480	35233774	ZNF181	1.14553646	152
chr19.fa	35248979	35264134	ZNF599	0.542634238	76
chr19.fa	35307058	35323773	LOC400685	0	0
chr19.fa	35417807	35436076	ZNF30	3.802442708	456
chr19.fa	35447258	35454953	ZNF792	9.128931496	1596
chr19.fa	35491246	35517373	GRAMD1A	10.2435556	1216
chr19.fa	35521592	35531353	SCN1B	0.853981752	76
chr19.fa	35531410	35557477	HPN	10.47461993	1064
chr19.fa	35549963	35597175	LOC100128675	0	0
chr19.fa	35606732	35615228	FXYD3	58.03361984	5420
chr19.fa	35615417	35626178	LGI4	0	0
chr19.fa	35629732	35633954	FXYD1	0	0
chr19.fa	35634154	35645205	FXYD7	0	0
chr19.fa	35645625	35660788	FXYD5	0	0
chr19.fa	35715704	35719628	FAM187B	0	0
chr19.fa	35739559	35758867	LSR	0.741896647	76

chr19.fa	35759896	35770718	USF2	66.74334415	5129
chr19.fa	35773410	35776045	HAMP	0	0
chr19.fa	35782989	35804709	MAG	0	0
chr19.fa	35820072	35838264	CD22	0	0
chr19.fa	35842445	35843367	FFAR1	0	0
chr19.fa	35849488	35851389	FFAR3	0	0
chr19.fa	35940617	35942669	FFAR2	0.823291783	76
chr19.fa	35978228	35981356	KRTDAP	0	0
chr19.fa	35988119	36004560	DMKN	9.576159961	1092
chr19.fa	36014269	36019253	SBSN	0	0
chr19.fa	36024314	36036221	GAPDHS	0	0
chr19.fa	36036545	36038428	TMEM147	31.777684	1216
chr19.fa	36041095	36054560	ATP4A	0	0
chr19.fa	36103646	36116251	HAUS5	2.353564813	456
chr19.fa	36119980	36128587	RBM42	29.70611108	2204
chr19.fa	36132647	36135773	ETV2	2.273281633	152
chr19.fa	36139125	36149686	COX6B1	295.0640388	7828
chr19.fa	36157715	36169367	UPK1A	269.4877302	14820
chr19.fa	36203830	36207940	ZBTB32	0	0
chr19.fa	36208921	36229779	MLL4	10.77684941	4104
chr19.fa	36230151	36233351	TMEM149	5.652736504	304
chr19.fa	36233428	36236336	U2AF1L4	6.484034366	228
chr19.fa	36236494	36237903	PSENEN	17.7912865	532
chr19.fa	36239512	36245420	LIN37	5.633833263	228
chr19.fa	36245470	36247930	HSPB6	1.15999188	76
chr19.fa	36249044	36260077	C19orf55	3.936099748	380
chr19.fa	36266417	36279724	ARHGAP33	0.783038997	152
chr19.fa	36290892	36304201	PRODH2	3.023629141	228
chr19.fa	36316274	36342895	NPHS1	0	0
chr19.fa	36347810	36358048	KIRREL2	0	0
chr19.fa	36359401	36370699	APLP1	0	0
chr19.fa	36379143	36391552	NFKBID	0	0
chr19.fa	36393382	36395173	HCST	0	0
chr19.fa	36395303	36399211	TYROBP	0	0
chr19.fa	36428022	36436097	LRFN3	1.344131695	152
chr19.fa	36486090	36487220	SDHAF1	11.95529975	608
chr19.fa	36494002	36499672	C19orf46	8.774440113	608
chr19.fa	36500022	36505141	ALKBH6	14.39982251	608
chr19.fa	36505562	36523797	CLIP3	0	0
chr19.fa	36525887	36545664	THAP8	2.778331779	228
chr19.fa	36545783	36596012	WDR62	0	0
chr19.fa	36604611	36605887	POLR2I	48.90201965	1216
chr19.fa	36606207	36616849	TBCB	49.71085601	2584

chr19.fa	36630918	36641255	CAPNS1	252.8757836	16806
chr19.fa	36641824	36643771	COX7A1	2.218128645	76
chr19.fa	36673187	36692952	ZNF565	4.559906731	358
chr19.fa	36705504	36729675	ZNF146	14.4898909	2356
chr19.fa	36802245	36803570	LOC100134317	0	0
chr19.fa	36825355	36870105	ZFP14	1.127745173	380
chr19.fa	36882861	36909550	ZFP82	2.584406756	304
chr19.fa	36912439	36913686	LOC644189	0	0
chr19.fa	36936021	36980463	ZNF566	0.639151968	152
chr19.fa	36980529	36981942	LOC728752	1.195352062	76
chr19.fa	37001589	37019248	ZNF260	4.541893053	1140
chr19.fa	37034517	37096178	ZNF529	0.862877395	304
chr19.fa	37096221	37119499	ZNF382	0.600900702	76
chr19.fa	37128283	37157739	ZNF461	0.529513165	76
chr19.fa	37180302	37212225	ZNF567	1.257621565	152
chr19.fa	37234380	37263716	ZNF850	0.656720863	228
chr19.fa	37308331	37329284	ZNF790	2.228358635	304
chr19.fa	37341267	37370470	ZNF345	2.172538473	304
chr19.fa	37379026	37407193	ZNF829	0.322022286	76
chr19.fa	37407234	37442450	ZNF568	2.90398274	380
chr19.fa	37569382	37620651	ZNF420	3.979466009	532
chr19.fa	37641001	37663615	ZNF585A	2.424285177	456
chr19.fa	37672481	37701451	ZNF585B	4.354639763	1216
chr19.fa	37717366	37734574	ZNF383	3.991030345	304
chr19.fa	37756841	37759912	LOC284412	0	0
chr19.fa	37825580	37855357	HKR1	13.8491822	1824
chr19.fa	37862059	37883966	ZNF527	2.307974642	532
chr19.fa	37902060	37958339	ZNF569	1.249615486	228
chr19.fa	37959982	37976242	ZNF570	3.760855576	380
chr19.fa	37997841	38034239	ZNF793	0.949387526	304
chr19.fa	38042308	38105000	ZNF540	0.98919553	152
chr19.fa	38055155	38085673	ZNF571	2.970477673	304
chr19.fa	38123389	38146313	ZFP30	5.131674201	1064
chr19.fa	38158650	38183216	ZNF781	0.537964026	76
chr19.fa	38187264	38210691	ZNF607	4.0968885	836
chr19.fa	38229203	38270230	ZNF573	1.877203117	228
chr19.fa	38319707	38322556	LOC728853	0	0
chr19.fa	38375472	38397317	WDR87	0	0
chr19.fa	38397868	38699008	SIPA1L3	17.57289846	6308
chr19.fa	38701649	38720317	DPF1	0	0
chr19.fa	38741877	38747172	PPP1R14A	0	0
chr19.fa	38755098	38783254	SPINT2	17.82086452	1444
chr19.fa	38794200	38806606	YIF1B	1.244055709	152

chr19.fa	38810484	38819649	KCNK6	0	0
chr19.fa	38826443	38861589	CATSPERG	0	0
chr19.fa	38865190	38874464	PSMD8	85.66170865	5928
chr19.fa	38874992	38878668	GGN	0	0
chr19.fa	38880939	38886871	SPRED3	0	0
chr19.fa	38893775	38899697	FAM98C	21.73850581	1216
chr19.fa	38899729	38916945	RASGRP4	0	0
chr19.fa	38924340	39078204	RYR1	0	0
chr19.fa	39078281	39108643	MAP4K1	0	0
chr19.fa	39109722	39127595	EIF3K	111.5244571	4408
chr19.fa	39138267	39220831	ACTN4	78.10152384	12692
chr19.fa	39221172	39235114	CAPN12	0.606015697	76
chr19.fa	39261608	39264157	LGALS7	0	0
chr19.fa	39279850	39282394	LGALS7B	0	0
chr19.fa	39292311	39303740	LGALS4	7.250838815	417
chr19.fa	39306062	39322497	ECH1	78.62036223	4465
chr19.fa	39327028	39342979	HNRNPL	39.92542604	3876
chr19.fa	39358472	39368919	RINL	1.110398669	152
chr19.fa	39369195	39390463	SIRT2	17.98876978	1676
chr19.fa	39390615	39399534	NFKBIB	10.27402317	1045
chr19.fa	39405904	39421347	SARS2	10.33718224	905
chr19.fa	39421537	39423659	MRPS12	17.98565631	833
chr19.fa	39432042	39466380	FBXO17	7.25395229	760
chr19.fa	39514663	39523198	FBXO27	5.823088073	608
chr19.fa	39574945	39602128	PAPL	0	0
chr19.fa	39616420	39670046	PAK4	48.70164528	6688
chr19.fa	39687604	39692522	NCCRP1	12.84975668	1140
chr19.fa	39693562	39694906	SYCN	0	0
chr19.fa	39734272	39735611	IL28B	0	0
chr19.fa	39759157	39760732	IL28A	0	0
chr19.fa	39786965	39789312	IL29	0	0
chr19.fa	39797457	39805976	LRFN1	0	0
chr19.fa	39818999	39826726	GMFG	0	0
chr19.fa	39833108	39875537	SAMD4B	11.66930482	2356
chr19.fa	39876270	39881679	PAF1	23.31347944	2235
chr19.fa	39881963	39891203	MED29	17.05806311	2736
chr19.fa	39897487	39900045	ZFP36	20.34011069	1596
chr19.fa	39903750	39919055	PLEKHG2	0.22216869	76
chr19.fa	39923847	39926618	RPS16	1123.112545	29796
chr19.fa	39936186	39967308	SUPT5H	44.25404605	7697
chr19.fa	39971052	39981528	TIMM50	8.776219241	1015
chr19.fa	39989557	39999121	DLL3	0	0
chr19.fa	40005753	40011326	SELV	0	0

chr19.fa	40021630	40023494	EID2B	1.812487313	152
chr19.fa	40029447	40030838	EID2	8.499342345	532
chr19.fa	40093169	40098114	LGALS13	0	0
chr19.fa	40129325	40133041	LOC100129935	0	0
chr19.fa	40146558	40151287	LGALS16	0	0
chr19.fa	40170014	40177013	LGALS17A	0	0
chr19.fa	40194946	40200088	LGALS14	0	0
chr19.fa	40221895	40228668	CLC	0	0
chr19.fa	40267234	40276775	LEUTX	0	0
chr19.fa	40315990	40324841	DYRK1B	12.00111231	1368
chr19.fa	40325093	40337054	FBL	88.49830689	4636
chr19.fa	40353963	40440533	FCGBP	0.825070912	608
chr19.fa	40477073	40487353	PSMC4	57.71315429	3724
chr19.fa	40502943	40523514	ZNF546	0.735892088	152
chr19.fa	40534167	40562115	ZNF780B	2.145851543	836
chr19.fa	40575059	40596845	ZNF780A	2.477659037	836
chr19.fa	40697651	40721482	MAP3K10	3.443281112	532
chr19.fa	40721965	40724306	TTC9B	0	0
chr19.fa	40728115	40732597	CNTD2	0	0
chr19.fa	40736224	40791265	AKT2	19.26863046	4560
chr19.fa	40788450	40788548	MIR641	0	0
chr19.fa	40826971	40854293	C19orf47	8.749087529	836
chr19.fa	40854332	40884390	PLD3	10.23465995	1122
chr19.fa	40885178	40896094	HIPK4	0	0
chr19.fa	40899671	40919271	PRX	2.435849513	532
chr19.fa	40928409	40931932	SERTAD1	5.615152412	304
chr19.fa	40946748	40950282	SERTAD3	15.09990964	1140
chr19.fa	40953691	40971725	BLVRB	39.48998431	1520
chr19.fa	40973126	41082365	SPTBN4	0	0
chr19.fa	41082757	41097305	SHKBP1	18.58121962	1976
chr19.fa	41099072	41135725	LTBP4	0.931151457	228
chr19.fa	41171812	41196556	NUMBL	0.949832308	152
chr19.fa	41197434	41222790	ADCK4	9.476306365	1064
chr19.fa	41223008	41246760	ITPKC	19.00198355	2888
chr19.fa	41246766	41255828	C19orf54	5.535536405	684
chr19.fa	41256759	41271297	SNRPA	14.52569587	1064
chr19.fa	41281300	41283394	MIA	0	0
chr19.fa	41284171	41302847	RAB4B	28.16961109	1444
chr19.fa	41305048	41314337	EGLN2	24.4841461	2432
chr19.fa	41349443	41356352	CYP2A6	0	0
chr19.fa	41381344	41388657	CYP2A7	0	0
chr19.fa	41430170	41456565	CYP2B7P1	0	0
chr19.fa	41497204	41524301	CYP2B6	0.553753792	76

chr19.fa	41594368	41602099	CYP2A13	0	0
chr19.fa	41620353	41634281	CYP2F1	0	0
chr19.fa	41699115	41713444	CYP2S1	1.28964588	152
chr19.fa	41725108	41767671	AXL	0.356715294	76
chr19.fa	41768391	41813811	HNRNPUL1	54.26787166	10488
chr19.fa	41816094	41830788	CCDC97	11.19316551	1672
chr19.fa	41836812	41859831	TGFB1	0	0
chr19.fa	41860322	41870078	B9D2	5.627606312	228
chr19.fa	41882438	41889987	TMEM91	0	0
chr19.fa	41892276	41903256	EXOSC5	5.106321618	228
chr19.fa	41903694	41930910	BCKDHA	19.05491263	1520
chr19.fa	41931264	41934635	B3GNT8	0	0
chr19.fa	41937223	41945843	ATP5SL	14.78544865	1216
chr19.fa	41949063	41950670	C19orf69	5.35984745	228
chr19.fa	42082531	42093197	CEACAM21	0	0
chr19.fa	42125344	42133442	CEACAM4	0	0
chr19.fa	42177235	42192096	CEACAM7	0	0
chr19.fa	42212530	42234437	CEACAM5	0	0
chr19.fa	42259398	42276113	CEACAM6	0	0
chr19.fa	42300534	42315591	CEACAM3	0	0
chr19.fa	42341150	42348508	LYPD4	0	0
chr19.fa	42349086	42356397	DMRTC2	0	0
chr19.fa	42363988	42375484	RPS19	660.8864704	25230
chr19.fa	42381190	42385439	CD79A	0	0
chr19.fa	42387267	42411604	ARHGEF1	5.078745124	760
chr19.fa	42460833	42463528	RABAC1	25.13263849	912
chr19.fa	42470734	42498382	ATP1A3	0	0
chr19.fa	42502473	42569957	GRIK5	0.958060778	152
chr19.fa	42580290	42585720	ZNF574	4.318612408	608
chr19.fa	42592666	42636630	POU2F2	0.375840927	76
chr19.fa	42637597	42637665	MIR4323	0	0
chr19.fa	42702752	42721813	DEDD2	17.01825511	1444
chr19.fa	42724492	42732353	ZNF526	5.12945029	912
chr19.fa	42734338	42746736	GSK3A	39.55892554	3876
chr19.fa	42751717	42759309	ERF	8.747753183	1064
chr19.fa	42788817	42799949	CIC	11.11755255	2736
chr19.fa	42801185	42806952	PAFAH1B3	4.937304396	228
chr19.fa	42806284	42814973	PRR19	2.687151435	152
chr19.fa	42817477	42829214	TMEM145	0	0
chr19.fa	42829761	42882921	MEGF8	1.387053174	684
chr19.fa	42891171	42894444	CNFN	12.8043889	304
chr19.fa	42905666	42931578	LIPE	0.888229979	152
chr19.fa	42932695	42947136	CXCL17	3.962786678	152

chr19.fa	43011458	43032661	CEACAM1	0.93582167	152
chr19.fa	43084395	43099082	CEACAM8	0	0
chr19.fa	43225794	43244668	PSG3	0	0
chr19.fa	43256839	43269831	PSG8	0	0
chr19.fa	43326015	43331030	LOC100289650	0	0
chr19.fa	43341149	43359870	PSG10P	0	0
chr19.fa	43370613	43383871	PSG1	0	0
chr19.fa	43406240	43422043	PSG6	0	0
chr19.fa	43428284	43441330	PSG7	0	0
chr19.fa	43511809	43530631	PSG11	0	0
chr19.fa	43568362	43586893	PSG2	0	0
chr19.fa	43671895	43690688	PSG5	0	0
chr19.fa	43696854	43709790	PSG4	0	0
chr19.fa	43715943	43752798	LOC284344	0	0
chr19.fa	43757435	43773682	PSG9	0	0
chr19.fa	43853208	43853700	PRG1	0	0
chr19.fa	43857825	43867480	CD177	1.429974653	152
chr19.fa	43892763	43922767	TEX101	0.971849025	76
chr19.fa	43964946	43969831	LYPD3	14.86083923	1140
chr19.fa	43979255	44008985	PHLDB3	18.33458792	2052
chr19.fa	44010871	44031396	ETHE1	31.59198745	1368
chr19.fa	44037340	44040284	ZNF575	0	0
chr19.fa	44047464	44079730	XRCC1	9.648881844	912
chr19.fa	44080952	44086256	LOC390940	0	0
chr19.fa	44088519	44100287	IRGQ	6.979076913	3040
chr19.fa	44100544	44104587	ZNF576	10.68166603	988
chr19.fa	44111376	44124014	ZNF428	25.77423676	1378
chr19.fa	44116253	44118650	SRRM5	1.542059758	152
chr19.fa	44126522	44143991	CADM4	2.33021375	228
chr19.fa	44150248	44174502	PLAUR	0	0
chr19.fa	44220214	44224169	IRGC	0	0
chr19.fa	44235301	44259142	C19orf61	12.92959508	1368
chr19.fa	44270685	44285409	KCNN4	2.263718817	228
chr19.fa	44300079	44324808	LYPD5	0	0
chr19.fa	44331473	44353050	ZNF283	2.635779096	304
chr19.fa	44376515	44384288	ZNF404	0	0
chr19.fa	44416776	44439411	ZNF45	8.079690375	1418
chr19.fa	44455397	44471752	ZNF221	0	0
chr19.fa	44488355	44502477	ZNF155	3.906299343	456
chr19.fa	44507077	44518072	ZNF230	2.454307973	456
chr19.fa	44529494	44537262	ZNF222	1.975722366	152
chr19.fa	44556164	44572142	ZNF223	6.301451289	684
chr19.fa	44576297	44591623	ZNF284	2.296410305	228

chr19.fa	44598482	44605378	ZNF224	9.185641222	228
chr19.fa	44609492	44617336	LOC100379224	0	0
chr19.fa	44617548	44637255	ZNF225	1.856743138	228
chr19.fa	44645710	44664462	ZNF234	2.939342922	608
chr19.fa	44669215	44681838	ZNF226	6.426435076	912
chr19.fa	44716691	44741421	ZNF227	4.443373804	608
chr19.fa	44764076	44779468	ZNF233	2.430067345	304
chr19.fa	44790501	44809178	ZNF235	3.179080507	456
chr19.fa	44830706	44860856	ZFP112	1.810041011	304
chr19.fa	44889808	44905777	ZNF285	3.775533387	456
chr19.fa	44930426	44952665	ZNF229	7.490798791	1672
chr19.fa	44979859	45004574	ZNF180	5.415445221	760
chr19.fa	45010211	45033548	CEACAM20	0	0
chr19.fa	45041045	45060150	CEACAM22P	1.12085105	152
chr19.fa	45147098	45169428	PVR	1.43153139	380
chr19.fa	45174724	45187627	CEACAM19	0	0
chr19.fa	45202421	45213986	CEACAM16	0	0
chr19.fa	45251978	45263301	BCL3	11.78761687	988
chr19.fa	45281126	45303903	CBLC	16.99735034	1216
chr19.fa	45312338	45324678	BCAM	8.343001415	912
chr19.fa	45349393	45392485	PVRL2	4.255008558	684
chr19.fa	45394477	45406946	TOMM40	8.895643252	684
chr19.fa	45409039	45412650	APOE	54.42932758	2888
chr19.fa	45417921	45422606	APOC1	3.666339366	76
chr19.fa	45430060	45434643	APOC1P1	0	0
chr19.fa	45445495	45448751	APOC4	0	0
chr19.fa	45449243	45452818	APOC2	4.688448776	152
chr19.fa	45458599	45496604	CLPTM1	15.41859606	1596
chr19.fa	45504712	45541452	RELB	5.221075416	532
chr19.fa	45542298	45574214	CLASRP	6.052595669	608
chr19.fa	45574758	45579688	ZNF296	3.197094185	228
chr19.fa	45582518	45594782	GEMIN7	4.075094174	304
chr19.fa	45653008	45663408	NKPD1	0	0
chr19.fa	45666186	45681485	TRAPPC6A	29.76393276	1064
chr19.fa	45682003	45685058	BLOC1S3	3.900294784	456
chr19.fa	45715879	45737469	EXOC3L2	0	0
chr19.fa	45754516	45808541	MARK4	7.092051583	1672
chr19.fa	45809671	45826233	CKM	0	0
chr19.fa	45843998	45854648	KLC3	3.048981725	228
chr19.fa	45854779	45873845	ERCC2	9.15028104	1064
chr19.fa	45882892	45908312	PPP1R13L	11.37619337	1596
chr19.fa	45909467	45914024	CD3EAP	1.033673746	76
chr19.fa	45910591	45927177	ERCC1	17.4672627	1596

chr19.fa	45971253	45978437	FOSB	1.790470596	304
chr19.fa	45988550	46000311	RTN2	0	0
chr19.fa	46001731	46005764	PPM1N	0	0
chr19.fa	46010688	46030240	VASP	19.85018313	2044
chr19.fa	46031025	46088122	OPA3	4.595266913	1976
chr19.fa	46093023	46105466	GPR4	0	0
chr19.fa	46112658	46148775	EML2	39.64143263	5524
chr19.fa	46142252	46142345	MIR330	0	0
chr19.fa	46171502	46185717	GIPR	0	0
chr19.fa	46178186	46178282	MIR642A	0	0
chr19.fa	46190712	46195443	SNRPD2	87.62408755	3420
chr19.fa	46195741	46207248	QPCTL	0	0
chr19.fa	46213887	46234151	FBXO46	2.261050124	304
chr19.fa	46268043	46272497	SIX5	8.118608814	1216
chr19.fa	46272976	46285815	DMPK	2.348005036	380
chr19.fa	46286264	46296060	DMWD	7.664041444	1140
chr19.fa	46298968	46318605	RSPH6A	0.680516709	76
chr19.fa	46318700	46366548	SYMPK	24.21460811	4560
chr19.fa	46367518	46377055	FOXA3	0	0
chr19.fa	46386866	46389376	IRF2BP1	9.423599679	1064
chr19.fa	46393281	46405862	MYPOP	5.37408048	456
chr19.fa	46416473	46418036	NANOS2	0	0
chr19.fa	46442771	46476657	NOVA2	0	0
chr19.fa	46498339	46521874	CCDC61	6.482922411	532
chr19.fa	46522190	46522307	MIR769	0	0
chr19.fa	46522412	46526556	PGLYRP1	0	0
chr19.fa	46543006	46544274	IGFL4	0	0
chr19.fa	46623328	46627931	IGFL3	0	0
chr19.fa	46651039	46664561	IGFL2	0	0
chr19.fa	46713499	46718094	DKFZp434J0226	1.034340919	76
chr19.fa	46733009	46734500	IGFL1	70.33229142	2432
chr19.fa	46800305	46846690	HIF3A	0	0
chr19.fa	46850294	46894105	PPP5C	18.85253674	1748
chr19.fa	46913586	46916919	CCDC8	0.507051665	76
chr19.fa	46969748	46974820	PNMAL1	6.402194448	1064
chr19.fa	46994448	46999169	PNMAL2	0	0
chr19.fa	47104512	47114039	CALM3	133.6414725	13575
chr19.fa	47123725	47128354	PTGIR	0	0
chr19.fa	47137333	47137939	GNG8	0	0
chr19.fa	47150869	47164395	DACT3	0	0
chr19.fa	47177573	47220384	PRKD2	14.0809137	2280
chr19.fa	47212550	47212602	MIR320E	0	0
chr19.fa	47222768	47249720	STRN4	15.90007275	2280

chr19.fa	47249303	47261832	FKRP	1.481791775	228
chr19.fa	47278140	47291842	SLC1A5	13.12418727	1892
chr19.fa	47333842	47333961	SNAR-E	0	0
chr19.fa	47341423	47354203	AP2S1	97.95192937	3920
chr19.fa	47421933	47508333	GRLF1	28.33440289	11324
chr19.fa	47524143	47549017	NPAS1	0	0
chr19.fa	47549167	47551882	TMEM160	20.64322973	608
chr19.fa	47567447	47617009	ZC3H4	17.6865403	4864
chr19.fa	47634080	47713893	SAE1	29.05183651	3420
chr19.fa	47724079	47736023	BBC3	3.227116981	304
chr19.fa	47730199	47730278	MIR3190	0	0
chr19.fa	47759731	47775210	CCDC9	10.57380635	988
chr19.fa	47777603	47778980	PRR24	4.906169645	304
chr19.fa	47813104	47825327	C5AR1	0	0
chr19.fa	47840371	47845272	GPR77	0	0
chr19.fa	47852538	47885961	DHX34	2.319538978	456
chr19.fa	47906381	47922785	MEIS3	11.91504696	1056
chr19.fa	47931279	47975307	SLC8A2	0.330695538	76
chr19.fa	47978400	47987521	KPTN	3.003836335	228
chr19.fa	47990891	48018497	NAPA	73.34569057	6075
chr19.fa	48023947	48059113	ZNF541	0.364943764	76
chr19.fa	48111453	48206534	GLTSCR1	5.595581997	1444
chr19.fa	48216601	48246391	EHD2	0.471691483	76
chr19.fa	48248793	48260323	GLTSCR2	254.7854558	17437
chr19.fa	48259110	48259219	SNORD23	0	0
chr19.fa	48281842	48287943	SEPW1	263.8114202	10439
chr19.fa	48304500	48306861	TPRX1	0	0
chr19.fa	48325099	48346586	CRX	0	0
chr19.fa	48373723	48389654	SULT2A1	0	0
chr19.fa	48442798	48442917	SNAR-C4	0	0
chr19.fa	48453553	48453671	SNAR-C3	0	0
chr19.fa	48471303	48495427	BSPH1	0	0
chr19.fa	48497908	48528410	ELSPBP1	0	0
chr19.fa	48533210	48547304	CABP5	0	0
chr19.fa	48551100	48614109	PLA2G4C	3.950555168	456
chr19.fa	48618703	48673560	LIG1	1.096388031	152
chr19.fa	48711343	48759203	CARD8	2.599529349	674
chr19.fa	48774654	48790863	ZNF114	0	0
chr19.fa	48799709	48823332	CCDC114	0	0
chr19.fa	48828629	48833810	EMP3	0	0
chr19.fa	48835616	48867186	TMEM143	1.503141319	152
chr19.fa	48867651	48879634	SYNGR4	0	0
chr19.fa	48885827	48894810	KDELR1	37.99351472	2660

chr19.fa	48898132	48948188	GRIN2D	0	0
chr19.fa	48949030	48957164	GRWD1	13.76356163	1520
chr19.fa	48958766	48969367	KCNJ14	0.487036468	76
chr19.fa	48972465	48985571	CYTH2	16.12290861	3340
chr19.fa	48988528	49016446	LMTK3	2.719398142	608
chr19.fa	49055429	49102684	SULT2B1	30.07416832	1900
chr19.fa	49103857	49116694	FAM83E	0	0
chr19.fa	49110000	49110971	SPACA4	0	0
chr19.fa	49118588	49122433	RPL18	980.8798823	28272
chr19.fa	49122548	49133662	SPHK2	13.11973945	1748
chr19.fa	49133817	49140639	DBP	3.086121035	228
chr19.fa	49141272	49149451	CA11	12.01801403	836
chr19.fa	49141402	49185502	SEC1	0	0
chr19.fa	49164664	49176264	NTN5	0	0
chr19.fa	49199228	49209191	FUT2	0	0
chr19.fa	49216255	49222976	MAMSTR	0	0
chr19.fa	49223842	49243970	RASIP1	2.047554686	304
chr19.fa	49244145	49250166	IZUMO1	0	0
chr19.fa	49251268	49258647	FUT1	0.398080036	76
chr19.fa	49259344	49261582	FGF21	0	0
chr19.fa	49298319	49314320	BCAT2	46.2426671	4560
chr19.fa	49316274	49339934	HSD17B14	13.59165332	756
chr19.fa	49340354	49371884	PLEKHA4	9.319743044	1292
chr19.fa	49375649	49379319	PPP1R15A	17.02225814	1824
chr19.fa	49384222	49401996	TULP2	0	0
chr19.fa	49403307	49426540	NUCB1	31.45721845	3648
chr19.fa	49436939	49448226	DHDH	12.50816398	608
chr19.fa	49458117	49465055	BAX	25.39083453	1434
chr19.fa	49468566	49470136	FTL	4313.72314	168948
chr19.fa	49471382	49496610	GYS1	21.95644907	3572
chr19.fa	49497156	49519182	RUVBL2	33.76230201	2259
chr19.fa	49519237	49520347	LHB	0	0
chr19.fa	49526127	49527593	CGB	0	0
chr19.fa	49534926	49535044	SNAR-G2	0	0
chr19.fa	49535130	49536495	CGB2	0	0
chr19.fa	49538826	49540191	CGB1	0	0
chr19.fa	49540277	49540404	SNAR-G1	0	0
chr19.fa	49547102	49548568	CGB5	0	0
chr19.fa	49550895	49552368	CGB8	0	0
chr19.fa	49557531	49558997	CGB7	0	0
chr19.fa	49564397	49567124	NTF4	0	0
chr19.fa	49570675	49576198	KCNA7	0	0
chr19.fa	49588465	49611870	SNRNP70	35.78139063	3094

chr19.fa	49617618	49621653	LIN7B	7.338016119	228
chr19.fa	49621718	49622397	C19orf73	2.485442725	76
chr19.fa	49622663	49654280	PPFIA3	3.94054757	836
chr19.fa	49654456	49658681	HRC	0.708982767	76
chr19.fa	49661016	49715098	TRPM4	15.2404608	2807
chr19.fa	49792892	49828474	SLC6A16	0	0
chr19.fa	49812054	49812125	MIR4324	0	0
chr19.fa	49838677	49843854	CD37	0	0
chr19.fa	49843862	49865714	TEAD2	8.507348424	824
chr19.fa	49866987	49878373	DKKL1	0	0
chr19.fa	49891475	49921256	CCDC155	0	0
chr19.fa	49925671	49926698	PTH2	0	0
chr19.fa	49929688	49932075	LOC645971	0	0
chr19.fa	49932655	49944808	SLC17A7	0	0
chr19.fa	49949550	49955115	PIH1D1	22.86380468	1281
chr19.fa	49956473	49974305	ALDH16A1	5.441687368	760
chr19.fa	49977486	49989488	FLT3LG	0	0
chr19.fa	49993223	49993304	SNORD32A	0	0
chr19.fa	49993874	49993956	SNORD33	0	0
chr19.fa	49994164	49994229	SNORD34	0	0
chr19.fa	49994432	49994517	SNORD35A	0	0
chr19.fa	49999634	50002944	RPS11	1645.766946	45068
chr19.fa	50000976	50001062	SNORD35B	0	0
chr19.fa	50004042	50004125	MIR150	0	0
chr19.fa	50015536	50029685	FCGRT	4.826776029	456
chr19.fa	50030875	50046890	RCN3	0	0
chr19.fa	50058968	50083803	NOSIP	23.08975402	1140
chr19.fa	50084587	50094265	PRRG2	1.21336574	76
chr19.fa	50094912	50129696	PRR12	8.520247107	2660
chr19.fa	50138552	50143400	RRAS	32.66858267	1444
chr19.fa	50145382	50161906	SCAF1	17.63450079	3420
chr19.fa	50162826	50168103	IRF3	16.35308338	1178
chr19.fa	50168399	50177173	BCL2L12	7.348468499	532
chr19.fa	50180496	50191704	PRMT1	37.74910692	2502
chr19.fa	50191942	50194247	C19orf76	0	0
chr19.fa	50194365	50216988	CPT1C	0	0
chr19.fa	50243011	50266515	TSKS	0	0
chr19.fa	50270180	50310123	AP2A1	28.05685882	4104
chr19.fa	50310370	50316567	FUZ	3.382568347	228
chr19.fa	50321536	50340237	MED25	10.82066045	1140
chr19.fa	50354416	50363999	PTOV1	50.69893959	4295
chr19.fa	50364460	50370822	PNKP	16.69556565	1292
chr19.fa	50372297	50380644	AKT1S1	27.0149566	2964

chr19.fa	50380682	50392007	TBC1D17	16.38866595	1748
chr19.fa	50392914	50407578	IL4I1	0	0
chr19.fa	50410084	50432773	NUP62	15.04542382	2171
chr19.fa	50431959	50437193	ATF5	15.01629059	1553
chr19.fa	50452250	50464429	SIGLEC11	0.533960986	76
chr19.fa	50472912	50479076	SIGLEC16	0	0
chr19.fa	50479724	50528805	VRK3	29.67341959	2802
chr19.fa	50529212	50552031	ZNF473	6.126429508	1292
chr19.fa	50553937	50570052	FLJ26850	4.708019191	304
chr19.fa	50643459	50643577	SNAR-D	0	0
chr19.fa	50655805	50666538	IZUMO2	0	0
chr19.fa	50706885	50813801	MYH14	89.62249381	27855
chr19.fa	50818765	50832634	KCNC3	2.12872743	304
chr19.fa	50837057	50848005	NAPSB	1.270742639	76
chr19.fa	50861734	50868931	NAPSA	6.071054128	380
chr19.fa	50879685	50886267	NR1H2	23.71311622	2204
chr19.fa	50887593	50921271	POLD1	0.980744669	152
chr19.fa	50922195	50932250	SPIB	0	0
chr19.fa	50936160	50969583	MYBPC2	0.469689964	76
chr19.fa	50970045	50980003	FAM71E1	0	0
chr19.fa	50981186	50986608	C19orf63	6.381512078	532
chr19.fa	51009259	51014345	JOSD2	10.98945528	380
chr19.fa	51014857	51017947	ASPDH	10.75683421	608
chr19.fa	51020150	51071302	LRRC4B	0	0
chr19.fa	51108220	51108342	SNAR-F	0	0
chr19.fa	51125234	51143092	SYT3	0	0
chr19.fa	51152702	51162567	LOC342918	6.628143787	228
chr19.fa	51165084	51220195	SHANK1	0.254415397	76
chr19.fa	51226605	51228981	CLEC11A	1.201356621	76
chr19.fa	51273858	51274928	GPR32	0	0
chr19.fa	51293672	51298481	ACPT	0	0
chr19.fa	51300961	51307974	C19orf48	20.8782971	1565
chr19.fa	51302286	51302382	SNORD88B	0	0
chr19.fa	51302696	51302792	SNORD88A	0	0
chr19.fa	51305582	51305678	SNORD88C	0	0
chr19.fa	51322404	51327043	KLK1	9.669119433	380
chr19.fa	51328545	51334779	KLK15	0	0
chr19.fa	51358171	51364020	KLK3	391.0629297	33516
chr19.fa	51376689	51383823	KLK2	245.5479974	31302
chr19.fa	51385352	51399654	KLKP1	4.507200045	228
chr19.fa	51409608	51413994	KLK4	614.8361716	37240
chr19.fa	51446559	51456344	KLK5	0	0
chr19.fa	51461887	51472929	KLK6	0	0

chr19.fa	51479729	51487150	KLK7	0	0
chr19.fa	51499264	51504958	KLK8	0	0
chr19.fa	51505769	51512890	KLK9	0	0
chr19.fa	51516000	51523431	KLK10	0.514390571	76
chr19.fa	51525487	51531290	KLK11	12.83085344	836
chr19.fa	51532348	51538148	KLK12	0	0
chr19.fa	51559463	51568367	KLK13	0	0
chr19.fa	51581154	51587502	KLK14	1.491799373	76
chr19.fa	51600863	51611647	CTU1	4.010823151	380
chr19.fa	51628137	51639520	SIGLEC9	0	0
chr19.fa	51645558	51656783	SIGLEC7	0	0
chr19.fa	51670585	51676780	SIGLECP3	0	0
chr19.fa	51728335	51743274	CD33	0	0
chr19.fa	51760964	51772582	C19orf75	0	0
chr19.fa	51815102	51834102	IGLON5	0	0
chr19.fa	51834795	51845378	VSIG10L	0.460127147	76
chr19.fa	51848409	51869672	ETFB	53.61915687	3496
chr19.fa	51870352	51872257	CLDND2	0	0
chr19.fa	51874874	51875960	NKG7	0	0
chr19.fa	51883163	51891210	LIM2	0	0
chr19.fa	51891543	51893828	LOC147646	0	0
chr19.fa	51913275	51921057	SIGLEC10	0	0
chr19.fa	51919777	51919919	LOC100129083	0	0
chr19.fa	51954251	51961708	SIGLEC8	0	0
chr19.fa	51979838	51986611	CEACAM18	0	0
chr19.fa	51994613	52005043	SIGLEC12	0	0
chr19.fa	52022784	52035110	SIGLEC6	0	0
chr19.fa	52074531	52092991	ZNF175	9.823903625	1672
chr19.fa	52095036	52097633	FLJ30403	0	0
chr19.fa	52114781	52133727	SIGLEC5	0	0
chr19.fa	52145806	52150132	SIGLEC14	0	0
chr19.fa	52195865	52195934	MIR99B	0	0
chr19.fa	52196039	52196117	MIRLET7E	0	0
chr19.fa	52196507	52196592	MIR125A	0	0
chr19.fa	52196593	52208443	NCRNA00085	1.721084578	152
chr19.fa	52216365	52227221	HAS1	0	0
chr19.fa	52249023	52255150	FPR1	0	0
chr19.fa	52264453	52273779	FPR2	0	0
chr19.fa	52298411	52329334	FPR3	0	0
chr19.fa	52359056	52391229	ZNF577	5.48927906	836
chr19.fa	52392488	52408305	ZNF649	13.09394208	1900
chr19.fa	52430688	52449011	ZNF613	18.01745823	1976
chr19.fa	52467593	52490079	ZNF350	36.09940988	3800

chr19.fa	52494587	52511483	ZNF615	13.60321766	2503
chr19.fa	52516577	52531680	ZNF614	12.28955354	2584
chr19.fa	52536677	52552073	ZNF432	11.82408901	1368
chr19.fa	52567719	52599018	ZNF841	5.668748662	988
chr19.fa	52617653	52643191	ZNF616	7.211030811	988
chr19.fa	52658125	52674896	ZNF836	2.535480718	380
chr19.fa	52693055	52729678	PPP2R1A	106.3558659	11999
chr19.fa	52772824	52795976	ZNF766	10.7944183	1444
chr19.fa	52785050	52785143	MIR643	0	0
chr19.fa	52800426	52829174	ZNF480	3.20843613	684
chr19.fa	52839498	52870376	ZNF610	4.96688241	532
chr19.fa	52873170	52889046	ZNF880	20.92166336	2128
chr19.fa	52901121	52921657	ZNF528	5.09853793	912
chr19.fa	52934667	52942699	ZNF534	0	0
chr19.fa	52956829	53020131	ZNF578	0.752126637	228
chr19.fa	53030909	53059303	ZNF808	10.20952976	1520
chr19.fa	53073526	53090427	ZNF701	2.416279098	608
chr19.fa	53099937	53103405	ZNF137P	0.680516709	76
chr19.fa	53115618	53193886	ZNF83	7.523045498	1140
chr19.fa	53206066	53238297	ZNF611	9.343983672	1976
chr19.fa	53268748	53290034	ZNF600	8.626772435	988
chr19.fa	53300661	53324922	ZNF28	8.608758757	1824
chr19.fa	53341785	53360902	ZNF468	6.708649358	1216
chr19.fa	53379425	53394599	ZNF320	4.675772484	1292
chr19.fa	53430388	53445847	ZNF321	4.344854555	456
chr19.fa	53452632	53466114	ZNF816	8.146852481	988
chr19.fa	53471504	53496784	ZNF702P	13.5349436	1824
chr19.fa	53517344	53519833	HERV-V1	0	0
chr19.fa	53547991	53554380	HERV-V2	0	0
chr19.fa	53569867	53606687	ZNF160	6.236735484	1216
chr19.fa	53611132	53636173	ZNF415	7.953817023	1064
chr19.fa	53641957	53662322	ZNF347	3.70414585	760
chr19.fa	53666552	53696619	ZNF665	0.513501007	76
chr19.fa	53738638	53758111	ZNF677	0.967845986	152
chr19.fa	53761545	53762855	VN1R2	0	0
chr19.fa	53770013	53770918	VN1R4	0	0
chr19.fa	53784811	53788200	LOC646508	0	0
chr19.fa	53792854	53794875	BIRC8	0	0
chr19.fa	53837002	53858122	ZNF845	5.096758801	988
chr19.fa	53868968	53889841	ZNF525	8.597861594	2356
chr19.fa	53898397	53915262	ZNF765	2.954243124	608
chr19.fa	53935227	53961515	ZNF761	43.63068385	8185
chr19.fa	53945049	53947925	LOC147804	18.21182804	2356

chr19.fa	53970989	53997546	ZNF813	8.475101717	1596
chr19.fa	54024177	54083523	ZNF331	4.772734996	1216
chr19.fa	54102887	54106751	LOC284379	1.381938179	76
chr19.fa	54135310	54140263	DPRX	0	0
chr19.fa	54169927	54172508	MIR512-2	0	0
chr19.fa	54175222	54175294	MIR1323	0	0
chr19.fa	54177451	54177574	MIR498	0	0
chr19.fa	54178965	54179051	MIR520E	0	0
chr19.fa	54183194	54183277	MIR519E	0	0
chr19.fa	54185413	54185499	MIR520F	0	0
chr19.fa	54189723	54189809	MIR519C	0	0
chr19.fa	54191735	54191821	MIR1283-1	0	0
chr19.fa	54194135	54194219	MIR520A	0	0
chr19.fa	54197647	54197729	MIR526B	0	0
chr19.fa	54198467	54198547	MIR519B	0	0
chr19.fa	54200787	54200871	MIR525	0	0
chr19.fa	54201639	54201725	MIR523	0	0
chr19.fa	54203269	54203355	MIR518F	0	0
chr19.fa	54204481	54204541	MIR520B	0	0
chr19.fa	54205991	54206073	MIR518B	0	0
chr19.fa	54209506	54209590	MIR526A1	0	0
chr19.fa	54210707	54210793	MIR520C	0	0
chr19.fa	54211989	54212089	MIR518C	0	0
chr19.fa	54214256	54214342	MIR524	0	0
chr19.fa	54215522	54215608	MIR517A	0	0
chr19.fa	54216601	54216688	MIR519D	0	0
chr19.fa	54219848	54219934	MIR521-2	0	0
chr19.fa	54223350	54223436	MIR520D	0	0
chr19.fa	54224330	54224396	MIR517B	0	0
chr19.fa	54225420	54225509	MIR520G	0	0
chr19.fa	54228696	54228780	MIR516B2	0	0
chr19.fa	54230176	54230240	MIR526A2	0	0
chr19.fa	54233092	54233179	MIR518E	0	0
chr19.fa	54234260	54234344	MIR518A1	0	0
chr19.fa	54238131	54238217	MIR518D	0	0
chr19.fa	54240099	54240188	MIR516B1	0	0
chr19.fa	54242587	54242673	MIR518A2	0	0
chr19.fa	54244567	54244661	MIR517C	0	0
chr19.fa	54245766	54245853	MIR520H	0	0
chr19.fa	54251890	54251976	MIR521-1	0	0
chr19.fa	54254465	54254551	MIR522	0	0
chr19.fa	54255651	54255735	MIR519A1	0	0
chr19.fa	54257272	54257356	MIR527	0	0

chr19.fa	54259995	54260084	MIR516A1	0	0
chr19.fa	54261486	54261572	MIR1283-2	0	0
chr19.fa	54264387	54264476	MIR516A2	0	0
chr19.fa	54265598	54265684	MIR519A2	0	0
chr19.fa	54290929	54290995	MIR371	0	0
chr19.fa	54291144	54291210	MIR372	0	0
chr19.fa	54291959	54292027	MIR373	0	0
chr19.fa	54296855	54327648	NLRP12	0	0
chr19.fa	54369611	54379689	MYADM	1.464222879	228
chr19.fa	54385467	54410901	PRKCG	0	0
chr19.fa	54415991	54446969	CACNG7	0	0
chr19.fa	54466290	54493469	CACNG8	0	0
chr19.fa	54495542	54515920	CACNG6	0	0
chr19.fa	54544080	54567207	VSTM1	0	0
chr19.fa	54573201	54584634	TARM1	0	0
chr19.fa	54597933	54604148	OSCAR	0	0
chr19.fa	54606160	54610281	NDUFA3	105.038866	1672
chr19.fa	54610320	54618789	TFPT	26.99961162	1140
chr19.fa	54619056	54635150	PRPF31	19.39695011	1672
chr19.fa	54641436	54659378	CNOT3	8.993495328	1140
chr19.fa	54659447	54663446	LENG1	16.48940911	608
chr19.fa	54663834	54676944	TMC4	8.859838288	988
chr19.fa	54677106	54693733	MBOAT7	0.555755312	76
chr19.fa	54694119	54698394	TSEN34	17.5693402	1900
chr19.fa	54704726	54711515	RPS9	1168.473209	37462
chr19.fa	54720147	54726959	LILRB3	0	0
chr19.fa	54740469	54746724	LILRA6	0.430104351	76
chr19.fa	54754270	54761167	LILRB5	0	0
chr19.fa	54777675	54785033	LILRB2	0	0
chr19.fa	54799855	54804221	LILRA3	0	0
chr19.fa	54818353	54824409	LILRA5	0	0
chr19.fa	54844692	54850421	LILRA4	0	0
chr19.fa	54865235	54876721	LAIR1	0	0
chr19.fa	54926635	54948054	TTYH1	0.833076991	76
chr19.fa	54960065	54972978	LENG8	11.26188436	1900
chr19.fa	54973197	54974894	LENG9	10.9492025	836
chr19.fa	54976210	54984422	CDC42EP5	135.2137774	5320
chr19.fa	55014013	55021897	LAIR2	0	0
chr19.fa	55043909	55055195	KIR3DX1	0	0
chr19.fa	55085257	55099027	LILRA2	0	0
chr19.fa	55105113	55112540	LILRA1	0	0
chr19.fa	55128629	55149004	LILRB1	0	0
chr19.fa	55174124	55179846	LILRB4	0	0

chr19.fa	55219601	55224887	LILRP2	0	0
chr19.fa	55235984	55248003	KIR3DL3	0	0
chr19.fa	55249974	55264504	KIR2DL3	0	0
chr19.fa	55266474	55309738	KIR3DP1	0	0
chr19.fa	55281265	55295778	KIR2DL1	0	0
chr19.fa	55315067	55325972	KIR2DL4	0	0
chr19.fa	55327893	55342233	KIR3DL1	0	0
chr19.fa	55344174	55360023	KIR2DS4	0	0
chr19.fa	55361898	55378662	KIR3DL2	0	0
chr19.fa	55385549	55401839	FCAR	0	0
chr19.fa	55417508	55424439	NCR1	0	0
chr19.fa	55434877	55458873	NLRP7	0	0
chr19.fa	55476652	55512510	NLRP2	3.224670679	532
chr19.fa	55525075	55549632	GP6	0.745899687	76
chr19.fa	55555692	55580914	RDH13	6.719101739	836
chr19.fa	55587221	55599291	EPS8L1	14.21345879	1748
chr19.fa	55602281	55628927	PPP1R12C	12.48859356	1642
chr19.fa	55644161	55660606	TNNT1	0	0
chr19.fa	55663136	55669100	TNNI3	0	0
chr19.fa	55670031	55678018	C19orf51	0	0
chr19.fa	55684469	55691720	SYT5	0	0
chr19.fa	55692615	55720874	PTPRH	0	0
chr19.fa	55738002	55740632	TMEM86B	0.965177293	76
chr19.fa	55741147	55770038	PPP6R1	27.41815163	4484
chr19.fa	55773591	55791751	HSPBP1	4.429140775	380
chr19.fa	55795534	55823903	BRSK1	0	0
chr19.fa	55824227	55836708	TMEM150B	0	0
chr19.fa	55851221	55859489	SUV420H2	0	0
chr19.fa	55861070	55866182	COX6B2	0	0
chr19.fa	55866276	55874620	FAM71E2	0	0
chr19.fa	55875757	55881814	IL11	0	0
chr19.fa	55888204	55889612	TMEM190	0	0
chr19.fa	55890612	55895627	LOC388564	35.74380654	1064
chr19.fa	55897300	55903451	RPL28	141.8961846	30856
chr19.fa	55912650	55919325	UBE2S	7.09560984	380
chr19.fa	55940105	55954230	SHISA7	0	0
chr19.fa	55964346	55973049	ISOC2	28.56769113	1440
chr19.fa	55987699	55995854	ZNF628	1.318111939	228
chr19.fa	55996557	55998935	NAT14	1.252061788	76
chr19.fa	55999870	56030466	SSC5D	0	0
chr19.fa	56041100	56048435	SBK2	0	0
chr19.fa	56052023	56056909	LOC100130827	0	0
chr19.fa	56088891	56092211	ZNF579	1.559183871	152

chr19.fa	56102737	56110893	FIZ1	3.812450307	456
chr19.fa	56111730	56114504	ZNF524	12.19236864	608
chr19.fa	56124959	56129907	ZNF865	1.024555712	228
chr19.fa	56132107	56135941	ZNF784	6.774254727	608
chr19.fa	56152392	56154836	ZNF580	8.574065748	608
chr19.fa	56154986	56156989	ZNF581	22.05763701	1216
chr19.fa	56158954	56164526	CCDC106	3.393910292	304
chr19.fa	56165416	56186082	U2AF2	35.64395295	4999
chr19.fa	56186561	56207133	EPN1	37.29453955	5472
chr19.fa	56219798	56249768	NLRP9	0.970292288	152
chr19.fa	56270507	56274541	RFPL4A	0	0
chr19.fa	56296763	56347943	NLRP11	0	0
chr19.fa	56348129	56393220	NLRP4	0	0
chr19.fa	56407311	56443702	NLRP13	0	0
chr19.fa	56459198	56499995	NLRP8	0.429659569	76
chr19.fa	56511092	56573174	NLRP5	0	0
chr19.fa	56598732	56632649	ZNF787	20.16286499	1748
chr19.fa	56652556	56672262	ZNF444	7.445653402	684
chr19.fa	56687389	56697144	GALP	0	0
chr19.fa	56701058	56704421	ZSCAN5B	0	0
chr19.fa	56732679	56739659	ZSCAN5A	0.879334335	76
chr19.fa	56879468	56891196	ZNF542	5.867121507	988
chr19.fa	56894648	56904889	ZNF582	0.735892088	76
chr19.fa	56905045	56910539	LOC386758	0	0
chr19.fa	56915383	56936400	ZNF583	0.634036973	76
chr19.fa	56950693	56988770	ZNF667	0.757686414	152
chr19.fa	56989243	57006805	LOC100128252	26.55705336	2128
chr19.fa	57019212	57040269	ZNF471	1.701736554	380
chr19.fa	57050317	57068170	ZFP28	4.135584548	760
chr19.fa	57078890	57094262	ZNF470	1.656813556	532
chr19.fa	57106664	57135544	ZNF71	3.243129139	456
chr19.fa	57154527	57168614	LOC147670	0	0
chr19.fa	57174953	57184246	ZNF835	3.018069364	228
chr19.fa	57285923	57352097	ZIM2	0	0
chr19.fa	57321445	57351954	PEG3	3.351433595	947
chr19.fa	57352270	57359922	MIMT1	0	0
chr19.fa	57631509	57643293	USP29	0	0
chr19.fa	57645464	57656570	ZIM3	0	0
chr19.fa	57663094	57678856	DUXA	0	0
chr19.fa	57702868	57734214	ZNF264	6.808947736	3724
chr19.fa	57742377	57746916	AURKC	0	0
chr19.fa	57752053	57774106	ZNF805	2.336885482	1064
chr19.fa	57791853	57805436	ZNF460	12.29533571	2128

chr19.fa	57831865	57842144	ZNF543	3.22289155	532
chr19.fa	57862645	57871265	ZNF304	11.48205153	2280
chr19.fa	57874879	57876721	TRAPPC2P1	0	0
chr19.fa	57879912	57890925	ZNF547	0	0
chr19.fa	57901218	57913919	ZNF548	2.971812019	608
chr19.fa	57922529	57933307	ZNF17	2.524583555	304
chr19.fa	57946693	57957191	ZNF749	4.03928921	532
chr19.fa	57966542	57968107	VN1R1	0	0
chr19.fa	57980954	57988938	ZNF772	6.722215214	1638
chr19.fa	57999079	58006048	ZNF419	3.571823157	380
chr19.fa	58011309	58019510	ZNF773	3.09123603	304
chr19.fa	58038693	58052244	ZNF549	4.511425475	836
chr19.fa	58058343	58067725	ZNF550	2.949795302	152
chr19.fa	58082934	58090243	ZNF416	4.715580488	532
chr19.fa	58095628	58103758	ZIK1	5.684538429	684
chr19.fa	58111253	58119637	ZNF530	1.140421465	152
chr19.fa	58125830	58133636	ZNF134	13.53338686	1368
chr19.fa	58144535	58154147	ZNF211	6.055486753	684
chr19.fa	58180303	58190520	ZSCAN4	0.752571419	76
chr19.fa	58193357	58201169	ZNF551	6.393521196	1064
chr19.fa	58211810	58220579	ZNF154	0.615356122	76
chr19.fa	58231119	58238995	ZNF671	6.249634167	684
chr19.fa	58258164	58269527	ZNF776	7.345799806	1748
chr19.fa	58281025	58291984	ZNF586	5.341388991	532
chr19.fa	58318450	58326281	ZNF552	22.27691461	2356
chr19.fa	58336667	58338830	FKBP1AP1	1.028781142	76
chr19.fa	58361269	58376487	ZNF587	4.938416351	1520
chr19.fa	58380747	58400442	ZNF814	4.304156987	1216
chr19.fa	58417142	58427978	ZNF417	2.875071899	608
chr19.fa	58433252	58446740	ZNF418	5.490613406	912
chr19.fa	58452201	58459077	ZNF256	3.824014643	380
chr19.fa	58469805	58485902	C19orf18	0	0
chr19.fa	58488441	58514714	ZNF606	5.568227894	988
chr19.fa	58515751	58518574	LOC100128398	1.044570909	76
chr19.fa	58545434	58565999	ZSCAN1	0	0
chr19.fa	58570607	58581110	ZNF135	0	0
chr19.fa	58595209	58629793	ZSCAN18	8.623436568	1824
chr19.fa	58637695	58662148	ZNF329	10.39945174	1596
chr19.fa	58694396	58724927	ZNF274	12.50660724	1596
chr19.fa	58740070	58775008	ZNF544	10.11212247	1596
chr19.fa	58790318	58807254	ZNF8	4.58859518	456
chr19.fa	58838385	58853712	ZSCAN22	3.64031961	760
chr19.fa	58858172	58864865	A1BG	2.590411315	152

chr19.fa	58863336	58865223	A1BG-AS1	0	0
chr19.fa	58865725	58874120	ZNF497	0	0
chr19.fa	58878990	58892389	ZNF837	0.831297862	76
chr19.fa	58898636	58906171	RPS5	643.2235036	21432
chr19.fa	58907457	58908446	LOC646862	3.414592662	152
chr19.fa	58920063	58929692	ZNF584	5.237309965	532
chr19.fa	58944181	58951589	ZNF132	10.03739906	1368
chr19.fa	58962971	58969199	ZNF324B	2.257269475	304
chr19.fa	58978412	58984945	ZNF324	1.569413861	228
chr19.fa	58987795	58992597	ZNF446	5.993439641	532
chr19.fa	59009700	59023432	SLC27A5	2.858614959	304
chr19.fa	59024897	59030921	ZBTB45	5.030263868	532
chr19.fa	59055836	59062082	TRIM28	60.33647949	8028
chr19.fa	59062933	59066486	CHMP2A	95.63683821	4438
chr19.fa	59067079	59070343	UBE2M	32.05989828	2197
chr19.fa	59070553	59086164	LOC100131691	0.840415896	76
chr19.fa	59073284	59084942	MZF1	4.678218786	532
chr19.fa	59086766	59095762	MGC2752	6.070609346	912
chr2.fa	38814	46588	FAM110C	6.823847939	1216
chr2.fa	218136	264068	SH3YL1	66.08306503	5313
chr2.fa	264869	278282	ACP1	53.80129517	4028
chr2.fa	279561	288308	FAM150B	0	0
chr2.fa	667973	677439	TMEM18	11.93617412	1140
chr2.fa	779837	864112	LOC339822	0	0
chr2.fa	946554	1371384	SNTG2	0.895791275	76
chr2.fa	1417233	1546499	TPO	0	0
chr2.fa	1635659	1748291	PXDN	0.247743665	76
chr2.fa	1792885	2335045	MYT1L	0	0
chr2.fa	2323004	2330880	LOC730811	0	0
chr2.fa	3192741	3381653	TSSC1	9.82079015	760
chr2.fa	3383446	3483342	TTC15	14.73207479	1672
chr2.fa	3501690	3523350	ADI1	183.5389146	13832
chr2.fa	3592691	3605940	RNASEH1	16.53989189	1216
chr2.fa	3622853	3628509	RPS7	406.2913814	13300
chr2.fa	3642637	3692047	COLEC11	2.446079503	152
chr2.fa	3705786	3750260	ALLC	0	0
chr2.fa	4675808	4703812	LOC727982	0	0
chr2.fa	5832799	5841517	SOX11	0	0
chr2.fa	6072819	6120350	LOC150622	0	0
chr2.fa	6122110	6128364	LOC400940	0	0
chr2.fa	6988451	7005936	CMPK2	1.130858648	152
chr2.fa	7017796	7038363	RSAD2	0.4812543	76
chr2.fa	7052407	7058813	LOC386597	0	0

chr2.fa	7057523	7184309	RNF144A	6.773587554	1748
chr2.fa	8062556	8116945	LOC339788	0	0
chr2.fa	8147901	8468549	C2orf46	0	0
chr2.fa	8822113	8824583	ID2	60.71743541	3724
chr2.fa	8868987	8977755	KIDINS220	4.821883425	1596
chr2.fa	8996701	9143876	MBOAT2	7.553290685	1292
chr2.fa	9346894	9545812	ASAP2	44.84783024	11529
chr2.fa	9545815	9563643	ITGB1BP1	10.51976532	912
chr2.fa	9563868	9613227	CPSF3	13.23493803	1292
chr2.fa	9614670	9628591	IAH1	22.38633103	912
chr2.fa	9629411	9695917	ADAM17	3.32986166	532
chr2.fa	9724106	9771106	YWHAQ	100.3942282	9778
chr2.fa	9983571	10074545	TAF1B	5.146129621	532
chr2.fa	10091792	10142412	GRHL1	26.08847536	4229
chr2.fa	10183682	10194963	KLF11	3.934320619	760
chr2.fa	10196926	10220538	CYS1	14.29707783	1748
chr2.fa	10262695	10271546	RRM2	0	0
chr2.fa	10281509	10351856	C2orf48	0	0
chr2.fa	10332740	10332797	MIR4261	0	0
chr2.fa	10443040	10567743	HPCAL1	13.2531741	1292
chr2.fa	10580508	10588453	ODC1	225.4109298	20900
chr2.fa	10586840	10586975	SNORA80B	0	0
chr2.fa	10710894	10830112	NOL10	10.15548873	1596
chr2.fa	10861775	10925236	ATP6V1C2	0	0
chr2.fa	10927407	10952960	PDIA6	22.1875134	1292
chr2.fa	11052063	11054351	KCNF1	0	0
chr2.fa	11273179	11286916	C2orf50	0	0
chr2.fa	11295540	11318998	PQLC3	2.839044544	228
chr2.fa	11321778	11484711	ROCK2	15.79777285	4547
chr2.fa	11584501	11606297	E2F6	1.472228958	228
chr2.fa	11674242	11782912	GREB1	52.36286966	24320
chr2.fa	11798304	11810329	NTSR2	0	0
chr2.fa	11886740	11967533	LPIN1	3.786875332	912
chr2.fa	11977059	11977112	MIR4262	0	0
chr2.fa	12856998	12882858	TRIB2	19.56396581	3876
chr2.fa	12877493	12877570	MIR3125	0	0
chr2.fa	14772810	14780168	FAM84A	18.88900888	5396
chr2.fa	15307032	15701454	NBAS	13.22982304	4332
chr2.fa	15731770	15771225	DDX1	36.46524321	4437
chr2.fa	16080020	16081845	MYCNOS	0	0
chr2.fa	16080683	16087129	MYCN	6.488259797	760
chr2.fa	16730730	16847134	FAM49A	4.675994875	988
chr2.fa	17691986	17699706	RAD51AP2	0	0

chr2.fa	17721807	17837706	VSNL1	0	0
chr2.fa	17845079	17935096	SMC6	4.178061244	988
chr2.fa	17935177	17966632	GEN1	0.263088649	76
chr2.fa	17997786	17998367	MSGN1	0	0
chr2.fa	18059945	18114225	KCNS3	7.25395229	760
chr2.fa	18741446	18741959	RDH14	9.691803323	224
chr2.fa	18744137	18768259	NT5C1B	0	0
chr2.fa	19551246	19558372	OSR1	1.784688427	152
chr2.fa	20068615	20084808	FLJ12334	0	0
chr2.fa	20096514	20101744	TTC32	10.31850139	380
chr2.fa	20110029	20189884	WDR35	3.340314041	1043
chr2.fa	20191813	20212455	MATN3	0	0
chr2.fa	20232411	20251789	LAPTM4A	16.27924954	1292
chr2.fa	20400558	20425194	SDC1	21.55703468	3192
chr2.fa	20448453	20527144	PUM2	36.78504158	10108
chr2.fa	20646835	20649201	RHOB	44.98549032	4788
chr2.fa	20817564	20850864	HS1BP3	12.66050187	1368
chr2.fa	20866424	20871250	GDF7	0.847532411	76
chr2.fa	20884818	21022827	C2orf43	7.693397066	988
chr2.fa	21224301	21266945	APOB	0	0
chr2.fa	23608298	23931483	KLHL29	0.997424	228
chr2.fa	23971534	24149934	ATAD2B	5.247095172	1900
chr2.fa	24163376	24223693	UBXN2A	9.821457323	836
chr2.fa	24232953	24247145	MFSD2B	0	0
chr2.fa	24252206	24270296	C2orf44	4.340406734	760
chr2.fa	24272584	24286550	FKBP1B	13.50870145	605
chr2.fa	24290454	24299314	SF3B14	26.30619622	912
chr2.fa	24300305	24307728	TP53I3	6.054374797	456
chr2.fa	24338244	24346151	PFN4	0	0
chr2.fa	24346350	24392507	LOC375190	1.245612446	76
chr2.fa	24397972	24414567	C2orf84	0	0
chr2.fa	24425735	24583397	ITSN2	50.34000038	15252
chr2.fa	24807346	24993570	NCOA1	27.71971394	8664
chr2.fa	25013136	25016174	C2orf79	78.53318493	1748
chr2.fa	25016252	25045245	CENPO	3.747512111	532
chr2.fa	25042039	25142055	ADCY3	0	0
chr2.fa	25166505	25194963	DNAJC27	4.062862664	912
chr2.fa	25194981	25262563	LOC729723	1.084823695	228
chr2.fa	25264973	25382004	EFR3B	0.453233024	152
chr2.fa	25383722	25391559	POMC	0	0
chr2.fa	25455845	25565459	DNMT3A	9.250801809	2344
chr2.fa	25551509	25551590	MIR1301	0	0
chr2.fa	25600112	25896503	DTNB	9.564373233	1064

chr2.fa	25962253	26101312	ASXL2	17.90537313	5776
chr2.fa	26149455	26205443	KIF3C	3.112140792	760
chr2.fa	26256729	26360323	RAB10	38.76721329	6598
chr2.fa	26395960	26412532	FAM59B	0	0
chr2.fa	26413504	26467594	HADHA	82.4612786	11261
chr2.fa	26467616	26513333	HADHB	80.3067538	7901
chr2.fa	26531041	26569685	GPR113	0.351155517	76
chr2.fa	26568954	26618759	EPT1	1.911006562	684
chr2.fa	26624784	26679579	C2orf39	0	0
chr2.fa	26680071	26781566	OTOF	0	0
chr2.fa	26785481	26802395	C2orf70	0	0
chr2.fa	26804073	26864211	CIB4	0	0
chr2.fa	26915581	26954066	KCNK3	1.699512643	304
chr2.fa	26987142	27004099	C2orf18	2.160306964	380
chr2.fa	27008882	27017455	CENPA	0	0
chr2.fa	27070969	27173219	DPYSL5	0	0
chr2.fa	27193525	27250087	MAPRE3	12.65360774	1064
chr2.fa	27255774	27264565	TMEM214	8.339665549	1140
chr2.fa	27274491	27293341	AGBL5	18.90212996	2736
chr2.fa	27293491	27294567	OST4	422.5430545	6384
chr2.fa	27301435	27309265	EMILIN1	0	0
chr2.fa	27309611	27323461	KHK	17.20617557	1494
chr2.fa	27322221	27341995	CGREF1	0	0
chr2.fa	27346657	27353624	ABHD1	2.03198731	127
chr2.fa	27353681	27357542	PREB	28.06264098	2660
chr2.fa	27359715	27362332	C2orf53	0	0
chr2.fa	27371945	27375819	TCF23	0	0
chr2.fa	27422455	27434898	SLC5A6	3.314071894	456
chr2.fa	27435176	27440046	C2orf28	54.27854643	2736
chr2.fa	27440258	27466654	CAD	4.280138751	1368
chr2.fa	27477440	27485960	SLC30A3	0	0
chr2.fa	27498289	27504296	DNAJC5G	0	0
chr2.fa	27505297	27530264	TRIM54	0	0
chr2.fa	27530308	27531130	UCN	0	0
chr2.fa	27532360	27545969	MPV17	37.72041847	1708
chr2.fa	27548721	27579868	GTF3C2	17.67786705	3116
chr2.fa	27587219	27593324	EIF2B4	28.76072659	2280
chr2.fa	27593389	27599994	SNX17	50.22569136	4614
chr2.fa	27600102	27603593	ZNF513	9.495431998	912
chr2.fa	27604061	27632496	PPM1G	30.75757611	3116
chr2.fa	27615490	27616443	FTH1P3	1.771567354	76
chr2.fa	27651473	27665124	NRBP1	41.08008054	4062
chr2.fa	27665233	27669348	KRTCAP3	1.668377892	76

chr2.fa	27667240	27712571	IFT172	6.371949261	1520
chr2.fa	27714750	27718126	FNDC4	20.22935993	1520
chr2.fa	27719706	27746550	GCKR	0	0
chr2.fa	27799389	27805589	C2orf16	0.54508054	152
chr2.fa	27805893	27845963	ZNF512	12.8268504	1976
chr2.fa	27848506	27851862	CCDC121	4.933078965	608
chr2.fa	27851899	27873678	GPN1	21.23100936	1748
chr2.fa	27873714	27886337	SUPT7L	7.805926954	1444
chr2.fa	27886450	27917847	SLC4A1AP	9.929094607	1276
chr2.fa	27994584	28002608	MRPL33	99.77553623	2324
chr2.fa	28004266	28081438	RBKS	3.084341907	152
chr2.fa	28112323	28113981	LOC100302650	0	0
chr2.fa	28113557	28561767	BRE	21.6688974	1976
chr2.fa	28219234	28219316	MIR4263	0	0
chr2.fa	28615779	28637516	FOSL2	42.77314384	7676
chr2.fa	28718938	28866653	PLB1	0	0
chr2.fa	28974614	29025806	PPP1CB	76.76450866	17252
chr2.fa	29033700	29073475	SPDYA	0	0
chr2.fa	29072688	29093175	TRMT61B	11.08708497	684
chr2.fa	29117533	29171080	WDR43	16.89682957	2660
chr2.fa	29136528	29136616	SNORD92	0	0
chr2.fa	29149933	29150008	SNORD53	0	0
chr2.fa	29204164	29275096	FAM179A	0	0
chr2.fa	29284558	29297127	C2orf71	0.239959977	76
chr2.fa	29338308	29406679	CLIP4	5.137233978	988
chr2.fa	29415640	30144432	ALK	0	0
chr2.fa	30369750	30383399	YPEL5	56.07880223	6004
chr2.fa	30454397	30482899	LBH	9.7796478	1292
chr2.fa	30670123	30867091	LCLAT1	2.672251233	608
chr2.fa	30945638	31030311	CAPN13	14.86484227	1792
chr2.fa	31133333	31361571	GALNT14	2.49278163	304
chr2.fa	31395922	31440411	CAPN14	0	0
chr2.fa	31456880	31491260	EHD3	3.028966527	532
chr2.fa	31557188	31637611	XDH	0.886895632	228
chr2.fa	31749656	31806040	SRD5A2	0.691191481	76
chr2.fa	32092894	32235698	MEMO1	4.663763366	380
chr2.fa	32248972	32264844	DPY30	25.2969855	910
chr2.fa	32288680	32382706	SPAST	1.296984786	304
chr2.fa	32390910	32449181	SLC30A6	2.382920436	532
chr2.fa	32449518	32490812	NLRC4	0	0
chr2.fa	32502958	32531658	YIPF4	1.720195014	152
chr2.fa	32582096	32843965	BIRC6	14.20856618	10032
chr2.fa	32757220	32757313	MIR558	0	0

chr2.fa	32853087	33046118	TTC27	10.12457637	1216
chr2.fa	33050510	33162270	LOC285045	0	0
chr2.fa	33152194	33171202	LOC100271832	0	0
chr2.fa	33172369	33624575	LTBP1	1.863859652	532
chr2.fa	33661416	33789798	RASGRP3	0.319353593	76
chr2.fa	33808729	33824362	FAM98A	28.32350572	3496
chr2.fa	33951128	33953284	MYADML	0	0
chr2.fa	36581892	36582713	LOC100288911	4.721140265	152
chr2.fa	36583370	36778278	CRIM1	4.819659514	1216
chr2.fa	36779404	36825332	FEZ2	8.079245593	760
chr2.fa	36923833	37041937	VIT	0	0
chr2.fa	37075472	37193615	STRN	12.14544412	1900
chr2.fa	37208153	37311485	HEATR5B	13.68661432	4180
chr2.fa	37311594	37323738	CCDC75	1.344576478	76
chr2.fa	37333699	37384190	EIF2AK2	12.11386459	1596
chr2.fa	37394963	37415690	SULT6B1	0	0
chr2.fa	37428775	37458740	CEBPZ	18.28855296	2736
chr2.fa	37458774	37476303	C2orf56	4.465835304	444
chr2.fa	37477646	37544222	PRKD3	9.450286609	2508
chr2.fa	37571753	37600465	QPCT	0	0
chr2.fa	37870743	37899326	CDC42EP3	6.368391004	1140
chr2.fa	38152462	38294285	FAM82A1	1.53227455	228
chr2.fa	38294746	38303323	CYP1B1	6.232065271	1444
chr2.fa	38358247	38408993	C2orf58	0.964065337	76
chr2.fa	38522029	38604432	ATL2	9.382012547	1420
chr2.fa	38790328	38830178	HNRPLL	8.910321063	1216
chr2.fa	38893052	38961909	GALM	25.86652906	2888
chr2.fa	38970741	38978636	SRSF7	13.43820348	1504
chr2.fa	39005327	39009106	GEMIN6	17.09720394	532
chr2.fa	39024876	39103021	DHX57	10.85913411	2356
chr2.fa	39103103	39109850	MORN2	15.96656768	532
chr2.fa	39146504	39202590	ARHGEF33	0	0
chr2.fa	39186429	39186981	LOC375196	0	0
chr2.fa	39208690	39347604	SOS1	11.78539296	4408
chr2.fa	39405688	39456673	CDKL4	0	0
chr2.fa	39476422	39664219	MAP4K3	18.92459145	3500
chr2.fa	39892638	39945104	TMEM178	3.694360643	304
chr2.fa	39963200	40006416	THUMPD2	5.045386461	532
chr2.fa	40339286	40739575	SLC8A1	9.7447324	2736
chr2.fa	42104695	42121186	LOC388942	0	0
chr2.fa	42275161	42285668	PKDCC	0.675846496	76
chr2.fa	42396490	42559688	EML4	21.8383594	5449
chr2.fa	42577645	42588356	COX7A2L	115.3079965	5776

chr2.fa	42669157	42721237	KCNG3	0	0
chr2.fa	42795671	42936353	MTA3	26.39470788	2204
chr2.fa	42989639	42991401	OXER1	4.793417366	380
chr2.fa	42994229	43019751	HAAO	11.84699529	684
chr2.fa	43449541	43453745	ZFP36L2	51.25892033	8512
chr2.fa	43454350	43455994	LOC100129726	0	0
chr2.fa	43457975	43823185	THADA	10.56691223	3040
chr2.fa	43864439	43995126	PLEKHH2	0.243073452	76
chr2.fa	43902292	43903461	LOC728819	0	0
chr2.fa	44001178	44037149	DYNC2LI1	16.3699851	1032
chr2.fa	44039611	44065958	ABCG5	0	0
chr2.fa	44066103	44105605	ABCG8	0	0
chr2.fa	44113363	44223144	LRPPRC	23.7208999	7060
chr2.fa	44396000	44461742	PPM1B	15.85537214	3800
chr2.fa	44502597	44541090	SLC3A1	12.96295374	988
chr2.fa	44544748	44589001	PREPL	11.90059154	3116
chr2.fa	44589043	44999729	C2orf34	10.88337474	760
chr2.fa	45169037	45173216	SIX3	0	0
chr2.fa	45232324	45236542	SIX2	0	0
chr2.fa	45401480	45482080	UNQ6975	0	0
chr2.fa	45615819	45838433	SRBD1	10.06608751	1672
chr2.fa	45879043	46415129	PRKCE	5.205285649	1292
chr2.fa	46524541	46613842	EPAS1	25.51937658	5928
chr2.fa	46706704	46711564	LOC388946	0	0
chr2.fa	46738986	46747096	ATP6V1E2	0	0
chr2.fa	46769867	46811827	RHOQ	24.80839229	4712
chr2.fa	46815214	46844251	PIGF	0.654941734	24
chr2.fa	46844325	46852881	CRIPT	9.693360061	836
chr2.fa	46926099	46989927	SOCS5	4.350414332	912
chr2.fa	47043807	47049799	LOC388948	0	0
chr2.fa	47055003	47086145	LOC100134259	0	0
chr2.fa	47129009	47168994	MCFD2	7.442317536	1520
chr2.fa	47168313	47303275	TTC7A	4.390889509	988
chr2.fa	47314130	47382517	C2orf61	0	0
chr2.fa	47387221	47403740	CALM2	173.9329542	10183
chr2.fa	47596287	47614167	EPCAM	7.870420367	608
chr2.fa	47604814	47604909	MIR559	0	0
chr2.fa	47630263	47710360	MSH2	6.448896576	912
chr2.fa	47747915	47797470	KCNK12	0	0
chr2.fa	48010221	48034058	MSH6	11.02125721	2128
chr2.fa	48034093	48132932	FBXO11	20.12594808	3724
chr2.fa	48541795	48606434	FOXN2	2.16564435	532
chr2.fa	48667908	48742531	KLRAQ1	19.3200028	2793

chr2.fa	48757064	49003656	STON1-GTF2A1L	0	0
chr2.fa	48757308	48825654	STON1	6.311681278	980
chr2.fa	48844937	48845034	GTF2A1L	0	0
chr2.fa	48913913	48982880	LHCGR	0	0
chr2.fa	49189296	49381666	FSHR	0	0
chr2.fa	50145643	51259674	NRXN1	0	0
chr2.fa	53897118	54014067	ASB3	0	0
chr2.fa	53994929	54002287	CHAC2	1.325673236	76
chr2.fa	54014080	54046495	ERLEC1	7.610889975	1064
chr2.fa	54076259	54076342	MIR3682	0	0
chr2.fa	54080050	54082002	GPR75	0	0
chr2.fa	54087127	54087170	LOC100302652	0	0
chr2.fa	54091204	54197977	PSME4	21.59684269	6894
chr2.fa	54342410	54532435	ACYP2	7.534387443	414
chr2.fa	54480315	54483409	TSPYL6	0	0
chr2.fa	54558071	54588714	C2orf73	0	0
chr2.fa	54683454	54898583	SPTBN1	92.13395629	44391
chr2.fa	54756359	54756978	RPL23AP32	0	0
chr2.fa	54952149	55199156	EML6	0.861098267	304
chr2.fa	55199327	55277734	RTN4	19.13964363	4477
chr2.fa	55399687	55459449	C2orf63	1.264515688	304
chr2.fa	55459039	55462989	RPS27A	439.3462574	25702
chr2.fa	55463756	55496384	MTIF2	16.24077589	2204
chr2.fa	55509455	55511607	PRORSD1P	0.785040517	76
chr2.fa	55514978	55647057	CCDC88A	1.722418925	760
chr2.fa	55746740	55772216	CCDC104	33.82946411	1976
chr2.fa	55775514	55844796	SMEK2	25.40195409	5092
chr2.fa	55861198	55921011	PNPT1	2.583739583	532
chr2.fa	56093102	56150932	EFEMP1	0	0
chr2.fa	56210102	56210211	MIR217	0	0
chr2.fa	56216085	56216194	MIR216A	0	0
chr2.fa	56227849	56227930	MIR216B	0	0
chr2.fa	56411258	56613309	CCDC85A	0.424322183	76
chr2.fa	58273777	58387055	VRK2	7.977612868	617
chr2.fa	58386378	58468515	FANCL	6.306788675	363
chr2.fa	58747888	59290901	FLJ30838	0	0
chr2.fa	60678302	60780633	BCL11A	1.134861688	380
chr2.fa	60983365	61029221	PAPOLG	2.517244649	836
chr2.fa	61074895	61108449	FLJ16341	0	0
chr2.fa	61108752	61150178	REL	12.38940714	1444
chr2.fa	61167548	61245365	PUS10	3.105246668	532
chr2.fa	61244812	61279125	PEX13	8.984377293	1824
chr2.fa	61293006	61365169	KIAA1841	4.900832259	1216

chr2.fa	61368727	61372110	LOC339803	4.627068838	532
chr2.fa	61372243	61391964	C2orf74	1.73175935	76
chr2.fa	61404555	61414589	AHSA2	0.575992901	76
chr2.fa	61414687	61697849	USP34	23.28034317	11738
chr2.fa	61644379	61644512	SNORA70B	0	0
chr2.fa	61705069	61765418	XPO1	27.90251941	6060
chr2.fa	62051983	62081278	FAM161A	0	0
chr2.fa	62095262	62115791	CCT4	70.96699556	7448
chr2.fa	62132803	62363205	COMMD1	64.00370842	2052
chr2.fa	62423262	62451866	B3GNT2	4.918623545	608
chr2.fa	62727356	62733604	TMEM17	0	0
chr2.fa	62900986	63273616	EHBP1	13.81604593	3192
chr2.fa	63271100	63275656	LOC100132215	0	0
chr2.fa	63277192	63284966	OTX1	0	0
chr2.fa	63344986	63346677	DBIL5P2	0	0
chr2.fa	63348535	63815742	C2orf86	1.548286708	228
chr2.fa	63815852	63834330	MDH1	60.02313045	4788
chr2.fa	64068098	64118696	UGP2	49.93524861	5472
chr2.fa	64119667	64246214	VPS54	16.22698764	2888
chr2.fa	64319786	64371605	PELI1	13.46044259	2280
chr2.fa	64412213	64432619	LOC150992	0	0
chr2.fa	64681327	64688517	HSPC159	15.4575145	2660
chr2.fa	64751439	64820138	AFTPH	51.15595326	9484
chr2.fa	64834446	64843616	LOC339807	0	0
chr2.fa	64858755	64881046	SERTAD2	6.092848454	1520
chr2.fa	65128974	65159581	LOC400958	0	0
chr2.fa	65215579	65251000	SLC1A4	8.489779529	1824
chr2.fa	65283495	65313987	CEP68	5.923164059	1520
chr2.fa	65314143	65357435	RAB1A	72.68830253	8132
chr2.fa	65454829	65498390	ACTR2	65.68365064	11628
chr2.fa	65537985	65659656	SPRED2	11.26788892	2356
chr2.fa	66662532	66799891	MEIS1	4.250560737	608
chr2.fa	67624442	67637533	ETAA1	4.118682826	608
chr2.fa	68269332	68290159	C1D	15.79599372	912
chr2.fa	68357281	68384656	WDR92	6.025463957	608
chr2.fa	68385005	68403094	PNO1	15.35743851	1368
chr2.fa	68405989	68479651	PPP3R1	28.06664402	3800
chr2.fa	68511303	68547183	CNRIP1	0	0
chr2.fa	68592322	68624585	PLEK	0	0
chr2.fa	68689505	68694390	FBXO48	1.86964182	228
chr2.fa	68694691	68807294	APLF	3.51288952	608
chr2.fa	68872954	68882708	PROKR1	0	0
chr2.fa	68961913	69053957	ARHGAP25	0	0

chr2.fa	69092613	69098649	BMP10	0	0
chr2.fa	69172364	69180102	GKN2	0	0
chr2.fa	69201705	69208112	GKN1	0	0
chr2.fa	69240276	69476459	ANTXR1	1.89833027	608
chr2.fa	69330814	69330887	MIR3126	0	0
chr2.fa	69546905	69614382	GFPT1	29.71567389	11546
chr2.fa	69623253	69664753	NFU1	24.37050425	1292
chr2.fa	69685127	69870977	AAK1	9.378899072	8968
chr2.fa	69747177	69747303	SNORA36C	0	0
chr2.fa	69969127	70053596	ANXA4	48.1327689	4636
chr2.fa	70056818	70106727	GMCL1	12.24752163	1292
chr2.fa	70121090	70132349	SNRNP27	16.85346331	1064
chr2.fa	70142203	70170076	MXD1	4.236105317	1064
chr2.fa	70187224	70189394	ASPRV1	1.55695996	152
chr2.fa	70189398	70314147	LOC400960	0.709205158	76
chr2.fa	70314585	70316334	PCBP1	140.0427774	11020
chr2.fa	70351168	70352448	LOC100133985	2.557052653	76
chr2.fa	70377017	70418151	C2orf42	4.017939666	456
chr2.fa	70436576	70475779	TIA1	3.995700558	836
chr2.fa	70485231	70508317	PCYOX1	0.942938185	228
chr2.fa	70508506	70520869	SNRPG	11.67642133	304
chr2.fa	70523108	70529220	FAM136A	27.12504019	2204
chr2.fa	70674417	70781105	TGFA	3.173965512	608
chr2.fa	70889216	70995375	ADD2	0	0
chr2.fa	71004442	71017775	FIGLA	0	0
chr2.fa	71035777	71047732	CLEC4F	0	0
chr2.fa	71057343	71062953	CD207	0	0
chr2.fa	71127720	71160575	VAX2	0	0
chr2.fa	71162998	71192561	ATP6V1B1	11.33171516	988
chr2.fa	71205575	71212629	ANKRD53	1.594544053	152
chr2.fa	71213068	71222001	TEX261	5.521748158	836
chr2.fa	71251205	71257060	OR7E91P	1.374154491	76
chr2.fa	71295408	71305998	NAGK	31.90778278	2584
chr2.fa	71336806	71357394	MCEE	23.31281227	912
chr2.fa	71357444	71377232	MPHOSPH10	24.48548044	2736
chr2.fa	71409868	71454233	PAIP2B	15.02385189	4256
chr2.fa	71558889	71662189	ZNF638	20.61676519	6024
chr2.fa	71680753	71913893	DYSF	0.232176289	76
chr2.fa	72356367	72374963	CYP26B1	0	0
chr2.fa	72406444	73053177	EXOC6B	20.62743996	2406
chr2.fa	73114512	73119289	SPR	26.64423067	1748
chr2.fa	73144604	73162020	EMX1	5.407216751	532
chr2.fa	73169165	73298965	SFXN5	5.436127591	1012

chr2.fa	73300510	73340146	RAB11FIP5	12.06716246	2356
chr2.fa	73429386	73438340	NOTO	0	0
chr2.fa	73441366	73454355	SMYD5	8.524917319	984
chr2.fa	73455134	73460356	C2orf7	9.363776478	456
chr2.fa	73461364	73480150	CCT7	112.8372316	10310
chr2.fa	73481810	73496758	FBXO41	2.195667146	684
chr2.fa	73518057	73520829	EGR4	0	0
chr2.fa	73612886	73837046	ALMS1	6.80160883	3952
chr2.fa	73867850	73869537	NAT8	67.73298446	3268
chr2.fa	73872046	73912694	ALMS1P	1.061695022	76
chr2.fa	73927636	73928467	NAT8B	8.125725329	304
chr2.fa	73956957	73964517	TPRKB	17.98054131	608
chr2.fa	73989325	74007284	DUSP11	15.46841166	1140
chr2.fa	74011316	74044274	C2orf78	0	0
chr2.fa	74056086	74090011	STAMBP	15.79599372	1748
chr2.fa	74120093	74146780	ACTG2	0	0
chr2.fa	74153953	74186088	DGUOK	22.16127125	1140
chr2.fa	74273450	74335302	TET3	12.31223743	6080
chr2.fa	74362528	74375039	BOLA3	48.81373039	1216
chr2.fa	74383211	74405995	MOBKL1B	67.40340088	4028
chr2.fa	74425690	74442424	MTHFD2	11.09531344	1140
chr2.fa	74443369	74570534	SLC4A5	0.792824205	228
chr2.fa	74588281	74619120	DCTN1	30.13955129	6905
chr2.fa	74612845	74621008	LOC100189589	0	0
chr2.fa	74641303	74644844	C2orf81	1.516484783	152
chr2.fa	74648885	74652882	WDR54	6.041698506	304
chr2.fa	74652988	74669060	RTKN	10.60827697	1359
chr2.fa	74682199	74685087	INO80B	15.33986961	836
chr2.fa	74685577	74688010	WBP1	60.93315476	3192
chr2.fa	74688184	74692537	MOGS	4.086880901	532
chr2.fa	74699109	74699927	MRPL53	49.38816655	1368
chr2.fa	74699959	74710357	CCDC142	2.766989834	532
chr2.fa	74710449	74721691	TTC31	7.666487746	988
chr2.fa	74724644	74725445	LBX2	0	0
chr2.fa	74729744	74732169	LOC151534	0.951611437	76
chr2.fa	74732193	74734821	PCGF1	8.815137681	350
chr2.fa	74741596	74744275	TLX2	0	0
chr2.fa	74745258	74753408	DQX1	0	0
chr2.fa	74753775	74756531	AUP1	16.35641925	912
chr2.fa	74756627	74759841	HTRA2	3.496877362	228
chr2.fa	74759946	74781062	LOXL3	1.083044566	152
chr2.fa	74776147	74784678	DOK1	2.809911312	304
chr2.fa	74785010	74875164	C2orf65	0.664726942	76

chr2.fa	74881393	74909185	SEMA4F	0.791045076	152
chr2.fa	75059782	75120481	HK2	18.82473786	6004
chr2.fa	75185775	75196859	POLE4	32.27228176	1010
chr2.fa	75273590	75426645	TACR1	0	0
chr2.fa	75719444	75796848	FAM176A	1.608109909	152
chr2.fa	75873909	75889334	MRPL19	4.750718279	1672
chr2.fa	75889832	75938111	C2orf3	6.096184321	1216
chr2.fa	76974850	77749502	LRRTM4	0.350933126	76
chr2.fa	78182033	78182152	SNAR-H	0	0
chr2.fa	79252826	79255628	REG3G	0	0
chr2.fa	79312149	79315150	REG1B	0	0
chr2.fa	79347584	79350545	REG1A	0	0
chr2.fa	79362629	79365553	REG1P	0	0
chr2.fa	79384132	79386880	REG3A	0	0
chr2.fa	79740060	80875988	CTNNA2	0	0
chr2.fa	79876420	79876485	MIR4264	0	0
chr2.fa	80529003	80531487	LRRTM1	0	0
chr2.fa	83083927	83084893	LOC1720	0	0
chr2.fa	84517806	84519324	FUNDC2P2	0	0
chr2.fa	84650647	84686586	SUCLG1	85.81204502	5622
chr2.fa	84743579	85046713	DNAH6	0.396300907	228
chr2.fa	85048796	85108252	C2orf89	0	0
chr2.fa	85132763	85133799	TMSB10	515.4673887	11172
chr2.fa	85198231	85286595	KCMF1	9.398469487	3192
chr2.fa	85360583	85537511	TCF7L1	2.896199052	380
chr2.fa	85545146	85555374	TGOLN2	7.492800311	2128
chr2.fa	85569078	85581821	RETSAT	7.146537398	1064
chr2.fa	85581843	85618875	ELMOD3	6.126207117	684
chr2.fa	85621871	85637676	CAPG	228.8993563	14842
chr2.fa	85661918	85664152	SH2D6	0	0
chr2.fa	85766288	85772403	MAT2A	95.33082808	12084
chr2.fa	85776193	85788657	GGCX	3.643655476	532
chr2.fa	85804614	85809156	VAMP8	296.2158023	10336
chr2.fa	85811531	85820511	VAMP5	17.7912865	532
chr2.fa	85822837	85824831	RNF181	73.1777853	2356
chr2.fa	85825670	85829822	TMEM150A	7.613336277	532
chr2.fa	85832376	85839179	C2orf68	17.83309603	3420
chr2.fa	85843283	85876406	USP39	20.65857472	2052
chr2.fa	85884440	85895864	SFTPB	0	0
chr2.fa	85921414	85925875	GNLY	0	0
chr2.fa	85980909	86018506	ATOH8	4.612391026	1216
chr2.fa	86066271	86116157	ST3GAL5	27.61719165	3040
chr2.fa	86247339	86250991	LOC90784	5.552215736	912

chr2.fa	86253451	86333278	POLR1A	7.894438604	2356
chr2.fa	86333305	86369280	PTCD3	7.265516626	2200
chr2.fa	86362993	86363129	SNORD94	0	0
chr2.fa	86371055	86422893	IMMT	27.02629855	3647
chr2.fa	86426556	86440477	MRPL35	10.20952976	1520
chr2.fa	86441120	86565206	REEP1	0.86376696	152
chr2.fa	86668271	86719839	KDM3A	27.14994799	6004
chr2.fa	86790427	86790620	VPS24	62.24659648	543
chr2.fa	86830516	86851000	RNF103	3.410367232	760
chr2.fa	86915355	86948245	RNF103-VPS24	0	0
chr2.fa	86947414	87005164	RMND5A	19.95515172	5548
chr2.fa	87011728	87035519	CD8A	0	0
chr2.fa	87042460	87089047	CD8B	0	0
chr2.fa	87140935	88125286	RGPD2	0	0
chr2.fa	87144738	88285309	RGPD1	0	0
chr2.fa	87248970	88047609	PLGLB2	0	0
chr2.fa	87257798	87303536	LOC285074	0.767916404	76
chr2.fa	87754974	87821030	NCRNA00152	3.550696004	152
chr2.fa	88326724	88355248	KRCC1	16.39066747	1292
chr2.fa	88367299	88412902	SMYD1	0	0
chr2.fa	88422510	88427578	FABP1	48.38940821	1064
chr2.fa	88469835	88486146	THNSL2	2.400711723	228
chr2.fa	88747726	88752053	FOXI3	0	0
chr2.fa	88824169	88829103	C2orf51	0	0
chr2.fa	88856259	88927094	EIF2AK3	7.272633141	1520
chr2.fa	88991176	89050452	RPIA	10.96187879	904
chr2.fa	89065419	89106109	ANKRD36BP2	0	0
chr2.fa	91824709	91847975	LOC654342	13.96838382	380
chr2.fa	91963368	91970153	GGT8P	0	0
chr2.fa	92129159	92130496	ACTR3BP2	0	0
chr2.fa	95426673	95522820	ANKRD20A8P	0	0
chr2.fa	95534430	95613087	LOC442028	0.512611442	76
chr2.fa	95537232	95542568	TEKT4	0	0
chr2.fa	95691479	95719735	MAL	25.60877779	1216
chr2.fa	95752952	95787754	MRPS5	30.31257155	2264
chr2.fa	95813400	95825263	ZNF514	5.275116448	760
chr2.fa	95831183	95850064	ZNF2	2.137845465	380
chr2.fa	95940201	95957055	PROM2	5.000463463	1064
chr2.fa	95963072	96051825	KCNIP3	1.120406268	152
chr2.fa	96068448	96078879	FAHD2A	7.812821077	456
chr2.fa	96142715	96150479	TRIM43B	0	0
chr2.fa	96257766	96265469	TRIM43	0	0
chr2.fa	96676299	96688853	LOC729234	0.599343964	19

chr2.fa	96687694	96700727	GPAT2	0	0
chr2.fa	96778623	96781888	ADRA2B	0	0
chr2.fa	96789589	96804175	ASTL	0	0
chr2.fa	96808908	96811179	DUSP2	11.03371111	836
chr2.fa	96850603	96874573	STARD7	63.95300325	9717
chr2.fa	96905977	96907701	LOC285033	3.147500974	76
chr2.fa	96915946	96931751	TMEM127	4.41579731	912
chr2.fa	96931884	96939900	CIAO1	20.93878748	3800
chr2.fa	96940074	96971297	SNRNP200	27.65922356	8966
chr2.fa	96991062	96994091	ITPRIPL1	0	0
chr2.fa	97001484	97041274	NCAPH	0	0
chr2.fa	97163383	97173846	NEURL3	0	0
chr2.fa	97202464	97218371	ARID5A	0	0
chr2.fa	97258907	97304115	KIAA1310	15.0309684	3572
chr2.fa	97308574	97370624	FER1L5	0.260864738	76
chr2.fa	97371667	97405813	LMAN2L	3.460627616	380
chr2.fa	97426639	97477628	CNNM4	2.817027827	608
chr2.fa	97464015	97464090	MIR3127	0	0
chr2.fa	97481991	97501121	CNNM3	1.031449835	228
chr2.fa	97503651	97509758	ANKRD23	1.309216296	152
chr2.fa	97513724	97523756	ANKRD39	7.528605275	304
chr2.fa	97525473	97535735	SEMA4C	2.855056702	456
chr2.fa	97541619	97652301	FAM178B	0	0
chr2.fa	97749323	97760582	FAHD2B	4.008376849	228
chr2.fa	97779233	97930257	ANKRD36	0.244852581	69
chr2.fa	98121261	98206428	ANKRD36B	0.564650955	152
chr2.fa	98262521	98264657	COX5B	177.3844638	4028
chr2.fa	98272402	98280561	ACTR1B	31.43809282	3192
chr2.fa	98330031	98356323	ZAP70	0	0
chr2.fa	98372801	98612354	TMEM131	3.053651937	912
chr2.fa	98703595	98929410	VWA3B	0	0
chr2.fa	98962618	99015064	CNGA3	0	0
chr2.fa	99061321	99207496	INPP4A	44.72062254	13837
chr2.fa	99215865	99224955	C2orf64	19.32200432	1444
chr2.fa	99225042	99234977	UNC50	5.920050584	304
chr2.fa	99235569	99347589	MGAT4A	14.12294562	2812
chr2.fa	99410309	99552684	C2orf55	8.737078411	1520
chr2.fa	99613724	99771187	TSGA10	4.85168383	836
chr2.fa	99758185	99767928	C2orf15	3.601178779	228
chr2.fa	99771418	99779613	LIPT1	2.94112205	228
chr2.fa	99785726	99797492	MITD1	1.857410311	76
chr2.fa	99797578	99814089	MRPL30	17.76348762	2052
chr2.fa	99858711	99871570	LYG2	0	0

chr2.fa	99900701	99917639	LYG1	0	0
chr2.fa	99935487	99952860	TXNDC9	8.83759918	608
chr2.fa	99953834	100016728	EIF5B	33.81789978	7144
chr2.fa	100016938	100106480	REV1	11.39198314	2425
chr2.fa	100163716	100759037	AFF3	8.186660485	3023
chr2.fa	100889753	100939195	LONRF2	2.186771502	1368
chr2.fa	101008322	101034091	CHST10	1.801145367	228
chr2.fa	101086944	101099742	NMS	0	0
chr2.fa	101179418	101193201	PDCL3	20.43929711	988
chr2.fa	101436613	101613287	NPAS2	20.6839273	3724
chr2.fa	101618691	101636155	RPL31	814.8793955	44080
chr2.fa	101623690	101767846	TBC1D8	23.60659089	4402
chr2.fa	101869345	101886778	C2orf29	24.16457011	2736
chr2.fa	101889398	101889511	SNORD89	0	0
chr2.fa	101892063	101925178	RNF149	3.416816573	456
chr2.fa	101964816	102003965	CREG2	0	0
chr2.fa	102013823	102091165	RFX8	0	0
chr2.fa	102314488	102511152	MAP4K4	14.003744	4712
chr2.fa	102608306	102644884	IL1R2	0	0
chr2.fa	102770402	102796334	IL1R1	37.88854613	8360
chr2.fa	102803433	102855811	IL1RL2	0	0
chr2.fa	102927962	102968497	IL1RL1	0	0
chr2.fa	102979097	103015217	IL18R1	0	0
chr2.fa	103035254	103069024	IL18RAP	0	0
chr2.fa	103089762	103150431	SLC9A4	15.11280832	2812
chr2.fa	103236166	103327809	SLC9A2	13.04434887	3192
chr2.fa	103333666	103353337	MFSD9	3.747512111	456
chr2.fa	103378490	103434138	TMEM182	2.355343942	380
chr2.fa	105050805	105129215	LOC150568	0	0
chr2.fa	105471969	105473471	POU3F3	2.249041005	152
chr2.fa	105654483	105716418	MRPS9	18.54786096	1216
chr2.fa	105858200	105859924	GPR45	0	0
chr2.fa	105883540	105946148	TGFBRAP1	15.38078957	2052
chr2.fa	105954013	105961984	C2orf49	7.622009529	304
chr2.fa	105977283	106055230	FHL2	6.006783106	608
chr2.fa	106361520	106510730	NCK2	17.1107698	2052
chr2.fa	106682113	106694609	C2orf40	0	0
chr2.fa	106709759	106810795	UXS1	8.110157953	760
chr2.fa	107002769	107007851	PLGLA	0	0
chr2.fa	107021136	107084801	RGPD3	0	0
chr2.fa	107418056	107503563	ST6GAL2	0	0
chr2.fa	108439520	108442583	LOC729121	0	0
chr2.fa	108443388	108509000	RGPD4	0	0

chr2.fa	108602995	108630443	SLC5A7	0	0
chr2.fa	108863651	108881807	SULT1C3	0	0
chr2.fa	108905095	108926371	SULT1C2	1.169777088	152
chr2.fa	108938694	108970254	SULT1C2P1	2.705609895	228
chr2.fa	108994421	109004270	SULT1C4	0	0
chr2.fa	109065577	109125854	GCC2	19.62223228	6156
chr2.fa	109150857	109303702	LIMS1	5.264441677	1292
chr2.fa	109335937	109402267	RANBP2	15.15395067	7980
chr2.fa	109403219	109492847	CCDC138	1.539391065	152
chr2.fa	109510927	109605828	EDAR	0	0
chr2.fa	109743784	109746575	LOC100287216	0	0
chr2.fa	109757946	109758044	MIR4265	0	0
chr2.fa	109930027	109930081	MIR4266	0	0
chr2.fa	109964130	110262207	SH3RF3	0.671398674	152
chr2.fa	110300376	110371783	10-Sep	19.73765325	2888
chr2.fa	110371911	110376564	ANKRD57	6.900128079	1444
chr2.fa	110663457	111223204	LIMS3	0	0
chr2.fa	110690665	111195997	LIMS3-LOC440895	0	0
chr2.fa	110827538	110827619	MIR4267	0	0
chr2.fa	110841447	110874143	MALL	1.10439411	152
chr2.fa	110880914	110962639	NPHP1	1.843844455	228
chr2.fa	110969106	110980517	NCRNA00116	15.6594456	1140
chr2.fa	111003215	111024135	LOC100507334	0	0
chr2.fa	111132686	111142113	LOC151009	0.774588136	76
chr2.fa	111271379	111271397	RGPD6	0	0
chr2.fa	111395409	111435684	BUB1	1.930576977	304
chr2.fa	111490150	111875799	ACOXL	8.546934036	912
chr2.fa	111878491	111926022	BCL2L11	8.26182867	1976
chr2.fa	112124591	112252692	LOC541471	0	0
chr2.fa	112526640	112641741	ANAPC1	6.012565274	1706
chr2.fa	112656191	112786945	MERTK	2.796790238	456
chr2.fa	112812800	112876895	TMEM87B	1.25450809	294
chr2.fa	112895962	112945791	FBLN7	0	0
chr2.fa	112973439	113012664	ZC3H8	6.32235605	456
chr2.fa	113033178	113097640	ZC3H6	4.247669653	2204
chr2.fa	113125946	113125964	RGPD8	0	0
chr2.fa	113239743	113290222	TTL	5.89247409	1368
chr2.fa	113299492	113334727	POLR1B	9.686688328	2356
chr2.fa	113342036	113346617	CHCHD5	27.62542012	836
chr2.fa	113399407	113401757	FLJ42351	0	0
chr2.fa	113403527	113421400	SLC20A1	22.64541664	3344
chr2.fa	113495444	113522254	CKAP2L	0	0
chr2.fa	113531492	113542971	IL1A	0	0

chr2.fa	113587337	113594356	IL1B	3.384792257	228
chr2.fa	113670548	113676458	IL1F7	0	0
chr2.fa	113735606	113743227	IL1F9	0	0
chr2.fa	113763449	113765621	IL1F6	0	0
chr2.fa	113779668	113810440	IL1F8	0	0
chr2.fa	113816215	113822320	IL1F5	0	0
chr2.fa	113825547	113833427	IL1F10	0	0
chr2.fa	113875470	113891593	IL1RN	81.58906078	7752
chr2.fa	113917421	114205429	LOC440839	0	0
chr2.fa	113931560	113960677	PSD4	34.86580655	7900
chr2.fa	113973574	114036498	PAX8	16.24344458	2888
chr2.fa	113993846	114024580	LOC654433	0	0
chr2.fa	114195268	114253781	CBWD2	5.550658998	456
chr2.fa	114256661	114258727	FOXD4L1	0	0
chr2.fa	114334959	114336429	FAM138B	0	0
chr2.fa	114341230	114356604	WASH2P	2.601530869	228
chr2.fa	114356614	114361294	DDX11L2	0	0
chr2.fa	114368816	114384715	RPL23AP7	1.804703625	152
chr2.fa	114384817	114400975	RABL2A	0	0
chr2.fa	114471933	114514400	SLC35F5	1.186901201	152
chr2.fa	114647537	114716167	ACTR3	37.87698179	4619
chr2.fa	114735246	114737145	LOC100499194	0	0
chr2.fa	114737562	114764887	LOC440900	0	0
chr2.fa	115199899	116602326	DPP10	1.087269996	304
chr2.fa	115901625	115918920	LOC389023	2.315313547	76
chr2.fa	118572255	118589953	DDX18	15.26314469	2584
chr2.fa	118673054	118771739	CCDC93	8.53737122	2660
chr2.fa	118846050	118867597	INSIG2	4.58036671	532
chr2.fa	119599747	119605759	EN1	0	0
chr2.fa	119699745	119752236	MARCO	0	0
chr2.fa	119913819	119916471	C1QL2	0	0
chr2.fa	119981384	120023227	STEAP3	1.165106875	228
chr2.fa	120060020	120124258	C2orf76	7.116514602	304
chr2.fa	120124504	120130122	DBI	336.0275865	18585
chr2.fa	120189446	120196096	TMEM37	4.007487285	304
chr2.fa	120197419	120282028	SCTR	0	0
chr2.fa	120302025	120414237	PCDP1	1.708408287	228
chr2.fa	120436743	120439694	TMEM177	3.767082526	228
chr2.fa	120517207	120735037	PTPN4	6.429770943	1140
chr2.fa	120770604	120936697	EPB41L5	10.60805458	4739
chr2.fa	120978854	120980984	TMEM185B	2.379362179	228
chr2.fa	121010414	121052286	RALB	71.01569921	7220
chr2.fa	121103719	121109383	INHBB	0.527734036	76

chr2.fa	121221911	121223925	LOC84931	0	0
chr2.fa	121554867	121750229	GLI2	0	0
chr2.fa	121974164	122042778	TFCP2L1	12.89890511	5386
chr2.fa	122095352	122407052	CLASP1	10.58959612	3876
chr2.fa	122288456	122288585	RNU4ATAC	0	0
chr2.fa	122484521	122494503	MKI67IP	9.724717203	760
chr2.fa	122513121	122525428	TSN	9.425601199	1444
chr2.fa	124782864	125672863	CNTNAP5	1.279638282	304
chr2.fa	127413684	127454246	GYPC	1.578087113	76
chr2.fa	127805607	127864864	BIN1	13.30854948	1596
chr2.fa	127941412	127963343	CYP27C1	0	0
chr2.fa	128014866	128051752	ERCC3	19.56240908	2419
chr2.fa	128062139	128100770	MAP3K2	15.32118876	3404
chr2.fa	128175996	128186822	PROC	0	0
chr2.fa	128238383	128284087	IWS1	32.0094155	4318
chr2.fa	128293378	128395303	MYO7B	0.251746704	76
chr2.fa	128395996	128439360	LIMS2	0.583998979	76
chr2.fa	128403439	128410213	GPR17	0	0
chr2.fa	128458597	128461407	SFT2D3	3.607628121	456
chr2.fa	128461808	128568761	WDR33	7.081376811	3572
chr2.fa	128603840	128615729	POLR2D	6.542523221	684
chr2.fa	128619207	128643514	AMMECR1L	7.785466974	1672
chr2.fa	128698791	128784869	SAP130	10.93563664	2052
chr2.fa	128848754	128953249	UGGT1	2.313089637	1140
chr2.fa	129023054	129076171	HS6ST1	3.849144835	684
chr2.fa	130680435	130691890	LOC389033	0	0
chr2.fa	130724165	130736664	LOC100131320	2.371578491	228
chr2.fa	130737235	130740311	RAB6C	0.613576993	76
chr2.fa	130783572	130808704	LOC440905	0	0
chr2.fa	130831108	130886795	POTEF	0	0
chr2.fa	130887196	130896861	LOC285103	0	0
chr2.fa	130896987	130902631	CCDC74B	7.336014599	444
chr2.fa	130908965	130939330	SMPD4	6.03636112	1064
chr2.fa	130939501	130948300	MZT2B	77.89647926	3268
chr2.fa	130949318	130956034	TUBA3E	0	0
chr2.fa	131095816	131099922	CCDC115	18.50560666	1368
chr2.fa	131100489	131104197	IMP4	20.94590399	988
chr2.fa	131113580	131132982	PTPN18	27.51244545	4539
chr2.fa	131278667	131357251	CFC1B	0	0
chr2.fa	131307469	131328418	LOC646743	0	0
chr2.fa	131328422	131339833	LOC150527	0	0
chr2.fa	131350335	131350352	CFC1	0	0
chr2.fa	131437623	131443435	C2orf14	0	0

chr2.fa	131486643	131487909	GPR148	0	0
chr2.fa	131513077	131525707	FAM123C	0	0
chr2.fa	131674224	131804836	ARHGEF4	0.794158551	152
chr2.fa	131805449	131851004	FAM168B	34.70390585	8436
chr2.fa	131862420	131907425	PLEKHB2	51.00339298	9880
chr2.fa	131975924	132022416	POTEE	0.491261899	76
chr2.fa	132036863	132057392	LOC440910	0	0
chr2.fa	132118065	132121731	LOC150786	0.921811032	152
chr2.fa	132160474	132165801	LOC389043	0	0
chr2.fa	132199734	132202467	LOC401010	0	0
chr2.fa	132233666	132240507	TUBA3D	1.093274556	76
chr2.fa	132241533	132250064	MZT2A	51.2173332	1596
chr2.fa	132250386	132279149	LOC150776	1.784688427	304
chr2.fa	132285492	132291239	CCDC74A	4.640189911	304
chr2.fa	132349268	132384999	POTEKP	0.576215292	76
chr2.fa	132480064	132524977	C2orf27A	1.386608392	76
chr2.fa	132552534	132559234	C2orf27B	0	0
chr2.fa	132905164	133015542	ANKRD30BL	0	0
chr2.fa	133014539	133014653	MIR663B	0	0
chr2.fa	133174147	133404169	GPR39	1.029893097	76
chr2.fa	133402337	133429070	LYPD1	0	0
chr2.fa	133429372	134326031	NCKAP5	5.118997909	1748
chr2.fa	134884696	134884763	MIR3679	0	0
chr2.fa	135011830	135206468	MGAT5	9.075780028	988
chr2.fa	135206960	135212192	LOC151162	2.583961974	608
chr2.fa	135213330	135476571	TMEM163	0	0
chr2.fa	135596186	135659602	ACMSD	14.60153122	759
chr2.fa	135624203	135676176	LOC100129961	0	0
chr2.fa	135676392	135716915	CCNT2	10.12657789	3192
chr2.fa	135722273	135782248	YSK4	0	0
chr2.fa	135809835	135928279	RAB3GAP1	24.64404528	5472
chr2.fa	135957574	136288806	ZRANB3	1.829611426	304
chr2.fa	136289083	136482839	R3HDM1	6.91058046	1444
chr2.fa	136422967	136423048	MIR128-1	0	0
chr2.fa	136499189	136542633	UBXN4	16.00059352	2888
chr2.fa	136545415	136594750	LCT	0	0
chr2.fa	136597196	136634011	MCM6	3.604737037	608
chr2.fa	136664254	136743222	DARS	29.31692668	3061
chr2.fa	136871919	136875725	CXCR4	0.843084589	76
chr2.fa	137748462	138435287	THSD7B	0	0
chr2.fa	138721808	138773934	HNMT	11.63060877	2185
chr2.fa	139259350	139330805	SPOPL	12.96962547	3344
chr2.fa	139426727	139537811	NXPH2	0	0

chr2.fa	139654894	139656744	LOC647012	0	0
chr2.fa	140988996	142889270	LRP1B	0.204377404	152
chr2.fa	143635195	143799885	KYNU	7.552845903	684
chr2.fa	143886899	144525921	ARHGAP15	0	0
chr2.fa	144703581	145090083	GTDC1	5.893141263	760
chr2.fa	145141942	145277958	ZEB2	0.28999797	152
chr2.fa	145425534	145834291	DKFZp686O1327	0	0
chr2.fa	147344625	147348558	PABPC1P2	0	0
chr2.fa	148602570	148687965	ACVR2A	8.773772939	1900
chr2.fa	148688394	148779173	ORC4	4.652421421	1355
chr2.fa	148778580	149271044	MBD5	4.2140886	1140
chr2.fa	149402560	149545136	EPC2	15.31874246	2508
chr2.fa	149632819	149883273	KIF5C	13.65592435	4256
chr2.fa	149894981	150071772	LYPD6B	7.521488761	532
chr2.fa	150186499	150330659	LYPD6	3.923423456	760
chr2.fa	150426147	150444330	MMADHC	61.16132801	3952
chr2.fa	151324710	151344180	RND3	8.192442653	988
chr2.fa	152104728	152118389	RBM43	3.886506537	684
chr2.fa	152126982	152146430	NMI	7.999407194	532
chr2.fa	152214106	152236562	TNFAIP6	0	0
chr2.fa	152266397	152333860	RIF1	6.294334774	2881
chr2.fa	152341853	152591001	NEB	0.771029879	912
chr2.fa	152657480	152685009	ARL5A	13.33367967	1900
chr2.fa	152689286	152955593	CACNB4	1.212698566	456
chr2.fa	152973315	153032506	STAM2	6.818732944	1748
chr2.fa	153191751	153506348	FMNL2	1.818936654	456
chr2.fa	153508107	153573975	PRPF40A	13.24361128	4483
chr2.fa	153574423	153617767	ARL6IP6	0	0
chr2.fa	154333852	154335322	RPRM	0	0
chr2.fa	154728426	155310489	GALNT13	0	0
chr2.fa	155555093	155713014	KCNJ3	1.75444324	228
chr2.fa	157180944	157189287	NR4A2	2.871958424	456
chr2.fa	157291965	157442915	GPD2	18.94749774	5244
chr2.fa	158114340	158167913	GALNT5	0	0
chr2.fa	158175125	158184146	ERMN	0	0
chr2.fa	158271131	158300604	CYTIP	0.764802929	76
chr2.fa	158383279	158485399	ACVR1C	0	0
chr2.fa	158592958	158732374	ACVR1	4.887933576	684
chr2.fa	158851691	158992666	UPP2	0.655386517	76
chr2.fa	159027869	159313265	CCDC148	2.730517696	380
chr2.fa	159313476	159537940	PKP4	27.92720482	5764
chr2.fa	159651829	159672496	DAPL1	6.225393539	152
chr2.fa	159825146	160089170	TANC1	21.1851968	7136

chr2.fa	160092304	160143236	WDSUB1		9.840805347	912
chr2.fa	160175490	160473059	BAZ2B		23.20561977	8499
chr2.fa	160569010	160625084		7-Mar	22.8033143	3556
chr2.fa	160654643	160654766	CD302		0	0
chr2.fa	160659868	160661733	LY75		0.905798874	76
chr2.fa	160797260	160919126	PLA2R1		1.276747198	380
chr2.fa	160958233	161056590	ITGB6		5.640949777	608
chr2.fa	161128662	161350318	RBMS1		7.098278533	1368
chr2.fa	161993466	162092682	TANK		10.22910018	988
chr2.fa	162164786	162268226	PSMD14		29.5657823	2280
chr2.fa	162272620	162281573	TBR1		0	0
chr2.fa	162480845	162841786	SLC4A10		0	0
chr2.fa	162848755	162931052	DPP4		36.03313734	6319
chr2.fa	162999385	163008914	GCG		1.312329771	76
chr2.fa	163027200	163100045	FAP		0.608017216	76
chr2.fa	163123589	163175039	IFIH1		6.422654428	988
chr2.fa	163200583	163219148	GCA		5.240868222	760
chr2.fa	163227917	163695240	KCNH7		0	0
chr2.fa	164464118	164592513	FIGN		3.727052131	760
chr2.fa	165349323	165478360	GRB14		0	0
chr2.fa	165541258	165697928	COBLL1		51.51911789	11400
chr2.fa	165544153	165544287	SNORA70F		0	0
chr2.fa	165754709	165812035	SLC38A11		0	0
chr2.fa	165944030	166060577	SCN3A		0.183472642	76
chr2.fa	166095912	166248820	SCN2A		0.735669697	304
chr2.fa	166326157	166545917	CSRNP3		0.421208708	228
chr2.fa	166604313	166650803	GALNT3		2.586630667	380
chr2.fa	166730453	166810348	TTC21B		6.90435351	1520
chr2.fa	166845670	166930149	SCN1A		0	0
chr2.fa	167051697	167232497	SCN9A		0.173242652	76
chr2.fa	167261539	167343478	SCN7A		0	0
chr2.fa	167744997	168116261	XIRP2		0	0
chr2.fa	168675182	168727366	B3GALT1		0	0
chr2.fa	168810530	169104105	STK39		44.05700755	6486
chr2.fa	169312835	169631152	LASS6		1.065920453	300
chr2.fa	169643049	169721849	NOSTRIN		4.196964486	532
chr2.fa	169727401	169746944	SPC25		0	0
chr2.fa	169757750	169766510	G6PC2		0	0
chr2.fa	169779449	169887833	ABCB11		0	0
chr2.fa	169921299	169952677	DHRS9		0	0
chr2.fa	169983619	170219122	LRP2		3.222446768	2280
chr2.fa	170336006	170363165	BBS5		3.747512111	532
chr2.fa	170366212	170382772	KBTBD10		0	0

chr2.fa	170386263	170430424	FASTKD1	2.813247178	380
chr2.fa	170440850	170494254	PPIG	14.03710266	1708
chr2.fa	170501935	170550931	C2orf77	1.558516698	152
chr2.fa	170554375	170558218	PHOSPHO2	1.918567858	76
chr2.fa	170590356	170608396	KLHL23	5.391871766	988
chr2.fa	170655389	170668575	SSB	38.3631287	2660
chr2.fa	170668967	170681353	METTL5	22.21264359	836
chr2.fa	170684018	170940639	UBR3	11.05105761	3952
chr2.fa	171034655	171511674	MYO3B	6.404195968	1824
chr2.fa	171568949	171571077	LOC440925	0	0
chr2.fa	171571857	171574498	SP5	0	0
chr2.fa	171673200	171717659	GAD1	1.381938179	228
chr2.fa	171785711	171823640	GORASP2	36.52239772	4104
chr2.fa	171847333	172087824	TLK1	14.22746943	3859
chr2.fa	172173913	172291312	METTL8	1.459997449	532
chr2.fa	172290761	172341562	DCAF17	1.457551147	380
chr2.fa	172378866	172414643	CYBRD1	1.551622574	304
chr2.fa	172543982	172604919	DYNC1I2	31.82883395	3724
chr2.fa	172639915	172750813	SLC25A12	4.230767931	760
chr2.fa	172778935	172848600	HAT1	16.19318419	1216
chr2.fa	172864804	172945587	METAP1D	0	0
chr2.fa	172950208	172954401	DLX1	0	0
chr2.fa	172964166	172967478	DLX2	0	0
chr2.fa	173292314	173371181	ITGA6	6.109083003	1596
chr2.fa	173420779	173463862	PDK1	4.062862664	836
chr2.fa	173587918	173600934	LOC91149	0	0
chr2.fa	173600525	173917620	RAPGEF4	0.356715294	76
chr2.fa	173940565	174132737	ZAK	1.682166139	731
chr2.fa	174062441	174146764	LOC339751	0	0
chr2.fa	174219561	174233718	CDCA7	0	0
chr2.fa	174771187	174830430	SP3	13.28964624	3800
chr2.fa	174937175	175113365	OLA1	10.8642491	2128
chr2.fa	175199821	175202268	SP9	0	0
chr2.fa	175212878	175260443	CIR1	13.08549122	1055
chr2.fa	175260457	175294303	SCRN3	7.750551574	1064
chr2.fa	175296375	175351811	GPR155	0.904686919	304
chr2.fa	175424302	175547627	WIPF1	3.587612924	760
chr2.fa	175612323	175629200	CHRNA1	0	0
chr2.fa	175664042	175870170	CHN1	1.090605863	133
chr2.fa	175939006	176032897	ATF2	32.82670273	3116
chr2.fa	176032361	176032437	MIR933	0	0
chr2.fa	176040986	176046490	ATP5G3	53.15814016	6308
chr2.fa	176790410	176867018	KIAA1715	2.63867018	684

chr2.fa	176944835	176948690	EVX2	1.045238082	76
chr2.fa	176957532	176960666	HOXD13	6.536963444	684
chr2.fa	176964530	176965488	HOXD12	0	0
chr2.fa	176972084	176974316	HOXD11	15.0185145	988
chr2.fa	176981492	176984670	HOXD10	1.873867251	152
chr2.fa	176987413	176989645	HOXD9	1.79336168	152
chr2.fa	176994422	176997423	HOXD8	7.71474661	912
chr2.fa	177015031	177015140	MIR10B	0	0
chr2.fa	177016113	177017949	HOXD4	3.912526293	228
chr2.fa	177028805	177037826	HOXD3	2.940677268	304
chr2.fa	177037924	177042222	LOC401022	7.388721285	1216
chr2.fa	177053307	177055635	HOXD1	2.770770482	228
chr2.fa	177134123	177202753	MTX2	30.10374633	2155
chr2.fa	177465708	177465780	MIR1246	0	0
chr2.fa	178077422	178088685	HNRNPA3	16.87837112	4408
chr2.fa	178095033	178129859	NFE2L2	34.91673411	5244
chr2.fa	178120673	178120738	MIR3128	0	0
chr2.fa	178148236	178257419	LOC100130691	2.921996417	684
chr2.fa	178257471	178408564	AGPS	5.721455349	1972
chr2.fa	178414881	178417524	TTC30B	4.474730947	532
chr2.fa	178479026	178483694	TTC30A	3.258029341	684
chr2.fa	178487977	178973066	PDE11A	7.769010034	3420
chr2.fa	178977182	178994382	RBM45	1.894772013	152
chr2.fa	179059374	179260875	OSBPL6	2.109601797	380
chr2.fa	179246805	179541009	MIR548N	0	0
chr2.fa	179296141	179315958	PRKRA	12.49993551	1064
chr2.fa	179316163	179326110	DFNB59	0	0
chr2.fa	179328391	179343355	FKBP7	0.582664633	76
chr2.fa	179345199	179369782	PLEKHA3	6.683519166	742
chr2.fa	179390718	179672150	TTN	0.166126138	836
chr2.fa	179694484	179914786	CCDC141	0.740784692	304
chr2.fa	179966419	180129350	SESTD1	3.72060279	1748
chr2.fa	180306711	180726232	ZNF385B	13.32233773	2280
chr2.fa	180725563	180725635	MIR1258	0	0
chr2.fa	180809604	180871780	CWC22	11.33994363	1672
chr2.fa	181845112	181928150	UBE2E3	19.10873127	1465
chr2.fa	182321619	182401400	ITGA4	0	0
chr2.fa	182402469	182521834	CERKL	0.500824715	50
chr2.fa	182541194	182545381	NEUROD1	0	0
chr2.fa	182756472	182795464	SSFA2	14.7965682	3521
chr2.fa	182850551	182982419	PPP1R1C	0	0
chr2.fa	183007183	183387465	PDE1A	4.373765396	532
chr2.fa	183580999	183643255	DNAJC10	8.061899088	1520

chr2.fa	183698005	183731498	FRZB	0	0
chr2.fa	183789605	183903229	NCKAP1	39.82579484	8295
chr2.fa	183943287	183964722	DUSP19	0.630033933	152
chr2.fa	183989083	184026408	NUP35	3.105024277	228
chr2.fa	185463093	185804214	ZNF804A	0	0
chr2.fa	186603355	186698016	FSIP2	0.160566361	152
chr2.fa	187350885	187374087	ZC3H15	27.73372458	2660
chr2.fa	187454790	187545628	ITGAV	2.714727929	880
chr2.fa	187558789	187628512	FAM171B	0	0
chr2.fa	187692207	187713897	ZSWIM2	0	0
chr2.fa	188207849	188313021	CALCRL	0	0
chr2.fa	188328958	188419219	TFPI	1.632572928	304
chr2.fa	189157390	189460652	GULP1	9.65622075	1498
chr2.fa	189162219	189162315	MIR561	0	0
chr2.fa	189598465	189654831	DIRC1	0	0
chr2.fa	189839099	189877472	COL3A1	0	0
chr2.fa	189842818	189842886	MIR1245	0	0
chr2.fa	189860356	189860418	MIR3606	0	0
chr2.fa	189896641	190044605	COL5A2	0.401193511	125
chr2.fa	189997762	189997837	MIR3129	0	0
chr2.fa	190306159	190340264	WDR75	11.37730533	1368
chr2.fa	190425316	190445537	SLC40A1	19.14342428	2888
chr2.fa	190526125	190535557	ASNSD1	11.62816247	1292
chr2.fa	190540711	190611376	ANKAR	1.148205153	228
chr2.fa	190611386	190627924	OSGEPL1	2.189440195	228
chr2.fa	190634993	190647849	ORMDL1	9.755629563	912
chr2.fa	190648811	190742355	PMS1	0.988973139	152
chr2.fa	190920426	190927455	MSTN	0	0
chr2.fa	191002486	191068210	C2orf88	0.377842447	76
chr2.fa	191069360	191184771	HIBCH	32.96302846	2874
chr2.fa	191208196	191236391	INPP1	4.688448776	456
chr2.fa	191273081	191367041	MFSD6	4.228099238	912
chr2.fa	191371619	191399468	TMEM194B	0.46479736	76
chr2.fa	191513848	191557492	NAB1	9.076669592	1824
chr2.fa	191745547	191829776	GLS	19.50147392	3804
chr2.fa	191833762	191878976	STAT1	37.79136123	7788
chr2.fa	191894306	192015925	STAT4	0	0
chr2.fa	192110107	192290115	MYO1B	13.91968017	3189
chr2.fa	192542798	192553248	OBFC2A	1.296095222	228
chr2.fa	192699032	192712006	SDPR	0	0
chr2.fa	192814747	193059644	TMEFF2	1.868529865	152
chr2.fa	193614571	193641625	PCGEM1	0	0
chr2.fa	196521532	196602426	SLC39A10	5.200615436	1292

chr2.fa	196602427	196933536	DNAH7	0.818176788	456
chr2.fa	196998307	197036336	STK17B	7.962045493	1900
chr2.fa	197063977	197457335	HECW2	0	0
chr2.fa	197504356	197597530	CCDC150	0	0
chr2.fa	197565359	197577736	LOC100130452	0	0
chr2.fa	197629103	197664425	GTF3C3	14.39670904	1900
chr2.fa	197670009	197675000	C2orf66	0	0
chr2.fa	197697728	197791454	PGAP1	0.456346499	228
chr2.fa	197851386	198175521	ANKRD44	0.786152472	228
chr2.fa	198256698	198299771	SF3B1	55.11028907	11020
chr2.fa	198318231	198339851	COQ10B	11.98710167	1064
chr2.fa	198351308	198364640	HSPD1	85.92857795	8968
chr2.fa	198364721	198368187	HSPE1	94.79486558	3572
chr2.fa	198380320	198417515	MOBKL3	8.588298778	1140
chr2.fa	198435527	198540584	RFTN2	0	0
chr2.fa	198570028	198573114	MARS2	2.737634211	380
chr2.fa	198591603	198650938	BOLL	0.550640317	76
chr2.fa	198669426	199014608	PLCL1	2.81346957	836
chr2.fa	200134223	200329831	SATB2	5.000463463	1368
chr2.fa	200332821	200337481	FLJ32063	0	0
chr2.fa	200625259	200715896	LOC348751	1.775348002	76
chr2.fa	200775979	200792996	C2orf69	7.25395229	1216
chr2.fa	200793634	200820219	C2orf60	0.988305965	228
chr2.fa	200820040	200828847	C2orf47	17.28734831	988
chr2.fa	201170604	201346986	SPATS2L	18.64059804	5776
chr2.fa	201353684	201374792	KCTD18	10.9340799	1444
chr2.fa	201390865	201448818	SGOL2	0	0
chr2.fa	201450731	201536217	AOX1	51.01962753	11317
chr2.fa	201560446	201658941	AOX2P	0	0
chr2.fa	201676647	201688560	BZW1	59.8896958	7535
chr2.fa	201717732	201729467	CLK1	17.58512997	1893
chr2.fa	201735679	201753849	PPIL3	9.301284584	532
chr2.fa	201754050	201768655	NIF3L1	9.713597649	760
chr2.fa	201774894	201828424	ORC2	5.703441671	804
chr2.fa	201838441	201936392	FAM126B	3.078559738	1292
chr2.fa	201936462	201950473	NDUFB3	42.53407343	1444
chr2.fa	201980816	202028841	CFLAR	30.39663538	3525
chr2.fa	202047621	202094110	CASP10	3.985692959	1140
chr2.fa	202098166	202152434	CASP8	11.09108801	1748
chr2.fa	202153147	202222101	ALS2CR12	0	0
chr2.fa	202241930	202316319	TRAK2	17.60870343	5168
chr2.fa	202316392	202345574	STRADB	11.76804646	1216
chr2.fa	202352144	202483905	ALS2CR11	0	0

chr2.fa	202484907	202508224	ALS2CR4	0.92559168	228
chr2.fa	202509597	202563417	MPP4	0	0
chr2.fa	202564986	202645895	ALS2	6.484256757	2432
chr2.fa	202671198	202758263	CDK15	0	0
chr2.fa	202899310	202903160	FZD7	0.877777598	152
chr2.fa	203070903	203103322	SUMO1	34.31249754	2356
chr2.fa	203130515	203168384	NOP58	18.06282601	1596
chr2.fa	203141154	203141241	SNORD70	0	0
chr2.fa	203156040	203156151	SNORD11B	0	0
chr2.fa	203157774	203157857	SNORD11	0	0
chr2.fa	203241050	203432474	BMPR2	5.041161031	2736
chr2.fa	203499901	203634480	FAM117B	13.17467005	3420
chr2.fa	203637873	203736371	ICA1L	0.393409823	152
chr2.fa	203745323	203776949	WDR12	8.624770915	885
chr2.fa	203776978	203851060	ALS2CR8	1.972164109	532
chr2.fa	203879602	204082717	NBEAL1	11.75537017	4788
chr2.fa	204103164	204170563	CYP20A1	2.316870285	1140
chr2.fa	204193003	204296892	ABI2	4.955762856	1444
chr2.fa	204298539	204400058	RAPH1	7.662484706	3572
chr2.fa	204571198	204602557	CD28	0	0
chr2.fa	204732509	204738683	CTLA4	0	0
chr2.fa	204801471	204826298	ICOS	0	0
chr2.fa	205410516	206480537	PARD3B	21.65177328	3724
chr2.fa	206547224	206662857	NRP2	0.223280646	76
chr2.fa	206858445	206950906	INO80D	4.902166605	3116
chr2.fa	206980297	206981296	LOC100329109	0	0
chr2.fa	206987803	207024243	NDUFS1	40.19474164	6456
chr2.fa	207024318	207027653	EEF1B2	116.8940897	4636
chr2.fa	207026605	207026674	SNORD51	0	0
chr2.fa	207026952	207027083	SNORA41	0	0
chr2.fa	207040042	207082771	GPR1	0	0
chr2.fa	207139523	207179148	ZDBF2	1.165996439	532
chr2.fa	207308368	207482679	ADAM23	0	0
chr2.fa	207507142	207514173	LOC200726	0	0
chr2.fa	207516345	207583120	DYTN	0	0
chr2.fa	207602489	207630050	MDH1B	0.725439707	76
chr2.fa	207630112	207660911	FASTKD2	4.635297308	1444
chr2.fa	207804278	207834198	CPO	0	0
chr2.fa	207945529	208030614	KLF7	8.249819552	608
chr2.fa	207974711	207974797	MIR2355	0	0
chr2.fa	208394616	208470284	CREB1	6.740673674	2964
chr2.fa	208473839	208489973	FAM119A	2.054448809	456
chr2.fa	208576264	208620896	CCNYL1	7.716303348	1357

chr2.fa	208627310	208634143	FZD5	2.317315067	684
chr2.fa	208686012	208890284	PLEKHM3	7.927797266	3496
chr2.fa	208986331	208989313	CRYGD	0	0
chr2.fa	208992861	208994554	CRYGC	0	0
chr2.fa	209007297	209010877	CRYGB	0	0
chr2.fa	209025464	209028297	CRYGA	0	0
chr2.fa	209030071	209054773	C2orf80	0	0
chr2.fa	209100953	209119806	IDH1	436.8757149	45752
chr2.fa	209130991	209223475	PIKFYVE	12.79571564	6004
chr2.fa	209271556	209359231	PTH2R	0	0
chr2.fa	210288771	210598834	MAP2	1.691951347	760
chr2.fa	210636717	210864024	UNC80	1.744658033	1064
chr2.fa	210867352	210885434	RPE	17.95941416	1368
chr2.fa	210885951	211036051	C2orf67	13.8142668	3040
chr2.fa	211052716	211090215	ACADL	13.57564117	1520
chr2.fa	211154868	211179895	MYL1	0	0
chr2.fa	211295973	211341499	LANCL1	15.03185797	3116
chr2.fa	211342409	211543831	CPS1	0.549750753	152
chr2.fa	211482295	211484599	CPS1-IT	0	0
chr2.fa	212240442	213403352	ERBB4	0.425211747	228
chr2.fa	213290987	213291084	MIR548F2	0	0
chr2.fa	213864411	214016333	IKZF2	9.276154392	4028
chr2.fa	214149116	215275225	SPAG16	11.6822035	1142
chr2.fa	215276461	215440653	VWC2L	0	0
chr2.fa	215593275	215674428	BARD1	8.470431505	988
chr2.fa	215796266	216003151	ABCA12	0	0
chr2.fa	216176679	216214496	ATIC	41.36207243	3876
chr2.fa	216225179	216300791	FN1	0	0
chr2.fa	216476286	216708259	LOC646324	0	0
chr2.fa	216807314	216878346	MREG	5.786393544	836
chr2.fa	216903111	216946539	PECR	8.117052076	684
chr2.fa	216946589	216967506	TMEM169	0	0
chr2.fa	216974020	217071016	XRCC5	74.77655478	11533
chr2.fa	217081612	217084915	PKI55	0	0
chr2.fa	217122585	217236750	4-Mar	0	0
chr2.fa	217277137	217347774	SMARCAL1	11.13956926	1662
chr2.fa	217363520	217366188	RPL37A	4047.51768	76076
chr2.fa	217498127	217529158	IGFBP2	5.913823634	380
chr2.fa	217536828	217560272	IGFBP5	2.438073424	684
chr2.fa	217724182	217724782	TNP1	0	0
chr2.fa	218148746	218621316	DIRC3	0	0
chr2.fa	218664512	218808796	TNS1	9.210771414	4256
chr2.fa	218899657	218955304	RUFY4	0	0

chr2.fa	218923878	218926013	CXCR2P1	0	0
chr2.fa	218990013	219001976	CXCR2	0	0
chr2.fa	219027568	219031716	CXCR1	0	0
chr2.fa	219081874	219119071	ARPC2	107.7435863	7335
chr2.fa	219125738	219128582	GPBAR1	0.843751762	76
chr2.fa	219128852	219134893	AAMP	63.15751035	5092
chr2.fa	219135115	219211516	PNKD	24.82218054	4180
chr2.fa	219138917	219157280	TMBIM1	43.49369094	4594
chr2.fa	219221579	219232817	C2orf62	2.555051133	152
chr2.fa	219246752	219261617	SLC11A1	0.877555207	152
chr2.fa	219264478	219270664	CTDSP1	60.22595112	7144
chr2.fa	219267369	219267445	MIR26B	0	0
chr2.fa	219283838	219314248	VIL1	1.851850534	228
chr2.fa	219314974	219433084	USP37	3.787764897	1368
chr2.fa	219433678	219458999	RQCD1	20.65768515	836
chr2.fa	219472632	219501904	PLCD4	0	0
chr2.fa	219502640	219524261	ZNF142	3.398580504	905
chr2.fa	219524379	219528166	BCS1L	5.081636208	380
chr2.fa	219528587	219536761	RNF25	5.483941674	380
chr2.fa	219536782	219567440	STK36	4.482292244	988
chr2.fa	219575568	219620138	TTLL4	4.059749189	912
chr2.fa	219646472	219680016	CYP27A1	2.948460956	304
chr2.fa	219687106	219696512	PRKAG3	0	0
chr2.fa	219724546	219738954	WNT6	0	0
chr2.fa	219745255	219758651	WNT10A	0	0
chr2.fa	219824398	219826877	CDK5R2	0	0
chr2.fa	219841006	219842644	LOC151300	0	0
chr2.fa	219845809	219850379	FEV	10.79397352	912
chr2.fa	219854912	219858127	CRYBA2	0	0
chr2.fa	219866367	219866430	MIR375	0	0
chr2.fa	219867576	219906245	CCDC108	0	0
chr2.fa	219919142	219925238	IHH	0.815730486	76
chr2.fa	219923410	219923472	MIR3131	0	0
chr2.fa	219940046	220025587	NHEJ1	17.68120292	1672
chr2.fa	220026181	220034817	SLC23A3	4.194073402	456
chr2.fa	220036619	220041702	C2orf24	35.84632883	3343
chr2.fa	220042939	220050197	FAM134A	9.524120448	1976
chr2.fa	220071538	220074370	ZFAND2B	14.33110367	830
chr2.fa	220074488	220083712	ABCB6	0.559980743	76
chr2.fa	220084102	220094361	ATG9A	2.687818609	456
chr2.fa	220094479	220101391	ANKZF1	12.81884432	1520
chr2.fa	220101503	220110131	GLB1L	2.032209701	228
chr2.fa	220110192	220115000	STK16	17.62894101	2432

chr2.fa	220115060	220118638	TUBA4A	11.91104392	760
chr2.fa	220117965	220136910	TUBA4B	0	0
chr2.fa	220144040	220151622	DNAJB2	48.90468834	6872
chr2.fa	220154345	220174295	PTPRN	0	0
chr2.fa	220158833	220158922	MIR153-1	0	0
chr2.fa	220192131	220197899	RESP18	0	0
chr2.fa	220238180	220252662	DNPEP	29.8101901	3268
chr2.fa	220283099	220291461	DES	0	0
chr2.fa	220299700	220358354	SPEG	0.290442752	152
chr2.fa	220363587	220371718	GMPPA	17.16369887	1444
chr2.fa	220378892	220403494	ACCN4	0	0
chr2.fa	220403669	220408487	CHPF	1.596545573	228
chr2.fa	220408745	220415317	TMEM198	0.762134236	76
chr2.fa	220413795	220413869	MIR3132	0	0
chr2.fa	220415450	220436268	OBSL1	6.272762839	2052
chr2.fa	220436954	220440435	INHA	0	0
chr2.fa	220462596	220481173	STK11IP	3.254693475	527
chr2.fa	220492292	220506702	SLC4A3	1.178895122	228
chr2.fa	220771223	220771286	MIR4268	0	0
chr2.fa	222282747	222437010	EPHA4	0	0
chr2.fa	223064606	223163715	PAX3	0	0
chr2.fa	223162866	223169936	CCDC140	0	0
chr2.fa	223289322	223423617	SGPP2	0	0
chr2.fa	223436162	223520827	FARSB	15.89495775	1596
chr2.fa	223536457	223574649	MOGAT1	0	0
chr2.fa	223725732	223808119	ACSL3	9.301506975	1824
chr2.fa	223916862	223920355	KCNE4	0	0
chr2.fa	224461658	224467217	SCG2	0	0
chr2.fa	224620047	224702319	AP1S3	6.757353005	1216
chr2.fa	224740065	224810052	WDFY1	23.77894398	4926
chr2.fa	224822121	224832431	MRPL44	16.43759199	1292
chr2.fa	224839765	224904036	SERPINE2	0	0
chr2.fa	225243415	225266711	FAM124B	0	0
chr2.fa	225334869	225450110	CUL3	11.7095576	3572
chr2.fa	225629807	225907330	DOCK10	0.682295837	228
chr2.fa	226265602	226518734	KIAA1486	0	0
chr2.fa	227596033	227663506	IRS1	2.708500979	1064
chr2.fa	227700671	227863923	RHBDD1	9.832354486	2280
chr2.fa	227867427	228029275	COL4A4	0.653829779	304
chr2.fa	228029281	228179508	COL4A3	1.461109404	532
chr2.fa	228192228	228222549	MFF	25.21381123	2280
chr2.fa	228226874	228244022	TM4SF20	0	0
chr2.fa	228336888	228425938	AGFG1	4.835671672	1900

chr2.fa	228474806	228498036	C2orf83	0	0
chr2.fa	228549926	228582745	SLC19A3	0	0
chr2.fa	228678558	228682280	CCL20	0	0
chr2.fa	228736327	228789026	WDR69	0	0
chr2.fa	228844670	229046361	SPHKAP	0	0
chr2.fa	229888689	230136057	PID1	1.796697546	228
chr2.fa	230222345	230579286	DNER	0	0
chr2.fa	230631930	230786655	TRIP12	69.37089477	20051
chr2.fa	230787207	230877825	FBXO36	3.002057206	380
chr2.fa	230899690	230933715	SLC16A14	0.384291788	76
chr2.fa	231033645	231090444	SP110	5.989658993	836
chr2.fa	231090445	231177930	SP140	0.473915394	76
chr2.fa	231191894	231268445	SP140L	5.701440151	684
chr2.fa	231280871	231410317	SP100	12.18147148	3774
chr2.fa	231577557	231685790	CAB39	42.42843766	8280
chr2.fa	231729621	231743963	ITM2C	15.49131794	1444
chr2.fa	231772043	231789941	GPR55	0	0
chr2.fa	231849083	231860747	LOC348761	0	0
chr2.fa	231860839	231871999	SPATA3	0	0
chr2.fa	231902281	231914427	C2orf72	5.194610877	836
chr2.fa	231921578	232037540	PSMD1	28.3390731	4237
chr2.fa	231972950	231989824	HTR2B	0	0
chr2.fa	232063342	232209914	ARMC9	0	0
chr2.fa	232260335	232265875	B3GNT7	0	0
chr2.fa	232319459	232329205	NCL	156.1403334	19076
chr2.fa	232320511	232320647	SNORA75	0	0
chr2.fa	232321155	232321234	SNORD20	0	0
chr2.fa	232325079	232325153	SNORD82	0	0
chr2.fa	232373137	232379050	C2orf52	0	0
chr2.fa	232387871	232395182	NMUR1	0	0
chr2.fa	232457575	232458994	C2orf57	0	0
chr2.fa	232573235	232578250	PTMA	211.8348438	10716
chr2.fa	232597147	232645974	PDE6D	9.070665033	456
chr2.fa	232651162	232673434	COPS7B	6.839415314	608
chr2.fa	232756952	232757008	MIR1471	0	0
chr2.fa	232790135	232790959	NPPC	0	0
chr2.fa	232826293	233201908	DIS3L2	8.204674162	1292
chr2.fa	233243348	233247599	ALPP	0	0
chr2.fa	233250460	233251754	ECEL1P2	1.305213256	76
chr2.fa	233271552	233275424	ALPPL2	0	0
chr2.fa	233320833	233324742	ALPI	0	0
chr2.fa	233344537	233352532	ECEL1	0	0
chr2.fa	233385173	233390425	LOC646960	0	0

chr2.fa	233390922	233400205	CHRND	0	0
chr2.fa	233404437	233411038	CHRNG	0	0
chr2.fa	233412779	233415226	TIGD1	1.380826224	152
chr2.fa	233415357	233433920	EIF4E2	28.47673318	1292
chr2.fa	233470767	233547491	EFHD1	25.66949056	2432
chr2.fa	233562015	233725289	GIGYF2	27.97079347	10048
chr2.fa	233630512	233641275	KCNJ13	0	0
chr2.fa	233734994	233741107	C2orf82	0	0
chr2.fa	233743396	233877951	NGEF	1.47267374	228
chr2.fa	233897382	233899767	NEU2	0	0
chr2.fa	233925036	234116549	INPP5D	4.895939655	1064
chr2.fa	234160217	234204320	ATG16L1	21.8408057	3344
chr2.fa	234184372	234184649	SCARNA5	0	0
chr2.fa	234197322	234197587	SCARNA6	0	0
chr2.fa	234216309	234255701	SAG	0	0
chr2.fa	234263153	234380743	DGKD	8.719064733	2508
chr2.fa	234384165	234474236	USP40	18.3550479	4636
chr2.fa	234526291	234527208	UGT1A8	0	0
chr2.fa	234545123	234681951	UGT1A10	0	0
chr2.fa	234580544	234581435	UGT1A9	5.684538429	228
chr2.fa	234590584	234591438	UGT1A7	0	0
chr2.fa	234600321	234602511	UGT1A6	4.424470562	228
chr2.fa	234621638	234622504	UGT1A5	0	0
chr2.fa	234627438	234628333	UGT1A4	0	0
chr2.fa	234637773	234638639	UGT1A3	0	0
chr2.fa	234651396	234652661	DNAJB3	0	0
chr2.fa	234668919	234669797	UGT1A1	0	0
chr2.fa	234745486	234763212	HJURP	1.108397149	152
chr2.fa	234774090	234777055	MSL3L2	4.246557697	380
chr2.fa	234826043	234928166	TRPM8	27.82156905	7032
chr2.fa	234959346	234985776	SPP2	0	0
chr2.fa	235401686	235405693	ARL4C	7.590652387	1368
chr2.fa	235860628	235964358	SH3BP4	16.95709756	3952
chr2.fa	236402736	237034120	AGAP1	10.836895	2204
chr2.fa	237074307	237076652	GBX2	0	0
chr2.fa	237103515	237172988	ASB18	0	0
chr2.fa	237232794	237416092	IQCA1	1.060360676	152
chr2.fa	237478380	237490994	CXCR7	1.609666646	152
chr2.fa	237994084	238007489	COPS8	11.56478101	1208
chr2.fa	238232655	238322850	COL6A3	0	0
chr2.fa	238395878	238463961	MLPH	325.9425957	55049
chr2.fa	238475217	238475818	PRLH	0	0
chr2.fa	238482965	238499769	RAB17	12.68274098	1140

chr2.fa	238536224	238690290	LRRFIP1	19.56974798	6840
chr2.fa	238707388	238751451	RBM44	0	0
chr2.fa	238768187	238820755	RAMP1	1.859411831	76
chr2.fa	238875700	238950682	UBE2F	33.75340636	2052
chr2.fa	238969632	239008054	SCLY	3.435275033	380
chr2.fa	239008951	239041928	ESPNL	0	0
chr2.fa	239047363	239061547	KLHL30	0	0
chr2.fa	239079043	239112324	ILKAP	12.8842273	836
chr2.fa	239133754	239140318	LOC151174	0	0
chr2.fa	239140327	239142985	LOC643387	0.63559371	76
chr2.fa	239146908	239148681	HES6	3.717489315	228
chr2.fa	239152679	239197207	PER2	6.129542983	1748
chr2.fa	239229185	239309541	TRAF3IP1	13.16977744	2473
chr2.fa	239335626	239360891	ASB1	6.925925445	2128
chr2.fa	239756673	239795893	TWIST2	0	0
chr2.fa	239840998	239847965	FLJ43879	0	0
chr2.fa	239969864	240322643	HDAC4	5.837321102	2356
chr2.fa	240115027	240117153	MGC16025	0	0
chr2.fa	240227157	240227240	MIR4269	0	0
chr2.fa	240896789	240964819	NDUFA10	20.21201342	4467
chr2.fa	240968908	240969846	OR6B2	0	0
chr2.fa	240981230	240982399	PRR21	0	0
chr2.fa	240984494	240985489	OR6B3	0	0
chr2.fa	241065980	241075764	MYEOV2	47.85122179	2128
chr2.fa	241078446	241080073	OTOS	0	0
chr2.fa	241375115	241407495	GPC1	0.45857041	76
chr2.fa	241388836	241396117	PP14571	5.86578716	532
chr2.fa	241395418	241395506	MIR149	0	0
chr2.fa	241418839	241497405	ANKMY1	0.992753787	152
chr2.fa	241499471	241503431	DUSP28	3.260698034	228
chr2.fa	241508104	241518143	RNPEPL1	31.34246465	4104
chr2.fa	241526133	241538526	CAPN10	4.474730947	532
chr2.fa	241544825	241570676	GPR35	0.651161086	76
chr2.fa	241615835	241622317	AQP12B	0	0
chr2.fa	241631262	241637900	AQP12A	0	0
chr2.fa	241653184	241759624	KIF1A	5.176374808	2052
chr2.fa	241808162	241818536	AGXT	4.230767931	304
chr2.fa	241825465	241835573	C2orf54	6.423988774	760
chr2.fa	241894036	241906868	LOC200772	0	0
chr2.fa	241938255	242033643	SNED1	0.755462503	228
chr2.fa	242026509	242041747	MTERFD2	15.2131067	3040
chr2.fa	242045515	242088878	PASK	5.223521718	1064
chr2.fa	242089902	242122439	PPP1R7	116.1662037	6754

chr2.fa	242127924	242164791	ANO7		3.419930048	684
chr2.fa	242166679	242255254	HDLBP		172.4415997	51184
chr2.fa	242254723	242293441		2-Sep	73.49491498	12416
chr2.fa	242295664	242434256	FARP2		15.30072878	2783
chr2.fa	242434432	242448034	STK25		29.20884462	2812
chr2.fa	242483801	242484259	BOK-AS		0	0
chr2.fa	242498870	242513553	BOK		19.8510727	2128
chr2.fa	242523820	242576725	THAP4		21.87460915	2280
chr2.fa	242577027	242613271	ATG4B		15.16885087	1976
chr2.fa	242615157	242626383	DTYMK		12.58199782	684
chr2.fa	242641456	242668896	ING5		6.503159999	1520
chr2.fa	242674030	242708231	D2HGDH		3.26803694	380
chr2.fa	242716240	242743702	GAL3ST2		0	0
chr2.fa	242750160	242758739	NEU4		0	0
chr2.fa	242792033	242801058	PDCD1		0	0
chr2.fa	242811886	242815482	C2orf85		0	0
chr2.fa	243030844	243102469	LOC728323		4.495190926	228
chr20.fa	68351	77296	DEFB125		0	0
chr20.fa	123252	126392	DEFB126		0	0
chr20.fa	138186	139804	DEFB127		0	0
chr20.fa	168527	170264	DEFB128		0	0
chr20.fa	207899	210527	DEFB129		0	0
chr20.fa	238377	241736	DEFB132		1.594544053	152
chr20.fa	251524	271390	C20orf96		4.347745639	304
chr20.fa	278204	280963	ZCCHC3		9.185641222	1140
chr20.fa	306239	310867	SOX12		10.2235404	2128
chr20.fa	327370	335512	NRSN2		5.666969534	608
chr20.fa	361308	378203	TRIB3		11.71823086	1292
chr20.fa	388709	411610	RBCK1		31.20880761	3876
chr20.fa	416124	443187	TBC1D20		20.86161777	4180
chr20.fa	463338	524482	CSNK2A1		29.20261767	3724
chr20.fa	584637	590910	TCF15		1.377490358	76
chr20.fa	627268	633890	SRXN1		20.4270656	2356
chr20.fa	642240	656823	SCRT2		0	0
chr20.fa	740724	749228	C20orf54		5.644285643	684
chr20.fa	814356	826922	FAM110A		15.81245066	1368
chr20.fa	853297	896960	ANGPT4		0	0
chr20.fa	939096	982907	RSPO4		0	0
chr20.fa	1093906	1148426	PSMF1		32.47310091	5610
chr20.fa	1161215	1165117	C20orf46		0	0
chr20.fa	1184098	1188918	C20orf202		0	0
chr20.fa	1206764	1235145	RAD21L1		0	0
chr20.fa	1246960	1289971	SNPH		0.338479226	76

chr20.fa	1306474	1309879	SDCBP2	0	0
chr20.fa	1349621	1356247	FKBP1A	130.5171002	10200
chr20.fa	1422811	1448417	NSFL1C	15.30606617	2508
chr20.fa	1455236	1472233	SIRPB2	0	0
chr20.fa	1514897	1538343	SIRPD	0	0
chr20.fa	1545029	1600689	SIRPB1	0.377397665	76
chr20.fa	1609798	1638425	SIRPG	0	0
chr20.fa	1754011	1760392	LOC100289473	0	0
chr20.fa	1874813	1920540	SIRPA	1.132415386	228
chr20.fa	1959402	1974931	PDYN	0	0
chr20.fa	2082528	2129198	STK35	17.40010059	5016
chr20.fa	2276613	2321725	TGM3	1.26273656	152
chr20.fa	2361554	2413399	TGM6	0	0
chr20.fa	2442281	2451499	SNRPB	50.35267667	2280
chr20.fa	2443598	2443693	SNORD119	0	0
chr20.fa	2462467	2489778	ZNF343	4.192739056	684
chr20.fa	2517253	2622430	TMC2	0	0
chr20.fa	2633254	2639039	NOP56	18.30812338	1672
chr20.fa	2633423	2633488	MIR1292	0	0
chr20.fa	2634858	2634932	SNORD110	0	0
chr20.fa	2635713	2635844	SNORA51	0	0
chr20.fa	2637270	2637340	SNORD56	0	0
chr20.fa	2637585	2637656	SNORD57	0	0
chr20.fa	2639041	2644843	IDH3B	39.71793516	2720
chr20.fa	2673524	2740754	EBF4	11.03170959	1444
chr20.fa	2774715	2781292	CPXM1	0	0
chr20.fa	2795657	2796476	C20orf141	0	0
chr20.fa	2796976	2797804	LOC100288797	0	0
chr20.fa	2815971	2821332	FAM113A	13.52804948	1216
chr20.fa	2821373	2847378	VPS16	12.98207937	1272
chr20.fa	2854142	3019315	PTPRA	16.76361732	2584
chr20.fa	3024268	3026391	GNRH2	0	0
chr20.fa	3026675	3028896	MRPS26	58.56224344	2736
chr20.fa	3052266	3053162	OXT	0	0
chr20.fa	3063202	3065370	AVP	0	0
chr20.fa	3088219	3104052	UBOX5	4.087770465	772
chr20.fa	3127165	3129906	FASTKD5	6.780481678	836
chr20.fa	3143273	3149207	ProSAPiP1	8.375248122	1976
chr20.fa	3171012	3185295	DDRGK1	14.82614622	836
chr20.fa	3190056	3204506	ITPA	23.60570132	1140
chr20.fa	3208063	3219887	SLC4A11	0.497266458	76
chr20.fa	3229948	3388255	C20orf194	7.088715716	2185
chr20.fa	3451665	3631769	ATRN	3.678126094	1444

chr20.fa	3639939	3644046	GFRA4	0	0
chr20.fa	3648620	3662738	ADAM33	0	0
chr20.fa	3667617	3687775	SIGLEC1	0	0
chr20.fa	3713317	3733758	HSPA12B	0	0
chr20.fa	3734158	3748452	C20orf27	8.598306376	532
chr20.fa	3758151	3762102	SPEF1	0	0
chr20.fa	3764498	3767337	CENPB	28.56635678	3648
chr20.fa	3776401	3786761	CDC25B	0.920031903	152
chr20.fa	3801203	3805954	C20orf29	2.767657007	228
chr20.fa	3827449	3856762	MAVS	18.6839643	9880
chr20.fa	3869486	3904502	PANK2	15.09701855	1672
chr20.fa	3898141	3898218	MIR103-2	0	0
chr20.fa	3912069	3996216	RNF24	3.798217278	608
chr20.fa	4129450	4168369	SMOX	3.71637736	380
chr20.fa	4173737	4176600	LOC728228	0	0
chr20.fa	4201278	4229659	ADRA1D	0	0
chr20.fa	4666797	4682234	PRNP	2.853055182	380
chr20.fa	4702556	4709106	PRND	0	0
chr20.fa	4711928	4721314	PRNT	0	0
chr20.fa	4760669	4804291	RASSF2	0.620471117	152
chr20.fa	4833002	4990939	SLC23A2	2.395374337	760
chr20.fa	5080484	5093733	C20orf30	80.9777077	6536
chr20.fa	5095599	5107268	PCNA	15.97524093	760
chr20.fa	5107482	5171989	CDS2	4.375544525	532
chr20.fa	5282686	5295015	PROKR2	0	0
chr20.fa	5451842	5457780	LOC643406	0	0
chr20.fa	5479218	5485242	LOC149837	0.470579528	76
chr20.fa	5525080	5591672	GPCPD1	4.012824671	985
chr20.fa	5731043	5844559	C20orf196	4.389999945	228
chr20.fa	5891974	5906005	CHGB	0	0
chr20.fa	5918486	5931173	TRMT6	10.97032965	1140
chr20.fa	5931298	5975831	MCM8	4.509423956	760
chr20.fa	5986739	6020697	CRLS1	13.0934973	2421
chr20.fa	6021425	6034694	LRRN4	0	0
chr20.fa	6055492	6104191	FERMT1	7.784355019	1803
chr20.fa	6748745	6760910	BMP2	0.536629679	76
chr20.fa	7863631	7921093	HAO1	0	0
chr20.fa	7961716	8000393	TMX4	2.144072415	228
chr20.fa	8113296	8865547	PLCB1	9.592616901	2943
chr20.fa	9049701	9461462	PLCB4	8.627661999	2195
chr20.fa	9495005	9511171	C20orf103	3.310736027	304
chr20.fa	9518037	9819687	PAK7	0.35382421	76
chr20.fa	10015697	10037407	ANKRD5	3.536907757	608

chr20.fa	10199477	10288065	SNAP25	0	0
chr20.fa	10385833	10414866	MKKS	17.06629158	2128
chr20.fa	10415951	10604027	C20orf94	7.207472554	456
chr20.fa	10618332	10654694	JAG1	7.057580965	1900
chr20.fa	11871477	11907243	BTBD3	8.14040314	1824
chr20.fa	12989627	13147411	SPTLC3	18.02257323	3116
chr20.fa	13202418	13281297	ISM1	0	0
chr20.fa	13370036	13619583	TASP1	7.189236485	760
chr20.fa	13694969	13765532	ESF1	9.98535955	1444
chr20.fa	13765672	13799067	C20orf7	3.50666257	380
chr20.fa	13830050	13971262	SEL1L2	0	0
chr20.fa	13976146	16033841	MACROD2	4.290146349	985
chr20.fa	14304639	14318313	FLRT3	5.880242581	1064
chr20.fa	16252749	16554079	KIF16B	15.69480578	4408
chr20.fa	16710609	16722417	SNRPB2	11.64706571	828
chr20.fa	16729003	16732809	OTOR	0	0
chr20.fa	17207631	17465222	PCSK2	0	0
chr20.fa	17474550	17539605	BFSP1	0	0
chr20.fa	17550599	17588652	DSTN	136.4916366	10716
chr20.fa	17594323	17662928	RRBP1	7.577308922	1292
chr20.fa	17674320	17716517	BANF2	0	0
chr20.fa	17922244	17949490	SNX5	29.8302053	2959
chr20.fa	17943353	17943589	SNORD17	21.39468919	228
chr20.fa	17949762	17971762	C20orf72	8.740859059	836
chr20.fa	18004796	18038521	OVOL2	15.21688735	1064
chr20.fa	18118499	18123812	PET117	14.63355554	304
chr20.fa	18125743	18169031	CSRP2BP	6.540521701	927
chr20.fa	18269121	18297640	ZNF133	6.22761745	760
chr20.fa	18359693	18362127	MGC44328	0	0
chr20.fa	18364011	18447829	C20orf12	3.863155473	608
chr20.fa	18448033	18465286	POLR3F	2.355121551	228
chr20.fa	18451259	18451335	MIR3192	0	0
chr20.fa	18467188	18477887	RBBP9	10.40745782	1805
chr20.fa	18488188	18542059	SEC23B	38.33532981	6459
chr20.fa	18548073	18550203	LOC388789	65.2708928	1444
chr20.fa	18568556	18744560	DTD1	13.60054897	836
chr20.fa	18768615	18774447	HSPC072	0	0
chr20.fa	18774693	18776709	LOC100270804	1.056357636	76
chr20.fa	18794370	18795035	C20orf79	0	0
chr20.fa	19193290	19703541	SLC24A3	0	0
chr20.fa	19222946	19265240	LOC100130264	0	0
chr20.fa	19870210	19983100	RIN2	17.52308286	3344
chr20.fa	19997937	20014269	NAA20	56.99905653	3132

chr20.fa	20015012	20036690	CRNKL1	16.79230577	3268
chr20.fa	20037262	20341346	C20orf26	0	0
chr20.fa	20348765	20351592	INSM1	0	0
chr20.fa	20370272	20693266	RALGAPA2	52.33951859	22184
chr20.fa	21106624	21227258	PLK1S1	5.382753732	532
chr20.fa	21283942	21370463	XRN2	28.04662883	4332
chr20.fa	21376005	21378047	NKX2-4	0	0
chr20.fa	21491660	21494664	NKX2-2	0	0
chr20.fa	21686297	21696620	PAX1	0	0
chr20.fa	22380971	22401281	LOC284788	0	0
chr20.fa	22541192	22559280	NCRNA00261	0.344261394	76
chr20.fa	22561642	22566101	FOXA2	0	0
chr20.fa	23016057	23017314	SSTR4	0	0
chr20.fa	23026270	23030301	THBD	0.419207188	76
chr20.fa	23059993	23066977	CD93	0	0
chr20.fa	23105705	23113273	LOC200261	0	0
chr20.fa	23331373	23335408	NXT1	3.026297834	152
chr20.fa	23345021	23353683	GZF1	4.27791484	912
chr20.fa	23355156	23402156	NAPB	1.746437161	304
chr20.fa	23420322	23425567	CSTL1	0	0
chr20.fa	23431041	23433482	CST11	0	0
chr20.fa	23471766	23476655	CST8	0	0
chr20.fa	23499783	23522655	CSTT	0	0
chr20.fa	23545370	23549386	CST9L	0	0
chr20.fa	23583047	23586610	CST9	0	0
chr20.fa	23614294	23618574	CST3	69.78765566	2432
chr20.fa	23666277	23669662	CST4	0	0
chr20.fa	23728190	23731574	CST1	0	0
chr20.fa	23804404	23807312	CST2	0	0
chr20.fa	23856572	23860380	CST5	0	0
chr20.fa	23965690	23969416	GGTLC1	0	0
chr20.fa	24449835	24647167	TMEM90B	0	0
chr20.fa	24929866	24940564	CST7	0	0
chr20.fa	24943580	24973425	C20orf3	3.80488901	380
chr20.fa	24986873	25039616	ACSS1	29.47326761	5928
chr20.fa	25056099	25062767	VSX1	0	0
chr20.fa	25121434	25129426	LOC284798	0	0
chr20.fa	25176339	25207360	ENTPD6	11.56233471	1436
chr20.fa	25228706	25278648	PYGB	49.50647861	9196
chr20.fa	25275379	25371477	ABHD12	18.7464562	1900
chr20.fa	25388323	25429191	GINS1	1.027669187	152
chr20.fa	25433338	25566153	NINL	254.9133306	56991
chr20.fa	25593573	25604648	NANP	0.445004554	76

chr20.fa	25654851	25677469	ZNF337	6.344150376	912
chr20.fa	25744102	25781927	FAM182B	0	0
chr20.fa	25990435	26002430	LOC100134868	3.262921945	228
chr20.fa	26035250	26067553	FAM182A	0	0
chr20.fa	26084052	26094677	C20orf191	0	0
chr20.fa	26188822	26188914	MIR663	0	0
chr20.fa	29611879	29634007	FRG1B	8.933227345	380
chr20.fa	29845467	29847435	DEFB115	0	0
chr20.fa	29891015	29896388	DEFB116	0	0
chr20.fa	29956421	29961705	DEFB118	0	0
chr20.fa	29964967	29978406	DEFB119	0	0
chr20.fa	29992648	30000641	DEFB121	0	0
chr20.fa	30009242	30016977	DEFB122	0	0
chr20.fa	30028411	30038060	DEFB123	0	0
chr20.fa	30053309	30060816	DEFB124	0	0
chr20.fa	30063105	30072708	REM1	0	0
chr20.fa	30073581	30075377	NCRNA00028	0	0
chr20.fa	30102241	30157370	HM13	4.820326687	722
chr20.fa	30135185	30136019	PSIMCT-1	8.096592097	304
chr20.fa	30193092	30194313	ID1	106.5001978	5852
chr20.fa	30194989	30195043	MIR3193	0	0
chr20.fa	30225691	30232800	COX4I2	0	0
chr20.fa	30252261	30310656	BCL2L1	75.2949484	8664
chr20.fa	30326904	30389603	TPX2	0	0
chr20.fa	30407178	30422500	MYLK2	0	0
chr20.fa	30432103	30433420	FOXS1	0	0
chr20.fa	30448870	30458479	DUSP15	1.847847495	144
chr20.fa	30458505	30530858	TTLL9	0	0
chr20.fa	30532758	30539883	PDRG1	12.31001352	760
chr20.fa	30555805	30586256	XKR7	0	0
chr20.fa	30598245	30619984	C20orf160	0	0
chr20.fa	30639991	30689657	HCK	0	0
chr20.fa	30697309	30755061	TM9SF4	5.09853793	912
chr20.fa	30776949	30778163	TSPY26P	1.391056214	76
chr20.fa	30780307	30795546	PLAGL2	8.06834843	2052
chr20.fa	30795696	30826467	POFUT1	3.581385973	988
chr20.fa	30865454	30922811	KIF3B	22.67210357	6232
chr20.fa	30946147	31027122	ASXL1	15.25135796	5092
chr20.fa	31030862	31071288	C20orf112	7.59954803	2052
chr20.fa	31175281	31196694	LOC149950	0	0
chr20.fa	31219427	31239783	C20orf203	0	0
chr20.fa	31290493	31331814	COMMD7	30.90902443	2656
chr20.fa	31350191	31397162	DNMT3B	0.364943764	76

chr20.fa	31407699	31438211	MAPRE1	46.39433782	5472
chr20.fa	31571581	31592239	SUN5	0	0
chr20.fa	31595407	31611515	BPIL1	0	0
chr20.fa	31619454	31631853	BPIL3	0	0
chr20.fa	31643230	31661434	C20orf185	0	0
chr20.fa	31669318	31699557	C20orf186	0	0
chr20.fa	31755960	31769223	C20orf70	0	0
chr20.fa	31781411	31798268	BASE	0	0
chr20.fa	31805135	31815559	C20orf71	0	0
chr20.fa	31823802	31831115	PLUNC	0	0
chr20.fa	31870941	31897684	C20orf114	0	0
chr20.fa	31946645	31989337	CDK5RAP1	6.399525755	602
chr20.fa	31995763	32031698	SNTA1	6.500713697	684
chr20.fa	32077928	32237837	CBFA2T2	13.02277694	4636
chr20.fa	32244893	32262264	NECAB3	30.34481826	2643
chr20.fa	32250092	32251721	C20orf144	0	0
chr20.fa	32254304	32256331	C20orf134	1.666821154	152
chr20.fa	32263292	32274210	E2F1	1.252061788	152
chr20.fa	32290550	32308136	PXMP4	7.972497873	2052
chr20.fa	32319808	32380075	ZNF341	3.05832215	456
chr20.fa	32399110	32442173	CHMP4B	80.04455472	5928
chr20.fa	32581732	32668067	RALY	59.0219258	4180
chr20.fa	32676115	32700085	EIF2S2	31.21370022	3638
chr20.fa	32848171	32857148	ASIP	0	0
chr20.fa	32868071	32899608	AHCY	101.2239693	11552
chr20.fa	32951062	33099197	ITCH	28.95376205	8305
chr20.fa	33054130	33054223	MIR644	0	0
chr20.fa	33104204	33128762	DYNLRB1	175.1856832	5396
chr20.fa	33134692	33148149	MAP1LC3A	30.15467389	1520
chr20.fa	33148346	33265089	PIGU	2.767657007	228
chr20.fa	33292148	33301237	TP53INP2	22.95098198	4256
chr20.fa	33302578	33413433	NCOA6	18.18936654	5776
chr20.fa	33421378	33422265	HMGB3P1	0	0
chr20.fa	33432523	33460661	GGT7	1.280527846	152
chr20.fa	33462766	33515769	ACSS2	6.881447229	988
chr20.fa	33516236	33543601	GSS	25.79736543	2204
chr20.fa	33543704	33590206	MYH7B	0	0
chr20.fa	33578179	33578300	MIR499	0	0
chr20.fa	33590241	33680618	TRPC4AP	18.46579865	2584
chr20.fa	33703160	33735161	EDEM2	1.767119532	152
chr20.fa	33759774	33765165	PROCR	0	0
chr20.fa	33814539	33864804	MMP24	9.361774958	1824
chr20.fa	33866725	33872594	EIF6	88.87437021	4332

chr20.fa	33873534	33880225	FAM83C	1.067699581	152
chr20.fa	33890369	33999945	UQCC	7.594655426	836
chr20.fa	34021149	34026027	GDF5	0	0
chr20.fa	34043223	34099803	CEP250	6.663281578	2494
chr20.fa	34108570	34117481	C20orf173	0	0
chr20.fa	34129778	34145405	ERGIC3	89.34717365	5488
chr20.fa	34146507	34195484	FER1L4	10.69189602	3163
chr20.fa	34203809	34208965	SPAG4	0	0
chr20.fa	34213953	34220845	CPNE1	33.7000325	2764
chr20.fa	34236847	34243123	RBM12	11.18182357	2964
chr20.fa	34256610	34287231	NFS1	10.34385397	1140
chr20.fa	34287288	34288902	ROMO1	166.9305263	3040
chr20.fa	34291531	34330193	RBM39	79.99229281	10039
chr20.fa	34359923	34538288	PHF20	14.3502293	3800
chr20.fa	34541546	34542428	SCAND1	28.35130461	988
chr20.fa	34556529	34618622	C20orf152	0	0
chr20.fa	34633540	34638882	LOC647979	63.26670437	15200
chr20.fa	34700348	34820721	EPB41L1	30.43421948	8664
chr20.fa	34824447	34844853	C20orf4	13.40907025	1596
chr20.fa	34995444	35157040	DLGAP4	12.59400693	3344
chr20.fa	35169897	35178226	MYL9	7.119405686	380
chr20.fa	35201876	35222355	TGIF2	6.116421909	912
chr20.fa	35234137	35238161	C20orf24	25.49713747	540
chr20.fa	35240961	35274558	SLA2	0	0
chr20.fa	35280169	35374481	NDRG3	73.74777364	9829
chr20.fa	35380194	35402230	DSN1	5.37207896	608
chr20.fa	35405845	35492087	C20orf117	7.74766049	3040
chr20.fa	35504570	35522632	C20orf118	0.920476685	76
chr20.fa	35520227	35580246	SAMHD1	6.796938618	912
chr20.fa	35626178	35724410	RBL1	1.152875365	228
chr20.fa	35729629	35807974	C20orf132	0.965622075	152
chr20.fa	35807456	35870025	RPN2	7.720973561	836
chr20.fa	35879490	35885299	GHRH	0	0
chr20.fa	35918051	35945663	MANBAL	8.997053585	532
chr20.fa	35973088	36033821	SRC	35.80696561	6764
chr20.fa	36145819	36156333	BLCAP	11.70377544	1292
chr20.fa	36149607	36152090	NNAT	0	0
chr20.fa	36322434	36500520	CTNNBL1	25.06614356	2128
chr20.fa	36531499	36573747	VSTM2L	0.861987831	76
chr20.fa	36611423	36661833	TTI1	11.22741374	1976
chr20.fa	36661948	36720766	RPRD1B	16.58748358	2888
chr20.fa	36756864	36793700	TGM2	25.23871903	4636
chr20.fa	36838907	36889174	KIAA1755	0.527289254	152

chr20.fa	36932552	36965905	BPI	3.664337847	304
chr20.fa	36974885	37005653	LBP	0	0
chr20.fa	37049239	37064018	LOC388796	9.073111335	532
chr20.fa	37053843	37053978	SNORA71B	0	0
chr20.fa	37055949	37056086	SNORA71A	0	0
chr20.fa	37058310	37058447	SNORA71C	0	0
chr20.fa	37062505	37062642	SNORA71D	0	0
chr20.fa	37075297	37079564	SNHG11	17.10387567	836
chr20.fa	37076726	37076861	SNORA39	0	0
chr20.fa	37078012	37078147	SNORA60	0	0
chr20.fa	37101486	37207504	RALGAPB	23.2896836	9044
chr20.fa	37209838	37217104	ADIG	0	0
chr20.fa	37230577	37279295	ARHGAP40	10.12435398	1292
chr20.fa	37353105	37358015	SLC32A1	0	0
chr20.fa	37377097	37401089	ACTR5	4.09088394	456
chr20.fa	37434348	37551667	PPP1R16B	4.326173705	1216
chr20.fa	37554955	37581703	FAM83D	0	0
chr20.fa	37590981	37668366	DHX35	3.540243623	532
chr20.fa	37842424	37853391	LOC339568	0	0
chr20.fa	39314517	39317876	MAFB	9.55747911	1444
chr20.fa	39657462	39753126	TOP1	37.78802536	6343
chr20.fa	39766161	39804357	PLCG1	8.117941641	1900
chr20.fa	39807089	39928739	ZHX3	8.627661999	3876
chr20.fa	39969560	39988605	LPIN3	0	0
chr20.fa	39989223	39995498	EMILIN3	0	0
chr20.fa	40031170	40247133	CHD6	24.49971347	11445
chr20.fa	40701392	41818557	PTPRT	0	0
chr20.fa	42086504	42092244	SRSF6	32.00118703	5776
chr20.fa	42136320	42170534	L3MBTL1	0.474137785	76
chr20.fa	42187635	42214273	SGK2	11.62193552	1284
chr20.fa	42219579	42275862	IFT52	11.09953887	836
chr20.fa	42295709	42345122	MYBL2	0	0
chr20.fa	42354801	42355642	GTSF1L	0	0
chr20.fa	42543492	42698254	TOX2	0	0
chr20.fa	42740337	42816218	JPH2	0	0
chr20.fa	42825136	42839431	C20orf111	13.97727946	988
chr20.fa	42875908	42909013	GDAP1L1	0	0
chr20.fa	42935197	42939889	FITM2	0	0
chr20.fa	42965626	42979875	R3HDML	0	0
chr20.fa	42984441	43058311	HNF4A	19.71674849	1368
chr20.fa	43036760	43036843	MIR3646	0	0
chr20.fa	43104547	43123244	TTPAL	4.89505009	1368
chr20.fa	43124864	43150726	SERINC3	9.923979612	1216

chr20.fa	43160436	43247678	PKIG	11.79473339	760
chr20.fa	43248163	43280376	ADA	0	0
chr20.fa	43285092	43300380	LOC79015	0	0
chr20.fa	43343885	43356452	WISP2	0	0
chr20.fa	43374488	43379668	KCNK15	1.356585596	76
chr20.fa	43380449	43438912	RIMS4	0	0
chr20.fa	43514344	43537161	YWHAB	134.4516432	18772
chr20.fa	43538703	43567962	PABPC1L	0	0
chr20.fa	43570771	43589114	TOMM34	44.54337685	4104
chr20.fa	43595120	43708593	STK4	11.21451506	3192
chr20.fa	43720950	43729753	KCNS1	0.744787731	152
chr20.fa	43738093	43743803	WFDC5	0	0
chr20.fa	43752067	43753106	WFDC12	0	0
chr20.fa	43803540	43805185	PI3	0	0
chr20.fa	43835638	43838414	SEMG1	2.032654483	152
chr20.fa	43850010	43853099	SEMG2	1.705517202	152
chr20.fa	43880879	43883206	SLPI	0	0
chr20.fa	43922087	43936967	MATN4	0	0
chr20.fa	43935491	43946464	RBPJL	0	0
chr20.fa	43953929	43977064	SDC4	24.1547849	2837
chr20.fa	43990577	44005442	SYS1	5.627606312	1140
chr20.fa	44002520	44006957	TP53TG5	0	0
chr20.fa	44034633	44037676	DBNDD2	0	0
chr20.fa	44044707	44054884	PIGT	0.75279381	76
chr20.fa	44098394	44110172	WFDC2	5.972312488	152
chr20.fa	44141101	44144264	SPINT3	0	0
chr20.fa	44162836	44168134	WFDC6	0	0
chr20.fa	44164919	44166189	SPINLW1-WFDC6	0	0
chr20.fa	44169265	44170793	SPINLW1	0	0
chr20.fa	44179791	44207965	WFDC8	0	0
chr20.fa	44236578	44259907	WFDC9	0	0
chr20.fa	44258385	44259831	WFDC10A	0	0
chr20.fa	44277202	44298878	WFDC11	0	0
chr20.fa	44313290	44333658	WFDC10B	0	0
chr20.fa	44330655	44337456	WFDC13	0	0
chr20.fa	44350988	44354335	SPINT4	0	0
chr20.fa	44402847	44420547	WFDC3	0	0
chr20.fa	44420576	44440066	DNTTIP1	16.94664518	983
chr20.fa	44441255	44445596	UBE2C	0	0
chr20.fa	44451855	44455953	TNNC2	0	0
chr20.fa	44462470	44471914	SNX21	1.723975662	228
chr20.fa	44472166	44486048	ACOT8	10.37031851	436
chr20.fa	44486220	44507769	ZSWIM3	1.706184376	228

chr20.fa	44509848	44513905	ZSWIM1	8.490224311	1064
chr20.fa	44515130	44516238	C20orf165	2.350673729	76
chr20.fa	44517267	44519590	NEURL2	1.782909299	76
chr20.fa	44519902	44527396	CTSA	7.242387954	608
chr20.fa	44527459	44540786	PLTP	0	0
chr20.fa	44563317	44576662	PCIF1	15.66722929	1900
chr20.fa	44577292	44600833	ZNF335	1.898107879	380
chr20.fa	44637547	44645200	MMP9	0	0
chr20.fa	44650329	44688789	SLC12A5	0	0
chr20.fa	44689626	44718580	NCOA5	21.08111777	3040
chr20.fa	44746906	44758384	CD40	1.045905255	76
chr20.fa	44802376	44880334	CDH22	0	0
chr20.fa	44978177	44993064	SLC35C2	6.905020683	684
chr20.fa	44994690	45035271	ELMO2	18.82051243	3116
chr20.fa	45092299	45093931	LOC100240726	0	0
chr20.fa	45129707	45142194	ZNF334	1.45821832	228
chr20.fa	45169585	45179213	C20orf123	0	0
chr20.fa	45186462	45298778	SLC13A3	3.26203238	608
chr20.fa	45313004	45318276	TP53RK	11.32615538	1672
chr20.fa	45338279	45364985	SLC2A10	1.596323182	304
chr20.fa	45523263	45817492	EYA2	10.00848822	1216
chr20.fa	45795609	45795700	MIR3616	0	0
chr20.fa	45838381	45985474	ZMYND8	27.93743481	5844
chr20.fa	45947246	45949498	LOC100131496	0	0
chr20.fa	46130601	46285621	NCOA3	22.77751694	8208
chr20.fa	46286150	46415360	SULF2	0	0
chr20.fa	46988654	46999381	LOC284749	0	0
chr20.fa	47240793	47444420	PREX1	5.320929011	1587
chr20.fa	47538275	47653230	ARFGEF2	28.09043987	11373
chr20.fa	47662838	47713486	CSE1L	20.32810157	3255
chr20.fa	47729876	47804904	STAU1	42.62058356	7296
chr20.fa	47835832	47860614	DDX27	21.32307927	2584
chr20.fa	47862439	47894714	ZNFX1	23.5289764	7752
chr20.fa	47894757	47905795	NCRNA00275	100.8654749	4472
chr20.fa	47895482	47895560	SNORD12C	0	0
chr20.fa	47896850	47896952	SNORD12B	0	0
chr20.fa	47897220	47897309	SNORD12	0	0
chr20.fa	47988505	48099181	KCNB1	0	0
chr20.fa	48120411	48184707	PTGIS	0	0
chr20.fa	48249483	48330421	B4GALT5	4.64819599	988
chr20.fa	48429250	48508772	SLC9A8	3.782872293	1064
chr20.fa	48519929	48532080	SPATA2	6.28321522	1216
chr20.fa	48552914	48570422	RNF114	38.36624217	4256

chr20.fa	48599513	48605420	SNAI1	0	0
chr20.fa	48729644	48732494	UBE2V1	4.899275521	102
chr20.fa	48740274	48767852	TMEM189	2.184992374	152
chr20.fa	48807376	48809212	CEBPB	4.600381908	380
chr20.fa	48909257	48931456	LOC284751	0	0
chr20.fa	49126891	49201086	PTPN1	21.91708584	3268
chr20.fa	49202323	49202416	MIR645	0	0
chr20.fa	49202647	49253426	FAM65C	0	0
chr20.fa	49348081	49370278	PARD6B	19.04423786	3952
chr20.fa	49411467	49493714	BCAS4	2.518801387	152
chr20.fa	49506883	49547527	ADNP	50.92399936	10792
chr20.fa	49551405	49575060	DPM1	6.457125046	304
chr20.fa	49575363	49577820	MOCS3	5.501065787	608
chr20.fa	49620193	49639675	KCNG1	0.761244671	76
chr20.fa	50007765	50179168	NFATC2	1.525380427	228
chr20.fa	50069442	50069514	MIR3194	0	0
chr20.fa	50213314	50384908	ATP9A	8.027206079	2736
chr20.fa	50400583	50419048	SALL4	1.940584575	304
chr20.fa	50700550	50808524	ZFP64	4.069534397	912
chr20.fa	51588946	52111869	TSHZ2	0.80616767	456
chr20.fa	52183610	52199636	ZNF217	60.28822062	15276
chr20.fa	52491040	52492248	SUMO1P1	0	0
chr20.fa	52560079	52687304	BCAS1	190.8916085	29828
chr20.fa	52769988	52790516	CYP24A1	6.7275526	988
chr20.fa	52824502	52836492	PFDN4	5.022702571	304
chr20.fa	53092266	53267710	DOK5	0	0
chr20.fa	54572496	54580012	CBLN4	0	0
chr20.fa	54823788	54824871	MC3R	0	0
chr20.fa	54933983	54943718	C20orf108	71.32949302	9728
chr20.fa	54944445	54967351	AURKA	1.327229973	152
chr20.fa	54967427	54979582	CSTF1	11.82853683	1368
chr20.fa	54987168	55034396	CASS4	0.518838393	76
chr20.fa	55043647	55093942	C20orf43	45.86727095	3401
chr20.fa	55066548	55100859	GCNT7	0	0
chr20.fa	55099785	55101208	C20orf106	0	0
chr20.fa	55108302	55111576	C20orf107	0	0
chr20.fa	55204358	55214338	TFAP2C	7.669156439	988
chr20.fa	55743809	55841707	BMP7	0	0
chr20.fa	55896558	55896647	MIR4325	0	0
chr20.fa	55904831	55919049	SPO11	0	0
chr20.fa	55926145	55953519	RAE1	6.230953316	608
chr20.fa	55933496	55934878	MTRNR2L3	0	0
chr20.fa	55966463	55984386	RBM38	4.97310936	532

chr20.fa	56072224	56100154	CTCFL	0	0
chr20.fa	56136137	56141513	PCK1	43.43453492	5244
chr20.fa	56178902	56195632	ZBP1	0	0
chr20.fa	56223452	56286541	PMEPA1	76.81187796	17912
chr20.fa	56725983	56736183	C20orf85	0	0
chr20.fa	56807833	56884495	PPP4R1L	1.409737064	152
chr20.fa	56884771	56942563	RAB22A	8.007635664	3115
chr20.fa	56964175	57026156	VAPB	17.03382248	6080
chr20.fa	57034426	57089949	APCDD1L	0.543079021	76
chr20.fa	57090435	57194948	LOC149773	0	0
chr20.fa	57226328	57254582	STX16	13.45154694	2962
chr20.fa	57267862	57290900	NPEPL1	6.961730409	684
chr20.fa	57392670	57392749	MIR296	0	0
chr20.fa	57393281	57393368	MIR298	0	0
chr20.fa	57393973	57425958	GNAS-AS	0	0
chr20.fa	57414795	57486250	GNAS	122.0193146	30945
chr20.fa	57556311	57570188	TH1L	41.93606381	4256
chr20.fa	57570242	57582309	CTSZ	19.15521101	1292
chr20.fa	57594309	57601709	TUBB1	0.483478211	76
chr20.fa	57603733	57607400	ATP5E	445.8487502	8360
chr20.fa	57608200	57617901	SLMO2	26.37802854	3040
chr20.fa	57766075	57834167	ZNF831	0	0
chr20.fa	57875499	57901047	EDN3	0	0
chr20.fa	58152564	58422766	PHACTR3	0	0
chr20.fa	58438618	58507209	SYCP2	0.925369289	228
chr20.fa	58508819	58523702	C20orf177	5.258214726	1216
chr20.fa	58511887	58515352	PPP1R3D	9.606182757	1444
chr20.fa	58533471	58588168	CDH26	9.883949217	1520
chr20.fa	58630980	58648008	C20orf197	0	0
chr20.fa	59827559	60512299	CDH4	0	0
chr20.fa	60528602	60528718	MIR1257	0	0
chr20.fa	60549854	60640866	TAF4	6.511166078	1355
chr20.fa	60697517	60710434	LSM14B	18.24385235	2128
chr20.fa	60711791	60718474	PSMA7	91.03578913	4028
chr20.fa	60718822	60757566	SS18L1	4.087992856	836
chr20.fa	60758081	60777810	GTPBP5	4.029726393	532
chr20.fa	60790017	60795323	HRH3	0	0
chr20.fa	60813580	60871269	OSBPL2	28.09155183	5016
chr20.fa	60878027	60883918	ADRM1	39.69791997	2508
chr20.fa	60884121	60942368	LAMA5	0.443670207	228
chr20.fa	60962121	60963576	RPS21	744.1116624	13250
chr20.fa	60963686	60982339	CABLES2	0.893122582	152
chr20.fa	60985293	61002629	C20orf151	7.159213689	912

chr20.fa	61038553	61051026	GATA5	0	0
chr20.fa	61141438	61148768	C20orf200	0	0
chr20.fa	61147660	61167971	C20orf166	0	0
chr20.fa	61151513	61151583	MIR1-1	0	0
chr20.fa	61162119	61162195	MIR133A2	0	0
chr20.fa	61273797	61303647	SLCO4A1	1.391056214	152
chr20.fa	61294379	61297973	LOC100127888	0	0
chr20.fa	61340189	61394123	NTSR1	0	0
chr20.fa	61427805	61431945	C20orf20	5.165477645	380
chr20.fa	61436177	61445352	OGFR	9.777868671	1064
chr20.fa	61448414	61472466	COL9A3	0.692748218	76
chr20.fa	61472512	61493115	TCFL5	5.608258288	608
chr20.fa	61476476	61477543	DPH3P1	0	0
chr20.fa	61509090	61569304	DIDO1	9.891510514	5928
chr20.fa	61569441	61579827	C20orf11	28.63396367	5700
chr20.fa	61583999	61599949	SLC17A9	0	0
chr20.fa	61637331	61638387	BHLHE23	0	0
chr20.fa	61640735	61716423	LOC63930	0	0
chr20.fa	61665569	61668380	NCRNA00029	0	0
chr20.fa	61669363	61685215	LOC100144597	0	0
chr20.fa	61726845	61727535	HAR1B	0	0
chr20.fa	61732644	61735737	HAR1A	0	0
chr20.fa	61809852	61809938	MIR124-3	0	0
chr20.fa	61826782	61847538	YTHDF1	24.94716433	3648
chr20.fa	61867276	61871854	BIRC7	0	0
chr20.fa	61870131	61870194	MIR3196	0	0
chr20.fa	61872136	61881424	NKAIN4	1.31433129	76
chr20.fa	61885330	61892967	FLJ16779	0	0
chr20.fa	61904165	61921142	ARFGAP1	7.729424422	1140
chr20.fa	61918160	61918218	MIR4326	0	0
chr20.fa	61924538	61962285	COL20A1	0	0
chr20.fa	61974665	61992695	CHRNA4	0.308011648	76
chr20.fa	62037542	62103993	KCNQ2	0	0
chr20.fa	62119366	62130505	EEF1A2	48.60512756	4028
chr20.fa	62152133	62153524	PPDPF	515.5352179	18012
chr20.fa	62159776	62168707	PTK6	15.49376424	1748
chr20.fa	62172163	62178857	SRMS	3.344761863	228
chr20.fa	62184373	62188035	C20orf195	0	0
chr20.fa	62189439	62205592	PRIC285	3.886728928	1748
chr20.fa	62218955	62258381	GMEB2	6.824959894	1292
chr20.fa	62271061	62284780	STMN3	3.747512111	380
chr20.fa	62289647	62326287	RTEL1	1.390611431	228
chr20.fa	62328021	62329994	TNFRSF6B	0	0

chr20.fa	62330036	62339355	ARFRP1	11.8194188	1368
chr20.fa	62338794	62367494	ZGPAT	19.72964717	1824
chr20.fa	62367978	62370460	LIME1	19.73520695	1064
chr20.fa	62371211	62375403	SLC2A4RG	35.59814038	3648
chr20.fa	62375878	62436856	ZBTB46	2.703608375	532
chr20.fa	62492566	62494341	ABHD16B	4.758279575	380
chr20.fa	62496590	62522890	TPD52L2	30.00567186	3253
chr20.fa	62526455	62567384	DNAJC5	28.41957867	6764
chr20.fa	62550778	62550833	MIR941-1	0	0
chr20.fa	62550850	62551301	MIR941-2	0	0
chr20.fa	62550895	62551201	MIR941-3	0	0
chr20.fa	62571182	62582527	UCKL1	2.248373832	212
chr20.fa	62572818	62572897	MIR1914	0	0
chr20.fa	62573984	62574079	MIR647	0	0
chr20.fa	62584737	62588054	UCKL1-AS1	0	0
chr20.fa	62588339	62601218	ZNF512B	9.598176678	2432
chr20.fa	62605466	62610995	SAMD10	6.974629092	684
chr20.fa	62612431	62664453	PRPF6	30.52228634	4256
chr20.fa	62665697	62671315	NCRNA00176	0	0
chr20.fa	62679079	62680979	SOX18	0	0
chr20.fa	62688439	62703700	TCEA2	2.861061261	304
chr20.fa	62704535	62711324	RGS19	0	0
chr20.fa	62711471	62731996	OPRL1	0	0
chr20.fa	62714733	62715712	C20orf201	0	0
chr20.fa	62737183	62738184	NPBWR2	0	0
chr20.fa	62795827	62873606	MYT1	0.305342955	76
chr20.fa	62887048	62907579	PCMTD2	10.36920656	1824
chr21.fa	9825832	9826011	MIR3648	0	0
chr21.fa	9826203	9826263	MIR3687	0	0
chr21.fa	9907194	9968585	LOC100132288	6.147334269	442
chr21.fa	10906743	10990920	TPTE	0	0
chr21.fa	11057796	11098937	BAGE	0	0
chr21.fa	11097542	11097545	BAGE4	0	0
chr21.fa	14410487	14490571	ANKRD30BP2	0	0
chr21.fa	14778705	14778781	MIR3156-3	0	0
chr21.fa	14982498	15013906	POTED	0	0
chr21.fa	15215454	15220685	C21orf15	0	0
chr21.fa	15316096	15352765	C21orf81	0	0
chr21.fa	15481135	15579254	LIPI	0	0
chr21.fa	15588466	15600693	RBM11	0.859319138	76
chr21.fa	15646120	15673692	ABCC13	0	0
chr21.fa	15743437	15755509	HSPA13	0.847977193	152
chr21.fa	15857549	15918664	SAMSN1	0	0

chr21.fa	16333556	16437126	NRIP1	16.77051144	5698
chr21.fa	17102496	17252377	USP25	15.30962443	3420
chr21.fa	17442842	17982094	C21orf34	3.556478172	608
chr21.fa	17911409	17911489	MIR99A	0	0
chr21.fa	17912148	17912231	MIRLET7C	0	0
chr21.fa	17962557	17962645	MIR125B2	0	0
chr21.fa	18811208	18821503	C21orf37	0	0
chr21.fa	18885330	18939266	CXADR	5.469708645	608
chr21.fa	18965968	18985268	BTG3	5.301580987	380
chr21.fa	19161284	19191703	C21orf91	2.177653468	532
chr21.fa	19207989	19257925	NCRNA00157	0	0
chr21.fa	19617150	19639687	CHODL	0	0
chr21.fa	19641433	19775970	TMPRSS15	0	0
chr21.fa	22114913	22175426	C21orf131	0	0
chr21.fa	22370633	22912517	NCAM2	0.337812052	76
chr21.fa	26758133	26804013	NCRNA00158	0	0
chr21.fa	26934457	26947480	MIR155HG	0	0
chr21.fa	26955087	26955536	C21orf71	0	0
chr21.fa	26957968	26979801	MRPL39	10.10344921	532
chr21.fa	27011589	27087235	JAM2	0	0
chr21.fa	27096791	27107965	ATP5J	59.1068792	3192
chr21.fa	27113884	27144771	GABPA	5.064956877	1052
chr21.fa	27252861	27543138	APP	15.22400386	2487
chr21.fa	27838528	27945581	CYYR1	0.548861189	76
chr21.fa	28208606	28217728	ADAMTS1	11.62882964	2432
chr21.fa	28290231	28339439	ADAMTS5	0.524842952	228
chr21.fa	29094698	29123552	NCRNA00113	0	0
chr21.fa	29385682	29395528	C21orf94	0	0
chr21.fa	29911640	29912677	NCRNA00161	23.0179217	532
chr21.fa	30244513	30257693	N6AMT1	2.084471605	456
chr21.fa	30300466	30365277	LTN1	7.627124524	2660
chr21.fa	30378080	30391685	RWDD2B	19.8261649	1520
chr21.fa	30396938	30426807	USP16	24.96406605	3408
chr21.fa	30428648	30446010	CCT8	78.38885312	6676
chr21.fa	30452873	30548202	C21orf7	0	0
chr21.fa	30565801	30660526	NCRNA00189	0	0
chr21.fa	30671220	30734217	BACH1	23.6036998	7144
chr21.fa	30909254	31312282	GRIK1	0	0
chr21.fa	30968360	31003067	NCRNA00258	0	0
chr21.fa	31120494	31136325	GRIK1-AS1	0	0
chr21.fa	31538241	31538971	CLDN17	0	0
chr21.fa	31586324	31588469	CLDN8	2.362682848	228
chr21.fa	31653627	31655276	KRTAP24-1	0	0

chr21.fa	31661463	31661832	KRTAP25-1	0	0
chr21.fa	31691450	31692607	KRTAP26-1	0	0
chr21.fa	31709331	31710012	KRTAP27-1	0	0
chr21.fa	31720717	31720924	KRTAP23-1	0	0
chr21.fa	31743709	31744557	KRTAP13-2	0	0
chr21.fa	31747612	31747696	MIR4327	0	0
chr21.fa	31768392	31769138	KRTAP13-1	0	0
chr21.fa	31797711	31798230	KRTAP13-3	0	0
chr21.fa	31802594	31803076	KRTAP13-4	0	0
chr21.fa	31812646	31813098	KRTAP15-1	0	0
chr21.fa	31852364	31852636	KRTAP19-1	0	0
chr21.fa	31859509	31859667	KRTAP19-2	0	0
chr21.fa	31863782	31864275	KRTAP19-3	0	0
chr21.fa	31869174	31869428	KRTAP19-4	0	0
chr21.fa	31874190	31874408	KRTAP19-5	0	0
chr21.fa	31913854	31914181	KRTAP19-6	0	0
chr21.fa	31933417	31933608	KRTAP19-7	0	0
chr21.fa	31962424	31962716	KRTAP22-2	0	0
chr21.fa	31964759	31965374	KRTAP6-3	0	0
chr21.fa	31971005	31971193	KRTAP6-2	0	0
chr21.fa	31973440	31973586	KRTAP22-1	0	0
chr21.fa	31986005	31986223	KRTAP6-1	0	0
chr21.fa	31988774	31988944	KRTAP20-1	0	0
chr21.fa	31992946	31993169	KRTAP20-4	0	0
chr21.fa	32007583	32007780	KRTAP20-2	0	0
chr21.fa	32015183	32015455	KRTAP20-3	0	0
chr21.fa	32090843	32091095	KRTAP21-3	0	0
chr21.fa	32119269	32119520	KRTAP21-2	0	0
chr21.fa	32127457	32127696	KRTAP21-1	0	0
chr21.fa	32185015	32185570	KRTAP8-1	0	0
chr21.fa	32201358	32202051	KRTAP7-1	0	0
chr21.fa	32252964	32253874	KRTAP11-1	0	0
chr21.fa	32410478	32410795	KRTAP19-8	0	0
chr21.fa	32490736	32931290	TIAM1	33.293724	10776
chr21.fa	33031935	33041243	SOD1	223.9582712	9718
chr21.fa	33043313	33104431	SFRS15	17.02448206	3268
chr21.fa	33245628	33376377	HUNK	0.915361691	304
chr21.fa	33452629	33528816	NCRNA00159	0	0
chr21.fa	33640530	33651376	C21orf45	4.303489814	304
chr21.fa	33664124	33679050	MRAP	0	0
chr21.fa	33683330	33765312	URB1	9.301506975	4170
chr21.fa	33749496	33749631	SNORA80	0	0
chr21.fa	33765442	33766266	C21orf119	16.38955552	608

chr21.fa	33784752	33887697	C21orf63	0.874441732	76
chr21.fa	33947151	33957845	TCP10L	0	0
chr21.fa	33973984	33984918	C21orf59	36.7172123	2356
chr21.fa	34001069	34100351	SYNJ1	4.693563771	1520
chr21.fa	34106210	34144169	GCFC1	3.934320619	760
chr21.fa	34144411	34170016	C21orf49	1.406178807	76
chr21.fa	34162984	34186053	C21orf62	0	0
chr21.fa	34398239	34401500	OLIG2	0	0
chr21.fa	34442450	34444728	OLIG1	0	0
chr21.fa	34537776	34542541	C21orf54	0	0
chr21.fa	34602231	34636820	IFNAR2	2.157193489	304
chr21.fa	34638672	34669520	IL10RB	6.114420389	532
chr21.fa	34697214	34732128	IFNAR1	4.437591636	1216
chr21.fa	34775202	34809828	IFNGR2	25.11284568	2508
chr21.fa	34821448	34852281	TMEM50B	5.798180272	608
chr21.fa	34860238	34864023	DNAJC28	2.501454882	228
chr21.fa	34876238	34915198	GART	12.98074502	2584
chr21.fa	34915350	34949812	SON	44.44441282	18392
chr21.fa	34950211	34961014	DONSON	0	0
chr21.fa	34961648	35014160	CRYZL1	8.895643252	684
chr21.fa	35014784	35261609	ITSN1	9.073556117	3244
chr21.fa	35275757	35288158	ATP5O	146.5408224	5311
chr21.fa	35445823	35515334	MRPS6	24.17880314	885
chr21.fa	35467162	35478561	SLC5A3	1.186011637	608
chr21.fa	35552978	35562220	C21orf82	0	0
chr21.fa	35736323	35743440	KCNE2	0	0
chr21.fa	35747749	35761452	FAM165B	15.02385189	608
chr21.fa	35818988	35883613	KCNE1	0	0
chr21.fa	35888784	35987382	RCAN1	9.328193905	1216
chr21.fa	36041688	36090519	CLIC6	28.46605841	4864
chr21.fa	36096105	36109479	NCRNA00160	0	0
chr21.fa	36160098	36421595	RUNX1	8.894531297	3192
chr21.fa	36410233	36411723	C21orf96	0	0
chr21.fa	37093013	37093106	MIR802	0	0
chr21.fa	37406839	37432816	SETD4	4.343520209	608
chr21.fa	37442285	37445462	CBR1	42.08462105	2356
chr21.fa	37507263	37518860	CBR3	4.559906731	228
chr21.fa	37536839	37666572	DOPEY2	23.9303923	8263
chr21.fa	37692487	37748944	MORC3	28.66042821	5472
chr21.fa	37757689	37789125	CHAF1B	0.741674256	76
chr21.fa	37832920	37948867	CLDN14	0.72388297	76
chr21.fa	38071991	38122510	SIM2	2.832372811	532
chr21.fa	38123189	38362536	HLCS	13.49891624	3648

chr21.fa	38378863	38391958	DSCR6	0	0
chr21.fa	38437664	38445458	PIGP	13.6746052	760
chr21.fa	38445571	38575408	TTC3	41.29424315	16899
chr21.fa	38580955	38592893	DSCR9	0	0
chr21.fa	38595726	38639833	DSCR3	22.87536901	3344
chr21.fa	38739859	38887679	DYRK1A	22.68522464	6916
chr21.fa	38996786	39288696	KCNJ6	0	0
chr21.fa	39426313	39493454	DSCR4	0	0
chr21.fa	39493545	39528605	DSCR8	0	0
chr21.fa	39578250	39580738	DSCR10	0	0
chr21.fa	39628664	39673746	KCNJ15	13.45866346	1824
chr21.fa	39751950	40033704	ERG	0	0
chr21.fa	40110879	40145401	NCRNA00114	0	0
chr21.fa	40177849	40196878	ETS2	2.301970083	380
chr21.fa	40547384	40555440	PSMG1	4.495190926	228
chr21.fa	40557404	40685556	BRWD1	8.514909721	6478
chr21.fa	40685861	40686888	NCRNA00257	0	0
chr21.fa	40714241	40721047	HMGN1	61.54984522	3609
chr21.fa	40752213	40769815	WRB	6.097963449	456
chr21.fa	40777770	40816128	LCA5L	0.714987326	76
chr21.fa	40817797	40887433	SH3BGR	2.628662581	152
chr21.fa	40969075	40984749	C21orf88	0	0
chr21.fa	41029254	41034815	B3GALT5	1.766007577	228
chr21.fa	41117334	41174023	IGSF5	2.454307973	228
chr21.fa	41239347	41301322	PCP4	3.03986369	76
chr21.fa	41384343	42219039	DSCAM	0	0
chr21.fa	42513427	42519991	C21orf130	0	0
chr21.fa	42539484	42539556	MIR3197	0	0
chr21.fa	42539728	42648524	BACE2	7.341129594	988
chr21.fa	42547158	42557166	PLAC4	0.168794831	76
chr21.fa	42688661	42729654	FAM3B	9.753628044	607
chr21.fa	42733950	42780869	MX2	2.854167137	380
chr21.fa	42792520	42831141	MX1	3.281825187	532
chr21.fa	42836478	42880085	TMPRSS2	170.3502339	25431
chr21.fa	43099462	43117496	NCRNA00111	0	0
chr21.fa	43131680	43135935	C21orf129	0	0
chr21.fa	43136596	43137742	NCRNA00112	0	0
chr21.fa	43159529	43187249	RIPK4	19.5568493	3405
chr21.fa	43218385	43299582	PRDM15	1.769565834	608
chr21.fa	43305219	43373999	C2CD2	6.273430012	1824
chr21.fa	43406940	43430496	ZNF295	4.519876336	1520
chr21.fa	43442113	43445060	C21orf121	0	0
chr21.fa	43483068	43563105	UMODL1	0	0

chr21.fa	43522244	43528644	C21orf128	0	0
chr21.fa	43619799	43717354	ABCG1	3.788654461	608
chr21.fa	43731777	43735706	TFF3	0	0
chr21.fa	43766467	43771208	TFF2	0	0
chr21.fa	43782391	43786644	TFF1	3.435275033	76
chr21.fa	43791996	43816200	TMPRSS3	1.285198059	152
chr21.fa	43824019	43867774	UBASH3A	0	0
chr21.fa	43892597	43916401	RSPH1	4.949313514	304
chr21.fa	43919742	44001550	SLC37A1	21.08912385	2934
chr21.fa	44073862	44195618	PDE9A	43.14275782	4297
chr21.fa	44263204	44299678	WDR4	2.182768463	228
chr21.fa	44313378	44329773	NDUFV3	19.83772923	1900
chr21.fa	44394643	44453688	PKNOX1	5.774384426	1292
chr21.fa	44473301	44496472	CBS	10.08454597	1140
chr21.fa	44513066	44527688	U2AF1	39.26737083	1801
chr21.fa	44589141	44592913	CRYAA	13.65481239	684
chr21.fa	44834398	44847002	SIK1	11.14090361	2356
chr21.fa	44869904	44873771	C21orf125	0	0
chr21.fa	44881974	44898103	C21orf84	0	0
chr21.fa	44949072	45079374	HSF2BP	0	0
chr21.fa	45079432	45115960	RRP1B	9.967568264	2280
chr21.fa	45138978	45182188	PDXK	25.42085733	8437
chr21.fa	45193831	45196259	CSTB	285.1202664	8436
chr21.fa	45209418	45223983	RRP1	5.612038937	456
chr21.fa	45225639	45232448	LOC284837	0.735224915	152
chr21.fa	45285116	45407475	AGPAT3	6.393521196	1900
chr21.fa	45432206	45526432	TRAPPC10	14.06668067	4408
chr21.fa	45527208	45551063	PWP2	8.295409724	1216
chr21.fa	45553494	45565605	C21orf33	85.07415141	6377
chr21.fa	45646722	45660834	ICOSLG	1.565855603	228
chr21.fa	45666223	45682099	DNMT3L	0	0
chr21.fa	45705763	45718110	AIRE	0	0
chr21.fa	45719925	45747256	PFKL	59.08152661	9022
chr21.fa	45748827	45759285	C21orf2	14.21190205	1427
chr21.fa	45773484	45862964	TRPM2	0	0
chr21.fa	45875393	45878739	LRRC3	1.349469081	152
chr21.fa	45917775	46131495	C21orf29	0	0
chr21.fa	45937098	45938860	C21orf90	0	0
chr21.fa	45959068	45960078	KRTAP10-1	0	0
chr21.fa	45970318	45971388	KRTAP10-2	0	0
chr21.fa	45977906	45978643	KRTAP10-3	0	0
chr21.fa	45993606	45994987	KRTAP10-4	0	0
chr21.fa	45999564	46000481	KRTAP10-5	0	0

chr21.fa	46011149	46012386	KRTAP10-6	0	0
chr21.fa	46020497	46022091	KRTAP10-7	0	0
chr21.fa	46031996	46032871	KRTAP10-8	0	0
chr21.fa	46047040	46048295	KRTAP10-9	0	0
chr21.fa	46057273	46058372	KRTAP10-10	0	0
chr21.fa	46066331	46067566	KRTAP10-11	0	0
chr21.fa	46074130	46074576	KRTAP12-4	0	0
chr21.fa	46077849	46078258	KRTAP12-3	0	0
chr21.fa	46086106	46086844	KRTAP12-2	0	0
chr21.fa	46101491	46102078	KRTAP12-1	0	0
chr21.fa	46117087	46117959	KRTAP10-12	0	0
chr21.fa	46188955	46221738	UBE2G2	14.52703021	1976
chr21.fa	46225532	46238044	SUMO3	83.24587433	6764
chr21.fa	46269500	46293818	PTTG1IP	8.931892998	1135
chr21.fa	46305868	46348753	ITGB2	0.537741635	76
chr21.fa	46353199	46359828	C21orf67	0.773476181	76
chr21.fa	46359955	46396888	C21orf70	7.673826651	304
chr21.fa	46409779	46414001	NCRNA00163	0	0
chr21.fa	46419127	46424642	NCRNA00162	0	0
chr21.fa	46490871	46493126	C21orf122	0	0
chr21.fa	46494493	46646478	ADARB1	7.752997876	1292
chr21.fa	46683843	46707811	POFUT2	2.088697036	453
chr21.fa	46707967	46717269	LOC642852	0.707648421	228
chr21.fa	46825097	46933634	COL18A1	1.941251749	528
chr21.fa	46839631	46844985	NCRNA00175	0	0
chr21.fa	46934629	46962351	SLC19A1	2.382253263	304
chr21.fa	47269875	47362368	PCBP3	0	0
chr21.fa	47401663	47424963	COL6A1	1.200022275	228
chr21.fa	47518033	47552763	COL6A2	0.778591176	152
chr21.fa	47556176	47575481	FTCD	4.005040983	380
chr21.fa	47581062	47604373	C21orf56	8.757315999	912
chr21.fa	47608360	47648738	LSS	41.79062004	8708
chr21.fa	47649158	47671604	MCM3AP-AS1	0.855316099	76
chr21.fa	47655048	47705236	MCM3AP	23.13645614	6008
chr21.fa	47706267	47717665	YBEY	5.776385946	380
chr21.fa	47721047	47743785	C21orf58	0.569543559	76
chr21.fa	47744036	47865682	PCNT	16.6393007	7901
chr21.fa	47878862	47989926	DIP2A	9.811894507	3268
chr21.fa	48018531	48025035	S100B	0	0
chr21.fa	48055527	48084863	PRMT2	33.9544479	3420
chr22.fa	16256332	16287937	POTEH	4.978446746	456
chr22.fa	16448824	16449804	OR11H1	0	0
chr22.fa	17071648	17073700	CCT8L2	0	0

chr22.fa	17082801	17129720	psiTPTE22	0	0
chr22.fa	17264306	17302584	XKR3	0	0
chr22.fa	17308364	17310225	HSFY1P1	0	0
chr22.fa	17442827	17489112	GAB4	0	0
chr22.fa	17517460	17539682	CECR7	0.620026335	76
chr22.fa	17565851	17596584	IL17RA	0.785485299	304
chr22.fa	17597189	17602257	CECR6	1.333679315	304
chr22.fa	17618410	17646158	CECR5	25.42975297	2128
chr22.fa	17640279	17646335	CECR4	0	0
chr22.fa	17660192	17690779	CECR1	4.187846452	760
chr22.fa	17956628	18033845	CECR2	1.799366239	456
chr22.fa	18043183	18073647	SLC25A18	3.23934849	304
chr22.fa	18074903	18111588	ATP6V1E1	87.54758502	5531
chr22.fa	18121485	18211989	BCL2L13	30.63904166	4636
chr22.fa	18216906	18257258	BID	13.64080175	1596
chr22.fa	18246946	18247025	MIR3198	0	0
chr22.fa	18270416	18507325	MICAL3	2.40226846	1596
chr22.fa	18463634	18463727	MIR648	0	0
chr22.fa	18512151	18520734	FLJ41941	0	0
chr22.fa	18560686	18573797	PEX26	12.48792639	2432
chr22.fa	18593453	18614498	TUBA8	0	0
chr22.fa	18632758	18660162	USP18	0.830630689	76
chr22.fa	18761202	18779474	GGT3P	0	0
chr22.fa	18893736	18899601	DGCR6	2.784558729	152
chr22.fa	18900287	18923964	PRODH	2.931781625	304
chr22.fa	18958027	18982141	DGCR5	0	0
chr22.fa	19005347	19007761	DGCR9	0	0
chr22.fa	19010137	19011063	DGCR10	0	0
chr22.fa	19023795	19109967	DGCR2	5.654070851	1140
chr22.fa	19033675	19035888	DGCR11	0	0
chr22.fa	19117792	19132190	DGCR14	2.367797843	380
chr22.fa	19136504	19137796	GSC2	0	0
chr22.fa	19163094	19166301	SLC25A1	79.88131967	6232
chr22.fa	19166987	19279239	CLTCL1	1.226042031	304
chr22.fa	19318224	19419219	HIRA	11.79295426	2128
chr22.fa	19420036	19423596	MRPL40	34.93897322	1216
chr22.fa	19428410	19435755	C22orf39	10.31694465	1292
chr22.fa	19437464	19466738	UFD1L	14.11938736	1132
chr22.fa	19467414	19508135	CDC45	0	0
chr22.fa	19510547	19512860	CLDN5	0	0
chr22.fa	19553653	19554362	LOC150185	0	0
chr22.fa	19701987	19702314	5-Sep	9.44228053	76
chr22.fa	19704743	19711065	SEPT5-GP1BB	0	0

chr22.fa	19744226	19771112	TBX1	2.411164103	304
chr22.fa	19775934	19842462	GNB1L	1.21336574	76
chr22.fa	19833661	19842342	C22orf29	9.867269886	2888
chr22.fa	19863040	19929262	TXNRD2	26.11049207	2192
chr22.fa	19929360	19957401	COMT	35.82386733	3800
chr22.fa	19957499	20004309	ARVCF	2.574621548	456
chr22.fa	20008631	20053447	C22orf25	7.442317536	760
chr22.fa	20020662	20020743	MIR185	0	0
chr22.fa	20067755	20099397	DGCR8	10.5053099	2052
chr22.fa	20099401	20104768	TRMT2A	5.241757786	684
chr22.fa	20105024	20114704	RANBP1	30.59144997	1216
chr22.fa	20119364	20135530	ZDHHC8	0.67451215	152
chr22.fa	20193855	20196060	LOC150197	0	0
chr22.fa	20228938	20255816	RTN4R	0	0
chr22.fa	20236657	20236734	MIR1286	0	0
chr22.fa	20301761	20307628	DGCR6L	33.33130809	1824
chr22.fa	20383731	20398695	PI4KAP1	0	0
chr22.fa	20455994	20461786	RIMBP3	0	0
chr22.fa	20748480	20762752	ZNF74	3.495765407	608
chr22.fa	20778874	20792146	SCARF2	0	0
chr22.fa	20795806	20850170	KLHL22	7.106951785	836
chr22.fa	20861886	20941919	MED15	18.75335032	2846
chr22.fa	21043843	21046009	POM121L4P	0	0
chr22.fa	21055402	21058891	TMEM191A	2.589744142	228
chr22.fa	21061979	21213070	PI4KA	21.11425404	6382
chr22.fa	21128383	21142008	SERPIND1	0	0
chr22.fa	21213292	21245501	SNAP29	11.89992437	2280
chr22.fa	21271714	21308037	CRKL	23.48783405	5624
chr22.fa	21319418	21335649	AIFM3	0	0
chr22.fa	21336558	21353326	LZTR1	8.988380333	1744
chr22.fa	21354061	21356404	THAP7	7.839285616	380
chr22.fa	21356405	21364663	FLJ39582	0.849311539	76
chr22.fa	21362496	21368576	MGC16703	0	0
chr22.fa	21369442	21382302	P2RX6	0	0
chr22.fa	21383007	21386847	SLC7A4	0	0
chr22.fa	21396681	21398538	P2RX6P	0	0
chr22.fa	21400249	21418457	LOC400891	0	0
chr22.fa	21457305	21476575	BCRP2	0	0
chr22.fa	21636714	21652015	POM121L8P	0	0
chr22.fa	21737663	21738039	RIMBP3C	0	0
chr22.fa	21771693	21805750	HIC2	0.497488849	152
chr22.fa	21827287	21871780	PI4KAP2	0	0
chr22.fa	21921957	21978323	UBE2L3	36.42610238	4940

chr22.fa	21982378	21984340	YDJC	3.789544025	228
chr22.fa	21987086	21991616	CCDC116	0	0
chr22.fa	21996542	21998588	SDF2L1	9.669119433	380
chr22.fa	22007270	22007347	MIR301B	0	0
chr22.fa	22007593	22007674	MIR130B	0	0
chr22.fa	22020273	22051825	PPIL2	12.50171464	2094
chr22.fa	22052203	22090071	YPEL1	0.785485299	138
chr22.fa	22113947	22221970	MAPK1	31.13475138	8512
chr22.fa	22273792	22307250	PPM1F	6.845419873	1596
chr22.fa	22311403	22337147	TOP3B	3.2640339	456
chr22.fa	22599200	22599926	VPREB1	0	0
chr22.fa	22652463	22677324	LOC96610	5.042717768	456
chr22.fa	22838772	22863505	ZNF280B	0.295112965	76
chr22.fa	22868061	22874613	ZNF280A	0	0
chr22.fa	22890123	22901696	PRAME	0	0
chr22.fa	22901756	22909007	LOC648691	0	0
chr22.fa	22974028	22987012	POM121L1P	0	0
chr22.fa	22988782	22990368	GGTLC2	0	0
chr22.fa	23165270	23165365	MIR650	0	0
chr22.fa	23229960	23238013	IGLL5	0	0
chr22.fa	23401593	23484241	RTDR1	0	0
chr22.fa	23412669	23467221	GNAZ	1.053688943	152
chr22.fa	23487513	23506531	RAB36	3.537797321	608
chr22.fa	23522552	23660224	BCR	12.68785597	3952
chr22.fa	23604954	23607186	FBXW4P1	0	0
chr22.fa	23732792	23744799	ZDHHC8P1	23.21673932	2660
chr22.fa	23915313	23922495	IGLL1	0	0
chr22.fa	23950639	23974508	C22orf43	0	0
chr22.fa	23980675	24059610	LOC91316	4.692007033	684
chr22.fa	24033048	24041363	RGL4	0.623362201	76
chr22.fa	24083772	24093279	ZNF70	2.103819629	380
chr22.fa	24094930	24096630	VPREB3	0	0
chr22.fa	24105208	24108020	C22orf15	0	0
chr22.fa	24108051	24110141	CHCHD10	41.47660384	1216
chr22.fa	24115036	24126503	MMP11	0.747456424	76
chr22.fa	24129150	24176689	SMARCB1	33.06199249	2508
chr22.fa	24176706	24181199	DERL3	0.530847511	76
chr22.fa	24199059	24227725	SLC2A11	3.293389523	380
chr22.fa	24236565	24237409	MIF	409.7386656	10336
chr22.fa	24309026	24314748	DDTL	0	0
chr22.fa	24315957	24322019	DDT	9.058655915	189
chr22.fa	24322314	24326106	GSTT2	0	0
chr22.fa	24340595	24347258	GSTTP1	0	0

chr22.fa	24373117	24374043	LOC391322	3.002057206	76
chr22.fa	24376139	24384284	GSTT1	143.5051841	6840
chr22.fa	24385938	24401899	GSTTP2	0	0
chr22.fa	24407765	24574596	CABIN1	12.70809356	4332
chr22.fa	24577444	24585074	SUSD2	1.592987315	228
chr22.fa	24615622	24641110	GGT5	0	0
chr22.fa	24647589	24661492	POM121L9P	0	0
chr22.fa	24666786	24813708	SPECC1L	32.41705836	9880
chr22.fa	24823530	24838325	ADORA2A	0	0
chr22.fa	24825178	24890783	C22orf45	1.403732505	228
chr22.fa	24891251	24922553	UPB1	4.088437639	380
chr22.fa	24936406	24951275	C22orf13	47.79517924	7752
chr22.fa	24951618	24968509	SNRPD3	78.10174623	4864
chr22.fa	24979718	25024972	GGT1	9.743398054	1140
chr22.fa	24981591	24989035	C22orf36	10.9042795	608
chr22.fa	25028882	25049017	BCRP3	0	0
chr22.fa	25041133	25055114	POM121L10P	0	0
chr22.fa	25115001	25170683	PIWIL3	0	0
chr22.fa	25160468	25161986	TOP1P2	0	0
chr22.fa	25202136	25322813	SGSM1	6.773365163	1881
chr22.fa	25331208	25335314	TMEM211	0	0
chr22.fa	25423941	25593415	KIAA1671	18.03880778	8512
chr22.fa	25595825	25603324	CRYBB3	0	0
chr22.fa	25615612	25627836	CRYBB2	0	0
chr22.fa	25714224	25716193	IGLL3P	0	0
chr22.fa	25747385	25777544	LRP5L	3.047202596	228
chr22.fa	25844054	25857645	CRYBB2P1	7.882207094	532
chr22.fa	25960861	26125258	ADRBK2	21.94688625	8936
chr22.fa	26138120	26427007	MYO18B	0	0
chr22.fa	26565440	26779563	SEZ6L	0	0
chr22.fa	26825280	26840978	ASPHD2	0	0
chr22.fa	26847446	26879820	HPS4	6.733779551	1444
chr22.fa	26879850	26887893	SRRD	19.63713248	1132
chr22.fa	26887905	26908437	TFIP11	11.01658699	1444
chr22.fa	26921714	26986089	TPST2	3.258251732	304
chr22.fa	26951178	26951289	MIR548J	0	0
chr22.fa	26995362	27013991	CRYBB1	0	0
chr22.fa	27017928	27026636	CRYBA4	0	0
chr22.fa	27053446	27072440	MIAT	0	0
chr22.fa	28144265	28197486	MN1	1.342130176	456
chr22.fa	28247657	28315255	PITPNB	18.49070645	2432
chr22.fa	28315364	28398667	TTC28-AS1	0.312459469	76
chr22.fa	28316513	28316600	MIR3199-1	0	0

chr22.fa	28374002	29075853	TTC28	4.182064284	2128
chr22.fa	29083731	29137822	CHEK2	3.402361153	304
chr22.fa	29138043	29153496	HSCB	1.553401703	76
chr22.fa	29168662	29185283	CCDC117	7.63446343	1368
chr22.fa	29190548	29196560	XBP1	44.13106378	3560
chr22.fa	29279890	29453475	ZNRF3	1.033673746	304
chr22.fa	29454660	29457907	C22orf31	0	0
chr22.fa	29469066	29564321	KREMEN1	8.57651205	1064
chr22.fa	29601953	29655586	EMID1	0	0
chr22.fa	29655844	29663914	RHBDD3	4.862358602	380
chr22.fa	29663998	29696515	EWSR1	37.20069052	4938
chr22.fa	29702997	29708774	GAS2L1	8.026094124	912
chr22.fa	29708922	29711748	RASL10A	0	0
chr22.fa	29723669	29784572	AP1B1	84.47992244	15871
chr22.fa	29729147	29729256	MIR3653	0	0
chr22.fa	29833004	29837530	RFPL1S	0	0
chr22.fa	29838119	29838444	RFPL1	0	0
chr22.fa	29876181	29887277	NEFH	520.3675537	87020
chr22.fa	29904156	29949736	THOC5	12.27420856	1520
chr22.fa	29950798	29977144	NIPSNAP1	50.87707484	4699
chr22.fa	29999545	30094589	NF2	16.37621205	4631
chr22.fa	30116344	30127820	CABP7	0	0
chr22.fa	30134319	30162969	ZMAT5	10.66365235	304
chr22.fa	30163358	30166402	UQCR10	95.94863051	3952
chr22.fa	30184601	30234254	ASCC2	59.58168416	7448
chr22.fa	30279158	30426857	MTMR3	19.86463855	8056
chr22.fa	30476453	30573062	HORMAD2	0	0
chr22.fa	30636442	30642796	LIF	0.431216307	76
chr22.fa	30658819	30662829	OSM	0	0
chr22.fa	30681107	30685616	GATSL3	12.50438333	912
chr22.fa	30687979	30722894	TBC1D10A	8.730184287	760
chr22.fa	30727977	30749061	SF3A1	31.84417893	6876
chr22.fa	30752627	30772818	CCDC157	1.203802923	152
chr22.fa	30774803	30783302	RNF215	2.64089409	228
chr22.fa	30792933	30821278	SEC14L2	40.4751768	8807
chr22.fa	30821720	30825041	MTFP1	15.90407579	836
chr22.fa	30855216	30868034	SEC14L3	0	0
chr22.fa	30877277	30877743	SDC4P	0	0
chr22.fa	30884898	30901698	SEC14L4	0	0
chr22.fa	30920917	30942669	LOC730005	0	0
chr22.fa	30950624	30960876	GAL3ST1	2.831038465	228
chr22.fa	30972612	31003000	PES1	14.53392434	1824
chr22.fa	31003070	31023047	TCN2	2.447191459	228

chr22.fa	31031793	31043862	SLC35E4	0	0
chr22.fa	31058039	31063872	DUSP18	0.68896757	76
chr22.fa	31090793	31303811	OSBP2	3.894512616	760
chr22.fa	31127544	31127628	MIR3200	0	0
chr22.fa	31318295	31322599	C22orf27	4.847458399	456
chr22.fa	31322641	31364187	MORC2	15.05898968	2997
chr22.fa	31365634	31375380	TUG1	9.278823085	2964
chr22.fa	31477305	31500609	SMTN	3.171074428	456
chr22.fa	31500763	31503551	SELM	69.71048595	2166
chr22.fa	31518961	31530683	INPP5J	6.806056652	684
chr22.fa	31530793	31536469	PLA2G3	0	0
chr22.fa	31556048	31556105	MIR3928	0	0
chr22.fa	31556138	31603005	RNF185	28.72936945	4276
chr22.fa	31608250	31676066	LIMK2	17.45858945	3893
chr22.fa	31677579	31688520	PIK3IP1	7.517930503	836
chr22.fa	31721790	31742249	PATZ1	10.50175164	2244
chr22.fa	31795539	31830172	DRG1	33.80344436	2204
chr22.fa	31835345	31885874	EIF4ENIF1	17.89692227	3116
chr22.fa	31892261	32014476	SFI1	3.626976145	684
chr22.fa	32014535	32026810	PISD	3.224225897	380
chr22.fa	32077334	32146120	C22orf30	19.19946683	9348
chr22.fa	32150069	32303001	DEPDC5	4.482514635	1213
chr22.fa	32329507	32341336	C22orf24	0	0
chr22.fa	32340479	32353590	YWHAH	9.426490763	760
chr22.fa	32439019	32509011	SLC5A1	0.969402723	228
chr22.fa	32545519	32555243	C22orf42	3.508441699	127
chr22.fa	32586422	32600718	RFPL2	4.967549583	608
chr22.fa	32614463	32651318	SLC5A4	0	0
chr22.fa	32750872	32757148	RFPL3	0	0
chr22.fa	32755893	32766972	RFPL3S	0	0
chr22.fa	32783562	32808274	C22orf28	68.70883652	6383
chr22.fa	32809834	32853373	BPIL2	0	0
chr22.fa	32870707	32894818	FBXO7	56.59208085	5891
chr22.fa	32908540	33454377	SYN3	0	0
chr22.fa	33196802	33259028	TIMP3	0.616245686	152
chr22.fa	33669062	34316416	LARGE	3.21866612	608
chr22.fa	35462130	35483380	ISX	0	0
chr22.fa	35653445	35691800	HMGXB4	11.53031039	2204
chr22.fa	35695268	35743987	TOM1	17.15991822	1946
chr22.fa	35731633	35731751	MIR3909	0	0
chr22.fa	35777060	35790207	HMOX1	6.377953821	456
chr22.fa	35796116	35820495	MCM5	5.97475879	684
chr22.fa	35937352	35950045	RASD2	0.568653995	76

chr22.fa	36002811	36019401	MB	15.81867761	988
chr22.fa	36044424	36064456	APOL6	7.001316021	3192
chr22.fa	36113919	36125529	APOL5	0	0
chr22.fa	36134783	36424585	RBFOX2	14.04488635	4621
chr22.fa	36536371	36562225	APOL3	3.394799856	608
chr22.fa	36585176	36600879	APOL4	8.908764326	1368
chr22.fa	36622255	36636000	APOL2	16.36064468	1976
chr22.fa	36649117	36663577	APOL1	3.318297324	456
chr22.fa	36677323	36784063	MYH9	105.8083391	35707
chr22.fa	36863093	36877687	TXN2	69.58038717	4180
chr22.fa	36883233	36903148	FOXRED2	2.33443918	532
chr22.fa	36906897	36925277	EIF3D	91.68361435	8035
chr22.fa	36956916	37098690	CACNG2	0	0
chr22.fa	37154246	37172172	IFT27	12.77792436	836
chr22.fa	37196745	37215517	PVALB	29.5484358	760
chr22.fa	37257030	37274059	NCF4	4.114902177	304
chr22.fa	37309675	37336479	CSF2RB	0.348709215	76
chr22.fa	37387160	37403877	C22orf33	0	0
chr22.fa	37406906	37415491	TST	47.9481843	2432
chr22.fa	37415702	37425863	MPST	34.55735012	2508
chr22.fa	37447779	37459430	KCTD17	6.996645809	532
chr22.fa	37461479	37499693	TMPRSS6	0	0
chr22.fa	37521880	37545962	IL2RB	0	0
chr22.fa	37576206	37584330	C1QTNF6	1.727089137	228
chr22.fa	37602245	37608353	SSTR3	0	0
chr22.fa	37621310	37640305	RAC2	8.059452786	532
chr22.fa	37678495	37711389	CYTH4	0	0
chr22.fa	37764000	37823505	ELFN2	0.404306986	152
chr22.fa	37865101	37882478	MFNG	0	0
chr22.fa	37886400	37915210	CARD10	3.034526304	532
chr22.fa	37956471	37965410	CDC42EP1	3.908745645	380
chr22.fa	37966253	37976024	LGALS2	32.01074985	760
chr22.fa	38004481	38029571	GGA1	11.19316551	1824
chr22.fa	38035684	38052050	SH3BP1	1.968383461	228
chr22.fa	38054737	38062939	PDXP	9.272818526	836
chr22.fa	38071613	38075809	LGALS1	93.89840713	2356
chr22.fa	38082344	38089485	NOL12	2.975592668	380
chr22.fa	38092995	38172563	TRIOBP	4.352860634	2128
chr22.fa	38201114	38203443	H1F0	155.9601966	16340
chr22.fa	38203912	38213183	GCAT	29.55488514	2432
chr22.fa	38219389	38221502	GALR3	0	0
chr22.fa	38226862	38240278	ANKRD54	5.022702571	456
chr22.fa	38240354	38240378	MIR658	0	0

chr22.fa	38243685	38243781	MIR659	0	0
chr22.fa	38245370	38284638	EIF3L	228.1534566	19995
chr22.fa	38302346	38338026	MICALL1	13.11907228	2280
chr22.fa	38339533	38349603	C22orf23	2.166978696	152
chr22.fa	38349674	38365319	POLR2F	9.648881844	912
chr22.fa	38368319	38380539	SOX10	0	0
chr22.fa	38453262	38471708	PICK1	16.70312694	1584
chr22.fa	38474144	38479170	SLC16A8	0	0
chr22.fa	38480896	38506676	BAIAP2L2	3.151726404	304
chr22.fa	38507502	38577836	PLA2G6	5.170370249	760
chr22.fa	38597939	38612517	MAFF	11.47471262	1292
chr22.fa	38615298	38669040	TMEM184B	7.845067784	1368
chr22.fa	38686697	38714089	CSNK1E	18.70820493	2432
chr22.fa	38740670	38794931	LOC400927	0	0
chr22.fa	38822333	38851203	KCNJ4	0	0
chr22.fa	38864083	38879442	KDELR3	0	0
chr22.fa	38879446	38902345	DDX17	80.32632422	18811
chr22.fa	38914954	38966189	DMC1	0	0
chr22.fa	38974125	39052634	LOC646851	0.327582063	152
chr22.fa	39052658	39069855	CBY1	30.58544541	1748
chr22.fa	39077954	39080766	TOMM22	58.83133665	3648
chr22.fa	39081548	39096459	JOSD1	15.25335948	2356
chr22.fa	39101807	39129592	GTPBP1	12.72566245	2888
chr22.fa	39130719	39152024	SUN2	7.146537398	1368
chr22.fa	39174513	39190161	DNAL4	14.77633061	988
chr22.fa	39214457	39240017	NPTXR	1.162882964	304
chr22.fa	39260248	39268258	CBX6	32.4059388	4762
chr22.fa	39353527	39359188	APOBEC3A	1.170444261	76
chr22.fa	39378405	39388154	APOBEC3B	0	0
chr22.fa	39410265	39414825	APOBEC3C	35.05305984	1748
chr22.fa	39417118	39429256	APOBEC3D	6.752682793	760
chr22.fa	39436673	39451975	APOBEC3F	4.031505522	912
chr22.fa	39473010	39483748	APOBEC3G	11.0588413	912
chr22.fa	39493229	39500072	APOBEC3H	0	0
chr22.fa	39526779	39548538	CBX7	19.08449064	3400
chr22.fa	39619685	39640957	PDGFB	0.984970099	152
chr22.fa	39708887	39715670	RPL3	1555.125234	93563
chr22.fa	39709824	39709916	SNORD83B	18.17379916	76
chr22.fa	39711218	39711312	SNORD83A	0	0
chr22.fa	39712847	39712901	RNU86	0	0
chr22.fa	39715057	39715118	SNORD43	0	0
chr22.fa	39745954	39781593	SYNGR1	1.892992884	456
chr22.fa	39795759	39833132	TAB1	10.50330838	1824

chr22.fa	39853325	39888199	MGAT3		4.542560227	1064
chr22.fa	39898284	39914137	SMCR7L		11.56633775	2964
chr22.fa	39916569	39918691	ATF4		214.2471199	13680
chr22.fa	39925098	39928860	RPS19BP1		87.51711744	3648
chr22.fa	39966758	40085740	CACNA1I		0	0
chr22.fa	40139049	40289794	ENTHD1		0	0
chr22.fa	40297086	40367384	GRAP2		0	0
chr22.fa	40390953	40426043	FAM83F		13.87386761	1292
chr22.fa	40440821	40731812	TNRC6B		9.583276475	8056
chr22.fa	40742504	40762575	ADSL		20.54582244	1444
chr22.fa	40766595	40806291	SGSM3		35.02259227	4704
chr22.fa	40806294	41032690	MKL1		14.31064369	2888
chr22.fa	41075182	41078818	MCHR1		0	0
chr22.fa	41165639	41215392	SLC25A17		5.22819193	532
chr22.fa	41220601	41252687	ST13		65.00446828	9336
chr22.fa	41253094	41323880	XPNPEP3		6.0817289	836
chr22.fa	41255554	41258130	DNAJB7		0.655831299	76
chr22.fa	41347351	41369019	RBX1		48.50393961	1976
chr22.fa	41488517	41488570	MIR1281		0	0
chr22.fa	41488614	41576081	EP300		54.59634329	21508
chr22.fa	41601313	41627275	L3MBTL2		10.88448669	1505
chr22.fa	41625514	41636935	CHADL		2.798346976	304
chr22.fa	41641615	41682216	RANGAP1		25.7357631	3496
chr22.fa	41697567	41756151	ZC3H7B		40.23343769	10616
chr22.fa	41763392	41795330	TEF		7.387164548	1520
chr22.fa	41829492	41843027	TOB2		30.36016325	6232
chr22.fa	41855721	41864708	PHF5A		22.15548908	1061
chr22.fa	41865129	41921352	ACO2		50.27817566	4031
chr22.fa	41921803	41940476	POLR3H		12.12542893	1748
chr22.fa	41957014	41972670	CSDC2		3.340314041	380
chr22.fa	41972890	41985871	PMM1		25.02989381	1444
chr22.fa	41994032	42017061	PPPDE2		13.81004137	2356
chr22.fa	42017295	42060052	XRCC6		76.06953653	7296
chr22.fa	42069937	42084913	NHP2L1		60.75457472	4636
chr22.fa	42086547	42094140	C22orf46		11.89058394	2660
chr22.fa	42095518	42195459	MEI1		0	0
chr22.fa	42196678	42222303	CCDC134		3.976797316	228
chr22.fa	42229106	42302375	SREBF2		22.7659526	4408
chr22.fa	42296948	42297016	MIR33A		0	0
chr22.fa	42321036	42322821	TNFRSF13C		0	0
chr22.fa	42334741	42343148	CENPM		1.671713758	76
chr22.fa	42348191	42354946	LOC339674		0	0
chr22.fa	42372931	42394225		3-Sep	1.45199137	304

chr22.fa	42394729	42424477	WBP2NL	0	0
chr22.fa	42454338	42466846	NAGA	3.175299859	532
chr22.fa	42470255	42475442	FAM109B	7.24772534	760
chr22.fa	42475699	42480288	C22orf32	80.8952006	5700
chr22.fa	42481530	42486888	NDUFA6	59.95552356	3192
chr22.fa	42486937	42521354	LOC100132273	1.019440717	76
chr22.fa	42522501	42526883	CYP2D6	0	0
chr22.fa	42536214	42540575	CYP2D7P1	0	0
chr22.fa	42556019	42611445	TCF20	15.72082554	5236
chr22.fa	42665759	42670868	LOC388906	0	0
chr22.fa	42776414	42828401	NFAM1	0.301562306	76
chr22.fa	42896585	42901148	SERHL	0	0
chr22.fa	42904341	42915829	RRP7A	6.79938492	1444
chr22.fa	42949925	42968535	SERHL2	13.43464522	560
chr22.fa	42969266	42978017	RRP7B	0	0
chr22.fa	42979727	43010962	POLDIP3	26.07824537	4028
chr22.fa	43011251	43011399	RNU12	11.34350188	76
chr22.fa	43013846	43045405	CYB5R3	25.95081528	4104
chr22.fa	43035809	43036607	ATP5L2	0	0
chr22.fa	43088127	43116876	A4GALT	8.079245593	760
chr22.fa	43192532	43253408	ARFGAP3	53.07407633	6761
chr22.fa	43265772	43411184	PACSIN2	34.62095397	5472
chr22.fa	43435523	43485434	TTLL1	6.306566283	532
chr22.fa	43506757	43525718	BIK	5.337385951	228
chr22.fa	43528212	43539403	MCAT	9.029522683	836
chr22.fa	43547535	43559248	TSPO	115.1503212	4484
chr22.fa	43562628	43583137	TTLL12	31.47523213	4788
chr22.fa	43599230	43739355	SCUBE1	0	0
chr22.fa	43808020	43902800	MPPED1	0	0
chr22.fa	43924624	44208217	EFCAB6	2.40026694	532
chr22.fa	44220387	44258378	SULT4A1	0	0
chr22.fa	44275558	44287893	PNPLA5	0	0
chr22.fa	44319619	44343448	PNPLA3	0.602457439	76
chr22.fa	44351261	44392412	SAMM50	28.81209893	2275
chr22.fa	44395173	44565103	PARVB	2.598862176	228
chr22.fa	44568836	44603035	PARVG	0	0
chr22.fa	44639557	44708731	KIAA1644	0	0
chr22.fa	44888450	44894005	LDOC1L	9.523898057	2280
chr22.fa	44965220	44968329	NCRNA00207	0	0
chr22.fa	45064427	45133561	PRR5	5.674308439	718
chr22.fa	45148438	45204978	ARHGAP8	1.773568873	13
chr22.fa	45277043	45405809	PHF21B	0.966066857	152
chr22.fa	45559726	45583890	NUP50	12.90446488	3192

chr22.fa	45588123	45636650	C22orf9	13.16955505	3800
chr22.fa	45596835	45596900	MIR1249	0	0
chr22.fa	45680868	45691755	UPK3A	56.55449676	2660
chr22.fa	45705081	45737836	FAM118A	12.93760115	1957
chr22.fa	45739945	45809500	SMC1B	0	0
chr22.fa	45809574	45828295	RIBC2	0	0
chr22.fa	45898719	45997014	FBLN1	0.696084084	152
chr22.fa	46067678	46241187	ATXN10	39.0687756	5850
chr22.fa	46316248	46373008	WNT7B	3.024296315	532
chr22.fa	46402496	46406657	LOC730668	0	0
chr22.fa	46435787	46440748	LOC100271722	0	0
chr22.fa	46446339	46450024	C22orf26	0	0
chr22.fa	46452727	46454402	LOC150381	2.166978696	152
chr22.fa	46481877	46509808	LOC400931	0	0
chr22.fa	46486924	46487006	MIR3619	0	0
chr22.fa	46546499	46639653	PPARA	8.241368691	3724
chr22.fa	46639910	46646193	C22orf40	3.398580504	228
chr22.fa	46651560	46659219	PKDREJ	0	0
chr22.fa	46663861	46689905	TTC38	22.55445868	2647
chr22.fa	46691040	46692557	CN5H6-4	0	0
chr22.fa	46692638	46726707	GTSE1	0	0
chr22.fa	46731298	46753237	TRMU	5.163476126	456
chr22.fa	46756731	46933067	CELSR1	1.484015686	760
chr22.fa	47022658	47075688	GRAMD4	1.174892083	228
chr22.fa	47080307	47134152	CERK	2.661798852	532
chr22.fa	47158518	47571342	TBC1D22A	7.123853507	1215
chr22.fa	48016792	48027318	FLJ46257	0	0
chr22.fa	48670176	48670227	MIR3201	0	0
chr22.fa	48885288	49147744	FAM19A5	2.358012635	304
chr22.fa	50013290	50051190	C22orf34	0	0
chr22.fa	50166937	50218452	BRD1	17.97275763	3724
chr22.fa	50171538	50173958	LOC90834	0	0
chr22.fa	50247497	50283726	ZBED4	7.118071339	2204
chr22.fa	50296854	50312106	ALG12	4.985785652	532
chr22.fa	50312283	50321186	CRELD2	3.219333293	228
chr22.fa	50354143	50357720	PIM3	25.5763087	2736
chr22.fa	50432942	50451055	IL17REL	0	0
chr22.fa	50453552	50493055	TTLL8	0	0
chr22.fa	50497820	50524358	MLC1	0	0
chr22.fa	50528435	50600116	MOV10L1	0	0
chr22.fa	50609160	50618724	PANX2	7.280416828	988
chr22.fa	50624360	50638027	TRABD	15.33208593	1596
chr22.fa	50639408	50656045	SELO	2.19032976	228

chr22.fa	50656118	50683400	TUBGCP6	5.054504496	1368
chr22.fa	50683613	50689834	HDAC10	6.246075909	760
chr22.fa	50691331	50700089	MAPK12	1.927241111	152
chr22.fa	50702142	50708779	MAPK11	4.246557697	456
chr22.fa	50713408	50746002	PLXNB2	22.6638751	6457
chr22.fa	50750392	50765489	FAM116B	0	0
chr22.fa	50781760	50883430	PPP6R2	29.21151331	5149
chr22.fa	50883515	50913464	SBF1	15.55314266	5548
chr22.fa	50920012	50924866	ADM2	1.990400178	380
chr22.fa	50925213	50928750	MIOX	74.00663686	4782
chr22.fa	50941376	50946135	LMF2	0	0
chr22.fa	50946645	50963209	NCAPH2	6.78515189	760
chr22.fa	50963871	50964034	SCO2	0	0
chr22.fa	50964182	50968514	TYMP	3.94277148	228
chr22.fa	50968838	50971008	ODF3B	5.227302366	228
chr22.fa	50986462	50989452	KLHDC7B	0	0
chr22.fa	50989541	51001328	C22orf41	0	0
chr22.fa	51007596	51007764	CHKB-CPT1B	0	0
chr22.fa	51016815	51017096	CPT1B	0	0
chr22.fa	51017387	51017464	CHKB	21.6688974	76
chr22.fa	51021455	51022355	LOC100144603	2.695602296	76
chr22.fa	51039131	51049979	MAPK8IP2	1.46844831	228
chr22.fa	51061182	51066601	ARSA	3.133490336	608
chr22.fa	51113070	51171641	SHANK3	1.18267577	380
chr22.fa	51176652	51183727	ACR	0	0
chr22.fa	51195514	51238065	RPL23AP82	0	0
chr22.fa	51205920	51222087	RABL2B	2.070460967	228
chr3.fa	238650	451097	CHL1	0.221056735	76
chr3.fa	1134629	1445278	CNTN6	0	0
chr3.fa	2142247	3098040	CNTN4	0.43610891	76
chr3.fa	3111401	3152058	IL5RA	0.580218331	76
chr3.fa	3168600	3190706	TRNT1	14.1158291	1444
chr3.fa	3191317	3221401	CRBN	24.81706554	2888
chr3.fa	3841121	3889387	LRRN1	3.094794287	532
chr3.fa	4344988	4358949	SETMAR	11.28189955	1216
chr3.fa	4402829	4508966	SUMF1	3.128597732	304
chr3.fa	4535032	4889524	ITPR1	3.300506038	1520
chr3.fa	4790878	4793274	EGOT	2.312200072	152
chr3.fa	5021097	5026865	BHLHE40	11.14157078	1520
chr3.fa	5163930	5222601	ARL8B	18.11909096	2432
chr3.fa	5229359	5261650	EDEM1	8.240701518	2280
chr3.fa	6902802	7783218	GRM7	0	0
chr3.fa	8262834	8543344	LOC100288428	0	0

chr3.fa	8543511	8609806	LMCD1	2.924220328	228
chr3.fa	8613468	8615580	LOH3CR2A	0.799940719	76
chr3.fa	8661320	8693737	C3orf32	0	0
chr3.fa	8775486	8788451	CAV3	0	0
chr3.fa	8792095	8811300	OXTR	0	0
chr3.fa	8918880	9005159	RAD18	2.356011115	608
chr3.fa	9022278	9291311	SRGAP3	3.973683841	1596
chr3.fa	9404717	9428475	THUMPD3	14.43874095	2584
chr3.fa	9430537	9439174	LOC440944	1.817379916	152
chr3.fa	9439403	9519838	SETD5	31.1941298	9576
chr3.fa	9540045	9595486	LHFPL4	0	0
chr3.fa	9691117	9744078	MTMR14	12.71810116	1444
chr3.fa	9745510	9771592	CPNE9	0	0
chr3.fa	9773434	9789699	BRPF1	6.81806577	1444
chr3.fa	9791628	9808353	OGG1	5.467707125	608
chr3.fa	9799090	9811661	CAMK1	18.51049926	1117
chr3.fa	9821654	9834178	TADA3	50.84549531	6612
chr3.fa	9834809	9848789	ARPC4	97.44443292	4925
chr3.fa	9851644	9874929	TTLL3	0	0
chr3.fa	9879533	9885702	RPUSD3	17.90737465	988
chr3.fa	9908394	9921938	CIDEC	0	0
chr3.fa	9932271	9936031	JAGN1	8.155303342	608
chr3.fa	9944296	9958084	IL17RE	1.775348002	228
chr3.fa	9958764	9975314	IL17RC	10.27958295	1126
chr3.fa	9975524	9987097	CRELD1	1.169777088	152
chr3.fa	9987226	9994078	PRRT3	0.449452375	76
chr3.fa	10005636	10028522	TMEM111	10.95476227	532
chr3.fa	10048102	10052779	LOC401052	0	0
chr3.fa	10059237	10067820	CIDECP	0	0
chr3.fa	10068113	10143614	FANCD2	2.035100785	456
chr3.fa	10123004	10149915	C3orf24	0	0
chr3.fa	10157333	10168874	C3orf10	247.2021423	13072
chr3.fa	10183319	10193746	VHL	23.47471298	3116
chr3.fa	10206563	10285427	IRAK2	5.359402668	836
chr3.fa	10290177	10322906	TATDN2	17.22196534	3800
chr3.fa	10326103	10327430	C3orf42	0	0
chr3.fa	10327434	10333135	GHRL	0	0
chr3.fa	10329098	10335133	GHRLOS	0	0
chr3.fa	10342615	10362858	SEC13	43.4523262	3177
chr3.fa	10365707	10547268	ATP2B2	0	0
chr3.fa	10436173	10436246	MIR885	0	0
chr3.fa	10801169	10805877	LOC285370	0	0
chr3.fa	10857917	10980146	SLC6A11	0.848866757	76

chr3.fa	11034420	11080935	SLC6A1	0	0
chr3.fa	11178779	11304939	HRH1	1.052354597	228
chr3.fa	11314010	11597543	ATG7	10.27313361	1596
chr3.fa	11599140	11762220	VGLL4	17.94473635	2432
chr3.fa	11831919	11888352	C3orf31	2.697826207	152
chr3.fa	12045862	12233532	SYN2	0.509497967	76
chr3.fa	12194568	12200647	TIMP4	1.18267577	76
chr3.fa	12329349	12475855	PPARG	95.5996989	9728
chr3.fa	12525931	12574820	TSEN2	2.956244644	304
chr3.fa	12581280	12586963	LOC100129480	4.677551613	228
chr3.fa	12598594	12625099	MKRN2	16.55790557	1976
chr3.fa	12625211	12705700	RAF1	36.85909781	5244
chr3.fa	12775392	12800808	TMEM40	0.988305965	76
chr3.fa	12838171	12876313	CAND2	1.840286198	380
chr3.fa	12876444	12883081	RPL32	496.5072146	36860
chr3.fa	12881811	12881949	SNORA7A	0	0
chr3.fa	12938719	13114617	IQSEC1	21.13938423	4940
chr3.fa	13357737	13461809	NUP210	0.235067373	76
chr3.fa	13521715	13547924	HDAC11	11.77227189	1596
chr3.fa	13590625	13679922	FBLN2	2.536147891	532
chr3.fa	13692221	13788132	LOC285375	0	0
chr3.fa	13860082	13921618	WNT7A	0	0
chr3.fa	13974553	13978444	LOC100132526	0	0
chr3.fa	13978807	14107481	TPRXL	0.747901206	76
chr3.fa	14153577	14166371	CHCHD4	14.75208999	1064
chr3.fa	14166440	14185180	TMEM43	2.022869275	304
chr3.fa	14186648	14220172	XPC	18.62814414	3116
chr3.fa	14220228	14239869	LSM3	65.66141154	2052
chr3.fa	14444106	14530618	SLC6A6	1.348134735	380
chr3.fa	14530858	14583588	GRIP2	0	0
chr3.fa	14693253	14714166	C3orf19	16.55901752	2204
chr3.fa	14716606	14814543	C3orf20	0.50794123	76
chr3.fa	14860469	14976072	FGD5	1.719750232	456
chr3.fa	14989236	15090018	NR2C2	12.72988788	4332
chr3.fa	15090781	15106816	MRPS25	7.553290685	1292
chr3.fa	15111580	15140655	ZFYVE20	7.339795247	2204
chr3.fa	15206869	15247466	COL6A4P1	0	0
chr3.fa	15247733	15294423	CAPN7	9.673567254	1900
chr3.fa	15296357	15382901	SH3BP5	25.05191053	3340
chr3.fa	15451377	15469042	METTL6	2.705387504	304
chr3.fa	15469064	15484120	EAF1	19.95959955	4028
chr3.fa	15491640	15563258	COLQ	0	0
chr3.fa	15537746	15537815	MIR4270	0	0

chr3.fa	15602240	15643130	HACL1	11.6621883	1064
chr3.fa	15643255	15687325	BTD	1.637020749	152
chr3.fa	15708744	15901053	ANKRD28	13.97505555	4160
chr3.fa	15915278	15915356	MIR563	0	0
chr3.fa	16216184	16271253	GALNTL2	0	0
chr3.fa	16298568	16306496	DPH3	8.731518634	1596
chr3.fa	16306667	16347594	OXNAD1	5.537315533	988
chr3.fa	16357352	16555222	RFTN1	1.125966045	152
chr3.fa	16628301	16647006	DAZL	0	0
chr3.fa	16926452	17132098	PLCL2	3.941659525	760
chr3.fa	17198654	17784240	TBC1D5	11.56567057	4408
chr3.fa	18389134	18480265	SATB1	23.65084671	6232
chr3.fa	19190017	19577135	KCNH8	0	0
chr3.fa	19920966	19975706	EFHB	0.592005058	76
chr3.fa	19988572	20026667	RAB5A	21.544136	2356
chr3.fa	20021453	20053765	C3orf48	0	0
chr3.fa	20081524	20195896	KAT2B	16.46717001	3572
chr3.fa	20202085	20227724	SGOL1	0	0
chr3.fa	21447218	21448177	VENTXP7	0	0
chr3.fa	21462490	21792816	ZNF385D	0.932708195	76
chr3.fa	23244784	23632296	UBE2E2	12.48414574	988
chr3.fa	23847439	23932807	UBE2E1	42.08128519	2774
chr3.fa	23933572	23958537	NKIRAS1	6.777590594	608
chr3.fa	23958639	23962332	RPL15	391.3458112	35036
chr3.fa	23986751	24022109	NR1D2	10.97299834	2660
chr3.fa	24141465	24144738	LOC152024	0	0
chr3.fa	24158645	24536313	THRB	12.53774199	4332
chr3.fa	25469754	25639422	RARB	0	0
chr3.fa	25639475	25705788	TOP2B	116.2271389	27119
chr3.fa	25760435	25831530	NGLY1	15.9561153	2052
chr3.fa	25831563	25836025	OXSM	4.427361647	304
chr3.fa	25900023	25915186	LOC285326	0	0
chr3.fa	26664300	26752265	LRRC3B	0	0
chr3.fa	27257097	27410912	NEK10	0.581552678	69
chr3.fa	27414214	27498245	SLC4A7	2.832595203	988
chr3.fa	27757886	27763785	EOMES	0	0
chr3.fa	28283124	28361263	CMC1	13.52137774	380
chr3.fa	28363845	28390618	AZI2	13.28275211	2189
chr3.fa	28431987	28566632	ZCWPW2	2.037547087	152
chr3.fa	29322803	30051886	RBMS3	0.202153493	76
chr3.fa	30647994	30735633	TGFBR2	2.515687912	532
chr3.fa	30767692	30936153	GADL1	0	0
chr3.fa	31574491	31677556	STT3B	16.18851398	1806

chr3.fa	31702317	32023265	OSBPL10	7.355585014	1368
chr3.fa	32023343	32033120	ZNF860	3.356770981	456
chr3.fa	32148003	32210207	GPD1L	102.7751471	18772
chr3.fa	32280171	32411813	CMTM8	4.333735001	228
chr3.fa	32433163	32496333	CMTM7	1.250949832	76
chr3.fa	32522804	32544403	CMTM6	4.017050102	608
chr3.fa	32567463	32612366	DYNC1LI1	10.12479876	1140
chr3.fa	32726698	32815354	CNOT10	14.79901451	1824
chr3.fa	32859510	32933771	TRIM71	0	0
chr3.fa	32993066	32996403	CCR4	0	0
chr3.fa	33038100	33138694	GLB1	7.773902638	912
chr3.fa	33131908	33135795	TMPPE	0.434774564	76
chr3.fa	33155450	33189265	CRTAP	10.92718578	3268
chr3.fa	33191537	33260707	SUSD5	0	0
chr3.fa	33318934	33428757	FBXL2	1.147760371	152
chr3.fa	33429829	33481897	UBP1	18.22183564	3420
chr3.fa	33537739	33759848	CLASP2	11.17626379	3648
chr3.fa	33840066	33911195	PDCD6IP	19.77101191	6080
chr3.fa	35683849	35835988	ARPP21	0.311792296	76
chr3.fa	35785968	35786051	MIR128-2	0	0
chr3.fa	36422097	36589496	STAC	1.140866247	152
chr3.fa	36753913	36781352	DCLK3	7.906892505	1900
chr3.fa	36868308	36986548	TRANK1	5.642506515	2660
chr3.fa	37027357	37034795	EPM2AIP1	5.680090607	1900
chr3.fa	37034841	37092337	MLH1	14.61242839	1824
chr3.fa	37094117	37217851	LRRFIP2	26.90976562	4615
chr3.fa	37284682	37408370	GOLGA4	36.64471281	13103
chr3.fa	37440968	37476988	C3orf35	0	0
chr3.fa	37493813	37861281	ITGA9	0.853092188	152
chr3.fa	37903669	38025960	CTDSPL	20.10748962	4028
chr3.fa	38010895	38010971	MIR26A1	0	0
chr3.fa	38035078	38048676	VILL	12.12898718	1520
chr3.fa	38048987	38071154	PLCD1	32.47198895	4560
chr3.fa	38080696	38164200	DLEC1	0.605126132	152
chr3.fa	38164229	38178733	ACAA1	136.5016442	10993
chr3.fa	38179969	38184512	MYD88	28.476066	3648
chr3.fa	38207026	38296979	OXSR1	23.83698805	4848
chr3.fa	38307298	38319806	SLC22A13	0	0
chr3.fa	38347445	38359859	SLC22A14	0	0
chr3.fa	38388251	38456467	XYLB	3.67123197	608
chr3.fa	38492518	38496311	LOC100128640	2.014418414	228
chr3.fa	38495790	38534633	ACVR2B	3.41815092	1748
chr3.fa	38537763	38567796	EXOG	2.686261871	380

chr3.fa	38589553	38691163	SCN5A	0.393187432	152
chr3.fa	38738837	38835501	SCN10A	0	0
chr3.fa	38887260	38992052	SCN11A	0.780147913	228
chr3.fa	39093507	39137881	WDR48	25.6434708	4256
chr3.fa	39138091	39149130	GORASP1	22.11545869	3040
chr3.fa	39149152	39180394	TTC21A	2.77855417	532
chr3.fa	39183342	39195102	CSRNP1	19.08604738	2736
chr3.fa	39224706	39234077	XIRP1	0	0
chr3.fa	39304985	39323226	CX3CR1	0	0
chr3.fa	39371197	39375171	CCR8	0	0
chr3.fa	39424815	39438819	SLC25A38	28.64708475	2736
chr3.fa	39448204	39454032	RPSA	145.8823224	7524
chr3.fa	39449882	39450030	SNORA6	0	0
chr3.fa	39452545	39452698	SNORA62	0	0
chr3.fa	39509070	39567857	MOBP	0	0
chr3.fa	39851303	40301811	MYRIP	15.27359707	3344
chr3.fa	40214638	40351172	FLJ33065	1.613447295	152
chr3.fa	40351190	40353915	EIF1B	27.17863644	1216
chr3.fa	40428673	40470110	ENTPD3	4.841231449	608
chr3.fa	40498801	40503859	RPL14	408.0851879	17359
chr3.fa	40518604	40531728	ZNF619	2.827702599	532
chr3.fa	40547530	40559199	ZNF620	0.746789251	76
chr3.fa	40566376	40581043	ZNF621	4.515873297	1672
chr3.fa	41240942	41281939	CTNNB1	97.94970546	15041
chr3.fa	41288090	42003660	ULK4	4.652866203	912
chr3.fa	42132746	42267268	TRAK1	47.6939913	11628
chr3.fa	42299318	42307662	CCK	2.094479204	152
chr3.fa	42438575	42452065	LYZL4	0	0
chr3.fa	42544104	42579065	VIPR1	7.269519666	912
chr3.fa	42589473	42623428	SEC22C	23.85166586	2204
chr3.fa	42632298	42636490	SS18L2	35.25521334	1292
chr3.fa	42642147	42690233	NKTR	10.09922378	3316
chr3.fa	42695176	42709072	ZBTB47	5.524416851	1368
chr3.fa	42727011	42733938	KBTBD5	0	0
chr3.fa	42734155	42744319	HHATL	0	0
chr3.fa	42749874	42814745	CCDC13	0	0
chr3.fa	42824400	42846027	HIGD1A	30.98196871	4256
chr3.fa	42850964	42908775	CCBP2	0.571100297	76
chr3.fa	42913684	42917633	CYP8B1	0	0
chr3.fa	42947402	42960825	ZNF662	3.446394587	912
chr3.fa	43020759	43099207	FAM198A	0.492373854	76
chr3.fa	43120729	43147565	C3orf39	2.663800372	304
chr3.fa	43328004	43392634	SNRK	8.102374265	1900

chr3.fa	43407818	43663560	ANO10	1.854519227	228
chr3.fa	43732375	43764217	ABHD5	6.946385424	1672
chr3.fa	44155704	44155802	MIR138-1	0	0
chr3.fa	44283378	44373590	C3orf77	0	0
chr3.fa	44379611	44450940	C3orf23	7.105395048	1748
chr3.fa	44481262	44519162	ZNF445	6.761356045	3116
chr3.fa	44596713	44624973	ZNF167	0.850645886	152
chr3.fa	44626456	44637557	ZNF660	0.766582057	76
chr3.fa	44666511	44689963	ZNF197	4.809207133	1368
chr3.fa	44690233	44702283	ZNF35	5.74236011	684
chr3.fa	44754135	44765323	ZNF502	0.517059264	76
chr3.fa	44771124	44778575	ZNF501	0.551752273	76
chr3.fa	44790236	44803173	KIAA1143	9.205434028	2128
chr3.fa	44803209	44894748	KIF15	0	0
chr3.fa	44903380	44903407	MIR564	0	0
chr3.fa	44903474	44907154	TMEM42	14.94067762	608
chr3.fa	44916098	44956088	TGM4	595.7025325	80332
chr3.fa	44956753	45017674	ZDHHC3	8.39348419	4788
chr3.fa	45017741	45054158	EXOSC7	26.68692976	1824
chr3.fa	45067759	45077563	CLEC3B	3.96745689	152
chr3.fa	45123769	45187914	CDCP1	3.791545545	1064
chr3.fa	45265956	45267814	TMEM158	0	0
chr3.fa	45430075	45590328	LARS2	8.021423911	1516
chr3.fa	45636323	45722755	LIMD1	19.56997037	3952
chr3.fa	45729868	45730374	LOC644714	0	0
chr3.fa	45730754	45786900	SACM1L	15.42393344	2571
chr3.fa	45796941	45838035	SLC6A20	0.311125123	76
chr3.fa	45865446	45883621	LZTFL1	5.405882404	826
chr3.fa	45927996	45944667	CCR9	0	0
chr3.fa	45959395	46037307	FYCO1	26.43073523	10108
chr3.fa	45984973	45989845	CXCR6	0	0
chr3.fa	46062291	46068979	XCR1	0	0
chr3.fa	46243200	46249832	CCR1	0	0
chr3.fa	46283872	46308197	CCR3	0	0
chr3.fa	46395235	46402413	CCR2	0	0
chr3.fa	46411633	46417697	CCR5	0	0
chr3.fa	46448721	46451014	CCRL2	0	0
chr3.fa	46477496	46506598	LTF	3.026742616	380
chr3.fa	46539485	46542439	RTP3	0	0
chr3.fa	46556878	46608011	LRRC2	0	0
chr3.fa	46616045	46623952	TDGF1	0.771029879	76
chr3.fa	46653925	46668033	LOC100132146	0	0
chr3.fa	46710485	46735194	ALS2CL	2.858170177	684

chr3.fa	46742823	46752413	TMIE	0	0
chr3.fa	46753606	46759373	PRSS50	0	0
chr3.fa	46783581	46786245	PRSS45	0	0
chr3.fa	46871894	46875585	PRSS42	0	0
chr3.fa	46899357	46904973	MYL3	1.819381436	76
chr3.fa	46919236	46945289	PTH1R	14.17676426	1368
chr3.fa	46963220	47018270	CCDC12	30.69308269	1209
chr3.fa	47021173	47051193	NBEAL2	28.33862832	11248
chr3.fa	47053032	47054957	NRADDP	0	0
chr3.fa	47057898	47205467	SETD2	29.75770581	11284
chr3.fa	47205860	47285606	FLJ39534	1.501139799	152
chr3.fa	47269516	47324329	KIF9	4.599714735	684
chr3.fa	47324338	47388306	KLHL18	7.523045498	1596
chr3.fa	47422491	47454931	PTPN23	15.03030123	3557
chr3.fa	47455184	47517445	SCAP	9.533238482	1824
chr3.fa	47537130	47555199	C3orf75	18.79360311	1140
chr3.fa	47603728	47620359	CSPG5	0	0
chr3.fa	47627378	47823405	SMARCC1	48.3720617	12522
chr3.fa	47844399	47891686	DHX30	18.37595266	3401
chr3.fa	47891045	47891119	MIR1226	0	0
chr3.fa	47892180	48130769	MAP4	56.81758541	22263
chr3.fa	48198668	48229801	CDC25A	2.280398148	380
chr3.fa	48264862	48266975	CAMP	0	0
chr3.fa	48282596	48312479	ZNF589	5.526640761	836
chr3.fa	48335589	48342848	NME6	7.229044489	380
chr3.fa	48348336	48369831	SPINK8	3.841361147	76
chr3.fa	48413709	48436190	FBXW12	0	0
chr3.fa	48445261	48471460	PLXNB1	7.569302843	2503
chr3.fa	48473580	48481529	CCDC51	4.489186367	304
chr3.fa	48481686	48485537	CCDC72	136.5567972	2843
chr3.fa	48488218	48507054	ATRIP	6.062825658	684
chr3.fa	48507229	48509044	TREX1	10.23777343	836
chr3.fa	48509197	48541661	SHISA5	18.72777535	1824
chr3.fa	48555117	48594227	PFKFB4	3.778646862	593
chr3.fa	48599151	48601201	UCN2	0	0
chr3.fa	48601506	48632593	COL7A1	0	0
chr3.fa	48616335	48616410	MIR711	0	0
chr3.fa	48636432	48647098	UQCRC1	50.17209511	3648
chr3.fa	48658275	48659189	TMEM89	0	0
chr3.fa	48663156	48672926	SLC26A6	0.634926537	76
chr3.fa	48673896	48700348	CELSR3	0.141218337	76
chr3.fa	48711278	48723334	NCKIPSD	3.994366211	532
chr3.fa	48725436	48754711	IP6K2	22.52821654	3040

chr3.fa	48788093	48885270	PRKAR2A	41.02225886	4392
chr3.fa	48894356	48936402	SLC25A20	11.76248668	988
chr3.fa	48955221	48956818	C3orf71	0	0
chr3.fa	48960181	49022971	ARIH2	16.30260061	2867
chr3.fa	49027341	49044581	P4HTM	2.962693985	304
chr3.fa	49044637	49053386	WDR6	51.42304495	8056
chr3.fa	49053406	49058467	DALRD3	7.336681772	545
chr3.fa	49057581	49057667	MIR425	0	0
chr3.fa	49057908	49060926	NDUFAF3	48.23795988	2720
chr3.fa	49058051	49058142	MIR191	0	0
chr3.fa	49061762	49066875	IMPDH2	72.96428986	5548
chr3.fa	49067142	49131504	QRICH1	26.51257515	3952
chr3.fa	49133365	49142171	QARS	74.12116827	8099
chr3.fa	49145479	49158371	USP19	20.23024949	4864
chr3.fa	49158547	49170599	LAMB2	1.745992379	456
chr3.fa	49190292	49191834	LAMB2P1	0	0
chr3.fa	49199968	49203785	CCDC71	9.327749123	760
chr3.fa	49209018	49213919	KLHDC8B	14.4749907	1292
chr3.fa	49215069	49229291	LOC646498	0	0
chr3.fa	49235861	49295537	CCDC36	0	0
chr3.fa	49306030	49314508	C3orf62	3.296280607	529
chr3.fa	49311574	49311619	MIR4271	0	0
chr3.fa	49314577	49377536	USP4	15.99925917	2964
chr3.fa	49394609	49395791	GPX1	108.5824454	5776
chr3.fa	49396579	49449526	RHOA	223.4792409	19304
chr3.fa	49449639	49453909	TCTA	36.99964898	3572
chr3.fa	49454211	49459704	AMT	20.7163964	1755
chr3.fa	49459766	49466757	NICN1	3.485980199	456
chr3.fa	49506146	49573048	DAG1	4.418466003	1292
chr3.fa	49591922	49708982	BSN	0.42365501	304
chr3.fa	49711435	49720934	APEH	40.85768946	5067
chr3.fa	49721380	49726196	MST1	1.450212241	152
chr3.fa	49726990	49758931	RNF123	4.801645836	912
chr3.fa	49754267	49757238	AMIGO3	0.568653995	76
chr3.fa	49758963	49761384	GMPPB	28.28547685	2049
chr3.fa	49761728	49823973	IP6K1	17.77927739	3572
chr3.fa	49828167	49837254	CDHR4	0	0
chr3.fa	49840687	49842463	C3orf54	0	0
chr3.fa	49842638	49851391	UBA7	7.142311967	1064
chr3.fa	49866028	49893992	TRAIP	0.83507851	76
chr3.fa	49895422	49907369	CAMKV	0	0
chr3.fa	49924436	49941306	MST1R	0	0
chr3.fa	49946302	49967445	MON1A	3.789544025	380

chr3.fa	49977477	50114685	RBM6	15.60718369	2624
chr3.fa	50126352	50156392	RBM5	32.01297376	4484
chr3.fa	50192848	50226508	SEMA3F	3.845586578	608
chr3.fa	50229043	50235129	GNAT1	0	0
chr3.fa	50242692	50258406	SLC38A3	0	0
chr3.fa	50273647	50296786	GNAI2	65.47549259	7013
chr3.fa	50305040	50314572	SEMA3B	1.558071916	205
chr3.fa	50316518	50325162	C3orf45	0	0
chr3.fa	50325546	50330026	IFRD2	40.09733435	3112
chr3.fa	50330262	50336899	HYAL3	2.707166633	228
chr3.fa	50333833	50334958	NAT6	1.501139799	76
chr3.fa	50337320	50349812	HYAL1	2.437851033	228
chr3.fa	50355221	50360281	HYAL2	1.400841421	152
chr3.fa	50362341	50365668	TUSC2	34.16527465	2584
chr3.fa	50367217	50378367	RASSF1	9.422042941	988
chr3.fa	50378537	50383156	ZMYND10	0	0
chr3.fa	50384919	50388486	NPRL2	12.03358141	836
chr3.fa	50388774	50391496	CYB561D2	3.127040994	152
chr3.fa	50392180	50396939	TMEM115	5.647399119	532
chr3.fa	50400231	50540892	CACNA2D2	0.946496442	228
chr3.fa	50595456	50608361	C3orf18	4.952871772	608
chr3.fa	50606909	50622421	HEMK1	2.639114962	684
chr3.fa	50643885	50649262	CISH	3.726162567	380
chr3.fa	50654601	50686719	MAPKAPK3	20.28206661	2280
chr3.fa	50712672	51421629	DOCK3	4.247224871	1672
chr3.fa	51422692	51426828	MANF	8.999944669	380
chr3.fa	51428699	51435336	RBM15B	15.39368826	3724
chr3.fa	51436166	51534018	VPRBP	15.71882402	3240
chr3.fa	51575596	51697612	RAD54L2	12.93226377	2736
chr3.fa	51705222	51738339	TEX264	10.72747859	684
chr3.fa	51741081	51752625	GRM2	0	0
chr3.fa	51812577	51813203	IQCF6	0	0
chr3.fa	51862569	51864876	IQCF3	0	0
chr3.fa	51895645	51897440	IQCF2	0	0
chr3.fa	51907737	51909600	IQCF5	0	0
chr3.fa	51928892	51937386	IQCF1	0	0
chr3.fa	51967446	51975922	RRP9	5.476825159	380
chr3.fa	51976361	51982883	PARP3	4.297040473	456
chr3.fa	51989330	51991469	GPR62	0	0
chr3.fa	51991521	52001482	PCBP4	14.57039647	1444
chr3.fa	52002526	52008646	ABHD14B	38.84660691	3918
chr3.fa	52014409	52015216	ABHD14A	30.9348218	836
chr3.fa	52017300	52017605	ACY1	21.94844299	302

chr3.fa	52027644	52029958	RPL29	502.9879131	15719
chr3.fa	52082937	52090461	DUSP7	4.182286675	608
chr3.fa	52096110	52099128	C3orf74	0	0
chr3.fa	52109249	52188706	POC1A	0	0
chr3.fa	52232116	52248343	ALAS1	61.08215678	6688
chr3.fa	52255098	52260179	TLR9	0	0
chr3.fa	52262626	52273183	TWF2	18.72199318	1368
chr3.fa	52280225	52284615	PPM1M	4.954206118	456
chr3.fa	52288438	52312659	WDR82	37.01677309	7144
chr3.fa	52302294	52302377	MIRLET7G	0	0
chr3.fa	52321836	52329272	GLYCTK	5.228414321	836
chr3.fa	52350335	52434513	DNAH1	0.644711745	380
chr3.fa	52435025	52444009	BAP1	21.95378037	3538
chr3.fa	52444527	52457657	PHF7	0.74256382	76
chr3.fa	52467268	52479043	SEMA3G	0.359606378	76
chr3.fa	52485107	52488057	TNNC1	4.856798824	152
chr3.fa	52489524	52527088	NISCH	17.42122774	4104
chr3.fa	52529356	52558384	STAB1	0	0
chr3.fa	52558512	52569093	NT5DC2	3.78442903	380
chr3.fa	52570621	52574586	C3orf78	30.1144211	608
chr3.fa	52579368	52719866	PBRM1	17.71078093	6156
chr3.fa	52719936	52728503	GNL3	80.06924013	7676
chr3.fa	52723256	52723331	SNORD19	0	0
chr3.fa	52724754	52724846	SNORD19B	0	0
chr3.fa	52726752	52726828	SNORD69	0	0
chr3.fa	52728511	52739856	GLT8D1	6.827406196	684
chr3.fa	52740049	52742197	SPCS1	32.21179139	1292
chr3.fa	52744796	52804965	NEK4	9.525899576	1596
chr3.fa	52811608	52826077	ITIH1	0	0
chr3.fa	52828784	52843025	ITIH3	0	0
chr3.fa	52847006	52864717	ITIH4	0.514835353	76
chr3.fa	52868956	52869235	MUSTN1	0	0
chr3.fa	52870772	52874515	TMEM110	4.989788691	836
chr3.fa	52937583	53080089	SFMBT1	3.011620023	684
chr3.fa	53122501	53164470	RFT1	0.331585102	76
chr3.fa	53195223	53226733	PRKCD	43.8664184	5592
chr3.fa	53258724	53290130	TKT	32.53514802	4484
chr3.fa	53317445	53381654	DCP1A	10.40189805	2812
chr3.fa	53529031	53846492	CACNA1D	1.073481749	380
chr3.fa	53850324	53880420	CHDH	10.48952013	1748
chr3.fa	53880577	53899827	IL17RB	6.628143787	608
chr3.fa	53901094	53916229	ACTR8	9.317296742	1520
chr3.fa	53919226	53925989	SELK	37.19201726	1368

chr3.fa	54156693	55108584	CACNA2D3	0	0
chr3.fa	54666151	54673884	ESRG	0	0
chr3.fa	54952381	54962072	LRTM1	0	0
chr3.fa	55499743	55521331	WNT5A	1.737096736	456
chr3.fa	55542336	56502391	ERC2	0	0
chr3.fa	55691243	55693497	C3orf51	0	0
chr3.fa	55886520	55886622	MIR3938	0	0
chr3.fa	56591184	56653929	CCDC66	8.311421881	1064
chr3.fa	56654160	56717135	C3orf63	9.626642736	3466
chr3.fa	56761446	57113336	ARHGEF3	11.33616298	2356
chr3.fa	57094469	57109460	SPATA12	0	0
chr3.fa	57124010	57199403	IL17RD	0.581552678	228
chr3.fa	57231944	57234280	HESX1	0	0
chr3.fa	57261765	57307498	APPL1	8.97948469	2379
chr3.fa	57302379	57326710	ASB14	0	0
chr3.fa	57327727	57530071	DNAH12	0	0
chr3.fa	57541981	57547768	PDE12	7.195908218	1292
chr3.fa	57557090	57583215	ARF4	93.2508043	7296
chr3.fa	57611181	57678816	FAM116A	6.526511063	1367
chr3.fa	57743174	57914894	SLMAP	19.88443136	3952
chr3.fa	57994127	58157982	FLNB	38.54148634	16568
chr3.fa	58178354	58200398	DNASE1L3	0	0
chr3.fa	58223259	58280461	ABHD6	0.706314074	76
chr3.fa	58291972	58305920	RPP14	6.277877834	988
chr3.fa	58318617	58410878	PXK	8.338331202	760
chr3.fa	58413357	58419579	PDHB	37.69929132	2497
chr3.fa	58477823	58488087	KCTD6	6.204043995	532
chr3.fa	58490863	58522929	ACOX2	8.05189149	836
chr3.fa	58549845	58563491	FAM107A	3.903408259	608
chr3.fa	58619670	58652561	FAM3D	0	0
chr3.fa	58727737	59035715	C3orf67	0	0
chr3.fa	59735036	61237133	FHIT	7.832169101	380
chr3.fa	61547243	62280573	PTPRG	0.254193006	76
chr3.fa	62109159	62110392	ID2B	1.440871816	76
chr3.fa	62305396	62319320	C3orf14	22.03606507	760
chr3.fa	62355347	62359190	FEZF2	1.620563809	152
chr3.fa	62384021	62861064	CADPS	0.913360171	228
chr3.fa	63088364	63110737	LOC285401	0	0
chr3.fa	63263914	63602597	SYNPR	0	0
chr3.fa	63638344	63650891	SNTN	0	0
chr3.fa	63805041	63834312	C3orf49	0	0
chr3.fa	63819546	63849597	THOC7	21.42271047	971
chr3.fa	63850233	63989136	ATXN7	8.311644272	2812

chr3.fa	63996231	64009120	PSMD6	41.34984092	2432
chr3.fa	64064037	64073039	LOC100287879	0	0
chr3.fa	64079526	64211131	PRICKLE2	3.655664594	1368
chr3.fa	64501331	64673365	ADAMTS9	2.542597232	836
chr3.fa	64705683	64941858	MIR548A2	0	0
chr3.fa	65339906	66024509	MAGI1	25.86919775	10661
chr3.fa	66119285	66429220	SLC25A26	6.433773982	760
chr3.fa	66429352	66550845	LRIG1	3.284716271	684
chr3.fa	67048727	67061632	KBTBD8	0	0
chr3.fa	67275888	67275951	MIR4272	0	0
chr3.fa	67410884	67705038	SUCLG2	39.3505451	4712
chr3.fa	68053454	68594771	FAM19A1	0	0
chr3.fa	68780917	68981711	FAM19A4	0	0
chr3.fa	69024368	69062774	C3orf64	2.859059741	532
chr3.fa	69068978	69101484	TMF1	16.70757476	5168
chr3.fa	69098109	69098186	MIR3136	0	0
chr3.fa	69103881	69129524	UBA3	23.67686647	2256
chr3.fa	69134090	69155239	ARL6IP5	7.148316526	684
chr3.fa	69156039	69171746	LMOD3	0	0
chr3.fa	69219146	69435430	FRMD4B	7.253729899	1716
chr3.fa	69788586	70017488	MITF	3.874942201	1062
chr3.fa	71004736	71633140	FOXP1	41.39632066	11928
chr3.fa	71591121	71591240	MIR1284	0	0
chr3.fa	71728440	71778269	EIF4E3	7.818603245	2204
chr3.fa	71803201	71804328	GPR27	2.03398883	76
chr3.fa	71820806	71834357	PROK2	0	0
chr3.fa	72423744	72495774	RYBP	20.13462133	4028
chr3.fa	72798428	72897598	SHQ1	4.747604804	608
chr3.fa	72937385	73024522	GXYLT2	0	0
chr3.fa	73046119	73115011	PPP4R2	16.38266139	1077
chr3.fa	73110810	73112471	EBLN2	0	0
chr3.fa	73431652	73674072	PDZRN3	5.68676234	1064
chr3.fa	74311722	74570343	CNTN3	3.711707147	834
chr3.fa	75470703	75484266	FAM86DP	1.970162589	222
chr3.fa	75679914	75680009	MIR1324	0	0
chr3.fa	75713487	75716368	FRG2C	0	0
chr3.fa	75786029	75834255	ZNF717	9.062658954	1216
chr3.fa	77089294	77699114	ROBO2	0	0
chr3.fa	78646388	79817059	ROBO1	1.28341893	456
chr3.fa	81538850	81810950	GBE1	9.817009502	1368
chr3.fa	84687556	84918726	LOC440970	0	0
chr3.fa	85008133	86123579	CADM2	0.350488344	152
chr3.fa	86987123	87040257	VGLL3	43.57130543	20368

chr3.fa	87276413	87304698	CHMP2B	27.01184313	3192
chr3.fa	87308783	87325737	POU1F1	0	0
chr3.fa	88031726	88042919	HTR1F	0.531737075	76
chr3.fa	88101100	88198892	CGGBP1	20.20422974	4180
chr3.fa	88188262	88193814	ZNF654	2.448303414	537
chr3.fa	88199017	88207115	C3orf38	4.046628115	456
chr3.fa	89156674	89531284	EPHA3	0	0
chr3.fa	93591881	93692934	PROS1	0.944272531	152
chr3.fa	93698983	93774522	ARL13B	0.441223905	76
chr3.fa	93733215	93747454	STX19	1.461998968	76
chr3.fa	93776766	93782067	DHFRL1	1.256732	228
chr3.fa	93783281	93845630	NSUN3	6.465798298	380
chr3.fa	94657107	94710169	LOC255025	0	0
chr3.fa	96533425	97467786	EPHA6	0	0
chr3.fa	97483595	97517373	ARL6	2.326433101	152
chr3.fa	97660661	97691295	MINA	8.542708606	2052
chr3.fa	97705527	97753831	GABRR3	0	0
chr3.fa	97806017	97806946	OR5AC2	0	0
chr3.fa	97851542	97852483	OR5H1	0	0
chr3.fa	97868230	97869162	OR5H14	0	0
chr3.fa	97887544	97888485	OR5H15	0	0
chr3.fa	97983129	97984106	OR5H6	0	0
chr3.fa	98001732	98002676	OR5H2	0	0
chr3.fa	98072698	98073663	OR5K4	0	0
chr3.fa	98109510	98110475	OR5K3	0	0
chr3.fa	98188421	98189372	OR5K1	0	0
chr3.fa	98216525	98217475	OR5K2	0	0
chr3.fa	98234317	98241910	CLDND1	6.667284617	760
chr3.fa	98250878	98251960	GPR15	0	0
chr3.fa	98298290	98312455	CPOX	1.87164334	228
chr3.fa	98451572	98513236	ST3GAL6	0.807057234	76
chr3.fa	98514814	98620533	DCBLD2	1.387053174	380
chr3.fa	99273153	99717059	MIR548G	0	0
chr3.fa	99357454	99515158	COL8A1	0	0
chr3.fa	99536678	99897476	C3orf26	2.602198042	152
chr3.fa	99551988	99833349	FILIP1L	0	0
chr3.fa	99683158	99683242	MIR3921	0	0
chr3.fa	99904668	99913030	TMEM30C	0	0
chr3.fa	99979686	100044094	TBC1D23	10.3732096	1800
chr3.fa	100053562	100074478	NIT2	34.29470626	1960
chr3.fa	100082307	100120036	TOMM70A	37.28030652	6992
chr3.fa	100120226	100175170	LNP1	2.500342927	228
chr3.fa	100211463	100296285	TMEM45A	3.242017183	228

chr3.fa	100328433	100414323	GPR128	0	0
chr3.fa	100428134	100467810	TFG	40.79453039	3876
chr3.fa	100468179	100712334	ABI3BP	0	0
chr3.fa	100945288	101039419	IMPG2	0.379399185	76
chr3.fa	101043118	101232085	SENP7	4.796753233	1064
chr3.fa	101237716	101242724	FAM172B	0	0
chr3.fa	101280712	101285089	RG9MTD1	5.242424959	380
chr3.fa	101293042	101313281	PCNP	42.09440626	4238
chr3.fa	101368283	101391057	ZBTB11	7.751441139	1596
chr3.fa	101395274	101398057	LOC100009676	1.506477185	152
chr3.fa	101399934	101405563	RPL24	1018.685699	25743
chr3.fa	101431278	101432260	LOC285359	0	0
chr3.fa	101443494	101486181	CEP97	3.831798331	760
chr3.fa	101498029	101547075	FAM55C	1.71730393	684
chr3.fa	101548457	101579869	NFKBIZ	12.24752163	2204
chr3.fa	101659703	101716770	LOC152225	0	0
chr3.fa	102153859	102198685	ZPLD1	0	0
chr3.fa	103903476	103946105	MIR548A3	0	0
chr3.fa	105085713	105295744	ALCAM	14.41961532	3074
chr3.fa	105377109	105587887	CBLB	8.927000394	1596
chr3.fa	106828637	106959485	LOC100302640	0	0
chr3.fa	106959539	107045811	LOC344595	0.842195025	152
chr3.fa	107096188	107097481	CCDC54	0	0
chr3.fa	107241783	107530049	BBX	19.94469934	7980
chr3.fa	107560509	107596915	LOC151658	0	0
chr3.fa	107602052	107647753	LOC285205	0	0
chr3.fa	107761941	107809935	CD47	3.12414991	751
chr3.fa	107879659	107941417	IFT57	57.8924015	8338
chr3.fa	108021332	108097126	HHLA2	0	0
chr3.fa	108099216	108248169	MYH15	0	0
chr3.fa	108268718	108308336	KIAA1524	0.411201109	76
chr3.fa	108308492	108413693	DZIP3	2.803017189	679
chr3.fa	108474486	108476130	RETNLB	0	0
chr3.fa	108541631	108573714	TRAT1	0	0
chr3.fa	108626642	108672677	GUCA1C	0	0
chr3.fa	108677087	108836993	MORC1	0	0
chr3.fa	108855561	108868951	FLJ22763	0	0
chr3.fa	108897012	108904108	C3orf66	0	0
chr3.fa	109012635	109035364	DPPA2	0	0
chr3.fa	109044988	109056419	DPPA4	0	0
chr3.fa	109128837	109214014	FLJ25363	0	0
chr3.fa	110790865	110853062	PVRL3	9.219222275	684
chr3.fa	111260926	111371206	CD96	0.754350548	152

chr3.fa	111311747	111314166	ZBED2	0.772809008	76
chr3.fa	111393523	111565294	PLCXD2	0	0
chr3.fa	111451327	111695364	PHLDB2	8.374358557	2419
chr3.fa	111697828	111712210	ABHD10	16.62039746	1976
chr3.fa	111717586	111732735	TAGLN3	0	0
chr3.fa	111758465	111800116	TMPRSS7	1.289201098	152
chr3.fa	111805182	111837044	C3orf52	5.193721313	532
chr3.fa	111831648	111831745	MIR567	0	0
chr3.fa	111839688	111852152	GCET2	0	0
chr3.fa	111859752	112013074	SLC9A10	0	0
chr3.fa	112051916	112081658	CD200	0.738115999	76
chr3.fa	112182813	112218408	BTLA	0	0
chr3.fa	112251359	112280485	ATG3	26.71561821	1492
chr3.fa	112280895	112303003	SLC35A5	4.599047561	608
chr3.fa	112323409	112359977	CCDC80	0	0
chr3.fa	112534556	112564797	CD200R1L	0	0
chr3.fa	112641532	112693937	CD200R1	0	0
chr3.fa	112709800	112720221	GTPBP8	2.491002502	152
chr3.fa	112721292	112738555	C3orf17	7.65092037	1672
chr3.fa	112931375	113005776	BOC	0	0
chr3.fa	113006306	113160361	WDR52	1.519598259	684
chr3.fa	113161565	113234034	SPICE1	8.996831194	2204
chr3.fa	113251218	113348422	SIDT1	2.365351541	532
chr3.fa	113367233	113415493	KIAA2018	6.73978411	4154
chr3.fa	113435307	113465120	NAA50	19.56352103	5396
chr3.fa	113465866	113530905	ATP6V1A	32.38303252	6688
chr3.fa	113557681	113666021	GRAMD1C	6.589447739	1216
chr3.fa	113666748	113681827	ZDHHC23	0.895124102	152
chr3.fa	113682984	113775460	KIAA1407	3.400804415	608
chr3.fa	113775611	113807268	QTRTD1	6.769139733	1216
chr3.fa	113847557	113897899	DRD3	0	0
chr3.fa	113953480	113956425	ZNF80	0	0
chr3.fa	114012833	114029135	TIGIT	0	0
chr3.fa	114035322	114035416	MIR568	0	0
chr3.fa	114056947	114866127	ZBTB20	14.50657023	3058
chr3.fa	115342151	115440334	GAP43	0	0
chr3.fa	115521210	116164385	LSAMP	1.604996434	684
chr3.fa	116428635	116435887	NCRNA00295	0	0
chr3.fa	118619479	118864898	IGSF11	0.875553687	152
chr3.fa	118864997	118870302	C3orf30	0	0
chr3.fa	118892425	118924000	UPK1B	18.06794101	1672
chr3.fa	118930589	118959752	B4GALT4	16.58437011	1748
chr3.fa	119013220	119138323	ARHGAP31	0.418317624	152

chr3.fa	119149701	119182471	TMEM39A	4.001037944	532
chr3.fa	119187785	119213554	POGLUT1	0.459904756	76
chr3.fa	119217368	119243125	C3orf1	61.5380585	4408
chr3.fa	119243140	119278481	CD80	0	0
chr3.fa	119298523	119308792	ADPRH	4.423358607	684
chr3.fa	119316722	119348658	PLA1A	0	0
chr3.fa	119360908	119379404	POPDC2	0	0
chr3.fa	119388372	119396243	COX17	91.90066805	1748
chr3.fa	119421869	119485949	C3orf15	2.287514662	456
chr3.fa	119499331	119537332	NR1I2	0	0
chr3.fa	119540802	119813264	GSK3B	17.33382805	5548
chr3.fa	119884328	119963142	GPR156	0	0
chr3.fa	120043576	120068186	LRRC58	5.499731441	1900
chr3.fa	120113061	120169918	FSTL1	16.10533972	2736
chr3.fa	120315128	120321258	NDUFB4	116.3725826	3192
chr3.fa	120347015	120401418	HGD	34.60894485	3128
chr3.fa	120405528	120461384	RABL3	10.23465995	1787
chr3.fa	120461558	120501916	GTF2E1	7.801701523	1064
chr3.fa	120627050	121143608	STXBP5L	0.180581558	76
chr3.fa	121150273	121264853	POLQ	0.384958962	152
chr3.fa	121286778	121309469	ARGFX	0	0
chr3.fa	121312170	121349139	FBXO40	0	0
chr3.fa	121350246	121379791	HCLS1	0.831297862	76
chr3.fa	121382048	121468602	GOLGB1	28.55990744	14364
chr3.fa	121488610	121553926	IQCB1	8.470431505	988
chr3.fa	121554034	121605373	EAF2	34.79775488	1596
chr3.fa	121613171	121663034	SLC15A2	7.956708107	1976
chr3.fa	121706170	121741127	ILDR1	1.755777587	228
chr3.fa	121774221	121839983	CD86	0	0
chr3.fa	121902530	122005344	CASR	0	0
chr3.fa	122044011	122060815	CSTA	12.26242183	456
chr3.fa	122078436	122102074	CCDC58	16.47806717	532
chr3.fa	122103023	122128961	FAM162A	177.4883205	6688
chr3.fa	122130700	122134882	WDR5B	2.020200583	380
chr3.fa	122140748	122233786	KPNA1	10.8469026	3420
chr3.fa	122246760	122283523	PARP9	6.508942167	2280
chr3.fa	122283195	122294049	DTX3L	20.10370897	5046
chr3.fa	122296449	122355536	PARP15	0	0
chr3.fa	122399672	122449687	PARP14	14.0335444	4864
chr3.fa	122458846	122512650	HSPBAP1	1.745992379	152
chr3.fa	122513901	122599986	DIRC2	0.506384492	76
chr3.fa	122605360	122611263	LOC100129550	0.286217322	76
chr3.fa	122628043	122746576	SEMA5B	0	0

chr3.fa	122785856	122880953	PDIA5	6.353935584	532
chr3.fa	122920774	122992982	SEC22A	0.989640312	152
chr3.fa	123001143	123167392	ADCY5	0.44433738	125
chr3.fa	123213363	123303924	PTPLB	2.847717796	152
chr3.fa	123331143	123603149	MYLK	2.372690446	836
chr3.fa	123632274	123680255	CCDC14	1.212476175	228
chr3.fa	123687879	123710199	ROPN1	0	0
chr3.fa	123813558	124440036	KALRN	8.265386928	5472
chr3.fa	124449213	124468119	UMPS	6.181137714	1900
chr3.fa	124481795	124606144	ITGB5	5.012472581	760
chr3.fa	124624289	124653595	MUC13	1.175336865	152
chr3.fa	124684554	124774802	HEG1	0	0
chr3.fa	124801480	124931609	SLC12A8	0.962953382	152
chr3.fa	124944513	125094198	ZNF148	9.918197444	4256
chr3.fa	125165494	125239058	SNX4	23.88680365	2711
chr3.fa	125247702	125314381	OSBPL11	7.288422907	1520
chr3.fa	125509247	125509395	MIR548I1	0	0
chr3.fa	125635444	125648867	LOC100125556	0	0
chr3.fa	125649400	125655887	ALG1L	2.691376866	76
chr3.fa	125688028	125702296	ROPN1B	1.690172218	76
chr3.fa	125725200	125820391	SLC41A3	5.783280069	608
chr3.fa	125822408	125899485	ALDH1L1	14.60308796	2052
chr3.fa	126061478	126076236	KLF15	9.371337775	1064
chr3.fa	126113782	126155398	CCDC37	0	0
chr3.fa	126156444	126194762	ZXDC	4.797865188	2964
chr3.fa	126200008	126236616	UROC1	0	0
chr3.fa	126243176	126262134	CHST13	0	0
chr3.fa	126268519	126277758	C3orf22	0	0
chr3.fa	126290622	126291485	TXNRD3IT1	0	0
chr3.fa	126325895	126373967	TXNRD3	7.98828764	912
chr3.fa	126380924	126390782	C3orf46	0	0
chr3.fa	126423118	126679244	CHCHD6	0	0
chr3.fa	126707437	126756235	PLXNA1	1.864304435	760
chr3.fa	127291908	127309496	TPRA1	2.757204626	228
chr3.fa	127317253	127341278	MCM2	2.460979706	380
chr3.fa	127348039	127391652	PODXL2	3.114142311	304
chr3.fa	127391781	127399769	ABTB1	3.033191958	271
chr3.fa	127407909	127542051	MGLL	6.063048049	1292
chr3.fa	127641902	127706514	KBTBD12	0	0
chr3.fa	127771212	127790526	SEC61A1	12.08584331	1976
chr3.fa	127799800	127842671	RUVBL1	7.647806895	608
chr3.fa	127872313	128127489	EEFSEC	4.576363671	456
chr3.fa	128081008	128081101	MIR1280	0	0

chr3.fa	128181282	128186091	DNAJB8	0	0
chr3.fa	128198265	128212030	GATA2	88.01104803	14896
chr3.fa	128226678	128229429	LOC90246	1.842510109	228
chr3.fa	128290843	128294929	C3orf27	0	0
chr3.fa	128338813	128369719	RPN1	15.16173436	1596
chr3.fa	128444979	128533641	RAB7A	145.3014369	14583
chr3.fa	128580351	128590384	LOC653712	0	0
chr3.fa	128598333	128631957	ACAD9	19.04601698	2356
chr3.fa	128689782	128712986	KIAA1257	0	0
chr3.fa	128720472	128759585	CCDC48	0	0
chr3.fa	128779645	128781253	GP9	0	0
chr3.fa	128806418	128840619	RAB43	4.940862653	988
chr3.fa	128846259	128880073	ISY1	8.637669598	1493
chr3.fa	128886658	128902810	CNBP	92.40015841	14060
chr3.fa	128968453	128996616	COPG	114.2122757	15787
chr3.fa	128997684	129024135	C3orf37	19.10317149	1596
chr3.fa	129033614	129035113	H1FX	77.74792202	5244
chr3.fa	129035121	129043412	C3orf47	0.77325379	76
chr3.fa	129101677	129118282	RPL32P3	0.814618531	76
chr3.fa	129116053	129116191	SNORA7B	0	0
chr3.fa	129120164	129147494	C3orf25	0	0
chr3.fa	129149793	129158852	MBD4	6.84275118	760
chr3.fa	129158968	129239191	IFT122	5.672974093	1064
chr3.fa	129247482	129254187	RHO	0	0
chr3.fa	129262057	129270204	H1FOO	0	0
chr3.fa	129274056	129325582	PLXND1	0.242183888	76
chr3.fa	129366635	129612419	TMCC1	18.42599065	5214
chr3.fa	129693114	129696776	TRH	0	0
chr3.fa	129800674	129816093	ALG1L2	0	0
chr3.fa	129816625	129830276	LOC729375	0	0
chr3.fa	129931663	129992649	COL6A4P2	0	0
chr3.fa	130064359	130203688	COL6A5	0	0
chr3.fa	130279178	130395888	COL6A6	0	0
chr3.fa	130397778	130465696	PIK3R4	9.056209613	2052
chr3.fa	130569369	130735555	ATP2C1	13.84584633	3771
chr3.fa	130732721	130745646	ASTE1	4.099112411	456
chr3.fa	130745694	131069309	NEK11	3.050760853	456
chr3.fa	131080689	131083966	NUDT16P1	4.289701567	456
chr3.fa	131100515	131107674	NUDT16	24.19170182	7372
chr3.fa	131181045	131221860	MRPL3	38.32598939	3040
chr3.fa	131197941	131198077	SNORA58	0	0
chr3.fa	131253577	131753844	CPNE4	49.22715541	5691
chr3.fa	132036211	132087146	ACPP	244.8049888	42028

chr3.fa	132136553	132257876	DNAJC13	25.19757668	8551
chr3.fa	132276982	132378975	ACAD11	7.604885416	1216
chr3.fa	132316094	132321382	CCRL1	0	0
chr3.fa	132373290	132396944	UBA5	18.0930712	2649
chr3.fa	132399454	132441276	NPHP3	3.803777055	912
chr3.fa	132446337	132593050	NCRNA00119	0	0
chr3.fa	132757171	133116619	TMEM108	0	0
chr3.fa	133118790	133194056	BFSP2	0	0
chr3.fa	133292434	133309118	CDV3	25.74465874	5016
chr3.fa	133319449	133380737	TOPBP1	6.639263341	1596
chr3.fa	133464977	133497850	TF	0	0
chr3.fa	133502877	133540336	SRPRB	1.195796844	152
chr3.fa	133543080	133614691	RAB6B	7.883763832	1971
chr3.fa	133646990	133648656	C3orf36	0	0
chr3.fa	133651540	133748920	SLCO2A1	0.400303946	76
chr3.fa	133875978	133969586	RYK	9.803221255	1292
chr3.fa	134074190	134093406	AMOTL2	15.59940001	3496
chr3.fa	134196546	134204863	ANAPC13	42.26942804	3081
chr3.fa	134204866	134293855	CEP63	4.311940675	1251
chr3.fa	134318765	134369864	KY	0.29622492	76
chr3.fa	134514099	134979307	EPHB1	0	0
chr3.fa	135684515	135866752	PPP2R3A	3.946329738	1292
chr3.fa	135867760	135914688	MSL2	9.212772934	1976
chr3.fa	135969167	136049013	PCCB	17.01136098	1435
chr3.fa	136055999	136471245	STAG1	13.74132252	3192
chr3.fa	136537861	136574734	TMEM22	0	0
chr3.fa	136581050	136667968	NCK1	6.573435581	608
chr3.fa	136676707	136729926	IL20RB	0	0
chr3.fa	137483579	137484396	SOX14	0	0
chr3.fa	137717658	137752494	CLDN18	0.465464533	76
chr3.fa	137780827	137834451	DZIP1L	2.161863701	380
chr3.fa	137842560	137851229	A4GNT	0	0
chr3.fa	137879830	137893791	DBR1	6.911470025	836
chr3.fa	137906148	138016219	ARMC8	8.465983683	2128
chr3.fa	137980279	138048728	TXNDC6	0	0
chr3.fa	138066625	138124377	MRAS	3.709705627	760
chr3.fa	138153415	138197256	ESYT3	0	0
chr3.fa	138213187	138313129	CEP70	21.58750226	2584
chr3.fa	138327542	138352213	FAIM	8.084582978	602
chr3.fa	138374231	138478185	PIK3CB	28.93219011	4180
chr3.fa	138663066	138665982	FOXL2	0	0
chr3.fa	138666076	138672830	C3orf72	0	0
chr3.fa	138722804	138725110	PRR23A	0	0

chr3.fa	138737873	138739768	PRR23B	0	0
chr3.fa	138760944	138763734	PRR23C	0	0
chr3.fa	138823027	138844005	BPESC1	0	0
chr3.fa	138951834	138952364	PISRT1	0	0
chr3.fa	139062861	139075887	MRPS22	27.11503259	1368
chr3.fa	139076433	139108522	COPB2	70.7317058	10184
chr3.fa	139171726	139195352	RBP2	0	0
chr3.fa	139236276	139258671	RBP1	0.864878915	76
chr3.fa	139279033	139396840	NMNAT3	2.721622053	228
chr3.fa	139654027	140286919	CLSTN2	3.808224876	836
chr3.fa	140396866	140419992	TRIM42	0	0
chr3.fa	140660662	140698785	SLC25A36	15.23000842	3192
chr3.fa	140770743	140867453	SPSB4	0	0
chr3.fa	140950682	141013486	ACPL2	5.844660008	836
chr3.fa	141043055	141168632	ZBTB38	18.74823533	7068
chr3.fa	141205926	141331197	RASA2	5.216627594	608
chr3.fa	141457051	141465243	RNF7	43.78279935	3148
chr3.fa	141497043	141535892	GRK7	0	0
chr3.fa	141595470	141645382	ATP1B3	7.020886437	585
chr3.fa	141663270	141868386	TFDP2	13.33078859	6077
chr3.fa	141876369	141944449	GK5	5.101651405	2505
chr3.fa	142025449	142166853	XRN1	9.378676681	4256
chr3.fa	142168077	142297668	ATR	4.0968885	1520
chr3.fa	142315229	142432505	PLS1	27.43260705	5092
chr3.fa	142443266	142526729	TRPC1	0.41542654	76
chr3.fa	142536702	142608045	PCOLCE2	0	0
chr3.fa	142680074	142682178	PAQR9	0	0
chr3.fa	142719687	142720309	LOC100289361	0	0
chr3.fa	142720372	142779567	SR140	12.96695678	4352
chr3.fa	142838668	142841812	CHST2	0.555532921	76
chr3.fa	142984064	143567373	SLC9A9	0	0
chr3.fa	143690640	143711210	C3orf58	1.839841416	380
chr3.fa	145787228	145879282	PLOD2	2.914879903	532
chr3.fa	145910124	145968966	PLSCR4	1.477788735	228
chr3.fa	146151082	146213722	PLSCR2	0	0
chr3.fa	146232967	146262628	PLSCR1	31.084491	3089
chr3.fa	146303625	146324003	PLSCR5	0	0
chr3.fa	147103835	147124407	ZIC4	0	0
chr3.fa	147127181	147134506	ZIC1	0	0
chr3.fa	148415658	148460790	AGTR1	0.657388036	76
chr3.fa	148545588	148577972	CPB1	0	0
chr3.fa	148583043	148614872	CPA3	0	0
chr3.fa	148709195	148745456	GYG1	30.92792768	2812

chr3.fa	148747904	148804341	HLTF	9.237458344	1875
chr3.fa	148847371	148890289	HPS3	16.72959148	2584
chr3.fa	148891306	148939832	CP	0	0
chr3.fa	149036285	149051548	TM4SF18	0.877555207	152
chr3.fa	149086805	149095568	TM4SF1	6.996645809	532
chr3.fa	149192434	149221068	TM4SF4	0	0
chr3.fa	149235022	149421060	WWTR1	11.33705254	2736
chr3.fa	149456257	149470286	COMMD2	5.47282212	912
chr3.fa	149478890	149510610	C3orf16	0	0
chr3.fa	149530475	149679925	RNF13	11.40465943	1520
chr3.fa	149682691	149688741	PFN2	57.78365226	5472
chr3.fa	149689066	149691029	LOC646903	0	0
chr3.fa	150126788	150177615	TSC22D2	17.38475561	3037
chr3.fa	150259780	150264428	SERP1	128.4146149	18316
chr3.fa	150264574	150303803	EIF2A	39.65077306	6916
chr3.fa	150321066	150348234	SELT	10.65431192	1672
chr3.fa	150377675	150421742	FAM194A	0	0
chr3.fa	150458910	150481263	SIAH2	16.12757882	1900
chr3.fa	150643950	150690464	CLRN1	0	0
chr3.fa	150690787	150797617	CLRN1OS	0	0
chr3.fa	150804676	151153777	MED12L	1.574528856	684
chr3.fa	150915619	150920988	GPR171	0	0
chr3.fa	150929905	150996230	P2RY14	0	0
chr3.fa	151011876	151034636	GPR87	0	0
chr3.fa	151044096	151047337	P2RY13	0	0
chr3.fa	151055376	151102544	P2RY12	0	0
chr3.fa	151154466	151176497	IGSF10	0	0
chr3.fa	151347320	151542394	MIR548H2	0	0
chr3.fa	151451704	151475556	AADACL2	0	0
chr3.fa	151488244	151502682	LOC201651	0	0
chr3.fa	151531861	151546276	AADAC	0	0
chr3.fa	151591431	151599876	SUCNR1	2.048666641	152
chr3.fa	151980405	151987415	LOC401093	0.300672742	76
chr3.fa	151986862	152183569	MBNL1	16.93241215	4180
chr3.fa	152057487	152058779	TMEM14E	0	0
chr3.fa	152552736	152555843	P2RY1	2.719175751	380
chr3.fa	152880029	152886263	RAP2B	4.608387987	1292
chr3.fa	153202284	153220486	C3orf79	0	0
chr3.fa	153839149	153975616	ARHGEF26	8.48177345	1976
chr3.fa	153993457	154042286	DHX36	21.77186447	3494
chr3.fa	154055461	154147504	GPR149	0	0
chr3.fa	154797436	154901518	MME	14.12806061	3832
chr3.fa	155197671	155421997	PLCH1	4.913286159	1444

chr3.fa	155480401	155524055	C3orf33	0.875331296	76
chr3.fa	155544301	155572248	SLC33A1	6.213829203	684
chr3.fa	155588325	155655520	GMPS	26.83904526	2964
chr3.fa	155838337	156256927	KCNAB1	0	0
chr3.fa	156257929	156272973	SSR3	23.19672413	3876
chr3.fa	156390960	156393502	LOC100287227	0.996534435	76
chr3.fa	156394453	156424557	TIPARP	13.73109253	2274
chr3.fa	156527060	156529810	PA2G4P4	0	0
chr3.fa	156544096	156763918	LEKR1	0	0
chr3.fa	156799456	156840791	LOC339894	0	0
chr3.fa	156807670	156818924	LOC100498859	0	0
chr3.fa	156865586	156878482	CCNL1	14.1707597	1672
chr3.fa	156977532	157221415	VEPH1	3.837358108	836
chr3.fa	157154580	157161417	PTX3	0	0
chr3.fa	157261159	157319021	C3orf55	0	0
chr3.fa	157813800	157823952	SHOX2	0	0
chr3.fa	157827892	158262576	RSRC1	12.17146388	891
chr3.fa	158288953	158324248	MLF1	1.303656519	152
chr3.fa	158362317	158410360	GFM1	10.19262804	1589
chr3.fa	158384203	158390482	LXN	6.057933055	304
chr3.fa	158414897	158450275	RARRES1	5.059174708	379
chr3.fa	158519715	158547508	MFSD1	7.861747115	835
chr3.fa	158983004	158984096	IQCJ	0	0
chr3.fa	158991036	159558003	SCHIP1	0	0
chr3.fa	159000435	159000523	MIR3919	0	0
chr3.fa	159706629	159713806	IL12A	0	0
chr3.fa	159943423	159946000	LOC401097	0	0
chr3.fa	159974774	160117320	IFT80	1.765118012	380
chr3.fa	160117430	160152741	SMC4	2.196334319	532
chr3.fa	160122376	160122473	MIR15B	0	0
chr3.fa	160122533	160122613	MIR16-2	0	0
chr3.fa	160153291	160167626	TRIM59	0.43610891	76
chr3.fa	160217962	160283376	KPNA4	26.67291912	4560
chr3.fa	160232695	160233024	SCARNA7	0	0
chr3.fa	160394948	160396235	ARL14	7.873533842	456
chr3.fa	160473996	160788817	PPM1L	2.765433096	380
chr3.fa	160801671	160823160	B3GALNT1	1.856743138	304
chr3.fa	160939099	160969795	NMD3	19.12496582	2346
chr3.fa	161062580	161089871	C3orf57	43.97672437	4560
chr3.fa	161214596	161221730	OTOL1	0	0
chr3.fa	162895031	163021089	LOC647107	0	0
chr3.fa	164696686	164796283	SI	0	0
chr3.fa	164904508	164914469	SLITRK3	0	0

chr3.fa	165490692	165555253	BCHE	0	0
chr3.fa	166958077	167098085	ZBBX	0	0
chr3.fa	167159723	167191818	SERPINI2	0	0
chr3.fa	167196473	167371289	WDR49	0	0
chr3.fa	167401695	167452630	PDCD10	15.18530781	1057
chr3.fa	167453432	167543357	SERPINI1	0	0
chr3.fa	167613736	167641797	LOC646168	0	0
chr3.fa	167727654	167813417	GOLIM4	2.977816579	380
chr3.fa	167967310	168548374	EGFEM1P	0	0
chr3.fa	168269642	168269737	MIR551B	0	0
chr3.fa	168801287	169381563	MECOM	39.26959474	12048
chr3.fa	169482398	169482848	TERC	0	0
chr3.fa	169484711	169487683	ARPM1	1.687725916	152
chr3.fa	169490853	169507504	MYNN	4.723808958	1140
chr3.fa	169511216	169530574	LRRC34	0	0
chr3.fa	169539710	169555560	LRRIQ4	0	0
chr3.fa	169557029	169587660	LRRC31	0.685631704	76
chr3.fa	169629482	169656948	SAMD7	0	0
chr3.fa	169661772	169684522	LOC100128164	0	0
chr3.fa	169684580	169716161	SEC62	13.36192334	3930
chr3.fa	169755735	169803183	GPR160	36.10408009	3268
chr3.fa	169805368	169899537	PHC3	12.5375196	7144
chr3.fa	169940220	170023770	PRKCI	17.30313808	3800
chr3.fa	170075473	170110950	SKIL	41.92449947	6764
chr3.fa	170136653	170152479	CLDN11	0	0
chr3.fa	170177342	170303863	SLC7A14	0	0
chr3.fa	170582665	170588045	RPL22L1	9.476083974	836
chr3.fa	170606204	170626426	EIF5A2	0.305342955	76
chr3.fa	170714137	170744768	SLC2A2	0	0
chr3.fa	170780292	171178197	TNIK	3.603625081	987
chr3.fa	170824453	170824548	MIR569	0	0
chr3.fa	171318618	171528273	PLD1	3.627643318	912
chr3.fa	171561139	171577108	TMEM212	0	0
chr3.fa	171757418	172118492	FNDC3B	26.7182869	8512
chr3.fa	172161081	172166246	GHSR	0	0
chr3.fa	172223298	172241297	TNFSF10	8.100150354	1116
chr3.fa	172348435	172429008	NCEH1	4.690005514	910
chr3.fa	172472298	172539263	ECT2	3.45729175	608
chr3.fa	172607147	172859058	SPATA16	0	0
chr3.fa	173116244	174001116	NLGN1	0	0
chr3.fa	174577111	175523428	NAALADL2	4.817213212	1064
chr3.fa	176738542	176915048	TBL1XR1	35.86434251	10563
chr3.fa	178254224	178562217	KCNMB2	0	0

chr3.fa	178735011	178789656	ZMAT3	3.70814889	1520
chr3.fa	178866311	178952497	PIK3CA	8.658129577	1444
chr3.fa	178957537	178984838	KCNMB3	0.967178813	152
chr3.fa	179041551	179053320	ZNF639	5.019144314	680
chr3.fa	179065480	179111008	MFN1	13.47133975	2128
chr3.fa	179113876	179169371	GNB4	0.788821165	228
chr3.fa	179280708	179306193	ACTL6A	16.37421053	1368
chr3.fa	179306255	179322434	MRPL47	20.05278141	1064
chr3.fa	179322575	179342288	NDUFB5	28.7280351	1368
chr3.fa	179370933	179507189	USP13	2.982709182	1064
chr3.fa	179518050	179754517	PEX5L	0	0
chr3.fa	180319918	180328918	TTC14	0.706091683	152
chr3.fa	180331796	180397283	CCDC39	0	0
chr3.fa	180630234	180700539	FXR1	13.46978301	5396
chr3.fa	180701498	180707562	DNAJC19	9.529680225	1064
chr3.fa	181328151	181459005	SOX2OT	1.354806467	152
chr3.fa	181429722	181432223	SOX2	0	0
chr3.fa	182511291	182639421	ATP11B	13.62301047	4484
chr3.fa	182660559	182698326	DCUN1D1	8.043440628	1140
chr3.fa	182733006	182817365	MCCC1	33.28416118	3794
chr3.fa	182840003	182880667	LAMP3	0	0
chr3.fa	182895831	183145855	MCF2L2	0	0
chr3.fa	182971032	182991179	B3GNT5	2.869512122	532
chr3.fa	183205319	183273499	KLHL6	0	0
chr3.fa	183353411	183402304	KLHL24	14.06934937	4636
chr3.fa	183415606	183530413	YEATS2	11.15713816	3264
chr3.fa	183533664	183543393	MAP6D1	3.162178785	304
chr3.fa	183547173	183602693	PARL	33.51611508	2128
chr3.fa	183637724	183735727	ABCC5	3.105913841	986
chr3.fa	183749332	183757157	HTR3D	0	0
chr3.fa	183770835	183778461	HTR3C	0	0
chr3.fa	183817967	183824783	HTR3E	0	0
chr3.fa	183852810	183863099	EIF2B5	18.07972774	2356
chr3.fa	183873284	183891314	DVL3	17.02870749	3876
chr3.fa	183892634	183901879	AP2M1	149.7730543	13072
chr3.fa	183903863	183911795	ABCF3	23.60525654	2730
chr3.fa	183948317	183960117	VWA5B2	0.420541535	76
chr3.fa	183959193	183959277	MIR1224	0	0
chr3.fa	183960117	183967313	ALG3	3.688356083	304
chr3.fa	183967445	184010819	ECE2	0.775032918	152
chr3.fa	183977003	183979251	CAMK2N2	2.485442725	152
chr3.fa	184017022	184026840	PSMD2	58.90850635	7740
chr3.fa	184032283	184053146	EIF4G1	71.02704115	19760

chr3.fa	184043484	184043559	SNORD66	0	0
chr3.fa	184053717	184063975	FAM131A	1.866305954	228
chr3.fa	184064064	184079391	CLCN2	1.635686403	228
chr3.fa	184081194	184086363	POLR2H	24.70409088	912
chr3.fa	184089773	184095932	THPO	0	0
chr3.fa	184097861	184107617	CHRD	0	0
chr3.fa	184279587	184300196	EPHB3	2.002631687	380
chr3.fa	184428155	184429836	MAGEF1	28.13603004	2128
chr3.fa	184529931	184770402	VPS8	12.63003429	2864
chr3.fa	184795838	184870802	C3orf70	2.864174736	760
chr3.fa	184908412	184971886	EHHADH	13.18779112	2356
chr3.fa	185080970	185200628	MAP3K13	7.127411765	1140
chr3.fa	185207389	185216845	TMEM41A	1.793584071	228
chr3.fa	185225570	185270369	LIPH	15.02985645	1672
chr3.fa	185304031	185348885	SENP2	17.87334881	2567
chr3.fa	185361527	185542827	IGF2BP2	2.762542012	456
chr3.fa	185431040	185435955	C3orf65	0	0
chr3.fa	185632358	185655924	TRA2B	18.13154486	3496
chr3.fa	185686267	185697578	LOC344887	0	0
chr3.fa	185764106	185826901	ETV5	1.23604963	228
chr3.fa	185864990	186080023	DGKG	0	0
chr3.fa	186172770	186211450	LOC253573	0	0
chr3.fa	186256232	186262167	CRYGS	0	0
chr3.fa	186263856	186288332	TBCCD1	5.365629619	760
chr3.fa	186288467	186303589	DNAJB11	4.002817072	304
chr3.fa	186330850	186339107	AHSG	0	0
chr3.fa	186358149	186370797	FETUB	0	0
chr3.fa	186383798	186396023	HRG	2.59752783	228
chr3.fa	186435098	186462199	KNG1	8.675698473	836
chr3.fa	186501361	186507681	EIF4A2	197.5106343	16750
chr3.fa	186502585	186502654	SNORD2	0	0
chr3.fa	186504461	186504463	MIR1248	0	0
chr3.fa	186504567	186504641	SNORA81	0	0
chr3.fa	186505088	186505222	SNORA63	12.51972831	76
chr3.fa	186505402	186505538	SNORA4	12.33692284	76
chr3.fa	186507686	186524484	RFC4	1.069033928	76
chr3.fa	186560463	186576252	ADIPOQ	0	0
chr3.fa	186648315	186796341	ST6GAL1	5.35762354	1140
chr3.fa	186838741	186857263	RPL39L	7.212587549	228
chr3.fa	186915274	186919253	RTP1	0	0
chr3.fa	186933873	187009810	MASP1	0.169017222	76
chr3.fa	187086168	187089369	RTP4	3.343649907	152
chr3.fa	187386694	187388201	SST	0	0

chr3.fa	187416047	187420345	RTP2	0	0
chr3.fa	187433410	187450203	LOC100131635	0	0
chr3.fa	187439165	187463513	BCL6	28.00170583	5005
chr3.fa	187868994	187871876	LOC339929	3.706592152	456
chr3.fa	187896331	187898596	FLJ42393	0	0
chr3.fa	187930721	188608460	LPP	13.59209811	11206
chr3.fa	188889763	189041271	TPRG1	0.462128667	76
chr3.fa	189349216	189615068	TP63	1.813599268	532
chr3.fa	189547711	189547798	MIR944	0	0
chr3.fa	189674517	189840226	LEPREL1	18.47424951	3268
chr3.fa	190023490	190040235	CLDN1	0.489705161	76
chr3.fa	190105661	190129932	CLDN16	0	0
chr3.fa	190146444	190167665	TMEM207	0	0
chr3.fa	190231840	190374986	IL1RAP	0	0
chr3.fa	190570526	190580465	GEMC1	0.466354097	76
chr3.fa	190595719	190595839	SNAR-I	0	0
chr3.fa	190930322	190967910	OSTN	4.204303392	76
chr3.fa	190984944	191048325	UTS2D	0	0
chr3.fa	191046874	191116459	CCDC50	5.47704755	2204
chr3.fa	191178952	191179245	PYDC2	0	0
chr3.fa	191857182	192445388	FGF12	0.262643867	76
chr3.fa	192514605	192635950	C3orf59	6.023684828	912
chr3.fa	192958918	192988644	HRASLS	1.591430578	76
chr3.fa	192959568	192961761	MGC2889	0	0
chr3.fa	192992831	193096514	ATP13A5	0	0
chr3.fa	193119866	193272696	ATP13A4	0	0
chr3.fa	193310933	193415600	OPA1	15.35810568	4484
chr3.fa	193675161	193721448	LOC647323	0	0
chr3.fa	193710883	193712027	LOC100128023	0	0
chr3.fa	193853934	193856396	HES1	73.28342107	4788
chr3.fa	194018989	194030593	LOC100131551	0	0
chr3.fa	194060494	194072057	CPN2	1.116403228	152
chr3.fa	194075976	194090472	LRRC15	0	0
chr3.fa	194115550	194119995	GP5	0	0
chr3.fa	194123403	194188968	ATP13A3	5.074297302	1672
chr3.fa	194207869	194209274	FLJ34208	1.505142838	76
chr3.fa	194308402	194354150	TMEM44	0.671843457	76
chr3.fa	194361517	194393206	LSG1	19.40006359	3116
chr3.fa	194406622	194409766	FAM43A	4.836783627	684
chr3.fa	194789013	194991895	C3orf21	1.24227658	152
chr3.fa	194995465	195163817	ACAP2	14.5872982	4703
chr3.fa	195241221	195270224	PPP1R2	10.72503229	1672
chr3.fa	195295573	195311076	APOD	0	0

chr3.fa	195384910	195415735	SDHAP2	5.137233978	532
chr3.fa	195426272	195426368	MIR570	17.42456361	76
chr3.fa	195447753	195464540	MUC20	0.508608403	152
chr3.fa	195473638	195539148	MUC4	0.09874164	76
chr3.fa	195590236	195635880	TNK2	13.97705707	3040
chr3.fa	195686792	195717150	SDHAP1	7.463667079	836
chr3.fa	195776155	195809032	TFRC	8.618543965	2052
chr3.fa	195869507	195887761	LOC401109	2.773883957	228
chr3.fa	195924323	195938300	ZDHHC19	0	0
chr3.fa	195943383	195960301	OSTalpha	0	0
chr3.fa	195965253	196014584	PCYT1A	21.35399163	1520
chr3.fa	196018090	196045159	TCTEX1D2	10.04562753	304
chr3.fa	196050419	196065258	TM4SF19	0	0
chr3.fa	196080369	196159345	UBXN7	9.253692893	1969
chr3.fa	196195657	196230639	RNF168	10.12079572	2432
chr3.fa	196233750	196242237	C3orf43	0	0
chr3.fa	196281059	196295413	WDR53	1.854519227	131
chr3.fa	196295725	196315930	FBXO45	6.485813495	1672
chr3.fa	196366656	196388874	LRRC33	0	0
chr3.fa	196433148	196439123	C3orf34	3.129932078	304
chr3.fa	196439245	196462876	PIGX	3.789099243	527
chr3.fa	196466728	196559518	PAK2	19.73943238	5449
chr3.fa	196594727	196661584	SENP5	10.18328761	2888
chr3.fa	196662273	196669464	NCBP2	31.15521136	3040
chr3.fa	196669494	196670884	LOC152217	10.93563664	684
chr3.fa	196673214	196695704	PIGZ	0.625808503	76
chr3.fa	196728612	196756686	MFI2	0.702755817	152
chr3.fa	196769431	197025447	DLG1	28.79497482	6562
chr3.fa	197236654	197300194	BDH1	11.91237827	1976
chr3.fa	197340898	197354752	LOC220729	1.106840412	76
chr3.fa	197398259	197476568	KIAA0226	12.0262425	3800
chr3.fa	197476624	197511317	FYTTD1	14.50612545	2812
chr3.fa	197518145	197598456	LRCH3	23.20428542	2356
chr3.fa	197615946	197686886	IQCG	3.603847472	380
chr3.fa	197677052	197682721	RPL35A	1623.270754	35255
chr3.fa	197687071	197770591	LMLN	1.441983771	456
chr3.fa	197784404	197807542	LOC348840	0	0
chr3.fa	197879237	197907728	FAM157A	0	0
chr4.fa	53227	88099	ZNF595	4.099557193	466
chr4.fa	154702	156490	ZNF718	0	0
chr4.fa	206389	249773	ZNF876P	0.648270002	76
chr4.fa	264464	289944	ZNF732	0	0
chr4.fa	331596	367691	ZNF141	3.088122555	228

chr4.fa	419224	467998	ABCA11P	1.911896126	152
chr4.fa	433779	493442	ZNF721	4.404677756	941
chr4.fa	494185	533320	PIGG	3.997034904	532
chr4.fa	619363	664681	PDE6B	1.429974653	228
chr4.fa	666225	668127	ATP5I	147.3265301	2418
chr4.fa	671711	674929	MYL5	10.33584789	244
chr4.fa	675618	682973	MFSD7	0.97696402	76
chr4.fa	699573	764427	PCGF3	15.44772929	3935
chr4.fa	773937	775636	LOC100129917	1.988398658	152
chr4.fa	778745	819945	CPLX1	2.36935458	228
chr4.fa	843065	926174	GAK	20.14329458	4104
chr4.fa	926262	952443	TMEM175	0.953835348	76
chr4.fa	952675	967344	DGKQ	3.645656996	760
chr4.fa	972863	987183	SLC26A1	0.432550653	76
chr4.fa	980785	998317	IDUA	0	0
chr4.fa	1005610	1020686	FGFRL1	1.566522777	228
chr4.fa	1065266	1107352	RNF212	0.55531053	76
chr4.fa	1108985	1116952	TMED11P	0	0
chr4.fa	1160721	1166999	SPON2	9.344428454	608
chr4.fa	1189571	1198904	LOC100130872	0	0
chr4.fa	1205228	1242908	CTBP1	53.09431392	5928
chr4.fa	1243228	1246795	C4orf42	9.806557121	1292
chr4.fa	1283672	1333925	MAEA	21.61930419	2128
chr4.fa	1341104	1381837	KIAA1530	3.623195497	760
chr4.fa	1385340	1389782	CRIPAK	1.902110918	380
chr4.fa	1641608	1685988	FAM53A	0.596008098	76
chr4.fa	1694527	1714030	SLBP	20.57606762	1596
chr4.fa	1717679	1723084	TMEM129	7.744991797	988
chr4.fa	1723266	1746897	TACC3	1.216034433	152
chr4.fa	1795039	1810599	FGFR3	6.855205081	1368
chr4.fa	1813206	1857974	LETM1	8.973924913	2204
chr4.fa	1873123	1983934	WHSC1	2.474323171	1900
chr4.fa	1976363	1976487	SCARNA22	0	0
chr4.fa	1984443	2010959	WHSC2	8.990381853	988
chr4.fa	1988130	1988204	MIR943	0	0
chr4.fa	2043720	2045697	C4orf48	3.129932078	76
chr4.fa	2061239	2070816	NAT8L	0.287774059	76
chr4.fa	2073645	2214853	POLN	0.611130691	76
chr4.fa	2230096	2243860	HAUS3	1.545840406	380
chr4.fa	2249160	2263739	MXD4	39.68035107	6732
chr4.fa	2271324	2420370	ZFYVE28	0.864656524	228
chr4.fa	2420701	2464690	LOC402160	0	0
chr4.fa	2470795	2517586	RNF4	23.57078592	3894

chr4.fa	2627159	2734302	FAM193A	11.7845034	2508
chr4.fa	2743387	2758103	TNIP2	16.57836555	1520
chr4.fa	2794750	2842823	SH3BP2	3.275598236	1442
chr4.fa	2845584	2931789	ADD1	60.66005851	11096
chr4.fa	2932288	2936586	MFSD10	3.059211714	282
chr4.fa	2937278	2952794	C4orf10	4.239663574	684
chr4.fa	2939870	2965118	NOP14	23.86723324	2844
chr4.fa	2965343	3042474	GRK4	1.460887013	152
chr4.fa	3076408	3245687	HTT	16.17895116	9803
chr4.fa	3250767	3258342	C4orf44	0	0
chr4.fa	3315874	3441640	RGS12	2.859059741	903
chr4.fa	3443726	3451213	HGFAC	0	0
chr4.fa	3465033	3496209	DOK7	0	0
chr4.fa	3514290	3534224	LRPAP1	10.76550746	760
chr4.fa	3675320	3679582	LOC100133461	0	0
chr4.fa	3768296	3770253	ADRA2C	0.863322178	76
chr4.fa	3943669	3957148	LOC348926	1.485572423	228
chr4.fa	4190530	4228621	OTOP1	0	0
chr4.fa	4237269	4249934	TMEM128	1.951704129	152
chr4.fa	4269429	4291896	LYAR	1.053688943	76
chr4.fa	4291924	4323513	ZBTB49	3.445727414	456
chr4.fa	4387983	4420695	D4S234E	4.048407244	456
chr4.fa	4420786	4543775	STX18	17.93539592	1667
chr4.fa	4861392	4865660	MSX1	0	0
chr4.fa	5016314	5021197	CYTL1	0	0
chr4.fa	5053527	5502725	STK32B	0.986304446	143
chr4.fa	5526883	5529527	C4orf6	0	0
chr4.fa	5564146	5711275	EVC2	2.306195513	532
chr4.fa	5712924	5816031	EVC	6.045701545	1748
chr4.fa	5822491	5894785	CRMP1	1.44576442	228
chr4.fa	6027926	6202318	JAKMIP1	10.22487474	1520
chr4.fa	6271577	6304992	WFS1	0.928705156	152
chr4.fa	6322305	6474326	PPP2R2C	6.339924946	1292
chr4.fa	6576902	6624188	MAN2B2	1.593654489	304
chr4.fa	6642445	6644449	MRFAP1	193.9181288	13376
chr4.fa	6675821	6677774	LOC93622	16.21275461	1216
chr4.fa	6695566	6698897	S100P	440.7704499	10108
chr4.fa	6709429	6711606	MRFAP1L1	52.05441323	3724
chr4.fa	6717842	6719387	CNO	9.83924861	684
chr4.fa	6784459	6885899	KIAA0232	19.79080472	6992
chr4.fa	6911171	7032280	TBC1D14	26.9775949	3268
chr4.fa	7034846	7047958	LOC100129931	0	0
chr4.fa	7042576	7044728	CCDC96	1.570081034	152

chr4.fa	7045156	7059677	TADA2B	13.33323489	2584
chr4.fa	7061780	7069800	GRPEL1	28.0946653	1900
chr4.fa	7099151	7105103	FLJ36777	0.78971073	76
chr4.fa	7194374	7744564	SORCS2	0	0
chr4.fa	7432021	7436700	PSAPL1	0	0
chr4.fa	7461755	7461845	MIR4274	0	0
chr4.fa	7755817	7780654	AFAP1-AS1	0.26731408	76
chr4.fa	7760440	7941653	AFAP1	3.93654453	1292
chr4.fa	7967037	8160559	ABLIM2	0.699642342	152
chr4.fa	8201060	8242830	SH3TC1	5.157693957	988
chr4.fa	8271489	8308838	HTRA3	0	0
chr4.fa	8368009	8442452	ACOX3	24.11164103	3116
chr4.fa	8442532	8478282	C4orf23	7.720306387	988
chr4.fa	8582291	8589520	GPR78	0	0
chr4.fa	8594387	8621488	CPZ	0	0
chr4.fa	8868773	8873543	HMX1	0	0
chr4.fa	8951477	8952127	LOC650293	0	0
chr4.fa	9217131	9366447	USP17	0	0
chr4.fa	9369600	9370796	USP17L6P	0	0
chr4.fa	9446260	9452240	DEFB131	0	0
chr4.fa	9557789	9557937	MIR548I2	0	0
chr4.fa	9783258	9785633	DRD5	0	0
chr4.fa	9827848	10041872	SLC2A9	2.438962989	228
chr4.fa	10075963	10118573	WDR1	81.20143312	11476
chr4.fa	10080235	10080316	MIR3138	0	0
chr4.fa	10441504	10459032	ZNF518B	7.066698999	2204
chr4.fa	10491838	10686386	CLNK	0	0
chr4.fa	11370451	11370545	MIR572	0	0
chr4.fa	11399988	11430537	HS3ST1	0.860208702	76
chr4.fa	13335037	13339925	HSP90AB2P	0	0
chr4.fa	13369347	13485989	RAB28	2.691376866	228
chr4.fa	13527943	13533041	LOC285547	0	0
chr4.fa	13542454	13546114	NKX3-2	0	0
chr4.fa	13547700	13549448	LOC285548	0	0
chr4.fa	13570366	13629328	BOD1L	11.38330989	5396
chr4.fa	14113592	14141676	LOC152742	0	0
chr4.fa	15004298	15071777	CPEB2	6.871662021	2128
chr4.fa	15341560	15447791	C1QTNF7	0	0
chr4.fa	15471489	15603180	CC2D2A	3.68969043	1064
chr4.fa	15606007	15657035	FBXL5	45.9184209	7524
chr4.fa	15683352	15692070	FAM200B	6.167127076	1216
chr4.fa	15704573	15733796	BST1	0	0
chr4.fa	15779931	15850706	CD38	4.534331757	304

chr4.fa	15937193	15939971	FGFBP1	0	0
chr4.fa	15961863	15964859	FGFBP2	0	0
chr4.fa	15969849	16085623	PROM1	5.431902161	1052
chr4.fa	16162128	16228161	TAPT1	4.420689914	912
chr4.fa	16228286	16259810	FLJ39653	0	0
chr4.fa	16503165	16900424	LDB2	0	0
chr4.fa	17488016	17513857	QDPR	63.39413446	4712
chr4.fa	17516788	17528727	CLRN2	0	0
chr4.fa	17578927	17609590	LAP3	25.49024335	2407
chr4.fa	17616273	17626160	MED28	17.15235693	988
chr4.fa	17633709	17783135	FAM184B	0.439889559	76
chr4.fa	17802278	17812381	DCAF16	9.6624477	1976
chr4.fa	17812525	17844002	NCAPG	0	0
chr4.fa	17844839	18023483	LCORL	2.192998453	608
chr4.fa	20255235	20620788	SLIT2	1.307437167	291
chr4.fa	20529898	20530007	MIR218-1	0	0
chr4.fa	20702036	20729980	PACRGL	4.076650911	380
chr4.fa	20730239	21950374	KCNIP4	0.533960986	76
chr4.fa	21844964	21854811	NCRNA00099	0	0
chr4.fa	22388999	22517672	GPR125	1.85318488	380
chr4.fa	22694548	22821192	GBA3	17.31892785	1672
chr4.fa	23793644	23891700	PPARGC1A	5.082748163	1444
chr4.fa	24521815	24521913	MIR573	0	0
chr4.fa	24529088	24586184	DHX15	49.89232713	6764
chr4.fa	24797085	24802467	SOD3	6.632369218	456
chr4.fa	24807739	24981826	CCDC149	9.229452265	1753
chr4.fa	25000471	25032414	LGI2	0	0
chr4.fa	25121627	25162204	SEPSECS	4.790526282	1216
chr4.fa	25235653	25280831	PI4K2B	8.448414787	1368
chr4.fa	25314396	25372005	ZCCHC4	1.812932095	228
chr4.fa	25378848	25420120	ANAPC4	3.795326193	456
chr4.fa	25657435	25680368	SLC34A2	0	0
chr4.fa	25749049	25864610	SEL1L3	3.715487795	760
chr4.fa	25915814	25931501	C4orf52	21.31796427	972
chr4.fa	26321332	26433278	RBPJ	14.51835696	1824
chr4.fa	26483018	26492042	CCKAR	0	0
chr4.fa	26585546	26756918	TBC1D19	2.304861167	228
chr4.fa	26862313	27027003	STIM2	3.866268948	912
chr4.fa	28821204	28821290	MIR4275	0	0
chr4.fa	30722037	31148423	PCDH7	0	0
chr4.fa	36067622	36245979	ARAP2	8.442410228	2794
chr4.fa	36283244	36347378	DTHD1	0	0
chr4.fa	37246690	37451087	KIAA1239	0	0

chr4.fa	37455552	37595132	C4orf19	4.005040983	608
chr4.fa	37612256	37687999	RELL1	2.67380797	435
chr4.fa	37828282	37864559	PGM2	17.74725307	2584
chr4.fa	37892720	38140794	TBC1D1	55.86352767	14288
chr4.fa	37962056	37962631	PTTG2	0	0
chr4.fa	38614322	38666249	FLJ13197	1.623677285	152
chr4.fa	38665790	38703129	KLF3	18.37061527	4402
chr4.fa	38773860	38784611	TLR10	0	0
chr4.fa	38797876	38806412	TLR1	0.592894623	76
chr4.fa	38825329	38858437	TLR6	0	0
chr4.fa	38869354	38947365	FAM114A1	14.20434075	2643
chr4.fa	38869653	38869748	MIR574	0	0
chr4.fa	38968441	39034041	TMEM156	0	0
chr4.fa	39046451	39127853	KLHL5	2.96380594	1064
chr4.fa	39184024	39287430	WDR19	4.098445237	833
chr4.fa	39289076	39367995	RFC1	22.49863852	4940
chr4.fa	39408473	39453153	KLB	0	0
chr4.fa	39455745	39460568	RPL9	24.99408885	1007
chr4.fa	39460665	39479271	LIAS	0.978075976	76
chr4.fa	39481875	39483523	LOC401127	0	0
chr4.fa	39500375	39529218	UGDH	33.31685267	4788
chr4.fa	39552546	39640481	C4orf34	76.3346267	5691
chr4.fa	39699664	39784410	UBE2K	19.96560411	4707
chr4.fa	39824483	39979576	PDS5A	22.45571704	7668
chr4.fa	40044537	40058819	LOC344967	0	0
chr4.fa	40058524	40157019	N4BP2	2.757204626	836
chr4.fa	40198527	40246281	RHOH	0	0
chr4.fa	40337469	40356973	CHRNA9	0	0
chr4.fa	40425272	40631883	RBM47	73.4297544	17328
chr4.fa	40751914	40812002	NSUN7	6.398636191	1064
chr4.fa	40812044	41216635	APBB2	2.761652448	1140
chr4.fa	41258898	41270446	UCHL1	4.511203084	228
chr4.fa	41362804	41702061	LIMCH1	50.1376245	14343
chr4.fa	41746099	41750987	PHOX2B	0	0
chr4.fa	41937137	41962824	TMEM33	1.75221933	608
chr4.fa	41983713	41988484	DCAF4L1	0	0
chr4.fa	41992523	42089551	SLC30A9	31.09783446	4560
chr4.fa	42112870	42154895	BEND4	4.201634699	1672
chr4.fa	42399856	42404504	SHISA3	0.857540009	76
chr4.fa	42410392	42659122	ATP8A1	1.620786201	608
chr4.fa	42895283	43032673	GRXCR1	0	0
chr4.fa	44175920	44450824	KCTD8	0	0
chr4.fa	44624354	44653658	YIPF7	0	0

chr4.fa	44680433	44702697	GUF1	7.216145806	1368
chr4.fa	44704168	44728612	GNPDA2	5.287347958	456
chr4.fa	46037787	46126082	GABRG1	0	0
chr4.fa	46251581	46392056	GABRA2	0	0
chr4.fa	46736847	46911252	COX7B2	0	0
chr4.fa	46920917	46995580	GABRA4	0	0
chr4.fa	47033295	47428447	GABRB1	0	0
chr4.fa	47452811	47465676	COMMD8	13.17644918	836
chr4.fa	47487410	47595503	ATP10D	2.838377371	836
chr4.fa	47596018	47840059	CORIN	0	0
chr4.fa	47849258	47916633	NFXL1	12.19659407	2044
chr4.fa	47937994	48014961	CNGA1	6.154228393	836
chr4.fa	48018791	48039080	NIPAL1	14.64334075	1442
chr4.fa	48068410	48136273	TXK	2.8999797	380
chr4.fa	48137800	48271814	TEC	4.202079481	684
chr4.fa	48343613	48428215	SLAIN2	21.65088372	5852
chr4.fa	48485360	48491541	SLC10A4	0	0
chr4.fa	48492309	48496422	ZAR1	0	0
chr4.fa	48499380	48782316	FRYL	8.807576384	4636
chr4.fa	48833060	48863834	OCIAD1	8.848941125	910
chr4.fa	48887405	48908815	OCIAD2	77.45836883	2661
chr4.fa	48988265	49064095	CWH43	10.26423797	1140
chr4.fa	52709276	52783003	DCUN1D4	9.740284579	1868
chr4.fa	52859866	52883786	LRRC66	0	0
chr4.fa	52886861	52904485	SGCB	1.975055193	380
chr4.fa	52917593	52963458	SPATA18	3.14460989	608
chr4.fa	53457127	53525502	USP46	9.410256214	3496
chr4.fa	53578621	53580305	KIAA0114	140.3534577	5396
chr4.fa	53579416	53579537	SNORA26	0	0
chr4.fa	53728495	53733002	RASL11B	24.1823614	2128
chr4.fa	53739151	54232242	SCFD2	5.325154442	760
chr4.fa	54243820	54326103	FIP1L1	22.44504227	2508
chr4.fa	54326437	54457724	LNX1	12.15611889	1900
chr4.fa	54851666	54853449	RPL21P44	0	0
chr4.fa	54875958	54930788	CHIC2	9.030412247	456
chr4.fa	54966248	54968122	GSX2	0	0
chr4.fa	55095264	55164412	PDGFRA	0	0
chr4.fa	55524095	55606881	KIT	0.327359672	76
chr4.fa	55944426	55991762	KDR	0	0
chr4.fa	56212409	56237866	SRD5A3	0	0
chr4.fa	56262090	56292342	TMEM165	10.32161487	912
chr4.fa	56298660	56412997	CLOCK	9.323523692	2432
chr4.fa	56422692	56458379	PDCL2	0	0

chr4.fa	56461398	56502465	NMU	0	0
chr4.fa	56686237	56703430	LOC644145	0	0
chr4.fa	56719816	56771244	EXOC1	15.78687569	2432
chr4.fa	56815037	56899527	CEP135	2.384699565	608
chr4.fa	57036361	57196890	KIAA1211	0	0
chr4.fa	57204457	57253638	AASDH	5.695657983	912
chr4.fa	57259529	57301845	PPAT	2.256602302	380
chr4.fa	57301915	57327534	PAICS	11.52719692	1824
chr4.fa	57333762	57369847	SRP72	15.28516141	2664
chr4.fa	57371375	57390058	ARL9	0	0
chr4.fa	57514154	57547872	HOPX	24.9602854	2128
chr4.fa	57676034	57687893	SPINK2	0	0
chr4.fa	57774042	57802010	REST	16.76584123	5624
chr4.fa	57829516	57843826	C4orf14	14.13428756	1444
chr4.fa	57845109	57897243	POLR2B	32.81447122	5396
chr4.fa	57897335	57976539	IGFBP7	10.96321313	456
chr4.fa	57975928	58071465	LOC255130	0	0
chr4.fa	62362839	62938168	LPHN3	0.275987332	76
chr4.fa	65144177	65275178	TECRL	0	0
chr4.fa	65779999	65870218	LOC401134	0	0
chr4.fa	66185281	66535653	EPHA5	0.645378918	228
chr4.fa	66535679	66559104	LOC100144602	4.307937636	228
chr4.fa	68337989	68411256	CENPC1	7.430753199	1119
chr4.fa	68424446	68472616	STAP1	2.237254278	152
chr4.fa	68481479	68566889	UBA6	6.205600733	1799
chr4.fa	68566996	68588222	LOC550112	12.54708242	1292
chr4.fa	68603099	68621804	GNRHR	0.289553188	76
chr4.fa	68686594	68749716	TMPRSS11D	0	0
chr4.fa	68776019	68829232	TMPRSS11A	0	0
chr4.fa	68857405	68863301	LOC644759	0	0
chr4.fa	68918916	68995587	TMPRSS11F	0	0
chr4.fa	68926328	68929015	SYT14L	0	0
chr4.fa	69048010	69078079	FTLP10	0	0
chr4.fa	69054242	69083798	TMPRSS11BNL	0	0
chr4.fa	69092371	69111412	TMPRSS11B	0	0
chr4.fa	69176105	69215824	YTHDC1	26.06846016	7325
chr4.fa	69313167	69363322	TMPRSS11E	0	0
chr4.fa	69402903	69434245	UGT2B17	0	0
chr4.fa	69512315	69536374	UGT2B15	0	0
chr4.fa	69681713	69886113	UGT2B10	0	0
chr4.fa	69794177	69817509	UGT2A3	1.102614981	152
chr4.fa	69962193	69978705	UGT2B7	15.22667255	1292
chr4.fa	70066051	70080449	UGT2B11	0	0

chr4.fa	70146217	70160768	UGT2B28	0	0
chr4.fa	70345883	70361626	UGT2B4	0	0
chr4.fa	70454135	70513417	UGT2A1	0	0
chr4.fa	70504617	70505334	UGT2A2	0	0
chr4.fa	70592686	70626430	SULT1B1	0	0
chr4.fa	70706930	70725870	SULT1E1	0	0
chr4.fa	70796799	70812288	CSN1S1	0	0
chr4.fa	70820974	70826726	CSN2	0	0
chr4.fa	70861648	70868173	STATH	0	0
chr4.fa	70894130	70902255	HTN3	0	0
chr4.fa	70916159	70924559	HTN1	0	0
chr4.fa	70933103	70950988	CSN1S2AP	0	0
chr4.fa	70999321	71012421	CSN1S2BP	0	0
chr4.fa	71019904	71032326	C4orf40	0	0
chr4.fa	71062244	71070293	ODAM	3.936766921	228
chr4.fa	71091815	71100968	C4orf7	0	0
chr4.fa	71108333	71117145	CSN3	0	0
chr4.fa	71200671	71202833	C4orf35	0	0
chr4.fa	71226493	71232823	SMR3A	0	0
chr4.fa	71248795	71255961	SMR3B	0	0
chr4.fa	71263599	71275914	PROL1	0	0
chr4.fa	71296209	71348714	MUC7	0	0
chr4.fa	71384298	71398459	AMTN	0	0
chr4.fa	71458001	71473004	AMBN	0	0
chr4.fa	71494461	71512536	ENAM	0	0
chr4.fa	71521258	71532348	IGJ	0	0
chr4.fa	71554196	71556268	UTP3	20.38325456	1900
chr4.fa	71570654	71674336	RUFY3	4.754943709	1520
chr4.fa	71681499	71705627	GRSF1	12.78570805	3952
chr4.fa	71768064	71853891	MOBKL1A	7.044459891	2204
chr4.fa	71859265	71896629	DCK	3.892955878	456
chr4.fa	72053003	72437804	SLC4A4	19.83483815	7144
chr4.fa	72607411	72649888	GC	8.545822081	684
chr4.fa	72897521	73013918	NPFFR2	0	0
chr4.fa	73146687	73434516	ADAMTS3	0	0
chr4.fa	73920416	73935472	COX18	3.363220322	684
chr4.fa	73940502	74124502	ANKRD17	45.61218838	19228
chr4.fa	74269972	74287129	ALB	39.05454257	3946
chr4.fa	74301933	74321492	AFP	0	0
chr4.fa	74347462	74369718	AFM	0.846420455	76
chr4.fa	74438862	74486340	RASSF6	5.313812497	1064
chr4.fa	74606275	74609433	IL8	2.044885993	152
chr4.fa	74702273	74704477	CXCL6	0	0

chr4.fa	74719013	74720198	PF4V1		0	0
chr4.fa	74735109	74736955	CXCL1		0	0
chr4.fa	74846796	74847715	PF4		0	0
chr4.fa	74852156	74853907	PPBP		0	0
chr4.fa	74861359	74864416	CXCL5		0	0
chr4.fa	74902312	74904490	CXCL3		0	0
chr4.fa	74919755	74921116	PPBPL2		0	0
chr4.fa	74962754	74964997	CXCL2		2.809911312	152
chr4.fa	75023829	75168814	MTHFD2L		3.582497929	380
chr4.fa	75174204	75179307	EPGN		0	0
chr4.fa	75230860	75254477	EREG		0	0
chr4.fa	75310853	75490485	AREG		0	0
chr4.fa	75671448	75719882	BTC		1.277636762	76
chr4.fa	75858285	75975325	PARM1		7.357364143	1672
chr4.fa	76279286	76287776	LOC441025		0	0
chr4.fa	76404347	76439628	RCHY1		8.048110841	1563
chr4.fa	76439654	76455236	THAP6		2.361793283	380
chr4.fa	76481258	76489927	C4orf26		0	0
chr4.fa	76501704	76555721	CDKL2		0.358494423	76
chr4.fa	76567953	76598667	G3BP2		60.53574189	12388
chr4.fa	76649829	76735366	USO1		23.82386698	4104
chr4.fa	76781026	76823681	PPEF2		0	0
chr4.fa	76831808	76862166	NAAA		1.319446285	152
chr4.fa	76871068	76912113	SDAD1		19.35047037	2626
chr4.fa	76922623	76928641	CXCL9		0	0
chr4.fa	76932337	77033955	ART3		0	0
chr4.fa	76942271	76944650	CXCL10		0	0
chr4.fa	76954840	76957350	CXCL11		0	0
chr4.fa	77035817	77069655	NUP54		11.48805609	1216
chr4.fa	77079894	77135035	SCARB2		13.78001857	2942
chr4.fa	77172853	77204929	FAM47E		6.555199512	456
chr4.fa	77227677	77232283	STBD1		8.957245581	988
chr4.fa	77234192	77328458	CCDC158		0	0
chr4.fa	77356253	77704405	SHROOM3		18.86654738	9348
chr4.fa	77816082	77819002	ANKRD56		12.15122629	1596
chr4.fa	77870895	77959768		11-Sep	13.38749831	3344
chr4.fa	77969177	77997125	CCNI		274.3258481	23104
chr4.fa	78078357	78091213	CCNG2		36.12542964	8892
chr4.fa	78432907	78532988	CXCL13		0	0
chr4.fa	78634541	78740544	CNOT6L		6.919031321	2736
chr4.fa	78783805	78873944	MRPL1		3.731055171	228
chr4.fa	78978724	79465423	FRAS1		1.736651954	1292
chr4.fa	79472742	79531605	ANXA3		73.47979239	5346

chr4.fa	79697532	79833341	BMP2K	4.884820101	836
chr4.fa	79839094	79860582	PAQR3	0.904019745	152
chr4.fa	80238272	80247171	NAA11	0	0
chr4.fa	80327507	80329372	GK2	0	0
chr4.fa	80748625	80784401	GDEP	75.1726333	7068
chr4.fa	80822771	80994477	ANTXR2	3.937878877	1444
chr4.fa	81106424	81125482	PRDM8	15.62386303	2964
chr4.fa	81187742	81212171	FGF5	0	0
chr4.fa	81256874	81884902	C4orf22	0	0
chr4.fa	81952119	81978685	BMP3	1.473785696	380
chr4.fa	82009837	82126215	PRKG2	7.61800649	1140
chr4.fa	82348219	82393061	RASGEF1B	5.959191414	608
chr4.fa	83274467	83295149	HNRNPD	67.99829702	6901
chr4.fa	83344347	83351378	HNRPDL	21.50699669	3496
chr4.fa	83351726	83382244	ENOPH1	27.69391657	2508
chr4.fa	83405604	83483126	TMEM150C	0.895124102	76
chr4.fa	83534266	83542590	C4orf11	0	0
chr4.fa	83550690	83720010	SCD5	0	0
chr4.fa	83674490	83674583	MIR575	0	0
chr4.fa	83739814	83812419	SEC31A	62.74608685	12008
chr4.fa	83814605	83822069	LOC100499177	7.212587549	684
chr4.fa	83825889	83841284	THAP9	0.440334341	76
chr4.fa	83845756	83934040	LIN54	1.901221354	532
chr4.fa	83956239	83996971	COPS4	19.33846126	1520
chr4.fa	84011211	84035911	PLAC8	2.474545562	228
chr4.fa	84184977	84206067	COQ2	2.060008586	152
chr4.fa	84213614	84256306	HPSE	0	0
chr4.fa	84328499	84377025	HELQ	4.705350498	760
chr4.fa	84377118	84380950	MRPS18C	14.38670144	262
chr4.fa	84382094	84406290	FAM175A	1.60432926	152
chr4.fa	84457653	84527026	AGPAT9	1.333901706	152
chr4.fa	85414436	85419387	NKX6-1	0	0
chr4.fa	85504057	85572493	CDS1	5.214181292	1064
chr4.fa	85590693	85887544	WDFY3	22.89783051	14741
chr4.fa	85887971	85928168	NCRNA00247	2.154524796	152
chr4.fa	86396284	86923823	ARHGAP24	5.056728407	1292
chr4.fa	86936276	87374283	MAPK10	10.95743097	2128
chr4.fa	87515468	87736328	PTPN13	25.64947536	9875
chr4.fa	87744621	87770416	SLC10A6	0	0
chr4.fa	87797358	87813575	C4orf36	0	0
chr4.fa	87856154	88062206	AFF1	42.19670616	18422
chr4.fa	88082214	88141674	KLHL8	7.756556134	1596
chr4.fa	88224941	88244056	HSD17B13	0	0

chr4.fa	88257691	88312455	HSD17B11	7.21903689	608
chr4.fa	88343735	88379499	NUDT9	13.62034177	836
chr4.fa	88394488	88450655	SPARCL1	0	0
chr4.fa	88529681	88538025	DSPP	0	0
chr4.fa	88571454	88585512	DMP1	0	0
chr4.fa	88720702	88733601	IBSP	0	0
chr4.fa	88742563	88767968	MEPE	0	0
chr4.fa	88812995	88815167	HSP90AB3P	0	0
chr4.fa	88896802	88904563	SPP1	15.60162392	1140
chr4.fa	88928820	88998929	PKD2	3.008506548	684
chr4.fa	89011416	89080011	ABCG2	0.381400704	76
chr4.fa	89181532	89205888	PPM1K	7.715636175	1292
chr4.fa	89299891	89364249	HERC6	9.126707585	1596
chr4.fa	89378268	89427314	HERC5	0.481031909	76
chr4.fa	89442135	89444955	PIGY	56.2142384	3420
chr4.fa	89513647	89629686	HERC3	14.85038684	3268
chr4.fa	89617066	89619023	NAP1L5	3.452843928	304
chr4.fa	89630940	89651254	FAM13AOS	0	0
chr4.fa	89647106	89978323	FAM13A	14.07490914	3360
chr4.fa	90033968	90036052	TIGD2	7.295761813	684
chr4.fa	90165429	90229161	GPRIN3	0.532181858	152
chr4.fa	90645250	90759447	SNCA	5.829759805	912
chr4.fa	90816052	90875780	MMRN1	0	0
chr4.fa	91048684	92523370	FAM190A	6.066161525	2204
chr4.fa	91759642	91760269	TMSL3	2.691376866	76
chr4.fa	93225550	94693649	GRID2	0	0
chr4.fa	94750078	94751142	ATOH1	0	0
chr4.fa	95128759	95212443	SMARCAD1	8.082359068	1900
chr4.fa	95219707	95264027	HPGDS	0	0
chr4.fa	95373038	95589377	PDLIM5	50.29552216	16695
chr4.fa	95679128	96079601	BMPR1B	7.295761813	1824
chr4.fa	96083656	96470361	UNC5C	0.342259874	152
chr4.fa	96761239	96762625	PDHA2	0	0
chr4.fa	98480025	99064391	C4orf37	0	0
chr4.fa	99182527	99365012	RAP1GDS1	8.538483175	1444
chr4.fa	99391518	99579812	TSPAN5	1.990177787	304
chr4.fa	99799607	99851786	EIF4E	4.694898117	1064
chr4.fa	99916788	99983960	METAP1	32.18643881	4009
chr4.fa	99918538	99918611	MIR3684	0	0
chr4.fa	99992130	100009931	ADH5	53.07763459	6308
chr4.fa	100044833	100065449	ADH4	0	0
chr4.fa	100081751	100082804	PCNAP1	0	0
chr4.fa	100123795	100140403	ADH6	1.124631698	152

chr4.fa	100197523	100212185	ADH1A	1.126855609	76
chr4.fa	100227527	100242572	ADH1B	0	0
chr4.fa	100257649	100273917	ADH1C	0	0
chr4.fa	100333418	100356667	ADH7	0	0
chr4.fa	100432161	100463460	C4orf17	0	0
chr4.fa	100467864	100485189	RG9MTD2	0.881780637	152
chr4.fa	100485240	100545154	MTTP	0	0
chr4.fa	100737981	100791346	DAPP1	12.66050187	1672
chr4.fa	100799495	100815703	MAPKSP1	14.37224602	2766
chr4.fa	100820710	100867879	DNAJB14	3.055208675	380
chr4.fa	100869244	100871512	H2AFZ	42.83430139	1824
chr4.fa	100871636	100873620	LOC256880	0	0
chr4.fa	101107027	101111655	DDIT4L	0.64159827	76
chr4.fa	101316498	101439250	EMCN	0	0
chr4.fa	101944587	102268628	PPP3CA	85.3038814	17936
chr4.fa	102268934	102270040	FLJ20021	27.70770482	988
chr4.fa	102711764	102995969	BANK1	9.500769384	1520
chr4.fa	103172198	103266655	SLC39A8	2.333772007	608
chr4.fa	103422486	103538459	NFKB1	15.7117075	2886
chr4.fa	103552643	103682151	MANBA	2.55238244	380
chr4.fa	103717133	103790032	UBE2D3	53.16414472	6947
chr4.fa	103790135	103813963	CISD2	1.755110414	456
chr4.fa	103806205	103940896	NHEDC1	0	0
chr4.fa	103946652	103998170	NHEDC2	0	0
chr4.fa	103998782	104021024	BDH2	11.57656774	1520
chr4.fa	104026963	104119566	CENPE	0	0
chr4.fa	104510625	104640973	TACR3	0	0
chr4.fa	105393343	105412467	CXXC4	4.519209163	152
chr4.fa	106067943	106200958	TET2	8.898089554	6004
chr4.fa	106290234	106395227	PPA2	20.25849316	1524
chr4.fa	106405863	106407507	EEF1A1P9	14.38447753	1064
chr4.fa	106473777	106552837	ARHGEF38	2.352452858	152
chr4.fa	106603785	106629881	INTS12	18.32947292	1444
chr4.fa	106629941	106766910	GSTCD	9.92976178	1064
chr4.fa	106816597	106892828	NPNT	1.071257839	228
chr4.fa	106967233	107237861	TBCK	9.861710109	1470
chr4.fa	107236767	107270381	AIMP1	13.8367283	1790
chr4.fa	107279340	107288592	LOC100507096	0	0
chr4.fa	107842959	107957453	DKK2	0	0
chr4.fa	108534822	108641419	PAPSS1	35.90459529	4104
chr4.fa	108745721	108836204	SGMS2	3.099909282	912
chr4.fa	108852717	108874613	CYP2U1	1.067032408	228
chr4.fa	108910870	108956331	HADH	51.54491526	4712

chr4.fa	108968701	109090112	LEF1	0	0
chr4.fa	109088681	109097586	LOC641518	0	0
chr4.fa	109459346	109541613	LOC285456	0	0
chr4.fa	109541722	109551639	RPL34	756.7436982	34504
chr4.fa	109571741	109588980	OSTC	12.51972831	608
chr4.fa	109663202	109684235	AGXT2L1	3.157730963	304
chr4.fa	109734972	110223799	COL25A1	0	0
chr4.fa	110354971	110461615	SEC24B	21.09979862	4408
chr4.fa	110481355	110608872	CCDC109B	0	0
chr4.fa	110609785	110624629	CASP6	20.35123024	1520
chr4.fa	110631145	110651242	PLA2G12A	9.362886914	2204
chr4.fa	110661848	110723335	CFI	1.162882964	113
chr4.fa	110736666	110745893	GAR1	15.0907916	684
chr4.fa	110749150	110765861	RRH	0	0
chr4.fa	110772486	110793471	LRIT3	0	0
chr4.fa	110834040	110934118	EGF	4.238329227	1064
chr4.fa	110970229	111119820	ELOVL6	6.477585025	988
chr4.fa	111397229	111484493	ENPEP	1.691951347	380
chr4.fa	111538580	111558508	PITX2	0.548861189	76
chr4.fa	113066553	113110237	C4orf32	7.440316016	912
chr4.fa	113152895	113191211	AP1AR	8.137067274	1064
chr4.fa	113196782	113207059	TIFA	11.00013005	1520
chr4.fa	113218499	113363764	ALPK1	3.435275033	836
chr4.fa	113434672	113437328	NEUROG2	0	0
chr4.fa	113460489	113558119	C4orf21	0.50393819	152
chr4.fa	113558152	113578742	LARP7	17.59135692	1900
chr4.fa	113569030	113569097	MIR367	0	0
chr4.fa	113569160	113569227	MIR302D	0	0
chr4.fa	113569339	113569407	MIR302A	0	0
chr4.fa	113569519	113569586	MIR302C	0	0
chr4.fa	113569641	113569713	MIR302B	0	0
chr4.fa	113739239	114304896	ANK2	3.375451832	2204
chr4.fa	114028019	114028111	MIR1243	0	0
chr4.fa	114372188	114683083	CAMK2D	18.50582905	5071
chr4.fa	114821440	114900878	ARSJ	3.974351014	836
chr4.fa	115519611	115598202	UGT8	1.483570903	228
chr4.fa	115577915	115578010	MIR577	0	0
chr4.fa	115748927	116035032	NDST4	0	0
chr4.fa	117220881	117220924	MIR1973	0	0
chr4.fa	118004710	118006736	TRAM1L1	0	0
chr4.fa	118955500	119179789	NDST3	0.284215802	76
chr4.fa	119199917	119200978	SNHG8	303.0122961	8434
chr4.fa	119200345	119200475	SNORA24	0	0

chr4.fa	119201193	119273922	PRSS12	0	0
chr4.fa	119437495	119475359	CEP170P1	0	0
chr4.fa	119606574	119632077	METTL14	8.823810933	836
chr4.fa	119643978	119757326	SEC24D	14.14362799	2584
chr4.fa	119809996	119982402	SYNPO2	0.38873961	152
chr4.fa	120056939	120108938	MYOZ2	0	0
chr4.fa	120133782	120216673	USP53	9.738727841	2888
chr4.fa	120217574	120225600	C4orf3	41.69832774	5700
chr4.fa	120238405	120243316	FABP2	0	0
chr4.fa	120326678	120331815	FLJ14186	0.350710735	76
chr4.fa	120375938	120420747	LOC645513	0.720769494	28
chr4.fa	120415550	120549981	PDE5A	0.480587127	152
chr4.fa	120980579	120988013	MAD2L1	0	0
chr4.fa	121615929	121844013	PRDM5	3.407698539	380
chr4.fa	121956782	121993673	C4orf31	0	0
chr4.fa	122052564	122137655	TNIP3	0	0
chr4.fa	122249797	122302181	QRFPR	0	0
chr4.fa	122589152	122618147	ANXA5	134.380478	9662
chr4.fa	122680085	122685027	TMEM155	0	0
chr4.fa	122685740	122687963	LOC100192379	0	0
chr4.fa	122722472	122737598	EXOSC9	4.863915339	304
chr4.fa	122737603	122745088	CCNA2	0	0
chr4.fa	122745635	122791642	BBS7	3.281825187	608
chr4.fa	122800183	122872909	TRPC3	0	0
chr4.fa	123091758	123283914	KIAA1109	6.077058688	4256
chr4.fa	123300121	123350947	ADAD1	0	0
chr4.fa	123372626	123377650	IL2	0	0
chr4.fa	123533783	123542211	IL21	0	0
chr4.fa	123651344	123653613	CETN4P	0	0
chr4.fa	123653857	123666098	BBS12	3.447284151	532
chr4.fa	123747863	123819390	FGF2	0	0
chr4.fa	123833723	123844123	NUDT6	7.264404671	228
chr4.fa	123844225	124240604	SPATA5	2.699382945	988
chr4.fa	124317956	124324907	SPRY1	1.939027838	228
chr4.fa	124573940	124851518	LOC285419	0	0
chr4.fa	125585204	125633887	ANKRD50	7.685168596	3040
chr4.fa	126237567	126414087	FAT4	0.209937181	152
chr4.fa	126428414	126428462	MIR2054	0	0
chr4.fa	128554087	128637934	INTU	2.064234017	304
chr4.fa	128651555	128695447	SLC25A31	0	0
chr4.fa	128703453	128754526	HSPA4L	3.618080502	532
chr4.fa	128802016	128820377	PLK4	0.846198064	152
chr4.fa	128838960	128887139	MFSD8	1.115736055	228

chr4.fa	128886461	128952455	C4orf29	7.728312466	760
chr4.fa	128982503	129132289	LARP1B	9.144276481	2280
chr4.fa	129190392	129209984	PGRMC2	9.365110825	1596
chr4.fa	129730779	129796379	PHF17	7.026890996	2584
chr4.fa	129805152	130014764	SCLT1	4.982449785	684
chr4.fa	130014829	130033843	C4orf33	3.477751729	304
chr4.fa	134070470	134112732	PCDH10	0	0
chr4.fa	135117489	135122903	PABPC4L	0.342037483	76
chr4.fa	138440074	138453629	PCDH18	0.286217322	76
chr4.fa	139085248	139163503	SLC7A11	0.175244172	76
chr4.fa	139936943	139967095	CCRN4L	0.863989351	76
chr4.fa	139978871	140060606	ELF2	11.68865284	1900
chr4.fa	140187317	140201492	C4orf49	0	0
chr4.fa	140211071	140223705	NDUFC1	45.36600146	2964
chr4.fa	140222676	140311935	NAA15	15.21577539	3800
chr4.fa	140374961	140397069	RAB33B	3.495765407	608
chr4.fa	140427192	140477577	SETD7	31.33512575	9880
chr4.fa	140586922	140625406	MGST2	40.14159017	1444
chr4.fa	140637546	141075233	MAML3	11.92283065	3800
chr4.fa	141178440	141303710	SCOC	21.5766051	2280
chr4.fa	141204880	141294546	LOC100129858	0	0
chr4.fa	141309607	141348815	CLGN	1.197798364	152
chr4.fa	141445312	141474924	ELMOD2	0	0
chr4.fa	141481050	141489959	UCP1	0	0
chr4.fa	141541936	141677471	TBC1D9	47.41311136	11696
chr4.fa	141786725	142054616	RNF150	0.725217316	152
chr4.fa	142142049	142155850	ZNF330	14.30841978	1216
chr4.fa	142557754	142654612	IL15	0.868881955	76
chr4.fa	142949182	143767604	INPP4B	36.02046105	6678
chr4.fa	144106070	144143141	USP38	10.73637423	2508
chr4.fa	144257983	144395718	GAB1	4.273467018	1520
chr4.fa	144434616	144474567	SMARCA5	38.6224367	6688
chr4.fa	144480625	144482613	LOC441046	0	0
chr4.fa	144498561	144621828	FREM3	0	0
chr4.fa	144792019	144826716	GYPE	0	0
chr4.fa	144917257	144940496	GYPB	0	0
chr4.fa	145030456	145061904	GYPA	0	0
chr4.fa	145567173	145659881	HHIP	0	0
chr4.fa	145916313	146017263	ANAPC10	4.322615447	152
chr4.fa	146019156	146050676	ABCE1	12.3429274	2280
chr4.fa	146054802	146100832	OTUD4	10.18929217	3406
chr4.fa	146402951	146480325	SMAD1	8.675920864	1216
chr4.fa	146540540	146581187	MMAA	0.568876386	152

chr4.fa	146601356	146653948	C4orf51	0	0
chr4.fa	146681888	146859607	ZNF827	52.11801708	9803
chr4.fa	147096835	147111213	LSM6	25.77134567	912
chr4.fa	147175137	147443123	SLC10A7	0.690079525	152
chr4.fa	147560045	147563623	POU4F2	0	0
chr4.fa	147628179	147867034	TTC29	0	0
chr4.fa	148402069	148466106	EDNRA	0	0
chr4.fa	148538539	148556672	TMEM184C	10.23310321	1368
chr4.fa	148559534	148605280	PRMT10	6.571878843	836
chr4.fa	148653453	148993927	ARHGAP10	14.93289394	2052
chr4.fa	148999915	149363672	NR3C2	8.310532317	2204
chr4.fa	150999426	151178606	DCLK2	4.613725373	912
chr4.fa	151185596	151936649	LRBA	39.6147457	18164
chr4.fa	151503077	151505845	MAB21L2	0	0
chr4.fa	152020754	152025804	RPS3A	125.1287867	5092
chr4.fa	152024979	152025043	SNORD73A	0	0
chr4.fa	152041433	152147660	SH3D19	8.880075876	2052
chr4.fa	152198325	152212605	PRSS48	0	0
chr4.fa	152330398	152584784	FAM160A1	9.972683259	2052
chr4.fa	152591809	152682175	PET112L	2.271724895	228
chr4.fa	153242410	153456172	FBXW7	17.58735388	3724
chr4.fa	153410479	153410568	MIR3140	0	0
chr4.fa	153457416	153460415	DKFZP434I0714	1.126855609	152
chr4.fa	153547266	153601317	TMEM154	8.408829175	1216
chr4.fa	153690506	153700916	TIGD4	0	0
chr4.fa	153701112	153833063	ARFIP1	26.0924784	3467
chr4.fa	153864135	153900848	FHDC1	11.47649175	3344
chr4.fa	154074270	154260474	TRIM2	29.59624988	9196
chr4.fa	154228621	154229963	ANXA2P1	0	0
chr4.fa	154265801	154336243	MND1	0	0
chr4.fa	154387498	154557862	KIAA0922	0	0
chr4.fa	154605441	154627242	TLR2	0	0
chr4.fa	154631312	154681387	RNF175	0	0
chr4.fa	154701742	154710228	SFRP2	0	0
chr4.fa	155155527	155412877	DCHS2	0	0
chr4.fa	155457662	155471523	PLRG1	26.54215316	2128
chr4.fa	155484132	155493915	FGB	0	0
chr4.fa	155504280	155511897	FGA	0	0
chr4.fa	155525286	155533902	FGG	0	0
chr4.fa	155665163	155674270	LRAT	0	0
chr4.fa	155702427	155749965	RBM46	0	0
chr4.fa	156129781	156138228	NPY2R	0	0
chr4.fa	156263812	156298122	MAP9	3.227784154	1064

chr4.fa	156587862	156658214	GUCY1A3		17.09453525	7636
chr4.fa	156680126	156728783	GUCY1B3		2.601086087	380
chr4.fa	156750881	156787425	ACCN5		0	0
chr4.fa	156824847	156841550	TDO2		0	0
chr4.fa	156845270	156875048	CTSO		1.152430583	152
chr4.fa	157682763	157892546	PDGFC		2.115828747	292
chr4.fa	157997277	158093242	GLRB		0	0
chr4.fa	158141736	158287226	GRIA2		0	0
chr4.fa	158493642	158497303	LOC340017		0	0
chr4.fa	159045732	159094202	FAM198B		0	0
chr4.fa	159131401	159176439	TMEM144		1.018773543	152
chr4.fa	159443047	159573263	RXFP1		0	0
chr4.fa	159587831	159593202	C4orf46		1.917900685	304
chr4.fa	159593277	159629841	ETFDH		17.27600637	1824
chr4.fa	159630279	159644552	PPID		16.58837315	1368
chr4.fa	159690182	159827954	FNIP2		9.931985691	2584
chr4.fa	159814684	159956333	C4orf45		0	0
chr4.fa	160049954	160050046	MIR3688		0	0
chr4.fa	160188998	160281301	RAPGEF2		11.89569894	3648
chr4.fa	162305049	163085186	FSTL5		0	0
chr4.fa	164047860	164088073	NAF1		4.384440168	456
chr4.fa	164245117	164253748	NPY1R		0.612687429	76
chr4.fa	164265091	164273086	NPY5R		0	0
chr4.fa	164392247	164395047	TKTL2		0	0
chr4.fa	164415673	164441691	C4orf43		5.287347958	456
chr4.fa	164445450	165304407		1-Mar	0	0
chr4.fa	165118159	165118863	ANP32C		0	0
chr4.fa	165875598	165898818	TRIM61		0	0
chr4.fa	165878100	165880273	C4orf39		1.55495844	152
chr4.fa	165953151	165962896	TRIM60		0	0
chr4.fa	165997230	166034024	TMEM192		6.768250168	1368
chr4.fa	166128770	166244308	KLHL2		6.660168103	988
chr4.fa	166198944	166201175	GK3P		0	0
chr4.fa	166248818	166264225	SC4MOL		13.43286609	1292
chr4.fa	166300097	166419482	CPE		13.92234886	1520
chr4.fa	166307394	166307489	MIR578		0	0
chr4.fa	166794410	167024993	TLL1		0.50794123	152
chr4.fa	167654536	168155741	SPOCK3		1.697066341	228
chr4.fa	169013707	169108893	ANXA10		0	0
chr4.fa	169137442	169239958	DDX60		3.896291744	1064
chr4.fa	169277886	169401638	DDX60L		2.25215448	684
chr4.fa	169418217	169849608	PALLD		4.356641283	1368
chr4.fa	169908742	169931468	CBR4		6.276098705	988

chr4.fa	170015407	170192249	SH3RF1	28.57080461	6764
chr4.fa	170314421	170533778	NEK1	7.738097674	1976
chr4.fa	170541722	170642157	CLCN3	12.51661484	2280
chr4.fa	170650619	170679093	C4orf27	6.949721291	380
chr4.fa	170907748	170947429	MFAP3L	8.312756228	2356
chr4.fa	170981373	171011372	AADAT	8.819585502	836
chr4.fa	171502621	171526135	HSP90AA6P	0	0
chr4.fa	172734575	173961558	GALNTL6	1.186456419	152
chr4.fa	174089904	174245118	GALNT7	7.063585524	1368
chr4.fa	174252527	174255595	HMGB2	9.794992785	684
chr4.fa	174292093	174298683	SAP30	1.52404608	76
chr4.fa	174309299	174320617	SCRG1	0	0
chr4.fa	174447652	174451378	HAND2	0	0
chr4.fa	174451609	174462981	NBLA00301	0	0
chr4.fa	174537087	174537794	MORF4	0	0
chr4.fa	175157810	175205402	FBXO8	10.08832662	988
chr4.fa	175205055	175254531	KIAA1712	3.364332278	939
chr4.fa	175344946	175345015	MIR4276	0	0
chr4.fa	175411328	175444044	HPGD	134.7887881	18316
chr4.fa	175563198	175750465	GLRA3	0	0
chr4.fa	175839509	175899331	ADAM29	0	0
chr4.fa	176554088	176923648	GPM6A	1.12085105	152
chr4.fa	176986985	177103979	WDR17	0.449452375	152
chr4.fa	177105725	177116822	SPATA4	1.370818625	76
chr4.fa	177134826	177190373	ASB5	0	0
chr4.fa	177241090	177253396	SPCS3	5.884023229	1216
chr4.fa	177604691	177713895	VEGFC	0.813061793	76
chr4.fa	178230991	178284092	NEIL3	0.703645381	76
chr4.fa	178351929	178363657	AGA	0	0
chr4.fa	178649911	178911904	LOC285501	0	0
chr4.fa	181985243	182080302	NCRNA00290	0	0
chr4.fa	183060159	183065668	MGC45800	0	0
chr4.fa	183090446	183090531	MIR1305	0	0
chr4.fa	183245137	183724177	ODZ3	0	0
chr4.fa	183811244	183838630	DCTD	28.96955181	2660
chr4.fa	183958818	183961272	FAM92A3	0	0
chr4.fa	184018174	184020352	C4orf38	0	0
chr4.fa	184020463	184241929	WWC2	11.91437979	3268
chr4.fa	184242917	184243579	CLDN24	0	0
chr4.fa	184365789	184369049	CDKN2AIP	9.255472022	988
chr4.fa	184415890	184425668	LOC389247	0	0
chr4.fa	184426220	184432249	ING2	12.11586611	608
chr4.fa	184560789	184580331	RWDD4	2.607313037	304

chr4.fa	184580446	184634745	C4orf41	14.57373234	2888
chr4.fa	184826509	184938875	STOX2	6.536963444	1368
chr4.fa	185009859	185139114	ENPP6	1.717748712	304
chr4.fa	185308876	185395726	IRF2	22.64764055	2328
chr4.fa	185548850	185570629	CASP3	8.852276991	1064
chr4.fa	185570767	185612866	CCDC111	6.367723831	532
chr4.fa	185615219	185655286	MLF1IP	0.799940719	76
chr4.fa	185676749	185747215	ACSL1	82.95387484	14193
chr4.fa	185719451	185720200	SLED1	0	0
chr4.fa	185772167	185772264	MIR3945	0	0
chr4.fa	185940083	185941926	HELT	1.717748712	76
chr4.fa	186064417	186071538	SLC25A4	17.97231284	3572
chr4.fa	186080819	186125182	KIAA1430	9.353324097	2052
chr4.fa	186131284	186285031	SNX25	10.27958295	1444
chr4.fa	186285121	186300152	LRP2BP	1.000315084	228
chr4.fa	186317840	186320182	ANKRD37	20.75642679	476
chr4.fa	186320694	186347139	UFSP2	16.42625005	1444
chr4.fa	186350545	186370821	C4orf47	0	0
chr4.fa	186366338	186392913	CCDC110	0.583554197	76
chr4.fa	186421815	186456712	PDLIM3	0	0
chr4.fa	186506598	186877870	SORBS2	9.949109804	3927
chr4.fa	186990309	187006252	TLR3	1.111510624	152
chr4.fa	187065995	187093817	FAM149A	21.25213651	2584
chr4.fa	187112674	187134617	CYP4V2	2.155859142	456
chr4.fa	187148672	187179625	KLKB1	0.752126637	76
chr4.fa	187187118	187210835	F11	1.239163105	152
chr4.fa	187207252	187422212	LOC285441	4.499193966	456
chr4.fa	187454809	187476537	MTNR1A	1.529605857	76
chr4.fa	187508937	187644987	FAT1	18.88900888	12540
chr4.fa	188916925	188926199	ZFP42	0	0
chr4.fa	189012427	189026408	TRIML2	0	0
chr4.fa	189060598	189068649	TRIML1	0	0
chr4.fa	189376732	189523062	LOC401164	0	0
chr4.fa	190394299	190396344	HSP90AA4P	0	0
chr4.fa	190861974	190884359	FRG1	8.102374265	380
chr4.fa	190903678	190906024	TUBB4Q	0	0
chr4.fa	190988867	191013476	DUX2	0	0
chr4.fa	191001979	191003583	DUX4L4	0	0
chr4.fa	191005267	191011865	DUX4	0	0
chr5.fa	140373	190087	PLEKHG4B	0.293556227	152
chr5.fa	191626	195468	LRRC14B	0	0
chr5.fa	204875	218297	CCDC127	4.760948268	304
chr5.fa	218356	256814	SDHA	24.76191256	2660

chr5.fa	271736	315089	PDCD6	50.84393857	2492
chr5.fa	304291	438405	AHRR	0.644489354	164
chr5.fa	403812	404662	LOC100310782	0	0
chr5.fa	441643	443258	C5orf55	3.137715766	228
chr5.fa	443334	467409	EXOC3	21.41781787	2660
chr5.fa	470625	473080	LOC25845	11.63639094	912
chr5.fa	473334	524549	SLC9A3	2.434515167	304
chr5.fa	612405	653666	CEP72	1.400396639	152
chr5.fa	659977	693510	TPPP	0.841972634	228
chr5.fa	795720	851101	ZDHHC11	0	0
chr5.fa	863850	892939	BRD9	12.71676681	1672
chr5.fa	892969	918164	TRIP13	0	0
chr5.fa	1009168	1038925	NKD2	0.940047101	76
chr5.fa	1050489	1112172	SLC12A7	6.373283608	1520
chr5.fa	1201710	1225230	SLC6A19	2.614429552	608
chr5.fa	1225470	1246304	SLC6A18	0	0
chr5.fa	1253287	1295162	TERT	0	0
chr5.fa	1318000	1345002	CLPTM1L	25.37771346	2450
chr5.fa	1392905	1445543	SLC6A3	0	0
chr5.fa	1461542	1524076	LPCAT1	2.141626113	380
chr5.fa	1572073	1594646	SDHAP3	1.467113963	76
chr5.fa	1597672	1634120	LOC728613	1.312329771	152
chr5.fa	1708900	1708983	MIR4277	0	0
chr5.fa	1798499	1799956	MRPL36	60.55998252	1672
chr5.fa	1801496	1816167	NDUFS6	125.4399118	3091
chr5.fa	1877541	1882880	IRX4	1.52204456	152
chr5.fa	2746279	2751769	IRX2	2.201671705	228
chr5.fa	2752262	2755511	C5orf38	5.697214721	228
chr5.fa	3417266	3536208	LOC285577	0	0
chr5.fa	3596168	3601517	IRX1	3.638762872	304
chr5.fa	5034472	5070115	LOC340094	0.966289248	76
chr5.fa	5140443	5320412	ADAMTS16	1.019440717	228
chr5.fa	5422807	5490338	KIAA0947	14.66402312	5221
chr5.fa	6310554	6337405	FLJ33360	0	0
chr5.fa	6372039	6378639	MED10	13.15309811	608
chr5.fa	6448736	6496834	UBE2QL1	0	0
chr5.fa	6582287	6588613	LOC255167	1.02210941	152
chr5.fa	6599352	6633473	NSUN2	25.04212532	3797
chr5.fa	6633500	6669675	SRD5A1	2.23970058	228
chr5.fa	6714718	6757161	PAPD7	4.549009568	836
chr5.fa	6827966	6828034	MIR4278	0	0
chr5.fa	7299487	7306827	LOC442132	0	0
chr5.fa	7396343	7830194	ADCY2	2.321318107	684

chr5.fa	7831511	7851264	C5orf49	14.82614622	684
chr5.fa	7859272	7869150	FASTKD3	5.548435087	608
chr5.fa	7869217	7901235	MTRR	10.84779216	1596
chr5.fa	9035138	9546233	SEMA5A	2.719620533	1444
chr5.fa	9548939	9549026	SNORD123	0	0
chr5.fa	9629109	9630463	TAS2R1	0	0
chr5.fa	9641427	9903936	LOC285692	0	0
chr5.fa	10226438	10250014	FAM173B	10.0714249	836
chr5.fa	10250282	10266501	CCT5	37.67505069	5765
chr5.fa	10277707	10308168	CMBL	17.54065176	3192
chr5.fa	10353828	10435491	6-Mar	7.991845898	1657
chr5.fa	10442009	10465138	ROPN1L	0	0
chr5.fa	10564435	10657928	ANKRD33B	0.362275071	152
chr5.fa	10679342	10761387	DAP	136.7202546	14398
chr5.fa	10971952	11904110	CTNND2	4.663763366	1140
chr5.fa	12574969	12805295	TAG	0	0
chr5.fa	13690437	13944589	DNAH5	1.519375867	1064
chr5.fa	14143829	14509458	TRIO	19.48835285	8962
chr5.fa	14581891	14616287	FAM105A	0.239737586	76
chr5.fa	14664783	14699842	FAM105B	5.137233978	1824
chr5.fa	14704909	14871887	ANKH	19.1527647	7068
chr5.fa	15500305	15939900	FBXL7	2.223021249	456
chr5.fa	16067474	16179897	11-Mar	0	0
chr5.fa	16451628	16465894	ZNF622	12.98586002	988
chr5.fa	16473147	16617118	FAM134B	1.416408797	228
chr5.fa	16662016	16936385	MYO10	10.18951456	5237
chr5.fa	17130137	17217531	LOC285696	0.509720358	76
chr5.fa	17217750	17276943	BASP1	248.3058692	20064
chr5.fa	17379015	17387419	LOC401177	0	0
chr5.fa	19473155	19988353	CDH18	0	0
chr5.fa	21459589	21589481	GUSBP1	0.556422485	152
chr5.fa	21750973	22853731	CDH12	0	0
chr5.fa	22142461	22152379	PMCHL1	0	0
chr5.fa	23507724	23528706	PRDM9	0	0
chr5.fa	24487209	24645085	CDH10	0.46679888	76
chr5.fa	26880709	27038689	CDH9	0.549750753	76
chr5.fa	28926977	28927420	LOC729862	0	0
chr5.fa	31193796	31325237	CDH6	0.373839408	76
chr5.fa	31400602	31532282	DROSHA	12.87733317	3183
chr5.fa	31532373	31555165	C5orf22	7.954928978	1292
chr5.fa	31799031	32111038	PDZD2	3.339202086	1748
chr5.fa	31936208	31936265	MIR4279	0	0
chr5.fa	32124824	32174425	GOLPH3	96.63537417	11628

chr5.fa	32227111	32313114	MTMR12	15.64076475	3648
chr5.fa	32354456	32444844	ZFR	30.82807408	6536
chr5.fa	32585605	32604185	SUB1	22.16927733	3496
chr5.fa	32711665	32787254	NPR3	5.113882914	608
chr5.fa	32788945	32791819	C5orf23	7.054689881	912
chr5.fa	32947549	32962573	LOC340113	0	0
chr5.fa	33440898	33468196	TARS	32.02431571	4104
chr5.fa	33527287	33892124	ADAMTS12	0	0
chr5.fa	33936491	33939023	RXFP3	0	0
chr5.fa	33944721	33984780	SLC45A2	0	0
chr5.fa	33987091	34008220	AMACR	10.8517952	1520
chr5.fa	34017963	34043317	C1QTNF3	1.792249724	304
chr5.fa	34656433	34832717	RAI14	10.65631344	2806
chr5.fa	34839269	34899564	TTC23L	0	0
chr5.fa	34905366	34915731	RAD1	5.83687632	1216
chr5.fa	34915820	34925787	BRIX1	18.51806056	1060
chr5.fa	34929698	34959069	DNAJC21	10.34585549	2888
chr5.fa	34998206	35048240	AGXT2	11.94217867	1292
chr5.fa	35055802	35230794	PRLR	0.573546599	304
chr5.fa	35617989	35814713	SPEF2	0.899349533	228
chr5.fa	35856991	35876923	IL7R	0	0
chr5.fa	35904398	35938881	CAPSL	0	0
chr5.fa	35953191	36001130	UGT3A1	0.970292288	228
chr5.fa	36035119	36067023	UGT3A2	0	0
chr5.fa	36103414	36152015	LMBRD2	4.17717168	608
chr5.fa	36147994	36148090	MIR580	0	0
chr5.fa	36152189	36184147	SKP2	3.558257301	288
chr5.fa	36192694	36242258	C5orf33	11.14779773	1976
chr5.fa	36249104	36302011	RANBP3L	9.038418326	1064
chr5.fa	36606457	36688436	SLC1A3	2.026649924	380
chr5.fa	36876861	37065921	NIPBL	21.98380317	9728
chr5.fa	37106330	37249530	C5orf42	4.37665648	2204
chr5.fa	37291941	37371197	NUP155	9.44228053	1900
chr5.fa	37379412	37752774	WDR70	9.557923892	912
chr5.fa	37812779	37839782	GDNF	0	0
chr5.fa	38258533	38465581	EGFLAM	0	0
chr5.fa	38475065	38595507	LIFR	9.260809407	4332
chr5.fa	38557604	38557663	MIR3650	0	0
chr5.fa	38845960	38935743	OSMR	0.824848521	228
chr5.fa	38938022	39074501	RICTOR	6.007005497	2575
chr5.fa	39105357	39219667	FYB	0	0
chr5.fa	39284378	39364655	C9	0	0
chr5.fa	39371780	39425335	DAB2	59.03971709	12204

chr5.fa	40680032	40693837	PTGER4	0.989640312	152
chr5.fa	40711678	40756072	TTC33	2.762764403	684
chr5.fa	40759481	40798297	PRKAA1	20.09748202	4636
chr5.fa	40825365	40829244	LOC100506548	8.276506482	1444
chr5.fa	40831430	40835387	RPL37	569.4910749	40204
chr5.fa	40832758	40832837	SNORD72	0	0
chr5.fa	40841410	40855456	CARD6	4.95042547	912
chr5.fa	40909599	40983042	C7	0	0
chr5.fa	40998122	41071444	HEATR7B2	0	0
chr5.fa	41142336	41261540	C6	0	0
chr5.fa	41307048	41510730	PLCXD3	0	0
chr5.fa	41730167	41870791	OXCT1	3.325191448	532
chr5.fa	41904470	41921738	C5orf51	7.444319055	1748
chr5.fa	41925356	41941672	FBXO4	5.600252209	380
chr5.fa	42424026	42721926	GHR	3.876498938	760
chr5.fa	42756920	42802539	CCDC152	0.82885156	76
chr5.fa	42804758	42812024	SEPP1	46.01805211	1554
chr5.fa	43014831	43018913	LOC648987	1.591430578	228
chr5.fa	43039182	43040447	C5orf39	0	0
chr5.fa	43042236	43045370	LOC153684	3.739283641	380
chr5.fa	43065289	43067073	LOC100132356	0.946941224	76
chr5.fa	43121642	43175823	ZNF131	11.2583261	1216
chr5.fa	43192327	43280952	MGC42105	0.730777093	76
chr5.fa	43289493	43313595	HMGCS1	85.21181149	13449
chr5.fa	43381600	43412488	CCL28	2.071350531	76
chr5.fa	43444354	43483992	C5orf28	1.224262903	152
chr5.fa	43486803	43515273	C5orf34	0	0
chr5.fa	43526370	43557521	PAIP1	9.406253175	1216
chr5.fa	43602791	43705668	NNT	10.11456877	2204
chr5.fa	44305097	44388784	FGF10	0	0
chr5.fa	44809027	44815618	MRPS30	16.17383617	1216
chr5.fa	45259352	45696220	HCN1	0	0
chr5.fa	49692031	49737234	EMB	1.176671211	228
chr5.fa	49961733	50142356	PARP8	5.511295777	1821
chr5.fa	50678958	50690563	ISL1	0.622027854	76
chr5.fa	52083774	52098452	PELO	9.028633119	1140
chr5.fa	52145199	52249485	ITGA1	0	0
chr5.fa	52285156	52390609	ITGA2	1.073926532	380
chr5.fa	52393895	52405598	MOCS2	28.97444442	2052
chr5.fa	52405687	52410952	LOC257396	3.594507047	380
chr5.fa	52776595	52781904	FST	0	0
chr5.fa	52856465	52979171	NDUFS4	35.00346663	1064
chr5.fa	53180614	53606403	ARL15	8.713060174	974

chr5.fa	53247334	53247429	MIR581	0	0
chr5.fa	53751431	53752213	HSPB3	0	0
chr5.fa	53813589	53842416	SNX18	8.802906171	2128
chr5.fa	54273695	54281414	ESM1	0	0
chr5.fa	54320107	54329960	GZMK	0	0
chr5.fa	54398474	54406080	GZMA	0	0
chr5.fa	54408799	54469005	CDC20B	0	0
chr5.fa	54455984	54463129	GPX8	0.450786722	76
chr5.fa	54466360	54466450	MIR449A	0	0
chr5.fa	54466474	54466570	MIR449B	0	0
chr5.fa	54468090	54468181	MIR449C	0	0
chr5.fa	54515425	54523143	LOC345643	0.803276586	76
chr5.fa	54526981	54529508	CCNO	16.52410212	1064
chr5.fa	54552073	54603521	DHX29	24.18302857	4864
chr5.fa	54603576	54721409	SKIV2L2	30.30100722	5074
chr5.fa	54721691	54830873	PPAP2A	44.63255567	2603
chr5.fa	54824670	54830370	RNF138P1	0	0
chr5.fa	54921676	55008163	SLC38A9	4.003484246	456
chr5.fa	55033845	55112974	DDX4	0	0
chr5.fa	55147334	55212981	IL31RA	0	0
chr5.fa	55230925	55290821	IL6ST	5.786393544	2356
chr5.fa	55395507	55529186	ANKRD55	0	0
chr5.fa	56110900	56191978	MAP3K1	10.1261331	3420
chr5.fa	56205087	56221359	C5orf35	0	0
chr5.fa	56215429	56247954	MIER3	7.149428482	1672
chr5.fa	56469775	56560506	GPBP1	25.47623271	5320
chr5.fa	56775843	56778636	ACTBL2	0	0
chr5.fa	57749810	57755913	PLK2	26.09603665	3268
chr5.fa	57787330	57792185	GAPT	0	0
chr5.fa	57878939	58147406	RAB3C	0	0
chr5.fa	58264866	59481514	PDE4D	6.483367193	3192
chr5.fa	59783540	59843484	PART1	10.67432712	4484
chr5.fa	59892739	59995993	DEPDC1B	0.674956932	76
chr5.fa	60047616	60140101	ELOVL7	11.75848364	2052
chr5.fa	60169659	60240905	ERCC8	0.82685004	76
chr5.fa	60240956	60448864	NDUFAF2	16.45516089	532
chr5.fa	60453536	60458302	C5orf43	22.22376315	2888
chr5.fa	60628100	60841999	ZSWIM6	7.678496864	1900
chr5.fa	60933636	61002362	FLJ37543	0	0
chr5.fa	61601989	61682210	KIF2A	12.0462577	1820
chr5.fa	61684351	61699728	DIMT1L	7.598658466	532
chr5.fa	61708573	61924416	IPO11	5.83687632	1216
chr5.fa	61874562	61877275	LRRC70	0	0

chr5.fa	63256278	63257546	HTR1A	0	0
chr5.fa	63461671	63668696	RNF180	3.07678061	684
chr5.fa	63802452	63908121	RGS7BP	3.100354064	532
chr5.fa	63986135	64013977	FAM159B	0	0
chr5.fa	64014018	64064496	SREK1IP1	2.232806456	684
chr5.fa	64064755	64314590	CWC27	15.02207276	1405
chr5.fa	64444563	64777704	ADAMTS6	0	0
chr5.fa	64813593	64858995	CENPK	0	0
chr5.fa	64859131	64883370	PPWD1	8.067681256	760
chr5.fa	64885507	64920187	TRIM23	12.59756519	1368
chr5.fa	64920558	64961754	C5orf44	10.20396998	1900
chr5.fa	64961955	65017941	SGTB	1.286977187	304
chr5.fa	65018023	65125111	NLN	5.268889498	2052
chr5.fa	65222384	65376850	ERBB2IP	30.3038983	9424
chr5.fa	65240633	65241401	LOC100303749	0	0
chr5.fa	65440085	65476714	SREK1	12.43321818	2508
chr5.fa	65892176	66465423	MAST4	39.52534449	19456
chr5.fa	66478104	66492617	CD180	0	0
chr5.fa	67522462	67597649	PIK3R1	15.35855047	4788
chr5.fa	68389818	68425880	SLC30A5	6.409088572	1064
chr5.fa	68462913	68474070	CCNB1	2.42495235	228
chr5.fa	68485375	68506184	CENPH	0	0
chr5.fa	68513573	68525985	MRPS36	16.53299777	988
chr5.fa	68530622	68573257	CDK7	11.14157078	760
chr5.fa	68576519	68616410	CCDC125	17.76170849	2888
chr5.fa	68647553	68665840	TAF9	20.72662639	2042
chr5.fa	68665124	68710630	RAD17	7.870420367	1216
chr5.fa	68710939	68737890	MARVELD2	6.259864156	608
chr5.fa	68788119	68850133	OCLN	10.2435556	1216
chr5.fa	68856051	68888729	GTF2H2C	0	0
chr5.fa	68858500	68858597	GTF2H2D	9.758075865	43
chr5.fa	68926980	68929028	LOC100272216	0	0
chr5.fa	68935290	69006272	GUSBP3	0	0
chr5.fa	69321072	70203944	SERF1A	0	0
chr5.fa	69423289	69521622	LOC100170939	0	0
chr5.fa	69711197	69746189	GTF2H2B	0	0
chr5.fa	69776870	69881549	SMA5	0	0
chr5.fa	69812079	70555122	LOC100049076	0	0
chr5.fa	70264310	70320941	NAIP	0	0
chr5.fa	70330951	70363497	GTF2H2	0	0
chr5.fa	70370030	70388897	LOC647859	0	0
chr5.fa	70671612	70681820	PMCHL2	0	0
chr5.fa	70751442	70863649	BDP1	13.73509557	6840

chr5.fa	70883115	70954530	MCCC2	92.0140875	15197
chr5.fa	71014990	71016875	CARTPT	18.47180321	760
chr5.fa	71403118	71505397	MAP1B	0	0
chr5.fa	71515236	71616084	MRPS27	15.19398107	1900
chr5.fa	71616200	71655180	PTCD2	4.018384448	380
chr5.fa	71739234	71803249	ZNF366	0	0
chr5.fa	72112418	72210215	TNPO1	19.95604129	7814
chr5.fa	72251808	72386349	FCHO2	8.143739006	1824
chr5.fa	72416388	72427644	TMEM171	0	0
chr5.fa	72469023	72470970	TMEM174	22.1125676	1748
chr5.fa	72742085	72744352	FOXD1	0.745232513	76
chr5.fa	72794250	72801448	BTF3	231.5691612	10496
chr5.fa	72848025	72861511	ANKRA2	6.920588059	684
chr5.fa	72861598	72877794	UTP15	4.064419402	668
chr5.fa	72921983	73237818	RGNEF	15.14238633	4325
chr5.fa	73923234	73937249	ENC1	61.5778665	15276
chr5.fa	73980969	74017030	HEXB	5.578235492	456
chr5.fa	74017114	74063042	GFM2	11.85322224	1672
chr5.fa	74063103	74072734	NSA2	76.54767736	3800
chr5.fa	74073399	74162615	FAM169A	4.551011088	1216
chr5.fa	74323289	74326724	GCNT4	11.31370148	1748
chr5.fa	74364122	74532703	ANKRD31	0	0
chr5.fa	74632993	74657926	HMGCR	16.2303235	3344
chr5.fa	74666928	74807806	COL4A3BP	18.31368315	4558
chr5.fa	74842835	74895646	POLK	4.810319089	958
chr5.fa	74970024	75013313	POC5	4.525213722	456
chr5.fa	75379305	75621416	SV2C	0	0
chr5.fa	75699149	76003957	IQGAP2	9.082229369	2356
chr5.fa	75911307	75919240	F2RL2	0	0
chr5.fa	76007763	76008686	NCRUPAR	0	0
chr5.fa	76011868	76031595	F2R	0	0
chr5.fa	76114833	76131140	F2RL1	0.58622289	76
chr5.fa	76145826	76217056	S100Z	0	0
chr5.fa	76248680	76265299	CRHBP	0	0
chr5.fa	76326210	76361058	AGGF1	12.63403733	2561
chr5.fa	76372532	76383030	ZBED3	8.337664029	532
chr5.fa	76376259	76376396	SNORA47	0	0
chr5.fa	76382623	76444176	LOC728723	0	0
chr5.fa	76506706	76724080	PDE8B	22.42080164	4440
chr5.fa	76728069	76788332	WDR41	12.43232862	1430
chr5.fa	76924537	76934522	OTP	0	0
chr5.fa	76986995	77072185	TBCA	102.0574911	3116
chr5.fa	77298150	77590528	AP3B1	20.35323176	3648

chr5.fa	77656339	77776562	SCAMP1	3.248244133	912
chr5.fa	77781038	77944648	LHFPL2	5.025371264	1140
chr5.fa	78073037	78282357	ARSB	2.02576036	608
chr5.fa	78293429	78365449	DMGDH	9.319743044	1292
chr5.fa	78365547	78385897	BHMT2	21.67712587	2584
chr5.fa	78407604	78428113	BHMT	100.7342642	11324
chr5.fa	78531925	78623038	JMY	7.405623007	3040
chr5.fa	78669786	78809700	HOMER1	11.63149833	2204
chr5.fa	78908243	78982471	PAPD4	9.859708589	1440
chr5.fa	78985659	79096049	CMYA5	14.29018371	8284
chr5.fa	79272539	79287088	MTX3	6.177801847	2204
chr5.fa	79331170	79379107	THBS4	0	0
chr5.fa	79407050	79551898	SERINC5	8.809355512	2780
chr5.fa	79594917	79596297	LOC644936	0	0
chr5.fa	79615790	79617660	SPZ1	0	0
chr5.fa	79646424	79647785	CRSP8P	0	0
chr5.fa	79703838	79775498	ZFYVE16	10.52843857	3420
chr5.fa	79783800	79838206	FAM151B	0	0
chr5.fa	79852574	79866304	ANKRD34B	0	0
chr5.fa	79922045	79950800	DHFR	1.972831282	304
chr5.fa	79945819	79946854	MTRNR2L2	0	0
chr5.fa	79952230	80172634	MSH3	11.81408141	2194
chr5.fa	80256558	80521953	RASGRF2	0.414536976	76
chr5.fa	80501462	80501507	RNU5E	0	0
chr5.fa	80529139	80562217	CKMT2	3.088122555	228
chr5.fa	80533384	80597388	LOC100131067	1.742434122	228
chr5.fa	80597402	80608965	ZCCHC9	8.256268893	532
chr5.fa	80625947	80689988	ACOT12	0	0
chr5.fa	80715672	81047072	SSBP2	3.390574425	297
chr5.fa	81267844	81551216	ATG10	2.78678264	304
chr5.fa	81569139	81574235	RPS23	253.4749051	37772
chr5.fa	81601166	81614147	ATP6AP1L	0	0
chr5.fa	82348665	82373272	TMEM167A	11.05417109	2280
chr5.fa	82360023	82360156	SCARNA18	0	0
chr5.fa	82373317	82649579	XRCC4	0	0
chr5.fa	82767493	82878122	VCAN	0	0
chr5.fa	82934017	83016896	HAPLN1	0	0
chr5.fa	83238126	83680611	EDIL3	0	0
chr5.fa	85578262	85593362	NBPF22P	0	0
chr5.fa	85913784	85916583	COX7C	635.3717641	11628
chr5.fa	85916314	85916392	MIR3607	0	0
chr5.fa	86410696	86410771	MIR4280	0	0
chr5.fa	86564151	86687733	RASA1	25.19757668	4940

chr5.fa	86690079	86708850	CCNH	4.127356078	304
chr5.fa	87491023	87564665	TMEM161B	0.651605868	76
chr5.fa	87836597	87980620	LOC645323	0.445004554	76
chr5.fa	88014058	88199922	MEF2C	0.492373854	152
chr5.fa	89312438	89312537	MIR3660	0	0
chr5.fa	89689529	89705603	CETN3	8.614540925	380
chr5.fa	89754020	89770585	MBLAC2	3.948998431	760
chr5.fa	89770681	89810369	POLR3G	3.601623562	532
chr5.fa	89811445	89825401	LYSMD3	3.173965512	608
chr5.fa	89854617	90460033	GPR98	0.34959878	304
chr5.fa	90664541	90679149	ARRDC3	27.21488618	5092
chr5.fa	90676164	90716532	LOC100129716	0	0
chr5.fa	92745065	92916953	FLJ42709	0	0
chr5.fa	92919043	92929786	NR2F1	0	0
chr5.fa	92953431	93447404	FAM172A	6.57521471	1368
chr5.fa	93076015	93077309	POU5F2	0	0
chr5.fa	93486556	93954309	C5orf36	0.204155013	76
chr5.fa	93954391	94031573	ANKRD32	0.443225425	76
chr5.fa	94042289	94620279	MCTP1	1.952593694	380
chr5.fa	94727048	94786144	FAM81B	0	0
chr5.fa	94799599	94890709	TTC37	21.31907623	5468
chr5.fa	94890825	94940806	ARSK	0	0
chr5.fa	94955980	94957284	GPR150	1.295205657	76
chr5.fa	94982481	94992849	RFESD	0	0
chr5.fa	94994020	95018714	SPATA9	0	0
chr5.fa	95066850	95132071	RHOBTB3	25.20380363	6308
chr5.fa	95149553	95158577	GLRX	60.54663906	4484
chr5.fa	95187936	95195836	C5orf27	0	0
chr5.fa	95220802	95297775	ELL2	33.4358319	9090
chr5.fa	95414842	95414916	MIR583	0	0
chr5.fa	95726040	95768985	PCSK1	0	0
chr5.fa	95997741	96110187	CAST	47.50518127	10311
chr5.fa	96096514	96149848	ERAP1	2.316870285	760
chr5.fa	96211644	96255406	ERAP2	0	0
chr5.fa	96271346	96365115	LNPEP	3.524898639	760
chr5.fa	96427574	96478520	LIX1	0	0
chr5.fa	96496571	96519005	RIOK2	5.051835803	1064
chr5.fa	98104999	98132198	RGMB	3.759298838	760
chr5.fa	98105330	98108788	FLJ35946	0	0
chr5.fa	98190908	98262238	CHD1	13.05346691	3790
chr5.fa	98264838	98266713	LOC100289230	0	0
chr5.fa	99715209	99723958	LOC100133050	0	0
chr5.fa	99871124	99922440	FAM174A	1.367482759	76

chr5.fa	100142639	100238970	ST8SIA4	0	0
chr5.fa	101569692	101632253	SLCO4C1	1.901888527	456
chr5.fa	101707652	101834720	SLCO6A1	0	0
chr5.fa	102201527	102366808	PAM	1.900109399	456
chr5.fa	102421704	102455842	GIN1	1.428640306	228
chr5.fa	102465257	102538909	PPIP5K2	7.99851763	1899
chr5.fa	102594442	102614361	C5orf30	8.422839813	1140
chr5.fa	102884556	102898490	NUDT12	12.59867715	1976
chr5.fa	104435175	104435799	RAB9BP1	0	0
chr5.fa	106712590	107006596	EFNA5	3.168183344	760
chr5.fa	107194734	107717799	FBXL17	8.125725329	1900
chr5.fa	108083523	108523373	FER	5.156359611	684
chr5.fa	108670410	108745675	PJA2	32.97726149	7220
chr5.fa	109025156	109203429	MAN2A1	6.591894041	1520
chr5.fa	109218883	109221200	LOC100289673	0	0
chr5.fa	109755198	110062450	TMEM232	0	0
chr5.fa	110074754	110098484	SLC25A46	17.22908185	1802
chr5.fa	110407390	110413722	TSLP	0	0
chr5.fa	110427870	110466200	WDR36	3.589614443	1064
chr5.fa	110559947	110820748	CAMK4	0	0
chr5.fa	110834022	110848157	STARD4	0.74656686	76
chr5.fa	111065000	111312628	C5orf13	6.371504479	836
chr5.fa	111496223	111498198	NCRNA00219	36.80438961	1976
chr5.fa	111497182	111497314	SNORA13	0	0
chr5.fa	111498315	111755010	EPB41L4A	10.64941932	2254
chr5.fa	111755280	111756677	FLJ11235	4.835894063	304
chr5.fa	112043202	112181935	APC	4.069089615	2052
chr5.fa	112196993	112203604	SRP19	28.61461565	1140
chr5.fa	112212081	112258031	REEP5	8.678589557	1216
chr5.fa	112312433	112356667	DCP2	6.234066791	2508
chr5.fa	112357796	112824527	MCC	1.276747198	532
chr5.fa	112768251	112770728	TSSK1B	0	0
chr5.fa	112849391	112930984	YTHDC2	7.502363128	2128
chr5.fa	113698016	113832197	KCNN2	7.53772331	988
chr5.fa	114460459	114516243	TRIM36	3.800441188	836
chr5.fa	114546527	114598569	PGGT1B	12.99942588	1594
chr5.fa	114602885	114632458	CCDC112	2.937341402	380
chr5.fa	114856608	114880591	FEM1C	5.233529316	1368
chr5.fa	114916895	114938176	TICAM2	3.373672703	76
chr5.fa	114948905	114952014	TMED7	1.086825214	152
chr5.fa	115140430	115152405	CDO1	1.047239602	76
chr5.fa	115163894	115177548	ATG12	7.039344896	1444
chr5.fa	115177619	115249778	AP3S1	15.73483618	912

chr5.fa	115298151	115363299	AQPEP		0	0
chr5.fa	115387163	115394827	LOC644100		0	0
chr5.fa	115420727	115628978	COMMD10		17.8037404	1140
chr5.fa	115779251	115910551	SEMA6A		2.46387079	760
chr5.fa	118172569	118324240	DTWD2		2.378695006	532
chr5.fa	118407084	118584822	DMXL1		33.9568942	17060
chr5.fa	118604418	118730294	TNFAIP8		2.315313547	228
chr5.fa	118788138	118878030	HSD17B4		75.70993015	10002
chr5.fa	118965254	118971517	FAM170A		0	0
chr5.fa	119800019	120022964	PRR16		0.958727951	76
chr5.fa	121187650	121188523	FTMT		0	0
chr5.fa	121297656	121364295	SRFBP1		9.527678705	1215
chr5.fa	121398890	121414055	LOX		0.326470107	76
chr5.fa	121465215	121489266	ZNF474		0	0
chr5.fa	121495871	121518356	LOC728460		0	0
chr5.fa	121647820	121799794	SNCAIP		0	0
chr5.fa	122110750	122165802	SNX2		44.13417726	4104
chr5.fa	122181160	122344902	SNX24		5.536425969	532
chr5.fa	122359078	122372425	PPIC		0	0
chr5.fa	122424841	122523745	PRDM6		0	0
chr5.fa	122680579	122759286	CEP120		6.816731424	1444
chr5.fa	122847793	122952464	CSNK1G3		11.74514018	2356
chr5.fa	123972610	124080805	ZNF608		10.17995175	2584
chr5.fa	125695788	125829853	GRAMD3		9.614188836	1520
chr5.fa	125878918	125931082	ALDH7A1		50.38403381	8045
chr5.fa	125936607	125962944	PHAX		14.3902597	2356
chr5.fa	125967414	125971974	C5orf48		0	0
chr5.fa	126112315	126172712	LMNB1		0.496599285	76
chr5.fa	126203406	126366440		3-Mar	1.634574448	304
chr5.fa	126378250	126409184	FLJ44606		0.801719848	228
chr5.fa	126626456	126796910	MEGF10		0	0
chr5.fa	126853309	126890780	PRRC1		26.66802651	5624
chr5.fa	126984713	126994322	CTXN3		10.9042795	912
chr5.fa	127357244	127418766	FLJ33630		2.861728434	380
chr5.fa	127419483	127525380	SLC12A2		5.420337825	1672
chr5.fa	127593601	127873735	FBN2		11.66285548	5624
chr5.fa	128301210	128369335	SLC27A6		0	0
chr5.fa	128430442	128449719	ISOC1		17.42456361	1520
chr5.fa	128796103	129074376	ADAMTS19		0.332029884	76
chr5.fa	129240523	129522327	CHSY3		0	0
chr5.fa	130494875	130501034	HINT1		322.5775963	14592
chr5.fa	130506641	130541119	LYRM7		2.99249439	836
chr5.fa	130599702	130730382	CDC42SE2		40.22009423	6612

chr5.fa	130759614	130970929	RAPGEF6		4.684890519	1976
chr5.fa	130977407	131132756	FNIP1		8.433069803	2508
chr5.fa	131289152	131347349	ACSL6		0	0
chr5.fa	131396347	131398896	IL3		0	0
chr5.fa	131409485	131411859	CSF2		0	0
chr5.fa	131528304	131563556	P4HA2		3.749068849	456
chr5.fa	131593351	131609147	PDLIM4		7.364480657	760
chr5.fa	131630145	131679899	SLC22A4		0.771029879	76
chr5.fa	131701182	131701291	MIR3936		0	0
chr5.fa	131705401	131731306	SLC22A5		3.607183339	532
chr5.fa	131746673	131798059	C5orf56		0	0
chr5.fa	131817301	131826465	IRF1		6.18247206	988
chr5.fa	131877136	131879214	IL5		0	0
chr5.fa	131892616	131980313	RAD50		21.52100733	6384
chr5.fa	131993865	131996801	IL13		0	0
chr5.fa	132009373	132018368	IL4		0	0
chr5.fa	132028323	132073265	KIF3A		6.212939638	1748
chr5.fa	132083137	132089856	CCNI2		1.691951347	152
chr5.fa	132086509	132113561		8-Sep	59.12244657	14744
chr5.fa	132149033	132152489	ANKRD43		6.355937104	988
chr5.fa	132157833	132166590	SHROOM1		33.38579391	6152
chr5.fa	132196878	132200477	GDF9		0.836635248	76
chr5.fa	132202319	132204536	UQCRQ		146.0907028	10412
chr5.fa	132209358	132210582	LEAP2		14.34377996	456
chr5.fa	132211071	132299354	AFF4		32.49511763	13998
chr5.fa	132332678	132362240	ZCCHC10		4.686892038	447
chr5.fa	132387662	132440709	HSPA4		60.36427837	9188
chr5.fa	132532152	132948223	FSTL4		0.314016207	76
chr5.fa	132763288	132763398	MIR1289-2		0	0
chr5.fa	133291198	133304406	C5orf15		2.96380594	304
chr5.fa	133307566	133340824	VDAC1		79.63802382	8025
chr5.fa	133450402	133483920	TCF7		0.807057234	152
chr5.fa	133492082	133512724	SKP1		67.63201891	8208
chr5.fa	133532148	133561950	PPP2CA		37.43153246	4292
chr5.fa	133561448	133561450	MIR3661		0	0
chr5.fa	133634115	133702765	CDKL3		0.747234033	76
chr5.fa	133706870	133727799	UBE2B		13.17778352	1559
chr5.fa	133737756	133747598	CDKN2AIPNL		20.21735081	1140
chr5.fa	133861798	133918918	PHF15		10.7221412	3116
chr5.fa	133936839	133968533	SAR1B		17.02626118	5092
chr5.fa	133984479	134063601	SEC24A		9.23056422	2651
chr5.fa	134074206	134087843	CAMLG		10.76061486	1064
chr5.fa	134094461	134166812	DDX46		11.6050338	2964

chr5.fa	134181370	134195425	C5orf24	9.205434028	2128
chr5.fa	134209460	134237323	TXNDC15	1.832724901	304
chr5.fa	134240810	134298336	PCBD2	2.843047583	304
chr5.fa	134303596	134347397	CATSPER3	0	0
chr5.fa	134363424	134369964	PITX1	3.546248182	380
chr5.fa	134670071	134735577	H2AFY	44.00252174	4256
chr5.fa	134779904	134783038	C5orf20	0	0
chr5.fa	134784558	134788089	TIFAB	0	0
chr5.fa	134869972	134871639	NEUROG1	0	0
chr5.fa	134906371	134914969	CXCL14	10.27980534	912
chr5.fa	134984372	134989624	LOC340074	0	0
chr5.fa	135170365	135224326	SLC25A48	2.819251738	152
chr5.fa	135227935	135231516	IL9	0	0
chr5.fa	135266006	135277367	FBXL21	0	0
chr5.fa	135282600	135290723	LECT2	0	0
chr5.fa	135364584	135399507	TGFBI	2.212124086	279
chr5.fa	135416187	135416286	VTRNA2	0	0
chr5.fa	135465203	135470579	SMAD5OS	0	0
chr5.fa	135468536	135518422	SMAD5	6.431994853	2052
chr5.fa	135527156	135528851	LOC389332	7.972497873	608
chr5.fa	135548999	135701164	TRPC7	0	0
chr5.fa	136310987	136835018	SPOCK1	6.164458383	1338
chr5.fa	136953189	137071779	KLHL3	3.725050612	1140
chr5.fa	136983261	136983338	MIR874	0	0
chr5.fa	137087073	137090039	HNRNPA0	39.87605522	5320
chr5.fa	137136882	137146439	NPY6R	0	0
chr5.fa	137203545	137223540	MYOT	0	0
chr5.fa	137225125	137276156	PKD2L2	0	0
chr5.fa	137273642	137368802	FAM13B	15.08745574	3637
chr5.fa	137419774	137427199	WNT8A	0	0
chr5.fa	137450861	137475132	NME5	2.757204626	152
chr5.fa	137475459	137514358	BRD8	14.57684581	3061
chr5.fa	137514417	137523336	KIF20A	0	0
chr5.fa	137523405	137549032	CDC23	8.212680241	1140
chr5.fa	137588069	137610253	GFRA3	0	0
chr5.fa	137620959	137667516	CDC25C	0	0
chr5.fa	137673224	137685418	FAM53C	15.06521663	3192
chr5.fa	137688285	137772716	KDM3B	40.68511398	12464
chr5.fa	137774776	137782658	REEP2	0.805278105	76
chr5.fa	137801181	137805004	EGR1	3.772642303	532
chr5.fa	137841784	137878932	ETF1	32.95769108	5467
chr5.fa	137890571	137911318	HSPA9	67.00287454	10563
chr5.fa	137896732	137896799	SNORD63	0	0

chr5.fa	138089107	138270723	CTNNA1	104.2593852	17632
chr5.fa	138205079	138211057	LRRTM2	0	0
chr5.fa	138282410	138534065	SIL1	3.400804415	304
chr5.fa	138614469	138614668	SNORA74A	0	0
chr5.fa	138618428	138618873	SNHG4	0	0
chr5.fa	138629333	138667366	MATR3	29.48705586	7144
chr5.fa	138677519	138705409	PAIP2	63.31763193	4712
chr5.fa	138702885	138719039	SLC23A1	5.727015126	608
chr5.fa	138723257	138725605	MZB1	0	0
chr5.fa	138727635	138730885	LOC389333	2.079579001	304
chr5.fa	138732456	138739776	SPATA24	4.985785652	152
chr5.fa	138745892	138775214	DNAJC18	0	0
chr5.fa	138784245	138842320	ECSCR	0	0
chr5.fa	138855113	138862343	TMEM173	0	0
chr5.fa	138940751	139008018	UBE2D2	23.06929404	2973
chr5.fa	139028301	139062680	CXXC5	17.52085895	1140
chr5.fa	139175406	139224048	PSD2	1.499138279	304
chr5.fa	139226364	139422884	NRG2	0	0
chr5.fa	139493708	139499001	PURA	10.21642388	2432
chr5.fa	139505521	139508391	C5orf53	7.653144281	988
chr5.fa	139554653	139623374	C5orf32	59.43468365	2432
chr5.fa	139624635	139682689	PFDN1	38.29685616	2280
chr5.fa	139712428	139726188	HBEGF	0.716766455	76
chr5.fa	139739787	139754722	SLC4A9	0.516614482	76
chr5.fa	139851824	139919441	ANKHD1	2.11227049	70
chr5.fa	139921757	139921866	ANKHD1-EIF4EBP3	11.92816804	59
chr5.fa	139927251	139927425	EIF4EBP3	9.65822227	76
chr5.fa	139929653	139937678	SRA1	1.735317607	152
chr5.fa	139937853	139944189	APBB3	2.356233506	228
chr5.fa	139944420	139948683	SLC35A4	32.44218855	3876
chr5.fa	140011313	140013286	CD14	0	0
chr5.fa	140019012	140024947	TMCO6	1.830278599	152
chr5.fa	140024990	140027370	NDUFA2	108.6705123	3572
chr5.fa	140027384	140042065	IK	55.28597803	4609
chr5.fa	140027510	140027511	MIR3655	0	0
chr5.fa	140044384	140050380	WDR55	18.25697343	1976
chr5.fa	140050554	140053171	DND1	3.5458034	228
chr5.fa	140053490	140070971	HARS	24.95672714	2204
chr5.fa	140071018	140078890	HARS2	9.502993295	1064
chr5.fa	140080032	140086239	ZMAT2	68.188219	4636
chr5.fa	140090861	140090958	VTRNA1-1	0	0
chr5.fa	140098511	140098598	VTRNA1-2	0	0
chr5.fa	140105744	140105831	VTRNA1-3	0	0

chr5.fa	140165876	140168269	PCDHA1	0	0
chr5.fa	140174444	140177062	PCDHA2	0	0
chr5.fa	140180783	140183176	PCDHA3	0	0
chr5.fa	140186672	140189169	PCDHA4	0	0
chr5.fa	140201361	140203712	PCDHA5	0	0
chr5.fa	140207650	140210070	PCDHA6	0	0
chr5.fa	140213969	140216323	PCDHA7	0	0
chr5.fa	140220907	140223300	PCDHA8	0	0
chr5.fa	140227357	140230474	PCDHA9	0	0
chr5.fa	140235634	140238021	PCDHA10	0	0
chr5.fa	140247831	140251079	PCDHA11	0	0
chr5.fa	140254931	140257436	PCDHA12	0	0
chr5.fa	140261854	140264280	PCDHA13	0	0
chr5.fa	140306302	140308910	PCDHAC1	0	0
chr5.fa	140345747	140349006	PCDHAC2	0	0
chr5.fa	140430979	140433512	PCDHB1	0	0
chr5.fa	140474237	140476964	PCDHB2	0	0
chr5.fa	140480234	140483406	PCDHB3	0.53262664	76
chr5.fa	140501581	140505201	PCDHB4	0	0
chr5.fa	140514800	140517704	PCDHB5	0.581775069	76
chr5.fa	140529839	140532868	PCDHB6	0	0
chr5.fa	140535580	140537990	PCDHB17	0	0
chr5.fa	140552243	140555957	PCDHB7	0	0
chr5.fa	140557430	140560021	PCDHB8	0	0
chr5.fa	140560980	140565796	PCDHB16	0	0
chr5.fa	140566893	140571111	PCDHB9	0	0
chr5.fa	140571952	140575213	PCDHB10	0	0
chr5.fa	140579348	140582618	PCDHB11	0	0
chr5.fa	140588291	140591698	PCDHB12	0	0
chr5.fa	140593509	140596993	PCDHB13	0	0
chr5.fa	140603078	140605860	PCDHB14	0	0
chr5.fa	140613938	140617101	PCDHB18	0	0
chr5.fa	140619689	140624312	PCDHB19P	0	0
chr5.fa	140625147	140627801	PCDHB15	0	0
chr5.fa	140682196	140683612	SLC25A2	0	0
chr5.fa	140698057	140700351	TAF7	47.13334338	4864
chr5.fa	140710252	140712672	PCDHGA1	0	0
chr5.fa	140718354	140720962	PCDHGA2	0	0
chr5.fa	140723601	140726024	PCDHGA3	0	0
chr5.fa	140729828	140732236	PCDHGB1	0	0
chr5.fa	140734768	140737296	PCDHGA4	0	0
chr5.fa	140739703	140742138	PCDHGB2	0	0
chr5.fa	140743898	140746318	PCDHGA5	0	0

chr5.fa	140749962	140752406	PCDHGB3	0	0
chr5.fa	140753651	140756107	PCDHGA6	0	0
chr5.fa	140762467	140765187	PCDHGA7	0	0
chr5.fa	140767452	140769863	PCDHGB4	0	0
chr5.fa	140771483	140774804	PCDHGA8	0	0
chr5.fa	140777695	140780151	PCDHGB5	0.687855614	76
chr5.fa	140782520	140784943	PCDHGA9	0	0
chr5.fa	140787770	140790187	PCDHGB6	0	0
chr5.fa	140792743	140795178	PCDHGA10	0	0
chr5.fa	140797282	140799841	PCDHGB7	0	0
chr5.fa	140800537	140803227	PCDHGA11	0	0
chr5.fa	140805853	140807825	PCDHGB8P	0	0
chr5.fa	140810158	140812750	PCDHGA12	0	0
chr5.fa	140855569	140855713	PCDHGC3	0	0
chr5.fa	140864741	140867182	PCDHGC4	0	0
chr5.fa	140868808	140871267	PCDHGC5	0	0
chr5.fa	140894588	140998622	DIAPH1	119.6110416	31141
chr5.fa	141000443	141016423	HDAC3	29.22530156	2556
chr5.fa	141016517	141018588	RELL2	0	0
chr5.fa	141018869	141030986	FCHSD1	0.988305965	148
chr5.fa	141032968	141061800	ARAP3	0	0
chr5.fa	141232673	141257944	PCDH1	11.69999479	2812
chr5.fa	141275191	141276260	LOC729080	0	0
chr5.fa	141303385	141321612	KIAA0141	7.304435065	1368
chr5.fa	141324530	141338627	PCDH12	0	0
chr5.fa	141346437	141368754	RNF14	26.28573625	3800
chr5.fa	141380234	141392620	GNPDA1	17.65963098	1824
chr5.fa	141488324	141534008	NDFIP1	12.21705405	1976
chr5.fa	141689992	141704620	SPRY4	18.96017403	4256
chr5.fa	141971743	142077635	FGF1	1.594099271	304
chr5.fa	142150292	142608572	ARHGAP26	3.362553149	1367
chr5.fa	142657496	142815077	NR3C1	6.655053108	2356
chr5.fa	143191726	143200284	HMHB1	0	0
chr5.fa	143537729	143550278	YIPF5	6.374395563	988
chr5.fa	143550437	143856944	KCTD16	0	0
chr5.fa	145138582	145214899	PRELID2	2.846605841	304
chr5.fa	145239296	145252531	GRXCR2	0	0
chr5.fa	145316126	145442879	SH3RF2	0.555532921	76
chr5.fa	145463876	145483946	PLAC8L1	0	0
chr5.fa	145492589	145562294	LARS	29.2791202	6384
chr5.fa	145583163	145668784	RBM27	14.47232201	4256
chr5.fa	145718587	145720083	POU4F3	0	0
chr5.fa	145826873	145891069	TCERG1	16.49007629	3115

chr5.fa	145894417	145895676	GPR151	0	0
chr5.fa	145969068	146461033	PPP2R2B	3.914750204	608
chr5.fa	146614579	146763768	STK32A	0.924702116	76
chr5.fa	146770371	146889619	DPYSL3	9.007950748	2280
chr5.fa	146970706	147162252	JAKMIP2	1.012768984	152
chr5.fa	147204143	147211260	SPINK1	225.4671947	4471
chr5.fa	147258274	147261756	SCGB3A2	0	0
chr5.fa	147272271	147286101	C5orf46	0	0
chr5.fa	147443535	147516925	SPINK5	0	0
chr5.fa	147549296	147554961	SPINK14	0	0
chr5.fa	147582357	147594700	SPINK6	0	0
chr5.fa	147648423	147665773	SPINK13	0	0
chr5.fa	147691990	147695481	SPINK7	0	0
chr5.fa	147715122	147719412	SPINK9	0	0
chr5.fa	147763546	147822399	FBXO38	22.40167601	4408
chr5.fa	147830595	148033739	HTR4	0	0
chr5.fa	148206156	148208197	ADRB2	0.827739605	76
chr5.fa	148361713	148442737	SH3TC2	6.740451283	8056
chr5.fa	148521054	148639999	ABLIM3	19.10450584	3724
chr5.fa	148651401	148721367	AFAP1L1	0.401193511	76
chr5.fa	148724977	148734146	GRPEL2	5.352508545	988
chr5.fa	148737570	148749221	PCYOX1L	0.662503031	76
chr5.fa	148753830	148758838	IL17B	0	0
chr5.fa	148786440	148812399	LOC728264	0	0
chr5.fa	148874845	148931007	CSNK1A1	80.51001925	11400
chr5.fa	148961135	149014527	ARHGEF37	34.74148994	7600
chr5.fa	149109815	149234585	PPARGC1B	2.341555695	1140
chr5.fa	149237519	149324356	PDE6A	0	0
chr5.fa	149340300	149366963	SLC26A2	1.255842436	456
chr5.fa	149372683	149380168	TIGD6	2.455197538	380
chr5.fa	149380218	149432706	HMGXB3	21.77008534	5244
chr5.fa	149432854	149492935	CSF1R	0	0
chr5.fa	149493402	149535422	PDGFRB	0	0
chr5.fa	149546344	149564121	CDX1	0	0
chr5.fa	149569520	149590635	SLC6A7	0	0
chr5.fa	149599054	149669403	CAMK2A	0	0
chr5.fa	149675909	149682525	ARSI	0	0
chr5.fa	149737202	149779871	TCOF1	8.177320059	2204
chr5.fa	149781200	149792323	CD74	6.738227372	456
chr5.fa	149823792	149829319	RPS14	1376.221616	48640
chr5.fa	149887674	149937773	NDST1	7.787913276	2812
chr5.fa	149980642	150038792	SYNPO	3.277599756	1216
chr5.fa	150040404	150058925	MYOZ3	0	0

chr5.fa	150070352	150080669	RBM22	13.68861584	1444
chr5.fa	150088309	150138657	DCTN4	25.42574993	4931
chr5.fa	150157508	150176298	C5orf62	3.620749195	380
chr5.fa	150226085	150228231	IRGM	0	0
chr5.fa	150273954	150284545	ZNF300	0.508608403	76
chr5.fa	150309998	150326146	ZNF300P1	2.793676763	304
chr5.fa	150399999	150408554	GPX3	474.1311136	37544
chr5.fa	150409506	150460997	TNIP1	32.5829621	4788
chr5.fa	150480267	150537443	ANXA6	10.30916096	1444
chr5.fa	150560613	150603654	CCDC69	13.92323843	2128
chr5.fa	150632613	150649953	GM2A	3.220222857	532
chr5.fa	150655926	150683334	SLC36A3	0	0
chr5.fa	150694539	150727151	SLC36A2	0.988083574	152
chr5.fa	150827163	150871940	SLC36A1	5.562668117	1444
chr5.fa	150883653	150948505	FAT2	1.395281644	912
chr5.fa	151041009	151066517	SPARC	2.153190449	304
chr5.fa	151122383	151138210	ATOX1	76.0404033	1672
chr5.fa	151151476	151184915	G3BP1	38.75164592	4940
chr5.fa	151202074	151304397	GLRA1	0	0
chr5.fa	151771102	151784840	NMUR2	0	0
chr5.fa	152870084	153193429	GRIA1	0	0
chr5.fa	153371269	153418497	FAM114A2	12.32424655	1596
chr5.fa	153418519	153437014	MFAP3	1.896995923	456
chr5.fa	153570295	153800543	GALNT10	3.97346145	1064
chr5.fa	153825517	153840613	SAP30L	12.40186104	3496
chr5.fa	153854532	153857824	HAND1	0	0
chr5.fa	153975572	153975632	MIR3141	0	0
chr5.fa	154092462	154197163	LARP1	57.18542025	16948
chr5.fa	154198052	154230213	C5orf4	0.563983782	76
chr5.fa	154238198	154256352	CNOT8	14.29463153	1596
chr5.fa	154266976	154317776	GEMIN5	9.382902111	2280
chr5.fa	154320633	154348971	MRPL22	5.270668627	752
chr5.fa	154393260	154397685	KIF4B	0	0
chr5.fa	155753767	156194798	SGCD	0.690746699	304
chr5.fa	156277549	156279539	PPP1R2P3	0	0
chr5.fa	156346293	156390266	TIMD4	1.234492892	76
chr5.fa	156351100	156352013	LOC100286948	0	0
chr5.fa	156456531	156485970	HAVCR1	0	0
chr5.fa	156512843	156536138	HAVCR2	0	0
chr5.fa	156565451	156569921	MED7	3.437721335	228
chr5.fa	156589344	156593279	FAM71B	0	0
chr5.fa	156607907	156682109	ITK	0	0
chr5.fa	156693091	156822606	CYFIP2	23.50429099	7524

chr5.fa	156768607	156772729	C5orf40	0	0
chr5.fa	156887027	156901730	NIPAL4	6.185363144	912
chr5.fa	156904312	157002783	ADAM19	0	0
chr5.fa	157052687	157079428	SOX30	0.517726437	76
chr5.fa	157098561	157107162	C5orf52	0	0
chr5.fa	157158323	157166772	THG1L	14.24659506	836
chr5.fa	157170755	157183746	LSM11	0.660056729	76
chr5.fa	157212751	157286183	CLINT1	45.86193357	8220
chr5.fa	158122923	158526788	EBF1	0	0
chr5.fa	158584417	158637061	RNF145	1.959932599	380
chr5.fa	158690089	158713048	UBLCP1	5.652736504	608
chr5.fa	158741791	158757481	IL12B	0	0
chr5.fa	158875564	158893284	LOC285627	0	0
chr5.fa	159343740	159400017	ADRA1B	0	0
chr5.fa	159436180	159492550	TTC1	34.44148437	2179
chr5.fa	159502892	159546452	PWWP2A	7.907559678	2052
chr5.fa	159614374	159665729	FABP6	0	0
chr5.fa	159678671	159739573	CCNJL	5.678756261	836
chr5.fa	159774775	159797648	C1QTNF2	0	0
chr5.fa	159820155	159827060	C5orf54	1.806482753	228
chr5.fa	159828648	159846168	SLU7	17.93495114	2871
chr5.fa	159848865	159855746	PTTG1	0	0
chr5.fa	159901409	159901490	MIR3142	0	0
chr5.fa	159912359	159912457	MIR146A	0	0
chr5.fa	159990127	160279219	ATP10B	6.031468516	2052
chr5.fa	160358786	160365633	LOC285629	0	0
chr5.fa	160715436	160975130	GABRB2	0	0
chr5.fa	161112658	161129598	GABRA6	0	0
chr5.fa	161274197	161326965	GABRA1	0	0
chr5.fa	161494648	161582545	GABRG2	0	0
chr5.fa	162864577	162872022	CCNG1	73.94881518	7874
chr5.fa	162880586	162887143	NUDCD2	15.57671612	664
chr5.fa	162887517	162918953	HMMR	0	0
chr5.fa	162930231	162946328	MAT2B	36.45167735	3724
chr5.fa	166711843	167691162	ODZ2	0.175244172	76
chr5.fa	167719065	167899308	WWC1	61.69795768	18696
chr5.fa	167913463	167946309	RARS	36.36472244	3496
chr5.fa	167956582	167957639	FBLL1	0	0
chr5.fa	167982628	168006614	PANK3	22.95476263	3496
chr5.fa	167987901	167987978	MIR103-1	0	0
chr5.fa	168093071	168728133	SLIT3	0.314238598	76
chr5.fa	168195151	168195260	MIR218-2	0	0
chr5.fa	168690605	168690698	MIR585	0	0

chr5.fa	169010638	169031781	CCDC99	4.43625729	532
chr5.fa	169064251	169510386	DOCK2	0	0
chr5.fa	169290719	169407744	FAM196B	0.476361696	76
chr5.fa	169532917	169536729	FOXI1	3.680794787	380
chr5.fa	169659950	169673235	C5orf58	0	0
chr5.fa	169675088	169724822	LCP2	0	0
chr5.fa	169758435	169762104	LOC257358	0	0
chr5.fa	169780881	170163636	KCNIP1	0	0
chr5.fa	169805167	169816638	KCNMB1	0	0
chr5.fa	170210723	170241050	GABRP	0.510832314	76
chr5.fa	170288896	170727019	RANBP17	2.591078488	532
chr5.fa	170736288	170739138	TLX3	0	0
chr5.fa	170813660	170813764	MIR3912	0	0
chr5.fa	170814708	170837888	NPM1	111.0956871	8892
chr5.fa	170846667	170884630	FGF18	0	0
chr5.fa	171288556	171433877	FBXW11	17.4921705	3648
chr5.fa	171469074	171615346	STK10	6.177801847	1672
chr5.fa	171621176	171630458	EFCAB9	0	0
chr5.fa	171636650	171710795	UBTD2	13.45421564	1824
chr5.fa	171760503	171881527	SH3PXD2B	6.51983933	2280
chr5.fa	172068276	172118533	NEURL1B	1.319001503	380
chr5.fa	172195093	172198203	DUSP1	36.74278728	3344
chr5.fa	172261223	172379688	ERGIC1	55.8401766	7372
chr5.fa	172381786	172386371	LOC100268168	0	0
chr5.fa	172386439	172396774	RPL26L1	21.42471199	684
chr5.fa	172410763	172461900	ATP6V0E1	108.6740706	4310
chr5.fa	172447729	172447932	SNORA74B	0	0
chr5.fa	172483355	172566291	C5orf41	5.044719288	1976
chr5.fa	172571445	172591390	BNIP1	6.097296276	380
chr5.fa	172659107	172662315	NKX2-5	0	0
chr5.fa	172741726	172756506	STC2	0.632702626	152
chr5.fa	173006646	173012075	LOC285593	0	0
chr5.fa	173034148	173043666	BOD1	10.48707383	912
chr5.fa	173315331	173387313	CPEB4	22.19040448	7752
chr5.fa	173416162	173433143	C5orf47	0	0
chr5.fa	173472724	173536182	HMP19	0	0
chr5.fa	174151575	174157902	MSX2	15.30962443	1520
chr5.fa	174867675	174871163	DRD1	0	0
chr5.fa	174905514	174955621	SFXN1	16.387554	2204
chr5.fa	175085040	175113245	HRH2	0	0
chr5.fa	175223610	175311023	CPLX2	0	0
chr5.fa	175386534	175395545	THOC3	0.924702116	76
chr5.fa	175511909	175543457	FAM153B	0	0

chr5.fa	175570088	175626298	LOC643201	0	0
chr5.fa	175665370	175772990	C5orf25	3.285161053	304
chr5.fa	175773066	175788809	KIAA1191	54.40597652	6916
chr5.fa	175792502	175800503	ARL10	0	0
chr5.fa	175811232	175815540	NOP16	3.147500974	76
chr5.fa	175815784	175816751	HIGD2A	85.75333378	2356
chr5.fa	175819456	175843540	CLTB	36.10808313	1924
chr5.fa	175875356	175937075	FAF2	6.738227372	1368
chr5.fa	175953700	175964421	RNF44	8.139513576	1520
chr5.fa	175969512	176022769	CDHR2	0.386293308	76
chr5.fa	176022803	176037131	GPRIN1	0.401638293	76
chr5.fa	176047210	176057557	SNCB	0	0
chr5.fa	176056440	176056501	MIR4281	0	0
chr5.fa	176057683	176073642	EIF4E1B	0	0
chr5.fa	176074388	176086059	TSPAN17	10.31182966	1200
chr5.fa	176237560	176307869	UNC5A	0	0
chr5.fa	176307900	176326333	HK3	0	0
chr5.fa	176332006	176433795	UIMC1	10.79797656	1444
chr5.fa	176449697	176493745	ZNF346	4.400007544	608
chr5.fa	176513921	176525126	FGFR4	0.537741635	76
chr5.fa	176560080	176727214	NSD1	22.13191563	12996
chr5.fa	176728199	176730744	RAB24	14.5272526	1053
chr5.fa	176730835	176731851	PRELID1	68.04210806	1438
chr5.fa	176732501	176739292	MXD3	0.692748218	76
chr5.fa	176758563	176778885	LMAN2	14.76143041	1216
chr5.fa	176784844	176799599	RGS14	1.405066852	152
chr5.fa	176811432	176825849	SLC34A1	1.953483258	304
chr5.fa	176827108	176827637	PFN3	0	0
chr5.fa	176829139	176836577	F12	0.824181347	76
chr5.fa	176853687	176869850	GRK6	8.282511041	1140
chr5.fa	176873796	176883287	PRR7	0	0
chr5.fa	176883614	176900694	DBN1	3.567820117	532
chr5.fa	176910395	176924602	PDLIM7	2.444745157	228
chr5.fa	176928906	176937427	DOK3	0	0
chr5.fa	176938578	176943967	DDX41	18.47625103	1748
chr5.fa	176946790	176981548	FAM193B	3.2989493	684
chr5.fa	177019213	177023099	TMED9	15.77330983	988
chr5.fa	177027119	177037346	B4GALT7	3.914750204	304
chr5.fa	177045501	177099278	LOC202181	1.183120553	152
chr5.fa	177150365	177207505	FAM153A	0	0
chr5.fa	177302262	177311269	LOC728554	2.775218304	228
chr5.fa	177419236	177423243	PROP1	0	0
chr5.fa	177435689	177474656	FAM153C	0	0

chr5.fa	177540556	177553107	N4BP3	1.693952866	456
chr5.fa	177558028	177575479	RMND5B	37.39328119	3040
chr5.fa	177576465	177580961	NHP2	91.03979217	3496
chr5.fa	177611511	177614433	GMCL1L	0	0
chr5.fa	177631508	177638184	HNRNPAB	78.1798055	6384
chr5.fa	177635540	177659803	AGXT2L2	15.1132531	1368
chr5.fa	177664617	178017556	COL23A1	0	0
chr5.fa	178029665	178054054	CLK4	4.045071378	456
chr5.fa	178138522	178157703	ZNF354A	1.98461801	228
chr5.fa	178191864	178203277	AACSP1	0	0
chr5.fa	178286954	178311424	ZNF354B	0.76925075	76
chr5.fa	178322916	178360210	ZFP2	0	0
chr5.fa	178368194	178393218	ZNF454	0.799495937	76
chr5.fa	178405330	178422124	GRM6	0	0
chr5.fa	178450776	178461388	ZNF879	0.649159566	76
chr5.fa	178487607	178507691	ZNF354C	0.712318633	76
chr5.fa	178537852	178772431	ADAMTS2	0	0
chr5.fa	178977571	179037019	RUFY1	17.94762743	2204
chr5.fa	179041179	179050722	HNRNPH1	57.11558945	5776
chr5.fa	179068558	179072047	C5orf60	0	0
chr5.fa	179105559	179107975	CBY3	0	0
chr5.fa	179125930	179158639	CANX	49.83361589	11092
chr5.fa	179159851	179204287	MAML1	7.678496864	1976
chr5.fa	179220986	179223513	LTC4S	0	0
chr5.fa	179224598	179233952	MGAT4B	18.63637261	1976
chr5.fa	179225278	179225346	MIR1229	0	0
chr5.fa	179233388	179265077	SQSTM1	145.6485894	17100
chr5.fa	179267872	179285840	C5orf45	11.63639094	304
chr5.fa	179289071	179334856	TBC1D9B	26.78166836	6220
chr5.fa	179382474	179499109	RNF130	24.70742674	2052
chr5.fa	179442303	179442397	MIR340	0	0
chr5.fa	179527795	179636130	RASGEF1C	1.525380427	152
chr5.fa	179660595	179719071	MAPK9	10.44926735	2736
chr5.fa	179727700	179780315	GFPT2	0	0
chr5.fa	179921417	180005353	CNOT6	16.14648207	4484
chr5.fa	180017105	180018487	SCGB3A1	7.25395229	152
chr5.fa	180028506	180076624	FLT4	0	0
chr5.fa	180166123	180167058	OR2Y1	0	0
chr5.fa	180217541	180237137	MGAT1	9.44228053	1672
chr5.fa	180256959	180262726	LOC729678	8.181990272	1064
chr5.fa	180274611	180288286	ZFP62	9.660446181	1748
chr5.fa	180326077	180377906	BTNL8	0	0
chr5.fa	180415845	180433727	BTNL3	0	0

chr5.fa	180467225	180488523	BTNL9	0	0
chr5.fa	180581943	180582890	OR2V2	0	0
chr5.fa	180620924	180632177	TRIM7	0.437220866	76
chr5.fa	180650306	180662808	TRIM41	13.77356923	2242
chr5.fa	180663928	180670906	GNB2L1	1258.865843	62776
chr5.fa	180668818	180668889	SNORD96A	0	0
chr5.fa	180670314	180670376	SNORD95	0	0
chr5.fa	180683386	180688119	TRIM52	0.758575978	76
chr6.fa	292101	351355	DUSP22	9.129821061	608
chr6.fa	391739	411443	IRF4	0	0
chr6.fa	485138	693109	EXOC2	14.77944409	2952
chr6.fa	655939	656964	HUS1B	0	0
chr6.fa	961241	1101567	LOC285768	1.444652464	152
chr6.fa	1312675	1314993	FOXQ1	46.64563974	4864
chr6.fa	1390069	1395832	FOXF2	0.772809008	76
chr6.fa	1610681	1614129	FOXC1	3.920309981	608
chr6.fa	1624041	2245846	GMDS	10.1087866	760
chr6.fa	2622972	2635298	C6orf195	0	0
chr6.fa	2663863	2751154	MYLK4	0.586890064	152
chr6.fa	2765666	2785979	WRNIP1	17.84488275	2128
chr6.fa	2833734	2842081	SERPINB1	17.00646838	988
chr6.fa	2854891	2876744	MGC39372	1.349913863	76
chr6.fa	2887504	2903545	SERPINB9	1.63101619	304
chr6.fa	2948393	2972399	SERPINB6	61.07059244	5624
chr6.fa	2988201	2991405	DKFZP686I15217	0.601790266	76
chr6.fa	3000067	3019994	NQO2	37.09772344	1900
chr6.fa	3020390	3025005	LOC401233	2.197001492	456
chr6.fa	3045618	3046034	FAM136BP	0	0
chr6.fa	3077058	3115421	RIPK1	18.80894809	3268
chr6.fa	3118926	3153432	BPHL	19.29642934	1600
chr6.fa	3153902	3157783	TUBB2A	4.175837334	304
chr6.fa	3224495	3227968	TUBB2B	0.83708003	76
chr6.fa	3259162	3268300	PSMG4	18.01879258	760
chr6.fa	3269207	3456793	SLC22A23	10.31538791	3040
chr6.fa	3722836	3752246	C6orf145	8.473989762	836
chr6.fa	3849632	3851551	FAM50B	4.178506027	304
chr6.fa	4021569	4065217	PRPF4B	11.06907129	3724
chr6.fa	4068601	4079391	C6orf146	0	0
chr6.fa	4079440	4130999	C6orf201	0	0
chr6.fa	4115927	4135831	PECI	43.3002107	2757
chr6.fa	4706393	4955778	CDYL	13.11484685	2432
chr6.fa	4995280	5004271	RPP40	5.833318062	304
chr6.fa	5085720	5087455	PPP1R3G	1.947256308	152

chr6.fa	5108653	5261172	LYRM4	3.393910292	304
chr6.fa	5148467	5148556	MIR3691	0	0
chr6.fa	5261584	5771816	FARS2	9.180748618	760
chr6.fa	5998233	6007633	NRN1	0	0
chr6.fa	6144311	6320924	F13A1	0	0
chr6.fa	6346698	6623059	LY86-AS1	0	0
chr6.fa	6588934	6655216	LY86	0	0
chr6.fa	7107830	7252213	RREB1	26.38225397	10564
chr6.fa	7281288	7313541	SSR1	3.429048083	1520
chr6.fa	7326887	7389942	CAGE1	0	0
chr6.fa	7390062	7418270	RIOK1	3.933653446	456
chr6.fa	7541870	7586946	DSP	58.36564972	25536
chr6.fa	7590432	7612200	SNRNP48	3.240238055	608
chr6.fa	7727011	7881482	BMP6	0.643599789	76
chr6.fa	7910046	7911047	TXNDC5	2.98626744	76
chr6.fa	7986335	7990577	PIP5K1P1	0	0
chr6.fa	8054503	8054605	MUTED	0	0
chr6.fa	8073593	8080263	EEF1E1	6.584110353	233
chr6.fa	8086641	8086766	SCARNA27	0	0
chr6.fa	8413301	8435794	SLC35B3	9.156730381	912
chr6.fa	8652442	8654080	HULC	0	0
chr6.fa	10396916	10419797	TFAP2A	4.843677751	891
chr6.fa	10414914	10416402	LOC100130275	1.4786783	76
chr6.fa	10428018	10435055	C6orf218	0	0
chr6.fa	10521568	10629601	GCNT2	5.322930531	1748
chr6.fa	10671651	10695030	C6orf52	0	0
chr6.fa	10695188	10709970	PAK1IP1	63.90585634	4296
chr6.fa	10723148	10731362	TMEM14C	65.19972765	3480
chr6.fa	10747995	10757214	TMEM14B	62.48455494	2731
chr6.fa	10762956	10831110	MAK	0	0
chr6.fa	10873456	10882098	GCM2	0	0
chr6.fa	10887064	10974541	SYCP2L	0	0
chr6.fa	10980993	11044624	ELOVL2	2.489890546	456
chr6.fa	11094266	11138969	LOC221710	2.882410805	608
chr6.fa	11102722	11112071	ERVFRDE1	0	0
chr6.fa	11183531	11382581	NEDD9	12.71254138	3192
chr6.fa	11538511	11583757	TMEM170B	0.808391581	304
chr6.fa	11713888	11779280	C6orf105	3.638762872	304
chr6.fa	12012724	12165232	HIVEP1	10.28492034	4104
chr6.fa	12290529	12297427	EDN1	0.804833323	76
chr6.fa	12717833	13287528	PHACTR1	6.288997388	608
chr6.fa	13305184	13328787	TBC1D7	19.51704129	1140
chr6.fa	13363819	13487787	GFOD1	0.564428564	76

chr6.fa	13469509	13486415	C6orf114	0	0
chr6.fa	13574792	13614785	SIRT5	6.444893536	1520
chr6.fa	13615559	13621127	NOL7	42.69708609	1678
chr6.fa	13621730	13711796	RANBP9	34.64808568	4850
chr6.fa	13791020	13814789	CCDC90A	34.72481061	1919
chr6.fa	13924677	13980240	RNF182	0.445226945	76
chr6.fa	14117865	14137148	CD83	0.682295837	76
chr6.fa	15246527	15522253	JARID2	9.690024194	2508
chr6.fa	15523032	15663289	DTNBP1	5.944513603	672
chr6.fa	16129317	16148478	MYLIP	26.96780969	3724
chr6.fa	16238811	16295780	GMPR	74.58552085	5081
chr6.fa	16299343	16761721	ATXN1	5.218851505	2496
chr6.fa	17102489	17131603	FLJ23152	0	0
chr6.fa	17281809	17294099	RBM24	0	0
chr6.fa	17393736	17558023	CAP2	2.233473629	304
chr6.fa	17600518	17611950	FAM8A1	3.918308461	836
chr6.fa	17615269	17706818	NUP153	21.6564435	5538
chr6.fa	17760585	17987799	KIF13A	9.413147298	2736
chr6.fa	18120718	18122851	NHLRC1	3.168183344	304
chr6.fa	18128545	18155374	TPMT	10.4105713	1520
chr6.fa	18155619	18224084	KDM1B	6.404863141	1116
chr6.fa	18224400	18264799	DEK	18.24985691	2356
chr6.fa	18387581	18469105	RNF144B	18.4546791	4179
chr6.fa	18572015	18572111	MIR548A1	0	0
chr6.fa	19837617	19840915	ID4	25.2371623	2660
chr6.fa	20100935	20212670	MBOAT1	3.096573416	456
chr6.fa	20402137	20493945	E2F3	5.736355551	1292
chr6.fa	20534688	21232634	CDKAL1	6.64927094	978
chr6.fa	21593972	21598849	SOX4	38.8067989	8512
chr6.fa	21666675	22194616	FLJ22536	0	0
chr6.fa	22134831	22147422	LOC729177	0	0
chr6.fa	22287477	22303082	PRL	0	0
chr6.fa	22569678	22570750	HDGFL1	1.575196029	76
chr6.fa	24126414	24147757	NRSN1	0	0
chr6.fa	24171983	24383520	DCDC2	7.785022192	1479
chr6.fa	24357131	24358512	KAAG1	0	0
chr6.fa	24403153	24425816	MRS2	5.048277545	760
chr6.fa	24428405	24489799	GPLD1	0.426990876	76
chr6.fa	24495197	24537435	ALDH5A1	14.38447753	3344
chr6.fa	24544332	24646383	KIAA0319	0.72388297	228
chr6.fa	24650205	24667115	TDP2	85.16711089	7372
chr6.fa	24667263	24705089	ACOT13	16.14692685	3040
chr6.fa	24705296	24719403	C6orf62	84.28488547	8588

chr6.fa	24775164	24786278	GMNN	23.64840041	1292
chr6.fa	24804513	24911195	FAM65B	0.276654505	76
chr6.fa	25081295	25138620	CMAH	0	0
chr6.fa	25279656	25620758	LRRC16A	9.913527231	2421
chr6.fa	25652429	25702008	SCGN	0	0
chr6.fa	25726291	25726790	HIST1H2AA	0	0
chr6.fa	25727137	25727573	HIST1H2BA	0	0
chr6.fa	25754927	25781403	SLC17A4	0	0
chr6.fa	25783126	25832287	SLC17A1	6.419540953	532
chr6.fa	25845328	25874471	SLC17A3	1.86964182	152
chr6.fa	25912984	25930946	SLC17A2	0	0
chr6.fa	25963071	25985352	TRIM38	11.37107838	1672
chr6.fa	26017260	26018040	HIST1H1A	0	0
chr6.fa	26020718	26021186	HIST1H3A	3.603847472	76
chr6.fa	26021907	26022278	HIST1H4A	0	0
chr6.fa	26027124	26027480	HIST1H4B	227.250104	3648
chr6.fa	26031817	26032288	HIST1H3B	3.580941191	76
chr6.fa	26033320	26033796	HIST1H2AB	7.086714197	152
chr6.fa	26043455	26043885	HIST1H2BB	0	0
chr6.fa	26045639	26046097	HIST1H3C	18.41153523	380
chr6.fa	26055968	26056699	HIST1H1C	212.4259593	6992
chr6.fa	26087509	26095469	HFE	0	0
chr6.fa	26104176	26104565	HIST1H4C	138.6808544	2432
chr6.fa	26107640	26108364	HIST1H1T	0	0
chr6.fa	26123695	26124132	HIST1H2BC	189.0831243	3724
chr6.fa	26124373	26124918	HIST1H2AC	241.4531104	5928
chr6.fa	26156559	26157343	HIST1H1E	122.725851	4332
chr6.fa	26158349	26171576	HIST1H2BD	118.707689	4788
chr6.fa	26184024	26184458	HIST1H2BE	3.885394581	76
chr6.fa	26188938	26189304	HIST1H4D	87.50199485	1444
chr6.fa	26197012	26197497	HIST1H3D	3.477751729	76
chr6.fa	26199012	26199471	HIST1H2AD	20.61187259	76
chr6.fa	26199787	26200216	HIST1H2BF	11.7918423	228
chr6.fa	26204873	26205249	HIST1H4E	304.8585868	5168
chr6.fa	26216428	26216872	HIST1H2BG	79.76100609	1596
chr6.fa	26217148	26217711	HIST1H2AE	47.9481843	1216
chr6.fa	26225383	26225844	HIST1H3E	3.658333287	76
chr6.fa	26234440	26235216	HIST1H1D	2.175207166	76
chr6.fa	26240654	26241021	HIST1H4F	13.77868422	228
chr6.fa	26246839	26247205	HIST1H4G	0	0
chr6.fa	26250370	26250835	HIST1H3F	7.25395229	152
chr6.fa	26251879	26252303	HIST1H2BH	7.953817023	152
chr6.fa	26271146	26271612	HIST1H3G	0	0

chr6.fa	26273204	26273640	HIST1H2BI	3.867603295	76
chr6.fa	26285354	26285727	HIST1H4H	153.6519996	2584
chr6.fa	26365398	26378548	BTN3A2	2.46809622	456
chr6.fa	26383324	26395100	BTN2A2	0	0
chr6.fa	26402465	26415444	BTN3A1	2.663800372	502
chr6.fa	26421619	26430816	BTN2A3	0	0
chr6.fa	26440700	26453643	BTN3A3	3.378120525	456
chr6.fa	26458153	26476849	BTN2A1	2.315313547	380
chr6.fa	26501495	26510652	BTN1A1	0	0
chr6.fa	26521934	26527612	HCG11	0.892900191	228
chr6.fa	26538572	26547164	HMGN4	31.77657204	2812
chr6.fa	26597180	26600277	ABT1	8.99816554	912
chr6.fa	26634611	26659963	ZNF322A	2.817027827	608
chr6.fa	26839266	26924333	GUSBL1	0	0
chr6.fa	26924772	26991753	NCRNA00240	0	0
chr6.fa	26987145	26988085	LOC100270746	0	0
chr6.fa	27100095	27100575	HIST1H2BJ	7.02778056	152
chr6.fa	27100817	27101314	HIST1H2AG	0	0
chr6.fa	27106072	27114637	HIST1H2BK	68.49333957	2584
chr6.fa	27107088	27107457	HIST1H4I	0	0
chr6.fa	27114908	27115346	HIST1H2AH	0	0
chr6.fa	27115405	27115467	MIR3143	0	0
chr6.fa	27215502	27224399	PRSS16	8.259159977	1064
chr6.fa	27276842	27280011	POM121L2	0	0
chr6.fa	27292578	27293741	FKSG83	0	0
chr6.fa	27325602	27343153	ZNF204P	0	0
chr6.fa	27356524	27369227	ZNF391	0	0
chr6.fa	27418521	27440897	ZNF184	1.634574448	228
chr6.fa	27775257	27775709	HIST1H2BL	7.462110342	152
chr6.fa	27775977	27776445	HIST1H2AI	0	0
chr6.fa	27777842	27778314	HIST1H3H	0	0
chr6.fa	27782080	27782518	HIST1H2AJ	3.850034399	76
chr6.fa	27782822	27783267	HIST1H2BM	0	0
chr6.fa	27791903	27792258	HIST1H4J	4.747604804	76
chr6.fa	27798952	27799305	HIST1H4K	4.774514124	76
chr6.fa	27805658	27806117	HIST1H2AK	0	0
chr6.fa	27806440	27806888	HIST1H2BN	3.764413833	76
chr6.fa	27833107	27833576	HIST1H2AL	3.596063785	76
chr6.fa	27834570	27835359	HIST1H1B	2.139402202	76
chr6.fa	27839623	27840099	HIST1H3I	10.6300713	228
chr6.fa	27840926	27841289	HIST1H4L	9.286606773	152
chr6.fa	27858093	27858570	HIST1H3J	0	0
chr6.fa	27860477	27860963	HIST1H2AM	0	0

chr6.fa	27861203	27861669	HIST1H2BO	0	0
chr6.fa	27878963	27880174	OR2B2	0	0
chr6.fa	27925019	27925960	OR2B6	0	0
chr6.fa	28048482	28057340	ZNF165	1.422190965	152
chr6.fa	28058929	28063493	ZSCAN12P1	0	0
chr6.fa	28092387	28097856	ZSCAN16	10.6049411	608
chr6.fa	28109716	28125236	ZNF192	5.043384942	1216
chr6.fa	28129551	28137376	ZNF389	1.030560271	76
chr6.fa	28183116	28186707	LOC222699	0	0
chr6.fa	28193029	28201264	ZNF193	1.842065326	152
chr6.fa	28212490	28220002	ZKSCAN4	5.914935589	608
chr6.fa	28227098	28228736	NKAPL	1.031227444	76
chr6.fa	28234788	28245980	ZNF187	3.601178779	456
chr6.fa	28249314	28270326	PGBD1	0.539075981	76
chr6.fa	28292515	28324048	ZNF323	1.549621054	304
chr6.fa	28317691	28334524	ZKSCAN3	4.418688394	456
chr6.fa	28346598	28367544	ZSCAN12	3.386571386	912
chr6.fa	28400432	28411279	ZSCAN23	0.534628159	76
chr6.fa	28471073	28483570	GPX6	0	0
chr6.fa	28493789	28502728	GPX5	0	0
chr6.fa	28539407	28555112	SCAND3	3.119034915	684
chr6.fa	28827402	28831454	LOC401242	0	0
chr6.fa	28870779	28891768	TRIM27	21.77475555	2907
chr6.fa	28962594	28973037	ZNF311	1.879427028	228
chr6.fa	29004000	29044517	LOC100129636	0	0
chr6.fa	29011990	29012952	OR2W1	0	0
chr6.fa	29053985	29055090	OR2B3	0	0
chr6.fa	29079668	29080603	OR2J3	0	0
chr6.fa	29141311	29142351	OR2J2	0	0
chr6.fa	29274467	29275432	OR14J1	0	0
chr6.fa	29323007	29324054	OR5V1	0	0
chr6.fa	29341200	29343068	OR12D3	0	0
chr6.fa	29364416	29365448	OR12D2	0	0
chr6.fa	29393281	29395509	OR11A1	0	0
chr6.fa	29407793	29408731	OR10C1	0	0
chr6.fa	29426230	29432099	OR2H1	0	0
chr6.fa	29454543	29455679	MAS1L	0	0
chr6.fa	29497183	29501345	LOC100507362	0	0
chr6.fa	29523389	29527702	UBD	8.536259265	380
chr6.fa	29550029	29550105	SNORD32B	0	0
chr6.fa	29555683	29556745	OR2H2	0	0
chr6.fa	29570005	29600962	GABBR1	0.372505061	76
chr6.fa	29624758	29640149	MOG	0	0

chr6.fa	29640169	29644931	ZFP57	0	0
chr6.fa	29691117	29695073	HLA-F	1.172890563	76
chr6.fa	29694378	29716826	LOC285830	0	0
chr6.fa	29718584	29718925	IFITM4P	0	0
chr6.fa	29758808	29760850	HCG4	0.827294822	76
chr6.fa	29794756	29798899	HLA-G	0	0
chr6.fa	29855383	29858856	HLA-H	1.838062287	152
chr6.fa	29866808	29870431	HCG2P7	0	0
chr6.fa	29892369	29893428	HCG4P6	1.594544053	76
chr6.fa	29910309	29913661	HLA-A	11.94506976	832
chr6.fa	29942892	29946177	HCG9	0	0
chr6.fa	29968788	30028961	ZNRD1-AS1	0	0
chr6.fa	29973748	29977733	HLA-J	0	0
chr6.fa	30029036	30032686	ZNRD1	22.74793892	760
chr6.fa	30034932	30038042	PPP1R11	52.9502045	3800
chr6.fa	30038109	30043626	RNF39	0.887785197	76
chr6.fa	30070674	30080867	TRIM31	0.833744164	76
chr6.fa	30104510	30116512	TRIM40	0	0
chr6.fa	30119723	30128711	TRIM10	0	0
chr6.fa	30130983	30140473	TRIM15	0.600011137	60
chr6.fa	30152232	30181153	TRIM26	21.80544552	3420
chr6.fa	30227339	30234728	HLA-L	0	0
chr6.fa	30255174	30294927	HCG18	3.805556183	1140
chr6.fa	30295008	30308138	TRIM39	4.767842392	152
chr6.fa	30312906	30314341	RPP21	0	0
chr6.fa	30457183	30461982	HLA-E	16.43292178	1967
chr6.fa	30509155	30525371	GNL1	6.367279049	2052
chr6.fa	30525927	30532473	PRR3	2.539706148	304
chr6.fa	30539170	30559309	ABCF1	25.82160606	4022
chr6.fa	30552109	30552194	MIR877	0	0
chr6.fa	30568182	30585020	PPP1R10	32.15552645	6534
chr6.fa	30585486	30594174	MRPS18B	25.34146371	1748
chr6.fa	30594613	30614598	ATAT1	2.360903719	348
chr6.fa	30614816	30620895	C6orf136	10.06052774	608
chr6.fa	30620988	30640830	DHX16	15.45306668	2341
chr6.fa	30644166	30655672	KIAA1949	0.735002524	152
chr6.fa	30655826	30658769	NRM	0	0
chr6.fa	30667584	30685458	MDC1	3.211549605	1064
chr6.fa	30688157	30693195	TUBB	65.34695055	7296
chr6.fa	30695511	30710453	FLOT1	52.31661231	4305
chr6.fa	30710976	30712327	IER3	5.452139749	304
chr6.fa	30851861	30867933	DDR1	5.985878344	1064
chr6.fa	30875977	30881880	GTF2H4	5.909598203	456

chr6.fa	30881982	30894235	VARS2	4.52276742	760
chr6.fa	30899127	30899952	SFTA2	3.722826701	76
chr6.fa	30908777	30921998	DPCR1	0	0
chr6.fa	30951485	30957675	MUC21	1.386830783	228
chr6.fa	30973729	31003179	PBMUCL1	0	0
chr6.fa	31021984	31027653	HCG22	0	0
chr6.fa	31079000	31080332	C6orf15	0	0
chr6.fa	31082608	31107869	PSORS1C1	0	0
chr6.fa	31082865	31088252	CDSN	0	0
chr6.fa	31105311	31107127	PSORS1C2	0	0
chr6.fa	31110216	31126015	CCHCR1	5.205952822	684
chr6.fa	31126303	31131992	TCF19	2.556163088	380
chr6.fa	31132114	31138451	POU5F1	0	0
chr6.fa	31141512	31145676	PSORS1C3	0	0
chr6.fa	31165537	31171745	HCG27	0	0
chr6.fa	31236529	31239855	HLA-C	45.3824584	3112
chr6.fa	31321649	31324989	HLA-B	16.87748155	1193
chr6.fa	31367561	31383036	MICA	2.919105333	304
chr6.fa	31430957	31433586	HCP5	11.98220907	1368
chr6.fa	31439006	31440185	HCG26	0	0
chr6.fa	31465855	31478901	MICB	0	0
chr6.fa	31496739	31497995	MCCD1	0	0
chr6.fa	31498009	31510225	BAT1	49.90945125	4466
chr6.fa	31504151	31504226	SNORD117	0	0
chr6.fa	31508878	31508955	SNORD84	0	0
chr6.fa	31512240	31514627	ATP6V1G2	0	0
chr6.fa	31514628	31526606	NFKBIL1	8.419281556	608
chr6.fa	31539876	31542098	LTA	0	0
chr6.fa	31543350	31546112	TNF	0	0
chr6.fa	31548336	31550202	LTB	0	0
chr6.fa	31553956	31556659	LST1	0	0
chr6.fa	31556687	31560762	NCR3	0	0
chr6.fa	31582994	31584798	AIF1	0	0
chr6.fa	31588450	31605554	PRRC2A	43.0384564	13452
chr6.fa	31590856	31590987	SNORA38	0	0
chr6.fa	31606805	31620477	BAG6	43.50169702	7752
chr6.fa	31623671	31625987	APOM	15.54691571	532
chr6.fa	31626075	31628549	C6orf47	5.463259303	608
chr6.fa	31629006	31634060	GPANK1	5.874238021	836
chr6.fa	31634598	31637843	CSNK2B	122.9691469	4418
chr6.fa	31638728	31640227	LY6G5B	0	0
chr6.fa	31644461	31648150	LY6G5C	4.656202069	152
chr6.fa	31654726	31671137	ABHD16A	2.724957919	304

chr6.fa	31674684	31678372	LY6G6F	0	0
chr6.fa	31679753	31681842	LY6G6E	0	0
chr6.fa	31683133	31685581	LY6G6D	0	0
chr6.fa	31686425	31689510	LY6G6C	0	0
chr6.fa	31691161	31692851	C6orf25	0	0
chr6.fa	31694817	31698039	DDAH2	59.68309449	3572
chr6.fa	31698358	31704341	CLIC1	205.3605947	11552
chr6.fa	31707725	31730455	MSH5	2.46387079	304
chr6.fa	31730773	31732624	C6orf26	0	0
chr6.fa	31733371	31745108	C6orf27	0.57999594	76
chr6.fa	31745297	31763712	VARS	15.7517379	3040
chr6.fa	31765174	31774743	LSM2	24.73033302	953
chr6.fa	31777396	31782835	HSPA1L	1.332344968	152
chr6.fa	31783291	31785719	HSPA1A	17.39565277	1900
chr6.fa	31795512	31798031	HSPA1B	31.52304621	3572
chr6.fa	31802693	31807541	C6orf48	141.9130864	5373
chr6.fa	31803040	31803103	SNORD48	0	0
chr6.fa	31804853	31804916	SNORD52	0	0
chr6.fa	31826829	31830709	NEU1	4.080653951	380
chr6.fa	31830970	31846823	SLC44A4	40.56902583	5002
chr6.fa	31847537	31865464	EHMT2	19.17255751	3420
chr6.fa	31867394	31869769	ZBTB12	0.980299886	76
chr6.fa	31895266	31913449	C2	0.595563316	76
chr6.fa	31913721	31919861	CFB	1.286754796	152
chr6.fa	31919864	31926231	RDBP	25.25317445	1550
chr6.fa	31924616	31924717	MIR1236	0	0
chr6.fa	31926581	31937532	SKIV2L	9.782094102	1748
chr6.fa	31937588	31939458	DOM3Z	6.691080463	380
chr6.fa	31938952	31981961	STK19	6.15578513	532
chr6.fa	31973348	32009419	CYP21A2	0	0
chr6.fa	31978762	32077151	TNXB	0	0
chr6.fa	32083045	32096017	ATF6B	5.814859603	684
chr6.fa	32096484	32098067	FKBPL	7.545506997	456
chr6.fa	32116140	32119720	PRRT1	0	0
chr6.fa	32120579	32122142	LOC100507547	1.737096736	76
chr6.fa	32121622	32131452	PPT2	2.529031377	228
chr6.fa	32132405	32135982	EGFL8	1.421523792	76
chr6.fa	32136063	32145888	AGPAT1	16.99045622	1748
chr6.fa	32146162	32146231	RNF5	72.43588865	228
chr6.fa	32148746	32152023	AGER	1.172000998	76
chr6.fa	32152510	32157963	PBX2	11.57300948	1672
chr6.fa	32158543	32162606	GPSM3	6.222947237	380
chr6.fa	32162620	32191844	NOTCH4	0	0

chr6.fa	32260475	32339656	C6orf10	0	0
chr6.fa	32362513	32374900	BTNL2	0	0
chr6.fa	32407619	32412826	HLA-DRA	5.26933428	304
chr6.fa	32485154	32498006	HLA-DRB5	0	0
chr6.fa	32520490	32527779	HLA-DRB6	0	0
chr6.fa	32546547	32557562	HLA-DRB1	7.25395229	380
chr6.fa	32605183	32611429	HLA-DQA1	0	0
chr6.fa	32627657	32634466	HLA-DQB1	0	0
chr6.fa	32709163	32714664	HLA-DQA2	0	0
chr6.fa	32723875	32731330	HLA-DQB2	0	0
chr6.fa	32780540	32784825	HLA-DOB	0	0
chr6.fa	32789610	32806547	TAP2	0.274208203	76
chr6.fa	32808494	32812712	PSMB8	31.68383496	2204
chr6.fa	32811863	32814277	LOC100507463	2.5359255	152
chr6.fa	32812986	32821748	TAP1	4.740043507	532
chr6.fa	32821938	32827628	PSMB9	12.90201858	608
chr6.fa	32844255	32847851	PPP1R2P1	0	0
chr6.fa	32861953	32871535	LOC100294145	5.648066292	912
chr6.fa	32902406	32908847	HLA-DMB	1.209807482	76
chr6.fa	32916391	32920899	HLA-DMA	0	0
chr6.fa	32936437	32949282	BRD2	79.853076	19228
chr6.fa	32971960	32977389	HLA-DOA	0	0
chr6.fa	33032794	33041378	HLA-DPA1	5.843325661	304
chr6.fa	33043760	33054976	HLA-DPB1	17.61648711	1189
chr6.fa	33080293	33096890	HLA-DPB2	4.199188397	152
chr6.fa	33130469	33160245	COL11A2	0	0
chr6.fa	33161365	33168432	RXRB	14.10915737	1824
chr6.fa	33168603	33172214	SLC39A7	4.132471073	456
chr6.fa	33172419	33174607	HSD17B8	11.310588	503
chr6.fa	33175612	33175721	MIR219-1	0	0
chr6.fa	33176286	33180499	RING1	17.5046244	1368
chr6.fa	33218049	33239662	VPS52	20.03854838	2659
chr6.fa	33239852	33244281	RPS18	2070.616863	50464
chr6.fa	33244917	33246602	B3GALT4	2.004855598	152
chr6.fa	33246880	33257304	WDR46	7.152764348	760
chr6.fa	33257378	33258711	PFDN6	23.18471501	760
chr6.fa	33259431	33267176	RGL2	26.71606299	4028
chr6.fa	33267472	33282164	TAPBP	9.113586512	1672
chr6.fa	33282182	33285719	ZBTB22	13.00609761	1596
chr6.fa	33286335	33290793	DAXX	20.57940349	2356
chr6.fa	33332514	33334139	LYPLA2P1	0	0
chr6.fa	33359313	33377699	KIFC1	0	0
chr6.fa	33378773	33384230	PHF1	15.46552058	1596

chr6.fa	33384319	33386065	CUTA	57.98202511	2675
chr6.fa	33387847	33421466	SYNGAP1	0.562427045	152
chr6.fa	33422356	33425320	ZBTB9	4.364202579	532
chr6.fa	33540323	33548070	BAK1	6.936377826	684
chr6.fa	33551476	33556803	GGNBP1	0	0
chr6.fa	33553883	33561115	C6orf227	0	0
chr6.fa	33589156	33664348	ITPR3	5.240201049	2128
chr6.fa	33665346	33679504	C6orf125	46.7637294	1064
chr6.fa	33689443	33714762	IP6K3	0	0
chr6.fa	33738990	33756906	LEMD2	4.350414332	608
chr6.fa	33762449	33771793	MLN	0	0
chr6.fa	33967749	33967828	MIR1275	0	0
chr6.fa	33989628	34101443	GRM4	0	0
chr6.fa	34204577	34214008	HMGA1	14.08469435	1520
chr6.fa	34214157	34216904	C6orf1	7.007320581	380
chr6.fa	34255997	34360441	NUDT3	49.07704143	2988
chr6.fa	34385231	34393876	RPS10	278.9604783	7752
chr6.fa	34433853	34504039	PACSIN1	2.142515677	532
chr6.fa	34505580	34524091	SPDEF	125.8024093	10714
chr6.fa	34555066	34664625	C6orf106	46.29025879	9196
chr6.fa	34724871	34741634	SNRPC	20.81380369	1139
chr6.fa	34759794	34845291	UHRF1BP1	7.193684307	3116
chr6.fa	34845555	34855819	TAF11	15.43527539	1064
chr6.fa	34857038	35059190	ANKS1A	32.20133901	9196
chr6.fa	35085849	35109187	TCP11	0	0
chr6.fa	35182190	35218609	SCUBE3	0	0
chr6.fa	35227510	35263760	ZNF76	13.37860267	1596
chr6.fa	35265595	35289548	DEF6	6.625252703	684
chr6.fa	35310335	35395968	PPARD	6.157341868	1064
chr6.fa	35420138	35434881	FANCE	1.323004543	152
chr6.fa	35436178	35438558	RPL10A	1137.751438	36784
chr6.fa	35441374	35464861	TEAD3	14.12717105	1900
chr6.fa	35465651	35480647	TULP1	0	0
chr6.fa	35541362	35688325	FKBP5	16.17783921	7676
chr6.fa	35694539	35704724	LOC285847	0	0
chr6.fa	35704859	35716685	C6orf81	0	0
chr6.fa	35744392	35747329	C6orf126	0	0
chr6.fa	35748831	35755841	C6orf127	0	0
chr6.fa	35762760	35765102	CLPS	0	0
chr6.fa	35773071	35791852	LHFPL5	0	0
chr6.fa	35800811	35888957	SRPK1	16.27391216	3192
chr6.fa	35911291	35992413	SLC26A8	0	0
chr6.fa	35995454	36079013	MAPK14	14.78433669	2947

chr6.fa	36098262	36107842	MAPK13	29.11810906	2472
chr6.fa	36164550	36200567	BRPF3	12.62781038	3420
chr6.fa	36210945	36276372	PNPLA1	0.650271522	76
chr6.fa	36283535	36304662	C6orf222	0	0
chr6.fa	36333971	36355467	ETV7	0	0
chr6.fa	36358329	36410543	PXT1	0	0
chr6.fa	36410667	36458315	KCTD20	15.89606971	3496
chr6.fa	36461669	36515247	STK38	28.80008981	4631
chr6.fa	36562090	36572244	SRSF3	32.17865512	5209
chr6.fa	36590213	36590289	MIR3925	0	0
chr6.fa	36644237	36655116	CDKN1A	35.25187747	4180
chr6.fa	36708555	36807220	CPNE5	0	0
chr6.fa	36822606	36842800	PPIL1	14.60397753	1140
chr6.fa	36853640	36896740	C6orf89	3.755295799	1140
chr6.fa	36916039	36932613	PI16	0	0
chr6.fa	36935917	36953949	MTCH1	121.1268592	10245
chr6.fa	36973423	36996845	FGD2	1.110176278	152
chr6.fa	37137922	37143204	PIM1	13.72597754	1672
chr6.fa	37179954	37225931	TMEM217	0	0
chr6.fa	37237370	37300746	TBC1D22B	9.332196945	1368
chr6.fa	37321748	37362514	RNF8	4.502307441	1140
chr6.fa	37400907	37449284	FTSJD2	9.426268372	1748
chr6.fa	37450697	37467700	C6orf129	27.26092114	760
chr6.fa	37600284	37665766	MDGA1	0.189699592	76
chr6.fa	37787307	38122399	ZFAND3	36.97185009	5541
chr6.fa	38136227	38607924	BTBD9	7.424081467	3116
chr6.fa	38643702	38670952	GLO1	140.2389263	12902
chr6.fa	38690552	38998567	DNAH8	0.350265953	228
chr6.fa	39016557	39055520	GLP1R	0	0
chr6.fa	39071840	39082865	C6orf64	7.922682271	684
chr6.fa	39156747	39197251	KCNK5	4.467836823	760
chr6.fa	39266777	39282236	KCNK17	0	0
chr6.fa	39282474	39290330	KCNK16	0	0
chr6.fa	39302876	39693181	KIF6	0	0
chr6.fa	39760793	39872033	DAAM2	0.301339915	76
chr6.fa	39872642	39902290	MOCS1	6.187587055	988
chr6.fa	40346163	40347631	TDRG1	0	0
chr6.fa	40359373	40555126	LRFN2	0	0
chr6.fa	40994640	41006938	UNC5CL	0.540410328	76
chr6.fa	41010237	41012076	TSPO2	0	0
chr6.fa	41020940	41032630	APOBEC2	0	0
chr6.fa	41034531	41040188	C6orf130	27.35365822	1444
chr6.fa	41040707	41070146	NFYA	8.494672132	2280

chr6.fa	41072849	41108573	LOC221442	1.956596733	380
chr6.fa	41117342	41122070	TREML1	0	0
chr6.fa	41126246	41130922	TREM2	0	0
chr6.fa	41157552	41168925	TREML2	0	0
chr6.fa	41176292	41185685	TREML3	0	0
chr6.fa	41196062	41206120	TREML4	0	0
chr6.fa	41217115	41217327	TREML2P1	0	0
chr6.fa	41243712	41254457	TREM1	0	0
chr6.fa	41303528	41318625	NCR2	0	0
chr6.fa	41514164	41570122	FOXP4	14.48966851	3876
chr6.fa	41606195	41621982	MDFI	4.180952328	304
chr6.fa	41651716	41703997	TFEB	3.424155479	380
chr6.fa	41704449	41715139	PGC	2.796790238	228
chr6.fa	41737914	41747630	FRS3	0.780370304	76
chr6.fa	41748500	41755110	PRICKLE4	4.107340881	304
chr6.fa	41755181	41757634	TOMM6	118.747497	4560
chr6.fa	41765383	41863099	USP49	0	0
chr6.fa	41873092	41888877	MED20	7.50280791	836
chr6.fa	41888965	41900784	BYSL	14.43874095	1292
chr6.fa	41902671	42016610	CCND3	22.32161522	2432
chr6.fa	42018251	42048644	TAF8	8.992383372	1748
chr6.fa	42068857	42110715	C6orf132	41.86578823	12692
chr6.fa	42123144	42147794	GUCA1A	0	0
chr6.fa	42151022	42162694	GUCA1B	0	0
chr6.fa	42174539	42185633	MRPS10	15.00628299	1444
chr6.fa	42192669	42419783	TRERF1	11.37196794	3876
chr6.fa	42531760	42661243	UBR2	13.85029415	5531
chr6.fa	42664333	42690358	PRPH2	0	0
chr6.fa	42695314	42695932	LOC442211	0	0
chr6.fa	42712234	42713884	TBCC	17.40343646	1292
chr6.fa	42788794	42836296	KIAA0240	9.800330171	2871
chr6.fa	42847671	42854731	RPL7L1	22.53555544	1292
chr6.fa	42858003	42858554	C6orf226	6.123760815	152
chr6.fa	42883727	42893575	PTCRA	0	0
chr6.fa	42896860	42907008	CNPY3	7.660927969	608
chr6.fa	42928500	42931610	GNMT	84.50861089	4028
chr6.fa	42931619	42946981	PEX6	17.43434882	2736
chr6.fa	42952330	42979966	PPP2R5D	19.7287576	2452
chr6.fa	42980081	42981618	MEA1	51.69413967	1748
chr6.fa	42981977	42989031	KLHDC3	61.18712537	5167
chr6.fa	42989385	42997337	RRP36	26.2494865	1368
chr6.fa	43005355	43021683	CUL7	13.89343802	3496
chr6.fa	43021767	43027242	MRPL2	17.0856396	988

chr6.fa	43027372	43042833	KLC4	7.655812974	988
chr6.fa	43044029	43129457	PTK7	0.795492898	152
chr6.fa	43138920	43149244	SRF	9.394244056	1824
chr6.fa	43149922	43192325	CUL9	6.307455848	2204
chr6.fa	43193367	43197211	C6orf108	75.77131009	2201
chr6.fa	43211222	43255997	TTBK1	0	0
chr6.fa	43265998	43273210	SLC22A7	5.423673691	608
chr6.fa	43273277	43276530	CRIP3	0	0
chr6.fa	43303808	43337181	ZNF318	6.966845404	2508
chr6.fa	43395292	43418089	ABCC10	2.508571397	608
chr6.fa	43418164	43423786	DLK2	0	0
chr6.fa	43445261	43474294	TJAP1	6.578772967	912
chr6.fa	43474707	43478424	C6orf154	3.187531368	304
chr6.fa	43479565	43484728	YIPF3	14.95780174	1060
chr6.fa	43484791	43497114	POLR1C	22.39411471	1444
chr6.fa	43490068	43543812	XPO5	13.86830783	3345
chr6.fa	43543878	43588217	POLH	2.423395613	912
chr6.fa	43588261	43596936	GTPBP2	14.18721664	1873
chr6.fa	43597279	43608688	MAD2L1BP	10.37543351	760
chr6.fa	43612767	43638748	RSPH9	0	0
chr6.fa	43638934	43655549	MRPS18A	28.1122342	1503
chr6.fa	43737946	43754223	VEGFA	18.61724698	3007
chr6.fa	43858765	43905944	LOC100132354	0	0
chr6.fa	43968337	43973694	C6orf223	1.399507075	228
chr6.fa	44081373	44095191	MRPL14	21.83680266	760
chr6.fa	44095376	44123256	TMEM63B	7.925128573	1140
chr6.fa	44126548	44152139	CAPN11	0	0
chr6.fa	44187242	44201888	SLC29A1	2.98626744	380
chr6.fa	44214849	44221614	HSP90AB1	463.4988184	52896
chr6.fa	44221838	44225283	SLC35B2	2.485442725	228
chr6.fa	44225903	44233525	NFKBIE	3.27426389	380
chr6.fa	44238480	44247182	TMEM151B	0	0
chr6.fa	44247897	44265458	TCTE1	0	0
chr6.fa	44266463	44281063	AARS2	3.534461455	760
chr6.fa	44310397	44344904	SPATS1	0	0
chr6.fa	44355302	44414780	CDC5L	31.54995553	4256
chr6.fa	44796470	45345575	SUPT3H	6.633925955	684
chr6.fa	45165411	45165507	MIR586	0	0
chr6.fa	45296054	45518819	RUNX2	3.114809485	836
chr6.fa	45866190	46048085	CLIC5	2.393595208	684
chr6.fa	46097701	46114436	ENPP4	0.361163116	76
chr6.fa	46127762	46138717	ENPP5	1.148649935	152
chr6.fa	46188469	46293531	RCAN2	7.131637195	1064

chr6.fa	46517445	46620523	CYP39A1	3.719490835	380
chr6.fa	46620679	46645927	SLC25A27	1.142422985	152
chr6.fa	46655612	46672052	TDRD6	0.188365246	76
chr6.fa	46672057	46703430	PLA2G7	4.270353543	380
chr6.fa	46714699	46726942	LOC100287718	0	0
chr6.fa	46761094	46807519	MEP1A	0	0
chr6.fa	46820242	46922675	GPR116	0.561315089	152
chr6.fa	46967813	47010082	GPR110	4.58036671	760
chr6.fa	47199268	47277680	TNFRSF21	24.18525248	3952
chr6.fa	47445525	47594996	CD2AP	41.62071326	10123
chr6.fa	47624326	47665533	GPR111	0	0
chr6.fa	47666289	47689757	GPR115	0	0
chr6.fa	47749775	47794116	OPN5	0	0
chr6.fa	47845764	48036425	C6orf138	0	0
chr6.fa	49398073	49431041	MUT	13.48312648	2356
chr6.fa	49431096	49460820	CENPQ	0.978075976	76
chr6.fa	49467671	49495777	GLYATL3	0	0
chr6.fa	49518113	49519808	C6orf141	0	0
chr6.fa	49572890	49604587	RHAG	0	0
chr6.fa	49660072	49681299	CRISP2	0	0
chr6.fa	49695092	49712150	CRISP3	0.758575978	76
chr6.fa	49753364	49755053	PGK2	0	0
chr6.fa	49801979	49834218	CRISP1	0	0
chr6.fa	49913814	49917157	DEFB133	0	0
chr6.fa	49928005	49931818	DEFB114	0	0
chr6.fa	49936390	49937338	DEFB113	0	0
chr6.fa	49976851	49989694	DEFB110	0	0
chr6.fa	50011288	50016364	DEFB112	0	0
chr6.fa	50681257	50740746	TFAP2D	0	0
chr6.fa	50786439	50815326	TFAP2B	1.171778607	304
chr6.fa	51480145	51952423	PKHD1	5.922941668	4636
chr6.fa	52009147	52009232	MIR206	0	0
chr6.fa	52013721	52013839	MIR133B	0	0
chr6.fa	52051185	52055436	IL17A	0	0
chr6.fa	52101484	52109298	IL17F	0	0
chr6.fa	52128812	52149582	MCM3	29.33760905	4104
chr6.fa	52226926	52272575	PAQR8	6.42109769	1368
chr6.fa	52284994	52360583	EFHC1	1.202246186	380
chr6.fa	52362200	52441862	TRAM2	0.239515195	76
chr6.fa	52529199	52533951	LOC730101	2.853499964	456
chr6.fa	52535884	52551385	TMEM14A	18.33525509	836
chr6.fa	52604261	52609957	GSTA7P	0	0
chr6.fa	52614885	52628361	GSTA2	7.747215708	456

chr6.fa	52656178	52668664	GSTA1	44.30163774	2508
chr6.fa	52696541	52710893	GSTA5	0	0
chr6.fa	52761439	52774496	GSTA3	0	0
chr6.fa	52842746	52860178	GSTA4	22.37187561	1349
chr6.fa	52866098	52926600	ICK	10.56691223	2964
chr6.fa	52929796	52965670	FBXO9	19.19479662	4300
chr6.fa	52991760	53013624	GCM1	0.611575474	76
chr6.fa	53132196	53213942	ELOVL5	21.63909699	2885
chr6.fa	53362140	53409927	GCLC	37.68750459	6460
chr6.fa	53512699	53530506	KLHL31	0	0
chr6.fa	53659778	53788919	LRRC1	15.94499575	2280
chr6.fa	53883714	54131078	C6orf142	0	0
chr6.fa	54173203	54254950	TINAG	13.70840864	1112
chr6.fa	54711569	54806819	FAM83B	8.541596651	1216
chr6.fa	55039071	55147418	HCRTR2	0	0
chr6.fa	55192267	55267291	GFRAL	0	0
chr6.fa	55299171	55444012	HMGCLL1	0	0
chr6.fa	55620238	55740375	BMP5	0	0
chr6.fa	55921388	56112378	COL21A1	0.404974159	76
chr6.fa	56322785	56507694	DST	5.772605297	5766
chr6.fa	56819773	56892142	BEND6	0.626475676	76
chr6.fa	56911384	56920023	KIAA1586	3.501769966	456
chr6.fa	56954828	57035098	ZNF451	10.31961335	2432
chr6.fa	57037104	57050012	BAG2	2.470987304	228
chr6.fa	57053582	57087078	RAB23	13.40551199	1900
chr6.fa	57182422	57513376	PRIM2	45.81345231	4701
chr6.fa	58246159	58287724	GUSBP4	0	0
chr6.fa	62284008	62284534	MTRNR2L9	0	0
chr6.fa	62389865	62996100	KHDRBS2	0.734780133	76
chr6.fa	63985856	64029882	LGSN	0.648492393	152
chr6.fa	64281920	64293489	PTP4A1	21.61774745	4940
chr6.fa	64356431	64424405	PHF3	17.27400485	5396
chr6.fa	64429876	66417118	EYS	0.121870313	76
chr6.fa	66497772	66499376	MCART3P	0	0
chr6.fa	69345632	70099403	BAI3	0	0
chr6.fa	70385641	70507049	LMBRD1	6.590782085	684
chr6.fa	70576448	70922157	COL19A1	0	0
chr6.fa	70925743	71012786	COL9A1	0.826405258	149
chr6.fa	71123107	71270877	FAM135A	22.98656455	6612
chr6.fa	71276625	71298606	C6orf57	28.97444442	684
chr6.fa	71377479	71571716	SMAP1	16.30971712	2052
chr6.fa	71603831	71666788	B3GAT2	0	0
chr6.fa	71998477	72011973	OGFRL1	2.913990338	228

chr6.fa	72086663	72086734	MIR30C2	0	0
chr6.fa	72113254	72113324	MIR30A	0	0
chr6.fa	72124149	72130448	C6orf155	1.728868266	228
chr6.fa	72596650	73112845	RIMS1	4.21831403	1672
chr6.fa	73331571	73908573	KCNQ5	0	0
chr6.fa	73677410	73677476	MIR4282	0	0
chr6.fa	73933268	73935175	KHDC1L	0	0
chr6.fa	73951038	73972907	KHDC1	1.491799373	76
chr6.fa	73983862	74020088	C6orf147	0	0
chr6.fa	74062785	74063999	DPPA5	0	0
chr6.fa	74072400	74073898	C6orf221	0	0
chr6.fa	74078280	74079515	OOEP	0	0
chr6.fa	74104285	74127289	DDX43	0	0
chr6.fa	74134856	74162043	C6orf150	0	0
chr6.fa	74171454	74211179	MTO1	10.29603989	1444
chr6.fa	74225473	74230755	EEF1A1	612.6393925	96748
chr6.fa	74303102	74363737	SLC17A5	5.610037417	836
chr6.fa	74405508	74538041	CD109	0	0
chr6.fa	75794042	75915623	COL12A1	1.021887019	532
chr6.fa	75947391	75953644	COX7A2	127.7716823	4332
chr6.fa	75962638	75994632	TMEM30A	3.347652947	684
chr6.fa	76017800	76203496	FILIP1	0.365610938	76
chr6.fa	76311622	76427994	SENP6	19.84840401	5928
chr6.fa	76458909	76629254	MYO6	28.42669518	11072
chr6.fa	76631062	76782335	IMPG1	0	0
chr6.fa	78171948	78173120	HTR1B	0	0
chr6.fa	79577189	79608320	IRAK1BP1	0	0
chr6.fa	79644136	79788011	PHIP	7.158991298	3852
chr6.fa	79910962	79944455	HMGN3	17.84443797	739
chr6.fa	80194708	80247147	LCA5	3.939880396	836
chr6.fa	80341000	80413369	SH3BGRL2	52.02216652	10868
chr6.fa	80451636	80451669	RNY4	0	0
chr6.fa	80624529	80657315	ELOVL4	0	0
chr6.fa	80714322	80752244	TTK	0	0
chr6.fa	80816344	81055987	BCKDHB	26.17031527	1824
chr6.fa	82455447	82462428	FAM46A	6.324579961	1596
chr6.fa	82879956	82957448	IBTK	18.73088882	4864
chr6.fa	83072923	83077133	TPBG	0.486146904	76
chr6.fa	83602186	83775545	UBE2CBP	5.532422929	456
chr6.fa	83777385	83874438	DOPEY1	6.384847944	2128
chr6.fa	83874593	83903655	PGM3	24.68696676	6384
chr6.fa	83903032	83906256	RWDD2A	22.69478746	1444
chr6.fa	83920110	84140938	ME1	2.88352276	456

chr6.fa	84222194	84235421	PRSS35	0	0
chr6.fa	84262605	84419127	SNAP91	1.429974653	304
chr6.fa	84562985	84567234	RIPPLY2	0	0
chr6.fa	84569370	84670146	CYB5R4	5.265331241	532
chr6.fa	84743420	84800605	MRAP2	0.763690973	76
chr6.fa	84833960	84937335	KIAA1009	4.605274512	1064
chr6.fa	85444157	85473899	TBX18	0	0
chr6.fa	86159302	86205498	NT5E	0.416538495	76
chr6.fa	86215215	86303629	SNX14	1.447098766	228
chr6.fa	86317502	86353043	SYNCRIP	19.87486854	7448
chr6.fa	86386725	86388451	SNHG5	3.333642309	76
chr6.fa	86387012	86387086	SNORD50A	0	0
chr6.fa	86387307	86387377	SNORD50B	0	0
chr6.fa	87647024	87726397	HTR1E	0	0
chr6.fa	87795222	87804824	CGA	2.400711723	76
chr6.fa	87865269	87973406	ZNF292	12.71810116	5776
chr6.fa	87992697	88038996	GJB7	1.482681339	152
chr6.fa	88032306	88052043	C6orf162	1.388832303	152
chr6.fa	88054571	88075181	C6orf163	0	0
chr6.fa	88106842	88109459	C6orf164	0	0
chr6.fa	88117690	88174191	C6orf165	0	0
chr6.fa	88182643	88222057	SLC35A1	0.886673241	76
chr6.fa	88224096	88299735	RARS2	34.07498387	2807
chr6.fa	88299785	88377172	ORC3	8.456420866	978
chr6.fa	88384578	88411985	AKIRIN2	13.12240814	1140
chr6.fa	88410020	88410933	NCRNA00120	7.396727364	304
chr6.fa	88757507	88776550	SPACA1	0	0
chr6.fa	88849585	88875767	CNR1	0	0
chr6.fa	89319989	89673348	RNGTT	3.408365712	684
chr6.fa	89790429	89794879	PNRC1	61.80648453	5928
chr6.fa	89805678	89827800	SRSF12	1.878537464	304
chr6.fa	89855769	89875284	PM20D2	13.2829745	2812
chr6.fa	89887223	89927496	GABRR1	0	0
chr6.fa	89967239	90024967	GABRR2	0	0
chr6.fa	90036344	90062619	UBE2J1	52.93952973	10336
chr6.fa	90074335	90121995	RRAGD	13.03834431	2888
chr6.fa	90142897	90341942	ANKRD6	5.439018675	912
chr6.fa	90343404	90348474	LYRM2	13.48223691	2508
chr6.fa	90353231	90529442	MDN1	4.079764386	3192
chr6.fa	90539619	90584155	CASP8AP2	5.344724857	1584
chr6.fa	90604188	90605819	GJA10	0	0
chr6.fa	90636247	91006562	BACH2	0.550195535	228
chr6.fa	91225353	91296907	MAP3K7	14.11760823	1900

chr6.fa	93949740	94129300	EPHA7	0.763468582	228
chr6.fa	94416801	94486199	TSG1	0	0
chr6.fa	96025413	96057326	MANEA	0	0
chr6.fa	96463845	96663488	FUT9	0.132100302	76
chr6.fa	96969702	97003151	KIAA0776	12.80394411	2421
chr6.fa	97010424	97064512	FHL5	0	0
chr6.fa	97245888	97285353	GPR63	0	0
chr6.fa	97337187	97345767	NDUFAF4	0.700976688	76
chr6.fa	97372496	97588630	KLHL32	0	0
chr6.fa	97537843	97862283	MIR548H3	0	0
chr6.fa	97590037	97731052	MMS22L	0.586667672	228
chr6.fa	98472407	98472495	MIR2113	0	0
chr6.fa	99282580	99286666	POU3F2	1.240719843	228
chr6.fa	99321601	99395849	FBXL4	9.129376278	1140
chr6.fa	99720793	99797531	C6orf168	0.865768479	380
chr6.fa	99817348	99842082	COQ3	1.336125616	76
chr6.fa	99847841	99873207	SFRS18	13.64302566	1976
chr6.fa	99880184	99963252	USP45	2.646676259	760
chr6.fa	99968870	99981059	LOC100130890	5.265331241	456
chr6.fa	99990263	100016690	CCNC	19.00020442	1971
chr6.fa	100054650	100063454	PRDM13	0	0
chr6.fa	100367786	100442114	MCHR2	0	0
chr6.fa	100836750	100911551	SIM1	2.115383965	380
chr6.fa	100956608	101329224	ASCC3	9.826572318	4636
chr6.fa	101846861	102517958	GRIK2	0	0
chr6.fa	105175968	105307794	HACE1	2.216127125	456
chr6.fa	105404923	105531206	LIN28B	0	0
chr6.fa	105544699	105585049	BVES	0.87622086	228
chr6.fa	105585562	105617819	C6orf112	0	0
chr6.fa	105605775	105627858	POPDC3	1.768898661	152
chr6.fa	105725442	105850999	PREP	17.22908185	2280
chr6.fa	106534195	106557811	PRDM1	0.635816101	152
chr6.fa	106632352	106773695	ATG5	13.54628554	1976
chr6.fa	106959730	107018334	AIM1	82.44259775	27996
chr6.fa	107018903	107077373	RTN4IP1	3.205989828	380
chr6.fa	107077441	107116292	QRSL1	7.387831721	1368
chr6.fa	107165327	107235300	LOC100422737	4.87436772	304
chr6.fa	107349407	107372547	C6orf203	15.30339748	684
chr6.fa	107386385	107435636	BEND3	3.806668139	1140
chr6.fa	107473761	107780779	PDSS2	12.84753277	2052
chr6.fa	107811317	107982513	SOBP	4.339294778	1216
chr6.fa	108023364	108145521	SCML4	0	0
chr6.fa	108188960	108279482	SEC63	4.885709665	1428

chr6.fa	108362613	108395941	OSTM1	0.378287229	76
chr6.fa	108487215	108510013	NR2E1	0	0
chr6.fa	108532717	108582464	SNX3	55.41540964	3648
chr6.fa	108616098	108844251	LACE1	2.998054167	304
chr6.fa	108881026	109005971	FOXO3	13.46044259	4560
chr6.fa	109072857	109091145	NCRNA00222	0	0
chr6.fa	109169619	109295352	ARMC2	0.990529876	152
chr6.fa	109307640	109415708	SESN1	22.80865169	3724
chr6.fa	109416356	109485115	CEP57L1	1.093719338	152
chr6.fa	109615506	109629423	CCDC162	0	0
chr6.fa	109687717	109703762	CD164	10.81932611	1514
chr6.fa	109711418	109761930	PPIL6	1.347912344	228
chr6.fa	109762560	109765122	SMPD2	13.6746052	760
chr6.fa	109765266	109777190	MICAL1	11.13178557	1824
chr6.fa	109783719	109804440	ZBTB24	6.201820084	1596
chr6.fa	109814059	110012415	AKD1	1.342797349	456
chr6.fa	110012424	110146634	FIG4	12.51972831	1748
chr6.fa	110300298	110301924	GPR6	0	0
chr6.fa	110421022	110501207	WASF1	2.099594199	304
chr6.fa	110501624	110553422	CDC40	7.459219258	1292
chr6.fa	110567149	110679475	C6orf186	0	0
chr6.fa	110713383	110736753	DDO	1.977946277	152
chr6.fa	110745907	110797844	SLC22A16	0	0
chr6.fa	110931181	111136412	CDK19	27.41570533	7568
chr6.fa	111195987	111216913	AMD1	386.9253437	59520
chr6.fa	111279763	111289091	GTF3C6	102.8383062	4393
chr6.fa	111303291	111346794	RPF2	5.342945728	228
chr6.fa	111367622	111368757	GSTM2P1	0	0
chr6.fa	111408781	111544606	SLC16A10	2.33844222	304
chr6.fa	111580482	111590261	KIAA1919	0.431438698	76
chr6.fa	111620234	111804414	REV3L	7.581311961	3648
chr6.fa	111804675	111923497	LOC643749	0.965844466	304
chr6.fa	111880143	111927474	TRAF3IP2	13.77379162	1824
chr6.fa	111982485	112194627	FYN	0.593339405	76
chr6.fa	112375278	112390887	WISP3	0	0
chr6.fa	112391860	112408673	TUBE1	2.144739588	214
chr6.fa	112408752	112423993	C6orf225	1.955484778	228
chr6.fa	112429134	112575828	LAMA4	0.22617173	76
chr6.fa	112668532	112672498	RFPL4B	0	0
chr6.fa	114178527	114184652	MARCKS	36.23773713	6992
chr6.fa	114225551	114242806	FLJ34503	0	0
chr6.fa	114257320	114292359	HDAC2	10.11479116	3040
chr6.fa	114376750	114384041	HS3ST5	2.46387079	304

chr6.fa	116262693	116381921	FRK	11.86500897	1520
chr6.fa	116359894	116361107	TPI1P3	0	0
chr6.fa	116421999	116566853	NT5DC1	16.18362138	2277
chr6.fa	116440085	116447296	COL10A1	0	0
chr6.fa	116571131	116575261	TSPYL4	15.1383833	2812
chr6.fa	116596022	116601280	TSPYL1	52.70735344	12464
chr6.fa	116601283	116759442	DSE	2.02175732	380
chr6.fa	116782556	116784934	FAM26F	0	0
chr6.fa	116817651	116866773	BET3L	0	0
chr6.fa	116832808	116839709	FAM26E	0	0
chr6.fa	116850195	116880031	FAM26D	0	0
chr6.fa	116892583	116914436	RWDD1	31.95693121	1881
chr6.fa	116937642	116954148	RSPH4A	0	0
chr6.fa	116956781	116989973	ZUFSP	4.586593661	456
chr6.fa	117002367	117063030	KPNA5	3.916084551	380
chr6.fa	117073360	117086886	FAM162B	0	0
chr6.fa	117113248	117150198	GPRC6A	0	0
chr6.fa	117198376	117253326	RFX6	0	0
chr6.fa	117586721	117594725	VGLL2	0	0
chr6.fa	117609530	117747018	ROS1	0	0
chr6.fa	117803820	117891020	DCBLD1	1.816490352	152
chr6.fa	117881433	117923705	GOPC	13.75777946	2790
chr6.fa	117996617	118031886	NUS1	5.625604793	1216
chr6.fa	118228689	118638839	SLC35F1	0.348264433	76
chr6.fa	118781935	119031238	C6orf204	0.840638287	304
chr6.fa	118822536	118824996	BRD7P3	0	0
chr6.fa	118869442	118881587	PLN	0	0
chr6.fa	119103871	119104581	LOC100287632	0	0
chr6.fa	119134612	119256327	MCM9	2.199670185	608
chr6.fa	119215241	119230335	ASF1A	14.15207885	1596
chr6.fa	119280996	119470358	FAM184A	1.219147908	228
chr6.fa	119390212	119390308	MIR548B	0	0
chr6.fa	119499267	119670926	MAN1A1	3.281158013	608
chr6.fa	121400627	121655644	C6orf170	1.975944757	456
chr6.fa	121756745	121770873	GJA1	0	0
chr6.fa	122720696	122754264	HSF2	2.506792268	304
chr6.fa	122764493	122792952	SERINC1	9.185641222	1292
chr6.fa	122793062	123047518	PKIB	1.550510619	152
chr6.fa	123100646	123105218	FABP7	0	0
chr6.fa	123109971	123130864	SMPDL3A	5.392538939	532
chr6.fa	123317582	123385063	CLVS2	0	0
chr6.fa	123537483	123957942	TRDN	0	0
chr6.fa	124125069	125146786	NKAIN2	0	0

chr6.fa	125229392	125284173	STL	0.316462509	76
chr6.fa	125304514	125404661	RNF217	0	0
chr6.fa	125474879	125584644	TPD52L1	12.2457425	897
chr6.fa	125596496	125623282	HDDC2	33.48942815	2432
chr6.fa	126070732	126082415	HEY2	0.632480235	76
chr6.fa	126102307	126253176	NCOA7	6.214718767	1976
chr6.fa	126277861	126301389	HINT3	15.98013354	2428
chr6.fa	126307576	126360420	TRMT11	0.866213262	76
chr6.fa	126661253	126669754	CENPW	3.072999961	76
chr6.fa	127440048	127518184	RSPO3	0	0
chr6.fa	127588020	127609504	RNF146	11.83587574	1140
chr6.fa	127609857	127664754	ECHDC1	19.08604738	2052
chr6.fa	127780320	127780535	KIAA0408	0	0
chr6.fa	127794225	127840500	C6orf174	0	0
chr6.fa	127898319	127912960	C6orf58	0	0
chr6.fa	128029339	128239776	THEMIS	0	0
chr6.fa	128289924	128841819	PTPRK	4.152708661	1140
chr6.fa	129204286	129837710	LAMA2	0.174354608	76
chr6.fa	129898240	130031370	ARHGAP18	16.88103981	2656
chr6.fa	130152389	130182416	C6orf191	0	0
chr6.fa	130339728	130462594	L3MBTL3	4.009933587	760
chr6.fa	130465461	130544099	SAMD3	0	0
chr6.fa	130758262	130764210	TMEM200A	0	0
chr6.fa	131148545	131158276	LOC285733	1.344576478	152
chr6.fa	131160488	131384462	EPB41L2	1.809596229	380
chr6.fa	131466461	131604673	AKAP7	3.790878372	532
chr6.fa	131894365	131905468	ARG1	0	0
chr6.fa	131895106	131949363	MED23	7.882429486	1976
chr6.fa	131958442	132068550	ENPP3	0.533960986	76
chr6.fa	132021609	132022541	OR2A4	0	0
chr6.fa	132029581	132032157	CTAGE9	0.655831299	76
chr6.fa	132129156	132216295	ENPP1	0.227061294	76
chr6.fa	132269317	132272518	CTGF	1.442650944	152
chr6.fa	132617194	132722664	MOXD1	0	0
chr6.fa	132778663	132834337	STX7	9.895513553	1900
chr6.fa	132859427	132860475	TAAR9	0	0
chr6.fa	132873832	132874860	TAAR8	0	0
chr6.fa	132891461	132892498	TAAR6	0	0
chr6.fa	132909731	132910877	TAAR5	0	0
chr6.fa	132929364	132930441	TAAR3	0	0
chr6.fa	132938289	132945414	TAAR2	0	0
chr6.fa	132966123	132967142	TAAR1	0	0
chr6.fa	133001997	133035194	VNN1	0.439667168	76

chr6.fa	133043926	133055904	VNN3	0	0
chr6.fa	133065009	133084598	VNN2	0.812172229	76
chr6.fa	133090507	133119747	C6orf192	1.393947298	152
chr6.fa	133135708	133138703	RPS12	1663.600264	37627
chr6.fa	133136446	133136518	SNORD101	0	0
chr6.fa	133137941	133138016	SNORD100	0	0
chr6.fa	133138358	133138490	SNORA33	0	0
chr6.fa	133409219	133427717	LOC285735	0	0
chr6.fa	133562495	133853258	EYA4	1.155321667	301
chr6.fa	134142285	134175130	MGC34034	0	0
chr6.fa	134210259	134216675	TCF21	0	0
chr6.fa	134274301	134308629	TBPL1	10.25133928	608
chr6.fa	134308719	134373789	SLC2A12	3.320521235	836
chr6.fa	134490384	134639196	SGK1	6.39418837	1140
chr6.fa	135238528	135271260	ALDH8A1	5.286013611	608
chr6.fa	135281517	135376036	HBS1L	4.890157487	2052
chr6.fa	135300476	135300570	MIR3662	0	0
chr6.fa	135502453	135540310	MYB	0	0
chr6.fa	135605110	135818903	AHI1	4.29859721	1140
chr6.fa	135818939	136011976	NCRNA00271	2.066235536	152
chr6.fa	136172834	136516709	PDE7B	2.509905744	608
chr6.fa	136552168	136571449	FAM54A	0	0
chr6.fa	136578001	136610989	BCLAF1	20.47843794	6688
chr6.fa	136663419	136871957	MAP7	40.62907142	9964
chr6.fa	136878187	137113656	MAP3K5	28.94286488	6761
chr6.fa	137143702	137235072	PEX7	20.36323936	1357
chr6.fa	137243402	137246776	SLC35D3	0	0
chr6.fa	137303296	137314368	NHEG1	0	0
chr6.fa	137321108	137366298	IL20RA	8.262718235	1368
chr6.fa	137464957	137494785	IL22RA2	0	0
chr6.fa	137518621	137540567	IFNGR1	3.189088106	304
chr6.fa	137813336	137815531	OLIG3	0	0
chr6.fa	138188581	138204449	TNFAIP3	9.91530636	1976
chr6.fa	138409642	138428660	PERP	9.822124497	1900
chr6.fa	138483053	138665800	KIAA1244	41.0636236	27479
chr6.fa	138537127	138539627	PBOV1	0	0
chr6.fa	138725336	138734582	HEBP2	112.0628659	6460
chr6.fa	138743181	138893668	NHSL1	11.65329266	4104
chr6.fa	138756350	138756431	MIR3145	0	0
chr6.fa	139012805	139018425	FLJ46906	0	0
chr6.fa	139094657	139114456	CCDC28A	20.33633004	1368
chr6.fa	139117248	139225207	ECT2L	0	0
chr6.fa	139225620	139309398	REPS1	10.94742337	1748

chr6.fa	139349819	139364439	C6orf115	9.843029258	374
chr6.fa	139456249	139501946	HECA	12.02757685	3040
chr6.fa	139561199	139613208	TXLNB	0	0
chr6.fa	139693397	139695785	CITED2	47.26077347	4484
chr6.fa	139790132	139795733	LOC645434	0	0
chr6.fa	140526389	140526463	MIR3668	0	0
chr6.fa	142396745	142409936	NMBR	0	0
chr6.fa	142468410	142542085	VTA1	9.899294202	1444
chr6.fa	142623056	142767403	GPR126	2.913767947	912
chr6.fa	142847592	142959026	LOC153910	0	0
chr6.fa	143072604	143266338	HIVEP2	16.5123154	7220
chr6.fa	143382023	143661441	AIG1	12.2034882	760
chr6.fa	143743969	143771841	ADAT2	0.540632719	152
chr6.fa	143771918	143811751	PEX3	3.658333287	456
chr6.fa	143815949	143833020	FUCA2	5.970088577	684
chr6.fa	143875467	143890476	LOC285740	0	0
chr6.fa	143929317	144152322	PHACTR2	16.40178703	7210
chr6.fa	144164508	144184943	LTV1	17.70299724	1444
chr6.fa	144185573	144259483	C6orf94	0	0
chr6.fa	144261437	144385735	PLAGL1	2.305973122	340
chr6.fa	144324034	144329867	HYMAI	0	0
chr6.fa	144416018	144416754	SF3B5	121.5456216	4028
chr6.fa	144471654	144513076	STX11	1.228265942	304
chr6.fa	144612873	145174170	UTRN	15.21733213	8506
chr6.fa	145946440	146056991	EPM2A	0.975629674	152
chr6.fa	146119272	146135921	FBXO30	2.684705133	532
chr6.fa	146205945	146285233	SHPRH	6.762690391	2128
chr6.fa	146348782	146758731	GRM1	0	0
chr6.fa	146864828	146876086	RAB32	8.429733937	456
chr6.fa	146920136	147136597	C6orf103	0	0
chr6.fa	147122805	147124960	LOC729176	0	0
chr6.fa	147162525	147495084	LOC729178	0.588891583	76
chr6.fa	147525508	147708707	STXBP5	16.68911631	4676
chr6.fa	147829828	147891157	SAMD5	0.26731408	76
chr6.fa	148663729	148873184	SASH1	23.57768005	8173
chr6.fa	149068271	149398126	UST	3.079449303	608
chr6.fa	149639436	149732747	TAB2	36.91847623	7296
chr6.fa	149721495	149722182	SUMO4	0	0
chr6.fa	149768766	149806148	ZC3H12D	0	0
chr6.fa	149825631	149867171	PPIL4	6.16601512	684
chr6.fa	149887528	149912067	C6orf72	9.144276481	456
chr6.fa	149916172	149959728	KATNA1	6.667284617	456
chr6.fa	149982051	150039392	LATS1	13.15176377	2812

chr6.fa	150045457	150067688	NUP43	9.663114874	1672
chr6.fa	150070831	150132557	PCMT1	11.38886966	888
chr6.fa	150139894	150185480	LRP11	7.007320581	1140
chr6.fa	150209601	150212097	RAET1E	0	0
chr6.fa	150238014	150244214	RAET1G	0	0
chr6.fa	150263136	150270368	ULBP2	0	0
chr6.fa	150285143	150294846	ULBP1	0	0
chr6.fa	150319155	150326280	RAET1K	0	0
chr6.fa	150341266	150346668	RAET1L	0	0
chr6.fa	150385743	150390202	ULBP3	0	0
chr6.fa	150464188	150571528	PPP1R14C	0.764358146	76
chr6.fa	150690028	150725765	IYD	2.900646873	988
chr6.fa	150920999	151164799	PLEKHG1	9.793658438	3187
chr6.fa	151186691	151423023	MTHFD1L	2.34867221	380
chr6.fa	151561134	151679694	AKAP12	59.03771557	23332
chr6.fa	151685250	151712677	ZBTB2	4.929298317	684
chr6.fa	151725989	151773316	RMND1	7.960043973	684
chr6.fa	151773422	151791232	C6orf211	3.94277148	456
chr6.fa	151815175	151942328	C6orf97	1.279638282	304
chr6.fa	152011631	152424408	ESR1	0	0
chr6.fa	152442822	152958534	SYNE1	2.891528839	3648
chr6.fa	153019030	153045715	MYCT1	0	0
chr6.fa	153071933	153080902	VIP	0	0
chr6.fa	153291658	153304740	FBXO5	0.685631704	76
chr6.fa	153308400	153323925	MTRF1L	1.743323686	304
chr6.fa	153332032	153452389	RGS17	1.152875365	76
chr6.fa	154331636	154568001	OPRM1	0	0
chr6.fa	154475618	154677900	IPCEF1	0.491261899	152
chr6.fa	154726433	154831753	CNKSR3	2.094479204	304
chr6.fa	155054512	155155194	RBM16	23.64350781	5320
chr6.fa	155411423	155575707	TIAM2	0.351822691	76
chr6.fa	155577264	155635617	TFB1M	2.164087612	152
chr6.fa	155585147	155597682	CLDN20	0	0
chr6.fa	155716502	155777037	NOX3	0	0
chr6.fa	157099064	157531913	ARID1B	24.37584164	10564
chr6.fa	157710054	157745253	C6orf35	1.344798869	304
chr6.fa	157802557	158094977	ZDHHC14	4.85168383	608
chr6.fa	157950164	157950232	MIR3692	0	0
chr6.fa	158244294	158366109	SNX9	28.16961109	5320
chr6.fa	158402888	158520207	SYNJ2	8.267610838	2812
chr6.fa	158530536	158589312	SERAC1	4.684223345	836
chr6.fa	158589379	158620376	GTF2H5	5.181045021	1744
chr6.fa	158733692	158932856	TULP4	21.28104735	10640

chr6.fa	158957468	159056467	TMEM181	4.071091134	988
chr6.fa	159057507	159065804	DYNLT1	93.59195222	3190
chr6.fa	159071046	159185901	SYTL3	10.8344487	1064
chr6.fa	159186773	159240456	EZR	208.3550906	29184
chr6.fa	159262158	159278664	OSTCL	0	0
chr6.fa	159309619	159331385	C6orf99	0	0
chr6.fa	159398266	159421198	RSPH3	14.75120042	1444
chr6.fa	159456027	159466184	TAGAP	0.948942744	152
chr6.fa	159590429	159693140	FNDC1	0	0
chr6.fa	160100149	160114353	SOD2	69.51122354	5548
chr6.fa	160148152	160177351	WTAP	22.48774136	2262
chr6.fa	160181291	160183364	LOC100129518	3.233788713	228
chr6.fa	160182989	160199312	ACAT2	61.00810055	2757
chr6.fa	160199530	160210735	TCP1	101.9365104	9424
chr6.fa	160201282	160201413	SNORA20	0	0
chr6.fa	160206626	160206765	SNORA29	0	0
chr6.fa	160211492	160219461	MRPL18	53.62871969	2356
chr6.fa	160221301	160241735	PNLDC1	0	0
chr6.fa	160327974	160329107	MAS1	0	0
chr6.fa	160390131	160527583	IGF2R	6.864323115	2806
chr6.fa	160514114	160517244	LOC729603	0	0
chr6.fa	160542863	160579750	SLC22A1	0	0
chr6.fa	160637794	160679963	SLC22A2	3.364109887	380
chr6.fa	160769425	160876014	SLC22A3	2.103597238	532
chr6.fa	160887587	160932156	LPAL2	0	0
chr6.fa	160952515	161087407	LPA	0	0
chr6.fa	161123225	161175085	PLG	7.167886941	1140
chr6.fa	161412822	161538417	MAP3K4	22.19040448	5472
chr6.fa	161551057	161695107	AGPAT4	0	0
chr6.fa	161581164	161583014	NCRNA00241	0	0
chr6.fa	161768590	163148834	PARK2	2.489890546	456
chr6.fa	163148164	163736524	PACRG	6.536518662	532
chr6.fa	163731017	163745505	LOC285796	0	0
chr6.fa	163759374	163768065	DKFZp451B082	0.342704656	76
chr6.fa	163834097	163834982	LOC100526820	0	0
chr6.fa	163835675	163999628	QKI	5.043607333	3496
chr6.fa	165693153	165723111	C6orf118	0	0
chr6.fa	165740778	166075584	PDE10A	19.46633613	7280
chr6.fa	166337536	166401038	C6orf176	0	0
chr6.fa	166401528	166403103	LOC441177	0	0
chr6.fa	166571086	166582131	T	0	0
chr6.fa	166719168	166721871	PRR18	0.62514133	76
chr6.fa	166733517	166755991	SFT2D1	12.02201707	373

chr6.fa	166778408	166796486	BRP44L	40.49363526	1748
chr6.fa	166822854	167275771	RPS6KA2	20.56005547	5596
chr6.fa	166922842	166922921	MIR1913	0	0
chr6.fa	167343004	167370077	RNASET2	6.760688871	380
chr6.fa	167411295	167411400	MIR3939	0	0
chr6.fa	167412816	167454066	FGFR1OP	6.111306914	518
chr6.fa	167525295	167552629	CCR6	0	0
chr6.fa	167570360	167571319	GPR31	0	0
chr6.fa	167584081	167596396	TCP10L2	0	0
chr6.fa	167704803	167729502	UNC93A	0	0
chr6.fa	167738574	167756177	TTLL2	0	0
chr6.fa	167786577	167797998	TCP10	0	0
chr6.fa	168185219	168197539	C6orf123	1.02433332	76
chr6.fa	168224570	168227476	C6orf124	0.731444266	76
chr6.fa	168227671	168365793	MLLT4	40.06775634	13745
chr6.fa	168376604	168377619	HGC6-3	0	0
chr6.fa	168418553	168445769	KIF25	0	0
chr6.fa	168456464	168479839	FRMD1	0.537964026	76
chr6.fa	168707584	168720402	DACT2	8.042995846	1064
chr6.fa	168841831	169068674	SMOC2	0.536629679	76
chr6.fa	169615875	169654137	THBS2	0.872884994	228
chr6.fa	169857307	170102159	WDR27	0.975184891	152
chr6.fa	170102257	170106402	C6orf120	1.844734019	304
chr6.fa	170110332	170124106	PHF10	15.5971761	836
chr6.fa	170140215	170151638	TCTE3	0	0
chr6.fa	170151721	170181617	C6orf70	8.133731407	760
chr6.fa	170188886	170198921	NCRNA00242	0	0
chr6.fa	170195280	170202969	C6orf208	0	0
chr6.fa	170563422	170571657	LOC154449	0	0
chr6.fa	170591294	170599697	DLL1	0	0
chr6.fa	170615844	170714237	FAM120B	23.48182949	3420
chr6.fa	170844204	170862417	PSMB1	97.68706159	4028
chr6.fa	170863421	170881958	TBP	2.663133199	228
chr6.fa	170884660	170893780	PDCD2	9.129821061	1824
chr7.fa	192969	300711	FAM20C	4.30059873	532
chr7.fa	330136	331454	LOC100288524	0	0
chr7.fa	419391	422845	LOC442497	0	0
chr7.fa	536897	559481	PDGFA	7.82772128	988
chr7.fa	560028	564869	FLJ44511	0.482143864	76
chr7.fa	588834	767313	PRKAR1B	11.77405102	1520
chr7.fa	766338	826116	HEATR2	9.707815481	1444
chr7.fa	855194	914557	SUN1	9.824570799	2052
chr7.fa	916191	936071	GET4	27.52200827	2584

chr7.fa	937537	994289	ADAP1	5.095202064	532
chr7.fa	1004486	1015235	COX19	3.110139272	684
chr7.fa	1022835	1029276	CYP2W1	0	0
chr7.fa	1036623	1177893	C7orf50	50.97959713	2964
chr7.fa	1062569	1062662	MIR339	0	0
chr7.fa	1097141	1098897	GPR146	0	0
chr7.fa	1126443	1133451	GPER	2.193665626	304
chr7.fa	1192543	1199855	ZFAND2A	18.7589101	760
chr7.fa	1272654	1276613	UNCX	0	0
chr7.fa	1473995	1499109	MICALL2	2.719842924	379
chr7.fa	1509913	1544018	INTS1	13.32945424	4180
chr7.fa	1570368	1581870	MAFK	9.312181747	1064
chr7.fa	1582680	1596066	TMEM184A	5.87401563	1444
chr7.fa	1606970	1609668	PSMG3	19.55217909	684
chr7.fa	1609709	1629261	KIAA1908	1.455104845	380
chr7.fa	1654106	1656328	TFAMP1	0	0
chr7.fa	1748798	1787590	ELFN1	0.451676286	76
chr7.fa	1855428	2272583	MAD1L1	6.790266885	836
chr7.fa	2273926	2281833	FTSJ2	14.77966648	1064
chr7.fa	2281857	2290780	NUDT1	2.016864716	76
chr7.fa	2291405	2354099	SNX8	5.005800849	1064
chr7.fa	2394474	2420377	EIF3B	53.51530024	7414
chr7.fa	2443259	2474216	CHST12	1.633907274	152
chr7.fa	2552163	2568810	LFNG	0	0
chr7.fa	2577444	2595392	C7orf27	5.609592635	760
chr7.fa	2598632	2654368	IQCE	5.186160016	1596
chr7.fa	2671603	2704436	TTYH3	1.401953377	304
chr7.fa	2719163	2755069	AMZ1	0	0
chr7.fa	2767741	2883959	GNA12	21.97535231	4332
chr7.fa	2945710	3083509	CARD11	21.31618514	4180
chr7.fa	3341080	4308631	SDK1	1.645471611	532
chr7.fa	4721930	4811074	FOXK1	11.78027797	5928
chr7.fa	4815264	4831399	KIAA0415	7.602883896	988
chr7.fa	4838740	4923335	RADIL	0	0
chr7.fa	4897369	4901625	PAPOLB	0	0
chr7.fa	4945620	4998844	MMD2	0	0
chr7.fa	5013616	5037800	RNF216L	4.868807943	532
chr7.fa	5085553	5109119	RBAK	3.096128634	912
chr7.fa	5111738	5112854	LOC389458	0	0
chr7.fa	5160941	5184177	ZNF890P	0	0
chr7.fa	5229835	5273486	WIPI2	21.83413397	4636
chr7.fa	5322561	5343704	SLC29A4	1.169332305	152
chr7.fa	5346423	5463177	TNRC18	15.0309684	7144

chr7.fa	5515428	5553399	FBXL18	5.134787676	1900
chr7.fa	5535450	5535548	MIR589	0	0
chr7.fa	5566779	5570232	ACTB	724.7591905	59052
chr7.fa	5632454	5646286	FSCN1	0	0
chr7.fa	5659678	5821292	RNF216	9.635315988	2428
chr7.fa	5862791	5894066	ZNF815	0.599121573	76
chr7.fa	5920429	5925994	OCM	0	0
chr7.fa	5938341	5965603	CCZ1	2.779888516	228
chr7.fa	6012870	6048737	PMS2	7.151652392	912
chr7.fa	6048882	6057676	AIMP2	9.840805347	304
chr7.fa	6061878	6098860	EIF2AK1	35.51474373	6276
chr7.fa	6144550	6201195	USP42	7.592653907	1748
chr7.fa	6201412	6312242	CYTH3	5.297133165	1064
chr7.fa	6369040	6388590	C7orf70	6.870550066	684
chr7.fa	6414126	6443598	RAC1	71.89214246	7752
chr7.fa	6448747	6487643	DAGLB	1.742434122	228
chr7.fa	6500712	6523849	KDELR2	20.6839273	2660
chr7.fa	6536409	6591067	GRID2IP	0.364943764	76
chr7.fa	6617065	6628610	ZDHHC4	25.07059138	1824
chr7.fa	6629915	6648355	C7orf26	13.25272932	1140
chr7.fa	6655527	6663921	ZNF853	2.361793283	380
chr7.fa	6728064	6746566	ZNF12	10.92852013	2432
chr7.fa	6774936	6791232	PMS2CL	4.862358602	380
chr7.fa	6838566	6865926	C7orf28B	4.60282821	380
chr7.fa	7115401	7136417	LOC100131257	0.080505571	76
chr7.fa	7222246	7288251	C1GALT1	1.361255809	380
chr7.fa	7398244	7575460	COL28A1	0.961619036	152
chr7.fa	7606616	7647110	MIOS	29.50039933	4408
chr7.fa	7676575	7758238	RPA3	11.39687574	760
chr7.fa	7712940	7918851	LOC729852	13.30854948	1292
chr7.fa	8008423	8128709	GLCCI1	5.700328196	1216
chr7.fa	8152815	8302185	ICA1	42.38351467	4755
chr7.fa	8473585	8792593	NXPH1	0	0
chr7.fa	9673900	9675447	PER4	0	0
chr7.fa	10971580	10979813	NDUFA4	90.73934182	8397
chr7.fa	11013499	11209250	PHF14	8.55182664	3344
chr7.fa	11410062	11871824	THSD7A	3.353212724	1596
chr7.fa	12250848	12276890	TMEM106B	2.335106354	684
chr7.fa	12370509	12443852	VWDE	0	0
chr7.fa	12610203	12693228	SCIN	11.79073035	1748
chr7.fa	12726452	12730556	ARL4A	1.480012646	228
chr7.fa	13930856	14031050	ETV1	0	0
chr7.fa	14184674	14881075	DGKB	0.22417021	76

chr7.fa	15239943	15601640	TMEM195	0.682963011	76
chr7.fa	15650837	15726308	MEOX2	0.712763416	76
chr7.fa	16127152	16460947	ISPD	1.529828248	380
chr7.fa	16501106	16505474	SOSTDC1	3.617190937	304
chr7.fa	16566505	16621114	LOC100129335	0	0
chr7.fa	16639401	16685442	ANKMY2	9.922645265	1140
chr7.fa	16685759	16746148	BZW2	18.20315479	1520
chr7.fa	16793351	16824161	TSPAN13	2.665801892	228
chr7.fa	16832264	16844738	AGR2	253.6570434	11212
chr7.fa	16899030	16921613	AGR3	0	0
chr7.fa	17338276	17385775	AHR	43.28909115	12160
chr7.fa	17830385	17980131	SNX13	3.326525794	953
chr7.fa	18066400	18067486	PRPS1L1	0	0
chr7.fa	18535369	19036992	HDAC9	1.778016695	380
chr7.fa	19155091	19157295	TWIST1	0	0
chr7.fa	19184405	19185044	FERD3L	0	0
chr7.fa	19735085	19748660	TWISTNB	3.908523254	684
chr7.fa	19744981	19745059	MIR3146	0	0
chr7.fa	19758938	19812404	TMEM196	0	0
chr7.fa	20174279	20257013	MACC1	15.13371308	6232
chr7.fa	20370725	20455382	ITGB8	3.08100604	1216
chr7.fa	20655245	20796637	ABCB5	0	0
chr7.fa	20821894	20826508	SP8	6.345484723	1064
chr7.fa	20866917	20867439	RPL23P8	6.463351996	152
chr7.fa	21467689	21554151	SP4	2.913990338	760
chr7.fa	21582833	21939738	DNAH11	0	0
chr7.fa	21940517	21985542	CDCA7L	3.96945841	380
chr7.fa	22157908	22396533	RAPGEF5	1.531384986	456
chr7.fa	22459063	22539901	MGC87042	0	0
chr7.fa	22766766	22771621	IL6	0	0
chr7.fa	22852252	22862421	TOMM7	354.0539403	11590
chr7.fa	22896232	22896305	SNORD93	0	0
chr7.fa	22980878	23053770	FAM126A	2.747864201	760
chr7.fa	23145353	23215038	KLHL7	6.607016634	1216
chr7.fa	23221446	23240630	NUPL2	6.583887962	532
chr7.fa	23286316	23314729	GPNMB	1.222038992	152
chr7.fa	23338940	23349180	C7orf30	20.1477424	684
chr7.fa	23349828	23509995	IGF2BP3	0	0
chr7.fa	23530007	23531031	RPS2P32	1.649029868	76
chr7.fa	23544401	23571656	TRA2A	23.89592169	1976
chr7.fa	23624335	23626146	CLK2P	0	0
chr7.fa	23636998	23684327	CCDC126	0.683407793	76
chr7.fa	23719749	23742269	C7orf46	10.33384637	684

chr7.fa	23749838	23872127	STK31	0	0
chr7.fa	24323807	24331484	NPY	27.16351384	684
chr7.fa	24613085	24727498	MPP6	31.68583648	3116
chr7.fa	24737974	24797639	DFNA5	1.31833433	152
chr7.fa	24836164	25019760	OSBPL3	3.276043019	988
chr7.fa	25158270	25164980	CYCS	11.29212954	2812
chr7.fa	25174316	25219817	C7orf31	1.873200078	304
chr7.fa	25264191	25268105	NPVF	0	0
chr7.fa	25989539	25989606	MIR148A	0	0
chr7.fa	26191847	26226756	NFE2L3	0.91113626	152
chr7.fa	26229556	26240413	HNRNPA2B1	134.3597957	22366
chr7.fa	26241099	26252976	CBX3	33.20921539	2888
chr7.fa	26331515	26413949	SNX10	1.681721357	228
chr7.fa	26443107	26535986	LOC441204	0	0
chr7.fa	26572740	26578444	KIAA0087	0	0
chr7.fa	26677490	26686924	C7orf71	0	0
chr7.fa	26706688	26904341	SKAP2	8.901203029	1589
chr7.fa	27132614	27135625	HOXA1	0.663614987	76
chr7.fa	27139973	27142394	HOXA2	0	0
chr7.fa	27145809	27166639	HOXA3	0.972516199	152
chr7.fa	27168126	27170399	HOXA4	0.978075976	76
chr7.fa	27180996	27183287	HOXA5	2.537704629	152
chr7.fa	27185116	27187368	HOXA6	0	0
chr7.fa	27193338	27196296	HOXA7	5.871569328	532
chr7.fa	27202057	27205149	HOXA9	8.216683281	760
chr7.fa	27209099	27209182	MIR196B	20.12105547	76
chr7.fa	27210210	27219876	HOXA10	21.7709749	3116
chr7.fa	27220776	27224835	HOXA11	7.007765363	836
chr7.fa	27225027	27228912	HOXA11-AS1	3.273374326	228
chr7.fa	27236499	27239725	HOXA13	15.46307427	1748
chr7.fa	27282164	27286192	EVX1	0	0
chr7.fa	27565059	27702620	HIBADH	50.7558717	4560
chr7.fa	27779738	27869386	TAX1BP1	89.58513211	13962
chr7.fa	27870193	28220437	JAZF1	3.789544025	532
chr7.fa	28220076	28280996	LOC100128081	0	0
chr7.fa	28338940	28865511	CREB5	0.191701112	76
chr7.fa	28992974	28998029	TRIL	0	0
chr7.fa	29035247	29186153	CPVL	1.900109399	152
chr7.fa	29234121	29553944	CHN2	0.878889553	152
chr7.fa	29603427	29606911	PRR15	7.050686842	532
chr7.fa	29685538	29690955	LOC646762	4.367316055	1064
chr7.fa	29724388	29724769	LOC100271874	0	0
chr7.fa	29725438	29782019	DPY19L2P3	0	0

chr7.fa	29874341	29945791	WIPF3	0	0
chr7.fa	29959719	30029905	SCRN1	34.40011963	8588
chr7.fa	30052884	30066268	FKBP14	0	0
chr7.fa	30067977	30157961	PLEKHA8	4.607498422	1824
chr7.fa	30174552	30202381	C7orf41	22.09366436	5776
chr7.fa	30323923	30407308	ZNRF2	6.350377326	988
chr7.fa	30409666	30412410	DKFZP586I1420	1.847180321	228
chr7.fa	30464143	30518393	NOD1	0.375840927	76
chr7.fa	30536237	30544457	GGCT	19.11962843	1140
chr7.fa	30634181	30673648	GARS	22.74882849	2812
chr7.fa	30692863	30722140	CRHR2	0	0
chr7.fa	30793347	30797218	INMT	0	0
chr7.fa	30805961	30806177	INMT-FAM188B	0	0
chr7.fa	30811033	30811172	FAM188B	0	0
chr7.fa	30951415	30965131	AQP1	13.85385241	1976
chr7.fa	31003636	31019146	GHRHR	0	0
chr7.fa	31092076	31151093	ADCYAP1R1	0	0
chr7.fa	31377080	31380538	NEUROD6	0	0
chr7.fa	31556978	31693303	CCDC129	0	0
chr7.fa	31726631	31748069	C7orf16	0	0
chr7.fa	31792632	32338383	PDE1C	0	0
chr7.fa	32524945	32534870	LSM5	4.406901667	647
chr7.fa	32535176	32623779	AVL9	28.94953662	3063
chr7.fa	32620553	32758780	DPY19L1P1	0.889119543	76
chr7.fa	32767562	32769595	LOC441208	4.154710181	380
chr7.fa	32772593	32772689	MIR550A2	0	0
chr7.fa	32797898	32802536	LOC401321	0	0
chr7.fa	32907778	32931468	KBTBD2	11.38931445	1900
chr7.fa	32956427	32982782	RP9P	2.606201082	152
chr7.fa	32997005	33046543	FKBP9	3.892066314	608
chr7.fa	33053742	33102409	NT5C3	5.428788686	456
chr7.fa	33134410	33149002	RP9	0	0
chr7.fa	33169152	33645680	BBS9	4.205415347	760
chr7.fa	33945112	34194112	BMPER	0	0
chr7.fa	34386124	34873943	AAA1	0	0
chr7.fa	34697897	34917944	NPSR1	0	0
chr7.fa	34968493	35077653	DPY19L1	1.389721867	304
chr7.fa	35129456	35147346	DPY19L2P1	0	0
chr7.fa	35242042	35293711	TBX20	0	0
chr7.fa	35672270	35734772	HERPUD2	7.550621992	988
chr7.fa	35840627	35944917	7-Sep	19.46589135	2204
chr7.fa	36192836	36341152	EEPD1	1.803146887	380
chr7.fa	36363759	36429734	KIAA0895	5.568895067	1216

chr7.fa	36429432	36493400	ANLN	0.360273552	76
chr7.fa	36552549	36764154	AOAH	0	0
chr7.fa	36893961	37488511	ELMO1	0	0
chr7.fa	36958962	36959037	MIR1200	0	0
chr7.fa	37779996	37780913	GPR141	0	0
chr7.fa	37888199	37940002	TXNDC3	0	0
chr7.fa	37945535	37956525	SFRP4	0.568431604	76
chr7.fa	37960163	37991542	EPDR1	7.156322605	836
chr7.fa	38217933	38270270	STARD3NL	14.63355554	1064
chr7.fa	38299244	38313248	TARP	357.3199758	16501
chr7.fa	38423297	38671167	AMPH	0.493263418	76
chr7.fa	38724946	38726689	FAM183B	0	0
chr7.fa	38763543	38948800	VPS41	22.47550985	4946
chr7.fa	39017609	39504390	POU6F2	0	0
chr7.fa	39606003	39612480	C7orf36	9.702478095	380
chr7.fa	39649086	39651687	LOC646999	0.649604348	76
chr7.fa	39663162	39747717	RALA	14.51835696	1824
chr7.fa	39773167	39834222	NCRNA00265	0.3640542	76
chr7.fa	39989959	40136733	CDK13	13.81226528	4332
chr7.fa	40172342	40174251	C7orf11	16.16538531	684
chr7.fa	40174575	40900366	C7orf10	6.894568302	532
chr7.fa	41728601	41742706	INHBA	0	0
chr7.fa	41733514	41818976	LOC285954	0	0
chr7.fa	42000548	42276618	GLI3	1.028781142	380
chr7.fa	42948872	42951689	C7orf25	14.81791775	1216
chr7.fa	42956462	42971805	PSMA2	70.42391654	4636
chr7.fa	42971939	42977453	MRPL32	28.42224736	1140
chr7.fa	43152198	43602938	HECW1	3.212883952	988
chr7.fa	43190494	43190593	MIR3943	0	0
chr7.fa	43622692	43666978	STK17A	0.859541529	152
chr7.fa	43678859	43769083	C7orf44	41.41611346	1596
chr7.fa	43798272	43846941	BLVRA	21.53968818	1047
chr7.fa	43906157	43909145	MRPS24	77.5813511	2428
chr7.fa	43915501	43965996	URGCP	13.36970703	2335
chr7.fa	43966035	43995735	UBE2D4	10.45415995	1563
chr7.fa	43980494	44058748	POLR2J4	0	0
chr7.fa	44040489	44049723	SPDYE1	0	0
chr7.fa	44068486	44078575	RASA4P	0	0
chr7.fa	44078698	44082082	FLJ35390	2.240812535	160
chr7.fa	44084239	44101315	DBNL	38.10137439	3800
chr7.fa	44102326	44105186	PGAM2	0	0
chr7.fa	44111847	44122129	POLM	5.160807433	608
chr7.fa	44143960	44154159	AEBP1	0	0

chr7.fa	44154286	44163147	POLD2	45.70603742	3648
chr7.fa	44178463	44180916	MYL7	0	0
chr7.fa	44183870	44229022	GCK	0	0
chr7.fa	44240578	44253893	YKT6	50.54860321	6287
chr7.fa	44256749	44365230	CAMK2B	0.736559261	152
chr7.fa	44421965	44530385	NUDCD3	16.4996391	3648
chr7.fa	44552135	44580914	NPC1L1	0	0
chr7.fa	44605403	44614137	DDX56	13.60299527	1520
chr7.fa	44618762	44621827	TMED4	11.57656774	532
chr7.fa	44646121	44748669	OGDH	48.85709665	9651
chr7.fa	44788530	44809479	ZMIZ2	8.873181753	2052
chr7.fa	44836241	44842716	PPIA	50.91732763	5211
chr7.fa	44866488	44887725	H2AFV	21.55836903	4560
chr7.fa	44915892	44924960	PURB	9.69113615	3952
chr7.fa	45002260	45018704	MYO1G	0	0
chr7.fa	45022627	45026259	C7orf40	8.419281556	304
chr7.fa	45024977	45025109	SNORA9	0	0
chr7.fa	45039345	45116069	CCM2	7.009989274	684
chr7.fa	45120036	45128493	NACAD	0.354936166	76
chr7.fa	45139699	45151317	TBRG4	15.79599372	1596
chr7.fa	45143948	45144081	SNORA5A	0	0
chr7.fa	45144505	45144641	SNORA5C	0	0
chr7.fa	45145567	45145698	SNORA5B	0	0
chr7.fa	45197367	45223850	RAMP3	0	0
chr7.fa	45614125	45762714	ADCY1	1.62278772	912
chr7.fa	45763386	45808617	SEPT7P2	3.777757298	380
chr7.fa	45927959	45933267	IGFBP1	0	0
chr7.fa	45951844	45960871	IGFBP3	5.139235498	608
chr7.fa	47314752	47621742	TNS3	24.50438368	8394
chr7.fa	47694842	47701246	C7orf65	0	0
chr7.fa	47814250	47988071	PKD1L1	0.931151457	380
chr7.fa	47834889	47859444	C7orf69	0	0
chr7.fa	48003783	48019246	HUS1	16.56257578	1596
chr7.fa	48026746	48068716	SUN3	0	0
chr7.fa	48075117	48100894	C7orf57	0	0
chr7.fa	48128355	48148330	UPP1	6.959061716	608
chr7.fa	48211057	48687091	ABCA13	0.491706681	380
chr7.fa	48964157	48967049	CDC14C	0	0
chr7.fa	49813257	49952138	VWC2	0	0
chr7.fa	49977024	50132860	ZPBP	0	0
chr7.fa	50135682	50199359	C7orf72	0	0
chr7.fa	50344378	50472798	IKZF1	0	0
chr7.fa	50511832	50518088	FIGNL1	3.829352029	608

chr7.fa	50526134	50633154	DDC		18.83808132	1706
chr7.fa	50599457	50611161	LOC100129427		0	0
chr7.fa	50657760	50861159	GRB10		10.8918256	2584
chr7.fa	51083909	51384515	COBL		23.55410659	5624
chr7.fa	53103349	53104618	POM121L12		0	0
chr7.fa	54268917	54270114	HPVC1		0	0
chr7.fa	54610019	54636948	VSTM2A		0	0
chr7.fa	54819940	54826939	SEC61G		119.6902128	2508
chr7.fa	55086725	55275031	EGFR		5.501288178	1596
chr7.fa	55433141	55501435	LANCL2		7.37737934	1444
chr7.fa	55538306	55640200	VOPP1		3.444615458	456
chr7.fa	55748767	55772260	FKBP9L		0	0
chr7.fa	55861237	55930482		14-Sep	0	0
chr7.fa	55980331	56008433	ZNF713		2.455419929	228
chr7.fa	56019611	56023033	MRPS17		17.3351624	456
chr7.fa	56032296	56067871	GBAS		47.48627803	4215
chr7.fa	56078744	56119268	PSPH		11.17203836	1064
chr7.fa	56119378	56131682	CCT6A		50.73919237	6078
chr7.fa	56128163	56128295	SNORA15		0	0
chr7.fa	56131917	56148365	SUMF2		18.1030788	1672
chr7.fa	56148675	56160689	PHKG1		0	0
chr7.fa	56169266	56174187	CHCHD2		120.2822178	4408
chr7.fa	56182374	56184090	LOC389493		14.08469435	380
chr7.fa	56491397	56516068	LOC650226		0	0
chr7.fa	56563916	56564977	DKFZp434L192		0	0
chr7.fa	57187326	57207571	ZNF479		0	0
chr7.fa	57233377	57247863	LOC642006		0	0
chr7.fa	57472731	57472796	MIR3147		0	0
chr7.fa	57509883	57533265	ZNF716		0	0
chr7.fa	62751670	62764434	LOC643955		0	0
chr7.fa	62856718	62859419	LOC100287834		0	0
chr7.fa	63505821	63538927	ZNF727		2.013306459	152
chr7.fa	63667581	63680668	ZNF735		0	0
chr7.fa	63688852	63727309	ZNF679		0	0
chr7.fa	63773186	63810017	ZNF736		2.41672388	228
chr7.fa	63894071	63895895	LOC649395		0.926036463	76
chr7.fa	63980255	64023505	ZNF680		3.44083481	760
chr7.fa	64042988	64044129	LOC641746		0	0
chr7.fa	64126511	64171401	ZNF107		3.991252736	988
chr7.fa	64254771	64294025	ZNF138		1.862747697	228
chr7.fa	64310071	64314178	LOC168474		0.4114235	76
chr7.fa	64363620	64391955	ZNF273		0.765914884	152
chr7.fa	64434830	64450732	ZNF117		2.36312763	608

chr7.fa	64451415	64467124	ERV3	6.540966483	760
chr7.fa	64498732	64535091	CCT6P3	0.753238592	76
chr7.fa	64601603	64694600	INTS4L1	0	0
chr7.fa	64838768	64865998	ZNF92	4.342185862	608
chr7.fa	65112777	65183632	INTS4L2	0	0
chr7.fa	65216092	65228662	CCT6P1	2.871291251	228
chr7.fa	65220513	65220646	SNORA22	0	0
chr7.fa	65338257	65419800	VKORC1L1	10.32383878	532
chr7.fa	65425673	65447301	GUSB	3.67745892	380
chr7.fa	65540776	65558329	ASL	19.00509703	1810
chr7.fa	65579805	65619553	CRCP	21.97290601	2888
chr7.fa	65670259	65825438	TPST1	3.296280607	304
chr7.fa	65841031	65865395	NCRNA00174	0.382067878	76
chr7.fa	66018553	66043498	LOC493754	3.080561258	456
chr7.fa	66093868	66108038	KCTD7	5.896254738	1292
chr7.fa	66205643	66274979	RABGEF1	7.229044489	760
chr7.fa	66276449	66309813	LOC729156	1.766007577	76
chr7.fa	66386203	66423538	C7orf42	9.571489748	1824
chr7.fa	66452690	66460588	SBDS	14.74297195	1064
chr7.fa	66461817	66704498	TYW1	2.537704629	380
chr7.fa	66757423	66767406	PMS2P4	11.8816883	304
chr7.fa	66767616	66786513	STAG3L4	7.124743072	684
chr7.fa	69063905	70257475	AUTS2	8.637002424	2432
chr7.fa	70597789	71178584	WBSCR17	0.519950348	76
chr7.fa	70772658	70772756	MIR3914-1	0	0
chr7.fa	71244476	71877360	CALN1	0	0
chr7.fa	72023729	72298813	TYW1B	1.023443756	152
chr7.fa	72299952	72307978	SBDSP1	2.823254777	228
chr7.fa	72333318	72339655	SPDYE7P	0	0
chr7.fa	72349936	72418831	POM121	4.514761341	1216
chr7.fa	72418839	72425302	NSUN5P2	1.920569378	152
chr7.fa	72440192	72443668	LOC541473	0	0
chr7.fa	72469027	72476455	STAG3L3	0	0
chr7.fa	72476680	74988215	PMS2L2	0	0
chr7.fa	72490260	74974606	SPDYE8P	0	0
chr7.fa	72569012	74653459	LOC100093631	0	0
chr7.fa	72569129	74653341	GTF2IP1	0	0
chr7.fa	72634674	72649979	NCF1B	0	0
chr7.fa	72656902	72685658	GTF2IRD2P1	0	0
chr7.fa	72716514	72722864	NSUN5	3.62386267	380
chr7.fa	72726535	72742085	TRIM50	0	0
chr7.fa	72742155	72772641	FKBP6	0	0
chr7.fa	72848109	72850450	FZD9	0	0

chr7.fa	72854728	72936615	BAZ1B	43.11807241	11856
chr7.fa	72950683	72972065	BCL7B	31.31555533	2736
chr7.fa	72983277	72993013	TBL2	3.894512616	532
chr7.fa	73007524	73038870	MLXIPL	1.033006573	152
chr7.fa	73082174	73086440	VPS37D	2.10604354	152
chr7.fa	73095248	73097781	DNAJC30	6.003002458	684
chr7.fa	73097898	73112542	WBSCR22	9.618859048	532
chr7.fa	73113535	73134017	STX1A	0.795270507	76
chr7.fa	73125647	73125727	MIR4284	0	0
chr7.fa	73149399	73150330	WBSCR26	0	0
chr7.fa	73150425	73153184	ABHD11	27.613411	1824
chr7.fa	73183327	73184600	CLDN3	7.960043973	456
chr7.fa	73245193	73247015	CLDN4	29.66830459	2432
chr7.fa	73248921	73256855	WBSCR27	16.35641925	684
chr7.fa	73275489	73280223	WBSCR28	0	0
chr7.fa	73442427	73484236	ELN	0.484812557	76
chr7.fa	73498156	73536854	LIMK1	8.623436568	1292
chr7.fa	73588706	73611429	EIF4H	61.73843286	7068
chr7.fa	73605528	73605624	MIR590	0	0
chr7.fa	73624087	73644164	LAT2	0	0
chr7.fa	73645832	73668738	RFC2	7.981838299	608
chr7.fa	73703805	73820273	CLIP2	10.02627951	2508
chr7.fa	73868120	74016920	GTF2IRD1	12.57843956	1996
chr7.fa	74072030	74175022	GTF2I	23.70733405	4828
chr7.fa	74188309	74203659	NCF1	0	0
chr7.fa	74210484	74267841	GTF2IRD2	1.903890047	304
chr7.fa	74299283	74306731	STAG3L2	3.007394592	152
chr7.fa	74306894	74336166	PMS2P5	0.835523292	76
chr7.fa	74379083	74438803	GATSL1	0	0
chr7.fa	74456283	74489699	WBSCR16	12.08272984	1292
chr7.fa	74508347	74565623	GTF2IRD2B	0.471469092	76
chr7.fa	74572384	74587816	NCF1C	0	0
chr7.fa	74807605	74867341	GATSL2	0	0
chr7.fa	74988447	74996990	STAG3L1	0	0
chr7.fa	75043758	75046064	NSUN5P1	0	0
chr7.fa	75046067	75115568	POM121C	3.460405225	912
chr7.fa	75124299	75133628	SPDYE5	0	0
chr7.fa	75137069	75157453	PMS2P3	5.630052614	380
chr7.fa	75163409	75368279	HIP1	2.101373327	684
chr7.fa	75398842	75419064	CCL26	0	0
chr7.fa	75441114	75443033	CCL24	0	0
chr7.fa	75508317	75518244	RHBDD2	17.99966695	1520
chr7.fa	75544420	75616154	POR	5.452139749	608

chr7.fa	75573101	75573234	SNORA14A	0	0
chr7.fa	75616174	75623992	TMEM120A	10.47617667	684
chr7.fa	75625655	75677321	STYXL1	2.387145867	152
chr7.fa	75677393	75695930	MDH2	134.6956062	7904
chr7.fa	75831216	75916605	SRRM3	0.467466053	76
chr7.fa	75931875	75933614	HSPB1	436.1542782	17592
chr7.fa	75956108	75988342	YWHAG	39.2433526	6612
chr7.fa	76018646	76039012	SRCRB4D	0	0
chr7.fa	76054272	76071388	ZP3	0	0
chr7.fa	76090972	76135312	DTX2	3.131044034	380
chr7.fa	76099337	76104317	FDPSL2A	0	0
chr7.fa	76139745	76157199	UPK3B	11.00769135	836
chr7.fa	76178658	76257299	LOC100133091	0.901795835	76
chr7.fa	76239303	76255000	POMZP3	6.852091606	228
chr7.fa	76610139	76653076	PMS2P11	0.959839907	76
chr7.fa	76668797	76682355	LOC100132832	0.724772534	76
chr7.fa	76751934	76924521	CCDC146	2.028429053	304
chr7.fa	76822688	76829150	FGL2	0.396078516	76
chr7.fa	76940068	77045717	PION	3.639207654	532
chr7.fa	77166773	77269388	PTPN12	15.37923284	2280
chr7.fa	77325743	77409120	RSBN1L	11.02281394	1596
chr7.fa	77423045	77427747	TMEM60	0	0
chr7.fa	77428109	77586821	PHTF2	0.256416917	76
chr7.fa	77646374	79082890	MAGI2	4.881039452	1510
chr7.fa	77976559	77988770	RPL13AP17	0	0
chr7.fa	79400572	79400606	MIR548M	0	0
chr7.fa	79764140	79848725	GNAI1	1.018773543	152
chr7.fa	80087987	80141242	GNAT3	0	0
chr7.fa	80231504	80308593	CD36	0	0
chr7.fa	80371854	80548667	SEMA3C	15.31896485	3564
chr7.fa	81331444	81399452	HGF	0	0
chr7.fa	81579418	82073031	CACNA2D1	2.18210129	375
chr7.fa	82383321	82792197	PCLO	10.00315084	10032
chr7.fa	82993222	83278479	SEMA3E	0.502381453	152
chr7.fa	83587659	83824217	SEMA3A	0	0
chr7.fa	84624872	84751247	SEMA3D	1.350358646	380
chr7.fa	86273230	86494192	GRM3	0	0
chr7.fa	86506223	86689014	KIAA1324L	4.025278572	1292
chr7.fa	86781677	86825477	DMTF1	5.936062742	1140
chr7.fa	86825649	86849031	C7orf23	7.752997876	304
chr7.fa	86954664	86974808	TP53TG1	47.67753436	1520
chr7.fa	86974951	87029112	CROT	24.9453852	3724
chr7.fa	87031361	87105019	ABCB4	0	0

chr7.fa	87132948	87342564	ABCB1	2.081580521	456
chr7.fa	87257729	87461613	RUNDC3B	0.412090674	76
chr7.fa	87463814	87505543	SLC25A40	3.95300147	532
chr7.fa	87505693	87538856	DBF4	1.34924669	228
chr7.fa	87563566	87832204	ADAM22	0.533071422	228
chr7.fa	87834432	87856308	SRI	43.40606886	4089
chr7.fa	87905744	87936209	STEAP4	10.62517869	2128
chr7.fa	88388753	88966346	ZNF804B	0	0
chr7.fa	88423420	88425031	MGC26647	0	0
chr7.fa	89748714	89754914	DPY19L2P4	0	0
chr7.fa	89783689	89794141	STEAP1	21.88350479	1292
chr7.fa	89841000	89866992	STEAP2	24.77347689	3040
chr7.fa	89874488	89940377	C7orf63	7.467002946	1292
chr7.fa	89975979	90020769	GTPBP10	2.465205136	836
chr7.fa	90032648	90045268	CLDN12	1.385496436	228
chr7.fa	90338712	90839904	CDK14	3.704368241	825
chr7.fa	90893783	90898132	FZD1	3.108360143	608
chr7.fa	91502021	91510016	MTERF	3.835134197	373
chr7.fa	91570189	91739987	AKAP9	9.764302816	5472
chr7.fa	91741463	91764059	CYP51A1	12.33692284	1824
chr7.fa	91774198	91794590	LOC401387	0	0
chr7.fa	91828283	91875414	KRIT1	7.331121995	1672
chr7.fa	91875548	92030698	ANKIB1	23.06929404	6308
chr7.fa	92076765	92088742	GATAD1	12.741897	2266
chr7.fa	92116337	92157845	PEX1	10.41412956	2052
chr7.fa	92158087	92166823	C7orf64	6.890565263	532
chr7.fa	92167789	92169079	MGC16142	0	0
chr7.fa	92190072	92214710	FAM133B	0	0
chr7.fa	92234235	92465941	CDK6	1.868085083	988
chr7.fa	92728826	92747336	SAMD9	1.232713764	380
chr7.fa	92759368	92777680	SAMD9L	2.369576971	760
chr7.fa	92817899	92855782	HEPACAM2	1.615004032	152
chr7.fa	92861653	92988338	CCDC132	8.420393511	1368
chr7.fa	93053799	93204042	CALCR	0.847977193	152
chr7.fa	93112072	93112167	MIR653	0	0
chr7.fa	93113248	93113331	MIR489	0	0
chr7.fa	93515745	93520065	TFPI2	0	0
chr7.fa	93535820	93540485	GNGT1	0	0
chr7.fa	93551016	93555826	GNG11	14.17342839	608
chr7.fa	93621000	93633690	BET1	11.58813207	1595
chr7.fa	94023873	94060544	COL1A2	0	0
chr7.fa	94139170	94186328	CASD1	2.035323176	355
chr7.fa	94214536	94285521	SGCE	3.538686886	288

chr7.fa	94285637	94299006	PEG10	2.550158529	760
chr7.fa	94536949	94925727	PPP1R9A	11.02036764	5168
chr7.fa	94927669	94953884	PON1	0	0
chr7.fa	94989184	95025687	PON3	0	0
chr7.fa	95034174	95064384	PON2	4.087548074	304
chr7.fa	95115213	95169543	ASB4	0	0
chr7.fa	95212809	95225925	PDK4	3.197538967	532
chr7.fa	95401818	95727736	DYNC1I1	0	0
chr7.fa	95749532	95951459	SLC25A13	13.77579314	1976
chr7.fa	95848974	95849068	MIR591	0	0
chr7.fa	96318079	96339203	SHFM1	139.1507667	3116
chr7.fa	96597827	96643377	DLX6-AS1	0	0
chr7.fa	96635290	96640352	DLX6	0	0
chr7.fa	96649702	96654143	DLX5	0	0
chr7.fa	96745905	96811075	ACN9	8.216683281	760
chr7.fa	97361271	97369784	TAC1	0	0
chr7.fa	97481429	97501854	ASNS	3.509553654	380
chr7.fa	97595908	97601638	MGC72080	14.178321	445
chr7.fa	97614013	97619416	OCM2	0	0
chr7.fa	97736197	97838944	LMTK2	9.825460363	3952
chr7.fa	97841566	97842271	BHLHA15	9.57593757	304
chr7.fa	97846047	97881468	TECPR1	6.593228387	1292
chr7.fa	97910979	97920839	BRI3	164.5300369	5852
chr7.fa	97920962	98030427	BAIAP2L1	24.16056707	3634
chr7.fa	98246597	98259181	NPTX2	0	0
chr7.fa	98444111	98467673	TMEM130	0	0
chr7.fa	98476113	98610864	TRRAP	10.33873898	5852
chr7.fa	98479273	98479352	MIR3609	0	0
chr7.fa	98625058	98741743	SMURF1	19.35647493	5016
chr7.fa	98771197	98805089	KPNA7	0	0
chr7.fa	98870924	98895594	MYH16	0	0
chr7.fa	98923496	98963885	ARPC1A	126.2107193	9120
chr7.fa	98972329	98992297	ARPC1B	52.17339245	3268
chr7.fa	98992405	99006305	PDAP1	40.61661752	4712
chr7.fa	99006601	99013883	BUD31	39.20932676	1146
chr7.fa	99036315	99036462	PTCD1	0	0
chr7.fa	99036563	99054996	CPSF4	11.10732256	912
chr7.fa	99055784	99063824	ATP5J2	111.4330543	4224
chr7.fa	99070515	99085217	ZNF789	1.914120037	152
chr7.fa	99090854	99097877	ZNF394	10.2435556	988
chr7.fa	99102273	99131445	ZKSCAN5	5.343835293	1064
chr7.fa	99143923	99149757	FAM200A	4.755388491	532
chr7.fa	99156045	99174076	ZNF655	7.491021182	2052

chr7.fa	99195902	99208455	LOC100289187	0	0
chr7.fa	99214571	99230030	ZNF498	5.052280585	988
chr7.fa	99245813	99277621	CYP3A5	2.701606856	380
chr7.fa	99302660	99332819	CYP3A7	0	0
chr7.fa	99354604	99381808	CYP3A4	0	0
chr7.fa	99425636	99464173	CYP3A43	0	0
chr7.fa	99473685	99474656	OR2AE1	0	0
chr7.fa	99488030	99517223	TRIM4	19.92379458	3098
chr7.fa	99520892	99527243	GJC3	0	0
chr7.fa	99564350	99573735	AZGP1	134.9464634	7676
chr7.fa	99578385	99581860	AZGP1P1	0	0
chr7.fa	99613219	99635403	ZKSCAN1	40.60838905	10032
chr7.fa	99647417	99662663	ZSCAN21	3.05632063	152
chr7.fa	99667594	99679371	ZNF3	14.36401755	1824
chr7.fa	99686583	99689822	COPS6	41.32582268	2648
chr7.fa	99690404	99699427	MCM7	13.63034937	1900
chr7.fa	99691183	99691266	MIR25	0	0
chr7.fa	99691391	99691470	MIR93	0	0
chr7.fa	99691616	99691697	MIR106B	0	0
chr7.fa	99699503	99704692	AP4M1	6.473359594	434
chr7.fa	99704804	99717021	TAF6	6.313682798	760
chr7.fa	99717265	99723128	CNPY4	1.297207177	76
chr7.fa	99724320	99726121	MBLAC1	4.510091129	304
chr7.fa	99746530	99751833	C7orf59	91.13675468	2508
chr7.fa	99752043	99756302	C7orf43	3.361441194	380
chr7.fa	99756865	99766373	GAL3ST4	0	0
chr7.fa	99767229	99774990	GPC2	0	0
chr7.fa	99775538	99812010	STAG3	0	0
chr7.fa	99798278	99869855	GATS	7.380715206	1596
chr7.fa	99816871	99819111	PVRIG	0	0
chr7.fa	99905325	99919819	SPDYE3	1.130191475	152
chr7.fa	99918263	99933687	PMS2P1	7.817713681	380
chr7.fa	99933931	99965454	PILRB	3.083229951	456
chr7.fa	99971068	99997722	PILRA	0	0
chr7.fa	99998495	100026302	ZCWPW1	4.339294778	456
chr7.fa	100026413	100031749	MEPCE	15.58005198	2204
chr7.fa	100032912	100034094	C7orf47	1.817379916	76
chr7.fa	100054238	100061894	C7orf61	0	0
chr7.fa	100064142	100076902	TSC22D4	27.66122508	2964
chr7.fa	100081550	100092422	C7orf51	0.471913875	76
chr7.fa	100136834	100165843	AGFG2	11.24209155	2432
chr7.fa	100169853	100171270	SAP25	0	0
chr7.fa	100171634	100183776	LRCH4	13.20024502	1900

chr7.fa	100183956	100198740	FBXO24	0.682963011	76
chr7.fa	100199882	100205798	PCOLCE	1.043236562	76
chr7.fa	100209725	100213000	MOSPD3	13.28942385	836
chr7.fa	100218039	100239173	TFR2	0	0
chr7.fa	100240726	100254084	ACTL6B	0	0
chr7.fa	100271363	100276792	GNB2	143.8325438	10762
chr7.fa	100277130	100286870	GIGYF1	12.81839954	3648
chr7.fa	100303676	100305123	POP7	21.76185687	912
chr7.fa	100318423	100321323	EPO	1.270742639	76
chr7.fa	100331249	100395419	ZAN	0	0
chr7.fa	100400187	100425143	EPHB4	5.438351502	1064
chr7.fa	100450358	100464634	SLC12A9	1.539391065	228
chr7.fa	100464950	100471076	TRIP6	25.0823781	1976
chr7.fa	100472701	100486285	SRRT	29.16992618	3952
chr7.fa	100486344	100487339	UFSP1	1.697066341	76
chr7.fa	100487615	100493541	ACHE	0	0
chr7.fa	100612904	100662230	MUC12	1.242721362	912
chr7.fa	100663364	100702140	MUC17	0	0
chr7.fa	100728786	100733889	TRIM56	20.60609042	3268
chr7.fa	100770379	100782547	SERPINE1	0	0
chr7.fa	100797686	100804557	AP1S1	49.51937729	2888
chr7.fa	100805790	100808852	VGF	0	0
chr7.fa	100813774	100823557	C7orf52	0	0
chr7.fa	100839013	100844302	MOGAT3	0	0
chr7.fa	100849258	100860984	PLOD3	2.291740093	304
chr7.fa	100861012	100867471	ZNHIT1	63.99970538	3344
chr7.fa	100875373	100882101	CLDN15	0.753238592	76
chr7.fa	100882893	100888371	FIS1	128.0779148	4256
chr7.fa	100956648	100965093	RABL5	19.49124393	2128
chr7.fa	101006122	101202304	EMID2	1.124854089	152
chr7.fa	101256605	101272576	MYL10	0	0
chr7.fa	101459292	101927250	CUX1	20.87918667	6863
chr7.fa	101928405	101962178	SH2B2	0	0
chr7.fa	101936369	101936453	MIR4285	0	0
chr7.fa	101986192	101996889	SPDYE6	0	0
chr7.fa	102036804	102067129	PRKRIP1	8.524472537	836
chr7.fa	102073996	102096666	ORAI2	0.831297862	380
chr7.fa	102097269	102105321	ALKBH4	2.225467551	152
chr7.fa	102105390	102113547	LRWD1	3.169517691	304
chr7.fa	102113613	102119381	POLR2J	31.53994793	1292
chr7.fa	102178366	102213068	POLR2J3	2.012194504	152
chr7.fa	102220093	102257205	RASA4	1.507366749	380
chr7.fa	102283077	102283238	UPK3BL	10.70768578	78

chr7.fa	102306524	102312182	POLR2J2	3.153283142	76
chr7.fa	102389399	102449672	FAM185A	2.326433101	211
chr7.fa	102453308	102715288	FBXL13	0	0
chr7.fa	102553344	102585556	LRRC17	0.764802929	76
chr7.fa	102715328	102740022	ARMC10	13.12885748	1444
chr7.fa	102740206	102789569	NAPEPLD	5.873126066	1368
chr7.fa	102781717	102782850	RPL19P12	0	0
chr7.fa	102815462	102920759	DPY19L2P2	0	0
chr7.fa	102937873	102955133	PMPCB	23.51385381	3800
chr7.fa	102956211	102985320	DNAJC2	8.202672643	696
chr7.fa	102988089	103008656	PSMC2	55.25551045	3794
chr7.fa	102993177	103086624	SLC26A5	0	0
chr7.fa	103112231	103629963	RELN	0.292221881	152
chr7.fa	103766788	103848495	ORC5	4.335069348	684
chr7.fa	103969104	104549003	LHFPL3	0	0
chr7.fa	104436954	104444539	LOC645591	0	0
chr7.fa	104535075	104567092	LOC723809	3.690357603	304
chr7.fa	104650989	104654588	LOC100216545	0.469467573	76
chr7.fa	104654637	104754532	MLL5	32.10793475	9845
chr7.fa	104756823	105029341	SRPK2	10.03206168	1748
chr7.fa	105096960	105162685	PUS7	3.883170671	608
chr7.fa	105172532	105208124	RINT1	9.028410728	1129
chr7.fa	105205580	105221976	EFCAB10	0	0
chr7.fa	105245221	105517031	ATXN7L1	3.342537952	1064
chr7.fa	105603657	105676877	CDHR3	0	0
chr7.fa	105730952	105753057	SYPL1	7.821271938	760
chr7.fa	105888732	105925638	NAMPT	22.13725301	4560
chr7.fa	106297211	106301634	FLJ36031	2.674252753	532
chr7.fa	106505924	106547592	PIK3CG	4.713356577	1140
chr7.fa	106685178	106802256	PRKAR2B	13.35191574	2198
chr7.fa	106809460	106842188	HBP1	49.63791174	4560
chr7.fa	106842975	107204959	COG5	12.36005152	3637
chr7.fa	107110502	107116125	GPR22	0	0
chr7.fa	107205033	107218968	DUS4L	5.002909765	456
chr7.fa	107220422	107263762	BCAP29	0.554420966	152
chr7.fa	107296961	107302083	LOC286002	0	0
chr7.fa	107301080	107358252	SLC26A4	0.708982767	152
chr7.fa	107384279	107402083	CBLL1	10.90694819	2128
chr7.fa	107405912	107443678	SLC26A3	0	0
chr7.fa	107531586	107561643	DLD	22.63674338	3643
chr7.fa	107564246	107643804	LAMB1	10.408125	2736
chr7.fa	107663996	107770801	LAMB4	0.288441232	76
chr7.fa	107788071	108096841	NRCAM	0.758575978	228

chr7.fa	108112071	108166638	PNPLA8	14.84282555	2356
chr7.fa	108202671	108210167	THAP5	4.616616457	760
chr7.fa	108210189	108215294	DNAJB9	1.997739083	228
chr7.fa	108524038	108524637	C7orf66	0	0
chr7.fa	109599284	109600270	EIF3IP1	0	0
chr7.fa	110303110	111202347	IMMP2L	2.225467551	152
chr7.fa	110731062	110765509	LRRN3	0	0
chr7.fa	111366164	111846462	DOCK4	1.824718822	684
chr7.fa	111846643	111983989	ZNF277	14.53370195	1756
chr7.fa	112063199	112117258	IFRD1	5.416779567	985
chr7.fa	112120908	112130943	C7orf53	0	0
chr7.fa	112405787	112430478	TMEM168	3.850034399	684
chr7.fa	112459202	112579932	C7orf60	5.145017666	912
chr7.fa	112720468	112727833	GPR85	0	0
chr7.fa	112756773	112758637	LOC401397	13.49735951	684
chr7.fa	113516882	113559082	PPP1R3A	0	0
chr7.fa	113726365	114333827	FOXP2	0.486814077	152
chr7.fa	114293400	114293510	MIR3666	0	0
chr7.fa	114562209	114659970	MDFIC	3.675012618	912
chr7.fa	115575202	115670798	TFEC	0.509720358	152
chr7.fa	115850581	115898837	TES	14.51835696	1824
chr7.fa	116139444	116148595	CAV2	2.560166128	380
chr7.fa	116164839	116201239	CAV1	0	0
chr7.fa	116312459	116438440	MET	2.78478112	836
chr7.fa	116502563	116559313	CAPZA2	47.06462454	4986
chr7.fa	116592501	116594388	ST7OT1	0	0
chr7.fa	116594515	116599867	ST7OT4	0	0
chr7.fa	116739816	116870075	ST7	2.831038465	304
chr7.fa	116752346	116785614	ST7OT2	0	0
chr7.fa	116822735	116839198	ST7OT3	0	0
chr7.fa	116916686	116963343	WNT2	0	0
chr7.fa	117003276	117067577	ASZ1	0	0
chr7.fa	117120017	117308718	CFTR	0.551307491	152
chr7.fa	117350706	117513561	CTTNBP2	11.32637777	3040
chr7.fa	117824086	117844093	NAA38	1.619896636	912
chr7.fa	117864712	117882784	ANKRD7	0	0
chr7.fa	119913722	120390387	KCND2	0.950721873	228
chr7.fa	120427374	120498177	TSPAN12	5.932726876	684
chr7.fa	120590817	120615711	ING3	5.580014621	759
chr7.fa	120628751	120937498	C7orf58	0	0
chr7.fa	120965421	120981158	WNT16	0	0
chr7.fa	120988905	121036422	FAM3C	0.664949333	76
chr7.fa	121513159	121702090	PTPRZ1	0	0

chr7.fa	121713598	121784344	AASS	4.000148379	1056
chr7.fa	121941448	121944565	FEZF1	0	0
chr7.fa	121943913	121950131	LOC154860	0	0
chr7.fa	121958478	122526813	CADPS2	9.93598873	2720
chr7.fa	122337766	122339208	RNF133	0	0
chr7.fa	122341720	122343021	RNF148	0	0
chr7.fa	122634759	122635754	TAS2R16	0	0
chr7.fa	122753588	122840025	SLC13A1	0.890231498	152
chr7.fa	123092236	123174718	IQUB	0.542856629	76
chr7.fa	123181083	123197958	NDUFA5	28.09310856	1958
chr7.fa	123249112	123277932	ASB15	0.624696547	76
chr7.fa	123295861	123304147	LMOD2	0	0
chr7.fa	123321997	123389116	WASL	27.72082589	5512
chr7.fa	123454193	123459484	HYALP1	0	0
chr7.fa	123485223	123517531	HYAL4	0	0
chr7.fa	123565286	123611461	SPAM1	0	0
chr7.fa	123670970	123673523	TMEM229A	0	0
chr7.fa	124386114	124405681	GPR37	0	0
chr7.fa	124417346	124430864	LOC154872	0	0
chr7.fa	124462440	124570037	POT1	2.752089631	532
chr7.fa	126078652	126892428	GRM8	0	0
chr7.fa	126698142	126698238	MIR592	0	0
chr7.fa	127010354	127032767	ZNF800	8.809133121	1672
chr7.fa	127220682	127225654	GCC1	13.08949426	2432
chr7.fa	127228406	127231759	ARF5	78.6486059	3876
chr7.fa	127233689	127241851	FSCN3	0	0
chr7.fa	127250346	127255780	PAX4	0	0
chr7.fa	127292202	127732659	SND1	81.90685763	12920
chr7.fa	127637562	127640130	C7orf54	0	0
chr7.fa	127667124	127671002	LRRC4	1.367927541	228
chr7.fa	127721913	127722012	MIR593	0	0
chr7.fa	127847925	127847996	MIR129-1	0	0
chr7.fa	127881331	127897682	LEP	0	0
chr7.fa	127950436	127983962	RBM28	13.23738433	1672
chr7.fa	127990379	128001739	PRRT4	0	0
chr7.fa	128032331	128050036	IMPDH1	12.27398617	1499
chr7.fa	128095884	128098472	C7orf68	2.39403999	152
chr7.fa	128116783	128142978	METTL2B	3.872940681	380
chr7.fa	128281295	128301052	FLJ45340	0.988083574	456
chr7.fa	128312346	128323309	FAM71F2	0	0
chr7.fa	128355443	128371797	FAM71F1	0.983858144	76
chr7.fa	128379346	128413477	CALU	3.547360138	988
chr7.fa	128413703	128415844	OPN1SW	0	0

chr7.fa	128432099	128462183	CCDC136	0.405418941	76
chr7.fa	128470483	128499328	FLNC	0	0
chr7.fa	128502857	128505903	ATP6V1F	76.54389671	2812
chr7.fa	128506464	128512101	LOC100130705	0.44433738	76
chr7.fa	128516919	128550773	KCP	0.940491883	228
chr7.fa	128577994	128590088	IRF5	8.260939106	1140
chr7.fa	128594234	128695227	TNPO3	30.26809334	6224
chr7.fa	128695277	128697293	TPI1P2	1.675939189	152
chr7.fa	128766325	128768050	LOC407835	0	0
chr7.fa	128784712	128809534	TSPAN33	10.3356255	1292
chr7.fa	128828713	128853385	SMO	1.809151446	304
chr7.fa	128864855	129070052	AHCYL2	6.993532334	1817
chr7.fa	129074274	129128239	FAM40B	0	0
chr7.fa	129142320	129152773	LOC100287482	0	0
chr7.fa	129251555	129396922	NRF1	4.197409268	684
chr7.fa	129410223	129410332	MIR182	0	0
chr7.fa	129414533	129414609	MIR96	0	0
chr7.fa	129414745	129414854	MIR183	0	0
chr7.fa	129472995	129592789	UBE2H	44.91832821	5510
chr7.fa	129658126	129691233	ZC3HC1	4.415352528	380
chr7.fa	129710349	129775560	KLHDC10	18.84008284	5454
chr7.fa	129804555	129845338	TMEM209	1.430419435	228
chr7.fa	129847704	129856684	C7orf45	0	0
chr7.fa	129906703	129929637	CPA2	0	0
chr7.fa	129932974	129964020	CPA4	0	0
chr7.fa	129984630	130008571	CPA5	0	0
chr7.fa	130020290	130027949	CPA1	0	0
chr7.fa	130036375	130080854	TSGA14	0.958727951	152
chr7.fa	130126046	130146079	MEST	3.069664095	380
chr7.fa	130126898	130131013	MESTIT1	0	0
chr7.fa	130135952	130136045	MIR335	0	0
chr7.fa	130146132	130353485	COPG2	15.08122879	1064
chr7.fa	130353599	130371406	TSGA13	0	0
chr7.fa	130417396	130418888	KLF14	0	0
chr7.fa	130561506	130561569	MIR29A	0	0
chr7.fa	130562218	130562298	MIR29B1	0	0
chr7.fa	130565751	130598069	LOC646329	0	0
chr7.fa	130628919	130793562	FLJ43663	0.52239665	76
chr7.fa	130794855	131181398	MKLN1	9.918642226	5092
chr7.fa	131185021	131241376	PODXL	0.282436673	76
chr7.fa	131808091	132333447	PLXNA4	0.109638803	76
chr7.fa	132469623	132766828	CHCHD3	26.53303513	1897
chr7.fa	132937823	133750513	EXOC4	20.6165428	4028

chr7.fa	133812105	133948933	LRGUK	0.626030894	76
chr7.fa	133974090	134001827	SLC35B4	1.983950836	608
chr7.fa	134127107	134143888	AKR1B1	12.08984635	760
chr7.fa	134212344	134226166	AKR1B10	0	0
chr7.fa	134233849	134264592	AKR1B15	0	0
chr7.fa	134331531	134364567	BPGM	16.05863759	1520
chr7.fa	134464164	134655480	CALD1	19.97783562	4329
chr7.fa	134671259	134820530	AGBL3	0	0
chr7.fa	134832766	134850531	TMEM140	16.70334933	1216
chr7.fa	134850968	134855532	C7orf49	22.51976567	1444
chr7.fa	134868590	134896316	WDR91	4.02505618	836
chr7.fa	134916731	134943244	STRA8	0	0
chr7.fa	135046547	135194875	CNOT4	10.03806624	2432
chr7.fa	135242662	135333499	NUP205	11.87034636	3344
chr7.fa	135347221	135361160	PL-5283	22.32806456	2508
chr7.fa	135365987	135412933	SLC13A4	0	0
chr7.fa	135414346	135433594	FAM180A	0	0
chr7.fa	136553399	136701771	CHRM2	0	0
chr7.fa	136587914	136588041	MIR490	0	0
chr7.fa	136912092	137028546	PTN	1.093941729	76
chr7.fa	137074385	137531609	DGKI	1.060805458	228
chr7.fa	137559725	137686846	CREB3L2	14.73652261	4940
chr7.fa	137761178	137803050	AKR1D1	0	0
chr7.fa	138145079	138270332	TRIM24	29.10120733	5106
chr7.fa	138279030	138363790	SVOPL	0.916918428	75
chr7.fa	138391039	138482941	ATP6V0A4	6.857651383	988
chr7.fa	138482739	138490769	TMEM213	1.566522777	228
chr7.fa	138516127	138666064	KIAA1549	1.088159561	608
chr7.fa	138710452	138720775	ZC3HAV1L	3.856706132	304
chr7.fa	138728266	138794465	ZC3HAV1	11.38375467	4028
chr7.fa	138818490	138874550	TTC26	3.897848482	377
chr7.fa	138916231	138992982	UBN2	5.967864667	3876
chr7.fa	139025897	139030541	C7orf55	55.7478843	1672
chr7.fa	139044634	139108200	LUC7L2	54.56342941	5908
chr7.fa	139102209	139112272	LOC100129148	0	0
chr7.fa	139138088	139168457	KLRG2	0	0
chr7.fa	139208674	139229731	CLEC2L	0	0
chr7.fa	139246316	139477693	HIPK2	33.7051475	22952
chr7.fa	139478047	139720125	TBXAS1	0	0
chr7.fa	139723549	139763521	PARP12	11.59169033	1976
chr7.fa	139784546	139876741	JHDM1D	8.839378308	3648
chr7.fa	139877061	139879440	LOC100134229	6.391297285	684
chr7.fa	140033552	140098311	SLC37A3	4.083767426	608

chr7.fa	140103843	140126050	RAB19	7.101614399	304
chr7.fa	140152840	140179369	MKRN1	57.89529259	9424
chr7.fa	140218220	140302342	DENND2A	1.952816085	304
chr7.fa	140372953	140394908	ADCK2	10.72436511	1140
chr7.fa	140395136	140396877	LOC100134713	0	0
chr7.fa	140396621	140406446	NDUFB2	214.7483894	3428
chr7.fa	140433813	140624564	BRAF	32.70194133	4332
chr7.fa	140705961	140714781	MRPS33	41.3751935	1520
chr7.fa	140774032	141180179	LOC100131199	1.921236551	912
chr7.fa	141251078	141354209	AGK	5.778387465	760
chr7.fa	141356528	141401953	KIAA1147	11.81452619	3876
chr7.fa	141404138	141438030	FLJ40852	0	0
chr7.fa	141408153	141431071	WEE2	0	0
chr7.fa	141438176	141450257	SSBP1	50.64000595	1430
chr7.fa	141463897	141464997	TAS2R3	0	0
chr7.fa	141478289	141479188	TAS2R4	0	0
chr7.fa	141490017	141491166	TAS2R5	0	0
chr7.fa	141536078	141541221	PRSS37	0	0
chr7.fa	141618676	141619620	OR9A4	0	0
chr7.fa	141627157	141646783	CLEC5A	0	0
chr7.fa	141672431	141673573	TAS2R38	0	0
chr7.fa	141695679	141806547	MGAM	0.521284695	152
chr7.fa	141811549	141843783	LOC93432	0	0
chr7.fa	141870970	141921088	LOC100124692	0	0
chr7.fa	141940556	141946886	MOXD2P	0	0
chr7.fa	141951963	141957878	TRYX3	0	0
chr7.fa	141968101	141972068	LOC730441	0	0
chr7.fa	142374131	142375525	MTRNR2L6	0	0
chr7.fa	142457319	142460927	PRSS1	0	0
chr7.fa	142478757	142482399	TRY6	0	0
chr7.fa	142479908	142480066	PRSS2	0	0
chr7.fa	142552792	142568847	EPHB6	2.090253773	380
chr7.fa	142568960	142583477	TRPV6	33.81967891	4401
chr7.fa	142605648	142630820	TRPV5	0	0
chr7.fa	142636603	142637957	C7orf34	0	0
chr7.fa	142638201	142659503	KEL	0	0
chr7.fa	142723287	142724219	OR9A2	0	0
chr7.fa	142749438	142750379	OR6V1	0	0
chr7.fa	142759381	142760882	OR6W1P	0	0
chr7.fa	142829174	142836834	PIP	0	0
chr7.fa	142880512	142881528	TAS2R39	0	0
chr7.fa	142919172	142920143	TAS2R40	0	0
chr7.fa	142960522	142966222	GSTK1	66.52806958	3165

chr7.fa	142982063	142985141	TMEM139	8.583183783	912
chr7.fa	142985402	143004785	CASP2	7.946922899	1444
chr7.fa	143013219	143049097	CLCN1	0	0
chr7.fa	143050493	143059840	FAM131B	0	0
chr7.fa	143078360	143088204	ZYX	11.7271265	1216
chr7.fa	143088207	143105985	EPHA1	1.508701096	228
chr7.fa	143104906	143220540	LOC285965	0	0
chr7.fa	143140546	143141502	TAS2R60	0	0
chr7.fa	143174966	143175889	TAS2R41	0	0
chr7.fa	143268890	143271244	CTAGE15P	0	0
chr7.fa	143318045	143427173	FAM115C	2.521914862	684
chr7.fa	143452182	143454843	CTAGE6P	0	0
chr7.fa	143509061	143533810	LOC154761	0	0
chr7.fa	143550049	143599172	FAM115A	17.00113099	3116
chr7.fa	143632326	143633279	OR2F2	0	0
chr7.fa	143657020	143658108	OR2F1	0	0
chr7.fa	143701090	143702025	OR6B1	0	0
chr7.fa	143747495	143748430	OR2A5	0	0
chr7.fa	143771313	143772245	OR2A25	0	0
chr7.fa	143792201	143793133	OR2A12	0	0
chr7.fa	143806676	143807632	OR2A2	0	0
chr7.fa	143826206	143827138	OR2A14	0	0
chr7.fa	143880548	143966392	CTAGE4	0	0
chr7.fa	143883176	143892791	ARHGEF35	2.049556205	228
chr7.fa	143947564	143997598	OR2A9P	0	0
chr7.fa	143948549	143996613	OR2A20P	0	0
chr7.fa	143955789	143956088	OR2A7	0	0
chr7.fa	143956722	143983096	LOC728377	2.360236546	379
chr7.fa	144015218	144015235	OR2A1	0	0
chr7.fa	144052489	144077725	ARHGEF5	5.851554131	1444
chr7.fa	144094333	144100786	NOBOX	0	0
chr7.fa	144149034	144533146	TPK1	2.771882437	304
chr7.fa	145813453	148118088	CNTNAP2	0.17079635	76
chr7.fa	146991342	147254962	MIR548I4	0	0
chr7.fa	147075109	147075213	MIR548F4	0	0
chr7.fa	147592371	147797648	MIR548F3	0	0
chr7.fa	147626685	148043927	MIR548T	0	0
chr7.fa	148287657	148312952	C7orf33	0	0
chr7.fa	148395933	148498202	CUL1	26.25171041	3788
chr7.fa	148504475	148581414	EZH2	2.517911822	304
chr7.fa	148700154	148725782	PDIA4	9.160733421	1216
chr7.fa	148766733	148787869	ZNF786	3.620304412	532
chr7.fa	148799878	148823438	ZNF425	7.353138712	1064

chr7.fa	148823508	148880118	ZNF398	23.05706253	6004
chr7.fa	148892577	148923339	ZNF282	19.07737413	3192
chr7.fa	148936742	148952700	ZNF212	7.819270418	988
chr7.fa	148959262	148982085	ZNF783	3.035193478	608
chr7.fa	148982372	148994403	LOC155060	0	0
chr7.fa	149128454	149158053	ZNF777	4.951759816	684
chr7.fa	149169884	149194898	ZNF746	7.567301323	1292
chr7.fa	149244245	149321881	ZNF767	2.846605841	304
chr7.fa	149412148	149431664	KRBA1	4.625956882	836
chr7.fa	149461453	149470295	ZNF467	2.224800377	228
chr7.fa	149473131	149531053	SSPO	0.108304457	76
chr7.fa	149535509	149564568	ZNF862	1.71530241	532
chr7.fa	149564783	149568885	LOC401431	0	0
chr7.fa	149570057	149577787	ATP6V0E2	25.90300119	2432
chr7.fa	149944301	150020758	ACTR3C	1.272744158	76
chr7.fa	150020296	150035245	LRRC61	4.516762861	380
chr7.fa	150026938	150029811	C7orf29	2.940454877	380
chr7.fa	150035407	150038763	RARRES2	4.561908251	152
chr7.fa	150065879	150071133	REPIN1	30.76713892	4636
chr7.fa	150076406	150095719	ZNF775	2.971812019	304
chr7.fa	150102840	150109558	LOC728743	1.505142838	228
chr7.fa	150130742	150145228	LOC285972	0	0
chr7.fa	150147962	150176483	GIMAP8	0	0
chr7.fa	150211945	150218161	GIMAP7	0	0
chr7.fa	150264458	150271041	GIMAP4	0	0
chr7.fa	150322464	150329680	GIMAP6	0	0
chr7.fa	150382794	150390728	GIMAP2	1.172000998	76
chr7.fa	150417495	150421368	GIMAP1	0	0
chr7.fa	150434436	150434592	GIMAP5	0	0
chr7.fa	150446824	150487837	LOC100128542	0	0
chr7.fa	150488376	150498448	TMEM176B	18.13154486	1364
chr7.fa	150497854	150502208	TMEM176A	35.82253298	1672
chr7.fa	150549573	150558379	ABP1	1.392168169	152
chr7.fa	150642049	150675014	KCNH2	0	0
chr7.fa	150688144	150711687	NOS3	0	0
chr7.fa	150709297	150721586	ATG9B	0	0
chr7.fa	150725510	150744869	ABCB8	0.360940725	76
chr7.fa	150745605	150749843	ACCN3	0	0
chr7.fa	150750899	150755052	CDK5	4.250115955	228
chr7.fa	150755299	150773614	SLC4A2	4.69334138	1216
chr7.fa	150773708	150777951	FASTK	21.90285281	1824
chr7.fa	150778172	150780620	TMUB1	8.014529788	684
chr7.fa	150783826	150841523	AGAP3	10.59915893	1976

chr7.fa	150845676	150864867	GBX1	0	0
chr7.fa	150872785	150884919	ASB10	0	0
chr7.fa	150904923	150924317	ABCF2	9.29639198	1596
chr7.fa	150929585	150935905	CHPF2	2.193888017	380
chr7.fa	150936059	150974231	SMARCD3	0.8802239	76
chr7.fa	151038858	151075532	NUB1	25.02611316	3493
chr7.fa	151078207	151107124	WDR86	0	0
chr7.fa	151106322	151110132	LOC100131176	0	0
chr7.fa	151127056	151137099	CRYGN	0	0
chr7.fa	151130575	151130725	MIR3907	0	0
chr7.fa	151163098	151217010	RHEB	26.23681021	2448
chr7.fa	151253201	151574316	PRKAG2	13.36770551	2322
chr7.fa	151653464	151717019	GALNTL5	0	0
chr7.fa	151722778	151819427	GALNT11	24.21638723	2964
chr7.fa	151832010	152133090	MLL3	19.11206714	14491
chr7.fa	152133980	152140100	FABP5P3	0	0
chr7.fa	152161209	152162630	LOC100128822	5.942956866	380
chr7.fa	152343587	152373250	XRCC2	0	0
chr7.fa	152456851	152552463	ACTR3B	1.537167154	152
chr7.fa	153584419	154685995	DPP6	0	0
chr7.fa	154720227	154741196	LOC100132707	5.068070352	304
chr7.fa	154735400	154794682	PAXIP1	9.41959664	1388
chr7.fa	154795143	154797413	LOC202781	3.721269963	380
chr7.fa	154862546	154877459	HTR5A	0	0
chr7.fa	155089486	155101945	INSIG1	18.6517176	2584
chr7.fa	155250824	155257526	EN2	0	0
chr7.fa	155293953	155326539	CNPY1	0	0
chr7.fa	155437203	155574179	RBM33	8.512241028	3952
chr7.fa	155595558	155604967	SHH	0	0
chr7.fa	156333185	156333795	NCRNA00244	0	0
chr7.fa	156431060	156433348	C7orf13	2.215237561	228
chr7.fa	156433401	156469820	RNF32	1.652143343	152
chr7.fa	156473570	156685902	LMBR1	4.147593666	912
chr7.fa	156742417	156765876	NOM1	3.6156342	988
chr7.fa	156797547	156803347	MNX1	0	0
chr7.fa	156931655	157062066	UBE3C	27.07678132	6308
chr7.fa	157129710	157210133	DNAJB6	17.4846092	2508
chr7.fa	157331750	158380482	PTPRN2	3.851591137	836
chr7.fa	157367028	157367114	MIR153-2	0	0
chr7.fa	158325410	158325505	MIR595	0	0
chr7.fa	158424003	158497520	NCAPG2	3.00895133	532
chr7.fa	158523689	158622319	ESYT2	5.3536205	1434
chr7.fa	158649269	158738883	WDR60	14.26194004	2419

chr7.fa	158801045	158818929	LOC154822	0	0
chr7.fa	158820866	158937649	VIPR2	0.857095227	152
chr8.fa	116086	117024	OR4F21	0	0
chr8.fa	158345	182199	RPL23AP53	0.596230489	152
chr8.fa	182319	197339	ZNF596	1.724642835	228
chr8.fa	356808	419875	FBXO25	31.15854723	3420
chr8.fa	441646	495331	C8orf42	13.44954542	1140
chr8.fa	614200	681226	ERICH1	9.389796235	760
chr8.fa	1244294	1250827	LOC286083	0	0
chr8.fa	1449569	1656642	DLGAP2	0	0
chr8.fa	1711870	1734736	CLN8	3.300728429	1064
chr8.fa	1765397	1765473	MIR596	0	0
chr8.fa	1772149	1906807	ARHGEF10	3.022961968	760
chr8.fa	1922044	1955109	KBTBD11	2.767879398	836
chr8.fa	1993158	2093380	MYOM2	1.34924669	304
chr8.fa	2792875	4852328	CSMD1	0.236179328	152
chr8.fa	6264113	6501140	MCPH1	7.065364653	1572
chr8.fa	6357175	6420784	ANGPT2	0.325802934	76
chr8.fa	6565878	6619021	AGPAT5	2.143627633	532
chr8.fa	6666041	6693166	XKR5	0	0
chr8.fa	6728097	6735529	DEFB1	102.2133873	2128
chr8.fa	6782216	6783598	DEFA6	0	0
chr8.fa	6793345	6795786	DEFA4	0	0
chr8.fa	6825663	6826635	DEFA10P	0	0
chr8.fa	6912829	6914259	DEFA5	0	0
chr8.fa	7118141	7143880	LOC349196	0	0
chr8.fa	7144914	7147924	FAM90A5	0	0
chr8.fa	7152536	7155546	FAM90A20	0	0
chr8.fa	7159133	7212876	FAM66B	1.180229468	76
chr8.fa	7170368	7855043	DEFB109P1B	0	0
chr8.fa	7215498	7220490	ZNF705G	0	0
chr8.fa	7286416	7740105	DEFB103A	0	0
chr8.fa	7305276	7707686	SPAG11B	0	0
chr8.fa	7413660	7440175	FAM90A7	0	0
chr8.fa	7595909	7598918	FAM90A8	0	0
chr8.fa	7618852	7621862	FAM90A9	0	0
chr8.fa	7626499	7629510	FAM90A10	0	0
chr8.fa	7718168	7721319	SPAG11A	0	0
chr8.fa	7752199	7754237	DEFB4A	0	0
chr8.fa	7783859	7809935	LOC100132396	0	0
chr8.fa	7812535	7866277	FAM66E	0	0
chr8.fa	7946463	7946611	MIR548I3	0	0
chr8.fa	8086092	8102387	FLJ10661	0	0

chr8.fa	8175258	8239257	SGK223	6.209826163	1292
chr8.fa	8559666	8561617	CLDN23	3.4635187	304
chr8.fa	8641999	8751131	MFHAS1	6.111084523	1444
chr8.fa	8860314	8890849	ERI1	3.662336327	760
chr8.fa	8993767	9008220	PPP1R3B	21.27904583	5320
chr8.fa	9413445	9639856	TNKS	17.4223397	7520
chr8.fa	9599182	9599278	MIR597	0	0
chr8.fa	9757574	9760839	LOC157627	0	0
chr8.fa	9760898	9760982	MIR124-1	0	0
chr8.fa	9911830	10286401	MSRA	14.03665788	1292
chr8.fa	10383056	10411676	PRSS55	0	0
chr8.fa	10463860	10512617	RP1L1	0.2119387	76
chr8.fa	10530147	10558103	C8orf74	0	0
chr8.fa	10581278	10588022	SOX7	0	0
chr8.fa	10622884	10697299	PINX1	4.843010577	228
chr8.fa	10682883	10682953	MIR1322	0	0
chr8.fa	10753657	11058875	XKR6	0	0
chr8.fa	10892716	10892812	MIR598	0	0
chr8.fa	11142000	11185654	MTMR9	6.162679254	2052
chr8.fa	11188495	11189695	AMAC1L2	0	0
chr8.fa	11197146	11225910	TDH	0	0
chr8.fa	11241322	11296166	C8orf12	0	0
chr8.fa	11278973	11324276	FAM167A	2.064678799	380
chr8.fa	11351521	11422108	BLK	0	0
chr8.fa	11561717	11617509	GATA4	0	0
chr8.fa	11627172	11644854	NEIL2	17.83643189	1900
chr8.fa	11660190	11696818	FDFT1	153.2872782	14516
chr8.fa	11700034	11725646	CTSB	10.59270959	1900
chr8.fa	11831446	11832108	DEFB136	0	0
chr8.fa	11839830	11842099	DEFB135	0	0
chr8.fa	11851489	11853760	DEFB134	0	0
chr8.fa	11946847	11973025	ZNF705D	0	0
chr8.fa	11973291	12008698	FAM66D	0	0
chr8.fa	11985367	11986804	LOC392196	0	0
chr8.fa	11994677	11996269	USP17L2	0	0
chr8.fa	12039613	12051624	FAM86B1	0	0
chr8.fa	12219528	12268510	FAM66A	0	0
chr8.fa	12250793	12257874	DEFB109P1	0	0
chr8.fa	12272030	12277095	FAM90A25P	0	0
chr8.fa	12283124	12293852	FAM86B2	0	0
chr8.fa	12579406	12612992	LONRF1	12.73277897	2052
chr8.fa	12584741	12584813	MIR3926-1	0	0
chr8.fa	12623571	12668910	LOC340357	0	0

chr8.fa	12803183	12887284	C8orf79	0.506606883	228
chr8.fa	12940872	13372429	DLC1	1.572749727	608
chr8.fa	13424352	13425797	C8orf48	0	0
chr8.fa	13947373	15095792	SGCZ	0	0
chr8.fa	14710947	14711019	MIR383	0	0
chr8.fa	15397730	15621995	TUSC3	7.454993827	532
chr8.fa	15965387	16050300	MSR1	0.307789257	76
chr8.fa	16850334	16859674	FGF20	0	0
chr8.fa	16884747	16980148	EFHA2	0.847754802	152
chr8.fa	17013836	17080241	ZDHHC2	14.92777894	2693
chr8.fa	17086740	17104387	CNOT7	25.16266128	4028
chr8.fa	17104401	17154305	VPS37A	7.048685322	1064
chr8.fa	17155534	17271040	MTMR7	0.416316104	76
chr8.fa	17354597	17428077	SLC7A2	6.45601309	2280
chr8.fa	17433942	17500642	PDGFRL	0	0
chr8.fa	17501303	17658426	MTUS1	36.19414848	12312
chr8.fa	17721900	17753047	FGL1	0	0
chr8.fa	17780366	17887457	PCM1	28.86547279	11400
chr8.fa	17913925	17942507	ASAH1	34.59004161	4607
chr8.fa	18027971	18081198	NAT1	3.808002485	532
chr8.fa	18248755	18258723	NAT2	0	0
chr8.fa	18384813	18871196	PSD3	13.42663914	7141
chr8.fa	19171081	19253729	SH2D4A	30.87944642	4864
chr8.fa	19261672	19540261	CSGALNACT1	16.60082705	3648
chr8.fa	19674918	19709586	INTS10	35.3508415	4028
chr8.fa	19796582	19824770	LPL	1.35324973	228
chr8.fa	20002366	20040717	SLC18A1	0	0
chr8.fa	20054704	20079207	ATP6V1B2	31.05735929	4251
chr8.fa	20103676	20112803	LZTS1	0	0
chr8.fa	20831497	20852630	LOC286114	0	0
chr8.fa	21549530	21646346	GFRA2	0	0
chr8.fa	21766384	21771205	DOK2	0	0
chr8.fa	21777180	21864096	XPO7	24.32891712	5320
chr8.fa	21882354	21894408	NPM2	1.536499981	76
chr8.fa	21900428	21906319	FGF17	0	0
chr8.fa	21911081	21940036	EPB49	28.0746501	4147
chr8.fa	21946714	21961891	FAM160B2	10.41635347	1748
chr8.fa	21964383	21966932	NUDT18	4.444930542	304
chr8.fa	21971932	21988565	HR	3.983469048	988
chr8.fa	21995533	21999448	REEP4	0.997646391	76
chr8.fa	22004343	22014344	LGI3	0	0
chr8.fa	22019184	22021991	SFTPC	0	0
chr8.fa	22022653	22069840	BMP1	0	0

chr8.fa	22077216	22089851	PHYHIP	0.506384492	76
chr8.fa	22102475	22102556	MIR320A	0	0
chr8.fa	22102619	22108680	POLR3D	10.51420554	912
chr8.fa	22132810	22213584	PIWIL2	0.824626129	152
chr8.fa	22224762	22291640	SLC39A14	2.232361674	532
chr8.fa	22298596	22398637	PPP3CC	8.497118434	836
chr8.fa	22409251	22433008	SORBS3	34.98100513	5164
chr8.fa	22436254	22455538	PDLIM2	5.191719793	1368
chr8.fa	22457114	22461662	C8orf58	4.100446757	380
chr8.fa	22462145	22477946	KIAA1967	27.18886643	5092
chr8.fa	22477984	22526661	BIN3	3.735280602	304
chr8.fa	22497884	22499722	FLJ14107	0	0
chr8.fa	22545174	22550815	EGR3	0.375173754	76
chr8.fa	22570765	22785421	PEBP4	1.890546582	76
chr8.fa	22844930	22877647	RHOBTB2	8.802906171	2280
chr8.fa	22877711	22926700	TNFRSF10B	9.506996334	1748
chr8.fa	22960327	22974950	TNFRSF10C	0	0
chr8.fa	22993104	23021540	TNFRSF10D	2.392705644	380
chr8.fa	23048970	23082680	TNFRSF10A	0	0
chr8.fa	23082734	23088439	LOC389641	2.573954375	228
chr8.fa	23101150	23119512	CHMP7	16.27813759	2496
chr8.fa	23145612	23153792	R3HCC1	13.8889902	988
chr8.fa	23154410	23261722	LOXL2	0	0
chr8.fa	23289572	23315244	ENTPD4	6.143553621	826
chr8.fa	23386363	23430063	SLC25A37	17.21885186	1444
chr8.fa	23536206	23540450	NKX3-1	772.1938738	113924
chr8.fa	23559964	23563922	NKX2-6	0	0
chr8.fa	23699434	23712320	STC1	2.179654988	380
chr8.fa	24151580	24212726	ADAM28	1.574751247	228
chr8.fa	24241798	24263526	ADAMDEC1	0	0
chr8.fa	24298509	24366275	ADAM7	0	0
chr8.fa	24771274	24776606	NEFM	0	0
chr8.fa	24808469	24814131	NEFL	1.412850539	228
chr8.fa	25042287	25270619	DOCK5	12.28666246	4174
chr8.fa	25276774	25282556	GNRH1	0	0
chr8.fa	25285364	25315984	KCTD9	3.289164092	503
chr8.fa	25316513	25365425	CDCA2	0.454344979	76
chr8.fa	25701573	25902392	EBF2	0	0
chr8.fa	26149007	26230195	PPP2R2A	13.91945778	2508
chr8.fa	26240523	26270644	BNIP3L	42.9786332	6764
chr8.fa	26362196	26371483	PNMA2	0	0
chr8.fa	26371709	26515693	DPYSL2	1.681054184	380
chr8.fa	26605667	26722922	ADRA1A	0	0

chr8.fa	27093814	27115903	STMN4	0	0
chr8.fa	27142404	27168834	TRIM35	8.396820057	1596
chr8.fa	27168999	27316903	PTK2B	14.57595625	3108
chr8.fa	27317278	27336813	CHRNA2	8.727293203	1596
chr8.fa	27348645	27402486	EPHX2	79.39650711	7840
chr8.fa	27454451	27472327	CLU	2.068014665	304
chr8.fa	27491577	27534286	SCARA3	0	0
chr8.fa	27559190	27559284	MIR3622B	0	0
chr8.fa	27590833	27630170	CCDC25	13.81382201	2261
chr8.fa	27632058	27662424	ESCO2	0	0
chr8.fa	27667138	27695349	PBK	0	0
chr8.fa	27727399	27850369	SCARA5	0	0
chr8.fa	27743556	27743633	MIR4287	0	0
chr8.fa	27879481	27941388	C8orf80	0.869549128	152
chr8.fa	27950584	28048669	ELP3	12.60156823	1824
chr8.fa	28174649	28200868	PNOC	0	0
chr8.fa	28203102	28243977	ZNF395	32.0294307	6916
chr8.fa	28285929	28347784	FBXO16	3.924535412	228
chr8.fa	28351722	28431785	FZD3	0.245074972	152
chr8.fa	28362633	28362699	MIR4288	0	0
chr8.fa	28559153	28611202	EXTL3	3.833799851	1064
chr8.fa	28625175	28747698	INTS9	8.462870208	1064
chr8.fa	28747911	28910242	HMBOX1	9.608629059	1520
chr8.fa	28924795	29120610	KIF13B	51.81000543	20422
chr8.fa	29193617	29208185	DUSP4	2.402490851	456
chr8.fa	29578776	29605625	C8orf75	0	0
chr8.fa	29779029	29811123	LOC286135	0	0
chr8.fa	29814788	29814864	MIR3148	0	0
chr8.fa	29920631	29940649	TMEM66	57.27949168	5092
chr8.fa	29952922	29995222	LEPROTL1	3.222446768	456
chr8.fa	29989187	30002200	MBOAT4	0	0
chr8.fa	30013813	30041155	DCTN6	25.49802704	1291
chr8.fa	30241944	30429734	RBPMS	11.23208395	1748
chr8.fa	30436031	30515738	GTF2E2	11.00368831	760
chr8.fa	30535580	30585486	GSR	63.75440801	9059
chr8.fa	30601690	30624520	UBXN8	1.1744473	76
chr8.fa	30643126	30670352	PPP2CB	51.47730837	4560
chr8.fa	30689060	30706533	TEX15	0	0
chr8.fa	30853321	30890777	PURG	0	0
chr8.fa	30891232	31031277	WRN	6.068163044	1444
chr8.fa	31497268	32622558	NRG1	0	0
chr8.fa	33228344	33330664	FUT10	2.859059741	456
chr8.fa	33342685	33358778	MAK16	7.067143782	906

chr8.fa	33360947	33370703	C8orf41	10.40456674	684
chr8.fa	33405273	33424643	RNF122	0	0
chr8.fa	33448854	33457439	DUSP26	2.053781636	152
chr8.fa	35092975	35652181	UNC5D	0.463685405	152
chr8.fa	36641842	36793643	KCNU1	0	0
chr8.fa	37553301	37556396	ZNF703	10.8842643	1064
chr8.fa	37594097	37615319	ERLIN2	1.929020239	532
chr8.fa	37604074	37605564	LOC728024	0	0
chr8.fa	37620101	37637286	PROSC	36.72188252	4242
chr8.fa	37654401	37701397	GPR124	0	0
chr8.fa	37701505	37707431	BRF2	12.45301099	1060
chr8.fa	37716465	37757015	RAB11FIP1	124.353309	43928
chr8.fa	37791800	37797647	GOT1L1	0	0
chr8.fa	37820514	37824184	ADRB3	0	0
chr8.fa	37888020	37917883	EIF4EBP1	11.80563055	456
chr8.fa	37963011	37997228	ASH2L	21.03219173	2736
chr8.fa	38000218	38008600	STAR	0	0
chr8.fa	38020859	38034026	LSM1	18.39152003	760
chr8.fa	38034106	38068537	BAG4	1.549176272	152
chr8.fa	38089009	38120287	DDHD2	8.966363616	1976
chr8.fa	38120650	38126738	PPAPDC1B	7.726533338	912
chr8.fa	38132561	38239790	WHSC1L1	22.76884369	7220
chr8.fa	38243959	38266062	LETM2	0	0
chr8.fa	38268656	38326352	FGFR1	0	0
chr8.fa	38368352	38386180	C8orf86	0	0
chr8.fa	38457693	38458775	RNF5P1	0	0
chr8.fa	38585704	38710546	TACC1	31.90689322	11891
chr8.fa	38758753	38831430	PLEKHA2	7.191015614	1824
chr8.fa	38831668	38846181	HTRA4	0	0
chr8.fa	38846327	38854041	TM2D2	3.249356089	532
chr8.fa	38854505	38962779	ADAM9	5.774162035	1064
chr8.fa	38965050	39142436	ADAM32	0	0
chr8.fa	39172182	39260375	ADAM5P	0	0
chr8.fa	39308564	39380470	ADAM3A	0	0
chr8.fa	39442087	39587583	ADAM18	0	0
chr8.fa	39601255	39695779	ADAM2	0	0
chr8.fa	39771328	39785947	IDO1	0	0
chr8.fa	39792474	39873910	IDO2	0	0
chr8.fa	40010989	40012821	C8orf4	1.844066846	152
chr8.fa	40388111	40755343	ZMAT4	0	0
chr8.fa	41119476	41166990	SFRP1	1.513371308	304
chr8.fa	41348081	41368499	GOLGA7	24.28399412	2280
chr8.fa	41386725	41402565	GINS4	0	0

chr8.fa	41435707	41482520	AGPAT6	6.386182291	1812
chr8.fa	41503829	41504878	NKX6-3	0	0
chr8.fa	41510744	41754280	ANK1	0.546414887	228
chr8.fa	41517959	41518026	MIR486	0	0
chr8.fa	41786997	41909505	MYST3	17.29313048	7220
chr8.fa	42010464	42028701	AP3M2	4.605274512	760
chr8.fa	42032236	42065194	PLAT	1.59810231	228
chr8.fa	42128820	42190171	IKBKB	8.98371012	1672
chr8.fa	42196030	42229313	POLB	22.57469627	1278
chr8.fa	42231586	42234674	DKK4	0	0
chr8.fa	42249346	42263415	VDAC3	36.45479083	2349
chr8.fa	42273993	42330172	SLC20A2	4.283697008	684
chr8.fa	42396298	42408140	C8orf40	18.57321355	1216
chr8.fa	42552562	42592209	CHRNB3	0	0
chr8.fa	42607780	42623929	CHRNA6	0	0
chr8.fa	42691817	42698474	THAP1	2.339776566	228
chr8.fa	42704780	42751866	RNF170	1.85096097	456
chr8.fa	42752033	42885682	HOOK3	13.26651756	8614
chr8.fa	42911442	42940932	FNTA	28.87814908	2201
chr8.fa	42948657	42978323	SGK196	4.186289714	304
chr8.fa	42995592	43057970	HGSNAT	1.945032397	456
chr8.fa	43147585	43218328	POTEA	0	0
chr8.fa	47752508	47767407	NCRNA00293	0	0
chr8.fa	48100930	48104439	LOC100287846	0	0
chr8.fa	48173542	48648475	KIAA0146	25.19513038	3648
chr8.fa	48649476	48650726	CEBPD	6.755351486	380
chr8.fa	48685669	48872743	PRKDC	9.384236458	5700
chr8.fa	48873494	48890068	MCM4	9.398024705	1444
chr8.fa	48920995	48974454	UBE2V2	16.63552005	1140
chr8.fa	49627474	49647870	EFCAB1	0.917807993	152
chr8.fa	49830239	49833988	SNAI2	0.805500496	76
chr8.fa	49984903	49988642	C8orf22	0	0
chr8.fa	50824597	51705427	SNTG1	0	0
chr8.fa	52232137	52722005	PXDNL	0.3516003	76
chr8.fa	52730140	52811735	PCMTD1	35.53164545	6688
chr8.fa	53023392	53322439	ST18	0	0
chr8.fa	53446597	53478021	FAM150A	1.443318118	76
chr8.fa	53535018	53627026	RB1CC1	14.45030529	4332
chr8.fa	53852468	53853454	NPBWR1	0	0
chr8.fa	54138276	54164194	OPRK1	0	0
chr8.fa	54628115	54755850	ATP6V1H	23.08174794	2356
chr8.fa	54764368	54871863	RGS20	0	0
chr8.fa	54879116	54935008	TCEA1	9.129376278	1140

chr8.fa	54958938	55014577	LYPLA1	38.1709828	4418
chr8.fa	55047781	55061074	MRPL15	23.19272109	1824
chr8.fa	55370495	55373456	SOX17	0	0
chr8.fa	55528627	55543394	RP1	0	0
chr8.fa	56015017	56438710	XKR4	0	0
chr8.fa	56361757	56367881	SBF1P1	0	0
chr8.fa	56651320	56676539	TMEM68	2.992716781	304
chr8.fa	56685791	56738005	TGS1	10.23221365	1672
chr8.fa	56792386	56923940	LYN	3.215107862	447
chr8.fa	56980739	56987140	RPS20	945.5241482	56164
chr8.fa	56986398	56986460	SNORD54	0	0
chr8.fa	57025501	57026541	MOS	0	0
chr8.fa	57073468	57123859	PLAG1	0.69096909	228
chr8.fa	57124315	57131176	CHCHD7	24.42454529	1900
chr8.fa	57212570	57233241	SDR16C5	0	0
chr8.fa	57353513	57359282	PENK	0	0
chr8.fa	57870488	57906427	IMPAD1	1.870976167	608
chr8.fa	58192102	58197290	C8orf71	0	0
chr8.fa	58907113	59062277	FAM110B	1.934802407	380
chr8.fa	59323823	59364060	UBXN2B	12.67295577	2888
chr8.fa	59402737	59412720	CYP7A1	0	0
chr8.fa	59465728	59495419	SDCBP	26.24148042	2557
chr8.fa	59496064	59572404	NSMAF	11.84877442	2015
chr8.fa	59717977	60031767	TOX	2.045775557	380
chr8.fa	61101423	61193954	CA8	0	0
chr8.fa	61429559	61533629	RAB2A	103.0593629	5320
chr8.fa	61591339	61779465	CHD7	15.21221713	7144
chr8.fa	61878680	61880307	LOC100130298	0	0
chr8.fa	62200525	62413114	CLVS1	0	0
chr8.fa	62414205	62627199	ASPH	0.308901212	152
chr8.fa	63161501	63903628	NKAIN3	0	0
chr8.fa	63890420	63897460	LOC643763	0	0
chr8.fa	63927639	63951610	GGH	0	0
chr8.fa	63972048	63998612	TTPA	0	0
chr8.fa	64081121	64125346	YTHDF3	18.26586907	4234
chr8.fa	65285775	65291386	LOC100130155	0	0
chr8.fa	65291706	65291814	MIR124-2	0	0
chr8.fa	65486866	65489820	LOC401463	0	0
chr8.fa	65492814	65496186	BHLHE22	0	0
chr8.fa	65508529	65711348	CYP7B1	1.411516193	152
chr8.fa	66439243	66474901	LOC286186	0	0
chr8.fa	66514691	66546452	ARMC1	8.889861084	1216
chr8.fa	66556888	66622798	MTFR1	8.658129577	1064

chr8.fa	66629905	66753755	PDE7A	4.385774514	988
chr8.fa	66933791	67012755	DNAJC5B	0	0
chr8.fa	67039278	67087718	TRIM55	0	0
chr8.fa	67088612	67090846	CRH	0	0
chr8.fa	67341263	67342968	RRS1	17.83309603	1368
chr8.fa	67344718	67381044	ADHFE1	7.135195452	608
chr8.fa	67405491	67430759	C8orf46	0	0
chr8.fa	67474410	67525480	MYBL1	0.325802934	76
chr8.fa	67542488	67579452	VCPIP1	9.716043951	3572
chr8.fa	67588454	67593235	C8orf44	0	0
chr8.fa	67624891	67774257	SGK3	1.172668172	228
chr8.fa	67679632	67680240	PTTG3P	0	0
chr8.fa	67782984	67834164	C8orf45	1.681721357	380
chr8.fa	67834284	67837777	SNHG6	0	0
chr8.fa	67834709	67834784	SNORD87	0	0
chr8.fa	67858736	67874825	TCF24	0	0
chr8.fa	67900367	67940786	LRRC67	0	0
chr8.fa	67955315	67974562	COPS5	32.91810546	2204
chr8.fa	67976603	68108849	CSPP1	8.955911235	1900
chr8.fa	68109897	68255912	ARFGEF1	25.68972815	8331
chr8.fa	68334405	68658620	CPA6	0	0
chr8.fa	68864603	69143897	PREX2	0	0
chr8.fa	69242957	69731258	C8orf34	0.629589151	76
chr8.fa	70378859	70573147	SULF1	0	0
chr8.fa	70584568	70747299	SLCO5A1	1.783798863	304
chr8.fa	70964023	70983562	PRDM14	0	0
chr8.fa	71024267	71316020	NCOA2	53.74658696	14880
chr8.fa	71485453	71520694	TRAM1	21.27037258	2964
chr8.fa	71549501	71581447	LACTB2	6.53006932	456
chr8.fa	71581600	71648177	XKR9	1.065030888	152
chr8.fa	72109668	72274467	EYA1	0	0
chr8.fa	72753777	72756731	MSC	0	0
chr8.fa	72755358	72968547	LOC100132891	0	0
chr8.fa	72933486	72987819	TRPA1	0	0
chr8.fa	73117534	73163869	LOC392232	0	0
chr8.fa	73449626	73850584	KCNB2	0.471913875	76
chr8.fa	73921097	73959987	TERF1	2.855056702	380
chr8.fa	73976778	74005507	C8orf84	0	0
chr8.fa	74153564	74171737	LOC100130301	0	0
chr8.fa	74202874	74205869	RPL7	100.9659957	3800
chr8.fa	74207265	74237516	RDH10	9.09468327	1444
chr8.fa	74332604	74659943	STAU2	6.834077928	1814
chr8.fa	74702840	74791110	UBE2W	5.021590616	912

chr8.fa	74858634	74884346	TCEB1	54.1546746	1595
chr8.fa	74888377	74895018	TMEM70	11.64039398	1140
chr8.fa	74903564	74941307	LY96	0	0
chr8.fa	75146939	75233562	JPH1	1.159547098	228
chr8.fa	75262618	75279335	GDAP1	1.738431083	304
chr8.fa	75512101	75670587	FLJ39080	2.386033911	228
chr8.fa	75617928	75617982	MIR2052	0	0
chr8.fa	75736772	75767264	PI15	1.004318123	304
chr8.fa	75896708	75946793	CRISPLD1	1.171333825	228
chr8.fa	76452203	76479061	HNF4G	2.890639275	532
chr8.fa	77523114	77595510	LOC100192378	0	0
chr8.fa	77593515	77779521	ZFHX4	0.605348523	380
chr8.fa	77892494	77913280	PEX2	10.04940818	2052
chr8.fa	79428336	79515485	PKIA	13.92991016	1368
chr8.fa	79578282	79631997	FAM164A	2.5234716	380
chr8.fa	79645007	79717758	IL7	0.814173749	76
chr8.fa	80523049	80578410	STMN2	0	0
chr8.fa	80676245	80680098	HEY1	1.46644679	152
chr8.fa	80831095	80942506	MRPS28	19.12496582	608
chr8.fa	80947105	81083836	TPD52	86.71717672	17504
chr8.fa	81398448	81434610	ZBTB10	13.66415282	3724
chr8.fa	81540686	81787016	ZNF704	9.986471506	6460
chr8.fa	81880046	82024303	PAG1	3.618525284	1748
chr8.fa	82192785	82197008	FABP5	0	0
chr8.fa	82352564	82359719	PMP2	0	0
chr8.fa	82370618	82373758	FABP9	0	0
chr8.fa	82390732	82395473	FABP4	0	0
chr8.fa	82437281	82443550	FABP12	0	0
chr8.fa	82569151	82598589	IMPA1	4.698901157	760
chr8.fa	82605891	82607207	SLC10A5	0	0
chr8.fa	82613566	82633539	ZFAND1	10.77484789	1110
chr8.fa	82644688	82671748	CHMP4C	10.07698468	836
chr8.fa	82711818	82754521	SNX16	2.934227927	456
chr8.fa	85095453	85834078	RALYL	0	0
chr8.fa	86019323	86058314	LRRCC1	4.797865188	836
chr8.fa	86089619	86126287	E2F5	2.431846474	152
chr8.fa	86126754	86132643	C8orf59	40.93841742	1064
chr8.fa	86157716	86196302	CA13	5.261328201	912
chr8.fa	86240458	86290342	CA1	0	0
chr8.fa	86351056	86361267	CA3	0.965177293	76
chr8.fa	86376131	86393721	CA2	22.31916892	1672
chr8.fa	86566828	86840171	REXO1L2P	0	0
chr8.fa	86568695	86789306	REXO1L1	0	0

chr8.fa	87060691	87081851	PSKH2	0	0
chr8.fa	87111139	87166454	ATP6V0D2	7.131637195	760
chr8.fa	87226288	87242604	SLC7A13	0.906243656	76
chr8.fa	87354994	87480178	WWP1	69.10113439	12755
chr8.fa	87484578	87521009	FAM82B	11.03237676	1520
chr8.fa	87526656	87573726	CPNE3	31.7950305	6954
chr8.fa	87586163	87755903	CNGB3	0	0
chr8.fa	87878676	88394955	CNBD1	0	0
chr8.fa	88882971	88886296	DCAF4L2	0	0
chr8.fa	89049460	89339717	MMP16	0	0
chr8.fa	90769975	90803292	RIPK2	13.07681797	1520
chr8.fa	90914096	90940095	OSGIN2	11.63572376	2356
chr8.fa	90945564	90996899	NBN	20.48244098	4256
chr8.fa	91013580	91064227	DECR1	57.63331589	3242
chr8.fa	91070838	91095107	CALB1	15.35921764	1748
chr8.fa	91634223	91658133	TMEM64	9.469634633	2052
chr8.fa	91803921	91971630	NECAB1	0.737004043	152
chr8.fa	91972970	91997485	LOC100127983	3.380344436	76
chr8.fa	92006502	92053203	TMEM55A	6.737337808	728
chr8.fa	92082424	92099323	OTUD6B	2.050000987	304
chr8.fa	92114847	92231464	LRRC69	0	0
chr8.fa	92261516	92410378	SLC26A7	2.05266968	532
chr8.fa	92967195	93115454	RUNX1T1	0	0
chr8.fa	93895758	93978372	C8orf83	11.91704848	2204
chr8.fa	94358695	94712661	LOC642924	0	0
chr8.fa	94712773	94740671	FAM92A1	1.510480224	76
chr8.fa	94743731	94753224	RBM12B	2.650679298	608
chr8.fa	94752339	94753047	C8orf39	2.38381	76
chr8.fa	94767072	94831460	TMEM67	0.966066857	152
chr8.fa	94929083	94938296	PDP1	4.7776276	988
chr8.fa	95139394	95229531	CDH17	0	0
chr8.fa	95261485	95274547	GEM	0	0
chr8.fa	95384188	95487310	RAD54B	2.778998952	380
chr8.fa	95500600	95565688	KIAA1429	15.79332503	4256
chr8.fa	95649513	95651695	LOC100288748	1.548509099	152
chr8.fa	95653364	95719694	ESRP1	32.91321286	5615
chr8.fa	95732103	95806076	DPY19L4	1.084378912	304
chr8.fa	95835534	95892452	INTS8	12.3231346	1720
chr8.fa	95892722	95907482	CCNE2	0	0
chr8.fa	95938200	95961615	TP53INP1	29.85288919	7587
chr8.fa	96037221	96070938	C8orf38	2.831038465	228
chr8.fa	96085139	96085224	MIR3150B	0	0
chr8.fa	96145949	96168913	PLEKHF2	5.130339854	684

chr8.fa	96257141	96281462	C8orf37	0	0
chr8.fa	96959215	96960576	LOC100500773	0	0
chr8.fa	97154558	97173020	GDF6	0	0
chr8.fa	97239304	97247862	UQCRB	41.72590424	8505
chr8.fa	97251645	97273796	MTERFD1	1.202023794	76
chr8.fa	97274167	97346774	PTDSS1	9.370003428	1055
chr8.fa	97505882	97624037	SDC2	3.394799856	532
chr8.fa	97657499	98155722	PGCP	4.457162051	380
chr8.fa	98285714	98290176	TSPYL5	11.74002518	2356
chr8.fa	98656407	98742488	MTDH	4.188513625	1444
chr8.fa	98787809	98864830	LAPTM4B	4.531218281	456
chr8.fa	98881311	99048946	MATN2	0.4114235	76
chr8.fa	99053942	99057773	RPL30	1532.120433	34791
chr8.fa	99054314	99054445	SNORA72	0	0
chr8.fa	99076750	99105838	C8orf47	3.271372806	228
chr8.fa	99114567	99129418	HRSP12	36.77925942	1672
chr8.fa	99129521	99172069	POP1	1.793806462	380
chr8.fa	99204387	99306621	NIPAL2	6.745788669	684
chr8.fa	99439250	99443023	KCNS2	0	0
chr8.fa	99466861	99837909	STK3	7.177004976	912
chr8.fa	99956631	99964326	OSR2	0.903352572	76
chr8.fa	100025494	100889808	VPS13B	7.298875288	4712
chr8.fa	100548864	100548958	MIR599	0	0
chr8.fa	100549014	100549089	MIR875	0	0
chr8.fa	100890223	100906242	COX6C	78.91147216	3268
chr8.fa	100973276	101118344	RGS22	0	0
chr8.fa	101145588	101158099	FBXO43	0	0
chr8.fa	101162839	101166230	POLR2K	33.07244487	1444
chr8.fa	101170263	101254130	SPAG1	8.1588616	1444
chr8.fa	101269288	101322327	RNF19A	26.29907971	5396
chr8.fa	101533000	101572012	ANKRD46	6.081951291	760
chr8.fa	101585112	101661893	SNX31	103.9484825	11475
chr8.fa	101715144	101734315	PABPC1	821.4472713	105640
chr8.fa	101930804	101965623	YWHAZ	81.93932673	13452
chr8.fa	102064282	102088479	FLJ42969	0	0
chr8.fa	102209266	102217960	ZNF706	10.6374102	1368
chr8.fa	102381121	102381823	NACAP1	4.80853996	152
chr8.fa	102504668	102681952	GRHL2	47.04283021	11061
chr8.fa	102698770	103137135	NCALD	2.190774542	442
chr8.fa	103216729	103251346	RRM2B	8.125725329	1900
chr8.fa	103265569	103424495	UBR5	24.64916028	10412
chr8.fa	103563848	103573245	ODF1	0	0
chr8.fa	103661005	103667983	KLF10	3.456402186	532

chr8.fa	103838536	103876397	AZIN1	52.08910624	10184
chr8.fa	104033248	104085285	ATP6V1C1	17.48260768	4484
chr8.fa	104145191	104153570	C8orf56	0	0
chr8.fa	104152921	104242533	BAALC	0	0
chr8.fa	104166842	104166917	MIR3151	0	0
chr8.fa	104177737	104184368	LOC100499183	0	0
chr8.fa	104310661	104345094	FZD6	2.148742628	380
chr8.fa	104383786	104395217	CTHRC1	0	0
chr8.fa	104410866	104420012	SLC25A32	5.267332761	608
chr8.fa	104426942	104455680	DCAF13	13.92991016	1368
chr8.fa	104512976	105266656	RIMS2	0.92559168	304
chr8.fa	105352054	105368916	TM7SF4	0	0
chr8.fa	105391652	105479277	DPYS	16.71869432	1596
chr8.fa	105501459	105601252	LRP12	0.814840922	152
chr8.fa	106331147	106816767	ZFPM2	0	0
chr8.fa	107282406	107764921	OXR1	13.43887065	3268
chr8.fa	107771711	107782472	ABRA	0	0
chr8.fa	108261710	108510254	ANGPT1	1.558516698	304
chr8.fa	108911544	109095913	RSPO2	0.536629679	76
chr8.fa	109213972	109260959	EIF3E	159.3901343	10808
chr8.fa	109455853	109499136	TTC35	16.11356819	897
chr8.fa	109795346	109799770	TMEM74	0	0
chr8.fa	110099676	110131812	TRHR	0	0
chr8.fa	110253148	110346350	NUDCD1	3.726384958	684
chr8.fa	110346552	110358189	ENY2	8.554050551	1117
chr8.fa	110374706	110543500	PKHD1L1	0	0
chr8.fa	110551929	110577391	EBAG9	3.632758313	304
chr8.fa	110586405	110704020	SYBU	0.810170709	152
chr8.fa	110979233	110986959	KCNV1	0	0
chr8.fa	113235159	114449242	CSMD3	0	0
chr8.fa	113655722	113655812	MIR2053	0	0
chr8.fa	116420724	116681228	TRPS1	9.305287624	4180
chr8.fa	117657055	117768062	EIF3H	193.5413983	11096
chr8.fa	117778742	117786921	UTP23	5.952964464	988
chr8.fa	117858173	117879000	RAD21	30.9272605	4859
chr8.fa	117886663	117889107	NCRNA00255	0	0
chr8.fa	117886967	117887039	MIR3610	0	0
chr8.fa	117950464	117956239	C8orf85	0.946274051	76
chr8.fa	117962512	118188953	SLC30A8	0.289108406	76
chr8.fa	118532965	118552501	MED30	6.955503459	304
chr8.fa	118811602	119124058	EXT1	5.025816046	760
chr8.fa	119201695	119634184	SAMD12	6.798940137	3192
chr8.fa	119935796	119964383	TNFRSF11B	2.161418919	228

chr8.fa	120079424	120119202	COLEC10	0	0
chr8.fa	120220610	120257914	MAL2	33.56370677	4256
chr8.fa	120428552	120436678	NOV	0	0
chr8.fa	120569319	120651106	ENPP2	1.008765945	152
chr8.fa	120743014	120845074	TAF2	11.78405862	2660
chr8.fa	120846181	120868170	DSCC1	0.744787731	76
chr8.fa	120885900	121063157	DEPTOR	17.91982855	2120
chr8.fa	121137352	121384273	COL14A1	0	0
chr8.fa	121408083	121457647	MRPL13	21.09712993	1053
chr8.fa	121457666	121535875	MTBP	0.552419446	76
chr8.fa	121547985	121824309	SNTB1	8.493337786	1900
chr8.fa	122625271	122653429	HAS2	0	0
chr8.fa	122651586	122657564	HAS2-AS1	0	0
chr8.fa	123793901	123986755	ZHX2	13.958821	2736
chr8.fa	124025568	124054648	DERL1	2.138735029	304
chr8.fa	124084920	124164392	WDR67	3.376563787	532
chr8.fa	124194752	124222318	FAM83A	0	0
chr8.fa	124213412	124214983	LOC100131726	0	0
chr8.fa	124232231	124253617	C8orf76	3.94277148	228
chr8.fa	124260696	124286547	ZHX1	10.78485549	2432
chr8.fa	124332091	124408705	ATAD2	6.379065776	1596
chr8.fa	124428965	124454260	WDYHV1	11.28456825	684
chr8.fa	124515358	124553446	FBXO32	8.924998875	608
chr8.fa	124657915	124665190	KLHL38	0	0
chr8.fa	124693034	124749647	ANXA13	0	0
chr8.fa	124780882	124827690	FAM91A1	11.85055355	2837
chr8.fa	124864227	125132302	FER1L6	0.279323198	76
chr8.fa	125323159	125384940	TMEM65	1.49735915	290
chr8.fa	125463048	125465266	TRMT12	9.140273441	912
chr8.fa	125487008	125500734	RNF139	1.060583067	152
chr8.fa	125500860	125551329	TATDN1	9.674012037	445
chr8.fa	125551343	125562227	NDUFB9	39.96234296	1292
chr8.fa	125563028	125740730	MTSS1	10.75305356	2421
chr8.fa	125954250	125963337	LOC157381	0.472136266	76
chr8.fa	125985539	125991630	ZNF572	2.580403716	380
chr8.fa	126010720	126034525	SQLE	13.00565283	1748
chr8.fa	126036503	126104061	KIAA0196	17.04383008	3192
chr8.fa	126104083	126379367	NSMCE2	11.15624859	608
chr8.fa	126442563	126450644	TRIB1	24.19859595	3952
chr8.fa	127564683	127570711	FAM84B	24.66962026	6080
chr8.fa	128427857	128429441	POU5F1B	0	0
chr8.fa	128455595	128494384	LOC727677	0	0
chr8.fa	128748315	128753680	MYC	0.714320153	76

chr8.fa	128806779	129113499	PVT1	2.667358629	228
chr8.fa	128808208	128808274	MIR1204	0	0
chr8.fa	128972879	128972941	MIR1205	0	0
chr8.fa	129021144	129021202	MIR1206	0	0
chr8.fa	129061398	129061484	MIR1207	0	0
chr8.fa	129162362	129162434	MIR1208	0	0
chr8.fa	130228713	130253486	LOC728724	0	0
chr8.fa	130760442	130799134	GSDMC	0	0
chr8.fa	130853716	130952000	FAM49B	6.157341868	608
chr8.fa	131064353	131414217	ASAP1	9.757631083	2651
chr8.fa	131307601	131308779	ASAP1-IT	0	0
chr8.fa	131792547	132052835	ADCY8	0	0
chr8.fa	132916359	133025771	EFR3A	25.46822663	6065
chr8.fa	133036467	133071627	OC90	0	0
chr8.fa	133073733	133117512	HHLA1	0	0
chr8.fa	133141256	133493004	KCNQ3	0	0
chr8.fa	133572745	133573726	HPYR1	0	0
chr8.fa	133584447	133687813	LRRC6	3.205100264	228
chr8.fa	133722192	133772914	TMEM71	0	0
chr8.fa	133787604	133861052	PHF20L1	10.93452469	3192
chr8.fa	133879205	134147143	TG	0.199929582	76
chr8.fa	134048973	134115310	SLA	0	0
chr8.fa	134203312	134241571	WISP1	0	0
chr8.fa	134249414	134309547	NDRG1	430.8108877	67375
chr8.fa	134467091	134584183	ST3GAL1	17.45703271	5472
chr8.fa	135490031	135725292	ZFAT	5.79573397	1216
chr8.fa	135610314	135612932	ZFAT-AS1	0	0
chr8.fa	135812763	135812850	MIR30B	0	0
chr8.fa	135817119	135817188	MIR30D	0	0
chr8.fa	136246374	136311962	LOC286094	0	0
chr8.fa	136469716	136659848	KHDRBS3	2.592190444	228
chr8.fa	139142266	139509065	FAM135B	0.242851061	76
chr8.fa	139600478	139926236	COL22A1	0.266424515	76
chr8.fa	140624804	140715299	KCNK9	0	0
chr8.fa	140742586	141468678	TRAPPC9	10.30693705	2131
chr8.fa	141521401	141527252	CHRAC1	12.84530886	1444
chr8.fa	141541264	141645646	EIF2C2	11.58968881	1824
chr8.fa	141668481	142011412	PTK2	49.11173444	10313
chr8.fa	142138720	142205900	DENND3	2.173650429	532
chr8.fa	142220870	142238673	SLC45A4	3.003836335	456
chr8.fa	142350648	142354720	LOC731779	0	0
chr8.fa	142366587	142377365	GPR20	0	0
chr8.fa	142432007	142441620	PTP4A3	12.68896793	760

chr8.fa	142443929	142517330	FLJ43860	0	0
chr8.fa	143279717	143290364	NCRNA00051	0	0
chr8.fa	143293441	143484543	TSNARE1	2.661798852	228
chr8.fa	143545377	143626368	BAI1	0	0
chr8.fa	143692410	143695833	ARC	0	0
chr8.fa	143738874	143751401	JRK	5.931837311	2432
chr8.fa	143751726	143764145	PSCA	129.0006154	7448
chr8.fa	143781529	143785584	LY6K	0	0
chr8.fa	143808621	143818350	C8orf55	5.972312488	608
chr8.fa	143822362	143823829	SLURP1	0	0
chr8.fa	143831628	143833952	LYPD2	0	0
chr8.fa	143845756	143859639	LYNX1	0	0
chr8.fa	143866298	143868008	LY6D	0	0
chr8.fa	143916217	143928262	GML	0	0
chr8.fa	143953773	143961236	CYP11B1	0	0
chr8.fa	143991975	143999259	CYP11B2	0	0
chr8.fa	144063448	144099807	LOC100133669	0	0
chr8.fa	144099902	144103827	LY6E	15.80933719	836
chr8.fa	144120679	144135720	C8orf31	0	0
chr8.fa	144239331	144242053	LY6H	0	0
chr8.fa	144295068	144299044	GPIHBP1	0	0
chr8.fa	144329109	144344875	ZFP41	1.412628148	304
chr8.fa	144349607	144359101	GLI4	7.551066774	456
chr8.fa	144373559	144382120	ZNF696	2.886636235	608
chr8.fa	144391528	144417050	TOP1MT	12.40163865	1064
chr8.fa	144448793	144450805	C8orf51	1.679275055	152
chr8.fa	144451025	144466390	RHPN1	1.364814066	228
chr8.fa	144510230	144512602	MAFA	0	0
chr8.fa	144519825	144623620	ZC3H3	6.189143793	912
chr8.fa	144635557	144645231	GSDMD	12.83685799	1368
chr8.fa	144648363	144654928	C8orf73	1.560073435	228
chr8.fa	144656955	144660513	NAPRT1	23.93172665	1824
chr8.fa	144661867	144679845	EEF1D	99.63965528	14158
chr8.fa	144680074	144682485	TIGD5	3.503771486	380
chr8.fa	144686083	144691784	PYCRL	1.893437666	228
chr8.fa	144694788	144699732	TSTA3	31.11518097	1900
chr8.fa	144718373	144735900	ZNF623	8.125725329	1520
chr8.fa	144766622	144777555	ZNF707	6.605682288	679
chr8.fa	144779285	144780583	BREA2	0	0
chr8.fa	144788864	144790279	LOC100130274	0	0
chr8.fa	144798507	144804633	MAPK15	0	0
chr8.fa	144806103	144815914	FAM83H	18.42065326	4636
chr8.fa	144816310	144828507	LOC100128338	9.262810927	1140

chr8.fa	144873090	144897549	SCRIB	14.62643903	3420
chr8.fa	144895132	144895199	MIR937	0	0
chr8.fa	144898547	144911537	PUF60	44.66257847	3876
chr8.fa	144915755	144923146	NRBP2	8.040327153	1346
chr8.fa	144939912	144947434	EPPK1	6.290776517	2128
chr8.fa	144989321	145050913	PLEC	9.971793694	7372
chr8.fa	145019359	145019447	MIR661	0	0
chr8.fa	145051320	145060635	PARP10	9.128264323	1444
chr8.fa	145064226	145067583	GRINA	16.3177232	1444
chr8.fa	145086582	145102015	SPATC1	0	0
chr8.fa	145106167	145115584	OPLAH	5.477937115	988
chr8.fa	145133522	145135551	EXOSC4	16.99623839	684
chr8.fa	145137524	145141119	GPAA1	16.35397295	1520
chr8.fa	145149960	145152428	CYC1	58.66721203	3192
chr8.fa	145153536	145159140	SHARPIN	22.3371826	1824
chr8.fa	145159305	145162515	MAF1	41.89714537	3344
chr8.fa	145162629	145173218	KIAA1875	0.237513675	76
chr8.fa	145192672	145440828	C8orf30A	0	0
chr8.fa	145202919	145316843	HEATR7A	7.531273968	1976
chr8.fa	145486056	145515120	BOP1	3.561148385	380
chr8.fa	145515270	145538246	HSF1	20.15107827	1824
chr8.fa	145538386	145550567	DGAT1	0.473915394	76
chr8.fa	145554454	145559943	SCRT1	0	0
chr8.fa	145576886	145578505	C8ORFK29	0	0
chr8.fa	145579091	145582132	FBXL6	0	0
chr8.fa	145582225	145584946	GPR172A	1.744213251	152
chr8.fa	145597731	145618445	ADCK5	0.865323697	76
chr8.fa	145618454	145634733	CPSF1	6.044367199	1216
chr8.fa	145619376	145619445	MIR939	0	0
chr8.fa	145625476	145625559	MIR1234	0	0
chr8.fa	145637798	145642273	SLC39A4	5.180600239	608
chr8.fa	145649000	145653927	VPS28	75.10413685	3188
chr8.fa	145654163	145669812	TONSL	1.123074961	228
chr8.fa	145675315	145691031	CYHR1	9.221446186	1064
chr8.fa	145691738	145699114	KIFC2	4.722697002	608
chr8.fa	145699500	145701718	FOXH1	0	0
chr8.fa	145722109	145727504	PPP1R16A	17.09498003	1748
chr8.fa	145729465	145732555	GPT	5.376971564	456
chr8.fa	145734552	145736589	MFSD3	8.181990272	532
chr8.fa	145736667	145743210	RECQL4	7.970496354	1368
chr8.fa	145743363	145750559	LRRC14	6.013232447	988
chr8.fa	145751603	145754458	C8orf82	42.48047718	3551
chr8.fa	145754563	145838888	ARHGAP39	2.629107363	608

chr8.fa	145946294	145980970	ZNF251	3.372560748	456
chr8.fa	145998501	146012725	ZNF34	0.873552167	76
chr8.fa	146015154	146017805	RPL8	1402.409501	61484
chr8.fa	146024261	146034529	ZNF517	2.895309487	304
chr8.fa	146052903	146068606	ZNF7	5.255990815	532
chr8.fa	146075551	146078932	COMMD5	2.067569883	152
chr8.fa	146102336	146126846	ZNF250	2.662021243	760
chr8.fa	146155744	146176274	ZNF16	3.876498938	456
chr8.fa	146198975	146225155	ZNF252	12.098742	2854
chr8.fa	146220251	146224283	TMED10P1	0.42988196	76
chr8.fa	146228197	146231432	C8orf77	0.535962506	76
chr8.fa	146277824	146281416	C8orf33	26.47966127	3116
chr9.fa	14511	29739	WASH1	1.840063807	152
chr9.fa	34394	35864	FAM138C	0	0
chr9.fa	116234	118417	FOXD4	0	0
chr9.fa	121038	179075	CBWD1	8.353453796	684
chr9.fa	168877	170508	LOC642313	0	0
chr9.fa	213108	215893	C9orf66	1.934580016	228
chr9.fa	271627	465259	DOCK8	20.89697795	6897
chr9.fa	504703	746103	KANK1	46.51554096	11324
chr9.fa	841690	969090	DMRT1	0	0
chr9.fa	976964	991732	DMRT3	0	0
chr9.fa	1050346	1057554	DMRT2	0.575770509	76
chr9.fa	2015342	2193623	SMARCA2	72.67896211	18811
chr9.fa	2535655	2622373	FLJ35024	0	0
chr9.fa	2621793	2654485	VLDLR	3.903408259	608
chr9.fa	2717526	2729757	KCNV2	0	0
chr9.fa	2804155	2844130	KIAA0020	19.8037034	1976
chr9.fa	3224647	3525983	RFX3	2.138290247	380
chr9.fa	3824128	4300035	GLIS3	9.535240002	3268
chr9.fa	3898646	3901248	C9orf70	0	0
chr9.fa	4490427	4587469	SLC1A1	0.904019745	152
chr9.fa	4598316	4666674	C9orf68	0.451231504	76
chr9.fa	4662298	4665258	PPAPDC2	2.283289232	304
chr9.fa	4679566	4706594	CDC37L1	2.98626744	228
chr9.fa	4709557	4742043	AK3	19.30554738	3876
chr9.fa	4792834	4861064	RCL1	4.675550093	456
chr9.fa	4850297	4850375	MIR101-2	0	0
chr9.fa	4985245	5128183	JAK2	5.11699639	1216
chr9.fa	5163863	5185618	INSL6	0	0
chr9.fa	5231419	5233967	INSL4	0	0
chr9.fa	5299868	5304580	RLN2	3.802442708	152
chr9.fa	5334969	5339873	RLN1	0	0

chr9.fa	5357971	5437860	C9orf46	1.845178802	76
chr9.fa	5450503	5470567	CD274	0	0
chr9.fa	5510545	5571282	PDCD1LG2	0	0
chr9.fa	5629327	5776556	KIAA1432	5.406549577	1596
chr9.fa	5784572	5833081	ERMP1	9.49365287	2280
chr9.fa	5890909	5909822	MLANA	0	0
chr9.fa	5919008	6008003	KIAA2026	11.73068476	3800
chr9.fa	6011019	6015640	RANBP6	6.216497896	1292
chr9.fa	6215786	6257983	IL33	0	0
chr9.fa	6328349	6331900	TPD52L3	0	0
chr9.fa	6413151	6507051	UHRF2	11.66930482	1900
chr9.fa	6532464	6645692	GLDC	1.77490322	304
chr9.fa	6720863	7175648	KDM4C	8.874293708	2660
chr9.fa	7796491	7799799	C9orf123	9.51833828	1064
chr9.fa	8314246	10612723	PTPRD	0.610463518	279
chr9.fa	12693386	12710266	TYRP1	1.175336865	152
chr9.fa	12775012	12823059	C9orf150	0.628254805	76
chr9.fa	13105703	13250365	MPDZ	8.554272942	2861
chr9.fa	13406379	13431328	FLJ41200	0	0
chr9.fa	14081842	14398982	NFIB	33.96023007	13661
chr9.fa	14611069	14693480	ZDHHC21	2.395819119	988
chr9.fa	14719732	14722715	CER1	0	0
chr9.fa	14734664	14910993	FREM1	2.314201592	1064
chr9.fa	14993325	15019722	LOC389705	0	0
chr9.fa	15170842	15307358	TTC39B	0.474137785	76
chr9.fa	15422782	15461627	SNAPC3	9.829685793	1368
chr9.fa	15464065	15511003	PSIP1	3.445727414	608
chr9.fa	15553097	15971897	C9orf93	0.7754777	152
chr9.fa	16409501	16870786	BNC2	0.523063823	304
chr9.fa	17135038	17503917	CNTLN	0.550417926	228
chr9.fa	17578953	17797122	SH3GL2	8.054337791	988
chr9.fa	18474104	18910947	ADAMTSL1	1.72975783	608
chr9.fa	18573304	18573377	MIR3152	0	0
chr9.fa	18927891	19033256	FAM154A	0	0
chr9.fa	19049372	19051021	RRAGA	49.16866656	3648
chr9.fa	19053141	19102902	HAUS6	2.139179811	608
chr9.fa	19063654	19063784	SCARNA8	0	0
chr9.fa	19115759	19127573	PLIN2	30.7304444	2736
chr9.fa	19290749	19374139	DENND4C	13.95837622	4408
chr9.fa	19376254	19380235	RPS6	3031.708165	113012
chr9.fa	19408925	19452500	ACER2	53.92894765	6916
chr9.fa	19507450	19788591	SLC24A2	0	0
chr9.fa	20344968	20622514	MLLT3	7.372041954	1140

chr9.fa	20658309	20995954	KIAA1797	19.96604889	5472
chr9.fa	20716104	20716187	MIR491	0	0
chr9.fa	21006365	21031635	PTPLAD2	0	0
chr9.fa	21077104	21077943	IFNB1	0	0
chr9.fa	21140631	21142144	IFNW1	0	0
chr9.fa	21165636	21166659	IFNA21	0	0
chr9.fa	21186618	21187598	IFNA4	0	0
chr9.fa	21201468	21202204	IFNA7	0	0
chr9.fa	21206180	21207142	IFNA10	0	0
chr9.fa	21216372	21217310	IFNA16	0	0
chr9.fa	21227242	21228221	IFNA17	0	0
chr9.fa	21239201	21239978	IFNA14	0	0
chr9.fa	21277687	21278562	IFNA22P	0	0
chr9.fa	21304613	21305312	IFNA5	0	0
chr9.fa	21331018	21335429	KLHL9	16.08954995	3192
chr9.fa	21350317	21350886	IFNA6	0	0
chr9.fa	21367371	21368075	IFNA13	0	0
chr9.fa	21384254	21385396	IFNA2	0	0
chr9.fa	21409146	21410184	IFNA8	0	0
chr9.fa	21440440	21441315	IFNA1	0	0
chr9.fa	21454267	21559697	LOC554202	0	0
chr9.fa	21480839	21482312	IFNE	0	0
chr9.fa	21512114	21512184	MIR31	0	0
chr9.fa	21802635	21865969	MTAP	6.855649863	1517
chr9.fa	21967138	21967750	C9orf53	0	0
chr9.fa	21967754	21994490	CDKN2A	0	0
chr9.fa	21994790	22121093	CDKN2B-AS1	0	0
chr9.fa	22002902	22009312	CDKN2B	5.090976633	912
chr9.fa	22446840	22452472	DMRTA1	5.139457889	532
chr9.fa	23690103	23826063	ELAVL2	0	0
chr9.fa	25676387	25678856	TUSC1	8.211345895	912
chr9.fa	26840683	26892826	C9orf82	9.677570294	1292
chr9.fa	26903368	26947468	PLAA	11.16225315	2356
chr9.fa	26956371	27062931	IFT74	9.145388436	1033
chr9.fa	26993135	27005691	LRRC19	1.405289243	228
chr9.fa	27109147	27230172	TEK	0	0
chr9.fa	27245682	27282791	NCRNA00032	0	0
chr9.fa	27284654	27297137	C9orf11	0	0
chr9.fa	27325207	27529850	MOBKL2B	1.295205657	380
chr9.fa	27524312	27526496	IFNK	0	0
chr9.fa	27546544	27573842	C9orf72	5.739024244	1129
chr9.fa	27948528	28719303	LINGO2	0	0
chr9.fa	28863624	28863704	MIR876	0	0

chr9.fa	28888877	28888953	MIR873	0	0
chr9.fa	32384601	32450832	ACO1	23.81230264	3799
chr9.fa	32455300	32526322	DDX58	6.75223801	1444
chr9.fa	32540542	32552626	TOPORS	8.029207599	1490
chr9.fa	32551142	32553015	LOC100129250	6.636149866	299
chr9.fa	32553524	32573182	NDUFB6	26.91421344	1042
chr9.fa	32629452	32635667	TAF1L	0	0
chr9.fa	32783497	32789199	TMEM215	0	0
chr9.fa	32972604	33001639	APTX	8.536259265	912
chr9.fa	33025209	33039062	DNAJA1	125.2793454	8664
chr9.fa	33041850	33076714	SMU1	12.39452213	3952
chr9.fa	33110639	33167356	B4GALT1	15.29561379	2888
chr9.fa	33240196	33248565	SPINK4	0	0
chr9.fa	33252470	33264759	BAG1	32.24448288	5614
chr9.fa	33264877	33282067	CHMP5	49.49535905	4607
chr9.fa	33290418	33371155	NFX1	15.09234834	4175
chr9.fa	33384948	33402517	AQP7	1.345688433	76
chr9.fa	33441160	33447590	AQP3	12.05937877	988
chr9.fa	33461351	33473941	NOL6	18.80672418	4104
chr9.fa	33504535	33511164	SUGT1P1	2.197891056	76
chr9.fa	33624223	33625532	ANXA2P2	0	0
chr9.fa	33673502	33677418	PTENP1	1.294538484	228
chr9.fa	33750464	33799229	PRSS3	0	0
chr9.fa	33817182	33920401	UBE2R2	31.47500974	6308
chr9.fa	33921691	34048947	UBAP2	29.94295758	5768
chr9.fa	33934286	33934378	SNORD121B	0	0
chr9.fa	33952762	33952852	SNORD121A	0	0
chr9.fa	34086381	34126771	DCAF12	31.71385776	5235
chr9.fa	34179003	34252377	UBAP1	28.80008981	3876
chr9.fa	34252522	34329198	KIF24	0.833521773	228
chr9.fa	34329504	34343696	NUDT2	9.567041926	456
chr9.fa	34368907	34376894	KIAA1161	0.804388541	152
chr9.fa	34379017	34397849	C9orf24	0	0
chr9.fa	34398182	34458568	C9orf25	7.419188863	1216
chr9.fa	34458811	34520982	DNAI1	0	0
chr9.fa	34521040	34523037	ENHO	6.271428493	304
chr9.fa	34551431	34589722	CNTFR	0	0
chr9.fa	34568010	34583070	LOC415056	0	0
chr9.fa	34610492	34612101	C9orf23	21.84903417	1064
chr9.fa	34613548	34620496	DCTN3	28.61239174	1064
chr9.fa	34621455	34628011	ARID3C	0	0
chr9.fa	34634719	34637768	SIGMAR1	7.144535878	532
chr9.fa	34646635	34650573	GALT	3.80088597	228

chr9.fa	34652182	34661884	IL11RA	0.677625625	54
chr9.fa	34661893	34662689	CCL27	0	0
chr9.fa	34689567	34691274	CCL19	0	0
chr9.fa	34709002	34710147	CCL21	0	0
chr9.fa	34723050	34729535	C9orf144B	0	0
chr9.fa	34830264	34838583	C9orf144	0	0
chr9.fa	34958192	34982541	KIAA1045	0	0
chr9.fa	34989725	34998428	DNAJB5	0.582887024	76
chr9.fa	35041102	35045988	C9orf131	0	0
chr9.fa	35056065	35072739	VCP	93.72805556	16264
chr9.fa	35073835	35080013	FANCG	1.927241111	228
chr9.fa	35088688	35096579	PIGO	1.653477689	304
chr9.fa	35099889	35103154	STOML2	47.64773395	2736
chr9.fa	35104118	35115893	KIAA1539	8.37280182	1140
chr9.fa	35161989	35405332	UNC13B	40.56101975	11618
chr9.fa	35406752	35483026	ATP8B5P	0	0
chr9.fa	35490007	35561826	RUSC2	2.561500474	660
chr9.fa	35561896	35563896	FAM166B	0	0
chr9.fa	35605281	35609975	TESK1	6.890120481	760
chr9.fa	35610039	35618424	CD72	1.138197554	76
chr9.fa	35649297	35650947	SIT1	0	0
chr9.fa	35657748	35658015	RMRP	0	0
chr9.fa	35658287	35661500	CCDC107	0	0
chr9.fa	35659341	35665278	C9orf100	0.592449841	76
chr9.fa	35673915	35681154	CA9	0	0
chr9.fa	35681990	35690053	TPM2	0	0
chr9.fa	35697334	35732316	TLN1	19.61556054	7372
chr9.fa	35732393	35736862	CREB3	43.49702681	3192
chr9.fa	35737006	35749225	GBA2	15.10813811	2356
chr9.fa	35749277	35752987	RGP1	25.82205084	1672
chr9.fa	35753265	35754274	MSMP	29.57801381	532
chr9.fa	35792406	35809728	NPR2	1.017883979	152
chr9.fa	35807782	35812259	SPAG8	0.760355107	76
chr9.fa	35812957	35815042	HINT2	51.84581039	1520
chr9.fa	35817014	35828744	C9orf128	0.57376899	76
chr9.fa	35829222	35854844	TMEM8B	6.967957359	1216
chr9.fa	35860271	35865515	LOC92973	0	0
chr9.fa	35869460	35870398	OR13J1	0	0
chr9.fa	35906189	35907138	HRCT1	0	0
chr9.fa	35909480	35911617	LOC158376	0	0
chr9.fa	35957105	35958151	OR2S2	0	0
chr9.fa	36036910	36124452	RECK	1.53227455	304
chr9.fa	36136742	36163903	GLIPR2	48.47013617	4104

chr9.fa	36169389	36171331	CCIN	0	0
chr9.fa	36190853	36212059	CLTA	116.3954889	6469
chr9.fa	36214439	36277053	GNE	26.23525347	6460
chr9.fa	36336399	36401195	RNF38	16.11134428	4028
chr9.fa	36572905	36677679	MELK	0	0
chr9.fa	36838531	37034476	PAX5	0	0
chr9.fa	37079893	37090398	LOC100506710	24.33847994	4788
chr9.fa	37120469	37358145	ZCCHC7	11.33082559	1368
chr9.fa	37422707	37436986	GRHPR	80.74508662	4484
chr9.fa	37438100	37465407	ZBTB5	9.45251052	1976
chr9.fa	37485945	37503693	POLR1E	10.02805864	836
chr9.fa	37510889	37576250	FBXO10	1.847180321	380
chr9.fa	37588412	37592636	TOMM5	42.95461496	1748
chr9.fa	37651052	37746901	FRMPD1	0	0
chr9.fa	37753802	37778969	RG9MTD3	4.422691434	456
chr9.fa	37780308	37785065	EXOSC3	13.85385241	760
chr9.fa	37800790	37867665	DCAF10	7.261291196	2584
chr9.fa	37877572	37904350	MCART1	4.962434588	1053
chr9.fa	37915895	38069210	SHB	4.491410278	1216
chr9.fa	38392702	38398657	ALDH1B1	4.471395081	608
chr9.fa	38408991	38424444	IGFBPL1	0	0
chr9.fa	38571361	38620360	ANKRD18A	2.712948801	456
chr9.fa	38621085	38623277	FAM201A	1.957263907	152
chr9.fa	39072764	39288300	CNTNAP3	0	0
chr9.fa	39443814	41609544	LOC653501	0	0
chr9.fa	39900200	39907240	FAM74A1	0	0
chr9.fa	40700291	40706537	FAM75A3	0	0
chr9.fa	40715524	40722679	FAM74A3	0	0
chr9.fa	40771402	40792112	ZNF658	2.099594199	380
chr9.fa	41321107	65503365	FAM75A5	0	0
chr9.fa	41952399	41955076	MGC21881	1.309661078	152
chr9.fa	41958802	42019584	KGFLP2	0	0
chr9.fa	42411411	67970293	ANKRD20A3	0	0
chr9.fa	42468589	43033312	FAM95B1	0	0
chr9.fa	42858152	42893137	AQP7P3	3.195092665	76
chr9.fa	43140537	43145484	LOC642929	0	0
chr9.fa	43624502	43630730	FAM75A6	0	0
chr9.fa	44990236	44991492	FAM27C	0	0
chr9.fa	45727029	45728283	FAM27A	0	0
chr9.fa	46687566	46748386	KGFLP1	0	0
chr9.fa	66494269	66503030	LOC442421	0	0
chr9.fa	67017400	69664949	LOC100133920	0	0
chr9.fa	67270215	67289492	AQP7P1	0	0

chr9.fa	67792929	67794189	FAM27B	0	0
chr9.fa	68427783	68454375	LOC642236	0	0
chr9.fa	68726541	68748372	LOC100132352	0	0
chr9.fa	69080244	69147854	PGM5P2	0	0
chr9.fa	69174214	69181041	LOC440896	0	0
chr9.fa	69199480	69202204	FOXD4L6	0	0
chr9.fa	69204538	69262593	CBWD6	0	0
chr9.fa	69381981	69425109	ANKRD20A4	0	0
chr9.fa	70175707	70178815	FOXD4L5	0	0
chr9.fa	70432000	70914932	CBWD3	0	0
chr9.fa	70917783	70920000	FOXD4L3	0	0
chr9.fa	70970109	70972757	LOC572558	3.177078987	76
chr9.fa	70971815	71145977	PGM5	2.53170007	380
chr9.fa	71151498	71155783	C9orf71	4.079319604	228
chr9.fa	71320616	71624091	PIP5K1B	1.910561779	228
chr9.fa	71394964	71398609	FAM122A	6.953501939	1140
chr9.fa	71627449	71629039	PRKACG	0	0
chr9.fa	71650479	71715094	FXN	1.361922982	456
chr9.fa	71736224	71870124	TJP2	68.12639428	15954
chr9.fa	71939488	72007370	FAM189A2	0	0
chr9.fa	72042449	72287275	APBA1	7.701180754	2280
chr9.fa	72324438	72374876	PTAR1	5.717452309	2584
chr9.fa	72435731	72521148	C9orf135	0	0
chr9.fa	72658497	72841888	MAMDC2	0	0
chr9.fa	72873878	72969789	SMC5	10.48396035	2812
chr9.fa	72999513	73029573	KLF9	12.00778404	2812
chr9.fa	73149966	73736514	TRPM3	4.289701567	1292
chr9.fa	73424891	73425000	MIR204	0	0
chr9.fa	74298282	74383800	TMEM2	22.79819931	6687
chr9.fa	74477368	74526148	FAM108B1	8.813803334	1292
chr9.fa	74526423	74588371	C9orf85	5.141237017	304
chr9.fa	74666297	74675521	C9orf57	0	0
chr9.fa	74764293	74867140	GDA	0.93382015	228
chr9.fa	74966341	74980163	ZFAND5	15.84180629	4180
chr9.fa	75136717	75451267	TMC1	2.112048099	304
chr9.fa	75515587	75567969	ALDH1A1	14.5147987	1368
chr9.fa	75766781	75785307	ANXA1	78.25808716	4923
chr9.fa	77112252	77302117	RORB	0.46902279	76
chr9.fa	77337411	77503010	TRPM6	0.195704152	76
chr9.fa	77561499	77567802	C9orf40	0	0
chr9.fa	77597873	77643310	C9orf41	10.01916299	988
chr9.fa	77676116	77703133	C9orf95	13.98773184	912
chr9.fa	77703398	77762114	OSTF1	45.88395028	2709

chr9.fa	78118512	78338744	MIR548H3	0	0
chr9.fa	78505560	78977255	PCSK5	0.347374869	152
chr9.fa	79000433	79009444	RFK	9.365555607	1140
chr9.fa	79013515	79014954	RPSAP9	0	0
chr9.fa	79056582	79122332	GCNT1	2.012416895	608
chr9.fa	79226292	79521003	PRUNE2	7.923794227	4484
chr9.fa	79379354	79402465	PCA3	0	0
chr9.fa	79634571	79635869	FOXB2	0	0
chr9.fa	79791672	79792833	LOC100286938	0	0
chr9.fa	79792361	80032399	VPS13A	2.450082543	1368
chr9.fa	80037995	80263232	GNA14	7.463667079	836
chr9.fa	80335191	80646219	GNAQ	33.86504669	3344
chr9.fa	80850991	80881983	CEP78	1.843844455	228
chr9.fa	80912059	80945009	PSAT1	15.44950842	1520
chr9.fa	82186878	82341656	TLE4	10.56913614	2256
chr9.fa	84198598	84303596	TLE1	16.77629361	2466
chr9.fa	84528352	84534842	FAM75D5	0	0
chr9.fa	84543343	84549913	FAM75D4	0	0
chr9.fa	84558415	84565009	FAM75D3	0	0
chr9.fa	84603687	84610171	FAM75D1	0	0
chr9.fa	85597317	85678043	RASEF	45.33264279	5624
chr9.fa	85862307	86153348	FRMD3	14.0735748	1596
chr9.fa	86238033	86259045	C9orf103	36.66695192	1596
chr9.fa	86274878	86323168	UBQLN1	45.4173738	8512
chr9.fa	86354336	86432752	GKAP1	7.21903689	608
chr9.fa	86451615	86536342	KIF27	3.997479686	836
chr9.fa	86553227	86571663	C9orf64	8.884746089	988
chr9.fa	86582998	86595569	HNRNPK	138.1482277	19226
chr9.fa	86584663	86584772	MIR7-1	0	0
chr9.fa	86595637	86618987	RMI1	3.386126604	532
chr9.fa	86890765	86983413	SLC28A3	0.372505061	76
chr9.fa	87283466	87638505	NTRK2	0	0
chr9.fa	88161454	88356944	AGTPBP1	5.903148862	1173
chr9.fa	88420917	88457794	LOC389765	2.874404726	228
chr9.fa	88556057	88637217	NAA35	19.56062995	2337
chr9.fa	88641058	88715116	GOLM1	13.3652592	1961
chr9.fa	88842226	88874519	C9orf153	0	0
chr9.fa	88879463	88897490	ISCA1	12.66361534	1140
chr9.fa	88902648	88969402	ZCCHC6	25.86474993	6606
chr9.fa	89559277	89562104	GAS1	1.195352062	152
chr9.fa	89623366	89657041	LOC440173	0.838414376	76
chr9.fa	89698801	89700140	LOC494127	0	0
chr9.fa	89763559	89774641	C9orf170	0	0

chr9.fa	90112756	90323549	DAPK1	56.64278602	15124
chr9.fa	90340974	90346384	CTSL1	7.540614394	532
chr9.fa	90387830	90401799	CTSL3	0	0
chr9.fa	90459660	90462339	CTSL1P8	0	0
chr9.fa	90497772	90503814	C9orf79	0	0
chr9.fa	90532877	90538572	FAM75C1	0	0
chr9.fa	90581359	90589695	CDK20	7.146537398	760
chr9.fa	90744220	90749900	FAM75C2	0	0
chr9.fa	91003297	91093622	SPIN1	36.83619153	7510
chr9.fa	91150016	91190704	NXNL2	0	0
chr9.fa	91262094	91267075	LOC286238	0	0
chr9.fa	91360751	91360820	MIR4289	0	0
chr9.fa	91605778	91611057	C9orf47	0	0
chr9.fa	91606362	91619925	S1PR3	0.849311539	152
chr9.fa	91620686	91793682	SHC3	2.076465526	912
chr9.fa	91926113	91931618	CKS2	5.514409252	152
chr9.fa	91927140	91927221	MIR3153	0	0
chr9.fa	91933412	91974561	SECISBP2	21.93754582	3420
chr9.fa	91975706	92094611	SEMA4D	3.450620017	1054
chr9.fa	92219927	92221469	GADD45G	11.10910168	532
chr9.fa	92254698	92334674	LOC100129066	0	0
chr9.fa	92785723	92785817	MIR4290	0	0
chr9.fa	93224714	93345028	LOC340515	0	0
chr9.fa	93372114	93405108	DIRAS2	2.880631676	532
chr9.fa	93564012	93660842	SYK	8.274282571	1976
chr9.fa	93825576	93837414	LOC100129316	0	0
chr9.fa	93976097	94124206	AUH	41.11721985	2936
chr9.fa	94171327	94186144	NFIL3	13.78068574	1292
chr9.fa	94398533	94398643	MIR3910-1	0	0
chr9.fa	94484878	94712444	ROR2	1.237606367	228
chr9.fa	94793427	94877690	SPTLC1	4.118682826	608
chr9.fa	94895116	94900911	LOC100128076	0	0
chr9.fa	94903749	94921890	C9orf44	0	0
chr9.fa	94972625	95056038	IARS	24.74678996	5092
chr9.fa	95054740	95054742	MIR3651	0	0
chr9.fa	95054830	95054875	SNORA84	0	0
chr9.fa	95059640	95087740	NOL8	9.828796229	1900
chr9.fa	95087877	95375465	CENPP	0	0
chr9.fa	95146249	95166937	OGN	0	0
chr9.fa	95176527	95186836	OMD	0	0
chr9.fa	95218489	95244844	ASPN	0	0
chr9.fa	95255829	95298374	ECM2	0	0
chr9.fa	95377438	95432547	IPPK	3.853370266	407

chr9.fa	95380332	95382817	LOC100128361	0.702533426	76
chr9.fa	95473645	95527083	BICD2	112.4849641	32452
chr9.fa	95571893	95600739	ANKRD19	0.732556222	76
chr9.fa	95608351	95640270	ZNF484	5.342945728	684
chr9.fa	95709601	95798518	FGD3	37.71908413	6384
chr9.fa	95820989	95847415	SUSD3	0	0
chr9.fa	95858450	95875565	C9orf89	9.327749123	380
chr9.fa	95883771	95896570	NINJ1	19.7912495	1140
chr9.fa	95947212	96082854	WNK2	9.629089038	2959
chr9.fa	96080481	96108696	C9orf129	1.464667661	76
chr9.fa	96208782	96215874	FAM120AOS	28.1471496	3192
chr9.fa	96214173	96328397	FAM120A	59.78717351	12673
chr9.fa	96338909	96441869	PHF2	17.69476877	4256
chr9.fa	96581639	96581703	MIR4291	0	0
chr9.fa	96713909	96717608	BARX1	0	0
chr9.fa	96793076	96872136	PTPDC1	2.10404202	456
chr9.fa	96938239	96938318	MIRLET7A1	0	0
chr9.fa	96938629	96938715	MIRLET7F1	0	0
chr9.fa	96941116	96941202	MIRLET7D	0	0
chr9.fa	97021578	97065291	ZNF169	0	0
chr9.fa	97080478	97090926	FAM22F	0	0
chr9.fa	97094758	97123230	LOC100132077	0	0
chr9.fa	97136833	97223202	HIATL1	4.612391026	684
chr9.fa	97321003	97356075	FBP2	0	0
chr9.fa	97365421	97402531	FBP1	87.52000853	7068
chr9.fa	97488951	97849500	C9orf3	2.937786184	684
chr9.fa	97572244	97572339	MIR2278	0	0
chr9.fa	97847490	97847586	MIR23B	0	0
chr9.fa	97847727	97847823	MIR27B	0	0
chr9.fa	97848296	97848376	MIR3074	0	0
chr9.fa	97861336	98079991	FANCC	4.048852026	836
chr9.fa	98205264	98279247	PTCH1	4.319724363	1748
chr9.fa	98568370	98638259	C9orf130	3.030300874	1216
chr9.fa	98638260	98731122	C9orf102	6.308123021	1216
chr9.fa	98782014	98784037	NCRNA00092	2.946237045	228
chr9.fa	98828121	98864194	LOC158435	0	0
chr9.fa	98997589	99064434	HSD17B3	0	0
chr9.fa	99082988	99145992	SLC35D2	19.27107676	1409
chr9.fa	99148225	99180669	ZNF367	0	0
chr9.fa	99212414	99253618	HABP4	11.3181493	1368
chr9.fa	99262395	99382112	CDC14B	13.53783468	3496
chr9.fa	99403533	99417599	C9orf21	1.379714268	76
chr9.fa	99488103	99489749	LOC441455	0	0

chr9.fa	99518147	99540328	ZNF510	5.557553122	1288
chr9.fa	99579273	99616389	ZNF782	3.571155984	532
chr9.fa	99671357	99672737	LOC441454	0	0
chr9.fa	99691286	99704572	FAM22G	0.322022286	76
chr9.fa	99708327	99775862	HIATL2	0	0
chr9.fa	99794938	99801539	CTSL2	0	0
chr9.fa	99837953	99844227	LOC340508	0	0
chr9.fa	99959537	99961910	ZNF322B	0	0
chr9.fa	100054838	100059596	LOC100499484	0	0
chr9.fa	100069910	100070017	C9orf174	0	0
chr9.fa	100124534	100126332	LOC57653	0	0
chr9.fa	100153119	100158973	LOC286359	0	0
chr9.fa	100174302	100258405	TDRD7	15.2753762	2584
chr9.fa	100263462	100362361	TMOD1	0.959172734	76
chr9.fa	100364026	100395704	TSTD2	12.14010674	1368
chr9.fa	100395963	100436029	NCBP1	8.909431499	2052
chr9.fa	100437191	100459691	XPA	7.953817023	608
chr9.fa	100615537	100618997	FOXE1	0	0
chr9.fa	100666772	100684852	C9orf156	6.161122516	456
chr9.fa	100689073	100707134	HEMGN	0	0
chr9.fa	100745489	100778224	ANP32B	78.52495646	5706
chr9.fa	100818959	100845365	NANS	173.1063266	9652
chr9.fa	100831569	100881480	TRIM14	21.23590196	4636
chr9.fa	100883257	100954956	CORO2A	26.70694495	6916
chr9.fa	100961280	101018003	TBC1D2	18.85320392	2812
chr9.fa	101050364	101471479	GABBR2	5.840212186	1520
chr9.fa	101494291	101558794	ANKS6	8.927445177	2869
chr9.fa	101569981	101612359	GALNT12	2.475435126	304
chr9.fa	101706138	101833068	COL15A1	0	0
chr9.fa	101867412	101916473	TGFBR1	1.044348518	304
chr9.fa	101978707	101984246	ALG2	6.220056153	836
chr9.fa	101984570	101992901	SEC61B	135.334091	3420
chr9.fa	102584137	102629173	NR4A3	0.592005058	152
chr9.fa	102668915	102736818	STX17	9.786764315	3040
chr9.fa	102741463	102861330	ERP44	1.043236562	228
chr9.fa	102861511	103063426	INVS	6.098630622	1064
chr9.fa	103064357	103115259	TEX10	7.22726536	1064
chr9.fa	103189495	103214016	C9orf30	7.865750154	608
chr9.fa	103235520	103236022	TMEFF1	0	0
chr9.fa	103340336	103350173	MURC	0	0
chr9.fa	103791031	104087417	LPPR1	1.894772013	228
chr9.fa	104122699	104147287	BAAT	0	0
chr9.fa	104152249	104160919	MRPL50	13.2431665	608

chr9.fa	104161163	104172942	ZNF189	9.20788033	1368
chr9.fa	104182842	104198062	ALDOB	1208.520505	131834
chr9.fa	104237608	104249475	C9orf125	0	0
chr9.fa	104296133	104325626	RNF20	20.16197543	3572
chr9.fa	104331634	104500862	GRIN3A	0.217498478	76
chr9.fa	104353897	104357283	PPP3R2	0	0
chr9.fa	105757593	105780770	CYLC2	0	0
chr9.fa	106856541	106903700	SMC2	3.770863175	1064
chr9.fa	107266544	107267503	OR13F1	0	0
chr9.fa	107288534	107289490	OR13C4	0	0
chr9.fa	107298051	107299094	OR13C3	0	0
chr9.fa	107331449	107332411	OR13C8	0	0
chr9.fa	107360738	107361694	OR13C5	0	0
chr9.fa	107366952	107367908	OR13C2	0	0
chr9.fa	107379529	107380485	OR13C9	0	0
chr9.fa	107456703	107457743	OR13D1	0	0
chr9.fa	107509969	107522403	NIPSNAP3A	20.70016185	1520
chr9.fa	107526451	107536291	NIPSNAP3B	0	0
chr9.fa	107536633	107540045	LOC286367	0	0
chr9.fa	107543284	107690527	ABCA1	4.298819602	2030
chr9.fa	108006929	108153682	SLC44A1	6.701088062	1368
chr9.fa	108210315	108309083	FSD1L	0.805278105	76
chr9.fa	108320411	108403399	FKTN	0.674956932	76
chr9.fa	108424738	108425385	TAL2	0	0
chr9.fa	108456825	108537444	TMEM38B	0	0
chr9.fa	109625378	109773796	ZNF462	17.12389087	6384
chr9.fa	109653505	109848716	MIR548Q	0	0
chr9.fa	110045544	110094470	RAD23B	50.7460865	9424
chr9.fa	110247133	110252047	KLF4	6.33881299	836
chr9.fa	111616869	111618275	ACTL7B	0	0
chr9.fa	111624603	111626035	ACTL7A	0	0
chr9.fa	111629800	111696608	IKBKAP	7.740321585	2128
chr9.fa	111696673	111703237	C9orf6	7.960043973	684
chr9.fa	111704851	111775764	CTNNAL1	14.29841218	1572
chr9.fa	111777415	111882225	C9orf5	4.226987282	1520
chr9.fa	111808509	111808578	MIR32	0	0
chr9.fa	111899581	111929571	C9orf4	0	0
chr9.fa	111934254	112083021	EPB41L4B	21.14983661	7281
chr9.fa	112137974	112260593	PTPN3	16.33217862	5339
chr9.fa	112403068	112713756	PALM2	0.190589157	76
chr9.fa	112778234	112778319	PALM2-AKAP2	17.84310363	69
chr9.fa	112810878	112887876	AKAP2	0	0
chr9.fa	112961846	112970413	C9orf152	27.9999267	3344

chr9.fa	113006310	113018778	TXN	224.2736218	5123
chr9.fa	113065867	113100125	TXNDC8	0	0
chr9.fa	113127529	113342160	SVEP1	0	0
chr9.fa	113431051	113563278	MUSK	0	0
chr9.fa	113636054	113800365	LPAR1	0.533516204	76
chr9.fa	114089763	114090713	OR2K2	0	0
chr9.fa	114122973	114247025	KIAA0368	40.49141135	13457
chr9.fa	114287447	114340124	ZNF483	2.59975174	608
chr9.fa	114312002	114362135	PTGR1	122.8083581	8714
chr9.fa	114365111	114375833	C9orf29	0	0
chr9.fa	114405137	114416631	DNAJC25	1.598991875	152
chr9.fa	114423851	114431551	GNG10	128.7764452	857
chr9.fa	114448901	114545779	C9orf84	0	0
chr9.fa	114659206	114695433	UGCG	4.145147364	304
chr9.fa	114758832	115112545	MIR3134	0	0
chr9.fa	114803061	114937556	SUSD1	11.28278912	1520
chr9.fa	114980715	115095944	ROD1	26.26727779	8588
chr9.fa	115142189	115234685	HSDL2	39.58605725	6004
chr9.fa	115249248	115422706	KIAA1958	4.409792751	532
chr9.fa	115448791	115480387	C9orf80	12.23951555	836
chr9.fa	115513134	115637267	SNX30	13.55518119	4636
chr9.fa	115641200	115653193	SLC46A2	0	0
chr9.fa	115759400	115774472	ZNF883	2.131396123	228
chr9.fa	115804174	115818996	ZFP37	1.228265942	152
chr9.fa	115867003	115873957	NCRNA00256B	0	0
chr9.fa	115875176	115882126	NCRNA00256A	0	0
chr9.fa	115913238	115926422	SLC31A2	0	0
chr9.fa	115927800	115983641	FKBP15	14.03287723	2736
chr9.fa	115983808	116026772	SLC31A1	5.659853019	1216
chr9.fa	116029290	116037869	CDC26	9.713597649	380
chr9.fa	116037974	116055056	PRPF4	22.83266993	2808
chr9.fa	116059373	116061320	RNF183	0	0
chr9.fa	116077931	116102620	WDR31	2.700050118	304
chr9.fa	116111812	116133513	BSPRY	14.09359	1488
chr9.fa	116135698	116138341	HDHD3	15.71348663	1216
chr9.fa	116148592	116163618	ALAD	80.46732016	11517
chr9.fa	116169518	116173021	POLE3	14.22835899	1444
chr9.fa	116173030	116191879	C9orf43	0	0
chr9.fa	116207011	116360018	RGS3	4.386219296	1368
chr9.fa	116638562	116818875	ZNF618	9.273485699	3800
chr9.fa	116822408	116840752	AMBP	0	0
chr9.fa	116853918	116861337	KIF12	10.12591071	912
chr9.fa	116918231	117072975	COL27A1	2.111158535	530

chr9.fa	116971714	116971809	MIR455	0	0
chr9.fa	117085303	117088759	ORM1	0	0
chr9.fa	117092069	117095536	ORM2	6.00767267	228
chr9.fa	117096433	117156685	AKNA	2.747197027	912
chr9.fa	117164360	117267736	DFNB31	1.624344458	304
chr9.fa	117349994	117361152	ATP6V1G1	138.4871518	10032
chr9.fa	117373706	117408703	C9orf91	4.656202069	912
chr9.fa	117428714	117444369	LOC100505478	0	0
chr9.fa	117551611	117568408	TNFSF15	1.696176777	152
chr9.fa	117665124	117692770	TNFSF8	0	0
chr9.fa	117782805	117880486	TNC	0	0
chr9.fa	117904097	118164923	1-Dec	0	0
chr9.fa	118650544	118687377	C9orf27	0	0
chr9.fa	118916071	119164600	PAPPA	0.770362706	380
chr9.fa	119187504	120177317	ASTN2	2.635779096	912
chr9.fa	119266562	119324572	LOC100128505	0	0
chr9.fa	119449581	119463579	TRIM32	6.362608836	1064
chr9.fa	119943345	119943480	SNORA70C	0	0
chr9.fa	120466460	120479766	TLR4	1.46444527	380
chr9.fa	121928908	122131739	DBC1	0.528845991	76
chr9.fa	123007257	123007328	MIR147	0	0
chr9.fa	123151147	123342437	CDK5RAP2	14.65001248	4104
chr9.fa	123363196	123476765	MEGF9	4.626846446	1292
chr9.fa	123519254	123555740	FBXW2	13.24761432	5472
chr9.fa	123555775	123561009	LOC100288842	8.697270407	228
chr9.fa	123578332	123605206	PSMD5	21.96334319	3344
chr9.fa	123605320	123616651	LOC253039	6.675290696	1140
chr9.fa	123617931	123639606	PHF19	0.370948324	76
chr9.fa	123664671	123691451	TRAF1	0.369391586	76
chr9.fa	123714614	123812554	C5	0.928037982	228
chr9.fa	123850574	123939886	CEP110	5.003799329	1672
chr9.fa	123940415	123964365	RAB14	30.51183396	5986
chr9.fa	124030380	124095120	GSN	24.32669321	3496
chr9.fa	124101353	124132545	STOM	1.64947465	228
chr9.fa	124217319	124262306	GGTA1	0	0
chr9.fa	124329399	124547809	DAB2IP	9.836579917	2584
chr9.fa	124584204	124855885	TTLL11	2.389369777	380
chr9.fa	124906338	124922098	NDUFA8	54.13354745	2052
chr9.fa	124922190	124962367	MORN5	0	0
chr9.fa	124964858	124991019	LHX6	0	0
chr9.fa	125001834	125023787	RBM18	10.61272479	1497
chr9.fa	125026882	125085742	MRRF	13.71152212	1140
chr9.fa	125133229	125157981	PTGS1	0.331807493	76

chr9.fa	125239237	125240205	OR1J1	0	0
chr9.fa	125273081	125274022	OR1J2	0	0
chr9.fa	125281420	125282361	OR1J4	0	0
chr9.fa	125288637	125289572	OR1N1	0	0
chr9.fa	125315449	125316441	OR1N2	0	0
chr9.fa	125329827	125330756	OR1L8	0	0
chr9.fa	125377017	125377961	OR1Q1	0	0
chr9.fa	125390858	125391814	OR1B1	0	0
chr9.fa	125423995	125424927	OR1L1	0	0
chr9.fa	125437409	125438383	OR1L3	0	0
chr9.fa	125486269	125487204	OR1L4	0	0
chr9.fa	125512127	125513062	OR1L6	0	0
chr9.fa	125551212	125552174	OR5C1	0	0
chr9.fa	125562402	125563352	OR1K1	0	0
chr9.fa	125580376	125590935	PDCL	6.026575912	836
chr9.fa	125611732	125667562	RC3H2	20.13061829	3800
chr9.fa	125642492	125642598	SNORD90	0	0
chr9.fa	125670335	125675609	ZBTB6	5.351174198	988
chr9.fa	125680378	125693779	ZBTB26	1.769788225	152
chr9.fa	125703288	125867147	RABGAP1	41.58201721	9347
chr9.fa	125796846	125797895	GPR21	0	0
chr9.fa	125871773	125877756	NCRNA00287	1.435756821	380
chr9.fa	125883908	126030843	STRBP	7.432087546	2204
chr9.fa	126118448	126141032	CRB2	0	0
chr9.fa	126141933	126692417	DENND1A	7.207027772	1738
chr9.fa	126164804	126164882	MIR601	0	0
chr9.fa	126773889	126795442	LHX2	0	0
chr9.fa	127019885	127114719	NEK6	23.24097995	3410
chr9.fa	127115744	127177721	PSMB7	83.79851617	2242
chr9.fa	127115989	127121463	LOC100129034	14.28840458	3420
chr9.fa	127213423	127239379	GPR144	0	0
chr9.fa	127243515	127269699	NR5A1	0	0
chr9.fa	127284703	127533576	NR6A1	2.687151435	228
chr9.fa	127454721	127454830	MIR181A2	0	0
chr9.fa	127455989	127456077	MIR181B2	0	0
chr9.fa	127539437	127577159	OLFML2A	0	0
chr9.fa	127615755	127620157	WDR38	1.35324973	76
chr9.fa	127620161	127624240	RPL35	861.7289679	17708
chr9.fa	127631484	127639696	ARPC5L	29.68098088	1368
chr9.fa	127640573	127703386	GOLGA1	15.2331219	3344
chr9.fa	127704888	127905838	SCAI	2.136511118	1173
chr9.fa	127908852	127952218	PPP6C	13.55829466	2652
chr9.fa	127962821	127996438	RABEPK	3.047202596	228

chr9.fa	127997127	128003666	HSPA5	15.75218268	2812
chr9.fa	128024111	128127290	GAPVD1	20.85427887	6460
chr9.fa	128199673	128469513	MAPKAP1	33.88595145	5624
chr9.fa	128509617	128729655	PBX3	14.15741624	1976
chr9.fa	129089128	129269319	FAM125B	1.751774547	532
chr9.fa	129376722	129463311	LMX1B	0	0
chr9.fa	129567285	129600487	ZBTB43	5.377416346	1444
chr9.fa	129622944	129648156	ZBTB34	4.893048571	1444
chr9.fa	129677053	129985445	RALGPS1	2.669804931	912
chr9.fa	129849628	129885044	ANGPTL2	0	0
chr9.fa	130026756	130155828	GARNL3	5.425897602	912
chr9.fa	130159465	130170165	SLC2A8	2.396263901	228
chr9.fa	130186653	130207651	ZNF79	7.292203556	684
chr9.fa	130209955	130213684	RPL12	816.9611984	23180
chr9.fa	130210781	130210916	SNORA65	12.42765841	76
chr9.fa	130213765	130265780	LRSAM1	13.88498716	2154
chr9.fa	130267618	130341268	FAM129B	96.77370142	17480
chr9.fa	130374486	130454995	STXBP1	1.71330089	304
chr9.fa	130452966	130453053	MIR3911	0	0
chr9.fa	130469271	130476226	C9orf117	0	0
chr9.fa	130476304	130477936	PTRH1	0	0
chr9.fa	130478358	130493802	TTC16	0	0
chr9.fa	130493880	130497604	TOR2A	0	0
chr9.fa	130500596	130541048	SH2D3C	0	0
chr9.fa	130548197	130548286	MIR2861	0	0
chr9.fa	130548305	130553052	CDK9	22.56290954	2508
chr9.fa	130565154	130576556	FPGS	13.30343448	1593
chr9.fa	130577291	130617047	ENG	0	0
chr9.fa	130628759	130640022	AK1	0.745899687	76
chr9.fa	130647601	130661871	ST6GALNAC6	7.04824054	760
chr9.fa	130670165	130679305	ST6GALNAC4	0	0
chr9.fa	130683808	130693076	PIP5KL1	0.989640312	76
chr9.fa	130697374	130700763	DPM2	7.652699499	532
chr9.fa	130702861	130742812	FAM102A	24.55731276	4788
chr9.fa	130823512	130829599	NAIF1	1.989510613	304
chr9.fa	130830479	130871524	SLC25A25	8.656350448	1520
chr9.fa	130873450	130881013	LOC100289019	1.759780626	304
chr9.fa	130882972	130890712	PTGES2	13.70596234	1444
chr9.fa	130890808	130892913	LOC389791	0	0
chr9.fa	130911732	130915734	LCN2	12.33692284	456
chr9.fa	130922539	130926207	C9orf16	47.41022028	1520
chr9.fa	130928344	130966662	CIZ1	21.19342527	3040
chr9.fa	130965663	131017527	DNM1	0	0

chr9.fa	131007000	131007109	MIR199B	0	0
chr9.fa	131007226	131007309	MIR3154	0	0
chr9.fa	131018108	131038268	GOLGA2	37.0338972	7094
chr9.fa	131038425	131051268	C9orf119	25.84362278	1140
chr9.fa	131071396	131084697	TRUB2	23.49094753	1520
chr9.fa	131084791	131096351	COQ4	22.57869931	1596
chr9.fa	131102839	131123749	SLC27A4	2.115383965	304
chr9.fa	131133598	131153015	URM1	41.29646706	2544
chr9.fa	131154897	131154993	MIR219-2	0	0
chr9.fa	131182759	131199630	CERCAM	0.628477196	76
chr9.fa	131217434	131263571	ODF2	5.98098574	1292
chr9.fa	131266971	131304580	GLE1	11.63194312	1748
chr9.fa	131314837	131395939	SPTAN1	34.43103199	12234
chr9.fa	131395945	131419129	WDR34	24.23862634	1976
chr9.fa	131445934	131458675	SET	68.67481069	10104
chr9.fa	131464802	131483148	PKN3	0	0
chr9.fa	131483200	131486408	ZDHHC12	13.82872222	684
chr9.fa	131492067	131534198	ZER1	21.3097358	4104
chr9.fa	131549510	131572711	TBC1D13	20.85161017	3637
chr9.fa	131580779	131583127	ENDOG	10.61672783	380
chr9.fa	131584345	131592085	C9orf114	10.0938864	684
chr9.fa	131595392	131644354	CCBL1	3.511999956	304
chr9.fa	131644391	131680317	LRRC8A	5.324042486	1140
chr9.fa	131683174	131704320	PHYHD1	9.490984177	912
chr9.fa	131707809	131709976	DOLK	1.559183871	152
chr9.fa	131710013	131769374	NUP188	8.969699482	2280
chr9.fa	131770313	131790579	SH3GLB2	51.10280179	4559
chr9.fa	131799253	131834351	FAM73B	5.654293242	912
chr9.fa	131843383	131852717	DOLPP1	3.883615453	380
chr9.fa	131857073	131873070	CRAT	71.59992058	9224
chr9.fa	131873228	131911225	PPP2R4	59.60725913	8132
chr9.fa	131937831	131940540	IER5L	0	0
chr9.fa	132083295	132084882	C9orf106	0	0
chr9.fa	132374504	132383055	C9orf50	0	0
chr9.fa	132388435	132396585	METTL11A	10.26134688	281
chr9.fa	132396883	132404444	ASB6	6.800719266	1292
chr9.fa	132427920	132484951	PRRX2	0	0
chr9.fa	132500615	132515344	PTGES	0	0
chr9.fa	132565432	132573560	TOR1B	5.49350449	684
chr9.fa	132575221	132586441	TOR1A	6.420430517	608
chr9.fa	132589564	132597572	C9orf78	35.02236987	2863
chr9.fa	132597696	132644107	USP20	11.42556419	2277
chr9.fa	132649466	132805473	FNBP1	9.945106765	2426

chr9.fa	132815985	132902448	GPR107	5.014696492	1596
chr9.fa	132934857	132999583	NCS1	7.421857556	1672
chr9.fa	133320094	133376661	ASS1	200.4768865	16659
chr9.fa	133452737	133454881	LOC100272217	0	0
chr9.fa	133454960	133513739	FUBP3	24.51439128	3480
chr9.fa	133539981	133558384	PRDM12	0	0
chr9.fa	133569158	133580452	EXOSC2	7.703627056	760
chr9.fa	133589268	133763062	ABL1	30.89523619	8284
chr9.fa	133768815	133769225	QRFP	4.112233484	76
chr9.fa	133777825	133814455	FIBCD1	0	0
chr9.fa	133884504	133968446	LAMC3	0	0
chr9.fa	133971863	133998539	AIF1L	47.58435249	7568
chr9.fa	134000981	134109091	NUP214	14.33866497	4256
chr9.fa	134133465	134151906	FAM78A	0.429437178	76
chr9.fa	134165081	134184649	PPAPDC3	0	0
chr9.fa	134305477	134375575	PRRC2B	31.33067792	15580
chr9.fa	134378289	134399182	POMT1	0.550640317	76
chr9.fa	134399194	134406662	UCK1	19.48990958	1900
chr9.fa	134452157	134612925	RAPGEF1	20.0954805	5700
chr9.fa	134735499	134955253	MED27	3.663670673	228
chr9.fa	135037334	135118220	NTNG2	1.093719338	152
chr9.fa	135136827	135230372	SETX	15.18953324	7524
chr9.fa	135250937	135282221	TTF1	9.704257224	1368
chr9.fa	135285611	135448675	C9orf171	0	0
chr9.fa	135457993	135465640	BARHL1	0	0
chr9.fa	135469676	135545727	DDX31	5.898478649	988
chr9.fa	135545789	135565470	GTF3C4	19.99407016	3420
chr9.fa	135600965	135754198	AK8	2.407605846	228
chr9.fa	135754290	135765418	C9orf9	7.052243579	228
chr9.fa	135766735	135820020	TSC1	14.09292282	5460
chr9.fa	135854098	135867084	GFI1B	0	0
chr9.fa	135906062	135933890	GTF3C5	13.84829263	1520
chr9.fa	135937365	135947248	CEL	0.708982767	76
chr9.fa	135957926	135962478	CELP	0	0
chr9.fa	135973107	136024588	RALGDS	6.112641261	1064
chr9.fa	136028340	136039301	GBGT1	0	0
chr9.fa	136080666	136084628	OBP2B	0	0
chr9.fa	136130563	136150630	ABO	5.352063763	380
chr9.fa	136197552	136203047	SURF6	21.88394957	2280
chr9.fa	136207755	136214972	MED22	10.91273036	1900
chr9.fa	136215069	136218280	RPL7A	609.5514923	24394
chr9.fa	136216251	136216325	SNORD24	0	0
chr9.fa	136216949	136217019	SNORD36B	0	0

chr9.fa	136217311	136217382	SNORD36A	0	0
chr9.fa	136217701	136217767	SNORD36C	0	0
chr9.fa	136218666	136223361	SURF1	25.7133016	1199
chr9.fa	136223421	136228040	SURF2	18.0232404	684
chr9.fa	136228340	136242970	SURF4	10.24689146	1368
chr9.fa	136243284	136271185	C9orf96	0	0
chr9.fa	136271221	136283164	REXO4	16.72002867	1748
chr9.fa	136279459	136324508	ADAMTS13	0.645156527	152
chr9.fa	136325087	136335909	C9orf7	3.681017178	456
chr9.fa	136336216	136344276	SLC2A6	2.655349511	304
chr9.fa	136379708	136390068	TMEM8C	0	0
chr9.fa	136397286	136440641	ADAMTSL2	0	0
chr9.fa	136443537	136445368	FAM163B	0	0
chr9.fa	136501485	136524466	DBH	0	0
chr9.fa	136528684	136605077	SARDH	4.369984748	684
chr9.fa	136627016	136857446	VAV2	9.40736513	2052
chr9.fa	136890561	136895445	NCRNA00094	5.684538429	1140
chr9.fa	136896720	136933141	BRD3	22.86625098	4484
chr9.fa	137001210	137025094	WDR5	16.85235136	2412
chr9.fa	137029562	137029686	RNU6ATAC	0	0
chr9.fa	137218316	137332431	RXRA	41.93295033	10412
chr9.fa	137533652	137736688	COL5A1	0	0
chr9.fa	137741021	137742490	MIR3689A	0	0
chr9.fa	137741971	137742118	MIR3689B	0	0
chr9.fa	137772658	137779366	FCN2	0	0
chr9.fa	137801431	137809806	FCN1	0	0
chr9.fa	137967089	138013030	OLFM1	0	0
chr9.fa	138371648	138380739	KIAA0649	9.938212641	2204
chr9.fa	138387026	138391761	C9orf116	10.00092693	304
chr9.fa	138392483	138396519	MRPS2	19.09160716	1292
chr9.fa	138413286	138418378	LCN1	0	0
chr9.fa	138437985	138441815	OBP2A	0	0
chr9.fa	138453604	138458622	PAEP	0	0
chr9.fa	138466771	138478958	LOC100130954	0	0
chr9.fa	138515502	138531386	GLT6D1	0	0
chr9.fa	138555168	138557755	LCN9	0	0
chr9.fa	138585255	138591374	SOHLH1	0	0
chr9.fa	138594031	138684993	KCNT1	0	0
chr9.fa	138700333	138799005	CAMSAP1	12.18191626	4180
chr9.fa	138824815	138853226	UBAC1	14.48455352	1216
chr9.fa	138903203	138987131	NACC2	5.690765379	532
chr9.fa	139006427	139010731	C9orf69	13.66393043	1748
chr9.fa	139088096	139096955	LHX3	0	0

chr9.fa	139098182	139137687	QSOX2	0	0
chr9.fa	139216998	139221779	LOC26102	0	0
chr9.fa	139221932	139254057	GPSM1	1.479345473	304
chr9.fa	139256352	139258241	DNLZ	9.337979113	304
chr9.fa	139258408	139268133	CARD9	1.592320142	152
chr9.fa	139270029	139292889	SNAPC4	5.332493347	1115
chr9.fa	139296374	139305054	SDCCAG3	6.39418837	684
chr9.fa	139305116	139318213	PMPCA	17.06429006	1596
chr9.fa	139323073	139334256	INPP5E	2.003743643	304
chr9.fa	139334548	139377507	SEC16A	28.77896266	11701
chr9.fa	139377947	139380519	C9orf163	0	0
chr9.fa	139388896	139440238	NOTCH1	0.545525322	228
chr9.fa	139557377	139567130	EGFL7	1.101725417	76
chr9.fa	139565054	139565138	MIR126	0	0
chr9.fa	139567595	139581911	AGPAT2	3.275598236	228
chr9.fa	139607024	139619045	FAM69B	0.76502532	76
chr9.fa	139619171	139622636	SNHG7	8.821587022	906
chr9.fa	139632619	139637411	LCN10	0	0
chr9.fa	139638469	139640366	LCN6	0	0
chr9.fa	139640613	139644363	LOC100128593	0	0
chr9.fa	139648840	139652731	LCN8	0	0
chr9.fa	139654086	139658965	LCN15	0	0
chr9.fa	139685777	139687769	TMEM141	279.6218693	10788
chr9.fa	139690790	139702193	KIAA1984	4.180062764	228
chr9.fa	139698379	139703300	LOC100131193	0.897570404	76
chr9.fa	139702374	139735639	C9orf86	47.15447053	7964
chr9.fa	139725409	139725475	MIR4292	0	0
chr9.fa	139738867	139741797	C9orf172	0	0
chr9.fa	139743256	139745490	PHPT1	44.58496398	2508
chr9.fa	139746819	139755251	MAMDC4	0.923590161	152
chr9.fa	139756571	139760738	EDF1	237.5370259	8171
chr9.fa	139780965	139821067	TRAF2	5.938286653	608
chr9.fa	139834887	139839173	FBXW5	30.99242109	3268
chr9.fa	139839698	139841426	C8G	0	0
chr9.fa	139846768	139849949	LCN12	0	0
chr9.fa	139871956	139876194	PTGDS	0	0
chr9.fa	139877445	139880210	LCNL1	0	0
chr9.fa	139886870	139888428	C9orf142	14.46098006	528
chr9.fa	139889060	139891024	CLIC3	2.078911828	76
chr9.fa	139901686	139923374	ABCA2	1.212253784	456
chr9.fa	139921916	139931234	C9orf139	0	0
chr9.fa	139924626	139926449	FUT7	0	0
chr9.fa	139933909	139940676	NPDC1	46.45771927	3192

chr9.fa	139942553	139948505	ENTPD2	0	0
chr9.fa	139956579	139965028	C9orf140	7.937137692	1368
chr9.fa	139971953	139978990	UAP1L1	5.525084024	836
chr9.fa	139979398	139981269	LOC100289341	0.90290779	76
chr9.fa	139981417	140003639	MAN1B1	4.332178264	532
chr9.fa	140004992	140009195	DPP7	12.54307938	912
chr9.fa	140033609	140063211	GRIN1	0.38051114	76
chr9.fa	140063215	140064491	LRRC26	22.68678138	1216
chr9.fa	140063638	140063722	MIR3621	0	0
chr9.fa	140069236	140083053	ANAPC2	10.03072733	1216
chr9.fa	140083058	140084822	SSNA1	50.50679369	2128
chr9.fa	140086069	140095163	TPRN	3.854482221	456
chr9.fa	140098535	140100090	TMEM203	21.72449517	1520
chr9.fa	140100119	140113813	NDOR1	5.198391525	1140
chr9.fa	140114707	140115775	RNF208	12.64871514	608
chr9.fa	140119644	140120763	C9orf169	0	0
chr9.fa	140122018	140124090	LOC643596	0	0
chr9.fa	140125209	140131006	SLC34A3	1.467781137	152
chr9.fa	140135711	140138036	TUBB2C	145.0692606	9576
chr9.fa	140138160	140142222	FAM166A	0	0
chr9.fa	140145730	140147934	C9orf173	0	0
chr9.fa	140149759	140168000	COBRA1	35.56878476	4104
chr9.fa	140172280	140177093	C9orf167	0.83085308	152
chr9.fa	140194083	140196703	NRARP	6.448674184	760
chr9.fa	140201348	140317714	EXD3	0.586667672	76
chr9.fa	140317847	140328815	NOXA1	4.251895083	304
chr9.fa	140328859	140335901	ENTPD8	0	0
chr9.fa	140342023	140353786	NELF	9.2510242	1520
chr9.fa	140354405	140444986	PNPLA7	2.110046579	456
chr9.fa	140446309	140447007	MRPL41	111.9318775	3040
chr9.fa	140449361	140473387	WDR85	6.405530315	532
chr9.fa	140476531	140484937	ZMYND19	8.635890469	532
chr9.fa	140500096	140509783	ARRDC1	25.69751184	1813
chr9.fa	140509813	140513308	C9orf37	5.938731435	380
chr9.fa	140513444	140730578	EHMT1	21.30328646	5168
chr9.fa	140657474	140659224	FLJ40292	0	0
chr9.fa	140732871	140732968	MIR602	0	0
chr9.fa	140772241	141019076	CACNA1B	0	0
chr9.fa	141044565	141071885	TUBBP5	1.270742639	152
chr9.fa	141106637	141134172	FAM157B	0	0
chrX.fa	192991	220022	PLCXD1	0	0
chrX.fa	221426	230887	GTPBP6	0	0
chrX.fa	281385	282054	NCRNA00107	0	0

chrX.fa	294668	347690	PPP2R3B	0	0
chrX.fa	585079	620146	SHOX	0	0
chrX.fa	1314887	1331527	CRLF2	0	0
chrX.fa	1387693	1428828	CSF2RA	0	0
chrX.fa	1412811	1412885	MIR3690	0	0
chrX.fa	1455509	1501582	IL3RA	0	0
chrX.fa	1505045	1511039	SLC25A6	0	0
chrX.fa	1522032	1572655	ASMTL	0	0
chrX.fa	1532655	1534314	ASMTL-AS	0	0
chrX.fa	1581466	1656037	P2RY8	0	0
chrX.fa	1710486	1721407	AKAP17A	0	0
chrX.fa	1733941	1761974	ASMT	0	0
chrX.fa	2137555	2343345	DHRSX	0	0
chrX.fa	2404455	2418580	ZBED1	0	0
chrX.fa	2527306	2575270	CD99P1	0	0
chrX.fa	2609228	2659350	CD99	0	0
chrX.fa	2670093	2734541	XG	0	0
chrX.fa	2692781	2693037	XGPY2	0	0
chrX.fa	2746863	2800861	GYG2	1.991289742	304
chrX.fa	2822011	2847392	ARSD	5.08208099	1444
chrX.fa	2852673	2882494	ARSE	2.283956405	228
chrX.fa	2924654	2951426	ARSH	0	0
chrX.fa	2984853	3030747	ARSF	2.520135733	228
chrX.fa	3226609	3264684	MXRA5	0.863099787	380
chrX.fa	3522384	3631675	PRKX	1.944587615	532
chrX.fa	3735576	3761935	LOC389906	1.887433107	152
chrX.fa	5808083	6146706	NLGN4X	0	0
chrX.fa	6451659	6453159	VCX3A	0	0
chrX.fa	6966961	7066231	HDHD1	5.525751197	608
chrX.fa	7137472	7272682	STS	5.300913814	1520
chrX.fa	7810303	7812184	VCX	0	0
chrX.fa	7866804	7895780	PNPLA4	11.06973846	1444
chrX.fa	8095006	8095102	MIR651	0	0
chrX.fa	8137985	8139308	VCX2	0	0
chrX.fa	8432871	8434551	VCX3B	0	0
chrX.fa	8496915	8700227	KAL1	0	0
chrX.fa	8758837	8769424	FAM9A	0	0
chrX.fa	8993037	9001116	FAM9B	0	0
chrX.fa	9431335	9687780	TBL1X	12.07672528	3268
chrX.fa	9693453	9734005	GPR143	0.996534435	76
chrX.fa	9754496	9917481	SHROOM2	7.264627062	2432
chrX.fa	9935398	9936042	LOC100288814	0	0
chrX.fa	9983795	10112518	WWC3	12.04648009	3496

chrX.fa	10124985	10205699	CLCN4	0.497933631	152
chrX.fa	10413350	10851809	MID1	5.22196498	2052
chrX.fa	11129406	11141204	HCCS	6.619470535	684
chrX.fa	11155663	11683821	ARHGAP6	16.08065431	4332
chrX.fa	11311533	11318881	AMELX	0	0
chrX.fa	11776278	11793872	MSL3	8.744417317	1064
chrX.fa	12156585	12742642	FRMPD4	0	0
chrX.fa	12809474	12842346	PRPS2	16.69467608	1900
chrX.fa	12885202	12908480	TLR7	0	0
chrX.fa	12920936	12961419	LOC349408	0	0
chrX.fa	12924739	12941288	TLR8	0	0
chrX.fa	12993225	12993287	TMSL3	0	0
chrX.fa	13053736	13062917	FAM9C	0	0
chrX.fa	13336768	13338518	ATXN3L	0	0
chrX.fa	13587694	13651694	EGFL6	0	0
chrX.fa	13671307	13682247	TCEANC	3.458181314	304
chrX.fa	13707240	13727944	RAB9A	18.6141335	1140
chrX.fa	13730361	13752754	TRAPPC2	6.500713697	836
chrX.fa	13752832	13787480	OFD1	16.20274701	2660
chrX.fa	13789062	13956831	GPM6B	0	0
chrX.fa	14024845	14048035	GEMIN8	8.775552068	1286
chrX.fa	14547420	14749933	GLRA2	0	0
chrX.fa	14861529	14891184	FANCB	0.561982262	76
chrX.fa	14891527	14939459	MOSPD2	3.504438659	532
chrX.fa	15262109	15288589	ASB9	0.821290263	76
chrX.fa	15301627	15333727	ASB11	0	0
chrX.fa	15337573	15353676	PIGA	0.932263413	152
chrX.fa	15363718	15402577	FIGF	0	0
chrX.fa	15402924	15511464	PIR	19.03778851	1124
chrX.fa	15518900	15574652	BMX	0	0
chrX.fa	15579156	15620192	ACE2	1.44576442	228
chrX.fa	15645441	15683154	TMEM27	5.355399629	380
chrX.fa	15693039	15721474	CA5BP	3.400804415	152
chrX.fa	15756412	15805748	CA5B	2.438962989	456
chrX.fa	15808574	15841382	ZRSR2	7.82994519	532
chrX.fa	15843929	15873100	AP1S2	0.74033991	76
chrX.fa	16141424	16171641	GRPR	0	0
chrX.fa	16606122	16731059	CTPS2	3.828684856	836
chrX.fa	16668281	16672791	S100G	0	0
chrX.fa	16737707	16780807	SYAP1	27.24201789	4318
chrX.fa	16804555	16862642	TXLNG	4.998239552	988
chrX.fa	16862775	16888534	RBBP7	26.26483148	2812
chrX.fa	16964814	17171403	REPS2	14.8065758	5295

chrX.fa	17393543	17754113	NHS	0.74033991	304
chrX.fa	17755592	17773105	SCML1	1.161326227	152
chrX.fa	17818169	17879457	RAI2	6.170685333	760
chrX.fa	18181051	18239024	BEND2	0	0
chrX.fa	18257433	18372844	SCML2	0	0
chrX.fa	18443725	18671749	CDKL5	21.43627633	3391
chrX.fa	18657808	18690223	RS1	0	0
chrX.fa	18709045	18846034	PPEF1	0	0
chrX.fa	18908414	18913093	LOC100132163	1.758668671	76
chrX.fa	18910416	19002480	PHKA2	3.110806445	698
chrX.fa	19007425	19140755	GPR64	0.696528867	152
chrX.fa	19362011	19379825	PDHA1	17.87490555	2052
chrX.fa	19380856	19533379	MAP3K15	0.918919948	152
chrX.fa	19552083	19905744	SH3KBP1	8.997275976	2052
chrX.fa	19930980	19988382	CXorf23	3.065883447	895
chrX.fa	20004935	20007897	LOC729609	0	0
chrX.fa	20024831	20135114	MAP7D2	1.596323182	304
chrX.fa	20035206	20035305	MIR23C	0	0
chrX.fa	20142636	20159966	EIF1AX	9.536129566	1900
chrX.fa	20154184	20154531	SCARNA9L	0	0
chrX.fa	20168029	20284750	RPS6KA3	14.24926375	4940
chrX.fa	21392536	21672813	CNKSR2	0.476806478	152
chrX.fa	21673609	21676448	KLHL34	0	0
chrX.fa	21724091	21776230	SMPX	0	0
chrX.fa	21857656	21903541	MBTPS2	1.867640301	380
chrX.fa	21874105	21876845	YY2	0	0
chrX.fa	21958715	22012955	SMS	225.2459156	18383
chrX.fa	22050921	22266478	PHEX	0	0
chrX.fa	22291060	22292574	ZNF645	0	0
chrX.fa	23018087	23020206	DDX53	0	0
chrX.fa	23352985	23414918	PTCHD1	1.277859153	304
chrX.fa	23685645	23704514	PRDX4	5.505513609	228
chrX.fa	23721777	23761407	ACOT9	10.76550746	836
chrX.fa	23801275	23804327	SAT1	302.5390479	15971
chrX.fa	23851465	23926057	APOO	17.80707627	904
chrX.fa	23926123	23957624	CXorf58	0	0
chrX.fa	24001833	24045303	KLHL15	1.885209196	532
chrX.fa	24073065	24096927	EIF2S3	52.19296287	8132
chrX.fa	24167762	24234372	ZFX	9.073111335	3192
chrX.fa	24328979	24331432	FAM48B2	0	0
chrX.fa	24380878	24383541	FAM48B1	0	0
chrX.fa	24483344	24568583	PDK3	5.404103276	3192
chrX.fa	24576204	24690979	PCYT1B	0	0

chrX.fa	24712064	25015102	POLA1	4.039733992	988
chrX.fa	24762558	24762687	SCARNA23	0	0
chrX.fa	25021813	25034065	ARX	2.352452858	304
chrX.fa	26156460	26158853	MAGEB18	0	0
chrX.fa	26210557	26213763	MAGEB6	0	0
chrX.fa	26576454	26579169	VENTXP1	0	0
chrX.fa	27478328	27481458	SMEK3P	0	0
chrX.fa	27764926	27766938	DCAF8L2	0	0
chrX.fa	27826107	27841131	MAGEB10	0	0
chrX.fa	27996110	27999566	DCAF8L1	0	0
chrX.fa	28605681	29974017	IL1RAPL1	0	0
chrX.fa	30233675	30238206	MAGEB2	0	0
chrX.fa	30248553	30255610	MAGEB3	0	0
chrX.fa	30260057	30262308	MAGEB4	0	0
chrX.fa	30264406	30270155	MAGEB1	0	0
chrX.fa	30322539	30327495	NR0B1	0	0
chrX.fa	30576941	30596033	CXorf21	0	0
chrX.fa	30671476	30748725	GK	4.083545035	684
chrX.fa	30845559	30907511	TAB3	15.68902361	4788
chrX.fa	31089358	31090170	FTHL17	0	0
chrX.fa	31137345	33357726	DMD	0.430326742	304
chrX.fa	34147869	34150447	FAM47A	0	0
chrX.fa	34645181	34675405	TMEM47	0	0
chrX.fa	34960913	34963034	FAM47B	0	0
chrX.fa	35816459	35821852	MAGEB16	0	0
chrX.fa	35937851	36008269	CXorf22	0	0
chrX.fa	36065053	36163187	CXorf59	0	0
chrX.fa	36254051	36403434	CXorf30	0	0
chrX.fa	37026432	37029739	FAM47C	0.510832314	76
chrX.fa	37208528	37316548	PRRG1	1.109731496	304
chrX.fa	37430822	37536750	LANCL3	2.029096226	304
chrX.fa	37545133	37591383	XK	1.994403217	456
chrX.fa	37639270	37672714	CYBB	0.391408303	76
chrX.fa	37698089	37706889	DYNLT3	13.72886862	1368
chrX.fa	37850070	37850570	CXorf27	0	0
chrX.fa	37865835	37988073	SYTL5	33.2287858	7296
chrX.fa	38008588	38080177	SRPX	0.888229979	76
chrX.fa	38128423	38186788	RPGR	2.36512915	608
chrX.fa	38211736	38280703	OTC	0	0
chrX.fa	38420731	38548172	TSPAN7	1.87164334	152
chrX.fa	38660685	38665783	MID1IP1	20.29830116	2280
chrX.fa	39910499	40036582	BCOR	18.57054485	5548
chrX.fa	40440216	40465888	ATP6AP2	4.150707141	380

chrX.fa	40482818	40483386	LOC347411	0	0
chrX.fa	40486173	40506819	CXorf38	4.772290214	912
chrX.fa	40508795	40594804	MED14	12.98586002	3952
chrX.fa	40690470	40692449	LOC100132831	0	0
chrX.fa	40944888	41095832	USP9X	19.97605649	11096
chrX.fa	41192651	41209524	DDX3X	45.99492343	11160
chrX.fa	41306713	41334905	NYX	0	0
chrX.fa	41374189	41782287	CASK	3.45729175	1292
chrX.fa	41548226	41556530	GPR34	0	0
chrX.fa	41583408	41589388	GPR82	0	0
chrX.fa	42636617	42637486	PPP1R2P9	0	0
chrX.fa	43515409	43606068	MAOA	40.56880344	7430
chrX.fa	43625857	43741721	MAOB	5.85333326	683
chrX.fa	43808024	43832921	NDP	0	0
chrX.fa	44007128	44202923	EFHC2	0	0
chrX.fa	44382885	44402221	FUNDC1	17.3351624	912
chrX.fa	44703249	44704134	DUSP21	0	0
chrX.fa	44732423	44971845	KDM6A	12.80772476	3323
chrX.fa	45007618	45060146	CXorf36	0	0
chrX.fa	45605585	45605694	MIR221	0	0
chrX.fa	45606421	45606530	MIR222	0	0
chrX.fa	46306624	46334074	ZNF673	3.399470069	380
chrX.fa	46357160	46404892	ZNF674	1.652588125	304
chrX.fa	46404928	46407670	LOC401588	3.548916875	304
chrX.fa	46433192	46457838	CHST7	0.731444266	76
chrX.fa	46466373	46618472	SLC9A7	3.072999961	304
chrX.fa	46696347	46741791	RP2	3.53134798	608
chrX.fa	46771736	46920641	PHF16	4.079319604	912
chrX.fa	46937775	46952712	RGN	7.562186328	760
chrX.fa	47001615	47004609	NDUFB11	42.10396908	2128
chrX.fa	47004631	47046212	RBM10	16.43447852	2500
chrX.fa	47050199	47074527	UBA1	84.24774616	14206
chrX.fa	47064247	47065254	INE1	0	0
chrX.fa	47077528	47089394	CDK16	22.4792905	4026
chrX.fa	47092314	47107727	USP11	18.96751293	2806
chrX.fa	47229999	47273098	ZNF157	0	0
chrX.fa	47248049	47248175	SNORA11C	0	0
chrX.fa	47305561	47342345	ZNF41	5.022257789	988
chrX.fa	47420516	47431299	ARAF	16.50297497	1824
chrX.fa	47431321	47479256	SYN1	0	0
chrX.fa	47441690	47446190	TIMP1	12.91625161	532
chrX.fa	47483612	47489704	CFP	0	0
chrX.fa	47494920	47510003	ELK1	7.532385924	988

chrX.fa	47511195	47518231	UXT	150.7026491	2880
chrX.fa	47518252	47519776	LOC100133957	0	0
chrX.fa	47566590	47596027	CXXC1P1	0	0
chrX.fa	47696301	47781655	ZNF81	1.075260878	380
chrX.fa	47834250	47863394	ZNF182	2.813914352	456
chrX.fa	47863734	47991997	SPACA5	0	0
chrX.fa	47917567	47931025	ZNF630	3.487314546	456
chrX.fa	47967367	47980068	SSX6	0	0
chrX.fa	48045656	48056199	SSX5	0	0
chrX.fa	48114797	48126879	SSX1	0	0
chrX.fa	48205863	48216142	SSX3	0	0
chrX.fa	48261524	48261527	SSX4	0	0
chrX.fa	48316927	48328644	SLC38A5	0	0
chrX.fa	48334549	48344752	FTSJ1	13.14664877	1216
chrX.fa	48367371	48379202	PORCN	0	0
chrX.fa	48380164	48387104	EBP	13.14731594	684
chrX.fa	48398075	48420997	TBC1D25	3.547137747	608
chrX.fa	48432836	48436804	RBM3	65.9889936	4712
chrX.fa	48455880	48463582	WDR13	19.89154788	2508
chrX.fa	48542186	48549817	WAS	0	0
chrX.fa	48555131	48567406	SUV39H1	3.096795807	380
chrX.fa	48620154	48632064	GLOD5	0	0
chrX.fa	48644982	48652717	GATA1	0	0
chrX.fa	48660487	48683380	HDAC6	13.01566042	2399
chrX.fa	48687283	48688548	ERAS	0	0
chrX.fa	48689506	48693960	PCSK1N	6.948386944	304
chrX.fa	48750730	48755426	TIMM17B	18.71042884	912
chrX.fa	48755195	48760420	PQBP1	26.50768254	1509
chrX.fa	48760459	48768917	SLC35A2	3.922978674	456
chrX.fa	48770459	48776413	PIM2	0.772809008	76
chrX.fa	48779303	48815648	OTUD5	16.46272218	2280
chrX.fa	48818639	48828251	KCND1	0.358049641	76
chrX.fa	48830134	48858675	GRIPAP1	15.3801224	3054
chrX.fa	48886242	48900990	TFE3	14.44585747	2204
chrX.fa	48916516	48927510	CCDC120	8.433959367	1520
chrX.fa	48928818	48931662	PRAF2	0	0
chrX.fa	48932092	48958059	WDR45	17.33938783	1596
chrX.fa	48969022	48980079	GPKOW	9.090013057	1216
chrX.fa	49019181	49023836	MAGIX	3.467076957	456
chrX.fa	49028184	49031468	PLP2	19.35313907	912
chrX.fa	49031906	49042776	PRICKLE3	2.547934618	228
chrX.fa	49044265	49056661	SYP	0	0
chrX.fa	49061523	49089833	CACNA1F	0	0

chrX.fa	49091927	49106896	CCDC22	9.103356522	912
chrX.fa	49106988	49121288	FOXP3	0	0
chrX.fa	49126306	49144555	PPP1R3F	0	0
chrX.fa	49160125	49176311	GAGE10	0	0
chrX.fa	49178516	49185866	GAGE12J	0	0
chrX.fa	49188094	49188103	GAGE13	0	0
chrX.fa	49197563	49226170	GAGE2D	0	0
chrX.fa	49197596	49223953	GAGE2C	0	0
chrX.fa	49204807	49301555	GAGE2E	0	0
chrX.fa	49216634	49361430	GAGE2A	0	0
chrX.fa	49216649	49216656	GAGE4	0	0
chrX.fa	49216678	49216722	GAGE7	0	0
chrX.fa	49235708	49242997	GAGE2B	0	0
chrX.fa	49296769	49351917	GAGE12D	0	0
chrX.fa	49296815	49315999	GAGE12B	0	0
chrX.fa	49325480	49335064	GAGE6	0	0
chrX.fa	49332808	49332811	GAGE12G	0	0
chrX.fa	49342357	49342360	GAGE12E	0	0
chrX.fa	49363616	49373139	GAGE1	0	0
chrX.fa	49452054	49460596	PAGE1	0	0
chrX.fa	49593906	49598570	PAGE4	0	0
chrX.fa	49641327	49643959	LOC158572	1.476231998	152
chrX.fa	49644470	49647168	USP27X	2.504790749	304
chrX.fa	49687225	49863892	CLCN5	0.655831299	304
chrX.fa	49767754	49767844	MIR532	0	0
chrX.fa	49768109	49768194	MIR188	0	0
chrX.fa	49773039	49773122	MIR500A	0	0
chrX.fa	49773572	49773635	MIR362	0	0
chrX.fa	49774330	49774413	MIR501	0	0
chrX.fa	49775280	49775358	MIR500B	0	0
chrX.fa	49777849	49777945	MIR660	0	0
chrX.fa	49779206	49779291	MIR502	0	0
chrX.fa	49955411	49965664	AKAP4	0	0
chrX.fa	50027540	50094911	CCNB3	0.374728972	76
chrX.fa	50111897	50213737	DGKK	0	0
chrX.fa	50334643	50557044	SHROOM4	0.844196545	228
chrX.fa	50648432	50648882	LOC347376	0	0
chrX.fa	50653735	50659641	BMP15	0	0
chrX.fa	51075083	51080377	NUDT10	2.53392398	228
chrX.fa	51232863	51239459	NUDT11	7.759447218	836
chrX.fa	51486481	51489326	GSPT2	8.314312965	1064
chrX.fa	51546155	51645450	MAGED1	42.57699491	5688
chrX.fa	51811270	51934969	MAGED4B	0	0

chrX.fa	52112078	52387096	XAGE2	0	0
chrX.fa	52240600	52240615	XAGE1B	0	0
chrX.fa	52260211	52260363	XAGE1A	0	0
chrX.fa	52513551	52513566	XAGE1D	0	0
chrX.fa	52651985	52662998	SSX8	0	0
chrX.fa	52673111	52683950	SSX7	0	0
chrX.fa	52727836	52788723	SSX2	0	0
chrX.fa	52825186	52826388	SPANXN5	0	0
chrX.fa	52841228	52847322	XAGE5	0	0
chrX.fa	52891558	52897119	XAGE3	0	0
chrX.fa	52927862	52986176	FAM156A	0	0
chrX.fa	53078506	53109796	GPR173	0.397635253	76
chrX.fa	53111542	53117728	TSPYL2	8.964806878	1140
chrX.fa	53220503	53254604	KDM5C	19.1129567	5472
chrX.fa	53262058	53350522	IQSEC2	3.396356594	1064
chrX.fa	53401070	53449618	SMC1A	3.128375341	1368
chrX.fa	53449839	53458057	RIBC1	0	0
chrX.fa	53458206	53461323	HSD17B10	47.38798117	2052
chrX.fa	53559063	53713673	HUWE1	52.00993501	34458
chrX.fa	53583184	53583302	MIR98	0	0
chrX.fa	53584153	53584235	MIRLET7F2	0	0
chrX.fa	53963113	54071569	PHF8	10.51687423	3420
chrX.fa	54094757	54209714	FAM120C	1.601882959	608
chrX.fa	54219256	54384438	WNK3	5.364295272	2736
chrX.fa	54466853	54471731	TSR2	44.61231808	2660
chrX.fa	54471887	54522599	FGD1	2.767434616	532
chrX.fa	54556644	54593720	GNL3L	7.885987743	3040
chrX.fa	54775332	54824673	ITIH5L	0.680516709	152
chrX.fa	54834171	54842445	MAGED2	97.10795521	9995
chrX.fa	54840803	54840933	SNORA11	0	0
chrX.fa	54947249	54957865	TRO	0	0
chrX.fa	54959590	55020511	PFKFB1	0	0
chrX.fa	55026780	55034212	APEX2	11.11399429	988
chrX.fa	55035488	55057497	ALAS2	0	0
chrX.fa	55101504	55105336	PAGE2B	0	0
chrX.fa	55115497	55119260	PAGE2	0	0
chrX.fa	55169535	55187628	FAM104B	5.938286653	608
chrX.fa	55207824	55208944	MTRNR2L10	0	0
chrX.fa	55246791	55250541	PAGE5	0	0
chrX.fa	55284849	55291165	PAGE3	0	0
chrX.fa	55478538	55479999	MAGEH1	0	0
chrX.fa	55511049	55515631	USP51	0.767694013	152
chrX.fa	55649833	55652621	FOXR2	0	0

chrX.fa	55744110	55785207	RRAGB	6.892344392	684
chrX.fa	56258822	56314322	KLF8	3.11792296	760
chrX.fa	56590026	56593443	UBQLN2	19.77968516	3040
chrX.fa	56755718	56844004	LOC550643	58.44971355	1824
chrX.fa	56763221	56764017	LOC442454	0	0
chrX.fa	57002803	57021988	SPIN3	3.028966527	760
chrX.fa	57146115	57147979	SPIN2B	2.684927525	152
chrX.fa	57162083	57164058	SPIN2A	0	0
chrX.fa	57313110	57515629	FAAH2	3.360106847	304
chrX.fa	57618269	57623910	ZXDB	4.194073402	1064
chrX.fa	57931864	57937067	ZXDA	1.623899676	380
chrX.fa	62567107	62571218	SPIN4	1.233158546	228
chrX.fa	62646439	62780873	LOC92249	13.46978301	1748
chrX.fa	62854848	63005426	ARHGEF9	5.46059061	1444
chrX.fa	63005882	63005967	MIR1468	0	0
chrX.fa	63404997	63425624	FAM123B	3.00272438	1140
chrX.fa	63444076	63445503	ASB12	0	0
chrX.fa	63487961	63615333	MTMR8	5.081413817	608
chrX.fa	64136250	64254593	ZC4H2	5.079857079	532
chrX.fa	64708615	64727767	ZC3H12B	1.164662093	380
chrX.fa	64732462	64754686	LAS1L	9.319075871	1043
chrX.fa	64770502	64772301	FKSG43	0	0
chrX.fa	64887511	64961793	MSN	10.64341476	1900
chrX.fa	65238712	65238821	MIR223	0	0
chrX.fa	65241580	65259967	VSIG4	0	0
chrX.fa	65382433	65487230	HEPH	0	0
chrX.fa	65815482	65859108	EDA2R	0.968513159	152
chrX.fa	66763874	66944119	AR	16.91684477	3420
chrX.fa	67262186	67653299	OPHN1	10.45949734	3542
chrX.fa	67718624	67757127	YIPF6	3.497544536	988
chrX.fa	67867511	67945684	STARD8	1.646361175	380
chrX.fa	68048840	68062006	EFNB1	1.522711734	228
chrX.fa	68380581	68385365	PJA1	7.042458372	912
chrX.fa	68725078	68752351	FAM155B	1.682166139	304
chrX.fa	68835911	69259321	EDA	0.607350043	152
chrX.fa	69242707	69242773	MIR676	0	0
chrX.fa	69260392	69269788	AWAT2	0	0
chrX.fa	69282341	69284029	OTUD6A	0	0
chrX.fa	69353318	69386173	IGBP1	62.10871401	4636
chrX.fa	69397336	69425553	DGAT2L6	0	0
chrX.fa	69454505	69460511	AWAT1	0	0
chrX.fa	69478016	69479654	P2RY4	0	0
chrX.fa	69488185	69501690	ARR3	0	0

chrX.fa	69502022	69504852	RAB41	0	0
chrX.fa	69506211	69509798	PDZD11	28.4860736	1596
chrX.fa	69509879	69640774	KIF4A	0.375618536	76
chrX.fa	69642881	69653241	GDPD2	0	0
chrX.fa	69664705	69725339	DLG3	13.20847349	4028
chrX.fa	69748790	70128567	TEX11	0	0
chrX.fa	70145430	70150975	SLC7A3	0	0
chrX.fa	70280788	70288231	SNX12	47.38798117	1368
chrX.fa	70315999	70323384	FOXO4	15.93009554	2280
chrX.fa	70323739	70326638	CXorf65	0	0
chrX.fa	70327254	70331481	IL2RG	0	0
chrX.fa	70338406	70362304	MED12	9.943550027	3116
chrX.fa	70364681	70391051	NLGN3	0	0
chrX.fa	70430035	70948962	BCYRN1	0	0
chrX.fa	70435062	70445065	GJB1	2.925777066	228
chrX.fa	70459474	70475047	ZMYM3	10.5475642	3116
chrX.fa	70503042	70521018	NONO	73.41396463	10623
chrX.fa	70521628	70525204	ITGB1BP2	0	0
chrX.fa	70586114	70685855	TAF1	13.18378808	4560
chrX.fa	70711377	70712604	INGX	0	0
chrX.fa	70752912	70795747	OGT	18.19047849	4484
chrX.fa	70797874	70833433	ACRC	1.063696542	152
chrX.fa	70835766	70838367	CXCR3	0	0
chrX.fa	71130938	71363424	NHSL2	1.838062287	304
chrX.fa	71264259	71264811	RPS26P11	0	0
chrX.fa	71346961	71351751	RGAG4	1.558516698	304
chrX.fa	71364034	71381600	FLJ44635	0	0
chrX.fa	71401526	71483814	PIN4	12.43744361	1444
chrX.fa	71424507	71458858	ERCC6L	0	0
chrX.fa	71492453	71497141	RPS4X	1881.112085	80864
chrX.fa	71521488	71527037	CITED1	9.40736513	684
chrX.fa	71549366	71792953	HDAC8	8.109713171	1292
chrX.fa	71798664	71934029	PHKA1	6.584110353	1824
chrX.fa	72001689	72158798	LOC100129407	0	0
chrX.fa	72223352	72225551	PABPC1L2B	0	0
chrX.fa	72297177	72299351	PABPC1L2A	0	0
chrX.fa	72345876	72347919	NAP1L6	0.82685004	76
chrX.fa	72432137	72434710	NAP1L2	3.283159533	380
chrX.fa	72667090	72674421	CDX4	0	0
chrX.fa	72744111	72782921	LOC139201	0	0
chrX.fa	72782984	72906937	CHIC1	2.446301894	760
chrX.fa	73012040	73048902	TSIX	0	0
chrX.fa	73050901	73072588	XIST	0	0

chrX.fa	73164159	73290217	NCRNA00183	1.012101811	76
chrX.fa	73247971	73513409	NCRNA00182	1.434867257	152
chrX.fa	73438212	73438296	MIR421	0	0
chrX.fa	73438382	73438383	MIR374B	0	0
chrX.fa	73506939	73507044	MIR545	0	0
chrX.fa	73507121	73507192	MIR374A	0	0
chrX.fa	73524025	73524869	ZCCHC13	0	0
chrX.fa	73641085	73753752	SLC16A2	0.386515699	76
chrX.fa	73802811	73834461	RLIM	13.1982435	6308
chrX.fa	73952691	74145287	KIAA2022	0.721214277	380
chrX.fa	74273105	74376132	ABCB7	6.372616435	684
chrX.fa	74493894	74524732	UPRT	5.157471566	596
chrX.fa	74588262	74743337	ZDHHC15	0	0
chrX.fa	74960373	74962914	TTC3P1	0	0
chrX.fa	75002823	75005079	MAGEE2	0	0
chrX.fa	75392771	75398033	CXorf26	14.14362799	760
chrX.fa	75648046	75651746	MAGEE1	1.826720342	304
chrX.fa	76139698	76139785	MIR384	0	0
chrX.fa	76709647	76712013	FGF16	0	0
chrX.fa	76760356	77041719	ATRX	14.12005453	7101
chrX.fa	77081862	77151065	MAGT1	4.203413828	760
chrX.fa	77154961	77160881	COX7B	155.6737569	3192
chrX.fa	77166194	77305892	ATP7A	2.389592169	912
chrX.fa	77223458	77225135	PGAM4	0	0
chrX.fa	77359666	77382324	PGK1	80.6134311	8783
chrX.fa	77385245	77395179	TAF9B	8.171093109	988
chrX.fa	77528130	77583087	CYSLTR1	1.116403228	76
chrX.fa	77911566	77914825	ZCCHC5	0	0
chrX.fa	78003206	78012578	LPAR4	0	0
chrX.fa	78156691	78156746	MIR4328	0	0
chrX.fa	78200829	78217438	P2RY10	0	0
chrX.fa	78426469	78427726	GPR174	0	0
chrX.fa	78615881	78623049	ITM2A	0	0
chrX.fa	79270255	79287268	TBX22	0	0
chrX.fa	79591003	79700810	FAM46D	0	0
chrX.fa	79924987	80065233	BRWD3	4.359754758	2508
chrX.fa	80369200	80457302	HMGN5	2.551937658	228
chrX.fa	80457442	80554046	SH3BGRL	27.24668811	2356
chrX.fa	82763269	82764775	POU3F4	2.243036446	152
chrX.fa	83116170	83141626	CYLC1	0	0
chrX.fa	83313354	83442943	RPS6KA6	3.089901684	1140
chrX.fa	83572882	83757487	HDX	1.067254799	304
chrX.fa	84189157	84189896	UBE2DNL	0	0

chrX.fa	84258898	84348324	APOOL	7.370040434	2052
chrX.fa	84349134	84363974	SATL1	0	0
chrX.fa	84498997	84528368	ZNF711	0.405641332	76
chrX.fa	84532395	84634748	POF1B	21.75474035	3862
chrX.fa	85116185	85302566	CHM	4.260568336	1520
chrX.fa	85403455	86087605	DACH2	2.02175732	228
chrX.fa	86772715	86925050	KLHL4	0	0
chrX.fa	88002226	88009785	CPXCR1	0	0
chrX.fa	89176940	89177882	TGIF2LX	0	0
chrX.fa	90689597	90693583	PABPC5	0	0
chrX.fa	91034260	91878228	PCDH11X	0	0
chrX.fa	92925925	92928682	NAP1L3	0	0
chrX.fa	92929012	92967273	FAM133A	0	0
chrX.fa	95592085	95592901	LOC643486	0	0
chrX.fa	95939662	96855597	DIAPH2	4.284808963	988
chrX.fa	96138907	96140450	RPA4	0	0
chrX.fa	98716600	99194841	LOC442459	0	0
chrX.fa	99546642	99665271	PCDH19	0	0
chrX.fa	99839790	99854882	TNMD	0	0
chrX.fa	99883795	99891794	TSPAN6	13.07036863	1216
chrX.fa	99899163	99926296	SRPX2	0	0
chrX.fa	99929489	99987135	SYTL4	55.46344611	10108
chrX.fa	100075348	100095923	CSTF2	5.030263868	456
chrX.fa	100098313	100129334	NOX1	0	0
chrX.fa	100168431	100183898	XKRX	0	0
chrX.fa	100224697	100245820	ARL13A	0	0
chrX.fa	100264334	100307105	TRMT2B	7.062028787	1140
chrX.fa	100333836	100351355	TMEM35	0	0
chrX.fa	100354798	100417978	CENPI	0	0
chrX.fa	100474933	100519485	DRP2	0	0
chrX.fa	100523241	100548059	TAF7L	0	0
chrX.fa	100600644	100603957	TIMM8A	5.615152412	456
chrX.fa	100604435	100641212	BTK	0	0
chrX.fa	100650716	100651142	RPL36A	7.187457357	138
chrX.fa	100652779	100663001	GLA	0	0
chrX.fa	100663121	100663295	HNRNPH2	34.81999399	274
chrX.fa	100673266	100788446	ARMCX4	0.312459469	76
chrX.fa	100805514	100809675	ARMCX1	14.263052	1368
chrX.fa	100870108	100872991	ARMCX6	6.111084523	532
chrX.fa	100878120	100882831	ARMCX3	21.46251847	3344
chrX.fa	100910268	100914863	ARMCX2	11.74536257	1520
chrX.fa	101087085	101112549	NXF5	0	0
chrX.fa	101137260	101187039	ZMAT1	0.96873555	304

chrX.fa	101380660	101382684	TCEAL2	23.02681734	1140
chrX.fa	101394933	101397388	TCEAL6	0	0
chrX.fa	101408679	101410986	BEX5	11.63349985	532
chrX.fa	101485190	101711835	NXF2B	0	0
chrX.fa	101502170	101694853	NXF2	0	0
chrX.fa	101768610	101771699	TMSB15A	60.907135	1824
chrX.fa	101804893	101826621	NXF4	0	0
chrX.fa	101854096	101859085	ARMCX5	11.11555103	1379
chrX.fa	101854776	101972661	GPRASP2	6.095739538	1140
chrX.fa	101906294	101914010	GPRASP1	3.370781619	912
chrX.fa	101975642	102007369	BHLHB9	2.368909798	456
chrX.fa	102192200	102193228	RAB40AL	0	0
chrX.fa	102317581	102319168	BEX1	0	0
chrX.fa	102330750	102348022	NXF3	0	0
chrX.fa	102470020	102472095	BEX4	33.83057607	1900
chrX.fa	102507923	102510121	TCEAL8	32.07413131	1748
chrX.fa	102528618	102531797	TCEAL5	0	0
chrX.fa	102564274	102565974	BEX2	14.37224602	760
chrX.fa	102585114	102587251	TCEAL7	0	0
chrX.fa	102611380	102613397	WBP5	52.76940055	2584
chrX.fa	102631268	102633001	NGFRAP1	123.439504	6916
chrX.fa	102754681	102774417	RAB40A	0	0
chrX.fa	102840419	102842655	TCEAL4	78.85720874	4560
chrX.fa	102862834	102864855	TCEAL3	58.59271102	2964
chrX.fa	102883648	102885876	TCEAL1	14.66246638	836
chrX.fa	102930426	102943086	MORF4L2	69.78476457	6916
chrX.fa	102962272	102983552	GLRA4	0	0
chrX.fa	102965837	102968960	TMEM31	0	0
chrX.fa	103031439	103047547	PLP1	1.105728456	152
chrX.fa	103077255	103087212	RAB9B	0.889786716	152
chrX.fa	103139055	103139097	MIR1256	0	0
chrX.fa	103217200	103220563	TMSB15B	2.537704629	76
chrX.fa	103230502	103317502	H2BFXP	0	0
chrX.fa	103265719	103268256	H2BFWT	0	0
chrX.fa	103294516	103297021	H2BFM	0	0
chrX.fa	103343898	103401708	MCART6	0.272206684	76
chrX.fa	103357107	103360533	ZCCHC18	0	0
chrX.fa	103411301	103436127	FAM199X	17.45925662	2356
chrX.fa	103494719	103499599	ESX1	0	0
chrX.fa	103810996	105011822	IL1RAPL2	0	0
chrX.fa	104463611	104465358	TEX13A	0	0
chrX.fa	105066536	105202602	NRK	0.20971479	76
chrX.fa	105277190	105282718	SERPINA7	0	0

chrX.fa	105412298	105452949	MUM1L1	1.568969079	304
chrX.fa	105855160	105922673	CXorf57	0	0
chrX.fa	105937068	106040246	RNF128	0.495487329	76
chrX.fa	106045919	106119377	TBC1D8B	5.634722827	1596
chrX.fa	106143293	106146561	RIPPLY1	0	0
chrX.fa	106161590	106174091	CLDN2	6.003224849	836
chrX.fa	106183964	106243474	MORC4	11.90259306	2052
chrX.fa	106305118	106362057	RBM41	5.024704091	1444
chrX.fa	106366657	106449670	NUP62CL	0	0
chrX.fa	106449862	106487473	CXorf41	0	0
chrX.fa	106871654	106894256	PRPS1	21.16629355	2052
chrX.fa	106956452	107019017	TSC22D3	84.07606024	10612
chrX.fa	107069084	107174867	MID2	8.852721773	2888
chrX.fa	107224094	107225600	TEX13B	0	0
chrX.fa	107288200	107322414	VSIG1	0	0
chrX.fa	107327437	107334848	PSMD10	24.05159544	1672
chrX.fa	107334899	107397901	ATG4A	7.304212674	760
chrX.fa	107398837	107682704	COL4A6	0.247966056	76
chrX.fa	107683154	107940775	COL4A5	0.525510125	152
chrX.fa	107975727	107979607	IRS4	0	0
chrX.fa	108616135	108725285	GUCY2F	0	0
chrX.fa	108779010	108787913	NXT2	1.255620045	152
chrX.fa	108866929	108868393	KCNE1L	0	0
chrX.fa	108884564	108976621	ACSL4	36.39963784	8732
chrX.fa	109245863	109421016	TMEM164	10.35586309	2658
chrX.fa	109437414	109683461	AMMECR1	3.433495904	912
chrX.fa	109468217	109468288	SNORD96B	0	0
chrX.fa	109662285	109699562	RGAG1	0	0
chrX.fa	109763540	109766249	TDGF3	0	0
chrX.fa	109917084	110039286	CHRDL1	0	0
chrX.fa	110187513	110464173	PAK3	0	0
chrX.fa	110488327	110513774	CAPN6	0	0
chrX.fa	110537007	110655460	DCX	0	0
chrX.fa	110754890	110765627	DKFZp686D0853	0	0
chrX.fa	110924346	111003875	ALG13	5.30825272	1672
chrX.fa	111017542	111326004	TRPC5	0	0
chrX.fa	111119279	111147213	LOC100329135	0	0
chrX.fa	111326253	111700473	ZCCHC16	0	0
chrX.fa	111873879	111923375	LHFPL1	0	0
chrX.fa	112018105	112084043	AMOT	16.93374649	5624
chrX.fa	112023946	112024016	MIR4329	0	0
chrX.fa	113818551	114144624	HTR2C	0	0
chrX.fa	113865259	113865386	SNORA35	0	0

chrX.fa	113873918	113874002	MIR764	0	0
chrX.fa	113886019	113886098	MIR1912	0	0
chrX.fa	113887130	113887198	MIR1264	0	0
chrX.fa	113949650	113949761	MIR1298	0	0
chrX.fa	113997744	113997823	MIR1911	0	0
chrX.fa	114058017	114058127	MIR448	0	0
chrX.fa	114238538	114252207	IL13RA2	0	0
chrX.fa	114345183	114468635	LRCH2	0.686298877	152
chrX.fa	114423963	114427431	RBMXL3	0	0
chrX.fa	114524292	114542121	LUZP4	0	0
chrX.fa	114795177	114885179	PLS3	23.97731682	4028
chrX.fa	115301958	115306225	AGTR2	0	0
chrX.fa	115567747	115592625	SLC6A14	0	0
chrX.fa	115592852	115594137	CXorf61	0	0
chrX.fa	117031776	117251303	KLHL13	1.772456918	380
chrX.fa	117480036	117583923	WDR44	16.30660365	3040
chrX.fa	117520357	117520434	MIR1277	0	0
chrX.fa	117629872	117820123	DOCK11	1.782242126	532
chrX.fa	117861559	117928496	IL13RA1	2.95335356	532
chrX.fa	117957787	117960931	ZCCHC12	0	0
chrX.fa	118108713	118151949	LONRF3	0	0
chrX.fa	118212598	118284542	KIAA1210	0	0
chrX.fa	118370211	118378429	PGRMC1	7.939139211	684
chrX.fa	118533258	118588437	SLC25A43	4.650642292	532
chrX.fa	118599996	118603083	LOC100303728	1.181563815	152
chrX.fa	118603624	118605359	SLC25A5	174.2525302	8807
chrX.fa	118672112	118699397	CXorf56	7.641579945	836
chrX.fa	118708499	118718379	UBE2A	18.79226876	2508
chrX.fa	118722300	118739846	NKRF	3.526455376	532
chrX.fa	118749688	118827333	6-Sep	1.519598259	304
chrX.fa	118780701	118780811	MIR766	0	0
chrX.fa	118892576	118894165	ANKRD58	1.063029369	76
chrX.fa	118920469	118925606	RPL39	214.9596609	3876
chrX.fa	118921316	118921447	SNORA69	0	0
chrX.fa	118967989	118986991	UPF3B	1.406178807	152
chrX.fa	119004495	119005733	RNF113A	8.184881356	456
chrX.fa	119005792	119010629	NDUFA1	205.3479184	3952
chrX.fa	119029936	119054679	AKAP14	0	0
chrX.fa	119059013	119077735	NKAP	3.199095704	228
chrX.fa	119206229	119297945	RHOXF2B	0	0
chrX.fa	119243025	119249847	RHOXF1	0	0
chrX.fa	119370309	119379122	NKAPP1	0.698307995	76
chrX.fa	119384607	119392251	ZBTB33	5.410107835	1292

chrX.fa	119392505	119445391	FAM70A	0	0
chrX.fa	119495940	119517104	ATP1B4	0	0
chrX.fa	119560003	119603204	LAMP2	2.087362689	615
chrX.fa	119658446	119709684	CUL4B	8.531366661	2052
chrX.fa	119737744	119755016	MCTS1	1.357252769	608
chrX.fa	119759529	119764005	C1GALT1C1	0.887785197	76
chrX.fa	120006452	120009779	CT47B1	0	0
chrX.fa	120012623	120012778	CT47A1	0	0
chrX.fa	120087137	120090454	CT47A7	0	0
chrX.fa	120094341	120095337	CT47A6	0	0
chrX.fa	120181462	120183796	GLUD2	0.72388297	76
chrX.fa	122318096	122624766	GRIA3	0.319131202	76
chrX.fa	122734412	122866904	THOC2	11.13534383	2809
chrX.fa	122994048	123047822	XIAP	7.433199501	2812
chrX.fa	123094475	123236505	STAG2	18.1653483	5244
chrX.fa	123480132	123507010	SH2D1A	0	0
chrX.fa	123509756	124097666	ODZ1	0.52439817	304
chrX.fa	124453969	124456950	LOC100129520	0	0
chrX.fa	125297482	125300080	DCAF12L2	0	0
chrX.fa	125683366	125686842	DCAF12L1	0.997201609	152
chrX.fa	125953747	125955768	CXorf64	0	0
chrX.fa	127184941	127186382	ACTRT1	0	0
chrX.fa	128580478	128657460	SMARCA1	18.00455955	3308
chrX.fa	128674252	128726530	OCRL	17.05250333	3952
chrX.fa	128779236	128788933	APLN	0.524175779	76
chrX.fa	128872946	128903525	XPNPEP2	1.014103331	152
chrX.fa	128913892	128929176	SASH3	0	0
chrX.fa	128937264	128977910	ZDHHC9	2.195444755	456
chrX.fa	129040097	129063738	UTP14A	5.942067301	684
chrX.fa	129139164	129192058	BCORL1	4.980225875	1596
chrX.fa	129198895	129244688	ELF4	8.308530797	1596
chrX.fa	129263338	129299861	AIFM1	21.44383762	2508
chrX.fa	129305773	129318844	RAB33A	0	0
chrX.fa	129336673	129402922	ZNF280C	1.079931091	228
chrX.fa	129474047	129507335	SLC25A14	1.059693502	76
chrX.fa	129518319	129519511	GPR119	0	0
chrX.fa	129535943	129547317	RBMX2	5.195945223	357
chrX.fa	129628915	129631421	FAM45B	0.674289758	76
chrX.fa	129757357	130037208	ENOX2	10.103894	1900
chrX.fa	130192216	130223857	ARHGAP36	0	0
chrX.fa	130407483	130423403	IGSF1	0	0
chrX.fa	130678048	130678974	OR13H1	0	0
chrX.fa	130836678	130964671	LOC286467	0	0

chrX.fa	131157245	131209971	MST4	2.533256807	380
chrX.fa	131211021	131262050	FRMD7	0.527956427	76
chrX.fa	131337053	131352189	RAP2C	1.718860667	304
chrX.fa	131503343	131623996	MBNL3	1.712633717	912
chrX.fa	131760038	132095423	HS6ST2	0.370058759	76
chrX.fa	132159507	132162300	USP26	0	0
chrX.fa	132350697	132352376	TFDP3	0	0
chrX.fa	132435064	132549205	GPC4	0	0
chrX.fa	132669776	133119673	GPC3	1.417298361	152
chrX.fa	133303408	133303482	MIR363	0	0
chrX.fa	133303568	133303642	MIR92A2	0	0
chrX.fa	133303701	133303796	MIR19B2	0	0
chrX.fa	133303839	133303907	MIR20B	0	0
chrX.fa	133304071	133304141	MIR18B	0	0
chrX.fa	133304228	133304308	MIR106A	0	0
chrX.fa	133371077	133379808	CCDC160	0	0
chrX.fa	133507342	133562822	PHF6	5.335384431	1064
chrX.fa	133594175	133634698	HPRT1	14.06645828	895
chrX.fa	133674215	133674292	MIR450B	0	0
chrX.fa	133674371	133674461	MIR450A1	0	0
chrX.fa	133674538	133674637	MIR450A2	0	0
chrX.fa	133675371	133675467	MIR542	0	0
chrX.fa	133677407	133680643	MGC16121	0	0
chrX.fa	133680358	133680428	MIR503	0	0
chrX.fa	133680661	133680741	MIR424	0	0
chrX.fa	133699873	133792513	PLAC1	0	0
chrX.fa	133903596	133931262	FAM122B	7.431420373	1216
chrX.fa	133930443	133988641	FAM122C	1.254730481	304
chrX.fa	134021662	134049297	MOSPD1	7.167886941	760
chrX.fa	134124968	134126503	LOC644538	2.200782141	152
chrX.fa	134154534	134156566	FAM127C	7.48234793	684
chrX.fa	134166333	134167575	FAM127A	67.98762225	3800
chrX.fa	134184963	134186221	FAM127B	22.82199515	1292
chrX.fa	134229015	134232733	NCRNA00087	9.630645776	1216
chrX.fa	134252882	134254405	LOC100129515	0	0
chrX.fa	134290461	134305751	CXorf48	0	0
chrX.fa	134419723	134429965	ZNF75D	7.755444178	1140
chrX.fa	134478696	134497338	ZNF449	0.416316104	76
chrX.fa	134555868	134560225	NCRNA00086	2.934227927	456
chrX.fa	134654555	134716460	DDX26B	3.074556699	532
chrX.fa	134847185	134856988	CT45A1	0	0
chrX.fa	134873998	134874268	CT45A2	0	0
chrX.fa	134883488	134890044	CT45A3	0	0

chrX.fa	134928697	134936735	CT45A4	0	0
chrX.fa	134945651	134953994	CT45A5	0	0
chrX.fa	134963215	134971244	CT45A6	0	0
chrX.fa	134975785	134995220	SAGE1	0	0
chrX.fa	135044231	135056134	MMGT1	2.316647894	380
chrX.fa	135067583	135129428	SLC9A6	1.42930748	304
chrX.fa	135228861	135293518	FHL1	0	0
chrX.fa	135295379	135338641	MAP7D3	0	0
chrX.fa	135383122	135499047	GPR112	0	0
chrX.fa	135570125	135574598	BRS3	0	0
chrX.fa	135579238	135594503	HTATSF1	25.85919015	3643
chrX.fa	135614311	135638966	VGLL1	36.16835112	1976
chrX.fa	135633037	135633119	MIR934	0	0
chrX.fa	135730336	135742549	CD40LG	0	0
chrX.fa	135747712	135863503	ARHGEF6	0.64159827	152
chrX.fa	135951353	135962939	RBMX	20.98348808	3192
chrX.fa	135961358	135961430	SNORD61	0	0
chrX.fa	136112307	136113833	GPR101	0	0
chrX.fa	136648346	136654259	ZIC3	0	0
chrX.fa	137696892	137699799	LOC158696	0	0
chrX.fa	137713734	138287185	FGF13	4.896162046	760
chrX.fa	137749872	137749954	MIR504	0	0
chrX.fa	138528978	138531132	SRD5A1P1	0	0
chrX.fa	138612895	138645617	F9	0	0
chrX.fa	138663930	138790381	MCF2	0	0
chrX.fa	138808505	138914447	ATP11C	1.657480729	456
chrX.fa	139006307	139006390	MIR505	0	0
chrX.fa	139037884	139047677	CXorf66	0	0
chrX.fa	139585152	139587225	SOX3	0	0
chrX.fa	139791924	139796996	RP1-177G6-2	0	0
chrX.fa	139865425	139866723	CDR1	44.23847867	2584
chrX.fa	140008337	140008384	MIR320D2	0	0
chrX.fa	140269931	140271310	LDOC1	25.71997333	1596
chrX.fa	140335596	140336646	SPANXC	0	0
chrX.fa	140590843	140738069	CXorf18	0	0
chrX.fa	140671796	140678899	SPANXA2	0	0
chrX.fa	140786599	140786655	SPANXE	0	0
chrX.fa	140926102	140985618	MAGEC3	0	0
chrX.fa	140991642	140997187	MAGEC1	0	0
chrX.fa	141290128	141293076	MAGEC2	0	0
chrX.fa	142113704	142122066	SPANXN4	0	0
chrX.fa	142596564	142605307	SPANXN3	0	0
chrX.fa	142710595	142723926	SLITRK4	0	0

chrX.fa	142795055	142804516	SPANXN2	0	0
chrX.fa	142967173	142968357	UBE2NL	0	0
chrX.fa	144329107	144337728	SPANXN1	0	0
chrX.fa	144899347	144907360	SLITRK2	0	0
chrX.fa	144908928	144911370	CXorf1	0	0
chrX.fa	145075793	145075869	MIR890	0	0
chrX.fa	145076302	145076378	MIR888	0	0
chrX.fa	145078187	145078261	MIR892A	0	0
chrX.fa	145078716	145078792	MIR892B	0	0
chrX.fa	145082571	145082649	MIR891B	0	0
chrX.fa	145109312	145109390	MIR891A	0	0
chrX.fa	145891302	145896249	CXorf51	0	0
chrX.fa	146312238	146312361	MIR506	0	0
chrX.fa	146312502	146312595	MIR507	0	0
chrX.fa	146318431	146318545	MIR508	0	0
chrX.fa	146331669	146331748	MIR514B	0	0
chrX.fa	146341170	146342133	MIR509-3	0	0
chrX.fa	146342050	146342052	MIR509-1	0	0
chrX.fa	146353853	146353926	MIR510	0	0
chrX.fa	146360765	146360862	MIR514-1	0	0
chrX.fa	146990949	147003676	FMR1-AS1	0	0
chrX.fa	147007058	147032647	FMR1	14.03710266	2574
chrX.fa	147062849	147108187	FMR1NB	0	0
chrX.fa	147582139	148082193	AFF2	0	0
chrX.fa	148560295	148586884	IDS	24.64537963	6688
chrX.fa	148609134	148621312	LOC100131434	0	0
chrX.fa	148622519	148632086	CXorf40A	5.090976633	760
chrX.fa	148674172	148674182	HSFX2	0	0
chrX.fa	148678216	148713487	TMEM185A	0.618691988	76
chrX.fa	148769903	148798928	MAGEA11	0	0
chrX.fa	148863593	148863599	MAGEA9B	0	0
chrX.fa	149009941	149014609	MAGEA8	0	0
chrX.fa	149100415	149106716	CXorf40B	13.87453478	836
chrX.fa	149106766	149185018	LOC100272228	0.531069902	76
chrX.fa	149396239	149396318	MIR2114	0	0
chrX.fa	149531551	149682448	MAMLD1	0	0
chrX.fa	149737047	149841616	MTM1	5.90270408	912
chrX.fa	149861869	149933575	MTMR1	8.661243052	1064
chrX.fa	149934809	150067289	CD99L2	4.094664589	684
chrX.fa	150151763	150159248	HMGB3	9.50610677	1520
chrX.fa	150336694	150336798	MIR4330	0	0
chrX.fa	150345056	150349937	GPR50	0	0
chrX.fa	150565657	150577836	VMA21	4.282585053	912

chrX.fa	150732007	150845211	PASD1	0	0
chrX.fa	150863730	150870063	PRRG3	0	0
chrX.fa	150884508	150891664	FATE1	0	0
chrX.fa	150903218	150914036	CNGA2	0	0
chrX.fa	151081361	151093642	MAGEA4	0	0
chrX.fa	151121596	151143151	GABRE	0.536184897	76
chrX.fa	151128100	151128184	MIR452	0	0
chrX.fa	151282527	151286444	MAGEA5	0	0
chrX.fa	151302907	151307025	MAGEA10	0	0
chrX.fa	151335634	151619831	GABRA3	0	0
chrX.fa	151560691	151560771	MIR105-1	0	0
chrX.fa	151561893	151562001	MIR767	0	0
chrX.fa	151562884	151562964	MIR105-2	0	0
chrX.fa	151806637	151821825	GABRQ	0	0
chrX.fa	151867245	151870814	MAGEA6	0	0
chrX.fa	151883119	151922364	MAGEA2	0	0
chrX.fa	151885384	151885450	MAGEA2B	0	0
chrX.fa	151899293	151903184	MAGEA12	0	0
chrX.fa	151903228	151909518	CSAG1	0	0
chrX.fa	151928150	151928200	CSAG3	0	0
chrX.fa	151934652	151938240	MAGEA3	0	0
chrX.fa	151995871	151999301	CETN2	48.5141696	2356
chrX.fa	151999511	152037907	NSDHL	10.36920656	760
chrX.fa	152082986	152142025	ZNF185	10.94986967	2356
chrX.fa	152157368	152162671	PNMA5	0	0
chrX.fa	152224766	152228827	PNMA3	0	0
chrX.fa	152240819	152240838	LOC100287428	0	0
chrX.fa	152244152	152245958	PNMA6A	0	0
chrX.fa	152481522	152486116	MAGEA1	0	0
chrX.fa	152599613	152618384	ZNF275	3.209103303	912
chrX.fa	152683781	152687086	ZFP92	0	0
chrX.fa	152710178	152711945	TREX2	0	0
chrX.fa	152713123	152736603	HAUS7	2.700050118	228
chrX.fa	152760347	152775004	BGN	1.383050135	152
chrX.fa	152801580	152848387	ATP2B3	0	0
chrX.fa	152853383	152864632	FAM58A	9.634426424	532
chrX.fa	152907897	152916781	DUSP9	4.97110784	532
chrX.fa	152935188	152939816	PNCK	0	0
chrX.fa	152953752	152962048	SLC6A8	9.713597649	1596
chrX.fa	152965947	152990201	BCAP31	13.4028433	1444
chrX.fa	152990323	153010216	ABCD1	1.383939699	228
chrX.fa	153029651	153044801	PLXNB3	0	0
chrX.fa	153046456	153051187	SRPK3	0	0

chrX.fa	153051221	153059967	IDH3G	13.41885545	1033
chrX.fa	153060094	153063954	SSR4	100.8265565	2888
chrX.fa	153067623	153096003	PDZD4	0	0
chrX.fa	153126971	153141399	L1CAM	1.008321163	228
chrX.fa	153167985	153172620	AVPR2	0	0
chrX.fa	153172830	153191714	ARHGAP4	0.277099287	42
chrX.fa	153195377	153200468	NAA10	25.99596067	1031
chrX.fa	153200722	153210232	RENBP	11.49005761	760
chrX.fa	153213008	153236819	HCFC1	15.64610213	5928
chrX.fa	153237991	153248646	TMEM187	4.055523759	304
chrX.fa	153246548	153246628	MIR3202-1	0	0
chrX.fa	153275957	153285342	IRAK1	40.70423961	6536
chrX.fa	153285371	153285440	MIR718	0	0
chrX.fa	153287264	153363188	MECP2	11.05772934	5092
chrX.fa	153409725	153424507	OPN1LW	0	0
chrX.fa	153461638	153462352	OPN1MW	0	0
chrX.fa	153500056	153523438	TEX28	0	0
chrX.fa	153524027	153558713	TKTL1	0	0
chrX.fa	153576900	153603006	FLNA	0.595785707	228
chrX.fa	153607597	153609883	EMD	34.08121082	2052
chrX.fa	153626571	153629578	RPL10	17.64873382	965
chrX.fa	153628622	153628756	SNORA70	0	0
chrX.fa	153630681	153640422	DNASE1L1	2.081580521	228
chrX.fa	153639877	153650063	TAZ	0	0
chrX.fa	153656978	153664862	ATP6AP1	10.4679482	988
chrX.fa	153665259	153671814	GDI1	55.63802311	6207
chrX.fa	153672485	153679002	FAM50A	32.74464042	1973
chrX.fa	153686623	153701985	PLXNA3	0.250634749	76
chrX.fa	153705241	153707596	LAGE3	9.307066752	760
chrX.fa	153712056	153714932	UBL4A	16.86502765	1748
chrX.fa	153715650	153719002	SLC10A3	3.17552225	304
chrX.fa	153733327	153744566	FAM3A	7.57686414	1048
chrX.fa	153759606	153775233	G6PD	16.59348814	1787
chrX.fa	153770459	153793261	IKBKG	3.891399141	456
chrX.fa	153880246	153881853	CTAG2	1.702181336	76
chrX.fa	153903527	153979348	GAB3	0	0
chrX.fa	153991031	154005964	DKC1	17.70833463	2052
chrX.fa	153996803	153996932	SNORA36A	0	0
chrX.fa	154003273	154003401	SNORA56	0	0
chrX.fa	154006959	154033802	MPP1	16.92485085	1672
chrX.fa	154051623	154062937	LOC100132963	0	0
chrX.fa	154064064	154250998	F8	0.550862708	228
chrX.fa	154114635	154688276	F8A1	0	0

chrX.fa	154255064	154285191	FUNDC2	25.09038418	3344
chrX.fa	154289900	154292308	MTCP1NB	20.50089944	401
chrX.fa	154292309	154294332	MTCP1	0	0
chrX.fa	154299710	154351349	BRCC3	9.280379823	1216
chrX.fa	154444701	154468098	VBP1	13.49646994	988
chrX.fa	154487526	154493852	RAB39B	0.485257339	76
chrX.fa	154505500	154563986	CLIC2	0	0
chrX.fa	154718673	154842622	TMLHE	2.048221859	380
chrX.fa	154997451	155012117	SPRY3	0	0
chrX.fa	155110943	155173433	VAMP7	0	0
chrX.fa	155227246	155240482	IL9R	0	0
chrY.fa	142991	170022	PLCXD1	0	0
chrY.fa	171426	180887	GTPBP6	0	0
chrY.fa	231385	232054	NCRNA00107	0	0
chrY.fa	244668	297690	PPP2R3B	0	0
chrY.fa	535079	570146	SHOX	0	0
chrY.fa	1264887	1281527	CRLF2	0	0
chrY.fa	1337693	1378828	CSF2RA	0	0
chrY.fa	1362811	1362885	MIR3690	0	0
chrY.fa	1405509	1451582	IL3RA	0	0
chrY.fa	1455045	1461039	SLC25A6	0	0
chrY.fa	1472032	1522655	ASMTL	0	0
chrY.fa	1482655	1484314	ASMTL-AS	0	0
chrY.fa	1531466	1606037	P2RY8	0	0
chrY.fa	1660486	1671411	AKAP17A	0	0
chrY.fa	1683941	1711974	ASMT	0	0
chrY.fa	2087555	2293345	DHRSX	0	0
chrY.fa	2354455	2368580	ZBED1	0	0
chrY.fa	2477306	2525270	CD99P1	0	0
chrY.fa	2559228	2609350	CD99	0	0
chrY.fa	2620337	2643037	XGPY2	0	0
chrY.fa	2654896	2655792	SRY	0	0
chrY.fa	2709623	2734997	RPS4Y1	390.0397084	15732
chrY.fa	2803112	2850547	ZFY	8.727737986	2204
chrY.fa	3447126	3448082	TGIF2LY	0	0
chrY.fa	4868267	5610264	PCDH11Y	0	0
chrY.fa	6114264	6117053	TSPY2	0	0
chrY.fa	6258442	9611928	TTTY1B	0	0
chrY.fa	6325200	9545180	TTTY7	0	0
chrY.fa	6733959	6742068	AMELY	0	0
chrY.fa	6778727	6959724	TBL1Y	4.213199035	456
chrY.fa	7142013	7249588	PRKY	0.936711234	304
chrY.fa	7567398	7569288	TTTY16	0	0

chrY.fa	7672965	7678723	TTTY12	0	0
chrY.fa	8551411	8551919	TTTY18	0	0
chrY.fa	8572513	8573324	TTTY19	0	0
chrY.fa	8651359	8685423	TTTY11	0	0
chrY.fa	9154670	9160483	RBMY1A3P	0	0
chrY.fa	9167489	9172441	TTTY20	0	0
chrY.fa	9187189	9362877	FAM197Y2P	0	0
chrY.fa	9195452	9236312	TSPY4	0	0
chrY.fa	9236030	9365753	TSPY3	0	0
chrY.fa	9304564	9307358	TSPY1	0	0
chrY.fa	9448330	9452762	RBMY3AP	0	0
chrY.fa	9638762	9650854	TTTY22	0	0
chrY.fa	14517915	14533389	GYG2P1	2.051112943	76
chrY.fa	14774298	14804153	TTTY15	0	0
chrY.fa	14813160	14972768	USP9Y	8.080579939	3648
chrY.fa	15016019	15032390	DDX3Y	14.06690307	3033
chrY.fa	15360263	15592550	UTY	8.624548524	2736
chrY.fa	15815447	15817902	TMSB4Y	10.13903179	760
chrY.fa	16168098	16168101	VCY	0	0
chrY.fa	16634488	16955848	NLGN4Y	0.807946798	228
chrY.fa	19687041	20492736	NCRNA00230B	0	0
chrY.fa	19691333	20488439	NCRNA00230A	0	0
chrY.fa	19990140	19992099	CDY2B	0	0
chrY.fa	20137667	20139626	CDY2A	0	0
chrY.fa	20708557	20935621	HSFY2	0	0
chrY.fa	20933700	20934513	HSFY1	0	0
chrY.fa	21034387	21040114	NCRNA00185	0	0
chrY.fa	21094585	21239302	TTTY14	0	0
chrY.fa	21152526	21154705	CD24	106.2173163	10412
chrY.fa	21617317	21665039	BCORP1	0.46079432	76
chrY.fa	21729235	21752309	CYorf15A	9.769862593	380
chrY.fa	21758442	21767698	CYorf15B	0.509942749	76
chrY.fa	21867301	21906825	KDM5D	9.388239497	2356
chrY.fa	22627554	22681114	TTTY10	0	0
chrY.fa	22737611	22755040	EIF1AY	7.295761813	456
chrY.fa	22917954	22942918	RPS4Y2	0	0
chrY.fa	23557034	23563448	RBMY2EP	0	0
chrY.fa	23673224	24064214	RBMY1B	0	0
chrY.fa	23696805	23696912	RBMY1D	0	0
chrY.fa	23745486	23756552	TTTY13	0	0
chrY.fa	24291112	24587606	TTTY6	0	0
chrY.fa	24442945	24445023	TTTY5	0	0
chrY.fa	24455006	24462352	RBMY2FP	0	0

chrY.fa	24549623	24549729	RBMY1J	0	0
chrY.fa	24585737	24585739	TTTY6B	0	0
chrY.fa	25275502	25345239	DAZ1	0	0
chrY.fa	25365622	27037136	DAZ2	0	0
chrY.fa	25382975	26980276	DAZ3	0	0
chrY.fa	26986878	26988731	DAZ4	0	0
chrY.fa	59100457	59115123	SPRY3	0	0
chrY.fa	59213949	59276439	VAMP7	0	0
chrY.fa	59330252	59343488	IL9R	0	0
