# Supplementary material for: Massively Parallel Sequencing of Human Urinary Exosome/Microvesicle RNA Reveals a Predominance of Non-Coding RNA
Source: PLoS One. 2014 May 9;9(5):e96094. doi: 10.1371/journal.pone.0096094 (PMC4015934; doi:10.1371/journal.pone.0096094)
Supplement: Table S6 — Genes found within microvesicles (including exosomes) (-DNase). Raw and normalized counts (as per Casava's 1.7 read counts method) of genes identified in microvesicles. (ZIP) [file pone.0096094.s006.zip › PLOS Russo Supp Table S6 Final 040814.pdf]

Supp. Table S6: Genes found within microvesicles (including exosomes) (-DNase)
Raw and normalized counts (as per Casava's 1.7 read counts method) of genes identified in microvesicles

chr	start	end	hgnc	normal	raw
chr1.fa	14362	29370	WASH7P	0	0
chr1.fa	69091	70008	OR4F5	0	0
chr1.fa	661139	665731	LOC100133331	0.267928736	76
chr1.fa	700245	714068	LOC100288069	0	0
chr1.fa	761586	762902	NCRNA00115	0	0
chr1.fa	763064	789740	LOC643837	4.450870116	456
chr1.fa	803451	812182	FAM41C	0	0
chr1.fa	852953	854817	FLJ39609	0	0
chr1.fa	861121	879582	SAMD11	9.472688988	1368
chr1.fa	879962	894679	NOC2L	11.34683468	1824
chr1.fa	895967	901099	KLHL17	0	0
chr1.fa	901877	910484	PLEKHN1	3.758984442	608
chr1.fa	910579	917473	C1orf170	1.506063721	304
chr1.fa	934342	935552	HES4	8.804749727	608
chr1.fa	948847	949919	ISG15	25.77944332	1140
chr1.fa	955503	991492	AGRN	4.691689705	2280
chr1.fa	1017198	1051736	C1orf159	1.087980432	152
chr1.fa	1102484	1102578	MIR200B	0	0
chr1.fa	1103243	1103332	MIR200A	0	0
chr1.fa	1104385	1104467	MIR429	0	0
chr1.fa	1109286	1133313	TTLL10	0	0
chr1.fa	1138888	1142089	TNFRSF18	0	0
chr1.fa	1146706	1149548	TNFRSF4	0	0
chr1.fa	1152288	1167447	SDF4	11.32153281	1520
chr1.fa	1167629	1170420	B3GALT6	2.869653815	532
chr1.fa	1177833	1182102	FAM132A	0	0
chr1.fa	1189292	1209234	UBE2J2	10.25885425	1660
chr1.fa	1210638	1227409	SCNN1D	0.330580987	76
chr1.fa	1227764	1243269	ACAP3	4.230984812	1056
chr1.fa	1243994	1246964	PUSL1	5.98750693	456
chr1.fa	1247058	1260046	CPSF3L	17.67154928	2396
chr1.fa	1260143	1264276	GLTPD1	10.96067994	1596
chr1.fa	1266726	1269844	TAS1R3	0	0
chr1.fa	1270658	1284492	DVL1	10.17782802	1976
chr1.fa	1288071	1293915	MXRA8	1.00710481	152
chr1.fa	1309110	1310818	AURKAIP1	38.91864444	2964
chr1.fa	1321091	1334718	CCNL2	5.674245676	1368
chr1.fa	1334910	1337275	LOC148413	7.167206643	760

chr1.fa	1337427	1342693	MRPL20	98.3340631	3800
chr1.fa	1353800	1356650	LOC441869	5.104802983	684
chr1.fa	1361508	1363167	TMEM88B	0	0
chr1.fa	1370903	1378262	VWA1	2.211052149	684
chr1.fa	1385069	1405538	ATAD3C	2.076259446	532
chr1.fa	1407164	1431582	ATAD3B	0.940838007	152
chr1.fa	1447523	1470067	ATAD3A	3.669976076	684
chr1.fa	1470158	1475740	C1orf70	0	0
chr1.fa	1477053	1510262	SSU72	87.84402807	7600
chr1.fa	1550795	1565990	MIB2	1.921586702	456
chr1.fa	1567560	1568499	MMP23B	0	0
chr1.fa	1568159	1633247	MMP23A	0	0
chr1.fa	1571100	1654073	CDK11B	6.340076447	807
chr1.fa	1592939	1624243	SLC35E2B	10.29635524	4104
chr1.fa	1634170	1655791	CDK11A	2.440726867	311
chr1.fa	1656277	1677438	SLC35E2	1.411784132	684
chr1.fa	1682671	1711508	NADK	17.53766022	4864
chr1.fa	1716729	1822495	GNB1	168.276566	34950
chr1.fa	1846266	1848733	CALML6	0	0
chr1.fa	1849029	1850740	TMEM52	1.19476035	76
chr1.fa	1884752	1935276	KIAA1751	0.486006763	152
chr1.fa	1950768	1962192	GABRD	0	0
chr1.fa	1981909	2106752	PRKCZ	15.07434239	2201
chr1.fa	2115899	2139172	C1orf86	8.574623191	1092
chr1.fa	2120482	2125024	LOC100128003	0.531188674	152
chr1.fa	2160134	2241652	SKI	17.85001783	6764
chr1.fa	2252696	2322993	MORN1	2.789983044	304
chr1.fa	2281853	2284100	LOC100129534	0	0
chr1.fa	2323214	2336240	RER1	17.22259168	2812
chr1.fa	2336886	2344010	PEX10	20.0661906	1824
chr1.fa	2407754	2436964	PLCH2	2.839683146	912
chr1.fa	2439975	2458035	PANK4	5.19878136	912
chr1.fa	2460184	2461684	HES5	0.876378479	76
chr1.fa	2481359	2484284	LOC115110	0.51808592	76
chr1.fa	2487805	2495267	TNFRSF14	11.40587238	1292
chr1.fa	2517899	2522908	C1orf93	15.36380784	2584
chr1.fa	2522996	2564481	MMEL1	0	0
chr1.fa	2938046	2939467	ACTRT2	0	0
chr1.fa	2976181	2984289	FLJ42875	0.297447585	76
chr1.fa	2985742	3355185	PRDM16	5.248632069	3040
chr1.fa	3044539	3044599	MIR4251	0	0
chr1.fa	3371147	3397677	ARHGEF16	9.459586233	1824
chr1.fa	3404506	3528059	MEGF6	0.307688818	152

chr1.fa	3477260	3477354	MIR551A	0	0
chr1.fa	3541556	3546694	TPRG1L	41.50214615	6688
chr1.fa	3547331	3566671	WDR8	7.410586541	836
chr1.fa	3569129	3650467	TP73	0.367027729	76
chr1.fa	3652548	3663335	KIAA0495	3.960646374	988
chr1.fa	3668965	3688209	CCDC27	0	0
chr1.fa	3689352	3692546	LOC388588	0	0
chr1.fa	3696784	3713068	LRRC47	21.61276743	3800
chr1.fa	3728645	3773797	KIAA0562	12.20619464	5234
chr1.fa	3773845	3801993	DFFB	1.89010997	380
chr1.fa	3805697	3816857	C1orf174	8.931560293	988
chr1.fa	3816968	3832011	LOC100133612	0	0
chr1.fa	4472111	4484744	LOC284661	0	0
chr1.fa	4715105	4843851	AJAP1	0.345039199	76
chr1.fa	5922870	6052357	NPHP4	1.896586044	608
chr1.fa	6052532	6161253	KCNAB2	1.434826907	532
chr1.fa	6161847	6240194	CHD5	0.236903823	152
chr1.fa	6245080	6259679	RPL22	159.7510404	22116
chr1.fa	6266189	6281252	RNF207	0.592485468	152
chr1.fa	6281360	6296044	ICMT	8.995417394	2812
chr1.fa	6304252	6305638	HES3	0	0
chr1.fa	6307406	6321035	GPR153	1.401994718	380
chr1.fa	6324332	6453826	ACOT7	4.79108991	760
chr1.fa	6475294	6479979	HES2	0.806045304	228
chr1.fa	6484848	6521004	ESPN	1.522630422	357
chr1.fa	6489894	6489956	MIR4252	0	0
chr1.fa	6521214	6526151	TNFRSF25	0.632697369	76
chr1.fa	6526256	6580069	PLEKHG5	0.619745221	228
chr1.fa	6581407	6614658	NOL9	7.673997085	3420
chr1.fa	6615338	6639817	TAS1R1	0	0
chr1.fa	6640063	6649340	ZBTB48	5.874853364	912
chr1.fa	6650784	6662929	KLHL21	10.20373232	3040
chr1.fa	6673756	6684093	PHF13	5.925758318	1444
chr1.fa	6684925	6694227	THAP3	2.191623927	226
chr1.fa	6695647	6761966	DNAJC11	17.60016186	2190
chr1.fa	6845384	7829766	CAMTA1	3.005199549	1743
chr1.fa	7831329	7841492	VAMP3	51.6569314	7563
chr1.fa	7844763	7905237	PER3	7.381067692	3040
chr1.fa	7907672	7913551	UTS2	0	0
chr1.fa	7975931	8003225	TNFRSF9	0	0
chr1.fa	8021714	8045342	PARK7	237.7104711	15168
chr1.fa	8071779	8086393	ERRFI1	16.82709935	3496
chr1.fa	8384390	8404227	SLC45A1	0	0

chr1.fa	8412464	8877699	RERE	72.9665282	40314
chr1.fa	8921063	8938780	ENO1	198.1404541	23839
chr1.fa	9005922	9035148	CA6	0	0
chr1.fa	9063359	9086404	SLC2A7	0	0
chr1.fa	9097005	9148510	SLC2A5	2.846309827	532
chr1.fa	9164476	9189229	GPR157	0.992797205	76
chr1.fa	9211727	9211836	MIR34A	0	0
chr1.fa	9294863	9331394	H6PD	3.516056364	2128
chr1.fa	9352941	9429590	SPSB1	2.570549559	532
chr1.fa	9599528	9642831	SLC25A33	7.834392872	760
chr1.fa	9648932	9674935	TMEM201	0.297146372	76
chr1.fa	9711790	9789078	PIK3CD	0.645800124	228
chr1.fa	9712668	9714644	C1orf200	0	0
chr1.fa	9789173	9884550	CLSTN1	6.062057085	2052
chr1.fa	9908334	9970316	CTNNBIP1	36.30637692	7220
chr1.fa	9989776	10002826	LZIC	30.91331334	2888
chr1.fa	10003486	10045556	NMNAT1	4.540932726	1140
chr1.fa	10057255	10076078	RBP7	12.23240015	532
chr1.fa	10093041	10241296	UBE4B	28.0359786	10944
chr1.fa	10270764	10441661	KIF1B	7.218111597	7676
chr1.fa	10459085	10480201	PGD	55.54634096	7144
chr1.fa	10502322	10502872	APITD1	13.42068443	491
chr1.fa	10509776	10510379	CORT	0	0
chr1.fa	10520603	10532613	DFFA	9.445579841	2128
chr1.fa	10535003	10690815	PEX14	8.116779819	1038
chr1.fa	10696666	10856707	CASZ1	25.38621009	14363
chr1.fa	11006530	11042094	C1orf127	0	0
chr1.fa	11072679	11085549	TARDBP	21.17134016	5928
chr1.fa	11086580	11107285	MASP2	0	0
chr1.fa	11114649	11120091	SRM	14.38622188	1216
chr1.fa	11126676	11159938	EXOSC10	19.57295473	3648
chr1.fa	11166588	11322608	MTOR	14.56182891	8436
chr1.fa	11249398	11256038	ANGPTL7	0	0
chr1.fa	11333255	11348491	UBIAD1	2.192678172	532
chr1.fa	11539295	11597640	PTCHD2	0.439017575	152
chr1.fa	11708448	11714739	FBXO2	2.58380292	228
chr1.fa	11714914	11723384	FBXO44	2.413316507	532
chr1.fa	11724150	11734409	FBXO6	3.694675521	380
chr1.fa	11734537	11751678	MAD2L2	6.895211535	608
chr1.fa	11751781	11780336	C1orf187	0	0
chr1.fa	11796142	11810828	AGTRAP	12.4250257	1056
chr1.fa	11845787	11866160	MTHFR	9.925261136	4712
chr1.fa	11866207	11903201	CLCN6	3.663349396	1368

chr1.fa	11905767	11907840	NPPA	0	0
chr1.fa	11917521	11918992	NPPB	0	0
chr1.fa	11980123	11986480	KIAA2013	2.934866374	456
chr1.fa	11994746	12035594	PLOD1	3.048272972	608
chr1.fa	12040238	12073572	MFN2	37.44390684	11628
chr1.fa	12079512	12092106	MIIP	2.162406291	228
chr1.fa	12123434	12204264	TNFRSF8	0	0
chr1.fa	12227060	12269277	TNFRSF1B	0.311453978	76
chr1.fa	12290113	12572098	VPS13D	13.95006582	15124
chr1.fa	12627939	12677820	DHRS3	19.01345206	2280
chr1.fa	12704566	12727097	AADACL4	0.726675746	76
chr1.fa	12776118	12788726	AADACL3	0	0
chr1.fa	12806163	12821102	C1orf158	0	0
chr1.fa	12834984	12838048	PRAMEF12	0	0
chr1.fa	12851546	12856777	PRAMEF1	0	0
chr1.fa	12884468	12891264	PRAMEF11	0	0
chr1.fa	12907236	12907260	LOC649330	0	0
chr1.fa	12908238	12908578	HNRNPCL1	0	0
chr1.fa	12916941	12921764	PRAMEF2	0	0
chr1.fa	12939033	12946025	PRAMEF4	0	0
chr1.fa	12952727	12958094	PRAMEF10	0	0
chr1.fa	12976450	13611550	PRAMEF8	0	0
chr1.fa	12998302	13007406	PRAMEF6	0	0
chr1.fa	13035543	13038381	PRAMEF22	0	0
chr1.fa	13108514	13369057	PRAMEF5	0	0
chr1.fa	13182960	13184326	LOC440563	0	0
chr1.fa	13328196	13331692	PRAMEF3	0	0
chr1.fa	13449560	13449703	PRAMEF13	0	0
chr1.fa	13495254	13498259	PRAMEF16	0	0
chr1.fa	13516066	13737001	PRAMEF20	0	0
chr1.fa	13521963	13742807	PRAMEF21	0	0
chr1.fa	13668269	13673511	PRAMEF14	0	0
chr1.fa	13716088	13719064	PRAMEF17	0	0
chr1.fa	13801445	13840242	LRRC38	0	0
chr1.fa	13910252	13944452	PDPN	0	0
chr1.fa	14026735	14151574	PRDM2	24.96330739	15884
chr1.fa	14925213	15444544	KAZ	4.836874247	1900
chr1.fa	15438311	15478960	C1orf126	0.324556732	76
chr1.fa	15479028	15546974	TMEM51	2.557145592	380
chr1.fa	15573768	15724622	FHAD1	0.449710627	152
chr1.fa	15736391	15756839	EFHD2	24.13196022	3876
chr1.fa	15764938	15773153	CTRC	0	0
chr1.fa	15783223	15798586	CELA2A	0	0

chr1.fa	15802596	15817895	CELA2B	0	0
chr1.fa	15818769	15851285	CASP9	5.30586249	836
chr1.fa	15853352	15898228	DNAJC16	10.63868352	4256
chr1.fa	15899152	15911605	AGMAT	30.75216452	4484
chr1.fa	15943953	15986363	DDI2	44.52541846	4408
chr1.fa	15987553	15988217	RSC1A1	53.35772916	2356
chr1.fa	16010827	16061264	PLEKHM2	11.66265624	3192
chr1.fa	16062809	16067884	SLC25A34	2.236956445	456
chr1.fa	16068917	16074477	TMEM82	3.012127443	304
chr1.fa	16085255	16113084	FBLIM1	10.25328181	2280
chr1.fa	16133657	16134194	UQCRHL	2.127465613	76
chr1.fa	16160710	16163401	FLJ37453	0.850323577	152
chr1.fa	16174359	16266950	SPEN	32.44211862	26252
chr1.fa	16268364	16302627	ZBTB17	5.825002655	1064
chr1.fa	16330731	16333184	C1orf64	1.242803783	76
chr1.fa	16340523	16345285	HSPB7	0.395793546	76
chr1.fa	16348486	16360545	CLCNKA	0.902734595	152
chr1.fa	16370247	16383803	CLCNKB	3.132763147	608
chr1.fa	16384264	16400127	FAM131C	0	0
chr1.fa	16450832	16482582	EPHA2	5.197576509	1368
chr1.fa	16524599	16539104	ARHGEF19	0.755742775	152
chr1.fa	16558182	16563659	C1orf89	5.869732747	608
chr1.fa	16576559	16678948	FBXO42	14.60008293	2885
chr1.fa	16693583	16724640	C1orf144	41.54311108	10024
chr1.fa	16725138	16763919	SPATA21	0	0
chr1.fa	16767167	16786584	NECAP2	26.40129703	3648
chr1.fa	16793931	16819196	CROCCP3	0.213258623	76
chr1.fa	16875409	17185516	MIR3675	0	0
chr1.fa	16890412	16939982	NBPF1	5.275891822	1520
chr1.fa	16944751	16957401	CROCCP2	3.242555192	380
chr1.fa	16972069	16976915	MST1P2	0.868396342	152
chr1.fa	17017713	17046652	ESPNP	0	0
chr1.fa	17081401	17090975	MST1P9	0.781195252	228
chr1.fa	17248445	17299474	CROCC	1.375789209	608
chr1.fa	17300999	17308081	MFAP2	0	0
chr1.fa	17312453	17338423	ATP13A2	2.291475952	608
chr1.fa	17345225	17380665	SDHB	49.98294157	3800
chr1.fa	17393256	17445948	PADI2	0.2623563	76
chr1.fa	17531621	17572501	PADI1	0.595045776	152
chr1.fa	17575593	17610727	PADI3	30.77324941	6514
chr1.fa	17634690	17690495	PADI4	0	0
chr1.fa	17698741	17728195	PADI6	0	0
chr1.fa	17733251	17766220	RCC2	30.75562847	8436

chr1.fa	17866330	18024370	ARHGEF10L	17.48027919	5216
chr1.fa	18081808	18153558	ACTL8	0	0
chr1.fa	18434240	18704977	IGSF21	2.943902756	380
chr1.fa	18807424	18812480	KLHDC7A	18.33361489	6156
chr1.fa	18957500	19075360	PAX7	0	0
chr1.fa	19166093	19186155	TAS1R2	0	0
chr1.fa	19197924	19229293	ALDH4A1	37.43456925	6383
chr1.fa	19230774	19282826	IFFO2	7.650351885	2964
chr1.fa	19401000	19536746	UBR4	12.90049002	13604
chr1.fa	19544584	19578046	KIAA0090	2.160599015	608
chr1.fa	19578075	19586622	MRTO4	6.546407177	988
chr1.fa	19592476	19600568	AKR7L	0	0
chr1.fa	19609057	19615280	AKR7A3	14.75716537	1216
chr1.fa	19630459	19638640	AKR7A2	41.17894487	3724
chr1.fa	19638740	19655794	PQLC2	1.214489785	152
chr1.fa	19665267	19812066	CAPZB	198.6946855	22560
chr1.fa	19923471	19956314	C1orf151	30.25335621	8013
chr1.fa	19969726	19984945	NBL1	80.07545018	11628
chr1.fa	19991780	20006055	HTR6	0	0
chr1.fa	20008706	20126410	TMCO4	18.0738189	3569
chr1.fa	20140522	20141771	RNF186	1.831373485	152
chr1.fa	20208888	20239437	OTUD3	4.920008965	2128
chr1.fa	20246800	20250110	PLA2G2E	0	0
chr1.fa	20301924	20306932	PLA2G2A	36.59087235	2432
chr1.fa	20396701	20418394	PLA2G5	1.812999508	228
chr1.fa	20438434	20446008	PLA2G2D	0	0
chr1.fa	20465823	20476879	PLA2G2F	1.26283443	228
chr1.fa	20490484	20501687	PLA2G2C	0	0
chr1.fa	20512578	20519942	UBXN10	5.391858729	1064
chr1.fa	20617412	20681387	VWA5B1	1.534678932	456
chr1.fa	20686294	20755287	LOC339505	0	0
chr1.fa	20808884	20812728	CAMK2N1	39.43567612	6156
chr1.fa	20825941	20834674	MUL1	2.328374513	380
chr1.fa	20878932	20881513	FAM43B	0	0
chr1.fa	20915444	20945400	CDA	0	0
chr1.fa	20959948	20978004	PINK1	29.69099202	5244
chr1.fa	20978260	20988037	DDOST	18.84356807	2660
chr1.fa	20990507	21044317	KIF17	0	0
chr1.fa	21046225	21059133	SH2D5	0	0
chr1.fa	21069171	21113181	HP1BP3	48.75022841	12692
chr1.fa	21132785	21503381	EIF4G3	24.15063541	11172
chr1.fa	21543740	21672034	ECE1	2.647660022	988
chr1.fa	21766631	21811393	NBPF3	2.923420289	731

chr1.fa	21835858	21904905	ALPL	8.377328843	1444
chr1.fa	21922708	21995856	RAP1GAP	42.88260416	10239
chr1.fa	22004792	22109688	USP48	22.16714949	7636
chr1.fa	22138758	22151714	LDLRAD2	0	0
chr1.fa	22154356	22263750	HSPG2	1.812698295	1520
chr1.fa	22303418	22315847	CELA3B	0	0
chr1.fa	22328149	22339035	CELA3A	0	0
chr1.fa	22351707	22357715	HSPC157	5.737349746	456
chr1.fa	22379120	22419436	CDC42	74.00947733	15504
chr1.fa	22443798	22469519	WNT4	0.29308	76
chr1.fa	22778344	22857650	ZBTB40	17.05707528	10184
chr1.fa	22890004	22930087	EPHA8	0	0
chr1.fa	22963118	22966175	C1QA	0	0
chr1.fa	22970118	22974603	C1QC	0	0
chr1.fa	22979682	22988029	C1QB	0	0
chr1.fa	23037331	23241823	EPHB2	0.236753217	76
chr1.fa	23189686	23189719	MIR4253	0	0
chr1.fa	23345941	23410184	KDM1A	39.36022232	8120
chr1.fa	23370798	23370865	MIR3115	0	0
chr1.fa	23410516	23495351	LUZP1	13.89117873	7828
chr1.fa	23518388	23521222	HTR1D	0	0
chr1.fa	23636276	23670853	HNRNPR	40.92366707	7448
chr1.fa	23685941	23694879	ZNF436	15.38519394	4408
chr1.fa	23695464	23698330	C1orf213	0	0
chr1.fa	23707555	23751261	TCEA3	96.76414227	10190
chr1.fa	23755056	23810750	ASAP3	21.2669752	5863
chr1.fa	23832920	23857712	E2F2	0	0
chr1.fa	23884409	23886322	ID3	67.53897576	5776
chr1.fa	23953824	23967056	MDS2	0	0
chr1.fa	24018269	24022915	RPL11	2115.9007	89072
chr1.fa	24069856	24088549	TCEB3	29.40830386	9652
chr1.fa	24104876	24114722	C1orf128	52.06221314	5607
chr1.fa	24117646	24122029	LYPLA2	18.66811164	2013
chr1.fa	24122089	24127294	GALE	11.70482603	1444
chr1.fa	24128367	24151949	HMGCL	54.81017701	5852
chr1.fa	24171572	24194859	FUCA1	3.244061256	456
chr1.fa	24200460	24239817	CNR2	0	0
chr1.fa	24286301	24289949	PNRC2	51.68283569	8284
chr1.fa	24292937	24306953	SRSF10	9.615313222	3182
chr1.fa	24382531	24438665	MYOM3	2.960168244	1140
chr1.fa	24446261	24469611	IL22RA1	0	0
chr1.fa	24480647	24513751	IL28RA	4.025858933	1216
chr1.fa	24526730	24538180	LOC284632	0	0

chr1.fa	24645812	24690970	GRHL3	177.0966775	40239
chr1.fa	24683489	24741587	C1orf201	7.512998874	1520
chr1.fa	24742245	24799473	NIPAL3	39.61113254	14271
chr1.fa	24829387	24862427	RCAN3	145.5181358	16416
chr1.fa	24882567	24935818	C1orf130	1.434375088	380
chr1.fa	24969594	24999772	SRRM1	50.91670108	13300
chr1.fa	25071760	25170815	CLIC4	18.32819306	5396
chr1.fa	25226002	25291501	RUNX3	0	0
chr1.fa	25548767	25559013	SYF2	27.69726487	3268
chr1.fa	25568740	25573985	C1orf63	7.813006767	760
chr1.fa	25598981	25656936	RHD	0	0
chr1.fa	25664811	25688739	TMEM50A	7.394772872	1064
chr1.fa	25688853	25747363	RHCE	0	0
chr1.fa	25757388	25826698	TMEM57	8.719657127	2280
chr1.fa	25870076	25895377	LDLRAP1	15.59950681	3040
chr1.fa	25943959	26111258	MAN1C1	1.072769189	228
chr1.fa	26126667	26144713	SEPN1	12.6827132	3648
chr1.fa	26146397	26159433	FAM54B	44.11381125	5328
chr1.fa	26146521	26150097	LOC646471	0.745953361	152
chr1.fa	26160497	26185848	C1orf135	1.666158295	228
chr1.fa	26187975	26197744	PAQR7	21.58204373	4332
chr1.fa	26210677	26233368	STMN1	4.450870116	1045
chr1.fa	26232853	26232879	MIR3917	0	0
chr1.fa	26286258	26324648	PAFAH2	14.70309769	3496
chr1.fa	26348271	26362954	EXTL1	0	0
chr1.fa	26364514	26372604	SLC30A2	7.868279305	1292
chr1.fa	26377798	26394121	TRIM63	0	0
chr1.fa	26437656	26452026	PDIK1L	7.984999244	2432
chr1.fa	26485511	26489119	GRRP1	0	0
chr1.fa	26496388	26497364	ZNF593	7.054553077	304
chr1.fa	26503981	26516375	CNKSR1	7.221575544	1216
chr1.fa	26517119	26529033	CATSPER4	0	0
chr1.fa	26560693	26605299	CCDC21	2.036198151	532
chr1.fa	26606213	26608013	SH3BGRL3	58.5135877	4332
chr1.fa	26608773	26644756	UBXN11	3.355058152	456
chr1.fa	26644411	26647014	CD52	0	0
chr1.fa	26648350	26680621	AIM1L	11.70106087	4075
chr1.fa	26688125	26699266	ZNF683	0	0
chr1.fa	26737269	26756219	LIN28A	0	0
chr1.fa	26758802	26797795	DHDDS	19.42957746	4256
chr1.fa	26798902	26803133	HMGN2	26.35807301	3446
chr1.fa	26856249	26901520	RPS6KA1	21.99214488	4940
chr1.fa	26881033	26881084	MIR1976	0	0

chr1.fa	27022522	27108601	ARID1A	47.73092449	27208
chr1.fa	27114486	27124887	PIGV	3.387890341	532
chr1.fa	27153201	27182211	ZDHHC18	5.10495359	1064
chr1.fa	27189633	27190947	SFN	65.28183806	5700
chr1.fa	27205873	27216869	GPN2	9.854174928	988
chr1.fa	27216979	27226962	GPATCH3	4.838681524	684
chr1.fa	27237975	27240567	NR0B2	0.900626105	76
chr1.fa	27248224	27272887	NUDC	68.97018812	6077
chr1.fa	27276047	27286901	C1orf172	10.15734556	1216
chr1.fa	27320195	27327377	TRNP1	1.761793341	228
chr1.fa	27331511	27339333	FAM46B	0	0
chr1.fa	27425300	27481451	SLC9A1	8.011204753	2432
chr1.fa	27561007	27635110	WDTC1	20.7374432	6612
chr1.fa	27648636	27662891	TMEM222	7.92415427	976
chr1.fa	27650365	27653016	LOC644961	0	0
chr1.fa	27668483	27680423	SYTL1	303.5492515	39625
chr1.fa	27681670	27693337	MAP3K6	2.380183105	684
chr1.fa	27695601	27701315	FCN3	0	0
chr1.fa	27705596	27709805	CD164L2	0	0
chr1.fa	27719152	27722317	GPR3	0	0
chr1.fa	27732126	27816669	WASF2	44.22962755	12540
chr1.fa	27860756	27930143	AHDC1	2.466781769	1064
chr1.fa	27938801	27961727	FGR	0	0
chr1.fa	27992572	27998724	IFI6	0	0
chr1.fa	28052490	28089423	FAM76A	2.978241009	684
chr1.fa	28099694	28150963	STX12	32.6354972	6672
chr1.fa	28157252	28178183	PPP1R8	17.98616599	3406
chr1.fa	28160912	28161077	SCARNA1	0	0
chr1.fa	28199055	28213193	C1orf38	0	0
chr1.fa	28218049	28241236	RPA2	17.96764141	2052
chr1.fa	28261504	28285663	SMPDL3B	0.611160658	76
chr1.fa	28286504	28294604	XKR8	0.530435643	76
chr1.fa	28300819	28415131	EYA3	19.4351499	2639
chr1.fa	28421446	28423069	LOC653566	0	0
chr1.fa	28473677	28520437	PTAFR	0.253018705	76
chr1.fa	28526790	28559542	DNAJC8	32.74242773	3835
chr1.fa	28562611	28564611	ATPIF1	110.0950653	4408
chr1.fa	28586006	28609002	SESN2	6.240375029	1444
chr1.fa	28655513	28662478	MED18	7.348988534	912
chr1.fa	28696093	28826881	PHACTR4	35.21568557	15180
chr1.fa	28832732	28832839	SNHG3	0	0
chr1.fa	28843237	28865708	RCC1	21.8531352	4028
chr1.fa	28879529	28905049	TRNAU1AP	8.554592543	1140

chr1.fa	28905058	28908366	SNHG12	1.565854451	76
chr1.fa	28905255	28905334	SNORD99	0	0
chr1.fa	28906276	28906405	SNORA61	0	0
chr1.fa	28906893	28907024	SNORA44	0	0
chr1.fa	28907432	28907565	SNORA16A	0	0
chr1.fa	28918712	28921088	RAB42	0	0
chr1.fa	28929609	28969604	TAF12	10.26201698	988
chr1.fa	28975112	28975246	RNU11	0	0
chr1.fa	28995244	29041387	GMEB1	5.250439345	684
chr1.fa	29063133	29096287	YTHDF2	25.21903701	5129
chr1.fa	29138654	29190208	OPRD1	0	0
chr1.fa	29213603	29445936	EPB41	64.86526084	24149
chr1.fa	29446559	29450421	TMEM200B	0	0
chr1.fa	29474250	29508637	SRSF4	23.9778899	4028
chr1.fa	29519385	29557454	MECR	8.934873633	1444
chr1.fa	29563028	29653325	PTPRU	2.029420864	760
chr1.fa	31184124	31196432	MATN1	0	0
chr1.fa	31191805	31199593	LOC100129196	0	0
chr1.fa	31205315	31230683	LAPTM5	0	0
chr1.fa	31342313	31381480	SDC3	1.117499281	380
chr1.fa	31404353	31538564	PUM1	52.07591832	18620
chr1.fa	31441010	31441084	SNORD85	0	0
chr1.fa	31460912	31461481	PRO0611	0	0
chr1.fa	31652592	31712734	NKAIN1	0.783454348	152
chr1.fa	31732415	31769644	SNRNP40	30.19552337	3256
chr1.fa	31769842	31837780	ZCCHC17	27.15056374	2888
chr1.fa	31838100	31845923	FABP3	3.130202838	228
chr1.fa	31882412	31907527	SERINC2	12.57804177	2964
chr1.fa	31971839	31974167	LOC149086	0	0
chr1.fa	31984036	31989846	LOC284551	0	0
chr1.fa	32042086	32053287	TINAGL1	6.651681031	988
chr1.fa	32083301	32092919	HCRTR1	0	0
chr1.fa	32095463	32110838	PEF1	31.49344908	4636
chr1.fa	32117848	32169768	COL16A1	0	0
chr1.fa	32192718	32229648	BAI2	0.211451346	76
chr1.fa	32224261	32224336	MIR4254	0	0
chr1.fa	32256025	32281580	SPOCD1	0	0
chr1.fa	32372022	32403988	PTP4A2	97.88450308	25536
chr1.fa	32479491	32509472	KHDRBS1	44.3349014	7904
chr1.fa	32538503	32568467	TMEM39B	1.259219877	152
chr1.fa	32573644	32642168	KPNA6	29.96599926	14668
chr1.fa	32645345	32663886	TXLNA	31.98035948	10184
chr1.fa	32666202	32670991	CCDC28B	1.305154821	76

chr1.fa	32671236	32674288	IQCC	0.55754479	76
chr1.fa	32674695	32681797	DCDC2B	0	0
chr1.fa	32681798	32687926	C1orf91	1.057859158	76
chr1.fa	32687971	32697205	EIF3I	106.7672669	10336
chr1.fa	32697261	32707311	MTMR9LP	0	0
chr1.fa	32712818	32714461	FAM167B	0	0
chr1.fa	32716840	32751766	LCK	0	0
chr1.fa	32757708	32799224	HDAC1	68.972146	9576
chr1.fa	32799440	32801834	MARCKSL1	94.76710178	9728
chr1.fa	32826871	32827844	LOC100128071	0	0
chr1.fa	32827862	32829924	TSSK3	0	0
chr1.fa	32830705	32860062	BSDC1	36.37113766	6902
chr1.fa	32930658	32953459	ZBTB8B	1.139487812	304
chr1.fa	33004772	33071542	ZBTB8A	2.653533671	1292
chr1.fa	33087307	33116185	ZBTB8OS	6.656199223	316
chr1.fa	33116749	33151812	RBBP4	21.49318597	8360
chr1.fa	33160466	33168361	SYNC	0	0
chr1.fa	33207512	33240571	KIAA1522	352.7886494	131318
chr1.fa	33240840	33283633	YARS	23.63947738	4712
chr1.fa	33290914	33324476	S100PBP	4.41126064	1292
chr1.fa	33327869	33338082	FNDC5	0	0
chr1.fa	33352098	33360195	HPCA	0.845353567	76
chr1.fa	33360248	33366953	TMEM54	59.60232116	3724
chr1.fa	33402050	33430286	RNF19B	21.10688063	3648
chr1.fa	33473541	33502512	AK2	15.07373997	5776
chr1.fa	33546714	33585995	ADC	0.524561994	76
chr1.fa	33611003	33647671	TRIM62	9.237893654	2356
chr1.fa	33722174	33766320	ZNF362	12.89808032	2660
chr1.fa	33789224	33841194	PHC2	17.59669791	4864
chr1.fa	33798006	33798093	MIR3605	0	0
chr1.fa	33938232	33961995	ZSCAN20	2.44223293	684
chr1.fa	33979609	34631443	CSMD2	0	0
chr1.fa	34326076	34330392	HMGB4	0	0
chr1.fa	34632624	34684731	C1orf94	0	0
chr1.fa	35220721	35224112	GJB5	0	0
chr1.fa	35225342	35229325	GJB4	0	0
chr1.fa	35246790	35251965	GJB3	0	0
chr1.fa	35258599	35261348	GJA4	0	0
chr1.fa	35315963	35325417	C1orf212	5.388093569	2280
chr1.fa	35331037	35370984	DLGAP3	0	0
chr1.fa	35447127	35450948	LOC100130633	0	0
chr1.fa	35451767	35497569	ZMYM6	8.338321793	2848
chr1.fa	35544972	35581455	ZMYM1	5.692017228	1576

chr1.fa	35649201	35658743	SFPQ	57.98812207	11832
chr1.fa	35734568	35887545	ZMYM4	25.11692589	11664
chr1.fa	35899091	36023037	KIAA0319L	14.5097191	4636
chr1.fa	36023393	36032380	NCDN	7.335132748	1672
chr1.fa	36038971	36060927	TFAP2E	0	0
chr1.fa	36065143	36107445	PSMB2	16.76414589	5393
chr1.fa	36179477	36184790	C1orf216	3.12101585	608
chr1.fa	36197713	36235551	CLSPN	0	0
chr1.fa	36273828	36321188	EIF2C4	10.34424806	3268
chr1.fa	36348810	36389899	EIF2C1	25.10246768	12464
chr1.fa	36396772	36522063	EIF2C3	7.081361011	1672
chr1.fa	36549676	36553876	TEKT2	0.75845369	76
chr1.fa	36554453	36559533	ADPRHL2	15.56833129	1748
chr1.fa	36560844	36565850	COL8A2	0	0
chr1.fa	36602173	36615067	TRAPPC3	57.13975637	4788
chr1.fa	36621803	36646441	MAP7D1	20.31649839	4484
chr1.fa	36690017	36770957	THRAP3	64.57956055	19000
chr1.fa	36771994	36786948	C1orf113	1.331510936	228
chr1.fa	36787632	36789755	FAM176B	0	0
chr1.fa	36805225	36851485	STK40	19.01962692	4857
chr1.fa	36859031	36863493	LSM10	22.89216856	1292
chr1.fa	36883507	36916086	OSCP1	2.749771142	304
chr1.fa	36921362	36930040	MRPS15	55.76336474	3573
chr1.fa	36931644	36948915	CSF3R	0	0
chr1.fa	37261128	37499844	GRIK3	0	0
chr1.fa	37627164	37627235	MIR4255	0	0
chr1.fa	37940119	37949978	ZC3H12A	9.692875504	1748
chr1.fa	37958176	37980364	MEAF6	79.64531838	11354
chr1.fa	38002142	38019903	SNIP1	9.727514969	1596
chr1.fa	38022520	38032412	DNALI1	16.26985577	2812
chr1.fa	38032459	38061586	GNL2	19.91889756	3116
chr1.fa	38076951	38100491	RSPO1	0.383443823	76
chr1.fa	38147242	38156192	C1orf109	7.290553262	1140
chr1.fa	38158159	38175391	CDCA8	0.497001028	76
chr1.fa	38181646	38230824	EPHA10	0.344587379	152
chr1.fa	38259774	38267278	MANEAL	0.368383186	76
chr1.fa	38268614	38273472	YRDC	6.345648883	608
chr1.fa	38273866	38275126	C1orf122	46.2468493	2432
chr1.fa	38275239	38325292	MTF1	8.614684486	4560
chr1.fa	38326369	38412729	INPP5B	3.340750547	988
chr1.fa	38422652	38455761	SF3A3	19.63831789	3698
chr1.fa	38462442	38471234	FHL3	2.015113259	228
chr1.fa	38478384	38490497	UTP11L	7.258474105	988

chr1.fa	38509523	38512450	POU3F1	0	0
chr1.fa	38554903	38555001	MIR3659	0	0
chr1.fa	39305015	39325340	RRAGC	6.394445348	608
chr1.fa	39338963	39339050	MYCBP	36.2824305	212
chr1.fa	39339267	39339581	GJA9-MYCBP	0	0
chr1.fa	39339739	39340449	GJA9	1.609831512	76
chr1.fa	39351479	39407456	RHBDL2	1.869326291	228
chr1.fa	39456916	39471737	AKIRIN1	31.91138176	5776
chr1.fa	39491967	39500308	NDUFS5	192.4384968	7296
chr1.fa	39547118	39952789	MACF1	10.77573532	17247
chr1.fa	39875176	39882154	KIAA0754	7.872345678	3648
chr1.fa	39957318	39995541	BMP8A	0.210547708	76
chr1.fa	39987952	40025370	PPIEL	0.233891696	38
chr1.fa	40026485	40042521	PABPC4	68.23357235	14516
chr1.fa	40033046	40033182	SNORA55	0	0
chr1.fa	40089103	40105348	HEYL	1.112830484	304
chr1.fa	40124793	40137710	NT5C1A	0	0
chr1.fa	40144645	40157089	HPCAL4	0.537514142	152
chr1.fa	40204517	40229586	PPIE	9.935652976	3067
chr1.fa	40223903	40254533	BMP8B	0.309947914	76
chr1.fa	40235197	40237020	OXCT2	0	0
chr1.fa	40306706	40349177	TRIT1	16.67423388	2356
chr1.fa	40361096	40367687	MYCL1	53.89915906	16416
chr1.fa	40420784	40435628	MFSD2A	0.520043803	76
chr1.fa	40506255	40538321	CAP1	90.39635425	16644
chr1.fa	40538382	40563142	PPT1	7.770836983	1292
chr1.fa	40627041	40706593	RLF	14.46785053	6004
chr1.fa	40713573	40717365	TMCO2	0	0
chr1.fa	40723733	40759856	ZMPSTE24	4.354933856	912
chr1.fa	40766163	40782939	COL9A2	5.857232418	1101
chr1.fa	40839378	40888998	SMAP2	9.488653263	2052
chr1.fa	40916337	40929390	ZNF643	1.127138089	152
chr1.fa	40943302	40962015	ZNF642	1.06026886	152
chr1.fa	40974433	40982214	DEM1	4.074504792	596
chr1.fa	40997233	41013841	ZNF684	1.637543084	228
chr1.fa	41086352	41131324	RIMS3	1.58332479	760
chr1.fa	41154752	41157933	LOC100130557	0	0
chr1.fa	41161278	41237275	NFYC	14.25263403	2205
chr1.fa	41220027	41220118	MIR30E	0	0
chr1.fa	41222956	41223044	MIR30C1	0	0
chr1.fa	41249684	41306124	KCNQ4	0.279224214	76
chr1.fa	41326728	41328018	CITED4	8.866046521	760
chr1.fa	41445007	41478235	CTPS	13.9542828	2964

chr1.fa	41481269	41487427	SLFNL1	0	0
chr1.fa	41492871	41707815	SCMH1	30.03407334	7580
chr1.fa	41944446	41950344	EDN2	0	0
chr1.fa	41975684	42384496	HIVEP3	1.032105468	608
chr1.fa	42619092	42621495	GUCA2B	0	0
chr1.fa	42628362	42630395	GUCA2A	0	0
chr1.fa	42642210	42801548	FOXJ3	17.48404435	6688
chr1.fa	42846468	42889900	RIMKLA	1.191296404	836
chr1.fa	42896001	42921938	ZMYND12	5.80692989	684
chr1.fa	42922173	42926086	PPCS	54.48667452	3777
chr1.fa	43000560	43120335	CCDC30	1.108462899	228
chr1.fa	43124048	43142429	PPIH	8.565737415	455
chr1.fa	43148066	43168020	YBX1	204.9844595	21042
chr1.fa	43198764	43205925	CLDN19	0.400311737	76
chr1.fa	43212006	43232755	LEPRE1	1.163584831	209
chr1.fa	43232916	43241415	C1orf50	5.627407095	380
chr1.fa	43272723	43283059	CCDC23	6.237664114	304
chr1.fa	43282776	43310660	ERMAP	7.201544896	1643
chr1.fa	43312280	43318146	ZNF691	6.540684135	684
chr1.fa	43391046	43424719	SLC2A1	5.816719304	1368
chr1.fa	43424848	43449029	FLJ32224	0	0
chr1.fa	43613594	43622067	FAM183A	2.21391367	76
chr1.fa	43629845	43638241	EBNA1BP2	24.05334369	2204
chr1.fa	43638015	43720029	WDR65	0.559502672	152
chr1.fa	43735665	43739673	TMEM125	31.40308526	3724
chr1.fa	43747557	43751288	C1orf210	9.078250899	912
chr1.fa	43766664	43788779	TIE1	0	0
chr1.fa	43803475	43820135	MPL	0.628028572	152
chr1.fa	43824626	43828873	CDC20	0.683752929	76
chr1.fa	43829072	43833699	ELOVL1	17.72139999	1745
chr1.fa	43849588	43855483	MED8	17.10255841	2052
chr1.fa	43855556	43872565	C1orf84	7.707732913	608
chr1.fa	43888797	43918304	KIAA0467	4.764733795	3040
chr1.fa	43917099	43919660	HYI	9.943936326	412
chr1.fa	43996547	44089343	PTPRF	20.42629043	10488
chr1.fa	44115797	44171189	KDM4A	36.39975287	10569
chr1.fa	44165409	44173012	LOC100132774	0	0
chr1.fa	44173218	44396831	ST3GAL3	2.761669046	441
chr1.fa	44398992	44402912	ARTN	0	0
chr1.fa	44412478	44433694	IPO13	11.02905524	2949
chr1.fa	44435653	44439043	DPH2	5.500747136	912
chr1.fa	44440602	44443972	ATP6V0B	10.07978327	684
chr1.fa	44444866	44456840	B4GALT2	9.402355812	1444

chr1.fa	44457280	44462154	CCDC24	4.038811081	380
chr1.fa	44462199	44497134	SLC6A9	0.317628839	76
chr1.fa	44584522	44600809	KLF17	0	0
chr1.fa	44679125	44686351	DMAP1	14.90716932	1748
chr1.fa	44686742	44820939	ERI3	25.61091479	2964
chr1.fa	44870960	45117396	RNF220	14.57026287	2912
chr1.fa	45119501	45140099	TMEM53	1.430760535	152
chr1.fa	45140394	45191263	C1orf228	0.679234738	76
chr1.fa	45205490	45233438	KIF2C	0	0
chr1.fa	45241246	45244412	RPS8	1771.301874	82916
chr1.fa	45241537	45241610	SNORD55	0	0
chr1.fa	45242164	45242261	SNORD46	0	0
chr1.fa	45243514	45243584	SNORD38A	0	0
chr1.fa	45244062	45244130	SNORD38B	0	0
chr1.fa	45249257	45253426	BEST4	0	0
chr1.fa	45266036	45271585	PLK3	1.508774636	228
chr1.fa	45271668	45272957	TCTEX1D4	0	0
chr1.fa	45274154	45279801	BTBD19	1.456213012	152
chr1.fa	45285516	45308616	PTCH2	0	0
chr1.fa	45316194	45452361	EIF2B3	14.89271111	2128
chr1.fa	45468220	45477027	HECTD3	17.03659282	4104
chr1.fa	45477805	45481341	UROD	19.56437016	1894
chr1.fa	45482076	45672250	ZSWIM5	2.941944873	1140
chr1.fa	45769582	45771291	LOC400752	0	0
chr1.fa	45792545	45794346	HPDL	1.270364749	152
chr1.fa	45794914	45806142	MUTYH	2.322651471	283
chr1.fa	45805342	45809554	TOE1	7.174586355	958
chr1.fa	45809651	45956840	TESK2	34.95287745	6916
chr1.fa	45959598	45965751	CCDC163P	1.055449456	152
chr1.fa	45965856	45976706	MMACHC	0.812671984	152
chr1.fa	45976740	45987609	PRDX1	304.0217037	24244
chr1.fa	46016498	46035721	AKR1A1	107.3769215	10944
chr1.fa	46049660	46084578	NASP	8.694957682	1900
chr1.fa	46085716	46089731	CCDC17	0	0
chr1.fa	46092976	46152302	GPBP1L1	52.97413473	13148
chr1.fa	46111452	46112357	RPS15AP10	0	0
chr1.fa	46153847	46159997	TMEM69	24.40711806	2204
chr1.fa	46160109	46216485	IPP	13.74072297	3040
chr1.fa	46269285	46501796	MAST2	4.189116241	1596
chr1.fa	46505812	46598708	PIK3R3	21.62556897	8512
chr1.fa	46640749	46651634	TSPAN1	21.07931966	2280
chr1.fa	46654353	46664121	POMGNT1	15.02765442	2730
chr1.fa	46669006	46686928	C1orf190	3.668620619	304

chr1.fa	46713367	46744071	RAD54L	0	0
chr1.fa	46744146	46769038	LRRC41	34.64895379	6612
chr1.fa	46769380	46782447	UQCRH	170.0764627	5940
chr1.fa	46806390	46830690	NSUN4	11.61114887	3420
chr1.fa	46859939	46879520	FAAH	5.269415748	733
chr1.fa	46972668	46979886	DMBX1	0	0
chr1.fa	47011316	47016887	KNCN	0	0
chr1.fa	47023090	47069966	MKNK1	4.736269191	912
chr1.fa	47073387	47082563	MOBKL2C	8.052470899	1596
chr1.fa	47098411	47134099	ATPAF1	16.88809493	4630
chr1.fa	47137500	47139251	C1orf223	0	0
chr1.fa	47140831	47184736	KIAA0494	42.47370785	16264
chr1.fa	47264670	47285021	CYP4B1	8.486367857	1216
chr1.fa	47323906	47366147	CYP4Z2P	0	0
chr1.fa	47394846	47407156	CYP4A11	6.279231473	1064
chr1.fa	47489240	47516423	CYP4X1	1.456815438	228
chr1.fa	47533160	47583992	CYP4Z1	0	0
chr1.fa	47603107	47614526	CYP4A22	0	0
chr1.fa	47649261	47655771	PDZK1IP1	116.5093907	6916
chr1.fa	47681963	47695443	TAL1	0	0
chr1.fa	47715811	47779819	STIL	0.456337308	152
chr1.fa	47799469	47844511	CMPK1	81.2554511	15808
chr1.fa	47881744	47883724	FOXE3	0	0
chr1.fa	47897807	47900313	MGC12982	0	0
chr1.fa	47901689	47906363	FOXD2	0	0
chr1.fa	48226200	48462562	LOC388630	0.162805488	76
chr1.fa	48567387	48648100	SKINTL	0	0
chr1.fa	48688357	48714316	SLC5A9	0.34955739	76
chr1.fa	48761044	48937876	SPATA6	2.443889601	812
chr1.fa	48998527	50489626	AGBL4	0.383142611	76
chr1.fa	49193540	49242547	BEND5	0.861920268	76
chr1.fa	50513686	50667540	ELAVL4	0	0
chr1.fa	50883227	50889141	DMRTA2	0	0
chr1.fa	50906935	51425936	FAF1	40.66311805	6974
chr1.fa	51434367	51440306	CDKN2C	5.5495436	912
chr1.fa	51567906	51613754	C1orf185	0	0
chr1.fa	51701945	51739119	RNF11	42.07565521	8588
chr1.fa	51752930	51810785	TTC39A	34.30707733	6524
chr1.fa	51819935	51984995	EPS15	51.55527209	17869
chr1.fa	52082546	52254865	OSBPL9	38.03232594	10687
chr1.fa	52254892	52344609	NRD1	36.31044329	9557
chr1.fa	52302016	52302040	MIR761	0	0
chr1.fa	52373628	52456436	RAB3B	25.04162271	21356

chr1.fa	52485804	52521047	TXNDC12	7.158320867	760
chr1.fa	52497777	52499472	KTI12	3.374486374	380
chr1.fa	52521857	52554090	BTF3L4	13.66692585	2128
chr1.fa	52608046	52812358	ZFYVE9	39.12392093	12768
chr1.fa	52816265	52831877	CC2D1B	8.09765281	3040
chr1.fa	52838501	52870143	ORC1	0.36115408	76
chr1.fa	52870219	52883992	PRPF38A	16.83734058	3099
chr1.fa	52888948	53018762	ZCCHC11	7.826410734	3040
chr1.fa	53068043	53074723	GPX7	1.86269961	152
chr1.fa	53099066	53122737	FAM159A	0	0
chr1.fa	53152014	53164038	C1orf163	8.663029131	1216
chr1.fa	53192131	53293013	ZYG11B	18.00905816	9736
chr1.fa	53308183	53360247	ZYG11A	2.947969128	836
chr1.fa	53361582	53387446	ECHDC2	38.19151688	4032
chr1.fa	53392901	53517289	SCP2	96.96203905	23821
chr1.fa	53527724	53551174	PODN	0	0
chr1.fa	53552855	53608289	SLC1A7	0	0
chr1.fa	53662101	53679771	CPT2	15.28187797	3040
chr1.fa	53679870	53686289	C1orf123	18.11553687	1186
chr1.fa	53692564	53704207	MAGOH	14.90581386	674
chr1.fa	53711212	53793726	LRP8	0	0
chr1.fa	53904043	53905693	FLJ40434	0	0
chr1.fa	53925072	53933158	DMRTB1	0	0
chr1.fa	53971906	54199877	GLIS1	0.406486598	76
chr1.fa	54231134	54304225	TMEM48	2.134995931	684
chr1.fa	54317392	54355487	YIPF1	20.42644104	2581
chr1.fa	54359861	54376759	DIO1	7.548391371	836
chr1.fa	54387234	54411288	HSPB11	22.42799972	895
chr1.fa	54412037	54433839	LRRC42	16.02722891	1824
chr1.fa	54474506	54483803	LDLRAD1	4.374362078	228
chr1.fa	54497349	54519111	TMEM59	24.29597056	2736
chr1.fa	54519274	54565416	C1orf83	4.469997125	1216
chr1.fa	54604668	54619443	CDCP2	0	0
chr1.fa	54638027	54665746	CYB5RL	3.88775289	912
chr1.fa	54665840	54684056	MRPL37	49.39648036	4864
chr1.fa	54692195	54871979	SSBP3	20.41243465	2811
chr1.fa	55013807	55100417	ACOT11	14.38381218	2508
chr1.fa	55076085	55089200	FAM151A	5.647738955	456
chr1.fa	55117517	55181283	C1orf175-TTC4	0	0
chr1.fa	55175619	55175939	C1orf175	0	0
chr1.fa	55181495	55181692	TTC4	0	0
chr1.fa	55222571	55230226	PARS2	0.95936259	152
chr1.fa	55246752	55266941	TTC22	4.097246354	836

chr1.fa	55271736	55307937	C1orf177	0	0
chr1.fa	55315300	55352921	DHCR24	22.43281913	6384
chr1.fa	55446465	55457966	TMEM61	0	0
chr1.fa	55464617	55474465	BSND	0.835413546	76
chr1.fa	55505149	55530526	PCSK9	0	0
chr1.fa	55532032	55681039	USP24	17.79052831	12760
chr1.fa	56960419	57045257	PPAP2B	6.228175913	1368
chr1.fa	57110990	57181008	PRKAA2	2.692691327	1672
chr1.fa	57184477	57285369	C1orf168	2.646455171	608
chr1.fa	57320443	57383894	C8A	0	0
chr1.fa	57394883	57431688	C8B	0	0
chr1.fa	57463579	58716211	DAB1	4.880098276	836
chr1.fa	58946391	59012446	OMA1	38.37344937	4864
chr1.fa	59041095	59043166	TACSTD2	29.27817995	4028
chr1.fa	59125590	59165747	MYSM1	17.16069247	2950
chr1.fa	59246463	59249785	JUN	21.35598357	4712
chr1.fa	59250823	59365384	LOC100131060	2.038457247	152
chr1.fa	59597608	59612479	LOC729467	0	0
chr1.fa	59762625	60228402	FGGY	32.92255295	4560
chr1.fa	60280533	60342050	HOOK1	25.22536248	9810
chr1.fa	60358980	60392423	CYP2J2	1.232713156	152
chr1.fa	60456066	60539442	C1orf87	0	0
chr1.fa	61542946	61928460	NFIA	16.90134829	11248
chr1.fa	62146719	62191095	TM2D1	2.747060228	228
chr1.fa	62208149	62629591	INADL	78.31561472	44221
chr1.fa	62660474	62678001	L1TD1	0	0
chr1.fa	62701837	62785083	KANK4	0	0
chr1.fa	62901975	62917475	USP1	8.72492835	2432
chr1.fa	62920397	63153969	DOCK7	15.90629199	7448
chr1.fa	63063187	63071180	ANGPTL3	4.84545881	684
chr1.fa	63249803	63330050	ATG4C	3.167101399	380
chr1.fa	63788730	63790797	FOXD3	0	0
chr1.fa	63833261	63904233	ALG6	1.358168264	304
chr1.fa	63906462	63988835	ITGB3BP	3.563798584	208
chr1.fa	63989013	64038364	EFCAB7	2.908811471	456
chr1.fa	64014651	64016307	DLEU2L	0	0
chr1.fa	64058947	64125916	PGM1	21.50613812	4788
chr1.fa	64239690	64644707	ROR1	0	0
chr1.fa	64669490	64710027	UBE2U	0	0
chr1.fa	64936476	65158741	CACHD1	0.651071347	228
chr1.fa	65210778	65298905	RAVER2	7.364199778	2128
chr1.fa	65298915	65432187	JAK1	31.31558296	10488
chr1.fa	65523438	65523525	MIR3671	0	0

chr1.fa	65524117	65524191	MIR101-1	0	0
chr1.fa	65613232	65697828	AK4	22.49306168	10792
chr1.fa	65730430	65881552	DNAJC6	3.185023558	1216
chr1.fa	65886131	65901690	LEPROT	6.104678688	2019
chr1.fa	65991372	66103176	LEPR	0.340370401	152
chr1.fa	66258193	66840262	PDE4B	7.026239079	2432
chr1.fa	66999825	67210768	SGIP1	0	0
chr1.fa	67094123	67094200	MIR3117	0	0
chr1.fa	67218140	67244730	TCTEX1D1	0	0
chr1.fa	67263424	67266939	INSL5	0	0
chr1.fa	67278572	67390570	WDR78	2.025806312	532
chr1.fa	67390578	67454302	MIER1	10.59651374	4071
chr1.fa	67465015	67520080	SLC35D1	4.198755049	1801
chr1.fa	67557859	67594220	C1orf141	0	0
chr1.fa	67632169	67725650	IL23R	0	0
chr1.fa	67773047	67862583	IL12RB2	0	0
chr1.fa	67873493	67896123	SERBP1	21.12389915	9424
chr1.fa	68150860	68154021	GADD45A	10.78251261	988
chr1.fa	68167149	68299155	GNG12	28.29713005	8284
chr1.fa	68511645	68516460	DIRAS3	0.701825694	76
chr1.fa	68564142	68698229	WLS	9.409133099	1893
chr1.fa	68649201	68649293	MIR1262	0	0
chr1.fa	68894507	68915642	RPE65	0	0
chr1.fa	68939835	68962799	DEPDC1	0	0
chr1.fa	70225858	70589171	LRRC7	0	0
chr1.fa	70385005	70386000	PIN1P1	0	0
chr1.fa	70610485	70671361	LRRC40	3.109268553	608
chr1.fa	70671365	70717701	SRSF11	21.27480673	3944
chr1.fa	70724685	70820417	ANKRD13C	9.227200601	3472
chr1.fa	70820493	70833705	HHLA3	8.041777846	456
chr1.fa	70876901	70905534	CTH	8.02295205	1140
chr1.fa	71318036	71513491	PTGER3	1.558775952	1064
chr1.fa	71528974	71546972	ZRANB2	11.28237516	2356
chr1.fa	71533314	71533399	MIR186	0	0
chr1.fa	71868625	72748277	NEGR1	0	0
chr1.fa	74491702	74663871	LRRIQ3	0	0
chr1.fa	74670075	74674148	FPGT	5.338092254	1444
chr1.fa	74701071	74701175	TNNI3K	0	0
chr1.fa	74930319	74930609	FPGT-TNNI3K	0	0
chr1.fa	75033795	75139422	C1orf173	0	0
chr1.fa	75171172	75198835	CRYZ	42.73064233	5805
chr1.fa	75198840	75232360	TYW3	8.072049727	1900
chr1.fa	75594119	75627218	LHX8	0	0

chr1.fa	75667816	76076799	SLC44A5	0.270940863	76
chr1.fa	76190043	76229355	ACADM	29.31492791	5090
chr1.fa	76251886	76260764	RABGGTB	33.92664563	3370
chr1.fa	76252757	76252834	SNORD45C	0	0
chr1.fa	76253574	76253657	SNORD45A	0	0
chr1.fa	76255162	76255232	SNORD45B	0	0
chr1.fa	76262630	76378923	MSH4	0	0
chr1.fa	76384558	76398116	ASB17	0	0
chr1.fa	76540389	77096669	ST6GALNAC3	0.316122775	76
chr1.fa	77333186	77529737	ST6GALNAC5	0	0
chr1.fa	77554667	77685132	PIGK	0.989935684	304
chr1.fa	77747742	78025654	AK5	0.268982981	76
chr1.fa	78030190	78148343	ZZZ3	23.29986001	6688
chr1.fa	78161674	78225537	USP33	29.16311669	8857
chr1.fa	78245309	78344081	FAM73A	4.784613836	1672
chr1.fa	78354200	78409578	NEXN	0.675469579	152
chr1.fa	78413591	78444777	FUBP1	28.24125508	5408
chr1.fa	78470636	78482995	DNAJB4	4.605091041	684
chr1.fa	78511589	78603112	GIPC2	2.432744729	456
chr1.fa	78695283	78835147	MGC27382	0	0
chr1.fa	78956728	79006386	PTGFR	0.63224555	228
chr1.fa	79086088	79111830	IFI44L	0.779387976	304
chr1.fa	79115477	79129763	IFI44	6.631499778	760
chr1.fa	79355449	79472495	ELTD1	0	0
chr1.fa	82266082	82458107	LPHN2	5.014890979	1900
chr1.fa	84335057	84464833	TTLL7	13.17805756	3192
chr1.fa	84543745	84704181	PRKACB	14.08305125	5244
chr1.fa	84764049	84816481	SAMD13	4.443791616	532
chr1.fa	84830641	84863576	UOX	0	0
chr1.fa	84864215	84880691	DNASE2B	12.76509489	1140
chr1.fa	84944920	84964005	RPF1	10.58190492	1368
chr1.fa	84964034	84972262	GNG5	52.23119349	2660
chr1.fa	84971985	85031877	SPATA1	1.333318212	152
chr1.fa	85018808	85040163	CTBS	5.441257619	1064
chr1.fa	85093913	85100703	C1orf180	0	0
chr1.fa	85109390	85156240	SSX2IP	5.241854782	2128
chr1.fa	85279086	85358896	LPAR3	10.64139444	1216
chr1.fa	85391266	85462796	MCOLN2	0	0
chr1.fa	85483765	85514169	MCOLN3	0	0
chr1.fa	85527993	85598821	WDR63	0	0
chr1.fa	85623356	85666728	SYDE2	5.588249438	1748
chr1.fa	85715637	85725355	C1orf52	1.350637945	304
chr1.fa	85731460	85742587	BCL10	9.218766644	1900

chr1.fa	85784168	86044046	DDAH1		49.54347218	14744
chr1.fa	86046444	86049648	CYR61		2.495848799	380
chr1.fa	86115106	86174116	ZNHIT6		16.4288961	6760
chr1.fa	86194916	86622121	COL24A1		0	0
chr1.fa	86812507	86862025	ODF2L		2.833207072	1216
chr1.fa	86889769	86922240	CLCA2		0.568840268	152
chr1.fa	86934526	86965974	CLCA1		0	0
chr1.fa	87012759	87046432	CLCA4		0	0
chr1.fa	87099959	87121059	CLCA3P		0	0
chr1.fa	87170257	87213867	SH3GLB1		19.39704649	8208
chr1.fa	87328128	87380107		15-Sep	14.92960967	1824
chr1.fa	87380335	87575681	HS2ST1		2.048096055	988
chr1.fa	87595448	87634886	LOC339524		0	0
chr1.fa	87794151	87814607	LMO4		1.270364749	456
chr1.fa	89149922	89301938	PKN2		15.56396371	6308
chr1.fa	89318321	89357301	GTF2B		18.09113863	1976
chr1.fa	89401456	89435150	CCBL2		22.36625111	2489
chr1.fa	89445139	89449749	RBMXL1		7.260883807	2223
chr1.fa	89472360	89488549	GBP3		36.52204524	7372
chr1.fa	89517987	89531043	GBP1		6.37983653	1292
chr1.fa	89573310	89591799	GBP2		7.498390056	1292
chr1.fa	89597434	89641723	GBP7		0	0
chr1.fa	89646831	89664633	GBP4		6.891898195	2812
chr1.fa	89724634	89738544	GBP5		0	0
chr1.fa	89829436	89853719	GBP6		1.175934554	380
chr1.fa	89873238	89890493	LOC400759		0	0
chr1.fa	89990397	90063420	LRRC8B		2.129875315	1140
chr1.fa	90090408	90098453	FLJ27354		0	0
chr1.fa	90098644	90185094	LRRC8C		0.317628839	152
chr1.fa	90286573	90401989	LRRC8D		3.10731067	836
chr1.fa	90458824	90460525	GEMIN8P4		0.672457452	76
chr1.fa	90460678	90494094	ZNF326		11.29818882	2042
chr1.fa	91177579	91182794	BARHL2		0	0
chr1.fa	91380857	91487671	ZNF644		13.95142128	5396
chr1.fa	91726323	91870426	HFM1		0.232084419	76
chr1.fa	91966404	91991321	CDC7		1.00544814	228
chr1.fa	92100568	92109335	HSP90B3P		0	0
chr1.fa	92145900	92371559	TGFBR3		3.535333979	1596
chr1.fa	92414928	92479985	BRDT		0	0
chr1.fa	92495533	92529093	EPHX4		0	0
chr1.fa	92545862	92613401	BTBD8		2.373104606	228
chr1.fa	92632609	92650280	KIAA1107		5.024680393	1520
chr1.fa	92683573	92711367	C1orf146		0	0

chr1.fa	92711955	92764521	GLMN	3.986851883	526
chr1.fa	92764567	92853732	RPAP2	9.938966316	2024
chr1.fa	92940318	92952433	GFI1	0.786617082	152
chr1.fa	92974253	93257961	EVI5	8.349165452	4104
chr1.fa	93297594	93307481	RPL5	1188.943028	81391
chr1.fa	93302846	93302940	SNORD21	0	0
chr1.fa	93306276	93306408	SNORA66	0	0
chr1.fa	93307721	93427079	FAM69A	1.761642735	304
chr1.fa	93544792	93604638	MTF2	4.991095172	1368
chr1.fa	93615299	93645919	TMED5	1.953967072	760
chr1.fa	93646247	93744268	CCDC18	2.908510259	836
chr1.fa	93775666	93811368	LOC100131564	1.419013238	380
chr1.fa	93811478	93828148	DR1	19.26707319	4104
chr1.fa	93913688	94020218	FNBP1L	36.30712995	13300
chr1.fa	94027349	94147385	BCAR3	5.012029458	1012
chr1.fa	94057525	94065587	LOC100129046	0	0
chr1.fa	94312388	94312467	MIR760	0	0
chr1.fa	94335014	94344762	DNTTIP2	23.96870291	4332
chr1.fa	94352590	94375012	GCLM	9.593023479	1958
chr1.fa	94458394	94586705	ABCA4	0	0
chr1.fa	94634463	94703307	ARHGAP29	15.8189403	9576
chr1.fa	94883933	94984219	ABCD3	33.83387211	8530
chr1.fa	94994732	95007413	F3	0.956651676	152
chr1.fa	95285901	95360802	SLC44A3	4.893351637	836
chr1.fa	95362507	95392735	CNN3	55.95614089	7676
chr1.fa	95393584	95428826	LOC729970	8.416185287	532
chr1.fa	95448279	95538507	ALG14	4.331589869	304
chr1.fa	95558073	95663161	TMEM56	0.170787626	76
chr1.fa	95583479	95710509	TMEM56-RWDD3	0	0
chr1.fa	95699711	95712781	RWDD3	6.503484361	380
chr1.fa	95940293	95944912	FLJ31662	0	0
chr1.fa	97187175	97280605	PTBP2	5.063386231	1172
chr1.fa	97543300	98386615	DPYD	1.426694163	532
chr1.fa	98511626	98511727	MIR137	0	0
chr1.fa	99127236	99226056	SNX7	7.240551946	912
chr1.fa	99355801	99470449	LPPR5	0	0
chr1.fa	99469832	99614408	LOC100129620	0	0
chr1.fa	99729848	99775138	LPPR4	0	0
chr1.fa	100111431	100160097	PALMD	16.08355569	2736
chr1.fa	100174259	100231349	FRRS1	3.846637351	684
chr1.fa	100315640	100389579	AGL	7.939967939	4332
chr1.fa	100435540	100489006	SLC35A3	3.19571661	456
chr1.fa	100503789	100548929	HIAT1	7.813006767	1368

chr1.fa	100549102	100598511	SASS6	0.587364851	152
chr1.fa	100598706	100616054	CCDC76	3.94423028	760
chr1.fa	100617941	100643771	LRRC39	0	0
chr1.fa	100652478	100715409	DBT	10.47768531	7524
chr1.fa	100731714	100758325	RTCD1	21.22721512	3697
chr1.fa	100746797	100746864	MIR553	0	0
chr1.fa	100818023	100985833	CDC14A	5.590357927	1585
chr1.fa	101003728	101007583	GPR88	0	0
chr1.fa	101185196	101204601	VCAM1	1.786793999	380
chr1.fa	101337941	101360418	EXTL2	1.605915746	304
chr1.fa	101361632	101447311	SLC30A7	2.46301661	1292
chr1.fa	101455180	101491362	DPH5	15.03563656	1804
chr1.fa	101702305	101707076	S1PR1	0	0
chr1.fa	102268127	102462790	OLFM3	0	0
chr1.fa	102337567	102360299	HEJ1	0	0
chr1.fa	103342023	103574052	COL11A1	0	0
chr1.fa	104068578	104097859	RNPC3	5.061880167	608
chr1.fa	104097322	104122149	AMY2B	5.987055111	836
chr1.fa	104112026	104114008	LOC648740	0	0
chr1.fa	104159999	104168400	AMY2A	0	0
chr1.fa	104198141	104292440	AMY1A	0	0
chr1.fa	104198325	104292688	AMY1B	0	0
chr1.fa	104238890	104238905	AMY1C	0	0
chr1.fa	104615645	104619693	LOC100129138	0	0
chr1.fa	107599267	107601916	PRMT6	12.09399289	2128
chr1.fa	107682629	108024475	NTNG1	0.658451059	152
chr1.fa	108113782	108507545	VAV3	7.588753879	2403
chr1.fa	108677447	108742974	SLC25A24	15.99123399	3952
chr1.fa	108765963	108786703	NBPF4	0	0
chr1.fa	108992904	109013260	NBPF6	0	0
chr1.fa	109102971	109181949	FAM102B	6.430440271	2280
chr1.fa	109190910	109204148	C1orf59	6.729695132	912
chr1.fa	109234932	109244422	PRPF38B	10.97061996	2736
chr1.fa	109255556	109285367	FNDC7	0	0
chr1.fa	109289285	109352148	STXBP3	21.49288476	3572
chr1.fa	109358525	109399716	AKNAD1	0	0
chr1.fa	109399839	109401146	LOC642864	0	0
chr1.fa	109419603	109466836	GPSM2	0.990538109	152
chr1.fa	109472130	109506111	CLCC1	3.068604832	836
chr1.fa	109512838	109584850	WDR47	9.427055257	2736
chr1.fa	109606998	109618624	TAF13	35.70696356	1368
chr1.fa	109633403	109639554	TMEM167B	21.64349113	3952
chr1.fa	109642815	109643234	SCARNA2	0	0

chr1.fa	109648573	109656479	C1orf194	0	0
chr1.fa	109656585	109749403	KIAA1324	6.348058585	2964
chr1.fa	109756515	109780804	SARS	17.96959929	7524
chr1.fa	109792641	109818378	CELSR2	1.303196938	912
chr1.fa	109822176	109825790	PSRC1	2.095085243	228
chr1.fa	109834987	109849663	MYBPHL	0	0
chr1.fa	109852192	109940563	SORT1	9.133372831	4256
chr1.fa	109941653	109969108	PSMA5	17.77335919	4560
chr1.fa	110009100	110024764	SYPL2	0.908608243	228
chr1.fa	110026561	110035420	ATXN7L2	0	0
chr1.fa	110036658	110043063	CYB561D1	5.326796776	1824
chr1.fa	110049446	110052336	AMIGO1	1.389644996	228
chr1.fa	110082494	110088455	GPR61	0	0
chr1.fa	110091186	110138452	GNAI3	18.05047491	5669
chr1.fa	110141515	110141589	MIR197	0	0
chr1.fa	110145889	110155705	GNAT2	0	0
chr1.fa	110162459	110174677	AMPD2	3.754466251	1140
chr1.fa	110198698	110208123	GSTM4	11.34457559	1444
chr1.fa	110210644	110226619	GSTM2	0.567183597	76
chr1.fa	110230418	110236367	GSTM1	312.9562761	24624
chr1.fa	110254864	110260890	GSTM5	24.78770036	2584
chr1.fa	110276554	110283660	GSTM3	63.70935693	17458
chr1.fa	110292702	110306564	EPS8L3	1.022918479	152
chr1.fa	110453233	110472355	CSF1	0	0
chr1.fa	110527308	110566364	AHCYL1	57.71868727	15414
chr1.fa	110577241	110597263	FAM40A	14.38652309	3116
chr1.fa	110602997	110613322	ALX3	0.774417965	76
chr1.fa	110655062	110656569	UBL4B	0	0
chr1.fa	110693132	110744823	SLC6A17	0	0
chr1.fa	110753336	110776674	KCNC4	0.555135088	152
chr1.fa	110828999	110881793	LOC440600	0	0
chr1.fa	110881945	110889303	RBM15	6.00497727	1368
chr1.fa	110905505	110933636	SLC16A4	7.241455585	1216
chr1.fa	110943877	110950546	HBXIP	97.18373163	5472
chr1.fa	110993788	110999976	PROK1	0	0
chr1.fa	111023388	111033891	CYMP	0	0
chr1.fa	111059839	111061797	KCNA10	0	0
chr1.fa	111145776	111148345	KCNA2	0	0
chr1.fa	111214310	111217655	KCNA3	0	0
chr1.fa	111413821	111442558	CD53	0	0
chr1.fa	111489812	111506566	C1orf103	6.177722778	1368
chr1.fa	111659954	111682832	DRAM2	7.306065718	912
chr1.fa	111682249	111727724	CEPT1	6.353480415	988

chr1.fa	111729801	111743281	DENND2D	36.94359248	5009
chr1.fa	111770281	111786062	CHI3L2	0	0
chr1.fa	111823146	111828730	LOC149620	0	0
chr1.fa	111833484	111863185	CHIA	0	0
chr1.fa	111889195	111895639	C1orf88	19.51587491	3040
chr1.fa	111927141	111932473	LOC441897	3.765159303	152
chr1.fa	111956937	111970399	OVGP1	2.0420718	304
chr1.fa	111982512	111991742	WDR77	29.40724962	4530
chr1.fa	111991831	112004525	ATP5F1	49.64844482	6636
chr1.fa	112016604	112021134	C1orf162	0	0
chr1.fa	112025970	112106597	ADORA3	0	0
chr1.fa	112141629	112150940	LOC100129269	0	0
chr1.fa	112162405	112256101	RAP1A	38.24422911	4840
chr1.fa	112264686	112282046	C1orf183	0	0
chr1.fa	112298190	112310199	DDX20	5.030554042	1140
chr1.fa	112318454	112531777	KCND3	0.850022364	152
chr1.fa	112938800	113003786	CTTNBP2NL	18.62413458	7296
chr1.fa	113004392	113004455	MIR4256	0	0
chr1.fa	113010040	113063910	WNT2B	0	0
chr1.fa	113066141	113162040	ST7L	1.70953293	532
chr1.fa	113162075	113214241	CAPZA1	54.10835131	9844
chr1.fa	113217048	113243368	MOV10	9.708237354	2280
chr1.fa	113243749	113250025	RHOC	77.6723749	8360
chr1.fa	113252616	113257950	PPM1J	0.667788654	76
chr1.fa	113263189	113269856	FAM19A3	0	0
chr1.fa	113454470	113498975	SLC16A1	0.490374348	152
chr1.fa	113465972	113467295	AKR7A2P1	0	0
chr1.fa	113615831	113667342	LRIG2	4.27631733	1140
chr1.fa	113933475	114228545	MAGI3	9.938213284	4765
chr1.fa	114239824	114301777	PHTF1	3.528406086	760
chr1.fa	114304454	114355070	RSBN1	7.11795836	3116
chr1.fa	114356433	114414375	PTPN22	0	0
chr1.fa	114419436	114430169	BCL2L15	0.230126537	76
chr1.fa	114437677	114447741	AP4B1	5.529512953	912
chr1.fa	114447915	114456708	DCLRE1B	1.812999508	456
chr1.fa	114471996	114520422	HIPK1	26.16725473	14440
chr1.fa	114522030	114524875	OLFML3	3.154902283	380
chr1.fa	114631914	114696472	SYT6	1.833481974	532
chr1.fa	114935399	115053781	TRIM33	18.5300056	10260
chr1.fa	115110181	115124265	BCAS2	26.35897664	2277
chr1.fa	115127196	115212732	DENND2C	2.016167504	532
chr1.fa	115215720	115238239	AMPD1	0	0
chr1.fa	115247078	115259515	NRAS	15.14166344	4485

chr1.fa	115259537	115300624	CSDE1	238.9635161	67640
chr1.fa	115312105	115323308	SIKE1	7.487847609	2736
chr1.fa	115397455	115537990	SYCP1	0	0
chr1.fa	115572415	115576941	TSHB	0	0
chr1.fa	115590633	115632121	TSPAN2	1.069004029	228
chr1.fa	115828537	115880857	NGF	0	0
chr1.fa	116184574	116240845	VANGL1	3.39376399	1976
chr1.fa	116242626	116311426	CASQ2	0	0
chr1.fa	116378999	116383747	NHLH2	0	0
chr1.fa	116519119	116612675	SLC22A15	1.69748442	532
chr1.fa	116654376	116677861	C1orf161	0.708904194	152
chr1.fa	116915795	116947335	ATP1A1	62.26504182	16194
chr1.fa	116935611	116961244	ATP1A1OS	4.066070835	608
chr1.fa	117057156	117113715	CD58	0	0
chr1.fa	117117031	117210314	IGSF3	4.418790958	2128
chr1.fa	117214371	117214449	MIR320B1	0	0
chr1.fa	117297086	117311851	CD2	0	0
chr1.fa	117452689	117532972	PTGFRN	3.344666312	1368
chr1.fa	117544382	117579167	CD101	0	0
chr1.fa	117602949	117645491	TTF2	7.545831063	2432
chr1.fa	117637265	117637350	MIR942	0	0
chr1.fa	117653677	117664411	TRIM45	2.237408264	532
chr1.fa	117686209	117753549	VTCN1	2.636364544	456
chr1.fa	117910085	118068320	MAN1A2	24.85502141	8892
chr1.fa	118148604	118171011	FAM46C	15.80839785	6004
chr1.fa	118406107	118472302	GDAP2	5.448185512	3496
chr1.fa	118472372	118503049	WDR3	14.89572324	3792
chr1.fa	118496288	118727848	SPAG17	0.646553156	304
chr1.fa	119425666	119532179	TBX15	0	0
chr1.fa	119573839	119683295	WARS2	8.478536325	1596
chr1.fa	119911402	119936751	HAO2	50.61654258	5700
chr1.fa	119957554	119965662	HSD3B2	0	0
chr1.fa	120049826	120057681	HSD3B1	0	0
chr1.fa	120106503	120115199	HSD3BP4	0	0
chr1.fa	120140325	120141914	LOC644242	0.719898459	76
chr1.fa	120162000	120190390	ZNF697	9.913965658	3344
chr1.fa	120254419	120286849	PHGDH	79.15238372	10590
chr1.fa	120290619	120311555	HMGCS2	65.44599901	10716
chr1.fa	120336641	120354203	REG4	0.50603741	76
chr1.fa	120377388	120387779	NBPF7	0	0
chr1.fa	120436156	120439113	ADAM30	0	0
chr1.fa	120454176	120612317	NOTCH2	4.355837495	3420
chr1.fa	120839005	120855681	FAM72B	0	0

chr1.fa	120906034	120914842	HIST2H2BA	0	0
chr1.fa	120926908	120935944	FCGR1B	0	0
chr1.fa	121260910	121313686	EMBP1	0	0
chr1.fa	143687130	143714180	LOC100130000	0	0
chr1.fa	143767144	143767881	PPIAL4G	0	0
chr1.fa	143896452	143913143	FAM72D	0	0
chr1.fa	143915748	144094477	SRGAP2P2	16.62061802	1140
chr1.fa	144300512	149616786	LOC728855	0	0
chr1.fa	144341670	144521969	LOC728875	0	0
chr1.fa	144610815	144612727	C1orf152	0	0
chr1.fa	144614959	144828810	NBPF9	2.34554364	456
chr1.fa	144676437	144680025	LOC653513	0	0
chr1.fa	144851424	145076186	PDE4DIP	8.405040416	7192
chr1.fa	145096407	145116922	SEC22B	85.48417682	10336
chr1.fa	145209111	145285912	NOTCH2NL	5.536139633	1824
chr1.fa	145293371	145368684	NBPF10	2.120387113	1506
chr1.fa	145413191	145417545	HFE2	0	0
chr1.fa	145438462	145442628	TXNIP	313.6234624	60952
chr1.fa	145456236	145470387	POLR3GL	10.80751326	836
chr1.fa	145470508	145475647	ANKRD34A	0.950627421	228
chr1.fa	145477085	145499091	LIX1L	8.986983438	836
chr1.fa	145507638	145509819	RBM8A	23.64083284	1824
chr1.fa	145511445	145516076	GNRHR2	4.200863538	1292
chr1.fa	145516165	145523732	PEX11B	19.60141933	2508
chr1.fa	145524990	145543868	ITGA10	0	0
chr1.fa	145549209	145568526	ANKRD35	5.405413302	1207
chr1.fa	145575988	145586492	PIAS3	15.27208856	2888
chr1.fa	145586547	145589435	NUDT17	2.207588203	152
chr1.fa	145592605	145610884	POLR3C	19.15336538	2354
chr1.fa	145611036	145688776	RNF115	15.02464229	1671
chr1.fa	145695798	145715565	CD160	0	0
chr1.fa	145727726	145764073	PDZK1	9.778269317	1368
chr1.fa	145764595	145827103	GPR89A	1.689803495	228
chr1.fa	145883868	147465755	GPR89C	0	0
chr1.fa	145924388	147484331	PDZK1P1	0	0
chr1.fa	146032542	147624469	NBPF11	0	0
chr1.fa	146057587	147607526	NBPF24	0	0
chr1.fa	146490895	146514599	LOC728989	0	0
chr1.fa	146626685	146644129	PRKAB2	9.920140519	3572
chr1.fa	146649430	146651528	PDIA3P	0.545345673	76
chr1.fa	146655884	146697230	FMO5	9.724502842	2052
chr1.fa	146714291	146767443	CHD1L	20.15429532	4028
chr1.fa	147013182	147098015	BCL9	9.301298936	3876

chr1.fa	147119168	147142634	ACP6	20.25851493	2432
chr1.fa	147228332	147245484	GJA5	0	0
chr1.fa	147374946	147381395	GJA8	0	0
chr1.fa	147400506	147416212	GPR89B	0	0
chr1.fa	147906022	147931980	FLJ39739	0	0
chr1.fa	147954635	149553787	PPIAL4B	0	0
chr1.fa	148003642	148025848	NBPF14	4.343939591	1064
chr1.fa	148558188	148577251	NBPF15	0.108436588	7
chr1.fa	148577357	148758311	NBPF16	0.26069963	69
chr1.fa	148644011	148644795	PPIAL4E	0	0
chr1.fa	148928286	148953054	LOC645166	0	0
chr1.fa	149279476	149291742	LOC388692	0.466578541	380
chr1.fa	149369294	149378297	FCGR1C	0	0
chr1.fa	149397114	149783928	HIST2H2BF	3.916970526	1064
chr1.fa	149755559	149764074	FCGR1A	0.551821747	76
chr1.fa	149784780	149785236	HIST2H3D	0	0
chr1.fa	149821759	149822340	HIST2H2BC	0	0
chr1.fa	149856010	149858232	HIST2H2BE	131.8128054	19456
chr1.fa	149858525	149858961	HIST2H2AC	23.57321058	684
chr1.fa	149859019	149859466	HIST2H2AB	10.21969659	304
chr1.fa	149871155	149872348	BOLA1	12.02034638	684
chr1.fa	149874872	149889434	SV2A	0	0
chr1.fa	149895211	149899702	SF3B4	21.42902766	2204
chr1.fa	149900543	149908791	MTMR11	0	0
chr1.fa	149912232	149982686	OTUD7B	33.73838767	14364
chr1.fa	150039342	150117505	VPS45	13.40863592	2508
chr1.fa	150122170	150131825	PLEKHO1	0.708753587	76
chr1.fa	150190717	150208504	ANP32E	4.510209026	1064
chr1.fa	150230218	150237480	CA14	0	0
chr1.fa	150237799	150241532	APH1A	30.32760516	4408
chr1.fa	150245183	150253335	C1orf54	0	0
chr1.fa	150255229	150259501	C1orf51	0.657095602	76
chr1.fa	150266269	150280819	MRPS21	49.97751974	3116
chr1.fa	150293928	150325704	PRPF3	16.72995824	2736
chr1.fa	150336990	150449041	RPRD2	18.00800392	9348
chr1.fa	150459920	150479749	TARS2	5.30586249	836
chr1.fa	150480487	150486265	ECM1	1.601548161	228
chr1.fa	150521898	150533412	ADAMTSL4	0	0
chr1.fa	150524405	150524490	MIR4257	0	0
chr1.fa	150547027	150552214	MCL1	52.39701111	14212
chr1.fa	150594599	150602098	ENSA	39.21217626	10792
chr1.fa	150618701	150669672	GOLPH3L	29.23781745	6156
chr1.fa	150670535	150693364	HORMAD1	0	0

chr1.fa	150702672	150738433	CTSS	1.67218255	456
chr1.fa	150768684	150780917	CTSK	0.632396157	76
chr1.fa	150782181	150849244	ARNT	13.58439355	4408
chr1.fa	150898815	150937220	SETDB1	14.8878917	4395
chr1.fa	150937649	150947440	LASS2	42.49449153	7068
chr1.fa	150954499	150968114	ANXA9	57.04713345	6909
chr1.fa	150969301	150980854	FAM63A	38.40146216	5941
chr1.fa	150980973	151008189	PRUNE	17.30181064	3420
chr1.fa	151009029	151020076	BNIPL	6.169138215	988
chr1.fa	151020259	151023446	C1orf56	1.428049621	152
chr1.fa	151023872	151032125	CDC42SE1	41.50139312	7600
chr1.fa	151032151	151040973	MLLT11	1.060871285	152
chr1.fa	151043080	151091007	GABPB2	10.54937394	1368
chr1.fa	151104163	151119140	SEMA6C	0	0
chr1.fa	151129105	151132223	TNFAIP8L2	0	0
chr1.fa	151132226	151138424	LYSMD1	5.024228574	836
chr1.fa	151138517	151141614	SCNM1	21.34468809	1216
chr1.fa	151142463	151148547	TMOD4	0	0
chr1.fa	151148934	151162640	VPS72	25.93517031	2280
chr1.fa	151171021	151222007	PIP5K1A	16.83688877	4256
chr1.fa	151227197	151239954	PSMD4	45.53267388	4024
chr1.fa	151254791	151264272	ZNF687	14.5710159	4256
chr1.fa	151264382	151300133	PI4KB	44.72873706	12435
chr1.fa	151313116	151319769	RFX5	15.50191388	3724
chr1.fa	151336780	151345164	SELENBP1	30.20968037	3420
chr1.fa	151372041	151374412	PSMB4	193.0367053	11856
chr1.fa	151375200	151431941	POGZ	19.55217105	8567
chr1.fa	151483862	151511167	CGN	54.52658521	18468
chr1.fa	151512781	151556059	TUFT1	21.33956747	4408
chr1.fa	151518272	151518367	MIR554	0	0
chr1.fa	151584662	151671559	SNX27	24.39461773	11476
chr1.fa	151672534	151688882	CELF3	0.210397102	76
chr1.fa	151694013	151702082	C1orf230	0	0
chr1.fa	151732123	151736040	MRPL9	33.27933944	2508
chr1.fa	151735445	151743806	OAZ3	12.96931713	800
chr1.fa	151744041	151763010	TDRKH	8.067983355	1748
chr1.fa	151772765	151777882	LINGO4	0	0
chr1.fa	151778547	151804348	RORC	17.09427506	3648
chr1.fa	151810339	151813033	C2CD4D	0.839178705	76
chr1.fa	151814217	151816641	LOC100132111	6.068081339	166
chr1.fa	151819577	151826173	THEM5	0	0
chr1.fa	151846060	151882113	THEM4	5.188540126	760
chr1.fa	151955386	151966714	S100A10	133.4471857	9348

chr1.fa	152004982	152009511	S100A11	553.0347312	21812
chr1.fa	152056620	152061540	TCHHL1	0	0
chr1.fa	152078793	152086556	TCHH	0	0
chr1.fa	152126071	152131704	RPTN	0.641432539	152
chr1.fa	152184558	152196669	HRNR	0.118979034	76
chr1.fa	152274651	152297679	FLG	0.359196198	304
chr1.fa	152321213	152332482	FLG2	0	0
chr1.fa	152381719	152386750	CRNN	0.598359116	76
chr1.fa	152483320	152484653	LCE5A	0	0
chr1.fa	152486978	152488481	CRCT1	0	0
chr1.fa	152538130	152539248	LCE3E	0	0
chr1.fa	152551860	152552980	LCE3D	0	0
chr1.fa	152573138	152573562	LCE3C	0	0
chr1.fa	152586287	152586574	LCE3B	0	0
chr1.fa	152595310	152595579	LCE3A	0	0
chr1.fa	152635872	152637135	LCE2D	0	0
chr1.fa	152647771	152649049	LCE2C	0	0
chr1.fa	152658599	152659876	LCE2B	0	0
chr1.fa	152670840	152671918	LCE2A	0	0
chr1.fa	152681523	152681910	LCE4A	0	0
chr1.fa	152691998	152692905	C1orf68	0	0
chr1.fa	152730506	152734529	KPRP	0	0
chr1.fa	152748848	152749445	LCE1F	0	0
chr1.fa	152758753	152760901	LCE1E	0	0
chr1.fa	152769227	152770657	LCE1D	0	0
chr1.fa	152777311	152779107	LCE1C	0	0
chr1.fa	152784447	152785585	LCE1B	0	0
chr1.fa	152799949	152800573	LCE1A	0	0
chr1.fa	152815330	152816459	LCE6A	0	0
chr1.fa	152850798	152857523	SMCP	0	0
chr1.fa	152881039	152884362	IVL	8.041777846	1140
chr1.fa	152943128	152945069	SPRR4	0	0
chr1.fa	152956564	152958290	SPRR1A	5.242457207	228
chr1.fa	152974223	152976332	SPRR3	42.95188309	2812
chr1.fa	153003679	153005376	SPRR1B	1.849145037	76
chr1.fa	153012201	153013594	SPRR2D	0	0
chr1.fa	153028596	153029988	SPRR2A	1.690707134	76
chr1.fa	153042718	153044084	SPRR2B	0	0
chr1.fa	153065611	153067001	SPRR2E	0	0
chr1.fa	153084613	153085989	SPRR2F	0	0
chr1.fa	153112594	153113969	SPRR2C	0	0
chr1.fa	153122058	153123427	SPRR2G	0	0
chr1.fa	153175919	153177596	LELP1	0	0

chr1.fa	153190060	153191793	PRR9	0	0
chr1.fa	153232179	153234600	LOR	0	0
chr1.fa	153270338	153283194	PGLYRP3	0	0
chr1.fa	153302597	153321022	PGLYRP4	0	0
chr1.fa	153330330	153333503	S100A9	33.72332703	1292
chr1.fa	153346184	153348075	S100A12	0	0
chr1.fa	153362508	153363664	S100A8	13.13121898	456
chr1.fa	153389000	153395701	S100A7A	0	0
chr1.fa	153409471	153412503	S100A7L2	0	0
chr1.fa	153430220	153433137	S100A7	2.607297514	76
chr1.fa	153507076	153508717	S100A6	377.0671496	17100
chr1.fa	153509623	153514241	S100A5	3.224181215	152
chr1.fa	153516095	153518282	S100A4	32.47118565	1216
chr1.fa	153519809	153521734	S100A3	0	0
chr1.fa	153533585	153538306	S100A2	4.759311966	304
chr1.fa	153579367	153585514	S100A16	37.51062547	2660
chr1.fa	153586732	153588790	S100A14	5.492313179	380
chr1.fa	153591276	153606524	S100A13	12.22306256	1064
chr1.fa	153600873	153604509	S100A1	5.820032645	228
chr1.fa	153606569	153617979	C1orf77	37.57327772	3116
chr1.fa	153631145	153634326	SNAPIN	25.43575958	1748
chr1.fa	153634514	153643479	ILF2	84.71141552	9067
chr1.fa	153651164	153666468	NPR1	0	0
chr1.fa	153700567	153746555	INTS3	23.28645605	6930
chr1.fa	153747768	153752633	SLC27A3	0.474410072	76
chr1.fa	153777203	153895451	GATAD2B	13.01389662	6460
chr1.fa	153901977	153919154	DENND4B	6.23826654	2356
chr1.fa	153920148	153931132	CRTC2	13.21043793	2349
chr1.fa	153931588	153940188	SLC39A1	9.43232648	1520
chr1.fa	153940397	153946744	CREB3L4	17.93842377	1976
chr1.fa	153946833	153950451	JTB	83.25911828	8215
chr1.fa	153954128	153958806	RAB13	74.88525338	5733
chr1.fa	153963239	153964631	RPS27	45.65391201	1064
chr1.fa	153965168	154127592	NUP210L	0	0
chr1.fa	154127780	154164609	TPM3	20.20776059	13219
chr1.fa	154166141	154166219	MIR190B	0	0
chr1.fa	154171848	154178809	C1orf189	0	0
chr1.fa	154179183	154193273	C1orf43	112.2961774	14152
chr1.fa	154192655	154243329	UBAP2L	28.9554305	8569
chr1.fa	154245039	154248355	HAX1	48.54148798	3800
chr1.fa	154293592	154297801	AQP10	0	0
chr1.fa	154298036	154323780	ATP8B2	4.102668183	1717
chr1.fa	154377669	154440188	IL6R	0.822310792	228

chr1.fa	154451954	154474526	SHE	0.185095231	76
chr1.fa	154474695	154520623	TDRD10	0	0
chr1.fa	154521051	154531120	UBE2Q1	26.22885274	5613
chr1.fa	154540257	154552353	CHRNB2	0	0
chr1.fa	154554534	154600456	ADAR	69.09910717	32277
chr1.fa	154679915	154842754	KCNN3	0.356033464	76
chr1.fa	154897208	154909484	PMVK	56.39335119	4864
chr1.fa	154916559	154928567	PBXIP1	5.730271247	1216
chr1.fa	154929502	154934258	PYGO2	34.55422238	7296
chr1.fa	154934774	154946959	SHC1	28.87335003	7126
chr1.fa	154947118	154951725	CKS1B	1.40274775	76
chr1.fa	154948169	154948259	MIR4258	0	0
chr1.fa	154955770	154965587	FLAD1	8.863185	1596
chr1.fa	154966062	154966791	LENEP	0	0
chr1.fa	154975112	154990999	ZBTB7B	35.13586419	8436
chr1.fa	154991003	155006257	DCST2	0	0
chr1.fa	155006282	155023406	DCST1	0.498356485	76
chr1.fa	155023762	155035252	ADAM15	3.086225778	608
chr1.fa	155036213	155042029	EFNA4	3.64241511	304
chr1.fa	155051348	155060014	EFNA3	0.642336177	76
chr1.fa	155100349	155107386	EFNA1	5.847292398	608
chr1.fa	155108288	155111334	SLC50A1	17.89776005	1596
chr1.fa	155112367	155112996	DPM3	20.81108971	532
chr1.fa	155141884	155145804	KRTCAP2	20.92524934	760
chr1.fa	155146360	155157445	TRIM46	0.364316814	76
chr1.fa	155158300	155162700	MUC1	3.906578687	304
chr1.fa	155164968	155165063	MIR92B	0	0
chr1.fa	155165379	155177690	THBS3	5.680571144	1187
chr1.fa	155178490	155183614	MTX1	7.714811412	836
chr1.fa	155183616	155197325	GBAP1	0	0
chr1.fa	155204239	155214653	GBA	2.215720947	380
chr1.fa	155216996	155225274	FAM189B	3.58804621	760
chr1.fa	155225770	155232195	SCAMP3	6.474869151	684
chr1.fa	155232659	155243281	CLK2	8.034247528	1140
chr1.fa	155247374	155259083	HCN3	0	0
chr1.fa	155259639	155271225	PKLR	2.632298172	456
chr1.fa	155278539	155290250	FDPS	58.82022227	5624
chr1.fa	155290458	155291478	C1orf104	0	0
chr1.fa	155291487	155300909	RUSC1	8.478536325	2204
chr1.fa	155305052	155491409	ASH1L	35.29746483	26554
chr1.fa	155316141	155316173	MIR555	0	0
chr1.fa	155402971	155404053	POU5F1P4	0	0
chr1.fa	155531772	155533735	LOC645676	0	0

chr1.fa	155580007	155584758	MSTO1	3.752809581	608
chr1.fa	155629233	155658764	YY1AP1	27.09800211	5324
chr1.fa	155658882	155708800	DAP3	52.06341799	7664
chr1.fa	155715609	155721369	MSTO2P	0	0
chr1.fa	155719510	155826972	GON4L	16.42739004	7680
chr1.fa	155829260	155854990	SYT11	0	0
chr1.fa	155867601	155881177	RIT1	12.98949838	2954
chr1.fa	155882836	155904188	KIAA0907	7.384531638	1444
chr1.fa	155889700	155889833	SNORA42	0	0
chr1.fa	155895749	155895877	SCARNA4	0	0
chr1.fa	155911480	155912625	RXFP4	0	0
chr1.fa	155916630	155948336	ARHGEF2	7.47414243	2128
chr1.fa	155978839	155990758	SSR2	90.63175201	6764
chr1.fa	156005092	156023516	UBQLN4	16.31895345	3800
chr1.fa	156024517	156028301	ROBLD3	52.10408171	2356
chr1.fa	156030966	156040295	RAB25	85.44998917	6156
chr1.fa	156041804	156051789	MEX3A	0.560707523	228
chr1.fa	156084461	156109878	LMNA	43.7604887	9728
chr1.fa	156119735	156147542	SEMA4A	10.93884202	2508
chr1.fa	156163730	156182587	SLC25A44	10.71489035	2584
chr1.fa	156195348	156209868	PMF1	16.0459041	684
chr1.fa	156211951	156212086	BGLAP	0	0
chr1.fa	156213207	156217843	PAQR6	0	0
chr1.fa	156219015	156252620	SMG5	35.0226082	10611
chr1.fa	156252704	156262234	TMEM79	13.61466543	2128
chr1.fa	156262484	156265449	C1orf85	22.38673358	2356
chr1.fa	156268415	156269428	VHLL	0	0
chr1.fa	156278752	156308206	CCT3	78.60989957	12433
chr1.fa	156307105	156316785	C1orf182	0	0
chr1.fa	156339003	156355011	RHBG	0	0
chr1.fa	156374055	156399184	C1orf61	0	0
chr1.fa	156390133	156390221	MIR9-1	0	0
chr1.fa	156433519	156470529	MEF2D	13.05561458	5092
chr1.fa	156495197	156542396	IQGAP3	0.189161603	76
chr1.fa	156549519	156556562	TTC24	0	0
chr1.fa	156561558	156564091	APOA1BP	43.90552263	3268
chr1.fa	156564100	156571279	GPATCH4	13.27851201	1900
chr1.fa	156589086	156595517	HAPLN2	0.646251943	76
chr1.fa	156611740	156629320	BCAN	0	0
chr1.fa	156638556	156647189	NES	0.617937945	228
chr1.fa	156669400	156675608	CRABP2	32.45025136	2508
chr1.fa	156692413	156697705	ISG20L2	17.85830118	2432
chr1.fa	156698263	156706752	C1orf66	5.044560434	760

chr1.fa	156707094	156710923	MRPL24	34.57289757	2128
chr1.fa	156711899	156722240	HDGF	69.13465027	12844
chr1.fa	156737274	156770609	PRCC	22.10494906	3116
chr1.fa	156776035	156786640	SH2D2A	0	0
chr1.fa	156785542	156851642	NTRK1	0	0
chr1.fa	156810665	156828712	INSRR	0	0
chr1.fa	156863523	156886226	PEAR1	0	0
chr1.fa	156890424	156902880	C1orf92	0	0
chr1.fa	156904632	157015162	ARHGEF11	17.31461218	7920
chr1.fa	156905923	156906036	MIR765	0	0
chr1.fa	157061835	157069600	ETV3L	0	0
chr1.fa	157094459	157108177	ETV3	19.32897241	3496
chr1.fa	157098154	157098463	CYCSP52	0	0
chr1.fa	157483167	157522310	FCRL5	0	0
chr1.fa	157543539	157567870	FCRL4	0	0
chr1.fa	157647978	157670647	FCRL3	0	0
chr1.fa	157715523	157746922	FCRL2	0	0
chr1.fa	157764194	157789940	FCRL1	0	0
chr1.fa	157800704	157811634	CD5L	0	0
chr1.fa	157963063	158065844	KIRREL	0.316875807	76
chr1.fa	158101834	158110430	LOC646268	0	0
chr1.fa	158149737	158156216	CD1D	0	0
chr1.fa	158223927	158228058	CD1A	0	0
chr1.fa	158259563	158264564	CD1C	0	0
chr1.fa	158297740	158301321	CD1B	0	0
chr1.fa	158323486	158327343	CD1E	0	0
chr1.fa	158368312	158369256	OR10T2	0	0
chr1.fa	158389718	158390656	OR10K2	0	0
chr1.fa	158435352	158436293	OR10K1	0	0
chr1.fa	158449668	158450675	OR10R2	0	0
chr1.fa	158516918	158517895	OR6Y1	0	0
chr1.fa	158532441	158533394	OR6P1	0	0
chr1.fa	158548709	158549689	OR10X1	0	0
chr1.fa	158576229	158577170	OR10Z1	0	0
chr1.fa	158580496	158656506	SPTA1	0	0
chr1.fa	158669468	158670442	OR6K2	0	0
chr1.fa	158686958	158687905	OR6K3	0	0
chr1.fa	158724606	158725637	OR6K6	0	0
chr1.fa	158735534	158736472	OR6N1	0	0
chr1.fa	158746472	158747425	OR6N2	0	0
chr1.fa	158801168	158819270	MNDA	0	0
chr1.fa	158901342	158946843	PYHIN1	0	0
chr1.fa	158979682	159024945	IFI16	4.186555932	760

chr1.fa	159032275	159046647	AIM2	0	0
chr1.fa	159141377	159172932	CADM3	0	0
chr1.fa	159173803	159176290	DARC	0	0
chr1.fa	159259504	159278014	FCER1A	0	0
chr1.fa	159283460	159284449	OR10J3	0	0
chr1.fa	159409512	159410600	OR10J1	0	0
chr1.fa	159504868	159505797	OR10J5	0	0
chr1.fa	159557616	159558661	APCS	0	0
chr1.fa	159682079	159684379	CRP	0	0
chr1.fa	159750759	159752333	DUSP23	13.36767098	608
chr1.fa	159772173	159786047	FCRL6	0	0
chr1.fa	159796479	159807282	SLAMF8	0	0
chr1.fa	159804264	159825137	C1orf204	0	0
chr1.fa	159824106	159832447	VSIG8	0	0
chr1.fa	159842154	159869906	CCDC19	2.550669518	304
chr1.fa	159887903	159895284	TAGLN2	101.8364143	9196
chr1.fa	159896829	159915386	IGSF9	1.718569312	456
chr1.fa	159921282	159924044	SLAMF9	0	0
chr1.fa	159997462	160001783	PIGM	1.059365222	304
chr1.fa	160007257	160040051	KCNJ10	0.647155581	228
chr1.fa	160051360	160059212	KCNJ9	0	0
chr1.fa	160061130	160068408	IGSF8	1.00755663	152
chr1.fa	160085520	160113374	ATP1A2	0	0
chr1.fa	160121352	160156767	ATP1A4	0.289465447	76
chr1.fa	160160285	160171676	CASQ1	1.169157267	152
chr1.fa	160175125	160185162	PEA15	75.10182534	12312
chr1.fa	160185505	160232350	DCAF8	23.91719553	7448
chr1.fa	160246599	160254941	PEX19	47.2679605	11352
chr1.fa	160258377	160313354	COPA	47.21765797	17024
chr1.fa	160287055	160288260	SUMO1P3	0	0
chr1.fa	160314512	160328742	NCSTN	5.458577351	988
chr1.fa	160336861	160342638	NHLH1	0	0
chr1.fa	160370364	160398468	VANGL2	0.427722097	152
chr1.fa	160454820	160493052	SLAMF6	0	0
chr1.fa	160510884	160549306	CD84	0	0
chr1.fa	160579891	160617081	SLAMF1	0	0
chr1.fa	160648536	160681585	CD48	0	0
chr1.fa	160709077	160724601	SLAMF7	0	0
chr1.fa	160765928	160798045	LY9	0	0
chr1.fa	160799950	160832692	CD244	1.368108284	228
chr1.fa	160846330	160854960	ITLN1	1.936797946	152
chr1.fa	160914816	160924589	ITLN2	5.971843868	456
chr1.fa	160965001	160991133	F11R	6.872319367	2204

chr1.fa	161007422	161008774	TSTD1	136.7279949	5320
chr1.fa	161009041	161015757	USF1	18.38783319	2194
chr1.fa	161016732	161039760	ARHGAP30	1.509226455	380
chr1.fa	161040781	161059385	PVRL4	20.59120441	4788
chr1.fa	161068151	161070138	KLHDC9	5.020162202	456
chr1.fa	161070346	161087861	PFDN2	37.73427593	1596
chr1.fa	161087867	161095235	NIT1	10.61052013	1824
chr1.fa	161090985	161102478	DEDD	13.46752301	1640
chr1.fa	161123534	161128646	UFC1	147.5862625	10995
chr1.fa	161129254	161135516	USP21	8.75233871	1368
chr1.fa	161136181	161141010	PPOX	3.88443955	456
chr1.fa	161141100	161147758	B4GALT3	9.562299779	1520
chr1.fa	161159538	161168845	ADAMTS4	0	0
chr1.fa	161169105	161184184	NDUFS2	51.52048202	6982
chr1.fa	161185087	161189038	FCER1G	0	0
chr1.fa	161192083	161193418	APOA2	0	0
chr1.fa	161195833	161200407	TOMM40L	5.787652275	988
chr1.fa	161200603	161208000	NR1I3	3.033362941	228
chr1.fa	161228517	161255240	PCP4L1	2.411358624	228
chr1.fa	161274525	161279762	MPZ	0	0
chr1.fa	161284166	161334520	SDHC	32.19166022	6064
chr1.fa	161334536	161337664	C1orf192	7.540258627	380
chr1.fa	161475205	161489360	FCGR2A	0	0
chr1.fa	161494036	161496687	HSPA6	0	0
chr1.fa	161511551	161520413	FCGR3A	0	0
chr1.fa	161551129	161570032	FCGR2C	0	0
chr1.fa	161575849	161578341	HSPA7	0	0
chr1.fa	161592988	161601158	FCGR3B	0.499109517	76
chr1.fa	161632905	161648444	FCGR2B	0	0
chr1.fa	161653495	161655042	RPL31P11	0	0
chr1.fa	161676762	161684142	FCRLA	0	0
chr1.fa	161692457	161697933	FCRLB	5.179202531	608
chr1.fa	161719581	161726952	DUSP12	4.582048266	380
chr1.fa	161736084	161928852	ATF6	10.56910338	1746
chr1.fa	161952982	161993644	OLFML2B	0	0
chr1.fa	162039581	162339813	NOS1AP	1.496274307	684
chr1.fa	162312336	162312430	MIR556	0	0
chr1.fa	162343515	162346644	C1orf111	0	0
chr1.fa	162348696	162356608	C1orf226	4.576325223	1292
chr1.fa	162365056	162381928	SH2D1B	0	0
chr1.fa	162466964	162499419	UHMK1	25.11843196	14440
chr1.fa	162531296	162569633	UAP1	40.82622475	6308
chr1.fa	162602228	162750247	DDR2	0.70574146	152

chr1.fa	162760496	162782608	HSD17B7	3.767569005	380
chr1.fa	162824087	162838605	C1orf110	0	0
chr1.fa	163038396	163046592	RGS4	0.863275725	228
chr1.fa	163112089	163172963	RGS5	0.386305345	152
chr1.fa	163291723	163325553	NUF2	0	0
chr1.fa	164528802	164821045	PBX1	19.5249113	8664
chr1.fa	165171104	165325952	LMX1A	0	0
chr1.fa	165370159	165414592	RXRG	0	0
chr1.fa	165446079	165551341	LOC400794	0	0
chr1.fa	165513478	165533185	LRRC52	0	0
chr1.fa	165600450	165624855	MGST3	115.7230748	5079
chr1.fa	165631449	165667900	ALDH9A1	69.13434906	11476
chr1.fa	165667987	165679199	LOC440700	0	0
chr1.fa	165693528	165738135	TMCO1	2.283192601	674
chr1.fa	165796732	165880855	UCK2	9.648145411	3116
chr1.fa	166039256	166135958	FAM78B	0.772911902	76
chr1.fa	166123980	166124035	MIR921	0	0
chr1.fa	166573153	166594473	FMO9P	44.54062971	4028
chr1.fa	166808724	166823709	POGK	68.93795835	17632
chr1.fa	166825749	166845654	TADA1	7.071119778	1055
chr1.fa	166882441	166944561	ILDR2	0.281633916	152
chr1.fa	166958519	166991447	MAEL	0	0
chr1.fa	167022082	167059868	GPA33	0	0
chr1.fa	167064087	167098402	DUSP27	0	0
chr1.fa	167190066	167396582	POU2F1	6.00994728	5694
chr1.fa	167399877	167487847	CD247	0	0
chr1.fa	167510251	167523056	CREG1	11.3048155	1520
chr1.fa	167599474	167675486	RCSD1	0.382690792	76
chr1.fa	167691187	167761156	MPZL1	6.16853579	2052
chr1.fa	167778625	167883453	ADCY10	0.211150134	76
chr1.fa	167887310	167906278	BRP44	284.1342829	13659
chr1.fa	167905797	168045083	DCAF6	49.02237413	11552
chr1.fa	168053997	168105624	GPR161	0	0
chr1.fa	168148171	168171351	TIPRL	6.756051247	1444
chr1.fa	168195255	168212088	SFT2D2	16.69682484	949
chr1.fa	168214819	168216668	ANKRD36BP1	6.187060373	760
chr1.fa	168250278	168283664	TBX19	0	0
chr1.fa	168344762	168344859	MIR557	0	0
chr1.fa	168510003	168513235	XCL2	0	0
chr1.fa	168545711	168551315	XCL1	0	0
chr1.fa	168664695	168698442	DPT	0	0
chr1.fa	168756179	168762126	MGC4473	0	0
chr1.fa	169075947	169101768	ATP1B1	53.01223814	7068

chr1.fa	169101773	169337186	NME7	4.948925388	532
chr1.fa	169337194	169365780	BLZF1	11.36701594	1748
chr1.fa	169364114	169396670	C1orf114	3.066948162	380
chr1.fa	169433149	169455208	SLC19A2	4.721961585	1140
chr1.fa	169481192	169555769	F5	0	0
chr1.fa	169558088	169599377	SELP	0	0
chr1.fa	169659806	169680843	SELL	0	0
chr1.fa	169691781	169703220	SELE	0	0
chr1.fa	169761673	169764031	C1orf156	3.175083537	304
chr1.fa	169764550	169821077	C1orf112	1.867820227	380
chr1.fa	169821804	169863076	SCYL3	14.34028693	3026
chr1.fa	169890470	170043879	KIFAP3	14.8948196	2964
chr1.fa	170115188	170136923	METTL11B	0	0
chr1.fa	170240546	170253349	LOC284688	0.663571676	76
chr1.fa	170501263	170522974	GORAB	2.921613013	684
chr1.fa	170633313	170708541	PRRX1	0	0
chr1.fa	170904612	171033906	C1orf129	0	0
chr1.fa	171060018	171086959	FMO3	0	0
chr1.fa	171070869	171070947	MIR1295	0	0
chr1.fa	171106879	171130702	FMO6P	0	0
chr1.fa	171154388	171181822	FMO2	0.441879096	152
chr1.fa	171217663	171255113	FMO1	6.460561545	912
chr1.fa	171283486	171311223	FMO4	5.886299448	836
chr1.fa	171308035	171310463	TOP1P1	0	0
chr1.fa	171454666	171562650	PRRC2C	40.9715599	28249
chr1.fa	171604557	171621773	MYOC	0	0
chr1.fa	171669296	171711379	VAMP4	4.154476775	787
chr1.fa	171750761	171766856	METTL13	14.22763337	2784
chr1.fa	171810621	172381857	DNM3	0.42757149	217
chr1.fa	172107938	172108047	MIR214	0	0
chr1.fa	172113675	172113784	MIR199A2	0	0
chr1.fa	172389828	172437969	C1orf105	0	0
chr1.fa	172410597	172413230	PIGC	3.874800742	380
chr1.fa	172502260	172580973	C1orf9	3.23848882	1216
chr1.fa	172628185	172636012	FASLG	0	0
chr1.fa	173010360	173020103	TNFSF18	0	0
chr1.fa	173152870	173176471	TNFSF4	0.655438932	152
chr1.fa	173331510	173332878	LOC646870	0	0
chr1.fa	173446486	173457946	PRDX6	238.5172694	26448
chr1.fa	173469604	173572233	SLC9A11	0	0
chr1.fa	173577475	173639001	ANKRD45	0.428475129	76
chr1.fa	173604661	173606272	LOC730159	0	0
chr1.fa	173684080	173755840	KLHL20	9.912911413	2280

chr1.fa	173768688	173793777	CENPL	0	0
chr1.fa	173793797	173827682	DARS2	9.819987282	2183
chr1.fa	173832386	173833038	LOC100506046	0	0
chr1.fa	173833080	173837125	GAS5	11.31460492	444
chr1.fa	173833313	173833355	SNORD81	0	0
chr1.fa	173833507	173833583	SNORD47	0	0
chr1.fa	173833971	173834041	SNORD80	0	0
chr1.fa	173834488	173834568	SNORD79	0	0
chr1.fa	173834771	173834824	SNORD78	0	0
chr1.fa	173835106	173835166	SNORD44	0	0
chr1.fa	173835449	173835509	SNORD77	0	0
chr1.fa	173835773	173835853	SNORD76	0	0
chr1.fa	173836017	173836076	SNORD75	0	0
chr1.fa	173836812	173836883	SNORD74	0	0
chr1.fa	173837493	173855774	ZBTB37	3.483525387	532
chr1.fa	173872942	173886516	SERPINC1	0.715832087	76
chr1.fa	173900352	173962210	RC3H1	9.992883397	7296
chr1.fa	174128634	174927327	RABGAP1L	9.609740786	2112
chr1.fa	174417212	174418683	GPR52	0	0
chr1.fa	174968571	174981163	CACYBP	11.39427569	2508
chr1.fa	174982094	174992591	MRPS14	17.39684326	2584
chr1.fa	175036994	175117202	TNN	0	0
chr1.fa	175126123	175162229	KIAA0040	8.789990303	2812
chr1.fa	175291935	175712752	TNR	0	0
chr1.fa	175913967	176176370	RFWD2	24.75727788	4560
chr1.fa	175937533	175937676	SCARNA3	0	0
chr1.fa	176432307	176811970	PAPPA2	0.826979589	380
chr1.fa	176830203	177134024	ASTN1	0	0
chr1.fa	176998499	176998581	MIR488	0	0
chr1.fa	177140633	177251558	FAM5B	0	0
chr1.fa	177898242	177939050	SEC16B	1.137831141	304
chr1.fa	177975275	178007142	LOC730102	8.08229096	1596
chr1.fa	178060643	178062863	LOC100302401	0	0
chr1.fa	178063129	178448648	RASAL2	10.83522484	7752
chr1.fa	178482212	178492635	C1orf49	0	0
chr1.fa	178511931	178518024	C1orf220	0	0
chr1.fa	178694300	178889237	RALGPS2	8.634112708	3344
chr1.fa	178818670	178840215	ANGPTL1	0	0
chr1.fa	178995074	179045702	FAM20B	8.837280704	3489
chr1.fa	179051112	179065129	TOR3A	3.236380331	456
chr1.fa	179068462	179198819	ABL2	9.739412873	7980
chr1.fa	179263017	179324453	SOAT1	7.745535112	1745
chr1.fa	179334855	179523870	C1orf125	0.315972169	76

chr1.fa	179519674	179545084	NPHS2	1.236779528	152
chr1.fa	179560748	179660407	TDRD5	5.336134371	1520
chr1.fa	179712298	179785333	FAM163A	0	0
chr1.fa	179809102	179846941	TOR1AIP2	7.844634105	7676
chr1.fa	179851420	179889211	TOR1AIP1	20.3616803	5147
chr1.fa	179923908	180084015	CEP350	24.83845471	22192
chr1.fa	180123968	180167143	QSOX1	10.78507291	2356
chr1.fa	180167170	180169859	FLJ23867	7.233624053	1292
chr1.fa	180199433	180244188	LHX4	0	0
chr1.fa	180238798	180243247	LOC100527964	0	0
chr1.fa	180257352	180472022	ACBD6	14.65053606	1572
chr1.fa	180407449	180407525	MIR3121	0	0
chr1.fa	180601146	180859415	XPR1	5.927866807	3344
chr1.fa	180882313	180915239	KIAA1614	0.275459055	76
chr1.fa	180942176	180992046	STX6	11.61009462	3496
chr1.fa	181002561	181031074	MR1	1.164940288	608
chr1.fa	181057638	181059979	IER5	2.932456672	456
chr1.fa	181452716	181770715	CACNA1E	0.23539776	152
chr1.fa	182023705	182030847	ZNF648	0	0
chr1.fa	182350839	182361341	GLUL	66.59542684	21888
chr1.fa	182367252	182369751	TEDDM1	0	0
chr1.fa	182376756	182383948	NCRNA00272	0	0
chr1.fa	182419256	182529732	RGSL1	0	0
chr1.fa	182542769	182558394	RNASEL	7.826862553	2204
chr1.fa	182567758	182573548	RGS16	3.765159303	608
chr1.fa	182584275	182585764	LOC284648	0	0
chr1.fa	182615792	182642067	RGS8	0	0
chr1.fa	182761394	182798588	NPL	5.647738955	582
chr1.fa	182808439	182857117	DHX9	36.70247167	11388
chr1.fa	182869000	182922553	C1orf14	0	0
chr1.fa	182992595	183114727	LAMC1	6.819155317	3572
chr1.fa	183155174	183214262	LAMC2	0.203619815	76
chr1.fa	183217379	183387737	NMNAT2	0	0
chr1.fa	183441506	183523328	SMG7	28.25721936	11400
chr1.fa	183524697	183560056	NCF2	0	0
chr1.fa	183595332	183604985	ARPC5	41.58016025	5472
chr1.fa	183605208	183897666	RGL1	3.581871348	1216
chr1.fa	183615411	183622448	APOBEC4	0	0
chr1.fa	183904966	184006863	GLT25D2	0	0
chr1.fa	184020811	184043344	TSEN15	12.13556025	1672
chr1.fa	184356150	184598155	C1orf21	13.7675309	9423
chr1.fa	184659625	184724041	EDEM3	7.736950549	3407
chr1.fa	184760166	184943682	FAM129A	29.58195301	13604

chr1.fa	185014551	185071740	RNF2	7.091301032	1672
chr1.fa	185087218	185126116	C1orf25	9.869536778	2888
chr1.fa	185126192	185260913	C1orf26	3.191499632	836
chr1.fa	185265522	185286461	IVNS1ABP	40.36265834	11248
chr1.fa	185703683	186160085	HMCN1	0.062802857	76
chr1.fa	186029867	186446655	MIR548F1	0	0
chr1.fa	186265405	186283688	PRG4	0	0
chr1.fa	186280786	186344457	TPR	24.21931191	14531
chr1.fa	186344890	186390503	C1orf27	5.582074577	1444
chr1.fa	186369704	186370587	OCLM	0	0
chr1.fa	186412715	186430239	PDC	0	0
chr1.fa	186640944	186649559	PTGS2	0.509501357	152
chr1.fa	186798032	186958113	PLA2G4A	5.081910815	988
chr1.fa	190066797	190446759	FAM5C	1.190693978	228
chr1.fa	190594020	190770788	LOC440704	0	0
chr1.fa	192127592	192154945	RGS18	0	0
chr1.fa	192286122	192336414	RGS21	0	0
chr1.fa	192544857	192549159	RGS1	0	0
chr1.fa	192605268	192629440	RGS13	0	0
chr1.fa	192778169	192781407	RGS2	7.630773057	684
chr1.fa	192981496	193028523	UCHL5	2.86197289	1064
chr1.fa	193028552	193060906	TROVE2	23.86553755	3876
chr1.fa	193065600	193075244	GLRX2	15.81833787	1357
chr1.fa	193091088	193223942	CDC73	15.89499651	6268
chr1.fa	193105633	193105713	MIR1278	0	0
chr1.fa	193147860	193155743	B3GALT2	0	0
chr1.fa	196194913	196577499	KCNT2	0	0
chr1.fa	196621008	196716634	CFH	0.261603268	76
chr1.fa	196743930	196763203	CFHR3	0	0
chr1.fa	196788861	196801319	CFHR1	0	0
chr1.fa	196857212	196887763	CFHR4	0	0
chr1.fa	196912934	196928356	CFHR2	0	0
chr1.fa	196946667	196978803	CFHR5	0	0
chr1.fa	197008321	197036397	F13B	0	0
chr1.fa	197053257	197115824	ASPM	0	0
chr1.fa	197122814	197169672	ZBTB41	4.862627937	2736
chr1.fa	197237334	197447585	CRB1	0	0
chr1.fa	197473879	197744623	DENND1B	6.42547026	4664
chr1.fa	197871682	197876497	C1orf53	1.675797103	76
chr1.fa	197881635	197899273	LHX9	0	0
chr1.fa	198126108	198291548	NEK7	12.9757932	3560
chr1.fa	198492352	198510075	ATP6V1G3	13.50577703	608
chr1.fa	198608137	198726545	PTPRC	0	0

chr1.fa	198828002	198828111	MIR181B1	0	0
chr1.fa	198828173	198828282	MIR181A1	0	0
chr1.fa	199996770	200146550	NR5A2	0	0
chr1.fa	200182656	200183643	FAM58B	0	0
chr1.fa	200375420	200379166	ZNF281	7.801861895	1824
chr1.fa	200520625	200589862	KIF14	0.157233053	76
chr1.fa	200613165	200639126	DDX59	10.85405063	1672
chr1.fa	200708686	200829831	CAMSAP1L1	15.10371064	7372
chr1.fa	200842166	200843251	GPR25	0	0
chr1.fa	200860639	200884864	C1orf106	16.32904408	4712
chr1.fa	200938520	200992828	KIF21B	2.429130176	1596
chr1.fa	201008640	201081694	CACNA1S	0	0
chr1.fa	201103900	201123632	TMEM9	6.693549603	684
chr1.fa	201159953	201198080	IGFN1	0	0
chr1.fa	201252580	201302121	PKP1	27.3175862	9880
chr1.fa	201328142	201346805	TNNT2	0	0
chr1.fa	201349966	201368669	LAD1	40.19006343	7576
chr1.fa	201372895	201390874	TNNI1	0.372600165	152
chr1.fa	201434622	201438299	PHLDA3	15.1003973	1520
chr1.fa	201452658	201476387	CSRP1	65.99721833	10184
chr1.fa	201489032	201489720	RPS10P7	1.661188285	76
chr1.fa	201617450	201796102	NAV1	0.431487256	380
chr1.fa	201777739	201777830	MIR1231	0	0
chr1.fa	201798288	201853422	IPO9	8.910324794	6754
chr1.fa	201857797	201861715	SHISA4	11.0257419	1216
chr1.fa	201865584	201915716	LMOD1	0.288561809	76
chr1.fa	201924619	201939789	TIMM17A	16.55856819	1824
chr1.fa	201951766	201975275	RNPEP	45.06338442	7220
chr1.fa	201979690	201986315	ELF3	155.6346671	32376
chr1.fa	202092029	202098634	GPR37L1	0	0
chr1.fa	202101977	202113866	ARL8A	22.92816349	3570
chr1.fa	202116141	202130716	PTPN7	0	0
chr1.fa	202137179	202158577	PTPRVP	0	0
chr1.fa	202163118	202288889	LGR6	0	0
chr1.fa	202300785	202311094	UBE2T	1.260575335	76
chr1.fa	202317830	202557697	PPP1R12B	8.315881443	6500
chr1.fa	202559725	202679551	SYT2	0	0
chr1.fa	202696532	202777549	KDM5B	37.11950072	15732
chr1.fa	202830882	202844369	LOC148709	1.972642262	304
chr1.fa	202848086	202858263	RABIF	9.424494949	1520
chr1.fa	202860230	202896371	KLHL12	22.28868883	4940
chr1.fa	202909961	202927700	ADIPOR1	63.20226528	9652
chr1.fa	202931001	202936404	CYB5R1	6.966598956	760

chr1.fa	202955580	202976393	LOC401980	0	0
chr1.fa	202976534	202976535	TMEM183A	0	0
chr1.fa	203020311	203047864	PPFIA4	0	0
chr1.fa	203052257	203055377	MYOG	0	0
chr1.fa	203096836	203136533	ADORA1	0	0
chr1.fa	203136939	203144942	MYBPH	0	0
chr1.fa	203148059	203155922	CHI3L1	0	0
chr1.fa	203185207	203198860	CHIT1	0	0
chr1.fa	203267886	203274453	LOC730227	0	0
chr1.fa	203274664	203278729	BTG2	52.35393769	9424
chr1.fa	203309752	203320289	FMOD	8.433203807	1672
chr1.fa	203444883	203460479	PRELP	0	0
chr1.fa	203463271	203478077	OPTC	0	0
chr1.fa	203595928	203713209	ATP2B4	1.028039096	608
chr1.fa	203698709	203698833	SNORA77	0	0
chr1.fa	203734284	203745480	LAX1	0	0
chr1.fa	203764751	203823256	ZC3H11A	30.64448096	9722
chr1.fa	203766651	203769590	ZBED6	18.29822239	3572
chr1.fa	203830740	203840280	SNRPE	10.3097592	1059
chr1.fa	204001575	204010392	C1orf157	0	0
chr1.fa	204042246	204096871	SOX13	11.80302138	3192
chr1.fa	204100190	204121307	ETNK2	15.34919902	2660
chr1.fa	204123944	204135465	REN	0	0
chr1.fa	204159469	204165619	KISS1	0	0
chr1.fa	204167288	204183220	GOLT1A	15.55522854	912
chr1.fa	204187981	204329044	PLEKHA6	24.59688209	12084
chr1.fa	204337558	204338847	LOC127841	0.887222138	76
chr1.fa	204372492	204380944	PPP1R15B	9.11349279	3192
chr1.fa	204391758	204459474	PIK3C2B	34.27951636	17312
chr1.fa	204485511	204527248	MDM4	7.454864814	4986
chr1.fa	204586303	204654481	LRRN2	0.71402481	152
chr1.fa	204797782	204991950	NFASC	0	0
chr1.fa	205012340	205047171	CNTN2	0	0
chr1.fa	205052257	205053588	TMEM81	0	0
chr1.fa	205055270	205091150	RBBP5	13.4934273	3952
chr1.fa	205111631	205180727	DSTYK	5.933589849	3116
chr1.fa	205197038	205242471	TMCC2	0.285850894	76
chr1.fa	205271191	205290883	NUAK2	8.077170344	1824
chr1.fa	205305648	205326039	KLHDC8A	0	0
chr1.fa	205350506	205391214	LEMD1	0	0
chr1.fa	205417430	205417526	MIR135B	0	0
chr1.fa	205473684	205501921	CDK18	10.33174773	2128
chr1.fa	205523401	205525763	LOC284578	3.390751862	532

chr1.fa	205538112	205572046	MFSD4	1.959991327	532
chr1.fa	205585235	205602000	ELK4	258.2595057	63156
chr1.fa	205626981	205649630	SLC45A3	116.7622588	26220
chr1.fa	205681947	205719372	NUCKS1	65.16105175	27967
chr1.fa	205737114	205744610	RAB7L1	34.25526873	7524
chr1.fa	205758221	205782161	SLC41A1	6.838432933	2204
chr1.fa	205797150	205819276	PM20D1	2.62160512	380
chr1.fa	205882177	205912588	SLC26A9	2.624165428	836
chr1.fa	206138911	206155074	FAM72A	0	0
chr1.fa	206224283	206231482	AVPR1B	0.645649517	76
chr1.fa	206238872	206288647	C1orf186	1.374885571	152
chr1.fa	206317459	206332104	CTSE	1.027436671	152
chr1.fa	206516200	206637783	SRGAP2	12.4060493	4921
chr1.fa	206643586	206670223	IKBKE	2.965288861	684
chr1.fa	206680879	206762616	RASSF5	13.5146628	3724
chr1.fa	206764974	206785904	LGTN	18.5583196	2584
chr1.fa	206808881	206822542	DYRK3	1.518714657	228
chr1.fa	206858289	206907626	MAPKAPK2	20.70054464	4933
chr1.fa	206940948	206945839	IL10	0	0
chr1.fa	206972215	207016326	IL19	0	0
chr1.fa	207039154	207042568	IL20	0	0
chr1.fa	207070788	207076630	IL24	0	0
chr1.fa	207077485	207095378	FAIM3	1.026081213	152
chr1.fa	207101867	207119811	PIGR	8.032289645	2280
chr1.fa	207131312	207143970	FCAMR	0.847913875	152
chr1.fa	207191866	207206101	C1orf116	162.4753591	59356
chr1.fa	207217194	207224422	YOD1	3.654011801	1520
chr1.fa	207226620	207254368	PFKFB2	7.228051618	4388
chr1.fa	207262212	207273337	C4BPB	0	0
chr1.fa	207277607	207318317	C4BPA	0	0
chr1.fa	207494817	207534311	CD55	7.463148164	1444
chr1.fa	207627645	207663240	CR2	0	0
chr1.fa	207669473	207815110	CR1	0	0
chr1.fa	207818458	207897036	CR1L	0	0
chr1.fa	207925383	207968861	CD46	7.585741751	1709
chr1.fa	207975197	207975284	MIR29C	0	0
chr1.fa	207975788	207975868	MIR29B2	0	0
chr1.fa	207991724	207995941	LOC148696	0.271392683	76
chr1.fa	208059883	208084683	CD34	0	0
chr1.fa	208195588	208417665	PLXNA2	0.500163762	380
chr1.fa	209602168	209605892	LOC642587	0	0
chr1.fa	209605478	209605548	MIR205	0	0
chr1.fa	209757045	209787284	CAMK1G	0	0

chr1.fa	209788218	209825820	LAMB3	0.553478418	152
chr1.fa	209796789	209796849	MIR4260	0	0
chr1.fa	209848670	209849735	G0S2	5.942927444	380
chr1.fa	209859550	209908295	HSD11B1	0	0
chr1.fa	209929394	209955661	TRAF3IP3	1.020207565	152
chr1.fa	209955669	209957890	C1orf74	5.89247431	608
chr1.fa	209961262	209979479	IRF6	64.8786648	9348
chr1.fa	210001312	210030910	C1orf107	5.799851391	3268
chr1.fa	210111538	210337633	SYT14	0	0
chr1.fa	210404804	210407466	C1orf133	1.430760535	76
chr1.fa	210406195	210416440	SERTAD4	0	0
chr1.fa	210501596	210849638	HHAT	1.733328737	456
chr1.fa	210851657	211307457	KCNH1	0	0
chr1.fa	211432708	211489725	RCOR3	11.32559918	3484
chr1.fa	211499957	211548286	TRAF5	1.056654307	289
chr1.fa	211556097	211605877	C1orf97	22.65059594	1140
chr1.fa	211649864	211666259	RD3	0	0
chr1.fa	211748381	211752099	SLC30A1	4.537619386	608
chr1.fa	211836122	211848967	NEK2	0	0
chr1.fa	211916799	212004114	LPGAT1	3.382920331	1748
chr1.fa	212113741	212208918	INTS7	2.550970731	760
chr1.fa	212209003	212278187	DTL	0	0
chr1.fa	212250955	212251027	MIR3122	0	0
chr1.fa	212458879	212535205	PPP2R5A	14.41227678	3268
chr1.fa	212526160	212526292	SNORA16B	0	0
chr1.fa	212537816	212588243	TMEM206	1.6190185	228
chr1.fa	212606229	212619721	NENF	24.12247202	1520
chr1.fa	212738697	212794116	ATF3	3.219060598	532
chr1.fa	212797789	212800120	FAM71A	0	0
chr1.fa	212859759	212873327	BATF3	0	0
chr1.fa	212899495	212965139	NSL1	1.385277411	1216
chr1.fa	212965170	212990167	TATDN3	6.58074543	1140
chr1.fa	213003485	213020991	C1orf227	0	0
chr1.fa	213029946	213031480	NCRNA00292	1.21509221	76
chr1.fa	213031597	213070197	FLVCR1	1.667965571	380
chr1.fa	213123887	213164927	VASH2	0	0
chr1.fa	213165524	213189168	ANGEL2	7.632429727	2356
chr1.fa	213224588	213446808	RPS6KC1	15.48474476	4309
chr1.fa	214161860	214209762	PROX1	0	0
chr1.fa	214454565	214510477	SMYD2	30.16600452	3375
chr1.fa	214522039	214725024	PTPN14	10.46277528	9339
chr1.fa	214776532	214837914	CENPF	0.222295005	152
chr1.fa	215178885	215410436	KCNK2	0	0

chr1.fa	215740735	215795149	KCTD3	50.53039573	13189
chr1.fa	215796236	216596738	USH2A	0	0
chr1.fa	216676588	217311097	ESRRG	3.952061811	1596
chr1.fa	217603834	217804409	GPATCH2	11.26972422	1748
chr1.fa	217804695	218040484	SPATA17	2.780494842	228
chr1.fa	218458629	218511325	RRP15	2.651274575	1368
chr1.fa	218518676	218617961	TGFB2	0	0
chr1.fa	219347192	219386207	LYPLAL1	15.72932951	1976
chr1.fa	220087606	220101993	SLC30A10	0	0
chr1.fa	220141942	220220000	EPRS	29.21432285	9728
chr1.fa	220230824	220263191	BPNT1	11.16234188	1824
chr1.fa	220267455	220321383	IARS2	16.77077257	3952
chr1.fa	220291195	220291304	MIR215	0	0
chr1.fa	220291499	220291583	MIR194-1	0	0
chr1.fa	220321610	220445843	RAB3GAP2	15.91141261	7743
chr1.fa	220373884	220373887	MIR664	0	0
chr1.fa	220373962	220374018	SNORA36B	0	0
chr1.fa	220439521	220441057	AURKAPS1	0	0
chr1.fa	220701568	220837799	MARK1	10.33912745	3622
chr1.fa	220863628	220872499	C1orf115	51.60753251	10184
chr1.fa	220921676	220957596	MOSC2	4.365928122	456
chr1.fa	220960039	220987741	MOSC1	2.551573157	380
chr1.fa	221052743	221058400	HLX	0	0
chr1.fa	221503270	221509638	LOC400804	0	0
chr1.fa	221874766	221915461	DUSP10	3.182463249	684
chr1.fa	222695602	222721444	HHIPL2	0	0
chr1.fa	222731665	222763255	TAF1A	1.827457719	228
chr1.fa	222791444	222841351	MIA3	6.90454913	3724
chr1.fa	222841355	222885864	AIDA	6.092328965	1216
chr1.fa	222885906	222906106	BROX	33.36458265	3724
chr1.fa	222910558	222924002	FAM177B	0	0
chr1.fa	223101783	223179335	DISP1	4.343939591	1368
chr1.fa	223282748	223316624	TLR5	1.072919795	304
chr1.fa	223394161	223537544	SUSD4	3.912602942	836
chr1.fa	223566715	223568812	C1orf65	0	0
chr1.fa	223714972	223853436	CAPN8	0.544743248	76
chr1.fa	223889295	223963720	CAPN2	35.6372328	8819
chr1.fa	223967595	224033674	TP53BP2	16.96957298	5396
chr1.fa	224301791	224349749	FBXO28	11.7632613	4256
chr1.fa	224370928	224381142	DEGS1	4.47767805	608
chr1.fa	224415036	224517872	NVL	18.09761471	3492
chr1.fa	224444706	224444843	MIR320B2	0	0
chr1.fa	224544595	224563695	CNIH4	12.86072994	532

chr1.fa	224572845	224622001	WDR26	28.78885985	13136
chr1.fa	224804179	224928249	CNIH3	1.930171265	304
chr1.fa	225117356	225586996	DNAH14	1.468110916	1368
chr1.fa	225589204	225616519	LBR	3.811395459	988
chr1.fa	225674534	225840845	ENAH	12.76012488	11160
chr1.fa	225965515	225978168	SRP9	40.95198107	4405
chr1.fa	225997797	226033232	EPHX1	3.050682674	380
chr1.fa	226033263	226070420	TMEM63A	8.022349624	2356
chr1.fa	226073982	226076836	LEFTY1	1.416603536	152
chr1.fa	226107580	226111965	PYCR2	31.77583603	3572
chr1.fa	226124298	226129083	LEFTY2	0	0
chr1.fa	226170403	226187066	C1orf55	10.56383215	2812
chr1.fa	226250421	226250427	H3F3A	0	0
chr1.fa	226332380	226374423	ACBD3	25.04327938	5928
chr1.fa	226411383	226413513	MIXL1	0	0
chr1.fa	226418861	226497198	LIN9	1.031804255	228
chr1.fa	226548392	226595801	PARP1	23.84008507	6308
chr1.fa	226736501	226796915	C1orf95	0.148497883	76
chr1.fa	226819391	226926876	ITPKB	14.67448247	6004
chr1.fa	227058273	227083804	PSEN2	2.988482242	456
chr1.fa	227127938	227175246	ADCK3	10.56925398	2052
chr1.fa	227177566	227505826	CDC42BPA	13.50954219	9424
chr1.fa	227751220	227850164	ZNF678	0.527724728	304
chr1.fa	227884733	227885408	ZNF847P	0	0
chr1.fa	227916240	227922055	LOC100130093	0.826377164	76
chr1.fa	227918890	227922517	JMJD4	2.637418789	228
chr1.fa	227922697	227968932	SNAP47	16.87529339	2204
chr1.fa	228003418	228034171	PRSS38	0	0
chr1.fa	228109165	228135676	WNT9A	0	0
chr1.fa	228194752	228248961	WNT3A	0	0
chr1.fa	228270361	228286913	ARF1	231.3051821	32452
chr1.fa	228284964	228285042	MIR3620	0	0
chr1.fa	228288428	228291022	C1orf35	15.01394924	1286
chr1.fa	228294380	228297013	MRPL55	13.94313793	836
chr1.fa	228327929	228336655	GUK1	93.80382342	7499
chr1.fa	228337553	228347527	GJC2	0	0
chr1.fa	228353429	228369958	C1orf69	3.910042633	2052
chr1.fa	228395861	228566575	OBSCN	0.845353567	1444
chr1.fa	228581377	228594517	TRIM11	16.07361567	2872
chr1.fa	228595636	228604583	TRIM17	4.404031534	760
chr1.fa	228612546	228613026	HIST3H3	0	0
chr1.fa	228645065	228645560	HIST3H2A	18.4614797	608
chr1.fa	228645808	228646259	HIST3H2BB	10.12933277	304

chr1.fa	228675068	228683889	RNF187	52.24354321	10868
chr1.fa	228780657	228788159	DUSP5P	0.462963988	76
chr1.fa	228870869	228882411	RHOU	51.64247318	14820
chr1.fa	229406879	229440128	RAB4A	93.74282784	9044
chr1.fa	229440519	229441250	SPHAR	7.818428596	380
chr1.fa	229456752	229478688	C1orf96	0.670499569	228
chr1.fa	229566993	229569843	ACTA1	0	0
chr1.fa	229577044	229644088	NUP133	16.44034219	4552
chr1.fa	229652329	229694442	ABCB10	2.967547956	760
chr1.fa	229728866	229761794	TAF5L	4.305083147	1748
chr1.fa	229761981	229795946	URB2	5.70978878	2128
chr1.fa	230202956	230417875	GALNT2	3.593769252	1064
chr1.fa	230457392	230513391	PGBD5	0	0
chr1.fa	230778202	230829731	COG2	26.66651486	5241
chr1.fa	230838272	230850336	AGT	0.443535766	76
chr1.fa	230883130	230937518	CAPN9	1.428200227	224
chr1.fa	230972865	231005335	C1orf198	21.46110682	5888
chr1.fa	231041987	231114618	TTC13	1.045057616	228
chr1.fa	231114823	231136479	ARV1	6.336913714	608
chr1.fa	231154704	231175995	FAM89A	0	0
chr1.fa	231298674	231357314	TRIM67	0.134340884	76
chr1.fa	231359509	231376918	C1orf131	7.208924608	684
chr1.fa	231376925	231413719	GNPAT	30.81963617	5472
chr1.fa	231468482	231473578	EXOC8	8.533507651	2888
chr1.fa	231473682	231489989	C1orf124	2.86152107	532
chr1.fa	231499497	231560790	EGLN1	7.726558709	3641
chr1.fa	231611510	231612271	SNRPD2P2	0	0
chr1.fa	231700274	231702269	TSNAX	12.51779923	1659
chr1.fa	231727038	231747836	LOC100287814	0	0
chr1.fa	231752390	231753805	TSNAX-DISC1	0	0
chr1.fa	231762561	232095249	DISC1	0	0
chr1.fa	231950372	231954074	DISC2	0	0
chr1.fa	232533712	232651243	SIPA1L2	19.93531366	8588
chr1.fa	232940638	232946092	KIAA1383	0.209794676	76
chr1.fa	233086370	233114219	NTPCR	31.72417804	1900
chr1.fa	233119882	233431459	PCNXL2	5.87666064	2660
chr1.fa	233463514	233520894	KIAA1804	3.940766333	1520
chr1.fa	233749750	233808258	KCNK1	1.059365222	152
chr1.fa	234040679	234460262	SLC35F3	0	0
chr1.fa	234509429	234519791	C1orf31	12.49957585	532
chr1.fa	234527059	234614849	TARBP1	8.863185	3019
chr1.fa	234740015	234745271	IRF2BP2	19.14628688	5928
chr1.fa	234765057	234770526	NCRNA00184	0	0

chr1.fa	235272658	235292256	TOMM20	70.59146571	15866
chr1.fa	235291118	235291252	SNORA14B	0	0
chr1.fa	235294498	235324571	RBM34	16.89321555	2323
chr1.fa	235330210	235491532	ARID4B	16.79080322	6764
chr1.fa	235491753	235507844	GGPS1	24.28768721	4788
chr1.fa	235530728	235606226	TBCE	14.24917008	1440
chr1.fa	235610533	235667781	B3GALNT2	6.869759058	1976
chr1.fa	235710985	235814054	GNG4	25.64163849	9044
chr1.fa	235824345	236030220	LYST	4.242430896	3796
chr1.fa	236016300	236016360	MIR1537	0	0
chr1.fa	236139132	236228481	NID1	0.194583433	76
chr1.fa	236305832	236372209	GPR137B	14.39691493	1952
chr1.fa	236378422	236445339	ERO1LB	0.226511984	76
chr1.fa	236557680	236648008	EDARADD	6.828944731	1368
chr1.fa	236681565	236712304	LGALS8	26.75552322	3935
chr1.fa	236686369	236687808	LOC100287902	1.181205777	76
chr1.fa	236712997	236767841	HEATR1	11.89850582	6156
chr1.fa	236849770	236927558	ACTN2	0.252717492	76
chr1.fa	236958581	237067281	MTR	15.36154874	10769
chr1.fa	237205702	237997288	RYR2	0.13991332	152
chr1.fa	238025475	238091619	LOC100130331	0	0
chr1.fa	238041164	238054222	ZP4	0	0
chr1.fa	238643684	238649317	LOC339535	0.747610031	76
chr1.fa	239792373	240072717	CHRM3	0	0
chr1.fa	240170824	240176560	RPS7P5	0	0
chr1.fa	240255185	240638489	FMN2	0.178016732	76
chr1.fa	240652873	240775462	GREM2	0	0
chr1.fa	240938817	241520478	RGS7	0	0
chr1.fa	241295572	241295646	MIR3123	0	0
chr1.fa	241660857	241683085	FH	43.52825367	5396
chr1.fa	241695680	241758944	KMO	1.647934924	380
chr1.fa	241761048	241803701	OPN3	2.100205859	152
chr1.fa	241792167	241799232	CHML	1.133915376	532
chr1.fa	241815580	241965434	WDR64	0	0
chr1.fa	242011493	242053241	EXO1	0.330580987	76
chr1.fa	242158792	242162385	MAP1LC3C	0	0
chr1.fa	242251689	242687998	PLD5	0	0
chr1.fa	243219616	243265046	LOC731275	0.597154265	152
chr1.fa	243287730	243418708	CEP170	4.442586765	2128
chr1.fa	243419307	243663393	SDCCAG8	19.53334525	3354
chr1.fa	243651535	244006553	AKT3	44.26848399	11243
chr1.fa	244080704	244210619	LOC339529	0	0
chr1.fa	244214561	244220778	ZNF238	12.79627041	3724

chr1.fa	244515937	244552393	C1orf100	0	0
chr1.fa	244571794	244615436	ADSS	20.13863226	3720
chr1.fa	244624673	244803662	C1orf101	0.358292559	76
chr1.fa	244816352	244872334	PPPDE1	8.111809809	2204
chr1.fa	244998639	244999058	FAM36A	4.66156843	130
chr1.fa	245003940	245010243	NCRNA00201	0	0
chr1.fa	245013602	245027827	HNRNPU	55.50838815	25232
chr1.fa	245133171	245288530	EFCAB2	0.694295376	228
chr1.fa	245318287	245866428	KIF26B	0.314164892	152
chr1.fa	245912642	246670644	SMYD3	5.879823374	684
chr1.fa	246703863	246729565	TFB2M	10.25403485	1216
chr1.fa	246729639	246831884	CNST	5.917023148	2186
chr1.fa	246887378	246931440	SCCPDH	83.33547571	13695
chr1.fa	246952919	246954788	LOC149134	0	0
chr1.fa	247002402	247094726	AHCTF1	9.017857744	5168
chr1.fa	247148625	247171355	ZNF695	0	0
chr1.fa	247199700	247242115	ZNF670	1.877910854	304
chr1.fa	247263264	247267674	ZNF669	2.312410238	304
chr1.fa	247273462	247275719	C1orf229	0	0
chr1.fa	247319203	247335318	ZNF124	36.9228088	4104
chr1.fa	247365269	247365362	MIR3916	0	0
chr1.fa	247419374	247420447	VN1R5	0	0
chr1.fa	247463622	247495045	ZNF496	6.599872439	1064
chr1.fa	247579458	247612406	NLRP3	0	0
chr1.fa	247614331	247615284	OR2B11	0	0
chr1.fa	247654370	247655711	OR2W5	0	0
chr1.fa	247687981	247691724	LOC148824	0.674114122	76
chr1.fa	247693434	247697141	OR2C3	0	0
chr1.fa	247712451	247739848	C1orf150	0.437662117	76
chr1.fa	247751662	247752615	OR2G2	0	0
chr1.fa	247768888	247769817	OR2G3	0	0
chr1.fa	247835420	247836343	OR13G1	0	0
chr1.fa	247875131	247876057	OR6F1	0	0
chr1.fa	247920764	247921708	OR1C1	0	0
chr1.fa	247978102	247979031	OR14A16	0	0
chr1.fa	248004230	248005198	OR11L1	0	0
chr1.fa	248020501	248043438	TRIM58	1.288136301	441
chr1.fa	248058889	248059833	OR2W3	1.211176445	76
chr1.fa	248084320	248085258	OR2T8	0	0
chr1.fa	248100493	248264224	OR2L13	0	0
chr1.fa	248112160	248113098	OR2L8	0	0
chr1.fa	248128634	248129641	OR2AK2	0	0
chr1.fa	248153569	248154493	OR2L1P	0	0

chr1.fa	248201474	248202607	OR2L2	0	0
chr1.fa	248223984	248224922	OR2L3	0	0
chr1.fa	248285438	248286082	OR2M1P	0	0
chr1.fa	248308450	248309388	OR2M5	0	0
chr1.fa	248343288	248344331	OR2M2	0	0
chr1.fa	248366370	248367308	OR2M3	0	0
chr1.fa	248402231	248403166	OR2M4	0	0
chr1.fa	248436154	248437116	OR2T33	0	0
chr1.fa	248457918	248458880	OR2T12	0	0
chr1.fa	248486932	248487870	OR2M7	0	0
chr1.fa	248512077	248513015	OR14C36	0	0
chr1.fa	248524883	248525929	OR2T4	0	0
chr1.fa	248550910	248551836	OR2T6	0	0
chr1.fa	248569296	248570405	OR2T1	0	0
chr1.fa	248616099	248617073	OR2T2	0	0
chr1.fa	248636652	248637608	OR2T3	0	0
chr1.fa	248651890	248652837	OR2T5	0	0
chr1.fa	248684948	248685898	OR2G6	0	0
chr1.fa	248721845	248722774	OR2T29	0	0
chr1.fa	248737102	248738058	OR2T34	0	0
chr1.fa	248756131	248757069	OR2T10	0	0
chr1.fa	248789479	248790429	OR2T11	0	0
chr1.fa	248801588	248802559	OR2T35	0	0
chr1.fa	248813232	248814185	OR2T27	0	0
chr1.fa	248844670	248845605	OR14I1	0	0
chr1.fa	248902717	248903151	LOC646627	0	0
chr1.fa	249104651	249120154	SH3BP5L	14.02883296	2964
chr1.fa	249120576	249120642	MIR3124	0	0
chr1.fa	249132530	249143714	ZNF672	17.54338326	3420
chr1.fa	249144203	249153315	ZNF692	1.921285489	304
chr1.fa	249200442	249213345	PGBD2	3.80266029	684
chr10.fa	92828	95504	TUBB8	0	0
chr10.fa	180424	300577	ZMYND11	41.76314699	12692
chr10.fa	320130	735608	DIP2C	5.653461997	2964
chr10.fa	695888	711109	C10orf108	0	0
chr10.fa	855484	931702	LARP4B	36.48740578	13664
chr10.fa	1034349	1063708	GTPBP4	18.64476766	3116
chr10.fa	1064847	1071799	IDI2	0.904090052	76
chr10.fa	1068577	1090141	IDI2-AS1	0	0
chr10.fa	1085964	1095061	IDI1	63.2500075	8244
chr10.fa	1102776	1178237	WDR37	10.60223678	3246
chr10.fa	1205708	1210612	NCRNA00200	0	0
chr10.fa	1228073	1779718	ADARB2	0	0

chr10.fa	1568825	1599179	NCRNA00168	0	0
chr10.fa	3109752	3178996	PFKP	9.625855668	1672
chr10.fa	3179920	3215003	PITRM1	21.19649142	4864
chr10.fa	3818188	3827473	KLF6	38.05536872	11780
chr10.fa	4692377	4720262	LOC100216001	0	0
chr10.fa	4698348	4704606	LOC338588	0	0
chr10.fa	4868402	4890251	AKR1E2	4.273606416	456
chr10.fa	4913859	4958465	tAKR	0	0
chr10.fa	5005454	5020158	AKR1C1	3.320117474	304
chr10.fa	5031965	5060207	AKR1C2	10.16246617	1444
chr10.fa	5136568	5149878	AKR1C3	25.24870647	2052
chr10.fa	5196655	5227150	AKR1CL1	0	0
chr10.fa	5238798	5260912	AKR1C4	0	0
chr10.fa	5406976	5416169	UCN3	0	0
chr10.fa	5435061	5446793	TUBAL3	2.54645254	304
chr10.fa	5454518	5500426	NET1	32.08894668	7828
chr10.fa	5540658	5541533	CALML5	0	0
chr10.fa	5566924	5568225	CALML3	4.395597577	380
chr10.fa	5680820	5708558	ASB13	9.375397271	1748
chr10.fa	5726801	5805703	C10orf18	14.59616716	8360
chr10.fa	5807186	5855512	GDI2	95.35280996	15271
chr10.fa	5903689	5931860	ANKRD16	2.603381749	456
chr10.fa	5932214	5979556	FBXO18	21.69876367	5472
chr10.fa	5994334	6020142	IL15RA	0	0
chr10.fa	6052657	6104333	IL2RA	0	0
chr10.fa	6130949	6159422	RBM17	10.11397092	2660
chr10.fa	6186843	6277507	PFKFB3	23.67953868	7363
chr10.fa	6194159	6194240	MIR3155	0	0
chr10.fa	6469105	6622238	PRKCQ	0.351063453	76
chr10.fa	6622387	6627323	LOC439949	0	0
chr10.fa	7200586	7453448	SFMBT2	7.354711577	3938
chr10.fa	7601232	7708961	ITIH5	0.292176362	152
chr10.fa	7745236	7791483	ITIH2	0	0
chr10.fa	7797367	7829944	KIN	4.804192665	608
chr10.fa	7830093	7849762	ATP5C1	118.3692288	9007
chr10.fa	7860673	8056714	TAF3	9.025538669	1672
chr10.fa	8092413	8095447	FLJ45983	3.045562057	456
chr10.fa	8096667	8117164	GATA3	157.3371215	32072
chr10.fa	10826402	10836877	SFTA1P	0	0
chr10.fa	10976903	10994126	LOC254312	0	0
chr10.fa	11047259	11378672	CELF2	0.951531059	532
chr10.fa	11502509	11653679	USP6NL	11.04095314	3546
chr10.fa	11784356	11806065	ECHDC3	13.84042439	1520

chr10.fa	11865397	11914276	C10orf47	4.121042161	912
chr10.fa	11962021	12085023	UPF2	27.30824861	9572
chr10.fa	12110934	12165224	DHTKD1	28.1966756	9728
chr10.fa	12171640	12211957	SEC61A2	1.329402447	304
chr10.fa	12209573	12237960	NUDT5	26.61440505	1700
chr10.fa	12238144	12292589	CDC123	26.20445451	2375
chr10.fa	12391583	12871733	CAMK1D	1.703358069	304
chr10.fa	12875133	12877545	LOC283070	2.84615922	456
chr10.fa	12938625	13043704	CCDC3	0	0
chr10.fa	13142082	13180276	OPTN	27.0480008	6460
chr10.fa	13203554	13253104	MCM10	0	0
chr10.fa	13263767	13276328	UCMA	0	0
chr10.fa	13319796	13342130	PHYH	20.68217066	2660
chr10.fa	13359438	13390298	SEPHS1	23.5241129	5092
chr10.fa	13480484	13544976	BEND7	6.295195749	1292
chr10.fa	13628939	13672868	PRPF18	11.41249906	1292
chr10.fa	13685706	14372866	FRMD4A	9.757033818	4408
chr10.fa	14478575	14478660	MIR1265	0	0
chr10.fa	14560559	14816896	FAM107B	37.19600876	9348
chr10.fa	14861251	14879983	CDNF	0	0
chr10.fa	14880261	14913740	HSPA14	2.535157062	304
chr10.fa	14920782	14946304	SUV39H2	2.741638398	608
chr10.fa	14948871	14996094	DCLRE1C	4.180381071	1140
chr10.fa	15001438	15014850	MEIG1	1.831373485	76
chr10.fa	15085895	15115851	OLAH	0	0
chr10.fa	15117474	15130775	ACBD7	0.339617369	76
chr10.fa	15137384	15139181	C10orf111	0	0
chr10.fa	15139319	15146256	RPP38	9.850259163	760
chr10.fa	15147771	15210695	NMT2	2.973572211	988
chr10.fa	15196721	15197346	LOC100192204	0	0
chr10.fa	15253644	15413058	FAM171A1	7.823850426	2052
chr10.fa	15559088	15761770	ITGA8	0	0
chr10.fa	15820175	15902519	FAM188A	20.61560264	3251
chr10.fa	16478967	16555736	PTER	10.05086685	2584
chr10.fa	16555742	16564004	C1QL3	0	0
chr10.fa	16632617	16859453	RSU1	19.51903765	4864
chr10.fa	16865965	17171816	CUBN	12.46960519	9880
chr10.fa	17184982	17244070	TRDMT1	1.044605797	532
chr10.fa	17270258	17279592	VIM	12.32487246	1748
chr10.fa	17362676	17496254	ST8SIA6	0.818696239	76
chr10.fa	17428935	17450285	LOC100128098	0	0
chr10.fa	17631958	17659373	PTPLA	0	0
chr10.fa	17686124	17757907	STAM	13.81150796	2720

chr10.fa	17794251	18089854	FAM23A	0	0
chr10.fa	18240768	18332221	SLC39A12	0	0
chr10.fa	18429606	18830688	CACNB2	0.764026126	304
chr10.fa	18834264	18940550	NSUN6	7.077144033	1140
chr10.fa	18948313	18966940	ARL5B	4.464876508	1064
chr10.fa	20105372	20569115	PLXDC2	3.088786086	532
chr10.fa	21068903	21463116	NEBL	10.9548063	7220
chr10.fa	21414692	21435488	C10orf113	0	0
chr10.fa	21783421	21786213	C10orf114	1.726400844	228
chr10.fa	21785491	21785570	MIR1915	0	0
chr10.fa	21802409	21814611	C10orf140	1.214640391	532
chr10.fa	21823101	22032559	MLLT10	13.35291156	5231
chr10.fa	22045477	22292650	DNAJC1	2.189515438	304
chr10.fa	22497743	22498912	EBLN1	0	0
chr10.fa	22605299	22609237	COMMD3	25.09041917	1576
chr10.fa	22610006	22620414	BMI1	16.38371419	3876
chr10.fa	22634399	22706539	SPAG6	0.882553341	152
chr10.fa	22724354	22726858	LOC100499489	3.655367258	608
chr10.fa	22823766	23003503	PIP4K2A	10.75028284	2736
chr10.fa	23216954	23326514	ARMC3	0	0
chr10.fa	23384427	23410942	MSRB2	24.6491425	2964
chr10.fa	23481460	23483181	PTF1A	0	0
chr10.fa	23605520	23633772	C10orf67	0	0
chr10.fa	23728198	23731310	OTUD1	5.147575193	1064
chr10.fa	23983675	24836772	KIAA1217	48.82101341	27207
chr10.fa	24536054	24544975	PRINS	0	0
chr10.fa	24564614	24564710	MIR603	0	0
chr10.fa	24872538	25012597	ARHGAP21	9.546636716	4543
chr10.fa	25137554	25241533	PRTFDC1	0.594292744	76
chr10.fa	25270917	25305030	ENKUR	0	0
chr10.fa	25305508	25315593	THNSL1	2.73245141	684
chr10.fa	25447001	25448832	LOC100128811	0	0
chr10.fa	25464290	25891157	GPR158	0.566581172	228
chr10.fa	26223002	26501465	MYO3A	0	0
chr10.fa	26505236	26593491	GAD2	0	0
chr10.fa	26727266	26856732	APBB1IP	0.434047564	76
chr10.fa	26878794	26883251	NCRNA00264	0	0
chr10.fa	26932037	26942383	LOC731789	0	0
chr10.fa	26986595	27035524	PDSS1	5.658432007	532
chr10.fa	27035727	27150016	ABI1	13.46225179	3192
chr10.fa	27220135	27230930	NCRNA00202	0	0
chr10.fa	27293045	27389427	ANKRD26	4.190019879	1886
chr10.fa	27399383	27443321	YME1L1	51.1582737	13679

chr10.fa	27443753	27475848	MASTL	4.419393384	1064
chr10.fa	27484143	27531068	ACBD5	24.69492684	6667
chr10.fa	27534782	27541235	LOC387646	0.177414306	76
chr10.fa	27687117	27703297	PTCHD3	0	0
chr10.fa	27793249	27829099	RAB18	34.76356524	6440
chr10.fa	27961803	28034778	MKX	0	0
chr10.fa	28101097	28287977	ARMC4	0	0
chr10.fa	28339923	28571067	MPP7	24.62474427	8512
chr10.fa	28808846	28821283	LOC220906	2.767843907	988
chr10.fa	28821427	28909923	WAC	58.37864439	15222
chr10.fa	28966424	28971868	BAMBI	5.975307814	684
chr10.fa	29577990	29600158	LYZL1	0	0
chr10.fa	29698501	29711299	LOC387647	15.27660675	2052
chr10.fa	29746277	30024730	SVIL	15.24738911	8653
chr10.fa	29833933	29834026	MIR604	0	0
chr10.fa	29891193	29891275	MIR938	0	0
chr10.fa	30301729	30348488	KIAA1462	4.307643456	2660
chr10.fa	30598730	30638267	MTPAP	5.295320044	1976
chr10.fa	30653256	30663377	LOC729668	0	0
chr10.fa	30722866	30750761	MAP3K8	6.654693159	1368
chr10.fa	30900708	30918647	LYZL2	0	0
chr10.fa	31133565	31320866	ZNF438	2.388315849	608
chr10.fa	31605457	31608024	LOC220930	0.517182282	76
chr10.fa	31608101	31818742	ZEB1	0	0
chr10.fa	32095225	32217770	ARHGAP12	72.88098378	20189
chr10.fa	32297938	32345371	KIF5B	56.2657876	22001
chr10.fa	32557859	32636113	EPC1	31.43456199	6080
chr10.fa	32735041	32863492	CCDC7	1.981829251	262
chr10.fa	32871962	33171792	C10orf68	1.346571573	228
chr10.fa	33189246	33247293	ITGB1	11.48253102	3116
chr10.fa	33466419	33623833	NRP1	1.549137144	608
chr10.fa	34398488	35104253	PARD3	22.39576996	9120
chr10.fa	35297479	35379570	CUL2	17.08373261	5320
chr10.fa	35415801	35501886	CREM	3.418614041	836
chr10.fa	35535953	35860847	CCNY	19.93968124	5652
chr10.fa	35894338	35897863	GJD4	0	0
chr10.fa	35927177	35930362	FZD8	0	0
chr10.fa	37414785	37521495	ANKRD30A	0	0
chr10.fa	37890366	37891859	MTRNR2L7	0	0
chr10.fa	38117899	38146486	ZNF248	3.047218727	988
chr10.fa	38238795	38265453	ZNF25	3.982785511	988
chr10.fa	38299578	38348995	ZNF33A	11.96778475	4864
chr10.fa	38383264	38412278	ZNF37A	4.722413404	2204

chr10.fa	38464599	38503273	LOC100129055	0	0
chr10.fa	38645308	38667433	HSD17B7P2	4.9019362	456
chr10.fa	38671998	38691780	SEPT7L	0	0
chr10.fa	38717074	38741081	LOC399744	1.089486496	380
chr10.fa	42827314	42863493	LOC441666	0.343683741	152
chr10.fa	42970939	42990785	LOC84856	1.489045201	228
chr10.fa	43008961	43048280	ZNF37BP	1.055901275	608
chr10.fa	43084555	43133992	ZNF33B	9.413500684	3724
chr10.fa	43277954	43330385	BMS1	13.93786671	7448
chr10.fa	43572517	43625797	RET	0.17892037	76
chr10.fa	43633934	43680749	CSGALNACT2	3.689253692	912
chr10.fa	43689984	43762367	RASGEF1A	1.061473711	228
chr10.fa	43867092	43871783	FXYD4	49.42072798	2530
chr10.fa	43881065	43904696	HNRNPF	75.72684179	15728
chr10.fa	43932574	43978007	ZNF487P	0	0
chr10.fa	44051793	44070065	ZNF239	2.212859426	304
chr10.fa	44101855	44113352	ZNF485	1.670977699	228
chr10.fa	44139307	44144326	ZNF32	34.9021231	2964
chr10.fa	44282860	44285865	HNRNPA3P1	0	0
chr10.fa	44340754	44346070	C10orf136	0	0
chr10.fa	44404752	44465355	LOC283033	0	0
chr10.fa	44865605	44880545	CXCL12	0.502573464	152
chr10.fa	45306472	45455137	LOC220980	0	0
chr10.fa	45406764	45430642	TMEM72	3.164842304	228
chr10.fa	45455219	45490172	RASSF4	2.752632663	456
chr10.fa	45471709	45474330	C10orf10	3.88940956	532
chr10.fa	45493146	45496470	C10orf25	0.374256835	76
chr10.fa	45498728	45500777	ZNF22	9.631428104	1311
chr10.fa	45594924	45650044	LOC100133308	0	0
chr10.fa	45650110	45681489	LOC338579	0	0
chr10.fa	45659462	45659536	MIR3156-1	0	0
chr10.fa	45798102	45811056	OR13A1	0	0
chr10.fa	45869629	45941563	ALOX5	46.19699859	7828
chr10.fa	45952817	46090354	8-Mar	13.73530114	2508
chr10.fa	46111039	46168251	ANUBL1	4.94967842	1216
chr10.fa	46222648	46288412	FAM21C	7.561192913	2332
chr10.fa	46321050	46342921	AGAP4	0	0
chr10.fa	46897641	46939145	FAM35B	0	0
chr10.fa	46955444	46970601	SYT15	0.226813196	76
chr10.fa	46993546	47000568	GPRIN2	5.650901689	684
chr10.fa	47083534	47088320	PPYR1	0	0
chr10.fa	47096454	47151400	LOC643650	0.397902035	76
chr10.fa	47133295	47133836	LOC728643	0	0

chr10.fa	47157984	48271368	ANXA8	0	0
chr10.fa	47191844	48237508	AGAP9	0	0
chr10.fa	47242687	48186660	LOC642826	0	0
chr10.fa	47379720	47421238	FAM35B2	0	0
chr10.fa	47658234	47701446	ANTXRL	0	0
chr10.fa	47746920	47763040	ANXA8L2	0	0
chr10.fa	47894023	51837912	FAM21B	2.366477925	684
chr10.fa	48155943	48158691	CTSLL2	0	0
chr10.fa	48355089	48373866	ZNF488	0	0
chr10.fa	48381487	48390991	RBP3	0	0
chr10.fa	48413092	48416853	GDF2	0	0
chr10.fa	48425789	48439166	GDF10	0	0
chr10.fa	48844036	49365413	FRMPD2P1	0	0
chr10.fa	49218159	49239658	LOC399753	0	0
chr10.fa	49382999	49482941	FRMPD2	0	0
chr10.fa	49609687	49643183	MAPK8	14.60550476	1444
chr10.fa	49654079	49813138	ARHGAP22	0	0
chr10.fa	49893518	50191001	WDFY4	0.228620473	152
chr10.fa	50117529	50122280	LRRC18	0	0
chr10.fa	50193557	50193632	MIR4294	0	0
chr10.fa	50222333	50323559	C10orf72	0.142323022	76
chr10.fa	50339201	50342065	FAM170B	0	0
chr10.fa	50363891	50396407	C10orf128	0	0
chr10.fa	50507187	50535537	C10orf71	0.216571963	76
chr10.fa	50574161	50599907	DRGX	0	0
chr10.fa	50664491	50747147	ERCC6	10.05478262	4180
chr10.fa	50723151	50725167	PGBD3	2.269939241	304
chr10.fa	50817141	50873150	CHAT	0	0
chr10.fa	50818347	50820766	SLC18A3	0	0
chr10.fa	50887684	50916956	C10orf53	0	0
chr10.fa	50942687	50970425	OGDHL	9.127649789	2280
chr10.fa	51026325	51371331	PARG	8.195095133	2114
chr10.fa	51224688	51246535	AGAP8	0	0
chr10.fa	51253908	51352122	LOC728407	10.39349635	726
chr10.fa	51464162	51486327	AGAP7	0.472904008	76
chr10.fa	51549553	51562592	MSMB	2158.250158	81540
chr10.fa	51565108	51590734	NCOA4	103.7494164	28120
chr10.fa	51592088	51623336	TIMM23	48.9083651	3793
chr10.fa	51748078	51770259	AGAP6	0	0
chr10.fa	51827684	51829471	FAM21A	3.068604832	76
chr10.fa	51947000	52008370	ASAH2	0	0
chr10.fa	52065345	52383737	SGMS1	7.014642388	1748
chr10.fa	52499708	52514569	ASAH2B	0	0

chr10.fa	52559169	52645435	A1CF	2.0420718	1292
chr10.fa	52750945	54055274	PRKG1	0	0
chr10.fa	53059333	53059415	MIR605	0	0
chr10.fa	53455246	53459355	CSTF2T	8.633359676	2356
chr10.fa	54074041	54077417	DKK1	0	0
chr10.fa	54525140	54531460	MBL2	0	0
chr10.fa	55562533	56561051	PCDH15	0	0
chr10.fa	57358750	57360487	MTRNR2L5	0	0
chr10.fa	58117199	58121034	ZWINT	3.526146991	380
chr10.fa	59064239	59064319	MIR3924	0	0
chr10.fa	59951278	60027694	IPMK	0.933157082	380
chr10.fa	60028895	60049019	CISD1	5.516259592	760
chr10.fa	60094739	60130509	UBE2D1	5.612948883	988
chr10.fa	60145176	60155897	TFAM	10.0118598	1287
chr10.fa	60272904	60588845	BICC1	19.08288159	3952
chr10.fa	60474775	60477293	LOC728640	0	0
chr10.fa	60936348	61007534	PHYHIPL	1.617512437	228
chr10.fa	61011335	61122661	FAM13C	16.65299839	2238
chr10.fa	61410522	61469649	SLC16A9	4.880399489	1292
chr10.fa	61548506	61666414	CCDC6	37.32839176	14440
chr10.fa	61717975	61720671	C10orf40	0	0
chr10.fa	61788159	62149488	ANK3	11.6521138	12160
chr10.fa	62538089	62554610	CDK1	0	0
chr10.fa	62629198	62761198	RHOBTB1	2.627478768	836
chr10.fa	63166401	63213208	TMEM26	0.221993793	76
chr10.fa	63422719	63526091	C10orf107	1.828511964	152
chr10.fa	63661013	63856707	ARID5B	35.25333716	18588
chr10.fa	63952953	64028466	RTKN2	0.861769661	380
chr10.fa	64133916	64431771	ZNF365	0	0
chr10.fa	64564516	64568239	ADO	8.298712317	2052
chr10.fa	64571756	64578927	EGR2	0	0
chr10.fa	64893007	64914786	NRBF2	11.4701813	1444
chr10.fa	64926988	65140242	JMJD1C	24.46630637	14260
chr10.fa	65132717	65132808	MIR1296	0	0
chr10.fa	65224989	65226322	LOC84989	2.643443044	152
chr10.fa	65281123	65384883	REEP3	4.398157885	1520
chr10.fa	66585285	66586634	ANXA2P3	0	0
chr10.fa	67679725	69455949	CTNNA3	1.020207565	228
chr10.fa	68685792	68860867	LRRTM3	0	0
chr10.fa	69556427	69597937	DNAJC12	1.49823219	152
chr10.fa	69644427	69678147	SIRT1	5.838557228	1672
chr10.fa	69681656	69835103	HERC4	19.98200163	5993
chr10.fa	69869250	69971773	MYPN	0	0

chr10.fa	69990382	69991855	ATOH7	0	0
chr10.fa	70042417	70092684	PBLD	12.91811096	2448
chr10.fa	70091768	70102953	HNRNPH3	35.11643597	5624
chr10.fa	70103275	70167051	RUFY2	4.882507978	1973
chr10.fa	70173821	70231879	DNA2	2.332742098	684
chr10.fa	70242097	70287584	SLC25A16	34.62229646	5846
chr10.fa	70320117	70454239	TET1	4.299510712	2736
chr10.fa	70480971	70551309	CCAR1	30.0119342	7688
chr10.fa	70514929	70514995	SNORD98	0	0
chr10.fa	70587294	70655209	STOX1	1.274280515	304
chr10.fa	70661034	70706603	DDX50	14.77584056	2490
chr10.fa	70715892	70744279	DDX21	28.08688355	7745
chr10.fa	70748477	70776739	KIAA1279	21.13820675	3572
chr10.fa	70847828	70864567	SRGN	0	0
chr10.fa	70883908	70932616	VPS26A	26.27388404	4712
chr10.fa	70939993	70968849	SUPV3L1	6.477128246	1064
chr10.fa	70980059	71027315	HKDC1	0.310249127	76
chr10.fa	71029756	71161637	HK1	46.76839916	13148
chr10.fa	71163958	71176674	TACR2	0.947464687	152
chr10.fa	71211226	71267423	TSPAN15	4.71593733	532
chr10.fa	71331791	71333210	NEUROG3	0	0
chr10.fa	71390003	71393355	C10orf35	3.191349025	228
chr10.fa	71561644	71718904	COL13A1	0	0
chr10.fa	71812357	71872022	H2AFY2	6.356040723	912
chr10.fa	71872041	71892690	AIFM2	4.608253774	988
chr10.fa	71897733	71906496	TYSND1	5.720030013	1444
chr10.fa	71909961	71930285	SAR1A	36.5211416	7447
chr10.fa	71962586	71993190	PPA1	88.33952303	7637
chr10.fa	72014713	72043450	NPFFR1	0	0
chr10.fa	72058729	72142382	LRRC20	1.061172498	228
chr10.fa	72163861	72188374	EIF4EBP2	63.98617144	31996
chr10.fa	72191692	72201465	NODAL	0	0
chr10.fa	72238564	72328206	KIAA1274	0	0
chr10.fa	72357104	72362531	PRF1	0	0
chr10.fa	72432559	72522195	ADAMTS14	0	0
chr10.fa	72530995	72545157	C10orf27	0	0
chr10.fa	72575704	72640932	SGPL1	26.38021214	10105
chr10.fa	72643267	72648541	PCBD1	100.1247729	6688
chr10.fa	72972298	73060749	UNC5B	1.374132539	456
chr10.fa	73079010	73123147	SLC29A3	7.068559469	1064
chr10.fa	73156691	73575704	CDH23	1.129397185	912
chr10.fa	73471458	73497581	C10orf105	0	0
chr10.fa	73507314	73533337	C10orf54	0.239765344	76

chr10.fa	73576055	73611082	PSAP	33.6020889	6323
chr10.fa	73724120	73773322	CHST3	1.148976013	532
chr10.fa	73818792	73848790	SPOCK2	0	0
chr10.fa	73856274	73976183	ASCC1	8.247054331	1444
chr10.fa	73975806	73995618	ANAPC16	42.63169394	9044
chr10.fa	74033677	74035797	DDIT4	28.09260659	3268
chr10.fa	74092588	74114907	DNAJB12	8.231993694	1748
chr10.fa	74119698	74336541	MIR1256	0	0
chr10.fa	74127085	74385949	CBARA1	24.4080217	4332
chr10.fa	74451889	74647452	CCDC109A	8.927042102	1748
chr10.fa	74653339	74692787	OIT3	0	0
chr10.fa	74694938	74714510	PLA2G12B	1.04822035	76
chr10.fa	74766980	74856732	P4HA1	3.042098111	608
chr10.fa	74870210	74891581	NUDT13	0.543990216	76
chr10.fa	74894282	74927853	ECD	12.73964241	1976
chr10.fa	74927877	75001939	FAM149B1	11.6945848	2432
chr10.fa	75002582	75007025	DNAJC9	7.965269809	760
chr10.fa	75008601	75012451	MRPS16	28.68042326	5016
chr10.fa	75013516	75118617	TTC18	1.854115047	456
chr10.fa	75135189	75173841	ANXA7	46.0838932	6744
chr10.fa	75183337	75193319	ZMYND17	0.472452189	76
chr10.fa	75196563	75255782	PPP3CB	13.09371799	2736
chr10.fa	75257296	75335433	USP54	14.30067746	5852
chr10.fa	75391412	75401515	MYOZ1	0.742790627	76
chr10.fa	75404644	75415832	SYNPO2L	0	0
chr10.fa	75434033	75457554	AGAP5	0	0
chr10.fa	75458909	75490272	BMS1P4	2.335904832	304
chr10.fa	75504132	75531919	SEC24C	50.28189522	15124
chr10.fa	75532049	75535976	FUT11	1.670977699	228
chr10.fa	75541808	75543406	CHCHD1	28.47966497	1596
chr10.fa	75545605	75561551	KIAA0913	19.02775966	7372
chr10.fa	75561669	75571589	NDST2	1.718569312	456
chr10.fa	75572259	75634338	CAMK2G	25.75700297	6675
chr10.fa	75669727	75682535	C10orf55	0	0
chr10.fa	75672043	75677258	PLAU	1.788450669	228
chr10.fa	75757872	75879914	VCL	14.39631251	5244
chr10.fa	75880015	75910826	AP3M1	20.91802024	7296
chr10.fa	75910965	76469060	ADK	11.21912048	1653
chr10.fa	76586379	76792639	MYST4	18.20665372	10336
chr10.fa	76797594	76818272	DUPD1	0	0
chr10.fa	76854190	76868970	DUSP13	0	0
chr10.fa	76871393	76941881	SAMD8	1.796432807	836
chr10.fa	76969912	76991207	VDAC2	16.76821226	2347

chr10.fa	76993729	76995770	COMTD1	1.173976671	76
chr10.fa	77157603	77161285	ZNF503	6.201669192	1216
chr10.fa	77161514	77168740	NCRNA00245	2.708053177	456
chr10.fa	77542519	78317126	C10orf11	3.849649478	228
chr10.fa	78629359	79397577	KCNMA1	0.233439877	228
chr10.fa	79550549	79686348	DLG5	23.10949356	11476
chr10.fa	79686570	79689583	LOC100128292	0	0
chr10.fa	79734907	79789298	POLR3A	10.69561273	4712
chr10.fa	79793518	79816571	RPS24	345.4488479	67940
chr10.fa	80703083	80827205	LOC283050	0.470494307	76
chr10.fa	80828792	81076285	ZMIZ1	32.42178676	16264
chr10.fa	81107220	81115089	PPIF	16.128587	2356
chr10.fa	81142083	81205383	ZCCHC24	3.713953137	1216
chr10.fa	81272357	81276192	EIF5AL1	0.298351223	76
chr10.fa	81315608	81320163	SFTPA2	0.520043803	76
chr10.fa	81370695	81375199	SFTPA1	0	0
chr10.fa	81442731	81448650	LOC650623	0.773363721	304
chr10.fa	81585658	81587358	LOC642361	0	0
chr10.fa	81664654	81691557	LOC100288974	0	0
chr10.fa	81679934	81682875	MBL1P	0	0
chr10.fa	81697496	81708861	SFTPD	0	0
chr10.fa	81805989	81838949	LOC219347	0.861468449	228
chr10.fa	81838426	81852307	C10orf57	14.96921914	1964
chr10.fa	81892258	81904784	PLAC9	0	0
chr10.fa	81914880	81965328	ANXA11	106.8257022	19364
chr10.fa	82031576	82049434	MAT1A	0	0
chr10.fa	82095862	82116500	DYDC1	0	0
chr10.fa	82116558	82127829	DYDC2	0	0
chr10.fa	82168242	82192753	C10orf58	48.59796537	5776
chr10.fa	82214038	82282391	TSPAN14	6.154981216	2280
chr10.fa	82297658	82406316	SH2D4B	0	0
chr10.fa	83635070	84746935	NRG3	0	0
chr10.fa	85899185	85913311	GHITM	90.20387931	14668
chr10.fa	85933554	85945050	C10orf99	5.529512953	304
chr10.fa	85954412	85979376	CDHR1	0	0
chr10.fa	85980249	85985284	LRIT2	0	0
chr10.fa	85991276	86001217	LRIT1	0	0
chr10.fa	86004809	86018944	RGR	0	0
chr10.fa	86088410	86278276	FAM190B	11.33282829	5776
chr10.fa	87359312	88126250	GRID1	0	0
chr10.fa	88024451	88024545	MIR346	0	0
chr10.fa	88195013	88281541	WAPAL	35.05393433	14668
chr10.fa	88414314	88426216	OPN4	0	0

chr10.fa	88428206	88495824	LDB3	0	0
chr10.fa	88516396	88684945	BMPR1A	3.798443311	912
chr10.fa	88695298	88717425	MMRN2	7.1212717	1976
chr10.fa	88718288	88723017	SNCG	213.1098238	11688
chr10.fa	88728188	88730497	C10orf116	608.204255	20313
chr10.fa	88730667	88769960	AGAP11	4.161103456	825
chr10.fa	88780046	88784487	FAM25A	0	0
chr10.fa	88809959	88854776	GLUD1	83.38382035	19245
chr10.fa	88854953	88951222	FAM35A	12.37502439	2797
chr10.fa	88985205	88994733	FAM22A	0.348051326	76
chr10.fa	88998424	89102167	LOC728190	9.9882146	447
chr10.fa	89102316	89103331	LOC439994	1.126535664	76
chr10.fa	89117477	89130452	FAM22D	0	0
chr10.fa	89264223	89313218	MINPP1	0.693090525	152
chr10.fa	89419476	89507462	PAPSS2	2.373405818	608
chr10.fa	89512875	89577917	ATAD1	4.52707694	912
chr10.fa	89578070	89605369	CFLP1	0.539923844	76
chr10.fa	89618918	89623194	KILLIN	0.535255047	152
chr10.fa	89623195	89728532	PTEN	16.9204753	6232
chr10.fa	90033621	90343082	RNLS	5.746988554	1368
chr10.fa	90346519	90366733	LIPJ	0	0
chr10.fa	90424146	90438572	LIPF	0.750019733	76
chr10.fa	90484301	90512513	LIPK	0	0
chr10.fa	90521163	90537999	LIPN	0	0
chr10.fa	90562487	90577653	LIPM	0	0
chr10.fa	90579659	90611732	ANKRD22	0.624112806	152
chr10.fa	90640026	90683244	STAMBPL1	1.719472951	228
chr10.fa	90694831	90751147	ACTA2	0	0
chr10.fa	90750288	90775542	FAS	0	0
chr10.fa	90751181	90752732	FAS-AS1	0	0
chr10.fa	90965694	90967071	CH25H	0	0
chr10.fa	90973326	91011660	LIPA	5.169413117	912
chr10.fa	91061706	91069033	IFIT2	7.532427096	1748
chr10.fa	91087602	91100725	IFIT3	8.516489131	1520
chr10.fa	91137813	91144962	IFIT1B	0	0
chr10.fa	91152322	91163744	IFIT1	7.384531638	912
chr10.fa	91174325	91180759	IFIT5	7.661044937	2052
chr10.fa	91190051	91295313	SLC16A12	4.457647402	1368
chr10.fa	91342749	91405215	PANK1	10.60720679	2508
chr10.fa	91352504	91352584	MIR107	0	0
chr10.fa	91451057	91457685	FLJ37201	0.312508222	76
chr10.fa	91461367	91534700	KIF20B	0.725621501	304
chr10.fa	92500576	92617671	HTR7	0	0

chr10.fa	92631709	92668312	RPP30	3.063634822	869
chr10.fa	92671857	92681032	ANKRD1	0	0
chr10.fa	92911761	92912837	NUDT9P1	0	0
chr10.fa	92980369	93044021	PCGF5	9.283226172	4393
chr10.fa	93066719	93371217	LOC100188947	10.06984325	684
chr10.fa	93170096	93274520	HECTD2	1.176838192	456
chr10.fa	93388199	93392858	PPP1R3C	60.99241798	10412
chr10.fa	93558151	93625232	TNKS2	38.34046658	15972
chr10.fa	93666345	93669258	FGFBP3	1.339191861	228
chr10.fa	93683736	93790080	BTAF1	13.6612028	6384
chr10.fa	93808397	94050875	CPEB3	5.929674083	2356
chr10.fa	94050920	94113721	5-Mar	18.95049859	4940
chr10.fa	94211441	94333852	IDE	18.32894609	7285
chr10.fa	94352825	94415152	KIF11	0.450764872	152
chr10.fa	94449681	94455408	HHEX	0.650770134	76
chr10.fa	94594470	94819251	EXOC6	9.814414846	2432
chr10.fa	94821021	94828454	CYP26C1	0	0
chr10.fa	94833232	94837641	CYP26A1	0	0
chr10.fa	95066186	95242074	MYOF	33.99487032	15446
chr10.fa	95256369	95288849	CEP55	0	0
chr10.fa	95326422	95349829	GPR120	0	0
chr10.fa	95351593	95360993	RBP4	30.80216583	1900
chr10.fa	95372345	95425429	PDE6C	0	0
chr10.fa	95427640	95462329	FRA10AC1	4.076010855	836
chr10.fa	95517566	95557916	LGI1	0.483747667	76
chr10.fa	95653730	95662491	TMEM20	0	0
chr10.fa	95717897	95721672	PIPSL	0	0
chr10.fa	95753746	96088148	PLCE1	86.49022739	46890
chr10.fa	96039047	96046827	LOC100128054	0	0
chr10.fa	96092989	96122683	NOC3L	5.663100805	1292
chr10.fa	96162186	96296089	TBC1D12	3.782780249	1444
chr10.fa	96305574	96361856	HELLS	1.10801108	228
chr10.fa	96443251	96495947	CYP2C18	0	0
chr10.fa	96522463	96612671	CYP2C19	0	0
chr10.fa	96698415	96749148	CYP2C9	0	0
chr10.fa	96796529	96829254	CYP2C8	0	0
chr10.fa	96953957	96988686	C10orf129	2.004570813	228
chr10.fa	96997330	97050781	PDLIM1	82.03950788	7904
chr10.fa	97071530	97321171	SORBS1	2.250962838	1102
chr10.fa	97365686	97416567	ALDH18A1	18.17894215	4180
chr10.fa	97423153	97453900	TCTN3	7.10425318	1292
chr10.fa	97471536	97637023	ENTPD1	1.751552108	1520
chr10.fa	97667722	97698415	C10orf131	0	0

chr10.fa	97759848	97792441	CC2D2B	0	0
chr10.fa	97803159	97820625	CCNJ	2.212859426	608
chr10.fa	97824072	97824156	MIR3157	0	0
chr10.fa	97889472	97923517	ZNF518A	8.831256449	4864
chr10.fa	97951455	98031333	BLNK	28.87380185	3495
chr10.fa	98064085	98098321	DNTT	0	0
chr10.fa	98102975	98119092	OPALIN	0	0
chr10.fa	98124363	98273683	TLL2	0.16898035	76
chr10.fa	98277867	98346809	TM9SF3	17.15045123	6992
chr10.fa	98353069	98480279	PIK3AP1	2.141170793	684
chr10.fa	98592017	98724198	LCOR	6.769455214	3572
chr10.fa	98741041	98745585	C10orf12	11.836456	3572
chr10.fa	98757795	98945683	SLIT1	0.144280904	76
chr10.fa	98981930	99052413	ARHGAP19	4.190471698	1520
chr10.fa	99079022	99081672	FRAT1	4.749371945	836
chr10.fa	99092254	99094458	FRAT2	10.38189966	1520
chr10.fa	99116458	99161127	RRP12	5.862955461	1723
chr10.fa	99186027	99193198	PGAM1	9.982039738	1140
chr10.fa	99195666	99205768	EXOSC1	12.93904525	988
chr10.fa	99205930	99217127	ZDHHC16	9.254008535	1216
chr10.fa	99218081	99258366	MMS19	16.45796313	4028
chr10.fa	99258768	99330960	UBTD1	2.940589416	304
chr10.fa	99332256	99343641	ANKRD2	0	0
chr10.fa	99344102	99372555	HOGA1	8.762128124	1444
chr10.fa	99349450	99350691	C10orf62	0	0
chr10.fa	99374310	99393913	MORN4	11.56174998	1976
chr10.fa	99400443	99436187	PI4K2A	10.6594672	2962
chr10.fa	99437181	99447015	AVPI1	0.822310792	76
chr10.fa	99473465	99477909	MARVELD1	0	0
chr10.fa	99496878	99520664	ZFYVE27	2.241474636	456
chr10.fa	99526508	99531756	SFRP5	0	0
chr10.fa	99609996	99627782	GOLGA7B	0.413414491	76
chr10.fa	99624758	99790585	CRTAC1	0	0
chr10.fa	99894381	100004654	C10orf28	14.60053475	3268
chr10.fa	100007443	100028007	LOXL4	0.625920083	152
chr10.fa	100143322	100174978	PYROXD2	3.967724874	524
chr10.fa	100155051	100155064	MIR1287	0	0
chr10.fa	100175956	100206704	HPS1	11.56581635	3187
chr10.fa	100216834	100995632	HPSE2	0	0
chr10.fa	101088856	101154087	CNNM1	0.192023124	76
chr10.fa	101156627	101190530	GOT1	22.60164887	3192
chr10.fa	101292690	101296280	NKX2-3	0	0
chr10.fa	101370275	101380221	SLC25A28	11.04125435	1140

chr10.fa	101419263	101468504	ENTPD7	3.023573527	1216
chr10.fa	101471003	101492423	COX15	9.879175586	3268
chr10.fa	101496003	101515894	CUTC	11.43524062	880
chr10.fa	101542463	101611662	ABCC2	1.133011738	380
chr10.fa	101635334	101769676	DNMBP	24.43859479	9272
chr10.fa	101686966	101718755	NCRNA00093	0	0
chr10.fa	101802065	101841642	CPN1	0	0
chr10.fa	101909847	101945814	ERLIN1	2.276867134	532
chr10.fa	101948124	101989344	CHUK	15.59062104	3648
chr10.fa	101992055	102027437	CWF19L1	7.82490467	1368
chr10.fa	101996913	101997059	SNORA12	0	0
chr10.fa	102033713	102046439	BLOC1S2	13.44538387	1748
chr10.fa	102047903	102090243	PKD2L1	0	0
chr10.fa	102106772	102124588	SCD	26.37539274	9576
chr10.fa	102133333	102148111	NCRNA00263	23.17018793	1520
chr10.fa	102222812	102243399	WNT8B	0	0
chr10.fa	102246403	102279595	SEC31B	0.496398603	152
chr10.fa	102283497	102289636	NDUFB8	93.43739812	4256
chr10.fa	102295641	102313681	HIF1AN	19.20713185	8815
chr10.fa	102505468	102589698	PAX2	4.351018091	1216
chr10.fa	102672326	102724891	FAM178A	10.54651242	5168
chr10.fa	102732286	102745372	SEMA4G	5.112032089	1243
chr10.fa	102734742	102734841	MIR608	0	0
chr10.fa	102737579	102747272	MRPL43	13.6137618	1976
chr10.fa	102747293	102754158	C10orf2	5.057663189	1216
chr10.fa	102756864	102767439	LZTS2	10.19695503	1824
chr10.fa	102767594	102790914	PDZD7	0	0
chr10.fa	102790996	102800998	SFXN3	7.691166212	1578
chr10.fa	102820999	102825351	KAZALD1	0.455283063	76
chr10.fa	102849078	102890903	TLX1NB	0	0
chr10.fa	102891061	102897546	TLX1	0	0
chr10.fa	102986733	102988717	LBX1	0	0
chr10.fa	102989351	102998616	FLJ41350	0	0
chr10.fa	103113825	103317070	BTRC	12.52879349	5092
chr10.fa	103338639	103348027	POLL	5.976060846	1140
chr10.fa	103348089	103369410	DPCD	15.75809532	836
chr10.fa	103370421	103454743	FBXW4	53.26375078	7823
chr10.fa	103529887	103535759	FGF8	0	0
chr10.fa	103541082	103543170	NPM3	5.220619283	304
chr10.fa	103544200	103578222	MGEA5	58.00453816	20035
chr10.fa	103585731	103603677	KCNIP2	0	0
chr10.fa	103605356	103815932	C10orf76	12.45695425	3392
chr10.fa	103825147	103827793	HPS6	7.783487918	1368

chr10.fa	103867325	103880210	LDB1	21.04528262	3724
chr10.fa	103892787	103910090	PPRC1	12.00784605	4256
chr10.fa	103911933	103923627	NOLC1	24.86375658	6498
chr10.fa	103986143	103989344	ELOVL3	0	0
chr10.fa	103989946	104001231	PITX3	0	0
chr10.fa	104005255	104142656	GBF1	28.06323835	12000
chr10.fa	104154229	104162281	NFKB2	5.470023436	1216
chr10.fa	104162376	104178901	PSD	0	0
chr10.fa	104179571	104182893	FBXL15	5.330561935	836
chr10.fa	104183002	104192423	CUEDC2	17.85619269	1418
chr10.fa	104196269	104196341	MIR146B	0	0
chr10.fa	104209594	104211300	C10orf95	5.454209767	532
chr10.fa	104221170	104236802	TMEM180	0	0
chr10.fa	104238986	104262512	ACTR1A	36.16013813	6898
chr10.fa	104263719	104393214	SUFU	23.12334934	8208
chr10.fa	104404252	104418076	TRIM8	61.48294293	11202
chr10.fa	104433484	104474190	ARL3	10.02225164	2578
chr10.fa	104474298	104498946	SFXN2	11.02800099	1876
chr10.fa	104503727	104576021	C10orf26	17.70543571	5168
chr10.fa	104590288	104597290	CYP17A1	10.40554486	1292
chr10.fa	104629210	104629350	AS3MT	0	0
chr10.fa	104678114	104838240	CNNM2	1.850801707	532
chr10.fa	104845940	104953056	NT5C2	36.22023007	12893
chr10.fa	105005644	105007773	LOC729020	0	0
chr10.fa	105036920	105050108	INA	3.188336898	684
chr10.fa	105062553	105110891	PCGF6	2.053066065	304
chr10.fa	105127724	105148813	TAF5	2.107284359	456
chr10.fa	105148823	105156223	USMG5	49.86501678	1331
chr10.fa	105154152	105154158	MIR1307	0	0
chr10.fa	105156412	105206019	PDCD11	11.58885912	4937
chr10.fa	105206543	105212162	CALHM2	0.979092025	120
chr10.fa	105213144	105218648	CALHM1	0	0
chr10.fa	105232561	105238997	CALHM3	0	0
chr10.fa	105253735	105352309	NEURL	0	0
chr10.fa	105353784	105615164	SH3PXD2A	6.386011391	4769
chr10.fa	105637318	105678045	OBFC1	12.36267466	5320
chr10.fa	105727470	105787342	SLK	89.56048889	33337
chr10.fa	105791046	105845638	COL17A1	0	0
chr10.fa	105807863	105807944	MIR936	0	0
chr10.fa	105881816	105886143	C10orf78	3.743020167	380
chr10.fa	105889646	105992120	C10orf79	0.853335704	304
chr10.fa	105978547	105978641	MIR609	0	0
chr10.fa	106013952	106027222	GSTO1	44.56111217	3876

chr10.fa	106028631	106059176	GSTO2	13.66918494	1444
chr10.fa	106071899	106093663	ITPRIP	1.573685982	456
chr10.fa	106113522	106214848	CCDC147	1.036473053	228
chr10.fa	106400859	107024993	SORCS3	0	0
chr10.fa	108333421	108924292	SORCS1	0.159793361	76
chr10.fa	111624524	111683311	XPNPEP1	29.18992462	4921
chr10.fa	111765726	111895323	ADD3	61.08865545	18772
chr10.fa	111967363	112047123	MXI1	9.393771249	2392
chr10.fa	112052798	112064707	SMNDC1	7.333626684	988
chr10.fa	112257625	112271302	DUSP5	2.71663774	456
chr10.fa	112327449	112364392	SMC3	19.63154061	5360
chr10.fa	112404155	112599227	RBM20	0.316423988	152
chr10.fa	112628648	112630662	LOC282997	0.568087236	76
chr10.fa	112631553	112658487	PDCD4	105.9035394	17024
chr10.fa	112659765	112679124	BBIP1	10.64305111	836
chr10.fa	112679301	112773425	SHOC2	21.74951802	5700
chr10.fa	112696361	112697013	RPL13AP6	3.505664524	152
chr10.fa	112836790	112840662	ADRA2A	0	0
chr10.fa	113909622	113943525	GPAM	2.874473218	1216
chr10.fa	114043493	114063070	TECTB	0	0
chr10.fa	114067936	114116353	GUCY2GP	0	0
chr10.fa	114133916	114188138	ACSL5	5.156912788	1282
chr10.fa	114190058	114206672	ZDHHC6	18.35726009	2645
chr10.fa	114206756	114578504	VTI1A	7.036480312	2052
chr10.fa	114393929	114394013	MIR4295	0	0
chr10.fa	114610673	114615127	LOC143188	0.357087708	76
chr10.fa	114710009	114927436	TCF7L2	11.81597353	3394
chr10.fa	115310590	115349360	HABP2	0	0
chr10.fa	115349425	115423805	NRAP	0	0
chr10.fa	115438935	115490664	CASP7	12.27261205	2311
chr10.fa	115511213	115542192	C10orf81	1.08060072	304
chr10.fa	115594484	115613859	DCLRE1A	9.478562636	2812
chr10.fa	115614391	115672265	NHLRC2	13.07203068	5548
chr10.fa	115803806	115806667	ADRB1	2.799471245	532
chr10.fa	115881974	115934364	C10orf118	20.30098593	8360
chr10.fa	115939029	115992063	TDRD1	0	0
chr10.fa	115999018	116049751	VWA2	0	0
chr10.fa	116054583	116164515	AFAP1L2	18.42427993	4864
chr10.fa	116190869	116444414	ABLIM1	23.88150182	13166
chr10.fa	116581503	116659586	FAM160B1	10.0567405	4104
chr10.fa	116697952	116737439	TRUB1	8.320399635	1885
chr10.fa	116853124	117708496	ATRNL1	0.131027544	76
chr10.fa	117816442	118032796	GFRA1	0	0

chr10.fa	118083940	118139541	C10orf96	0	0
chr10.fa	118187424	118237468	PNLIPRP3	0	0
chr10.fa	118305428	118327367	PNLIP	0	0
chr10.fa	118350490	118368686	PNLIPRP1	0	0
chr10.fa	118380465	118404654	PNLIPRP2	0	0
chr10.fa	118423207	118429481	C10orf82	10.36367629	684
chr10.fa	118430703	118502085	HSPA12A	2.400364359	912
chr10.fa	118644306	118765088	KIAA1598	13.90714301	3641
chr10.fa	118888032	118897812	VAX1	0	0
chr10.fa	118927189	118927285	MIR3663	0	0
chr10.fa	118957000	118969810	KCNK18	0	0
chr10.fa	119000584	119038941	SLC18A2	0.295640308	76
chr10.fa	119042606	119134937	PDZD8	7.159375112	1824
chr10.fa	119243804	119304579	EMX2OS	0.157233053	76
chr10.fa	119301956	119309057	EMX2	23.71432875	4560
chr10.fa	119764427	119806114	RAB11FIP2	23.50167255	9458
chr10.fa	119806332	119969665	CASC2	0.439017575	152
chr10.fa	120068572	120101839	C10orf84	8.276271968	1520
chr10.fa	120352916	120355160	PRLHR	0	0
chr10.fa	120440494	120514758	C10orf46	19.5297307	8740
chr10.fa	120789228	120793854	NANOS1	1.731672067	532
chr10.fa	120794541	120840334	EIF3A	108.7432225	38304
chr10.fa	120819525	120819650	SNORA19	0	0
chr10.fa	120863611	120863628	FAM45A	0	0
chr10.fa	120897224	120897376	FAM45B	0	0
chr10.fa	120900425	120925204	SFXN4	20.84407251	1921
chr10.fa	120927215	120938345	PRDX3	110.0723237	11628
chr10.fa	120967197	121215131	GRK5	0	0
chr10.fa	121259339	121302222	RGS10	39.95556931	2584
chr10.fa	121332978	121356541	TIAL1	17.65016318	4560
chr10.fa	121410882	121437329	BAG3	19.60397964	3344
chr10.fa	121485609	121588659	INPP5F	3.658981811	1292
chr10.fa	121588972	121632394	MCMBP	12.14504845	3420
chr10.fa	121652223	121701245	SEC23IP	29.6739735	8360
chr10.fa	122216466	122349367	PPAPDC1A	2.257589518	228
chr10.fa	122521324	122610686	LOC283089	0	0
chr10.fa	122610692	122669038	WDR11	24.93017399	7828
chr10.fa	123237844	123357972	FGFR2	3.03381476	1064
chr10.fa	123502625	123687546	ATE1	27.84741942	4323
chr10.fa	123716603	123734743	NSMCE4A	18.73739058	1748
chr10.fa	123748689	124014057	TACC2	44.53144272	29852
chr10.fa	124030821	124097676	BTBD16	0.619444009	76
chr10.fa	124134094	124191871	PLEKHA1	10.29771069	2882

chr10.fa	124176481	124176583	MIR3941	0	0
chr10.fa	124214179	124216868	ARMS2	1.416603536	76
chr10.fa	124221041	124274424	HTRA1	2.69961922	380
chr10.fa	124320181	124403252	DMBT1	0	0
chr10.fa	124457225	124459338	C10orf120	0	0
chr10.fa	124516210	124557161	FLJ46361	0	0
chr10.fa	124591674	124605691	CUZD1	0	0
chr10.fa	124608610	124639148	FAM24B	1.500190073	76
chr10.fa	124639158	124658230	LOC399815	0	0
chr10.fa	124670217	124672627	FAM24A	0	0
chr10.fa	124690419	124713919	C10orf88	3.707778275	760
chr10.fa	124739556	124749907	PSTK	2.012854163	228
chr10.fa	124753197	124768311	IKZF5	20.02025565	2204
chr10.fa	124768429	124817806	ACADSB	25.62416815	10108
chr10.fa	124895567	124897247	HMX3	0	0
chr10.fa	124907638	124910188	HMX2	0	0
chr10.fa	124913760	124924886	BUB3	24.43272114	4484
chr10.fa	125425871	125456913	GPR26	0	0
chr10.fa	125505152	125651500	CPXM2	0	0
chr10.fa	125767182	125851930	CHST15	0.179522796	76
chr10.fa	126085872	126107545	OAT	35.01673455	4864
chr10.fa	126135998	126138550	NKX1-2	0	0
chr10.fa	126150341	126302710	LHPP	11.5480448	1292
chr10.fa	126307863	126432930	FAM53B	10.74712011	4096
chr10.fa	126447406	126480439	METTL10	7.544927425	1292
chr10.fa	126490354	126525239	FAM175B	6.503484361	1292
chr10.fa	126630692	126676005	ZRANB1	22.46595253	7372
chr10.fa	126676418	126849624	CTBP2	14.4648384	5244
chr10.fa	126721352	126721439	MIR4296	0	0
chr10.fa	127262940	127267014	LOC100169752	0	0
chr10.fa	127344263	127371713	C10orf122	0	0
chr10.fa	127371812	127398246	LOC283038	0	0
chr10.fa	127393859	127408062	FLJ37035	0	0
chr10.fa	127408084	127452712	C10orf137	9.998757046	3040
chr10.fa	127455027	127464390	MMP21	0	0
chr10.fa	127477147	127511837	UROS	42.26767834	3800
chr10.fa	127512104	127542264	BCCIP	34.74699854	3876
chr10.fa	127525125	127569884	DHX32	29.67803987	5624
chr10.fa	127585108	127698161	FANK1	0	0
chr10.fa	127702902	128077127	ADAM12	0	0
chr10.fa	128113574	128210010	C10orf90	0	0
chr10.fa	128594023	129250780	DOCK1	37.06603546	16615
chr10.fa	128933690	128994422	FAM196A	0	0

chr10.fa	129347613	129350935	NPS	0	0
chr10.fa	129535538	129539450	FOXI2	0	0
chr10.fa	129676114	129691211	CLRN3	3.004295911	228
chr10.fa	129705325	129884164	PTPRE	2.70007104	988
chr10.fa	129894925	129924468	MKI67	0.091568674	76
chr10.fa	131265454	131565783	MGMT	21.7159328	1824
chr10.fa	131633547	131762091	EBF3	0	0
chr10.fa	131641563	131641638	MIR4297	0	0
chr10.fa	131862162	131909081	LOC387723	3.428252849	608
chr10.fa	131934639	131978646	GLRX3	29.80123588	3166
chr10.fa	132760851	132760931	MIR378C	0	0
chr10.fa	132890655	133109984	TCERG1L	0.874721809	152
chr10.fa	133747960	133770053	PPP2R2D	26.82224184	3496
chr10.fa	133781204	133795435	BNIP3	64.91586458	6612
chr10.fa	133918313	133998313	JAKMIP3	0.529230792	228
chr10.fa	134000414	134019280	DPYSL4	0.42094481	76
chr10.fa	134020996	134121477	STK32C	1.080299507	152
chr10.fa	134145614	134195010	LRRC27	2.066018213	1292
chr10.fa	134210702	134231358	PWWP2B	9.653416634	1480
chr10.fa	134258714	134261825	C10orf91	0	0
chr10.fa	134351353	134596984	INPP5A	28.09637175	5481
chr10.fa	134598320	134599537	NKX6-2	0	0
chr10.fa	134742689	134756089	C10orf93	0	0
chr10.fa	134901409	134945179	GPR123	0	0
chr10.fa	134973971	135039916	KNDC1	0	0
chr10.fa	135043778	135045062	UTF1	0	0
chr10.fa	135051408	135055433	VENTX	0	0
chr10.fa	135061015	135061124	MIR202	0	0
chr10.fa	135075920	135090407	ADAM8	1.044304584	228
chr10.fa	135093138	135122644	TUBGCP2	17.81342048	3572
chr10.fa	135122894	135126666	ZNF511	13.95865039	836
chr10.fa	135138928	135150475	CALY	0	0
chr10.fa	135160844	135166187	PRAP1	9.156867425	456
chr10.fa	135168658	135171529	C10orf125	10.432654	532
chr10.fa	135175987	135186908	ECHS1	180.7276466	15948
chr10.fa	135185060	135185167	MIR3944	0	0
chr10.fa	135192741	135205198	PAOX	10.48973382	1292
chr10.fa	135207621	135234169	MTG1	12.65500163	1352
chr10.fa	135234175	135238121	SPRN	0.722609373	152
chr10.fa	135267432	135281953	LOC619207	0.233439877	76
chr10.fa	135340867	135352620	CYP2E1	5.493066211	608
chr10.fa	135367404	135379138	SYCE1	1.317203331	152
chr10.fa	135380223	135383462	SPRNP1	0	0

chr10.fa	135438603	135440299	FRG2B	0	0
chr10.fa	135480368	135480370	DUX4L2	0	0
chr10.fa	135480432	135498492	DUX2	0	0
chr11.fa	126987	131920	LOC100133161	0	0
chr11.fa	194501	194573	SCGB1C1	0	0
chr11.fa	196761	200258	ODF3	0	0
chr11.fa	202924	207422	BET1L	26.83624824	5016
chr11.fa	208530	215029	RIC8A	31.04178057	5396
chr11.fa	215111	236362	SIRT3	8.114972543	1520
chr11.fa	236808	252984	PSMD13	59.90729907	7327
chr11.fa	278570	285304	NLRP6	0	0
chr11.fa	289138	295688	ATHL1	3.152191369	684
chr11.fa	298203	299526	IFITM5	0	0
chr11.fa	308107	309410	IFITM2	18.81555528	912
chr11.fa	313991	315272	IFITM1	4.789132027	228
chr11.fa	319673	320914	IFITM3	47.97761772	2128
chr11.fa	369795	382117	B4GALNT4	0.331334019	76
chr11.fa	394217	404908	PKP3	10.59440525	1976
chr11.fa	405716	417397	SIGIRR	11.15481156	1368
chr11.fa	417930	442011	ANO9	0	0
chr11.fa	450280	491387	PTDSS2	3.147673177	511
chr11.fa	494512	507273	RNH1	57.77697193	8436
chr11.fa	532242	535550	HRAS	15.24784093	1140
chr11.fa	537522	554849	LRRC56	2.114212252	380
chr11.fa	554917	560779	C11orf35	0.563569045	76
chr11.fa	560971	564025	RASSF7	17.16912642	1824
chr11.fa	568089	568198	MIR210	0	0
chr11.fa	573808	575885	LOC143666	1.101685612	152
chr11.fa	576483	612222	PHRF1	12.02019577	4408
chr11.fa	612555	615999	IRF7	4.063058707	532
chr11.fa	616565	625067	CDHR5	2.85323772	661
chr11.fa	626313	627173	SCT	0	0
chr11.fa	637305	640705	DRD4	0	0
chr11.fa	644225	695615	DEAF1	19.87928809	3420
chr11.fa	695741	704129	TMEM80	11.64804743	1140
chr11.fa	706120	727727	EPS8L2	29.86720148	6229
chr11.fa	747432	765024	TALDO1	155.6226186	12844
chr11.fa	767223	777487	PDDC1	13.64508792	3952
chr11.fa	787110	790126	CEND1	0	0
chr11.fa	790475	798269	SLC25A22	3.540454596	760
chr11.fa	799179	805250	LRDD	1.522027997	304
chr11.fa	809936	812876	RPLP2	1083.266152	35460
chr11.fa	811681	811814	SNORA52	0	0

chr11.fa	818901	825571	PNPLA2	15.99771006	2578
chr11.fa	827585	831991	EFCAB4A	11.32032796	1368
chr11.fa	832952	838835	CD151	6.667645307	684
chr11.fa	839721	842529	POLR2L	104.5304611	6080
chr11.fa	842824	867116	TSPAN4	0	0
chr11.fa	867859	915058	CHID1	2.698866189	684
chr11.fa	925841	1012240	AP2A2	22.31685222	6840
chr11.fa	1012824	1036706	MUC6	0	0
chr11.fa	1074875	1104417	MUC2	0	0
chr11.fa	1244295	1283406	MUC5B	0	0
chr11.fa	1295598	1330892	TOLLIP	43.78277844	10640
chr11.fa	1330938	1331937	LOC255512	1.144608428	76
chr11.fa	1411129	1483328	BRSK2	0	0
chr11.fa	1490685	1785501	MOB2	9.958846357	1140
chr11.fa	1575281	1593150	DUSP8	0.767640679	228
chr11.fa	1593971	1620414	LOC338651	0	0
chr11.fa	1605572	1606513	KRTAP5-1	0	0
chr11.fa	1618407	1618477	KRTAP5-2	0	0
chr11.fa	1628795	1629693	KRTAP5-3	0	0
chr11.fa	1642188	1643368	KRTAP5-4	0	0
chr11.fa	1651033	1652159	KRTAP5-5	0	0
chr11.fa	1686829	1689086	FAM99A	0	0
chr11.fa	1704500	1706859	FAM99B	0	0
chr11.fa	1718425	1718985	KRTAP5-6	0	0
chr11.fa	1756030	1769349	LOC402778	0	0
chr11.fa	1773985	1785222	CTSD	22.13627518	3116
chr11.fa	1855674	1858750	SYT8	0.804388634	76
chr11.fa	1860233	1862910	TNNI2	0	0
chr11.fa	1874200	1913493	LSP1	0.447602138	76
chr11.fa	1880694	1880766	MIR4298	0	0
chr11.fa	1940799	1959936	TNNT3	0	0
chr11.fa	1968502	1977839	MRPL23	27.76684501	1368
chr11.fa	2004439	2011150	LOC100133545	0	0
chr11.fa	2016406	2019065	H19	0	0
chr11.fa	2150342	2156763	INS-IGF2	0	0
chr11.fa	2155364	2155439	MIR483	0	0
chr11.fa	2159459	2162341	IGF2	0	0
chr11.fa	2161758	2169896	IGF2AS	0	0
chr11.fa	2181009	2182397	INS	0	0
chr11.fa	2185159	2193035	TH	0.599262755	76
chr11.fa	2289728	2292182	ASCL2	0	0
chr11.fa	2317507	2323143	C11orf21	0	0
chr11.fa	2323243	2339430	TSPAN32	0	0

chr11.fa	2398547	2418649	CD81	15.2919686	1520
chr11.fa	2423523	2425106	TSSC4	11.25315752	1064
chr11.fa	2425746	2444275	TRPM5	0	0
chr11.fa	2466221	2870340	KCNQ1	0.338111305	76
chr11.fa	2661768	2721228	KCNQ1OT1	0.038555231	152
chr11.fa	2891263	2893336	KCNQ1DN	0	0
chr11.fa	2904448	2906995	CDKN1C	8.895866583	1140
chr11.fa	2909327	2925175	SLC22A18AS	2.374761276	228
chr11.fa	2921021	2946476	SLC22A18	2.776880289	281
chr11.fa	2949503	2950650	PHLDA2	17.41792815	1064
chr11.fa	2965660	3013607	NAP1L4	59.8417853	10005
chr11.fa	2985001	2985123	SNORA54	0	0
chr11.fa	3022152	3078681	CARS	15.64574297	2888
chr11.fa	3108346	3186582	OSBPL5	3.533677309	912
chr11.fa	3239174	3240043	MRGPRG	0	0
chr11.fa	3242800	3244361	C11orf36	0	0
chr11.fa	3249041	3253616	MRGPRE	0	0
chr11.fa	3380011	3400448	ZNF195	5.711144237	912
chr11.fa	3402191	3430378	LOC650368	0	0
chr11.fa	3647714	3658789	TRPC2	0	0
chr11.fa	3659736	3663546	ART5	0	0
chr11.fa	3666361	3685646	ART1	0	0
chr11.fa	3686817	3692614	CHRNA10	0	0
chr11.fa	3696240	3819022	NUP98	31.94662366	16416
chr11.fa	3819049	3847582	PGAP2	9.035328083	1672
chr11.fa	3848208	3862213	RHOG	13.01675814	1140
chr11.fa	3876933	4114440	STIM1	3.685036713	988
chr11.fa	4115924	4160106	RRM1	17.64233164	3738
chr11.fa	4388493	4389616	OR52B4	0	0
chr11.fa	4406127	4414926	TRIM21	18.39445987	2356
chr11.fa	4470525	4471591	OR52K2	0	0
chr11.fa	4510109	4511138	OR52K1	0	0
chr11.fa	4566421	4567374	OR52M1	0	0
chr11.fa	4592653	4599050	C11orf40	0	0
chr11.fa	4608021	4609135	OR52I2	0	0
chr11.fa	4615269	4616243	OR52I1	0	0
chr11.fa	4619902	4629437	TRIM68	16.27919337	3567
chr11.fa	4660945	4662068	OR51D1	0	0
chr11.fa	4665156	4676716	OR51E1	1.843723208	380
chr11.fa	4701401	4719076	OR51E2	3.698892499	684
chr11.fa	4790209	4791147	OR51F1	0	0
chr11.fa	4824663	4825610	OR52R1	0	0
chr11.fa	4842616	4843644	OR51F2	0	0

chr11.fa	4869467	4870438	OR51S1	0	0
chr11.fa	4903049	4904113	OR51T1	0	0
chr11.fa	4928600	4929538	OR51A7	0	0
chr11.fa	4935949	4936893	OR51G2	0	0
chr11.fa	4944604	4945569	OR51G1	0	0
chr11.fa	4967389	4968330	OR51A4	0	0
chr11.fa	4976002	4976943	OR51A2	0	0
chr11.fa	5009424	5013659	MMP26	0	0
chr11.fa	5020213	5021160	OR51L1	0	0
chr11.fa	5067756	5068691	OR52J3	0	0
chr11.fa	5079880	5080857	OR52E2	0	0
chr11.fa	5141850	5142847	OR52A4	0	0
chr11.fa	5152922	5153872	OR52A5	0	0
chr11.fa	5172661	5173599	OR52A1	0	0
chr11.fa	5220965	5221930	OR51V1	0	0
chr11.fa	5246696	5248301	HBB	0	0
chr11.fa	5254059	5255858	HBD	0	0
chr11.fa	5263185	5264822	HBBP1	0	0
chr11.fa	5269502	5271087	HBG1	0	0
chr11.fa	5274421	5276011	HBG2	0	0
chr11.fa	5289580	5291373	HBE1	0	0
chr11.fa	5322244	5323176	OR51B4	0	0
chr11.fa	5344528	5345582	OR51B2	0	0
chr11.fa	5363816	5364754	OR51B5	0	0
chr11.fa	5372738	5373676	OR51B6	0	0
chr11.fa	5410607	5411664	OR51M1	0	0
chr11.fa	5443341	5444436	OR51Q1	0	0
chr11.fa	5461772	5462783	OR51I1	0	0
chr11.fa	5474638	5475707	OR51I2	0	0
chr11.fa	5509915	5510978	OR52D1	0	0
chr11.fa	5528530	5531153	UBQLN3	0	0
chr11.fa	5535623	5537956	UBQLNL	0	0
chr11.fa	5565791	5566753	OR52H1	0	0
chr11.fa	5602107	5603114	OR52B6	0	0
chr11.fa	5617331	5634188	TRIM6	13.43017263	2018
chr11.fa	5641174	5646290	TRIM34	4.591687073	50
chr11.fa	5684425	5706339	TRIM5	11.44548186	2346
chr11.fa	5686626	5687610	TRIM78P	0	0
chr11.fa	5710817	5732093	TRIM22	20.1181498	4129
chr11.fa	5757678	5758768	OR56B1	0	0
chr11.fa	5775923	5776959	OR52N4	0	0
chr11.fa	5798864	5799897	OR52N5	0	0
chr11.fa	5809084	5810046	OR52N1	0	0

chr11.fa	5841566	5842531	OR52N2	0	0
chr11.fa	5862186	5863127	OR52E6	0	0
chr11.fa	5877979	5878932	OR52E8	0	0
chr11.fa	5905523	5906461	OR52E4	0	0
chr11.fa	5968577	5969524	OR56A3	0	0
chr11.fa	5988783	5989724	OR56A5	0	0
chr11.fa	6007122	6008215	OR52L1	0	0
chr11.fa	6023281	6024378	OR56A4	0	0
chr11.fa	6047901	6048971	OR56A1	0	0
chr11.fa	6129009	6129968	OR56B4	0	0
chr11.fa	6190585	6191556	OR52B2	0	0
chr11.fa	6220454	6221416	OR52W1	0	0
chr11.fa	6226798	6232365	C11orf42	0	0
chr11.fa	6232564	6255941	FAM160A2	6.883313632	1596
chr11.fa	6260330	6265707	CNGA4	0	0
chr11.fa	6280966	6293356	CCKBR	0	0
chr11.fa	6340176	6341740	PRKCDBP	0	0
chr11.fa	6411644	6416228	SMPD1	3.704163723	608
chr11.fa	6416355	6440644	APBB1	6.726984217	1210
chr11.fa	6452268	6462254	HPX	0.705289641	76
chr11.fa	6469843	6495205	TRIM3	4.522709355	912
chr11.fa	6496926	6502595	ARFIP2	28.39336752	4864
chr11.fa	6502677	6505911	FXC1	43.89949838	8284
chr11.fa	6518526	6593254	DNHD1	0.076959856	76
chr11.fa	6621152	6624811	RRP8	11.6237998	1292
chr11.fa	6624964	6632072	ILK	40.60950218	5320
chr11.fa	6632100	6633445	TAF10	102.5966752	5007
chr11.fa	6633997	6640692	TPP1	3.563798584	836
chr11.fa	6642558	6677074	DCHS1	0	0
chr11.fa	6701616	6704632	MRPL17	15.96457666	2508
chr11.fa	6734381	6743110	GVINP1	0.13117815	76
chr11.fa	6789238	6790188	OR2AG2	0	0
chr11.fa	6806248	6807245	OR2AG1	0	0
chr11.fa	6815756	6817139	OR6A2	0	0
chr11.fa	6866914	6867867	OR10A5	0	0
chr11.fa	6890986	6891897	OR10A2	0	0
chr11.fa	6897856	6898850	OR10A4	0	0
chr11.fa	6912805	6913731	OR2D2	0	0
chr11.fa	6942233	6943225	OR2D3	0	0
chr11.fa	6947654	6979278	ZNF215	1.564498994	380
chr11.fa	7020549	7041586	ZNF214	1.287082056	228
chr11.fa	7041700	7092757	NLRP14	0.299405468	76
chr11.fa	7110165	7112379	RBMXL2	0	0

chr11.fa	7273181	7490276	SYT9	3.470272027	912
chr11.fa	7506600	7532606	OLFML1	0.394287482	76
chr11.fa	7535001	7674991	PPFIBP2	158.6716446	37517
chr11.fa	7686326	7694821	CYB5R2	5.943831082	532
chr11.fa	7712444	7727941	OVCH2	0	0
chr11.fa	7817521	7818489	OR5P2	0	0
chr11.fa	7846584	7847519	OR5P3	0	0
chr11.fa	7870598	7871118	OR5E1P	0	0
chr11.fa	7872298	7927502	LOC283299	0.914632498	152
chr11.fa	7949265	7950209	OR10A6	0	0
chr11.fa	7960123	7961067	OR10A3	0	0
chr11.fa	7981156	7985059	NLRP10	0	0
chr11.fa	8008867	8017719	EIF3F	79.22106023	6612
chr11.fa	8060180	8127596	TUB	2.448709004	1064
chr11.fa	8127655	8190590	RIC3	0.199101624	76
chr11.fa	8245857	8285406	LMO1	0	0
chr11.fa	8413418	8615503	STK33	5.49683137	988
chr11.fa	8633584	8680383	TRIM66	4.107638193	2660
chr11.fa	8703995	8711419	RPL27A	77.24164068	25023
chr11.fa	8705774	8705903	SNORA3	0	0
chr11.fa	8706986	8707116	SNORA45	0	0
chr11.fa	8714899	8932498	ST5	6.232392891	1947
chr11.fa	8932701	8941622	C11orf17	3.066646949	304
chr11.fa	8941627	8954553	C11orf16	0.595798808	76
chr11.fa	8959119	8964580	ASCL3	0	0
chr11.fa	8968840	8985989	TMEM9B	5.223480805	608
chr11.fa	9002123	9025596	NRIP3	0.300459712	76
chr11.fa	9041047	9113150	SCUBE2	28.93735773	9044
chr11.fa	9115910	9117737	FLJ46111	0	0
chr11.fa	9160375	9286871	DENND5A	18.91540731	6232
chr11.fa	9302201	9336296	TMEM41B	2.577929272	684
chr11.fa	9406169	9469674	IPO7	36.2068261	14886
chr11.fa	9450313	9450501	SNORA23	0	0
chr11.fa	9481103	9482245	LOC644656	0	0
chr11.fa	9482512	9550071	ZNF143	4.660815398	912
chr11.fa	9595228	9611313	WEE1	35.8522987	9634
chr11.fa	9685628	9774507	SWAP70	24.5593811	7904
chr11.fa	9776317	9781080	LOC440028	0.495344358	76
chr11.fa	9779840	9832866	LOC283104	0.472904008	76
chr11.fa	9800214	10315754	SBF2	20.4374353	9704
chr11.fa	10326642	10328923	ADM	1.579860844	152
chr11.fa	10471868	10529126	AMPD3	4.652080229	1748
chr11.fa	10529434	10530723	MTRNR2L8	0	0

chr11.fa	10533225	10562774	RNF141	50.44801405	13680
chr11.fa	10562787	10621479	LOC100129827	0	0
chr11.fa	10579413	10590365	LYVE1	0	0
chr11.fa	10594638	10715535	MRVI1	0	0
chr11.fa	10772811	10801290	CTR9	30.81331071	8816
chr11.fa	10818593	10830582	EIF4G2	295.3046069	85470
chr11.fa	10823014	10823155	SNORD97	0	0
chr11.fa	10874251	10879620	ZBED5	8.35172576	1520
chr11.fa	10879764	10900823	LOC729013	22.62484225	1672
chr11.fa	11292421	11643561	GALNTL4	2.730644133	456
chr11.fa	11373321	11374904	CSNK2A1P	0.722609373	76
chr11.fa	11678198	11678269	MIR4299	0	0
chr11.fa	11862970	11980872	USP47	33.08611147	17085
chr11.fa	11984543	12030917	DKK3	0	0
chr11.fa	12132138	12285331	MICAL2	11.43147546	2964
chr11.fa	12308447	12380691	MICALCL	8.272506808	1672
chr11.fa	12399026	12556903	PARVA	24.88920906	14409
chr11.fa	12695969	12966284	TEAD1	21.51607814	13452
chr11.fa	13030970	13033653	RASSF10	0.426517246	76
chr11.fa	13299325	13408812	ARNTL	3.194963578	608
chr11.fa	13409556	13484838	BTBD10	20.08893216	3344
chr11.fa	13513601	13517567	PTH	0	0
chr11.fa	13690206	13753893	FAR1	14.32115993	5016
chr11.fa	13983914	14289655	SPON1	0	0
chr11.fa	14299466	14386052	RRAS2	6.238718359	1140
chr11.fa	14479049	14521441	COPB1	49.00369894	11476
chr11.fa	14526422	14665180	PSMA1	47.97882258	6553
chr11.fa	14665269	14893604	PDE3B	1.879416918	760
chr11.fa	14899556	14913751	CYP2R1	3.537141256	380
chr11.fa	14988215	14993832	CALCA	0	0
chr11.fa	15095146	15100177	CALCB	0	0
chr11.fa	15133970	15268754	INSC	0.758001871	152
chr11.fa	15987995	16497935	SOX6	2.200509703	1368
chr11.fa	16760148	16779901	C11orf58	29.20558768	7718
chr11.fa	16809207	17035963	PLEKHA7	25.19087362	8042
chr11.fa	17095939	17099220	RPS13	856.4064178	30081
chr11.fa	17108124	17191354	PIK3C2A	17.07770835	9398
chr11.fa	17298286	17353070	NUCB2	6.390529582	684
chr11.fa	17373317	17398871	DKFZp686O24166	0	0
chr11.fa	17406796	17410878	KCNJ11	0	0
chr11.fa	17414432	17498449	ABCC8	0	0
chr11.fa	17515442	17565963	USH1C	7.548391371	1664
chr11.fa	17741110	17743678	MYOD1	0	0

chr11.fa	17757495	17804602	KCNC1	0	0
chr11.fa	17809597	18034637	SERGEF	23.77201099	2355
chr11.fa	18042084	18062335	TPH1	0	0
chr11.fa	18101890	18127638	SAAL1	6.728640888	684
chr11.fa	18134019	18137679	SAA3P	0	0
chr11.fa	18142502	18160027	MRGPRX3	0	0
chr11.fa	18194384	18195827	MRGPRX4	0	0
chr11.fa	18230685	18235111	LOC494141	0	0
chr11.fa	18258264	18258384	SAA4	0	0
chr11.fa	18259780	18267062	SAA2	0	0
chr11.fa	18287808	18291523	SAA1	0	0
chr11.fa	18300217	18343721	HPS5	12.66569468	4104
chr11.fa	18343816	18388590	GTF2H1	18.56118112	4332
chr11.fa	18415936	18429765	LDHA	63.21566924	10716
chr11.fa	18433853	18472793	LDHC	0	0
chr11.fa	18477374	18501147	LDHAL6A	0	0
chr11.fa	18501858	18548489	TSG101	40.1846416	4093
chr11.fa	18553244	18610281	UEVLD	12.9638953	1824
chr11.fa	18627948	18656020	SPTY2D1	13.37444827	5092
chr11.fa	18686477	18688026	LOC100132715	0.738423043	76
chr11.fa	18720351	18726332	TMEM86A	0	0
chr11.fa	18727364	18747777	IGSF22	0.279224214	76
chr11.fa	18749475	18813389	PTPN5	0	0
chr11.fa	18955360	18956549	MRGPRX1	0	0
chr11.fa	19076003	19082228	MRGPRX2	0	0
chr11.fa	19138692	19197967	ZDHHC13	6.57743209	1063
chr11.fa	19203578	19223589	CSRP3	0	0
chr11.fa	19245610	19262507	E2F8	0.922765242	228
chr11.fa	19372271	20143147	NAV2	10.47557682	7455
chr11.fa	19532440	19545625	FLJ13439	0	0
chr11.fa	19732480	19736146	LOC100126784	0	0
chr11.fa	20177760	20181870	DBX1	0	0
chr11.fa	20385231	20405329	HTATIP2	13.83982196	2052
chr11.fa	20409076	20530879	PRMT3	5.813707177	1055
chr11.fa	20620946	20676610	SLC6A5	0	0
chr11.fa	20691117	21597229	NELL1	0	0
chr11.fa	22214722	22304913	ANO5	0.807250155	357
chr11.fa	22359667	22401046	SLC17A6	0	0
chr11.fa	22644079	22647387	FANCF	14.18215024	3116
chr11.fa	22688160	22834547	GAS2	1.803963125	304
chr11.fa	22843598	22851382	SVIP	20.36273454	1878
chr11.fa	22868468	22881972	LOC100500938	0	0
chr11.fa	24518556	25104186	LUZP2	0.653179836	228

chr11.fa	26353678	26684836	ANO3	0	0
chr11.fa	26580579	26593815	MUC15	2.74901811	608
chr11.fa	26688566	26743574	SLC5A12	2.562718028	1064
chr11.fa	27015628	27018632	FIBIN	0.380883515	76
chr11.fa	27062509	27149354	BBOX1	29.13103753	3648
chr11.fa	27360061	27384795	CCDC34	1.944629477	380
chr11.fa	27387508	27494334	LGR4	8.289525328	2888
chr11.fa	27515965	27528326	LIN7C	20.75024474	6685
chr11.fa	27528399	27719718	BDNF-AS1	1.65350736	304
chr11.fa	27676442	27743605	BDNF	0	0
chr11.fa	28042163	28129746	KIF18A	0	0
chr11.fa	28078362	28078457	MIR610	0	0
chr11.fa	28129798	28355054	METT5D1	2.073548531	608
chr11.fa	30031765	30038488	KCNA4	0	0
chr11.fa	30252563	30256824	FSHB	0	0
chr11.fa	30344649	30359165	C11orf46	14.87343349	1748
chr11.fa	30406040	30607930	MPPED2	6.828342306	3268
chr11.fa	30886486	30953499	DCDC5	0.645800124	152
chr11.fa	31284171	31391321	DCDC1	0.670951388	76
chr11.fa	31391377	31454382	DNAJC24	1.616156979	304
chr11.fa	31455007	31531169	IMMP1L	9.976467303	416
chr11.fa	31531297	31805329	ELP4	5.728162758	596
chr11.fa	31806340	31839509	PAX6	0	0
chr11.fa	31838114	31908587	DKFZp686K1684	0	0
chr11.fa	32112477	32127272	RCN1	7.733787815	1292
chr11.fa	32409322	32457081	WT1	0	0
chr11.fa	32457285	32461620	WT1-AS	0	0
chr11.fa	32605391	32623368	EIF3M	101.7419841	7073
chr11.fa	32623626	32816187	CCDC73	0.299254861	76
chr11.fa	32851489	32876105	PRRG4	2.911070567	380
chr11.fa	32914792	33001814	QSER1	5.927414988	3648
chr11.fa	33037410	33055128	DEPDC7	1.06026886	152
chr11.fa	33060963	33095109	TCP11L1	5.325591925	1064
chr11.fa	33097696	33101000	NCRNA00294	2.424310772	532
chr11.fa	33106130	33183037	CSTF3	11.79850319	2492
chr11.fa	33183203	33213142	LOC338739	0	0
chr11.fa	33279168	33375939	HIPK3	68.29517036	21508
chr11.fa	33563877	33695646	C11orf41	0	0
chr11.fa	33719654	33722286	C11orf91	0	0
chr11.fa	33724556	33758025	CD59	4.593946169	2432
chr11.fa	33762490	33796071	FBXO3	10.24529968	1900
chr11.fa	33880123	33913836	LMO2	1.89507998	304
chr11.fa	34073230	34124157	CAPRIN1	57.69459025	21307

chr11.fa	34127111	34168458	NAT10	15.01214196	4028
chr11.fa	34172534	34379555	ABTB2	1.634079138	532
chr11.fa	34460472	34493607	CAT	104.5077195	15960
chr11.fa	34500342	34535330	ELF5	15.66110482	2660
chr11.fa	34642588	34684834	EHF	185.7377181	67213
chr11.fa	34903843	34916657	APIP	30.09371346	2184
chr11.fa	34937677	35017675	PDHX	15.7008649	2943
chr11.fa	35160417	35253949	CD44	2.995259529	1140
chr11.fa	35272752	35441532	SLC1A2	0	0
chr11.fa	35453376	35547176	PAMR1	0	0
chr11.fa	35639735	35642421	FJX1	0	0
chr11.fa	35684353	35830930	TRIM44	27.99606791	8062
chr11.fa	35965612	36252841	LDLRAD3	5.397280558	1064
chr11.fa	36293842	36310999	COMMD9	11.90573493	2351
chr11.fa	36317725	36486754	PRR5L	2.987879817	836
chr11.fa	36510723	36531822	TRAF6	12.89567061	2204
chr11.fa	36589563	36601310	RAG1	0.695801439	304
chr11.fa	36613493	36619829	RAG2	0	0
chr11.fa	36616093	36680817	C11orf74	13.95865039	760
chr11.fa	40135753	40315664	LRRC4C	0	0
chr11.fa	43283054	43290919	LOC399881	0.608148531	76
chr11.fa	43333505	43366080	API5	29.28766816	7448
chr11.fa	43380435	43516483	TTC17	6.576829664	1976
chr11.fa	43581206	43581303	MIR670	0	0
chr11.fa	43602944	43603033	MIR129-2	0	0
chr11.fa	43702143	43878169	HSD17B12	8.095393715	1368
chr11.fa	43902357	43941825	ALKBH3	11.49939894	1198
chr11.fa	43918853	43921424	LOC729799	0	0
chr11.fa	43964106	43965433	C11orf96	0	0
chr11.fa	44069531	44081527	ACCSL	0	0
chr11.fa	44087729	44105569	ACCS	2.009390217	301
chr11.fa	44117099	44266980	EXT2	1.485731861	380
chr11.fa	44282278	44331716	ALX4	0	0
chr11.fa	44587141	44641315	CD82	4.738227074	532
chr11.fa	44785976	44953898	TSPAN18	0.550918109	152
chr11.fa	44953978	44972608	TP53I11	9.300545904	2204
chr11.fa	44995453	44999578	LOC221122	0	0
chr11.fa	45115564	45246903	PRDM11	2.567537432	380
chr11.fa	45261853	45307884	SYT13	1.109818356	380
chr11.fa	45670427	45687172	CHST1	0.421095416	76
chr11.fa	45792983	45793909	DKFZp779M0652	0	0
chr11.fa	45825623	45834567	SLC35C1	4.364271452	1140
chr11.fa	45868669	45904799	CRY2	21.22555845	6156

chr11.fa	45907202	45928016	MAPK8IP1	6.754997003	1368
chr11.fa	45928086	45928833	C11orf94	0	0
chr11.fa	45931220	45939674	PEX16	6.20091616	730
chr11.fa	45943196	45950647	GYLTL1B	3.622233856	608
chr11.fa	45950870	46142985	PHF21A	10.60344163	5167
chr11.fa	46299228	46342972	CREB3L1	6.856505698	1216
chr11.fa	46354455	46402104	DGKZ	1.597481789	575
chr11.fa	46402618	46405375	MDK	5.713553939	456
chr11.fa	46406640	46408107	CHRM4	0	0
chr11.fa	46417964	46612914	AMBRA1	12.91088186	4306
chr11.fa	46473355	46473439	MIR3160-1	0	0
chr11.fa	46624856	46638777	HARBI1	5.264295131	532
chr11.fa	46638826	46696368	ATG13	23.11310811	7480
chr11.fa	46698632	46722120	ARHGAP1	36.56617291	8119
chr11.fa	46722317	46727466	ZNF408	1.803210093	304
chr11.fa	46740743	46761056	F2	0	0
chr11.fa	46765084	46867843	CKAP5	22.21549413	9880
chr11.fa	46783939	46784049	SNORD67	0	0
chr11.fa	46878268	46940173	LRP4	0.695650833	380
chr11.fa	46958251	47185851	C11orf49	17.13659545	2204
chr11.fa	47185932	47198419	ARFGAP2	22.60782373	4026
chr11.fa	47199073	47208010	PACSIN3	11.26369997	1596
chr11.fa	47236493	47260769	DDB2	4.865338852	597
chr11.fa	47260853	47270448	ACP2	6.958164999	1064
chr11.fa	47270458	47290401	NR1H3	10.73853555	1292
chr11.fa	47290927	47351582	MADD	25.99044285	10822
chr11.fa	47352957	47374253	MYBPC3	0	0
chr11.fa	47376409	47400127	SPI1	0	0
chr11.fa	47430046	47438051	SLC39A13	6.073503169	988
chr11.fa	47440320	47448024	PSMC3	39.08144993	4100
chr11.fa	47459315	47470730	RAPSN	0	0
chr11.fa	47487489	47574792	CELF1	25.12581167	9793
chr11.fa	47586982	47593748	PTPMT1	5.027541914	456
chr11.fa	47595014	47600561	KBTBD4	10.1867138	836
chr11.fa	47600568	47606115	NDUFS3	44.43912101	2812
chr11.fa	47608230	47610746	FAM180B	0	0
chr11.fa	47611216	47615961	C1QTNF4	0	0
chr11.fa	47638858	47664206	MTCH2	37.58276592	6558
chr11.fa	47681143	47736302	AGBL2	0.33750888	76
chr11.fa	47738069	47788993	FNBP4	16.14470188	4408
chr11.fa	47799670	47870057	NUP160	11.69488601	4180
chr11.fa	48002110	48192394	PTPRJ	3.413794637	2052
chr11.fa	48238362	48239291	OR4B1	0	0

chr11.fa	48266656	48267567	OR4X2	0	0
chr11.fa	48285413	48286330	OR4X1	0	0
chr11.fa	48327775	48328704	OR4S1	0	0
chr11.fa	48346493	48347482	OR4C3	0	0
chr11.fa	48366900	48373999	OR4C45	0	0
chr11.fa	48510345	48511274	OR4A47	0	0
chr11.fa	49168187	49230222	FOLH1	18.85335748	3420
chr11.fa	49580080	49831969	LOC440040	0	0
chr11.fa	49973943	49974971	OR4C13	0	0
chr11.fa	50003009	50004071	OR4C12	0	0
chr11.fa	50238999	50257633	LOC441601	0	0
chr11.fa	50368318	50379802	LOC646813	0	0
chr11.fa	51411378	51412448	OR4A5	0	0
chr11.fa	51515282	51516211	OR4C46	0	0
chr11.fa	55029658	55038595	TRIM48	0	0
chr11.fa	55110677	55111663	OR4A16	0	0
chr11.fa	55135360	55136394	OR4A15	0	0
chr11.fa	55321783	55322895	OR4C15	0	0
chr11.fa	55339604	55340536	OR4C16	0	0
chr11.fa	55370917	55371849	OR4C11	0	0
chr11.fa	55405834	55406772	OR4P4	0	0
chr11.fa	55418380	55419315	OR4S2	0	0
chr11.fa	55432643	55433572	OR4C6	0	0
chr11.fa	55540914	55541858	OR5D13	0	0
chr11.fa	55563032	55563976	OR5D14	0	0
chr11.fa	55578943	55579878	OR5L1	0	0
chr11.fa	55587106	55588047	OR5D18	0	0
chr11.fa	55594695	55595630	OR5L2	0	0
chr11.fa	55606228	55607214	OR5D16	0	0
chr11.fa	55650773	55659284	SPRYD5	0	0
chr11.fa	55681126	55682058	OR5W2	0	0
chr11.fa	55702932	55703876	OR5I1	0	0
chr11.fa	55735034	55735939	OR10AG1	0	0
chr11.fa	55746179	55753881	OR7E5P	0	0
chr11.fa	55761157	55762101	OR5F1	0	0
chr11.fa	55797895	55798869	OR5AS1	0	0
chr11.fa	55860784	55861716	OR8I2	0	0
chr11.fa	55872519	55873457	OR8H2	0	0
chr11.fa	55889849	55890787	OR8H3	0	0
chr11.fa	55904247	55905194	OR8J3	0	0
chr11.fa	55926870	55927793	OR8K5	0	0
chr11.fa	55944094	55945032	OR5J2	0	0
chr11.fa	55999582	56000661	OR5T2	0	0

chr11.fa	56019676	56020698	OR5T3	0	0
chr11.fa	56043115	56044095	OR5T1	0	0
chr11.fa	56057603	56058538	OR8H1	0	0
chr11.fa	56085783	56086721	OR8K3	0	0
chr11.fa	56113515	56114474	OR8K1	0	0
chr11.fa	56127723	56128673	OR8J1	0	0
chr11.fa	56143898	56144029	OR8U1	0	0
chr11.fa	56184734	56185708	OR5R1	0	0
chr11.fa	56229945	56230877	OR5M9	0	0
chr11.fa	56237050	56237973	OR5M3	0	0
chr11.fa	56257911	56258846	OR5M8	0	0
chr11.fa	56309816	56310733	OR5M11	0	0
chr11.fa	56344250	56345197	OR5M10	0	0
chr11.fa	56380031	56380978	OR5M1	0	0
chr11.fa	56408965	56409915	OR5AP2	0	0
chr11.fa	56431162	56432094	OR5AR1	0	0
chr11.fa	56508508	56508560	OR8U8	0	0
chr11.fa	56510304	56511287	OR9G4	0	0
chr11.fa	56756389	56757318	OR5AK2	0	0
chr11.fa	56805009	56805935	OR5AK4P	0	0
chr11.fa	56949221	56959188	LRRC55	0	0
chr11.fa	57001052	57004927	APLNR	0	0
chr11.fa	57067103	57092413	TNKS1BP1	20.45355019	7904
chr11.fa	57093459	57103351	SSRP1	38.90448744	7282
chr11.fa	57105949	57137549	P2RX3	0	0
chr11.fa	57144242	57148623	PRG3	0	0
chr11.fa	57154834	57158130	PRG2	0	0
chr11.fa	57174427	57195053	SLC43A3	0	0
chr11.fa	57228339	57244384	RTN4RL2	0.906198541	76
chr11.fa	57252004	57283192	SLC43A1	2.168581152	380
chr11.fa	57295936	57298232	TIMM10	25.47341118	1140
chr11.fa	57310114	57317747	SMTNL1	0	0
chr11.fa	57319129	57335453	UBE2L6	43.58066469	4028
chr11.fa	57365027	57382326	SERPING1	1.154397842	152
chr11.fa	57408671	57408759	MIR130A	0	0
chr11.fa	57412560	57417417	YPEL4	0	0
chr11.fa	57425216	57429337	CLP1	5.536139633	608
chr11.fa	57435474	57468659	ZDHHC5	22.08898478	6688
chr11.fa	57471187	57479673	MED19	6.80409468	684
chr11.fa	57505308	57508445	TMX2	10.85284578	1042
chr11.fa	57508722	57510883	C11orf31	52.18842128	4484
chr11.fa	57510986	57519253	BTBD18	0	0
chr11.fa	57529234	57583473	CTNND1	102.2665461	2526

chr11.fa	57791353	57949038	OR9Q1	0	0
chr11.fa	57798425	57799378	OR6Q1	0	0
chr11.fa	57885972	57886916	OR9I1	0	0
chr11.fa	57957906	57958990	OR9Q2	0	0
chr11.fa	57970676	57971653	OR1S2	0	0
chr11.fa	57982217	57983194	OR1S1	0	0
chr11.fa	57995354	57996390	OR10Q1	0	0
chr11.fa	58034264	58035732	OR10W1	0	0
chr11.fa	58125598	58126542	OR5B17	0	0
chr11.fa	58169938	58170882	OR5B3	0	0
chr11.fa	58189738	58190786	OR5B2	0	0
chr11.fa	58206593	58207646	OR5B12	0	0
chr11.fa	58274649	58275578	OR5B21	0	0
chr11.fa	58294344	58345639	LPXN	6.742948493	912
chr11.fa	58385042	58389023	ZFP91	22.70827818	6004
chr11.fa	58390146	58390238	CNTF	0	0
chr11.fa	58476230	58499447	GLYAT	21.26486671	3496
chr11.fa	58601540	58611997	GLYATL2	0	0
chr11.fa	58701116	58825925	LOC283194	0.439770607	76
chr11.fa	58710722	58724516	GLYATL1	36.41345805	5000
chr11.fa	58874658	58894888	FAM111B	0.321393998	76
chr11.fa	58910318	58922511	FAM111A	17.40542782	4528
chr11.fa	58939812	58975982	DTX4	39.84201211	15124
chr11.fa	58976061	58980494	MPEG1	0.258139322	76
chr11.fa	59131932	59132867	OR5AN1	0	0
chr11.fa	59189452	59190426	OR5A2	0	0
chr11.fa	59210642	59211589	OR5A1	0	0
chr11.fa	59224434	59225378	OR4D6	0	0
chr11.fa	59244903	59245838	OR4D10	0	0
chr11.fa	59271049	59271984	OR4D11	0	0
chr11.fa	59282386	59283330	OR4D9	0	0
chr11.fa	59341871	59383617	OSBP	85.00976675	28691
chr11.fa	59362550	59362631	MIR3162	0	0
chr11.fa	59404192	59436511	PATL1	19.69163255	5396
chr11.fa	59480389	59481318	OR10V1	0	0
chr11.fa	59522532	59573355	STX3	9.89935684	4256
chr11.fa	59573608	59578345	MRPL16	16.46218011	1292
chr11.fa	59596746	59612974	GIF	0	0
chr11.fa	59620281	59634041	TCN1	0	0
chr11.fa	59807748	59815516	PLAC1L	0	0
chr11.fa	59824101	59838588	MS4A3	0	0
chr11.fa	59856137	59865940	MS4A2	0	0
chr11.fa	59939080	59950674	MS4A6A	0	0

chr11.fa	60048139	60076445	MS4A4A	0	0
chr11.fa	60102355	60108441	MS4A6E	0	0
chr11.fa	60145958	60163426	MS4A7	0	0
chr11.fa	60163487	60185228	MS4A14	0	0
chr11.fa	60197062	60215265	MS4A5	0	0
chr11.fa	60223282	60238225	MS4A1	0	0
chr11.fa	60260251	60274901	MS4A12	0	0
chr11.fa	60282886	60310191	MS4A13	0	0
chr11.fa	60383224	60454621	C11orf64	0	0
chr11.fa	60467047	60483285	MS4A8B	0	0
chr11.fa	60524340	60544204	MS4A15	0	0
chr11.fa	60552821	60568778	MS4A10	0	0
chr11.fa	60609429	60618397	CCDC86	12.36749407	1520
chr11.fa	60618562	60623444	GPR44	0	0
chr11.fa	60635015	60643164	ZP1	0	0
chr11.fa	60658020	60674061	PRPF19	48.11677801	7476
chr11.fa	60681371	60690915	TMEM109	7.189797599	1140
chr11.fa	60691913	60704554	TMEM132A	0.332388263	76
chr11.fa	60704632	60719257	SLC15A3	0	0
chr11.fa	60739115	60787848	CD6	0	0
chr11.fa	60869930	60895323	CD5	0	0
chr11.fa	60897728	60928916	VPS37C	23.81056622	4256
chr11.fa	60970984	60980350	PGA3	0	0
chr11.fa	60989821	60999179	PGA4	0	0
chr11.fa	61008644	61018929	PGA5	0	0
chr11.fa	61025758	61062788	VWCE	0	0
chr11.fa	61066919	61100653	DDB1	72.51395605	20824
chr11.fa	61100667	61116219	DAK	11.07047199	3099
chr11.fa	61116232	61129472	CYBASC3	14.13561288	2888
chr11.fa	61129756	61136975	TMEM138	5.873497907	608
chr11.fa	61159832	61166335	TMEM216	6.937080107	608
chr11.fa	61170120	61197464	CPSF7	18.23105195	4560
chr11.fa	61197597	61214239	SDHAF2	24.41329292	1976
chr11.fa	61248585	61258400	C11orf66	0	0
chr11.fa	61276272	61278490	LRRC10B	0.515826825	76
chr11.fa	61282785	61348298	SYT7	19.19463152	5776
chr11.fa	61382508	61406921	RPLP0P2	0.649414677	152
chr11.fa	61447905	61514474	DAGLA	0.795201645	304
chr11.fa	61520121	61555989	C11orf9	0.191270093	76
chr11.fa	61521501	61525136	DKFZP434K028	0	0
chr11.fa	61556602	61560085	C11orf10	142.3880836	3829
chr11.fa	61559967	61560027	MIR611	0	0
chr11.fa	61560150	61564710	FEN1	4.071492664	608

chr11.fa	61567097	61584529	FADS1	2.848568922	836
chr11.fa	61582633	61582712	MIR1908	0	0
chr11.fa	61595713	61634825	FADS2	17.08373261	3572
chr11.fa	61640998	61659006	FADS3	0	0
chr11.fa	61664773	61684997	RAB3IL1	1.503352807	228
chr11.fa	61717356	61731756	BEST1	0	0
chr11.fa	61731936	61735132	FTH1	1530.505027	106704
chr11.fa	61891445	61920635	INCENP	3.988508553	1098
chr11.fa	61957710	61961009	SCGB1D1	0	0
chr11.fa	61976140	61981411	SCGB2A1	0	0
chr11.fa	62009724	62012280	SCGB1D2	0	0
chr11.fa	62037630	62040628	SCGB2A2	0	0
chr11.fa	62063754	62066536	SCGB1D4	0	0
chr11.fa	62104774	62160887	ASRGL1	27.91730078	4484
chr11.fa	62186507	62190678	SCGB1A1	7.596886623	228
chr11.fa	62201016	62314332	AHNAK	35.88964908	45904
chr11.fa	62327073	62341460	EEF1G	496.1392584	50666
chr11.fa	62342517	62359109	TUT1	11.34081043	2128
chr11.fa	62360675	62369303	MTA2	34.78705983	7068
chr11.fa	62369691	62380212	EML3	4.937780517	1059
chr11.fa	62380238	62382592	ROM1	3.706272212	456
chr11.fa	62382768	62389448	B3GAT3	2.38123735	228
chr11.fa	62392298	62414104	GANAB	12.54611322	3268
chr11.fa	62414320	62420774	INTS5	12.19520038	2660
chr11.fa	62430289	62439125	C11orf48	19.01345206	1520
chr11.fa	62432779	62434923	METTL12	4.969407855	456
chr11.fa	62432894	62433042	SNORA57	0	0
chr11.fa	62439242	62441162	C11orf83	7.489203067	912
chr11.fa	62443972	62446527	UBXN1	47.14476449	4035
chr11.fa	62453876	62457371	LRRN4CL	0	0
chr11.fa	62457747	62477046	BSCL2	12.52141378	2002
chr11.fa	62475201	62476618	GNG3	0	0
chr11.fa	62482220	62494821	HNRNPUL2	62.82318904	12464
chr11.fa	62495952	62506108	TTC9C	14.06889425	1064
chr11.fa	62518435	62521656	ZBTB3	4.233545121	836
chr11.fa	62529011	62534187	POLR2G	56.71579944	3152
chr11.fa	62538875	62554813	TAF6L	5.112333302	684
chr11.fa	62554874	62557786	TMEM179B	10.76428924	684
chr11.fa	62557873	62559486	TMEM223	5.380111432	304
chr11.fa	62559598	62572964	NXF1	7.829874681	2156
chr11.fa	62574369	62599560	STX5	26.61214596	3170
chr11.fa	62600383	62607628	WDR74	11.72244697	1064
chr11.fa	62619460	62623360	SNHG1	0	0

chr11.fa	62620382	62620507	SNORD22	0	0
chr11.fa	62620798	62620865	SNORD31	0	0
chr11.fa	62621135	62621204	SNORD30	0	0
chr11.fa	62621376	62621440	SNORD29	0	0
chr11.fa	62622093	62622167	SNORD28	0	0
chr11.fa	62622484	62622555	SNORD27	0	0
chr11.fa	62622764	62622838	SNORD26	0	0
chr11.fa	62623037	62623103	SNORD25	0	0
chr11.fa	62623484	62656355	SLC3A2	8.215426993	1583
chr11.fa	62676151	62689012	CHRM1	4.460057104	836
chr11.fa	62744069	62752469	SLC22A6	1.563745962	228
chr11.fa	62760296	62783317	SLC22A8	4.720756734	599
chr11.fa	62847412	62911693	SLC22A24	0	0
chr11.fa	62931296	62997124	SLC22A25	0	0
chr11.fa	63057430	63079246	SLC22A10	0	0
chr11.fa	63137261	63177712	SLC22A9	0	0
chr11.fa	63228876	63258680	HRASLS5	0.367630154	76
chr11.fa	63273524	63284246	LGALS12	0	0
chr11.fa	63304273	63313930	RARRES3	25.43575958	1292
chr11.fa	63320242	63330855	HRASLS2	6.170343066	304
chr11.fa	63341944	63381941	PLA2G16	13.51496402	988
chr11.fa	63396437	63439083	ATL3	18.63889401	2792
chr11.fa	63448922	63527363	RTN3	2.990289519	988
chr11.fa	63527364	63536113	C11orf95	0.61522703	228
chr11.fa	63580923	63595190	C11orf84	3.502049971	456
chr11.fa	63606400	63678492	MARK2	34.89911098	12001
chr11.fa	63678693	63684316	RCOR2	0.43962	76
chr11.fa	63706442	63724446	NAA40	5.832834186	1292
chr11.fa	63742079	63744015	COX8A	288.9741692	9728
chr11.fa	63753325	63765892	OTUB1	21.04392716	3496
chr11.fa	63766030	63933585	MACROD1	9.980533675	833
chr11.fa	63871362	63886645	FLRT1	0	0
chr11.fa	63953587	63972020	STIP1	52.20242768	7629
chr11.fa	63974152	63991270	FERMT3	0	0
chr11.fa	63991364	63993726	TRPT1	22.40390271	1437
chr11.fa	63993738	63997488	NUDT22	16.38085267	1216
chr11.fa	63997753	64001753	DNAJC4	16.92424046	1444
chr11.fa	64002266	64006259	VEGFB	46.28660938	3436
chr11.fa	64008413	64011607	FKBP2	11.45632552	712
chr11.fa	64011951	64014413	PPP1R14B	23.20889376	1521
chr11.fa	64018995	64036924	PLCB3	11.18508344	4254
chr11.fa	64037300	64052152	BAD	37.88518352	2757
chr11.fa	64052177	64056972	GPR137	4.508100537	760

chr11.fa	64058793	64067503	KCNK4	0	0
chr11.fa	64067863	64072239	C11orf20	0	0
chr11.fa	64073044	64084164	ESRRA	15.26139551	2204
chr11.fa	64084211	64085033	TRMT112	109.1122081	3876
chr11.fa	64085569	64089283	PRDX5	128.4583496	7540
chr11.fa	64107690	64125006	CCDC88B	2.093729785	684
chr11.fa	64126625	64139687	RPS6KA4	8.757007507	1824
chr11.fa	64136074	64136175	MIR1237	0	0
chr11.fa	64323098	64338999	SLC22A11	1.366903433	228
chr11.fa	64358282	64369820	SLC22A12	12.13872299	2584
chr11.fa	64373646	64490660	NRXN2	0	0
chr11.fa	64494383	64512928	RASGRP2	0.443234553	76
chr11.fa	64513861	64528187	PYGM	0	0
chr11.fa	64532076	64546316	SF1	55.79770299	14060
chr11.fa	64556609	64570713	MAP4K2	4.260804874	836
chr11.fa	64570986	64578766	MEN1	2.928239693	684
chr11.fa	64591662	64612041	CDC42BPG	27.05974809	8874
chr11.fa	64620208	64646191	EHD1	11.2573745	2584
chr11.fa	64658609	64658718	MIR192	0	0
chr11.fa	64658827	64658911	MIR194-2	0	0
chr11.fa	64662004	64684722	ATG2A	5.747289767	2432
chr11.fa	64692143	64701942	PPP2R5B	3.756875953	684
chr11.fa	64701951	64703360	GPHA2	0	0
chr11.fa	64705929	64727609	C11orf85	0	0
chr11.fa	64755417	64764517	BATF2	2.67431735	380
chr11.fa	64787891	64789657	ARL2	20.43939319	741
chr11.fa	64794790	64794879	ARL2-SNX15	0	0
chr11.fa	64808376	64812294	SAC3D1	0.733302426	76
chr11.fa	64812301	64826009	NAALADL1	0	0
chr11.fa	64844927	64851615	CDCA5	0.888728202	152
chr11.fa	64851694	64855874	ZFPL1	10.73582463	988
chr11.fa	64863683	64879180	C11orf2	38.30567651	6384
chr11.fa	64879341	64883707	TM7SF2	13.71225836	1444
chr11.fa	64883875	64885170	ZNHIT2	5.299085203	456
chr11.fa	64888099	64889654	FAU	243.9445206	9022
chr11.fa	64889673	64894750	MRPL49	39.58462582	5396
chr11.fa	64894842	64902003	SYVN1	3.866969211	760
chr11.fa	64937707	64940688	SPDYC	0	0
chr11.fa	64948686	64979477	CAPN1	106.1648414	23946
chr11.fa	64981311	65010228	SLC22A20	0	0
chr11.fa	65029432	65065088	POLA2	3.149480454	518
chr11.fa	65082331	65089900	CDC42EP2	5.77786286	760
chr11.fa	65101346	65120451	DPF2	19.95579613	3192

chr11.fa	65122282	65125082	TIGD3	0	0
chr11.fa	65142663	65150142	SLC25A45	1.969931347	299
chr11.fa	65154041	65180995	FRMD8	6.713279038	1672
chr11.fa	65190269	65194003	NEAT1	6.742044855	1672
chr11.fa	65211929	65212028	MIR612	0	0
chr11.fa	65265233	65273939	MALAT1	0	0
chr11.fa	65292548	65306029	SCYL1	33.93477837	5624
chr11.fa	65306183	65325699	LTBP3	1.509678274	456
chr11.fa	65337943	65339239	SSSCA1	21.00205859	912
chr11.fa	65339820	65341669	FAM89B	15.70101551	1520
chr11.fa	65343509	65360116	EHBP1L1	3.314545038	1140
chr11.fa	65360326	65363467	KCNK7	0	0
chr11.fa	65365226	65381720	MAP3K11	15.69709974	3724
chr11.fa	65383783	65404910	PCNXL3	2.44057626	1064
chr11.fa	65405578	65418391	SIPA1	2.77974181	684
chr11.fa	65421067	65430443	RELA	26.47584719	4532
chr11.fa	65479489	65484453	KAT5	17.07996745	1717
chr11.fa	65485144	65488409	RNASEH2C	14.99241253	1976
chr11.fa	65543378	65547822	DKFZp761E198	10.55765729	3116
chr11.fa	65554529	65564685	OVOL1	24.09295317	4788
chr11.fa	65601410	65621172	SNX32	0	0
chr11.fa	65622285	65625804	CFL1	322.3479389	26904
chr11.fa	65627872	65633911	MUS81	7.162688452	1140
chr11.fa	65633915	65640340	EFEMP2	4.006129499	532
chr11.fa	65647284	65651210	CTSW	1.769173053	152
chr11.fa	65651213	65656010	FIBP	25.29524384	2432
chr11.fa	65657875	65659106	CCDC85B	4.645302942	380
chr11.fa	65659692	65667997	FOSL1	0	0
chr11.fa	65684283	65686531	C11orf68	13.2239925	1368
chr11.fa	65686728	65689048	DRAP1	26.1949663	1748
chr11.fa	65713115	65727434	TSGA10IP	0	0
chr11.fa	65729160	65747607	SART1	10.489282	2508
chr11.fa	65764017	65769549	EIF1AD	12.60334365	2349
chr11.fa	65769579	65771617	BANF1	51.98133752	3876
chr11.fa	65779462	65780976	CST6	0	0
chr11.fa	65784223	65793988	CATSPER1	0	0
chr11.fa	65808236	65816651	GAL3ST3	0	0
chr11.fa	65819816	65836382	SF3B2	59.29026476	11393
chr11.fa	65837824	66012213	PACS1	37.92675087	11302
chr11.fa	66024765	66035331	KLC2	4.903442264	1064
chr11.fa	66036056	66044963	RAB1B	104.8004983	13604
chr11.fa	66045696	66051683	CNIH2	0	0
chr11.fa	66052051	66056638	YIF1A	8.034096921	598

chr11.fa	66059373	66064135	TMEM151A	0	0
chr11.fa	66081958	66084515	CD248	0	0
chr11.fa	66099542	66104000	RIN1	0	0
chr11.fa	66104804	66112582	BRMS1	25.69751346	2312
chr11.fa	66112843	66115161	B3GNT1	1.658778583	228
chr11.fa	66129992	66139291	SLC29A2	1.36539737	228
chr11.fa	66188475	66194177	NPAS4	0	0
chr11.fa	66202550	66206310	MRPL11	6.788883436	760
chr11.fa	66234336	66244808	PELI3	3.336985387	608
chr11.fa	66247880	66277130	DPP3	11.92742225	2128
chr11.fa	66278119	66301084	BBS1	13.02278239	2914
chr11.fa	66306735	66313671	ZDHHC24	1.738148141	152
chr11.fa	66314391	66330797	ACTN3	0	0
chr11.fa	66330935	66336047	CTSF	0.568388448	76
chr11.fa	66357640	66360554	CCDC87	0.785261624	152
chr11.fa	66360690	66373490	CCS	8.872974414	608
chr11.fa	66391685	66397397	RBM14	7.336337599	2402
chr11.fa	66406088	66435858	RBM4	11.64127014	2390
chr11.fa	66432470	66445275	RBM4B	5.982235707	684
chr11.fa	66452720	66488870	SPTBN2	19.04266969	9947
chr11.fa	66512207	66610882	C11orf80	13.85202108	2040
chr11.fa	66610988	66614003	RCE1	2.513770957	228
chr11.fa	66615997	66725847	PC	8.906409029	2508
chr11.fa	66624876	66627946	LRFN4	0.452722755	76
chr11.fa	66742754	66744479	C11orf86	0	0
chr11.fa	66790190	66818334	SYT12	0	0
chr11.fa	66824289	66839488	RHOD	13.13317686	988
chr11.fa	66886740	67025550	KDM2A	36.4950867	17876
chr11.fa	67033905	67054029	ADRBK1	40.8438457	9340
chr11.fa	67056762	67069955	ANKRD13D	8.545556161	1292
chr11.fa	67070919	67080078	SSH3	37.2277867	7114
chr11.fa	67085310	67159158	LOC100130987	5.386436899	304
chr11.fa	67119019	67121052	POLD4	62.45495646	3952
chr11.fa	67131639	67141648	CLCF1	1.160271491	152
chr11.fa	67159423	67165651	RAD9A	3.63367994	456
chr11.fa	67165884	67169376	PPP1CA	93.38077012	7676
chr11.fa	67171412	67177560	TBC1D10C	0	0
chr11.fa	67183149	67193078	CARNS1	0.288411203	76
chr11.fa	67195935	67202879	RPS6KB2	30.34251519	3566
chr11.fa	67202981	67205153	PTPRCAP	0	0
chr11.fa	67205518	67211263	CORO1B	49.09767731	6357
chr11.fa	67218772	67220200	GPR152	0	0
chr11.fa	67222818	67229245	CABP4	0.854239343	228

chr11.fa	67231819	67236731	TMEM134	43.05836179	2636
chr11.fa	67250505	67258579	AIP	36.20411519	2964
chr11.fa	67259239	67272843	PITPNM1	10.021348	2812
chr11.fa	67273968	67276102	CDK2AP2	37.37839307	3420
chr11.fa	67286418	67290899	CABP2	0	0
chr11.fa	67351066	67354124	GSTP1	101.3684803	6448
chr11.fa	67374323	67380012	NDUFV1	37.89632839	4104
chr11.fa	67380995	67383135	DOC2GP	0	0
chr11.fa	67395409	67397401	NUDT8	11.3188219	608
chr11.fa	67398774	67407031	TBX10	0.739477287	76
chr11.fa	67410026	67418130	ACY3	19.37023855	1672
chr11.fa	67429633	67448685	ALDH3B2	13.62129211	2432
chr11.fa	67559238	67572807	LOC645332	1.231357699	152
chr11.fa	67758575	67771593	UNC93B1	0.988881439	152
chr11.fa	67776048	67796743	ALDH3B1	11.71958545	2280
chr11.fa	67798084	67804114	NDUFS8	50.89516437	2717
chr11.fa	67806462	67818366	TCIRG1	1.881977226	380
chr11.fa	67820326	67888858	CHKA	10.87001491	1961
chr11.fa	67923507	67980784	SUV420H1	20.15926533	7797
chr11.fa	68028803	68039469	C11orf24	3.811696672	532
chr11.fa	68080108	68216743	LRP5	6.473061874	2204
chr11.fa	68228186	68382801	PPP6R3	34.37334413	11932
chr11.fa	68451983	68458643	GAL	0	0
chr11.fa	68474908	68518988	MTL5	1.570071429	532
chr11.fa	68522088	68609399	CPT1A	7.095066191	2584
chr11.fa	68658746	68671303	MRPL21	43.46123384	2046
chr11.fa	68671319	68708069	IGHMBP2	6.079075605	1596
chr11.fa	68747490	68748455	MRGPRD	0	0
chr11.fa	68771862	68780850	MRGPRF	0	0
chr11.fa	68816350	68858072	TPCN2	0.455433669	152
chr11.fa	69061622	69064754	MYEOV	0	0
chr11.fa	69455873	69469242	CCND1	74.72380335	21280
chr11.fa	69480332	69490165	ORAOV1	4.076613281	684
chr11.fa	69513006	69519106	FGF19	0	0
chr11.fa	69587797	69590171	FGF4	0	0
chr11.fa	69624736	69634192	FGF3	0	0
chr11.fa	69924408	70035652	ANO1	0.558599034	152
chr11.fa	70049269	70053508	FADD	10.5010293	1292
chr11.fa	70116823	70230502	PPFIA1	26.34813298	9120
chr11.fa	70130061	70130176	MIR548K	0	0
chr11.fa	70244612	70282690	CTTN	116.0322697	18475
chr11.fa	70313961	70935808	SHANK2	17.61160794	12220
chr11.fa	70718375	70718473	MIR3664	0	0

chr11.fa	71145457	71159477	DHCR7	3.865463147	684
chr11.fa	71164217	71212581	NADSYN1	15.07298694	2432
chr11.fa	71238313	71239210	KRTAP5-7	0	0
chr11.fa	71249071	71250253	KRTAP5-8	0.967495335	76
chr11.fa	71259466	71260653	KRTAP5-9	0.963428963	76
chr11.fa	71276609	71277666	KRTAP5-10	0	0
chr11.fa	71292901	71293921	KRTAP5-11	0	0
chr11.fa	71498557	71512280	FAM86C	3.133064359	456
chr11.fa	71544246	71548608	DEFB108B	0	0
chr11.fa	71576555	71639493	LOC100133315	0	0
chr11.fa	71639768	71708643	RNF121	3.649945429	665
chr11.fa	71709958	71713574	IL18BP	0	0
chr11.fa	71713911	71780957	NUMA1	139.5121043	65881
chr11.fa	71783274	71783348	MIR3165	0	0
chr11.fa	71791382	71821828	LRTOMT	7.694479552	2508
chr11.fa	71808338	71814433	C11orf59	88.34554728	6916
chr11.fa	71822203	71823822	C11orf51	23.42772482	532
chr11.fa	71846771	71850934	FOLR3	0	0
chr11.fa	71900602	71907367	FOLR1	8.241180683	684
chr11.fa	71927819	71932994	FOLR2	0	0
chr11.fa	71935882	71950120	INPPL1	23.89219487	7372
chr11.fa	71950189	71955220	PHOX2A	0	0
chr11.fa	72003470	72145568	CLPB	3.674494267	760
chr11.fa	72287185	72385494	PDE2A	0	0
chr11.fa	72326107	72326174	MIR139	0	0
chr11.fa	72396114	72463434	ARAP1	17.80814926	6676
chr11.fa	72465774	72504750	STARD10	51.15917734	6753
chr11.fa	72525451	72540680	ATG16L2	0	0
chr11.fa	72547790	72853143	FCHSD2	5.863256673	1748
chr11.fa	72929344	72947395	P2RY2	2.002462324	380
chr11.fa	72975570	73008850	P2RY6	0	0
chr11.fa	73019663	73080425	ARHGEF17	3.222976364	1672
chr11.fa	73087405	73108519	RELT	0	0
chr11.fa	73117028	73309228	FAM168A	7.773246685	912
chr11.fa	73357223	73373864	PLEKHB1	7.519926767	1444
chr11.fa	73386683	73472201	RAB6A	52.53647261	12258
chr11.fa	73498917	73575656	MRPL48	22.19139711	1444
chr11.fa	73583713	73587890	CHCHD8	40.6784799	2204
chr11.fa	73588033	73638779	PAAF1	30.9384646	3256
chr11.fa	73661364	73681332	DNAJB13	0	0
chr11.fa	73685716	73693889	UCP2	126.5605588	13832
chr11.fa	73711326	73720282	UCP3	0	0
chr11.fa	73745480	73882064	C2CD3	9.29241316	3876

chr11.fa	73882368	73965748	PPME1	32.60176137	5310
chr11.fa	73977702	74022699	P4HA3	0	0
chr11.fa	74041361	74109502	PGM2L1	2.288915644	1292
chr11.fa	74165886	74178600	KCNE3	4.101162119	836
chr11.fa	74202923	74204755	LIPT2	3.576901338	228
chr11.fa	74303629	74353765	POLD3	7.675201936	1748
chr11.fa	74407474	74442186	CHRDL2	0	0
chr11.fa	74459913	74551954	RNF169	9.056864794	3800
chr11.fa	74553459	74660232	XRRA1	15.71095553	3648
chr11.fa	74660292	74690076	SPCS2	2.5360607	456
chr11.fa	74699950	74718743	NEU3	6.664331967	1216
chr11.fa	74799796	74800758	OR2AT4	0	0
chr11.fa	74862032	74917445	SLCO2B1	0.771405838	228
chr11.fa	74951950	74954748	LOC441617	0.4088963	76
chr11.fa	74976482	75062873	ARRB1	32.79995936	4800
chr11.fa	75046136	75046230	MIR326	0	0
chr11.fa	75110562	75116733	RPS3	1184.07769	66120
chr11.fa	75111435	75111582	SNORD15A	0	0
chr11.fa	75115465	75115610	SNORD15B	0	0
chr11.fa	75133438	75141674	KLHL35	0	0
chr11.fa	75145685	75236599	GDPD5	0	0
chr11.fa	75273170	75283846	SERPINH1	4.154627382	608
chr11.fa	75297963	75379479	MAP6	2.6589555	988
chr11.fa	75428934	75442331	MOGAT2	0	0
chr11.fa	75479778	75512579	DGAT2	1.400488654	228
chr11.fa	75526212	75855282	UVRAG	9.084124548	3116
chr11.fa	75897370	75917574	WNT11	0	0
chr11.fa	76061004	76091880	PRKRIR	7.534987404	1596
chr11.fa	76156069	76262589	C11orf30	7.892376325	2888
chr11.fa	76368568	76381791	LRRC32	0	0
chr11.fa	76391210	76432833	GUCY2E	0	0
chr11.fa	76494285	76509198	TSKU	0.422300267	76
chr11.fa	76571917	76734850	ACER3	1.826403475	532
chr11.fa	76745435	76753005	B3GNT6	0	0
chr11.fa	76777992	76837198	CAPN5	78.74077651	22952
chr11.fa	76813886	76814377	OMP	0	0
chr11.fa	76839310	76926286	MYO7A	0.306634574	152
chr11.fa	76927603	76998463	GDPD4	0	0
chr11.fa	77033060	77185108	PAK1	21.58445343	4986
chr11.fa	77300680	77321401	AQP11	1.063280987	152
chr11.fa	77327196	77348851	CLNS1A	47.51826829	4332
chr11.fa	77377274	77531880	RSF1	28.93027923	9862
chr11.fa	77532208	77583398	C11orf67	11.35526864	380

chr11.fa	77589766	77705717	INTS4	9.450549851	1976
chr11.fa	77726761	77734320	KCTD14	5.47981285	608
chr11.fa	77774907	77779395	THRSP	0.976682323	76
chr11.fa	77779398	77791265	NDUFC2	19.92642788	3268
chr11.fa	77811988	77850699	ALG8	0	0
chr11.fa	77882298	77899664	KCTD21	6.776834927	1520
chr11.fa	77899858	77925757	USP35	1.062076136	304
chr11.fa	77926336	78128868	GAB2	8.810322163	3648
chr11.fa	78147016	78285909	NARS2	10.28445733	1714
chr11.fa	78364328	79151695	ODZ4	0.422450874	380
chr11.fa	79113066	79113153	MIR708	0	0
chr11.fa	81601783	81601878	MIR4300	0	0
chr11.fa	82443046	82444906	FAM181B	0.615076424	76
chr11.fa	82535409	82611557	PRCP	8.301724444	1216
chr11.fa	82612737	82645699	C11orf82	0.642486784	152
chr11.fa	82692478	82782884	RAB30	10.71730005	1140
chr11.fa	82752506	82752640	SNORA70E	0	0
chr11.fa	82868137	82896835	PCF11	25.74977387	10108
chr11.fa	82905291	82960013	ANKRD42	6.254682634	1064
chr11.fa	82972502	82997377	CCDC90B	21.62692443	2266
chr11.fa	83166056	85338314	DLG2	1.036473053	608
chr11.fa	85339622	85347583	TMEM126B	28.27258121	2508
chr11.fa	85358963	85367597	TMEM126A	16.79125503	912
chr11.fa	85368608	85376182	CREBZF	5.137484566	2584
chr11.fa	85394893	85397320	CCDC89	0	0
chr11.fa	85405265	85522178	SYTL2	89.02734233	41367
chr11.fa	85566144	85631051	CCDC83	0	0
chr11.fa	85668485	85780108	PICALM	55.53338881	14281
chr11.fa	85955815	85989780	EED	9.748599862	1292
chr11.fa	86013253	86056985	C11orf73	10.19695503	912
chr11.fa	86085778	86134151	CCDC81	0.411607215	76
chr11.fa	86152150	86383678	ME3	4.40418214	684
chr11.fa	86511491	86522273	PRSS23	1.203646126	304
chr11.fa	86656721	86666433	FZD4	3.720730423	1824
chr11.fa	86748886	87039876	TMEM135	3.391956713	2045
chr11.fa	87846415	87908635	RAB38	0.783906167	76
chr11.fa	88026760	88070941	CTSC	0.453023967	228
chr11.fa	88237744	88796816	GRM5	0	0
chr11.fa	88911040	89028927	TYR	0	0
chr11.fa	89057522	89322779	NOX4	13.97853043	4547
chr11.fa	89392465	89431886	FOLH1B	1.159669065	152
chr11.fa	89443467	89451040	TRIM77	0	0
chr11.fa	89530823	89541743	TRIM49	0	0

chr11.fa	89575165	89735676	TRIM53	0	0
chr11.fa	89603606	89609185	TRIM64B	0	0
chr11.fa	89644579	89666229	TRIM49L1	0	0
chr11.fa	89701672	89707240	TRIM64	0	0
chr11.fa	89764274	89775193	TRIM49L2	0	0
chr11.fa	89819118	89820299	UBTFL1	0	0
chr11.fa	89867818	89925779	NAALAD2	0	0
chr11.fa	89933597	89956532	CHORDC1	2.349158192	528
chr11.fa	92085262	92629635	FAT3	0.661011367	836
chr11.fa	92702789	92715948	MTNR1B	0	0
chr11.fa	92880850	92931095	SLC36A4	2.321747833	380
chr11.fa	93063883	93171636	CCDC67	1.287985694	228
chr11.fa	93211639	93276546	C11orf75	76.30727875	4940
chr11.fa	93394816	93463522	KIAA1731	8.537574023	4560
chr11.fa	93454680	93455032	SCARNA9	0	0
chr11.fa	93463681	93463812	SNORA25	0	0
chr11.fa	93464145	93464265	SNORA32	0	0
chr11.fa	93464669	93464739	SNORD6	0	0
chr11.fa	93465170	93465299	SNORA1	0	0
chr11.fa	93465527	93465665	SNORA8	0	0
chr11.fa	93466394	93466466	SNORD5	0	0
chr11.fa	93466632	93466763	SNORA18	0	0
chr11.fa	93466840	93466930	MIR1304	0	0
chr11.fa	93468276	93468402	SNORA40	0	0
chr11.fa	93469096	93474703	TAF1D	6.111606581	532
chr11.fa	93474793	93496247	C11orf54	44.06019538	6992
chr11.fa	93517405	93546496	MED17	13.09251314	3040
chr11.fa	93553735	93583668	C11orf90	0	0
chr11.fa	93754378	93847374	HEPHL1	0.384648674	152
chr11.fa	93862094	93915137	PANX1	3.721634062	684
chr11.fa	94038803	94040858	FOLR4	0	0
chr11.fa	94110477	94134585	GPR83	0	0
chr11.fa	94150469	94227040	MRE11A	6.328630363	2204
chr11.fa	94227153	94232744	ANKRD49	4.806752973	608
chr11.fa	94245695	94265289	LOC643037	0	0
chr11.fa	94277017	94283064	FUT4	1.324733649	532
chr11.fa	94300474	94354587	PIWIL4	0	0
chr11.fa	94501508	94609918	AMOTL1	33.83326968	20216
chr11.fa	94695787	94706776	CWC15	32.70311946	3420
chr11.fa	94706845	94732676	KDM4D	0.385100494	76
chr11.fa	94758422	94760760	KDM4DL	0	0
chr11.fa	94800056	94804388	SRSF8	19.81211765	5700
chr11.fa	94822974	94865815	ENDOD1	4.634007464	1444

chr11.fa	94906133	94964246	SESN3	14.97720128	1976
chr11.fa	95502106	95522954	FAM76B	1.448532087	380
chr11.fa	95523642	95565854	CEP57	16.67257721	3496
chr11.fa	95566044	95657371	MTMR2	24.95442162	8056
chr11.fa	95711440	96076344	MAML2	23.15377183	8208
chr11.fa	96085929	96123083	CCDC82	6.289020887	1140
chr11.fa	96123158	96126727	JRKL	4.488672315	1064
chr11.fa	98891871	100227473	CNTN5	0	0
chr11.fa	100558407	100861656	ARHGAP42	6.981960806	2203
chr11.fa	100862811	100864666	TMEM133	3.083514863	380
chr11.fa	100900355	101000544	PGR	0.087803515	76
chr11.fa	101322295	101454659	TRPC6	0.248199301	76
chr11.fa	101390551	101390636	MIR3920	0	0
chr11.fa	101761405	101787253	ANGPTL5	0	0
chr11.fa	101785746	101871793	KIAA1377	0.487512827	228
chr11.fa	101918169	101955291	C11orf70	0	0
chr11.fa	101981192	102104154	YAP1	32.91803476	12441
chr11.fa	102188194	102208465	BIRC3	2.10472405	373
chr11.fa	102217966	102249401	BIRC2	18.3479225	4560
chr11.fa	102267056	102323775	TMEM123	8.927343314	2128
chr11.fa	102391239	102401478	MMP7	1.022918479	76
chr11.fa	102447566	102496063	MMP20	0	0
chr11.fa	102562415	102576468	MMP27	0	0
chr11.fa	102582526	102595685	MMP8	0	0
chr11.fa	102641233	102651359	MMP10	0	0
chr11.fa	102660641	102668966	MMP1	0	0
chr11.fa	102706528	102714342	MMP3	0	0
chr11.fa	102733464	102745764	MMP12	0	0
chr11.fa	102813721	102826463	MMP13	0	0
chr11.fa	102921413	102962944	DCUN1D5	1.168705448	988
chr11.fa	102980160	103350591	DYNC2H1	2.06903034	1882
chr11.fa	103777914	104035027	PDGFD	0	0
chr11.fa	103907308	103909922	DDI1	0	0
chr11.fa	104756445	104769397	CASP12	0	0
chr11.fa	104772276	104788902	LOC643733	0	0
chr11.fa	104813594	104839325	CASP4	21.79439872	2052
chr11.fa	104864967	104893895	CASP5	0	0
chr11.fa	104896237	104905201	CASP1	4.299811924	380
chr11.fa	104912053	104916051	CARD16	6.040219161	304
chr11.fa	104963196	104971506	CARD17	0	0
chr11.fa	105008448	105010461	CARD18	0	0
chr11.fa	105480800	105852819	GRIA4	0.153016074	76
chr11.fa	105878629	105892954	KIAA1826	3.64075844	988

chr11.fa	105921825	105948291	KBTBD3	0.282085735	76
chr11.fa	105948466	105969419	AASDHPPT	12.35348767	2204
chr11.fa	106557910	106889171	GUCY1A2	1.549890176	304
chr11.fa	107197072	107328572	CWF19L2	5.585086704	1216
chr11.fa	107373453	107436461	ALKBH8	3.362437864	908
chr11.fa	107461817	107537505	ELMOD1	0	0
chr11.fa	107462471	107463949	LOC643923	0	0
chr11.fa	107578101	107582787	SLN	0	0
chr11.fa	107661717	107729914	SLC35F2	2.166472663	456
chr11.fa	107799277	107834208	RAB39	0.642336177	76
chr11.fa	107879408	107978488	CUL5	13.08227191	5548
chr11.fa	107992258	108018891	ACAT1	49.88218591	7068
chr11.fa	108028119	108093365	NPAT	8.098707055	3192
chr11.fa	108093559	108239826	ATM	2.611815705	2280
chr11.fa	108253727	108338258	C11orf65	0	0
chr11.fa	108342833	108369159	KDELC2	4.248153939	1216
chr11.fa	108376158	108464374	EXPH5	16.60555738	11232
chr11.fa	108535816	108811648	DDX10	10.04363774	2128
chr11.fa	109292846	109299893	C11orf87	0	0
chr11.fa	109964087	110042566	ZC3H12C	5.978470548	3496
chr11.fa	110100166	110167437	RDX	17.58977002	5244
chr11.fa	110300594	110335605	FDX1	8.178377826	1748
chr11.fa	110447766	110583451	ARHGAP20	0	0
chr11.fa	111126707	111156972	C11orf53	0	0
chr11.fa	111164114	111170539	C11orf92	3.557473116	1268
chr11.fa	111169976	111179353	C11orf93	5.352851678	456
chr11.fa	111222981	111250157	POU2AF1	0	0
chr11.fa	111338256	111383064	BTG4	0	0
chr11.fa	111383663	111383746	MIR34B	0	0
chr11.fa	111384164	111384240	MIR34C	0	0
chr11.fa	111385510	111407756	C11orf88	0	0
chr11.fa	111411233	111431788	LAYN	0	0
chr11.fa	111473170	111597632	SIK2	17.12951695	6458
chr11.fa	111597632	111637169	PPP2R1B	13.63319002	5320
chr11.fa	111652919	111742305	ALG9	3.090743969	1368
chr11.fa	111744780	111749947	FDXACB1	2.8291407	532
chr11.fa	111750154	111754797	C11orf1	29.46222094	1520
chr11.fa	111779350	111782473	CRYAB	72.8838453	3344
chr11.fa	111784165	111784817	HSPB2	0	0
chr11.fa	111789402	111789600	HSPB2-C11ORF52	0	0
chr11.fa	111807927	111893307	DIXDC1	5.550447238	2352
chr11.fa	111895538	111935002	DLAT	8.860022266	2432
chr11.fa	111938523	111944895	PIH1D2	0.920807359	76

chr11.fa	111944968	111955538	C11orf57	15.46320805	3496
chr11.fa	111955875	111957522	TIMM8B	45.55662029	2432
chr11.fa	111957571	111966518	SDHD	28.51038867	2584
chr11.fa	112013976	112034840	IL18	5.62966619	428
chr11.fa	112038095	112043279	TEX12	0	0
chr11.fa	112046208	112089649	BCO2	0.381335334	76
chr11.fa	112097088	112104695	PTS	15.59272953	967
chr11.fa	112118876	112131583	C11orf34	0	0
chr11.fa	112831995	113149158	NCAM1	0	0
chr11.fa	113140254	113144623	LOC100288346	0	0
chr11.fa	113185251	113237114	TTC12	19.23725312	2957
chr11.fa	113258513	113271140	ANKK1	0	0
chr11.fa	113280317	113346001	DRD2	0	0
chr11.fa	113320745	113320810	MIR4301	0	0
chr11.fa	113558268	113577068	TMPRSS5	0	0
chr11.fa	113603909	113644425	ZW10	9.922098402	1900
chr11.fa	113650518	113651207	CLDN25	0	0
chr11.fa	113668597	113746256	USP28	6.672012892	2052
chr11.fa	113775589	113817283	HTR3B	0	0
chr11.fa	113845797	113861034	HTR3A	0	0
chr11.fa	113930431	114121397	ZBTB16	11.80015986	1900
chr11.fa	114166535	114183238	NNMT	0	0
chr11.fa	114262170	114271139	C11orf71	7.076391001	304
chr11.fa	114271384	114279635	RBM7	7.762101813	988
chr11.fa	114310108	114321000	REXO2	74.06971988	5705
chr11.fa	114392437	114430580	FAM55A	0	0
chr11.fa	114441313	114466484	FAM55D	0	0
chr11.fa	114549200	114577652	FAM55B	0	0
chr11.fa	115044345	115375241	CADM1	1.594469662	456
chr11.fa	115626051	115630918	LOC283143	0.510254389	76
chr11.fa	116618886	116643714	BUD13	8.820714003	1292
chr11.fa	116649276	116658739	ZNF259	20.56650497	2428
chr11.fa	116660086	116663136	APOA5	0	0
chr11.fa	116691418	116694011	APOA4	0	0
chr11.fa	116700624	116703787	APOC3	0	0
chr11.fa	116706469	116708338	APOA1	0	0
chr11.fa	116714118	116968993	SIK3	11.88178852	4788
chr11.fa	117015000	117047592	PAFAH1B2	31.67839371	13224
chr11.fa	117049939	117068161	SIDT2	7.593723889	1976
chr11.fa	117070040	117075508	TAGLN	0	0
chr11.fa	117075788	117102811	PCSK7	5.879070342	1360
chr11.fa	117103404	117156404	RNF214	13.81572494	2508
chr11.fa	117156415	117186972	BACE1	4.708708225	1824

chr11.fa	117198571	117283982	CEP164	6.711471761	2508
chr11.fa	117298489	117667976	DSCAML1	0.49775406	228
chr11.fa	117690790	117698807	FXYD2	125.932982	5594
chr11.fa	117707693	117748145	FXYD6	1.455610587	228
chr11.fa	117771360	117800115	TMPRSS13	0	0
chr11.fa	117857106	117872199	IL10RA	0	0
chr11.fa	117947727	117990556	TMPRSS4	0	0
chr11.fa	118004092	118023630	SCN4B	0	0
chr11.fa	118033519	118047337	SCN2B	0	0
chr11.fa	118064442	118095809	AMICA1	0	0
chr11.fa	118100336	118123011	MPZL3	4.657652664	304
chr11.fa	118124131	118135251	MPZL2	4.670454206	988
chr11.fa	118175295	118186890	CD3E	0	0
chr11.fa	118209789	118213459	CD3D	0	0
chr11.fa	118215059	118224497	CD3G	0	0
chr11.fa	118230302	118269926	UBE4A	6.730749377	2736
chr11.fa	118272104	118280562	ATP5L	92.16115972	8512
chr11.fa	118307205	118397539	MLL	17.78706437	19598
chr11.fa	118398210	118401740	TTC36	3.371474246	152
chr11.fa	118401803	118417313	TMEM25	1.4110311	282
chr11.fa	118415243	118436791	IFT46	21.9523848	2733
chr11.fa	118443102	118473747	ARCN1	84.09950183	22420
chr11.fa	118477213	118528741	PHLDB1	1.183314266	456
chr11.fa	118528942	118550381	TREH	2.470848141	304
chr11.fa	118618473	118661972	DDX6	75.32246368	31138
chr11.fa	118754541	118766850	CXCR5	0	0
chr11.fa	118766972	118781613	BCL9L	15.92858174	8056
chr11.fa	118827008	118829269	UPK2	107.8431988	6688
chr11.fa	118842417	118851995	FOXR1	0	0
chr11.fa	118868852	118886421	CCDC84	1.959991327	152
chr11.fa	118873671	118874303	RPL23AP64	0	0
chr11.fa	118886456	118889057	RPS25	732.0522424	22262
chr11.fa	118889241	118894385	TRAPPC4	56.48386562	4403
chr11.fa	118889681	118889722	MIR3656	0	0
chr11.fa	118895061	118901616	SLC37A4	9.080961814	1672
chr11.fa	118914896	118927925	HYOU1	9.681128207	2964
chr11.fa	118938493	118952688	VPS11	22.02542889	4712
chr11.fa	118955587	118964259	HMBS	8.522814599	906
chr11.fa	118964585	118966177	H2AFX	6.466736406	684
chr11.fa	118967213	118972785	DPAGT1	3.740610465	532
chr11.fa	118978093	118987827	C2CD2L	1.711490813	380
chr11.fa	118992288	119005765	HINFP	4.976486354	760
chr11.fa	119019750	119033374	ABCG4	0.581792416	152

chr11.fa	119039440	119054725	NLRX1	6.725779367	1672
chr11.fa	119056166	119060932	PDZD3	3.503405428	532
chr11.fa	119060963	119066584	CCDC153	0	0
chr11.fa	119076990	119178859	CBL	7.034673036	5244
chr11.fa	119179234	119187840	MCAM	0	0
chr11.fa	119205237	119208022	RNF26	3.697537042	684
chr11.fa	119225925	119252436	USP2	3.095864585	836
chr11.fa	119252488	119369944	LOC100499227	0	0
chr11.fa	119288655	119294246	THY1	4.775125635	760
chr11.fa	119508808	119599435	PVRL1	3.744827443	1520
chr11.fa	119981994	120008863	TRIM29	2.274758645	456
chr11.fa	120081747	120100650	OAF	5.53252508	684
chr11.fa	120110951	120190653	POU2F3	0	0
chr11.fa	120195838	120204388	TMEM136	1.115390792	304
chr11.fa	120207618	120360645	ARHGEF12	51.97907843	33923
chr11.fa	120531028	120856969	GRIK4	0	0
chr11.fa	120894803	120960354	TBCEL	10.76880743	2888
chr11.fa	120973375	121061515	TECTA	0.176962487	76
chr11.fa	121163388	121184119	SC5DL	5.917324361	2812
chr11.fa	121322912	121504471	SORL1	16.06397686	11704
chr11.fa	121959811	122073770	LOC399959	0.371395314	76
chr11.fa	121970465	121970552	MIR125B1	0	0
chr11.fa	121986062	121986923	BLID	0	0
chr11.fa	122017230	122017301	MIRLET7A2	0	0
chr11.fa	122022937	122023016	MIR100	0	0
chr11.fa	122526398	122685187	UBASH3B	11.7727495	5396
chr11.fa	122709255	122743347	CRTAM	0	0
chr11.fa	122753473	122830430	C11orf63	6.332546129	1520
chr11.fa	122848357	122852379	BSX	0	0
chr11.fa	122888274	122890319	LOC341056	0	0
chr11.fa	122928200	122932844	HSPA8	805.4595955	116054
chr11.fa	122943033	123066007	ASAM	0	0
chr11.fa	123396528	123493518	GRAMD1B	1.658326764	304
chr11.fa	123499895	123525315	SCN3B	0.201963145	76
chr11.fa	123594997	123612363	ZNF202	4.516383887	1216
chr11.fa	123624288	123625226	OR6X1	0	0
chr11.fa	123676116	123677057	OR6M1	0	0
chr11.fa	123753633	123756340	TMEM225	0	0
chr11.fa	123777139	123778083	OR8D4	0	0
chr11.fa	123810324	123811280	OR4D5	0	0
chr11.fa	123813574	123814545	OR6T1	0	0
chr11.fa	123847403	123848398	OR10S1	0	0
chr11.fa	123886282	123887217	OR10G4	0	0

chr11.fa	123893720	123894655	OR10G9	0	0
chr11.fa	123900330	123901265	OR10G8	0	0
chr11.fa	123908773	123909708	OR10G7	0	0
chr11.fa	123986111	124017618	VWA5A	16.70043939	3921
chr11.fa	124095398	124096312	OR8G2	0	0
chr11.fa	124120423	124121283	OR8G1	0	0
chr11.fa	124134723	124135763	OR8G5	0	0
chr11.fa	124179736	124180662	OR8D1	0	0
chr11.fa	124189158	124190093	OR8D2	0	0
chr11.fa	124252298	124253239	OR8B2	0	0
chr11.fa	124266306	124267247	OR8B3	0	0
chr11.fa	124293838	124294767	OR8B4	0	0
chr11.fa	124310046	124310981	OR8B8	0	0
chr11.fa	124412618	124413550	OR8B12	0	0
chr11.fa	124439965	124440945	OR8A1	0	0
chr11.fa	124481453	124490251	PANX3	0	0
chr11.fa	124492742	124505692	TBRG1	10.4633777	3496
chr11.fa	124505823	124543717	SIAE	3.80266029	684
chr11.fa	124543740	124564687	SPA17	3.664704853	228
chr11.fa	124609829	124617102	NRGN	0	0
chr11.fa	124617370	124622109	VSIG2	18.37909802	1368
chr11.fa	124623019	124632223	ESAM	1.224881625	152
chr11.fa	124636394	124670299	C11orf61	1.543263495	228
chr11.fa	124735305	124751370	ROBO3	0.50272407	152
chr11.fa	124754114	124767831	ROBO4	0	0
chr11.fa	124792284	124806308	HEPACAM	0	0
chr11.fa	124824017	124911385	CCDC15	0.587967277	152
chr11.fa	124933013	124960412	SLC37A2	0	0
chr11.fa	124966399	124973205	TMEM218	4.898472253	684
chr11.fa	125034559	125303285	PKNOX2	2.761819652	684
chr11.fa	125315641	125366206	FEZ1	0	0
chr11.fa	125439298	125454575	EI24	48.0527703	7214
chr11.fa	125462739	125491280	STT3A	8.983670097	1672
chr11.fa	125495036	125546150	CHEK1	2.47672179	532
chr11.fa	125542229	125550793	ACRV1	0	0
chr11.fa	125616188	125619743	PATE1	0	0
chr11.fa	125646028	125648714	PATE2	0	0
chr11.fa	125658006	125661495	PATE3	0	0
chr11.fa	125703211	125709967	PATE4	0	0
chr11.fa	125753509	125770541	HYLS1	5.909342223	988
chr11.fa	125763380	125773116	PUS3	9.310787138	1140
chr11.fa	125774272	125793005	DDX25	0	0
chr11.fa	125826713	125933187	CDON	0.568990874	304

chr11.fa	126071989	126081587	RPUSD4	12.86238661	2083
chr11.fa	126081619	126132813	FAM118B	4.524215419	608
chr11.fa	126132880	126138877	SRPR	19.66934281	3952
chr11.fa	126138935	126148027	FOXRED1	9.38624093	1368
chr11.fa	126152982	126164828	TIRAP	2.924926353	456
chr11.fa	126173647	126215644	DCPS	5.417311205	532
chr11.fa	126211612	126225482	FLJ39051	1.522027997	76
chr11.fa	126226096	126284533	ST3GAL4	18.38723076	2128
chr11.fa	126293396	126870766	KIRREL3	0	0
chr11.fa	126858354	126858438	MIR3167	0	0
chr11.fa	128328656	128457453	ETS1	3.718320721	1368
chr11.fa	128562389	128683162	FLI1	0	0
chr11.fa	128707915	128737268	KCNJ1	5.65044987	1064
chr11.fa	128761313	128787951	KCNJ5	0.789629209	152
chr11.fa	128769460	128775592	C11orf45	0	0
chr11.fa	128804627	128813294	TP53AIP1	0	0
chr11.fa	128834955	129062093	ARHGAP32	33.96791178	23560
chr11.fa	129245881	129322174	BARX2	0.647758007	76
chr11.fa	129685741	129729898	TMEM45B	6.223507115	912
chr11.fa	129733670	129765490	NFRKB	8.942102739	2999
chr11.fa	129769601	129830977	PRDM10	4.252370917	1824
chr11.fa	129872519	129875381	NCRNA00167	0	0
chr11.fa	129939716	130014706	APLP2	22.51986961	5700
chr11.fa	130029682	130080257	ST14	6.937080107	1520
chr11.fa	130096574	130184607	ZBTB44	12.74506424	7904
chr11.fa	130274818	130298539	ADAMTS8	0	0
chr11.fa	130318869	130346539	ADAMTS15	0.604985797	228
chr11.fa	130745766	130786382	SNX19	10.16924346	4104
chr11.fa	131240371	132206716	NTM	0	0
chr11.fa	132284875	133402403	OPCML	0	0
chr11.fa	133710517	133715392	SPATA19	0	0
chr11.fa	133766330	133771635	LOC283174	0	0
chr11.fa	133785185	133826880	IGSF9B	0.313261254	152
chr11.fa	133902167	133911236	LOC100128239	0	0
chr11.fa	133938820	134021649	JAM3	1.873844482	456
chr11.fa	134022337	134094426	NCAPD3	43.4675593	16177
chr11.fa	134094561	134117686	VPS26B	35.87624512	8740
chr11.fa	134118173	134123260	THYN1	29.43601543	1976
chr11.fa	134123434	134135746	ACAD8	29.64234616	4332
chr11.fa	134146275	134189458	GLB1L3	1.166446352	228
chr11.fa	134201768	134246218	GLB1L2	0.36281075	76
chr11.fa	134248398	134281812	B3GAT1	0.626221295	152
chr11.fa	134306376	134375555	LOC283177	1.656218274	301

chr12.fa	87984	91263	LOC100288778	0.93737406	76
chr12.fa	147946	149412	FAM138D	0	0
chr12.fa	176049	287625	IQSEC3	0.162956095	76
chr12.fa	246577	258332	LOC574538	0	0
chr12.fa	299250	323371	SLC6A12	1.200483392	304
chr12.fa	329789	372039	SLC6A13	2.596303249	532
chr12.fa	389223	498515	KDM5A	21.86804523	15580
chr12.fa	498621	551806	CCDC77	1.073522221	171
chr12.fa	569543	671058	B4GALNT3	0.99882146	228
chr12.fa	673462	772755	NINJ2	0	0
chr12.fa	862089	1020618	WNK1	35.53994109	28190
chr12.fa	1021255	1058863	RAD52	3.42569254	608
chr12.fa	1100404	1605099	ERC1	21.08037391	13118
chr12.fa	1609657	1613590	LOC100292680	0	0
chr12.fa	1675159	1703331	FBXL14	5.964313549	836
chr12.fa	1726222	1756378	WNT5B	0	0
chr12.fa	1769481	1769546	MIR3649	0	0
chr12.fa	1800247	1897845	ADIPOR2	11.83585357	3116
chr12.fa	1901123	2027870	CACNA2D4	0.214162261	76
chr12.fa	1929433	1945918	LRTM2	0	0
chr12.fa	2038368	2045742	LOC100271702	0	0
chr12.fa	2055214	2113677	DCP1B	4.377072993	608
chr12.fa	2162416	2807115	CACNA1C	0.40392629	380
chr12.fa	2870366	2880835	LOC283440	0	0
chr12.fa	2904108	2914587	FKBP4	79.85646851	19836
chr12.fa	2921787	2934237	ITFG2	14.04042965	2202
chr12.fa	2934514	2944221	NRIP2	0.412209641	76
chr12.fa	2966849	2986303	FOXM1	0.631341912	152
chr12.fa	2986365	2998626	C12orf32	15.77933082	1976
chr12.fa	3000033	3050306	TULP3	6.864186623	912
chr12.fa	3068478	3149842	TEAD4	1.059365222	152
chr12.fa	3186521	3395730	TSPAN9	4.209146888	1216
chr12.fa	3600425	3703138	PRMT8	0	0
chr12.fa	3724494	3862366	EFCAB4B	24.17970244	6080
chr12.fa	3918027	3982608	PARP11	10.95691479	3268
chr12.fa	4382902	4414522	CCND2	5.967024464	2584
chr12.fa	4430359	4469190	C12orf5	2.504885181	1368
chr12.fa	4477393	4488894	FGF23	0	0
chr12.fa	4543308	4554780	FGF6	0	0
chr12.fa	4596901	4647637	C12orf4	7.7925243	1976
chr12.fa	4647950	4669213	RAD51AP1	1.008912087	152
chr12.fa	4699244	4723041	DYRK4	4.34273474	532
chr12.fa	4724676	4754343	AKAP3	1.13707811	228

chr12.fa	4758264	4796720	NDUFA9	29.10106686	3109
chr12.fa	4829752	4881892	GALNT8	0	0
chr12.fa	4918342	4960278	KCNA6	0	0
chr12.fa	5019073	5027422	KCNA1	0	0
chr12.fa	5153085	5155949	KCNA5	0	0
chr12.fa	5541280	5604465	NTF3	0	0
chr12.fa	5671817	6055398	ANO2	0	0
chr12.fa	6058040	6233836	VWF	2.980349498	1748
chr12.fa	6309555	6347427	CD9	31.61393418	2584
chr12.fa	6419602	6437672	PLEKHG6	18.54386139	4408
chr12.fa	6437923	6451283	TNFRSF1A	8.22717429	1216
chr12.fa	6456009	6486523	SCNN1A	16.32768862	3724
chr12.fa	6493357	6500732	LTBR	11.41174603	1595
chr12.fa	6548167	6560433	LOC678655	1.814957391	228
chr12.fa	6554051	6560884	CD27	0	0
chr12.fa	6561177	6571403	TAPBPL	1.981377432	228
chr12.fa	6571489	6579843	VAMP1	1.295817226	228
chr12.fa	6601316	6602471	MRPL51	155.5450563	6992
chr12.fa	6603298	6641132	NCAPD2	9.288346788	2964
chr12.fa	6619388	6619717	SCARNA10	0	0
chr12.fa	6643657	6647536	GAPDH	741.1213063	64464
chr12.fa	6648694	6665249	IFFO1	0	0
chr12.fa	6666037	6677480	NOP2	6.710718729	1216
chr12.fa	6679248	6716551	CHD4	56.199822	24244
chr12.fa	6690639	6690775	SCARNA11	0	0
chr12.fa	6728001	6745297	LPAR5	1.959991327	380
chr12.fa	6747242	6756580	ACRBP	0	0
chr12.fa	6759704	6772308	ING4	22.9237959	2204
chr12.fa	6775643	6798669	ZNF384	10.32677772	2356
chr12.fa	6802957	6809812	C12orf53	0	0
chr12.fa	6833150	6841041	COPS7A	38.73294678	5928
chr12.fa	6857158	6862636	MLF2	79.3835645	10136
chr12.fa	6875541	6880118	PTMS	228.3978767	17440
chr12.fa	6881670	6887621	LAG3	0	0
chr12.fa	6898638	6929976	CD4	0.331334019	76
chr12.fa	6930963	6936583	GPR162	0	0
chr12.fa	6937572	6949018	LEPREL2	0.434047564	76
chr12.fa	6949375	6956557	GNB3	0	0
chr12.fa	6957972	6960456	CDCA3	0	0
chr12.fa	6961285	6975795	USP5	29.06371648	6156
chr12.fa	6976584	6980099	TPI1	94.9187624	11628
chr12.fa	6980111	6982521	SPSB2	8.073254578	684
chr12.fa	6993145	6993768	RPL13P5	0	0

chr12.fa	6993846	6994902	DSTNP2	0	0
chr12.fa	7013897	7023406	LRRC23	4.921515028	532
chr12.fa	7023614	7032859	ENO2	0.944753772	152
chr12.fa	7033626	7051484	ATN1	36.52174403	10716
chr12.fa	7053203	7055165	C12orf57	141.2315773	5092
chr12.fa	7055740	7070479	PTPN6	11.66130079	1976
chr12.fa	7072862	7072929	MIR200C	0	0
chr12.fa	7073260	7073354	MIR141	12.04850977	76
chr12.fa	7074515	7079891	PHB2	86.37034471	8132
chr12.fa	7076500	7076769	SCARNA12	0	0
chr12.fa	7079944	7085165	EMG1	23.49790739	1671
chr12.fa	7085347	7125842	LPCAT3	8.969513098	1368
chr12.fa	7167980	7178335	C1S	0	0
chr12.fa	7187515	7245043	C1R	0.505133772	76
chr12.fa	7247146	7261869	C1RL	3.193306908	684
chr12.fa	7261076	7274447	LOC283314	0.67336109	228
chr12.fa	7276280	7281466	RBP5	57.23042141	3572
chr12.fa	7282967	7311530	CLSTN3	2.830044339	752
chr12.fa	7341759	7371169	PEX5	16.68357148	3053
chr12.fa	7456928	7480969	ACSM4	0	0
chr12.fa	7507557	7596749	CD163L1	0	0
chr12.fa	7623410	7656414	CD163	0	0
chr12.fa	7801996	7818502	APOBEC1	0	0
chr12.fa	7842381	7848360	GDF3	0	0
chr12.fa	7864089	7870152	DPPA3	0	0
chr12.fa	7882011	7902069	CLEC4C	0	0
chr12.fa	7917812	7926717	NANOGNB	0	0
chr12.fa	7941995	7948655	NANOG	0	0
chr12.fa	7966397	8025495	SLC2A14	0	0
chr12.fa	8071824	8088892	SLC2A3	0	0
chr12.fa	8185359	8208118	FOXJ2	6.263869623	2280
chr12.fa	8210919	8218955	C3AR1	0	0
chr12.fa	8234807	8250373	NECAP1	22.5124899	3876
chr12.fa	8276228	8291203	CLEC4A	0	0
chr12.fa	8286365	8287448	POU5F1P3	0	0
chr12.fa	8325150	8332642	ZNF705A	0	0
chr12.fa	8332805	8353596	FAM66C	1.75230514	380
chr12.fa	8373856	8380214	FAM90A1	0	0
chr12.fa	8383645	8395542	LOC653113	1.068250998	152
chr12.fa	8509560	8543348	LOC389634	0	0
chr12.fa	8608591	8630926	CLEC6A	0	0
chr12.fa	8666136	8674960	CLEC4D	0	0
chr12.fa	8685901	8693558	CLEC4E	0	0

chr12.fa	8754762	8765442	AICDA	0	0
chr12.fa	8798540	8815433	MFAP5	0	0
chr12.fa	8850518	8929787	RIMKLB	2.944957001	1140
chr12.fa	8975150	9029377	A2ML1	3.088786086	1064
chr12.fa	9067316	9092963	PHC1	1.964208305	532
chr12.fa	9094061	9102252	M6PR	10.12180245	912
chr12.fa	9142221	9163340	KLRG1	0	0
chr12.fa	9208185	9217666	C12orf33	0	0
chr12.fa	9217773	9220651	LOC144571	0	0
chr12.fa	9220779	9268558	A2M	0	0
chr12.fa	9301436	9360966	PZP	0	0
chr12.fa	9392599	9395645	LOC100499405	0	0
chr12.fa	9436253	9466684	LOC642846	0	0
chr12.fa	9570287	9600768	DDX12	0	0
chr12.fa	9747870	9760497	KLRB1	0	0
chr12.fa	9800643	9811008	LOC374443	3.505664524	608
chr12.fa	9822308	9852151	CLEC2D	0.421095416	152
chr12.fa	9875091	9885860	CLECL1	0	0
chr12.fa	9905082	9913497	CD69	0	0
chr12.fa	9980077	9997603	KLRF1	0.922313423	76
chr12.fa	10004968	10022458	CLEC2B	0	0
chr12.fa	10034088	10048432	KLRF2	0	0
chr12.fa	10065826	10084980	CLEC2A	0	0
chr12.fa	10124008	10138194	CLEC12A	0	0
chr12.fa	10145662	10151899	CLEC1B	0	0
chr12.fa	10163231	10171399	CLEC12B	0	0
chr12.fa	10183276	10218565	CLEC9A	0	0
chr12.fa	10223080	10251605	CLEC1A	0	0
chr12.fa	10269376	10282868	CLEC7A	0	0
chr12.fa	10310899	10324790	OLR1	0.45498185	76
chr12.fa	10331557	10344403	C12orf59	11.71265756	2169
chr12.fa	10365489	10375724	GABARAPL1	131.197729	16264
chr12.fa	10457050	10469850	KLRD1	0	0
chr12.fa	10524952	10562745	KLRK1	1.959991327	228
chr12.fa	10559983	10562356	KLRC4	0	0
chr12.fa	10564914	10573194	KLRC3	0	0
chr12.fa	10583206	10588592	KLRC2	0	0
chr12.fa	10598638	10607215	KLRC1	0	0
chr12.fa	10741077	10752434	KLRAP1	0	0
chr12.fa	10756364	10766208	MAGOHB	2.943601543	593
chr12.fa	10771538	10826891	STYK1	1.91089365	380
chr12.fa	10851676	10875953	CSDA	33.60028162	4404
chr12.fa	10954131	10955226	TAS2R7	0	0

chr12.fa	10958650	10959579	TAS2R8	0	0
chr12.fa	10961693	10962767	TAS2R9	0	0
chr12.fa	10977945	10978868	TAS2R10	0	0
chr12.fa	10998448	11002075	PRR4	0	0
chr12.fa	11033560	11187484	PRH1	1.860741728	96
chr12.fa	11060525	11062161	TAS2R13	0	0
chr12.fa	11081834	11087444	PRH2	0	0
chr12.fa	11090853	11091806	TAS2R14	0	0
chr12.fa	11138512	11139511	TAS2R50	0	0
chr12.fa	11148561	11150474	TAS2R20	0	0
chr12.fa	11174271	11175170	TAS2R19	0	0
chr12.fa	11182986	11184006	TAS2R31	0	0
chr12.fa	11213964	11214893	TAS2R46	0	0
chr12.fa	11243886	11244912	TAS2R43	0	0
chr12.fa	11285884	11286843	TAS2R30	0	0
chr12.fa	11323780	11325723	LOC100129361	11.09366537	1292
chr12.fa	11338599	11339543	TAS2R42	0	0
chr12.fa	11418857	11422641	PRB3	0	0
chr12.fa	11460015	11463366	PRB4	0	0
chr12.fa	11504757	11508524	PRB1	0	0
chr12.fa	11544474	11548498	PRB2	0	0
chr12.fa	11700964	11717335	LOC338817	0	0
chr12.fa	11802788	12048325	ETV6	23.16792883	9196
chr12.fa	12223878	12252627	BCL2L14	2.565278336	380
chr12.fa	12268961	12419811	LRP6	3.632625696	2432
chr12.fa	12482218	12503169	MANSC1	1.993275335	304
chr12.fa	12508342	12510001	LOH12CR2	2.23258886	228
chr12.fa	12510020	12619838	LOH12CR1	8.234554002	1140
chr12.fa	12626216	12715448	DUSP16	76.52490495	32072
chr12.fa	12764831	12798041	CREBL2	42.44946023	10564
chr12.fa	12813995	12849121	GPR19	0	0
chr12.fa	12870302	12875305	CDKN1B	22.76882194	3648
chr12.fa	12878851	12944399	APOLD1	0	0
chr12.fa	12917583	12917677	MIR613	0	0
chr12.fa	12966280	12982915	DDX47	20.78819754	2508
chr12.fa	13028411	13029070	RPL13AP20	1.734232375	76
chr12.fa	13043956	13066600	GPRC5A	4.014714062	760
chr12.fa	13068763	13068852	MIR614	0	0
chr12.fa	13093709	13103318	GPRC5D	1.102739857	76
chr12.fa	13127799	13153243	HEBP1	39.79592656	3192
chr12.fa	13153376	13157764	HTR7P1	1.043099733	304
chr12.fa	13197315	13236383	KIAA1467	5.345170753	1672
chr12.fa	13237042	13256619	GSG1	0	0

chr12.fa	13349602	13369708	EMP1	3.673891842	684
chr12.fa	13523605	13529679	C12orf36	0	0
chr12.fa	13714410	14133022	GRIN2B	0	0
chr12.fa	14518611	14651697	ATF7IP	32.38308092	10022
chr12.fa	14656597	14720791	PLBD1	10.07165053	1292
chr12.fa	14765568	14849519	GUCY2C	1.191748223	304
chr12.fa	14923654	14924065	HIST4H4	61.11983097	1672
chr12.fa	14927270	14930936	H2AFJ	34.02303371	8284
chr12.fa	14939412	14956401	WBP11	30.21706008	5365
chr12.fa	14956506	14976791	C12orf60	0	0
chr12.fa	14957584	14967116	C12orf69	2.176111471	304
chr12.fa	14982245	14996413	ART4	0	0
chr12.fa	15034115	15038853	MGP	1.554107154	152
chr12.fa	15066976	15091463	ERP27	70.0476261	7144
chr12.fa	15094950	15114562	ARHGDIB	51.7585907	4179
chr12.fa	15125956	15134799	PDE6H	0	0
chr12.fa	15260716	15374411	RERG	1.969178316	304
chr12.fa	15475487	15750335	PTPRO	0.178769764	76
chr12.fa	15773075	15942510	EPS8	14.86138498	4028
chr12.fa	16035288	16056410	STRAP	52.27351388	6612
chr12.fa	16064186	16190315	DERA	10.62286985	1216
chr12.fa	16341419	16430619	SLC15A5	0	0
chr12.fa	16500076	16517344	MGST1	37.14962199	2812
chr12.fa	16701307	16761024	LMO3	0.589774553	152
chr12.fa	17141681	17143562	LOC728622	0	0
chr12.fa	18233803	18243114	RERGL	0	0
chr12.fa	18414474	18801352	PIK3C2G	2.835466168	912
chr12.fa	18836116	18890918	PLCZ1	0	0
chr12.fa	18891045	18892122	CAPZA3	0	0
chr12.fa	19282626	19529333	PLEKHA5	14.4954115	8796
chr12.fa	19592608	19675173	AEBP2	7.525951022	2548
chr12.fa	20522197	20833881	PDE3A	0.832702631	228
chr12.fa	20848289	20906320	SLCO1C1	0	0
chr12.fa	20963638	21069658	SLCO1B3	0	0
chr12.fa	21168630	21243040	LST-3TM12	0	0
chr12.fa	21284128	21392730	SLCO1B1	0	0
chr12.fa	21417534	21548371	SLCO1A2	0	0
chr12.fa	21525802	21532914	IAPP	0	0
chr12.fa	21590538	21624182	PYROXD1	8.505043047	1444
chr12.fa	21624362	21654603	RECQL	5.154051267	757
chr12.fa	21654699	21671337	GOLT1B	2.798567607	608
chr12.fa	21679256	21684210	C12orf39	1.963304667	76
chr12.fa	21689123	21757781	GYS2	0	0

chr12.fa	21788275	21810789	LDHB	273.2752622	29032
chr12.fa	21917889	21927747	KCNJ8	0	0
chr12.fa	21950324	22089628	ABCC9	0	0
chr12.fa	22199159	22218602	CMAS	39.44651978	4560
chr12.fa	22346325	22487648	ST8SIA1	0.117774183	76
chr12.fa	22601518	22697452	KIAA0528	13.76542241	4028
chr12.fa	22778076	22843608	ETNK1	5.170015542	2508
chr12.fa	23685231	24715380	SOX5	0.728483022	228
chr12.fa	24365355	24365429	MIR920	0	0
chr12.fa	24719898	24737102	C12orf67	0	0
chr12.fa	24962958	25102393	BCAT1	0	0
chr12.fa	25070636	25071266	DAD1P1	0	0
chr12.fa	25146365	25150373	C12orf77	0	0
chr12.fa	25205241	25261222	LRMP	1.908483948	350
chr12.fa	25261270	25348094	CASC1	1.294311162	228
chr12.fa	25348150	25357949	LYRM5	12.10152321	912
chr12.fa	25358180	25403854	KRAS	9.501454805	3420
chr12.fa	25629016	25801488	IFLTD1	0	0
chr12.fa	26026953	26027012	MIR4302	0	0
chr12.fa	26111964	26232825	RASSF8	12.79340889	6004
chr12.fa	26272959	26278003	BHLHE41	17.56281148	4408
chr12.fa	26348269	26387708	SSPN	1.937099158	608
chr12.fa	26488285	26986131	ITPR2	5.337339222	4454
chr12.fa	27058112	27091254	C12orf11	8.84616648	1748
chr12.fa	27091305	27119581	FGFR1OP2	8.362720025	1748
chr12.fa	27124506	27167339	TM7SF3	8.960777929	2584
chr12.fa	27175483	27182682	MED21	16.97650087	1976
chr12.fa	27233990	27235455	C12orf71	0	0
chr12.fa	27397078	27478890	STK38L	6.738279695	2280
chr12.fa	27485988	27573466	ARNTL2	0.593087893	76
chr12.fa	27619743	27655118	C12orf70	0	0
chr12.fa	27677045	27848497	PPFIBP1	11.42078241	4672
chr12.fa	27849428	27850566	REP15	0	0
chr12.fa	27863706	27909237	MRPS35	32.58248376	4104
chr12.fa	27915599	27924209	LOC100287284	0	0
chr12.fa	27933187	27955973	KLHDC5	4.600422243	1976
chr12.fa	28111017	28124916	PTHLH	0	0
chr12.fa	28410133	28703099	CCDC91	10.57798915	1640
chr12.fa	29376598	29487006	FAR2	1.639049148	228
chr12.fa	29493579	29534143	ERGIC2	13.8148213	1630
chr12.fa	29580489	29650619	OVCH1	0	0
chr12.fa	29653746	29937692	TMTC1	0.622606742	380
chr12.fa	30781915	30848929	IPO8	15.89168317	6004

chr12.fa	30862486	30907448	CAPRIN2	7.055155502	2101
chr12.fa	31079838	31149537	TSPAN11	0.617335519	228
chr12.fa	31226779	31257725	DDX11	0.582997267	152
chr12.fa	31433520	31479121	FAM60A	16.1522322	3344
chr12.fa	31477250	31478879	FLJ13224	0	0
chr12.fa	31535157	31743952	DENND5B	4.509757207	2812
chr12.fa	31800094	31822016	C12orf72	2.403677699	380
chr12.fa	31824071	31882108	AMN1	7.594627527	1064
chr12.fa	31944119	31945175	H3F3C	0	0
chr12.fa	32112353	32146043	C12orf35	9.550703089	3952
chr12.fa	32260185	32531141	BICD1	7.567066561	1748
chr12.fa	32655041	32798984	FGD4	9.631879923	5305
chr12.fa	32832137	32898584	DNM1L	28.66159747	8777
chr12.fa	32899477	32908874	YARS2	6.885723334	988
chr12.fa	32943680	33049780	PKP2	7.477756983	2204
chr12.fa	33528348	33592754	SYT10	0.696705077	152
chr12.fa	34175216	34181236	ALG10	0.365973484	76
chr12.fa	38710557	38723528	ALG10B	0.454530031	304
chr12.fa	39046002	39299420	CPNE8	1.322926373	304
chr12.fa	39687030	39837192	KIF21A	14.27597801	6295
chr12.fa	39945022	40013843	ABCD2	0	0
chr12.fa	40019972	40115720	C12orf40	0	0
chr12.fa	40148823	40499661	SLC2A13	0.163407914	76
chr12.fa	40618813	40763086	LRRK2	7.569476263	4636
chr12.fa	41086358	41464094	CNTN1	0.329677349	75
chr12.fa	41582250	41968392	PDZRN4	0.279525427	76
chr12.fa	42475648	42538673	GXYLT1	2.90429328	1444
chr12.fa	42550907	42632151	YAF2	0.375612292	228
chr12.fa	42705888	42719932	ZCRB1	23.88963457	2925
chr12.fa	42719947	42842422	PPHLN1	9.354011167	2580
chr12.fa	42852140	42983572	PRICKLE1	1.192802467	380
chr12.fa	43748012	43945724	ADAMTS20	0	0
chr12.fa	44122412	44152596	PUS7L	1.301540268	380
chr12.fa	44152747	44183346	IRAK4	4.746058605	1368
chr12.fa	44187526	44200178	TWF1	20.67433913	4180
chr12.fa	44229962	44783541	TMEM117	0.848817513	152
chr12.fa	44902058	45307711	NELL2	0	0
chr12.fa	45408539	45444882	DBX2	0	0
chr12.fa	45456401	45459194	RACGAP1P	0	0
chr12.fa	45566817	45609769	PLEKHA9	4.333246539	532
chr12.fa	45580874	45581252	RNY5	0	0
chr12.fa	45609790	45834187	ANO6	6.314021545	2964
chr12.fa	46119503	46121704	LOC400027	9.356420868	1368

chr12.fa	46123620	46301819	ARID2	15.53745699	8734
chr12.fa	46312914	46384401	SRSF2IP	23.3325416	11768
chr12.fa	46576841	46663208	SLC38A1	13.35652611	7524
chr12.fa	46751971	46766645	SLC38A2	30.41736655	9864
chr12.fa	47158544	47219780	SLC38A4	1.149427832	304
chr12.fa	47469490	47473734	AMIGO2	2.027914801	532
chr12.fa	47602203	47610047	LOC100233209	0	0
chr12.fa	47610052	47630443	FAM113B	0	0
chr12.fa	48055715	48099844	RPAP3	9.934146912	2432
chr12.fa	48103518	48119355	ENDOU	0	0
chr12.fa	48128453	48152889	RAPGEF3	4.716991575	1976
chr12.fa	48166967	48176506	SLC48A1	6.85846358	1368
chr12.fa	48176537	48213763	HDAC7	9.900109872	2799
chr12.fa	48235320	48298814	VDR	3.116196446	988
chr12.fa	48357330	48362661	TMEM106C	25.48365241	2687
chr12.fa	48366748	48398285	COL2A1	0	0
chr12.fa	48436757	48499641	SENP1	7.265853817	2280
chr12.fa	48499656	48540187	PFKM	11.02318159	2733
chr12.fa	48541572	48551377	ASB8	10.95104114	1900
chr12.fa	48577366	48579709	C12orf68	0	0
chr12.fa	48596122	48597075	OR10AD1	0	0
chr12.fa	48722763	48724062	H1FNT	0	0
chr12.fa	48733793	48745029	ZNF641	7.575349912	2356
chr12.fa	48866448	48866843	ANP32D	0	0
chr12.fa	48876286	48890297	C12orf54	0	0
chr12.fa	48919415	48921886	OR8S1	0	0
chr12.fa	48961467	48963829	LALBA	0	0
chr12.fa	49046995	49076035	C12orf41	14.3914931	2280
chr12.fa	49048165	49048226	SNORA34	0	0
chr12.fa	49048302	49048313	MIR1291	0	0
chr12.fa	49050431	49050565	SNORA2A	0	0
chr12.fa	49061240	49061376	SNORA2B	0	0
chr12.fa	49082245	49086229	LOC144438	9.191356285	2432
chr12.fa	49086752	49110781	CCNT1	12.03450338	2052
chr12.fa	49121218	49159568	LOC255411	0	0
chr12.fa	49159975	49182820	ADCY6	14.58698017	5852
chr12.fa	49212512	49222724	CACNB3	6.787829192	1216
chr12.fa	49223539	49245957	DDX23	30.13046141	6536
chr12.fa	49250916	49259653	RND1	0	0
chr12.fa	49297893	49315359	CCDC65	0.63058888	76
chr12.fa	49315742	49319330	FKBP11	0	0
chr12.fa	49329992	49351252	ARF3	82.84419772	19456
chr12.fa	49359123	49365641	WNT10B	0	0

chr12.fa	49372236	49376396	WNT1	0	0
chr12.fa	49388933	49393088	DDN	0	0
chr12.fa	49396055	49412592	PRKAG1	47.69056198	5513
chr12.fa	49412758	49449107	MLL2	18.96089043	24448
chr12.fa	49458468	49463775	RHEBL1	0	0
chr12.fa	49483206	49488602	DHH	0	0
chr12.fa	49490923	49504680	LMBR1L	4.394844545	684
chr12.fa	49521567	49525304	TUBA1B	43.14345439	5016
chr12.fa	49578583	49582861	TUBA1A	5.499692891	608
chr12.fa	49658865	49667113	TUBA1C	29.48119734	3040
chr12.fa	49688909	49692481	PRPH	0	0
chr12.fa	49716971	49725514	TROAP	0	0
chr12.fa	49726200	49730971	C1QL4	0	0
chr12.fa	49741041	49745685	DNAJC22	1.879416918	228
chr12.fa	49760688	49921207	SPATS2	8.732458669	2201
chr12.fa	49782957	49786116	LOC100335030	0	0
chr12.fa	49932940	49952077	KCNH3	0.296995766	76
chr12.fa	49952086	49961911	MCRS1	17.24051384	2254
chr12.fa	49976658	49999433	FAM186B	0.339617369	76
chr12.fa	50017403	50035807	PRPF40B	6.169138215	988
chr12.fa	50031724	50101197	FMNL3	0.583750298	380
chr12.fa	50135293	50158717	TMBIM6	44.90374167	10006
chr12.fa	50184929	50222208	NCKAP5L	4.905400147	1596
chr12.fa	50222326	50234937	LOC100286844	0	0
chr12.fa	50229826	50236912	BCDIN3D	2.166924482	382
chr12.fa	50260680	50297720	FAIM2	0	0
chr12.fa	50302666	50305646	LOC283332	0	0
chr12.fa	50344524	50352664	AQP2	33.14138401	9196
chr12.fa	50355279	50359461	AQP5	0	0
chr12.fa	50366620	50370922	AQP6	0	0
chr12.fa	50382945	50419307	RACGAP1	1.323679405	304
chr12.fa	50451487	50477394	ACCN2	1.211929477	304
chr12.fa	50478983	50494494	SMARCD1	10.0141189	2280
chr12.fa	50497801	50505095	GPD1	15.12344007	2888
chr12.fa	50505900	50514234	C12orf62	68.56219545	2736
chr12.fa	50523581	50561097	LASS5	3.534580947	456
chr12.fa	50569563	50677353	LIMA1	33.83311907	8404
chr12.fa	50627925	50627995	MIR1293	0	0
chr12.fa	50721095	50790405	FAM186A	0	0
chr12.fa	50794592	50873788	LARP4	18.76901791	8184
chr12.fa	50898768	51142450	DIP2B	13.79042307	7980
chr12.fa	51157789	51214943	ATF1	5.940065923	988
chr12.fa	51236701	51281663	TMPRSS12	0	0

chr12.fa	51318534	51326300	METTL7A	7.090548	1596
chr12.fa	51347782	51364289	HIGD1C	0	0
chr12.fa	51373566	51422058	SLC11A2	5.021216447	1742
chr12.fa	51442084	51454207	LETMD1	37.04404693	5244
chr12.fa	51454990	51477333	CSRNP2	8.964241875	2660
chr12.fa	51487539	51566926	TFCP2	22.91325346	5652
chr12.fa	51580719	51611477	POU6F1	3.022971101	1064
chr12.fa	51632508	51640501	DAZAP2	109.7399355	20140
chr12.fa	51639133	51664202	SMAGP	14.55008161	1140
chr12.fa	51674822	51717938	BIN2	0.517634101	76
chr12.fa	51722227	51740463	CELA1	0	0
chr12.fa	51745833	51785200	GALNT6	0.253169312	76
chr12.fa	51818594	51902980	SLC4A8	0	0
chr12.fa	51985020	52202299	SCN8A	1.905773033	912
chr12.fa	52211676	52225701	FIGNL2	0.487663433	152
chr12.fa	52281793	52285505	ANKRD33	0	0
chr12.fa	52301202	52317145	ACVRL1	0.259946598	76
chr12.fa	52345451	52390863	ACVR1B	8.095845534	2584
chr12.fa	52400748	52409671	GRASP	1.18482033	152
chr12.fa	52445191	52453287	NR4A1	13.28950628	2356
chr12.fa	52463758	52471279	C12orf44	16.72709672	1596
chr12.fa	52562780	52585784	KRT80	6.822016838	1748
chr12.fa	52604714	52617597	LOC283404	0	0
chr12.fa	52626954	52642709	KRT7	313.5037303	35429
chr12.fa	52679697	52685299	KRT81	0	0
chr12.fa	52695649	52702947	KRT86	0	0
chr12.fa	52708085	52715182	KRT83	0	0
chr12.fa	52753790	52761309	KRT85	0	0
chr12.fa	52771596	52779417	KRT84	0	0
chr12.fa	52787735	52800176	KRT82	0	0
chr12.fa	52817854	52828110	KRT75	0	0
chr12.fa	52840435	52845910	KRT6B	0	0
chr12.fa	52862300	52867569	KRT6C	0	0
chr12.fa	52880958	52887181	KRT6A	0	0
chr12.fa	52908359	52914243	KRT5	0	0
chr12.fa	52937693	52946931	KRT71	0	0
chr12.fa	52959603	52967609	KRT74	0	0
chr12.fa	52979373	52995322	KRT72	0	0
chr12.fa	53001354	53012343	KRT73	0	0
chr12.fa	53038342	53045959	KRT2	0	0
chr12.fa	53068520	53074191	KRT1	0	0
chr12.fa	53083411	53097247	KRT77	0	0
chr12.fa	53161939	53171129	KRT76	0	0

chr12.fa	53183469	53189892	KRT3	0	0
chr12.fa	53200333	53208335	KRT4	2.666033999	456
chr12.fa	53215231	53228077	KRT79	0	0
chr12.fa	53232746	53242778	KRT78	3.557623722	420
chr12.fa	53290971	53298868	KRT8	269.4227512	32129
chr12.fa	53342655	53346685	KRT18	320.6394602	32680
chr12.fa	53400062	53435993	EIF4B	151.1372596	40422
chr12.fa	53436973	53447358	LOC283335	5.07588656	912
chr12.fa	53440810	53458099	TENC1	2.740433547	906
chr12.fa	53458163	53473204	SPRYD3	31.11874043	5928
chr12.fa	53491436	53496128	IGFBP6	1.18482033	76
chr12.fa	53497274	53518323	SOAT2	0	0
chr12.fa	53551447	53574430	CSAD	1.274129908	228
chr12.fa	53574535	53584654	ZNF740	15.32962019	4332
chr12.fa	53585107	53601000	ITGB7	0.826075951	152
chr12.fa	53604353	53626036	RARG	15.9443954	3572
chr12.fa	53645370	53648190	MFSD5	6.714785101	988
chr12.fa	53662083	53687427	ESPL1	0.345641624	152
chr12.fa	53689235	53693234	PFDN5	396.4652504	18085
chr12.fa	53693470	53700965	C12orf10	25.43575958	2052
chr12.fa	53701240	53715412	AAAS	11.21550593	1368
chr12.fa	53720360	53730004	SP7	0	0
chr12.fa	53773979	53810226	SP1	25.27717107	12920
chr12.fa	53817639	53825318	AMHR2	0	0
chr12.fa	53835433	53840427	PRR13	73.87905221	5862
chr12.fa	53845886	53874275	PCBP2	251.4463747	41739
chr12.fa	53874947	53893444	MAP3K12	0	0
chr12.fa	53894705	53900215	TARBP2	9.212139964	1216
chr12.fa	53900474	53901422	NPFF	0	0
chr12.fa	53905843	54020199	ATF7	13.11329682	5852
chr12.fa	54058944	54070512	ATP5G2	202.528672	17307
chr12.fa	54104902	54121307	CALCOCO1	23.67381563	4788
chr12.fa	54332576	54340328	HOXC13	0	0
chr12.fa	54348714	54350350	HOXC12	0	0
chr12.fa	54356096	54362515	HOTAIR	0	0
chr12.fa	54366910	54370203	HOXC11	0.561912374	76
chr12.fa	54378946	54384062	HOXC10	9.932791455	1292
chr12.fa	54385522	54385631	MIR196A2	0	0
chr12.fa	54393877	54397120	HOXC9	0.742790627	76
chr12.fa	54402890	54406545	HOXC8	0	0
chr12.fa	54422194	54424607	HOXC6	0.680891408	76
chr12.fa	54426832	54429144	HOXC5	0	0
chr12.fa	54427734	54427829	MIR615	0	0

chr12.fa	54446944	54449814	HOXC4	0.641884358	76
chr12.fa	54452038	54516018	FLJ12825	0	0
chr12.fa	54472623	54475607	LOC100240735	0	0
chr12.fa	54495993	54496483	LOC100240734	0	0
chr12.fa	54519855	54526626	LOC400043	1.641007031	152
chr12.fa	54575252	54582757	SMUG1	14.58095592	1520
chr12.fa	54624731	54673915	CBX5	20.54451643	16188
chr12.fa	54674488	54677018	HNRNPA1	29.44685909	393
chr12.fa	54685896	54694791	NFE2	0	0
chr12.fa	54718911	54745633	COPZ1	117.5714668	14809
chr12.fa	54731000	54731098	MIR148B	0	0
chr12.fa	54756229	54758270	GPR84	0	0
chr12.fa	54762920	54785083	ZNF385A	19.59946145	3040
chr12.fa	54789045	54813050	ITGA5	0	0
chr12.fa	54849736	54867386	GTSF1	2.81920068	152
chr12.fa	54891495	54936899	NCKAP1L	0	0
chr12.fa	54943177	54973023	PDE1B	0	0
chr12.fa	54973024	54982443	PPP1R1A	4.992902449	608
chr12.fa	55001971	55004246	GLYCAM1	0	0
chr12.fa	55024623	55028663	LACRT	0	0
chr12.fa	55038375	55042149	DCD	0	0
chr12.fa	55248299	55252174	MUCL1	0	0
chr12.fa	55343865	55378456	KIAA0748	0	0
chr12.fa	55413729	55423801	NEUROD4	0	0
chr12.fa	55523553	55524560	OR9K2	0	0
chr12.fa	55614809	55615759	OR10A7	0	0
chr12.fa	55641072	55642010	OR6C74	0	0
chr12.fa	55688072	55689016	OR6C6	0	0
chr12.fa	55714384	55715322	OR6C1	0	0
chr12.fa	55725485	55726420	OR6C3	0	0
chr12.fa	55758895	55759833	OR6C75	0	0
chr12.fa	55794313	55795251	OR6C65	0	0
chr12.fa	55820038	55820976	OR6C76	0	0
chr12.fa	55845998	55846936	OR6C2	0	0
chr12.fa	55862984	55863922	OR6C70	0	0
chr12.fa	55886147	55887100	OR6C68	0	0
chr12.fa	55945011	55945940	OR6C4	0	0
chr12.fa	56030676	56031617	OR10P1	0	0
chr12.fa	56075330	56078353	METTL7B	1.563745962	152
chr12.fa	56078395	56106089	ITGA7	0.523658356	152
chr12.fa	56110076	56113491	BLOC1S1	17.40874116	734
chr12.fa	56114151	56115278	RDH5	0	0
chr12.fa	56119230	56122910	CD63	75.40680325	5092

chr12.fa	56137064	56146665	GDF11	1.474285777	380
chr12.fa	56151054	56211540	SARNP	43.82163489	2706
chr12.fa	56211806	56214743	ORMDL2	7.166604218	532
chr12.fa	56214960	56223391	DNAJC14	15.21199662	3116
chr12.fa	56223421	56229213	LOC440104	11.6356977	2052
chr12.fa	56230031	56236735	MMP19	1.409073218	228
chr12.fa	56295197	56321697	WIBG	32.29301831	2736
chr12.fa	56324946	56347807	DGKA	11.2478863	2183
chr12.fa	56347889	56359826	SILV	0.53841778	76
chr12.fa	56360556	56366568	CDK2	14.47613388	2204
chr12.fa	56367862	56388488	RAB5B	126.4837495	27664
chr12.fa	56391043	56399309	SUOX	22.78056924	3724
chr12.fa	56414689	56432219	IKZF4	1.889808757	684
chr12.fa	56435686	56438007	RPS26	6.713279038	304
chr12.fa	56473892	56497128	ERBB3	28.67771235	11324
chr12.fa	56498103	56507694	PA2G4	28.21715806	4920
chr12.fa	56510374	56511616	RPL41	73.49801809	2850
chr12.fa	56512030	56516278	ZC3H10	3.237886394	456
chr12.fa	56521986	56538460	ESYT1	5.271373631	1497
chr12.fa	56546204	56551771	MYL6B	21.00567315	1484
chr12.fa	56552045	56555366	MYL6	539.6510959	28988
chr12.fa	56555636	56583351	SMARCC2	34.81898839	12489
chr12.fa	56598286	56615735	RNF41	31.59149383	7220
chr12.fa	56618125	56623638	OBFC2B	10.62091197	988
chr12.fa	56623820	56631590	SLC39A5	7.745083293	1064
chr12.fa	56631630	56652143	ANKRD52	24.15786451	13822
chr12.fa	56660642	56664750	COQ10A	5.707228472	684
chr12.fa	56665483	56694175	CS	74.50361683	14722
chr12.fa	56704213	56710006	CNPY2	18.61148365	1824
chr12.fa	56710129	56727837	PAN2	6.940845266	2508
chr12.fa	56732663	56734194	IL23A	0	0
chr12.fa	56735382	56754037	STAT2	23.12530723	6968
chr12.fa	56754355	56756583	APOF	0	0
chr12.fa	56810157	56843200	TIMELESS	4.232641482	1444
chr12.fa	56843286	56848435	MIP	0	0
chr12.fa	56862301	56864735	SPRYD4	9.386542143	1292
chr12.fa	56864768	56882181	GLS2	0.883456979	152
chr12.fa	56915609	56989379	RBMS2	7.919033653	4146
chr12.fa	56989981	57030163	BAZ2A	40.78285012	22565
chr12.fa	57031959	57039852	ATP5B	317.3601571	38836
chr12.fa	57037464	57037538	SNORD59B	0	0
chr12.fa	57038811	57038885	SNORD59A	0	0
chr12.fa	57057125	57082078	PTGES3	51.87907579	6600

chr12.fa	57106211	57119326	NACA	105.454883	48531
chr12.fa	57125364	57146146	PRIM1	0	0
chr12.fa	57157108	57181574	HSD17B6	0.756947626	76
chr12.fa	57316938	57328189	SDR9C7	0	0
chr12.fa	57345216	57351418	RDH16	0.664324707	76
chr12.fa	57388355	57390469	GPR182	0	0
chr12.fa	57392617	57400297	ZBTB39	6.055129191	2508
chr12.fa	57403781	57410344	TAC3	9.2413576	586
chr12.fa	57422301	57443893	MYO1A	0	0
chr12.fa	57449426	57472574	TMEM194A	0.611311264	228
chr12.fa	57482677	57489186	NAB2	4.338969581	760
chr12.fa	57489260	57505196	STAT6	60.27131467	16784
chr12.fa	57522282	57607125	LRP1	0.76884553	760
chr12.fa	57588287	57588359	MIR1228	0	0
chr12.fa	57610578	57620232	NXPH4	0.634504646	76
chr12.fa	57623356	57628685	SHMT2	6.487369479	1216
chr12.fa	57628719	57634475	NDUFA4L2	0	0
chr12.fa	57637242	57644969	STAC3	3.483224175	380
chr12.fa	57647548	57704246	R3HDM2	46.93662648	12385
chr12.fa	57828543	57844609	INHBC	0	0
chr12.fa	57849096	57851791	INHBE	0	0
chr12.fa	57853918	57866037	GLI1	0.31717702	76
chr12.fa	57866048	57873633	ARHGAP9	0	0
chr12.fa	57881851	57910370	MARS	20.51816032	3722
chr12.fa	57910434	57914300	DDIT3	8.098707055	456
chr12.fa	57916659	57923931	MBD6	12.68000229	3494
chr12.fa	57924094	57940978	DCTN2	59.02323966	6729
chr12.fa	57943847	57978554	KIF5A	0	0
chr12.fa	57984942	57997211	PIP4K2C	23.11265629	4940
chr12.fa	57998604	58003583	DTX3	9.025990488	1216
chr12.fa	58003963	58011026	ARHGEF25	0	0
chr12.fa	58013693	58019550	SLC26A10	0	0
chr12.fa	58019935	58026985	B4GALNT1	0	0
chr12.fa	58087738	58115340	OS9	14.01241686	2660
chr12.fa	58118994	58135940	AGAP2	0	0
chr12.fa	58120748	58122100	LOC100130776	0	0
chr12.fa	58138784	58142002	TSPAN31	6.670205615	760
chr12.fa	58142027	58146164	CDK4	47.59116177	4484
chr12.fa	58148900	58153689	9-Mar	4.18414623	684
chr12.fa	58156117	58160976	CYP27B1	0.460253073	76
chr12.fa	58162351	58165914	METTL1	6.130281771	532
chr12.fa	58166383	58176324	FAM119B	9.269671598	1748
chr12.fa	58176528	58196639	TSFM	6.263267198	912

chr12.fa	58191371	58209852	AVIL	0	0
chr12.fa	58213710	58240747	CTDSP2	54.12823136	18042
chr12.fa	58218392	58218475	MIR26A2	0	0
chr12.fa	58335445	58351052	XRCC6BP1	3.946941194	304
chr12.fa	59265937	59314262	LRIG3	7.459383005	2052
chr12.fa	60083126	60175408	SLC16A7	0.323201275	76
chr12.fa	62102041	62586551	FAM19A2	0.564472683	152
chr12.fa	62654187	62799898	USP15	7.943431885	2432
chr12.fa	62860597	62991363	MON2	7.828519223	5395
chr12.fa	62995531	62997214	C12orf61	0	0
chr12.fa	62997466	62997549	MIRLET7I	0	0
chr12.fa	63037763	63328665	PPM1H	32.65236512	13442
chr12.fa	63540216	63546590	AVPR1A	0	0
chr12.fa	63952693	64062354	DPY19L2	1.410729888	380
chr12.fa	64173637	64202887	TMEM5	15.77677051	1520
chr12.fa	64238541	64541613	SRGAP1	5.652859571	3181
chr12.fa	64586419	64616076	C12orf66	5.723042141	988
chr12.fa	64660763	64784345	C12orf56	1.106505016	152
chr12.fa	64798153	64842463	XPOT	26.61455566	6975
chr12.fa	64845840	64895899	TBK1	16.61203345	3405
chr12.fa	65004293	65089192	RASSF3	58.21493526	5168
chr12.fa	65107222	65153226	GNS	2.232137041	760
chr12.fa	65218352	65274798	TBC1D30	9.205061464	4782
chr12.fa	65277554	65371302	FLJ41278	0.361605899	76
chr12.fa	65444404	65515346	WIF1	0.511007421	76
chr12.fa	65563351	65642141	LEMD3	6.441133323	2052
chr12.fa	65672423	65860687	MSRB3	0	0
chr12.fa	66151801	66220754	RPSAP52	0	0
chr12.fa	66218240	66360071	HMGA2	0	0
chr12.fa	66516849	66524533	LLPH	11.28614031	912
chr12.fa	66530717	66563807	TMBIM4	6.933164341	836
chr12.fa	66582978	66648394	IRAK3	0	0
chr12.fa	66696356	66731958	HELB	0.679686557	152
chr12.fa	66741211	67072925	GRIP1	4.298004648	1444
chr12.fa	67663061	67708388	CAND1	31.38561492	12312
chr12.fa	68042512	68056444	DYRK2	13.93816792	5700
chr12.fa	68548550	68553521	IFNG	0	0
chr12.fa	68595129	68619571	IL26	0	0
chr12.fa	68642025	68647281	IL22	0	0
chr12.fa	68688346	68726161	MDM1	1.786040967	532
chr12.fa	69004652	69054374	RAP1B	10.09288603	1412
chr12.fa	69021014	69021155	SNORA70G	0	0
chr12.fa	69080731	69136473	NUP107	12.44279725	2581

chr12.fa	69139936	69159853	SLC35E3	4.876935542	760
chr12.fa	69201971	69239212	MDM2	26.38683882	12902
chr12.fa	69244956	69357020	CPM	8.338623006	3724
chr12.fa	69633317	69668138	CPSF6	12.74898001	5548
chr12.fa	69742134	69748013	LYZ	0	0
chr12.fa	69753532	69784576	YEATS4	3.305659262	304
chr12.fa	69864129	69973562	FRS2	12.06598011	5548
chr12.fa	69978502	69978603	MIR3913-1	0	0
chr12.fa	69979208	69995357	CCT2	61.19287506	9272
chr12.fa	70002345	70004942	LRRC10	0	0
chr12.fa	70047389	70093196	BEST3	0	0
chr12.fa	70132631	70216984	RAB3IP	8.905655997	5928
chr12.fa	70636774	70748773	CNOT2	17.51958745	4788
chr12.fa	70760062	70824978	KCNMB4	0.701825694	76
chr12.fa	70910632	71031219	PTPRB	0	0
chr12.fa	71031862	71314584	PTPRR	0.655890751	152
chr12.fa	71518877	71551779	TSPAN8	37.4783957	2812
chr12.fa	71833813	71978621	LGR5	0	0
chr12.fa	72003379	72057676	ZFC3H1	11.10887661	5226
chr12.fa	72057750	72074428	THAP2	0.960416835	304
chr12.fa	72079878	72097839	TMEM19	3.194511759	988
chr12.fa	72148658	72181150	RAB21	27.38084088	4776
chr12.fa	72233487	72320629	TBC1D15	9.499798135	3714
chr12.fa	72242074	72244763	MRS2P2	0	0
chr12.fa	72332626	72426221	TPH2	0	0
chr12.fa	72647287	72665942	LOC283392	0	0
chr12.fa	72666529	73059422	TRHDE	0.899120042	287
chr12.fa	74931551	74935232	ATXN7L3B	64.03843185	15656
chr12.fa	75433896	75603511	KCNC2	0	0
chr12.fa	75669759	75723836	CAPS2	0.536158685	76
chr12.fa	75728463	75764169	GLIPR1L1	0	0
chr12.fa	75784889	75817820	GLIPR1L2	0	0
chr12.fa	75874513	75895702	GLIPR1	0.945506804	166
chr12.fa	75891419	75905418	KRR1	23.48992525	3266
chr12.fa	76419227	76425556	PHLDA1	1.935743701	760
chr12.fa	76438672	76478738	NAP1L1	34.23252717	11649
chr12.fa	76738266	76742222	BBS10	3.513947875	836
chr12.fa	76745578	76953589	OSBPL8	11.67877113	5615
chr12.fa	77157854	77247474	ZDHHC17	3.118756754	988
chr12.fa	77252496	77272799	CSRP2	27.94832569	1672
chr12.fa	77415026	77459360	E2F7	0	0
chr12.fa	78225069	78606790	NAV3	0.234644728	152
chr12.fa	79257773	79845788	SYT1	0.42967998	152

chr12.fa	79813037	79813101	MIR1252	0	0
chr12.fa	79985745	80084790	PAWR	6.476676427	839
chr12.fa	80167343	80329235	PPP1R12A	22.32242466	8810
chr12.fa	80603233	80772870	C12orf64	0.424860576	228
chr12.fa	80838126	81073968	PTPRQ	0.151660617	76
chr12.fa	81101408	81103256	MYF6	0	0
chr12.fa	81110708	81113447	MYF5	0	0
chr12.fa	81191171	81331694	LIN7A	6.514026807	532
chr12.fa	81226312	81226408	MIR617	0	0
chr12.fa	81329515	81329612	MIR618	0	0
chr12.fa	81471809	81649582	ACSS3	16.22753538	3268
chr12.fa	81653356	82153109	PPFIA2	0.282537554	76
chr12.fa	82746083	82752199	CCDC59	10.61789984	1140
chr12.fa	82752276	82873016	C12orf26	11.17875797	1460
chr12.fa	83080934	83528067	TMTC2	6.146246047	1976
chr12.fa	85253267	85306606	SLC6A15	0	0
chr12.fa	85408094	85430055	TSPAN19	0	0
chr12.fa	85430099	85638883	LRRIQ1	0.420492991	152
chr12.fa	85674036	85695561	ALX1	0	0
chr12.fa	86198331	86230318	RASSF9	1.911947894	228
chr12.fa	86268073	86276770	NTS	0	0
chr12.fa	86373037	87232681	MGAT4C	0	0
chr12.fa	88176663	88178488	MKRN9P	0	0
chr12.fa	88373816	88423176	C12orf50	0	0
chr12.fa	88429268	88443937	C12orf29	6.945514063	1140
chr12.fa	88444131	88535993	CEP290	7.577157188	3800
chr12.fa	88536073	88593664	TMTC3	2.383647052	1140
chr12.fa	88886570	88974250	KITLG	2.102916774	760
chr12.fa	89741837	89746296	DUSP6	7.711949891	1444
chr12.fa	89813498	89919344	POC1B	9.136384959	1900
chr12.fa	89917451	89918583	GALNT4	4.040919571	304
chr12.fa	89981826	90049844	ATP2B1	3.136829519	1444
chr12.fa	90102732	90105729	LOC338758	0.763574307	152
chr12.fa	91345992	91348953	C12orf12	0	0
chr12.fa	91357456	91398803	EPYC	0	0
chr12.fa	91444271	91452131	KERA	0	0
chr12.fa	91497232	91505542	LUM	0	0
chr12.fa	91539035	91576806	DCN	0	0
chr12.fa	92534054	92539673	BTG1	16.8755946	5244
chr12.fa	92813870	92821924	CLLU1OS	0	0
chr12.fa	92815307	92824778	CLLU1	0	0
chr12.fa	93096619	93102325	C12orf74	0	0
chr12.fa	93130265	93165868	PLEKHG7	0.350310422	76

chr12.fa	93166285	93323107	EEA1	30.93982006	16441
chr12.fa	93771701	93772054	NUDT4	0	0
chr12.fa	93802088	93836026	UBE2N	35.88121513	6080
chr12.fa	93861270	93896432	MRPL42	24.5970327	3335
chr12.fa	93963598	93969978	SOCS2	34.18282707	5016
chr12.fa	94071151	94244531	CRADD	20.23291185	1596
chr12.fa	94542499	94701451	PLXNC1	0.150756979	76
chr12.fa	94702056	94853764	CCDC41	6.03359248	1292
chr12.fa	94853779	94856344	LOC144486	0	0
chr12.fa	94960900	95044324	TMCC3	4.685364237	1824
chr12.fa	95228174	95228229	MIR492	0	0
chr12.fa	95228290	95228804	KRT19P2	0	0
chr12.fa	95365110	95397511	NDUFA12	47.77158821	1900
chr12.fa	95414058	95467404	NR2C1	1.788450669	532
chr12.fa	95470525	95611240	FGD6	3.820281235	2356
chr12.fa	95611522	95696566	VEZT	8.279735914	2508
chr12.fa	95702196	95702289	MIR331	0	0
chr12.fa	95703699	95703760	MIR3685	0	0
chr12.fa	95867822	95909613	METAP2	20.54511886	4780
chr12.fa	95910888	95945263	USP44	0.323201275	76
chr12.fa	96051583	96184536	NTN4	6.332546129	1520
chr12.fa	96252709	96260238	SNRPF	18.49822766	974
chr12.fa	96260826	96336428	CCDC38	0	0
chr12.fa	96337071	96362370	AMDHD1	1.071714944	152
chr12.fa	96367142	96390071	HAL	0	0
chr12.fa	96394611	96429365	LTA4H	59.72340869	8169
chr12.fa	96588207	96661606	ELK3	3.724947402	532
chr12.fa	96672039	96794366	CDK17	6.314473364	1976
chr12.fa	97301001	97347469	NEDD1	7.262088658	1976
chr12.fa	97858799	97927544	RMST	0	0
chr12.fa	97885687	97885756	MIR1251	0	0
chr12.fa	97957590	97957689	MIR135A2	0	0
chr12.fa	98389161	98389226	MIR4303	0	0
chr12.fa	98847619	98850923	LOC121456	0	0
chr12.fa	98906751	98910004	LOC100128191	0	0
chr12.fa	98909676	98944157	TMPO	4.796662346	2074
chr12.fa	98987403	98995778	SLC25A3	202.2872499	27360
chr12.fa	98993413	98993662	SNORA53	0	0
chr12.fa	99007182	99038589	IKBIP	0	0
chr12.fa	99039078	99129070	APAF1	7.992830775	3724
chr12.fa	99129212	100378432	ANKS1B	1.086173156	454
chr12.fa	100041535	100043826	FAM71C	0	0
chr12.fa	100430863	100536642	UHRF1BP1L	12.84190414	4636

chr12.fa	100550175	100567121	GOLGA2B	1.275485366	380
chr12.fa	100583662	100583727	MIR1827	0	0
chr12.fa	100594574	100618201	ACTR6	7.749149665	903
chr12.fa	100646042	100660857	DEPDC4	0.991893567	76
chr12.fa	100661549	100733914	SCYL2	21.48640869	5427
chr12.fa	100750857	100815837	SLC17A8	0	0
chr12.fa	100867679	100957643	NR1H4	1.068250998	152
chr12.fa	100967489	101018685	GAS2L3	0	0
chr12.fa	101188374	101522419	ANO4	0	0
chr12.fa	101549994	101604016	SLC5A8	0.696705077	152
chr12.fa	101673905	101780397	UTP20	5.843677845	3496
chr12.fa	101786903	101801572	ARL1	47.78122702	10184
chr12.fa	101871335	101880775	SPIC	0	0
chr12.fa	101988747	102079658	MYBPC1	12.62232005	3344
chr12.fa	102091417	102122846	CHPT1	14.62508358	1444
chr12.fa	102122426	102133250	SYCP3	0	0
chr12.fa	102139275	102224645	GNPTAB	10.30704829	3853
chr12.fa	102271105	102317401	DRAM1	2.584555952	608
chr12.fa	102406717	102455899	CCDC53	13.79042307	836
chr12.fa	102467973	102512361	NUP37	15.19814083	1216
chr12.fa	102513956	102591298	C12orf48	0	0
chr12.fa	102591300	102591614	PMCH	0	0
chr12.fa	102789645	102874378	IGF1	0.14654	76
chr12.fa	103203061	103218177	LOC283432	0	0
chr12.fa	103232104	103311381	PAH	11.10029205	1976
chr12.fa	103351452	103354294	ASCL1	0	0
chr12.fa	103695733	103889749	C12orf42	0	0
chr12.fa	103981069	104160502	STAB2	0	0
chr12.fa	104166081	104234975	NT5DC3	5.69939694	2730
chr12.fa	104237527	104323989	GNN	0.760411573	228
chr12.fa	104324189	104341703	HSP90B1	31.92358088	5615
chr12.fa	104343981	104350993	C12orf73	0.676072004	76
chr12.fa	104359593	104382656	TDG	4.939738399	1064
chr12.fa	104382765	104443915	GLT8D2	0	0
chr12.fa	104458236	104500304	HCFC2	3.792117844	1444
chr12.fa	104510858	104532040	NFYB	5.259475727	1216
chr12.fa	104609559	104744062	TXNRD1	14.87207803	4180
chr12.fa	104697549	104698982	EID3	0.798213772	76
chr12.fa	104850692	105155792	CHST11	0	0
chr12.fa	104985411	104985494	MIR3922	0	0
chr12.fa	105197275	105322472	SLC41A2	0.968850792	228
chr12.fa	105380098	105388505	C12orf45	9.200995092	380
chr12.fa	105413562	105478341	ALDH1L2	0.151811223	76

chr12.fa	105501492	105562906	KIAA1033	13.9944947	5395
chr12.fa	105567075	105630008	APPL2	29.477884	6322
chr12.fa	105724414	105765296	C12orf75	14.69677222	1333
chr12.fa	106457125	106533811	NUAK1	0.503477102	228
chr12.fa	106631659	106641713	CKAP4	7.540258627	1520
chr12.fa	106696581	106740792	TCP11L2	12.38526562	1824
chr12.fa	106751436	106903976	POLR3B	8.038765719	2356
chr12.fa	106977033	107156581	RFX4	0.259795992	76
chr12.fa	107168399	107283094	RIC8B	4.359150835	1444
chr12.fa	107349544	107367813	C12orf23	15.62842324	3496
chr12.fa	107371069	107380929	MTERFD3	10.37602601	1216
chr12.fa	107385143	107487598	CRY1	10.1915332	2204
chr12.fa	107712197	108053419	BTBD11	2.51738551	988
chr12.fa	108079590	108106257	PWP1	25.8948078	3186
chr12.fa	108126643	108154914	PRDM4	18.58934451	5168
chr12.fa	108168162	108170421	ASCL4	0	0
chr12.fa	108296927	108297548	LOC728739	0	0
chr12.fa	108523511	108644313	WSCD2	4.691990917	1444
chr12.fa	108681821	108733094	CMKLR1	0	0
chr12.fa	108909051	108913380	FICD	2.77311513	304
chr12.fa	108915991	108955165	SART3	18.8584781	5472
chr12.fa	108956294	108963160	ISCU	77.08787157	6004
chr12.fa	108983622	108991894	TMEM119	0	0
chr12.fa	109015680	109027670	SELPLG	0	0
chr12.fa	109038887	109125295	CORO1C	9.305817127	2356
chr12.fa	109176466	109251359	SSH1	12.20453797	7448
chr12.fa	109273857	109294710	DAO	6.45453729	684
chr12.fa	109304658	109458845	SVOP	0	0
chr12.fa	109490380	109525831	USP30	3.659885449	912
chr12.fa	109525993	109531293	ALKBH2	8.606099923	684
chr12.fa	109535399	109548798	UNG	17.41581966	2736
chr12.fa	109577202	109706030	ACACB	5.940216529	3648
chr12.fa	109715783	109747025	FOXN4	0	0
chr12.fa	109826524	109886176	MYO1H	0.268832374	76
chr12.fa	109886460	109915155	KCTD10	19.52189917	5146
chr12.fa	109915439	109974507	UBE3B	18.7048596	6688
chr12.fa	109991521	110011358	MMAB	14.79858213	4066
chr12.fa	110011500	110035071	MVK	8.122502862	1118
chr12.fa	110152187	110208312	C12orf34	1.822638315	380
chr12.fa	110171130	110211289	MGC14436	0	0
chr12.fa	110220892	110271212	TRPV4	4.593946169	988
chr12.fa	110288748	110318293	GLTP	63.34293163	9909
chr12.fa	110338079	110355874	TCHP	9.682483664	2128

chr12.fa	110367607	110434194	GIT2	11.60316673	4940
chr12.fa	110437235	110477235	ANKRD13A	22.52544205	5845
chr12.fa	110478983	110505500	C12orf76	3.529159118	456
chr12.fa	110562140	110656600	IFT81	7.965571022	1748
chr12.fa	110719032	110788897	ATP2A2	19.90052359	5740
chr12.fa	110810705	110841535	ANAPC7	12.56433659	3040
chr12.fa	110872706	110888158	ARPC3	107.3666803	6302
chr12.fa	110890291	110906089	GPN3	9.828270632	988
chr12.fa	110906232	110928192	C12orf24	0	0
chr12.fa	110929330	110939916	VPS29	39.80601719	2910
chr12.fa	110940005	110969891	RAD9B	0	0
chr12.fa	110972237	111021064	PPTC7	7.329409706	1824
chr12.fa	111051832	111085698	TCTN1	5.289898215	752
chr12.fa	111086491	111127617	HVCN1	0	0
chr12.fa	111157615	111180757	PPP1CC	46.55875509	7676
chr12.fa	111284811	111345338	CCDC63	0	0
chr12.fa	111348624	111358404	MYL2	0	0
chr12.fa	111374406	111375250	LOC100131138	0	0
chr12.fa	111471828	111788358	CUX2	14.13425742	6423
chr12.fa	111798455	111806925	FAM109A	2.109242242	456
chr12.fa	111843752	111889427	SH2B3	1.05861219	380
chr12.fa	111890018	112037480	ATXN2	27.42436612	8562
chr12.fa	112079950	112123790	BRAP	10.8035975	2888
chr12.fa	112123857	112194911	ACAD10	5.573941832	1520
chr12.fa	112204346	112247784	ALDH2	40.06129499	6384
chr12.fa	112277573	112280706	C12orf47	8.052470899	1064
chr12.fa	112303054	112331228	MAPKAPK5	24.54130834	2850
chr12.fa	112336867	112339706	ADAM1	0.806045304	152
chr12.fa	112369087	112451023	TMEM116	7.140398709	760
chr12.fa	112451152	112461024	ERP29	55.98626217	5472
chr12.fa	112464493	112546635	NAA25	10.42527429	4028
chr12.fa	112563349	112591408	TRAFD1	28.7569313	6070
chr12.fa	112597992	112819896	C12orf51	12.16553092	12388
chr12.fa	112842994	112847443	RPL6	327.815402	24596
chr12.fa	112856536	112947717	PTPN11	32.7915254	13680
chr12.fa	113229549	113336684	RPH3A	0	0
chr12.fa	113344739	113357712	OAS1	21.33670595	2356
chr12.fa	113376249	113411054	OAS3	7.081511617	3116
chr12.fa	113416274	113449528	OAS2	12.98467898	4028
chr12.fa	113495662	113535833	DTX1	0.348051326	76
chr12.fa	113536624	113574044	RASAL1	0.892643968	228
chr12.fa	113587663	113597081	CCDC42B	0	0
chr12.fa	113594978	113623284	DDX54	13.68273951	3525

chr12.fa	113623555	113630119	C12orf52	13.54177195	1596
chr12.fa	113633246	113658880	IQCD	0.847913875	76
chr12.fa	113659260	113736389	TPCN1	8.075061854	2868
chr12.fa	113736571	113772925	SLC24A6	3.076888183	608
chr12.fa	113796371	113827458	PLBD2	2.8291407	532
chr12.fa	113830251	113841692	SDS	0	0
chr12.fa	113860216	113876081	SDSL	12.28993178	1140
chr12.fa	113900694	113909877	LHX5	0	0
chr12.fa	114254543	114404176	RBM19	5.676354166	2128
chr12.fa	114791735	114846247	TBX5	0	0
chr12.fa	115108059	115121969	TBX3	86.39007415	27436
chr12.fa	116396381	116714991	MED13L	24.53257317	15276
chr12.fa	116586365	116586459	MIR620	0	0
chr12.fa	116971227	116974318	NCRNA00173	0	0
chr12.fa	116997186	117014425	MAP1LC3B2	1.375789209	76
chr12.fa	117153596	117175843	C12orf49	12.37412075	2280
chr12.fa	117176096	117291436	RNFT2	0	0
chr12.fa	117299027	117319232	HRK	0	0
chr12.fa	117348761	117468953	FBXW8	4.424062181	1368
chr12.fa	117476728	117537251	TESC	25.7317011	1741
chr12.fa	117581585	117628300	FBXO21	24.45380604	6839
chr12.fa	117648777	117799607	NOS1	0.122442981	76
chr12.fa	117890817	118406028	KSR2	14.39917403	16261
chr12.fa	118454508	118470039	RFC5	5.443366108	892
chr12.fa	118470492	118498951	WSB2	80.71477423	15124
chr12.fa	118501398	118541810	VSIG10	5.719578194	1900
chr12.fa	118573870	118583390	PEBP1	471.3545701	46664
chr12.fa	118587606	118810750	TAOK3	39.42423003	11476
chr12.fa	118814358	118855840	SUDS3	22.79156351	7155
chr12.fa	119419396	119600857	SRRM4	0	0
chr12.fa	119616595	119632551	HSPB8	75.42352056	10026
chr12.fa	119721630	119741185	LOC144742	0	0
chr12.fa	119772517	119978852	CCDC60	0.467180966	76
chr12.fa	120031264	120079363	TMEM233	0	0
chr12.fa	120105761	120119429	PRKAB1	25.74299658	4104
chr12.fa	120123598	120315092	CIT	1.336932765	760
chr12.fa	120151454	120151529	MIR1178	0	0
chr12.fa	120427648	120532299	CCDC64	26.3768988	5396
chr12.fa	120532899	120554643	RAB35	15.93505781	3116
chr12.fa	120565014	120632513	GCN1L1	18.73196875	10781
chr12.fa	120634503	120639014	RPLP0	120.7352549	9652
chr12.fa	120648250	120703563	PXN	26.30957775	7068
chr12.fa	120740124	120751045	SIRT4	0.945958623	76

chr12.fa	120759914	120765592	PLA2G1B	0	0
chr12.fa	120779133	120806958	MSI1	4.26803398	836
chr12.fa	120875904	120878529	COX6A1	65.10623103	2356
chr12.fa	120881764	120884215	TRIAP1	24.20922129	1900
chr12.fa	120884241	120901556	GATC	10.40283394	2660
chr12.fa	120901753	120907558	SRSF9	116.246432	5156
chr12.fa	120907660	120936298	DYNLL1	105.8530862	6916
chr12.fa	120941082	120966964	COQ5	12.13631329	1216
chr12.fa	120972132	121015397	RNF10	93.24914015	23819
chr12.fa	121016848	121019201	POP5	20.00564684	1064
chr12.fa	121078422	121105127	CABP1	0.602726701	76
chr12.fa	121124949	121139667	MLEC	13.684848	5769
chr12.fa	121147831	121161443	UNC119B	25.21903701	8056
chr12.fa	121163571	121177811	ACADS	4.776631698	608
chr12.fa	121201035	121342151	SPPL3	6.390680189	1444
chr12.fa	121407641	121410095	NCRNA00262	0	0
chr12.fa	121416549	121440314	HNF1A	3.181409005	684
chr12.fa	121440848	121454300	C12orf43	8.346153324	1064
chr12.fa	121458095	121476780	OASL	0.62893221	76
chr12.fa	121570622	121624354	P2RX7	0	0
chr12.fa	121647664	121671909	P2RX4	5.079651719	684
chr12.fa	121675495	121736111	CAMKK2	14.76710539	6139
chr12.fa	121746048	121792012	ANAPC5	32.67917305	5928
chr12.fa	121837902	121862155	RNF34	12.13556025	1672
chr12.fa	121866900	122018920	KDM2B	2.261204071	836
chr12.fa	122064455	122079946	ORAI1	0	0
chr12.fa	122089293	122107560	MORN3	0.98842962	76
chr12.fa	122150658	122219974	TMEM120B	1.254099261	446
chr12.fa	122231058	122231594	RHOF	0	0
chr12.fa	122233173	122241390	LOC338799	1.505009477	152
chr12.fa	122242638	122270562	SETD1B	9.777064466	5320
chr12.fa	122277433	122326517	HPD	82.35261852	10619
chr12.fa	122326646	122355771	PSMD9	15.14422375	2356
chr12.fa	122356463	122441832	WDR66	1.865410525	532
chr12.fa	122459861	122499950	BCL7A	8.7780924	2128
chr12.fa	122516760	122628967	MLXIP	12.58045148	4484
chr12.fa	122652266	122688018	LRRC43	0.547905982	76
chr12.fa	122656577	122658746	IL31	0	0
chr12.fa	122688228	122692084	B3GNT4	0.761013998	76
chr12.fa	122692209	122712068	DIABLO	13.16209329	2052
chr12.fa	122716094	122751068	VPS33A	10.95842085	1894
chr12.fa	122755981	122907116	CLIP1	30.91527122	12070
chr12.fa	122957435	122985518	ZCCHC8	11.80031047	2244

chr12.fa	122989190	123011560	RSRC2	24.52398861	3952
chr12.fa	123011809	123110947	KNTC1	1.314643022	608
chr12.fa	123185840	123187904	GPR109A	0	0
chr12.fa	123199303	123201439	GPR109B	0	0
chr12.fa	123212153	123215129	GPR81	0.768996136	152
chr12.fa	123237371	123255953	DENR	16.41865487	3337
chr12.fa	123259073	123311927	CCDC62	0	0
chr12.fa	123320039	123347507	HIP1R	27.75765802	8244
chr12.fa	123349875	123380712	VPS37B	17.90408552	3268
chr12.fa	123413539	123459752	ABCB9	0	0
chr12.fa	123460358	123464588	OGFOD2	4.539577269	532
chr12.fa	123464880	123467460	ARL6IP4	84.34860477	7516
chr12.fa	123468027	123594975	PITPNM2	1.699291697	760
chr12.fa	123495214	123495275	MIR4304	0	0
chr12.fa	123640946	123706441	MPHOSPH9	3.607775644	865
chr12.fa	123717844	123742506	C12orf65	6.587673323	912
chr12.fa	123745540	123756687	CDK2AP1	75.21914771	8056
chr12.fa	123773656	123834988	SBNO1	19.38770889	14136
chr12.fa	123868704	123893900	SETD8	23.69956932	4329
chr12.fa	123899936	123921264	RILPL2	12.76223337	1216
chr12.fa	123942651	123950937	SNRNP35	15.58806073	1064
chr12.fa	123955909	124018265	RILPL1	3.925856302	684
chr12.fa	124020956	124021081	MIR3908	0	0
chr12.fa	124069076	124082688	TMED2	20.4586708	2888
chr12.fa	124086672	124105482	DDX55	6.98467172	1216
chr12.fa	124105570	124118323	EIF2B1	26.93203389	3344
chr12.fa	124118381	124145334	GTF2H3	8.806255791	880
chr12.fa	124155660	124192948	TCTN2	3.540002777	684
chr12.fa	124196865	124246301	ATP6V0A2	2.099904647	912
chr12.fa	124247042	124420267	DNAH10	0.167323679	152
chr12.fa	124420955	124457163	CCDC92	24.87987146	2962
chr12.fa	124457670	124499969	ZNF664	47.28121386	16036
chr12.fa	124773710	124800568	FAM101A	2.664377329	380
chr12.fa	124808961	125052010	NCOR2	17.24488143	10161
chr12.fa	125262174	125348519	SCARB1	2.922064832	532
chr12.fa	125396192	125399587	UBC	193.5734665	33212
chr12.fa	125431370	125473667	DHX37	6.036152789	1824
chr12.fa	125478194	125510349	BRI3BP	0.899873073	76
chr12.fa	125549925	125627871	AACS	13.44161871	2964
chr12.fa	125811162	126143589	TMEM132B	0	0
chr12.fa	126443234	126467920	LOC400084	0	0
chr12.fa	126927027	126957331	LOC100128554	0	0
chr12.fa	127215247	127256808	LOC387895	0	0

chr12.fa	127354040	127359236	LOC440117	0	0
chr12.fa	128366162	128383184	FLJ37505	0	0
chr12.fa	128751948	129192460	TMEM132C	0	0
chr12.fa	128778637	128778723	MIR3612	0	0
chr12.fa	129277739	129308541	SLC15A4	3.283218912	608
chr12.fa	129338081	129469509	GLT1D1	1.682574389	304
chr12.fa	129556271	130388212	TMEM132D	0	0
chr12.fa	130517999	130526887	LOC100190940	0.324406126	76
chr12.fa	130636138	130646223	FLJ31485	0	0
chr12.fa	130647004	130650285	FZD10	0	0
chr12.fa	130822433	130856877	PIWIL1	0	0
chr12.fa	130880681	131002410	RIMBP2	0.181028859	76
chr12.fa	131274147	131323811	STX2	2.650370937	608
chr12.fa	131356617	131360826	RAN	92.40288295	6571
chr12.fa	131438452	131626008	GPR133	0.212656197	76
chr12.fa	131649556	131697476	LOC116437	0	0
chr12.fa	132195635	132284282	SFSWAP	5.996392706	1292
chr12.fa	132312941	132336316	MMP17	0	0
chr12.fa	132379279	132407707	ULK1	10.52979511	3651
chr12.fa	132413745	132428406	PUS1	3.848294021	532
chr12.fa	132434508	132565005	EP400	10.25448667	8351
chr12.fa	132515769	132515905	SNORA49	0	0
chr12.fa	132568828	132610885	EP400NL	2.231233403	228
chr12.fa	132621140	132628880	DDX51	1.698538665	532
chr12.fa	132628993	132636986	NOC4L	0.701373875	76
chr12.fa	132680917	132905905	GALNT9	0.783906167	152
chr12.fa	132851977	132857486	LOC100130238	0	0
chr12.fa	133067157	133161773	FBRSL1	6.707254783	2025
chr12.fa	133179736	133187037	LOC645277	0	0
chr12.fa	133195403	133198972	P2RX2	0.625468263	76
chr12.fa	133200348	133263945	POLE	2.627930587	1368
chr12.fa	133264192	133281577	PXMP2	43.05730755	2736
chr12.fa	133287393	133299323	PGAM5	11.25616965	2432
chr12.fa	133302254	133338451	ANKLE2	3.844528861	1138
chr12.fa	133345495	133405426	GOLGA3	30.78906308	19597
chr12.fa	133416938	133464204	CHFR	8.229583992	1824
chr12.fa	133498019	133532868	ZNF605	4.735817372	1976
chr12.fa	133563056	133589153	ZNF26	0.748363063	152
chr12.fa	133613878	133639885	ZNF84	3.741062284	1748
chr12.fa	133657037	133684258	ZNF140	5.975157208	1216
chr12.fa	133707214	133736049	ZNF10	1.039786393	304
chr12.fa	133757995	133783697	ZNF268	6.631951597	2584
chr12.fa	133794898	133812422	LOC647589	0	0

chr13.fa	19408543	19446109	LOC284232	0	0
chr13.fa	19582399	19586774	LOC348021	0	0
chr13.fa	19620293	19692423	PHF2P1	0	0
chr13.fa	19747918	19755936	TUBA3C	0	0
chr13.fa	19836940	19919113	LOC100101938	0	0
chr13.fa	19919189	19920889	LOC100287114	0	0
chr13.fa	19997019	20110903	TPTE2	0	0
chr13.fa	20207788	20247599	MPHOSPH8	21.72286069	6156
chr13.fa	20248896	20357083	PSPC1	22.00253672	3496
chr13.fa	20397624	20437776	ZMYM5	4.286859776	988
chr13.fa	20532810	20665968	ZMYM2	26.8662189	18488
chr13.fa	20712395	20735183	GJA3	0	0
chr13.fa	20761604	20767114	GJB2	0.490826167	76
chr13.fa	20796101	20806534	GJB6	1.825499837	304
chr13.fa	20977806	21100012	CRYL1	78.54408459	7828
chr13.fa	21141208	21265576	IFT88	8.330942081	1748
chr13.fa	21277482	21297237	IL17D	1.845078665	228
chr13.fa	21303073	21348057	N6AMT2	14.43893411	836
chr13.fa	21351468	21476913	XPO4	6.484357352	4256
chr13.fa	21547176	21635722	LATS2	7.848248658	2888
chr13.fa	21714653	21723224	SAP18	55.71321282	8512
chr13.fa	21727734	21746820	SKA3	0	0
chr13.fa	21750372	21753220	MRP63	12.261919	1976
chr13.fa	21946710	22033508	ZDHHC20	9.927670838	3520
chr13.fa	22066828	22178355	EFHA1	17.14096303	2200
chr13.fa	22245215	22278640	FGF9	1.516003742	456
chr13.fa	23471169	23472320	BASP1P1	0	0
chr13.fa	23755060	23899304	SGCG	0	0
chr13.fa	23902965	24007841	SACS	0.439168181	456
chr13.fa	24144723	24250232	TNFRSF19	3.996490691	1140
chr13.fa	24304328	24463587	MIPEP	14.60806506	2356
chr13.fa	24463028	24466242	PCOTH	3.704163723	76
chr13.fa	24465428	24471125	C1QTNF9B	0	0
chr13.fa	24734861	24881212	SPATA13	13.53439224	7600
chr13.fa	24736555	24736643	MIR2276	0	0
chr13.fa	24883716	24896669	C1QTNF9	0	0
chr13.fa	24995069	25086948	PARP4	42.02053328	15259
chr13.fa	25154346	25171812	LOC374491	0	0
chr13.fa	25254549	25285923	ATP12A	1.226086475	304
chr13.fa	25338301	25454058	RNF17	0.224704707	76
chr13.fa	25456412	25497085	CENPJ	1.770076692	608
chr13.fa	25502893	25542607	TPTE2P1	0	0
chr13.fa	25670276	25672704	PABPC3	0	0

chr13.fa	25742672	25745857	FAM123A	0	0
chr13.fa	25820341	25861704	MTMR6	8.65685427	2997
chr13.fa	25875666	25916561	NUPL1	10.78040412	3103
chr13.fa	25946209	26595420	ATP8A2	0	0
chr13.fa	26618735	26625198	SHISA2	0	0
chr13.fa	26786905	26796508	RNF6	22.69171148	5852
chr13.fa	26828756	26978569	CDK8	16.82303298	1976
chr13.fa	27131840	27263082	WASF3	6.866746931	2204
chr13.fa	27329339	27334922	GPR12	0.236150791	76
chr13.fa	27640287	27746033	USP12	6.100461709	1824
chr13.fa	27825692	27825722	RPL21	0	0
chr13.fa	27829201	27829272	SNORD102	0	0
chr13.fa	27829538	27829663	SNORA27	0	0
chr13.fa	27844464	27847827	RASL11A	5.192606498	532
chr13.fa	27998681	28009775	GTF3A	111.4464563	9864
chr13.fa	28009847	28024739	MTIF3	22.67529539	1748
chr13.fa	28120050	28194720	LNX2	7.862556263	2508
chr13.fa	28196003	28241548	POLR1D	20.44752593	3420
chr13.fa	28366780	28368089	GSX1	0	0
chr13.fa	28494168	28500451	PDX1	0	0
chr13.fa	28519343	28519710	ATP5EP2	3.110322797	76
chr13.fa	28536278	28543317	CDX2	0	0
chr13.fa	28552243	28562774	PRHOXNB	0	0
chr13.fa	28577411	28674729	FLT3	0	0
chr13.fa	28710980	28713311	LOC100288730	2.08107885	228
chr13.fa	28712643	28869475	PAN3	16.39485906	5542
chr13.fa	28874483	29069265	FLT1	0	0
chr13.fa	29233141	29253093	POMP	36.51255704	3542
chr13.fa	29274218	29293150	SLC46A3	0	0
chr13.fa	29598748	30080084	MTUS2	3.499339056	1672
chr13.fa	30083551	30169825	SLC7A1	3.117551903	1520
chr13.fa	30338545	30424820	UBL3	27.65389023	7980
chr13.fa	30510668	30524625	LOC440131	0	0
chr13.fa	30776767	30881584	KATNAL1	1.806975253	912
chr13.fa	30914407	30948036	LOC100188949	0	0
chr13.fa	31032879	31040081	HMGB1	13.36375522	3040
chr13.fa	31191830	31233686	USPL1	4.691087279	1216
chr13.fa	31309669	31338556	ALOX5AP	0	0
chr13.fa	31480312	31499709	C13orf33	0	0
chr13.fa	31506834	31549153	C13orf26	0	0
chr13.fa	31710763	31736117	HSPH1	60.86681226	14820
chr13.fa	31774112	31906411	B3GALTL	1.086775581	304
chr13.fa	32313679	32377009	RXFP2	0	0

chr13.fa	32420920	32533721	EEF1DP3	0	0
chr13.fa	32605437	32870776	FRY	9.719081013	6916
chr13.fa	32877908	32886091	ZAR1L	0	0
chr13.fa	32889617	32973809	BRCA2	0.201059507	152
chr13.fa	32974860	33002315	N4BP2L1	0.367328942	76
chr13.fa	33006930	33112936	N4BP2L2	15.20536994	4940
chr13.fa	33078643	33083532	CG030	0	0
chr13.fa	33160564	33352158	PDS5B	8.548718895	4256
chr13.fa	33590571	33640282	KL	8.002620189	2660
chr13.fa	33677272	33859901	STARD13	4.85916399	2052
chr13.fa	34392206	34540695	RFC3	3.196168429	608
chr13.fa	35516424	36246873	NBEA	4.22029176	3116
chr13.fa	36047926	36050832	MAB21L1	0	0
chr13.fa	36168361	36515382	MIR548F5	0	0
chr13.fa	36342790	36705514	DCLK1	0	0
chr13.fa	36788616	36788752	SOHLH2	0	0
chr13.fa	36801179	36869994	C13orf38	5.402702387	339
chr13.fa	36875775	36944317	SPG20	16.12421941	5776
chr13.fa	37005967	37017019	CCNA1	0	0
chr13.fa	37248049	37271975	C13orf36	0	0
chr13.fa	37393339	37403740	RFXAP	2.431088059	456
chr13.fa	37418968	37494409	SMAD9	20.0592627	7448
chr13.fa	37523908	37573504	ALG5	9.602361074	760
chr13.fa	37574678	37583450	EXOSC8	10.31171709	760
chr13.fa	37583752	37633850	FAM48A	21.95509572	3971
chr13.fa	37677397	37679801	CSNK1A1L	0	0
chr13.fa	38136719	38172981	POSTN	0	0
chr13.fa	38210773	38443939	TRPC4	0	0
chr13.fa	38923942	38937143	UFM1	23.87924273	4124
chr13.fa	39261173	39461267	FREM2	5.311585532	5700
chr13.fa	39540062	39564996	STOML3	0	0
chr13.fa	39584002	39612252	C13orf23	15.81547635	5469
chr13.fa	39612455	39624246	NHLRC3	1.612542426	380
chr13.fa	39917029	40177356	LHFP	0	0
chr13.fa	40229764	40365802	COG6	6.675627445	3105
chr13.fa	40238171	40238272	MIR4305	0	0
chr13.fa	40768646	40794639	FLJ42392	0	0
chr13.fa	40921271	41055143	LOC646982	0.217776814	76
chr13.fa	41129801	41240734	FOXO1	4.189116241	1596
chr13.fa	41301964	41302011	MIR320D1	0	0
chr13.fa	41303432	41345347	MRPS31	18.80606708	1672
chr13.fa	41363547	41386596	SLC25A15	3.122220701	760
chr13.fa	41371127	41495886	SUGT1P3	0.874571203	228

chr13.fa	41506055	41593508	ELF1	39.49034623	9804
chr13.fa	41635697	41658139	WBP4	6.868704814	1064
chr13.fa	41701709	41706936	KBTBD6	2.627328162	912
chr13.fa	41765711	41768702	KBTBD7	9.181416264	1824
chr13.fa	41790516	41837713	MTRF1	6.317937311	912
chr13.fa	41885341	41951166	NAA16	3.98820734	1216
chr13.fa	42005476	42017723	OR7E37P	0	0
chr13.fa	42031542	42045013	C13orf15	3.099177926	228
chr13.fa	42140961	42535221	KIAA0564	11.08116504	5455
chr13.fa	42622889	42803888	DGKH	3.233368203	912
chr13.fa	42846289	42897402	AKAP11	11.19336679	7372
chr13.fa	43136872	43182149	TNFSF11	0	0
chr13.fa	43355686	43365685	C13orf30	0	0
chr13.fa	43462122	43566377	EPSTI1	7.276546869	760
chr13.fa	43597362	43683306	DNAJC15	5.772591637	1064
chr13.fa	43787666	44361106	ENOX1	2.940589416	608
chr13.fa	44410489	44449063	CCDC122	1.762696979	228
chr13.fa	44453420	44468068	C13orf31	6.628035831	1976
chr13.fa	44596471	44604599	NCRNA00284	0	0
chr13.fa	44947978	44971850	SERP2	4.379934514	228
chr13.fa	45007655	45150701	TSC22D1	61.83611487	21186
chr13.fa	45513384	45563613	NUFIP1	1.641308243	380
chr13.fa	45563687	45602404	KIAA1704	9.867277683	912
chr13.fa	45694631	45858239	GTF2F2	10.23038965	987
chr13.fa	45766988	45775175	KCTD4	0	0
chr13.fa	45911304	45915297	TPT1	4719.211664	259765
chr13.fa	45911615	45911744	SNORA31	0	0
chr13.fa	45915480	45965618	LOC100190939	1.500190073	152
chr13.fa	45967454	45992516	SLC25A30	21.5315906	5244
chr13.fa	46039071	46110760	COG3	29.10980203	8663
chr13.fa	46115432	46189874	FAM194B	0	0
chr13.fa	46276446	46288693	SPERT	0	0
chr13.fa	46354416	46425846	SIAH3	0	0
chr13.fa	46536314	46626894	ZC3H13	25.7175441	10944
chr13.fa	46627322	46679211	CPB2	0	0
chr13.fa	46700058	46756459	LCP1	126.5411305	31844
chr13.fa	46916137	46961635	C13orf18	0.287808777	76
chr13.fa	47127296	47327175	LRCH1	8.598117785	4614
chr13.fa	47345391	47371367	ESD	96.55479942	7642
chr13.fa	47407513	47471169	HTR2A	0	0
chr13.fa	48516791	48575462	SUCLA2	23.27305208	3344
chr13.fa	48611703	48621282	NUDT15	17.54835327	2356
chr13.fa	48650092	48669240	MED4	19.64133002	2637

chr13.fa	48807274	48836232	ITM2B	21.12932098	2660
chr13.fa	48877883	49056026	RB1	11.1249915	3525
chr13.fa	48985182	49018840	LPAR6	1.875199939	380
chr13.fa	49063099	49107316	RCBTB2	5.407672398	1140
chr13.fa	49280951	49283498	CYSLTR2	0	0
chr13.fa	49550048	49783915	FNDC3A	21.88506375	9271
chr13.fa	49794474	49796513	MLNR	0	0
chr13.fa	49822047	49867622	CDADC1	2.692390115	608
chr13.fa	49882786	50018221	CAB39L	64.25876898	17024
chr13.fa	50018429	50069139	SETDB2	3.49587511	1444
chr13.fa	50069801	50103117	PHF11	11.94880835	1509
chr13.fa	50106082	50159719	RCBTB1	11.35240712	3040
chr13.fa	50202435	50208008	ARL11	0	0
chr13.fa	50234810	50265623	EBPL	8.192534825	532
chr13.fa	50273443	50367057	KPNA3	10.96052934	3256
chr13.fa	50464545	50467516	CTAGE10P	0.385100494	76
chr13.fa	50486842	50510625	C13orf1	6.434356036	1368
chr13.fa	50556688	50699677	DLEU2	0	0
chr13.fa	50570551	50570637	MIR3613	0	0
chr13.fa	50571143	50592603	TRIM13	7.694931371	3062
chr13.fa	50592959	50595058	KCNRG	0	0
chr13.fa	50623109	50623197	MIR16-1	0	0
chr13.fa	50623255	50623337	MIR15A	0	0
chr13.fa	50656414	50679433	DLEU1	0	0
chr13.fa	50746154	50747751	ST13P4	0	0
chr13.fa	51286759	51417885	DLEU7	0	0
chr13.fa	51483892	51544596	RNASEH2B	13.54011528	1870
chr13.fa	51568647	51640293	GUCY1B2	0	0
chr13.fa	51796507	51855616	FAM124A	1.087980432	152
chr13.fa	51915168	51935500	SERPINE3	0	0
chr13.fa	51935701	52027275	INTS6	9.196778114	5320
chr13.fa	52158484	52336171	WDFY2	6.310557599	1596
chr13.fa	52342131	52378293	DHRS12	8.108647075	1444
chr13.fa	52387483	52419286	NCRNA00282	0	0
chr13.fa	52436117	52440372	CCDC70	0	0
chr13.fa	52506806	52585630	ATP7B	3.791816631	1672
chr13.fa	52586523	52598826	ALG11	5.769127691	380
chr13.fa	52603781	52607736	UTP14C	4.050708985	1064
chr13.fa	52638900	52703214	NEK5	0	0
chr13.fa	52706779	52733996	NEK3	1.334071244	228
chr13.fa	52741847	52768602	THSD1P1	0.983007791	228
chr13.fa	52793481	52864119	TPTE2P2	0.03238037	152
chr13.fa	52951303	52980629	THSD1	0.683903536	152

chr13.fa	52986737	53024763	VPS36	19.60985329	5759
chr13.fa	53029495	53050763	CKAP2	5.821086889	1444
chr13.fa	53063128	53161225	TPTE2P3	0.876077267	152
chr13.fa	53191605	53217919	HNRNPA1L2	3.871788615	608
chr13.fa	53226831	53262433	SUGT1	20.26348494	2403
chr13.fa	53277400	53313947	LECT1	0	0
chr13.fa	53384185	53384275	MIR759	0	0
chr13.fa	53418109	53422774	PCDH8	0	0
chr13.fa	53602972	53626188	OLFM4	0.406637205	76
chr13.fa	54886107	54886183	MIR1297	0	0
chr13.fa	58205789	58303065	PCDH17	0.142925447	76
chr13.fa	60239723	60738119	DIAPH3	0.229674717	76
chr13.fa	60970591	61148013	TDRD3	13.09703133	2728
chr13.fa	61773932	61774014	MIR3169	0	0
chr13.fa	61983819	61989655	PCDH20	0	0
chr13.fa	64311568	64316701	OR7E156P	0	0
chr13.fa	66876966	67804468	PCDH9	0.367630154	152
chr13.fa	70274725	70682625	KLHL1	0	0
chr13.fa	70689273	70713885	ATXN8OS	0	0
chr13.fa	72012098	72441330	DACH1	8.52070611	2964
chr13.fa	73282495	73301938	MZT1	4.270895501	684
chr13.fa	73302042	73329539	C13orf34	1.153795417	152
chr13.fa	73330329	73356229	DIS3	11.2290605	5076
chr13.fa	73356345	73590591	PIBF1	7.253955914	1444
chr13.fa	73633142	73651676	KLF5	46.40920297	10323
chr13.fa	74260149	74708066	KLF12	0.629534635	456
chr13.fa	75126980	75131257	LOC338864	0	0
chr13.fa	75811889	75814517	CTAGE11P	0	0
chr13.fa	75858809	76056250	TBC1D4	10.25343242	4332
chr13.fa	76099350	76111991	COMMD6	23.47426219	2684
chr13.fa	76123927	76180068	UCHL3	23.26085296	1302
chr13.fa	76194570	76434006	LMO7	22.94141685	12963
chr13.fa	77454304	77460540	KCTD12	2.202166373	912
chr13.fa	77502585	77503224	BTF3P11	0	0
chr13.fa	77566059	77576652	CLN5	4.496654453	836
chr13.fa	77579389	77601331	FBXL3	27.09137543	6384
chr13.fa	77618792	77901177	MYCBP2	13.25531863	13224
chr13.fa	78109809	78219398	SCEL	1.064485838	228
chr13.fa	78272147	78272251	MIR3665	0	0
chr13.fa	78272470	78338377	SLAIN1	6.897771843	1216
chr13.fa	78469616	78549664	EDNRB	1.069154636	304
chr13.fa	79173230	79177695	POU4F1	0	0
chr13.fa	79188421	79233314	RNF219	6.477730672	1520

chr13.fa	79894100	79979923	RBM26	17.10737781	4104
chr13.fa	80055259	80130212	NDFIP2	3.419818892	1057
chr13.fa	80910112	80915086	SPRY2	7.594627527	1064
chr13.fa	84451343	84456528	SLITRK1	0	0
chr13.fa	86366922	86373483	SLITRK6	0.821256547	228
chr13.fa	88096242	88323218	LOC642345	0	0
chr13.fa	88324870	88331870	SLITRK5	0	0
chr13.fa	90883436	90883531	MIR622	0	0
chr13.fa	91543208	91578851	LOC144776	0	0
chr13.fa	92000074	92006829	MIR17HG	0	0
chr13.fa	92050935	93519487	GPC5	1.192350648	228
chr13.fa	93879078	95060273	GPC6	0.967043515	456
chr13.fa	95091841	95131936	DCT	0	0
chr13.fa	95226308	95248511	TGDS	3.610787772	456
chr13.fa	95254104	95281946	GPR180	1.751100289	456
chr13.fa	95361879	95364389	SOX21	0	0
chr13.fa	95672083	95953687	ABCC4	36.89283813	15315
chr13.fa	96085853	96230455	CLDN10	15.14015738	1334
chr13.fa	96232011	96296957	DZIP1	2.096741913	826
chr13.fa	96329393	96447243	DNAJC3	14.12597407	5244
chr13.fa	96453836	96705736	UGGT2	3.018904729	988
chr13.fa	96743093	97491812	HS6ST3	0.293531819	152
chr13.fa	97637973	97646604	OXGR1	0	0
chr13.fa	97874574	98046374	MBNL2	26.16725473	8173
chr13.fa	98086475	98120252	RAP2A	5.252849047	1520
chr13.fa	98605929	98676550	IPO5	37.14465198	14803
chr13.fa	98795434	99102023	FARP1	48.89194901	18634
chr13.fa	98828039	98829521	RNF113B	0	0
chr13.fa	98860778	98860854	MIR3170	0	0
chr13.fa	99102455	99229396	STK24	44.25146547	14506
chr13.fa	99336055	99404929	SLC15A1	0.365973484	76
chr13.fa	99445741	99738660	DOCK9	16.66082992	8902
chr13.fa	99848628	99853009	LOC100289373	2.850828018	304
chr13.fa	99853012	100038753	UBAC2	11.53057446	2280
chr13.fa	99906967	99910682	GPR18	0	0
chr13.fa	99946789	99959749	GPR183	1.282413259	152
chr13.fa	100003674	100004281	FKSG29	0	0
chr13.fa	100008385	100008482	MIR623	0	0
chr13.fa	100153728	100215276	TM9SF2	13.21345006	2096
chr13.fa	100258919	100549388	CLYBL	3.372377885	1292
chr13.fa	100295313	100295403	MIR4306	0	0
chr13.fa	100615275	100624178	ZIC5	0	0
chr13.fa	100634319	100639019	ZIC2	0	0

chr13.fa	100741269	101182417	PCCA	42.48545515	6460
chr13.fa	101182692	101241046	A2LD1	8.707608617	1520
chr13.fa	101256090	101327103	TMTC4	5.488246807	1368
chr13.fa	101706130	102068813	NALCN	0	0
chr13.fa	102104966	102368794	ITGBL1	0	0
chr13.fa	102373205	103054124	FGF14	0	0
chr13.fa	102944670	103046869	LOC283480	0	0
chr13.fa	103046928	103048055	LOC283481	1.014785735	76
chr13.fa	103249286	103331523	TPP2	16.03912681	5016
chr13.fa	103338097	103346854	C13orf39	0	0
chr13.fa	103381717	103411422	C13orf40	0	0
chr13.fa	103418463	103426149	C13orf27	1.99056442	152
chr13.fa	103436631	103451398	KDELC1	0	0
chr13.fa	103451405	103493888	BIVM	8.852341341	2280
chr13.fa	103498174	103528345	ERCC5	32.13623708	8740
chr13.fa	103532449	103548383	LOC121952	0.495043145	76
chr13.fa	103696348	103719196	SLC10A2	0.302869414	76
chr13.fa	106118216	106143383	DAOA	0	0
chr13.fa	107028911	107030142	LOC728192	0	0
chr13.fa	107142096	107187337	EFNB2	3.975255192	1140
chr13.fa	107195662	107220514	ARGLU1	10.45901012	1216
chr13.fa	107820879	108519460	FAM155A	0.296543947	76
chr13.fa	108859792	108870716	LIG4	3.829920043	1064
chr13.fa	108870763	108886603	ABHD13	1.295365407	456
chr13.fa	108921977	108959365	TNFSF13B	0	0
chr13.fa	109248500	109860355	MYO16	0.152112436	76
chr13.fa	110406184	110438914	IRS2	8.014518093	3724
chr13.fa	110801310	110959496	COL4A1	1.026081213	445
chr13.fa	110959631	111165373	COL4A2	1.827608326	760
chr13.fa	111175413	111214071	RAB20	19.72160322	1976
chr13.fa	111268008	111292342	CARKD	28.25917724	4993
chr13.fa	111293757	111358480	CARS2	15.98264942	1976
chr13.fa	111365083	111373421	ING1	5.015342798	1368
chr13.fa	111516334	111522655	C13orf29	0	0
chr13.fa	111530887	111567416	ANKRD10	11.46897645	1900
chr13.fa	111767624	111958081	ARHGEF7	14.58201016	8501
chr13.fa	111973015	111996594	C13orf16	0	0
chr13.fa	112721913	112726020	SOX1	0	0
chr13.fa	113030651	113089009	C13orf28	0	0
chr13.fa	113139328	113242481	TUBGCP3	7.650351885	1976
chr13.fa	113301358	113338811	C13orf35	0	0
chr13.fa	113344643	113541482	ATP11A	2.197497576	1292
chr13.fa	113621798	113622756	LOC100289410	5.967777496	380

chr13.fa	113622953	113752862	MCF2L	12.73647968	4636
chr13.fa	113760105	113774995	F7	0	0
chr13.fa	113777113	113803843	F10	0	0
chr13.fa	113812968	113826694	PROZ	0	0
chr13.fa	113831925	113863029	PCID2	24.61164151	2783
chr13.fa	113863086	113919392	CUL4A	36.83726438	9500
chr13.fa	113951469	113977741	LAMP1	14.41438527	2431
chr13.fa	113978505	114018463	GRTP1	7.326849398	684
chr13.fa	114076260	114107839	ADPRHL1	1.580162056	228
chr13.fa	114110134	114145023	DCUN1D2	3.023121708	608
chr13.fa	114145308	114204544	TMCO3	6.305286376	1292
chr13.fa	114239003	114295788	TFDP1	23.3680847	4180
chr13.fa	114303122	114312513	ATP4B	0	0
chr13.fa	114321597	114438637	GRK1	0	0
chr13.fa	114451484	114454062	LOC100130386	0	0
chr13.fa	114462216	114514899	FAM70B	0	0
chr13.fa	114523522	114567046	GAS6	2.749620536	532
chr13.fa	114586610	114626485	FLJ44054	0	0
chr13.fa	114747194	114898095	RASA3	1.090088921	304
chr13.fa	115000362	115038150	CDC16	39.71731003	6047
chr13.fa	115047078	115071283	UPF3A	2.911221173	456
chr13.fa	115079965	115092803	ZNF828	12.59129514	3192
chr14.fa	19377594	19378574	OR11H12	0	0
chr14.fa	19553365	19584942	POTEG	1.15906664	152
chr14.fa	19983954	20020272	POTEM	0.171691264	76
chr14.fa	20181063	20182491	OR11H2	0	0
chr14.fa	20215587	20216528	OR4Q3	0	0
chr14.fa	20248482	20249423	OR4M1	0	0
chr14.fa	20295608	20296531	OR4N2	0	0
chr14.fa	20344427	20345371	OR4K2	0	0
chr14.fa	20388766	20389737	OR4K5	0	0
chr14.fa	20403767	20404842	OR4K1	0	0
chr14.fa	20443678	20444724	OR4K15	0	0
chr14.fa	20482420	20483352	OR4K14	0	0
chr14.fa	20502003	20502917	OR4K13	0	0
chr14.fa	20528204	20529142	OR4L1	0	0
chr14.fa	20585566	20586597	OR4K17	0	0
chr14.fa	20611895	20612821	OR4N5	0	0
chr14.fa	20665495	20666532	OR11G2	0	0
chr14.fa	20691869	20692861	OR11H6	0	0
chr14.fa	20710951	20711925	OR11H4	0	0
chr14.fa	20757301	20774153	TTC5	12.8882909	1596
chr14.fa	20779529	20801457	CCNB1IP1	19.43108353	2432

chr14.fa	20794600	20794698	SNORD126	0	0
chr14.fa	20811230	20811570	RPPH1	1037.196566	23484
chr14.fa	20811773	20826063	PARP2	4.335656241	532
chr14.fa	20833826	20881579	TEP1	4.709461256	3344
chr14.fa	20896970	20903801	KLHL33	0	0
chr14.fa	20915207	20923267	OSGEP	11.97441144	1292
chr14.fa	20923290	20925926	APEX1	51.056765	5058
chr14.fa	20926012	20929637	TMEM55B	18.03059487	2052
chr14.fa	20937538	20946165	PNP	16.54742332	2660
chr14.fa	20978631	20979281	RNASE10	0	0
chr14.fa	21024252	21029090	RNASE9	0	0
chr14.fa	21051052	21055784	RNASE11	0	0
chr14.fa	21058240	21058982	RNASE12	0	0
chr14.fa	21108855	21109850	OR6S1	0	0
chr14.fa	21152336	21162345	ANG	3.386384277	152
chr14.fa	21167514	21168758	RNASE4	10.11306728	836
chr14.fa	21214099	21216539	EDDM3A	0	0
chr14.fa	21236586	21239107	EDDM3B	0	0
chr14.fa	21249210	21250626	RNASE6	0	0
chr14.fa	21269515	21271036	RNASE1	0	0
chr14.fa	21359562	21360507	RNASE3	0	0
chr14.fa	21387500	21388335	ECRP	0	0
chr14.fa	21423630	21424594	RNASE2	0	0
chr14.fa	21457965	21465194	METT11D1	8.972224013	1064
chr14.fa	21467414	21470034	SLC39A2	3.228850012	304
chr14.fa	21484922	21493935	NDRG2	133.8609014	19545
chr14.fa	21498345	21500332	TPPP2	0	0
chr14.fa	21500979	21502944	RNASE13	0	0
chr14.fa	21510385	21512392	RNASE7	1.528202858	152
chr14.fa	21526052	21526516	RNASE8	0	0
chr14.fa	21538527	21558036	ARHGEF40	5.03311435	1824
chr14.fa	21558205	21572863	ZNF219	4.039865326	912
chr14.fa	21567174	21571883	C14orf176	0	0
chr14.fa	21623096	21624184	OR5AU1	0	0
chr14.fa	21677296	21737638	HNRNPC	77.925243	16821
chr14.fa	21756136	21819460	RPGRIP1	0	0
chr14.fa	21819631	21852425	SUPT16H	24.19220277	7524
chr14.fa	21853353	21905457	CHD8	31.91605056	17765
chr14.fa	21860310	21860412	SNORD9	0	0
chr14.fa	21865452	21865560	SNORD8	0	0
chr14.fa	21927179	21945132	RAB2B	10.78898868	2066
chr14.fa	21945335	21967319	TOX4	23.81809654	6609
chr14.fa	21967450	21979457	METTL3	29.61614065	3166

chr14.fa	21989232	22005337	SALL2	4.427375521	1444
chr14.fa	22037934	22038875	OR10G3	0	0
chr14.fa	22102066	22102998	OR10G2	0	0
chr14.fa	22133297	22134238	OR4E2	0	0
chr14.fa	23033807	23058143	DAD1	42.6970571	1976
chr14.fa	23067147	23081265	ABHD4	28.74066581	4788
chr14.fa	23235731	23240998	OXA1L	54.60023173	6232
chr14.fa	23242432	23289014	SLC7A7	29.07486135	5168
chr14.fa	23299092	23304246	MRPL52	13.87988326	1199
chr14.fa	23305793	23316803	MMP14	2.264668018	532
chr14.fa	23340960	23347291	LRP10	10.21337113	2204
chr14.fa	23352432	23356889	REM2	0	0
chr14.fa	23369854	23388396	RBM23	27.59967194	4772
chr14.fa	23389733	23398661	PRMT5	25.93230879	4253
chr14.fa	23415437	23426351	HAUS4	10.48175168	1140
chr14.fa	23440410	23451848	JUB	5.067904422	1596
chr14.fa	23456110	23479360	C14orf93	3.047821153	444
chr14.fa	23495060	23504429	PSMB5	57.4665722	5548
chr14.fa	23511376	23513269	PSMB11	0	0
chr14.fa	23516270	23526747	CDH24	0	0
chr14.fa	23527774	23564787	ACIN1	32.42148555	10994
chr14.fa	23564824	23569665	C14orf119	8.572364095	1596
chr14.fa	23586515	23588474	CEBPE	0	0
chr14.fa	23594504	23652849	SLC7A8	28.88434429	8360
chr14.fa	23742844	23755309	HOMEZ	10.09680179	2508
chr14.fa	23765112	23772057	PPP1R3E	2.536211307	760
chr14.fa	23776104	23780968	BCL2L2	53.85051321	10716
chr14.fa	23789397	23791029	PABPN1	3.246320351	352
chr14.fa	23815527	23822080	SLC22A17	1.424886887	228
chr14.fa	23825611	23834842	EFS	9.544528227	1976
chr14.fa	23842018	23845612	IL25	0	0
chr14.fa	23846017	23848981	CMTM5	0	0
chr14.fa	23851199	23877486	MYH6	0	0
chr14.fa	23857805	23857875	MIR208A	0	0
chr14.fa	23881947	23904870	MYH7	0	0
chr14.fa	23887196	23887272	MIR208B	0	0
chr14.fa	23938898	23947402	NGDN	12.89823092	1360
chr14.fa	23990065	24020858	ZFHX2	1.122017472	684
chr14.fa	24025198	24028776	THTPA	26.21725605	3116
chr14.fa	24028788	24037243	AP1G2	18.16960455	3413
chr14.fa	24037280	24048009	JPH4	0	0
chr14.fa	24105573	24114848	DHRS2	611.6207606	68510
chr14.fa	24391457	24403777	C14orf165	0	0

chr14.fa	24407940	24424243	C14orf167	11.99112874	2633
chr14.fa	24424299	24438488	DHRS4	14.01121201	988
chr14.fa	24439083	24475617	DHRS4L2	4.106734555	532
chr14.fa	24505710	24520580	DHRS4L1	0	0
chr14.fa	24521206	24538937	LRRC16B	0.497603454	152
chr14.fa	24540756	24547296	CPNE6	1.094004687	146
chr14.fa	24549316	24553832	NRL	1.159669065	152
chr14.fa	24563483	24573339	PCK2	11.3377983	1672
chr14.fa	24583906	24594451	DCAF11	40.06957834	11898
chr14.fa	24600675	24602058	FITM1	0	0
chr14.fa	24605378	24608173	PSME1	137.3371977	9046
chr14.fa	24608177	24610797	FAM158A	11.00586186	608
chr14.fa	24612574	24615855	PSME2	79.20916233	4360
chr14.fa	24616659	24629870	RNF31	14.16272202	3344
chr14.fa	24630422	24635774	IRF9	25.92086271	2888
chr14.fa	24641234	24649424	REC8	1.99764292	304
chr14.fa	24649464	24658124	IPO4	9.32148019	2204
chr14.fa	24658353	24664882	TM9SF1	3.294966209	608
chr14.fa	24674926	24677454	TSSK4	1.730316609	152
chr14.fa	24678787	24683036	CHMP4A	40.51718047	3648
chr14.fa	24683468	24685276	MDP1	4.38550695	76
chr14.fa	24686057	24686429	NEDD8	112.9345978	2797
chr14.fa	24701648	24708447	GMPR2	33.27075488	4891
chr14.fa	24708851	24711880	TINF2	19.79856307	2432
chr14.fa	24718320	24732416	TGM1	0	0
chr14.fa	24734744	24740803	RABGGTA	12.351831	1596
chr14.fa	24759804	24769039	DHRS1	11.94368774	1368
chr14.fa	24769098	24774374	C14orf21	15.09738517	2052
chr14.fa	24774393	24778036	CIDEB	27.10914698	2736
chr14.fa	24779357	24781259	LTB4R2	0.917192806	76
chr14.fa	24782313	24787242	LTB4R	0	0
chr14.fa	24787555	24804277	ADCY4	0.335099178	76
chr14.fa	24805227	24809242	RIPK3	3.418312828	436
chr14.fa	24836145	24848810	NFATC4	0.869450586	304
chr14.fa	24867992	24888494	NYNRIN	1.89387513	988
chr14.fa	24895740	24898731	CBLN3	2.441630505	380
chr14.fa	24899141	24910547	KHNYN	25.53425615	9196
chr14.fa	24910880	24912007	SDR39U1	28.6152107	684
chr14.fa	24974712	24977471	CMA1	0	0
chr14.fa	25042724	25045466	CTSG	0	0
chr14.fa	25075693	25078864	GZMH	0	0
chr14.fa	25100161	25103432	GZMB	0	0
chr14.fa	25281306	25519095	STXBP6	11.11264177	1444

chr14.fa	26915089	27066960	NOVA1	0	0
chr14.fa	27377848	27377931	MIR4307	0	0
chr14.fa	29236287	29238871	FOXG1	0	0
chr14.fa	29241910	29264000	C14orf23	0	0
chr14.fa	30045687	30396899	PRKD1	19.86513109	4850
chr14.fa	31028329	31089046	G2E3	2.255932848	836
chr14.fa	31091521	31205018	SCFD1	33.53024966	4800
chr14.fa	31343741	31359822	COCH	0.397149003	76
chr14.fa	31363005	31495607	STRN3	17.62184918	4940
chr14.fa	31483852	31483948	MIR624	0	0
chr14.fa	31494683	31562638	AP4S1	0	0
chr14.fa	31569324	31676689	HECTD1	66.47358629	40315
chr14.fa	31760994	31858181	HEATR5A	14.52462913	6612
chr14.fa	31915243	31926680	C14orf126	7.260582594	1292
chr14.fa	31952150	31957151	GPR33	0	0
chr14.fa	32030591	32330429	NUBPL	4.663978132	947
chr14.fa	32544625	32545905	C14orf128	0.893547606	76
chr14.fa	32546495	32628934	ARHGAP5	24.43196811	15580
chr14.fa	32798479	33302268	AKAP6	0.550918109	380
chr14.fa	33408459	34273382	NPAS3	0.76508037	304
chr14.fa	34393421	34420284	EGLN3	9.250996408	1672
chr14.fa	34902144	34931468	C14orf147	9.485641136	1596
chr14.fa	34985135	35008943	EAPP	19.5297307	1748
chr14.fa	35030618	35099366	SNX6	23.00858729	4545
chr14.fa	35179588	35184029	CFL2	3.257314616	760
chr14.fa	35221937	35344853	BAZ1A	19.21631884	7676
chr14.fa	35409128	35409702	C14orf19	0	0
chr14.fa	35452104	35498773	SRP54	28.10088994	4256
chr14.fa	35514113	35552589	FAM177A1	16.64426322	3710
chr14.fa	35554678	35591519	PPP2R3C	12.48210552	1520
chr14.fa	35591776	35743270	KIAA0391	14.47206751	2508
chr14.fa	35761574	35786682	PSMA6	56.10825333	3800
chr14.fa	35870716	35873960	NFKBIA	39.44411007	4104
chr14.fa	36003248	36006260	INSM2	0	0
chr14.fa	36007558	36278432	RALGAPA1	5.821990527	3040
chr14.fa	36295597	36341169	BRMS1L	0.885866681	152
chr14.fa	36767764	36789882	MBIP	3.317406559	363
chr14.fa	36942494	36982990	SFTA3	0	0
chr14.fa	36985604	36989430	NKX2-1	0	0
chr14.fa	37049216	37051786	NKX2-8	0	0
chr14.fa	37126773	37147011	PAX9	4.422254905	912
chr14.fa	37147126	37344209	SLC25A21	1.646880679	152
chr14.fa	37641231	37642998	LOC100129794	0.824720494	76

chr14.fa	37667118	38020464	MIPOL1	2.818297042	1195
chr14.fa	38059191	38064489	FOXA1	103.0515065	21280
chr14.fa	38677204	38682268	SSTR1	0	0
chr14.fa	38723319	38725574	CLEC14A	0	0
chr14.fa	39501123	39572437	SEC23A	10.72784249	2736
chr14.fa	39583488	39606177	SIP1	0.842190833	76
chr14.fa	39617015	39639634	TRAPPC6B	8.197806048	1824
chr14.fa	39644387	39652422	PNN	37.11287404	8886
chr14.fa	39703125	39722575	MIA2	0.501820432	76
chr14.fa	39734476	39820397	CTAGE5	8.405793448	2266
chr14.fa	39865577	39901704	FBXO33	6.446555153	2204
chr14.fa	42076764	42373752	LRFN5	0	0
chr14.fa	44973354	44976499	FSCB	0	0
chr14.fa	45366507	45376460	C14orf28	1.969328922	380
chr14.fa	45393527	45431179	KLHL28	3.144962263	1368
chr14.fa	45431416	45543634	FAM179B	11.9191389	4940
chr14.fa	45553302	45584801	PRPF39	6.392186252	1216
chr14.fa	45580078	45580176	SNORD127	0	0
chr14.fa	45584805	45603732	FKBP3	37.47237145	3344
chr14.fa	45605136	45670093	FANCM	0.80288257	380
chr14.fa	45672393	45722605	C14orf106	4.312914679	1368
chr14.fa	47120220	47121028	RPL10L	0	0
chr14.fa	47308828	48144157	MDGA2	0	0
chr14.fa	48230198	48230307	MIR548Y	0	0
chr14.fa	50044042	50053094	RPS29	486.389303	26676
chr14.fa	50065415	50081390	PPIL5	5.903468575	684
chr14.fa	50085407	50087349	RPL36AL	269.7066442	9348
chr14.fa	50087489	50090199	MGAT2	0.844449929	152
chr14.fa	50091892	50101948	C14orf104	1.931526723	380
chr14.fa	50110270	50155098	POLE2	1.765407894	228
chr14.fa	50159823	50219870	KLHDC1	3.402800372	608
chr14.fa	50234787	50249856	KLHDC2	32.32283837	3876
chr14.fa	50250532	50319539	SDCCAG1	17.16204792	4696
chr14.fa	50359736	50363772	ARF6	49.39919127	12920
chr14.fa	50448430	50474238	C14orf182	0	0
chr14.fa	50550369	50559361	C14orf183	0	0
chr14.fa	50575350	50583297	C14orf138	3.207915726	380
chr14.fa	50583846	50698099	SOS2	21.32812139	7524
chr14.fa	50709152	50778947	L2HGDH	3.906578687	1581
chr14.fa	50779047	50792946	ATP5S	6.693549603	836
chr14.fa	50796720	50862617	CDKL1	8.75233871	684
chr14.fa	50885243	50999376	MAP4K5	17.22590502	5036
chr14.fa	50999800	51099784	ATL1	3.525544565	684

chr14.fa	51100360	51135023	SAV1	12.46192426	2508
chr14.fa	51186481	51297839	NIN	65.51377188	47576
chr14.fa	51338878	51371688	ABHD12B	0	0
chr14.fa	51371935	51411248	PYGL	26.45928049	5000
chr14.fa	51441981	51562422	TRIM9	0.331334019	152
chr14.fa	51706886	51724372	TMX1	1.328800021	362
chr14.fa	51955855	52197444	FRMD6	0	0
chr14.fa	52116236	52118462	C14orf82	0.514019548	76
chr14.fa	52327050	52436518	GNG2	0	0
chr14.fa	52456228	52471420	C14orf166	137.3225889	9583
chr14.fa	52471520	52535946	NID2	0.226813196	76
chr14.fa	52734431	52743442	PTGDR	0	0
chr14.fa	52781016	52795322	PTGER2	0	0
chr14.fa	52897308	53019301	TXNDC16	3.946037556	1216
chr14.fa	53019866	53104431	GPR137C	0.883155766	228
chr14.fa	53108605	53162419	ERO1L	3.11228068	684
chr14.fa	53173896	53194716	PSMC6	19.96739282	2112
chr14.fa	53196883	53241705	STYX	2.711968943	831
chr14.fa	53241911	53258386	GNPNAT1	13.69569166	3572
chr14.fa	53323989	53417815	FERMT2	5.654817454	1292
chr14.fa	53503458	53620046	DDHD1	2.116019528	1824
chr14.fa	54416455	54423554	BMP4	0	0
chr14.fa	54863673	54886934	CDKN3	0	0
chr14.fa	54893647	54908148	CNIH	7.239196489	684
chr14.fa	54941209	54955744	GMFB	11.93901894	3270
chr14.fa	54976587	55005334	CGRRF1	2.595550217	228
chr14.fa	55034637	55260033	SAMD4A	2.03664997	912
chr14.fa	55308724	55369542	GCH1	6.261158708	1216
chr14.fa	55344831	55344911	MIR4308	0	0
chr14.fa	55405656	55493819	WDHD1	0.759056116	304
chr14.fa	55493844	55516206	SOCS4	3.754466251	1748
chr14.fa	55518362	55536912	MAPK1IP1L	11.500905	4940
chr14.fa	55595935	55612148	LGALS3	51.64909986	6996
chr14.fa	55614834	55658396	DLGAP5	0.371094101	76
chr14.fa	55738021	55820329	FBXO34	15.60538046	3724
chr14.fa	55833110	55878576	ATG14	6.275767527	1976
chr14.fa	55880930	55907263	TBPL2	0	0
chr14.fa	56042875	56046810	C14orf33	0	0
chr14.fa	56046925	56151302	KTN1	25.99209952	8479
chr14.fa	56232963	56234435	RPL13AP3	0	0
chr14.fa	56247853	56263392	C14orf34	0	0
chr14.fa	56585093	56768031	PELI2	1.205754615	456
chr14.fa	57046511	57116232	C14orf101	2.685010402	760

chr14.fa	57267425	57277184	OTX2	0	0
chr14.fa	57279901	57397550	OTX2OS1	0	0
chr14.fa	57669194	57735605	EXOC5	8.344647261	4709
chr14.fa	57735618	57756797	MUDENG	15.96442605	3268
chr14.fa	57857271	57879466	NAA30	8.952193366	2660
chr14.fa	57936595	57960576	C14orf105	7.746137538	1292
chr14.fa	58030640	58063615	SLC35F4	0	0
chr14.fa	58470808	58618847	C14orf37	0.742790627	152
chr14.fa	58666833	58702353	ACTR10	22.95195929	3720
chr14.fa	58711593	58738726	PSMA3	44.45086831	2736
chr14.fa	58732083	58764855	FLJ31306	4.157790115	720
chr14.fa	58765222	58840451	ARID4A	10.40554486	3876
chr14.fa	58862644	58875369	TOMM20L	0	0
chr14.fa	58875420	58894232	TIMM9	14.47462782	988
chr14.fa	58894710	59015549	KIAA0586	5.32288101	1976
chr14.fa	59104757	59115038	DACT1	0	0
chr14.fa	59655399	59836471	DAAM1	32.21259451	9103
chr14.fa	59930240	59932059	GPR135	0	0
chr14.fa	59939406	59951073	C14orf149	2.486511204	228
chr14.fa	59951161	59972081	JKAMP	0.538568387	76
chr14.fa	59977364	60043549	C14orf38	0	0
chr14.fa	60062694	60337557	RTN1	2.185599672	532
chr14.fa	60558629	60601532	C14orf135	6.113112645	1596
chr14.fa	60611500	60632211	DHRS7	60.19917422	5548
chr14.fa	60712470	60765805	PPM1A	13.03257181	9877
chr14.fa	60902674	60952764	C14orf39	0	0
chr14.fa	60975938	60978525	SIX6	0	0
chr14.fa	61111417	61116155	SIX1	5.963711124	1064
chr14.fa	61176256	61190852	SIX4	5.289898215	2204
chr14.fa	61201459	61435398	MNAT1	14.06964728	1292
chr14.fa	61438167	61447782	TRMT5	4.551625778	1596
chr14.fa	61447832	61550451	SLC38A6	3.03878477	456
chr14.fa	61744089	61748530	TMEM30B	4.638224442	1368
chr14.fa	61788515	62017698	PRKCH	38.52360393	8968
chr14.fa	62162119	62214977	HIF1A	50.75871499	13680
chr14.fa	62229075	62263146	SNAPC1	2.154725366	380
chr14.fa	62462541	62568427	SYT16	3.283821338	684
chr14.fa	62584075	62600903	FLJ43390	0	0
chr14.fa	63173945	63568584	KCNH5	0	0
chr14.fa	63671145	63758559	RHOJ	0	0
chr14.fa	63779642	63784563	GPHB5	0	0
chr14.fa	63841355	64010079	PPP2R5E	13.78093487	3080
chr14.fa	64063757	64108591	WDR89	6.753942758	1444

chr14.fa	64150935	64194756	SGPP1	2.422051677	532
chr14.fa	64319683	64693167	SYNE2	8.326725102	12312
chr14.fa	64693751	64805268	ESR2	1.008761481	228
chr14.fa	64812191	64814329	TEX21P	0	0
chr14.fa	64854759	64926725	MTHFD1	17.48042979	4002
chr14.fa	64932217	64941221	AKAP5	0.529381398	228
chr14.fa	64953555	64970554	ZBTB25	9.43563982	988
chr14.fa	64971292	65000408	ZBTB1	10.70299244	4028
chr14.fa	65007186	65009954	HSPA2	3.720278604	684
chr14.fa	65016620	65056096	C14orf50	1.62941034	152
chr14.fa	65171193	65211060	PLEKHG3	8.536670385	2494
chr14.fa	65213001	65289866	SPTB	0.33043038	228
chr14.fa	65381140	65401913	CHURC1	14.55731072	3268
chr14.fa	65405872	65409531	GPX2	14.96967096	988
chr14.fa	65412532	65438875	RAB15	38.46215653	8660
chr14.fa	65453507	65529370	FNTB	9.744834702	1824
chr14.fa	65472892	65569227	MAX	31.96198551	6112
chr14.fa	65877311	65879335	LOC645431	0	0
chr14.fa	65879535	66209962	FUT8	1.689652889	380
chr14.fa	66953109	66965271	NCRNA00238	0	0
chr14.fa	66974125	67648525	GPHN	8.776586336	2507
chr14.fa	67656110	67695267	FAM71D	0	0
chr14.fa	67708021	67802536	MPP5	32.04993963	11400
chr14.fa	67804581	67826720	ATP6V1D	49.21816241	5902
chr14.fa	67827034	67853233	EIF2S1	20.61123506	5700
chr14.fa	67853700	67878828	PLEK2	7.055757928	684
chr14.fa	67936983	67982021	TMEM229B	5.346074391	1444
chr14.fa	68000008	68056022	PLEKHH1	17.60377641	7372
chr14.fa	68056256	68067017	PIGH	8.548869501	684
chr14.fa	68086579	68118436	ARG2	160.4396128	17556
chr14.fa	68120154	68141602	VTI1B	80.47937647	5333
chr14.fa	68143519	68162510	RDH11	30.33257517	5168
chr14.fa	68168603	68201168	RDH12	3.047369334	380
chr14.fa	68213237	68283306	ZFYVE26	9.937610859	6384
chr14.fa	68286496	69062738	RAD51L1	2.969957658	760
chr14.fa	69254375	69259785	ZFP36L1	28.78569712	5776
chr14.fa	69261464	69263190	C14orf181	0	0
chr14.fa	69340840	69446083	ACTN1	34.72169667	8740
chr14.fa	69517637	69619914	DCAF5	12.62232005	5016
chr14.fa	69658194	69710737	EXD2	2.333344523	836
chr14.fa	69726681	69821190	GALNTL1	0.531339281	152
chr14.fa	69846840	69865021	ERH	84.30944712	4484
chr14.fa	69865407	69929107	SLC39A9	11.86778212	4256

chr14.fa	69951471	69995215	UPF0639	1.263436856	152
chr14.fa	70036531	70037918	C14orf162	0	0
chr14.fa	70078310	70181861	KIAA0247	6.367185594	2280
chr14.fa	70233000	70234430	LOC100289511	0	0
chr14.fa	70233834	70238722	SRSF5	78.41064734	7898
chr14.fa	70242552	70264006	SLC10A1	0	0
chr14.fa	70346143	70499083	SMOC1	0	0
chr14.fa	70510934	70655787	SLC8A3	0	0
chr14.fa	70712470	70714518	ADAM21P1	0	0
chr14.fa	70791798	70826444	COX16	22.5953234	2570
chr14.fa	70833213	70883807	SYNJ2BP	22.85978819	10716
chr14.fa	70924217	70926622	ADAM21	0	0
chr14.fa	70989078	71001732	ADAM20	0	0
chr14.fa	71050957	71067384	MED6	9.368017559	836
chr14.fa	71108504	71142077	TTC9	8.995417394	3116
chr14.fa	71194854	71275888	MAP3K9	7.56254837	2812
chr14.fa	71374122	71582099	PCNX	4.42993583	3800
chr14.fa	71865054	71865124	SNORD56B	0	0
chr14.fa	71954578	71956420	LOC145474	0	0
chr14.fa	71996042	72206120	SIPA1L1	15.0580769	6004
chr14.fa	72399786	73033238	RGS6	0	0
chr14.fa	73136660	73360809	DPF3	0	0
chr14.fa	73393040	73426357	DCAF4	2.710161666	456
chr14.fa	73436159	73493920	ZFYVE1	15.34196992	4636
chr14.fa	73525221	73588076	RBM25	29.3477601	8385
chr14.fa	73603143	73690399	PSEN1	6.402879305	2584
chr14.fa	73704205	73741347	PAPLN	0.784960412	304
chr14.fa	73741918	73925286	NUMB	31.41076618	7600
chr14.fa	73945192	74025651	HEATR4	1.342655808	304
chr14.fa	73957644	73960105	C14orf169	5.715963641	912
chr14.fa	74003928	74010498	ACOT1	9.28247314	988
chr14.fa	74035772	74042359	ACOT2	14.89873536	1748
chr14.fa	74058410	74062470	ACOT4	7.013588144	760
chr14.fa	74083548	74086592	ACOT6	0	0
chr14.fa	74111578	74168649	DNAL1	3.225687278	1444
chr14.fa	74178486	74181128	PNMA1	8.228379141	1444
chr14.fa	74181825	74253896	C14orf43	15.36953088	8360
chr14.fa	74318534	74352168	PTGR2	7.684840744	1368
chr14.fa	74353586	74398803	ZNF410	37.31272869	5396
chr14.fa	74400705	74413308	FAM161B	8.008042019	1140
chr14.fa	74416643	74429813	COQ6	10.9075159	1216
chr14.fa	74433181	74486026	ENTPD5	26.0104735	3537
chr14.fa	74486059	74532796	C14orf45	4.452225573	836

chr14.fa	74526872	74551196	ALDH6A1	102.1999781	12900
chr14.fa	74551656	74667117	LIN52	6.365980743	760
chr14.fa	74706175	74729441	VSX2	0	0
chr14.fa	74751980	74769767	ABCD4	3.263037659	684
chr14.fa	74815166	74826711	VRTN	0	0
chr14.fa	74872596	74892805	TMEM90A	0	0
chr14.fa	74946643	74960084	NPC2	56.14575432	3344
chr14.fa	74960450	74962271	ISCA2	16.18205226	1140
chr14.fa	74964886	75079034	LTBP2	0.267627523	152
chr14.fa	75127955	75179807	KIAA0317	10.68205816	3862
chr14.fa	75179850	75203390	FCF1	15.22976817	2432
chr14.fa	75230069	75304013	YLPM1	27.85208822	15048
chr14.fa	75321835	75330537	PROX2	2.573561687	304
chr14.fa	75348594	75370450	DLST	28.90241706	5454
chr14.fa	75372346	75389145	RPS6KL1	0	0
chr14.fa	75408538	75422291	PGF	0.661613793	76
chr14.fa	75469612	75476294	EIF2B2	15.56878311	1593
chr14.fa	75480467	75518235	MLH3	7.808789788	4094
chr14.fa	75519928	75530736	ACYP1	1.668567997	76
chr14.fa	75536299	75544799	FAM164C	3.666210917	380
chr14.fa	75548818	75593778	NEK9	19.37535917	7131
chr14.fa	75598171	75643349	TMED10	10.49590868	2888
chr14.fa	75745481	75748937	FOS	7.425647178	1064
chr14.fa	75894509	75939404	JDP2	2.14252625	608
chr14.fa	75988784	76013334	BATF	3.652957556	228
chr14.fa	76044940	76114512	FLVCR2	1.499286435	380
chr14.fa	76117233	76127538	C14orf1	26.75702929	2736
chr14.fa	76127551	76421425	TTLL5	12.24610533	3868
chr14.fa	76424442	76448092	TGFB3	2.157586887	456
chr14.fa	76452096	76550092	C14orf179	20.80581849	1282
chr14.fa	76618259	76669134	C14orf118	8.042380272	1343
chr14.fa	76837690	76968180	ESRRB	1.139337205	228
chr14.fa	77228235	77249363	VASH1	0.569442693	228
chr14.fa	77253586	77279283	ANGEL1	4.176314699	1140
chr14.fa	77292725	77336645	C14orf166B	0	0
chr14.fa	77490886	77495034	C14orf4	32.00159498	8816
chr14.fa	77564578	77583630	KIAA1737	32.37961698	9348
chr14.fa	77597613	77608134	ZDHHC22	0	0
chr14.fa	77648102	77725838	TMEM63C	0	0
chr14.fa	77731834	77737655	NGB	0	0
chr14.fa	77741299	77787225	POMT2	1.409073218	456
chr14.fa	77787230	77797940	GSTZ1	18.55033746	2121
chr14.fa	77808114	77843396	TMED8	7.022172707	456

chr14.fa	77843762	77857587	SAMD15	3.391504894	456
chr14.fa	77860424	77889379	C14orf148	0	0
chr14.fa	77893018	77923983	VIPAR	9.516214229	1876
chr14.fa	77924373	77935815	AHSA1	47.20440461	4388
chr14.fa	77940738	77965210	ISM2	0	0
chr14.fa	77973270	78083110	SPTLC2	5.221522922	2508
chr14.fa	78138749	78174356	ALKBH1	3.099479138	532
chr14.fa	78174425	78183941	C14orf156	89.37750215	2356
chr14.fa	78183944	78227497	SNW1	31.59043959	4451
chr14.fa	78227173	78236085	C14orf178	0	0
chr14.fa	78266426	78400297	ADCK1	2.56181439	380
chr14.fa	78870093	80330760	NRXN3	0	0
chr14.fa	80663869	80678525	DIO2	0	0
chr14.fa	80962821	81405884	C14orf145	2.311355993	684
chr14.fa	81421869	81612646	TSHR	0	0
chr14.fa	81646394	81687575	GTF2A1	19.49614548	2356
chr14.fa	81669039	81669178	SNORA79	0	0
chr14.fa	81736909	81864927	STON2	15.68490063	3040
chr14.fa	81939239	82000205	SEL1L	5.329206478	2328
chr14.fa	85996488	86094270	FLRT2	0	0
chr14.fa	88399358	88459907	GALC	1.617813649	456
chr14.fa	88471468	88481155	GPR65	0	0
chr14.fa	88646452	88793256	KCNK10	0.699867811	380
chr14.fa	88851988	88904804	SPATA7	6.68119988	893
chr14.fa	88932122	89021123	PTPN21	9.378108186	3870
chr14.fa	89029253	89079853	ZC3H14	18.32563275	6101
chr14.fa	89081174	89259096	EML5	31.38139794	14942
chr14.fa	89290978	89344335	TTC8	20.78277572	3040
chr14.fa	89622516	90085494	FOXN3	19.65774612	10562
chr14.fa	89883698	89886137	LOC400236	6.376673796	456
chr14.fa	90042560	90043820	PRO1768	0	0
chr14.fa	90263469	90421089	C14orf143	11.52846597	532
chr14.fa	90422246	90511108	TDP1	3.60129957	896
chr14.fa	90528108	90652195	KCNK13	0.454680637	76
chr14.fa	90722894	90738966	PSMC1	2.165117206	228
chr14.fa	90744398	90798279	C14orf102	9.017405925	2280
chr14.fa	90863327	90874619	CALM1	101.3427266	30166
chr14.fa	90921574	90925249	LOC400238	0	0
chr14.fa	91006932	91282761	TTC7B	17.30181064	3945
chr14.fa	91337167	91526993	RPS6KA5	3.7154592	1064
chr14.fa	91580357	91691703	C14orf159	6.857861155	1596
chr14.fa	91592770	91592896	SNORA11B	0	0
chr14.fa	91698876	91720224	GPR68	0	0

chr14.fa	91737667	91884133	CCDC88C	27.96910937	13906
chr14.fa	91923956	91976644	SMEK1	34.6939851	8968
chr14.fa	92038788	92041383	C14orf184	0	0
chr14.fa	92047118	92198413	CATSPERB	0.952284091	228
chr14.fa	92246267	92333880	TC2N	33.88071069	11932
chr14.fa	92335755	92414046	FBLN5	3.912452335	684
chr14.fa	92434243	92506403	TRIP11	14.37161306	7718
chr14.fa	92524896	92572965	ATXN3	4.365024483	2052
chr14.fa	92582468	92588153	NDUFB1	76.48936185	2128
chr14.fa	92588298	92630543	CPSF2	19.84434741	6683
chr14.fa	92788925	92967825	SLC24A4	0	0
chr14.fa	92980125	93155334	RIN3	0.892643968	228
chr14.fa	93170152	93215047	LGMN	3.157613198	456
chr14.fa	93260650	93306304	GOLGA5	42.73862446	8204
chr14.fa	93389445	93401638	CHGA	0	0
chr14.fa	93403259	93582263	ITPK1	20.0969143	6652
chr14.fa	93533797	93538478	ITPK1-AS1	0	0
chr14.fa	93648541	93651249	MOAP1	33.62317379	5396
chr14.fa	93651296	93653425	C14orf109	20.25851493	1824
chr14.fa	93669237	93673400	C14orf142	8.197806048	608
chr14.fa	93673460	93695561	UBR7	14.79963637	3572
chr14.fa	93703896	93799385	BTBD7	19.82160585	12540
chr14.fa	93799565	94173689	KIAA1409	0	0
chr14.fa	93813537	93814700	COX8C	0	0
chr14.fa	94184644	94254766	PRIMA1	0.627124934	152
chr14.fa	94371076	94392718	C14orf86	0	0
chr14.fa	94385258	94395944	FAM181A	0	0
chr14.fa	94400513	94423767	ASB2	0	0
chr14.fa	94463616	94478041	C14orf48	0	0
chr14.fa	94492724	94515276	OTUB2	0.591130011	152
chr14.fa	94517268	94547558	DDX24	52.80425074	10336
chr14.fa	94547639	94569060	IFI27L1	3.201740865	152
chr14.fa	94577079	94583033	IFI27	0	0
chr14.fa	94594118	94595957	IFI27L2	4.9019362	152
chr14.fa	94640649	94746072	PPP4R4	0	0
chr14.fa	94749650	94759596	SERPINA10	0	0
chr14.fa	94770585	94789688	SERPINA6	0.76718886	76
chr14.fa	94843084	94857029	SERPINA1	5.277548492	1292
chr14.fa	94908801	94919122	SERPINA11	0	0
chr14.fa	94929058	94942670	SERPINA9	0	0
chr14.fa	94953620	94984181	SERPINA12	0	0
chr14.fa	95027783	95036243	SERPINA4	0	0
chr14.fa	95047731	95059457	SERPINA5	5.44577581	836

chr14.fa	95078714	95090390	SERPINA3	10.07827721	1064
chr14.fa	95107062	95113331	SERPINA13	0	0
chr14.fa	95234560	95236499	GSC	0	0
chr14.fa	95552565	95623759	DICER1	24.61992486	17024
chr14.fa	95604256	95604323	MIR3173	0	0
chr14.fa	95624025	95646270	FLJ45244	0	0
chr14.fa	95648276	95786245	CLMN	20.70807495	17531
chr14.fa	95873604	95876427	C14orf139	2.837273445	532
chr14.fa	95883831	95942173	C14orf49	1.048521563	228
chr14.fa	95999249	96001209	SNHG10	1.721280227	152
chr14.fa	95999692	95999966	SCARNA13	0	0
chr14.fa	96001323	96011055	GLRX5	42.03092512	3268
chr14.fa	96117515	96139789	TCL6	0.692186886	152
chr14.fa	96152754	96158980	TCL1B	0	0
chr14.fa	96176304	96180533	TCL1A	0	0
chr14.fa	96505662	96560135	C14orf132	0.458445797	228
chr14.fa	96671135	96710666	BDKRB2	0	0
chr14.fa	96722559	96731100	BDKRB1	0.88797517	76
chr14.fa	96747595	96829678	ATG2B	9.590764384	7068
chr14.fa	96846022	96853563	C14orf129	13.84388833	1900
chr14.fa	96858448	96955112	AK7	0.864480576	152
chr14.fa	96968720	97033448	PAPOLA	42.62009725	12760
chr14.fa	97263684	97347951	VRK1	7.393266808	836
chr14.fa	98098984	98152995	LOC100129345	0	0
chr14.fa	98391947	98444461	C14orf64	0.785111018	76
chr14.fa	99177950	99184103	C14orf177	0	0
chr14.fa	99635625	99737822	BCL11B	0.585858788	304
chr14.fa	99864083	99947226	SETD3	57.5680809	12614
chr14.fa	99947739	99977602	CCNK	28.90663403	4522
chr14.fa	99977853	100070727	CCDC85C	15.83580821	5700
chr14.fa	100111480	100143011	HHIPL1	0	0
chr14.fa	100150755	100193638	CYP46A1	0	0
chr14.fa	100259745	100408395	EML1	13.30803086	3994
chr14.fa	100531751	100610573	EVL	14.2920929	1748
chr14.fa	100612753	100626012	DEGS2	0.814629867	76
chr14.fa	100705102	100744804	YY1	29.58677241	5092
chr14.fa	100757453	100772860	SLC25A29	4.829645141	760
chr14.fa	100774196	100774293	MIR345	0	0
chr14.fa	100789679	100796715	SLC25A47	0	0
chr14.fa	100800125	100842680	WARS	30.85201654	6080
chr14.fa	100842755	100996640	WDR25	2.081982488	304
chr14.fa	101003484	101036131	BEGAIN	0	0
chr14.fa	101123605	101139081	C14orf70	0	0

chr14.fa	101193202	101201467	DLK1	0	0
chr14.fa	101292445	101327360	MEG3	0	0
chr14.fa	101318727	101318824	MIR770	0	0
chr14.fa	101335397	101335485	MIR493	0	0
chr14.fa	101340830	101340922	MIR337	0	0
chr14.fa	101341370	101341441	MIR665	0	0
chr14.fa	101346992	101351184	RTL1	0	0
chr14.fa	101361107	101373305	MEG8	0	0
chr14.fa	101391158	101391227	SNORD113-1	0	0
chr14.fa	101393679	101393749	SNORD113-2	0	0
chr14.fa	101402828	101402901	SNORD113-4	0	0
chr14.fa	101404524	101404600	SNORD113-5	0	0
chr14.fa	101405893	101405966	SNORD113-6	0	0
chr14.fa	101407463	101407538	SNORD113-7	0	0
chr14.fa	101411986	101412056	SNORD113-9	0	0
chr14.fa	101416170	101416240	SNORD114-1	0	0
chr14.fa	101418193	101418269	SNORD114-2	0	0
chr14.fa	101419686	101419759	SNORD114-3	0	0
chr14.fa	101420681	101420784	SNORD114-4	0	0
chr14.fa	101421707	101421775	SNORD114-5	0	0
chr14.fa	101423503	101423573	SNORD114-6	0	0
chr14.fa	101429391	101429466	SNORD114-7	0	0
chr14.fa	101431118	101431188	SNORD114-8	0	0
chr14.fa	101432366	101432436	SNORD114-9	0	0
chr14.fa	101433389	101433459	SNORD114-10	0	0
chr14.fa	101434448	101434521	SNORD114-11	0	0
chr14.fa	101435285	101435358	SNORD114-12	0	0
chr14.fa	101436216	101436288	SNORD114-13	0	0
chr14.fa	101438440	101438513	SNORD114-14	0	0
chr14.fa	101439007	101439077	SNORD114-15	0	0
chr14.fa	101439932	101440000	SNORD114-16	0	0
chr14.fa	101441143	101441216	SNORD114-17	0	0
chr14.fa	101442162	101442232	SNORD114-18	0	0
chr14.fa	101442814	101442887	SNORD114-19	0	0
chr14.fa	101447341	101447411	SNORD114-20	0	0
chr14.fa	101448312	101448382	SNORD114-21	0	0
chr14.fa	101449263	101449333	SNORD114-22	0	0
chr14.fa	101450213	101450283	SNORD114-23	0	0
chr14.fa	101451114	101451184	SNORD114-24	0	0
chr14.fa	101452394	101452464	SNORD114-25	0	0
chr14.fa	101453383	101453453	SNORD114-26	0	0
chr14.fa	101454498	101454566	SNORD114-27	0	0
chr14.fa	101455467	101455537	SNORD114-28	0	0

chr14.fa	101456428	101456496	SNORD114-29	0	0
chr14.fa	101458256	101458326	SNORD114-30	0	0
chr14.fa	101459573	101459646	SNORD114-31	0	0
chr14.fa	101488403	101488469	MIR379	0	0
chr14.fa	101489662	101489757	MIR411	0	0
chr14.fa	101490131	101490193	MIR299	0	0
chr14.fa	101491354	101491414	MIR380	0	0
chr14.fa	101491901	101491988	MIR1197	0	0
chr14.fa	101492069	101492154	MIR323	0	0
chr14.fa	101492357	101492444	MIR758	0	0
chr14.fa	101493122	101493201	MIR329-1	0	0
chr14.fa	101493437	101493520	MIR329-2	0	0
chr14.fa	101495971	101496051	MIR494	0	0
chr14.fa	101496389	101496466	MIR1193	0	0
chr14.fa	101498324	101498401	MIR543	0	0
chr14.fa	101500092	101500173	MIR495	0	0
chr14.fa	101506027	101506092	MIR376C	0	0
chr14.fa	101506406	101506485	MIR376A2	0	0
chr14.fa	101506556	101506636	MIR654	0	0
chr14.fa	101506773	101506872	MIR376B	0	0
chr14.fa	101507119	101507186	MIR376A1	0	0
chr14.fa	101507700	101507781	MIR300	0	0
chr14.fa	101509314	101509398	MIR1185-1	0	0
chr14.fa	101510535	101510619	MIR1185-2	0	0
chr14.fa	101512257	101512331	MIR381	0	0
chr14.fa	101512792	101512875	MIR487B	0	0
chr14.fa	101513658	101513735	MIR539	0	0
chr14.fa	101514238	101514316	MIR889	0	0
chr14.fa	101515887	101515983	MIR655	0	0
chr14.fa	101518783	101518862	MIR487A	0	0
chr14.fa	101520643	101520718	MIR382	0	0
chr14.fa	101521024	101521096	MIR134	0	0
chr14.fa	101521595	101521660	MIR668	0	0
chr14.fa	101521756	101521828	MIR485	0	0
chr14.fa	101522556	101522637	MIR323B	0	0
chr14.fa	101526092	101526175	MIR154	0	0
chr14.fa	101526910	101527011	MIR496	0	0
chr14.fa	101528387	101528455	MIR377	0	0
chr14.fa	101530832	101530915	MIR541	0	0
chr14.fa	101531637	101531715	MIR409	0	0
chr14.fa	101531784	101531874	MIR412	0	0
chr14.fa	101531935	101532004	MIR369	0	0
chr14.fa	101532249	101532328	MIR410	0	0

chr14.fa	101533061	101533138	MIR656	0	0
chr14.fa	102018560	102022013	DIO3OS	0	0
chr14.fa	102026624	102026759	MIR1247	0	0
chr14.fa	102027688	102029789	DIO3	0	0
chr14.fa	102196774	102198862	NCRNA00239	0	0
chr14.fa	102228135	102394328	PPP2R5C	20.00504441	6688
chr14.fa	102430865	102517135	DYNC1H1	29.92066674	28489
chr14.fa	102547075	102606086	HSP90AA1	318.6023584	86184
chr14.fa	102606212	102690010	WDR20	11.91567495	2584
chr14.fa	102695178	102771531	RAGE	1.775498521	228
chr14.fa	102786096	102809044	ZNF839	5.17302767	1140
chr14.fa	102814619	102829253	CINP	34.74699854	2584
chr14.fa	102829300	102968818	TECPR2	7.068559469	4256
chr14.fa	102973198	102976128	ANKRD9	9.15129499	988
chr14.fa	103005981	103006063	MIR4309	0	0
chr14.fa	103059233	103196913	RCOR1	12.65108587	4636
chr14.fa	103243816	103377837	TRAF3	8.243138566	4256
chr14.fa	103388993	103397179	AMN	4.294691308	444
chr14.fa	103398716	103523742	CDC42BPB	53.80563251	23940
chr14.fa	103566481	103576894	C14orf73	0.887673957	152
chr14.fa	103592664	103603776	TNFAIP2	25.22250096	6992
chr14.fa	103653558	103655365	LOC100131366	0	0
chr14.fa	103800493	103811361	EIF5	45.95768506	18544
chr14.fa	103804186	103804311	SNORA28	0	0
chr14.fa	103851701	103970166	MARK3	26.5588313	6225
chr14.fa	103985996	103989170	CKB	552.6443595	52510
chr14.fa	103995509	104003410	TRMT61A	3.531719426	760
chr14.fa	104022881	104029151	BAG5	12.63557341	4408
chr14.fa	104029299	104057236	C14orf153	15.75568562	1292
chr14.fa	104095525	104167888	KLC1	19.07610431	4000
chr14.fa	104163954	104181823	XRCC3	0.433746352	76
chr14.fa	104182081	104200005	ZFYVE21	29.62156248	2964
chr14.fa	104200088	104313927	PPP1R13B	15.69544307	5168
chr14.fa	104378625	104387903	C14orf2	110.1830194	6738
chr14.fa	104394817	104519004	TDRD9	1.922038521	608
chr14.fa	104552048	104578925	ASPG	0	0
chr14.fa	104583742	104583851	MIR203	0	0
chr14.fa	104605060	104647235	KIF26A	0.338864337	152
chr14.fa	105046056	105056183	C14orf180	0	0
chr14.fa	105060420	105071097	TMEM179	0	0
chr14.fa	105155943	105185947	INF2	12.63692887	3952
chr14.fa	105190534	105213647	ADSSL1	6.059044957	836
chr14.fa	105219470	105225996	SIVA1	19.29945356	988

chr14.fa	105235687	105262080	AKT1	83.26845587	15796
chr14.fa	105266933	105271048	ZBTB42	8.499621217	2052
chr14.fa	105287538	105290055	MGC23270	1.3637407	228
chr14.fa	105331654	105363087	KIAA0284	11.47861526	5092
chr14.fa	105391187	105399573	PLD4	0	0
chr14.fa	105403591	105444694	AHNAK2	0	0
chr14.fa	105452616	105461855	C14orf79	4.239268163	532
chr14.fa	105475910	105487425	CDCA4	4.666086621	760
chr14.fa	105515737	105531754	GPR132	0	0
chr14.fa	105608076	105635161	JAG2	0.225457739	76
chr14.fa	105639276	105647660	NUDT14	22.61701072	1299
chr14.fa	105675623	105767329	BRF1	6.983617476	2076
chr14.fa	105714879	105717430	BTBD6	37.44315381	4940
chr14.fa	105781081	105864484	PACS2	7.516011001	3160
chr14.fa	105864920	105880196	LOC647310	0	0
chr14.fa	105886186	105937057	MTA1	16.38115388	3089
chr14.fa	105941131	105946500	CRIP2	24.63905187	1973
chr14.fa	105953257	105955124	CRIP1	0	0
chr14.fa	105956192	105965585	C14orf80	0	0
chr14.fa	105992953	105996539	TMEM121	0	0
chr14.fa	106383838	106398502	KIAA0125	0	0
chr14.fa	106435819	106438358	ADAM6	0	0
chr14.fa	106744269	106744966	NCRNA00226	0	0
chr14.fa	106938455	106951529	NCRNA00221	0	0
chr15.fa	20613650	20711433	HERC2P3	0	0
chr15.fa	20737094	20747114	GOLGA6L6	0	0
chr15.fa	20767674	20781026	GOLGA8C	0	0
chr15.fa	20874797	20961480	BCL8	37.59662171	1368
chr15.fa	21040701	22083137	POTEB	0	0
chr15.fa	21122021	22145802	NF1P1	0	0
chr15.fa	21932514	21940739	LOC646214	0.139160288	76
chr15.fa	22014420	22016878	CXADRP2	0	0
chr15.fa	22278032	22371088	LOC727924	0	0
chr15.fa	22368478	22369561	OR4M2	0	0
chr15.fa	22382473	22383423	OR4N4	0	0
chr15.fa	22413462	22414395	OR4N3P	0	0
chr15.fa	22546565	22570831	REREP3	0	0
chr15.fa	22702285	22715728	GOLGA8DP	0	0
chr15.fa	22736246	22746002	GOLGA6L1	0	0
chr15.fa	22833395	22873891	TUBGCP5	10.1135191	2584
chr15.fa	22892684	23003603	CYFIP1	35.60108728	12084
chr15.fa	23004684	23034427	NIPA2	12.08977592	2812
chr15.fa	23043279	23086843	NIPA1	3.126889498	1368

chr15.fa	23187729	23208357	WHAMML1	2.871159878	684
chr15.fa	23255242	23262743	GOLGA8IP	0	0
chr15.fa	23282265	23378259	HERC2P2	0	0
chr15.fa	23390722	23393943	HERC2P7	0	0
chr15.fa	23435070	23448423	GOLGA8E	0	0
chr15.fa	23810454	23813166	MKRN3	0	0
chr15.fa	23888696	23892993	MAGEL2	0	0
chr15.fa	23930554	23932450	NDN	2.413467113	304
chr15.fa	24409926	24415053	PWRN2	0	0
chr15.fa	24803304	24832926	PWRN1	0	0
chr15.fa	24920541	24928593	C15orf2	0	0
chr15.fa	25068794	25165271	SNRPN	0.497452847	22
chr15.fa	25200070	25213976	SNURF	0	0
chr15.fa	25227216	25228937	PAR-SN	0	0
chr15.fa	25230007	25233379	PAR5	0.353623762	76
chr15.fa	25296623	25296719	SNORD116-1	0	0
chr15.fa	25299356	25299452	SNORD116-2	0	0
chr15.fa	25304684	25304781	SNORD116-4	0	0
chr15.fa	25315578	25315674	SNORD116-8	0	0
chr15.fa	25319260	25319363	SNORD116-10	0	0
chr15.fa	25321075	25321168	SNORD116-11	0	0
chr15.fa	25322197	25322290	SNORD116-12	0	0
chr15.fa	25324204	25324297	SNORD116-13	0	0
chr15.fa	25325288	25325381	SNORD116-14	0	0
chr15.fa	25326433	25326526	SNORD116-15	0	0
chr15.fa	25327914	25328007	SNORD116-16	0	0
chr15.fa	25330531	25330624	SNORD116-18	0	0
chr15.fa	25332808	25332901	SNORD116-20	0	0
chr15.fa	25333950	25334043	SNORD116-21	0	0
chr15.fa	25335069	25335162	SNORD116-22	0	0
chr15.fa	25336932	25337025	SNORD116-23	0	0
chr15.fa	25339183	25339276	SNORD116-24	0	0
chr15.fa	25342809	25342902	SNORD116-25	0	0
chr15.fa	25344645	25344742	SNORD116-26	0	0
chr15.fa	25346721	25346814	SNORD116-27	0	0
chr15.fa	25349788	25349880	SNORD116-28	0	0
chr15.fa	25351667	25351751	SNORD116-29	0	0
chr15.fa	25361692	25367623	IPW	0	0
chr15.fa	25380789	25383200	PAR1	0	0
chr15.fa	25415870	25415951	SNORD115-1	0	0
chr15.fa	25417782	25417863	SNORD115-2	0	0
chr15.fa	25420074	25420155	SNORD115-3	0	0
chr15.fa	25421979	25422060	SNORD115-4	0	0

chr15.fa	25423885	25423887	SNORD115-5	0	0
chr15.fa	25425644	25425725	SNORD115-6	0	0
chr15.fa	25427532	25427613	SNORD115-7	0	0
chr15.fa	25429453	25429534	SNORD115-8	0	0
chr15.fa	25432683	25432763	SNORD115-10	0	0
chr15.fa	25438468	25438549	SNORD115-13	0	0
chr15.fa	25440068	25440148	SNORD115-14	0	0
chr15.fa	25444595	25444676	SNORD115-16	0	0
chr15.fa	25451434	25451490	SNORD115-20	0	0
chr15.fa	25455065	25455146	SNORD115-22	0	0
chr15.fa	25456839	25457180	PAR4	0	0
chr15.fa	25458806	25458876	SNORD115-24	0	0
chr15.fa	25460688	25460769	SNORD115-25	0	0
chr15.fa	25463764	25463845	SNORD115-26	0	0
chr15.fa	25465650	25465725	HBII-52-27	0	0
chr15.fa	25467501	25467574	HBII-52-28	0	0
chr15.fa	25470350	25470431	SNORD115-30	0	0
chr15.fa	25472256	25472337	SNORD115-31	0	0
chr15.fa	25474114	25474195	SNORD115-32	0	0
chr15.fa	25475985	25476066	SNORD115-33	0	0
chr15.fa	25477534	25477557	SNORD115-34	0	0
chr15.fa	25477560	25477615	SNORD115-21	0	0
chr15.fa	25479394	25479475	SNORD115-35	0	0
chr15.fa	25483133	25483214	SNORD115-37	0	0
chr15.fa	25484985	25485066	SNORD115-38	0	0
chr15.fa	25486893	25486974	SNORD115-39	0	0
chr15.fa	25488761	25488842	SNORD115-40	0	0
chr15.fa	25490625	25490706	SNORD115-41	0	0
chr15.fa	25496006	25496087	SNORD115-44	0	0
chr15.fa	25509674	25509726	HBII-52-45	0	0
chr15.fa	25513664	25513696	HBII-52-46	0	0
chr15.fa	25514930	25515005	SNORD115-48	0	0
chr15.fa	25582396	25684128	UBE3A	24.00665572	8284
chr15.fa	25923860	26108349	ATP10A	0	0
chr15.fa	26788694	27018935	GABRB3	1.663748593	684
chr15.fa	27111866	27194357	GABRA5	0	0
chr15.fa	27216429	27778373	GABRG3	3.426897391	456
chr15.fa	28000023	28344458	OCA2	0.364768633	76
chr15.fa	28356188	28567295	HERC2	6.494899798	6612
chr15.fa	28764757	28771064	GOLGA8G	0	0
chr15.fa	28899588	28930410	HERC2P9	0	0
chr15.fa	28982729	29003508	WHAMML2	0.43630666	152
chr15.fa	29033389	29034538	LOC100289656	0	0

chr15.fa	29034980	29101720	LOC646278	1.635133382	228
chr15.fa	29213840	29410516	APBA2	0.314767318	76
chr15.fa	29412455	29862927	FAM189A1	2.377321584	743
chr15.fa	29560358	29562017	NDNL2	0	0
chr15.fa	29992357	30114706	TJP1	67.90314197	32300
chr15.fa	30395935	32713929	FAM7A3	0	0
chr15.fa	30427990	30439395	LOC653075	0	0
chr15.fa	30488239	30506743	DKFZP434L187	0	0
chr15.fa	30653443	30685864	CHRFAM7A	0	0
chr15.fa	30918879	30931013	ARHGAP11B	0	0
chr15.fa	31196055	31235310	FAN1	11.47093433	3956
chr15.fa	31231144	31283807	MTMR10	18.41057475	5221
chr15.fa	31293551	31393924	TRPM1	0	0
chr15.fa	31357235	31357344	MIR211	0	0
chr15.fa	31619083	31670102	KLF13	17.0007485	7676
chr15.fa	31775329	31947542	OTUD7A	0	0
chr15.fa	32322691	32462384	CHRNA7	0	0
chr15.fa	32812049	32825942	LOC100288615	6.573968143	684
chr15.fa	32907691	32931868	ARHGAP11A	1.362234636	532
chr15.fa	32933870	32989298	SCG5	0	0
chr15.fa	33010205	33026870	GREM1	0	0
chr15.fa	33057747	33360085	FMN1	3.503405428	2874
chr15.fa	33603177	34158303	RYR3	0	0
chr15.fa	34158428	34331303	AVEN	3.738050156	380
chr15.fa	34261089	34357287	CHRM5	0.376666537	76
chr15.fa	34376224	34394053	C15orf24	8.696011927	608
chr15.fa	34394274	34396591	PGBD4	2.468890258	380
chr15.fa	34432875	34502297	C15orf29	2.924173321	532
chr15.fa	34517245	34522196	TMEM85	28.95994869	1596
chr15.fa	34522353	34630265	SLC12A6	5.423184854	2867
chr15.fa	34633917	34635362	NOP10	70.60200816	2508
chr15.fa	34638066	34649931	C15orf55	0	0
chr15.fa	34651089	34659395	LPCAT4	2.16285811	274
chr15.fa	34671270	34729667	GOLGA8A	0.701675088	304
chr15.fa	34817484	34875771	GOLGA8B	0.33043038	143
chr15.fa	35044679	35046689	GJD2	0	0
chr15.fa	35080297	35087927	ACTC1	0	0
chr15.fa	35148552	35261995	AQR	16.29696492	5497
chr15.fa	35270542	35280497	ZNF770	13.02594513	4712
chr15.fa	35529527	35530264	LOC723972	0	0
chr15.fa	35663170	35838404	ATPBD4	0.847160843	228
chr15.fa	35664457	35664565	MIR3942	0	0
chr15.fa	36871812	37102449	C15orf41	3.164390485	532

chr15.fa	37091301	37110707	CSNK1A1P1	0.677276855	76
chr15.fa	37156644	37178734	LOC145845	0	0
chr15.fa	37183232	37393500	MEIS2	3.225687278	988
chr15.fa	38227457	38243623	TMCO5A	0	0
chr15.fa	38545052	38649450	SPRED1	1.735437226	836
chr15.fa	38746328	38777063	FAM98B	21.55433216	2204
chr15.fa	38780302	38857007	RASGRP1	1.594469662	532
chr15.fa	38988799	38992239	C15orf53	0	0
chr15.fa	39542885	39547048	C15orf54	0	0
chr15.fa	39873280	39889668	THBS1	6.128323888	2356
chr15.fa	39892232	40075039	FSIP1	0.406938417	76
chr15.fa	40092931	40213093	GPR176	3.251742181	608
chr15.fa	40226347	40327797	EIF2AK4	26.08833699	9548
chr15.fa	40327891	40331389	SRP14	179.7378615	13438
chr15.fa	40380092	40401075	BMF	5.042301339	1596
chr15.fa	40453210	40513337	BUB1B	0.641281933	152
chr15.fa	40509629	40569688	PAK6	4.325866827	1140
chr15.fa	40542868	40545110	C15orf56	1.07728738	152
chr15.fa	40573645	40574787	LOC100131244	0	0
chr15.fa	40580098	40600174	PLCB2	2.202618192	684
chr15.fa	40623653	40633168	C15orf52	13.49719246	4774
chr15.fa	40643234	40648634	PHGR1	0	0
chr15.fa	40650434	40663256	DISP2	0	0
chr15.fa	40674922	40686489	C15orf23	1.689050463	228
chr15.fa	40697686	40713512	IVD	35.76133246	11077
chr15.fa	40733411	40760441	BAHD1	9.862910098	2964
chr15.fa	40763160	40765357	CHST14	0.520796835	76
chr15.fa	40824083	40824749	MRPL42P5	1.716009004	76
chr15.fa	40845298	40857252	C15orf57	16.54064603	1900
chr15.fa	40861537	40866661	RPUSD2	3.111979467	380
chr15.fa	40886447	40954881	CASC5	0	0
chr15.fa	40987327	41024356	RAD51	0	0
chr15.fa	41028086	41047458	FAM82A2	9.661248166	1444
chr15.fa	41056285	41059911	GCHFR	16.05343442	760
chr15.fa	41060067	41072195	DNAJC17	3.781726004	228
chr15.fa	41062159	41064648	C15orf62	0	0
chr15.fa	41099274	41106767	ZFYVE19	10.0543308	1444
chr15.fa	41107643	41120907	PPP1R14D	2.622207545	152
chr15.fa	41136246	41149853	SPINT1	20.09480581	3361
chr15.fa	41164412	41166487	RHOV	26.85296554	3040
chr15.fa	41186628	41196173	VPS18	15.28097434	3952
chr15.fa	41221531	41231258	DLL4	0	0
chr15.fa	41245636	41248717	CHAC1	0.803183783	76

chr15.fa	41271079	41408340	INO80	20.59045138	8512
chr15.fa	41474931	41522895	EXD1	0	0
chr15.fa	41523437	41574083	CHP	167.9578829	35999
chr15.fa	41576201	41591795	LOC729082	20.54737796	2584
chr15.fa	41601466	41624819	OIP5	0	0
chr15.fa	41624926	41673248	NUSAP1	0.472904008	76
chr15.fa	41679551	41694642	NDUFAF1	12.56945721	1216
chr15.fa	41709302	41775761	RTF1	24.16418998	8056
chr15.fa	41786122	41795747	ITPKA	0	0
chr15.fa	41795840	41806085	LTK	0	0
chr15.fa	41809375	41836464	RPAP1	7.351398236	2280
chr15.fa	41851220	41871536	TYRO3	2.59419476	684
chr15.fa	41952610	42062141	MGA	16.53898936	13224
chr15.fa	41983783	41983876	MIR626	0	0
chr15.fa	42066632	42120053	MAPKBP1	4.420598235	2128
chr15.fa	42128979	42129785	JMJD7	16.74381403	756
chr15.fa	42131011	42131120	PLA2G4B	0	0
chr15.fa	42140347	42186275	SPTBN5	1.074275252	836
chr15.fa	42158693	42158749	MIR4310	0	0
chr15.fa	42191639	42264755	EHD4	46.6864693	8968
chr15.fa	42275952	42302445	PLA2G4E	0	0
chr15.fa	42359881	42386752	PLA2G4D	0.30678518	73
chr15.fa	42433332	42448839	PLA2G4F	4.916695625	1140
chr15.fa	42450899	42500502	VPS39	23.82517504	7644
chr15.fa	42491768	42491864	MIR627	0	0
chr15.fa	42502726	42565755	TMEM87A	7.541463478	2033
chr15.fa	42566366	42645864	GANC	3.301743496	912
chr15.fa	42646538	42704515	CAPN3	7.05485429	1899
chr15.fa	42705021	42749730	ZFP106	22.8385527	15862
chr15.fa	42787835	42825256	SNAP23	37.2892341	5712
chr15.fa	42834720	42841002	LRRC57	7.109373796	1140
chr15.fa	42841011	42862190	HAUS2	2.902184791	760
chr15.fa	42867857	43013196	STARD9	0	0
chr15.fa	43015760	43029417	CDAN1	5.323784648	1672
chr15.fa	43036542	43213007	TTBK2	6.715086314	2508
chr15.fa	43235098	43398286	UBR1	9.565311907	4919
chr15.fa	43425722	43477341	TMEM62	3.721634062	684
chr15.fa	43477466	43488229	CCNDBP1	17.60949945	3178
chr15.fa	43489426	43513323	EPB42	0	0
chr15.fa	43524793	43559055	TGM5	0	0
chr15.fa	43568479	43594453	TGM7	0	0
chr15.fa	43619974	43622820	LCMT2	5.628611946	1064
chr15.fa	43622872	43646096	ADAL	4.327975316	1140

chr15.fa	43650370	43662258	ZSCAN29	14.28968319	5320
chr15.fa	43663313	43698240	TUBGCP4	6.649421936	1355
chr15.fa	43699412	43802707	TP53BP1	16.74772979	7068
chr15.fa	43809806	43823818	MAP1A	0.556942364	380
chr15.fa	43825660	43882451	PPIP5K1	8.127322266	3192
chr15.fa	43885252	43891604	CKMT1B	0.643390422	76
chr15.fa	43891761	43910998	STRC	0	0
chr15.fa	43922772	43941039	CATSPER2	0.429228161	76
chr15.fa	43985084	43991420	CKMT1A	0.643390422	76
chr15.fa	44028146	44038496	CATSPER2P1	0	0
chr15.fa	44038590	44064797	PDIA3	6.386011391	1292
chr15.fa	44064805	44069293	ELL3	3.016946846	380
chr15.fa	44069503	44088287	SERF2	181.7434865	32920
chr15.fa	44088353	44092255	SERINC4	0	0
chr15.fa	44092619	44092692	C15orf63	0	0
chr15.fa	44096733	44116951	MFAP1	28.85106028	3952
chr15.fa	44119112	44160617	WDR76	0.284194224	76
chr15.fa	44165730	44487429	FRMD5	2.041168161	304
chr15.fa	44168024	44170369	LOC728758	0	0
chr15.fa	44580909	44707959	CASC4	26.42509284	6992
chr15.fa	44719579	44819429	CTDSPL2	7.746589357	2507
chr15.fa	44826703	44829098	LOC645212	4.129174905	380
chr15.fa	44829266	44854893	EIF3J	25.21587428	4102
chr15.fa	44855002	44955876	SPG11	33.08626207	16872
chr15.fa	44957930	44969086	PATL2	0	0
chr15.fa	45003685	45010357	B2M	186.9532622	12252
chr15.fa	45028560	45060025	TRIM69	1.204248552	152
chr15.fa	45248903	45271421	C15orf43	0	0
chr15.fa	45315302	45367287	SORD	229.8019799	44219
chr15.fa	45384852	45406359	DUOX2	0.541128695	228
chr15.fa	45406523	45410301	DUOXA2	0	0
chr15.fa	45409686	45422057	DUOXA1	1.414043228	152
chr15.fa	45422192	45457774	DUOX1	0.403173258	152
chr15.fa	45459412	45493373	SHF	0.915686743	152
chr15.fa	45544428	45568132	SLC28A2	0	0
chr15.fa	45653322	45670897	GATM	90.97754424	15120
chr15.fa	45670981	45672321	LOC145663	0	0
chr15.fa	45694519	45713616	SPATA5L1	6.657253467	1140
chr15.fa	45722763	45725647	C15orf48	0	0
chr15.fa	45774680	45815002	SLC30A4	27.28068764	7635
chr15.fa	45803334	45848928	HMGN2P46	42.66241764	5266
chr15.fa	45879417	45901909	PLDN	37.1758275	9728
chr15.fa	45927256	45983479	SQRDL	81.86073811	9088

chr15.fa	47476403	48066420	SEMA6D	1.576396897	684
chr15.fa	48413169	48431372	SLC24A5	0	0
chr15.fa	48431629	48470558	MYEF2	3.291502263	1079
chr15.fa	48483867	48495951	CTXN2	0	0
chr15.fa	48498498	48596275	SLC12A1	10.24304058	3268
chr15.fa	48623621	48635570	DUT	16.3248271	2092
chr15.fa	48700503	48937985	FBN1	0.097894142	76
chr15.fa	49030135	49103343	CEP152	1.62444033	608
chr15.fa	49115934	49255641	SHC4	0	0
chr15.fa	49170290	49172380	EID1	61.30854075	8512
chr15.fa	49280835	49338760	SECISBP2L	33.7415504	16077
chr15.fa	49417471	49447854	COPS2	11.69142206	3189
chr15.fa	49447976	49622002	GALK2	8.552935873	1748
chr15.fa	49448495	49450822	LOC100306975	0	0
chr15.fa	49623977	49913118	C15orf33	0.668240473	76
chr15.fa	49715375	49779523	FGF7	0	0
chr15.fa	49913226	49937333	DTWD1	2.497354863	456
chr15.fa	50150435	50411419	ATP8B4	1.209670381	456
chr15.fa	50474393	50528589	SLC27A2	5.744578852	912
chr15.fa	50534146	50558162	HDC	0	0
chr15.fa	50569389	50638592	GABPB1	6.851535687	1292
chr15.fa	50641135	50646370	FLJ10038	1.967371039	684
chr15.fa	50647077	50650503	LOC100129387	1.143403577	228
chr15.fa	50716579	50793277	USP8	28.02001432	10108
chr15.fa	50821994	50838902	USP50	0	0
chr15.fa	50849352	50979012	TRPM7	7.767071823	5365
chr15.fa	50999737	51057910	SPPL2A	4.57150582	608
chr15.fa	51200946	51298097	AP4E1	3.597233198	1596
chr15.fa	51236326	51238762	LOC100132724	0	0
chr15.fa	51348799	51397473	TNFAIP8L3	0.506941049	76
chr15.fa	51500254	51630795	CYP19A1	0	0
chr15.fa	51633713	51700209	GLDN	0	0
chr15.fa	51739921	51914967	DMXL2	10.80224204	7600
chr15.fa	51973550	52013223	SCG3	0	0
chr15.fa	52015261	52043650	LYSMD2	6.715086314	836
chr15.fa	52043758	52108558	TMOD2	0.498507092	304
chr15.fa	52121825	52204331	TMOD3	24.53076589	7600
chr15.fa	52230222	52263958	LEO1	19.18333604	2736
chr15.fa	52311411	52358462	MAPK6	13.54568772	3800
chr15.fa	52401822	52404972	BCL2L10	1.290395396	76
chr15.fa	52413123	52483565	GNB5	10.47120923	2204
chr15.fa	52484515	52587995	MYO5C	35.91449914	16633
chr15.fa	52569314	52569397	MIR1266	0	0

chr15.fa	52599480	52821247	MYO5A	10.34710958	8399
chr15.fa	52839432	52861213	ARPP19	25.94330306	8892
chr15.fa	52873518	52970820	KIAA1370	26.59979623	7448
chr15.fa	53049353	53082209	ONECUT1	0	0
chr15.fa	53805938	54051859	WDR72	11.11881664	5396
chr15.fa	54305101	54920806	UNC13C	3.234121235	1748
chr15.fa	55473512	55489231	RSL24D1	44.11863065	4315
chr15.fa	55495800	55582001	RAB27A	68.10826785	13300
chr15.fa	55611133	55647176	PIGB	4.91895472	599
chr15.fa	55647438	55700574	CCPG1	2.890889313	1216
chr15.fa	55665138	55665232	MIR628	0	0
chr15.fa	55700723	55710910	FLJ27352	7.610441196	380
chr15.fa	55709954	55800432	DYX1C1	0.965537452	152
chr15.fa	55838221	55881050	PYGO1	6.358902244	532
chr15.fa	55903739	56035177	PRTG	1.912098501	1520
chr15.fa	56119122	56285835	NEDD4	4.936425059	2493
chr15.fa	56382731	56535483	RFX7	9.36922241	4476
chr15.fa	56657644	56738072	TEX9	5.581170938	524
chr15.fa	56720929	56757335	MNS1	2.263161954	304
chr15.fa	56922374	57025787	ZNF280D	4.452225573	1787
chr15.fa	57178368	57210697	LOC145783	0	0
chr15.fa	57210833	57580714	TCF12	19.92477121	6509
chr15.fa	57592563	57599967	LOC283663	0	0
chr15.fa	57668705	57842921	CGNL1	55.18473506	26448
chr15.fa	57884102	57977562	GCOM1	34.46114764	5487
chr15.fa	57998901	58001556	GRINL1A	47.78152823	2849
chr15.fa	58245627	58357906	ALDH1A2	5.751807958	1292
chr15.fa	58430408	58478110	AQP9	0.378925632	76
chr15.fa	58724175	58861073	LIPC	0.713572991	76
chr15.fa	58888510	59042177	ADAM10	15.15657347	3952
chr15.fa	58983302	58985324	HSP90AB4P	0	0
chr15.fa	59063393	59149734	FAM63B	13.06675945	4332
chr15.fa	59171244	59225852	SLTM	41.01267544	11293
chr15.fa	59279865	59389253	RNF111	22.325738	8208
chr15.fa	59397320	59417244	CCNB2	0.756495807	76
chr15.fa	59428563	59665071	MYO1E	34.22906323	10716
chr15.fa	59463382	59463461	MIR2116	0	0
chr15.fa	59499042	59500709	LDHAL6B	0	0
chr15.fa	59730372	59815751	FAM81A	9.2413576	2128
chr15.fa	59903982	59912210	GCNT3	0.515073793	76
chr15.fa	59930261	59949737	GTF2A2	20.98202795	2204
chr15.fa	59955062	59981642	BNIP2	9.54979945	1596
chr15.fa	60296421	60298142	FOXB1	0.784508592	76

chr15.fa	60639350	60690185	ANXA2	116.6949377	14040
chr15.fa	60711808	60771344	NARG2	7.894635421	3695
chr15.fa	60780483	61521502	RORA	4.06832993	3116
chr15.fa	62144590	62352664	VPS13C	8.542393427	9677
chr15.fa	62359176	62363116	C2CD4A	0	0
chr15.fa	62455737	62457482	C2CD4B	0	0
chr15.fa	62929371	62937380	MGC15885	0	0
chr15.fa	62939510	63136829	TLN2	4.618193795	3572
chr15.fa	63116156	63116240	MIR190	0	0
chr15.fa	63334838	63364113	TPM1	35.54476049	7904
chr15.fa	63413999	63434260	LACTB	6.38480654	836
chr15.fa	63445539	63449741	RPS27L	89.75251201	6460
chr15.fa	63481728	63559973	RAB8B	12.1319457	3927
chr15.fa	63569749	63601325	APH1B	1.637844297	456
chr15.fa	63615730	63674075	CA12	5.607527053	1480
chr15.fa	63796810	63883663	USP3	33.8029978	4554
chr15.fa	63879909	63893026	LOC100130855	1.611036363	152
chr15.fa	63889552	63894620	FBXL22	0.75348368	76
chr15.fa	63900817	64126147	HERC1	17.00014607	17154
chr15.fa	64199235	64338521	DAPK2	8.200818175	1431
chr15.fa	64364761	64386207	FAM96A	52.9191634	3837
chr15.fa	64388177	64436433	SNX1	24.32488698	13278
chr15.fa	64443916	64449680	SNX22	8.269193468	1701
chr15.fa	64452303	64455354	PPIB	49.4470841	1681
chr15.fa	64457716	64648442	CSNK1G1	8.693602225	4712
chr15.fa	64657211	64673702	KIAA0101	0.770200987	76
chr15.fa	64680003	64747502	TRIP4	20.14962653	2736
chr15.fa	64791619	64978266	ZNF609	23.24549111	13300
chr15.fa	64979773	64995462	OAZ2	43.60988233	5548
chr15.fa	65032095	65067770	RBPMS2	8.610467507	1140
chr15.fa	65054586	65054714	MIR1272	0	0
chr15.fa	65107833	65117838	PIF1	0	0
chr15.fa	65134082	65160201	PLEKHO2	6.152119695	1520
chr15.fa	65204101	65251041	ANKDD1A	2.206835171	456
chr15.fa	65255363	65282251	SPG21	76.02112664	9424
chr15.fa	65293850	65321977	MTFMT	6.669301977	1216
chr15.fa	65337708	65345674	OSTBETA	1.30063663	76
chr15.fa	65345735	65360388	RASL12	0	0
chr15.fa	65369154	65372276	KBTBD13	0	0
chr15.fa	65385342	65398697	LOC390595	0	0
chr15.fa	65409717	65426174	PDCD7	5.219263826	988
chr15.fa	65442784	65477563	CLPX	38.94876572	6080
chr15.fa	65488337	65503840	CILP	0	0

chr15.fa	65550437	65579018	PARP16	9.033972626	1520
chr15.fa	65619465	65670378	IGDCC3	2.299909909	684
chr15.fa	65673825	65715410	IGDCC4	0	0
chr15.fa	65737998	65810035	DPP8	16.24500572	4636
chr15.fa	65822827	65870693	PTPLAD1	6.741593036	1436
chr15.fa	65871118	65903469	C15orf44	25.08725644	4256
chr15.fa	65914270	65948598	SLC24A1	4.564126107	1748
chr15.fa	65952957	66084631	DENND4A	9.826915175	4788
chr15.fa	66161796	66181792	RAB11A	133.4498966	21204
chr15.fa	66187634	66546075	MEGF11	0	0
chr15.fa	66332571	66332670	MIR4311	0	0
chr15.fa	66585633	66626236	DIS3L	13.71496928	3496
chr15.fa	66629008	66649054	TIPIN	2.682751307	228
chr15.fa	66639544	66639680	SCARNA14	0	0
chr15.fa	66679211	66783882	MAP2K1	34.86823667	5487
chr15.fa	66782666	66790146	SNAPC5	4.023298625	152
chr15.fa	66791653	66797184	RPL4	837.6742984	79648
chr15.fa	66793590	66793656	SNORD18C	0	0
chr15.fa	66794360	66794429	SNORD18B	0	0
chr15.fa	66795149	66795248	SNORD16	0	0
chr15.fa	66795583	66795652	SNORD18A	0	0
chr15.fa	66797431	66841822	ZWILCH	2.272499549	456
chr15.fa	66840537	66857835	LCTL	0	0
chr15.fa	66994674	67074337	SMAD6	3.98820734	836
chr15.fa	67358195	67487533	SMAD3	11.92486194	5244
chr15.fa	67493367	67547074	AAGAB	38.1094364	7199
chr15.fa	67547138	67794142	IQCH	1.203796732	380
chr15.fa	67813522	67819641	C15orf61	5.295018831	456
chr15.fa	67835021	68099452	MAP2K5	7.702311083	1209
chr15.fa	68117941	68126174	SKOR1	0	0
chr15.fa	68346572	68480404	PIAS1	24.57263446	3720
chr15.fa	68483043	68498448	CALML4	3.210325428	988
chr15.fa	68499330	68522080	CLN6	2.552627401	380
chr15.fa	68570141	68583640	FEM1B	22.36444384	3800
chr15.fa	68594042	68724492	ITGA11	0	0
chr15.fa	68871573	69020144	CORO2B	1.533474081	380
chr15.fa	69070875	69113261	ANP32A	80.76311887	13224
chr15.fa	69094189	69094264	MIR4312	0	0
chr15.fa	69096160	69099440	C15orf28	0	0
chr15.fa	69116303	69489862	MIR548H4	0	0
chr15.fa	69237938	69239150	SPESP1	0	0
chr15.fa	69267068	69349501	NOX5	0	0
chr15.fa	69373190	69388163	NCRNA00277	0	0

chr15.fa	69452973	69564544	GLCE	1.815710422	608
chr15.fa	69591294	69699976	PAQR5	4.719551883	1748
chr15.fa	69706688	69740764	KIF23	1.265394739	304
chr15.fa	69745159	69747884	RPLP1	2139.43415	72732
chr15.fa	69854059	69863779	LOC145837	12.08390227	1368
chr15.fa	70127573	70135306	C15orf50	0	0
chr15.fa	70340543	70390256	TLE3	21.1487492	7486
chr15.fa	70371711	70371807	MIR629	0	0
chr15.fa	70946893	71055850	UACA	12.16131394	5864
chr15.fa	71123889	71146498	LARP6	8.800984568	1368
chr15.fa	71173681	71184772	THAP10	1.118402919	152
chr15.fa	71184928	71342436	LRRC49	3.891969869	760
chr15.fa	71402583	71407839	CT62	0.625167051	76
chr15.fa	71433788	72075722	THSD4	2.252920721	1368
chr15.fa	72102894	72110597	NR2E3	0	0
chr15.fa	72118361	72410422	MYO9A	12.18556157	7023
chr15.fa	72406599	72433311	SENP8	2.992398008	456
chr15.fa	72452147	72490136	GRAMD2	5.21534806	1140
chr15.fa	72491370	72523684	PKM2	190.5666102	35948
chr15.fa	72533522	72563628	PARP6	16.39696755	2859
chr15.fa	72577068	72612525	CELF6	0	0
chr15.fa	72635778	72668453	HEXA	9.176145041	1444
chr15.fa	72668521	72671129	C15orf34	0	0
chr15.fa	72690668	72700708	TMEM202	0	0
chr15.fa	72766667	72878896	ARIH1	22.19365621	7738
chr15.fa	72879558	72879654	MIR630	0	0
chr15.fa	72947038	72959738	GOLGA6B	0	0
chr15.fa	72968123	72978490	HIGD2B	0	0
chr15.fa	72978526	73030817	BBS4	21.43655798	3547
chr15.fa	73043708	73076126	ADPGK	2.477474822	456
chr15.fa	73344825	73597547	NEO1	6.459356694	3040
chr15.fa	73612200	73661605	HCN4	0.158287297	76
chr15.fa	73735499	73852343	C15orf60	0	0
chr15.fa	73852354	73925753	NPTN	7.493420045	1216
chr15.fa	73976622	74006859	CD276	5.048325594	1140
chr15.fa	74032141	74043816	C15orf59	0	0
chr15.fa	74165968	74181556	TBC1D21	0	0
chr15.fa	74218789	74244478	LOXL1	0.484801912	76
chr15.fa	74275560	74284635	STOML1	1.728961152	228
chr15.fa	74287014	74340155	PML	7.463148164	3496
chr15.fa	74362198	74374891	GOLGA6A	0	0
chr15.fa	74418714	74421619	LOC283731	0	0
chr15.fa	74421715	74429143	ISLR2	0	0

chr15.fa	74466087	74469212	ISLR	0	0
chr15.fa	74471808	74502046	STRA6	0.428625735	152
chr15.fa	74528667	74628482	CCDC33	0	0
chr15.fa	74630103	74660081	CYP11A1	0	0
chr15.fa	74701630	74726299	SEMA7A	0.332087051	76
chr15.fa	74738318	74753529	UBL7	20.70973163	1976
chr15.fa	74833548	74890472	ARID3B	2.968752807	836
chr15.fa	74900713	74922542	CLK3	12.89823092	2356
chr15.fa	74922899	74988386	EDC3	15.7528241	4256
chr15.fa	75011883	75017877	CYP1A1	7.899906644	1368
chr15.fa	75041184	75048941	CYP1A2	0	0
chr15.fa	75074425	75095539	CSK	22.75707465	3356
chr15.fa	75105194	75118099	LMAN1L	2.637418789	304
chr15.fa	75118951	75124136	CPLX3	0.568840268	76
chr15.fa	75128459	75135552	ULK3	16.889601	2955
chr15.fa	75137197	75165670	SCAMP2	7.979426808	684
chr15.fa	75182410	75190565	MPI	29.08374713	3420
chr15.fa	75192328	75199462	C15orf17	37.60490506	8864
chr15.fa	75212617	75230495	COX5A	61.0260032	3116
chr15.fa	75247443	75249775	RPP25	6.378029253	988
chr15.fa	75287876	75313836	SCAMP5	15.19678537	3572
chr15.fa	75315927	75343067	PPCDC	3.610787772	532
chr15.fa	75494221	75504510	C15orf39	8.790743335	2584
chr15.fa	75550899	75565796	GOLGA6C	0	0
chr15.fa	75575182	75588148	GOLGA6D	0	0
chr15.fa	75628374	75632614	COMMD4	38.13187675	2342
chr15.fa	75639331	75647588	NEIL1	1.209971594	152
chr15.fa	75645952	75646026	MIR631	0	0
chr15.fa	75648133	75660941	MAN2C1	13.36661674	2888
chr15.fa	75661720	75748124	SIN3A	28.81581839	13376
chr15.fa	75759462	75871625	PTPN9	23.83391021	6232
chr15.fa	75890424	75918719	SNUPN	10.60374284	1368
chr15.fa	75931426	75932664	IMP3	56.35283808	4636
chr15.fa	75941348	75950968	SNX33	20.5342752	4408
chr15.fa	75966663	76005189	CSPG4	0	0
chr15.fa	76016319	76020027	ODF3L1	0	0
chr15.fa	76025203	76032502	DNM1P35	0	0
chr15.fa	76054556	76054656	MIR4313	0	0
chr15.fa	76135622	76193388	UBE2Q2	19.59373841	4612
chr15.fa	76196200	76227608	FBXO22	5.111429664	1308
chr15.fa	76225024	76225025	FBXO22OS	0	0
chr15.fa	76234328	76304785	NRG4	8.396907672	1216
chr15.fa	76352299	76497304	C15orf27	0	0

chr15.fa	76508629	76603810	ETFA	73.54576031	6612
chr15.fa	76551630	76552493	TYRO3P	0	0
chr15.fa	76629147	76634816	ISL2	3.81967881	456
chr15.fa	76640527	77176217	SCAPER	10.68446786	3572
chr15.fa	77223962	77242601	RCN2	5.68945692	836
chr15.fa	77287465	77329671	PSTPIP1	0	0
chr15.fa	77336360	77363570	TSPAN3	4.455990732	1140
chr15.fa	77400471	77577333	PEAK1	5.240499325	4028
chr15.fa	77516250	77517746	C15orf5	0	0
chr15.fa	77713243	77777945	HMG20A	13.84720167	3572
chr15.fa	77905369	77924709	LINGO1	0	0
chr15.fa	78206559	78219188	LOC645752	0	0
chr15.fa	78285575	78286567	LOC91450	0	0
chr15.fa	78287327	78369994	TBC1D2B	7.942227034	3172
chr15.fa	78384927	78396393	SH2D7	0	0
chr15.fa	78396991	78423878	CIB2	16.18024498	1662
chr15.fa	78441719	78462884	IDH3A	14.17341507	2508
chr15.fa	78463187	78527049	ACSBG1	0	0
chr15.fa	78556487	78574538	DNAJA4	70.60456847	15808
chr15.fa	78575578	78591940	WDR61	68.34246076	5400
chr15.fa	78632666	78640572	CRABP1	2.965288861	152
chr15.fa	78730518	78793798	IREB2	23.5501678	9956
chr15.fa	78799906	78829715	AGPHD1	5.246523579	456
chr15.fa	78832747	78841563	PSMA4	82.10351558	6242
chr15.fa	78857862	78887611	CHRNA5	0	0
chr15.fa	78885395	78913637	CHRNA3	0	0
chr15.fa	78916636	78933587	CHRNB4	0	0
chr15.fa	79044379	79045734	LOC646938	0	0
chr15.fa	79051545	79103773	ADAMTS7	0	0
chr15.fa	79165172	79190074	MORF4L1	81.32894701	10487
chr15.fa	79214092	79237420	CTSH	35.31388092	3482
chr15.fa	79252289	79383215	RASGRF1	0	0
chr15.fa	79502130	79502213	MIR184	0	0
chr15.fa	79575146	79590581	ANKRD34C	0	0
chr15.fa	79603491	79615189	TMED3	20.61620507	1900
chr15.fa	79724858	79764642	KIAA1024	0.339617369	152
chr15.fa	80189133	80189627	MTHFS	19.84555226	506
chr15.fa	80191182	80207598	ST20	0	0
chr15.fa	80215113	80217196	C15orf37	2.986524359	304
chr15.fa	80253232	80263643	BCL2A1	0	0
chr15.fa	80352021	80430710	ZFAND6	34.10631903	3800
chr15.fa	80445341	80478682	FAH	11.69880177	1124
chr15.fa	80555410	80634147	LOC283688	0	0

chr15.fa	80696692	80890277	ARNT2	5.934192275	2584
chr15.fa	80987652	81047962	FAM108C1	26.1901469	4104
chr15.fa	81071712	81243999	KIAA1199	0	0
chr15.fa	81134319	81134414	MIR549	0	0
chr15.fa	81268095	81282205	MESDC2	2.697661338	760
chr15.fa	81293295	81296345	MESDC1	5.627407095	1140
chr15.fa	81426644	81441516	C15orf26	0	0
chr15.fa	81489219	81605006	IL16	0.138859075	76
chr15.fa	81605105	81616524	STARD5	5.366406252	434
chr15.fa	81624760	81666418	TMC3	0	0
chr15.fa	82334128	82338361	MEX3B	0	0
chr15.fa	82422561	82555104	EFTUD1	14.28817713	3496
chr15.fa	82555152	82577267	FAM154B	0	0
chr15.fa	82633123	83018198	GOLGA6L10	0	0
chr15.fa	82647543	83084257	UBE2Q2P2	0	0
chr15.fa	82707817	83084729	UBE2Q2P3	0	0
chr15.fa	82722185	83108111	GOLGA6L9	0	0
chr15.fa	82753447	83145927	LOC440297	0	0
chr15.fa	82763613	83142538	LOC727849	0	0
chr15.fa	82769979	83146651	LOC80154	0	0
chr15.fa	83211951	83316728	CPEB1	0	0
chr15.fa	83328033	83378635	AP3B2	0.308893669	76
chr15.fa	83379223	83382745	LOC338963	0	0
chr15.fa	83394650	83408532	LOC283693	0	0
chr15.fa	83424697	83424823	SCARNA15	0	0
chr15.fa	83428024	83474806	FSD2	0	0
chr15.fa	83477973	83503613	WHAMM	9.401903993	2660
chr15.fa	83517736	83621473	HOMER2	119.0632229	15408
chr15.fa	83654995	83659423	FAM103A1	3.886548039	248
chr15.fa	83657715	83680393	C15orf40	3.087882448	359
chr15.fa	83685181	83736106	BTBD1	20.87434439	4560
chr15.fa	83776324	83806111	TM6SF1	0.599563967	76
chr15.fa	83806816	83876321	HDGFRP3	23.20542982	3040
chr15.fa	83924655	83953468	BNC1	0	0
chr15.fa	84116091	84287493	SH3GL3	0.676072004	76
chr15.fa	84322838	84708593	ADAMTSL3	0.624263412	304
chr15.fa	84748939	84795353	LOC648809	0	0
chr15.fa	84860600	84878025	LOC440300	0	0
chr15.fa	84867600	84898920	LOC388152	0.235096547	38
chr15.fa	85045806	85047737	DNM1P41	0	0
chr15.fa	85050250	85060078	GOLGA6L5	0.95439258	304
chr15.fa	85070427	85114026	UBE2Q2P1	2.661817021	380
chr15.fa	85144249	85166947	ZSCAN2	2.597809313	760

chr15.fa	85174691	85185694	SCAND2	3.553858563	1064
chr15.fa	85186012	85197521	WDR73	7.45667209	912
chr15.fa	85198360	85201802	NMB	1.11132442	76
chr15.fa	85212775	85259674	SEC11A	65.60037054	6024
chr15.fa	85291818	85349663	ZNF592	8.536670385	4636
chr15.fa	85359911	85416713	ALPK3	1.782426414	1292
chr15.fa	85427913	85489027	SLC28A1	2.350965469	532
chr15.fa	85525205	85682372	PDE8A	25.92266998	6742
chr15.fa	85923871	86292586	AKAP13	20.88036864	18603
chr15.fa	86302559	86338189	KLHL25	2.176713896	532
chr15.fa	86313727	86313809	MIR1276	0	0
chr15.fa	86685242	87572283	AGBL1	1.310727257	304
chr15.fa	88120160	88122917	NCRNA00052	0	0
chr15.fa	88419988	88799661	NTRK3	0.224704707	76
chr15.fa	89002709	89010633	MRPL46	11.44608428	760
chr15.fa	89010684	89021861	MRPS11	12.20107403	1216
chr15.fa	89055714	89089912	DET1	3.419668286	532
chr15.fa	89151338	89151428	MIR1179	0	0
chr15.fa	89155056	89155165	MIR7-2	0	0
chr15.fa	89164527	89175512	AEN	7.346578832	1520
chr15.fa	89182039	89198879	ISG20	2.394641317	152
chr15.fa	89346674	89418585	ACAN	0	0
chr15.fa	89420519	89438770	HAPLN3	0	0
chr15.fa	89441914	89456663	MFGE8	0	0
chr15.fa	89631381	89745591	ABHD2	10.02616741	6080
chr15.fa	89753098	89764922	RLBP1	0	0
chr15.fa	89787194	89860362	FANCI	3.711091616	1064
chr15.fa	89859536	89878026	POLG	10.82769452	2888
chr15.fa	89911248	89911337	MIR9-3	0	0
chr15.fa	89921273	89941718	LOC254559	0	0
chr15.fa	90014638	90039799	RHCG	2.363616404	304
chr15.fa	90048161	90067265	LOC283761	0	0
chr15.fa	90118818	90171200	C15orf42	0	0
chr15.fa	90171254	90198682	KIF7	1.524437699	456
chr15.fa	90207600	90222648	PLIN1	0.772309476	152
chr15.fa	90226287	90233958	PEX11A	22.69487422	1748
chr15.fa	90234028	90286869	WDR93	0	0
chr15.fa	90293098	90294540	MESP1	29.55092809	2280
chr15.fa	90319589	90321982	MESP2	0.710560864	76
chr15.fa	90328126	90358072	ANPEP	45.60285645	11052
chr15.fa	90420538	90437617	AP3S2	6.202874042	257
chr15.fa	90443832	90445035	C15orf38	20.17673567	1613
chr15.fa	90544752	90625432	ZNF710	11.21912048	3344

chr15.fa	90627212	90645708	IDH2	59.51165613	6840
chr15.fa	90728152	90772892	SEMA4B	4.645754761	1216
chr15.fa	90773477	90777279	CIB1	136.1778299	8834
chr15.fa	90777487	90785312	C15orf58	7.625050014	684
chr15.fa	90792764	90802312	TTLL13	0	0
chr15.fa	90808895	90815443	NGRN	130.6669921	11704
chr15.fa	90889763	90892679	GABARAPL3	0	0
chr15.fa	90895477	90904715	ZNF774	0.622757349	76
chr15.fa	90931473	91045475	IQGAP1	44.13323947	21128
chr15.fa	91073198	91188577	CRTC3	7.694931371	2642
chr15.fa	91260579	91358686	BLM	0.252717492	76
chr15.fa	91411885	91426687	FURIN	9.857940088	2736
chr15.fa	91427665	91439006	FES	2.039511491	380
chr15.fa	91447420	91465815	MAN2A2	1.643266126	684
chr15.fa	91473410	91497323	UNC45A	13.85864776	3724
chr15.fa	91474155	91475774	HDDC3	9.742575607	676
chr15.fa	91498106	91506355	RCCD1	2.481993013	456
chr15.fa	91509268	91537804	PRC1	0.367630154	76
chr15.fa	91541774	91565833	VPS33B	9.804474826	1718
chr15.fa	91643182	91844539	SV2B	1.173976671	912
chr15.fa	92396938	92715665	SLCO3A1	0.802430751	152
chr15.fa	92937140	93011958	ST8SIA2	0	0
chr15.fa	93014907	93044347	C15orf32	0.663119856	76
chr15.fa	93111048	93115493	LOC100144604	0	0
chr15.fa	93160679	93199031	FAM174B	10.4490701	1824
chr15.fa	93338714	93340338	ASB9P1	0	0
chr15.fa	93426073	93441977	LOC100507217	18.56118112	2508
chr15.fa	93443551	93571237	CHD2	54.53215765	34474
chr15.fa	93447629	93447705	MIR3175	0	0
chr15.fa	93586636	93632443	RGMA	0.294586064	76
chr15.fa	94841430	95027181	MCTP2	5.987808143	3420
chr15.fa	95822519	95870329	LOC400456	0	0
chr15.fa	95976322	96051076	LOC145820	0	0
chr15.fa	96869157	96883492	NR2F2	8.804749727	3572
chr15.fa	96876490	96876536	MIR1469	0	0
chr15.fa	97326679	97328845	SPATA8	0	0
chr15.fa	98285846	98417659	LOC91948	0	0
chr15.fa	98503933	98517068	ARRDC4	12.38330774	3344
chr15.fa	98980391	99057611	FAM169B	0	0
chr15.fa	99192761	99507759	IGF1R	16.69772848	12464
chr15.fa	99511459	99551024	PGPEP1L	0	0
chr15.fa	99645286	99675800	SYNM	0.933910114	456
chr15.fa	99676528	99789815	TTC23	2.104121625	532

chr15.fa	99791652	99926498	LRRC28	20.52403397	1976
chr15.fa	100106133	100256629	MEF2A	8.316634475	3268
chr15.fa	100267612	100273626	LYSMD4	1.985142591	380
chr15.fa	100330361	100347132	C15orf51	0	0
chr15.fa	100511643	100882183	ADAMTS17	0.361605899	152
chr15.fa	100884662	100890923	FLJ42289	0.362660144	76
chr15.fa	100940600	101084925	LASS3	0	0
chr15.fa	101109429	101142445	LINS	4.102517577	684
chr15.fa	101142755	101191904	ASB7	6.68827838	2204
chr15.fa	101420009	101456830	ALDH1A3	101.8520773	23636
chr15.fa	101459460	101610317	LRRK1	1.9499007	988
chr15.fa	101715928	101792137	CHSY1	2.767241481	836
chr15.fa	101811214	101817700	SELS	2.447052334	304
chr15.fa	101821715	101835460	SNRPA1	9.727514969	684
chr15.fa	101844133	102030187	PCSK6	0.855293587	380
chr15.fa	102182049	102192594	TM2D3	2.44057626	228
chr15.fa	102193955	102264645	TARSL2	7.626405472	1668
chr15.fa	102345923	102346861	OR4F6	0	0
chr15.fa	102358390	102359328	OR4F15	0	0
chr15.fa	102382322	102390527	GPCRLTM7	0	0
chr15.fa	102462345	102463262	OR4F4	0	0
chr15.fa	102495088	102496558	FAM138E	0	0
chr15.fa	102501016	102516760	WASH3P	0.913427647	152
chr15.fa	102516809	102519296	DDX11L9	0	0
chr16.fa	96979	103632	POLR3K	13.89087752	760
chr16.fa	103829	107669	SNRNP25	25.29524384	1824
chr16.fa	108058	122629	RHBDF1	1.147620556	228
chr16.fa	127018	135803	MPG	7.636345492	684
chr16.fa	135844	188672	NPRL3	9.844686727	1744
chr16.fa	202854	204504	HBZ	0	0
chr16.fa	215973	216767	HBM	0	0
chr16.fa	222846	223709	HBA2	0	0
chr16.fa	226679	227520	HBA1	0	0
chr16.fa	230333	231178	HBQ1	0	0
chr16.fa	238970	279449	LUC7L	8.557454064	1183
chr16.fa	284801	316119	ITFG3	6.203777681	1216
chr16.fa	318310	325914	RGS11	0.481789784	76
chr16.fa	330606	333003	ARHGDIG	0	0
chr16.fa	333118	337209	PDIA2	0	0
chr16.fa	337440	402676	AXIN1	5.629214371	1368
chr16.fa	417384	420569	MRPL28	14.05654453	1064
chr16.fa	420776	431950	TMEM8A	17.84534903	4332
chr16.fa	432241	442960	LOC100134368	1.45711665	152

chr16.fa	447192	450754	NME4	139.8081965	9348
chr16.fa	451858	462487	DECR2	19.5226522	2052
chr16.fa	475668	572481	RAB11FIP3	14.92524208	4408
chr16.fa	576847	577407	NCRNA00235	0	0
chr16.fa	577856	604636	SOLH	6.514478626	2052
chr16.fa	593277	593366	MIR3176	0	0
chr16.fa	610422	615528	C16orf11	1.033009106	152
chr16.fa	617032	619495	NHLRC4	2.799923064	380
chr16.fa	620004	634109	PIGQ	4.700575481	912
chr16.fa	639357	679273	RAB40C	9.756280786	1900
chr16.fa	681012	684116	WFIKKN1	0	0
chr16.fa	684429	686347	C16orf13	21.67662453	1209
chr16.fa	691849	698474	FAM195A	17.60934885	1064
chr16.fa	699363	717829	WDR90	0.206631943	76
chr16.fa	718133	724171	RHOT2	13.01540268	2151
chr16.fa	726075	728267	RHBDL1	0	0
chr16.fa	730115	731603	STUB1	28.76822678	1786
chr16.fa	731667	734439	JMJD8	9.989268844	912
chr16.fa	734702	740400	WDR24	4.339722613	912
chr16.fa	742500	755825	FBXL16	1.292202673	304
chr16.fa	765173	767480	METRN	5.240951144	380
chr16.fa	771158	772581	FAM173A	2.69630588	152
chr16.fa	772591	776473	CCDC78	0	0
chr16.fa	776958	779715	HAGHL	0	0
chr16.fa	779769	790997	NARFL	5.473939201	760
chr16.fa	810765	818865	MSLN	0	0
chr16.fa	819428	832926	MSLNL	0	0
chr16.fa	834974	838383	RPUSD1	5.025132212	684
chr16.fa	838622	848040	CHTF18	0	0
chr16.fa	848075	850733	GNG13	0	0
chr16.fa	855443	863861	PRR25	0	0
chr16.fa	903635	1031318	LMF1	0.244885961	76
chr16.fa	1031808	1036979	SOX8	0	0
chr16.fa	1114082	1128731	LOC146336	0	0
chr16.fa	1122756	1131454	SSTR5	0	0
chr16.fa	1138226	1146244	C1QTNF8	0	0
chr16.fa	1203241	1271650	CACNA1H	2.155327792	1140
chr16.fa	1271773	1275254	TPSG1	0	0
chr16.fa	1278336	1280185	TPSB2	0	0
chr16.fa	1290678	1292555	TPSAB1	0	0
chr16.fa	1306273	1308494	TPSD1	0	0
chr16.fa	1359180	1375390	UBE2I	37.88699079	4103
chr16.fa	1383606	1399240	BAIAP3	1.897037863	608

chr16.fa	1399443	1401873	C16orf42	19.41948683	1292
chr16.fa	1401900	1413205	GNPTG	11.46686796	836
chr16.fa	1413353	1464705	UNKL	3.891969869	1520
chr16.fa	1469944	1479345	C16orf91	1.068702817	76
chr16.fa	1484389	1494490	CCDC154	1.044756403	152
chr16.fa	1494935	1525085	CLCN7	3.183216281	893
chr16.fa	1535940	1538468	PTX4	0	0
chr16.fa	1543352	1560427	TELO2	4.515932068	988
chr16.fa	1560461	1662109	IFT140	6.778039778	2356
chr16.fa	1584231	1605243	TMEM204	0	0
chr16.fa	1664641	1727909	CRAMP1L	11.93389832	6080
chr16.fa	1728278	1752073	HN1L	91.6927739	22423
chr16.fa	1756221	1820318	MAPK8IP3	6.70198356	2508
chr16.fa	1784986	1785067	MIR3177	0	0
chr16.fa	1820321	1821710	NME3	19.18966151	1292
chr16.fa	1821896	1823140	MRPS34	28.52966629	1900
chr16.fa	1823229	1826239	EME2	1.714804153	152
chr16.fa	1826713	1832581	SPSB3	17.79188377	1824
chr16.fa	1832933	1839192	NUBP2	16.56459244	1520
chr16.fa	1840414	1844909	IGFALS	0	0
chr16.fa	1859104	1877195	HAGH	29.14835726	3116
chr16.fa	1877225	1890203	FAHD1	9.809595442	1672
chr16.fa	1883984	1922179	C16orf73	0	0
chr16.fa	1928286	1934232	NCRNA00254	0	0
chr16.fa	1961465	1968231	HS3ST6	2.155629004	152
chr16.fa	1988234	1993294	SEPX1	32.65522664	2964
chr16.fa	1994580	2004679	RPL3L	0	0
chr16.fa	2009517	2011976	NDUFB10	55.83460155	2584
chr16.fa	2012062	2014827	RPS2	235.7260815	14744
chr16.fa	2012335	2012467	SNORA10	0	0
chr16.fa	2012974	2013107	SNORA64	0	0
chr16.fa	2014997	2015505	SNHG9	29.47487188	456
chr16.fa	2015185	2015311	SNORA78	0	0
chr16.fa	2016875	2018976	RNF151	0	0
chr16.fa	2022064	2028751	TBL3	9.562450386	1647
chr16.fa	2028918	2031184	NOXO1	0	0
chr16.fa	2034150	2037750	GFER	9.027647158	1444
chr16.fa	2039946	2044276	SYNGR3	0	0
chr16.fa	2047768	2059763	ZNF598	7.518571309	1596
chr16.fa	2069521	2070756	NPW	0	0
chr16.fa	2076888	2089027	SLC9A3R2	31.3330533	4525
chr16.fa	2089816	2097867	NTHL1	2.145538377	152
chr16.fa	2097990	2138710	TSC2	9.150391351	3420

chr16.fa	2138714	2185899	PKD1	1.457869682	1368
chr16.fa	2140196	2140285	MIR1225	0	0
chr16.fa	2185978	2186130	MIR3180-5	0	0
chr16.fa	2198651	2204141	RAB26	2.114362858	228
chr16.fa	2205024	2205106	SNORD60	0	0
chr16.fa	2205799	2227183	TRAF7	23.97517899	4330
chr16.fa	2228131	2246465	CASKIN1	0.241120802	76
chr16.fa	2255178	2259253	MLST8	16.3343153	2052
chr16.fa	2259419	2261069	C16orf79	0	0
chr16.fa	2261603	2264822	PGP	5.47484284	1140
chr16.fa	2273567	2285743	E4F1	5.33824286	912
chr16.fa	2286468	2288712	DNASE1L2	0	0
chr16.fa	2289873	2301602	DCI	40.29548789	2812
chr16.fa	2303100	2318413	RNPS1	34.0629444	5548
chr16.fa	2320714	2320773	MIR3677	0	0
chr16.fa	2321748	2321841	MIR940	0	0
chr16.fa	2325879	2390747	ABCA3	1.209820987	532
chr16.fa	2390923	2476700	ABCA17P	0	0
chr16.fa	2479395	2508859	CCNF	5.606924628	1596
chr16.fa	2510115	2514964	C16orf59	0	0
chr16.fa	2521500	2524146	NTN3	0	0
chr16.fa	2525147	2555734	TBC1D24	5.900908266	2584
chr16.fa	2563727	2570224	ATP6V0C	31.61393418	2584
chr16.fa	2570363	2579732	AMDHD2	4.152368286	437
chr16.fa	2580036	2581409	CEMP1	1.693267442	76
chr16.fa	2581923	2582006	MIR3178	0	0
chr16.fa	2587970	2653189	PDPK1	20.20986908	9710
chr16.fa	2653385	2680495	LOC652276	0.421697842	76
chr16.fa	2688983	2696130	FLJ42627	0.320189147	152
chr16.fa	2732495	2759031	KCTD5	9.759142308	1596
chr16.fa	2762423	2770552	PRSS27	1.565854451	152
chr16.fa	2787077	2799660	LOC100128788	0	0
chr16.fa	2802330	2821413	SRRM2	43.31770596	26444
chr16.fa	2821415	2827297	TCEB2	56.65239415	3724
chr16.fa	2833954	2836708	PRSS33	0	0
chr16.fa	2848486	2855133	PRSS41	0	0
chr16.fa	2867164	2871718	PRSS21	0	0
chr16.fa	2880173	2882285	ZG16B	105.0607461	5776
chr16.fa	2889574	2892752	PRSS30P	0	0
chr16.fa	2902728	2908171	PRSS22	2.477474822	228
chr16.fa	2933196	2949383	FLYWCH2	19.02083177	1292
chr16.fa	2961980	3001209	FLYWCH1	3.395119447	1140
chr16.fa	3014217	3018381	KREMEN2	1.114487154	152

chr16.fa	3019342	3023485	PAQR4	2.445696877	380
chr16.fa	3024001	3030540	PKMYT1	0	0
chr16.fa	3039055	3044510	LOC283875	0	0
chr16.fa	3062457	3064506	CLDN9	0	0
chr16.fa	3064713	3068188	CLDN6	0	0
chr16.fa	3070313	3072383	TNFRSF12A	3.327346579	228
chr16.fa	3072626	3074287	HCFC1R1	15.9111114	898
chr16.fa	3074288	3077756	THOC6	7.866773242	608
chr16.fa	3077868	3085542	CCDC64B	22.75315888	2736
chr16.fa	3096682	3110724	MMP25	0	0
chr16.fa	3115313	3119668	IL32	1.918875787	152
chr16.fa	3138895	3142861	ZSCAN10	0	0
chr16.fa	3160461	3165599	MGC3771	1.138885386	76
chr16.fa	3162563	3170518	ZNF205	3.565304647	437
chr16.fa	3185057	3192805	ZNF213	5.47981285	1216
chr16.fa	3254247	3255185	OR1F1	0	0
chr16.fa	3265562	3266546	OR1F2P	0	0
chr16.fa	3272325	3285457	ZNF200	4.380687546	1064
chr16.fa	3292028	3306627	MEFV	0	0
chr16.fa	3313768	3317566	FLJ39639	0.46868703	76
chr16.fa	3333487	3341459	ZNF263	11.14984155	2432
chr16.fa	3348808	3355432	TIGD7	1.671881337	380
chr16.fa	3355440	3368576	ZNF75A	7.028799387	988
chr16.fa	3405889	3406924	OR2C1	0	0
chr16.fa	3421053	3422283	MTRNR2L4	0	0
chr16.fa	3432085	3451025	ZNF434	7.562698976	1520
chr16.fa	3451190	3459364	ZNF174	3.496326929	608
chr16.fa	3486110	3493490	ZNF597	2.582296857	304
chr16.fa	3493668	3536963	NAT15	15.56471674	2964
chr16.fa	3543484	3545421	C16orf90	0	0
chr16.fa	3550963	3586585	CLUAP1	10.54334969	1216
chr16.fa	3589036	3627392	NLRC3	0.894300638	380
chr16.fa	3631184	3661585	SLX4	2.9827592	1444
chr16.fa	3702940	3708037	DNASE1	2.63651515	532
chr16.fa	3708097	3767598	TRAP1	28.12167362	4177
chr16.fa	3775056	3930121	CREBBP	26.54512612	17971
chr16.fa	4012650	4166186	ADCY9	1.185121542	608
chr16.fa	4239375	4292081	SRL	0	0
chr16.fa	4307187	4323001	TFAP4	4.813831472	684
chr16.fa	4382225	4389598	GLIS2	5.251945409	1292
chr16.fa	4390252	4401300	PAM16	4.479937145	152
chr16.fa	4404543	4466639	CORO7	2.254426784	526
chr16.fa	4421849	4433529	VASN	0.815834718	152

chr16.fa	4475858	4506775	DNAJA3	24.0747298	4332
chr16.fa	4511695	4524896	NMRAL1	27.87196826	2280
chr16.fa	4526341	4560348	HMOX2	18.70832355	2508
chr16.fa	4560677	4588816	C16orf5	12.29053421	2356
chr16.fa	4606491	4650318	LOC342346	0	0
chr16.fa	4658884	4664927	FAM100A	10.53069875	988
chr16.fa	4674826	4740975	MGRN1	8.485464218	3647
chr16.fa	4743694	4745860	NUDT16L1	22.04425469	1976
chr16.fa	4746513	4784163	ANKS3	2.579887155	456
chr16.fa	4784289	4799397	C16orf71	0.423053299	76
chr16.fa	4800815	4817166	ZNF500	5.978922367	1291
chr16.fa	4827672	4838397	12-Sep	0	0
chr16.fa	4838400	4846282	LOC440335	23.09744505	1368
chr16.fa	4846969	4852674	ROGDI	13.34598367	1292
chr16.fa	4853204	4897303	GLYR1	40.90423885	10136
chr16.fa	4897912	4932363	UBN1	17.80513713	8012
chr16.fa	4932508	4987136	PPL	115.494605	47837
chr16.fa	5008318	5069156	SEC14L5	0.3545274	152
chr16.fa	5074845	5083942	NAGPA	3.62705326	532
chr16.fa	5094123	5116111	C16orf89	3.780069334	380
chr16.fa	5121810	5137380	ALG1	0.775773423	152
chr16.fa	5134301	5147789	FAM86A	2.338615746	216
chr16.fa	6069132	7763340	RBFOX1	0	0
chr16.fa	8619502	8622226	TMEM114	0	0
chr16.fa	8715527	8740079	C16orf68	2.916191184	304
chr16.fa	8768444	8878432	ABAT	42.20020668	16616
chr16.fa	8889037	8891505	TMEM186	6.323810959	608
chr16.fa	8891670	8943194	PMM2	12.31824578	1900
chr16.fa	8946802	8962863	CARHSP1	7.870538401	1596
chr16.fa	8985951	9057341	USP7	70.98424713	25508
chr16.fa	9185537	9213555	C16orf72	21.28700585	5624
chr16.fa	9328774	9328803	MIR548X	0	0
chr16.fa	9847265	10276611	GRIN2A	0	0
chr16.fa	10522725	10577495	ATF7IP2	4.454936488	1064
chr16.fa	10622279	10674539	EMP2	6.185855522	2128
chr16.fa	10721361	10788802	TEKT5	4.268335192	456
chr16.fa	10837698	10863208	NUBP1	21.37149602	1551
chr16.fa	10860533	10912621	FAM18A	0	0
chr16.fa	10971055	11018840	CIITA	0	0
chr16.fa	11022748	11036257	DEXI	19.82672647	1976
chr16.fa	11038345	11276046	CLEC16A	7.640713077	3496
chr16.fa	11348274	11350039	SOCS1	0	0
chr16.fa	11361714	11363160	TNP2	0	0

chr16.fa	11367144	11367452	PRM3	0	0
chr16.fa	11369493	11370337	PRM2	0	0
chr16.fa	11374693	11375192	PRM1	0	0
chr16.fa	11439311	11445617	C16orf75	3.197222674	304
chr16.fa	11641578	11681322	LITAF	112.6613979	21888
chr16.fa	11762289	11772942	SNN	13.15230387	2812
chr16.fa	11773016	11836648	TXNDC11	12.31583608	2432
chr16.fa	11844442	11891114	ZC3H7A	23.89731549	6055
chr16.fa	11913692	11922689	BCAR4	0	0
chr16.fa	11928055	11945442	RSL1D1	28.7545216	9804
chr16.fa	11961985	12010519	GSPT1	37.86470105	19545
chr16.fa	12058964	12061925	TNFRSF17	0	0
chr16.fa	12070602	12147141	RUNDC2A	11.32770767	1672
chr16.fa	12155385	12668146	SNX29	14.76273781	6840
chr16.fa	12753656	12897744	CPPED1	6.894157291	2812
chr16.fa	12995477	13334273	SHISA9	1.5032022	988
chr16.fa	14014014	14046205	ERCC4	4.909617125	2204
chr16.fa	14165196	14360630	MKL2	13.2569753	7600
chr16.fa	14397824	14397906	MIR193B	0	0
chr16.fa	14403142	14403228	MIR365-1	0	0
chr16.fa	14529558	14724124	PARN	15.19768901	3106
chr16.fa	14726668	14763093	BFAR	8.275217723	1672
chr16.fa	14766405	14788526	PLA2G10	0	0
chr16.fa	14916289	14918559	ABCC6P2	0	0
chr16.fa	14927643	14990014	NOMO1	1.886495417	532
chr16.fa	15031300	15045931	NPIP	5.16594917	378
chr16.fa	15068833	15131552	PDXDC1	89.93579997	23952
chr16.fa	15131715	15149833	NTAN1	66.5731371	4911
chr16.fa	15153879	15188158	RRN3	13.74990996	3420
chr16.fa	15248707	15248859	MIR3180-4	0	0
chr16.fa	15489611	15503543	MPV17L	1.982883495	304
chr16.fa	15528325	15682116	C16orf45	0.977435355	152
chr16.fa	15688226	15737023	KIAA0430	30.09024951	15504
chr16.fa	15737124	15820208	NDE1	1.660284646	380
chr16.fa	15796992	15950887	MYH11	0	0
chr16.fa	15959577	15982447	C16orf63	13.73530114	2052
chr16.fa	16043434	16236930	ABCC1	4.178724401	1821
chr16.fa	16243422	16317328	ABCC6	0.615528243	228
chr16.fa	16326389	16388667	NOMO3	0.265217821	76
chr16.fa	16411466	16444465	PKD1P1	3.45475957	1444
chr16.fa	17196181	17564738	XYLT1	3.515152725	2179
chr16.fa	18511182	18573434	NOMO2	2.042975438	532
chr16.fa	18582570	18609607	ABCC6P1	0.420191778	76

chr16.fa	18794277	18801656	RPS15A	868.4690846	31312
chr16.fa	18802991	18812857	ARL6IP1	30.83966682	4636
chr16.fa	18816175	18937726	SMG1	14.95852609	15956
chr16.fa	18995256	19075262	TMC7	3.169962921	988
chr16.fa	19078917	19091417	COQ7	18.2937042	3344
chr16.fa	19125254	19132952	ITPRIPL2	11.44759035	5852
chr16.fa	19179638	19278554	SYT17	14.96605641	1900
chr16.fa	19297105	19315716	LOC728276	0	0
chr16.fa	19422057	19510434	TMC5	9.762154435	3554
chr16.fa	19513015	19533450	GDE1	45.20751472	8816
chr16.fa	19535179	19564728	CP110	2.868147751	1064
chr16.fa	19566737	19712485	C16orf62	21.61246622	5588
chr16.fa	19717674	19726359	C16orf88	13.42776293	1727
chr16.fa	19727778	19868859	IQCK	9.894085617	1668
chr16.fa	19870293	19896151	GPRC5B	3.218608779	608
chr16.fa	20043043	20085100	GPR139	0	0
chr16.fa	20321811	20338835	GP2	4.708407012	760
chr16.fa	20344373	20364037	UMOD	10.38310451	1596
chr16.fa	20370492	20416033	PDILT	0	0
chr16.fa	20420856	20452281	ACSM5	0.473205221	76
chr16.fa	20462859	20498991	ACSM2A	8.109550714	1596
chr16.fa	20548083	20587695	ACSM2B	10.78492231	1748
chr16.fa	20634559	20702578	ACSM1	2.232287648	304
chr16.fa	20744989	20753199	THUMPD1	29.82774261	8740
chr16.fa	20775312	20803657	ACSM3	39.96927449	4984
chr16.fa	20791515	20817766	ERI2	3.040140228	760
chr16.fa	20817796	20860990	LOC81691	8.750230221	1596
chr16.fa	20869396	20911556	DCUN1D3	5.597135214	1064
chr16.fa	20911562	20936328	LYRM1	19.63199243	2508
chr16.fa	20944476	21170762	DNAH3	0.092622919	76
chr16.fa	21169912	21191937	TMEM159	31.81137913	4028
chr16.fa	21208773	21222868	ZP2	0	0
chr16.fa	21245016	21263750	ANKS4B	3.150986518	608
chr16.fa	21269839	21314404	CRYM	69.92292403	6992
chr16.fa	21312170	21329912	NCRNA00169	1.490400659	228
chr16.fa	21413455	21436658	NPIPL3	0.311453978	75
chr16.fa	21443345	21445776	LOC100190986	0	0
chr16.fa	21458004	21513552	LOC100271836	0.641733752	211
chr16.fa	21529230	21531765	SLC7A5P2	0	0
chr16.fa	21610856	21668792	METTL9	64.84327231	13756
chr16.fa	21652606	21663972	IGSF6	0	0
chr16.fa	21689835	21772050	OTOA	0	0
chr16.fa	21807951	21830495	RRN3P1	1.422778397	152

chr16.fa	21964609	21994668	UQCRC2	111.9921032	12448
chr16.fa	21995186	22012431	PDZD9	0	0
chr16.fa	22019456	22095972	C16orf52	2.834411923	836
chr16.fa	22103863	22168287	VWA3A	0	0
chr16.fa	22217592	22300066	EEF2K	10.16367102	5002
chr16.fa	22308741	22345343	POLR3E	22.99277362	3977
chr16.fa	22357257	22385938	CDR2	21.60433348	3876
chr16.fa	22430867	22449036	RRN3P3	0.570346331	76
chr16.fa	22466023	22503541	LOC641298	0.253018705	76
chr16.fa	22524844	22547841	LOC100132247	0	0
chr16.fa	22557019	22588186	LOC653786	0	0
chr16.fa	22825860	22927659	HS3ST2	0	0
chr16.fa	23072728	23160591	USP31	10.91323894	7752
chr16.fa	23194040	23228200	SCNN1G	31.39540433	7294
chr16.fa	23313591	23392620	SCNN1B	5.28884397	912
chr16.fa	23399814	23464512	COG7	11.30195398	2204
chr16.fa	23474863	23521815	GGA2	14.64707211	5809
chr16.fa	23533334	23568696	EARS2	9.24467094	2432
chr16.fa	23568862	23585710	UBFD1	20.42704346	6688
chr16.fa	23592335	23607639	NDUFAB1	56.6298032	2508
chr16.fa	23614483	23652678	PALB2	6.454085471	1739
chr16.fa	23652687	23685068	DCTN5	15.88716498	8284
chr16.fa	23690201	23701625	PLK1	0	0
chr16.fa	23701689	23724821	ERN2	0.321845817	76
chr16.fa	23765948	23770256	CHP2	0.483747667	76
chr16.fa	23847300	24231932	PRKCB	0	0
chr16.fa	24266874	24373737	CACNG3	0	0
chr16.fa	24550908	24584183	RBBP6	8.973428864	4073
chr16.fa	24741049	24837547	TNRC6A	15.08202332	8434
chr16.fa	24857552	24922944	SLC5A11	0.974122015	152
chr16.fa	24930712	25026675	ARHGAP17	8.556550426	1976
chr16.fa	25123047	25189551	LCMT1	21.64559962	1969
chr16.fa	25228285	25240253	AQP8	0	0
chr16.fa	25247322	25268855	ZKSCAN2	3.854920701	1824
chr16.fa	25703347	26149009	HS3ST4	2.858810156	608
chr16.fa	27078219	27080487	C16orf82	0	0
chr16.fa	27214807	27233089	JMJD5	2.141923824	380
chr16.fa	27236315	27280113	NSMCE1	43.11137523	3040
chr16.fa	27279526	27301789	FLJ21408	1.904568182	76
chr16.fa	27325251	27376099	IL4R	0.296543947	76
chr16.fa	27413483	27463363	IL21R	0	0
chr16.fa	27458991	27464714	LOC283888	0	0
chr16.fa	27471934	27561251	GTF3C1	13.8124116	6518

chr16.fa	27561468	27791692	KIAA0556	9.332474455	4104
chr16.fa	27798850	28074830	GSG1L	1.368108284	456
chr16.fa	28109316	28223190	XPO6	23.29594425	6840
chr16.fa	28303840	28335170	SBK1	3.43939772	1140
chr16.fa	28390900	28747053	EIF3CL	0	0
chr16.fa	28415059	28722784	EIF3C	0	0
chr16.fa	28488600	28503623	CLN3	8.974934928	1140
chr16.fa	28505993	28510282	APOB48R	2.759560557	684
chr16.fa	28510683	28518155	IL27	0	0
chr16.fa	28548662	28550495	NUPR1	69.02787036	4180
chr16.fa	28565249	28603111	CCDC101	2.983361625	228
chr16.fa	28603264	28608391	SULT1A2	1.637543084	152
chr16.fa	28616916	28634866	SULT1A1	1.707575047	228
chr16.fa	28834414	28848558	ATXN2L	24.42985962	6370
chr16.fa	28853732	28857729	TUFM	45.32001768	6232
chr16.fa	28875078	28885534	SH2B1	5.866268801	1976
chr16.fa	28889809	28915741	ATP2A1	0	0
chr16.fa	28915831	28936532	RABEP2	17.97517173	2615
chr16.fa	28943260	28950668	CD19	0	0
chr16.fa	28962318	28977767	NFATC2IP	7.713757168	1976
chr16.fa	28986096	28995869	SPNS1	1.551697452	228
chr16.fa	28996147	29002104	LAT	0	0
chr16.fa	29086163	29128038	RRN3P2	0	0
chr16.fa	29302541	29385597	RUNDC2C	0.199854656	76
chr16.fa	29460666	30200575	LOC606724	0	0
chr16.fa	29476281	29476288	SLX1B-SULT1A4	0	0
chr16.fa	29538929	29577540	LOC440354	0.295489702	76
chr16.fa	29624424	29625038	SLC7A5P1	0	0
chr16.fa	29674300	29681823	SPN	0	0
chr16.fa	29690441	29709314	QPRT	36.82777618	3800
chr16.fa	29753786	29757340	C16orf54	0	0
chr16.fa	29789561	29791840	ZG16	0	0
chr16.fa	29802058	29816706	KIF22	43.687294	6080
chr16.fa	29817855	29822504	MAZ	193.8590161	35771
chr16.fa	29823409	29827202	PRRT2	0.891740329	152
chr16.fa	29827528	29833816	C16orf53	14.50821304	3572
chr16.fa	29841795	29859360	MVP	30.16419724	5624
chr16.fa	29869678	29874578	CDIPT	14.65881941	1824
chr16.fa	29875004	29879374	LOC440356	2.826128573	152
chr16.fa	29882480	29910580	SEZ6L2	0.966742303	228
chr16.fa	29912147	29917377	ASPHD1	1.548835931	152
chr16.fa	29917661	29937545	KCTD13	2.004570813	228
chr16.fa	29973351	29984373	TMEM219	98.53482139	6536

chr16.fa	29985222	30003581	TAOK2		8.954603068	3876
chr16.fa	30004311	30007412	HIRIP3		2.410756199	380
chr16.fa	30007531	30016834	INO80E		7.355916427	443
chr16.fa	30017113	30022401	DOC2A		0	0
chr16.fa	30034655	30035747	C16orf92		0	0
chr16.fa	30036024	30042186	FAM57B		0	0
chr16.fa	30064491	30081735	ALDOA		93.49267066	17301
chr16.fa	30087384	30096695	PPP4C		39.21880294	3466
chr16.fa	30097115	30103205	TBX6		3.811094247	456
chr16.fa	30103635	30107537	YPEL3		42.52792615	2660
chr16.fa	30116131	30124878	GDPD3		6.254682634	456
chr16.fa	30125426	30134630	MAPK3		23.9424974	2356
chr16.fa	30194731	30199897	CORO1A		0	0
chr16.fa	30234350	30256932	LOC613037		0.331334019	76
chr16.fa	30346296	30346695	LOC595101		2.86152107	76
chr16.fa	30362087	30366682	CD2BP2		12.23676774	2964
chr16.fa	30368422	30381522	TBC1D10B		11.83600418	2584
chr16.fa	30386123	30389310	MYLPF		0	0
chr16.fa	30389456	30394171		1-Sep	2.159695376	228
chr16.fa	30407057	30410715	ZNF48		2.597809313	380
chr16.fa	30418735	30429916	ZNF771		4.720003703	456
chr16.fa	30435019	30441373	DCTPP1		29.63722555	2204
chr16.fa	30454952	30457224	SEPHS2		71.00292232	10716
chr16.fa	30483983	30534506	ITGAL		0	0
chr16.fa	30535322	30537910	ZNF768		21.75493985	3344
chr16.fa	30542779	30546194	ZNF747		3.8619992	608
chr16.fa	30565085	30569642	ZNF764		0.409348119	76
chr16.fa	30581019	30583728	ZNF688		5.012029458	608
chr16.fa	30591994	30597092	ZNF785		2.103368593	456
chr16.fa	30614700	30621682	ZNF689		5.072121401	912
chr16.fa	30662241	30667734	PRR14		8.625377538	1213
chr16.fa	30675778	30682131	FBRS		20.68096581	3860
chr16.fa	30710462	30751450	SRCAP		29.28721634	20368
chr16.fa	30721858	30721986	SNORA30		0	0
chr16.fa	30759620	30772497	PHKG2		4.688677577	608
chr16.fa	30768744	30773565	C16orf93		1.463743331	152
chr16.fa	30773630	30786538	RNF40		24.88273298	6992
chr16.fa	30789770	30798523	ZNF629		10.53039754	4256
chr16.fa	30899116	30905399	BCL7C		28.06143107	1444
chr16.fa	30907928	30914881	CTF1		6.190825533	684
chr16.fa	30930640	30934590	NCRNA00095		1.158765427	304
chr16.fa	30935896	30960104	FBXL19		5.427552439	1368
chr16.fa	30960405	30966259	ORAI3		13.07820554	1900

chr16.fa	30968615	30995981	SETD1A	4.26095548	1824
chr16.fa	30996519	31000473	HSD3B7	6.738580908	1064
chr16.fa	31000577	31021829	STX1B	0	0
chr16.fa	31044903	31051485	STX4	12.38752471	1140
chr16.fa	31072164	31085641	ZNF668	0.87261332	228
chr16.fa	31085743	31094744	ZNF646	5.050584689	2052
chr16.fa	31094834	31100130	PRSS53	0	0
chr16.fa	31102175	31106276	VKORC1	8.028373879	532
chr16.fa	31119662	31124112	BCKDK	19.40427559	2801
chr16.fa	31128985	31142714	MYST1	10.04589684	1216
chr16.fa	31142754	31147151	PRSS8	2.950077617	380
chr16.fa	31150248	31161415	PRSS36	0.403323865	76
chr16.fa	31191431	31206192	FUS	14.71348953	5001
chr16.fa	31212807	31214097	PYCARD	13.32655544	684
chr16.fa	31225342	31236510	TRIM72	0	0
chr16.fa	31227283	31228395	PYDC1	0	0
chr16.fa	31271288	31344213	ITGAM	0	0
chr16.fa	31366509	31394318	ITGAX	0	0
chr16.fa	31404633	31437826	ITGAD	0	0
chr16.fa	31439055	31439721	COX6A2	0	0
chr16.fa	31446885	31454348	ZNF843	0	0
chr16.fa	31470317	31478488	ARMC5	1.959539508	608
chr16.fa	31483476	31489281	TGFB1I1	0	0
chr16.fa	31494439	31500659	SLC5A2	2.039059672	228
chr16.fa	31500813	31519706	C16orf58	6.320949438	912
chr16.fa	31539203	31540124	AHSP	0	0
chr16.fa	31579088	31580845	CSDAP1	0	0
chr16.fa	31711934	31718745	KIAA0664L3	1.933484605	228
chr16.fa	31724550	31772886	ZNF720	3.300990464	608
chr16.fa	31885079	31928627	ZNF267	4.205532335	912
chr16.fa	32162609	32163874	HERC2P4	0	0
chr16.fa	32300868	33298702	LOC390705	0	0
chr16.fa	32684841	33261514	TP53TG3B	0	0
chr16.fa	32888797	32896463	SLC6A10P	0	0
chr16.fa	34403802	34404762	UBE2MP1	0	0
chr16.fa	34597787	34626084	LOC283914	0	0
chr16.fa	34711785	34714967	LOC146481	0	0
chr16.fa	34739459	34740840	LOC100130700	0	0
chr16.fa	34980923	34990995	FLJ26245	0	0
chr16.fa	46503249	46603009	ANKRD26P1	0	0
chr16.fa	46614468	46655311	SHCBP1	0.708301768	152
chr16.fa	46693589	46723144	VPS35	48.08394582	10488
chr16.fa	46723558	46732306	ORC6	0	0

chr16.fa	46736194	46782221	MYLK3	0.429378767	228
chr16.fa	46835959	46865074	C16orf87	11.58675063	1064
chr16.fa	46918308	46965201	GPT2	28.26866544	7904
chr16.fa	46989274	47007625	DNAJA2	32.29542801	6536
chr16.fa	47115442	47177908	NETO2	0.626673114	152
chr16.fa	47189298	47495015	ITFG1	2.053066065	304
chr16.fa	47495210	47735434	PHKB	32.84559309	12418
chr16.fa	48116884	48180681	ABCC12	0	0
chr16.fa	48200822	48269088	ABCC11	0.750471552	228
chr16.fa	48278211	48387407	LONP2	57.46762644	16568
chr16.fa	48394446	48419229	SIAH1	12.82985563	2128
chr16.fa	48572637	48644120	N4BP1	21.10100698	9956
chr16.fa	49311829	49315742	CBLN1	0	0
chr16.fa	49407808	49433319	C16orf78	0	0
chr16.fa	49524521	49860918	ZNF423	0.237506249	76
chr16.fa	50059189	50070999	TMEM188	1.637543084	228
chr16.fa	50099881	50139375	HEATR3	10.53325906	1824
chr16.fa	50186829	50269219	PAPD5	4.652682654	2506
chr16.fa	50321823	50352043	ADCY7	0.372901377	152
chr16.fa	50352929	50402845	BRD7	25.63953	3936
chr16.fa	50582241	50668641	NKD1	0	0
chr16.fa	50700211	50715264	SNX20	0	0
chr16.fa	50731050	50766987	NOD2	0.510404995	152
chr16.fa	50775961	50835846	CYLD	10.9195644	6384
chr16.fa	51169886	51185183	SALL1	2.776127257	988
chr16.fa	52471918	52581714	TOX3	6.426524505	1444
chr16.fa	52585999	52640887	LOC643714	0	0
chr16.fa	53088945	53361414	CHD9	19.56888836	14893
chr16.fa	53468351	53525191	RBL2	53.46737059	16036
chr16.fa	53525561	53537170	AKTIP	12.97187744	1596
chr16.fa	53633818	53737771	RPGRIP1L	2.03664997	836
chr16.fa	53737875	54148379	FTO	16.80104445	4788
chr16.fa	54317212	54320378	IRX3	5.951210795	912
chr16.fa	54952778	54963079	CRNDE	1.747485736	152
chr16.fa	54965111	54968395	IRX5	2.775524832	380
chr16.fa	55358471	55364672	IRX6	0	0
chr16.fa	55513081	55540586	MMP2	0	0
chr16.fa	55542913	55620582	LPCAT2	0.212505591	76
chr16.fa	55600584	55601592	CAPNS2	4.537619386	304
chr16.fa	55689542	55740104	SLC6A2	0	0
chr16.fa	55758837	55784123	CES1P2	0	0
chr16.fa	55794511	55808826	CES1P1	0	0
chr16.fa	55836764	55867075	CES1	0	0

chr16.fa	55880066	55989943	CES5A	0	0
chr16.fa	56126899	56225006	LOC283856	0	0
chr16.fa	56225251	56391356	GNAO1	0	0
chr16.fa	56226529	56228437	DKFZP434H168	0	0
chr16.fa	56279432	56279535	MIR3935	0	0
chr16.fa	56395364	56459444	AMFR	11.46521129	2736
chr16.fa	56463048	56485261	NUDT21	17.65453076	5166
chr16.fa	56485424	56511407	OGFOD1	19.70850046	3952
chr16.fa	56518259	56554008	BBS2	21.96473452	4104
chr16.fa	56598961	56602869	MT4	0	0
chr16.fa	56623267	56625000	MT3	0	0
chr16.fa	56642478	56643409	MT2A	457.965513	12741
chr16.fa	56651373	56652730	MT1L	29.55559689	836
chr16.fa	56659585	56661024	MT1E	217.0993866	7294
chr16.fa	56666534	56667898	MT1M	49.42630042	1444
chr16.fa	56669651	56670998	MT1JP	2.805344894	76
chr16.fa	56672578	56673999	MT1A	17.34262496	456
chr16.fa	56677599	56678853	MT1DP	0	0
chr16.fa	56685811	56687116	MT1B	0	0
chr16.fa	56691855	56693215	MT1F	129.5692223	3794
chr16.fa	56700653	56701977	MT1G	458.398657	12053
chr16.fa	56703726	56705041	MT1H	63.42922908	1672
chr16.fa	56709649	56711653	MT1IP	0	0
chr16.fa	56716382	56718108	MT1X	203.9895538	5472
chr16.fa	56764017	56878677	NUP93	14.21874759	2584
chr16.fa	56892430	56892513	MIR138-2	0	0
chr16.fa	56899119	56949762	SLC12A3	5.371828081	1976
chr16.fa	56965748	56977793	HERPUD1	60.49165179	8740
chr16.fa	56995835	57017756	CETP	0	0
chr16.fa	57023410	57117436	NLRC5	0.671101994	304
chr16.fa	57126455	57181878	CPNE2	1.534377719	228
chr16.fa	57186378	57219976	FAM192A	18.04836642	3413
chr16.fa	57220241	57272947	RSPRY1	21.91036562	3010
chr16.fa	57279038	57287545	ARL2BP	13.62626212	1900
chr16.fa	57290009	57318584	PLLP	8.410612852	836
chr16.fa	57392695	57400102	CCL22	0	0
chr16.fa	57406414	57418956	CX3CL1	1.049123988	228
chr16.fa	57438679	57449974	CCL17	0	0
chr16.fa	57462087	57481336	CIAPIN1	16.02015041	2204
chr16.fa	57481370	57495187	COQ9	24.88273298	2736
chr16.fa	57496551	57505869	POLR2C	38.50266964	4479
chr16.fa	57505922	57520385	DOK4	12.30303454	2204
chr16.fa	57546090	57570477	CCDC102A	1.413742015	228

chr16.fa	57576601	57611100	GPR114	0	0
chr16.fa	57653910	57698944	GPR56	4.503130527	1350
chr16.fa	57702157	57723290	GPR97	0	0
chr16.fa	57728713	57765367	CCDC135	0	0
chr16.fa	57769660	57791162	KATNB1	3.460633219	608
chr16.fa	57792129	57836439	KIFC3	9.569227672	2356
chr16.fa	57916244	58005020	CNGB1	0	0
chr16.fa	58010339	58022017	TEPP	1.245062878	134
chr16.fa	58028573	58033762	ZNF319	9.221929378	2584
chr16.fa	58035277	58055527	C16orf57	17.01641156	2584
chr16.fa	58059282	58080804	MMP15	0.773966146	228
chr16.fa	58147497	58163296	C16orf80	17.73209304	1520
chr16.fa	58191812	58231782	CSNK2A2	31.43832715	3461
chr16.fa	58283840	58317734	CCDC113	4.065167196	1216
chr16.fa	58318494	58328951	PRSS54	0	0
chr16.fa	58426298	58440048	GINS3	0.957404708	152
chr16.fa	58497549	58547523	NDRG4	0.291724543	76
chr16.fa	58549383	58553854	SETD6	5.718222737	912
chr16.fa	58554432	58663750	CNOT1	72.73474499	39278
chr16.fa	58582403	58582537	SNORA46	0	0
chr16.fa	58593700	58593835	SNORA50	0	0
chr16.fa	58700298	58718674	SLC38A7	1.651097658	304
chr16.fa	58741035	58768246	GOT2	97.76175888	15884
chr16.fa	59788045	59789095	LOC644649	0	0
chr16.fa	61687235	62070036	CDH8	0.390823536	76
chr16.fa	64980683	65155919	CDH11	0	0
chr16.fa	65318402	65610203	LOC283867	0	0
chr16.fa	66400525	66438689	CDH5	0	0
chr16.fa	66460816	66527432	BEAN1	0.879541213	228
chr16.fa	66541906	66584315	TK2	7.162236633	2432
chr16.fa	66586466	66600154	CKLF	1.365849189	76
chr16.fa	66600294	66613038	CMTM1	1.023822118	76
chr16.fa	66613351	66622177	CMTM2	0	0
chr16.fa	66637935	66647795	CMTM3	0.882553341	152
chr16.fa	66648653	66730610	CMTM4	17.06264772	9196
chr16.fa	66754799	66785525	DYNC1LI2	48.62402027	13999
chr16.fa	66788879	66835523	CCDC79	0.467933998	76
chr16.fa	66836781	66864879	NAE1	24.15530421	3065
chr16.fa	66878282	66888049	CA7	0	0
chr16.fa	66914383	66925002	PDP2	10.58310977	4940
chr16.fa	66942025	66952766	CDH16	16.56504426	3116
chr16.fa	66955582	66959439	RRAD	0.77547221	76
chr16.fa	66965958	66968320	FAM96B	42.98697437	1978

chr16.fa	66968347	66978994	CES2	15.06425177	4156
chr16.fa	66995132	67009052	CES3	0.264916609	76
chr16.fa	67022492	67043659	CES4A	2.089663413	362
chr16.fa	67063050	67134958	CBFB	10.70073335	2204
chr16.fa	67143915	67182004	C16orf70	8.097502204	1292
chr16.fa	67182443	67184902	B3GNT9	2.52446401	380
chr16.fa	67188089	67193812	TRADD	13.902173	1368
chr16.fa	67193891	67198077	FBXL8	5.694577537	380
chr16.fa	67198344	67203848	HSF4	0.565526927	76
chr16.fa	67204405	67209504	NOL3	18.91375064	2204
chr16.fa	67209644	67217883	KIAA0895L	3.158968655	684
chr16.fa	67218282	67224107	EXOC3L	0.916740987	152
chr16.fa	67226068	67232821	E2F4	14.00639261	1953
chr16.fa	67233028	67237927	ELMO3	12.30273333	2052
chr16.fa	67236224	67236298	MIR328	0	0
chr16.fa	67241042	67260901	LRRC29	1.533323475	152
chr16.fa	67261016	67263182	TMEM208	7.207870364	380
chr16.fa	67263292	67281425	FHOD1	2.375966127	608
chr16.fa	67282855	67306094	SLC9A5	0	0
chr16.fa	67311413	67323392	PLEKHG4	0	0
chr16.fa	67323404	67360661	KCTD19	0	0
chr16.fa	67360747	67419109	LRRC36	0	0
chr16.fa	67423712	67427421	TPPP3	1.011171182	76
chr16.fa	67428322	67450339	ZDHHC1	1.139487812	152
chr16.fa	67465036	67471454	HSD11B2	17.0292131	2128
chr16.fa	67471917	67515089	ATP6V0D1	59.6292797	6612
chr16.fa	67516474	67517716	AGRP	0	0
chr16.fa	67562717	67580691	FAM65A	16.6676072	4958
chr16.fa	67596310	67673088	CTCF	32.81923698	8588
chr16.fa	67679030	67691414	RLTPR	0	0
chr16.fa	67691473	67694718	ACD	1.642362488	218
chr16.fa	67694851	67696681	PARD6A	1.812547689	152
chr16.fa	67696850	67700628	C16orf48	3.721031636	380
chr16.fa	67700717	67702661	C16orf86	0	0
chr16.fa	67708436	67753273	GFOD2	9.739864692	4408
chr16.fa	67757005	67840555	RANBP10	8.326574496	2933
chr16.fa	67841010	67861971	TSNAXIP1	0.467933998	76
chr16.fa	67862060	67881361	CENPT	4.065468409	633
chr16.fa	67876213	67878098	THAP11	12.74491364	1596
chr16.fa	67880819	67905219	NUTF2	74.92923044	4408
chr16.fa	67906999	67918406	EDC4	13.07564523	4104
chr16.fa	67918781	67920271	NRN1L	0	0
chr16.fa	67927175	67963473	PSKH1	22.3966736	5016

chr16.fa	67963582	67965778	CTRL	0	0
chr16.fa	67968407	67970753	PSMB10	27.8445579	1810
chr16.fa	67973787	67977115	LCAT	0	0
chr16.fa	67977377	68002597	SLC12A4	1.648236137	532
chr16.fa	68009566	68014452	DPEP3	0	0
chr16.fa	68021293	68033364	DPEP2	0	0
chr16.fa	68055177	68057770	DDX28	4.050859591	684
chr16.fa	68059318	68113184	DUS2L	1.831373485	228
chr16.fa	68119269	68262449	NFATC3	26.98067975	10412
chr16.fa	68263163	68270136	ESRP2	46.46131277	10205
chr16.fa	68279247	68294961	PLA2G15	1.681369538	304
chr16.fa	68298423	68335722	SLC7A6	3.91350658	1444
chr16.fa	68336284	68344868	SLC7A6OS	2.731698378	152
chr16.fa	68344877	68391169	PRMT7	13.0016975	2128
chr16.fa	68392230	68482409	SMPD3	0	0
chr16.fa	68573661	68601039	ZFP90	7.571584752	2204
chr16.fa	68678151	68732957	CDH3	0.267627523	76
chr16.fa	68771195	68869444	CDH1	20.91907448	6688
chr16.fa	68877509	69119085	TMCO7	4.513823579	1444
chr16.fa	69139467	69151570	HAS3	7.068559469	2128
chr16.fa	69151912	69166493	CHTF8	35.21207102	6156
chr16.fa	69166499	69202937	CIRH1A	29.45303395	4408
chr16.fa	69221050	69342955	SNTB2	12.30002241	7980
chr16.fa	69345287	69358946	VPS4A	39.97710602	5776
chr16.fa	69363900	69364498	PDF	0	0
chr16.fa	69364716	69373414	COG8	11.92305466	1444
chr16.fa	69373527	69377013	NIP7	9.335335976	1368
chr16.fa	69377149	69385712	TMED6	1.217652519	76
chr16.fa	69389464	69419874	TERF2	11.9429347	2352
chr16.fa	69458498	69500167	CYB5B	20.91124295	5951
chr16.fa	69598997	69738553	NFAT5	8.893005061	8360
chr16.fa	69743304	69760533	NQO1	37.54993373	6460
chr16.fa	69775774	69788829	NOB1	18.6765456	2128
chr16.fa	69796274	69975643	WWP2	28.53403387	9348
chr16.fa	69966984	69967083	MIR140	0	0
chr16.fa	69984626	69997889	CLEC18A	0.581340596	76
chr16.fa	70010202	70099851	PDXDC2P	3.468012931	963
chr16.fa	70147529	70195184	PDPR	5.008264299	2660
chr16.fa	70207928	70220798	CLEC18C	0	0
chr16.fa	70253484	70259936	LOC729513	0	0
chr16.fa	70284134	70285833	EXOSC6	6.733008472	760
chr16.fa	70286297	70323412	AARS	39.02075556	8664
chr16.fa	70333072	70367731	DDX19B	12.61283185	1520

chr16.fa	70380824	70407281	DDX19A	10.65675629	2052
chr16.fa	70413338	70472991	ST3GAL2	2.314970546	684
chr16.fa	70488498	70514177	FUK	5.273331514	1368
chr16.fa	70514472	70557457	COG4	19.46888573	3648
chr16.fa	70557691	70611571	SF3B3	20.13667438	12996
chr16.fa	70563402	70563502	MIR3647	0	0
chr16.fa	70571908	70572001	SNORD111	0	0
chr16.fa	70613798	70694585	IL34	0	0
chr16.fa	70695107	70719954	MTSS1L	7.819332235	2584
chr16.fa	70721342	70835061	VAC14	14.56890741	2933
chr16.fa	70788963	70807154	LOC100130894	0	0
chr16.fa	70841289	71264625	HYDIN	0.277416937	304
chr16.fa	71316203	71323509	FTSJD1	16.27015699	4560
chr16.fa	71392616	71424342	CALB2	0	0
chr16.fa	71481503	71496117	ZNF23	1.766913958	380
chr16.fa	71507976	71523254	ZNF19	3.338340845	608
chr16.fa	71560023	71572493	CHST4	0	0
chr16.fa	71600754	71610998	TAT	0	0
chr16.fa	71660070	71675868	MARVELD3	3.055050259	684
chr16.fa	71678852	71748704	PHLPP2	6.070641648	3192
chr16.fa	71732470	71732604	SNORA70D	0	0
chr16.fa	71762905	71842976	AP1G1	43.63322631	19834
chr16.fa	71792305	71792390	SNORD71	0	0
chr16.fa	71879899	71919174	ATXN1L	21.61803866	11476
chr16.fa	71893588	71917412	ZNF821	3.513194843	380
chr16.fa	71929446	71962906	KIAA0174	66.18607872	10323
chr16.fa	71963441	72033877	PKD1L3	0	0
chr16.fa	72042643	72058955	DHODH	2.216022159	304
chr16.fa	72088508	72094955	HP	0	0
chr16.fa	72097125	72111145	HPR	0	0
chr16.fa	72118756	72128215	TXNL4B	3.347979652	608
chr16.fa	72127769	72146811	DHX38	14.1079013	4028
chr16.fa	72152996	72206349	PMFBP1	0	0
chr16.fa	72816786	73092534	ZFHX3	32.84875582	35264
chr16.fa	73126248	73127672	HTA	0	0
chr16.fa	74330681	74340186	PSMD7	60.60505839	6688
chr16.fa	74366304	74402153	LOC283922	1.481966702	228
chr16.fa	74442529	74455368	CLEC18B	0.589021521	76
chr16.fa	74481326	74641042	GLG1	3.952061811	2204
chr16.fa	74655297	74700779	RFWD3	7.858791104	2584
chr16.fa	74705753	74734789	MLKL	1.804716157	304
chr16.fa	74746856	74808729	FA2H	0.943247709	152
chr16.fa	74907471	75019017	WDR59	14.57960046	3546

chr16.fa	75032915	75144892	ZNRF1	9.141053756	2812
chr16.fa	75145758	75150670	LDHD	18.78212067	2584
chr16.fa	75182421	75206132	ZFP1	2.436811101	532
chr16.fa	75237994	75241072	CTRB2	0	0
chr16.fa	75252884	75258822	CTRB1	0	0
chr16.fa	75262928	75301951	BCAR1	7.217509172	2584
chr16.fa	75327608	75467387	CFDP1	48.32597026	4104
chr16.fa	75480923	75498584	TMEM170A	15.20356266	1064
chr16.fa	75507022	75528926	CHST6	0	0
chr16.fa	75562428	75569068	CHST5	0	0
chr16.fa	75572015	75590170	TMEM231	0	0
chr16.fa	75600249	75611779	GABARAPL2	117.4189026	7812
chr16.fa	75632247	75657221	ADAT1	10.27692701	2419
chr16.fa	75661622	75681585	KARS	56.36925417	8208
chr16.fa	75681635	75691341	TERF2IP	33.10855182	4788
chr16.fa	76311176	76593135	CNTNAP4	0	0
chr16.fa	77224836	77233543	MON1B	19.0122472	4104
chr16.fa	77240338	77246976	SYCE1L	1.40274775	76
chr16.fa	77316025	77469011	ADAMTS18	0.193529188	76
chr16.fa	77756411	77776154	NUDT7	1.047166105	76
chr16.fa	77822483	78014001	VAT1L	0.303923659	76
chr16.fa	78056443	78065998	CLEC3A	2.957607936	380
chr16.fa	78133551	79246564	WWOX	8.333351783	1292
chr16.fa	79627745	79634622	MAF	17.80649259	8132
chr16.fa	80574854	80584541	DYNLRB2	2.303072643	76
chr16.fa	80637676	80838175	CDYL2	44.04302625	6536
chr16.fa	81009699	81031034	C16orf61	52.57924482	1976
chr16.fa	81040103	81066709	CENPN	19.91588544	6801
chr16.fa	81069458	81080951	ATMIN	21.64078022	6992
chr16.fa	81087102	81110872	C16orf46	2.056831224	304
chr16.fa	81115552	81115928	GCSH	3.036073856	76
chr16.fa	81134484	81253975	PKD1L2	0.140666352	76
chr16.fa	81272296	81324747	BCMO1	0.471548551	76
chr16.fa	81348571	81413803	GAN	21.15914104	6384
chr16.fa	81478775	81745367	CMIP	14.18440934	4241
chr16.fa	81812930	81991899	PLCG2	9.627361732	2711
chr16.fa	82031251	82045093	SDR42E1	6.187060373	1140
chr16.fa	82068858	82132139	HSD17B2	0	0
chr16.fa	82181767	82203829	MPHOSPH6	9.356420868	684
chr16.fa	82660578	83830199	CDH13	0.299104255	76
chr16.fa	83541951	83542013	MIR3182	0	0
chr16.fa	83841593	83846594	HSBP1	56.86565278	7140
chr16.fa	83932730	83949787	MLYCD	8.34344241	1216

chr16.fa	83982672	83999937	OSGIN1	0	0
chr16.fa	84002237	84036379	NECAB2	2.850828018	304
chr16.fa	84043389	84075762	SLC38A8	0	0
chr16.fa	84087369	84150517	MBTPS1	22.12317243	6384
chr16.fa	84155744	84178800	HSDL1	6.865090261	1672
chr16.fa	84178865	84211460	LRRC50	0	0
chr16.fa	84211525	84220662	TAF1C	2.847213465	684
chr16.fa	84224723	84230772	ADAD2	0	0
chr16.fa	84254741	84273356	KCNG4	0	0
chr16.fa	84328401	84363450	WFDC1	0	0
chr16.fa	84402133	84497793	ATP2C2	2.366929744	532
chr16.fa	84509966	84538288	KIAA1609	4.777685943	1596
chr16.fa	84599204	84651669	COTL1	82.16089661	9956
chr16.fa	84682131	84695916	KLHL36	16.88673947	2432
chr16.fa	84733555	84813527	USP10	29.43014178	6601
chr16.fa	84853587	84943116	CRISPLD2	4.739130712	1444
chr16.fa	85008067	85045141	ZDHHC7	8.742850508	1900
chr16.fa	85061410	85127828	KIAA0513	6.07094286	2964
chr16.fa	85131965	85146114	FAM92B	0	0
chr16.fa	85170756	85183049	LOC400548	0	0
chr16.fa	85645029	85709812	KIAA0182	9.60959018	4788
chr16.fa	85711280	85722588	GINS2	0	0
chr16.fa	85741124	85784689	C16orf74	0	0
chr16.fa	85775227	85775306	MIR1910	0	0
chr16.fa	85812231	85833148	COX4NB	10.49560747	1368
chr16.fa	85833196	85840607	COX4I1	274.3442663	14591
chr16.fa	85932774	85956211	IRF8	5.591863991	988
chr16.fa	86365456	86379285	LOC732275	0	0
chr16.fa	86508131	86542466	LOC400550	0	0
chr16.fa	86544133	86548070	FOXF1	0.893547606	152
chr16.fa	86563782	86588841	MTHFSD	3.132160721	684
chr16.fa	86588926	86590905	FLJ30679	0	0
chr16.fa	86600857	86602537	FOXC2	0	0
chr16.fa	86612115	86615304	FOXL1	0	0
chr16.fa	87336404	87351026	LOC100506581	1.978515911	152
chr16.fa	87362942	87425708	FBXO31	5.19170286	1292
chr16.fa	87425801	87438380	MAP1LC3B	24.51299434	3724
chr16.fa	87439852	87525460	ZCCHC14	9.440459224	4332
chr16.fa	87636499	87731748	JPH3	0.287356958	76
chr16.fa	87731762	87739290	LOC100129637	1.322323947	608
chr16.fa	87741418	87799598	KLHDC4	6.523665615	836
chr16.fa	87863629	87903100	SLC7A5	0.503928921	152
chr16.fa	87921625	87970112	CA5A	0	0

chr16.fa	87985038	88110924	BANP	5.556320887	988
chr16.fa	88493879	88507165	ZNF469	0	0
chr16.fa	88520014	88601574	ZFPM1	1.412687771	304
chr16.fa	88636789	88698372	ZC3H18	14.7413517	3648
chr16.fa	88705001	88706882	IL17C	0	0
chr16.fa	88709697	88717457	CYBA	6.654693159	304
chr16.fa	88718348	88729495	MVD	14.66634973	1748
chr16.fa	88729781	88753594	MGC23284	0.404980535	76
chr16.fa	88744090	88752882	SNAI3	0	0
chr16.fa	88762903	88772829	RNF166	0.844449929	152
chr16.fa	88772891	88781745	CTU2	3.32192475	371
chr16.fa	88781787	88851372	FAM38A	4.259901236	2204
chr16.fa	88870186	88875666	CDT1	0	0
chr16.fa	88875877	88878342	APRT	73.75419953	3952
chr16.fa	88880142	88923374	GALNS	0.484350093	76
chr16.fa	88923506	88927520	TRAPPC2L	32.75507866	1368
chr16.fa	88929748	88933068	PABPN1L	0	0
chr16.fa	88941263	89043504	CBFA2T3	2.011197493	608
chr16.fa	89160254	89222169	ACSF3	6.946568308	1672
chr16.fa	89225628	89230083	C16orf81	0	0
chr16.fa	89238163	89261900	CDH15	0.400311737	76
chr16.fa	89284111	89295363	ZNF778	2.350363043	456
chr16.fa	89334035	89556969	ANKRD11	12.91750854	7980
chr16.fa	89574805	89624174	SPG7	3.684133075	988
chr16.fa	89627090	89629848	RPL13	638.2957094	55181
chr16.fa	89627838	89627909	SNORD68	0	0
chr16.fa	89642176	89663654	CPNE7	0	0
chr16.fa	89679716	89704839	DPEP1	8.742248083	1064
chr16.fa	89710844	89724129	CHMP1A	24.76631426	3871
chr16.fa	89724210	89737675	C16orf55	2.090567052	304
chr16.fa	89753076	89762764	CDK10	8.825985226	1049
chr16.fa	89762773	89768121	SPATA2L	1.473080926	228
chr16.fa	89773541	89787392	C16orf7	5.369117166	831
chr16.fa	89778264	89784573	LOC100128881	1.5882948	141
chr16.fa	89787395	89807332	ZNF276	1.917219117	456
chr16.fa	89803959	89883065	FANCA	0.235849579	76
chr16.fa	89894907	89937727	SPIRE2	6.45785063	1391
chr16.fa	89939994	89977792	TCF25	31.41468195	4712
chr16.fa	89984287	89987385	MC1R	0.369286824	76
chr16.fa	89988417	90002505	TUBB3	1.141144482	152
chr16.fa	90015151	90034467	DEF8	9.142258607	2280
chr16.fa	90036183	90039240	CENPBD1	7.970842245	1520
chr16.fa	90044013	90067195	AFG3L1P	0.615829456	152

chr16.fa	90071279	90085937	DBNDD1	6.985424752	1064
chr16.fa	90089008	90111379	GAS8	8.803093057	1321
chr16.fa	90095316	90096309	C16orf3	0	0
chr16.fa	90106169	90114191	LOC100130015	3.963357289	380
chr16.fa	90122974	90142338	PRDM7	0	0
chr17.fa	6011	31420	DOC2B	4.266979735	304
chr17.fa	62180	202633	RPH3AL	4.156133445	760
chr17.fa	260118	264457	C17orf97	5.601653405	684
chr17.fa	289771	295731	FAM101B	3.161830176	760
chr17.fa	411908	618096	VPS53	6.538274433	5928
chr17.fa	635847	646075	FAM57A	1.650947051	228
chr17.fa	647661	655501	GEMIN4	9.484888104	2356
chr17.fa	655900	658576	DBIL5P	0	0
chr17.fa	662549	685512	GLOD4	35.17818458	4153
chr17.fa	685572	695741	RNMTL1	12.484214	1444
chr17.fa	702585	883010	NXN	12.19294128	2432
chr17.fa	900357	905390	TIMM22	9.578264055	1064
chr17.fa	906759	1090616	ABR	19.27761563	7584
chr17.fa	925716	925799	MIR3183	0	0
chr17.fa	1173858	1174565	BHLHA9	0	0
chr17.fa	1182957	1204281	TUSC5	0	0
chr17.fa	1247834	1303556	YWHAE	367.8762452	45433
chr17.fa	1325458	1359544	CRK	29.60605003	4712
chr17.fa	1367480	1396001	MYO1C	82.76693665	28000
chr17.fa	1397871	1420182	INPP5K	16.63281713	3724
chr17.fa	1420213	1421282	LOC100306951	7.69914835	228
chr17.fa	1421391	1466110	PITPNA	53.92928034	12651
chr17.fa	1477696	1532130	SLC43A2	3.432620434	684
chr17.fa	1537152	1549041	SCARF1	0	0
chr17.fa	1549445	1553392	RILP	6.390830795	760
chr17.fa	1553923	1588176	PRPF8	56.79893415	27512
chr17.fa	1606084	1613662	TLCD2	0.775924029	304
chr17.fa	1614798	1619566	C17orf91	5.090947197	456
chr17.fa	1619817	1641893	WDR81	6.335106437	3040
chr17.fa	1646130	1658559	SERPINF2	5.882383683	912
chr17.fa	1665259	1680868	SERPINF1	0	0
chr17.fa	1682829	1733175	SMYD4	5.282066683	1520
chr17.fa	1733273	1802848	RPA1	20.28426862	5852
chr17.fa	1837971	1928178	RTN4RL1	0.361756506	76
chr17.fa	1933431	1945169	DPH1	11.17439039	1015
chr17.fa	1945277	1945525	OVCA2	0	0
chr17.fa	1953202	1953302	MIR132	0	0
chr17.fa	1953565	1953674	MIR212	0	0

chr17.fa	1958393	1962981	HIC1	0	0
chr17.fa	1963133	2207069	SMG6	19.0642064	8195
chr17.fa	2207248	2228553	SRR	8.882462615	785
chr17.fa	2225982	2240678	TSR1	18.18240609	4508
chr17.fa	2232419	2232504	SNORD91B	0	0
chr17.fa	2233573	2233664	SNORD91A	0	0
chr17.fa	2240806	2284348	SGSM2	27.04544049	8740
chr17.fa	2287354	2304258	MNT	9.692122472	3116
chr17.fa	2310275	2318730	LOC284009	0	0
chr17.fa	2319348	2415200	METT10D	11.73856186	4484
chr17.fa	2496923	2588909	PAFAH1B1	38.53414637	14364
chr17.fa	2592680	2614927	KIAA0664	28.92199588	10032
chr17.fa	2651372	2651476	MIR1253	0	0
chr17.fa	2699732	2941035	RAP1GAP2	17.4995568	7742
chr17.fa	2965963	2966901	OR1D5	0	0
chr17.fa	2995352	2996290	OR1D2	0	0
chr17.fa	3029904	3030845	OR1G1	0	0
chr17.fa	3100813	3101742	OR1A2	0	0
chr17.fa	3118915	3119844	OR1A1	0	0
chr17.fa	3143970	3144559	OR1D4	0	0
chr17.fa	3181184	3182268	OR3A2	0	0
chr17.fa	3194929	3195876	OR3A1	0	0
chr17.fa	3213539	3214740	OR3A4	0	0
chr17.fa	3300760	3301704	OR1E1	0	0
chr17.fa	3323862	3324827	OR3A3	0	0
chr17.fa	3336164	3337135	OR1E2	0	0
chr17.fa	3343306	3375142	SPATA22	0.66628259	76
chr17.fa	3377404	3402700	ASPA	5.402702387	532
chr17.fa	3416486	3461289	TRPV3	0.669294718	152
chr17.fa	3468740	3500336	TRPV1	2.187557555	684
chr17.fa	3511556	3539616	SHPK	8.912433283	2128
chr17.fa	3539762	3566188	CTNS	2.388918275	608
chr17.fa	3566398	3571973	TAX1BP3	54.69119797	4256
chr17.fa	3572090	3572962	TMEM93	13.07293431	684
chr17.fa	3576522	3599583	P2RX5	0	0
chr17.fa	3617919	3704537	ITGAE	2.964535829	760
chr17.fa	3627197	3629992	GSG2	0	0
chr17.fa	3714460	3749540	C17orf85	9.344974784	2356
chr17.fa	3763617	3796337	CAMKK1	0.768243104	228
chr17.fa	3799885	3819960	P2RX1	0	0
chr17.fa	3827169	3867736	ATP2A3	2.356688511	760
chr17.fa	3907739	4046253	ZZEF1	12.65650769	9576
chr17.fa	4046462	4060991	CYB5D2	10.42919006	1216

chr17.fa	4066665	4167274	ANKFY1	20.97344338	11780
chr17.fa	4172512	4269969	UBE2G1	25.84074012	7220
chr17.fa	4337219	4391499	SPNS3	0	0
chr17.fa	4402129	4442190	SPNS2	6.222603477	1368
chr17.fa	4442734	4458681	MYBBP1A	12.21312254	3264
chr17.fa	4460222	4463876	GGT6	6.528183806	1140
chr17.fa	4487276	4511614	SMTNL2	3.334274473	532
chr17.fa	4534214	4544960	ALOX15	1.705767771	304
chr17.fa	4574679	4607632	PELP1	10.59244736	2584
chr17.fa	4613789	4624795	ARRB2	7.157567835	912
chr17.fa	4634723	4636827	MED11	16.54486301	836
chr17.fa	4636897	4643223	CXCL16	1.965864975	304
chr17.fa	4643319	4649410	ZMYND15	0	0
chr17.fa	4675187	4686506	TM4SF5	3.233368203	152
chr17.fa	4688580	4689729	VMO1	0	0
chr17.fa	4692254	4693884	GLTPD2	4.683406354	380
chr17.fa	4699457	4701790	PSMB6	59.58620628	3268
chr17.fa	4710421	4726727	PLD2	8.903547507	2052
chr17.fa	4736635	4801063	MINK1	22.17543284	6991
chr17.fa	4801357	4806369	CHRNE	0	0
chr17.fa	4802948	4806227	C17orf107	1.035418808	152
chr17.fa	4835592	4838325	GP1BA	0.457692765	76
chr17.fa	4840426	4843462	SLC25A11	27.4948499	3308
chr17.fa	4843630	4848517	RNF167	13.40291287	1520
chr17.fa	4848947	4851825	PFN1	292.1801271	15656
chr17.fa	4854384	4860426	ENO3	0.770803413	76
chr17.fa	4862521	4871132	SPAG7	42.43424899	2888
chr17.fa	4871287	4890960	CAMTA2	14.86801166	4864
chr17.fa	4891425	4900905	INCA1	0	0
chr17.fa	4901243	4931694	KIF1C	88.30367871	46419
chr17.fa	4935897	4938727	GPR172B	0	0
chr17.fa	4981754	4999669	ZFP3	3.437289231	1140
chr17.fa	5009031	5026397	ZNF232	1.101685612	152
chr17.fa	5031687	5078324	USP6	0.143527873	76
chr17.fa	5082831	5095178	ZNF594	0.469440062	152
chr17.fa	5095379	5138931	LOC100130950	0.738423043	76
chr17.fa	5113901	5124645	C17orf87	0	0
chr17.fa	5185558	5289132	RABEP1	39.8346324	14288
chr17.fa	5289346	5322960	NUP88	17.67546505	2812
chr17.fa	5323060	5336098	RPAIN	13.68740831	1216
chr17.fa	5336341	5342471	C1QBP	65.86784746	4028
chr17.fa	5344232	5372380	DHX33	6.927140086	2584
chr17.fa	5377613	5389494	DERL2	10.18340046	760

chr17.fa	5390247	5394130	MIS12	6.407246889	1064
chr17.fa	5402747	5404319	LOC728392	0	0
chr17.fa	5404719	5487832	NLRP1	0	0
chr17.fa	5973934	6027747	WSCD1	0	0
chr17.fa	6327059	6338519	AIPL1	0	0
chr17.fa	6347735	6354385	FAM64A	0	0
chr17.fa	6354583	6459877	PITPNM3	0.960416835	456
chr17.fa	6481645	6544221	KIAA0753	9.089998196	2812
chr17.fa	6544248	6546632	TXNDC17	36.29252113	1976
chr17.fa	6547862	6554954	MED31	18.7640479	532
chr17.fa	6555059	6556617	C17orf100	0.734206064	76
chr17.fa	6588039	6616740	SLC13A5	0	0
chr17.fa	6659156	6678964	XAF1	0.947464687	228
chr17.fa	6679552	6690965	FBXO39	0	0
chr17.fa	6703300	6735060	TEKT1	0	0
chr17.fa	6756895	6803668	ALOX12P2	0	0
chr17.fa	6899384	6914055	ALOX12	0.970808675	152
chr17.fa	6915798	6917851	RNASEK	90.28324887	4484
chr17.fa	6918073	6920839	C17orf49	36.3367994	2128
chr17.fa	6920934	6921020	MIR195	0	0
chr17.fa	6921230	6921341	MIR497	0	0
chr17.fa	6926369	6932961	BCL6B	0	0
chr17.fa	6939394	6943440	SLC16A13	2.619195418	304
chr17.fa	6944949	6947242	SLC16A11	0	0
chr17.fa	6977856	6983600	CLEC10A	0	0
chr17.fa	7004641	7018128	ASGR2	0.742338808	76
chr17.fa	7076751	7082883	ASGR1	0.761616424	76
chr17.fa	7093210	7123369	DLG4	2.487716055	760
chr17.fa	7123441	7128585	ACADVL	111.9857777	15429
chr17.fa	7126616	7126698	MIR324	0	0
chr17.fa	7128661	7137863	DVL2	6.451675769	1292
chr17.fa	7138347	7142825	PHF23	24.34687551	3293
chr17.fa	7143738	7145753	GABARAP	484.5038619	28728
chr17.fa	7146906	7155259	CTDNEP1	44.41065641	5470
chr17.fa	7155372	7163221	C17orf81	32.92405901	3019
chr17.fa	7163260	7166512	CLDN7	9.129457066	1216
chr17.fa	7185054	7191367	SLC2A4	0	0
chr17.fa	7191571	7197876	YBX2	0.723061193	76
chr17.fa	7210318	7215782	EIF5A	115.216435	13908
chr17.fa	7215978	7218658	GPS2	28.68945965	2204
chr17.fa	7218951	7232638	NEURL4	4.634007464	1596
chr17.fa	7239848	7254793	ACAP1	0.456337308	76
chr17.fa	7255208	7258262	KCTD11	2.622659364	532

chr17.fa	7258497	7260538	TMEM95	0	0
chr17.fa	7284365	7293054	TNK1	4.092276343	760
chr17.fa	7293093	7297843	PLSCR3	8.041777846	912
chr17.fa	7306295	7307418	C17orf61	123.753256	3673
chr17.fa	7311502	7323179	NLGN2	0.246542631	76
chr17.fa	7323679	7324951	SPEM1	0	0
chr17.fa	7328935	7330886	C17orf74	0	0
chr17.fa	7338762	7340998	TMEM102	1.740407236	228
chr17.fa	7342689	7348251	FGF11	0	0
chr17.fa	7348406	7360932	CHRNB1	2.348405161	380
chr17.fa	7362685	7387568	ZBTB4	38.01621106	15428
chr17.fa	7384721	7386383	AMAC1L3	0	0
chr17.fa	7387698	7417935	POLR2A	68.79879806	30780
chr17.fa	7456904	7461207	TNFSF12	13.32233847	912
chr17.fa	7461609	7463210	TNFSF13	7.279107178	507
chr17.fa	7465319	7475287	SENP3	16.05463927	2649
chr17.fa	7476057	7482324	EIF4A1	145.5508174	17821
chr17.fa	7478031	7478165	SNORA48	0	0
chr17.fa	7480129	7480276	SNORD10	0	0
chr17.fa	7481273	7481409	SNORA67	0	0
chr17.fa	7482805	7485429	CD68	3.700247957	456
chr17.fa	7486965	7491497	MPDU1	13.69493863	1444
chr17.fa	7491528	7493488	SOX15	0.839178705	76
chr17.fa	7494548	7518215	FXR2	22.22543415	4408
chr17.fa	7517382	7536700	SHBG	0	0
chr17.fa	7529556	7531194	SAT2	82.70051924	5288
chr17.fa	7554254	7561089	ATP1B2	0	0
chr17.fa	7571720	7590757	TP53	22.93479017	3868
chr17.fa	7589389	7606820	WRAP53	2.739680515	380
chr17.fa	7608520	7614693	EFNB3	0	0
chr17.fa	7623039	7737058	DNAH2	0.084791388	76
chr17.fa	7657638	7658286	RPL29P2	0	0
chr17.fa	7743235	7758118	KDM6B	14.17100537	6308
chr17.fa	7758384	7759417	TMEM88	0	0
chr17.fa	7760003	7761063	LSMD1	30.86587233	1824
chr17.fa	7761173	7765600	CYB5D1	8.811978833	2128
chr17.fa	7788123	7816075	CHD3	107.1455901	54097
chr17.fa	7809441	7809578	SCARNA21	0	0
chr17.fa	7816640	7819265	LOC284023	0.90665036	152
chr17.fa	7826027	7832753	KCNAB3	0	0
chr17.fa	7833663	7835317	TRAPPC1	87.4667591	4408
chr17.fa	7835473	7852896	CNTROB	6.677585327	1672
chr17.fa	7905988	7923658	GUCY2D	0.315972169	76

chr17.fa	7942358	7952451	ALOX15B	44.86970463	8050
chr17.fa	7975954	7991021	ALOX12B	0	0
chr17.fa	7991374	7991465	MIR4314	0	0
chr17.fa	7999218	8022234	ALOXE3	0.331334019	76
chr17.fa	8023908	8027410	HES7	0	0
chr17.fa	8043788	8055753	PER1	4.861423086	1520
chr17.fa	8062465	8066293	VAMP2	54.17642539	7752
chr17.fa	8076297	8079714	TMEM107	1.289642365	152
chr17.fa	8090493	8090577	MIR3676	0	0
chr17.fa	8091651	8093564	C17orf59	4.784162017	608
chr17.fa	8108049	8113883	AURKB	0.926078582	76
chr17.fa	8123948	8127361	C17orf44	1.093251655	152
chr17.fa	8128139	8151413	C17orf68	7.930630344	3724
chr17.fa	8152596	8173809	PFAS	5.526500825	1976
chr17.fa	8191082	8198170	SLC25A35	2.75278327	380
chr17.fa	8193131	8193409	RANGRF	30.93530187	304
chr17.fa	8213556	8225834	ARHGEF15	1.078341624	304
chr17.fa	8243188	8249363	ODF4	0	0
chr17.fa	8261731	8263859	LOC100128288	0.537664748	76
chr17.fa	8271973	8274858	KRBA2	3.496778748	456
chr17.fa	8280834	8286565	RPL26	1042.340978	40280
chr17.fa	8294023	8301144	RNF222	0	0
chr17.fa	8339179	8371481	NDEL1	15.67300272	2508
chr17.fa	8377530	8534036	MYH10	24.77821216	12535
chr17.fa	8633246	8648154	CCDC42	0	0
chr17.fa	8656424	8661877	SPDYE4	0	0
chr17.fa	8700428	8702667	MFSD6L	1.022014841	152
chr17.fa	8706055	8770994	PIK3R6	0	0
chr17.fa	8782233	8869024	PIK3R5	0.251813854	76
chr17.fa	8924859	9147317	NTN1	0.384498068	152
chr17.fa	9153788	9479275	STX8	26.01378684	1672
chr17.fa	9479944	9546776	WDR16	0.522001686	76
chr17.fa	9548950	9633003	USP43	16.31940527	4408
chr17.fa	9674755	9694601	DHRS7C	0	0
chr17.fa	9729381	9793022	GLP2R	0	0
chr17.fa	9801027	9808684	RCVRN	0	0
chr17.fa	9813926	10101868	GAS7	0	0
chr17.fa	10204183	10276322	MYH13	0	0
chr17.fa	10293642	10325267	MYH8	0.379076239	152
chr17.fa	10346608	10372876	MYH4	0.190366454	76
chr17.fa	10395627	10421859	MYH1	0	0
chr17.fa	10424465	10453017	MYH2	0	0
chr17.fa	10531843	10559465	MYH3	0.380431696	152

chr17.fa	10583649	10600885	SCO1	15.00912983	1748
chr17.fa	10600927	10614875	C17orf48	10.55630184	1064
chr17.fa	10616639	10633646	TMEM220	2.216172766	455
chr17.fa	10698230	10707416	LOC100289255	8.229282779	836
chr17.fa	10725792	10741418	PIRT	0	0
chr17.fa	11144740	11467380	SHISA6	0.308743063	152
chr17.fa	11501748	11873065	DNAH9	0.081628654	76
chr17.fa	11880762	11900689	ZNF18	4.136705223	760
chr17.fa	11924135	12047051	MAP2K4	26.51199272	6589
chr17.fa	11985216	11985313	MIR744	0	0
chr17.fa	12453285	12540504	FLJ34690	0	0
chr17.fa	12569207	12670651	MYOCD	0	0
chr17.fa	12692829	12894928	ARHGAP44	4.606597104	1292
chr17.fa	12894961	12921381	ELAC2	12.00618938	3007
chr17.fa	13399006	13505244	HS3ST3A1	0	0
chr17.fa	13927815	13928915	CDRT15P	0	0
chr17.fa	13972719	14111996	COX10	6.481797044	1292
chr17.fa	14139173	14140150	CDRT15	0	0
chr17.fa	14204506	14249492	HS3ST3B1	0.563267832	76
chr17.fa	14207057	14209062	MGC12916	0	0
chr17.fa	14934292	14935274	CDRT7	0	0
chr17.fa	15133096	15168644	PMP22	1.727304482	228
chr17.fa	15207129	15244958	TEKT3	0	0
chr17.fa	15339338	15370925	CDRT4	1.379554369	228
chr17.fa	15405578	15466945	FAM18B2	0.220337122	76
chr17.fa	15491977	15523018	CDRT1	0.828485653	152
chr17.fa	15531280	15586193	TRIM16	23.67336382	4560
chr17.fa	15602891	15624100	ZNF286A	2.270692273	836
chr17.fa	15635591	15648098	TBC1D26	0	0
chr17.fa	15668017	15669003	CDRT15L1	0	0
chr17.fa	15690164	15693019	MEIS3P1	1.603054225	304
chr17.fa	15848231	15879210	ADORA2B	0.607244892	76
chr17.fa	15879875	15897092	ZSWIM7	12.96540136	1578
chr17.fa	15902782	15932096	TTC19	9.157620457	1587
chr17.fa	15933408	16118874	NCOR1	39.1115712	26320
chr17.fa	16120509	16229573	PIGL	0	0
chr17.fa	16185328	16185402	MIR1288	0	0
chr17.fa	16245848	16256812	CENPV	2.950077617	228
chr17.fa	16284367	16286054	UBB	667.1970722	43016
chr17.fa	16318856	16340317	TRPV2	0	0
chr17.fa	16342301	16373962	NCRNA00188	252.5036314	38729
chr17.fa	16342823	16342841	SNORD49B	0	0
chr17.fa	16343350	16343420	SNORD49A	0	0

chr17.fa	16344540	16344612	SNORD65	0	0
chr17.fa	16345341	16395480	C17orf76	0.412962672	76
chr17.fa	16453631	16472520	ZNF287	3.745731081	1064
chr17.fa	16524048	16557167	ZNF624	1.618265469	456
chr17.fa	16593639	16678311	CCDC144A	0	0
chr17.fa	16690206	16707819	LOC162632	0.880294245	228
chr17.fa	16692057	16693815	FAM106C	0	0
chr17.fa	16733797	16736147	KRT16P2	0	0
chr17.fa	16842398	16875402	TNFRSF13B	0	0
chr17.fa	16946107	17088872	MPRIP	24.0274394	6222
chr17.fa	17104309	17109646	PLD6	0.888276383	152
chr17.fa	17115527	17140502	FLCN	18.0786383	4864
chr17.fa	17149938	17184617	COPS3	14.96967096	1976
chr17.fa	17206680	17250977	NT5M	0.720802097	76
chr17.fa	17380300	17396534	MED9	11.33282829	1672
chr17.fa	17397753	17399709	RASD1	2.855346209	317
chr17.fa	17408877	17494994	PEMT	1.891917247	152
chr17.fa	17584787	17714662	RAI1	19.53078494	9800
chr17.fa	17680000	17682843	SMCR5	0	0
chr17.fa	17714766	17740325	SREBF1	8.412871947	2736
chr17.fa	17717150	17717245	MIR33B	0	0
chr17.fa	17746822	17875784	TOM1L2	65.05276577	25087
chr17.fa	17876127	17920189	LRRC48	1.327896383	228
chr17.fa	17921334	17942480	ATPAF2	8.850082245	912
chr17.fa	17942611	17971718	C17orf39	7.843429254	2204
chr17.fa	17991283	18011292	DRG2	15.05265508	1887
chr17.fa	18012020	18083116	MYO15A	0.096538685	76
chr17.fa	18086867	18113267	ALKBH5	55.0131944	12540
chr17.fa	18128936	18148152	LLGL1	5.739458235	1596
chr17.fa	18148189	18162055	FLII	38.57917768	10564
chr17.fa	18163848	18169095	SMCR7	1.298226928	228
chr17.fa	18177235	18218321	TOP3A	4.171344689	1140
chr17.fa	18218594	18231186	SMCR8	17.74805732	9576
chr17.fa	18231371	18266856	SHMT1	32.84318339	5092
chr17.fa	18281079	18292960	EVPLL	0	0
chr17.fa	18325495	18328647	LOC339240	0	0
chr17.fa	18380099	18398259	LGALS9C	0	0
chr17.fa	18414576	18424566	LOC220594	0	0
chr17.fa	18427880	18430160	FAM106A	0	0
chr17.fa	18441115	18528930	CCDC144B	0.394889908	228
chr17.fa	18538842	18547740	TBC1D28	0	0
chr17.fa	18561742	18585572	ZNF286B	0	0
chr17.fa	18569236	18576494	FOXO3B	0.638721624	304

chr17.fa	18625402	18639431	TRIM16L	7.507577044	988
chr17.fa	18647326	18682662	FBXW10	0	0
chr17.fa	18684582	18710026	FAM18B1	6.398059901	760
chr17.fa	18761492	18834580	PRPSAP2	42.81528311	5373
chr17.fa	18855478	18923989	SLC5A10	10.24635392	1343
chr17.fa	18874532	18908060	FAM83G	13.18001544	2432
chr17.fa	18924004	18950336	GRAP	0	0
chr17.fa	19030782	19062148	GRAPL	0	0
chr17.fa	19140690	19240028	EPN2	19.17550451	6156
chr17.fa	19246483	19266046	B9D1	3.700247957	228
chr17.fa	19247819	19247887	MIR1180	0	0
chr17.fa	19281034	19286754	MAPK7	1.463291512	304
chr17.fa	19286858	19290532	MFAP4	0	0
chr17.fa	19314491	19320589	RNF112	0	0
chr17.fa	19437167	19482346	SLC47A1	3.839860064	836
chr17.fa	19552064	19580908	ALDH3A2	58.02306275	14744
chr17.fa	19581628	19620043	SLC47A2	0.972013526	152
chr17.fa	19641298	19651746	ALDH3A1	0	0
chr17.fa	19674143	19771239	ULK2	3.897391698	2365
chr17.fa	19808750	19881129	AKAP10	16.77017014	3247
chr17.fa	19990335	20218067	SPECC1	37.31724689	10937
chr17.fa	20224487	20305504	CCDC144C	0.544592642	152
chr17.fa	20353176	20370848	LGALS9B	0	0
chr17.fa	20404827	20407811	KRT16P3	0	0
chr17.fa	20483037	20484224	CDRT15L2	0	0
chr17.fa	20766708	20799453	CCDC144NL	0	0
chr17.fa	20902906	20946352	USP22	77.37191519	26817
chr17.fa	21030258	21094836	DHRS7B	3.329756281	304
chr17.fa	21101263	21117908	TMEM11	12.8836221	1444
chr17.fa	21142184	21156578	C17orf103	9.568324034	3040
chr17.fa	21187968	21218551	MAP2K3	17.16686733	2706
chr17.fa	21279699	21280225	KCNJ12	0	0
chr17.fa	21308448	21308639	KCNJ18	0	0
chr17.fa	21431571	21454941	C17orf51	1.736039652	912
chr17.fa	21825370	21826499	FAM27L	0	0
chr17.fa	21904062	21913070	FLJ36000	0	0
chr17.fa	22022437	22023991	MTRNR2L1	0	0
chr17.fa	25621106	25640645	WSB1	13.33754971	2508
chr17.fa	25745031	25758649	LOC440419	0	0
chr17.fa	25799036	25950718	KSR1	1.270967174	379
chr17.fa	25958174	25976586	LGALS9	3.099177926	363
chr17.fa	26083792	26127555	NOS2	0.544291429	152
chr17.fa	26205340	26220409	C17orf108	13.91151059	1368

chr17.fa	26369688	26523404	NLK	21.02871592	4940
chr17.fa	26553589	26555085	PYY2	0	0
chr17.fa	26574470	26575315	PPY2	0	0
chr17.fa	26603012	26634408	FLJ40504	1.760889703	228
chr17.fa	26646121	26655352	TMEM97	113.9945655	16720
chr17.fa	26655712	26662495	IFT20	15.84424217	647
chr17.fa	26662548	26673658	TNFAIP1	17.92185707	4084
chr17.fa	26674036	26684603	POLDIP2	41.52443589	6460
chr17.fa	26684687	26689089	TMEM199	10.98628303	1057
chr17.fa	26691290	26692265	SEBOX	0	0
chr17.fa	26694299	26697373	VTN	0	0
chr17.fa	26698987	26728065	SARM1	4.205231123	884
chr17.fa	26721661	26733228	SLC46A1	0	0
chr17.fa	26800664	26824798	SLC13A2	2.163761748	380
chr17.fa	26850959	26865175	FOXN1	0.424408757	76
chr17.fa	26873725	26879646	UNC119	23.15678396	2128
chr17.fa	26880406	26898887	PIGS	6.772768555	1292
chr17.fa	26900133	26903951	ALDOC	15.25220852	1672
chr17.fa	26904583	26926056	SPAG5	2.104121625	532
chr17.fa	26934982	26941211	SGK494	0.302869414	76
chr17.fa	26941458	26972173	KIAA0100	32.96276485	16264
chr17.fa	26975607	26988933	SDF2	6.388571699	456
chr17.fa	26989302	27029249	SUPT6H	39.1469637	15276
chr17.fa	27030534	27038872	PROCA1	0	0
chr17.fa	27041299	27045286	RAB34	5.639154392	492
chr17.fa	27047000	27051365	RPL23A	150.0735268	9576
chr17.fa	27047568	27047634	SNORD42B	0	0
chr17.fa	27049600	27049671	SNORD4A	0	0
chr17.fa	27050447	27050509	SNORD42A	0	0
chr17.fa	27050699	27050772	SNORD4B	0	0
chr17.fa	27051375	27053949	TLCD1	0.963428963	76
chr17.fa	27055832	27069784	NEK8	2.803387011	532
chr17.fa	27071023	27077976	TRAF4	15.77677051	3040
chr17.fa	27082996	27169841	C17orf63	11.45421703	3192
chr17.fa	27182043	27188072	ERAL1	20.49074996	2483
chr17.fa	27188387	27188458	MIR451	0	0
chr17.fa	27188551	27188636	MIR144	0	0
chr17.fa	27206357	27224715	FLOT2	42.18348938	7372
chr17.fa	27224799	27230089	DHRS13	2.979144647	380
chr17.fa	27232271	27278508	PHF12	19.50096488	6232
chr17.fa	27281947	27333081	SEZ6	0	0
chr17.fa	27369918	27384236	PIPOX	13.76180786	2204
chr17.fa	27400528	27507407	MYO18A	10.20343111	4193

chr17.fa	27401933	27402627	TIAF1	0	0
chr17.fa	27573875	27581502	CRYBA1	0	0
chr17.fa	27582854	27621166	NUFIP2	18.80230192	13583
chr17.fa	27717943	27878921	TAOK1	18.85411051	15124
chr17.fa	27887689	27894042	ABHD15	2.04704181	456
chr17.fa	27895739	27900175	TP53I13	0.787821933	76
chr17.fa	27900487	27916610	GIT1	10.35012171	2584
chr17.fa	27920527	27941773	ANKRD13B	0	0
chr17.fa	27941780	27948441	CORO6	0	0
chr17.fa	27952965	28257018	SSH2	10.72633643	6506
chr17.fa	28256874	28435470	EFCAB5	0.544291429	228
chr17.fa	28443834	28513486	CCDC55	24.17894941	4012
chr17.fa	28444097	28444190	MIR423	0	0
chr17.fa	28523378	28562954	SLC6A4	0	0
chr17.fa	28575223	28619074	BLMH	7.577006582	1216
chr17.fa	28643366	28661065	TMIGD1	0	0
chr17.fa	28705942	28796675	CPD	2.361809128	1444
chr17.fa	28804426	28853832	GOSR1	15.52902303	5371
chr17.fa	28886584	28890509	TBC1D29	0	0
chr17.fa	28903483	28964484	LRRC37BP1	2.48997515	760
chr17.fa	28951336	28953825	SH3GL1P2	0	0
chr17.fa	29058724	29085353	SUZ12P	0	0
chr17.fa	29109702	29151778	CRLF3	4.281136734	836
chr17.fa	29159023	29222295	ATAD5	0.183438561	76
chr17.fa	29226001	29233286	C17orf42	2.564525305	228
chr17.fa	29248754	29286211	ADAP2	0.416426619	76
chr17.fa	29297956	29326927	RNF135	19.09402646	2812
chr17.fa	29302353	29303017	DPRXP4	0	0
chr17.fa	29421945	29704695	NF1	19.62069695	16187
chr17.fa	29621668	29624380	OMG	0	0
chr17.fa	29630788	29641130	EVI2B	0	0
chr17.fa	29643428	29648767	EVI2A	0	0
chr17.fa	29718642	29865232	RAB11FIP4	30.16148633	17273
chr17.fa	29887015	29887102	MIR193A	0	0
chr17.fa	29902430	29902540	MIR365-2	0	0
chr17.fa	30178884	30186326	C17orf79	83.06649273	5168
chr17.fa	30190190	30228729	UTP6	18.29159571	2508
chr17.fa	30264044	30328057	SUZ12	14.53246067	4320
chr17.fa	30348155	30380519	LRRC37B	3.01513957	606
chr17.fa	30367355	30369851	SH3GL1P1	0	0
chr17.fa	30469473	30552745	RHOT1	14.86936712	3266
chr17.fa	30477387	30478590	ARGFXP2	0	0
chr17.fa	30593195	30651680	RHBDL3	0	0

chr17.fa	30658390	30669189	C17orf75	9.92842387	1064
chr17.fa	30677128	30677156	MIR632	0	0
chr17.fa	30677222	30697468	ZNF207	46.40257628	6957
chr17.fa	30771502	30808042	PSMD11	47.83017409	5021
chr17.fa	30814105	30818271	CDK5R1	0.296995766	76
chr17.fa	30819628	31203902	MYO1D	25.84314982	8892
chr17.fa	31254928	31268667	TMEM98	11.46114492	1366
chr17.fa	31318882	31324892	SPACA3	0	0
chr17.fa	31340106	32483825	ACCN1	0	0
chr17.fa	31856806	31860755	AA06	0	0
chr17.fa	32582296	32584220	CCL2	1.53226923	76
chr17.fa	32597240	32599256	CCL7	0	0
chr17.fa	32612687	32615199	CCL11	0	0
chr17.fa	32646066	32648421	CCL8	0	0
chr17.fa	32683471	32685629	CCL13	0	0
chr17.fa	32687399	32690252	CCL1	0	0
chr17.fa	32901142	32906388	C17orf102	0	0
chr17.fa	32907768	32966337	TMEM132E	0.523959569	152
chr17.fa	33254878	33288528	CCT6B	1.809234348	228
chr17.fa	33288549	33290205	ZNF830	8.289224116	912
chr17.fa	33307517	33332088	LIG3	17.90288067	4636
chr17.fa	33336131	33416294	RFFL	27.38776877	7805
chr17.fa	33426811	33446888	RAD51L3	5.317609787	912
chr17.fa	33448631	33457751	FNDC8	0	0
chr17.fa	33458368	33469322	NLE1	6.670205615	1140
chr17.fa	33474836	33516364	UNC45B	0	0
chr17.fa	33519539	33521412	AMAC1	0	0
chr17.fa	33570086	33594761	SLFN5	23.15332001	7144
chr17.fa	33677329	33700720	SLFN11	0	0
chr17.fa	33738081	33759543	SLFN12	6.225916817	988
chr17.fa	33762115	33775856	SLFN13	0.135093916	76
chr17.fa	33801942	33814758	SLFN12L	0	0
chr17.fa	33875144	33885110	SLFN14	0	0
chr17.fa	33900676	33900772	SNORD7	0	0
chr17.fa	33901814	33905656	PEX12	2.133038048	380
chr17.fa	33914282	34053436	AP2B1	46.07832077	17632
chr17.fa	34058679	34070540	RASL10B	0.716735725	152
chr17.fa	34071537	34079897	GAS2L2	0	0
chr17.fa	34087916	34092098	C17orf50	0	0
chr17.fa	34092876	34122640	MMP28	0.401968407	76
chr17.fa	34136488	34174237	TAF15	95.75703746	13689
chr17.fa	34181960	34195895	C17orf66	0	0
chr17.fa	34198496	34207377	CCL5	0	0

chr17.fa	34245085	34257780	RDM1	0	0
chr17.fa	34261523	34270703	LYZL6	0	0
chr17.fa	34303535	34308523	CCL16	0	0
chr17.fa	34324618	34324756	CCL15	8.234554002	76
chr17.fa	34340097	34345005	CCL23	0	0
chr17.fa	34391643	34398841	CCL18	0	0
chr17.fa	34415603	34417506	CCL3	0	0
chr17.fa	34431220	34433014	CCL4	0	0
chr17.fa	34493061	34503988	TBC1D3B	0	0
chr17.fa	34581085	34592027	TBC1D3C	0	0
chr17.fa	34745936	34757050	TBC1D3H	0	0
chr17.fa	34797175	34808103	TBC1D3G	0	0
chr17.fa	34842473	34851598	ZNHIT3	27.03806078	1596
chr17.fa	34851663	34891305	MYO19	4.868049766	1514
chr17.fa	34891403	34895150	PIGW	1.522781029	228
chr17.fa	34900737	34946276	GGNBP2	37.41604467	7068
chr17.fa	34948226	34957233	DHRS11	10.6310026	1128
chr17.fa	34958025	34965407	MRM1	0	0
chr17.fa	35294772	35301915	LHX1	3.336081749	760
chr17.fa	35306175	35414171	AATF	20.83593976	2962
chr17.fa	35391042	35391110	MIR2909	0	0
chr17.fa	35441927	35766902	ACACA	31.33757149	23679
chr17.fa	35732985	35749662	C17orf78	0	0
chr17.fa	35766977	35837226	TADA2A	3.607022612	456
chr17.fa	35849951	35873588	DUSP14	4.554186087	456
chr17.fa	35874900	35969486	SYNRG	25.48244756	13942
chr17.fa	35972363	36003493	DDX52	8.431245924	2204
chr17.fa	36046434	36105096	HNF1B	24.61962365	4636
chr17.fa	36202573	36244363	LOC284100	0.93240405	152
chr17.fa	36337528	36337710	TBC1D3	0	0
chr17.fa	36351796	36413256	LOC440434	5.497283189	603
chr17.fa	36452998	36479101	MRPL45	26.99543917	3040
chr17.fa	36481493	36499693	GPR179	0	0
chr17.fa	36508007	36561846	SOCS7	11.47771162	6080
chr17.fa	36584720	36668628	ARHGAP23	34.19653225	14416
chr17.fa	36686259	36762183	SRCIN1	0.324707338	152
chr17.fa	36827959	36831187	C17orf96	5.671685368	1216
chr17.fa	36861873	36886056	MLLT6	28.38628902	14136
chr17.fa	36886510	36891858	CISD3	41.5459726	2571
chr17.fa	36892343	36904558	PCGF2	42.87266413	2562
chr17.fa	36909002	36920478	PSMB3	75.90244882	3800
chr17.fa	36921944	36956158	PIP4K2B	14.09103339	5363
chr17.fa	36957446	36981589	CWC25	11.29864064	1748

chr17.fa	36991341	36997642	C17orf98	0	0
chr17.fa	37006321	37010053	RPL23	1832.960424	70224
chr17.fa	37009116	37009248	SNORA21	0	0
chr17.fa	37026112	37078023	LASP1	52.36960075	14288
chr17.fa	37092685	37123655	FBXO47	0	0
chr17.fa	37186159	37209458	FLJ43826	0.639474656	152
chr17.fa	37213272	37237704	LOC100131347	0	0
chr17.fa	37219556	37307902	PLXDC1	0	0
chr17.fa	37313147	37322414	ARL5C	0	0
chr17.fa	37329709	37353901	CACNB1	0	0
chr17.fa	37356536	37360980	RPL19	981.9865291	47728
chr17.fa	37366789	37382040	STAC2	0.349256177	76
chr17.fa	37408897	37557909	FBXL20	3.515303332	2423
chr17.fa	37560538	37607527	MED1	12.36192163	6688
chr17.fa	37617739	37690800	CDK12	16.8447203	9272
chr17.fa	37760021	37764175	NEUROD2	0	0
chr17.fa	37783179	37792877	PPP1R1B	14.4576093	1966
chr17.fa	37793333	37820454	STARD3	2.019480844	380
chr17.fa	37821599	37822807	TCAP	0	0
chr17.fa	37824507	37826728	PNMT	0.989333259	76
chr17.fa	37827375	37844310	PGAP3	1.277895068	228
chr17.fa	37844393	37884915	ERBB2	31.03184055	10564
chr17.fa	37885409	37886788	C17orf37	92.60093033	4636
chr17.fa	37894187	37903538	GRB7	9.484285678	1444
chr17.fa	37921199	38020441	IKZF3	12.21673709	1976
chr17.fa	38024455	38034149	ZPBP2	0	0
chr17.fa	38060848	38074903	GSDMB	3.599341687	380
chr17.fa	38077296	38083884	ORMDL3	11.23734385	1596
chr17.fa	38097727	38100986	LOC728129	0	0
chr17.fa	38119226	38134019	GSDMA	1.057859158	152
chr17.fa	38137060	38154212	PSMD3	27.62407017	3938
chr17.fa	38171614	38174066	CSF3	0	0
chr17.fa	38175350	38210889	MED24	15.46772624	3838
chr17.fa	38183795	38183898	SNORD124	0	0
chr17.fa	38218446	38250120	THRA	8.918758751	2001
chr17.fa	38249037	38256973	NR1D1	6.39188504	1064
chr17.fa	38278790	38293045	MSL1	17.97818385	4560
chr17.fa	38296507	38328431	CASC3	50.70901489	14074
chr17.fa	38334242	38351906	RAPGEFL1	7.063589459	1748
chr17.fa	38375574	38438439	WIPF2	24.23874014	8892
chr17.fa	38444146	38459413	CDC6	1.874597514	380
chr17.fa	38465423	38513895	RARA	4.19950808	1216
chr17.fa	38516905	38520945	GJD3	1.416302323	380

chr17.fa	38544773	38574202	TOP2A	0.397902035	152
chr17.fa	38599676	38613982	IGFBP4	22.01172371	3268
chr17.fa	38632080	38657854	TNS4	1.124427174	304
chr17.fa	38710022	38721736	CCR7	0	0
chr17.fa	38783976	38804103	SMARCE1	39.24094208	6308
chr17.fa	38811872	38821416	KRT222	0	0
chr17.fa	38854243	38860002	KRT24	0	0
chr17.fa	38904273	38911584	KRT25	0	0
chr17.fa	38922490	38928411	KRT26	0	0
chr17.fa	38933060	38938786	KRT27	0	0
chr17.fa	38948448	38956211	KRT28	0	0
chr17.fa	38974369	38978863	KRT10	4.423610362	608
chr17.fa	38975374	38992526	TMEM99	7.502155215	1064
chr17.fa	39017430	39023462	KRT12	0	0
chr17.fa	39032141	39041495	KRT20	15.21922572	1824
chr17.fa	39078952	39093836	KRT23	34.00737065	4848
chr17.fa	39114669	39123144	KRT39	0	0
chr17.fa	39133968	39143387	KRT40	0	0
chr17.fa	39149682	39150385	KRTAP3-3	0	0
chr17.fa	39155445	39156138	KRTAP3-2	0	0
chr17.fa	39164774	39165366	KRTAP3-1	0	0
chr17.fa	39182279	39183454	KRTAP1-5	0	0
chr17.fa	39190137	39191107	KRTAP1-3	0	0
chr17.fa	39196811	39197713	KRTAP1-1	0	0
chr17.fa	39202796	39203568	KRTAP2-1	0	0
chr17.fa	39210751	39211463	KRTAP2-2	0	0
chr17.fa	39215493	39216344	LOC730755	0	0
chr17.fa	39221368	39222131	KRTAP2-4	0	0
chr17.fa	39240459	39241396	KRTAP4-7	0	0
chr17.fa	39253234	39254375	KRTAP4-8	0	0
chr17.fa	39261641	39262740	KRTAP4-9	0	0
chr17.fa	39273434	39274606	KRTAP4-11	0	0
chr17.fa	39279345	39280419	KRTAP4-12	0	0
chr17.fa	39305176	39306054	KRTAP4-5	0	0
chr17.fa	39315906	39316983	KRTAP4-4	0	0
chr17.fa	39323483	39324424	KRTAP4-3	0	0
chr17.fa	39333698	39334460	KRTAP4-2	0	0
chr17.fa	39340352	39341147	KRTAP4-1	0	0
chr17.fa	39346139	39346891	KRTAP9-1	0	0
chr17.fa	39382900	39383904	KRTAP9-2	0	0
chr17.fa	39388715	39389706	KRTAP9-3	0	0
chr17.fa	39394270	39395256	KRTAP9-8	0	0
chr17.fa	39405939	39406905	KRTAP9-4	0	0

chr17.fa	39411636	39412616	KRTAP9-9	0	0
chr17.fa	39463952	39465505	KRTAP16-1	0	0
chr17.fa	39471169	39471947	KRTAP17-1	0	0
chr17.fa	39502371	39507056	KRT33A	0	0
chr17.fa	39519746	39526047	KRT33B	0	0
chr17.fa	39533921	39538636	KRT34	0	0
chr17.fa	39549977	39553844	KRT31	0	0
chr17.fa	39576809	39580822	KRT37	0	0
chr17.fa	39592621	39597596	KRT38	0	0
chr17.fa	39615765	39623638	KRT32	0	0
chr17.fa	39632941	39637392	KRT35	0	0
chr17.fa	39642388	39646116	KRT36	0	0
chr17.fa	39657233	39661865	KRT13	15.85101945	1805
chr17.fa	39669997	39675270	KRT15	1.24415924	152
chr17.fa	39679869	39684641	KRT19	174.2979593	17024
chr17.fa	39722094	39728310	KRT9	0	0
chr17.fa	39738531	39743147	KRT14	0	0
chr17.fa	39766031	39769079	KRT16	0	0
chr17.fa	39775692	39780882	KRT17	0	0
chr17.fa	39782579	39796451	KRT42P	0	0
chr17.fa	39845127	39847898	EIF1	341.8287225	30096
chr17.fa	39868613	39872221	GAST	0	0
chr17.fa	39878891	39890898	HAP1	0	0
chr17.fa	39910859	39942964	JUP	94.79616881	20211
chr17.fa	39958205	39968451	LEPREL4	0.439921213	76
chr17.fa	39968962	39979469	FKBP10	0	0
chr17.fa	39981334	39992523	NT5C3L	21.76638593	2204
chr17.fa	39994043	40004599	KLHL10	0	0
chr17.fa	40009799	40021629	KLHL11	10.33400683	1596
chr17.fa	40023179	40075272	ACLY	76.65246855	22155
chr17.fa	40086888	40117668	TTC25	1.648537349	228
chr17.fa	40118759	40129754	CNP	24.75336211	7904
chr17.fa	40133873	40169715	DNAJC7	45.31173433	5235
chr17.fa	40172087	40177656	NKIRAS2	14.25007372	2356
chr17.fa	40253422	40264751	DHX58	4.811120558	836
chr17.fa	40265129	40273382	KAT2A	5.149231863	1064
chr17.fa	40274756	40275371	HSPB9	0	0
chr17.fa	40276997	40307022	RAB5C	65.2401201	7676
chr17.fa	40308910	40333296	KCNH4	0	0
chr17.fa	40336078	40337470	HCRT	0	0
chr17.fa	40341105	40346550	GHDC	5.615358585	912
chr17.fa	40351195	40428424	STAT5B	11.45963886	3876
chr17.fa	40439565	40463960	STAT5A	4.791391123	1368

chr17.fa	40465343	40540405	STAT3	70.15470723	22429
chr17.fa	40554467	40575338	PTRF	21.41803339	5168
chr17.fa	40610862	40674597	ATP6V0A1	9.352655709	2584
chr17.fa	40687951	40696466	NAGLU	2.057132437	380
chr17.fa	40703984	40707232	HSD17B1	5.643521976	836
chr17.fa	40714092	40718295	COASY	17.9177907	2728
chr17.fa	40719078	40725221	MLX	26.88670137	3192
chr17.fa	40725329	40729747	PSMC3IP	0	0
chr17.fa	40731526	40761357	FAM134C	7.690262574	1900
chr17.fa	40761446	40767256	TUBG1	19.04613364	2356
chr17.fa	40811266	40819024	TUBG2	6.925634022	836
chr17.fa	40819933	40829048	PLEKHH3	10.84411061	2204
chr17.fa	40831420	40833845	CCR10	0	0
chr17.fa	40834632	40852011	CNTNAP1	0.629534635	228
chr17.fa	40852293	40897071	EZH1	13.5500553	4180
chr17.fa	40905947	40913211	LOC100190938	0	0
chr17.fa	40913276	40915060	RAMP2	0	0
chr17.fa	40925454	40931617	VPS25	31.76273328	2322
chr17.fa	40932649	40949084	WNK4	11.77305072	3266
chr17.fa	40949652	40950704	CCDC56	101.8442458	5092
chr17.fa	40950854	40963605	CNTD1	1.84613291	304
chr17.fa	40963673	40976310	BECN1	48.00156414	4290
chr17.fa	40985423	40995777	PSME3	39.24109268	8369
chr17.fa	40996609	41002724	AOC2	0.429529373	76
chr17.fa	41003201	41010140	AOC3	0	0
chr17.fa	41019162	41021234	LOC90586	0	0
chr17.fa	41026691	41050751	LOC388387	2.482896651	304
chr17.fa	41052815	41065386	G6PC	10.72151703	2204
chr17.fa	41102543	41132545	AARSD1	6.109949911	1368
chr17.fa	41132582	41145707	RUNDC1	14.95054395	3800
chr17.fa	41150446	41154971	RPL27	850.2805036	27664
chr17.fa	41158742	41166475	IFI35	22.91054254	1900
chr17.fa	41166622	41174459	VAT1	56.85691761	10412
chr17.fa	41177258	41184058	RND2	1.102739857	304
chr17.fa	41196312	41277500	BRCA1	0.933157082	456
chr17.fa	41277600	41297125	NBR2	5.027541914	456
chr17.fa	41322498	41363707	NBR1	92.30107304	31017
chr17.fa	41363894	41371589	TMEM106A	0.818997452	152
chr17.fa	41447213	41466266	LOC100130581	0.780592827	228
chr17.fa	41476353	41478504	ARL4D	2.888630217	304
chr17.fa	41522174	41522253	MIR2117	0	0
chr17.fa	41561334	41601680	DHX8	28.07016624	7828
chr17.fa	41605211	41623762	ETV4	0.463566413	76

chr17.fa	41717758	41739262	MEOX1	0	0
chr17.fa	41831099	41836156	SOST	0	0
chr17.fa	41843489	41856368	DUSP3	37.76484902	10336
chr17.fa	41857803	41862054	C17orf105	0	0
chr17.fa	41878167	41910547	MPP3	0.403775684	76
chr17.fa	41924516	41940997	CD300LG	0	0
chr17.fa	41952727	41985113	MPP2	1.297172683	380
chr17.fa	41994576	41995355	C17orf88	0	0
chr17.fa	42018172	42019833	PPY	0	0
chr17.fa	42030107	42081837	PYY	0	0
chr17.fa	42082032	42086436	NAGS	3.314545038	456
chr17.fa	42088556	42092345	TMEM101	15.61848322	1596
chr17.fa	42112003	42144987	LSM12	2.303976281	380
chr17.fa	42148098	42153712	G6PC3	6.459356694	760
chr17.fa	42154121	42201014	HDAC5	8.789086665	3107
chr17.fa	42219274	42239844	C17orf53	0	0
chr17.fa	42248074	42256451	ASB16	0.747158212	76
chr17.fa	42253352	42264082	C17orf65	2.096290094	228
chr17.fa	42264354	42269099	TMUB2	3.145715295	532
chr17.fa	42269173	42275529	ATXN7L3	21.13655008	5072
chr17.fa	42282401	42298250	UBTF	35.45454728	11361
chr17.fa	42325758	42345502	SLC4A1	0.924421912	304
chr17.fa	42385927	42396038	RUNDC3A	0	0
chr17.fa	42396993	42402217	SLC25A39	78.83912247	8428
chr17.fa	42422491	42430470	GRN	7.945239162	1216
chr17.fa	42431101	42441235	FAM171A2	0	0
chr17.fa	42449550	42466873	ITGA2B	0	0
chr17.fa	42472645	42580957	GPATCH8	16.89050463	7892
chr17.fa	42634812	42638630	FZD2	0	0
chr17.fa	42733762	42753165	C17orf104	0	0
chr17.fa	42754805	42767165	CCDC43	11.24261507	1596
chr17.fa	42785976	42829636	DBF4B	0.221843186	76
chr17.fa	42836568	42859214	ADAM11	0.259946598	76
chr17.fa	42875816	42908179	GJC1	0	0
chr17.fa	42925279	42926756	HIGD1B	0	0
chr17.fa	42927655	42976993	EFTUD2	29.60394154	7898
chr17.fa	42977154	42980629	CCDC103	2.733957473	228
chr17.fa	42982993	42992914	GFAP	0	0
chr17.fa	43003448	43025082	KIF18B	0	0
chr17.fa	43037061	43045644	C1QL1	0	0
chr17.fa	43100706	43138473	DCAKD	29.38932746	4256
chr17.fa	43138680	43186382	NMT1	25.31075629	8208
chr17.fa	43189008	43209891	PLCD3	28.78901046	6612

chr17.fa	43209967	43221543	ACBD4	4.83883213	836
chr17.fa	43224684	43229468	HEXIM1	39.70842426	12616
chr17.fa	43238264	43247406	HEXIM2	6.884819695	608
chr17.fa	43299292	43324683	FMNL1	0	0
chr17.fa	43325303	43345997	LOC100133991	1.843120782	152
chr17.fa	43331760	43339479	C17orf46	0	0
chr17.fa	43340488	43394414	MAP3K14	7.576705369	2204
chr17.fa	43471268	43503012	ARHGAP27	16.6203168	4028
chr17.fa	43506718	43510282	SH3D20	1.662393136	152
chr17.fa	43513266	43568146	PLEKHM1	9.855982205	3420
chr17.fa	43583249	43597889	LRRC37A4	1.581366907	760
chr17.fa	43677491	43679748	LOC644172	0	0
chr17.fa	43697712	43715329	MGC57346	3.040742653	532
chr17.fa	43716341	43723595	C17orf69	1.21509221	76
chr17.fa	43861646	43913194	CRHR1	0	0
chr17.fa	43920722	43972879	LOC100128977	0	0
chr17.fa	43922256	43924438	IMP5	0	0
chr17.fa	43971748	44105699	MAPT	5.038686786	2280
chr17.fa	43973149	43976164	LOC100130148	0	0
chr17.fa	44076616	44077060	STH	0	0
chr17.fa	44107282	44302740	KIAA1267	25.51512914	9489
chr17.fa	44270939	44274089	LOC644246	2.192678172	76
chr17.fa	44372497	44410022	LRRC37A	0.237807462	76
chr17.fa	44376500	44657088	ARL17A	0.232988058	152
chr17.fa	44450179	44500463	NSFP1	0	0
chr17.fa	44590076	44627885	LRRC37A2	0.713572991	228
chr17.fa	44668038	44834833	NSF	40.84023114	9209
chr17.fa	44841687	44896082	WNT3	0	0
chr17.fa	44928968	44954437	WNT9B	0.781797678	76
chr17.fa	45000486	45018733	GOSR2	24.15440057	2356
chr17.fa	45055522	45056614	RPRML	0	0
chr17.fa	45195311	45266665	CDC27	7.549144403	2812
chr17.fa	45286428	45301045	MYL4	0	0
chr17.fa	45331208	45390077	ITGB3	0.233891696	76
chr17.fa	45401327	45518677	C17orf57	5.762651617	1798
chr17.fa	45500843	45504058	LOC100272146	0.711765715	152
chr17.fa	45527593	45569986	MRPL45P2	1.63980218	152
chr17.fa	45608444	45700642	NPEPPS	31.48878028	9072
chr17.fa	45727275	45761004	KPNB1	72.72194345	20285
chr17.fa	45772630	45789429	TBKBP1	1.938153403	532
chr17.fa	45810610	45823485	TBX21	0.44504183	76
chr17.fa	45884733	45899147	OSBPL7	4.696207896	1140
chr17.fa	45900638	45908907	MRPL10	16.63010622	2204

chr17.fa	45908993	45915046	LRRC46	0	0
chr17.fa	45915080	45918699	SCRN2	21.63867173	2204
chr17.fa	45922280	45933240	SP6	6.63722282	1672
chr17.fa	45973516	46006323	SP2	5.517012624	1140
chr17.fa	46018889	46026674	PNPO	26.67012941	6136
chr17.fa	46029334	46035110	PRR15L	39.69742999	3648
chr17.fa	46048429	46059147	CDK5RAP3	31.97584129	4000
chr17.fa	46103533	46115152	COPZ2	4.903442264	294
chr17.fa	46114527	46114613	MIR152	0	0
chr17.fa	46125686	46138907	NFE2L1	22.30977372	7220
chr17.fa	46147414	46178883	CBX1	21.81427876	3876
chr17.fa	46184920	46200105	SNX11	10.48355896	1672
chr17.fa	46210802	46507594	SKAP1	4.137910074	433
chr17.fa	46233789	46233873	MIR1203	0	0
chr17.fa	46606807	46608272	HOXB1	0	0
chr17.fa	46620019	46622393	HOXB2	12.86072994	1368
chr17.fa	46626232	46651810	HOXB3	3.480061441	836
chr17.fa	46652869	46655743	HOXB4	2.815134308	380
chr17.fa	46657200	46657309	MIR10A	0	0
chr17.fa	46667823	46683774	LOC404266	1.216447668	76
chr17.fa	46668619	46671103	HOXB5	3.92886843	456
chr17.fa	46673099	46682334	HOXB6	9.096173057	912
chr17.fa	46684595	46688383	HOXB7	20.11031826	1824
chr17.fa	46689708	46692301	HOXB8	8.162262944	988
chr17.fa	46698519	46703835	HOXB9	6.782859182	1216
chr17.fa	46709852	46709921	MIR196A1	0	0
chr17.fa	46799082	46799882	PRAC	116.5527653	2964
chr17.fa	46800542	46802119	NCRNA00253	11.53840599	380
chr17.fa	46802127	46806111	HOXB13	140.6255372	28348
chr17.fa	46839593	46894469	TTLL6	0	0
chr17.fa	46908372	46942229	CALCOCO2	36.61451755	7979
chr17.fa	46970148	46973232	ATP5G1	53.02594332	2028
chr17.fa	46985731	47006422	UBE2Z	41.00966331	8512
chr17.fa	47007459	47022154	SNF8	27.30282678	2199
chr17.fa	47035918	47045955	GIP	0	0
chr17.fa	47074774	47133507	IGF2BP1	0.261000843	152
chr17.fa	47209822	47247351	B4GALNT2	0.542484152	76
chr17.fa	47283596	47286762	GNGT2	0	0
chr17.fa	47287589	47300587	ABI3	0	0
chr17.fa	47300732	47308128	PHOSPHO1	0	0
chr17.fa	47325605	47336027	FLJ40194	0	0
chr17.fa	47366568	47439835	ZNF652	14.4648384	11096
chr17.fa	47481420	47492242	PHB	63.93767619	7752

chr17.fa	47572655	47592382	NGFR	0	0
chr17.fa	47653298	47661171	NXPH3	0	0
chr17.fa	47676246	47755525	SPOP	22.14260065	4618
chr17.fa	47778690	47785282	SLC35B1	19.14960022	1508
chr17.fa	47787687	47841518	FAM117A	11.15029337	1748
chr17.fa	47865981	47906458	MYST2	21.97542758	5320
chr17.fa	47915671	47925379	TAC4	0	0
chr17.fa	48046562	48052323	DLX4	0	0
chr17.fa	48067369	48072588	DLX3	1.759534246	304
chr17.fa	48133340	48167849	ITGA3	3.87073437	1292
chr17.fa	48172101	48188733	PDK2	15.18729717	3116
chr17.fa	48190035	48207167	SAMD14	0.615678849	76
chr17.fa	48211101	48227877	PPP1R9B	10.74109585	2887
chr17.fa	48243366	48253293	SGCA	0	0
chr17.fa	48248789	48249837	HILS1	0	0
chr17.fa	48261457	48279000	COL1A1	0.193077369	76
chr17.fa	48348767	48358846	TMEM92	0	0
chr17.fa	48423393	48438512	XYLT2	0.641733752	152
chr17.fa	48445228	48450562	MRPL27	52.10167201	2432
chr17.fa	48450581	48458593	EME1	0	0
chr17.fa	48458821	48474914	LRRC59	46.84038901	8360
chr17.fa	48503519	48552200	ACSF2	19.42611352	2888
chr17.fa	48541853	48546227	CHAD	0	0
chr17.fa	48556190	48563336	RSAD1	13.23453495	2196
chr17.fa	48585745	48608862	MYCBPAP	1.076383742	228
chr17.fa	48610048	48621111	EPN3	23.02620823	6004
chr17.fa	48624562	48633213	SPATA20	25.90399479	4484
chr17.fa	48638449	48704431	CACNA1G	0	0
chr17.fa	48712218	48769063	ABCC3	0.66462592	228
chr17.fa	48770551	48785270	ANKRD40	17.84595146	4940
chr17.fa	48796926	48830072	LUC7L3	26.76034263	3575
chr17.fa	48834759	48844918	C17orf73	1.118854739	76
chr17.fa	48912605	48919709	WFIKKN2	0	0
chr17.fa	48939587	48941413	TOB1	74.55301572	9044
chr17.fa	49039535	49198226	SPAG9	112.3276542	61703
chr17.fa	49230897	49230919	NME1-NME2	0	0
chr17.fa	49239089	49239450	NME1	45.30676432	1089
chr17.fa	49242796	49244187	NME2	2.678534328	114
chr17.fa	49254786	49337427	MBTD1	14.24917008	5092
chr17.fa	49337897	49375292	UTP18	22.41504758	2804
chr17.fa	49707674	50237377	CA10	0.660860761	152
chr17.fa	51900239	51902573	KIF2B	0	0
chr17.fa	52978052	53037988	TOM1L1	64.01945545	6831

chr17.fa	53029259	53046064	COX11		3.461536857	456
chr17.fa	53046126	53241449	STXBP4		3.218458172	1292
chr17.fa	53342321	53402426	HLF		2.248703742	836
chr17.fa	53469974	53499341	MMD		0.420342385	76
chr17.fa	53796988	53809482	TMEM100		0.507995293	76
chr17.fa	53828340	53854748	PCTP		19.49328396	2964
chr17.fa	54230836	54560007	ANKFN1		11.32334009	1824
chr17.fa	54671060	54672951	NOG		0	0
chr17.fa	54869274	54893250	C17orf67		0	0
chr17.fa	54911460	54946036	DGKE		1.326089107	684
chr17.fa	54961463	54962274	MTVR2		0	0
chr17.fa	54965270	54991409	TRIM25		19.72250686	7522
chr17.fa	55015561	55038411	COIL		9.506575421	1662
chr17.fa	55055468	55084129	SCPEP1		4.04965474	523
chr17.fa	55122839	55124156	RNF126P1		0	0
chr17.fa	55162553	55198710	AKAP1		7.211484917	1900
chr17.fa	55333931	55757299	MSI2		27.32481531	5550
chr17.fa	55916287	55927433	MRPS23		52.16718578	5168
chr17.fa	55940337	55980750	CUEDC1		7.829121649	1140
chr17.fa	56048910	56065615	VEZF1		14.57628712	4484
chr17.fa	56078280	56084707	SRSF1		18.66103314	6992
chr17.fa	56160780	56167618	DYNLL2		165.4493831	16720
chr17.fa	56232515	56233447	OR4D1		0	0
chr17.fa	56234320	56236480	MSX2P1		0	0
chr17.fa	56247017	56247940	OR4D2		0	0
chr17.fa	56270089	56282535	EPX		0.421246023	76
chr17.fa	56282797	56296966	MKS1		2.251414657	380
chr17.fa	56315787	56345879	LPO		0.384196855	76
chr17.fa	56347217	56358296	MPO		0	0
chr17.fa	56378594	56406152	BZRAP1		1.636790052	836
chr17.fa	56408593	56408679	MIR142		0	0
chr17.fa	56422539	56429563	SUPT4H1		44.70659793	4420
chr17.fa	56431038	56494931	RNF43		6.781804937	2052
chr17.fa	56497529	56565759	HSF5		0.557394183	152
chr17.fa	56566893	56595251	MTMR4		35.14926816	13604
chr17.fa	56597611	56618179		4-Sep	0.987224769	152
chr17.fa	56618948	56621683	C17orf47		0	0
chr17.fa	56634039	56769416	TEX14		0.464620658	152
chr17.fa	56769963	56811692	RAD51C		16.77032075	1589
chr17.fa	56833232	57062534	PPM1E		1.46419515	608
chr17.fa	57075561	57184266	TRIM37		10.13821855	3040
chr17.fa	57187308	57232800	SKA2		4.000858276	836
chr17.fa	57215119	57215233	MIR454		0	0

chr17.fa	57228497	57228582	MIR301A		0	0
chr17.fa	57232860	57284070	PRR11		0	0
chr17.fa	57287371	57292611	C17orf71		5.607376447	1216
chr17.fa	57297828	57353330	GDPD1		0	0
chr17.fa	57409053	57479095	YPEL2		15.31395713	5320
chr17.fa	57642886	57685713	DHX40		15.54408367	3697
chr17.fa	57697050	57774317	CLTC		113.0592999	64372
chr17.fa	57774667	57784856	PTRH2		12.90907458	684
chr17.fa	57784863	57917952	TMEM49		15.26847401	2204
chr17.fa	57918627	57918698	MIR21		0	0
chr17.fa	57936841	57970306	TUBD1		3.520574555	608
chr17.fa	57970443	58027786	RPS6KB1		6.856355091	2421
chr17.fa	58029723	58042117	RNFT1		2.270993485	304
chr17.fa	58053385	58096336	DHX40P1		0	0
chr17.fa	58120552	58156292	HEATR6		10.47166105	2736
chr17.fa	58160927	58165828	LOC645638		40.54971145	1672
chr17.fa	58179121	58180280	LOC653653		0	0
chr17.fa	58227302	58236906	CA4		2.975530094	228
chr17.fa	58254691	58469586	USP32		18.2180998	8499
chr17.fa	58308877	58309006	SCARNA20		0	0
chr17.fa	58499865	58508787	C17orf64		0	0
chr17.fa	58520520	58603580	APPBP2		16.8115869	7220
chr17.fa	58677544	58743640	PPM1D		2.396147381	760
chr17.fa	58755172	59470199	BCAS3		16.09138722	3856
chr17.fa	59477257	59486827	TBX2		35.55741143	7980
chr17.fa	59489112	59490641	C17orf82		0	0
chr17.fa	59533807	59561664	TBX4		0	0
chr17.fa	59667794	59668563	NACA2		0	0
chr17.fa	59756547	59940920	BRIP1		0.140214532	76
chr17.fa	59942728	60005377	INTS2		5.572586375	2280
chr17.fa	60019966	60142643	MED13		29.42185843	20444
chr17.fa	60342067	60353016	TBC1D3P2		0	0
chr17.fa	60447579	60493839	EFCAB3		0	0
chr17.fa	60501246	60527454	METTL2A		4.526022695	757
chr17.fa	60556386	60692841	TLK2		11.103756	4010
chr17.fa	60704762	60770952	MRC2		0	0
chr17.fa	60778675	60885705		10-Mar	0	0
chr17.fa	60798858	61268734	MIR548W		0	0
chr17.fa	61086898	61505067	TANC2		39.6573687	30932
chr17.fa	61509665	61523722	CYB561		7.425948391	1672
chr17.fa	61554422	61575741	ACE		1.973847113	684
chr17.fa	61600695	61626338	KCNH6		3.197222674	760
chr17.fa	61627822	61671642	DCAF7		34.94158197	14744

chr17.fa	61678231	61685725	TACO1	15.58354254	1520
chr17.fa	61699801	61773248	MAP3K3	6.497610713	1900
chr17.fa	61773671	61777519	LIMD2	0.816738356	152
chr17.fa	61780192	61819330	STRADA	6.206036776	963
chr17.fa	61822610	61851088	CCDC47	8.369497312	1900
chr17.fa	61851567	61896677	DDX42	47.78438975	12669
chr17.fa	61896793	61905031	FTSJ3	11.92998256	2736
chr17.fa	61905044	61909387	PSMC5	101.3344432	10059
chr17.fa	61909441	61920351	SMARCD2	34.2969867	6308
chr17.fa	61934376	61941739	TCAM1P	0	0
chr17.fa	61949372	61951089	CSH2	0	0
chr17.fa	61957572	61959223	GH2	0	0
chr17.fa	61972273	61973987	CSH1	0	0
chr17.fa	61986965	61988618	CSHL1	0	0
chr17.fa	61994563	61996198	GH1	0	0
chr17.fa	62006098	62009704	CD79B	0	0
chr17.fa	62015914	62050278	SCN4A	0	0
chr17.fa	62075711	62081644	C17orf72	0	0
chr17.fa	62082467	62097994	ICAM2	0	0
chr17.fa	62120390	62207502	ERN1	2.57220623	684
chr17.fa	62223438	62223517	SNORD104	0	0
chr17.fa	62223699	62223831	SNORA76	0	0
chr17.fa	62224795	62340653	TEX2	9.89438683	3344
chr17.fa	62396777	62407083	PECAM1	0	0
chr17.fa	62461569	62464760	C17orf60	0	0
chr17.fa	62473902	62493184	POLG2	1.436182365	152
chr17.fa	62494374	62502484	DDX5	114.4953317	28577
chr17.fa	62503158	62534062	CCDC45	17.02650219	3040
chr17.fa	62540735	62658386	SMURF2	9.474195051	2432
chr17.fa	62745780	62778117	LOC146880	1.38241589	456
chr17.fa	62780959	62833302	PLEKHM1P	2.763777535	532
chr17.fa	62850488	62914903	LRRC37A3	1.010267544	380
chr17.fa	62962668	62971703	AMZ2P1	1.330155479	304
chr17.fa	63005407	63052920	GNA13	14.14660714	5928
chr17.fa	63133456	63223821	RGS9	0	0
chr17.fa	63524683	63557740	AXIN2	4.597861935	1292
chr17.fa	63631658	64188212	CCDC46	3.130504051	836
chr17.fa	64208147	64225556	APOH	1.906074246	152
chr17.fa	64298926	64806862	PRKCA	4.045889581	2356
chr17.fa	64783190	64783286	MIR634	0	0
chr17.fa	64873391	64881395	CACNG5	0	0
chr17.fa	64960980	65029518	CACNG4	17.6863087	4028
chr17.fa	65040652	65052911	CACNG1	0	0

chr17.fa	65066554	65241319	HELZ	10.97694543	10080
chr17.fa	65336619	65362721	PSMD12	22.93042258	2736
chr17.fa	65373924	65689647	PITPNC1	6.965845924	975
chr17.fa	65714061	65740266	NOL11	17.63826527	2874
chr17.fa	65736786	65736917	SNORA38B	0	0
chr17.fa	65821780	65980494	BPTF	34.15978429	25612
chr17.fa	65987217	65989765	C17orf58	4.391079386	456
chr17.fa	66031848	66042970	KPNA2	9.249339738	1216
chr17.fa	66097696	66132070	LOC100499466	7.362844321	5472
chr17.fa	66194801	66196443	LOC440461	0	0
chr17.fa	66244145	66253305	AMZ2	27.74801921	3648
chr17.fa	66255323	66417000	ARSG	0	0
chr17.fa	66263167	66287405	SLC16A6	0	0
chr17.fa	66417422	66453653	WIPI1	24.39130439	3116
chr17.fa	66420592	66420689	MIR635	11.67967476	76
chr17.fa	66508110	66528910	PRKAR1A	77.53065431	19526
chr17.fa	66531257	66597095	FAM20A	0.261753875	76
chr17.fa	66863431	66951533	ABCA8	0	0
chr17.fa	66970773	67057136	ABCA9	0	0
chr17.fa	67074847	67138015	ABCA6	0	0
chr17.fa	67144148	67225001	ABCA10	0.18931221	76
chr17.fa	67240576	67323323	ABCA5	1.384674985	836
chr17.fa	67410838	67538470	MAP2K6	1.837247134	228
chr17.fa	68071426	68131746	KCNJ16	7.312993611	2052
chr17.fa	68163102	68165543	FLJ36644	0	0
chr17.fa	68165676	68176183	KCNJ2	0	0
chr17.fa	70117161	70122560	SOX9	25.02189327	6536
chr17.fa	70399463	70588943	LOC100499467	4.564276714	684
chr17.fa	70594180	70636611	FLJ26484	0.505284378	76
chr17.fa	70642085	71088853	SLC39A11	1.645675828	304
chr17.fa	71161160	71168062	SSTR2	0	0
chr17.fa	71189173	71203395	COG1	14.90686811	2812
chr17.fa	71203492	71228533	FAM104A	12.74144969	2280
chr17.fa	71228776	71245095	C17orf80	4.298456467	1064
chr17.fa	71248769	71258019	CPSF4L	0	0
chr17.fa	71279763	71308143	CDC42EP4	19.93184971	4104
chr17.fa	71330523	71640227	SDK2	0.213559836	152
chr17.fa	71745409	71824676	C17orf54	0	0
chr17.fa	72199795	72206019	RPL38	1144.566258	27131
chr17.fa	72206135	72209460	MGC16275	1.381813464	228
chr17.fa	72209696	72258157	TTYH2	0	0
chr17.fa	72270386	72311023	DNAI2	0	0
chr17.fa	72322351	72351959	KIF19	0	0

chr17.fa	72352555	72357958	BTBD17	0.682096259	76
chr17.fa	72363645	72368739	GPR142	0	0
chr17.fa	72427667	72443568	GPRC5C	5.172877063	836
chr17.fa	72462522	72480933	CD300A	0	0
chr17.fa	72517313	72527613	CD300LB	0	0
chr17.fa	72537247	72542282	CD300C	0	0
chr17.fa	72576111	72584988	CD300LD	0	0
chr17.fa	72581057	72590348	C17orf77	0	0
chr17.fa	72606022	72619897	CD300E	0	0
chr17.fa	72667256	72743474	RAB37	0.953037123	228
chr17.fa	72690452	72709108	CD300LF	0	0
chr17.fa	72744752	72744762	MIR3615	0	0
chr17.fa	72744839	72765499	SLC9A3R1	68.45240341	8763
chr17.fa	72766686	72772470	NAT9	18.26554081	2274
chr17.fa	72772622	72835922	TMEM104	0.482090997	152
chr17.fa	72838168	72856007	GRIN2C	0	0
chr17.fa	72858619	72869156	FDXR	4.255081832	532
chr17.fa	72873473	72889705	FADS6	0.557846002	76
chr17.fa	72912176	72919351	USH1G	0	0
chr17.fa	72920370	72930006	OTOP2	0	0
chr17.fa	72931897	72945511	OTOP3	0	0
chr17.fa	72946839	72968900	C17orf28	35.27668115	7828
chr17.fa	72983727	73001892	CDR2L	7.451400867	1748
chr17.fa	73008780	73017356	ICT1	20.68051399	1207
chr17.fa	73034955	73043074	ATP5H	169.0326099	6981
chr17.fa	73043279	73061981	KCTD2	16.40464848	4028
chr17.fa	73084055	73102248	SLC16A5	10.70284184	1368
chr17.fa	73106082	73126322	ARMC7	5.481770733	760
chr17.fa	73126361	73127877	NT5C	13.70969805	822
chr17.fa	73131344	73150774	HN1	31.70279194	3568
chr17.fa	73163825	73179098	SUMO2	47.23045951	3343
chr17.fa	73201597	73231854	NUP85	6.934067979	1064
chr17.fa	73232694	73258444	GGA3	13.03211999	3572
chr17.fa	73257749	73262309	MRPS7	44.26667672	3724
chr17.fa	73262458	73267303	MIF4GD	4.531293918	380
chr17.fa	73267380	73269976	LOC100287042	0	0
chr17.fa	73273434	73285530	SLC25A19	1.067799178	76
chr17.fa	73314157	73401790	GRB2	45.03597406	9880
chr17.fa	73402150	73402243	MIR3678	0	0
chr17.fa	73452664	73496341	KIAA0195	3.039537802	1064
chr17.fa	73496534	73511627	CASKIN2	3.244513075	1064
chr17.fa	73512609	73520820	TSEN54	8.255789501	1064
chr17.fa	73521783	73571290	LLGL2	18.41825567	4339

chr17.fa	73584139	73622927	MYO15B	0.608449743	380
chr17.fa	73622950	73663269	RECQL5	4.190622304	1672
chr17.fa	73629514	73637486	LOC643008	3.354003907	1140
chr17.fa	73642646	73644057	LOC100130933	2.764681173	152
chr17.fa	73663399	73704139	SAP30BP	9.853120684	1648
chr17.fa	73717516	73753899	ITGB4	3.498736631	1412
chr17.fa	73754018	73761280	GALK1	1.681971964	152
chr17.fa	73772515	73775860	H3F3B	99.01600875	17784
chr17.fa	73780681	73821886	UNK	7.632580333	2052
chr17.fa	73823308	73840798	UNC13D	12.98211867	3785
chr17.fa	73841780	73851501	WBP2	74.77636497	9652
chr17.fa	73870245	73874656	TRIM47	3.540605202	532
chr17.fa	73885041	73893054	TRIM65	3.693320064	836
chr17.fa	73894724	73901181	MRPL38	17.44172396	1824
chr17.fa	73906618	73937119	FBF1	0.889180021	228
chr17.fa	73937589	73975300	ACOX1	85.42965731	43700
chr17.fa	73975516	73996667	C17orf106	22.0398871	1140
chr17.fa	73996987	74002080	CDK3	2.187105736	228
chr17.fa	74002927	74023507	EVPL	289.4011383	126574
chr17.fa	74035191	74068607	SRP68	43.18773267	7212
chr17.fa	74070892	74073573	GALR2	0	0
chr17.fa	74075263	74076505	ZACN	0	0
chr17.fa	74077086	74099868	EXOC7	34.62967618	9517
chr17.fa	74132415	74137380	FOXJ1	0	0
chr17.fa	74138534	74236390	RNF157	1.63814551	532
chr17.fa	74261286	74267379	FAM100B	23.63827253	2494
chr17.fa	74270130	74303761	QRICH2	0.854389949	304
chr17.fa	74306868	74350230	PRPSAP1	15.0415102	2356
chr17.fa	74380690	74383941	SPHK1	0	0
chr17.fa	74385613	74449288	UBE2O	10.22602206	3636
chr17.fa	74449433	74466199	AANAT	0	0
chr17.fa	74466975	74497509	RHBDF2	0.325309764	76
chr17.fa	74523440	74533782	CYGB	0	0
chr17.fa	74536121	74541458	PRCD	0	0
chr17.fa	74554874	74554951	SNORD1C	0	0
chr17.fa	74557190	74557275	SNORD1B	0	0
chr17.fa	74557715	74557788	SNORD1A	0	0
chr17.fa	74561461	74582145	ST6GALNAC2	1.631217617	228
chr17.fa	74620845	74639894	ST6GALNAC1	0.449861234	76
chr17.fa	74671809	74707056	MXRA7	2.350363043	608
chr17.fa	74708914	74722881	JMJD6	3.205656631	1216
chr17.fa	74722950	74729962	C17orf95	14.58095592	1064
chr17.fa	74730199	74733493	SRSF2	27.1971011	5320

chr17.fa	74732547	74732630	MIR636	0	0
chr17.fa	74733783	74775338	MFSD11	3.650999673	608
chr17.fa	74864798	74946471	MGAT5B	0.25738629	76
chr17.fa	75084725	75091068	C17orf86	2.110898912	304
chr17.fa	75085389	75085575	SCARNA16	0	0
chr17.fa	75137005	75213181	SEC14L1	23.2878115	8755
chr17.fa	75277492	75496678	9-Sep	24.51389798	10702
chr17.fa	75393066	75393136	MIR4316	0	0
chr17.fa	75875083	75880169	FLJ45079	0	0
chr17.fa	76000318	76104916	TNRC6C	4.042275028	2625
chr17.fa	76108999	76128439	TMC6	5.902113117	1140
chr17.fa	76126859	76139049	TMC8	0	0
chr17.fa	76142434	76162364	C17orf99	0	0
chr17.fa	76164671	76169009	SYNGR2	10.13520642	1140
chr17.fa	76170160	76183285	TK1	0.717639363	76
chr17.fa	76183398	76203782	AFMID	15.89620137	2264
chr17.fa	76210277	76221716	BIRC5	0	0
chr17.fa	76219545	76220144	EPR1	0	0
chr17.fa	76227391	76237068	LOC283999	0	0
chr17.fa	76352859	76356158	SOCS3	1.255906537	228
chr17.fa	76374735	76420639	PGS1	7.609236345	1060
chr17.fa	76419778	76573476	DNAH17	0	0
chr17.fa	76670130	76778376	CYTH1	23.80318651	5233
chr17.fa	76792965	76836969	USP36	9.840921568	3420
chr17.fa	76849059	76921472	TIMP2	3.131558296	760
chr17.fa	76967335	76976061	LGALS3BP	8.103375852	1216
chr17.fa	76987798	77005899	CANT1	19.41135409	4484
chr17.fa	77020251	77045870	C1QTNF1	0	0
chr17.fa	77071019	77084681	ENGASE	9.177199286	2736
chr17.fa	77085427	77478563	RBFOX3	0	0
chr17.fa	77704882	77716021	ENPP7	0.621401891	76
chr17.fa	77751977	77761449	CBX2	0	0
chr17.fa	77768176	77770915	CBX8	0.740380925	76
chr17.fa	77806955	77813213	CBX4	6.867951782	1208
chr17.fa	77913819	78009647	TBC1D16	27.39469666	5928
chr17.fa	78010435	78074412	CCDC40	1.332866393	380
chr17.fa	78075355	78093679	GAA	0.956501069	228
chr17.fa	78109013	78120982	EIF4A3	48.63004453	5599
chr17.fa	78152281	78182851	CARD14	2.266625901	608
chr17.fa	78183079	78194199	SGSH	2.481089374	456
chr17.fa	78194200	78227308	SLC26A11	1.874597514	380
chr17.fa	78234667	78370086	RNF213	19.46316268	26225
chr17.fa	78325631	78388966	LOC100294362	0	0

chr17.fa	78388969	78411884	FLJ35220	4.774824422	1064
chr17.fa	78440633	78450404	NPTX1	0	0
chr17.fa	78518625	78940173	RPTOR	13.02202936	5928
chr17.fa	78965641	78973933	CHMP6	15.06063721	1664
chr17.fa	79002933	79008519	FLJ90757	9.579468906	2812
chr17.fa	79008947	79091095	BAIAP2	16.26292788	5140
chr17.fa	79091233	79139872	AATK	0.442481521	152
chr17.fa	79099076	79099173	MIR657	0	0
chr17.fa	79099677	79099755	MIR3065	0	0
chr17.fa	79106996	79107108	MIR1250	0	0
chr17.fa	79139307	79156964	LOC388428	0	0
chr17.fa	79163393	79196751	AZI1	1.581366907	380
chr17.fa	79202077	79212891	C17orf56	2.502475479	380
chr17.fa	79213111	79215098	C17orf89	51.09863357	1900
chr17.fa	79218799	79269096	SLC38A10	2.329278151	760
chr17.fa	79276624	79283048	C17orf55	0	0
chr17.fa	79285072	79304474	TMEM105	0	0
chr17.fa	79373540	79433358	BAHCC1	1.237231347	836
chr17.fa	79418130	79418214	MIR3186	0	0
chr17.fa	79476997	79479892	ACTG1	674.2587038	94240
chr17.fa	79495417	79504156	FSCN2	1.314191203	152
chr17.fa	79506911	79519429	C17orf70	5.650148657	1444
chr17.fa	79523913	79604138	NPLOC4	26.64573118	7751
chr17.fa	79609349	79615779	TSPAN10	0	0
chr17.fa	79617489	79623607	PDE6G	0	0
chr17.fa	79632066	79633618	C17orf90	16.61534679	684
chr17.fa	79633761	79640936	CCDC137	5.972747506	836
chr17.fa	79648224	79650954	ARL16	3.793172088	304
chr17.fa	79650962	79669151	HGS	19.69238558	3886
chr17.fa	79670400	79674556	MRPL12	28.19230801	1900
chr17.fa	79679371	79688042	SLC25A10	25.64706032	3210
chr17.fa	79762010	79771889	GCGR	0.558147215	76
chr17.fa	79780293	79791167	FAM195B	22.40676423	1824
chr17.fa	79791368	79792926	DYSFIP1	0	0
chr17.fa	79801034	79818544	P4HB	63.93466406	10944
chr17.fa	79825597	79829282	ARHGDIA	82.7679909	12008
chr17.fa	79845711	79849462	THOC4	20.86801892	1520
chr17.fa	79849599	79858363	ANAPC11	32.02885474	2888
chr17.fa	79860072	79860776	NPB	1.870229929	76
chr17.fa	79860782	79869340	PCYT2	21.20582901	5014
chr17.fa	79869815	79876058	SIRT7	5.951210795	684
chr17.fa	79876146	79885588	MAFG	7.458178154	2584
chr17.fa	79885705	79888629	LOC92659	0	0

chr17.fa	79890267	79894968	PYCR1	21.1038685	2888
chr17.fa	79897521	79905109	MYADML2	0	0
chr17.fa	79910383	79919057	NOTUM	0	0
chr17.fa	79935426	79975282	ASPSCR1	6.709062059	821
chr17.fa	79976579	79980773	STRA13	65.79224306	3189
chr17.fa	79981280	79989027	LRRC45	5.284777598	912
chr17.fa	79989532	79992080	RAC3	6.478935523	456
chr17.fa	79993757	79995573	DCXR	249.4612321	13632
chr17.fa	80005778	80009650	RFNG	2.504583968	304
chr17.fa	80009763	80015346	GPS1	25.7905882	3192
chr17.fa	80015751	80023680	DUS1L	32.99770553	4084
chr17.fa	80036214	80056106	FASN	58.19129006	32680
chr17.fa	80059346	80170689	CCDC57	3.055351471	608
chr17.fa	80186293	80197369	SLC16A3	0	0
chr17.fa	80202240	80231573	CSNK1D	45.30706553	6152
chr17.fa	80272746	80275480	CD7	0	0
chr17.fa	80278900	80291921	SECTM1	2.006378089	304
chr17.fa	80317123	80321652	TEX19	0	0
chr17.fa	80332201	80333370	UTS2R	0	0
chr17.fa	80347086	80373503	C17orf101	3.105503393	884
chr17.fa	80376252	80400462	HEXDC	1.768118809	228
chr17.fa	80400517	80408707	C17orf62	3.54361733	684
chr17.fa	80416060	80446143	NARF	12.63241068	1748
chr17.fa	80477594	80562483	FOXK2	13.70397501	4788
chr17.fa	80572438	80606411	WDR45L	64.15469997	11020
chr17.fa	80614943	80656598	RAB40B	8.998730735	1051
chr17.fa	80674582	80685893	FN3KRP	13.79042307	1672
chr17.fa	80693452	80709073	FN3K	20.76756447	1976
chr17.fa	80709940	80901062	TBCD	13.65035914	4017
chr17.fa	80787310	80797931	ZNF750	19.7283805	4180
chr17.fa	80901667	81009686	B3GNTL1	1.655314636	152
chr17.fa	81037567	81052591	METRNL	12.88979696	1140
chr17.fa	81174666	81188573	FLJ43681	0.507392868	76
chr18.fa	109065	122222	ROCK1P1	0.930596773	152
chr18.fa	158483	213739	USP14	34.59669338	9547
chr18.fa	214520	268059	THOC1	9.661549378	1360
chr18.fa	319355	500729	COLEC12	1.826102262	380
chr18.fa	580369	581524	CETN1	0	0
chr18.fa	596998	650293	CLUL1	0	0
chr18.fa	649620	658340	C18orf56	0	0
chr18.fa	657604	673499	TYMS	0	0
chr18.fa	670324	712662	ENOSF1	9.400096716	2486
chr18.fa	721592	812327	YES1	15.39182063	4788

chr18.fa	904944	912173	ADCYAP1	0	0
chr18.fa	1268312	1359630	C18orf2	0	0
chr18.fa	2537524	2571489	METTL4	3.115443414	760
chr18.fa	2571510	2616634	NDC80	0.526971696	76
chr18.fa	2652722	2653231	LOC645158	2.244336157	76
chr18.fa	2655886	2805015	SMCHD1	13.31525997	7667
chr18.fa	2847028	2914090	EMILIN2	3.711694041	988
chr18.fa	2916992	3011945	LPIN2	11.02529008	4560
chr18.fa	2943213	2946621	LOC727896	0.335701603	76
chr18.fa	3066805	3220106	MYOM1	1.174579096	456
chr18.fa	3247528	3256234	MYL12A	149.0132579	12160
chr18.fa	3262111	3278282	MYL12B	317.7307993	24852
chr18.fa	3412072	3458406	TGIF1	17.59474003	2812
chr18.fa	3498837	3880135	DLGAP1	1.341902776	304
chr18.fa	3594112	3597377	FLJ35776	4.610663476	304
chr18.fa	4264602	4296000	LOC284215	0	0
chr18.fa	5143672	5197255	LOC642597	0	0
chr18.fa	5236723	5238028	C18orf18	0.876378479	76
chr18.fa	5238099	5246505	LOC339290	5.180557988	1368
chr18.fa	5289018	5296039	ZFP161	2.550820125	606
chr18.fa	5392388	5543986	EPB41L3	16.47663832	4864
chr18.fa	5890184	5892103	TMEM200C	0	0
chr18.fa	5954705	6414910	L3MBTL4	5.424690918	1292
chr18.fa	6374360	6374424	MIR4317	0	0
chr18.fa	6834432	6915712	ARHGAP28	2.897214781	1064
chr18.fa	6925473	6929868	LOC400643	0	0
chr18.fa	6941886	7117813	LAMA1	0.60152185	380
chr18.fa	7231137	7232042	LRRC30	0	0
chr18.fa	7567314	8406859	PTPRM	9.8109509	3648
chr18.fa	8360818	8367032	LOC100192426	0	0
chr18.fa	8609443	8639380	RAB12	11.24261507	1596
chr18.fa	8717369	8832775	KIAA0802	11.98028508	4846
chr18.fa	9102628	9134343	NDUFV2	84.92256566	5244
chr18.fa	9136758	9285206	ANKRD12	17.72139999	12388
chr18.fa	9334765	9402418	TWSG1	5.485385286	1368
chr18.fa	9475530	9538106	RALBP1	152.2113842	44004
chr18.fa	9546792	9614600	PPP4R1	44.34333536	11527
chr18.fa	9708228	9862553	RAB31	3.152341975	836
chr18.fa	9885723	9888156	TXNDC2	0	0
chr18.fa	9913955	9960018	VAPA	24.87565448	11552
chr18.fa	10454625	10488698	APCDD1	2.680642818	456
chr18.fa	10525873	10552766	NAPG	28.83208388	7112
chr18.fa	10670244	11148761	FAM38B	0	0

chr18.fa	11689136	11883144	GNAL	0.947916506	304
chr18.fa	11851389	11854448	CHMP1B	30.29838752	6156
chr18.fa	11883472	11908641	MPPE1	0.408143268	76
chr18.fa	11981455	12030876	IMPA2	37.39059219	3724
chr18.fa	12254318	12277594	CIDEA	0	0
chr18.fa	12308257	12326568	TUBB6	3.153245613	380
chr18.fa	12328943	12377275	AFG3L2	29.60499578	6308
chr18.fa	12407895	12432236	SLMO1	0	0
chr18.fa	12446511	12657912	SPIRE1	11.80377442	4331
chr18.fa	12658738	12725739	PSMG2	32.47329414	2499
chr18.fa	12672631	12702703	CEP76	3.191650238	608
chr18.fa	12785480	12884334	PTPN2	8.475524198	1596
chr18.fa	12947983	12987536	SEH1L	10.44711222	2432
chr18.fa	12991361	13125051	CEP192	12.33586673	6533
chr18.fa	13218786	13652753	C18orf1	6.381794413	4256
chr18.fa	13663346	13726591	C18orf19	3.916368101	1140
chr18.fa	13726704	13764554	RNMT	12.729853	5243
chr18.fa	13825543	13826861	MC5R	0	0
chr18.fa	13882043	13915535	MC2R	0	0
chr18.fa	14075989	14132489	ZNF519	0.38690777	228
chr18.fa	14337422	14342523	LOC284233	0	0
chr18.fa	14477954	14498705	CXADRP3	0	0
chr18.fa	14511737	14543599	POTEC	0	0
chr18.fa	14748239	14852737	ANKRD30B	0	0
chr18.fa	14830165	14830241	MIR3156-2	0	0
chr18.fa	15313555	15325918	LOC644669	0	0
chr18.fa	18529703	18691812	ROCK1	20.95311152	9249
chr18.fa	18822203	19102791	GREB1L	1.323830011	532
chr18.fa	19109262	19180693	ESCO1	10.17662317	3040
chr18.fa	19192260	19210208	SNRPD1	16.45374616	1748
chr18.fa	19230858	19284766	ABHD3	0	0
chr18.fa	19263471	19263558	MIR320C1	0	0
chr18.fa	19321545	19450912	MIB1	16.46865619	10176
chr18.fa	19405659	19405746	MIR133A1	0	0
chr18.fa	19408965	19409049	MIR1-2	0	0
chr18.fa	19749416	19782227	GATA6	0.327568859	76
chr18.fa	19993564	19997878	CTAGE1	0	0
chr18.fa	20513295	20606449	RBBP8	17.25090568	4072
chr18.fa	20714528	20840434	CABLES1	3.069207258	1064
chr18.fa	20875979	21017925	C18orf45	3.060472088	608
chr18.fa	21032787	21063099	RIOK3	17.59624609	4712
chr18.fa	21083462	21110576	C18orf8	12.1980619	1632
chr18.fa	21111463	21166581	NPC1	2.703083167	836

chr18.fa	21179978	21242849	ANKRD29	1.510732519	228
chr18.fa	21269562	21535029	LAMA3	1.390548634	988
chr18.fa	21572737	21715574	TTC39C	6.750026992	2660
chr18.fa	21718955	21741564	CABYR	0	0
chr18.fa	21742011	21977790	OSBPL1A	12.47788854	3952
chr18.fa	21901650	21901699	MIR320C2	0	0
chr18.fa	22006609	22033494	IMPACT	16.70375273	4168
chr18.fa	22040593	22059921	HRH4	0	0
chr18.fa	22641888	22932214	ZNF521	0.460554286	152
chr18.fa	23596217	23670611	SS18	22.31926192	5092
chr18.fa	23713816	23773319	PSMA8	0	0
chr18.fa	23806409	23971650	TAF4B	1.686038336	532
chr18.fa	24034875	24220308	KCTD1	6.141727855	1672
chr18.fa	24267585	24283602	LOC728606	3.524640927	608
chr18.fa	24432008	24445716	AQP4	0	0
chr18.fa	24445272	24515910	C18orf16	0	0
chr18.fa	24495595	24765289	CHST9	2.038457247	304
chr18.fa	25530930	25757445	CDH2	1.048370956	304
chr18.fa	27878876	27878926	MIR302F	0	0
chr18.fa	28570052	28622781	DSC3	1.148222981	532
chr18.fa	28645942	28682388	DSC2	3.491658131	1216
chr18.fa	28709214	28742819	DSC1	0.268982981	76
chr18.fa	28898052	28937393	DSG1	0.210246495	76
chr18.fa	28956740	28993880	DSG4	0	0
chr18.fa	29027732	29058665	DSG3	0	0
chr18.fa	29078027	29128814	DSG2	15.18579111	5700
chr18.fa	29171730	29178986	TTR	0	0
chr18.fa	29202209	29264686	B4GALT6	2.377622797	760
chr18.fa	29339659	29340843	MCART2	0	0
chr18.fa	29409136	29523091	TRAPPC8	19.52777282	8109
chr18.fa	29598445	29653154	RNF125	1.555161399	608
chr18.fa	29671818	29711524	RNF138	2.499162139	608
chr18.fa	29769987	29800366	MEP1B	0	0
chr18.fa	29847477	30050447	FAM59A	25.97101463	5244
chr18.fa	30091626	30094597	WBP11P1	0	0
chr18.fa	30252634	30352974	KLHL14	1.343107627	380
chr18.fa	30517366	31020685	C18orf34	0.602576095	137
chr18.fa	31158541	31327399	ASXL3	1.647633711	836
chr18.fa	31431064	31803515	NOL4	0	0
chr18.fa	32073254	32471808	DTNA	0.106629311	76
chr18.fa	32556892	32722378	MAPRE2	3.998900393	988
chr18.fa	32820994	32838397	ZNF397	7.219165842	1520
chr18.fa	32831023	32870196	ZSCAN30	4.926635645	1368

chr18.fa	32870236	32890730	ZNF271	9.140752544	3116
chr18.fa	32912178	32924426	ZNF24	24.12186959	10108
chr18.fa	32946661	32957301	ZNF396	1.642211882	304
chr18.fa	33048291	33077955	INO80C	11.57229242	836
chr18.fa	33234533	33291798	GALNT1	14.9115369	3800
chr18.fa	33484781	33484889	MIR187	0	0
chr18.fa	33552588	33559241	C18orf21	11.0164043	760
chr18.fa	33569792	33647373	RPRD1A	30.31194209	8588
chr18.fa	33688494	33709357	SLC39A6	11.82260021	2888
chr18.fa	33709887	33754607	ELP2	54.73517504	9064
chr18.fa	33767480	33848685	MOCOS	5.036728903	912
chr18.fa	33877702	34360018	FHOD3	4.400567587	1444
chr18.fa	34376034	34409079	C18orf10	29.96961381	3952
chr18.fa	34414283	34805288	KIAA1328	2.22099217	704
chr18.fa	34823008	35146000	CELF4	0	0
chr18.fa	35237098	35237178	MIR4318	0	0
chr18.fa	36786888	37331959	LOC647946	0	0
chr18.fa	39060236	39100561	KC6	0.668541686	76
chr18.fa	39535199	39661446	PIK3C3	25.00442293	5092
chr18.fa	40323192	40695657	RIT2	0	0
chr18.fa	40847857	40857615	SYT4	0	0
chr18.fa	42260138	42648475	SETBP1	6.363571042	4636
chr18.fa	42550047	42550131	MIR4319	0	0
chr18.fa	43194766	43263060	SLC14A2	0	0
chr18.fa	43304092	43332485	SLC14A1	9.005206809	2653
chr18.fa	43405545	43422521	SIGLEC15	1.521124358	152
chr18.fa	43427574	43547305	KIAA1632	9.014092585	7600
chr18.fa	43563502	43652250	PSTPIP2	2.649617905	532
chr18.fa	43664110	43684199	ATP5A1	253.0497301	32680
chr18.fa	43684298	43708299	HAUS1	7.859995955	608
chr18.fa	43753988	43846955	C18orf25	10.8910998	3952
chr18.fa	43914187	44040783	RNF165	0	0
chr18.fa	44056935	44236996	LOXHD1	0.146991819	76
chr18.fa	44259081	44337039	ST8SIA5	1.309070587	228
chr18.fa	44392060	44497466	PIAS2	24.65366069	4022
chr18.fa	44526787	44628614	KATNAL2	1.616156979	304
chr18.fa	44542731	44544371	TCEB3CL	0	0
chr18.fa	44550299	44556449	TCEB3C	0	0
chr18.fa	44558943	44561988	TCEB3B	0	0
chr18.fa	44633781	44676871	HDHD2	35.62172035	5168
chr18.fa	44681413	44702745	IER3IP1	4.637170198	456
chr18.fa	45359466	45457512	SMAD2	15.44393043	10999
chr18.fa	45553639	45663680	ZBTB7C	19.75835117	5472

chr18.fa	46065427	46389586	KIAA0427	22.03446527	8512
chr18.fa	46446223	46477081	SMAD7	11.48554315	2432
chr18.fa	46570172	46987079	DYM	18.29280057	3192
chr18.fa	47013413	47013644	C18orf32	0	0
chr18.fa	47013743	47013792	MIR1539	0	0
chr18.fa	47014851	47018827	RPL17	26.12086797	666
chr18.fa	47015605	47015694	SNORD58C	0	0
chr18.fa	47017653	47017717	SNORD58A	0	0
chr18.fa	47018034	47018099	SNORD58B	69.37019864	304
chr18.fa	47088427	47119278	LIPG	0.552875992	152
chr18.fa	47309874	47340251	ACAA2	47.92309822	6154
chr18.fa	47340393	47340813	SCARNA17	0	0
chr18.fa	47349156	47721451	MYO5B	43.758079	27660
chr18.fa	47652869	47652933	MIR4320	0	0
chr18.fa	47753563	47792865	CCDC11	2.510156404	304
chr18.fa	47795216	47808144	MBD1	12.09007713	2617
chr18.fa	47808713	47814692	CXXC1	8.342689378	1643
chr18.fa	47901392	47920538	SKA1	0	0
chr18.fa	48086484	48258196	MAPK4	5.075284134	1596
chr18.fa	48321490	48351754	MRO	1.716762036	608
chr18.fa	48405432	48476162	ME2	13.12504412	4028
chr18.fa	48494387	48514490	ELAC1	7.176243026	1064
chr18.fa	48556583	48611411	SMAD4	15.39031456	8963
chr18.fa	48700920	48724051	MEX3C	9.671941218	2660
chr18.fa	49866542	51062273	DCC	0	0
chr18.fa	51680575	51751158	MBD2	30.04115184	6004
chr18.fa	51748654	51748782	SNORA37	0	0
chr18.fa	51795849	51824604	POLI	8.313471741	3344
chr18.fa	51851062	51880943	STARD6	0	0
chr18.fa	51885171	51908404	C18orf54	0	0
chr18.fa	52258390	52266724	C18orf26	0	0
chr18.fa	52495840	52557211	RAB27B	83.16634475	7372
chr18.fa	52568740	52626739	CCDC68	2.179123598	608
chr18.fa	52889562	53255860	TCF4	2.331687853	1290
chr18.fa	54270053	54305920	TXNL1	36.04432183	3420
chr18.fa	54318616	54697036	WDR7	11.00284973	5320
chr18.fa	54814293	54817639	BOD1P	0	0
chr18.fa	55019721	55036161	ST8SIA3	0	0
chr18.fa	55102917	55158530	ONECUT2	0.142021809	152
chr18.fa	55212073	55253969	FECH	9.278858587	4484
chr18.fa	55267894	55289177	NARS	54.73351837	10412
chr18.fa	55313659	55470327	ATP8B1	35.55756203	14017
chr18.fa	55711619	56068772	NEDD4L	96.32693198	61497

chr18.fa	56118306	56118390	MIR122	0	0
chr18.fa	56148482	56296189	ALPK2	0.783906167	380
chr18.fa	56338618	56417370	MALT1	12.25152716	4091
chr18.fa	56530061	56653709	ZNF532	52.23691653	22420
chr18.fa	56702971	56720446	LOC390858	8.622214804	304
chr18.fa	56807125	56826063	SEC11C	80.47124372	4173
chr18.fa	56887400	56898002	GRP	0	0
chr18.fa	56934267	56940625	RAX	0	0
chr18.fa	56962636	56985881	CPLX4	0	0
chr18.fa	56995056	57026508	LMAN1	8.99767649	2888
chr18.fa	57098171	57364644	CCBE1	0	0
chr18.fa	57567192	57571538	PMAIP1	0.591280617	76
chr18.fa	58038564	58040001	MC4R	0	0
chr18.fa	59157775	59222365	CDH20	0	0
chr18.fa	59482304	59560304	RNF152	21.56050702	2584
chr18.fa	59711458	59854289	PIGN	3.052339344	988
chr18.fa	59854524	59974355	KIAA1468	21.15778558	7662
chr18.fa	59992548	60053504	TNFRSF11A	1.825951656	379
chr18.fa	60190658	60245818	ZCCHC2	17.52500928	6450
chr18.fa	60382672	60647666	PHLPP1	14.65836759	6230
chr18.fa	60790579	60986613	BCL2	5.705722408	2508
chr18.fa	60994971	61034506	KDSR	2.202015767	760
chr18.fa	61056425	61089752	VPS4B	32.86773223	7372
chr18.fa	61144144	61172318	SERPINB5	5.216552911	912
chr18.fa	61223393	61234244	SERPINB12	0	0
chr18.fa	61254534	61266433	SERPINB13	0	0
chr18.fa	61304493	61311502	SERPINB4	0	0
chr18.fa	61322431	61329197	SERPINB3	0	0
chr18.fa	61370194	61391122	SERPINB11	29.77307249	3420
chr18.fa	61420277	61472603	SERPINB7	0	0
chr18.fa	61554939	61571124	SERPINB2	0	0
chr18.fa	61582745	61602476	SERPINB10	0	0
chr18.fa	61616588	61627645	HMSD	0	0
chr18.fa	61637263	61656608	SERPINB8	1.518413444	380
chr18.fa	61747243	61816260	C18orf20	0	0
chr18.fa	61771325	62090827	LOC284294	0	0
chr18.fa	61880318	61927290	LOC400654	0	0
chr18.fa	63417488	63548174	CDH7	0.387058376	76
chr18.fa	64171321	64271216	CDH19	0	0
chr18.fa	65173819	65183782	DSEL	1.258316239	760
chr18.fa	65183968	65566856	LOC643542	0	0
chr18.fa	66340925	66382353	TMX3	1.447327236	456
chr18.fa	66382491	66722426	CCDC102B	0.78420738	152

chr18.fa	67068284	67516322	DOK6	0.25738629	152
chr18.fa	67530193	67624232	CD226	0.434047564	76
chr18.fa	67671043	67872962	RTTN	1.129547791	532
chr18.fa	67956137	67997434	SOCS6	7.048528822	2736
chr18.fa	70203915	70211723	CBLN2	0	0
chr18.fa	70414787	70534810	NETO1	0.292025756	76
chr18.fa	70821293	70931733	LOC400655	0	0
chr18.fa	71740588	71815100	FBXO15	0.618690977	76
chr18.fa	71815746	71826204	C18orf55	9.70010461	988
chr18.fa	71920527	71959221	CYB5A	190.249132	10510
chr18.fa	71983110	72026422	DKFZP781G0119	0	0
chr18.fa	72102963	72124503	FAM69C	0	0
chr18.fa	72163500	72190689	CNDP2	101.3022135	36100
chr18.fa	72201692	72252261	CNDP1	1.562239898	228
chr18.fa	72259010	72265071	LOC400657	4.612771966	1064
chr18.fa	72342919	72777628	ZNF407	6.079527424	3648
chr18.fa	72909278	72921281	ZADH2	11.45647612	4180
chr18.fa	72922731	73001901	TSHZ1	5.991573302	1976
chr18.fa	73121827	73139589	C18orf62	0	0
chr18.fa	74069637	74207146	ZNF516	4.9090147	2812
chr18.fa	74240612	74271784	LOC284276	0	0
chr18.fa	74536116	74682682	ZNF236	6.059044957	3268
chr18.fa	74690789	74844774	MBP	11.77787012	5396
chr18.fa	74962008	74982096	GALR1	0	0
chr18.fa	76740275	76758193	SALL3	3.116798871	988
chr18.fa	76829397	77138282	ATP9B	6.768099757	1958
chr18.fa	77155772	77289323	NFATC1	2.346447278	760
chr18.fa	77439801	77514507	CTDP1	10.37783328	2584
chr18.fa	77623668	77659816	KCNG2	0	0
chr18.fa	77662420	77711653	PQLC1	5.848949068	988
chr18.fa	77724582	77730822	HSBP1L1	19.83982922	988
chr18.fa	77732867	77748532	TXNL4A	32.18443112	2964
chr18.fa	77794346	77810652	RBFA	1.8033607	680
chr18.fa	77866915	77898228	ADNP2	10.4112679	3724
chr18.fa	77905807	77936315	LOC100130522	0.306333361	76
chr18.fa	77915117	78005397	PARD6G	1.492659754	380
chr19.fa	60951	70966	WASH5P	0	0
chr19.fa	110679	111596	OR4F17	0	0
chr19.fa	197016	202209	FLJ45445	0	0
chr19.fa	281044	291435	PPAP2C	3.745429868	380
chr19.fa	305575	344791	MIER2	9.095269419	1668
chr19.fa	362057	376009	THEG	0	0
chr19.fa	405443	409170	C2CD4C	0	0

chr19.fa	416583	460996	SHC2	5.507373816	912
chr19.fa	463346	474983	ODF3L2	0	0
chr19.fa	496490	505343	MADCAM1	0	0
chr19.fa	507497	519654	C19orf20	3.082460618	228
chr19.fa	531733	542087	CDC34	9.505370571	912
chr19.fa	544027	549919	GZMM	0	0
chr19.fa	571325	583493	BSG	13.67445616	2052
chr19.fa	589893	617159	HCN2	0.33585221	76
chr19.fa	617223	633568	POLRMT	7.530318606	1900
chr19.fa	639926	643604	FGF22	2.231233403	76
chr19.fa	647526	663233	RNF126	5.5495436	608
chr19.fa	676389	683392	FSTL3	0	0
chr19.fa	685546	695461	PRSSL1	0	0
chr19.fa	708953	748330	PALM	5.973952357	1140
chr19.fa	751146	764318	C19orf21	7.226545554	1368
chr19.fa	797392	812327	PTBP1	40.92216101	8915
chr19.fa	812518	821952	LPPR3	0	0
chr19.fa	827831	832017	AZU1	0	0
chr19.fa	840985	848175	PRTN3	0	0
chr19.fa	852291	856246	ELANE	0	0
chr19.fa	859665	863610	CFD	0	0
chr19.fa	867962	893218	MED16	12.13510843	2356
chr19.fa	896503	913225	C19orf22	20.23908671	2431
chr19.fa	917342	921015	KISS1R	0	0
chr19.fa	926037	972803	ARID3A	1.62941034	304
chr19.fa	984328	994569	WDR18	12.47773793	1285
chr19.fa	1000437	1009649	GRIN3B	0	0
chr19.fa	1009724	1021141	C19orf6	4.780095645	836
chr19.fa	1026298	1039064	CNN2	15.83264548	2584
chr19.fa	1040102	1065570	ABCA7	0.167926105	76
chr19.fa	1067174	1086577	HMHA1	1.086474369	304
chr19.fa	1086628	1095391	POLR2E	25.9443573	4851
chr19.fa	1103936	1106787	GPX4	119.8549606	9701
chr19.fa	1107633	1174282	SBNO2	10.30373495	3496
chr19.fa	1205798	1228434	STK11	13.97566891	3040
chr19.fa	1229947	1237990	C19orf26	0	0
chr19.fa	1241749	1244824	ATP5D	47.41947051	2204
chr19.fa	1248552	1259142	MIDN	19.61497391	4940
chr19.fa	1267470	1270259	C19orf23	0	0
chr19.fa	1269267	1274809	CIRBP	246.9759257	26566
chr19.fa	1275520	1279243	C19orf24	10.23114268	608
chr19.fa	1286168	1299944	EFNA2	0	0
chr19.fa	1354976	1378430	MUM1	6.744002738	1887

chr19.fa	1383883	1395588	NDUFS7	24.698692	1289
chr19.fa	1397088	1401552	GAMT	16.658119	1512
chr19.fa	1407584	1435682	DAZAP1	27.18595623	4103
chr19.fa	1438363	1440492	RPS15	409.5585165	14440
chr19.fa	1450148	1473199	APC2	0.113255992	76
chr19.fa	1473244	1479228	C19orf25	8.275518936	1216
chr19.fa	1481427	1490407	PCSK4	0	0
chr19.fa	1491165	1497924	REEP6	8.336514516	760
chr19.fa	1505017	1513188	ADAMTSL5	0.40061295	76
chr19.fa	1524078	1535455	PLK5P	0	0
chr19.fa	1554668	1568057	MEX3D	11.24532599	2128
chr19.fa	1576678	1592652	MBD3	14.76047871	2508
chr19.fa	1597154	1605483	UQCR11	64.30380028	5700
chr19.fa	1609292	1652326	TCF3	13.87777477	4306
chr19.fa	1753662	1775444	ONECUT3	0	0
chr19.fa	1782074	1812270	ATP8B3	0	0
chr19.fa	1815245	1848452	REXO1	2.303072643	684
chr19.fa	1816158	1816223	MIR1909	0	0
chr19.fa	1822088	1824542	LOC100288123	0	0
chr19.fa	1852398	1863564	KLF16	7.136482943	1368
chr19.fa	1876975	1885518	FAM108A1	13.34041123	1520
chr19.fa	1905373	1926012	SCAMP4	5.028294946	836
chr19.fa	1911889	1913446	ADAT3	2.938631533	304
chr19.fa	1941161	1981336	CSNK1G2	11.66371049	2249
chr19.fa	1952526	1954548	C19orf34	0	0
chr19.fa	1985447	2015702	BTBD2	42.84630802	7448
chr19.fa	2037470	2051243	MKNK2	43.85296101	11021
chr19.fa	2071035	2096269	MOBKL2A	12.37035559	2736
chr19.fa	2096868	2099583	IZUMO4	4.510811452	304
chr19.fa	2100993	2151556	AP3D1	53.26796776	17734
chr19.fa	2164148	2232577	DOT1L	6.920664012	3417
chr19.fa	2233155	2236328	PLEKHJ1	25.43575958	2052
chr19.fa	2234061	2234148	MIR1227	0	0
chr19.fa	2236816	2248678	SF3A2	18.00890755	1973
chr19.fa	2249113	2252072	AMH	0	0
chr19.fa	2250638	2250650	MIR4321	0	0
chr19.fa	2252250	2256422	JSRP1	0	0
chr19.fa	2269520	2273487	OAZ1	312.6199721	23788
chr19.fa	2274631	2282181	C19orf35	0	0
chr19.fa	2289774	2308156	LINGO3	0	0
chr19.fa	2321520	2328614	LSM7	19.81046098	684
chr19.fa	2328629	2355100	SPPL2B	0.91538553	228
chr19.fa	2389784	2425491	TMPRSS9	0	0

chr19.fa	2425622	2427875	TIMM13	21.05462022	2508
chr19.fa	2428164	2456958	LMNB2	9.630976285	2964
chr19.fa	2476123	2478257	GADD45B	20.81108971	1900
chr19.fa	2511218	2702746	GNG7	5.930577722	1672
chr19.fa	2714565	2721390	DIRAS1	3.031555665	684
chr19.fa	2732523	2740074	SLC39A3	0.870053012	228
chr19.fa	2754712	2783354	SGTA	13.84403894	2128
chr19.fa	2785506	2813599	THOP1	13.46601694	2280
chr19.fa	2819872	2836733	ZNF554	2.785615459	684
chr19.fa	2841433	2860472	ZNF555	1.741009662	988
chr19.fa	2867333	2878501	ZNF556	0.701373875	76
chr19.fa	2900896	2918474	ZNF57	4.57150582	608
chr19.fa	2933216	2944969	ZNF77	2.805344894	380
chr19.fa	2977536	2995182	TLE6	2.269939241	304
chr19.fa	2997636	3047633	TLE2	28.89759765	5597
chr19.fa	3052908	3062964	AES	278.4044634	35862
chr19.fa	3094408	3121454	GNA11	19.32731574	2052
chr19.fa	3136191	3163766	GNA15	9.663657868	1368
chr19.fa	3178736	3180330	S1PR4	0	0
chr19.fa	3185875	3209573	NCLN	1.226538295	304
chr19.fa	3224701	3297073	CELF5	0	0
chr19.fa	3359616	3463603	NFIC	27.08866452	4484
chr19.fa	3474405	3480540	C19orf77	41.59913665	2508
chr19.fa	3490819	3500938	DOHH	5.00073398	684
chr19.fa	3506295	3536755	FZR1	7.236636181	1737
chr19.fa	3538263	3557571	C19orf28	4.06170325	760
chr19.fa	3539155	3544028	C19orf71	0	0
chr19.fa	3572943	3579081	HMG20B	49.00415076	5154
chr19.fa	3585569	3593539	GIPC3	0	0
chr19.fa	3594504	3606831	TBXA2R	0	0
chr19.fa	3610627	3626813	C19orf29	4.105981523	988
chr19.fa	3630179	3700477	PIP5K1C	8.67718613	2928
chr19.fa	3728374	3750682	TJP3	15.94560026	3027
chr19.fa	3750771	3761673	APBA3	2.121591964	304
chr19.fa	3762665	3767563	MRPL54	38.58234041	1596
chr19.fa	3769089	3772219	RAX2	0	0
chr19.fa	3777967	3801810	MATK	0	0
chr19.fa	3804022	3869027	ZFR2	0.399859918	152
chr19.fa	3880618	3928080	ATCAY	0	0
chr19.fa	3933101	3942414	ITGB1BP3	0	0
chr19.fa	3958452	3969826	DAPK3	10.62467713	1485
chr19.fa	3961412	3961510	MIR637	0	0
chr19.fa	3976054	3985461	EEF2	1000.354482	209760

chr19.fa	3982505	3982570	SNORD37	0	0
chr19.fa	4007749	4038067	PIAS4	5.271674844	608
chr19.fa	4045216	4066816	ZBTB7A	24.22729405	7144
chr19.fa	4090320	4124126	MAP2K2	35.42246812	4076
chr19.fa	4153629	4173048	CREB3L3	0	0
chr19.fa	4174106	4182596	SIRT6	4.879044031	530
chr19.fa	4183351	4224811	ANKRD24	0.284344831	76
chr19.fa	4229540	4237524	EBI3	0	0
chr19.fa	4247111	4269085	CCDC94	9.012737127	836
chr19.fa	4278598	4290720	SHD	0	0
chr19.fa	4292225	4302428	TMIGD2	0	0
chr19.fa	4304591	4323843	FSD1	0	0
chr19.fa	4324040	4338847	STAP2	40.71432422	3782
chr19.fa	4343524	4360083	MPND	11.72500728	1290
chr19.fa	4360364	4400415	SH3GL1	27.58807525	4193
chr19.fa	4402660	4443394	CHAF1A	13.01073388	2888
chr19.fa	4445003	4457791	UBXN6	58.00574301	7676
chr19.fa	4472255	4502191	HDGFRP2	15.57631343	2356
chr19.fa	4502223	4517716	PLIN4	4.888381627	2052
chr19.fa	4522544	4535208	PLIN5	5.157967033	836
chr19.fa	4537227	4540036	LRG1	0.643089209	76
chr19.fa	4542600	4559771	SEMA6B	0	0
chr19.fa	4639527	4655580	TNFAIP8L1	0.867191491	228
chr19.fa	4657557	4670415	C19orf10	10.89064798	760
chr19.fa	4675244	4723855	DPP9	8.505796079	2411
chr19.fa	4769117	4772568	C19orf30	0	0
chr19.fa	4770682	4770791	MIR7-3	0	0
chr19.fa	4791728	4795571	FEM1A	16.67483631	4256
chr19.fa	4815938	4831737	TICAM1	5.504964114	988
chr19.fa	4838346	4867780	PLIN3	19.56602684	3040
chr19.fa	4890449	4902879	ARRDC5	0	0
chr19.fa	4909510	4962165	UHRF1	0	0
chr19.fa	4969124	5153608	KDM4B	18.18135185	6764
chr19.fa	5205519	5340814	PTPRS	4.050558378	1976
chr19.fa	5455426	5456867	ZNRF4	0	0
chr19.fa	5558178	5568005	PLAC2	10.84787577	2660
chr19.fa	5587010	5622938	SAFB2	22.13702821	4877
chr19.fa	5623164	5668489	SAFB	29.91464248	5935
chr19.fa	5678433	5680911	C19orf70	16.53326632	988
chr19.fa	5681035	5688533	HSD11B1L	0.663872888	76
chr19.fa	5690272	5691678	RPL36	456.7062932	18316
chr19.fa	5691845	5720176	LONP1	16.5564597	3420
chr19.fa	5720688	5778742	TMEM146	0	0

chr19.fa	5782971	5784776	PRR22	0.822310792	76
chr19.fa	5785153	5791249	DUS3L	1.084365879	152
chr19.fa	5823818	5828335	NRTN	0	0
chr19.fa	5830637	5839742	FUT6	5.837954803	1216
chr19.fa	5842899	5851485	FUT3	0.441879096	76
chr19.fa	5865837	5870551	FUT5	0	0
chr19.fa	5891287	5904024	NDUFA11	19.08980949	3724
chr19.fa	5904852	5910263	VMAC	6.16853579	684
chr19.fa	5914193	5916151	CAPS	54.32793541	5548
chr19.fa	5916223	5978320	RANBP3	15.07870998	3333
chr19.fa	5993175	6110664	RFX2	0.278320576	76
chr19.fa	6135710	6193112	ACSBG2	0.421999055	76
chr19.fa	6210392	6279959	MLLT1	19.5604544	5851
chr19.fa	6306510	6333640	ACER1	1.051985509	76
chr19.fa	6361463	6368915	CLPP	24.86119627	1900
chr19.fa	6372444	6375261	ALKBH7	24.07894678	2128
chr19.fa	6375305	6375860	PSPN	0	0
chr19.fa	6379580	6393291	GTF2F1	19.72461534	3341
chr19.fa	6413119	6424822	KHSRP	41.95336284	8992
chr19.fa	6416421	6416500	MIR3940	0	0
chr19.fa	6426048	6433790	SLC25A41	0	0
chr19.fa	6440075	6459781	SLC25A23	38.37420241	8816
chr19.fa	6464260	6467217	CRB3	20.94437635	1520
chr19.fa	6467231	6481798	DENND1C	13.57731505	2508
chr19.fa	6494330	6502330	TUBB4	0	0
chr19.fa	6531010	6535939	TNFSF9	0	0
chr19.fa	6585850	6591163	CD70	0	0
chr19.fa	6663148	6670599	TNFSF14	0	0
chr19.fa	6677846	6720662	C3	0.897613978	304
chr19.fa	6729925	6737633	GPR108	11.90302401	1881
chr19.fa	6739707	6751529	TRIP10	19.26948289	2573
chr19.fa	6752173	6767523	SH2D3A	6.801534372	1064
chr19.fa	6772722	6857371	VAV1	2.37792401	456
chr19.fa	6887582	6940464	EMR1	0	0
chr19.fa	6952511	6990857	EMR4P	0	0
chr19.fa	7004964	7006426	FLJ25758	0	0
chr19.fa	7030594	7033022	MBD3L5	0	0
chr19.fa	7037755	7040184	MBD3L4	0	0
chr19.fa	7049351	7051746	MBD3L2	0	0
chr19.fa	7056216	7058645	MBD3L3	0	0
chr19.fa	7069471	7087978	ZNF557	5.722138503	2280
chr19.fa	7112266	7294011	INSR	5.054048636	3040
chr19.fa	7459999	7537371	ARHGEF18	11.84654663	4622

chr19.fa	7537723	7538247	LOC100128573	0	0
chr19.fa	7541761	7553905	PEX11G	6.466736406	456
chr19.fa	7562445	7573336	C19orf45	0	0
chr19.fa	7581004	7585911	ZNF358	30.47595243	3952
chr19.fa	7587496	7598895	MCOLN1	2.727782612	380
chr19.fa	7599038	7626653	PNPLA6	3.869981338	1292
chr19.fa	7660788	7683196	KIAA1543	11.48704921	3192
chr19.fa	7684411	7694439	XAB2	12.93347281	2280
chr19.fa	7694671	7696506	LOC100131801	14.59993232	412
chr19.fa	7696511	7698570	PCP2	0	0
chr19.fa	7701991	7712759	STXBP2	48.87131594	6133
chr19.fa	7733972	7735340	RETN	0	0
chr19.fa	7741943	7744719	C19orf59	0	0
chr19.fa	7745707	7747748	TRAPPC5	33.66489176	1900
chr19.fa	7753643	7767032	FCER2	0	0
chr19.fa	7793843	7797057	CLEC4G	0	0
chr19.fa	7804881	7812464	CD209	0	0
chr19.fa	7828035	7834491	CLEC4M	0	0
chr19.fa	7852370	7855898	CLEC4GP1	0	0
chr19.fa	7895161	7929862	EVI5L	3.571328902	912
chr19.fa	7933605	7939326	FLJ22184	1.520371327	456
chr19.fa	7943517	7945117	LOC388499	0	0
chr19.fa	7953390	7966908	LRRC8E	1.588897226	380
chr19.fa	7968765	7979363	MAP2K7	10.52286722	2356
chr19.fa	7981032	7983980	LOC645781	0	0
chr19.fa	7985194	7988136	SNAPC2	6.102720805	684
chr19.fa	7989381	7991051	CTXN1	5.437643066	456
chr19.fa	7991603	8008708	TIMM44	7.40230319	983
chr19.fa	8023457	8070529	ELAVL1	15.49317871	6232
chr19.fa	8117934	8127547	CCL25	0	0
chr19.fa	8130287	8212385	FBN3	0	0
chr19.fa	8274217	8327304	LASS4	5.042301339	608
chr19.fa	8367011	8373240	CD320	1.829867421	152
chr19.fa	8376234	8386280	NDUFA7	59.20095518	2103
chr19.fa	8386384	8387280	RPS28	91.86205547	2330
chr19.fa	8387468	8408146	KANK3	0	0
chr19.fa	8429011	8439257	ANGPTL4	0.60152185	76
chr19.fa	8455205	8469317	RAB11B	54.83999707	5928
chr19.fa	8478187	8503899	2-Mar	24.95818678	2736
chr19.fa	8509803	8553996	HNRNPM	53.11148774	8513
chr19.fa	8554940	8567538	PRAM1	0	0
chr19.fa	8575462	8579048	ZNF414	5.142605183	532
chr19.fa	8585674	8642331	MYO1F	0	0

chr19.fa	8645126	8675588	ADAMTS10	0	0
chr19.fa	8807751	8809172	ACTL9	0	0
chr19.fa	8841391	8842335	OR2Z1	0	0
chr19.fa	8920382	8933565	ZNF558	4.557198214	912
chr19.fa	8953269	8954016	MBD3L1	0	0
chr19.fa	8959520	9092018	MUC16	0	0
chr19.fa	9203921	9204862	OR1M1	0	0
chr19.fa	9212945	9213982	OR7G2	0	0
chr19.fa	9225504	9226439	OR7G1	0	0
chr19.fa	9236688	9237626	OR7G3	0	0
chr19.fa	9251056	9274091	ZNF317	14.04434541	3800
chr19.fa	9296270	9299493	OR7D2	0	0
chr19.fa	9324526	9325547	OR7D4	0	0
chr19.fa	9361720	9362739	OR7E24	0	0
chr19.fa	9405986	9415795	ZNF699	3.279754966	456
chr19.fa	9434928	9454521	ZNF559	4.077667525	760
chr19.fa	9466333	9493293	ZNF177	1.190693978	228
chr19.fa	9523271	9546234	ZNF266	6.992955071	1672
chr19.fa	9577031	9609279	ZNF560	0	0
chr19.fa	9638681	9649303	ZNF426	8.942253345	1368
chr19.fa	9676292	9695209	ZNF121	8.606099923	988
chr19.fa	9718002	9731916	ZNF561	12.35363828	3724
chr19.fa	9759338	9785776	ZNF562	19.39343193	7448
chr19.fa	9800814	9811493	ZNF812	1.138282961	152
chr19.fa	9868151	9879410	ZNF846	5.902866149	836
chr19.fa	9920943	9929731	FBXL12	5.562345142	684
chr19.fa	9938568	9940797	UBL5	237.197807	8174
chr19.fa	9945999	9960358	PIN1	50.51428085	3344
chr19.fa	9964394	10047070	OLFM2	0.615076424	76
chr19.fa	10070237	10121147	COL5A3	0	0
chr19.fa	10123925	10132954	RDH8	0	0
chr19.fa	10152032	10184813	C3P1	0	0
chr19.fa	10196806	10203928	C19orf66	2.723565634	304
chr19.fa	10204025	10213425	ANGPTL6	0	0
chr19.fa	10218327	10218411	SNORD105	0	0
chr19.fa	10220425	10220516	SNORD105B	0	0
chr19.fa	10221761	10221975	PPAN	10.6475693	152
chr19.fa	10222197	10222416	P2RY11	15.60824198	228
chr19.fa	10226155	10230599	EIF3G	66.84181887	4487
chr19.fa	10244022	10305755	DNMT1	5.003444895	1794
chr19.fa	10332109	10341948	S1PR2	0.319436115	76
chr19.fa	10341089	10341161	MIR4322	0	0
chr19.fa	10362640	10370721	MRPL4	13.67084161	1200

chr19.fa	10381517	10397291	ICAM1	0.352569517	76
chr19.fa	10397650	10399198	ICAM4	0	0
chr19.fa	10400655	10407454	ICAM5	0	0
chr19.fa	10415479	10420233	ZGLP1	0	0
chr19.fa	10420891	10426691	FDX1L	10.87513553	608
chr19.fa	10426889	10444314	RAVER1	9.241056387	2204
chr19.fa	10444452	10450345	ICAM3	10.28867431	1216
chr19.fa	10461204	10491248	TYK2	9.969539409	2812
chr19.fa	10501809	10514271	CDC37	41.74356816	4407
chr19.fa	10531333	10580307	PDE4A	3.311081091	1216
chr19.fa	10596796	10614054	KEAP1	24.57880932	4408
chr19.fa	10623418	10628668	S1PR5	0	0
chr19.fa	10654647	10663760	ATG4D	7.98349318	828
chr19.fa	10662798	10662880	MIR1238	0	0
chr19.fa	10664095	10676702	KRI1	8.914541773	1581
chr19.fa	10677138	10679655	CDKN2D	1.608777267	152
chr19.fa	10683347	10697991	AP1M2	54.17009993	6298
chr19.fa	10713121	10755235	SLC44A2	38.68008395	9120
chr19.fa	10762538	10764548	LOC147727	4.553433055	608
chr19.fa	10764937	10803095	ILF3	33.66022296	16702
chr19.fa	10812112	10824043	QTRT1	11.09788235	983
chr19.fa	10828729	10942586	DNM2	49.9434827	12618
chr19.fa	10829080	10829179	MIR638	0	0
chr19.fa	10928102	10928172	MIR199A1	0	0
chr19.fa	10943114	10946983	TMED1	6.747918503	608
chr19.fa	10959106	10980360	C19orf38	1.051081871	76
chr19.fa	10982253	11033443	CARM1	9.271177662	1824
chr19.fa	11033449	11039357	YIPF2	6.87216876	684
chr19.fa	11039424	11040916	C19orf52	4.919557146	456
chr19.fa	11071598	11172958	SMARCA4	31.86695289	12431
chr19.fa	11200038	11244505	LDLR	8.883065041	3116
chr19.fa	11257831	11266484	SPC24	0	0
chr19.fa	11274943	11308243	KANK2	2.857153486	988
chr19.fa	11309971	11373157	DOCK6	5.017903107	2128
chr19.fa	11350295	11352619	LOC55908	0	0
chr19.fa	11406824	11437672	TSPAN16	0	0
chr19.fa	11432722	11450344	RAB3D	49.02026564	13908
chr19.fa	11453452	11456981	TMEM205	16.2444033	988
chr19.fa	11457181	11465620	CCDC159	19.14101565	1351
chr19.fa	11466062	11476374	LPPR2	6.29594878	1140
chr19.fa	11485383	11487627	C19orf39	0.68872294	76
chr19.fa	11487881	11495018	EPOR	1.276087791	228
chr19.fa	11504732	11530018	RGL3	4.050859591	684

chr19.fa	11531272	11545980	CCDC151	0	0
chr19.fa	11546269	11561782	PRKCSH	42.13288563	6384
chr19.fa	11562143	11591803	ELAVL3	0	0
chr19.fa	11594242	11616738	ZNF653	0.511007421	76
chr19.fa	11616744	11639987	ECSIT	10.14047764	1216
chr19.fa	11649579	11661138	CNN1	0	0
chr19.fa	11663858	11670051	ELOF1	57.28659759	3876
chr19.fa	11685475	11689801	ACP5	3.794226333	456
chr19.fa	11708235	11729974	ZNF627	12.38933199	2356
chr19.fa	11784813	11797384	ZNF833P	0	0
chr19.fa	11832080	11849760	ZNF823	23.82035563	3788
chr19.fa	11877815	11894893	ZNF441	4.117277001	1216
chr19.fa	11909391	11919306	ZNF491	0.405432354	76
chr19.fa	11925107	11946016	ZNF440	25.46000721	7068
chr19.fa	11976844	11980306	ZNF439	18.78151824	3180
chr19.fa	11998670	12025365	ZNF69	12.3825547	1364
chr19.fa	12035900	12061578	ZNF700	9.188795976	1748
chr19.fa	12075869	12091198	ZNF763	2.618291779	456
chr19.fa	12125532	12146525	ZNF433	5.891871884	908
chr19.fa	12154620	12167127	ZNF878	0	0
chr19.fa	12175546	12188626	ZNF844	10.09710301	1900
chr19.fa	12203078	12225494	ZNF788	0.607395499	152
chr19.fa	12242803	12251140	ZNF20	1.466755458	228
chr19.fa	12255709	12267529	ZNF625	4.786270506	532
chr19.fa	12273872	12300064	ZNF136	3.034115973	608
chr19.fa	12382625	12405714	ZNF44	9.832035792	1824
chr19.fa	12428304	12444534	ZNF563	8.255337682	1520
chr19.fa	12460185	12476475	ZNF442	2.8362192	532
chr19.fa	12500828	12512088	ZNF799	2.653684277	456
chr19.fa	12540520	12551926	ZNF443	2.148249292	380
chr19.fa	12571998	12595632	ZNF709	1.401844112	456
chr19.fa	12636184	12662356	ZNF564	8.930054229	1748
chr19.fa	12686920	12721623	ZNF490	6.194138873	2508
chr19.fa	12721732	12740676	ZNF791	9.114848248	1510
chr19.fa	12757322	12777591	MAN2B1	7.10048802	1520
chr19.fa	12777618	12786533	WDR83	5.307368554	456
chr19.fa	12778881	12780465	C19orf56	80.88917641	4098
chr19.fa	12786647	12792677	DHPS	31.90550812	2432
chr19.fa	12799730	12807455	FBXW9	1.317956362	152
chr19.fa	12810008	12834810	TNPO2	16.67724601	6004
chr19.fa	12817263	12817332	SNORD41	0	0
chr19.fa	12841454	12845529	C19orf43	118.2342855	7144
chr19.fa	12848306	12859137	ASNA1	33.08626207	2812

chr19.fa	12863407	12869271	BEST2	0	0
chr19.fa	12873817	12886434	HOOK2	29.33646462	5084
chr19.fa	12902310	12904125	JUNB	34.66597231	4180
chr19.fa	12907634	12912694	PRDX2	129.2158997	11248
chr19.fa	12917428	12924462	RNASEH2A	1.002285407	76
chr19.fa	12936296	12946230	RTBDN	0	0
chr19.fa	12949259	12985766	MAST1	0	0
chr19.fa	12986025	12992335	DNASE2	2.280029868	304
chr19.fa	12995237	12998017	KLF1	0	0
chr19.fa	13001974	13010783	GCDH	13.19251577	1596
chr19.fa	13009894	13030086	SYCE2	1.226839507	76
chr19.fa	13033284	13044558	FARSA	24.09054347	2964
chr19.fa	13049414	13055304	CALR	41.32804518	5244
chr19.fa	13056654	13064455	RAD23A	41.49446522	4940
chr19.fa	13064972	13068050	GADD45GIP1	35.43195632	1748
chr19.fa	13080432	13085567	DAND5	0	0
chr19.fa	13106584	13209610	NFIX	67.74048709	25080
chr19.fa	13209844	13213974	LYL1	0	0
chr19.fa	13215714	13227563	TRMT1	7.46706393	1063
chr19.fa	13229109	13251959	NACC1	19.11511136	5700
chr19.fa	13255224	13260987	STX10	7.630773057	380
chr19.fa	13261282	13265718	IER2	27.97182028	3800
chr19.fa	13317256	13617274	CACNA1A	0	0
chr19.fa	13858753	13874106	CCDC130	6.312214269	684
chr19.fa	13875337	13885096	MRI1	3.504610279	760
chr19.fa	13885257	13889586	C19orf53	48.79375365	2945
chr19.fa	13906274	13943044	ZSWIM4	3.956881215	1140
chr19.fa	13945330	13947100	LOC284454	0.646251943	76
chr19.fa	13947104	13947173	MIR24-2	0	0
chr19.fa	13947254	13947331	MIR27A	0	0
chr19.fa	13947401	13947473	MIR23A	0	0
chr19.fa	13985513	13985622	MIR181C	0	0
chr19.fa	13985689	13985825	MIR181D	0	0
chr19.fa	13987950	13991571	NANOS3	0	0
chr19.fa	13993168	14016909	C19orf57	0.472904008	76
chr19.fa	14016956	14041693	CC2D1A	9.024032605	2181
chr19.fa	14042000	14064204	PODNL1	0	0
chr19.fa	14063319	14072256	DCAF15	5.576050322	836
chr19.fa	14072342	14117134	RFX1	5.673944464	1649
chr19.fa	14139017	14141783	RLN3	0	0
chr19.fa	14142262	14164026	IL27RA	0	0
chr19.fa	14164179	14169971	PALM3	33.9331217	5092
chr19.fa	14183821	14185874	LOC113230	10.56563943	912

chr19.fa	14199229	14201232	SAMD1	14.97946038	1292
chr19.fa	14202507	14228559	PRKACA	23.12801814	4177
chr19.fa	14230321	14247440	ASF1B	0	0
chr19.fa	14258549	14316997	LPHN1	1.311179076	684
chr19.fa	14492213	14519535	CD97	2.832303434	608
chr19.fa	14519610	14530195	DDX39	9.66230241	988
chr19.fa	14544166	14582679	PKN1	14.23456126	2964
chr19.fa	14583278	14586174	PTGER1	1.611036363	152
chr19.fa	14588571	14606944	GIPC1	33.2285851	4256
chr19.fa	14599758	14600690	LOC100130932	0	0
chr19.fa	14625582	14629201	DNAJB1	73.8126348	10944
chr19.fa	14640355	14640381	MIR639	0	0
chr19.fa	14640453	14676792	TECR	15.72270282	1140
chr19.fa	14676890	14682889	NDUFB7	44.02344742	1596
chr19.fa	14693896	14721951	CLEC17A	0	0
chr19.fa	14730051	14785730	EMR3	0	0
chr19.fa	14800870	14831772	ZNF333	2.430786846	608
chr19.fa	14843205	14889353	EMR2	0	0
chr19.fa	14909986	14910948	OR7C1	0	0
chr19.fa	14937139	14939276	OR7A5	0	0
chr19.fa	14951760	14952689	OR7A10	0	0
chr19.fa	14991238	14992167	OR7A17	0	0
chr19.fa	15052301	15053260	OR7C2	0	0
chr19.fa	15060991	15083730	SLC1A6	0	0
chr19.fa	15121539	15134083	CCDC105	0	0
chr19.fa	15160291	15169103	CASP14	0	0
chr19.fa	15197877	15198944	OR1I1	0	0
chr19.fa	15218214	15225784	SYDE1	0	0
chr19.fa	15225790	15236577	ILVBL	8.985176161	1368
chr19.fa	15270444	15311792	NOTCH3	4.679942408	2508
chr19.fa	15337730	15343858	EPHX3	0	0
chr19.fa	15348301	15391262	BRD4	18.07969255	8947
chr19.fa	15464335	15490603	AKAP8	18.10604866	4245
chr19.fa	15490859	15529833	AKAP8L	14.93247119	2096
chr19.fa	15532318	15560762	WIZ	6.316280641	1748
chr19.fa	15560359	15560419	MIR1470	0	0
chr19.fa	15562438	15575382	RASAL3	0	0
chr19.fa	15579457	15590315	PGLYRP2	0	0
chr19.fa	15619336	15663128	CYP4F22	6.142029068	1064
chr19.fa	15726029	15740447	CYP4F8	1.201688243	152
chr19.fa	15751707	15771570	CYP4F3	1.826704688	380
chr19.fa	15783886	15807984	CYP4F12	1.327143351	152
chr19.fa	15838834	15839862	OR10H2	0	0

chr19.fa	15852203	15853153	OR10H3	0	0
chr19.fa	15870144	15890798	CYP4F24P	0	0
chr19.fa	15904859	15905806	OR10H5	0	0
chr19.fa	15917817	15918936	OR10H1	6.131787835	456
chr19.fa	15939757	15946230	UCA1	0	0
chr19.fa	15988834	16008884	CYP4F2	0.970055643	152
chr19.fa	16023180	16045676	CYP4F11	0	0
chr19.fa	16059818	16060768	OR10H4	0	0
chr19.fa	16126444	16138272	LOC126536	0	0
chr19.fa	16144523	16152918	FLJ25328	0	0
chr19.fa	16178317	16213813	TPM4	109.7125251	21876
chr19.fa	16222490	16244445	RAB8A	43.00956533	6217
chr19.fa	16244838	16269377	HSH2D	9.687754887	1520
chr19.fa	16272179	16284286	CIB3	0	0
chr19.fa	16296235	16302857	FAM32A	43.4461732	4180
chr19.fa	16308665	16346156	AP1M1	9.574348289	1520
chr19.fa	16435651	16438339	KLF2	0.694144769	76
chr19.fa	16472408	16582762	EPS15L1	19.24659072	3545
chr19.fa	16589875	16607003	CALR3	0	0
chr19.fa	16607205	16632163	C19orf44	1.908935767	380
chr19.fa	16628700	16653263	CHERP	16.96174145	4220
chr19.fa	16660648	16683193	SLC35E1	7.416460189	2508
chr19.fa	16685718	16739015	MED26	5.751807958	1216
chr19.fa	16756959	16770968	C19orf42	33.68627786	3071
chr19.fa	16771938	16799816	TMEM38A	0.704386002	76
chr19.fa	16830787	16928774	NWD1	16.0199998	8132
chr19.fa	16940218	16991164	SIN3B	12.49716615	4256
chr19.fa	16999826	17002830	F2RL3	0	0
chr19.fa	17003762	17137625	CPAMD8	4.778740188	1900
chr19.fa	17160571	17186343	HAUS8	1.572330525	152
chr19.fa	17186591	17324104	MYO9B	14.56469043	7372
chr19.fa	17326155	17330638	USE1	5.3486347	304
chr19.fa	17337055	17340028	OCEL1	35.32547761	2559
chr19.fa	17342694	17356151	NR2F6	41.24144652	4940
chr19.fa	17360849	17375544	USHBP1	0	0
chr19.fa	17378232	17390162	C19orf62	35.22803529	3394
chr19.fa	17392454	17398454	ANKLE1	0	0
chr19.fa	17402940	17414282	ABHD8	2.190569683	304
chr19.fa	17416477	17417652	MRPL34	23.64896558	1520
chr19.fa	17420337	17434031	DDA1	11.41777028	3034
chr19.fa	17434107	17445638	ANO8	0.561460555	152
chr19.fa	17445791	17453539	GTPBP3	1.252291984	228
chr19.fa	17462264	17488137	PLVAP	0	0

chr19.fa	17513755	17516384	BST2	1.219007976	76
chr19.fa	17530912	17536140	FAM125A	13.38167738	1060
chr19.fa	17546318	17559376	TMEM221	0	0
chr19.fa	17566234	17571725	NXNL1	0	0
chr19.fa	17581300	17616977	SLC27A1	0.96809776	228
chr19.fa	17622432	17632097	PGLS	46.4927895	3192
chr19.fa	17634110	17664648	FAM129C	0	0
chr19.fa	17666511	17693965	GLT25D1	2.548561029	608
chr19.fa	17712137	17799008	UNC13A	0	0
chr19.fa	17830303	17845324	MAP1S	3.84332401	836
chr19.fa	17858527	17899377	FCHO1	1.376542241	304
chr19.fa	17905919	17924385	B3GNT3	2.945710032	532
chr19.fa	17927322	17932320	INSL3	0	0
chr19.fa	17935593	17958841	JAK3	0	0
chr19.fa	17970727	17970730	RPL18AP3	0	0
chr19.fa	17973397	17973529	SNORA68	0	0
chr19.fa	17974119	17974124	RPL18A	0	0
chr19.fa	17982782	18005983	SLC5A5	0	0
chr19.fa	18043824	18054794	CCDC124	21.13775493	1520
chr19.fa	18062111	18109930	KCNN1	0	0
chr19.fa	18111944	18124911	ARRDC2	5.057813795	988
chr19.fa	18170371	18197697	IL12RB1	0	0
chr19.fa	18208603	18262499	MAST3	2.719198049	1064
chr19.fa	18264016	18281343	PIK3R2	16.85812426	4408
chr19.fa	18284579	18288927	IFI30	3.327346579	228
chr19.fa	18304040	18307550	MPV17L2	5.900004628	532
chr19.fa	18307611	18314874	RAB3A	0.762520062	76
chr19.fa	18318771	18359010	PDE4C	0	0
chr19.fa	18360760	18366229	LOC729966	0	0
chr19.fa	18367906	18385319	KIAA1683	0.257687503	76
chr19.fa	18390563	18392432	JUND	45.29471581	5624
chr19.fa	18392887	18392971	MIR3188	0	0
chr19.fa	18417717	18434001	LSM4	72.35145178	5515
chr19.fa	18451408	18480763	PGPEP1	20.30128714	9549
chr19.fa	18496968	18499986	GDF15	11.44608428	912
chr19.fa	18497372	18497444	MIR3189	0	0
chr19.fa	18501954	18508415	LRRC25	0	0
chr19.fa	18530221	18545197	SSBP4	5.203148944	531
chr19.fa	18545373	18549111	ISYNA1	2.039360885	304
chr19.fa	18553473	18632937	ELL	7.10590985	1900
chr19.fa	18642568	18654383	FKBP8	83.60957931	9804
chr19.fa	18668572	18680197	C19orf50	49.34437055	5216
chr19.fa	18682614	18688270	UBA52	139.6658734	26068

chr19.fa	18699495	18703147	C19orf60	15.60824198	912
chr19.fa	18704035	18717660	CRLF1	0	0
chr19.fa	18723682	18731849	TMEM59L	0	0
chr19.fa	18747838	18781302	KLHL26	3.666210917	760
chr19.fa	18794425	18893143	CRTC1	1.637091265	760
chr19.fa	18893583	18902114	COMP	0	0
chr19.fa	18942744	18979039	UPF1	32.33594113	11476
chr19.fa	18988718	18989738	LASS1	0	0
chr19.fa	19010323	19030199	COPE	74.69232662	5624
chr19.fa	19030484	19039442	DDX49	20.57298104	2508
chr19.fa	19040010	19052041	HOMER3	1.671730731	228
chr19.fa	19101697	19144380	SUGP2	20.91425508	6985
chr19.fa	19144387	19168987	ARMC6	18.40199018	3040
chr19.fa	19174803	19223704	SLC25A42	12.79099918	2660
chr19.fa	19230430	19249267	TMEM161A	1.286780843	152
chr19.fa	19281014	19281098	MEF2B	0	0
chr19.fa	19287713	19293381	LOC729991	1.993727154	166
chr19.fa	19303769	19310043	RFXANK	22.16233008	1267
chr19.fa	19312224	19314238	NR2C2AP	1.061774924	76
chr19.fa	19322782	19363061	NCAN	0	0
chr19.fa	19366452	19373596	HAPLN4	0	0
chr19.fa	19375174	19384074	TM6SF2	0	0
chr19.fa	19387322	19431307	SUGP1	9.929176902	1368
chr19.fa	19431630	19469563	MAU2	8.43184835	2717
chr19.fa	19496642	19619741	GATAD2A	20.78533602	7828
chr19.fa	19625028	19626469	TSSK6	0	0
chr19.fa	19627019	19639013	NDUFA13	219.463003	7898
chr19.fa	19639670	19648393	YJEFN3	0	0
chr19.fa	19649074	19657468	CILP2	0	0
chr19.fa	19672522	19729439	PBX4	0.795954677	76
chr19.fa	19734466	19739039	LPAR2	1.914056383	228
chr19.fa	19740285	19754455	GMIP	7.159977537	1672
chr19.fa	19756010	19774503	ATP13A1	3.850854329	988
chr19.fa	19779663	19791138	ZNF101	5.525747793	532
chr19.fa	19821281	19843921	ZNF14	10.4300937	2052
chr19.fa	19867181	19887222	LOC284440	2.413768326	380
chr19.fa	19903520	19932560	ZNF506	10.33039228	2280
chr19.fa	19976714	20004293	ZNF253	5.406015728	836
chr19.fa	20011722	20046382	ZNF93	1.638597329	304
chr19.fa	20115227	20150277	ZNF682	1.043702159	228
chr19.fa	20188803	20231977	ZNF90	1.829867421	456
chr19.fa	20278083	20308984	ZNF486	32.3377484	3268
chr19.fa	20578626	20607771	ZNF826P	0	0

chr19.fa	20720798	20748626	ZNF737	6.138414515	3420
chr19.fa	20802745	20844402	ZNF626	14.40384282	6536
chr19.fa	21106059	21133503	ZNF85	1.631368223	380
chr19.fa	21203426	21242852	ZNF430	2.628533013	684
chr19.fa	21264953	21307883	ZNF714	1.301992087	760
chr19.fa	21324840	21368805	ZNF431	2.380936137	608
chr19.fa	21473963	21512212	ZNF708	4.574969766	1216
chr19.fa	21541735	21571384	ZNF738	0.683602323	532
chr19.fa	21579921	21610296	ZNF493	2.367833383	988
chr19.fa	21666517	21686040	LOC400680	0	0
chr19.fa	21688437	21721079	ZNF429	7.272028678	1140
chr19.fa	21906843	21950430	ZNF100	1.280304769	380
chr19.fa	21933547	21936240	LOC641367	0	0
chr19.fa	21987752	22018970	ZNF43	3.510333322	1216
chr19.fa	22148897	22193745	ZNF208	0.881649702	532
chr19.fa	22235266	22273903	ZNF257	0.975477472	228
chr19.fa	22361903	22379753	ZNF676	1.560432622	304
chr19.fa	22573899	22605148	ZNF98	1.012978459	152
chr19.fa	22779059	22786352	LOC440518	0.562966619	76
chr19.fa	22817126	22850472	ZNF492	0	0
chr19.fa	22939007	22952784	ZNF99	0	0
chr19.fa	23540498	23578269	ZNF91	32.45461895	11628
chr19.fa	23835708	23870017	ZNF675	7.772343046	1140
chr19.fa	23921997	23941693	ZNF681	1.057106126	456
chr19.fa	23945816	24010919	RPSAP58	2.490125757	228
chr19.fa	24097684	24106517	ZNF726	7.360886438	304
chr19.fa	24269976	24312654	ZNF254	6.891747589	1824
chr19.fa	24344995	24346249	LOC100101266	0	0
chr19.fa	28281401	28284848	LOC148189	1.674291039	228
chr19.fa	29456038	29460055	LOC148145	0	0
chr19.fa	29698167	29704136	UQCRFS1	45.82168751	3724
chr19.fa	30017491	30055226	VSTM2B	0	0
chr19.fa	30097170	30108162	POP4	15.23488879	2624
chr19.fa	30156327	30166383	PLEKHF1	17.7714013	2052
chr19.fa	30189793	30206452	C19orf12	7.578663252	2432
chr19.fa	30302901	30315219	CCNE1	2.881702324	380
chr19.fa	30433425	30506613	C19orf2	45.07603536	6809
chr19.fa	30863328	31048965	ZNF536	0	0
chr19.fa	31640783	31641311	DKFZp566F0947	0	0
chr19.fa	31765851	31840190	TSHZ3	0.931952231	304
chr19.fa	32836514	32878573	ZNF507	14.97870735	7676
chr19.fa	32896655	32976799	DPY19L3	2.58922475	1064
chr19.fa	33072094	33078358	PDCD5	19.84811257	796

chr19.fa	33087907	33166102	ANKRD27	23.27922694	6883
chr19.fa	33166313	33169206	RGS9BP	1.581969333	304
chr19.fa	33182867	33204702	NUDT19	13.11645956	2584
chr19.fa	33210679	33281714	TDRD12	0	0
chr19.fa	33321419	33360683	SLC7A9	2.580790793	304
chr19.fa	33369904	33462869	CCDC123	13.47068574	2330
chr19.fa	33463148	33467960	C19orf40	4.023298625	304
chr19.fa	33469498	33555824	RHPN2	24.31193483	5700
chr19.fa	33571786	33621318	GPATCH1	15.66095421	3496
chr19.fa	33622998	33666703	WDR88	0	0
chr19.fa	33685599	33699569	LRP3	5.241553569	1216
chr19.fa	33699774	33716756	SLC7A10	0	0
chr19.fa	33790840	33793430	CEBPA	24.2970248	4180
chr19.fa	33793763	33795963	LOC80054	4.680394227	684
chr19.fa	33864609	33873592	CEBPG	13.17399119	3268
chr19.fa	33877855	34012799	PEPD	46.17094368	6162
chr19.fa	34112861	34264414	CHST8	0	0
chr19.fa	34287751	34306666	KCTD15	4.409453363	1444
chr19.fa	34663352	34720420	LSM14A	45.82078387	11476
chr19.fa	34745456	34846471	KIAA0355	10.79004292	4788
chr19.fa	34855645	34893318	GPI	33.48747745	9948
chr19.fa	34895303	34917072	PDCD2L	6.925031597	532
chr19.fa	34919268	34960798	UBA2	26.62886326	4712
chr19.fa	34972880	34992085	WTIP	1.55802292	228
chr19.fa	35067638	35068596	LOC643719	0	0
chr19.fa	35084346	35085490	SCGBL	0	0
chr19.fa	35168567	35177302	ZNF302	8.291031392	1444
chr19.fa	35225480	35233774	ZNF181	3.4909051	684
chr19.fa	35248979	35264134	ZNF599	0.367479548	76
chr19.fa	35307058	35323773	LOC400685	0	0
chr19.fa	35417807	35436076	ZNF30	1.716762036	304
chr19.fa	35447258	35454953	ZNF792	3.827209129	988
chr19.fa	35491246	35517373	GRAMD1A	6.937080107	1216
chr19.fa	35521592	35531353	SCN1B	0.578328469	76
chr19.fa	35531410	35557477	HPN	9.627060519	1444
chr19.fa	35549963	35597175	LOC100128675	0	0
chr19.fa	35606732	35615228	FXYD3	60.8226846	8388
chr19.fa	35615417	35626178	LGI4	0.396395971	76
chr19.fa	35629732	35633954	FXYD1	0	0
chr19.fa	35634154	35645205	FXYD7	0	0
chr19.fa	35645625	35660788	FXYD5	0.699566599	76
chr19.fa	35715704	35719628	FAM187B	0	0
chr19.fa	35739559	35758867	LSR	4.019684072	608

chr19.fa	35759896	35770718	USF2	53.21013491	6038
chr19.fa	35773410	35776045	HAMP	0	0
chr19.fa	35782989	35804709	MAG	0	0
chr19.fa	35820072	35838264	CD22	0	0
chr19.fa	35842445	35843367	FFAR1	0	0
chr19.fa	35849488	35851389	FFAR3	0	0
chr19.fa	35940617	35942669	FFAR2	0	0
chr19.fa	35978228	35981356	KRTDAP	0	0
chr19.fa	35988119	36004560	DMKN	8.658661546	1458
chr19.fa	36014269	36019253	SBSN	0	0
chr19.fa	36024314	36036221	GAPDHS	0	0
chr19.fa	36036545	36038428	TMEM147	34.89956279	1972
chr19.fa	36041095	36054560	ATP4A	0	0
chr19.fa	36103646	36116251	HAUS5	1.593867236	456
chr19.fa	36119980	36128587	RBM42	20.81108971	2280
chr19.fa	36132647	36135773	ETV2	1.539498336	152
chr19.fa	36139125	36149686	COX6B1	228.9216856	8968
chr19.fa	36157715	36169367	UPK1A	297.6168665	24168
chr19.fa	36203830	36207940	ZBTB32	0	0
chr19.fa	36208921	36229779	MLL4	10.67708815	6004
chr19.fa	36230151	36233351	TMEM149	0.957103495	76
chr19.fa	36233428	36236336	U2AF1L4	0	0
chr19.fa	36236494	36237903	PSENEN	18.93332947	836
chr19.fa	36239512	36245420	LIN37	2.543591019	152
chr19.fa	36245470	36247930	HSPB6	0	0
chr19.fa	36249044	36260077	C19orf55	2.132435623	304
chr19.fa	36266417	36279724	ARHGAP33	0.530285036	152
chr19.fa	36290892	36304201	PRODH2	3.412740392	380
chr19.fa	36316274	36342895	NPHS1	0.227867441	76
chr19.fa	36347810	36358048	KIRREL2	0	0
chr19.fa	36359401	36370699	APLP1	0	0
chr19.fa	36379143	36391552	NFKBID	0.624112806	76
chr19.fa	36393382	36395173	HCST	2.257589518	76
chr19.fa	36395303	36399211	TYROBP	0	0
chr19.fa	36428022	36436097	LRFN3	0.910264913	152
chr19.fa	36486090	36487220	SDHAF1	5.060223497	380
chr19.fa	36494002	36499672	C19orf46	8.913186315	912
chr19.fa	36500022	36505141	ALKBH6	3.656873322	228
chr19.fa	36505562	36523797	CLIP3	0.339466763	76
chr19.fa	36525887	36545664	THAP8	2.508800947	304
chr19.fa	36545783	36596012	WDR62	0	0
chr19.fa	36604611	36605887	POLR2I	47.55125108	1746
chr19.fa	36606207	36616849	TBCB	27.7240728	2128

chr19.fa	36630918	36641255	CAPNS1	277.9085167	27273
chr19.fa	36641824	36643771	COX7A1	4.50629326	228
chr19.fa	36673187	36692952	ZNF565	3.277797083	380
chr19.fa	36705504	36729675	ZNF146	24.05710885	5776
chr19.fa	36802245	36803570	LOC100134317	0	0
chr19.fa	36825355	36870105	ZFP14	2.596905675	1292
chr19.fa	36882861	36909550	ZFP82	4.375416323	760
chr19.fa	36912439	36913686	LOC644189	0	0
chr19.fa	36936021	36980463	ZNF566	2.16451478	760
chr19.fa	36980529	36981942	LOC728752	0.80950925	76
chr19.fa	37001589	37019248	ZNF260	6.151667876	2280
chr19.fa	37034517	37096178	ZNF529	1.606969991	836
chr19.fa	37096221	37119499	ZNF382	0	0
chr19.fa	37128283	37157739	ZNF461	1.434375088	304
chr19.fa	37180302	37212225	ZNF567	2.554886497	456
chr19.fa	37234380	37263716	ZNF850	0.741284564	380
chr19.fa	37308331	37329284	ZNF790	5.658883826	1140
chr19.fa	37341267	37370470	ZNF345	0.735561521	152
chr19.fa	37379026	37407193	ZNF829	0.43630666	152
chr19.fa	37407234	37442450	ZNF568	3.146618933	608
chr19.fa	37569382	37620651	ZNF420	1.539950155	304
chr19.fa	37641001	37663615	ZNF585A	3.283670732	912
chr19.fa	37672481	37701451	ZNF585B	6.451073344	2660
chr19.fa	37717366	37734574	ZNF383	6.081184094	684
chr19.fa	37756841	37759912	LOC284412	0	0
chr19.fa	37825580	37855357	HKR1	16.80375536	3268
chr19.fa	37862059	37883966	ZNF527	1.116445037	380
chr19.fa	37902060	37958339	ZNF569	0.282085735	76
chr19.fa	37959982	37976242	ZNF570	3.565756467	532
chr19.fa	37997841	38034239	ZNF793	1.285877205	608
chr19.fa	38042308	38105000	ZNF540	1.674893464	380
chr19.fa	38055155	38085673	ZNF571	1.508774636	228
chr19.fa	38123389	38146313	ZFP30	4.219990547	1292
chr19.fa	38158650	38183216	ZNF781	0.364316814	76
chr19.fa	38187264	38210691	ZNF607	2.017824174	608
chr19.fa	38229203	38270230	ZNF573	3.390149437	608
chr19.fa	38319707	38322556	LOC728853	0.401667194	76
chr19.fa	38375472	38397317	WDR87	0	0
chr19.fa	38397868	38699008	SIPA1L3	18.63000823	9875
chr19.fa	38701649	38720317	DPF1	0	0
chr19.fa	38741877	38747172	PPP1R14A	0	0
chr19.fa	38755098	38783254	SPINT2	17.06656348	2042
chr19.fa	38794200	38806606	YIF1B	1.685134698	304

chr19.fa	38810484	38819649	KCNK6	3.017850485	532
chr19.fa	38826443	38861589	CATSPERG	0.614775211	152
chr19.fa	38865190	38874464	PSMD8	85.52950934	8740
chr19.fa	38874992	38878668	GGN	0.531339281	76
chr19.fa	38880939	38886871	SPRED3	0	0
chr19.fa	38893775	38899697	FAM98C	14.72162227	1216
chr19.fa	38899729	38916945	RASGRP4	0	0
chr19.fa	38924340	39078204	RYR1	0	0
chr19.fa	39078281	39108643	MAP4K1	0	0
chr19.fa	39109722	39127595	EIF3K	119.7996881	6992
chr19.fa	39138267	39220831	ACTN4	67.46035924	16188
chr19.fa	39221172	39235114	CAPN12	1.636187627	303
chr19.fa	39261608	39264157	LGALS7	0	0
chr19.fa	39279850	39282394	LGALS7B	0	0
chr19.fa	39292311	39303740	LGALS4	8.054278175	684
chr19.fa	39306062	39322497	ECH1	55.91397111	4689
chr19.fa	39327028	39342979	HNRNPL	52.48556765	7524
chr19.fa	39358472	39368919	RINL	2.632147566	532
chr19.fa	39369195	39390463	SIRT2	16.36172566	2251
chr19.fa	39390615	39399534	NFKBIB	11.63840862	1748
chr19.fa	39405904	39421347	SARS2	4.115168512	532
chr19.fa	39421537	39423659	MRPS12	14.41724679	986
chr19.fa	39432042	39466380	FBXO17	8.351273941	1292
chr19.fa	39514663	39523198	FBXO27	8.380039758	1292
chr19.fa	39574945	39602128	PAPL	0	0
chr19.fa	39616420	39670046	PAK4	37.47899813	7600
chr19.fa	39687604	39692522	NCCRP1	15.08352938	1976
chr19.fa	39693562	39694906	SYCN	0	0
chr19.fa	39734272	39735611	IL28B	0	0
chr19.fa	39759157	39760732	IL28A	0	0
chr19.fa	39786965	39789312	IL29	0	0
chr19.fa	39797457	39805976	LRFN1	0	0
chr19.fa	39818999	39826726	GMFG	1.742214513	76
chr19.fa	39833108	39875537	SAMD4B	13.51089764	4028
chr19.fa	39876270	39881679	PAF1	13.13212262	1859
chr19.fa	39881963	39891203	MED29	17.61507189	4172
chr19.fa	39897487	39900045	ZFP36	24.26961444	2812
chr19.fa	39903750	39919055	PLEKHG2	0.601823063	304
chr19.fa	39923847	39926618	RPS16	890.0326037	34867
chr19.fa	39936186	39967308	SUPT5H	29.40092415	7551
chr19.fa	39971052	39981528	TIMM50	6.892048801	1177
chr19.fa	39989557	39999121	DLL3	0	0
chr19.fa	40005753	40011326	SELV	0	0

chr19.fa	40021630	40023494	EID2B	0	0
chr19.fa	40029447	40030838	EID2	4.933714145	456
chr19.fa	40093169	40098114	LGALS13	0	0
chr19.fa	40129325	40133041	LOC100129935	0	0
chr19.fa	40146558	40151287	LGALS16	0	0
chr19.fa	40170014	40177013	LGALS17A	0	0
chr19.fa	40194946	40200088	LGALS14	0	0
chr19.fa	40221895	40228668	CLC	0	0
chr19.fa	40267234	40276775	LEUTX	0	0
chr19.fa	40315990	40324841	DYRK1B	6.321250651	1064
chr19.fa	40325093	40337054	FBL	81.54717565	6308
chr19.fa	40353963	40440533	FCGBP	1.257111388	1368
chr19.fa	40477073	40487353	PSMC4	63.01325428	6004
chr19.fa	40502943	40523514	ZNF546	0.747610031	228
chr19.fa	40534167	40562115	ZNF780B	1.717515068	988
chr19.fa	40575059	40596845	ZNF780A	3.050682674	1520
chr19.fa	40697651	40721482	MAP3K10	4.330535624	988
chr19.fa	40721965	40724306	TTC9B	0	0
chr19.fa	40728115	40732597	CNTD2	0	0
chr19.fa	40736224	40791265	AKT2	17.59037245	6147
chr19.fa	40788450	40788548	MIR641	0	0
chr19.fa	40826971	40854293	C19orf47	7.002292666	988
chr19.fa	40854332	40884390	PLD3	8.710168926	1410
chr19.fa	40885178	40896094	HIPK4	0	0
chr19.fa	40899671	40919271	PRX	2.120989539	684
chr19.fa	40928409	40931932	SERTAD1	9.506726028	760
chr19.fa	40946748	40950282	SERTAD3	15.6796294	1748
chr19.fa	40953691	40971725	BLVRB	44.12631158	2508
chr19.fa	40973126	41082365	SPTBN4	0.125304502	76
chr19.fa	41082757	41097305	SHKBP1	13.44312478	2111
chr19.fa	41099072	41135725	LTBP4	0.420342385	152
chr19.fa	41171812	41196556	NUMBL	1.929719446	456
chr19.fa	41197434	41222790	ADCK4	4.101463332	680
chr19.fa	41223008	41246760	ITPKC	15.91608141	3572
chr19.fa	41246766	41255828	C19orf54	6.664331967	1216
chr19.fa	41256759	41271297	SNRPA	8.431697744	912
chr19.fa	41281300	41283394	MIA	0	0
chr19.fa	41284171	41302847	RAB4B	16.01186706	1212
chr19.fa	41305048	41314337	EGLN2	20.72629833	3040
chr19.fa	41349443	41356352	CYP2A6	0	0
chr19.fa	41381344	41388657	CYP2A7	0	0
chr19.fa	41430170	41456565	CYP2B7P1	0	0
chr19.fa	41497204	41524301	CYP2B6	1.875199939	380

chr19.fa	41594368	41602099	CYP2A13	0	0
chr19.fa	41620353	41634281	CYP2F1	0	0
chr19.fa	41699115	41713444	CYP2S1	0.436758479	76
chr19.fa	41725108	41767671	AXL	0	0
chr19.fa	41768391	41813811	HNRNPUL1	45.27302849	12920
chr19.fa	41816094	41830788	CCDC97	12.74852819	2812
chr19.fa	41836812	41859831	TGFB1	0.520345016	76
chr19.fa	41860322	41870078	B9D2	1.270364749	76
chr19.fa	41882438	41889987	TMEM91	1.770528511	152
chr19.fa	41892276	41903256	EXOSC5	5.763404649	380
chr19.fa	41903694	41930910	BCKDHA	17.42078967	2052
chr19.fa	41931264	41934635	B3GNT8	0	0
chr19.fa	41937223	41945843	ATP5SL	17.52275018	2128
chr19.fa	41949063	41950670	C19orf69	1.209971594	76
chr19.fa	42082531	42093197	CEACAM21	0	0
chr19.fa	42125344	42133442	CEACAM4	0	0
chr19.fa	42177235	42192096	CEACAM7	0.500013155	76
chr19.fa	42212530	42234437	CEACAM5	0	0
chr19.fa	42259398	42276113	CEACAM6	0	0
chr19.fa	42300534	42315591	CEACAM3	0	0
chr19.fa	42341150	42348508	LYPD4	0	0
chr19.fa	42349086	42356397	DMRTC2	0	0
chr19.fa	42363988	42375484	RPS19	480.8938271	27109
chr19.fa	42381190	42385439	CD79A	0	0
chr19.fa	42387267	42411604	ARHGEF1	5.846840579	1292
chr19.fa	42460833	42463528	RABAC1	22.69366937	1216
chr19.fa	42470734	42498382	ATP1A3	0	0
chr19.fa	42502473	42569957	GRIK5	1.297775109	304
chr19.fa	42580290	42585720	ZNF574	9.13924648	1900
chr19.fa	42592666	42636630	POU2F2	0.475614923	142
chr19.fa	42637597	42637665	MIR4323	0	0
chr19.fa	42702752	42721813	DEDD2	12.13149388	1520
chr19.fa	42724492	42732353	ZNF526	2.315874184	608
chr19.fa	42734338	42746736	GSK3A	32.56802555	4712
chr19.fa	42751717	42759309	ERF	11.42499939	2052
chr19.fa	42788817	42799949	CIC	10.66594327	3876
chr19.fa	42801185	42806952	PAFAH1B3	10.51458387	717
chr19.fa	42806284	42814973	PRR19	0	0
chr19.fa	42817477	42829214	TMEM145	0	0
chr19.fa	42829761	42882921	MEGF8	0.626221295	456
chr19.fa	42891171	42894444	CNFN	8.671312482	304
chr19.fa	42905666	42931578	LIPE	1.202893094	304
chr19.fa	42932695	42947136	CXCL17	1.341902776	76

chr19.fa	43011458	43032661	CEACAM1	0	0
chr19.fa	43084395	43099082	CEACAM8	0	0
chr19.fa	43225794	43244668	PSG3	0	0
chr19.fa	43256839	43269831	PSG8	0	0
chr19.fa	43326015	43331030	LOC100289650	0	0
chr19.fa	43341149	43359870	PSG10P	0	0
chr19.fa	43370613	43383871	PSG1	0	0
chr19.fa	43406240	43422043	PSG6	0	0
chr19.fa	43428284	43441330	PSG7	0	0
chr19.fa	43511809	43530631	PSG11	0	0
chr19.fa	43568362	43586893	PSG2	0	0
chr19.fa	43671895	43690688	PSG5	0	0
chr19.fa	43696854	43709790	PSG4	0	0
chr19.fa	43715943	43752798	LOC284344	0	0
chr19.fa	43757435	43773682	PSG9	0	0
chr19.fa	43853208	43853700	PRG1	0	0
chr19.fa	43857825	43867480	CD177	1.936797946	304
chr19.fa	43892763	43922767	TEX101	0	0
chr19.fa	43964946	43969831	LYPD3	8.051115441	912
chr19.fa	43979255	44008985	PHLDB3	13.79599551	2280
chr19.fa	44010871	44031396	ETHE1	23.77170978	1520
chr19.fa	44037340	44040284	ZNF575	0	0
chr19.fa	44047464	44079730	XRCC1	2.722661995	380
chr19.fa	44080952	44086256	LOC390940	4.774221996	304
chr19.fa	44088519	44100287	IRGQ	8.389377353	5396
chr19.fa	44100544	44104587	ZNF576	11.12890726	1520
chr19.fa	44111376	44124014	ZNF428	17.31536521	1367
chr19.fa	44116253	44118650	SRRM5	0.522152292	76
chr19.fa	44126522	44143991	CADM4	3.682175192	532
chr19.fa	44150248	44174502	PLAUR	0	0
chr19.fa	44220214	44224169	IRGC	0	0
chr19.fa	44235301	44259142	C19orf61	8.269645287	1292
chr19.fa	44270685	44285409	KCNN4	1.533022262	228
chr19.fa	44300079	44324808	LYPD5	0	0
chr19.fa	44331473	44353050	ZNF283	2.166623269	369
chr19.fa	44376515	44384288	ZNF404	1.242803783	152
chr19.fa	44416776	44439411	ZNF45	6.451826376	1672
chr19.fa	44455397	44471752	ZNF221	3.774496898	608
chr19.fa	44488355	44502477	ZNF155	6.596257886	1137
chr19.fa	44507077	44518072	ZNF230	1.385126804	380
chr19.fa	44529494	44537262	ZNF222	2.006980515	228
chr19.fa	44556164	44572142	ZNF223	6.164017598	988
chr19.fa	44576297	44591623	ZNF284	1.036774266	152

chr19.fa	44598482	44605378	ZNF224	4.147097063	152
chr19.fa	44609492	44617336	LOC100379224	0.800472868	76
chr19.fa	44617548	44637255	ZNF225	2.095537062	380
chr19.fa	44645710	44664462	ZNF234	2.239516754	684
chr19.fa	44669215	44681838	ZNF226	6.43751877	1349
chr19.fa	44716691	44741421	ZNF227	12.03661187	2432
chr19.fa	44764076	44779468	ZNF233	4.114264874	760
chr19.fa	44790501	44809178	ZNF235	3.946941194	836
chr19.fa	44830706	44860856	ZFP112	2.145237165	532
chr19.fa	44889808	44905777	ZNF285	5.113688759	912
chr19.fa	44930426	44952665	ZNF229	6.917501278	2280
chr19.fa	44979859	45004574	ZNF180	4.4008688	912
chr19.fa	45010211	45033548	CEACAM20	0.632396157	76
chr19.fa	45041045	45060150	CEACAM22P	3.036073856	608
chr19.fa	45147098	45169428	PVR	0.775622816	304
chr19.fa	45174724	45187627	CEACAM19	0	0
chr19.fa	45202421	45213986	CEACAM16	0	0
chr19.fa	45251978	45263301	BCL3	10.43897947	1292
chr19.fa	45281126	45303903	CBLC	20.86335012	2204
chr19.fa	45312338	45324678	BCAM	7.53348134	1216
chr19.fa	45349393	45392485	PVRL2	2.881551718	684
chr19.fa	45394477	45406946	TOMM40	4.016220126	456
chr19.fa	45409039	45412650	APOE	50.44033312	3952
chr19.fa	45417921	45422606	APOC1	0	0
chr19.fa	45430060	45434643	APOC1P1	0	0
chr19.fa	45445495	45448751	APOC4	0	0
chr19.fa	45449243	45452818	APOC2	0	0
chr19.fa	45458599	45496604	CLPTM1	12.43059814	1900
chr19.fa	45504712	45541452	RELB	2.525668861	380
chr19.fa	45542298	45574214	CLASRP	6.559509932	973
chr19.fa	45574758	45579688	ZNF296	0	0
chr19.fa	45582518	45594782	GEMIN7	3.449638954	380
chr19.fa	45653008	45663408	NKPD1	0	0
chr19.fa	45666186	45681485	TRAPPC6A	31.58004775	1667
chr19.fa	45682003	45685058	BLOC1S3	10.125417	1748
chr19.fa	45715879	45737469	EXOC3L2	0	0
chr19.fa	45754516	45808541	MARK4	5.81400839	2024
chr19.fa	45809671	45826233	CKM	0	0
chr19.fa	45843998	45854648	KLC3	1.376542241	152
chr19.fa	45854779	45873845	ERCC2	7.08181283	1216
chr19.fa	45882892	45908312	PPP1R13L	10.27210761	2128
chr19.fa	45909467	45914024	CD3EAP	0.700018418	76
chr19.fa	45910591	45927177	ERCC1	19.06300155	2572

chr19.fa	45971253	45978437	FOSB	1.212531902	304
chr19.fa	45988550	46000311	RTN2	0.501519219	76
chr19.fa	46001731	46005764	PPM1N	0	0
chr19.fa	46010688	46030240	VASP	27.62211229	4200
chr19.fa	46031025	46088122	OPA3	3.231711533	2052
chr19.fa	46093023	46105466	GPR4	0	0
chr19.fa	46112658	46148775	EML2	28.63900651	5893
chr19.fa	46142252	46142345	MIR330	0	0
chr19.fa	46171502	46185717	GIPR	0.565526927	76
chr19.fa	46178186	46178282	MIR642A	0	0
chr19.fa	46190712	46195443	SNRPD2	80.43901396	4636
chr19.fa	46195741	46207248	QPCTL	0.495344358	76
chr19.fa	46213887	46234151	FBXO46	1.914056383	380
chr19.fa	46268043	46272497	SIX5	5.15435248	1140
chr19.fa	46272976	46285815	DMPK	0.318080658	76
chr19.fa	46286264	46296060	DMWD	3.4601814	760
chr19.fa	46298968	46318605	RSPH6A	0	0
chr19.fa	46318700	46366548	SYMPK	19.67807798	5472
chr19.fa	46367518	46377055	FOXA3	0	0
chr19.fa	46386866	46389376	IRF2BP1	8.205035154	1368
chr19.fa	46393281	46405862	MYPOP	3.032911122	380
chr19.fa	46416473	46418036	NANOS2	0	0
chr19.fa	46442771	46476657	NOVA2	0	0
chr19.fa	46498339	46521874	CCDC61	5.644576221	684
chr19.fa	46522190	46522307	MIR769	0	0
chr19.fa	46522412	46526556	PGLYRP1	0	0
chr19.fa	46543006	46544274	IGFL4	0	0
chr19.fa	46623328	46627931	IGFL3	1.728961152	76
chr19.fa	46651039	46664561	IGFL2	0	0
chr19.fa	46713499	46718094	DKFZp434J0226	0.700470237	76
chr19.fa	46733009	46734500	IGFL1	60.79090665	3104
chr19.fa	46800305	46846690	HIF3A	0.189764029	76
chr19.fa	46850294	46894105	PPP5C	19.25306679	2636
chr19.fa	46913586	46916919	CCDC8	0.343382528	76
chr19.fa	46969748	46974820	PNMAL1	5.884040353	1444
chr19.fa	46994448	46999169	PNMAL2	0	0
chr19.fa	47104512	47114039	CALM3	111.5047409	16725
chr19.fa	47123725	47128354	PTGIR	0	0
chr19.fa	47137333	47137939	GNG8	0	0
chr19.fa	47150869	47164395	DACT3	0	0
chr19.fa	47177573	47220384	PRKD2	16.2066011	3875
chr19.fa	47212550	47212602	MIR320E	0	0
chr19.fa	47222768	47249720	STRN4	16.15162977	3420

chr19.fa	47249303	47261832	FKRP	0.668993505	152
chr19.fa	47278140	47291842	SLC1A5	11.67831931	2486
chr19.fa	47333842	47333961	SNAR-E	0	0
chr19.fa	47341423	47354203	AP2S1	70.64959977	4175
chr19.fa	47421933	47508333	GRLF1	31.29389564	18468
chr19.fa	47524143	47549017	NPAS1	0	0
chr19.fa	47549167	47551882	TMEM160	6.989942943	304
chr19.fa	47567447	47617009	ZC3H4	17.96643656	7296
chr19.fa	47634080	47713893	SAE1	25.35804669	4408
chr19.fa	47724079	47736023	BBC3	1.09264923	152
chr19.fa	47730199	47730278	MIR3190	0	0
chr19.fa	47759731	47775210	CCDC9	6.60981246	912
chr19.fa	47777603	47778980	PRR24	4.153121318	380
chr19.fa	47813104	47825327	C5AR1	0	0
chr19.fa	47840371	47845272	GPR77	0.889330627	76
chr19.fa	47852538	47885961	DHX34	3.141648923	912
chr19.fa	47906381	47922785	MEIS3	8.130183787	1064
chr19.fa	47931279	47975307	SLC8A2	0	0
chr19.fa	47978400	47987521	KPTN	3.211982098	360
chr19.fa	47990891	48018497	NAPA	71.94270608	8799
chr19.fa	48023947	48059113	ZNF541	0.494290113	152
chr19.fa	48111453	48206534	GLTSCR1	4.587168882	1748
chr19.fa	48216601	48246391	EHD2	0	0
chr19.fa	48248793	48260323	GLTSCR2	198.6975471	20080
chr19.fa	48259110	48259219	SNORD23	0	0
chr19.fa	48281842	48287943	SEPW1	276.8589409	16177
chr19.fa	48304500	48306861	TPRX1	0	0
chr19.fa	48325099	48346586	CRX	0	0
chr19.fa	48373723	48389654	SULT2A1	0	0
chr19.fa	48442798	48442917	SNAR-C4	0	0
chr19.fa	48453553	48453671	SNAR-C3	0	0
chr19.fa	48471303	48495427	BSPH1	0	0
chr19.fa	48497908	48528410	ELSPBP1	0	0
chr19.fa	48533210	48547304	CABP5	0	0
chr19.fa	48551100	48614109	PLA2G4C	2.675371594	456
chr19.fa	48618703	48673560	LIG1	4.826482408	988
chr19.fa	48711343	48759203	CARD8	3.573136179	1368
chr19.fa	48774654	48790863	ZNF114	0.518838952	76
chr19.fa	48799709	48823332	CCDC114	0	0
chr19.fa	48828629	48833810	EMP3	0	0
chr19.fa	48835616	48867186	TMEM143	2.544645263	380
chr19.fa	48867651	48879634	SYNGR4	0	0
chr19.fa	48885827	48894810	KDELR1	44.84334851	4636

chr19.fa	48898132	48948188	GRIN2D	0.898969435	304
chr19.fa	48949030	48957164	GRWD1	11.65121016	1900
chr19.fa	48958766	48969367	KCNJ14	0.329827955	76
chr19.fa	48972465	48985571	CYTH2	9.689562164	2964
chr19.fa	48988528	49016446	LMTK3	1.151084502	380
chr19.fa	49055429	49102684	SULT2B1	28.5134008	2660
chr19.fa	49103857	49116694	FAM83E	2.845858008	380
chr19.fa	49110000	49110971	SPACA4	0	0
chr19.fa	49118588	49122433	RPL18	773.9899422	32942
chr19.fa	49122548	49133662	SPHK2	11.58900973	2280
chr19.fa	49133817	49140639	DBP	6.269893878	684
chr19.fa	49141272	49149451	CA11	4.439273425	456
chr19.fa	49141402	49185502	SEC1	0	0
chr19.fa	49164664	49176264	NTN5	0	0
chr19.fa	49199228	49209191	FUT2	0.741133957	152
chr19.fa	49216255	49222976	MAMSTR	0	0
chr19.fa	49223842	49243970	RASIP1	1.386632868	304
chr19.fa	49244145	49250166	IZUMO1	0	0
chr19.fa	49251268	49258647	FUT1	0.269585406	76
chr19.fa	49259344	49261582	FGF21	0	0
chr19.fa	49298319	49314320	BCAT2	40.1891598	5852
chr19.fa	49316274	49339934	HSD17B14	14.80490759	1216
chr19.fa	49340354	49371884	PLEKHA4	5.197727115	1064
chr19.fa	49375649	49379319	PPP1R15A	15.85056763	2508
chr19.fa	49384222	49401996	TULP2	0.650318315	76
chr19.fa	49403307	49426540	NUCB1	20.85943436	3572
chr19.fa	49436939	49448226	DHDH	6.353028595	456
chr19.fa	49458117	49465055	BAX	20.96019002	1748
chr19.fa	49468566	49470136	FTL	4064.608296	235068
chr19.fa	49471382	49496610	GYS1	18.98182472	4560
chr19.fa	49497156	49519182	RUVBL2	18.4614797	1824
chr19.fa	49519237	49520347	LHB	0	0
chr19.fa	49526127	49527593	CGB	0	0
chr19.fa	49534926	49535044	SNAR-G2	0	0
chr19.fa	49535130	49536495	CGB2	0	0
chr19.fa	49538826	49540191	CGB1	0	0
chr19.fa	49540277	49540404	SNAR-G1	0	0
chr19.fa	49547102	49548568	CGB5	0	0
chr19.fa	49550895	49552368	CGB8	0	0
chr19.fa	49557531	49558997	CGB7	0	0
chr19.fa	49564397	49567124	NTF4	0	0
chr19.fa	49570675	49576198	KCNA7	0	0
chr19.fa	49588465	49611870	SNRNP70	19.91633726	2543

chr19.fa	49617618	49621653	LIN7B	8.282296222	380
chr19.fa	49621718	49622397	C19orf73	5.049681051	228
chr19.fa	49622663	49654280	PPFIA3	3.396474904	1064
chr19.fa	49654456	49658681	HRC	0	0
chr19.fa	49661016	49715098	TRPM4	12.29565483	3344
chr19.fa	49792892	49828474	SLC6A16	0.78044222	152
chr19.fa	49812054	49812125	MIR4324	0	0
chr19.fa	49838677	49843854	CD37	0	0
chr19.fa	49843862	49865714	TEAD2	6.376673796	912
chr19.fa	49866987	49878373	DKKL1	0	0
chr19.fa	49891475	49921256	CCDC155	0	0
chr19.fa	49925671	49926698	PTH2	0	0
chr19.fa	49929688	49932075	LOC645971	0	0
chr19.fa	49932655	49944808	SLC17A7	0	0
chr19.fa	49949550	49955115	PIH1D1	19.29117021	1596
chr19.fa	49956473	49974305	ALDH16A1	2.211052149	456
chr19.fa	49977486	49989488	FLT3LG	0	0
chr19.fa	49993223	49993304	SNORD32A	0	0
chr19.fa	49993874	49993956	SNORD33	0	0
chr19.fa	49994164	49994229	SNORD34	0	0
chr19.fa	49994432	49994517	SNORD35A	0	0
chr19.fa	49999634	50002944	RPS11	1355.111105	54796
chr19.fa	50000976	50001062	SNORD35B	0	0
chr19.fa	50004042	50004125	MIR150	0	0
chr19.fa	50015536	50029685	FCGRT	6.537521401	912
chr19.fa	50030875	50046890	RCN3	0	0
chr19.fa	50058968	50083803	NOSIP	19.75172449	1440
chr19.fa	50084587	50094265	PRRG2	4.108391225	380
chr19.fa	50094912	50129696	PRR12	9.891525309	4560
chr19.fa	50138552	50143400	RRAS	12.80846952	836
chr19.fa	50145382	50161906	SCAF1	14.86153559	4256
chr19.fa	50162826	50168103	IRF3	10.00282342	1064
chr19.fa	50168399	50177173	BCL2L12	3.554611595	380
chr19.fa	50180496	50191704	PRMT1	35.42412479	3467
chr19.fa	50191942	50194247	C19orf76	0	0
chr19.fa	50194365	50216988	CPT1C	0	0
chr19.fa	50243011	50266515	TSKS	0	0
chr19.fa	50270180	50310123	AP2A1	15.48188324	3344
chr19.fa	50310370	50316567	FUZ	4.58144584	456
chr19.fa	50321536	50340237	MED25	13.19010607	2052
chr19.fa	50354416	50363999	PTOV1	45.97334812	5751
chr19.fa	50364460	50370822	PNKP	6.650777393	760
chr19.fa	50372297	50380644	AKT1S1	18.29490905	2964

chr19.fa	50380682	50392007	TBC1D17	22.19727076	3496
chr19.fa	50392914	50407578	IL4I1	0	0
chr19.fa	50410084	50432773	NUP62	11.05736924	2356
chr19.fa	50431959	50437193	ATF5	25.98938861	3969
chr19.fa	50452250	50464429	SIGLEC11	0	0
chr19.fa	50472912	50479076	SIGLEC16	0.711313896	76
chr19.fa	50479724	50528805	VRK3	26.47087718	3691
chr19.fa	50529212	50552031	ZNF473	4.148904339	1292
chr19.fa	50553937	50570052	FLJ26850	2.39117737	228
chr19.fa	50643459	50643577	SNAR-D	0	0
chr19.fa	50655805	50666538	IZUMO2	0	0
chr19.fa	50706885	50813801	MYH14	68.67710812	31519
chr19.fa	50818765	50832634	KCNC3	1.441604194	304
chr19.fa	50837057	50848005	NAPSB	0	0
chr19.fa	50861734	50868931	NAPSA	2.466781769	228
chr19.fa	50879685	50886267	NR1H2	19.93516305	2736
chr19.fa	50887593	50921271	POLD1	1.328197596	304
chr19.fa	50922195	50932250	SPIB	0	0
chr19.fa	50936160	50969583	MYBPC2	0	0
chr19.fa	50970045	50980003	FAM71E1	1.095360144	76
chr19.fa	50981186	50986608	C19orf63	12.50183495	1539
chr19.fa	51009259	51014345	JOSD2	4.465328327	228
chr19.fa	51014857	51017947	ASPDH	10.92709472	912
chr19.fa	51020150	51071302	LRRC4B	0	0
chr19.fa	51108220	51108342	SNAR-F	0	0
chr19.fa	51125234	51143092	SYT3	0.402420226	76
chr19.fa	51152702	51162567	LOC342918	0	0
chr19.fa	51165084	51220195	SHANK1	0.17229369	76
chr19.fa	51226605	51228981	CLEC11A	3.254001276	304
chr19.fa	51273858	51274928	GPR32	0	0
chr19.fa	51293672	51298481	ACPT	0	0
chr19.fa	51300961	51307974	C19orf48	39.39079542	4360
chr19.fa	51302286	51302382	SNORD88B	0	0
chr19.fa	51302696	51302792	SNORD88A	0	0
chr19.fa	51305582	51305678	SNORD88C	0	0
chr19.fa	51322404	51327043	KLK1	18.33466914	1064
chr19.fa	51328545	51334779	KLK15	0	0
chr19.fa	51358171	51364020	KLK3	437.7857652	55404
chr19.fa	51376689	51383823	KLK2	235.1052821	44256
chr19.fa	51385352	51399654	KLKP1	4.069685388	304
chr19.fa	51409608	51413994	KLK4	595.6722988	53276
chr19.fa	51446559	51456344	KLK5	0	0
chr19.fa	51461887	51472929	KLK6	0	0

chr19.fa	51479729	51487150	KLK7	0.580738171	76
chr19.fa	51499264	51504958	KLK8	0	0
chr19.fa	51505769	51512890	KLK9	0	0
chr19.fa	51516000	51523431	KLK10	0.348352539	76
chr19.fa	51525487	51531290	KLK11	21.32812139	2052
chr19.fa	51532348	51538148	KLK12	0	0
chr19.fa	51559463	51568367	KLK13	0	0
chr19.fa	51581154	51587502	KLK14	0	0
chr19.fa	51600863	51611647	CTU1	1.086474369	152
chr19.fa	51628137	51639520	SIGLEC9	0	0
chr19.fa	51645558	51656783	SIGLEC7	0	0
chr19.fa	51670585	51676780	SIGLECP3	0	0
chr19.fa	51728335	51743274	CD33	0	0
chr19.fa	51760964	51772582	C19orf75	0	0
chr19.fa	51815102	51834102	IGLON5	0	0
chr19.fa	51834795	51845378	VSIG10L	0	0
chr19.fa	51848409	51869672	ETFB	47.8824345	4610
chr19.fa	51870352	51872257	CLDND2	0	0
chr19.fa	51874874	51875960	NKG7	0	0
chr19.fa	51883163	51891210	LIM2	0	0
chr19.fa	51891543	51893828	LOC147646	0	0
chr19.fa	51913275	51921057	SIGLEC10	0	0
chr19.fa	51919777	51919919	LOC100129083	0	0
chr19.fa	51954251	51961708	SIGLEC8	0	0
chr19.fa	51979838	51986611	CEACAM18	0	0
chr19.fa	51994613	52005043	SIGLEC12	0	0
chr19.fa	52022784	52035110	SIGLEC6	0	0
chr19.fa	52074531	52092991	ZNF175	15.12027734	3800
chr19.fa	52095036	52097633	FLJ30403	1.345818541	152
chr19.fa	52114781	52133727	SIGLEC5	0	0
chr19.fa	52145806	52150132	SIGLEC14	0	0
chr19.fa	52195865	52195934	MIR99B	0	0
chr19.fa	52196039	52196117	MIRLET7E	0	0
chr19.fa	52196507	52196592	MIR125A	0	0
chr19.fa	52196593	52208443	NCRNA00085	3.496778748	456
chr19.fa	52216365	52227221	HAS1	0	0
chr19.fa	52249023	52255150	FPR1	0	0
chr19.fa	52264453	52273779	FPR2	0	0
chr19.fa	52298411	52329334	FPR3	0	0
chr19.fa	52359056	52391229	ZNF577	9.800257847	2204
chr19.fa	52392488	52408305	ZNF649	19.8630226	4256
chr19.fa	52430688	52449011	ZNF613	28.626958	4636
chr19.fa	52467593	52490079	ZNF350	41.0709601	6384

chr19.fa	52494587	52511483	ZNF615	19.76798998	5371
chr19.fa	52516577	52531680	ZNF614	8.812280046	2736
chr19.fa	52536677	52552073	ZNF432	5.783134083	988
chr19.fa	52567719	52599018	ZNF841	6.79204617	1748
chr19.fa	52617653	52643191	ZNF616	9.39121094	1900
chr19.fa	52658125	52674896	ZNF836	4.464424689	988
chr19.fa	52693055	52729678	PPP2R1A	93.93545339	15649
chr19.fa	52772824	52795976	ZNF766	10.00327524	1976
chr19.fa	52785050	52785143	MIR643	0	0
chr19.fa	52800426	52829174	ZNF480	5.311434926	1672
chr19.fa	52839498	52870376	ZNF610	3.363642715	532
chr19.fa	52873170	52889046	ZNF880	27.32481531	4104
chr19.fa	52901121	52921657	ZNF528	3.165143517	836
chr19.fa	52934667	52942699	ZNF534	0	0
chr19.fa	52956829	53020131	ZNF578	1.528202858	684
chr19.fa	53030909	53059303	ZNF808	5.876811247	1292
chr19.fa	53073526	53090427	ZNF701	3.47719992	1292
chr19.fa	53099937	53103405	ZNF137P	2.303976281	380
chr19.fa	53115618	53193886	ZNF83	8.491187261	1900
chr19.fa	53206066	53238297	ZNF611	9.491815997	2964
chr19.fa	53268748	53290034	ZNF600	6.291581196	1064
chr19.fa	53300661	53324922	ZNF28	10.68823302	3344
chr19.fa	53341785	53360902	ZNF468	14.48155571	3876
chr19.fa	53379425	53394599	ZNF320	7.823248	3192
chr19.fa	53430388	53445847	ZNF321	5.884943991	912
chr19.fa	53452632	53466114	ZNF816	8.912433283	1596
chr19.fa	53471504	53496784	ZNF702P	14.51288184	2888
chr19.fa	53517344	53519833	HERV-V1	0	0
chr19.fa	53547991	53554380	HERV-V2	0	0
chr19.fa	53569867	53606687	ZNF160	9.211386932	2652
chr19.fa	53611132	53636173	ZNF415	5.771085574	1140
chr19.fa	53641957	53662322	ZNF347	3.01016956	912
chr19.fa	53666552	53696619	ZNF665	0.695650833	152
chr19.fa	53738638	53758111	ZNF677	1.310727257	304
chr19.fa	53761545	53762855	VN1R2	0	0
chr19.fa	53770013	53770918	VN1R4	0	0
chr19.fa	53784811	53788200	LOC646508	0	0
chr19.fa	53792854	53794875	BIRC8	0	0
chr19.fa	53837002	53858122	ZNF845	6.106636571	1748
chr19.fa	53868968	53889841	ZNF525	7.137386582	2888
chr19.fa	53898397	53915262	ZNF765	4.751480434	1444
chr19.fa	53935227	53961515	ZNF761	54.7555069	15168
chr19.fa	53945049	53947925	LOC147804	18.30093331	3496

chr19.fa	53970989	53997546	ZNF813	9.29241316	2584
chr19.fa	54024177	54083523	ZNF331	4.848320332	1824
chr19.fa	54102887	54106751	LOC284379	4.679490588	380
chr19.fa	54135310	54140263	DPRX	0	0
chr19.fa	54169927	54172508	MIR512-2	0	0
chr19.fa	54175222	54175294	MIR1323	0	0
chr19.fa	54177451	54177574	MIR498	0	0
chr19.fa	54178965	54179051	MIR520E	0	0
chr19.fa	54183194	54183277	MIR519E	0	0
chr19.fa	54185413	54185499	MIR520F	0	0
chr19.fa	54189723	54189809	MIR519C	0	0
chr19.fa	54191735	54191821	MIR1283-1	0	0
chr19.fa	54194135	54194219	MIR520A	0	0
chr19.fa	54197647	54197729	MIR526B	0	0
chr19.fa	54198467	54198547	MIR519B	0	0
chr19.fa	54200787	54200871	MIR525	0	0
chr19.fa	54201639	54201725	MIR523	0	0
chr19.fa	54203269	54203355	MIR518F	0	0
chr19.fa	54204481	54204541	MIR520B	0	0
chr19.fa	54205991	54206073	MIR518B	0	0
chr19.fa	54209506	54209590	MIR526A1	0	0
chr19.fa	54210707	54210793	MIR520C	0	0
chr19.fa	54211989	54212089	MIR518C	0	0
chr19.fa	54214256	54214342	MIR524	0	0
chr19.fa	54215522	54215608	MIR517A	0	0
chr19.fa	54216601	54216688	MIR519D	0	0
chr19.fa	54219848	54219934	MIR521-2	0	0
chr19.fa	54223350	54223436	MIR520D	0	0
chr19.fa	54224330	54224396	MIR517B	0	0
chr19.fa	54225420	54225509	MIR520G	0	0
chr19.fa	54228696	54228780	MIR516B2	0	0
chr19.fa	54230176	54230240	MIR526A2	0	0
chr19.fa	54233092	54233179	MIR518E	0	0
chr19.fa	54234260	54234344	MIR518A1	0	0
chr19.fa	54238131	54238217	MIR518D	0	0
chr19.fa	54240099	54240188	MIR516B1	0	0
chr19.fa	54242587	54242673	MIR518A2	0	0
chr19.fa	54244567	54244661	MIR517C	0	0
chr19.fa	54245766	54245853	MIR520H	0	0
chr19.fa	54251890	54251976	MIR521-1	0	0
chr19.fa	54254465	54254551	MIR522	0	0
chr19.fa	54255651	54255735	MIR519A1	0	0
chr19.fa	54257272	54257356	MIR527	0	0

chr19.fa	54259995	54260084	MIR516A1	0	0
chr19.fa	54261486	54261572	MIR1283-2	0	0
chr19.fa	54264387	54264476	MIR516A2	0	0
chr19.fa	54265598	54265684	MIR519A2	0	0
chr19.fa	54290929	54290995	MIR371	0	0
chr19.fa	54291144	54291210	MIR372	0	0
chr19.fa	54291959	54292027	MIR373	0	0
chr19.fa	54296855	54327648	NLRP12	0	0
chr19.fa	54369611	54379689	MYADM	0.661011367	152
chr19.fa	54385467	54410901	PRKCG	0	0
chr19.fa	54415991	54446969	CACNG7	0	0
chr19.fa	54466290	54493469	CACNG8	0	0
chr19.fa	54495542	54515920	CACNG6	0	0
chr19.fa	54544080	54567207	VSTM1	0	0
chr19.fa	54573201	54584634	TARM1	0	0
chr19.fa	54597933	54604148	OSCAR	0	0
chr19.fa	54606160	54610281	NDUFA3	64.66721346	1520
chr19.fa	54610320	54618789	TFPT	12.15754878	758
chr19.fa	54619056	54635150	PRPF31	12.53873351	1596
chr19.fa	54641436	54659378	CNOT3	8.526730364	1596
chr19.fa	54659447	54663446	LENG1	8.375220354	456
chr19.fa	54663834	54676944	TMC4	11.53840599	1900
chr19.fa	54677106	54693733	MBOAT7	2.25834255	456
chr19.fa	54694119	54698394	TSEN34	12.37412075	1976
chr19.fa	54704726	54711515	RPS9	871.7003443	41268
chr19.fa	54720147	54726959	LILRB3	0	0
chr19.fa	54740469	54746724	LILRA6	0	0
chr19.fa	54754270	54761167	LILRB5	0	0
chr19.fa	54777675	54785033	LILRB2	0	0
chr19.fa	54799855	54804221	LILRA3	0	0
chr19.fa	54818353	54824409	LILRA5	0	0
chr19.fa	54844692	54850421	LILRA4	0	0
chr19.fa	54865235	54876721	LAIR1	0	0
chr19.fa	54926635	54948054	TTYH1	0.56417147	76
chr19.fa	54960065	54972978	LENG8	5.796236838	1444
chr19.fa	54973197	54974894	LENG9	9.43729649	1064
chr19.fa	54976210	54984422	CDC42EP5	61.48188869	3572
chr19.fa	55014013	55021897	LAIR2	0	0
chr19.fa	55043909	55055195	KIR3DX1	0	0
chr19.fa	55085257	55099027	LILRA2	0	0
chr19.fa	55105113	55112540	LILRA1	0	0
chr19.fa	55128629	55149004	LILRB1	0	0
chr19.fa	55174124	55179846	LILRB4	0	0

chr19.fa	55219601	55224887	LILRP2	0	0
chr19.fa	55235984	55248003	KIR3DL3	0	0
chr19.fa	55249974	55264504	KIR2DL3	0	0
chr19.fa	55266474	55309738	KIR3DP1	0.884511224	76
chr19.fa	55281265	55295778	KIR2DL1	0	0
chr19.fa	55315067	55325972	KIR2DL4	0	0
chr19.fa	55327893	55342233	KIR3DL1	0	0
chr19.fa	55344174	55360023	KIR2DS4	0	0
chr19.fa	55361898	55378662	KIR3DL2	0	0
chr19.fa	55385549	55401839	FCAR	0	0
chr19.fa	55417508	55424439	NCR1	0	0
chr19.fa	55434877	55458873	NLRP7	0	0
chr19.fa	55476652	55512510	NLRP2	5.303452788	1292
chr19.fa	55525075	55549632	GP6	0	0
chr19.fa	55555692	55580914	RDH13	4.550270321	836
chr19.fa	55587221	55599291	EPS8L1	9.460339265	1718
chr19.fa	55602281	55628927	PPP1R12C	10.44560615	2028
chr19.fa	55644161	55660606	TNNT1	0	0
chr19.fa	55663136	55669100	TNNI3	0	0
chr19.fa	55670031	55678018	C19orf51	0	0
chr19.fa	55684469	55691720	SYT5	0	0
chr19.fa	55692615	55720874	PTPRH	0	0
chr19.fa	55738002	55740632	TMEM86B	0	0
chr19.fa	55741147	55770038	PPP6R1	27.32195379	6598
chr19.fa	55773591	55791751	HSPBP1	7.198833981	912
chr19.fa	55795534	55823903	BRSK1	0	0
chr19.fa	55824227	55836708	TMEM150B	0	0
chr19.fa	55851221	55859489	SUV420H2	1.487087318	228
chr19.fa	55861070	55866182	COX6B2	0	0
chr19.fa	55866276	55874620	FAM71E2	0	0
chr19.fa	55875757	55881814	IL11	0	0
chr19.fa	55888204	55889612	TMEM190	0	0
chr19.fa	55890612	55895627	LOC388564	3.45807291	152
chr19.fa	55897300	55903451	RPL28	98.69762688	31692
chr19.fa	55912650	55919325	UBE2S	0.961019261	76
chr19.fa	55940105	55954230	SHISA7	0	0
chr19.fa	55964346	55973049	ISOC2	25.52657522	1900
chr19.fa	55987699	55995854	ZNF628	1.190091553	304
chr19.fa	55996557	55998935	NAT14	1.695677144	152
chr19.fa	55999870	56030466	SSC5D	0	0
chr19.fa	56041100	56048435	SBK2	0	0
chr19.fa	56052023	56056909	LOC100130827	0.901981563	76
chr19.fa	56088891	56092211	ZNF579	4.2236051	608

chr19.fa	56102737	56110893	FIZ1	3.872692253	684
chr19.fa	56111730	56114504	ZNF524	8.256843746	608
chr19.fa	56124959	56129907	ZNF865	1.387687113	456
chr19.fa	56132107	56135941	ZNF784	3.440753178	456
chr19.fa	56152392	56154836	ZNF580	11.61295614	1216
chr19.fa	56154986	56156989	ZNF581	11.20330681	912
chr19.fa	56158954	56164526	CCDC106	4.02224438	532
chr19.fa	56165416	56186082	U2AF2	28.14155366	5828
chr19.fa	56186561	56207133	EPN1	30.86903506	6688
chr19.fa	56219798	56249768	NLRP9	0.985568099	228
chr19.fa	56270507	56274541	RFPL4A	0	0
chr19.fa	56296763	56347943	NLRP11	0	0
chr19.fa	56348129	56393220	NLRP4	0	0
chr19.fa	56407311	56443702	NLRP13	0	0
chr19.fa	56459198	56499995	NLRP8	0	0
chr19.fa	56511092	56573174	NLRP5	0	0
chr19.fa	56598732	56632649	ZNF787	7.124133221	912
chr19.fa	56652556	56672262	ZNF444	5.602557043	760
chr19.fa	56687389	56697144	GALP	0	0
chr19.fa	56701058	56704421	ZSCAN5B	0	0
chr19.fa	56732679	56739659	ZSCAN5A	1.786643393	228
chr19.fa	56879468	56891196	ZNF542	7.640863684	1900
chr19.fa	56894648	56904889	ZNF582	0.996562364	152
chr19.fa	56905045	56910539	LOC386758	0.687518089	76
chr19.fa	56915383	56936400	ZNF583	3.005350156	532
chr19.fa	56950693	56988770	ZNF667	3.07824364	912
chr19.fa	56989243	57006805	LOC100128252	26.33503023	3116
chr19.fa	57019212	57040269	ZNF471	1.613446065	532
chr19.fa	57050317	57068170	ZFP28	5.601201586	1520
chr19.fa	57078890	57094262	ZNF470	2.564525305	1216
chr19.fa	57106664	57135544	ZNF71	1.46419515	304
chr19.fa	57154527	57168614	LOC147670	0	0
chr19.fa	57174953	57184246	ZNF835	2.043879076	228
chr19.fa	57285923	57352097	ZIM2	0	0
chr19.fa	57321445	57351954	PEG3	3.460783825	1444
chr19.fa	57352270	57359922	MIMT1	0	0
chr19.fa	57631509	57643293	USP29	0	0
chr19.fa	57645464	57656570	ZIM3	0	0
chr19.fa	57663094	57678856	DUXA	0	0
chr19.fa	57702868	57734214	ZNF264	7.716618689	6232
chr19.fa	57742377	57746916	AURKC	0.879842426	76
chr19.fa	57752053	57774106	ZNF805	2.373707031	1596
chr19.fa	57791853	57805436	ZNF460	13.38197859	3420

chr19.fa	57831865	57842144	ZNF543	4.98868547	1216
chr19.fa	57862645	57871265	ZNF304	9.849506131	2888
chr19.fa	57874879	57876721	TRAPPC2P1	0	0
chr19.fa	57879912	57890925	ZNF547	1.945683722	335
chr19.fa	57901218	57913919	ZNF548	5.739608842	1734
chr19.fa	57922529	57933307	ZNF17	4.274058235	760
chr19.fa	57946693	57957191	ZNF749	2.735463537	532
chr19.fa	57966542	57968107	VN1R1	0	0
chr19.fa	57980954	57988938	ZNF772	6.125462367	2204
chr19.fa	57999079	58006048	ZNF419	2.90263661	456
chr19.fa	58011309	58019510	ZNF773	2.616785716	380
chr19.fa	58038693	58052244	ZNF549	5.14561731	1408
chr19.fa	58058343	58067725	ZNF550	3.995135233	304
chr19.fa	58082934	58090243	ZNF416	3.649644216	608
chr19.fa	58095628	58103758	ZIK1	3.421776775	608
chr19.fa	58111253	58119637	ZNF530	0	0
chr19.fa	58125830	58133636	ZNF134	13.23830011	1976
chr19.fa	58144535	58154147	ZNF211	8.20187242	1368
chr19.fa	58180303	58190520	ZSCAN4	0.509651963	76
chr19.fa	58193357	58201169	ZNF551	8.041024814	1976
chr19.fa	58211810	58220579	ZNF154	3.750098666	684
chr19.fa	58231119	58238995	ZNF671	4.23234027	684
chr19.fa	58258164	58269527	ZNF776	7.786500045	2736
chr19.fa	58281025	58291984	ZNF586	4.650724771	684
chr19.fa	58318450	58326281	ZNF552	14.59963111	2280
chr19.fa	58336667	58338830	FKBP1AP1	1.393259549	152
chr19.fa	58361269	58376487	ZNF587	6.855903272	3116
chr19.fa	58380747	58400442	ZNF814	3.461386251	1444
chr19.fa	58417142	58427978	ZNF417	3.407318563	1064
chr19.fa	58433252	58446740	ZNF418	4.64786325	1140
chr19.fa	58452201	58459077	ZNF256	3.107461276	456
chr19.fa	58469805	58485902	C19orf18	0	0
chr19.fa	58488441	58514714	ZNF606	5.221221709	1368
chr19.fa	58515751	58518574	LOC100128398	1.005297534	108
chr19.fa	58545434	58565999	ZSCAN1	0	0
chr19.fa	58570607	58581110	ZNF135	1.348529456	304
chr19.fa	58595209	58629793	ZSCAN18	11.67967476	3648
chr19.fa	58637695	58662148	ZNF329	9.054906911	2052
chr19.fa	58694396	58724927	ZNF274	13.71271018	2584
chr19.fa	58740070	58775008	ZNF544	16.30494706	3800
chr19.fa	58790318	58807254	ZNF8	5.697137845	836
chr19.fa	58838385	58853712	ZSCAN22	1.972190443	608
chr19.fa	58858172	58864865	A1BG	0.877131511	76

chr19.fa	58863336	58865223	A1BG-AS1	0	0
chr19.fa	58865725	58874120	ZNF497	0	0
chr19.fa	58878990	58892389	ZNF837	1.126083844	152
chr19.fa	58898636	58906171	RPS5	569.9871351	28044
chr19.fa	58907457	58908446	LOC646862	1.156205119	76
chr19.fa	58920063	58929692	ZNF584	5.066850178	760
chr19.fa	58944181	58951589	ZNF132	7.552608349	1520
chr19.fa	58962971	58969199	ZNF324B	1.146566311	228
chr19.fa	58978412	58984945	ZNF324	2.12550773	456
chr19.fa	58987795	58992597	ZNF446	5.798495933	760
chr19.fa	59009700	59023432	SLC27A5	1.935894307	304
chr19.fa	59024897	59030921	ZBTB45	5.353152891	836
chr19.fa	59055836	59062082	TRIM28	54.87794988	10782
chr19.fa	59062933	59066486	CHMP2A	96.75585892	6630
chr19.fa	59067079	59070343	UBE2M	33.68883817	3409
chr19.fa	59070553	59086164	LOC100131691	0.56914148	76
chr19.fa	59073284	59084942	MZF1	1.357716445	228
chr19.fa	59086766	59095762	MGC2752	5.824099017	1292
chr2.fa	38814	46588	FAM110C	5.487644381	1444
chr2.fa	218136	264068	SH3YL1	84.38339485	10018
chr2.fa	264869	278282	ACP1	64.62052548	7144
chr2.fa	279561	288308	FAM150B	0	0
chr2.fa	667973	677439	TMEM18	11.31671341	1596
chr2.fa	779837	864112	LOC339822	0	0
chr2.fa	946554	1371384	SNTG2	0	0
chr2.fa	1417233	1546499	TPO	0	0
chr2.fa	1635659	1748291	PXDN	0.503477102	228
chr2.fa	1792885	2335045	MYT1L	0	0
chr2.fa	2323004	2330880	LOC730811	0	0
chr2.fa	3192741	3381653	TSSC1	9.311238957	1064
chr2.fa	3383446	3483342	TTC15	15.87210435	2660
chr2.fa	3501690	3523350	ADI1	181.6621591	20216
chr2.fa	3592691	3605940	RNASEH1	13.30125357	1444
chr2.fa	3622853	3628509	RPS7	359.7796466	17391
chr2.fa	3642637	3692047	COLEC11	0.82818444	76
chr2.fa	3705786	3750260	ALLC	0	0
chr2.fa	4675808	4703812	LOC727982	0	0
chr2.fa	5832799	5841517	SOX11	0	0
chr2.fa	6072819	6120350	LOC150622	0.289314841	228
chr2.fa	6122110	6128364	LOC400940	0	0
chr2.fa	6988451	7005936	CMPK2	2.680642818	532
chr2.fa	7017796	7038363	RSAD2	0.325912189	76
chr2.fa	7052407	7058813	LOC386597	0	0

chr2.fa	7057523	7184309	RNF144A	7.379411022	2812
chr2.fa	8062556	8116945	LOC339788	0	0
chr2.fa	8147901	8468549	C2orf46	0	0
chr2.fa	8822113	8824583	ID2	59.38138162	5378
chr2.fa	8868987	8977755	KIDINS220	6.997322655	3420
chr2.fa	8996701	9143876	MBOAT2	13.54026589	3420
chr2.fa	9346894	9545812	ASAP2	49.09391215	18636
chr2.fa	9545815	9563643	ITGB1BP1	11.87350517	1520
chr2.fa	9563868	9613227	CPSF3	22.14350429	3192
chr2.fa	9614670	9628591	IAH1	31.33470997	1885
chr2.fa	9629411	9695917	ADAM17	4.510209026	1064
chr2.fa	9724106	9771106	YWHAQ	128.8359198	18529
chr2.fa	9983571	10074545	TAF1B	3.982936117	608
chr2.fa	10091792	10142412	GRHL1	39.02813527	9342
chr2.fa	10183682	10194963	KLF11	2.397954657	684
chr2.fa	10196926	10220538	CYS1	22.28356821	4023
chr2.fa	10262695	10271546	RRM2	0.333291902	76
chr2.fa	10281509	10351856	C2orf48	0	0
chr2.fa	10332740	10332797	MIR4261	0	0
chr2.fa	10443040	10567743	HPCAL1	10.03113742	1444
chr2.fa	10580508	10588453	ODC1	257.0096234	35188
chr2.fa	10586840	10586975	SNORA80B	0	0
chr2.fa	10710894	10830112	NOL10	15.34934963	3562
chr2.fa	10861775	10925236	ATP6V1C2	1.037226085	152
chr2.fa	10927407	10952960	PDIA6	17.67742293	1520
chr2.fa	11052063	11054351	KCNF1	0	0
chr2.fa	11273179	11286916	C2orf50	0	0
chr2.fa	11295540	11318998	PQLC3	6.408752953	760
chr2.fa	11321778	11484711	ROCK2	21.80629662	9268
chr2.fa	11584501	11606297	E2F6	2.32641663	532
chr2.fa	11674242	11782912	GREB1	58.06703981	39824
chr2.fa	11798304	11810329	NTSR2	0	0
chr2.fa	11886740	11967533	LPIN1	5.33989953	1899
chr2.fa	11977059	11977112	MIR4262	0	0
chr2.fa	12856998	12882858	TRIB2	23.90017701	6992
chr2.fa	12877493	12877570	MIR3125	0	0
chr2.fa	14772810	14780168	FAM84A	20.71937043	8740
chr2.fa	15307032	15701454	NBAS	10.80013355	5222
chr2.fa	15731770	15771225	DDX1	45.36007898	8150
chr2.fa	16080020	16081845	MYCNOS	0	0
chr2.fa	16080683	16087129	MYCN	3.075683332	532
chr2.fa	16730730	16847134	FAM49A	9.743479245	3040
chr2.fa	17691986	17699706	RAD51AP2	0	0

chr2.fa	17721807	17837706	VSNL1	0.570948757	76
chr2.fa	17845079	17935096	SMC6	3.047068121	1064
chr2.fa	17935177	17966632	GEN1	1.247020761	532
chr2.fa	17997786	17998367	MSGN1	0	0
chr2.fa	18059945	18114225	KCNS3	5.895034618	912
chr2.fa	18741446	18741959	RDH14	4.453731637	152
chr2.fa	18744137	18768259	NT5C1B	0	0
chr2.fa	19551246	19558372	OSR1	0.604383371	76
chr2.fa	20068615	20084808	FLJ12334	0	0
chr2.fa	20096514	20101744	TTC32	8.385461587	456
chr2.fa	20110029	20189884	WDR35	2.80218216	1292
chr2.fa	20191813	20212455	MATN3	0	0
chr2.fa	20232411	20251789	LAPTM4A	18.80666951	2204
chr2.fa	20400558	20425194	SDC1	20.50776848	4484
chr2.fa	20448453	20527144	PUM2	45.88915916	18620
chr2.fa	20646835	20649201	RHOB	38.68550578	6080
chr2.fa	20817564	20850864	HS1BP3	14.76605115	2356
chr2.fa	20866424	20871250	GDF7	1.148072375	152
chr2.fa	20884818	21022827	C2orf43	7.909997271	1500
chr2.fa	21224301	21266945	APOB	0	0
chr2.fa	23608298	23931483	KLHL29	0.900626105	304
chr2.fa	23971534	24149934	ATAD2B	8.101719182	4332
chr2.fa	24163376	24223693	UBXN2A	12.09308926	1520
chr2.fa	24232953	24247145	MFSD2B	0	0
chr2.fa	24252206	24270296	C2orf44	5.584936098	1444
chr2.fa	24272584	24286550	FKBP1B	12.64129645	836
chr2.fa	24290454	24299314	SF3B14	53.44477964	2736
chr2.fa	24300305	24307728	TP53I3	10.25026969	1140
chr2.fa	24338244	24346151	PFN4	0	0
chr2.fa	24346350	24392507	LOC375190	5.060976529	456
chr2.fa	24397972	24414567	C2orf84	0	0
chr2.fa	24425735	24583397	ITSN2	62.52016902	27971
chr2.fa	24807346	24993570	NCOA1	30.62821547	14136
chr2.fa	25013136	25016174	C2orf79	57.80844867	1900
chr2.fa	25016252	25045245	CENPO	3.263037659	684
chr2.fa	25042039	25142055	ADCY3	0	0
chr2.fa	25166505	25194963	DNAJC27	2.292831409	760
chr2.fa	25194981	25262563	LOC729723	0.734657883	228
chr2.fa	25264973	25382004	EFR3B	0.306935786	152
chr2.fa	25383722	25391559	POMC	0.883908798	76
chr2.fa	25455845	25565459	DNMT3A	6.753942758	2527
chr2.fa	25551509	25551590	MIR1301	0	0
chr2.fa	25600112	25896503	DTNB	16.15644918	2654

chr2.fa	25962253	26101312	ASXL2	14.99768375	7144
chr2.fa	26149455	26205443	KIF3C	3.372076672	1216
chr2.fa	26256729	26360323	RAB10	45.61957376	11465
chr2.fa	26395960	26412532	FAM59B	0	0
chr2.fa	26413504	26467594	HADHA	82.6475058	16666
chr2.fa	26467616	26513333	HADHB	96.60690922	14035
chr2.fa	26531041	26569685	GPR113	0.951531059	304
chr2.fa	26568954	26618759	EPT1	2.783205757	1471
chr2.fa	26624784	26679579	C2orf39	0	0
chr2.fa	26680071	26781566	OTOF	0	0
chr2.fa	26785481	26802395	C2orf70	0	0
chr2.fa	26804073	26864211	CIB4	3.072821811	152
chr2.fa	26915581	26954066	KCNK3	1.726400844	456
chr2.fa	26987142	27004099	C2orf18	2.340724236	608
chr2.fa	27008882	27017455	CENPA	0	0
chr2.fa	27070969	27173219	DPYSL5	0	0
chr2.fa	27193525	27250087	MAPRE3	10.40554486	1292
chr2.fa	27255774	27264565	TMEM214	13.93108942	2812
chr2.fa	27274491	27293341	AGBL5	25.6017278	5472
chr2.fa	27293491	27294567	OST4	235.0534735	5244
chr2.fa	27301435	27309265	EMILIN1	0	0
chr2.fa	27309611	27323461	KHK	18.22713619	2337
chr2.fa	27322221	27341995	CGREF1	0	0
chr2.fa	27346657	27353624	ABHD1	1.646880679	152
chr2.fa	27353681	27357542	PREB	28.7781668	4028
chr2.fa	27359715	27362332	C2orf53	0	0
chr2.fa	27371945	27375819	TCF23	0	0
chr2.fa	27422455	27434898	SLC5A6	4.449213445	904
chr2.fa	27435176	27440046	C2orf28	55.12373948	4103
chr2.fa	27440258	27466654	CAD	2.737572026	1292
chr2.fa	27477440	27485960	SLC30A3	0	0
chr2.fa	27498289	27504296	DNAJC5G	0	0
chr2.fa	27505297	27530264	TRIM54	0	0
chr2.fa	27530308	27531130	UCN	0	0
chr2.fa	27532360	27545969	MPV17	46.45318003	3106
chr2.fa	27548721	27579868	GTF3C2	18.88347876	4915
chr2.fa	27587219	27593324	EIF2B4	19.47716908	2280
chr2.fa	27593389	27599994	SNX17	47.79884796	6484
chr2.fa	27600102	27603593	ZNF513	4.822717248	684
chr2.fa	27604061	27632496	PPM1G	23.87773666	3572
chr2.fa	27615490	27616443	FTH1P3	2.399611327	152
chr2.fa	27651473	27665124	NRBP1	29.12139872	4252
chr2.fa	27665233	27669348	KRTCAP3	3.389697617	228

chr2.fa	27667240	27712571	IFT172	4.508251143	1588
chr2.fa	27714750	27718126	FNDC4	11.64473409	1292
chr2.fa	27719706	27746550	GCKR	0	0
chr2.fa	27799389	27805589	C2orf16	0.922915848	380
chr2.fa	27805893	27845963	ZNF512	10.356899	2356
chr2.fa	27848506	27851862	CCDC121	3.75823141	684
chr2.fa	27851899	27873678	GPN1	25.44103081	3093
chr2.fa	27873714	27886337	SUPT7L	5.007963086	1368
chr2.fa	27886450	27917847	SLC4A1AP	11.6143116	2204
chr2.fa	27994584	28002608	MRPL33	68.96491689	2372
chr2.fa	28004266	28081438	RBKS	9.399193078	684
chr2.fa	28112323	28113981	LOC100302650	0.859962385	76
chr2.fa	28113557	28561767	BRE	25.36843853	3416
chr2.fa	28219234	28219316	MIR4263	0	0
chr2.fa	28615779	28637516	FOSL2	44.45357922	11780
chr2.fa	28718938	28866653	PLB1	0	0
chr2.fa	28974614	29025806	PPP1CB	86.33796434	28652
chr2.fa	29033700	29073475	SPDYA	0	0
chr2.fa	29072688	29093175	TRMT61B	6.674121381	608
chr2.fa	29117533	29171080	WDR43	17.22876655	4005
chr2.fa	29136528	29136616	SNORD92	0	0
chr2.fa	29149933	29150008	SNORD53	0	0
chr2.fa	29204164	29275096	FAM179A	0	0
chr2.fa	29284558	29297127	C2orf71	0	0
chr2.fa	29338308	29406679	CLIP4	8.831407055	2508
chr2.fa	29415640	30144432	ALK	0	0
chr2.fa	30369750	30383399	YPEL5	66.82088458	10564
chr2.fa	30454397	30482899	LBH	12.85636235	2508
chr2.fa	30670123	30867091	LCLAT1	2.940740022	988
chr2.fa	30945638	31030311	CAPN13	16.80209869	2991
chr2.fa	31133333	31361571	GALNT14	3.376444257	608
chr2.fa	31395922	31440411	CAPN14	0	0
chr2.fa	31456880	31491260	EHD3	2.637418789	684
chr2.fa	31557188	31637611	XDH	0.600618212	228
chr2.fa	31749656	31806040	SRD5A2	0.936319816	152
chr2.fa	32092894	32235698	MEMO1	3.790159961	456
chr2.fa	32248972	32264844	DPY30	44.52300876	2365
chr2.fa	32288680	32382706	SPAST	4.983564854	1725
chr2.fa	32390910	32449181	SLC30A6	2.305331738	760
chr2.fa	32449518	32490812	NLRC4	0.32546037	76
chr2.fa	32502958	32531658	YIPF4	3.494971472	456
chr2.fa	32582096	32843965	BIRC6	15.01650955	15656
chr2.fa	32757220	32757313	MIR558	0	0

chr2.fa	32853087	33046118	TTC27	7.217358565	1280
chr2.fa	33050510	33162270	LOC285045	0	0
chr2.fa	33152194	33171202	LOC100271832	0	0
chr2.fa	33172369	33624575	LTBP1	2.163761748	912
chr2.fa	33661416	33789798	RASGRP3	0.864932395	304
chr2.fa	33808729	33824362	FAM98A	30.02247665	5472
chr2.fa	33951128	33953284	MYADML	0	0
chr2.fa	36581892	36582713	LOC100288911	1.59868664	76
chr2.fa	36583370	36778278	CRIM1	11.01565127	4104
chr2.fa	36779404	36825332	FEZ2	14.77267783	2052
chr2.fa	36923833	37041937	VIT	0	0
chr2.fa	37075472	37193615	STRN	22.37227537	5168
chr2.fa	37208153	37311485	HEATR5B	15.50417298	6992
chr2.fa	37311594	37323738	CCDC75	2.731698378	228
chr2.fa	37333699	37384190	EIF2AK2	17.87230757	3477
chr2.fa	37394963	37415690	SULT6B1	0	0
chr2.fa	37428775	37458740	CEBPZ	26.77600569	5915
chr2.fa	37458774	37476303	C2orf56	3.106105819	456
chr2.fa	37477646	37544222	PRKD3	7.1756406	2812
chr2.fa	37571753	37600465	QPCT	0.672156239	76
chr2.fa	37870743	37899326	CDC42EP3	13.22579978	3496
chr2.fa	38152462	38294285	FAM82A1	1.037677904	228
chr2.fa	38294746	38303323	CYP1B1	7.996445328	2736
chr2.fa	38358247	38408993	C2orf58	0.652878623	76
chr2.fa	38522029	38604432	ATL2	6.800931946	1520
chr2.fa	38790328	38830178	HNRPLL	15.46260562	3116
chr2.fa	38893052	38961909	GALM	28.58057124	4712
chr2.fa	38970741	38978636	SRSF7	23.35031315	3859
chr2.fa	39005327	39009106	GEMIN6	11.57846728	532
chr2.fa	39024876	39103021	DHX57	8.302929295	2660
chr2.fa	39103103	39109850	MORN2	9.268014928	456
chr2.fa	39146504	39202590	ARHGEF33	0.602425489	152
chr2.fa	39186429	39186981	LOC375196	0	0
chr2.fa	39208690	39347604	SOS1	15.05340811	8314
chr2.fa	39405688	39456673	CDKL4	0	0
chr2.fa	39476422	39664219	MAP4K3	17.41883179	4757
chr2.fa	39892638	39945104	TMEM178	5.003746108	608
chr2.fa	39963200	40006416	THUMPD2	3.416806764	532
chr2.fa	40339286	40739575	SLC8A1	14.48170632	6004
chr2.fa	42104695	42121186	LOC388942	0	0
chr2.fa	42275161	42285668	PKDCC	0.457692765	76
chr2.fa	42396490	42559688	EML4	25.16527054	9272
chr2.fa	42577645	42588356	COX7A2L	104.8026068	7752

chr2.fa	42669157	42721237	KCNG3	0	0
chr2.fa	42795671	42936353	MTA3	25.49027909	3143
chr2.fa	42989639	42991401	OXER1	0.64926407	76
chr2.fa	42994229	43019751	HAAO	8.914391166	760
chr2.fa	43449541	43453745	ZFP36L2	54.23952946	13300
chr2.fa	43454350	43455994	LOC100129726	0	0
chr2.fa	43457975	43823185	THADA	10.91293772	4636
chr2.fa	43864439	43995126	PLEKHH2	1.152138747	532
chr2.fa	43902292	43903461	LOC728819	0	0
chr2.fa	44001178	44037149	DYNC2LI1	13.84674985	1289
chr2.fa	44039611	44065958	ABCG5	0	0
chr2.fa	44066103	44105605	ABCG8	0	0
chr2.fa	44113363	44223144	LRPPRC	27.32255621	12008
chr2.fa	44396000	44461742	PPM1B	16.69381271	5908
chr2.fa	44502597	44541090	SLC3A1	21.65358176	2437
chr2.fa	44544748	44589001	PREPL	15.90373168	6149
chr2.fa	44589043	44999729	C2orf34	2.948119734	304
chr2.fa	45169037	45173216	SIX3	0	0
chr2.fa	45232324	45236542	SIX2	0	0
chr2.fa	45401480	45482080	UNQ6975	0	0
chr2.fa	45615819	45838433	SRBD1	13.32384453	3268
chr2.fa	45879043	46415129	PRKCE	5.183871329	1900
chr2.fa	46524541	46613842	EPAS1	27.03098228	9272
chr2.fa	46706704	46711564	LOC388946	0	0
chr2.fa	46738986	46747096	ATP6V1E2	1.156807544	152
chr2.fa	46769867	46811827	RHOQ	28.99458816	8132
chr2.fa	46815214	46844251	PIGF	3.307767751	179
chr2.fa	46844325	46852881	CRIPT	11.93540438	1520
chr2.fa	46926099	46989927	SOCS5	4.173754391	1292
chr2.fa	47043807	47049799	LOC388948	0	0
chr2.fa	47055003	47086145	LOC100134259	0.945958623	76
chr2.fa	47129009	47168994	MCFD2	6.552130219	1976
chr2.fa	47168313	47303275	TTC7A	5.48975287	1824
chr2.fa	47314130	47382517	C2orf61	0.480133114	76
chr2.fa	47387221	47403740	CALM2	181.2597389	15670
chr2.fa	47596287	47614167	EPCAM	7.065697948	806
chr2.fa	47604814	47604909	MIR559	0	0
chr2.fa	47630263	47710360	MSH2	10.19047896	2128
chr2.fa	47747915	47797470	KCNK12	0	0
chr2.fa	48010221	48034058	MSH6	13.06148823	3724
chr2.fa	48034093	48132932	FBXO11	21.41788279	5852
chr2.fa	48541795	48606434	FOXN2	4.190471698	1520
chr2.fa	48667908	48742531	KLRAQ1	25.43681383	5430

chr2.fa	48757064	49003656	STON1-GTF2A1L	0	0
chr2.fa	48757308	48825654	STON1	8.526880971	1955
chr2.fa	48844937	48845034	GTF2A1L	0	0
chr2.fa	48913913	48982880	LHCGR	0	0
chr2.fa	49189296	49381666	FSHR	0	0
chr2.fa	50145643	51259674	NRXN1	0	0
chr2.fa	53897118	54014067	ASB3	2.896311142	85
chr2.fa	53994929	54002287	CHAC2	0	0
chr2.fa	54014080	54046495	ERLEC1	3.313490793	684
chr2.fa	54076259	54076342	MIR3682	0	0
chr2.fa	54080050	54082002	GPR75	1.172169394	152
chr2.fa	54087127	54087170	LOC100302652	0	0
chr2.fa	54091204	54197977	PSME4	27.8682031	13136
chr2.fa	54342410	54532435	ACYP2	4.683406354	380
chr2.fa	54480315	54483409	TSPYL6	0	0
chr2.fa	54558071	54588714	C2orf73	0	0
chr2.fa	54683454	54898583	SPTBN1	101.7517735	72392
chr2.fa	54756359	54756978	RPL23AP32	0	0
chr2.fa	54952149	55199156	EML6	2.186653917	1140
chr2.fa	55199327	55277734	RTN4	19.24704254	6648
chr2.fa	55399687	55459449	C2orf63	1.71284627	608
chr2.fa	55459039	55462989	RPS27A	408.5473454	35292
chr2.fa	55463756	55496384	MTIF2	19.72160322	3952
chr2.fa	55509455	55511607	PRORSD1P	0.531640494	76
chr2.fa	55514978	55647057	CCDC88A	0.699867811	456
chr2.fa	55746740	55772216	CCDC104	49.00791592	4227
chr2.fa	55775514	55844796	SMEK2	23.87803788	7068
chr2.fa	55861198	55921011	PNPT1	3.999502818	1216
chr2.fa	56093102	56150932	EFEMP1	1.52353406	304
chr2.fa	56210102	56210211	MIR217	0	0
chr2.fa	56216085	56216194	MIR216A	0	0
chr2.fa	56227849	56227930	MIR216B	0	0
chr2.fa	56411258	56613309	CCDC85A	0	0
chr2.fa	58273777	58387055	VRK2	12.83663292	1466
chr2.fa	58386378	58468515	FANCL	6.906657619	587
chr2.fa	58747888	59290901	FLJ30838	0	0
chr2.fa	60678302	60780633	BCL11A	0.614925817	304
chr2.fa	60983365	61029221	PAPOLG	3.409276446	1672
chr2.fa	61074895	61108449	FLJ16341	0	0
chr2.fa	61108752	61150178	REL	17.66371775	3040
chr2.fa	61167548	61245365	PUS10	2.102916774	532
chr2.fa	61244812	61279125	PEX13	7.85894171	2356
chr2.fa	61293006	61365169	KIAA1841	7.115398051	2607

chr2.fa	61368727	61372110	LOC339803	3.133516179	532
chr2.fa	61372243	61391964	C2orf74	3.518315459	228
chr2.fa	61404555	61414589	AHSA2	1.093402262	213
chr2.fa	61414687	61697849	USP34	29.28043905	21800
chr2.fa	61644379	61644512	SNORA70B	0	0
chr2.fa	61705069	61765418	XPO1	36.25773106	11628
chr2.fa	62051983	62081278	FAM161A	1.240092868	304
chr2.fa	62095262	62115791	CCT4	95.98415187	14875
chr2.fa	62132803	62363205	COMMD1	33.71218216	1596
chr2.fa	62423262	62451866	B3GNT2	2.914534513	532
chr2.fa	62727356	62733604	TMEM17	1.189187914	152
chr2.fa	62900986	63273616	EHBP1	12.84762718	4383
chr2.fa	63271100	63275656	LOC100132215	0	0
chr2.fa	63277192	63284966	OTX1	1.55094442	304
chr2.fa	63344986	63346677	DBIL5P2	0	0
chr2.fa	63348535	63815742	C2orf86	2.796007299	608
chr2.fa	63815852	63834330	MDH1	61.94048509	7296
chr2.fa	64068098	64118696	UGP2	41.80140101	6764
chr2.fa	64119667	64246214	VPS54	25.34584758	6661
chr2.fa	64319786	64371605	PELI1	15.80026511	3952
chr2.fa	64412213	64432619	LOC150992	0	0
chr2.fa	64681327	64688517	HSPC159	25.72161048	6536
chr2.fa	64751439	64820138	AFTPH	59.61060451	16319
chr2.fa	64834446	64843616	LOC339807	0	0
chr2.fa	64858755	64881046	SERTAD2	7.220822512	2660
chr2.fa	65128974	65159581	LOC400958	0	0
chr2.fa	65215579	65251000	SLC1A4	8.384557949	2660
chr2.fa	65283495	65313987	CEP68	7.821892543	2964
chr2.fa	65314143	65357435	RAB1A	79.5889916	13148
chr2.fa	65454829	65498390	ACTR2	69.19398919	18088
chr2.fa	65537985	65659656	SPRED2	11.56912969	3572
chr2.fa	66662532	66799891	MEIS1	1.799143721	380
chr2.fa	67624442	67637533	ETAA1	3.486537515	760
chr2.fa	68269332	68290159	C1D	9.676760622	825
chr2.fa	68357281	68384656	WDR92	5.610840394	836
chr2.fa	68385005	68403094	PNO1	7.511342204	988
chr2.fa	68405989	68479651	PPP3R1	26.86501405	5371
chr2.fa	68511303	68547183	CNRIP1	0	0
chr2.fa	68592322	68624585	PLEK	0	0
chr2.fa	68689505	68694390	FBXO48	1.26614777	228
chr2.fa	68694691	68807294	APLF	4.46065953	1140
chr2.fa	68872954	68882708	PROKR1	0	0
chr2.fa	68961913	69053957	ARHGAP25	0.335550997	76

chr2.fa	69092613	69098649	BMP10	0	0
chr2.fa	69172364	69180102	GKN2	0	0
chr2.fa	69201705	69208112	GKN1	0	0
chr2.fa	69240276	69476459	ANTXR1	3.351292993	1585
chr2.fa	69330814	69330887	MIR3126	0	0
chr2.fa	69546905	69614382	GFPT1	34.25722662	19655
chr2.fa	69623253	69664753	NFU1	35.8568169	2807
chr2.fa	69685127	69870977	AAK1	10.7114264	15124
chr2.fa	69747177	69747303	SNORA36C	0	0
chr2.fa	69969127	70053596	ANXA4	38.17886594	5430
chr2.fa	70056818	70106727	GMCL1	8.294194126	1292
chr2.fa	70121090	70132349	SNRNP27	13.85925018	1292
chr2.fa	70142203	70170076	MXD1	8.810924589	3268
chr2.fa	70187224	70189394	ASPRV1	0	0
chr2.fa	70189398	70314147	LOC400960	1.441001769	228
chr2.fa	70314585	70316334	PCBP1	143.8936955	16720
chr2.fa	70351168	70352448	LOC100133985	3.463193527	152
chr2.fa	70377017	70418151	C2orf42	6.348811617	1064
chr2.fa	70436576	70475779	TIA1	5.903920394	1824
chr2.fa	70485231	70508317	PCYOX1	2.554434678	912
chr2.fa	70508506	70520869	SNRPG	15.29467952	588
chr2.fa	70523108	70529220	FAM136A	25.33726302	3040
chr2.fa	70674417	70781105	TGFA	3.761695357	1064
chr2.fa	70889216	70995375	ADD2	0	0
chr2.fa	71004442	71017775	FIGLA	0	0
chr2.fa	71035777	71047732	CLEC4F	0	0
chr2.fa	71057343	71062953	CD207	0	0
chr2.fa	71127720	71160575	VAX2	0	0
chr2.fa	71162998	71192561	ATP6V1B1	2.361206702	304
chr2.fa	71205575	71212629	ANKRD53	0	0
chr2.fa	71213068	71222001	TEX261	11.89820461	2660
chr2.fa	71251205	71257060	OR7E91P	0	0
chr2.fa	71295408	71305998	NAGK	27.32827925	3268
chr2.fa	71336806	71357394	MCEE	17.10331144	988
chr2.fa	71357444	71377232	MPHOSPH10	24.41223868	4028
chr2.fa	71409868	71454233	PAIP2B	17.98676842	7524
chr2.fa	71558889	71662189	ZNF638	24.00484844	10357
chr2.fa	71680753	71913893	DYSF	0.471849764	228
chr2.fa	72356367	72374963	CYP26B1	0	0
chr2.fa	72406444	73053177	EXOC6B	29.8890394	5148
chr2.fa	73114512	73119289	SPR	30.59613632	2964
chr2.fa	73144604	73162020	EMX1	7.32383727	1064
chr2.fa	73169165	73298965	SFXN5	3.976158831	1093

chr2.fa	73300510	73340146	RAB11FIP5	16.08039296	4636
chr2.fa	73429386	73438340	NOTO	0	0
chr2.fa	73441366	73454355	SMYD5	10.10297666	1722
chr2.fa	73455134	73460356	C2orf7	20.08079942	1444
chr2.fa	73461364	73480150	CCT7	128.6824519	17362
chr2.fa	73481810	73496758	FBXO41	3.139088614	1444
chr2.fa	73518057	73520829	EGR4	0	0
chr2.fa	73612886	73837046	ALMS1	8.14916019	6992
chr2.fa	73867850	73869537	NAT8	61.87075434	4408
chr2.fa	73872046	73912694	ALMS1P	0.718994821	76
chr2.fa	73927636	73928467	NAT8B	9.630072647	532
chr2.fa	73956957	73964517	TPRKB	16.74291039	836
chr2.fa	73989325	74007284	DUSP11	14.66544609	1596
chr2.fa	74011316	74044274	C2orf78	0	0
chr2.fa	74056086	74090011	STAMBP	19.06902581	3116
chr2.fa	74120093	74146780	ACTG2	0	0
chr2.fa	74153953	74186088	DGUOK	33.01758557	2508
chr2.fa	74273450	74335302	TET3	11.56912969	8436
chr2.fa	74362528	74375039	BOLA3	35.12336386	1292
chr2.fa	74383211	74405995	MOBKL1B	46.50785014	4104
chr2.fa	74425690	74442424	MTHFD2	9.517569687	1444
chr2.fa	74443369	74570534	SLC4A5	0.894752457	380
chr2.fa	74588281	74619120	DCTN1	34.33057192	11614
chr2.fa	74612845	74621008	LOC100189589	0	0
chr2.fa	74641303	74644844	C2orf81	0.513567729	76
chr2.fa	74648885	74652882	WDR54	3.068604832	228
chr2.fa	74652988	74669060	RTKN	9.240453962	1748
chr2.fa	74682199	74685087	INO80B	16.05478988	1292
chr2.fa	74685577	74688010	WBP1	52.07230377	4028
chr2.fa	74688184	74692537	MOGS	3.558376754	684
chr2.fa	74699109	74699927	MRPL53	46.45333063	1900
chr2.fa	74699959	74710357	CCDC142	2.676877658	760
chr2.fa	74710449	74721691	TTC31	7.96647466	1516
chr2.fa	74724644	74725445	LBX2	0	0
chr2.fa	74729744	74732169	LOC151534	3.222373938	380
chr2.fa	74732193	74734821	PCGF1	5.18507618	304
chr2.fa	74741596	74744275	TLX2	0	0
chr2.fa	74745258	74753408	DQX1	0	0
chr2.fa	74753775	74756531	AUP1	8.307598093	684
chr2.fa	74756627	74759841	HTRA2	6.979852316	672
chr2.fa	74759946	74781062	LOXL3	0.989182652	205
chr2.fa	74776147	74784678	DOK1	2.854442571	456
chr2.fa	74785010	74875164	C2orf65	0	0

chr2.fa	74881393	74909185	SEMA4F	0.803635602	228
chr2.fa	75059782	75120481	HK2	21.30101224	10032
chr2.fa	75185775	75196859	POLE4	35.42276933	1637
chr2.fa	75273590	75426645	TACR1	0	0
chr2.fa	75719444	75796848	FAM176A	2.178069354	304
chr2.fa	75873909	75889334	MRPL19	7.896894516	4104
chr2.fa	75889832	75938111	C2orf3	10.32105468	3040
chr2.fa	76974850	77749502	LRRTM4	0	0
chr2.fa	78182033	78182152	SNAR-H	0	0
chr2.fa	79252826	79255628	REG3G	0	0
chr2.fa	79312149	79315150	REG1B	0	0
chr2.fa	79347584	79350545	REG1A	0	0
chr2.fa	79362629	79365553	REG1P	0	0
chr2.fa	79384132	79386880	REG3A	0	0
chr2.fa	79740060	80875988	CTNNA2	0	0
chr2.fa	79876420	79876485	MIR4264	0	0
chr2.fa	80529003	80531487	LRRTM1	0	0
chr2.fa	83083927	83084893	LOC1720	0	0
chr2.fa	84517806	84519324	FUNDC2P2	0	0
chr2.fa	84650647	84686586	SUCLG1	87.95532617	8509
chr2.fa	84743579	85046713	DNAH6	0.35784074	304
chr2.fa	85048796	85108252	C2orf89	0	0
chr2.fa	85132763	85133799	TMSB10	467.8171277	14972
chr2.fa	85198231	85286595	KCMF1	9.547239142	4788
chr2.fa	85360583	85537511	TCF7L1	1.569017185	304
chr2.fa	85545146	85555374	TGOLN2	9.967280314	4180
chr2.fa	85569078	85581821	RETSAT	6.568245101	1444
chr2.fa	85581843	85618875	ELMOD3	6.453633652	1064
chr2.fa	85621871	85637676	CAPG	196.7804785	18841
chr2.fa	85661918	85664152	SH2D6	0	0
chr2.fa	85766288	85772403	MAT2A	123.4341211	23104
chr2.fa	85776193	85788657	GGCX	3.525092746	760
chr2.fa	85804614	85809156	VAMP8	306.8021985	15808
chr2.fa	85811531	85820511	VAMP5	13.76979	608
chr2.fa	85822837	85824831	RNF181	70.33904943	3344
chr2.fa	85825670	85829822	TMEM150A	11.00962702	1136
chr2.fa	85832376	85839179	C2orf68	16.10238149	4560
chr2.fa	85843283	85876406	USP39	18.65365343	2736
chr2.fa	85884440	85895864	SFTPB	0	0
chr2.fa	85921414	85925875	GNLY	0	0
chr2.fa	85980909	86018506	ATOH8	1.171416362	456
chr2.fa	86066271	86116157	ST3GAL5	30.85954686	5016
chr2.fa	86247339	86250991	LOC90784	3.446626826	836

chr2.fa	86253451	86333278	POLR1A	12.41704356	5472
chr2.fa	86333305	86369280	PTCD3	7.924003663	3543
chr2.fa	86362993	86363129	SNORD94	0	0
chr2.fa	86371055	86422893	IMMT	25.92583272	5166
chr2.fa	86426556	86440477	MRPL35	9.333829913	2052
chr2.fa	86441120	86565206	REEP1	2.925076959	760
chr2.fa	86668271	86719839	KDM3A	26.99769827	8816
chr2.fa	86790427	86790620	VPS24	36.25426711	467
chr2.fa	86830516	86851000	RNF103	1.665254657	548
chr2.fa	86915355	86948245	RNF103-VPS24	0	0
chr2.fa	86947414	87005164	RMND5A	20.73367804	8512
chr2.fa	87011728	87035519	CD8A	0	0
chr2.fa	87042460	87089047	CD8B	0	0
chr2.fa	87140935	88125286	RGPD2	0	0
chr2.fa	87144738	88285309	RGPD1	0	0
chr2.fa	87248970	88047609	PLGLB2	0	0
chr2.fa	87257798	87303536	LOC285074	3.120262818	456
chr2.fa	87754974	87821030	NCRNA00152	2.404581337	152
chr2.fa	88326724	88355248	KRCC1	15.0177144	1748
chr2.fa	88367299	88412902	SMYD1	0.258440535	76
chr2.fa	88422510	88427578	FABP1	56.17708044	1824
chr2.fa	88469835	88486146	THNSL2	1.08391406	152
chr2.fa	88747726	88752053	FOXI3	0	0
chr2.fa	88824169	88829103	C2orf51	0	0
chr2.fa	88856259	88927094	EIF2AK3	5.171371	1596
chr2.fa	88991176	89050452	RPIA	10.56051881	1286
chr2.fa	89065419	89106109	ANKRD36BP2	0	0
chr2.fa	91824709	91847975	LOC654342	0	0
chr2.fa	91963368	91970153	GGT8P	0	0
chr2.fa	92129159	92130496	ACTR3BP2	0	0
chr2.fa	95426673	95522820	ANKRD20A8P	0.311905797	76
chr2.fa	95534430	95613087	LOC442028	0	0
chr2.fa	95537232	95542568	TEKT4	0	0
chr2.fa	95691479	95719735	MAL	30.34944308	2128
chr2.fa	95752952	95787754	MRPS5	28.50737654	3144
chr2.fa	95813400	95825263	ZNF514	6.073201956	1292
chr2.fa	95831183	95850064	ZNF2	3.764255665	988
chr2.fa	95940201	95957055	PROM2	3.628258111	1140
chr2.fa	95963072	96051825	KCNIP3	0.379377451	76
chr2.fa	96068448	96078879	FAHD2A	6.172752768	532
chr2.fa	96142715	96150479	TRIM43B	0	0
chr2.fa	96257766	96265469	TRIM43	0	0
chr2.fa	96676299	96688853	LOC729234	6.280586931	294

chr2.fa	96687694	96700727	GPAT2	0	0
chr2.fa	96778623	96781888	ADRA2B	0	0
chr2.fa	96789589	96804175	ASTL	0	0
chr2.fa	96808908	96811179	DUSP2	14.94436909	1672
chr2.fa	96850603	96874573	STARD7	71.73306201	16094
chr2.fa	96905977	96907701	LOC285033	0	0
chr2.fa	96915946	96931751	TMEM127	7.226997373	2204
chr2.fa	96931884	96939900	CIAO1	26.65838211	7144
chr2.fa	96940074	96971297	SNRNP200	32.2312697	15428
chr2.fa	96991062	96994091	ITPRIPL1	0	0
chr2.fa	97001484	97041274	NCAPH	0.509350751	152
chr2.fa	97163383	97173846	NEURL3	2.050053937	228
chr2.fa	97202464	97218371	ARID5A	0	0
chr2.fa	97258907	97304115	KIAA1310	16.02662648	5624
chr2.fa	97308574	97370624	FER1L5	0	0
chr2.fa	97371667	97405813	LMAN2L	2.812272787	456
chr2.fa	97426639	97477628	CNNM4	4.292281606	1368
chr2.fa	97464015	97464090	MIR3127	0	0
chr2.fa	97481991	97501121	CNNM3	1.164187257	380
chr2.fa	97503651	97509758	ANKRD23	0.886619713	152
chr2.fa	97513724	97523756	ANKRD39	1.274581727	76
chr2.fa	97525473	97535735	SEMA4C	1.933484605	456
chr2.fa	97541619	97652301	FAM178B	0	0
chr2.fa	97749323	97760582	FAHD2B	4.524215419	380
chr2.fa	97779233	97930257	ANKRD36	0.655890751	273
chr2.fa	98121261	98206428	ANKRD36B	0	0
chr2.fa	98262521	98264657	COX5B	138.2596617	4636
chr2.fa	98272402	98280561	ACTR1B	32.17554534	4824
chr2.fa	98330031	98356323	ZAP70	0	0
chr2.fa	98372801	98612354	TMEM131	6.376221977	2812
chr2.fa	98703595	98929410	VWA3B	0	0
chr2.fa	98962618	99015064	CNGA3	0	0
chr2.fa	99061321	99207496	INPP4A	43.28653045	19777
chr2.fa	99215865	99224955	C2orf64	12.39641049	1368
chr2.fa	99225042	99234977	UNC50	8.018283252	608
chr2.fa	99235569	99347589	MGAT4A	16.50615717	4853
chr2.fa	99410309	99552684	C2orf55	10.94607113	2812
chr2.fa	99613724	99771187	TSGA10	6.209801936	1580
chr2.fa	99758185	99767928	C2orf15	5.690511165	532
chr2.fa	99771418	99779613	LIPT1	2.65564216	304
chr2.fa	99785726	99797492	MITD1	3.773442654	228
chr2.fa	99797578	99814089	MRPL30	26.28728801	4484
chr2.fa	99858711	99871570	LYG2	0	0

chr2.fa	99900701	99917639	LYG1	0	0
chr2.fa	99935487	99952860	TXNDC9	13.46601694	1368
chr2.fa	99953834	100016728	EIF5B	49.94559119	15580
chr2.fa	100016938	100106480	REV1	17.65031378	5548
chr2.fa	100163716	100759037	AFF3	8.561068617	4668
chr2.fa	100889753	100939195	LONRF2	1.563294143	1444
chr2.fa	101008322	101034091	CHST10	0.813274409	152
chr2.fa	101086944	101099742	NMS	0	0
chr2.fa	101179418	101193201	PDCL3	7.453208144	532
chr2.fa	101436613	101613287	NPAS2	18.58136237	4940
chr2.fa	101618691	101636155	RPL31	701.227335	56012
chr2.fa	101623690	101767846	TBC1D8	26.77284295	7372
chr2.fa	101869345	101886778	C2orf29	23.18314007	3876
chr2.fa	101889398	101889511	SNORD89	10.04047501	76
chr2.fa	101892063	101925178	RNF149	2.313916301	456
chr2.fa	101964816	102003965	CREG2	0	0
chr2.fa	102013823	102091165	RFX8	0	0
chr2.fa	102314488	102511152	MAP4K4	19.15231113	9516
chr2.fa	102608306	102644884	IL1R2	0	0
chr2.fa	102770402	102796334	IL1R1	43.84061129	14284
chr2.fa	102803433	102855811	IL1RL2	0	0
chr2.fa	102927962	102968497	IL1RL1	0	0
chr2.fa	102979097	103015217	IL18R1	0.650167708	152
chr2.fa	103035254	103069024	IL18RAP	0	0
chr2.fa	103089762	103150431	SLC9A4	22.12874486	6080
chr2.fa	103236166	103327809	SLC9A2	13.25064983	4788
chr2.fa	103333666	103353337	MFSD9	5.07588656	912
chr2.fa	103378490	103434138	TMEM182	3.509128471	836
chr2.fa	105050805	105129215	LOC150568	0	0
chr2.fa	105471969	105473471	POU3F3	2.284698665	228
chr2.fa	105654483	105716418	MRPS9	15.48414233	1499
chr2.fa	105858200	105859924	GPR45	0	0
chr2.fa	105883540	105946148	TGFBRAP1	13.05561458	2572
chr2.fa	105954013	105961984	C2orf49	9.032918381	532
chr2.fa	105977283	106055230	FHL2	7.62730911	1140
chr2.fa	106361520	106510730	NCK2	22.74623099	4028
chr2.fa	106682113	106694609	C2orf40	0	0
chr2.fa	106709759	106810795	UXS1	9.662151804	1337
chr2.fa	107002769	107007851	PLGLA	0	0
chr2.fa	107021136	107084801	RGPD3	0.194884646	76
chr2.fa	107418056	107503563	ST6GAL2	0.16190185	76
chr2.fa	108439520	108442583	LOC729121	0	0
chr2.fa	108443388	108509000	RGPD4	0.79791256	380

chr2.fa	108602995	108630443	SLC5A7	0.444891223	152
chr2.fa	108863651	108881807	SULT1C3	0	0
chr2.fa	108905095	108926371	SULT1C2	3.960646374	760
chr2.fa	108938694	108970254	SULT1C2P1	1.221568284	152
chr2.fa	108994421	109004270	SULT1C4	0.687819302	76
chr2.fa	109065577	109125854	GCC2	24.28000628	11248
chr2.fa	109150857	109303702	LIMS1	5.662197167	2052
chr2.fa	109335937	109402267	RANBP2	22.38191417	17404
chr2.fa	109403219	109492847	CCDC138	4.691087279	684
chr2.fa	109510927	109605828	EDAR	0.271693895	76
chr2.fa	109743784	109746575	LOC100287216	0	0
chr2.fa	109757946	109758044	MIR4265	0	0
chr2.fa	109930027	109930081	MIR4266	0	0
chr2.fa	109964130	110262207	SH3RF3	0	0
chr2.fa	110300376	110371783	10-Sep	18.64296038	4028
chr2.fa	110371911	110376564	ANKRD57	7.624146376	2356
chr2.fa	110663457	111223204	LIMS3	0	0
chr2.fa	110690665	111195997	LIMS3-LOC440895	0	0
chr2.fa	110827538	110827619	MIR4267	0	0
chr2.fa	110841447	110874143	MALL	1.869627504	380
chr2.fa	110880914	110962639	NPHP1	2.497354863	456
chr2.fa	110969106	110980517	NCRNA00116	7.776861238	836
chr2.fa	111003215	111024135	LOC100507334	0	0
chr2.fa	111132686	111142113	LOC151009	0	0
chr2.fa	111271379	111271397	RGPD6	0	0
chr2.fa	111395409	111435684	BUB1	2.614827833	608
chr2.fa	111490150	111875799	ACOXL	8.17446206	1288
chr2.fa	111878491	111926022	BCL2L11	8.177323581	2888
chr2.fa	112124591	112252692	LOC541471	0	0
chr2.fa	112526640	112641741	ANAPC1	7.243865287	3035
chr2.fa	112656191	112786945	MERTK	2.841038604	684
chr2.fa	112812800	112876895	TMEM87B	5.224384443	1808
chr2.fa	112895962	112945791	FBLN7	0	0
chr2.fa	112973439	113012664	ZC3H8	2.854442571	304
chr2.fa	113033178	113097640	ZC3H6	5.356466231	4104
chr2.fa	113125946	113125964	RGPD8	0	0
chr2.fa	113239743	113290222	TTL	8.424318031	2888
chr2.fa	113299492	113334727	POLR1B	12.06191374	4332
chr2.fa	113342036	113346617	CHCHD5	17.00752579	760
chr2.fa	113399407	113401757	FLJ42351	0	0
chr2.fa	113403527	113421400	SLC20A1	21.26095095	4636
chr2.fa	113495444	113522254	CKAP2L	0	0
chr2.fa	113531492	113542971	IL1A	1.563745962	304

chr2.fa	113587337	113594356	IL1B	1.528202858	152
chr2.fa	113670548	113676458	IL1F7	0	0
chr2.fa	113735606	113743227	IL1F9	0	0
chr2.fa	113763449	113765621	IL1F6	0	0
chr2.fa	113779668	113810440	IL1F8	0	0
chr2.fa	113816215	113822320	IL1F5	0	0
chr2.fa	113825547	113833427	IL1F10	0	0
chr2.fa	113875470	113891593	IL1RN	91.54698694	12844
chr2.fa	113917421	114205429	LOC440839	0	0
chr2.fa	113931560	113960677	PSD4	27.59274404	9232
chr2.fa	113973574	114036498	PAX8	18.76675882	4927
chr2.fa	113993846	114024580	LOC654433	0	0
chr2.fa	114195268	114253781	CBWD2	5.432371843	659
chr2.fa	114256661	114258727	FOXD4L1	0	0
chr2.fa	114334959	114336429	FAM138B	0	0
chr2.fa	114341230	114356604	WASH2P	1.174579096	152
chr2.fa	114356614	114361294	DDX11L2	0	0
chr2.fa	114368816	114384715	RPL23AP7	0	0
chr2.fa	114384817	114400975	RABL2A	1.582421152	228
chr2.fa	114471933	114514400	SLC35F5	5.224685656	988
chr2.fa	114647537	114716167	ACTR3	48.79164517	8786
chr2.fa	114735246	114737145	LOC100499194	0	0
chr2.fa	114737562	114764887	LOC440900	0	0
chr2.fa	115199899	116602326	DPP10	0.736314553	304
chr2.fa	115901625	115918920	LOC389023	4.703888821	228
chr2.fa	118572255	118589953	DDX18	21.88897952	5472
chr2.fa	118673054	118771739	CCDC93	12.2524308	5637
chr2.fa	118846050	118867597	INSIG2	5.760693734	988
chr2.fa	119599747	119605759	EN1	0	0
chr2.fa	119699745	119752236	MARCO	0	0
chr2.fa	119913819	119916471	C1QL2	0	0
chr2.fa	119981384	120023227	STEAP3	1.841012293	532
chr2.fa	120060020	120124258	C2orf76	5.675450527	358
chr2.fa	120124504	120130122	DBI	246.4311825	20126
chr2.fa	120189446	120196096	TMEM37	8.141780477	912
chr2.fa	120197419	120282028	SCTR	0	0
chr2.fa	120302025	120414237	PCDP1	0.385702919	76
chr2.fa	120436743	120439694	TMEM177	1.70079776	152
chr2.fa	120517207	120735037	PTPN4	8.945416079	2342
chr2.fa	120770604	120936697	EPB41L5	14.16769203	9346
chr2.fa	120978854	120980984	TMEM185B	2.148550505	304
chr2.fa	121010414	121052286	RALB	80.99836603	12160
chr2.fa	121103719	121109383	INHBB	1.429405078	304

chr2.fa	121221911	121223925	LOC84931	0	0
chr2.fa	121554867	121750229	GLI2	0.169130956	76
chr2.fa	121974164	122042778	TFCP2L1	15.97045031	9847
chr2.fa	122095352	122407052	CLASP1	12.65530284	6840
chr2.fa	122288456	122288585	RNU4ATAC	0	0
chr2.fa	122484521	122494503	MKI67IP	12.51297982	1444
chr2.fa	122513121	122525428	TSN	13.43830537	3040
chr2.fa	124782864	125672863	CNTNAP5	1.733328737	608
chr2.fa	127413684	127454246	GYPC	1.068702817	76
chr2.fa	127805607	127864864	BIN1	9.441814681	1672
chr2.fa	127941412	127963343	CYP27C1	0	0
chr2.fa	128014866	128051752	ERCC3	17.89745884	3268
chr2.fa	128062139	128100770	MAP3K2	13.57008595	4452
chr2.fa	128175996	128186822	PROC	1.68377924	199
chr2.fa	128238383	128284087	IWS1	26.70747979	5320
chr2.fa	128293378	128395303	MYO7B	0.85228146	380
chr2.fa	128395996	128439360	LIMS2	0	0
chr2.fa	128403439	128410213	GPR17	0	0
chr2.fa	128458597	128461407	SFT2D3	4.479033507	836
chr2.fa	128461808	128568761	WDR33	8.060603643	6004
chr2.fa	128603840	128615729	POLR2D	18.70757051	2888
chr2.fa	128619207	128643514	AMMECR1L	11.50361592	3648
chr2.fa	128698791	128784869	SAP130	18.92594975	5244
chr2.fa	128848754	128953249	UGGT1	1.148825407	836
chr2.fa	129023054	129076171	HS6ST1	3.765159303	988
chr2.fa	130680435	130691890	LOC389033	0	0
chr2.fa	130724165	130736664	LOC100131320	1.070660699	152
chr2.fa	130737235	130740311	RAB6C	0	0
chr2.fa	130783572	130808704	LOC440905	0	0
chr2.fa	130831108	130886795	POTEF	0	0
chr2.fa	130887196	130896861	LOC285103	0	0
chr2.fa	130896987	130902631	CCDC74B	4.889737084	437
chr2.fa	130908965	130939330	SMPD4	7.007865102	1824
chr2.fa	130939501	130948300	MZT2B	28.21655564	1748
chr2.fa	130949318	130956034	TUBA3E	0	0
chr2.fa	131095816	131099922	CCDC115	22.27950184	2432
chr2.fa	131100489	131104197	IMP4	27.27857915	1900
chr2.fa	131113580	131132982	PTPN18	30.8273171	7510
chr2.fa	131278667	131357251	CFC1B	0	0
chr2.fa	131307469	131328418	LOC646743	0	0
chr2.fa	131328422	131339833	LOC150527	0	0
chr2.fa	131350335	131350352	CFC1	0	0
chr2.fa	131437623	131443435	C2orf14	0	0

chr2.fa	131486643	131487909	GPR148	0	0
chr2.fa	131513077	131525707	FAM123C	0	0
chr2.fa	131674224	131804836	ARHGEF4	0.806647729	228
chr2.fa	131805449	131851004	FAM168B	43.19270268	15504
chr2.fa	131862420	131907425	PLEKHB2	64.49010036	18447
chr2.fa	131975924	132022416	POTEE	0.332689476	76
chr2.fa	132036863	132057392	LOC440910	0	0
chr2.fa	132118065	132121731	LOC150786	0.312207009	76
chr2.fa	132160474	132165801	LOC389043	0	0
chr2.fa	132199734	132202467	LOC401010	0.418685715	76
chr2.fa	132233666	132240507	TUBA3D	2.221142776	228
chr2.fa	132241533	132250064	MZT2A	13.21329945	608
chr2.fa	132250386	132279149	LOC150776	1.510883125	380
chr2.fa	132285492	132291239	CCDC74A	5.499090466	532
chr2.fa	132349268	132384999	POTEKP	0	0
chr2.fa	132480064	132524977	C2orf27A	0.93903073	76
chr2.fa	132552534	132559234	C2orf27B	0	0
chr2.fa	132905164	133015542	ANKRD30BL	0	0
chr2.fa	133014539	133014653	MIR663B	0	0
chr2.fa	133174147	133404169	GPR39	0.697458109	76
chr2.fa	133402337	133429070	LYPD1	0	0
chr2.fa	133429372	134326031	NCKAP5	7.385585883	3724
chr2.fa	134884696	134884763	MIR3679	0	0
chr2.fa	135011830	135206468	MGAT5	11.34683468	1824
chr2.fa	135206960	135212192	LOC151162	3.280959817	1140
chr2.fa	135213330	135476571	TMEM163	1.209369168	152
chr2.fa	135596186	135659602	ACMSD	6.905000949	530
chr2.fa	135624203	135676176	LOC100129961	3.492260557	304
chr2.fa	135676392	135716915	CCNT2	11.72410364	5457
chr2.fa	135722273	135782248	YSK4	0	0
chr2.fa	135809835	135928279	RAB3GAP1	25.46091085	8348
chr2.fa	135957574	136288806	ZRANB3	0.929391922	228
chr2.fa	136289083	136482839	R3HDM1	8.620859347	2660
chr2.fa	136422967	136423048	MIR128-1	0	0
chr2.fa	136499189	136542633	UBXN4	29.65605134	7904
chr2.fa	136545415	136594750	LCT	0	0
chr2.fa	136597196	136634011	MCM6	2.136050176	532
chr2.fa	136664254	136743222	DARS	37.25595009	5744
chr2.fa	136871919	136875725	CXCR4	0	0
chr2.fa	137748462	138435287	THSD7B	0	0
chr2.fa	138721808	138773934	HNMT	13.8999139	3856
chr2.fa	139259350	139330805	SPOPL	12.57593329	4788
chr2.fa	139426727	139537811	NXPH2	0	0

chr2.fa	139654894	139656744	LOC647012	0	0
chr2.fa	140988996	142889270	LRP1B	0	0
chr2.fa	143635195	143799885	KYNU	7.814512831	1045
chr2.fa	143886899	144525921	ARHGAP15	0.650770134	76
chr2.fa	144703581	145090083	GTDC1	8.780050282	1672
chr2.fa	145141942	145277958	ZEB2	0.589172128	456
chr2.fa	145425534	145834291	DKFZp686O1327	0	0
chr2.fa	147344625	147348558	PABPC1P2	0	0
chr2.fa	148602570	148687965	ACVR2A	6.892350014	2204
chr2.fa	148688394	148779173	ORC4	5.478306786	2356
chr2.fa	148778580	149271044	MBD5	4.566234597	1824
chr2.fa	149402560	149545136	EPC2	24.20620916	5852
chr2.fa	149632819	149883273	KIF5C	16.49907867	7593
chr2.fa	149894981	150071772	LYPD6B	10.18716562	1064
chr2.fa	150186499	150330659	LYPD6	2.922667258	836
chr2.fa	150426147	150444330	MMADHC	66.00685714	6298
chr2.fa	151324710	151344180	RND3	12.37637984	2204
chr2.fa	152104728	152118389	RBM43	2.924323928	760
chr2.fa	152126982	152146430	NMI	5.417311205	532
chr2.fa	152214106	152236562	TNFAIP6	0	0
chr2.fa	152266397	152333860	RIF1	8.7710139	5928
chr2.fa	152341853	152591001	NEB	0.435101809	760
chr2.fa	152657480	152685009	ARL5A	18.05951129	3800
chr2.fa	152689286	152955593	CACNB4	1.095058932	608
chr2.fa	152973315	153032506	STAM2	9.819384856	3717
chr2.fa	153191751	153506348	FMNL2	3.692868245	1367
chr2.fa	153508107	153573975	PRPF40A	18.55169292	9273
chr2.fa	153574423	153617767	ARL6IP6	0.814629867	152
chr2.fa	154333852	154335322	RPRM	1.556215643	152
chr2.fa	154728426	155310489	GALNT13	0	0
chr2.fa	155555093	155713014	KCNJ3	0.396094759	76
chr2.fa	157180944	157189287	NR4A2	3.241651554	760
chr2.fa	157291965	157442915	GPD2	15.62104352	6384
chr2.fa	158114340	158167913	GALNT5	0	0
chr2.fa	158175125	158184146	ERMN	0	0
chr2.fa	158271131	158300604	CYTIP	1.035870627	152
chr2.fa	158383279	158485399	ACVR1C	0	0
chr2.fa	158592958	158732374	ACVR1	4.045889581	836
chr2.fa	158851691	158992666	UPP2	2.219034287	380
chr2.fa	159027869	159313265	CCDC148	6.656801648	1368
chr2.fa	159313476	159537940	PKP4	36.90669392	11248
chr2.fa	159651829	159672496	DAPL1	4.215924175	152
chr2.fa	159825146	160089170	TANC1	23.07244439	11476

chr2.fa	160092304	160143236	WDSUB1		10.39123725	1422
chr2.fa	160175490	160473059	BAZ2B		25.5023276	13792
chr2.fa	160569010	160625084		7-Mar	27.06396507	6232
chr2.fa	160654643	160654766	CD302		0	0
chr2.fa	160659868	160661733	LY75		0	0
chr2.fa	160797260	160919126	PLA2R1		4.150259797	1824
chr2.fa	160958233	161056590	ITGB6		6.207693446	988
chr2.fa	161128662	161350318	RBMS1		9.347083273	2660
chr2.fa	161993466	162092682	TANK		17.58480001	2508
chr2.fa	162164786	162268226	PSMD14		26.67886458	3038
chr2.fa	162272620	162281573	TBR1		0	0
chr2.fa	162480845	162841786	SLC4A10		0	0
chr2.fa	162848755	162931052	DPP4		39.66745932	10272
chr2.fa	162999385	163008914	GCG		0	0
chr2.fa	163027200	163100045	FAP		0.823515643	152
chr2.fa	163123589	163175039	IFIH1		10.70660699	2432
chr2.fa	163200583	163219148	GCA		7.033016366	1506
chr2.fa	163227917	163695240	KCNH7		0	0
chr2.fa	164464118	164592513	FIGN		4.795457495	1444
chr2.fa	165349323	165478360	GRB14		1.438592067	228
chr2.fa	165541258	165697928	COBLL1		62.10329058	20292
chr2.fa	165544153	165544287	SNORA70F		0	0
chr2.fa	165754709	165812035	SLC38A11		0.639474656	76
chr2.fa	165944030	166060577	SCN3A		0.496699815	304
chr2.fa	166095912	166248820	SCN2A		1.245364091	760
chr2.fa	166326157	166545917	CSRNP3		0.38028109	304
chr2.fa	166604313	166650803	GALNT3		7.00711207	1520
chr2.fa	166730453	166810348	TTC21B		7.247329233	2356
chr2.fa	166845670	166930149	SCN1A		0	0
chr2.fa	167051697	167232497	SCN9A		0.469138849	304
chr2.fa	167261539	167343478	SCN7A		0	0
chr2.fa	167744997	168116261	XIRP2		0	0
chr2.fa	168675182	168727366	B3GALT1		0.528025941	76
chr2.fa	168810530	169104105	STK39		51.35059803	11163
chr2.fa	169312835	169631152	LASS6		2.925980598	1216
chr2.fa	169643049	169721849	NOSTRIN		7.714660806	1444
chr2.fa	169727401	169746944	SPC25		0	0
chr2.fa	169757750	169766510	G6PC2		0	0
chr2.fa	169779449	169887833	ABCB11		0	0
chr2.fa	169921299	169952677	DHRS9		0	0
chr2.fa	169983619	170219122	LRP2		3.782629642	3952
chr2.fa	170336006	170363165	BBS5		2.533199179	531
chr2.fa	170366212	170382772	KBTBD10		0.464470052	76

chr2.fa	170386263	170430424	FASTKD1	3.048272972	608
chr2.fa	170440850	170494254	PPIG	23.81538562	4279
chr2.fa	170501935	170550931	C2orf77	1.583174184	228
chr2.fa	170554375	170558218	PHOSPHO2	4.803740845	281
chr2.fa	170590356	170608396	KLHL23	6.741291823	1824
chr2.fa	170655389	170668575	SSB	43.05278936	4408
chr2.fa	170668967	170681353	METTL5	13.44116689	747
chr2.fa	170684018	170940639	UBR3	11.38689598	6013
chr2.fa	171034655	171511674	MYO3B	8.854751043	3724
chr2.fa	171568949	171571077	LOC440925	0	0
chr2.fa	171571857	171574498	SP5	0	0
chr2.fa	171673200	171717659	GAD1	0.311905797	76
chr2.fa	171785711	171823640	GORASP2	27.48159654	4560
chr2.fa	171847333	172087824	TLK1	13.79720036	5526
chr2.fa	172173913	172291312	METTL8	2.384400084	1283
chr2.fa	172290761	172341562	DCAF17	3.158667443	1216
chr2.fa	172378866	172414643	CYBRD1	2.101711923	608
chr2.fa	172543982	172604919	DYNC1I2	28.15330096	4864
chr2.fa	172639915	172750813	SLC25A12	4.011099509	1064
chr2.fa	172778935	172848600	HAT1	19.19101697	2128
chr2.fa	172864804	172945587	METAP1D	1.492358541	152
chr2.fa	172950208	172954401	DLX1	0	0
chr2.fa	172964166	172967478	DLX2	0	0
chr2.fa	173292314	173371181	ITGA6	5.319115851	2052
chr2.fa	173420779	173463862	PDK1	3.742116528	1137
chr2.fa	173587918	173600934	LOC91149	0	0
chr2.fa	173600525	173917620	RAPGEF4	0	0
chr2.fa	173940565	174132737	ZAK	2.088307956	1340
chr2.fa	174062441	174146764	LOC339751	0	0
chr2.fa	174219561	174233718	CDCA7	0.407390237	76
chr2.fa	174771187	174830430	SP3	14.21980184	6004
chr2.fa	174937175	175113365	OLA1	12.35002373	3572
chr2.fa	175199821	175202268	SP9	0	0
chr2.fa	175212878	175260443	CIR1	17.55528116	2090
chr2.fa	175260457	175294303	SCRN3	8.622967836	1748
chr2.fa	175296375	175351811	GPR155	0.612666722	304
chr2.fa	175424302	175547627	WIPF1	2.186653917	684
chr2.fa	175612323	175629200	CHRNA1	0	0
chr2.fa	175664042	175870170	CHN1	0	0
chr2.fa	175939006	176032897	ATF2	28.73720187	4028
chr2.fa	176032361	176032437	MIR933	0	0
chr2.fa	176040986	176046490	ATP5G3	39.46926134	6916
chr2.fa	176790410	176867018	KIAA1715	2.581092006	988

chr2.fa	176944835	176948690	EVX2	0	0
chr2.fa	176957532	176960666	HOXD13	5.902564936	912
chr2.fa	176964530	176965488	HOXD12	0	0
chr2.fa	176972084	176974316	HOXD11	12.51794983	1216
chr2.fa	176981492	176984670	HOXD10	10.15177312	1216
chr2.fa	176987413	176989645	HOXD9	3.643318748	456
chr2.fa	176994422	176997423	HOXD8	8.707608617	1520
chr2.fa	177015031	177015140	MIR10B	0	0
chr2.fa	177016113	177017949	HOXD4	2.649617905	228
chr2.fa	177028805	177037826	HOXD3	0.995809333	152
chr2.fa	177037924	177042222	LOC401022	6.880150898	1672
chr2.fa	177053307	177055635	HOXD1	5.003746108	608
chr2.fa	177134123	177202753	MTX2	29.52532501	3121
chr2.fa	177465708	177465780	MIR1246	0	0
chr2.fa	178077422	178088685	HNRNPA3	16.16006373	6232
chr2.fa	178095033	178129859	NFE2L2	41.80908193	9272
chr2.fa	178120673	178120738	MIR3128	0	0
chr2.fa	178148236	178257419	LOC100130691	2.638322427	912
chr2.fa	178257471	178408564	AGPS	5.456468862	2777
chr2.fa	178414881	178417524	TTC30B	2.5975081	456
chr2.fa	178479026	178483694	TTC30A	4.902990445	1520
chr2.fa	178487977	178973066	PDE11A	11.69157267	7600
chr2.fa	178977182	178994382	RBM45	6.415982059	760
chr2.fa	179059374	179260875	OSBPL6	3.14300438	836
chr2.fa	179246805	179541009	MIR548N	0	0
chr2.fa	179296141	179315958	PRKRA	18.74431847	2356
chr2.fa	179316163	179326110	DFNB59	0.751073978	76
chr2.fa	179328391	179343355	FKBP7	0	0
chr2.fa	179345199	179369782	PLEKHA3	10.00990192	1641
chr2.fa	179390718	179672150	TTN	0.29639334	2204
chr2.fa	179694484	179914786	CCDC141	0.25075961	152
chr2.fa	179966419	180129350	SESTD1	4.163061338	2888
chr2.fa	180306711	180726232	ZNF385B	13.83394831	3496
chr2.fa	180725563	180725635	MIR1258	0	0
chr2.fa	180809604	180871780	CWC22	14.93668817	3252
chr2.fa	181845112	181928150	UBE2E3	26.13743467	2959
chr2.fa	182321619	182401400	ITGA4	0	0
chr2.fa	182402469	182521834	CERKL	1.546727442	228
chr2.fa	182541194	182545381	NEUROD1	0	0
chr2.fa	182756472	182795464	SSFA2	18.64371341	6551
chr2.fa	182850551	182982419	PPP1R1C	0	0
chr2.fa	183007183	183387465	PDE1A	4.231436631	760
chr2.fa	183580999	183643255	DNAJC10	9.072979676	2526

chr2.fa	183698005	183731498	FRZB	0	0
chr2.fa	183789605	183903229	NCKAP1	55.42721132	17047
chr2.fa	183943287	183964722	DUSP19	0.426667852	152
chr2.fa	183989083	184026408	NUP35	4.205532335	456
chr2.fa	185463093	185804214	ZNF804A	0	0
chr2.fa	186603355	186698016	FSIP2	0.486458582	680
chr2.fa	187350885	187374087	ZC3H15	37.02672719	5244
chr2.fa	187454790	187545628	ITGAV	5.143508821	2462
chr2.fa	187558789	187628512	FAM171B	0.321996424	76
chr2.fa	187692207	187713897	ZSWIM2	0	0
chr2.fa	188207849	188313021	CALCRL	0	0
chr2.fa	188328958	188419219	TFPI	1.382114677	380
chr2.fa	189157390	189460652	GULP1	10.94848083	2508
chr2.fa	189162219	189162315	MIR561	0	0
chr2.fa	189598465	189654831	DIRC1	0	0
chr2.fa	189839099	189877472	COL3A1	0	0
chr2.fa	189842818	189842886	MIR1245	0	0
chr2.fa	189860356	189860418	MIR3606	0	0
chr2.fa	189896641	190044605	COL5A2	0.330279774	152
chr2.fa	189997762	189997837	MIR3129	0	0
chr2.fa	190306159	190340264	WDR75	14.48050147	2571
chr2.fa	190425316	190445537	SLC40A1	19.44644538	4332
chr2.fa	190526125	190535557	ASNSD1	16.16382889	2652
chr2.fa	190540711	190611376	ANKAR	2.332742098	684
chr2.fa	190611386	190627924	OSGEPL1	1.976859241	304
chr2.fa	190634993	190647849	ORMDL1	6.171999736	852
chr2.fa	190648811	190742355	PMS1	5.692920866	1292
chr2.fa	190920426	190927455	MSTN	0	0
chr2.fa	191002486	191068210	C2orf88	0.255880226	76
chr2.fa	191069360	191184771	HIBCH	27.06878448	3485
chr2.fa	191208196	191236391	INPP1	4.233394514	608
chr2.fa	191273081	191367041	MFSD6	5.965217187	1900
chr2.fa	191371619	191399468	TMEM194B	0.314767318	76
chr2.fa	191513848	191557492	NAB1	9.732635586	2888
chr2.fa	191745547	191829776	GLS	22.49727866	6480
chr2.fa	191833762	191878976	STAT1	38.37947363	11679
chr2.fa	191894306	192015925	STAT4	0	0
chr2.fa	192110107	192290115	MYO1B	13.88711236	4698
chr2.fa	192542798	192553248	OBFC2A	1.462990299	380
chr2.fa	192699032	192712006	SDPR	1.0565037	228
chr2.fa	192814747	193059644	TMEFF2	4.429182798	532
chr2.fa	193614571	193641625	PCGEM1	0	0
chr2.fa	196521532	196602426	SLC39A10	9.115450673	3344

chr2.fa	196602427	196933536	DNAH7	1.108161686	912
chr2.fa	196998307	197036336	STK17B	10.35253141	3648
chr2.fa	197063977	197457335	HECW2	0.16521519	76
chr2.fa	197504356	197597530	CCDC150	0.311303371	76
chr2.fa	197565359	197577736	LOC100130452	0	0
chr2.fa	197629103	197664425	GTF3C3	14.81951641	2888
chr2.fa	197670009	197675000	C2orf66	0.825774738	76
chr2.fa	197697728	197791454	PGAP1	1.133011738	836
chr2.fa	197851386	198175521	ANKRD44	1.597331183	684
chr2.fa	198256698	198299771	SF3B1	78.50357147	23180
chr2.fa	198318231	198339851	COQ10B	11.59684126	1520
chr2.fa	198351308	198364640	HSPD1	101.5895704	15656
chr2.fa	198364721	198368187	HSPE1	61.46471956	3420
chr2.fa	198380320	198417515	MOBKL3	11.43840336	2242
chr2.fa	198435527	198540584	RFTN2	0	0
chr2.fa	198570028	198573114	MARS2	1.853964441	380
chr2.fa	198591603	198650938	BOLL	0	0
chr2.fa	198669426	199014608	PLCL1	3.464247772	1520
chr2.fa	200134223	200329831	SATB2	2.069482159	836
chr2.fa	200332821	200337481	FLJ32063	0	0
chr2.fa	200625259	200715896	LOC348751	1.202290669	76
chr2.fa	200775979	200792996	C2orf69	3.991370074	988
chr2.fa	200793634	200820219	C2orf60	1.561788079	532
chr2.fa	200820040	200828847	C2orf47	12.60786184	1064
chr2.fa	201170604	201346986	SPATS2L	22.9001507	10478
chr2.fa	201353684	201374792	KCTD18	8.96363945	1748
chr2.fa	201390865	201448818	SGOL2	1.223977986	380
chr2.fa	201450731	201536217	AOX1	49.48051871	16207
chr2.fa	201560446	201658941	AOX2P	0	0
chr2.fa	201676647	201688560	BZW1	63.73586365	11841
chr2.fa	201717732	201729467	CLK1	16.25599999	2584
chr2.fa	201735679	201753849	PPIL3	12.59792182	1064
chr2.fa	201754050	201768655	NIF3L1	11.84082358	1368
chr2.fa	201774894	201828424	ORC2	10.95314963	2280
chr2.fa	201838441	201936392	FAM126B	5.273632726	3268
chr2.fa	201936462	201950473	NDUFB3	54.57733956	2736
chr2.fa	201980816	202028841	CFLAR	31.24253887	5350
chr2.fa	202047621	202094110	CASP10	5.038385573	2128
chr2.fa	202098166	202152434	CASP8	7.837555606	1824
chr2.fa	202153147	202222101	ALS2CR12	0	0
chr2.fa	202241930	202316319	TRAK2	20.86847074	9044
chr2.fa	202316392	202345574	STRADB	13.448396	2052
chr2.fa	202352144	202483905	ALS2CR11	0	0

chr2.fa	202484907	202508224	ALS2CR4	1.044756403	380
chr2.fa	202509597	202563417	MPP4	0	0
chr2.fa	202564986	202645895	ALS2	7.410285328	4104
chr2.fa	202671198	202758263	CDK15	0	0
chr2.fa	202899310	202903160	FZD7	0.594443351	152
chr2.fa	203070903	203103322	SUMO1	38.97813396	3952
chr2.fa	203130515	203168384	NOP58	16.76218801	2187
chr2.fa	203141154	203141241	SNORD70	0	0
chr2.fa	203156040	203156151	SNORD11B	0	0
chr2.fa	203157774	203157857	SNORD11	0	0
chr2.fa	203241050	203432474	BMPR2	6.258899613	5016
chr2.fa	203499901	203634480	FAM117B	15.26666673	5852
chr2.fa	203637873	203736371	ICA1L	0.799418623	456
chr2.fa	203745323	203776949	WDR12	10.03158923	1520
chr2.fa	203776978	203851060	ALS2CR8	2.098850402	836
chr2.fa	203879602	204082717	NBEAL1	13.39462952	8056
chr2.fa	204103164	204170563	CYP20A1	1.987401687	1444
chr2.fa	204193003	204296892	ABI2	6.888886068	2964
chr2.fa	204298539	204400058	RAPH1	10.26804124	7068
chr2.fa	204571198	204602557	CD28	0	0
chr2.fa	204732509	204738683	CTLA4	0	0
chr2.fa	204801471	204826298	ICOS	0	0
chr2.fa	205410516	206480537	PARD3B	19.75006782	5016
chr2.fa	206547224	206662857	NRP2	0	0
chr2.fa	206858445	206950906	INO80D	7.368416756	6916
chr2.fa	206980297	206981296	LOC100329109	0	0
chr2.fa	206987803	207024243	NDUFS1	43.57976105	10336
chr2.fa	207024318	207027653	EEF1B2	89.5442234	5244
chr2.fa	207026605	207026674	SNORD51	0	0
chr2.fa	207026952	207027083	SNORA41	0	0
chr2.fa	207040042	207082771	GPR1	0	0
chr2.fa	207139523	207179148	ZDBF2	2.256234061	1520
chr2.fa	207308368	207482679	ADAM23	0	0
chr2.fa	207507142	207514173	LOC200726	0	0
chr2.fa	207516345	207583120	DYTN	0	0
chr2.fa	207602489	207630050	MDH1B	0	0
chr2.fa	207630112	207660911	FASTKD2	3.799949375	1748
chr2.fa	207804278	207834198	CPO	0	0
chr2.fa	207945529	208030614	KLF7	8.380340971	912
chr2.fa	207974711	207974797	MIR2355	0	0
chr2.fa	208394616	208470284	CREB1	6.437669377	4180
chr2.fa	208473839	208489973	FAM119A	1.391301666	456
chr2.fa	208576264	208620896	CCNYL1	10.53596997	2736

chr2.fa	208627310	208634143	FZD5		1.046262467	456
chr2.fa	208686012	208890284	PLEKHM3		8.986983438	5852
chr2.fa	208986331	208989313	CRYGD		0	0
chr2.fa	208992861	208994554	CRYGC		0	0
chr2.fa	209007297	209010877	CRYGB		0	0
chr2.fa	209025464	209028297	CRYGA		0	0
chr2.fa	209030071	209054773	C2orf80		0	0
chr2.fa	209100953	209119806	IDH1		461.9716425	71440
chr2.fa	209130991	209223475	PIKFYVE		18.20695493	12615
chr2.fa	209271556	209359231	PTH2R		0	0
chr2.fa	210288771	210598834	MAP2		1.375036178	912
chr2.fa	210636717	210864024	UNC80		1.265997164	1140
chr2.fa	210867352	210885434	RPE		13.51360856	1520
chr2.fa	210885951	211036051	C2orf67		21.04919839	6840
chr2.fa	211052716	211090215	ACADL		18.34491037	3033
chr2.fa	211154868	211179895	MYL1		0	0
chr2.fa	211295973	211341499	LANCL1		17.13192665	5244
chr2.fa	211342409	211543831	CPS1		0.372298952	152
chr2.fa	211482295	211484599	CPS1-IT		0	0
chr2.fa	212240442	213403352	ERBB4		0.672005632	532
chr2.fa	213290987	213291084	MIR548F2		0	0
chr2.fa	213864411	214016333	IKZF2		17.36566774	11135
chr2.fa	214149116	215275225	SPAG16		12.10950535	1748
chr2.fa	215276461	215440653	VWC2L		0	0
chr2.fa	215593275	215674428	BARD1		7.059974906	1216
chr2.fa	215796266	216003151	ABCA12		0	0
chr2.fa	216176679	216214496	ATIC		47.78348611	6612
chr2.fa	216225179	216300791	FN1		0.259946598	152
chr2.fa	216476286	216708259	LOC646324		0	0
chr2.fa	216807314	216878346	MREG		7.481070323	1596
chr2.fa	216903111	216946539	PECR		8.55097799	1064
chr2.fa	216946589	216967506	TMEM169		0.331032806	76
chr2.fa	216974020	217071016	XRCC5		98.09610503	22341
chr2.fa	217081612	217084915	PKI55		1.039334574	228
chr2.fa	217122585	217236750		4-Mar	0	0
chr2.fa	217277137	217347774	SMARCAL1		11.51114623	2536
chr2.fa	217363520	217366188	RPL37A		2716.391348	75392
chr2.fa	217498127	217529158	IGFBP2		2.402924667	228
chr2.fa	217536828	217560272	IGFBP5		4.402977289	1824
chr2.fa	217724182	217724782	TNP1		0	0
chr2.fa	218148746	218621316	DIRC3		0	0
chr2.fa	218664512	218808796	TNS1		6.177572172	4215
chr2.fa	218899657	218955304	RUFY4		0	0

chr2.fa	218923878	218926013	CXCR2P1	0	0
chr2.fa	218990013	219001976	CXCR2	0	0
chr2.fa	219027568	219031716	CXCR1	0	0
chr2.fa	219081874	219119071	ARPC2	118.4955875	11912
chr2.fa	219125738	219128582	GPBAR1	0	0
chr2.fa	219128852	219134893	AAMP	50.43174856	6004
chr2.fa	219135115	219211516	PNKD	18.88513543	4696
chr2.fa	219138917	219157280	TMBIM1	37.83427856	5901
chr2.fa	219221579	219232817	C2orf62	1.730316609	152
chr2.fa	219246752	219261617	SLC11A1	1.188585489	304
chr2.fa	219264478	219270664	CTDSP1	59.44328083	10412
chr2.fa	219267369	219267445	MIR26B	0	0
chr2.fa	219283838	219314248	VIL1	2.508198521	456
chr2.fa	219314974	219433084	USP37	4.404633959	2349
chr2.fa	219433678	219458999	RQCD1	13.9896753	836
chr2.fa	219472632	219501904	PLCD4	0.741284564	152
chr2.fa	219502640	219524261	ZNF142	7.34462095	2888
chr2.fa	219524379	219528166	BCS1L	3.441355603	380
chr2.fa	219528587	219536761	RNF25	8.16075688	835
chr2.fa	219536782	219567440	STK36	3.268911307	1064
chr2.fa	219575568	219620138	TTLL4	5.95678323	1976
chr2.fa	219646472	219680016	CYP27A1	5.490957721	836
chr2.fa	219687106	219696512	PRKAG3	0	0
chr2.fa	219724546	219738954	WNT6	0	0
chr2.fa	219745255	219758651	WNT10A	0	0
chr2.fa	219824398	219826877	CDK5R2	0	0
chr2.fa	219841006	219842644	LOC151300	0	0
chr2.fa	219845809	219850379	FEV	7.309830878	912
chr2.fa	219854912	219858127	CRYBA2	5.173328883	304
chr2.fa	219866367	219866430	MIR375	0	0
chr2.fa	219867576	219906245	CCDC108	0	0
chr2.fa	219919142	219925238	IHH	0	0
chr2.fa	219923410	219923472	MIR3131	0	0
chr2.fa	219940046	220025587	NHEJ1	13.6068339	1900
chr2.fa	220026181	220034817	SLC23A3	2.840285572	456
chr2.fa	220036619	220041702	C2orf24	28.69804421	3952
chr2.fa	220042939	220050197	FAM134A	7.938311269	2432
chr2.fa	220071538	220074370	ZFAND2B	18.66208739	1596
chr2.fa	220074488	220083712	ABCB6	0.379226845	76
chr2.fa	220084102	220094361	ATG9A	4.247099694	1064
chr2.fa	220094479	220101391	ANKZF1	13.45577571	2356
chr2.fa	220101503	220110131	GLB1L	0.917494019	152
chr2.fa	220110192	220115000	STK16	16.41549214	3344

chr2.fa	220115060	220118638	TUBA4A	14.51935791	1368
chr2.fa	220117965	220136910	TUBA4B	0	0
chr2.fa	220144040	220151622	DNAJB2	39.8323733	8265
chr2.fa	220154345	220174295	PTPRN	0	0
chr2.fa	220158833	220158922	MIR153-1	0	0
chr2.fa	220192131	220197899	RESP18	0	0
chr2.fa	220238180	220252662	DNPEP	16.22196295	2626
chr2.fa	220283099	220291461	DES	0	0
chr2.fa	220299700	220358354	SPEG	0.098345961	76
chr2.fa	220363587	220371718	GMPPA	11.0117355	1368
chr2.fa	220378892	220403494	ACCN4	0	0
chr2.fa	220403669	220408487	CHPF	2.883208388	608
chr2.fa	220408745	220415317	TMEM198	0	0
chr2.fa	220413795	220413869	MIR3132	0	0
chr2.fa	220415450	220436268	OBSL1	6.765388842	3268
chr2.fa	220436954	220440435	INHA	0	0
chr2.fa	220462596	220481173	STK11IP	3.044809025	728
chr2.fa	220492292	220506702	SLC4A3	0.798364379	228
chr2.fa	220771223	220771286	MIR4268	0	0
chr2.fa	222282747	222437010	EPHA4	0.540978089	228
chr2.fa	223064606	223163715	PAX3	0	0
chr2.fa	223162866	223169936	CCDC140	0	0
chr2.fa	223289322	223423617	SGPP2	2.86152107	228
chr2.fa	223436162	223520827	FARSB	16.25449392	2410
chr2.fa	223536457	223574649	MOGAT1	0	0
chr2.fa	223725732	223808119	ACSL3	10.23611269	2964
chr2.fa	223916862	223920355	KCNE4	0	0
chr2.fa	224461658	224467217	SCG2	0	0
chr2.fa	224620047	224702319	AP1S3	7.722191125	2052
chr2.fa	224740065	224810052	WDFY1	25.0509603	7663
chr2.fa	224822121	224832431	MRPL44	17.02499612	1976
chr2.fa	224839765	224904036	SERPINE2	0.475012498	76
chr2.fa	225243415	225266711	FAM124B	0	0
chr2.fa	225334869	225450110	CUL3	20.41529617	9196
chr2.fa	225629807	225907330	DOCK10	0.924120699	456
chr2.fa	226265602	226518734	KIAA1486	0.74806185	228
chr2.fa	227596033	227663506	IRS1	3.406565531	1976
chr2.fa	227700671	227863923	RHBDD1	14.4268856	4940
chr2.fa	227867427	228029275	COL4A4	1.217501912	836
chr2.fa	228029281	228179508	COL4A3	0.957856527	515
chr2.fa	228192228	228222549	MFF	25.61287268	3420
chr2.fa	228226874	228244022	TM4SF20	0	0
chr2.fa	228336888	228425938	AGFG1	7.07352948	4104

chr2.fa	228474806	228498036	C2orf83	0	0
chr2.fa	228549926	228582745	SLC19A3	0	0
chr2.fa	228678558	228682280	CCL20	0	0
chr2.fa	228736327	228789026	WDR69	0	0
chr2.fa	228844670	229046361	SPHKAP	0	0
chr2.fa	229888689	230136057	PID1	1.622482447	304
chr2.fa	230222345	230579286	DNER	0	0
chr2.fa	230631930	230786655	TRIP12	89.27674648	38104
chr2.fa	230787207	230877825	FBXO36	5.285982449	988
chr2.fa	230899690	230933715	SLC16A14	0.780743433	228
chr2.fa	231033645	231090444	SP110	5.900004628	1216
chr2.fa	231090445	231177930	SP140	0	0
chr2.fa	231191894	231268445	SP140L	7.722191125	1368
chr2.fa	231280871	231410317	SP100	9.939116922	4547
chr2.fa	231577557	231685790	CAB39	46.63586556	13439
chr2.fa	231729621	231743963	ITM2C	12.69943051	1748
chr2.fa	231772043	231789941	GPR55	0	0
chr2.fa	231849083	231860747	LOC348761	0	0
chr2.fa	231860839	231871999	SPATA3	0	0
chr2.fa	231902281	231914427	C2orf72	4.477376837	1064
chr2.fa	231921578	232037540	PSMD1	35.81027953	7906
chr2.fa	231972950	231989824	HTR2B	0	0
chr2.fa	232063342	232209914	ARMC9	5.160527341	760
chr2.fa	232260335	232265875	B3GNT7	0	0
chr2.fa	232319459	232329205	NCL	151.6595625	27360
chr2.fa	232320511	232320647	SNORA75	0	0
chr2.fa	232321155	232321234	SNORD20	0	0
chr2.fa	232325079	232325153	SNORD82	0	0
chr2.fa	232373137	232379050	C2orf52	0	0
chr2.fa	232387871	232395182	NMUR1	0	0
chr2.fa	232457575	232458994	C2orf57	0	0
chr2.fa	232573235	232578250	PTMA	182.1198519	13604
chr2.fa	232597147	232645974	PDE6D	12.2855642	912
chr2.fa	232651162	232673434	COPS7B	9.842427631	1292
chr2.fa	232756952	232757008	MIR1471	0	0
chr2.fa	232790135	232790959	NPPC	0	0
chr2.fa	232826293	233201908	DIS3L2	9.47841203	2204
chr2.fa	233243348	233247599	ALPP	0	0
chr2.fa	233250460	233251754	ECEL1P2	1.76766699	152
chr2.fa	233271552	233275424	ALPPL2	0	0
chr2.fa	233320833	233324742	ALPI	0.897764584	152
chr2.fa	233344537	233352532	ECEL1	0	0
chr2.fa	233385173	233390425	LOC646960	0	0

chr2.fa	233390922	233400205	CHRND	0.657396814	76
chr2.fa	233404437	233411038	CHRNG	0	0
chr2.fa	233412779	233415226	TIGD1	1.40274775	228
chr2.fa	233415357	233433920	EIF4E2	36.30080448	2432
chr2.fa	233470767	233547491	EFHD1	27.705247	3876
chr2.fa	233562015	233725289	GIGYF2	31.03937087	16465
chr2.fa	233630512	233641275	KCNJ13	0	0
chr2.fa	233734994	233741107	C2orf82	0	0
chr2.fa	233743396	233877951	NGEF	1.994630792	456
chr2.fa	233897382	233899767	NEU2	0	0
chr2.fa	233925036	234116549	INPP5D	4.736570403	1520
chr2.fa	234160217	234204320	ATG16L1	15.12705462	3420
chr2.fa	234184372	234184649	SCARNA5	8.234554002	152
chr2.fa	234197322	234197587	SCARNA6	0	0
chr2.fa	234216309	234255701	SAG	0	0
chr2.fa	234263153	234380743	DGKD	12.34610796	5244
chr2.fa	234384165	234474236	USP40	22.58161822	8422
chr2.fa	234526291	234527208	UGT1A8	0	0
chr2.fa	234545123	234681951	UGT1A10	0	0
chr2.fa	234580544	234581435	UGT1A9	3.849649478	228
chr2.fa	234590584	234591438	UGT1A7	0	0
chr2.fa	234600321	234602511	UGT1A6	3.995135233	304
chr2.fa	234621638	234622504	UGT1A5	0	0
chr2.fa	234627438	234628333	UGT1A4	0	0
chr2.fa	234637773	234638639	UGT1A3	0	0
chr2.fa	234651396	234652661	DNAJB3	0	0
chr2.fa	234668919	234669797	UGT1A1	0	0
chr2.fa	234745486	234763212	HJURP	0	0
chr2.fa	234774090	234777055	MSL3L2	3.451145017	456
chr2.fa	234826043	234928166	TRPM8	29.92036553	11167
chr2.fa	234959346	234985776	SPP2	0	0
chr2.fa	235401686	235405693	ARL4C	8.852943766	2356
chr2.fa	235860628	235964358	SH3BP4	21.20055779	7296
chr2.fa	236402736	237034120	AGAP1	10.12255548	3040
chr2.fa	237074307	237076652	GBX2	0	0
chr2.fa	237103515	237172988	ASB18	0	0
chr2.fa	237232794	237416092	IQCA1	1.077136773	228
chr2.fa	237478380	237490994	CXCR7	0.545044461	76
chr2.fa	237994084	238007489	COPS8	17.58269152	2712
chr2.fa	238232655	238322850	COL6A3	0	0
chr2.fa	238395878	238463961	MLPH	310.4271433	77418
chr2.fa	238475217	238475818	PRLH	0	0
chr2.fa	238482965	238499769	RAB17	13.1696236	1748

chr2.fa	238536224	238690290	LRRFIP1	23.91147249	12341
chr2.fa	238707388	238751451	RBM44	0	0
chr2.fa	238768187	238820755	RAMP1	1.259219877	76
chr2.fa	238875700	238950682	UBE2F	32.6945349	2935
chr2.fa	238969632	239008054	SCLY	5.528308102	903
chr2.fa	239008951	239041928	ESPNL	0.244434142	76
chr2.fa	239047363	239061547	KLHL30	0.310550339	76
chr2.fa	239079043	239112324	ILKAP	16.65751658	1596
chr2.fa	239133754	239140318	LOC151174	0	0
chr2.fa	239140327	239142985	LOC643387	0	0
chr2.fa	239146908	239148681	HES6	0.839178705	76
chr2.fa	239152679	239197207	PER2	5.05344621	2128
chr2.fa	239229185	239309541	TRAF3IP1	11.51189927	3192
chr2.fa	239335626	239360891	ASB1	6.53300321	2964
chr2.fa	239756673	239795893	TWIST2	0	0
chr2.fa	239840998	239847965	FLJ43879	0	0
chr2.fa	239969864	240322643	HDAC4	5.605719777	3341
chr2.fa	240115027	240117153	MGC16025	0	0
chr2.fa	240227157	240227240	MIR4269	0	0
chr2.fa	240896789	240964819	NDUFA10	23.92547888	7808
chr2.fa	240968908	240969846	OR6B2	0	0
chr2.fa	240981230	240982399	PRR21	0	0
chr2.fa	240984494	240985489	OR6B3	0	0
chr2.fa	241065980	241075764	MYEOV2	49.38488367	3243
chr2.fa	241078446	241080073	OTOS	0	0
chr2.fa	241375115	241407495	GPC1	1.242050751	304
chr2.fa	241388836	241396117	PP14571	4.539878481	608
chr2.fa	241395418	241395506	MIR149	0	0
chr2.fa	241418839	241497405	ANKMY1	2.689227381	608
chr2.fa	241499471	241503431	DUSP28	0	0
chr2.fa	241508104	241518143	RNPEPL1	23.58390363	4560
chr2.fa	241526133	241538526	CAPN10	0.865836033	152
chr2.fa	241544825	241570676	GPR35	1.322775766	228
chr2.fa	241615835	241622317	AQP12B	0	0
chr2.fa	241631262	241637900	AQP12A	0	0
chr2.fa	241653184	241759624	KIF1A	2.856400454	1672
chr2.fa	241808162	241818536	AGXT	5.730271247	608
chr2.fa	241825465	241835573	C2orf54	7.395827116	1292
chr2.fa	241894036	241906868	LOC200772	0	0
chr2.fa	241938255	242033643	SNED1	0.341123433	152
chr2.fa	242026509	242041747	MTERFD2	10.56006699	3116
chr2.fa	242045515	242088878	PASK	4.295444339	1292
chr2.fa	242089902	242122439	PPP1R7	117.2470607	10066

chr2.fa	242127924	242164791	ANO7		1.544016527	456
chr2.fa	242166679	242255254	HDLBP		185.9958575	81521
chr2.fa	242254723	242293441		2-Sep	79.68462664	19878
chr2.fa	242295664	242434256	FARP2		11.73208578	3151
chr2.fa	242434432	242448034	STK25		25.12686591	3572
chr2.fa	242483801	242484259	BOK-AS		0	0
chr2.fa	242498870	242513553	BOK		16.32407407	2584
chr2.fa	242523820	242576725	THAP4		13.82611678	2128
chr2.fa	242577027	242613271	ATG4B		11.06279106	2128
chr2.fa	242615157	242626383	DTYMK		6.627132193	532
chr2.fa	242641456	242668896	ING5		6.606047301	2280
chr2.fa	242674030	242708231	D2HGDH		6.639331309	1140
chr2.fa	242716240	242743702	GAL3ST2		0	0
chr2.fa	242750160	242758739	NEU4		0.834208695	152
chr2.fa	242792033	242801058	PDCD1		0	0
chr2.fa	242811886	242815482	C2orf85		0	0
chr2.fa	243030844	243102469	LOC728323		1.014785735	76
chr20.fa	68351	77296	DEFB125		0	0
chr20.fa	123252	126392	DEFB126		0	0
chr20.fa	138186	139804	DEFB127		0	0
chr20.fa	168527	170264	DEFB128		0	0
chr20.fa	207899	210527	DEFB129		0	0
chr20.fa	238377	241736	DEFB132		3.779316302	532
chr20.fa	251524	271390	C20orf96		5.791869253	598
chr20.fa	278204	280963	ZCCHC3		11.6119019	2128
chr20.fa	306239	310867	SOX12		3.708983126	1140
chr20.fa	327370	335512	NRSN2		1.918875787	304
chr20.fa	361308	378203	TRIB3		10.26969791	1672
chr20.fa	388709	411610	RBCK1		22.79276836	4180
chr20.fa	416124	443187	TBC1D20		25.17310207	7448
chr20.fa	463338	524482	CSNK2A1		31.07717307	5852
chr20.fa	584637	590910	TCF15		0	0
chr20.fa	627268	633890	SRXN1		15.61848322	2660
chr20.fa	642240	656823	SCRT2		0	0
chr20.fa	740724	749228	C20orf54		4.671809663	836
chr20.fa	814356	826922	FAM110A		16.06262141	2052
chr20.fa	853297	896960	ANGPT4		0	0
chr20.fa	939096	982907	RSPO4		0	0
chr20.fa	1093906	1148426	PSMF1		37.16558627	9481
chr20.fa	1161215	1165117	C20orf46		0	0
chr20.fa	1184098	1188918	C20orf202		0	0
chr20.fa	1206764	1235145	RAD21L1		0	0
chr20.fa	1246960	1289971	SNPH		0.916740987	304

chr20.fa	1306474	1309879	SDCBP2	0	0
chr20.fa	1349621	1356247	FKBP1A	148.292757	17113
chr20.fa	1422811	1448417	NSFL1C	19.16059448	4636
chr20.fa	1455236	1472233	SIRPB2	0	0
chr20.fa	1514897	1538343	SIRPD	0	0
chr20.fa	1545029	1600689	SIRPB1	0	0
chr20.fa	1609798	1638425	SIRPG	0	0
chr20.fa	1754011	1760392	LOC100289473	0	0
chr20.fa	1874813	1920540	SIRPA	0.255579014	76
chr20.fa	1959402	1974931	PDYN	0	0
chr20.fa	2082528	2129198	STK35	15.8898759	6764
chr20.fa	2276613	2321725	TGM3	0.42757149	76
chr20.fa	2361554	2413399	TGM6	0	0
chr20.fa	2442281	2451499	SNRPB	39.85752456	2665
chr20.fa	2443598	2443693	SNORD119	0	0
chr20.fa	2462467	2489778	ZNF343	4.732353425	1140
chr20.fa	2517253	2622430	TMC2	0	0
chr20.fa	2633254	2639039	NOP56	20.2884856	2736
chr20.fa	2633423	2633488	MIR1292	0	0
chr20.fa	2634858	2634932	SNORD110	0	0
chr20.fa	2635713	2635844	SNORA51	0	0
chr20.fa	2637270	2637340	SNORD56	0	0
chr20.fa	2637585	2637656	SNORD57	0	0
chr20.fa	2639041	2644843	IDH3B	31.44630929	3180
chr20.fa	2673524	2740754	EBF4	5.11158027	988
chr20.fa	2774715	2781292	CPXM1	0	0
chr20.fa	2795657	2796476	C20orf141	0	0
chr20.fa	2796976	2797804	LOC100288797	0	0
chr20.fa	2815971	2821332	FAM113A	10.30659647	1368
chr20.fa	2821373	2847378	VPS16	11.88118609	1719
chr20.fa	2854142	3019315	PTPRA	14.27417074	3249
chr20.fa	3024268	3026391	GNRH2	0	0
chr20.fa	3026675	3028896	MRPS26	45.16745343	3116
chr20.fa	3052266	3053162	OXT	2.235600988	76
chr20.fa	3063202	3065370	AVP	0	0
chr20.fa	3088219	3104052	UBOX5	4.087908759	1140
chr20.fa	3127165	3129906	FASTKD5	9.183524753	1672
chr20.fa	3143273	3149207	ProSAPiP1	10.47090802	3648
chr20.fa	3171012	3185295	DDRGK1	6.113112645	509
chr20.fa	3190056	3204506	ITPA	25.15713779	1794
chr20.fa	3208063	3219887	SLC4A11	1.010267544	228
chr20.fa	3229948	3388255	C20orf194	11.86838455	5402
chr20.fa	3451665	3631769	ATRN	4.063962346	2356

chr20.fa	3639939	3644046	GFRA4	0	0
chr20.fa	3648620	3662738	ADAM33	0	0
chr20.fa	3667617	3687775	SIGLEC1	0	0
chr20.fa	3713317	3733758	HSPA12B	0	0
chr20.fa	3734158	3748452	C20orf27	3.327346579	304
chr20.fa	3758151	3762102	SPEF1	0.645348305	68
chr20.fa	3764498	3767337	CENPB	26.19707479	4940
chr20.fa	3776401	3786761	CDC25B	0.934663145	228
chr20.fa	3801203	3805954	C20orf29	3.123877371	380
chr20.fa	3827449	3856762	MAVS	18.29822239	14288
chr20.fa	3869486	3904502	PANK2	13.01223995	2128
chr20.fa	3898141	3898218	MIR103-2	0	0
chr20.fa	3912069	3996216	RNF24	5.144261853	1216
chr20.fa	4129450	4168369	SMOX	4.530089067	684
chr20.fa	4173737	4176600	LOC728228	0	0
chr20.fa	4201278	4229659	ADRA1D	0	0
chr20.fa	4666797	4682234	PRNP	5.4100821	1064
chr20.fa	4702556	4709106	PRND	0	0
chr20.fa	4711928	4721314	PRNT	0	0
chr20.fa	4760669	4804291	RASSF2	1.050328839	380
chr20.fa	4833002	4990939	SLC23A2	2.919956343	1368
chr20.fa	5080484	5093733	C20orf30	94.37447097	11248
chr20.fa	5095599	5107268	PCNA	15.14603103	1064
chr20.fa	5107482	5171989	CDS2	3.386384277	608
chr20.fa	5282686	5295015	PROKR2	0	0
chr20.fa	5451842	5457780	LOC643406	0	0
chr20.fa	5479218	5485242	LOC149837	0	0
chr20.fa	5525080	5591672	GPCPD1	4.822566642	1748
chr20.fa	5731043	5844559	C20orf196	2.972969786	228
chr20.fa	5891974	5906005	CHGB	0	0
chr20.fa	5918486	5931173	TRMT6	9.905682308	1520
chr20.fa	5931298	5975831	MCM8	5.19170286	1292
chr20.fa	5986739	6020697	CRLS1	16.96159084	4631
chr20.fa	6021425	6034694	LRRN4	0	0
chr20.fa	6055492	6104191	FERMT1	10.44394948	3572
chr20.fa	6748745	6760910	BMP2	2.543591019	532
chr20.fa	7863631	7921093	HAO1	0	0
chr20.fa	7961716	8000393	TMX4	7.113138956	1117
chr20.fa	8113296	8865547	PLCB1	9.056714188	4103
chr20.fa	9049701	9461462	PLCB4	6.603938811	2481
chr20.fa	9495005	9511171	C20orf103	1.121113834	152
chr20.fa	9518037	9819687	PAK7	0.239614738	76
chr20.fa	10015697	10037407	ANKRD5	2.994054678	760

chr20.fa	10199477	10288065	SNAP25	0	0
chr20.fa	10385833	10414866	MKKS	26.00444924	4788
chr20.fa	10415951	10604027	C20orf94	7.321578175	684
chr20.fa	10618332	10654694	JAG1	7.609537558	3025
chr20.fa	11871477	11907243	BTBD3	16.30886283	5396
chr20.fa	12989627	13147411	SPTLC3	22.92198863	5852
chr20.fa	13202418	13281297	ISM1	0	0
chr20.fa	13370036	13619583	TASP1	10.71097458	1672
chr20.fa	13694969	13765532	ESF1	12.81283711	2736
chr20.fa	13765672	13799067	C20orf7	2.762120865	442
chr20.fa	13830050	13971262	SEL1L2	0	0
chr20.fa	13976146	16033841	MACROD2	4.380085121	1485
chr20.fa	14304639	14318313	FLRT3	5.688854494	1520
chr20.fa	16252749	16554079	KIF16B	14.62177024	6064
chr20.fa	16710609	16722417	SNRPB2	13.65081096	1433
chr20.fa	16729003	16732809	OTOR	0	0
chr20.fa	17207631	17465222	PCSK2	0.241271408	76
chr20.fa	17474550	17539605	BFSP1	0	0
chr20.fa	17550599	17588652	DSTN	161.9233867	18772
chr20.fa	17594323	17662928	RRBP1	8.257144958	2079
chr20.fa	17674320	17716517	BANF2	0	0
chr20.fa	17922244	17949490	SNX5	35.08465803	5139
chr20.fa	17943353	17943589	SNORD17	9.659139676	152
chr20.fa	17949762	17971762	C20orf72	9.580221937	1353
chr20.fa	18004796	18038521	OVOL2	5.88870915	608
chr20.fa	18118499	18123812	PET117	0	0
chr20.fa	18125743	18169031	CSRP2BP	10.48325774	2194
chr20.fa	18269121	18297640	ZNF133	5.060976529	912
chr20.fa	18359693	18362127	MGC44328	0	0
chr20.fa	18364011	18447829	C20orf12	1.635133382	380
chr20.fa	18448033	18465286	POLR3F	2.658202468	380
chr20.fa	18451259	18451335	MIR3192	0	0
chr20.fa	18467188	18477887	RBBP9	10.57015762	2707
chr20.fa	18488188	18542059	SEC23B	44.87648192	11165
chr20.fa	18548073	18550203	LOC388789	83.75190233	2736
chr20.fa	18568556	18744560	DTD1	16.74622373	1520
chr20.fa	18768615	18774447	HSPC072	0	0
chr20.fa	18774693	18776709	LOC100270804	0	0
chr20.fa	18794370	18795035	C20orf79	0	0
chr20.fa	19193290	19703541	SLC24A3	0	0
chr20.fa	19222946	19265240	LOC100130264	0	0
chr20.fa	19870210	19983100	RIN2	19.14869658	5396
chr20.fa	19997937	20014269	NAA20	30.65125825	2487

chr20.fa	20015012	20036690	CRNKL1	15.60342258	4484
chr20.fa	20037262	20341346	C20orf26	0	0
chr20.fa	20348765	20351592	INSM1	0	0
chr20.fa	20370272	20693266	RALGAPA2	58.99146172	36921
chr20.fa	21106624	21227258	PLK1S1	11.97727296	1748
chr20.fa	21283942	21370463	XRN2	34.43554456	7854
chr20.fa	21376005	21378047	NKX2-4	0	0
chr20.fa	21491660	21494664	NKX2-2	0	0
chr20.fa	21686297	21696620	PAX1	0	0
chr20.fa	22380971	22401281	LOC284788	0	0
chr20.fa	22541192	22559280	NCRNA00261	0	0
chr20.fa	22561642	22566101	FOXA2	0	0
chr20.fa	23016057	23017314	SSTR4	0	0
chr20.fa	23026270	23030301	THBD	0	0
chr20.fa	23059993	23066977	CD93	0	0
chr20.fa	23105705	23113273	LOC200261	0	0
chr20.fa	23331373	23335408	NXT1	9.222381197	684
chr20.fa	23345021	23353683	GZF1	7.242811042	2280
chr20.fa	23355156	23402156	NAPB	3.252495212	836
chr20.fa	23420322	23425567	CSTL1	0	0
chr20.fa	23431041	23433482	CST11	0	0
chr20.fa	23471766	23476655	CST8	0	0
chr20.fa	23499783	23522655	CSTT	0	0
chr20.fa	23545370	23549386	CST9L	0	0
chr20.fa	23583047	23586610	CST9	0	0
chr20.fa	23614294	23618574	CST3	39.87665157	2052
chr20.fa	23666277	23669662	CST4	0	0
chr20.fa	23728190	23731574	CST1	0	0
chr20.fa	23804404	23807312	CST2	0	0
chr20.fa	23856572	23860380	CST5	0	0
chr20.fa	23965690	23969416	GGTLC1	0	0
chr20.fa	24449835	24647167	TMEM90B	0	0
chr20.fa	24929866	24940564	CST7	0	0
chr20.fa	24943580	24973425	C20orf3	3.092099426	456
chr20.fa	24986873	25039616	ACSS1	21.75087348	6460
chr20.fa	25056099	25062767	VSX1	0	0
chr20.fa	25121434	25129426	LOC284798	0	0
chr20.fa	25176339	25207360	ENTPD6	17.81974595	3268
chr20.fa	25228706	25278648	PYGB	39.56745669	10853
chr20.fa	25275379	25371477	ABHD12	27.000861	4041
chr20.fa	25388323	25429191	GINS1	0	0
chr20.fa	25433338	25566153	NINL	245.9954782	81211
chr20.fa	25593573	25604648	NANP	2.109091635	532

chr20.fa	25654851	25677469	ZNF337	8.950687302	1900
chr20.fa	25744102	25781927	FAM182B	0	0
chr20.fa	25990435	26002430	LOC100134868	0	0
chr20.fa	26035250	26067553	FAM182A	0	0
chr20.fa	26084052	26094677	C20orf191	0	0
chr20.fa	26188822	26188914	MIR663	0	0
chr20.fa	29611879	29634007	FRG1B	16.9393011	1064
chr20.fa	29845467	29847435	DEFB115	0	0
chr20.fa	29891015	29896388	DEFB116	0	0
chr20.fa	29956421	29961705	DEFB118	0	0
chr20.fa	29964967	29978406	DEFB119	0	0
chr20.fa	29992648	30000641	DEFB121	0	0
chr20.fa	30009242	30016977	DEFB122	0	0
chr20.fa	30028411	30038060	DEFB123	0	0
chr20.fa	30053309	30060816	DEFB124	0	0
chr20.fa	30063105	30072708	REM1	0	0
chr20.fa	30073581	30075377	NCRNA00028	0	0
chr20.fa	30102241	30157370	HM13	8.124912564	1797
chr20.fa	30135185	30136019	PSIMCT-1	1.370819199	76
chr20.fa	30193092	30194313	ID1	99.28679901	8056
chr20.fa	30194989	30195043	MIR3193	0	0
chr20.fa	30225691	30232800	COX4I2	0	0
chr20.fa	30252261	30310656	BCL2L1	68.88223399	11704
chr20.fa	30326904	30389603	TPX2	0	0
chr20.fa	30407178	30422500	MYLK2	0	0
chr20.fa	30432103	30433420	FOXS1	0	0
chr20.fa	30448870	30458479	DUSP15	1.147168737	132
chr20.fa	30458505	30530858	TTLL9	0	0
chr20.fa	30532758	30539883	PDRG1	15.00581649	1368
chr20.fa	30555805	30586256	XKR7	0	0
chr20.fa	30598245	30619984	C20orf160	0.452120329	76
chr20.fa	30639991	30689657	HCK	0	0
chr20.fa	30697309	30755061	TM9SF4	5.179202531	1368
chr20.fa	30776949	30778163	TSPY26P	0	0
chr20.fa	30780307	30795546	PLAGL2	7.083017681	2660
chr20.fa	30795696	30826467	POFUT1	4.85088064	1976
chr20.fa	30865454	30922811	KIF3B	32.01831229	12996
chr20.fa	30946147	31027122	ASXL1	11.87004122	5852
chr20.fa	31030862	31071288	C20orf112	9.339854168	3724
chr20.fa	31175281	31196694	LOC149950	0	0
chr20.fa	31219427	31239783	C20orf203	0	0
chr20.fa	31290493	31331814	COMMD7	31.13003591	3950
chr20.fa	31350191	31397162	DNMT3B	1.483322159	456

chr20.fa	31407699	31438211	MAPRE1	58.47412883	10184
chr20.fa	31571581	31592239	SUN5	0	0
chr20.fa	31595407	31611515	BPIL1	0	0
chr20.fa	31619454	31631853	BPIL3	0	0
chr20.fa	31643230	31661434	C20orf185	0	0
chr20.fa	31669318	31699557	C20orf186	0	0
chr20.fa	31755960	31769223	C20orf70	0	0
chr20.fa	31781411	31798268	BASE	0	0
chr20.fa	31805135	31815559	C20orf71	0	0
chr20.fa	31823802	31831115	PLUNC	0	0
chr20.fa	31870941	31897684	C20orf114	0	0
chr20.fa	31946645	31989337	CDK5RAP1	8.502181526	1181
chr20.fa	31995763	32031698	SNTA1	10.27210761	1596
chr20.fa	32077928	32237837	CBFA2T2	17.78284739	9348
chr20.fa	32244893	32262264	NECAB3	29.24263685	3761
chr20.fa	32250092	32251721	C20orf144	0	0
chr20.fa	32254304	32256331	C20orf134	1.693267442	228
chr20.fa	32263292	32274210	E2F1	1.271720206	228
chr20.fa	32290550	32308136	PXMP4	9.369523623	3561
chr20.fa	32319808	32380075	ZNF341	1.725949025	380
chr20.fa	32399110	32442173	CHMP4B	98.68512655	10792
chr20.fa	32581732	32668067	RALY	41.03180245	4291
chr20.fa	32676115	32700085	EIF2S2	31.28335319	5384
chr20.fa	32848171	32857148	ASIP	0	0
chr20.fa	32868071	32899608	AHCY	101.9233142	17176
chr20.fa	32951062	33099197	ITCH	37.47101599	15871
chr20.fa	33054130	33054223	MIR644	0	0
chr20.fa	33104204	33128762	DYNLRB1	202.1863437	9196
chr20.fa	33134692	33148149	MAP1LC3A	20.28682893	1510
chr20.fa	33148346	33265089	PIGU	0.624715232	76
chr20.fa	33292148	33301237	TP53INP2	29.42005116	8056
chr20.fa	33302578	33413433	NCOA6	23.13690392	10849
chr20.fa	33421378	33422265	HMGB3P1	0	0
chr20.fa	33432523	33460661	GGT7	1.30063663	228
chr20.fa	33462766	33515769	ACSS2	6.094136242	1292
chr20.fa	33516236	33543601	GSS	26.50672149	3344
chr20.fa	33543704	33590206	MYH7B	0.176510668	76
chr20.fa	33578179	33578300	MIR499	0	0
chr20.fa	33590241	33680618	TRPC4AP	32.54588641	6725
chr20.fa	33703160	33735161	EDEM2	3.590004092	456
chr20.fa	33759774	33765165	PROCR	0.789327996	76
chr20.fa	33814539	33864804	MMP24	7.660593118	2204
chr20.fa	33866725	33872594	EIF6	69.69023718	5016

chr20.fa	33873534	33880225	FAM83C	0	0
chr20.fa	33890369	33999945	UQCC	11.22168079	1824
chr20.fa	34021149	34026027	GDF5	0	0
chr20.fa	34043223	34099803	CEP250	7.121422306	3936
chr20.fa	34108570	34117481	C20orf173	0	0
chr20.fa	34129778	34145405	ERGIC3	81.60967729	7402
chr20.fa	34146507	34195484	FER1L4	11.11866603	4857
chr20.fa	34203809	34208965	SPAG4	0	0
chr20.fa	34213953	34220845	CPNE1	43.97645824	5326
chr20.fa	34236847	34243123	RBM12	15.14497678	5928
chr20.fa	34256610	34287231	NFS1	9.340004774	1520
chr20.fa	34287288	34288902	ROMO1	70.65487099	1900
chr20.fa	34291531	34330193	RBM39	85.57740216	15859
chr20.fa	34359923	34538288	PHF20	12.82804835	5016
chr20.fa	34541546	34542428	SCAND1	33.96896602	1748
chr20.fa	34556529	34618622	C20orf152	0.593991532	76
chr20.fa	34633540	34638882	LOC647979	61.91126745	21964
chr20.fa	34700348	34820721	EPB41L1	39.95556931	16796
chr20.fa	34824447	34844853	C20orf4	15.99936673	2812
chr20.fa	34995444	35157040	DLGAP4	14.73156229	5776
chr20.fa	35169897	35178226	MYL9	7.714359593	608
chr20.fa	35201876	35222355	TGIF2	4.487316858	988
chr20.fa	35234137	35238161	C20orf24	3.99694251	125
chr20.fa	35240961	35274558	SLA2	0	0
chr20.fa	35280169	35374481	NDRG3	86.84761631	17092
chr20.fa	35380194	35402230	DSN1	5.911751925	988
chr20.fa	35405845	35492087	C20orf117	10.48340835	6074
chr20.fa	35504570	35522632	C20orf118	1.246870155	152
chr20.fa	35520227	35580246	SAMHD1	9.877217703	1957
chr20.fa	35626178	35724410	RBL1	0.520495622	152
chr20.fa	35729629	35807974	C20orf132	0.980748695	228
chr20.fa	35807456	35870025	RPN2	12.21492981	1953
chr20.fa	35879490	35885299	GHRH	0	0
chr20.fa	35918051	35945663	MANBAL	16.53808572	1444
chr20.fa	35973088	36033821	SRC	30.24311498	8436
chr20.fa	36145819	36156333	BLCAP	14.45324171	2356
chr20.fa	36149607	36152090	NNAT	0	0
chr20.fa	36322434	36500520	CTNNBL1	27.28144067	3420
chr20.fa	36531499	36573747	VSTM2L	0.583750298	76
chr20.fa	36611423	36661833	TTI1	13.45216116	3496
chr20.fa	36661948	36720766	RPRD1B	27.78747808	7144
chr20.fa	36756864	36793700	TGM2	20.17417537	5472
chr20.fa	36838907	36889174	KIAA1755	0.178468551	76

chr20.fa	36932552	36965905	BPI	0.620347647	76
chr20.fa	36974885	37005653	LBP	0.627877965	76
chr20.fa	37049239	37064018	LOC388796	6.14443877	532
chr20.fa	37053843	37053978	SNORA71B	0	0
chr20.fa	37055949	37056086	SNORA71A	0	0
chr20.fa	37058310	37058447	SNORA71C	0	0
chr20.fa	37062505	37062642	SNORA71D	0	0
chr20.fa	37075297	37079564	SNHG11	4.212008409	304
chr20.fa	37076726	37076861	SNORA39	0	0
chr20.fa	37078012	37078147	SNORA60	0	0
chr20.fa	37101486	37207504	RALGAPB	34.19517679	19608
chr20.fa	37209838	37217104	ADIG	0	0
chr20.fa	37230577	37279295	ARHGAP40	6.426524505	1211
chr20.fa	37353105	37358015	SLC32A1	0	0
chr20.fa	37377097	37401089	ACTR5	3.693771883	608
chr20.fa	37434348	37551667	PPP1R16B	4.394543332	1824
chr20.fa	37554955	37581703	FAM83D	0	0
chr20.fa	37590981	37668366	DHX35	5.47981285	1216
chr20.fa	37842424	37853391	LOC339568	0	0
chr20.fa	39314517	39317876	MAFB	8.857160745	1976
chr20.fa	39657462	39753126	TOP1	39.62242802	9821
chr20.fa	39766161	39804357	PLCG1	5.00570399	1730
chr20.fa	39807089	39928739	ZHX3	6.186457948	4104
chr20.fa	39969560	39988605	LPIN3	2.138911697	532
chr20.fa	39989223	39995498	EMILIN3	0	0
chr20.fa	40031170	40247133	CHD6	24.8996009	17176
chr20.fa	40701392	41818557	PTPRT	0.090213217	76
chr20.fa	42086504	42092244	SRSF6	36.4997555	9728
chr20.fa	42136320	42170534	L3MBTL1	0.642336177	152
chr20.fa	42187635	42214273	SGK2	9.782938114	1596
chr20.fa	42219579	42275862	IFT52	14.34134118	1595
chr20.fa	42295709	42345122	MYBL2	0	0
chr20.fa	42354801	42355642	GTSF1L	0	0
chr20.fa	42543492	42698254	TOX2	0	0
chr20.fa	42740337	42816218	JPH2	0	0
chr20.fa	42825136	42839431	C20orf111	19.65925218	2052
chr20.fa	42875908	42909013	GDAP1L1	0	0
chr20.fa	42935197	42939889	FITM2	1.19476035	76
chr20.fa	42965626	42979875	R3HDML	0	0
chr20.fa	42984441	43058311	HNF4A	15.5779701	1596
chr20.fa	43036760	43036843	MIR3646	0	0
chr20.fa	43104547	43123244	TTPAL	7.550951679	3116
chr20.fa	43124864	43150726	SERINC3	8.400823437	1520

chr20.fa	43160436	43247678	PKIG	11.17198068	1063
chr20.fa	43248163	43280376	ADA	0	0
chr20.fa	43285092	43300380	LOC79015	0	0
chr20.fa	43343885	43356452	WISP2	0	0
chr20.fa	43374488	43379668	KCNK15	0	0
chr20.fa	43380449	43438912	RIMS4	0	0
chr20.fa	43514344	43537161	YWHAB	180.6306561	37240
chr20.fa	43538703	43567962	PABPC1L	0.537664748	76
chr20.fa	43570771	43589114	TOMM34	47.48257458	6460
chr20.fa	43595120	43708593	STK4	15.7315886	6612
chr20.fa	43720950	43729753	KCNS1	0.756495807	228
chr20.fa	43738093	43743803	WFDC5	0	0
chr20.fa	43752067	43753106	WFDC12	0	0
chr20.fa	43803540	43805185	PI3	0	0
chr20.fa	43835638	43838414	SEMG1	7.571132933	836
chr20.fa	43850010	43853099	SEMG2	2.310000536	304
chr20.fa	43880879	43883206	SLPI	0	0
chr20.fa	43922087	43936967	MATN4	0	0
chr20.fa	43935491	43946464	RBPJL	0	0
chr20.fa	43953929	43977064	SDC4	23.09262564	4005
chr20.fa	43990577	44005442	SYS1	4.319240146	1292
chr20.fa	44002520	44006957	TP53TG5	0	0
chr20.fa	44034633	44037676	DBNDD2	0	0
chr20.fa	44044707	44054884	PIGT	2.039360885	304
chr20.fa	44098394	44110172	WFDC2	0	0
chr20.fa	44141101	44144264	SPINT3	0	0
chr20.fa	44162836	44168134	WFDC6	0	0
chr20.fa	44164919	44166189	SPINLW1-WFDC6	0	0
chr20.fa	44169265	44170793	SPINLW1	0	0
chr20.fa	44179791	44207965	WFDC8	0	0
chr20.fa	44236578	44259907	WFDC9	0	0
chr20.fa	44258385	44259831	WFDC10A	0	0
chr20.fa	44277202	44298878	WFDC11	0	0
chr20.fa	44313290	44333658	WFDC10B	0	0
chr20.fa	44330655	44337456	WFDC13	0	0
chr20.fa	44350988	44354335	SPINT4	0	0
chr20.fa	44402847	44420547	WFDC3	1.144608428	76
chr20.fa	44420576	44440066	DNTTIP1	11.45316278	981
chr20.fa	44441255	44445596	UBE2C	0	0
chr20.fa	44451855	44455953	TNNC2	0	0
chr20.fa	44462470	44471914	SNX21	4.281136734	836
chr20.fa	44472166	44486048	ACOT8	18.36268192	1140
chr20.fa	44486220	44507769	ZSWIM3	3.466205655	684

chr20.fa	44509848	44513905	ZSWIM1	4.106885162	760
chr20.fa	44515130	44516238	C20orf165	0	0
chr20.fa	44517267	44519590	NEURL2	1.207411285	76
chr20.fa	44519902	44527396	CTSA	4.291528574	532
chr20.fa	44527459	44540786	PLTP	0	0
chr20.fa	44563317	44576662	PCIF1	11.45888582	2052
chr20.fa	44577292	44600833	ZNF335	6.941748904	2052
chr20.fa	44637547	44645200	MMP9	0	0
chr20.fa	44650329	44688789	SLC12A5	0	0
chr20.fa	44689626	44718580	NCOA5	17.48856254	3724
chr20.fa	44746906	44758384	CD40	1.416603536	152
chr20.fa	44802376	44880334	CDH22	0	0
chr20.fa	44978177	44993064	SLC35C2	10.63747867	1556
chr20.fa	44994690	45035271	ELMO2	18.65199676	4560
chr20.fa	45092299	45093931	LOC100240726	0.700922056	76
chr20.fa	45129707	45142194	ZNF334	3.291954082	760
chr20.fa	45169585	45179213	C20orf123	0	0
chr20.fa	45186462	45298778	SLC13A3	3.589853486	988
chr20.fa	45313004	45318276	TP53RK	10.45946194	2280
chr20.fa	45338279	45364985	SLC2A10	2.162255685	608
chr20.fa	45523263	45817492	EYA2	12.28481117	2204
chr20.fa	45795609	45795700	MIR3616	0	0
chr20.fa	45838381	45985474	ZMYND8	30.98244166	9570
chr20.fa	45947246	45949498	LOC100131496	0	0
chr20.fa	46130601	46285621	NCOA3	30.99328532	16492
chr20.fa	46286150	46415360	SULF2	0	0
chr20.fa	46988654	46999381	LOC284749	0.318683083	76
chr20.fa	47240793	47444420	PREX1	4.31411953	1900
chr20.fa	47538275	47653230	ARFGEF2	33.37467328	19953
chr20.fa	47662838	47713486	CSE1L	23.14292817	5472
chr20.fa	47729876	47804904	STAU1	52.89612063	13371
chr20.fa	47835832	47860614	DDX27	16.56383941	2964
chr20.fa	47862439	47894714	ZNFX1	23.74505245	11552
chr20.fa	47894757	47905795	NCRNA00275	114.8488048	7519
chr20.fa	47895482	47895560	SNORD12C	0	0
chr20.fa	47896850	47896952	SNORD12B	0	0
chr20.fa	47897220	47897309	SNORD12	0	0
chr20.fa	47988505	48099181	KCNB1	0.304676691	76
chr20.fa	48120411	48184707	PTGIS	0	0
chr20.fa	48249483	48330421	B4GALT5	7.990722286	2508
chr20.fa	48429250	48508772	SLC9A8	2.691938295	1118
chr20.fa	48519929	48532080	SPATA2	5.850756344	1672
chr20.fa	48552914	48570422	RNF114	45.46881678	7448

chr20.fa	48599513	48605420	SNAI1	0	0
chr20.fa	48729644	48732494	UBE2V1	2.960017638	91
chr20.fa	48740274	48767852	TMEM189	6.659060744	684
chr20.fa	48807376	48809212	CEBPB	3.738501975	456
chr20.fa	48909257	48931456	LOC284751	0	0
chr20.fa	49126891	49201086	PTPN1	19.67506585	4332
chr20.fa	49202323	49202416	MIR645	0	0
chr20.fa	49202647	49253426	FAM65C	0.267326311	76
chr20.fa	49348081	49370278	PARD6B	23.06581771	7068
chr20.fa	49411467	49493714	BCAS4	1.705767771	152
chr20.fa	49506883	49547527	ADNP	52.21537982	16340
chr20.fa	49551405	49575060	DPM1	9.839114291	684
chr20.fa	49575363	49577820	MOCS3	6.053623128	988
chr20.fa	49620193	49639675	KCNG1	0	0
chr20.fa	50007765	50179168	NFATC2	1.721732046	380
chr20.fa	50069442	50069514	MIR3194	0	0
chr20.fa	50213314	50384908	ATP9A	9.664260293	4864
chr20.fa	50400583	50419048	SALL4	1.314191203	304
chr20.fa	50700550	50808524	ZFP64	3.674494267	1216
chr20.fa	51588946	52111869	TSHZ2	1.000779343	836
chr20.fa	52183610	52199636	ZNF217	73.1249661	27360
chr20.fa	52491040	52492248	SUMO1P1	0	0
chr20.fa	52560079	52687304	BCAS1	199.4721156	46025
chr20.fa	52769988	52790516	CYP24A1	10.51383084	2280
chr20.fa	52824502	52836492	PFDN4	3.401444915	304
chr20.fa	53092266	53267710	DOK5	1.248828038	152
chr20.fa	54572496	54580012	CBLN4	0	0
chr20.fa	54823788	54824871	MC3R	0	0
chr20.fa	54933983	54943718	C20orf108	78.49604115	15808
chr20.fa	54944445	54967351	AURKA	0.449409414	76
chr20.fa	54967427	54979582	CSTF1	20.91621296	3572
chr20.fa	54987168	55034396	CASS4	0.351364666	76
chr20.fa	55043647	55093942	C20orf43	65.76814604	7201
chr20.fa	55066548	55100859	GCNT7	0	0
chr20.fa	55099785	55101208	C20orf106	0	0
chr20.fa	55108302	55111576	C20orf107	0	0
chr20.fa	55204358	55214338	TFAP2C	7.590711762	1444
chr20.fa	55743809	55841707	BMP7	0	0
chr20.fa	55896558	55896647	MIR4325	0	0
chr20.fa	55904831	55919049	SPO11	0	0
chr20.fa	55926145	55953519	RAE1	17.93390558	2584
chr20.fa	55933496	55934878	MTRNR2L3	0	0
chr20.fa	55966463	55984386	RBM38	1.924448223	304

chr20.fa	56072224	56100154	CTCFL	0	0
chr20.fa	56136137	56141513	PCK1	41.77715338	7448
chr20.fa	56178902	56195632	ZBP1	0	0
chr20.fa	56223452	56286541	PMEPA1	77.22281488	26591
chr20.fa	56725983	56736183	C20orf85	0	0
chr20.fa	56807833	56884495	PPP4R1L	2.863930772	456
chr20.fa	56884771	56942563	RAB22A	9.393921855	5396
chr20.fa	56964175	57026156	VAPB	16.72649429	8816
chr20.fa	57034426	57089949	APCDD1L	0.367780761	76
chr20.fa	57090435	57194948	LOC149773	1.092197411	152
chr20.fa	57226328	57254582	STX16	11.38855265	3703
chr20.fa	57267862	57290900	NPEPL1	6.809968329	988
chr20.fa	57392670	57392749	MIR296	0	0
chr20.fa	57393281	57393368	MIR298	0	0
chr20.fa	57393973	57425958	GNAS-AS	0	0
chr20.fa	57414795	57486250	GNAS	125.1180509	46855
chr20.fa	57556311	57570188	TH1L	42.59946418	6384
chr20.fa	57570242	57582309	CTSZ	14.49842363	1444
chr20.fa	57594309	57601709	TUBB1	0.327418253	76
chr20.fa	57603733	57607400	ATP5E	332.1286179	9196
chr20.fa	57608200	57617901	SLMO2	24.56239323	4180
chr20.fa	57766075	57834167	ZNF831	0	0
chr20.fa	57875499	57901047	EDN3	0	0
chr20.fa	58152564	58422766	PHACTR3	0	0
chr20.fa	58438618	58507209	SYCP2	0.208891038	76
chr20.fa	58508819	58523702	C20orf177	4.22857511	1444
chr20.fa	58511887	58515352	PPP1R3D	8.217384876	1824
chr20.fa	58533471	58588168	CDH26	4.92768989	1119
chr20.fa	58630980	58648008	C20orf197	0	0
chr20.fa	59827559	60512299	CDH4	0	0
chr20.fa	60528602	60528718	MIR1257	0	0
chr20.fa	60549854	60640866	TAF4	5.193811349	1596
chr20.fa	60697517	60710434	LSM14B	14.12010042	2432
chr20.fa	60711791	60718474	PSMA7	86.07831896	5624
chr20.fa	60718822	60757566	SS18L1	5.285078811	1596
chr20.fa	60758081	60777810	GTPBP5	6.237664114	1216
chr20.fa	60790017	60795323	HRH3	0.854239343	152
chr20.fa	60813580	60871269	OSBPL2	34.58901245	9120
chr20.fa	60878027	60883918	ADRM1	30.14266053	2812
chr20.fa	60884121	60942368	LAMA5	0.901529744	684
chr20.fa	60962121	60963576	RPS21	556.9012486	14643
chr20.fa	60963686	60982339	CABLES2	3.624944771	911
chr20.fa	60985293	61002629	C20orf151	5.252397228	988

chr20.fa	61038553	61051026	GATA5	0.441126064	76
chr20.fa	61141438	61148768	C20orf200	0	0
chr20.fa	61147660	61167971	C20orf166	0	0
chr20.fa	61151513	61151583	MIR1-1	0	0
chr20.fa	61162119	61162195	MIR133A2	0	0
chr20.fa	61273797	61303647	SLCO4A1	0.471096732	76
chr20.fa	61294379	61297973	LOC100127888	1.062829168	76
chr20.fa	61340189	61394123	NTSR1	0	0
chr20.fa	61427805	61431945	C20orf20	5.597135214	608
chr20.fa	61436177	61445352	OGFR	11.29547791	1815
chr20.fa	61448414	61472466	COL9A3	0	0
chr20.fa	61472512	61493115	TCFL5	4.747414062	760
chr20.fa	61476476	61477543	DPH3P1	0	0
chr20.fa	61509090	61569304	DIDO1	11.7655204	10412
chr20.fa	61569441	61579827	C20orf11	31.54329979	9272
chr20.fa	61583999	61599949	SLC17A9	0	0
chr20.fa	61637331	61638387	BHLHE23	0	0
chr20.fa	61640735	61716423	LOC63930	0	0
chr20.fa	61665569	61668380	NCRNA00029	0	0
chr20.fa	61669363	61685215	LOC100144597	0	0
chr20.fa	61726845	61727535	HAR1B	0	0
chr20.fa	61732644	61735737	HAR1A	0	0
chr20.fa	61809852	61809938	MIR124-3	0	0
chr20.fa	61826782	61847538	YTHDF1	23.13283755	4995
chr20.fa	61867276	61871854	BIRC7	0	0
chr20.fa	61870131	61870194	MIR3196	0	0
chr20.fa	61872136	61881424	NKAIN4	0	0
chr20.fa	61885330	61892967	FLJ16779	0	0
chr20.fa	61904165	61921142	ARFGAP1	3.838655213	836
chr20.fa	61918160	61918218	MIR4326	0	0
chr20.fa	61924538	61962285	COL20A1	0	0
chr20.fa	61974665	61992695	CHRNA4	0.834359302	304
chr20.fa	62037542	62103993	KCNQ2	0	0
chr20.fa	62119366	62130505	EEF1A2	26.70552191	3268
chr20.fa	62152133	62153524	PPDPF	378.5899307	19532
chr20.fa	62159776	62168707	PTK6	14.14223956	2356
chr20.fa	62172163	62178857	SRMS	3.775099324	380
chr20.fa	62184373	62188035	C20orf195	2.060445777	152
chr20.fa	62189439	62205592	PRIC285	3.433072253	2280
chr20.fa	62218955	62258381	GMEB2	6.797016181	1900
chr20.fa	62271061	62284780	STMN3	2.537867977	380
chr20.fa	62289647	62326287	RTEL1	3.436837412	832
chr20.fa	62328021	62329994	TNFRSF6B	0	0

chr20.fa	62330036	62339355	ARFRP1	6.225464998	1064
chr20.fa	62338794	62367494	ZGPAT	11.13432909	1520
chr20.fa	62367978	62370460	LIME1	7.637098524	608
chr20.fa	62371211	62375403	SLC2A4RG	36.66361523	5548
chr20.fa	62375878	62436856	ZBTB46	1.569318398	456
chr20.fa	62492566	62494341	ABHD16B	2.577929272	304
chr20.fa	62496590	62522890	TPD52L2	40.77185585	6527
chr20.fa	62526455	62567384	DNAJC5	22.70616969	7980
chr20.fa	62550778	62550833	MIR941-1	0	0
chr20.fa	62550850	62551301	MIR941-2	0	0
chr20.fa	62550895	62551201	MIR941-3	0	0
chr20.fa	62571182	62582527	UCKL1	5.249987526	731
chr20.fa	62572818	62572897	MIR1914	0	0
chr20.fa	62573984	62574079	MIR647	0	0
chr20.fa	62584737	62588054	UCKL1-AS1	0	0
chr20.fa	62588339	62601218	ZNF512B	6.500020415	2432
chr20.fa	62605466	62610995	SAMD10	6.297756057	912
chr20.fa	62612431	62664453	PRPF6	27.31412226	5624
chr20.fa	62665697	62671315	NCRNA00176	0	0
chr20.fa	62679079	62680979	SOX18	0	0
chr20.fa	62688439	62703700	TCEA2	3.836847936	602
chr20.fa	62704535	62711324	RGS19	1.353800679	152
chr20.fa	62711471	62731996	OPRL1	0	0
chr20.fa	62714733	62715712	C20orf201	0	0
chr20.fa	62737183	62738184	NPBWR2	0	0
chr20.fa	62795827	62873606	MYT1	0	0
chr20.fa	62887048	62907579	PCMTD2	18.14053752	4712
chr21.fa	9825832	9826011	MIR3648	0	0
chr21.fa	9826203	9826263	MIR3687	0	0
chr21.fa	9907194	9968585	LOC100132288	2.863328347	304
chr21.fa	10906743	10990920	TPTE	0	0
chr21.fa	11057796	11098937	BAGE	0	0
chr21.fa	11097542	11097545	BAGE4	0	0
chr21.fa	14410487	14490571	ANKRD30BP2	0	0
chr21.fa	14778705	14778781	MIR3156-3	0	0
chr21.fa	14982498	15013906	POTED	0	0
chr21.fa	15215454	15220685	C21orf15	0	0
chr21.fa	15316096	15352765	C21orf81	0	0
chr21.fa	15481135	15579254	LIPI	0	0
chr21.fa	15588466	15600693	RBM11	1.745678459	228
chr21.fa	15646120	15673692	ABCC13	0	0
chr21.fa	15743437	15755509	HSPA13	2.584405346	684
chr21.fa	15857549	15918664	SAMSN1	0.606190648	76

chr21.fa	16333556	16437126	NRIP1	21.81367633	10944
chr21.fa	17102496	17252377	USP25	14.74541808	4864
chr21.fa	17442842	17982094	C21orf34	3.561087669	899
chr21.fa	17911409	17911489	MIR99A	0	0
chr21.fa	17912148	17912231	MIRLET7C	0	0
chr21.fa	17962557	17962645	MIR125B2	0	0
chr21.fa	18811208	18821503	C21orf37	1.596427545	76
chr21.fa	18885330	18939266	CXADR	6.019435481	988
chr21.fa	18965968	18985268	BTG3	3.590305305	380
chr21.fa	19161284	19191703	C21orf91	2.317380248	836
chr21.fa	19207989	19257925	NCRNA00157	0	0
chr21.fa	19617150	19639687	CHODL	0	0
chr21.fa	19641433	19775970	TMPRSS15	0	0
chr21.fa	22114913	22175426	C21orf131	0	0
chr21.fa	22370633	22912517	NCAM2	0.228771079	76
chr21.fa	26758133	26804013	NCRNA00158	1.55094442	152
chr21.fa	26934457	26947480	MIR155HG	0	0
chr21.fa	26955087	26955536	C21orf71	0	0
chr21.fa	26957968	26979801	MRPL39	10.66202751	829
chr21.fa	27011589	27087235	JAM2	0	0
chr21.fa	27096791	27107965	ATP5J	57.09502628	4553
chr21.fa	27113884	27144771	GABPA	2.99646438	919
chr21.fa	27252861	27543138	APP	21.10898912	5092
chr21.fa	27838528	27945581	CYYR1	0	0
chr21.fa	28208606	28217728	ADAMTS1	15.25823277	4712
chr21.fa	28290231	28339439	ADAMTS5	0.355431038	228
chr21.fa	29094698	29123552	NCRNA00113	0	0
chr21.fa	29385682	29395528	C21orf94	0	0
chr21.fa	29911640	29912677	NCRNA00161	7.794030364	266
chr21.fa	30244513	30257693	N6AMT1	2.823267052	912
chr21.fa	30300466	30365277	LTN1	10.47798652	5396
chr21.fa	30378080	30391685	RWDD2B	18.74401726	2122
chr21.fa	30396938	30426807	USP16	19.60458207	3952
chr21.fa	30428648	30446010	CCT8	72.48820236	9116
chr21.fa	30452873	30548202	C21orf7	2.294939898	304
chr21.fa	30565801	30660526	NCRNA00189	0	0
chr21.fa	30671220	30734217	BACH1	30.43905387	13604
chr21.fa	30909254	31312282	GRIK1	0	0
chr21.fa	30968360	31003067	NCRNA00258	0	0
chr21.fa	31120494	31136325	GRIK1-AS1	0	0
chr21.fa	31538241	31538971	CLDN17	0	0
chr21.fa	31586324	31588469	CLDN8	2.666787031	380
chr21.fa	31653627	31655276	KRTAP24-1	0	0

chr21.fa	31661463	31661832	KRTAP25-1	0	0
chr21.fa	31691450	31692607	KRTAP26-1	0	0
chr21.fa	31709331	31710012	KRTAP27-1	0	0
chr21.fa	31720717	31720924	KRTAP23-1	0	0
chr21.fa	31743709	31744557	KRTAP13-2	0	0
chr21.fa	31747612	31747696	MIR4327	0	0
chr21.fa	31768392	31769138	KRTAP13-1	0	0
chr21.fa	31797711	31798230	KRTAP13-3	0	0
chr21.fa	31802594	31803076	KRTAP13-4	0	0
chr21.fa	31812646	31813098	KRTAP15-1	0	0
chr21.fa	31852364	31852636	KRTAP19-1	0	0
chr21.fa	31859509	31859667	KRTAP19-2	0	0
chr21.fa	31863782	31864275	KRTAP19-3	0	0
chr21.fa	31869174	31869428	KRTAP19-4	0	0
chr21.fa	31874190	31874408	KRTAP19-5	0	0
chr21.fa	31913854	31914181	KRTAP19-6	0	0
chr21.fa	31933417	31933608	KRTAP19-7	0	0
chr21.fa	31962424	31962716	KRTAP22-2	0	0
chr21.fa	31964759	31965374	KRTAP6-3	0	0
chr21.fa	31971005	31971193	KRTAP6-2	0	0
chr21.fa	31973440	31973586	KRTAP22-1	0	0
chr21.fa	31986005	31986223	KRTAP6-1	0	0
chr21.fa	31988774	31988944	KRTAP20-1	0	0
chr21.fa	31992946	31993169	KRTAP20-4	0	0
chr21.fa	32007583	32007780	KRTAP20-2	0	0
chr21.fa	32015183	32015455	KRTAP20-3	0	0
chr21.fa	32090843	32091095	KRTAP21-3	0	0
chr21.fa	32119269	32119520	KRTAP21-2	0	0
chr21.fa	32127457	32127696	KRTAP21-1	0	0
chr21.fa	32185015	32185570	KRTAP8-1	0	0
chr21.fa	32201358	32202051	KRTAP7-1	0	0
chr21.fa	32252964	32253874	KRTAP11-1	0	0
chr21.fa	32410478	32410795	KRTAP19-8	0	0
chr21.fa	32490736	32931290	TIAM1	41.18557155	19684
chr21.fa	33031935	33041243	SOD1	190.9657171	12236
chr21.fa	33043313	33104431	SFRS15	14.74662293	4180
chr21.fa	33245628	33376377	HUNK	1.549890176	760
chr21.fa	33452629	33528816	NCRNA00159	0	0
chr21.fa	33640530	33651376	C21orf45	0.728633628	76
chr21.fa	33664124	33679050	MRAP	0	0
chr21.fa	33683330	33765312	URB1	10.10282605	6688
chr21.fa	33749496	33749631	SNORA80	0	0
chr21.fa	33765442	33766266	C21orf119	6.937080107	380

chr21.fa	33784752	33887697	C21orf63	3.552804319	456
chr21.fa	33947151	33957845	TCP10L	0	0
chr21.fa	33973984	33984918	C21orf59	27.27165126	2584
chr21.fa	34001069	34100351	SYNJ1	9.853572503	4712
chr21.fa	34106210	34144169	GCFC1	8.792400005	2508
chr21.fa	34144411	34170016	C21orf49	0	0
chr21.fa	34162984	34186053	C21orf62	0.5554363	152
chr21.fa	34398239	34401500	OLIG2	0	0
chr21.fa	34442450	34444728	OLIG1	0	0
chr21.fa	34537776	34542541	C21orf54	0	0
chr21.fa	34602231	34636820	IFNAR2	1.46088181	304
chr21.fa	34638672	34669520	IL10RB	0.59158183	76
chr21.fa	34697214	34732128	IFNAR1	5.634786807	2280
chr21.fa	34775202	34809828	IFNGR2	28.85994606	4256
chr21.fa	34821448	34852281	TMEM50B	8.247204938	1277
chr21.fa	34860238	34864023	DNAJC28	0	0
chr21.fa	34876238	34915198	GART	14.97900856	4403
chr21.fa	34915350	34949812	SON	52.11251567	31844
chr21.fa	34950211	34961014	DONSON	2.109844667	304
chr21.fa	34961648	35014160	CRYZL1	15.17509806	1723
chr21.fa	35014784	35261609	ITSN1	5.710993631	3015
chr21.fa	35275757	35288158	ATP5O	126.240219	6756
chr21.fa	35445823	35515334	MRPS6	20.37071668	1101
chr21.fa	35467162	35478561	SLC5A3	1.405609271	1064
chr21.fa	35552978	35562220	C21orf82	0	0
chr21.fa	35736323	35743440	KCNE2	0	0
chr21.fa	35747749	35761452	FAM165B	11.44608428	684
chr21.fa	35818988	35883613	KCNE1	0	0
chr21.fa	35888784	35987382	RCAN1	8.686222513	1672
chr21.fa	36041688	36090519	CLIC6	34.0370401	8588
chr21.fa	36096105	36109479	NCRNA00160	0	0
chr21.fa	36160098	36421595	RUNX1	9.465459882	5016
chr21.fa	36410233	36411723	C21orf96	0	0
chr21.fa	37093013	37093106	MIR802	0	0
chr21.fa	37406839	37432816	SETD4	6.250616262	1292
chr21.fa	37442285	37445462	CBR1	54.24254159	4484
chr21.fa	37507263	37518860	CBR3	7.205310055	532
chr21.fa	37536839	37666572	DOPEY2	21.31125348	10866
chr21.fa	37692487	37748944	MORC3	39.03491256	11005
chr21.fa	37757689	37789125	CHAF1B	2.511210649	380
chr21.fa	37832920	37948867	CLDN14	0	0
chr21.fa	38071991	38122510	SIM2	3.288339529	912
chr21.fa	38123189	38362536	HLCS	18.66419588	7448

chr21.fa	38378863	38391958	DSCR6	0.512362878	76
chr21.fa	38437664	38445458	PIGP	10.1867138	836
chr21.fa	38445571	38575408	TTC3	51.22363686	30954
chr21.fa	38580955	38592893	DSCR9	0	0
chr21.fa	38595726	38639833	DSCR3	21.12480279	4560
chr21.fa	38739859	38887679	DYRK1A	23.80378893	10716
chr21.fa	38996786	39288696	KCNJ6	0	0
chr21.fa	39426313	39493454	DSCR4	0	0
chr21.fa	39493545	39528605	DSCR8	0	0
chr21.fa	39578250	39580738	DSCR10	0	0
chr21.fa	39628664	39673746	KCNJ15	15.19061051	3040
chr21.fa	39751950	40033704	ERG	0	0
chr21.fa	40110879	40145401	NCRNA00114	0	0
chr21.fa	40177849	40196878	ETS2	3.429758912	836
chr21.fa	40547384	40555440	PSMG1	9.132469193	684
chr21.fa	40557404	40685556	BRWD1	9.807788166	11018
chr21.fa	40685861	40686888	NCRNA00257	0	0
chr21.fa	40714241	40721047	HMGN1	52.37728167	4535
chr21.fa	40752213	40769815	WRB	8.947675175	988
chr21.fa	40777770	40816128	LCA5L	0.484199486	76
chr21.fa	40817797	40887433	SH3BGR	1.780167319	152
chr21.fa	40969075	40984749	C21orf88	1.044304584	152
chr21.fa	41029254	41034815	B3GALT5	2.392081009	456
chr21.fa	41117334	41174023	IGSF5	3.878114082	532
chr21.fa	41239347	41301322	PCP4	2.058638501	76
chr21.fa	41384343	42219039	DSCAM	0	0
chr21.fa	42513427	42519991	C21orf130	0.566882385	76
chr21.fa	42539484	42539556	MIR3197	0	0
chr21.fa	42539728	42648524	BACE2	8.030934187	1596
chr21.fa	42547158	42557166	PLAC4	0	0
chr21.fa	42688661	42729654	FAM3B	20.5886441	1892
chr21.fa	42733950	42780869	MX2	1.93288218	380
chr21.fa	42792520	42831141	MX1	9.525100005	2280
chr21.fa	42836478	42880085	TMPRSS2	191.6375722	42245
chr21.fa	43099462	43117496	NCRNA00111	0	0
chr21.fa	43131680	43135935	C21orf129	0	0
chr21.fa	43136596	43137742	NCRNA00112	0	0
chr21.fa	43159529	43187249	RIPK4	12.71132841	3268
chr21.fa	43218385	43299582	PRDM15	2.097193732	1064
chr21.fa	43305219	43373999	C2CD2	6.549720517	2812
chr21.fa	43406940	43430496	ZNF295	6.121697208	3040
chr21.fa	43442113	43445060	C21orf121	0	0
chr21.fa	43483068	43563105	UMODL1	0.218529846	76

chr21.fa	43522244	43528644	C21orf128	0	0
chr21.fa	43619799	43717354	ABCG1	1.924297617	456
chr21.fa	43731777	43735706	TFF3	3.257917042	228
chr21.fa	43766467	43771208	TFF2	0	0
chr21.fa	43782391	43786644	TFF1	4.652833261	152
chr21.fa	43791996	43816200	TMPRSS3	0.366425303	64
chr21.fa	43824019	43867774	UBASH3A	0	0
chr21.fa	43892597	43916401	RSPH1	3.351744812	304
chr21.fa	43919742	44001550	SLC37A1	19.16405843	3937
chr21.fa	44073862	44195618	PDE9A	50.15900042	7377
chr21.fa	44263204	44299678	WDR4	5.91280617	912
chr21.fa	44313378	44329773	NDUFV3	16.12120729	2280
chr21.fa	44394643	44453688	PKNOX1	6.900783971	2280
chr21.fa	44473301	44496472	CBS	15.00069588	2504
chr21.fa	44513066	44527688	U2AF1	49.37524486	3344
chr21.fa	44589141	44592913	CRYAA	10.27481853	760
chr21.fa	44834398	44847002	SIK1	10.95209538	3420
chr21.fa	44869904	44873771	C21orf125	0	0
chr21.fa	44881974	44898103	C21orf84	0	0
chr21.fa	44949072	45079374	HSF2BP	0	0
chr21.fa	45079432	45115960	RRP1B	9.124637662	3082
chr21.fa	45138978	45182188	PDXK	28.63780166	14035
chr21.fa	45193831	45196259	CSTB	219.1803149	9576
chr21.fa	45209418	45223983	RRP1	8.809719738	1057
chr21.fa	45225639	45232448	LOC284837	1.742515726	532
chr21.fa	45285116	45407475	AGPAT3	10.73778251	4712
chr21.fa	45432206	45526432	TRAPPC10	17.7382679	8208
chr21.fa	45527208	45551063	PWP2	11.93766348	2584
chr21.fa	45553494	45565605	C21orf33	80.10963782	8867
chr21.fa	45646722	45660834	ICOSLG	1.060419466	228
chr21.fa	45666223	45682099	DNMT3L	0.672457452	76
chr21.fa	45705763	45718110	AIRE	0	0
chr21.fa	45719925	45747256	PFKL	54.43742624	12275
chr21.fa	45748827	45759285	C21orf2	10.04936079	1490
chr21.fa	45773484	45862964	TRPM2	0	0
chr21.fa	45875393	45878739	LRRC3	0.913879466	152
chr21.fa	45917775	46131495	C21orf29	0	0
chr21.fa	45937098	45938860	C21orf90	1.095360144	76
chr21.fa	45959068	45960078	KRTAP10-1	0	0
chr21.fa	45970318	45971388	KRTAP10-2	0	0
chr21.fa	45977906	45978643	KRTAP10-3	0	0
chr21.fa	45993606	45994987	KRTAP10-4	0	0
chr21.fa	45999564	46000481	KRTAP10-5	0	0

chr21.fa	46011149	46012386	KRTAP10-6	0	0
chr21.fa	46020497	46022091	KRTAP10-7	0	0
chr21.fa	46031996	46032871	KRTAP10-8	0	0
chr21.fa	46047040	46048295	KRTAP10-9	0	0
chr21.fa	46057273	46058372	KRTAP10-10	0	0
chr21.fa	46066331	46067566	KRTAP10-11	0	0
chr21.fa	46074130	46074576	KRTAP12-4	0	0
chr21.fa	46077849	46078258	KRTAP12-3	0	0
chr21.fa	46086106	46086844	KRTAP12-2	0	0
chr21.fa	46101491	46102078	KRTAP12-1	0	0
chr21.fa	46117087	46117959	KRTAP10-12	0	0
chr21.fa	46188955	46221738	UBE2G2	20.07914274	4033
chr21.fa	46225532	46238044	SUMO3	84.87964284	10184
chr21.fa	46269500	46293818	PTTG1IP	18.63121308	3496
chr21.fa	46305868	46348753	ITGB2	0	0
chr21.fa	46353199	46359828	C21orf67	2.619195418	380
chr21.fa	46359955	46396888	C21orf70	2.598411738	152
chr21.fa	46409779	46414001	NCRNA00163	0	0
chr21.fa	46419127	46424642	NCRNA00162	0	0
chr21.fa	46490871	46493126	C21orf122	0.507392868	76
chr21.fa	46494493	46646478	ADARB1	8.647817888	2128
chr21.fa	46683843	46707811	POFUT2	1.898543927	608
chr21.fa	46707967	46717269	LOC642852	0.639022837	304
chr21.fa	46825097	46933634	COL18A1	2.743746887	1102
chr21.fa	46839631	46844985	NCRNA00175	0	0
chr21.fa	46934629	46962351	SLC19A1	1.613295458	304
chr21.fa	47269875	47362368	PCBP3	0	0
chr21.fa	47401663	47424963	COL6A1	0	0
chr21.fa	47518033	47552763	COL6A2	0.263561151	76
chr21.fa	47556176	47575481	FTCD	5.224836262	732
chr21.fa	47581062	47604373	C21orf56	11.36701594	1748
chr21.fa	47608360	47648738	LSS	37.87253258	11653
chr21.fa	47649158	47671604	MCM3AP-AS1	0	0
chr21.fa	47655048	47705236	MCM3AP	25.71648986	9861
chr21.fa	47706267	47717665	YBEY	6.258899613	608
chr21.fa	47721047	47743785	C21orf58	0.385702919	76
chr21.fa	47744036	47865682	PCNT	16.58387006	11628
chr21.fa	47878862	47989926	DIP2A	11.5694309	5690
chr21.fa	48018531	48025035	S100B	0	0
chr21.fa	48055527	48084863	PRMT2	28.6152107	4256
chr22.fa	16256332	16287937	POTEH	2.809561872	380
chr22.fa	16448824	16449804	OR11H1	0	0
chr22.fa	17071648	17073700	CCT8L2	0	0

chr22.fa	17082801	17129720	psiTPTE22	0.800472868	76
chr22.fa	17264306	17302584	XKR3	0	0
chr22.fa	17308364	17310225	HSFY1P1	0	0
chr22.fa	17442827	17489112	GAB4	0	0
chr22.fa	17517460	17539682	CECR7	0	0
chr22.fa	17565851	17596584	IL17RA	0.531941706	304
chr22.fa	17597189	17602257	CECR6	2.032282386	684
chr22.fa	17618410	17646158	CECR5	14.76123175	1824
chr22.fa	17640279	17646335	CECR4	3.771334164	152
chr22.fa	17660192	17690779	CECR1	1.417958994	380
chr22.fa	17956628	18033845	CECR2	3.505965737	1312
chr22.fa	18043183	18073647	SLC25A18	1.645374616	228
chr22.fa	18074903	18111588	ATP6V1E1	87.75908607	8187
chr22.fa	18121485	18211989	BCL2L13	31.29389564	6992
chr22.fa	18216906	18257258	BID	9.237743047	1596
chr22.fa	18246946	18247025	MIR3198	0	0
chr22.fa	18270416	18507325	MICAL3	3.563647977	3496
chr22.fa	18463634	18463727	MIR648	0	0
chr22.fa	18512151	18520734	FLJ41941	0	0
chr22.fa	18560686	18573797	PEX26	16.64983565	4788
chr22.fa	18593453	18614498	TUBA8	0	0
chr22.fa	18632758	18660162	USP18	2.812272787	380
chr22.fa	18761202	18779474	GGT3P	0.562213587	76
chr22.fa	18893736	18899601	DGCR6	2.828538275	228
chr22.fa	18900287	18923964	PRODH	0.496398603	76
chr22.fa	18958027	18982141	DGCR5	0	0
chr22.fa	19005347	19007761	DGCR9	0	0
chr22.fa	19010137	19011063	DGCR10	0	0
chr22.fa	19023795	19109967	DGCR2	4.594699201	1368
chr22.fa	19033675	19035888	DGCR11	0	0
chr22.fa	19117792	19132190	DGCR14	5.131309705	1216
chr22.fa	19136504	19137796	GSC2	0	0
chr22.fa	19163094	19166301	SLC25A1	54.75641053	6308
chr22.fa	19166987	19279239	CLTCL1	0.415221768	152
chr22.fa	19318224	19419219	HIRA	15.116964	4028
chr22.fa	19420036	19423596	MRPL40	42.8859175	2204
chr22.fa	19428410	19435755	C22orf39	9.041804157	1672
chr22.fa	19437464	19466738	UFD1L	19.29252566	2284
chr22.fa	19467414	19508135	CDC45	0	0
chr22.fa	19510547	19512860	CLDN5	1.345065509	152
chr22.fa	19553653	19554362	LOC150185	0	0
chr22.fa	19701987	19702314	5-Sep	6.73105059	80
chr22.fa	19704743	19711065	SEPT5-GP1BB	0.744146085	76

chr22.fa	19744226	19771112	TBX1	1.224580412	228
chr22.fa	19775934	19842462	GNB1L	0.821708366	76
chr22.fa	19833661	19842342	C22orf29	13.21646219	5712
chr22.fa	19863040	19929262	TXNRD2	20.05399148	2486
chr22.fa	19929360	19957401	COMT	32.02373412	5016
chr22.fa	19957499	20004309	ARVCF	2.615279652	684
chr22.fa	20008631	20053447	C22orf25	9.072226644	1368
chr22.fa	20020662	20020743	MIR185	0	0
chr22.fa	20067755	20099397	DGCR8	8.95866944	2584
chr22.fa	20099401	20104768	TRMT2A	4.338668368	836
chr22.fa	20105024	20114704	RANBP1	27.19092624	1596
chr22.fa	20119364	20135530	ZDHHC8	0.913427647	304
chr22.fa	20193855	20196060	LOC150197	0	0
chr22.fa	20228938	20255816	RTN4R	1.789354307	228
chr22.fa	20236657	20236734	MIR1286	0	0
chr22.fa	20301761	20307628	DGCR6L	20.69135765	1672
chr22.fa	20383731	20398695	PI4KAP1	1.455008161	228
chr22.fa	20455994	20461786	RIMBP3	0	0
chr22.fa	20748480	20762752	ZNF74	1.775498521	456
chr22.fa	20778874	20792146	SCARF2	0.326213402	76
chr22.fa	20795806	20850170	KLHL22	8.313321135	1444
chr22.fa	20861886	20941919	MED15	14.89994021	3339
chr22.fa	21043843	21046009	POM121L4P	0	0
chr22.fa	21055402	21058891	TMEM191A	0	0
chr22.fa	21061979	21213070	PI4KA	24.68137226	11016
chr22.fa	21128383	21142008	SERPIND1	0	0
chr22.fa	21213292	21245501	SNAP29	15.5802292	4408
chr22.fa	21271714	21308037	CRKL	28.37333687	10032
chr22.fa	21319418	21335649	AIFM3	0	0
chr22.fa	21336558	21353326	LZTR1	7.427303848	2128
chr22.fa	21354061	21356404	THAP7	8.494349994	608
chr22.fa	21356405	21364663	FLJ39582	0.575165735	76
chr22.fa	21362496	21368576	MGC16703	0	0
chr22.fa	21369442	21382302	P2RX6	0	0
chr22.fa	21383007	21386847	SLC7A4	0	0
chr22.fa	21396681	21398538	P2RX6P	0	0
chr22.fa	21400249	21418457	LOC400891	0	0
chr22.fa	21457305	21476575	BCRP2	0	0
chr22.fa	21636714	21652015	POM121L8P	0	0
chr22.fa	21737663	21738039	RIMBP3C	0	0
chr22.fa	21771693	21805750	HIC2	2.526421892	1140
chr22.fa	21827287	21871780	PI4KAP2	0	0
chr22.fa	21921957	21978323	UBE2L3	42.50533519	8512

chr22.fa	21982378	21984340	YDJC	9.410036737	836
chr22.fa	21987086	21991616	CCDC116	0	0
chr22.fa	21996542	21998588	SDF2L1	6.548063847	380
chr22.fa	22007270	22007347	MIR301B	0	0
chr22.fa	22007593	22007674	MIR130B	0	0
chr22.fa	22020273	22051825	PPIL2	17.16310217	4245
chr22.fa	22052203	22090071	YPEL1	0.878938788	228
chr22.fa	22113947	22221970	MAPK1	44.61713774	18012
chr22.fa	22273792	22307250	PPM1F	4.194387464	1444
chr22.fa	22311403	22337147	TOP3B	1.841915931	380
chr22.fa	22599200	22599926	VPREB1	0	0
chr22.fa	22652463	22677324	LOC96610	6.830149582	912
chr22.fa	22838772	22863505	ZNF280B	0.599413361	228
chr22.fa	22868061	22874613	ZNF280A	0	0
chr22.fa	22890123	22901696	PRAME	0	0
chr22.fa	22901756	22909007	LOC648691	0	0
chr22.fa	22974028	22987012	POM121L1P	0	0
chr22.fa	22988782	22990368	GGTLC2	0	0
chr22.fa	23165270	23165365	MIR650	0	0
chr22.fa	23229960	23238013	IGLL5	0	0
chr22.fa	23401593	23484241	RTDR1	0	0
chr22.fa	23412669	23467221	GNAZ	0.713572991	152
chr22.fa	23487513	23506531	RAB36	4.791692336	1216
chr22.fa	23522552	23660224	BCR	15.35627752	7063
chr22.fa	23604954	23607186	FBXW4P1	1.025177575	152
chr22.fa	23732792	23744799	ZDHHC8P1	27.85148579	4712
chr22.fa	23915313	23922495	IGLL1	0	0
chr22.fa	23950639	23974508	C22orf43	0	0
chr22.fa	23980675	24059610	LOC91316	1.059214615	228
chr22.fa	24033048	24041363	RGL4	0	0
chr22.fa	24083772	24093279	ZNF70	3.419367073	912
chr22.fa	24094930	24096630	VPREB3	0	0
chr22.fa	24105208	24108020	C22orf15	0	0
chr22.fa	24108051	24110141	CHCHD10	31.59962657	1368
chr22.fa	24115036	24126503	MMP11	0	0
chr22.fa	24129150	24176689	SMARCB1	24.42549204	2736
chr22.fa	24176706	24181199	DERL3	0.35949741	76
chr22.fa	24199059	24227725	SLC2A11	3.122521913	532
chr22.fa	24236565	24237409	MIF	259.1181126	9652
chr22.fa	24309026	24314748	DDTL	0	0
chr22.fa	24315957	24322019	DDT	5.550296632	171
chr22.fa	24322314	24326106	GSTT2	1.022918479	76
chr22.fa	24340595	24347258	GSTTP1	0	0

chr22.fa	24373117	24374043	LOC391322	0	0
chr22.fa	24376139	24384284	GSTT1	125.2590185	8816
chr22.fa	24385938	24401899	GSTTP2	0	0
chr22.fa	24407765	24574596	CABIN1	13.58861053	6840
chr22.fa	24577444	24585074	SUSD2	2.157586887	456
chr22.fa	24615622	24641110	GGT5	0	0
chr22.fa	24647589	24661492	POM121L9P	0	0
chr22.fa	24666786	24813708	SPECC1L	37.15157988	16720
chr22.fa	24823530	24838325	ADORA2A	0.473506434	76
chr22.fa	24825178	24890783	C22orf45	0.633751614	152
chr22.fa	24891251	24922553	UPB1	3.876306806	532
chr22.fa	24936406	24951275	C22orf13	54.26332527	12996
chr22.fa	24951618	24968509	SNRPD3	62.80888143	5776
chr22.fa	24979718	25024972	GGT1	6.598366376	1140
chr22.fa	24981591	24989035	C22orf36	10.153731	836
chr22.fa	25028882	25049017	BCRP3	0	0
chr22.fa	25041133	25055114	POM121L10P	0	0
chr22.fa	25115001	25170683	PIWIL3	0	0
chr22.fa	25160468	25161986	TOP1P2	0	0
chr22.fa	25202136	25322813	SGSM1	6.301220003	2584
chr22.fa	25331208	25335314	TMEM211	0	0
chr22.fa	25423941	25593415	KIAA1671	17.12439633	11932
chr22.fa	25595825	25603324	CRYBB3	0	0
chr22.fa	25615612	25627836	CRYBB2	0	0
chr22.fa	25714224	25716193	IGLL3P	0	0
chr22.fa	25747385	25777544	LRP5L	3.43939772	380
chr22.fa	25844054	25857645	CRYBB2P1	6.100461709	608
chr22.fa	25960861	26125258	ADRBK2	27.80103266	16715
chr22.fa	26138120	26427007	MYO18B	0	0
chr22.fa	26565440	26779563	SEZ6L	0	0
chr22.fa	26825280	26840978	ASPHD2	0.345491018	76
chr22.fa	26847446	26879820	HPS4	11.99097814	3797
chr22.fa	26879850	26887893	SRRD	13.58047779	1156
chr22.fa	26887905	26908437	TFIP11	10.99456638	2128
chr22.fa	26921714	26986089	TPST2	1.654862817	228
chr22.fa	26951178	26951289	MIR548J	0	0
chr22.fa	26995362	27013991	CRYBB1	0	0
chr22.fa	27017928	27026636	CRYBA4	0	0
chr22.fa	27053446	27072440	MIAT	0.112352354	76
chr22.fa	28144265	28197486	MN1	1.666308901	836
chr22.fa	28247657	28315255	PITPNB	23.16506731	4499
chr22.fa	28315364	28398667	TTC28-AS1	0.211601953	76
chr22.fa	28316513	28316600	MIR3199-1	0	0

chr22.fa	28374002	29075853	TTC28	4.932358687	3706
chr22.fa	29083731	29137822	CHEK2	1.72820812	228
chr22.fa	29138043	29153496	HSCB	5.260078153	380
chr22.fa	29168662	29185283	CCDC117	14.06287	3721
chr22.fa	29190548	29196560	XBP1	50.50434083	6016
chr22.fa	29279890	29453475	ZNRF3	1.399886229	608
chr22.fa	29454660	29457907	C22orf31	0	0
chr22.fa	29469066	29564321	KREMEN1	5.393214186	988
chr22.fa	29601953	29655586	EMID1	0.555586907	76
chr22.fa	29655844	29663914	RHBDD3	1.317203331	152
chr22.fa	29663998	29696515	EWSR1	31.81002368	6235
chr22.fa	29702997	29708774	GAS2L1	8.153075955	1368
chr22.fa	29708922	29711748	RASL10A	0.640528901	76
chr22.fa	29723669	29784572	AP1B1	89.31123534	24776
chr22.fa	29729147	29729256	MIR3653	0	0
chr22.fa	29833004	29837530	RFPL1S	0	0
chr22.fa	29838119	29838444	RFPL1	0	0
chr22.fa	29876181	29887277	NEFH	484.1271953	119548
chr22.fa	29904156	29949736	THOC5	13.15757509	2406
chr22.fa	29950798	29977144	NIPSNAP1	47.7336354	6510
chr22.fa	29999545	30094589	NF2	18.38211014	7676
chr22.fa	30116344	30127820	CABP7	0	0
chr22.fa	30134319	30162969	ZMAT5	14.44300048	608
chr22.fa	30163358	30166402	UQCR10	94.63863454	5756
chr22.fa	30184601	30234254	ASCC2	53.08603526	9799
chr22.fa	30279158	30426857	MTMR3	27.41276943	16416
chr22.fa	30476453	30573062	HORMAD2	0	0
chr22.fa	30636442	30642796	LIF	0	0
chr22.fa	30658819	30662829	OSM	0	0
chr22.fa	30681107	30685616	GATSL3	5.645479859	608
chr22.fa	30687979	30722894	TBC1D10A	13.59809873	1748
chr22.fa	30727977	30749061	SF3A1	28.72560518	9159
chr22.fa	30752627	30772818	CCDC157	0.815232292	152
chr22.fa	30774803	30783302	RNF215	1.788450669	228
chr22.fa	30792933	30821278	SEC14L2	29.54234353	9492
chr22.fa	30821720	30825041	MTFP1	13.70789078	1064
chr22.fa	30855216	30868034	SEC14L3	0	0
chr22.fa	30877277	30877743	SDC4P	0	0
chr22.fa	30884898	30901698	SEC14L4	0	0
chr22.fa	30920917	30942669	LOC730005	0	0
chr22.fa	30950624	30960876	GAL3ST1	1.917219117	228
chr22.fa	30972612	31003000	PES1	9.022375935	1672
chr22.fa	31003070	31023047	TCN2	2.762120865	380

chr22.fa	31031793	31043862	SLC35E4	0	0
chr22.fa	31058039	31063872	DUSP18	1.86646477	304
chr22.fa	31090793	31303811	OSBP2	3.692265819	1064
chr22.fa	31127544	31127628	MIR3200	0	0
chr22.fa	31318295	31322599	C22orf27	3.829920043	532
chr22.fa	31322641	31364187	MORC2	16.41157637	4823
chr22.fa	31365634	31375380	TUG1	8.5393813	4028
chr22.fa	31477305	31500609	SMTN	1.789504914	380
chr22.fa	31500763	31503551	SELM	30.55712927	1402
chr22.fa	31518961	31530683	INPP5J	9.218314825	1368
chr22.fa	31530793	31536469	PLA2G3	0.444439404	76
chr22.fa	31556048	31556105	MIR3928	0	0
chr22.fa	31556138	31603005	RNF185	30.63996277	6734
chr22.fa	31608250	31676066	LIMK2	18.44385876	6073
chr22.fa	31677579	31688520	PIK3IP1	7.405465924	1216
chr22.fa	31721790	31742249	PATZ1	10.11653123	3192
chr22.fa	31795539	31830172	DRG1	22.10284057	2128
chr22.fa	31835345	31885874	EIF4ENIF1	13.89373904	3572
chr22.fa	31892261	32014476	SFI1	3.274935562	912
chr22.fa	32014535	32026810	PISD	4.803740845	836
chr22.fa	32077334	32146120	C22orf30	22.83298026	16416
chr22.fa	32150069	32303001	DEPDC5	3.581268923	1431
chr22.fa	32329507	32341336	C22orf24	0	0
chr22.fa	32340479	32353590	YWHAH	21.06636751	2508
chr22.fa	32439019	32509011	SLC5A1	0	0
chr22.fa	32545519	32555243	C22orf42	10.68281119	571
chr22.fa	32586422	32600718	RFPL2	1.681971964	304
chr22.fa	32614463	32651318	SLC5A4	0	0
chr22.fa	32750872	32757148	RFPL3	0	0
chr22.fa	32755893	32766972	RFPL3S	0	0
chr22.fa	32783562	32808274	C22orf28	84.75795289	11627
chr22.fa	32809834	32853373	BPIL2	0	0
chr22.fa	32870707	32894818	FBXO7	63.80122682	9807
chr22.fa	32908540	33454377	SYN3	0	0
chr22.fa	33196802	33259028	TIMP3	0.208589825	76
chr22.fa	33669062	34316416	LARGE	3.269513733	912
chr22.fa	35462130	35483380	ISX	0.338412518	76
chr22.fa	35653445	35691800	HMGXB4	11.03944708	3116
chr22.fa	35695268	35743987	TOM1	13.61541847	2280
chr22.fa	35731633	35731751	MIR3909	0	0
chr22.fa	35777060	35790207	HMOX1	8.638480293	912
chr22.fa	35796116	35820495	MCM5	3.147070752	532
chr22.fa	35937352	35950045	RASD2	0	0

chr22.fa	36002811	36019401	MB	13.18483485	1216
chr22.fa	36044424	36064456	APOL6	6.434807856	4332
chr22.fa	36113919	36125529	APOL5	0	0
chr22.fa	36134783	36424585	RBFOX2	15.85312794	7702
chr22.fa	36536371	36562225	APOL3	3.735791061	988
chr22.fa	36585176	36600879	APOL4	9.966979101	2260
chr22.fa	36622255	36636000	APOL2	18.32397608	3268
chr22.fa	36649117	36663577	APOL1	1.49823219	304
chr22.fa	36677323	36784063	MYH9	104.1441557	51897
chr22.fa	36863093	36877687	TXN2	77.96364763	6916
chr22.fa	36883233	36903148	FOXRED2	2.936071225	988
chr22.fa	36906897	36925277	EIF3D	129.2865341	16731
chr22.fa	36956916	37098690	CACNG2	0	0
chr22.fa	37154246	37172172	IFT27	10.88914192	1052
chr22.fa	37196745	37215517	PVALB	12.00633999	456
chr22.fa	37257030	37274059	NCF4	4.179929252	456
chr22.fa	37309675	37336479	CSF2RB	0.472452189	152
chr22.fa	37387160	37403877	C22orf33	0	0
chr22.fa	37406906	37415491	TST	30.44176479	2280
chr22.fa	37415702	37425863	MPST	26.23954579	2812
chr22.fa	37447779	37459430	KCTD17	10.15327918	1140
chr22.fa	37461479	37499693	TMPRSS6	0	0
chr22.fa	37521880	37545962	IL2RB	0	0
chr22.fa	37576206	37584330	C1QTNF6	3.118756754	608
chr22.fa	37602245	37608353	SSTR3	0	0
chr22.fa	37621310	37640305	RAC2	5.457974926	532
chr22.fa	37678495	37711389	CYTH4	0	0
chr22.fa	37764000	37823505	ELFN2	0.136901192	76
chr22.fa	37865101	37882478	MFNG	0	0
chr22.fa	37886400	37915210	CARD10	3.229151225	836
chr22.fa	37956471	37965410	CDC42EP1	4.235352397	608
chr22.fa	37966253	37976024	LGALS2	34.68509932	1216
chr22.fa	38004481	38029571	GGA1	10.42271398	2508
chr22.fa	38035684	38052050	SH3BP1	2.666033999	456
chr22.fa	38054737	38062939	PDXP	6.279683292	836
chr22.fa	38071613	38075809	LGALS1	43.07673577	1596
chr22.fa	38082344	38089485	NOL12	2.821158563	532
chr22.fa	38092995	38172563	TRIOBP	3.474187792	2508
chr22.fa	38201114	38203443	H1F0	120.8470048	18696
chr22.fa	38203912	38213183	GCAT	19.38951617	2356
chr22.fa	38219389	38221502	GALR3	0	0
chr22.fa	38226862	38240278	ANKRD54	9.637602965	1292
chr22.fa	38240354	38240378	MIR658	0	0

chr22.fa	38243685	38243781	MIR659	0	0
chr22.fa	38245370	38284638	EIF3L	292.9135801	37906
chr22.fa	38302346	38338026	MICALL1	13.62279818	3496
chr22.fa	38339533	38349603	C22orf23	5.136129109	532
chr22.fa	38349674	38365319	POLR2F	11.42093302	1594
chr22.fa	38368319	38380539	SOX10	0	0
chr22.fa	38453262	38471708	PICK1	17.72426151	2482
chr22.fa	38474144	38479170	SLC16A8	0	0
chr22.fa	38480896	38506676	BAIAP2L2	2.668142489	380
chr22.fa	38507502	38577836	PLA2G6	4.551776385	988
chr22.fa	38597939	38612517	MAFF	5.485385286	912
chr22.fa	38615298	38669040	TMEM184B	5.607978873	1444
chr22.fa	38686697	38714089	CSNK1E	14.25323645	2736
chr22.fa	38740670	38794931	LOC400927	0	0
chr22.fa	38822333	38851203	KCNJ4	0	0
chr22.fa	38864083	38879442	KDELR3	3.362588471	380
chr22.fa	38879446	38902345	DDX17	87.72941662	30337
chr22.fa	38914954	38966189	DMC1	0	0
chr22.fa	38974125	39052634	LOC646851	0.665378952	456
chr22.fa	39052658	39069855	CBY1	24.31509757	2052
chr22.fa	39077954	39080766	TOMM22	43.99151887	4028
chr22.fa	39081548	39096459	JOSD1	20.6595797	4712
chr22.fa	39101807	39129592	GTPBP1	15.87526708	5320
chr22.fa	39130719	39152024	SUN2	7.52851133	2128
chr22.fa	39174513	39190161	DNAL4	18.47382942	1824
chr22.fa	39214457	39240017	NPTXR	1.771883968	684
chr22.fa	39260248	39268258	CBX6	29.770964	6460
chr22.fa	39353527	39359188	APOBEC3A	0	0
chr22.fa	39378405	39388154	APOBEC3B	0.963428963	76
chr22.fa	39410265	39414825	APOBEC3C	29.93120918	2204
chr22.fa	39417118	39429256	APOBEC3D	5.030252829	836
chr22.fa	39436673	39451975	APOBEC3F	4.095137865	1368
chr22.fa	39473010	39483748	APOBEC3G	8.113315873	988
chr22.fa	39493229	39500072	APOBEC3H	0	0
chr22.fa	39526779	39548538	CBX7	16.75601314	4408
chr22.fa	39619685	39640957	PDGFB	1.667513752	380
chr22.fa	39708887	39715670	RPL3	1365.272668	121292
chr22.fa	39709824	39709916	SNORD83B	0	0
chr22.fa	39711218	39711312	SNORD83A	0	0
chr22.fa	39712847	39712901	RNU86	0	0
chr22.fa	39715057	39715118	SNORD43	0	0
chr22.fa	39745954	39781593	SYNGR1	2.777633321	988
chr22.fa	39795759	39833132	TAB1	8.891348391	2280

chr22.fa	39853325	39888199	MGAT3		4.834163333	1672
chr22.fa	39898284	39914137	SMCR7L		14.05910484	5320
chr22.fa	39916569	39918691	ATF4		216.0246596	20368
chr22.fa	39925098	39928860	RPS19BP1		67.9109735	4180
chr22.fa	39966758	40085740	CACNA1I		0	0
chr22.fa	40139049	40289794	ENTHD1		0	0
chr22.fa	40297086	40367384	GRAP2		0	0
chr22.fa	40390953	40426043	FAM83F		12.15905485	1672
chr22.fa	40440821	40731812	TNRC6B		12.6140367	15658
chr22.fa	40742504	40762575	ADSL		16.66022749	1729
chr22.fa	40766595	40806291	SGSM3		29.85409872	5921
chr22.fa	40806294	41032690	MKL1		12.23842441	3647
chr22.fa	41075182	41078818	MCHR1		0	0
chr22.fa	41165639	41215392	SLC25A17		9.610041999	1444
chr22.fa	41220601	41252687	ST13		74.23749538	15744
chr22.fa	41253094	41323880	XPNPEP3		10.48386017	2128
chr22.fa	41255554	41258130	DNAJB7		0	0
chr22.fa	41347351	41369019	RBX1		48.00788961	2888
chr22.fa	41488517	41488570	MIR1281		0	0
chr22.fa	41488614	41576081	EP300		59.96754162	34884
chr22.fa	41601313	41627275	L3MBTL2		14.1691981	2893
chr22.fa	41625514	41636935	CHADL		1.89507998	304
chr22.fa	41641615	41682216	RANGAP1		20.83865068	4180
chr22.fa	41697567	41756151	ZC3H7B		31.06060637	12102
chr22.fa	41763392	41795330	TEF		11.50617622	3496
chr22.fa	41829492	41843027	TOB2		28.33312497	8588
chr22.fa	41855721	41864708	PHF5A		22.89503008	1619
chr22.fa	41865129	41921352	ACO2		32.81577303	3885
chr22.fa	41921803	41940476	POLR3H		10.71067337	2280
chr22.fa	41957014	41972670	CSDC2		3.166950793	532
chr22.fa	41972890	41985871	PMM1		24.08768195	2052
chr22.fa	41994032	42017061	PPPDE2		11.76582161	2964
chr22.fa	42017295	42060052	XRCC6		95.51817576	13528
chr22.fa	42069937	42084913	NHP2L1		61.37857272	6916
chr22.fa	42086547	42094140	C22orf46		7.592368432	2508
chr22.fa	42095518	42195459	MEI1		0	0
chr22.fa	42196678	42222303	CCDC134		5.386436899	456
chr22.fa	42229106	42302375	SREBF2		22.32859952	6384
chr22.fa	42296948	42297016	MIR33A		0	0
chr22.fa	42321036	42322821	TNFRSF13C		0	0
chr22.fa	42334741	42343148	CENPM		0	0
chr22.fa	42348191	42354946	LOC339674		0	0
chr22.fa	42372931	42394225		3-Sep	2.458347812	760

chr22.fa	42394729	42424477	WBP2NL	0.98842962	152
chr22.fa	42454338	42466846	NAGA	4.300715563	1064
chr22.fa	42470255	42475442	FAM109B	2.944957001	456
chr22.fa	42475699	42480288	C22orf32	70.85321959	7372
chr22.fa	42481530	42486888	NDUFA6	34.80227108	2736
chr22.fa	42486937	42521354	LOC100132273	2.761367833	304
chr22.fa	42522501	42526883	CYP2D6	0	0
chr22.fa	42536214	42540575	CYP2D7P1	0	0
chr22.fa	42556019	42611445	TCF20	15.08910182	7421
chr22.fa	42665759	42670868	LOC388906	0.921560391	76
chr22.fa	42776414	42828401	NFAM1	0	0
chr22.fa	42896585	42901148	SERHL	0	0
chr22.fa	42904341	42915829	RRP7A	8.482150878	2660
chr22.fa	42949925	42968535	SERHL2	7.229708288	445
chr22.fa	42969266	42978017	RRP7B	1.837247134	228
chr22.fa	42979727	43010962	POLDIP3	32.98866914	7524
chr22.fa	43011251	43011399	RNU12	7.681979223	76
chr22.fa	43013846	43045405	CYB5R3	25.38078826	5927
chr22.fa	43035809	43036607	ATP5L2	0	0
chr22.fa	43088127	43116876	A4GALT	3.829920043	532
chr22.fa	43192532	43253408	ARFGAP3	55.30913592	10404
chr22.fa	43265772	43411184	PACSIN2	46.56583359	10868
chr22.fa	43435523	43485434	TTLL1	6.101365348	760
chr22.fa	43506757	43525718	BIK	15.6630627	988
chr22.fa	43528212	43539403	MCAT	11.67395172	1596
chr22.fa	43547535	43559248	TSPO	104.4158496	6004
chr22.fa	43562628	43583137	TTLL12	23.00723183	5168
chr22.fa	43599230	43739355	SCUBE1	0	0
chr22.fa	43808020	43902800	MPPED1	0	0
chr22.fa	43924624	44208217	EFCAB6	2.554434678	836
chr22.fa	44220387	44258378	SULT4A1	0	0
chr22.fa	44275558	44287893	PNPLA5	0	0
chr22.fa	44319619	44343448	PNPLA3	0	0
chr22.fa	44351261	44392412	SAMM50	27.37677451	3192
chr22.fa	44395173	44565103	PARVB	8.120846192	1052
chr22.fa	44568836	44603035	PARVG	0	0
chr22.fa	44639557	44708731	KIAA1644	0.169733381	76
chr22.fa	44888450	44894005	LDOC1L	13.75939816	4864
chr22.fa	44965220	44968329	NCRNA00207	0	0
chr22.fa	45064427	45133561	PRR5	2.836520413	530
chr22.fa	45148438	45204978	ARHGAP8	3.972996097	43
chr22.fa	45277043	45405809	PHF21B	0.32711704	76
chr22.fa	45559726	45583890	NUP50	17.8942961	6536

chr22.fa	45588123	45636650	C22orf9	11.59413035	4940
chr22.fa	45596835	45596900	MIR1249	0	0
chr22.fa	45680868	45691755	UPK3A	32.82812275	2280
chr22.fa	45705081	45737836	FAM118A	6.406644464	1431
chr22.fa	45739945	45809500	SMC1B	0	0
chr22.fa	45809574	45828295	RIBC2	0	0
chr22.fa	45898719	45997014	FBLN1	0.706946311	228
chr22.fa	46067678	46241187	ATXN10	51.90272099	11476
chr22.fa	46316248	46373008	WNT7B	4.096192109	1064
chr22.fa	46402496	46406657	LOC730668	0	0
chr22.fa	46435787	46440748	LOC100271722	0	0
chr22.fa	46446339	46450024	C22orf26	0.650017102	76
chr22.fa	46452727	46454402	LOC150381	2.201112129	228
chr22.fa	46481877	46509808	LOC400931	0	0
chr22.fa	46486924	46487006	MIR3619	0	0
chr22.fa	46546499	46639653	PPARA	8.54269464	5700
chr22.fa	46639910	46646193	C22orf40	3.068604832	304
chr22.fa	46651560	46659219	PKDREJ	0	0
chr22.fa	46663861	46689905	TTC38	27.05146474	4688
chr22.fa	46691040	46692557	CN5H6-4	1.508021604	152
chr22.fa	46692638	46726707	GTSE1	0	0
chr22.fa	46731298	46753237	TRMU	5.828014782	760
chr22.fa	46756731	46933067	CELSR1	1.406964728	1064
chr22.fa	47022658	47075688	GRAMD4	1.057256732	303
chr22.fa	47080307	47134152	CERK	3.862601626	1140
chr22.fa	47158518	47571342	TBC1D22A	6.035399757	1520
chr22.fa	48016792	48027318	FLJ46257	0	0
chr22.fa	48670176	48670227	MIR3201	0	0
chr22.fa	48885288	49147744	FAM19A5	1.197772478	228
chr22.fa	50013290	50051190	C22orf34	0	0
chr22.fa	50166937	50218452	BRD1	15.15220589	4636
chr22.fa	50171538	50173958	LOC90834	0	0
chr22.fa	50247497	50283726	ZBED4	5.319115851	2432
chr22.fa	50296854	50312106	ALG12	2.411659837	380
chr22.fa	50312283	50321186	CRELD2	5.813857783	608
chr22.fa	50354143	50357720	PIM3	14.91500085	2356
chr22.fa	50432942	50451055	IL17REL	0	0
chr22.fa	50453552	50493055	TTLL8	0	0
chr22.fa	50497820	50524358	MLC1	0	0
chr22.fa	50528435	50600116	MOV10L1	0	0
chr22.fa	50609160	50618724	PANX2	2.654889128	532
chr22.fa	50624360	50638027	TRABD	14.33847966	2204
chr22.fa	50639408	50656045	SELO	5.43869731	836

chr22.fa	50656118	50683400	TUBGCP6	6.085250466	2432
chr22.fa	50683613	50689834	HDAC10	3.790159961	681
chr22.fa	50691331	50700089	MAPK12	3.915464463	456
chr22.fa	50702142	50708779	MAPK11	3.336232355	529
chr22.fa	50713408	50746002	PLXNB2	24.00770996	10100
chr22.fa	50750392	50765489	FAM116B	1.141144482	152
chr22.fa	50781760	50883430	PPP6R2	18.67985894	4862
chr22.fa	50883515	50913464	SBF1	11.10993086	5852
chr22.fa	50920012	50924866	ADM2	2.426118049	684
chr22.fa	50925213	50928750	MIOX	70.44025691	6721
chr22.fa	50941376	50946135	LMF2	0.880746064	152
chr22.fa	50946645	50963209	NCAPH2	5.477704361	906
chr22.fa	50963871	50964034	SCO2	10.83627908	118
chr22.fa	50964182	50968514	TYMP	7.483480025	639
chr22.fa	50968838	50971008	ODF3B	2.360001851	152
chr22.fa	50986462	50989452	KLHDC7B	0	0
chr22.fa	50989541	51001328	C22orf41	0	0
chr22.fa	51007596	51007764	CHKB-CPT1B	0	0
chr22.fa	51016815	51017096	CPT1B	0	0
chr22.fa	51017387	51017464	CHKB	14.67448247	76
chr22.fa	51021455	51022355	LOC100144603	0	0
chr22.fa	51039131	51049979	MAPK8IP2	1.3259385	304
chr22.fa	51061182	51066601	ARSA	1.856825962	532
chr22.fa	51113070	51171641	SHANK3	1.28150962	608
chr22.fa	51176652	51183727	ACR	0	0
chr22.fa	51195514	51238065	RPL23AP82	0.674415334	76
chr22.fa	51205920	51222087	RABL2B	2.804290649	456
chr3.fa	238650	451097	CHL1	0.277567544	141
chr3.fa	1134629	1445278	CNTN6	0.324255519	76
chr3.fa	2142247	3098040	CNTN4	0	0
chr3.fa	3111401	3152058	IL5RA	3.143456199	608
chr3.fa	3168600	3190706	TRNT1	9.559438258	1444
chr3.fa	3191317	3221401	CRBN	16.75992891	2880
chr3.fa	3841121	3889387	LRRN1	1.197621871	304
chr3.fa	4344988	4358949	SETMAR	12.41538689	1976
chr3.fa	4402829	4508966	SUMF1	3.707627669	532
chr3.fa	4535032	4889524	ITPR1	3.129148594	2128
chr3.fa	4790878	4793274	EGOT	0	0
chr3.fa	5021097	5026865	BHLHE40	12.44957454	2508
chr3.fa	5163930	5222601	ARL8B	19.17264299	3800
chr3.fa	5229359	5261650	EDEM1	9.487297806	3876
chr3.fa	6902802	7783218	GRM7	0.270037225	76
chr3.fa	8262834	8543344	LOC100288428	0	0

chr3.fa	8543511	8609806	LMCD1	1.320215458	152
chr3.fa	8613468	8615580	LOH3CR2A	0.541731121	76
chr3.fa	8661320	8693737	C3orf32	0	0
chr3.fa	8775486	8788451	CAV3	0	0
chr3.fa	8792095	8811300	OXTR	0	0
chr3.fa	8918880	9005159	RAD18	3.191047813	1216
chr3.fa	9022278	9291311	SRGAP3	4.357042346	2584
chr3.fa	9404717	9428475	THUMPD3	17.52410564	4631
chr3.fa	9430537	9439174	LOC440944	2.461510546	304
chr3.fa	9439403	9519838	SETD5	32.67149212	14810
chr3.fa	9540045	9595486	LHFPL4	0	0
chr3.fa	9691117	9744078	MTMR14	19.40879378	3254
chr3.fa	9745510	9771592	CPNE9	0	0
chr3.fa	9773434	9789699	BRPF1	8.748573551	2736
chr3.fa	9791628	9808353	OGG1	8.331092687	1368
chr3.fa	9799090	9811661	CAMK1	26.79934968	2388
chr3.fa	9821654	9834178	TADA3	55.40974098	10640
chr3.fa	9834809	9848789	ARPC4	72.54302308	5414
chr3.fa	9851644	9874929	TTLL3	2.698263763	208
chr3.fa	9879533	9885702	RPUSD3	6.21085618	506
chr3.fa	9908394	9921938	CIDEC	0	0
chr3.fa	9932271	9936031	JAGN1	9.665013325	1064
chr3.fa	9944296	9958084	IL17RE	2.003817781	380
chr3.fa	9958764	9975314	IL17RC	9.632331742	1558
chr3.fa	9975524	9987097	CRELD1	2.376417946	456
chr3.fa	9987226	9994078	PRRT3	0.304375478	76
chr3.fa	10005636	10028522	TMEM111	16.95722326	1216
chr3.fa	10048102	10052779	LOC401052	0.603329127	76
chr3.fa	10059237	10067820	CIDECP	0	0
chr3.fa	10068113	10143614	FANCD2	1.088131039	360
chr3.fa	10123004	10149915	C3orf24	0	0
chr3.fa	10157333	10168874	C3orf10	199.52784	15580
chr3.fa	10183319	10193746	VHL	24.42760053	4788
chr3.fa	10206563	10285427	IRAK2	5.939162285	1368
chr3.fa	10290177	10322906	TATDN2	21.92648051	7144
chr3.fa	10326103	10327430	C3orf42	0.861920268	76
chr3.fa	10327434	10333135	GHRL	0	0
chr3.fa	10329098	10335133	GHRLOS	1.524136486	152
chr3.fa	10342615	10362858	SEC13	47.02518303	5077
chr3.fa	10365707	10547268	ATP2B2	0	0
chr3.fa	10436173	10436246	MIR885	0	0
chr3.fa	10801169	10805877	LOC285370	0	0
chr3.fa	10857917	10980146	SLC6A11	0.574864522	76

chr3.fa	11034420	11080935	SLC6A1	0	0
chr3.fa	11178779	11304939	HRH1	0.237506249	76
chr3.fa	11314010	11597543	ATG7	13.25155347	3040
chr3.fa	11599140	11762220	VGLL4	18.60847152	3724
chr3.fa	11831919	11888352	C3orf31	6.394445348	532
chr3.fa	12045862	12233532	SYN2	0.345039199	76
chr3.fa	12194568	12200647	TIMP4	4.805849335	456
chr3.fa	12329349	12475855	PPARG	102.170158	15352
chr3.fa	12525931	12574820	TSEN2	7.507275831	1140
chr3.fa	12581280	12586963	LOC100129480	2.11180255	152
chr3.fa	12598594	12625099	MKRN2	14.23215156	2508
chr3.fa	12625211	12705700	RAF1	44.88672315	9430
chr3.fa	12775392	12800808	TMEM40	4.016220126	456
chr3.fa	12838171	12876313	CAND2	0.747760638	228
chr3.fa	12876444	12883081	RPL32	462.4190941	50692
chr3.fa	12881811	12881949	SNORA7A	0	0
chr3.fa	12938719	13114617	IQSEC1	17.80784805	6145
chr3.fa	13357737	13461809	NUP210	1.910140618	912
chr3.fa	13521715	13547924	HDAC11	7.203201566	1442
chr3.fa	13590625	13679922	FBLN2	0.981501727	304
chr3.fa	13692221	13788132	LOC285375	0	0
chr3.fa	13860082	13921618	WNT7A	0	0
chr3.fa	13974553	13978444	LOC100132526	0	0
chr3.fa	13978807	14107481	TPRXL	0	0
chr3.fa	14153577	14166371	CHCHD4	13.55833865	1444
chr3.fa	14166440	14185180	TMEM43	1.712394451	380
chr3.fa	14186648	14220172	XPC	16.30765797	4028
chr3.fa	14220228	14239869	LSM3	52.70138659	2432
chr3.fa	14444106	14530618	SLC6A6	1.825801049	760
chr3.fa	14530858	14583588	GRIP2	0.147895457	76
chr3.fa	14693253	14714166	C3orf19	13.14748447	2584
chr3.fa	14716606	14814543	C3orf20	0	0
chr3.fa	14860469	14976072	FGD5	1.164639076	456
chr3.fa	14989236	15090018	NR2C2	16.4855241	8284
chr3.fa	15090781	15106816	MRPS25	8.725982595	2204
chr3.fa	15111580	15140655	ZFYVE20	9.427055257	4180
chr3.fa	15206869	15247466	COL6A4P1	0	0
chr3.fa	15247733	15294423	CAPN7	14.92614572	4329
chr3.fa	15296357	15382901	SH3BP5	24.27473506	4779
chr3.fa	15451377	15469042	METTL6	5.038234967	836
chr3.fa	15469064	15484120	EAF1	23.20844195	6916
chr3.fa	15491640	15563258	COLQ	0	0
chr3.fa	15537746	15537815	MIR4270	0	0

chr3.fa	15602240	15643130	HACL1	14.1030819	1900
chr3.fa	15643255	15687325	BTD	1.662844955	228
chr3.fa	15708744	15901053	ANKRD28	15.57932556	6848
chr3.fa	15915278	15915356	MIR563	0	0
chr3.fa	16216184	16271253	GALNTL2	0.246693238	76
chr3.fa	16298568	16306496	DPH3	8.16572689	2204
chr3.fa	16306667	16347594	OXNAD1	2.884563845	760
chr3.fa	16357352	16555222	RFTN1	1.906375458	380
chr3.fa	16628301	16647006	DAZL	0	0
chr3.fa	16926452	17132098	PLCL2	6.13946876	1748
chr3.fa	17198654	17784240	TBC1D5	10.80329629	6080
chr3.fa	18389134	18480265	SATB1	27.73627192	10792
chr3.fa	19190017	19577135	KCNH8	0	0
chr3.fa	19920966	19975706	EFHB	0.801828325	152
chr3.fa	19988572	20026667	RAB5A	20.70837617	3344
chr3.fa	20021453	20053765	C3orf48	0	0
chr3.fa	20081524	20195896	KAT2B	18.26388414	5850
chr3.fa	20202085	20227724	SGOL1	0	0
chr3.fa	21447218	21448177	VENTXP7	0	0
chr3.fa	21462490	21792816	ZNF385D	1.89507998	228
chr3.fa	23244784	23632296	UBE2E2	10.11457335	1182
chr3.fa	23847439	23932807	UBE2E1	40.45618489	3938
chr3.fa	23933572	23958537	NKIRAS1	2.294939898	304
chr3.fa	23958639	23962332	RPL15	384.0272725	50768
chr3.fa	23986751	24022109	NR1D2	13.37610494	4788
chr3.fa	24141465	24144738	LOC152024	0	0
chr3.fa	24158645	24536313	THRB	11.91672919	6080
chr3.fa	25469754	25639422	RARB	0.731193937	152
chr3.fa	25639475	25705788	TOP2B	147.3208941	50758
chr3.fa	25760435	25831530	NGLY1	22.81219658	4332
chr3.fa	25831563	25836025	OXSM	5.247126005	532
chr3.fa	25900023	25915186	LOC285326	0	0
chr3.fa	26664300	26752265	LRRC3B	0	0
chr3.fa	27257097	27410912	NEK10	2.168581152	380
chr3.fa	27414214	27498245	SLC4A7	4.279178851	2204
chr3.fa	27757886	27763785	EOMES	0	0
chr3.fa	28283124	28361263	CMC1	9.156867425	380
chr3.fa	28363845	28390618	AZI2	13.42926899	3268
chr3.fa	28431987	28566632	ZCWPW2	1.879115705	207
chr3.fa	29322803	30051886	RBMS3	0.136901192	76
chr3.fa	30647994	30735633	TGFBR2	4.137458255	1292
chr3.fa	30767692	30936153	GADL1	0	0
chr3.fa	31574491	31677556	STT3B	15.07283633	2483

chr3.fa	31702317	32023265	OSBPL10	11.34638286	3116
chr3.fa	32023343	32033120	ZNF860	1.894477555	380
chr3.fa	32148003	32210207	GPD1L	121.1673446	32680
chr3.fa	32280171	32411813	CMTM8	5.869732747	456
chr3.fa	32433163	32496333	CMTM7	3.388944586	304
chr3.fa	32522804	32544403	CMTM6	2.380333711	532
chr3.fa	32567463	32612366	DYNC1LI1	12.72699148	2116
chr3.fa	32726698	32815354	CNOT10	12.52758864	2280
chr3.fa	32859510	32933771	TRIM71	0	0
chr3.fa	32993066	32996403	CCR4	0	0
chr3.fa	33038100	33138694	GLB1	8.335610878	1444
chr3.fa	33131908	33135795	TMPPE	0.883155766	228
chr3.fa	33155450	33189265	CRTAP	8.604744465	3800
chr3.fa	33191537	33260707	SUSD5	0	0
chr3.fa	33318934	33428757	FBXL2	1.165994533	228
chr3.fa	33429829	33481897	UBP1	25.70489317	7124
chr3.fa	33537739	33759848	CLASP2	14.44450654	6962
chr3.fa	33840066	33911195	PDCD6IP	29.79099465	13528
chr3.fa	35683849	35835988	ARPP21	0.844751141	304
chr3.fa	35785968	35786051	MIR128-2	0	0
chr3.fa	36422097	36589496	STAC	0.772610689	152
chr3.fa	36753913	36781352	DCLK3	8.781556346	3116
chr3.fa	36868308	36986548	TRANK1	4.585361606	3192
chr3.fa	37027357	37034795	EPM2AIP1	7.077897065	3496
chr3.fa	37034841	37092337	MLH1	18.55455444	3420
chr3.fa	37094117	37217851	LRRFIP2	27.07285085	6856
chr3.fa	37284682	37408370	GOLGA4	49.83158217	26311
chr3.fa	37440968	37476988	C3orf35	1.415398685	228
chr3.fa	37493813	37861281	ITGA9	0.866739672	228
chr3.fa	37903669	38025960	CTDSPL	24.92189064	7372
chr3.fa	38010895	38010971	MIR26A1	0	0
chr3.fa	38035078	38048676	VILL	6.571106622	1216
chr3.fa	38048987	38071154	PLCD1	23.82306655	4940
chr3.fa	38080696	38164200	DLEC1	0.614775211	228
chr3.fa	38164229	38178733	ACAA1	103.4567882	12303
chr3.fa	38179969	38184512	MYD88	24.5072713	4636
chr3.fa	38207026	38296979	OXSR1	27.11110487	8142
chr3.fa	38307298	38319806	SLC22A13	0.894150031	152
chr3.fa	38347445	38359859	SLC22A14	0	0
chr3.fa	38388251	38456467	XYLB	3.937904812	963
chr3.fa	38492518	38496311	LOC100128640	0.909511881	152
chr3.fa	38495790	38534633	ACVR2B	4.528884216	3420
chr3.fa	38537763	38567796	EXOG	2.183039364	456

chr3.fa	38589553	38691163	SCN5A	0.133136033	76
chr3.fa	38738837	38835501	SCN10A	0	0
chr3.fa	38887260	38992052	SCN11A	1.584830854	684
chr3.fa	39093507	39137881	WDR48	30.39055862	7448
chr3.fa	39138091	39149130	GORASP1	16.84893728	3420
chr3.fa	39149152	39180394	TTC21A	0.268832374	76
chr3.fa	39183342	39195102	CSRNP1	19.3880101	4104
chr3.fa	39224706	39234077	XIRP1	0	0
chr3.fa	39304985	39323226	CX3CR1	0	0
chr3.fa	39371197	39375171	CCR8	0	0
chr3.fa	39424815	39438819	SLC25A38	21.55568762	3040
chr3.fa	39448204	39454032	RPSA	145.6955502	11096
chr3.fa	39449882	39450030	SNORA6	0	0
chr3.fa	39452545	39452698	SNORA62	0	0
chr3.fa	39509070	39567857	MOBP	0	0
chr3.fa	39851303	40301811	MYRIP	19.04161545	6156
chr3.fa	40214638	40351172	FLJ33065	0	0
chr3.fa	40351190	40353915	EIF1B	24.1575633	1596
chr3.fa	40428673	40470110	ENTPD3	4.917749869	912
chr3.fa	40498801	40503859	RPL14	341.2523519	21435
chr3.fa	40518604	40531728	ZNF619	2.735614143	760
chr3.fa	40547530	40559199	ZNF620	3.540605202	532
chr3.fa	40566376	40581043	ZNF621	5.282367896	2888
chr3.fa	41240942	41281939	CTNNB1	110.1521451	24977
chr3.fa	41288090	42003660	ULK4	3.365299385	974
chr3.fa	42132746	42267268	TRAK1	43.48758995	15656
chr3.fa	42299318	42307662	CCK	2.836671019	304
chr3.fa	42438575	42452065	LYZL4	0	0
chr3.fa	42544104	42579065	VIPR1	7.794783396	1444
chr3.fa	42589473	42623428	SEC22C	23.39338657	3192
chr3.fa	42632298	42636490	SS18L2	19.66196309	1064
chr3.fa	42642147	42690233	NKTR	10.77874745	5226
chr3.fa	42695176	42709072	ZBTB47	4.156886477	1520
chr3.fa	42727011	42733938	KBTBD5	0	0
chr3.fa	42734155	42744319	HHATL	0	0
chr3.fa	42749874	42814745	CCDC13	0	0
chr3.fa	42824400	42846027	HIGD1A	33.34545564	6764
chr3.fa	42850964	42908775	CCBP2	1.160120885	228
chr3.fa	42913684	42917633	CYP8B1	0.86929998	228
chr3.fa	42947402	42960825	ZNF662	2.722963208	1064
chr3.fa	43020759	43099207	FAM198A	0.333442508	76
chr3.fa	43120729	43147565	C3orf39	0.901981563	152
chr3.fa	43328004	43392634	SNRK	9.218314825	3192

chr3.fa	43407818	43663560	ANO10	4.588674946	833
chr3.fa	43732375	43764217	ABHD5	7.697792892	2736
chr3.fa	44155704	44155802	MIR138-1	0	0
chr3.fa	44283378	44373590	C3orf77	0	0
chr3.fa	44379611	44450940	C3orf23	11.7159709	4256
chr3.fa	44481262	44519162	ZNF445	8.375973386	5700
chr3.fa	44596713	44624973	ZNF167	1.152138747	304
chr3.fa	44626456	44637557	ZNF660	0	0
chr3.fa	44666511	44689963	ZNF197	5.066247752	2128
chr3.fa	44690233	44702283	ZNF35	3.456717453	608
chr3.fa	44754135	44765323	ZNF502	3.15128773	684
chr3.fa	44771124	44778575	ZNF501	1.494768243	304
chr3.fa	44790236	44803173	KIAA1143	10.90947378	3724
chr3.fa	44803209	44894748	KIF15	0.709958438	228
chr3.fa	44903380	44903407	MIR564	0	0
chr3.fa	44903474	44907154	TMEM42	6.323810959	380
chr3.fa	44916098	44956088	TGM4	647.3010668	128896
chr3.fa	44956753	45017674	ZDHHC3	9.383530015	7904
chr3.fa	45017741	45054158	EXOSC7	17.31973279	1748
chr3.fa	45067759	45077563	CLEC3B	4.030377125	228
chr3.fa	45123769	45187914	CDCP1	2.567688038	1064
chr3.fa	45265956	45267814	TMEM158	0	0
chr3.fa	45430075	45590328	LARS2	4.357343558	1216
chr3.fa	45636323	45722755	LIMD1	18.04189035	5380
chr3.fa	45729868	45730374	LOC644714	0	0
chr3.fa	45730754	45786900	SACM1L	21.6097553	5319
chr3.fa	45796941	45838035	SLC6A20	0	0
chr3.fa	45865446	45883621	LZTFL1	6.736924238	1520
chr3.fa	45927996	45944667	CCR9	0	0
chr3.fa	45959395	46037307	FYCO1	32.03021019	18088
chr3.fa	45984973	45989845	CXCR6	0.586009394	76
chr3.fa	46062291	46068979	XCR1	0	0
chr3.fa	46243200	46249832	CCR1	0	0
chr3.fa	46283872	46308197	CCR3	0	0
chr3.fa	46395235	46402413	CCR2	0	0
chr3.fa	46411633	46417697	CCR5	0	0
chr3.fa	46448721	46451014	CCRL2	0	0
chr3.fa	46477496	46506598	LTF	1.63980218	304
chr3.fa	46539485	46542439	RTP3	0	0
chr3.fa	46556878	46608011	LRRC2	0.224102282	76
chr3.fa	46616045	46623952	TDGF1	0.522152292	76
chr3.fa	46653925	46668033	LOC100132146	0	0
chr3.fa	46710485	46735194	ALS2CL	2.795856692	988

chr3.fa	46742823	46752413	TMIE	0	0
chr3.fa	46753606	46759373	PRSS50	0	0
chr3.fa	46783581	46786245	PRSS45	0	0
chr3.fa	46871894	46875585	PRSS42	0	0
chr3.fa	46899357	46904973	MYL3	2.464221461	152
chr3.fa	46919236	46945289	PTH1R	14.4009813	2052
chr3.fa	46963220	47018270	CCDC12	26.09827701	1518
chr3.fa	47021173	47051193	NBEAL2	29.30574092	17176
chr3.fa	47053032	47054957	NRADDP	0	0
chr3.fa	47057898	47205467	SETD2	34.93254559	19560
chr3.fa	47205860	47285606	FLJ39534	0	0
chr3.fa	47269516	47324329	KIF9	3.114991595	684
chr3.fa	47324338	47388306	KLHL18	10.9171547	3420
chr3.fa	47422491	47454931	PTPN23	11.71536848	4094
chr3.fa	47455184	47517445	SCAP	10.49108928	2964
chr3.fa	47537130	47555199	C3orf75	10.99682547	985
chr3.fa	47603728	47620359	CSPG5	0.530435643	76
chr3.fa	47627378	47823405	SMARCC1	66.07116606	25256
chr3.fa	47844399	47891686	DHX30	15.01666015	4104
chr3.fa	47891045	47891119	MIR1226	0	0
chr3.fa	47892180	48130769	MAP4	58.95155103	34109
chr3.fa	48198668	48229801	CDC25A	1.853060803	456
chr3.fa	48264862	48266975	CAMP	0	0
chr3.fa	48282596	48312479	ZNF589	7.145368719	1596
chr3.fa	48335589	48342848	NME6	1.958334657	152
chr3.fa	48348336	48369831	SPINK8	0	0
chr3.fa	48413709	48436190	FBXW12	0	0
chr3.fa	48445261	48471460	PLXNB1	5.758886457	2812
chr3.fa	48473580	48481529	CCDC51	7.600350569	760
chr3.fa	48481686	48485537	CCDC72	134.5374253	4136
chr3.fa	48488218	48507054	ATRIP	4.105830917	684
chr3.fa	48507229	48509044	TREX1	8.193739676	988
chr3.fa	48509197	48541661	SHISA5	15.85327855	2280
chr3.fa	48555117	48594227	PFKFB4	5.890516427	1365
chr3.fa	48599151	48601201	UCN2	0	0
chr3.fa	48601506	48632593	COL7A1	0	0
chr3.fa	48616335	48616410	MIR711	0	0
chr3.fa	48636432	48647098	UQCRC1	55.920899	6004
chr3.fa	48658275	48659189	TMEM89	0	0
chr3.fa	48663156	48672926	SLC26A6	1.71992477	304
chr3.fa	48673896	48700348	CELSR3	0.095635046	76
chr3.fa	48711278	48723334	NCKIPSD	5.79653805	1140
chr3.fa	48725436	48754711	IP6K2	27.08007995	5396

chr3.fa	48788093	48885270	PRKAR2A	49.06559816	7757
chr3.fa	48894356	48936402	SLC25A20	16.54410998	2052
chr3.fa	48955221	48956818	C3orf71	3.955073938	380
chr3.fa	48960181	49022971	ARIH2	20.13983711	5230
chr3.fa	49027341	49044581	P4HTM	3.009416528	456
chr3.fa	49044637	49053386	WDR6	44.35191992	10260
chr3.fa	49053406	49058467	DALRD3	11.56897908	1269
chr3.fa	49057581	49057667	MIR425	0	0
chr3.fa	49057908	49060926	NDUFAF3	38.88867377	3238
chr3.fa	49058051	49058142	MIR191	0	0
chr3.fa	49061762	49066875	IMPDH2	78.39362882	8802
chr3.fa	49067142	49131504	QRICH1	19.6810901	4332
chr3.fa	49133365	49142171	QARS	69.04955767	11141
chr3.fa	49145479	49158371	USP19	16.91113771	6004
chr3.fa	49158547	49170599	LAMB2	1.970684379	760
chr3.fa	49190292	49191834	LAMB2P1	0	0
chr3.fa	49199968	49203785	CCDC71	6.948526191	836
chr3.fa	49209018	49213919	KLHDC8B	10.95601115	1444
chr3.fa	49215069	49229291	LOC646498	0	0
chr3.fa	49235861	49295537	CCDC36	0	0
chr3.fa	49306030	49314508	C3orf62	3.207012088	760
chr3.fa	49311574	49311619	MIR4271	0	0
chr3.fa	49314577	49377536	USP4	24.72580114	6764
chr3.fa	49394609	49395791	GPX1	83.20911696	6536
chr3.fa	49396579	49449526	RHOA	241.315084	30780
chr3.fa	49449639	49453909	TCTA	34.1126445	4863
chr3.fa	49454211	49459704	AMT	15.79604813	1976
chr3.fa	49459766	49466757	NICN1	12.19761008	2356
chr3.fa	49506146	49573048	DAG1	3.344214493	1444
chr3.fa	49591922	49708982	BSN	0.358593772	380
chr3.fa	49711435	49720934	APEH	42.59901236	7801
chr3.fa	49721380	49726196	MST1	0.490976773	76
chr3.fa	49726990	49758931	RNF123	6.232543498	1748
chr3.fa	49754267	49757238	AMIGO3	0	0
chr3.fa	49758963	49761384	GMPPB	31.96288914	3419
chr3.fa	49761728	49823973	IP6K1	21.00672739	6232
chr3.fa	49828167	49837254	CDHR4	0	0
chr3.fa	49840687	49842463	C3orf54	0	0
chr3.fa	49842638	49851391	UBA7	7.946293406	1748
chr3.fa	49866028	49893992	TRAIP	1.131053855	152
chr3.fa	49895422	49907369	CAMKV	0	0
chr3.fa	49924436	49941306	MST1R	0	0
chr3.fa	49946302	49967445	MON1A	2.053066065	304

chr3.fa	49977477	50114685	RBM6	22.00494643	5463
chr3.fa	50126352	50156392	RBM5	37.75551143	7809
chr3.fa	50192848	50226508	SEMA3F	2.604285387	608
chr3.fa	50229043	50235129	GNAT1	0	0
chr3.fa	50242692	50258406	SLC38A3	0	0
chr3.fa	50273647	50296786	GNAI2	42.14071717	6665
chr3.fa	50305040	50314572	SEMA3B	2.738325058	532
chr3.fa	50316518	50325162	C3orf45	0	0
chr3.fa	50325546	50330026	IFRD2	28.12302908	3223
chr3.fa	50330262	50336899	HYAL3	0.611160658	76
chr3.fa	50333833	50334958	NAT6	3.999201606	299
chr3.fa	50337320	50349812	HYAL1	2.751427812	380
chr3.fa	50355221	50360281	HYAL2	0.474410072	76
chr3.fa	50362341	50365668	TUSC2	30.62264304	3420
chr3.fa	50367217	50378367	RASSF1	4.417435501	684
chr3.fa	50378537	50383156	ZMYND10	1.291148428	152
chr3.fa	50384919	50388486	NPRL2	8.89014354	912
chr3.fa	50388774	50391496	CYB561D2	7.202900353	517
chr3.fa	50392180	50396939	TMEM115	2.731698378	380
chr3.fa	50400231	50540892	CACNA2D2	0.854691162	304
chr3.fa	50595456	50608361	C3orf18	2.096290094	380
chr3.fa	50606909	50622421	HEMK1	0.992947811	380
chr3.fa	50643885	50649262	CISH	4.037455624	608
chr3.fa	50654601	50686719	MAPKAPK3	20.60295171	3420
chr3.fa	50712672	51421629	DOCK3	1.820078007	1058
chr3.fa	51422692	51426828	MANF	17.06550924	1064
chr3.fa	51428699	51435336	RBM15B	14.4670975	5168
chr3.fa	51436166	51534018	VPRBP	14.48245935	4408
chr3.fa	51575596	51697612	RAD54L2	12.40695294	3876
chr3.fa	51705222	51738339	TEX264	5.65044987	532
chr3.fa	51741081	51752625	GRM2	0	0
chr3.fa	51812577	51813203	IQCF6	0	0
chr3.fa	51862569	51864876	IQCF3	0	0
chr3.fa	51895645	51897440	IQCF2	0	0
chr3.fa	51907737	51909600	IQCF5	0	0
chr3.fa	51928892	51937386	IQCF1	0	0
chr3.fa	51967446	51975922	RRP9	12.61072336	1292
chr3.fa	51976361	51982883	PARP3	3.394968841	532
chr3.fa	51989330	51991469	GPR62	0	0
chr3.fa	51991521	52001482	PCBP4	11.72591092	1716
chr3.fa	52002526	52008646	ABHD14B	39.32678771	5857
chr3.fa	52014409	52015216	ABHD14A	13.33152545	532
chr3.fa	52017300	52017605	ACY1	11.71386241	238

chr3.fa	52027644	52029958	RPL29	346.8713251	16007
chr3.fa	52082937	52090461	DUSP7	3.89437957	836
chr3.fa	52096110	52099128	C3orf74	0	0
chr3.fa	52109249	52188706	POC1A	1.009815725	152
chr3.fa	52232116	52248343	ALAS1	56.87785189	9196
chr3.fa	52255098	52260179	TLR9	0	0
chr3.fa	52262626	52273183	TWF2	19.72250686	2128
chr3.fa	52280225	52284615	PPM1M	10.06502385	1368
chr3.fa	52288438	52312659	WDR82	36.26902654	10336
chr3.fa	52302294	52302377	MIRLET7G	0	0
chr3.fa	52321836	52329272	GLYCTK	2.896913568	684
chr3.fa	52350335	52434513	DNAH1	0.785713443	684
chr3.fa	52435025	52444009	BAP1	20.43939319	4864
chr3.fa	52444527	52457657	PHF7	3.017398666	456
chr3.fa	52467268	52479043	SEMA3G	0	0
chr3.fa	52485107	52488057	TNNC1	1.644621584	76
chr3.fa	52489524	52527088	NISCH	15.88852044	5527
chr3.fa	52529356	52558384	STAB1	0	0
chr3.fa	52558512	52569093	NT5DC2	2.05035515	304
chr3.fa	52570621	52574586	C3orf78	2.549314061	76
chr3.fa	52579368	52719866	PBRM1	23.2475996	11932
chr3.fa	52719936	52728503	GNL3	112.0434599	15861
chr3.fa	52723256	52723331	SNORD19	0	0
chr3.fa	52724754	52724846	SNORD19B	0	0
chr3.fa	52726752	52726828	SNORD69	0	0
chr3.fa	52728511	52739856	GLT8D1	9.247231249	1368
chr3.fa	52740049	52742197	SPCS1	53.89418905	3192
chr3.fa	52744796	52804965	NEK4	18.12457325	4484
chr3.fa	52811608	52826077	ITIH1	0	0
chr3.fa	52828784	52843025	ITIH3	0	0
chr3.fa	52847006	52864717	ITIH4	0	0
chr3.fa	52868956	52869235	MUSTN1	0	0
chr3.fa	52870772	52874515	TMEM110	3.379155171	836
chr3.fa	52937583	53080089	SFMBT1	7.019009973	2354
chr3.fa	53122501	53164470	RFT1	0.898065797	304
chr3.fa	53195223	53226733	PRKCD	47.88062722	9013
chr3.fa	53258724	53290130	TKT	27.63491383	5624
chr3.fa	53317445	53381654	DCP1A	11.42319211	4560
chr3.fa	53529031	53846492	CACNA1D	0.872462714	456
chr3.fa	53850324	53880420	CHDH	14.51604457	3572
chr3.fa	53880577	53899827	IL17RB	3.366504236	456
chr3.fa	53901094	53916229	ACTR8	11.0423086	2660
chr3.fa	53919226	53925989	SELK	60.16890234	3268

chr3.fa	54156693	55108584	CACNA2D3	0.622907955	152
chr3.fa	54666151	54673884	ESRG	0	0
chr3.fa	54952381	54962072	LRTM1	0	0
chr3.fa	55499743	55521331	WNT5A	1.372475869	532
chr3.fa	55542336	56502391	ERC2	0.373353197	152
chr3.fa	55691243	55693497	C3orf51	0	0
chr3.fa	55886520	55886622	MIR3938	0	0
chr3.fa	56591184	56653929	CCDC66	6.030580353	1140
chr3.fa	56654160	56717135	C3orf63	13.15155084	6992
chr3.fa	56761446	57113336	ARHGEF3	14.36332971	4408
chr3.fa	57094469	57109460	SPATA12	0	0
chr3.fa	57124010	57199403	IL17RD	1.312685139	760
chr3.fa	57231944	57234280	HESX1	0	0
chr3.fa	57261765	57307498	APPL1	12.61448852	4935
chr3.fa	57302379	57326710	ASB14	0	0
chr3.fa	57327727	57530071	DNAH12	0.34082222	228
chr3.fa	57541981	57547768	PDE12	9.74619016	2584
chr3.fa	57557090	57583215	ARF4	109.1982044	12616
chr3.fa	57611181	57678816	FAM116A	6.266128719	1938
chr3.fa	57743174	57914894	SLMAP	24.98981411	7334
chr3.fa	57994127	58157982	FLNB	38.19347476	24244
chr3.fa	58178354	58200398	DNASE1L3	0	0
chr3.fa	58223259	58280461	ABHD6	0.478325838	76
chr3.fa	58291972	58305920	RPP14	10.13791733	2356
chr3.fa	58318617	58410878	PXK	11.67997598	1572
chr3.fa	58413357	58419579	PDHB	33.31126799	3258
chr3.fa	58477823	58488087	KCTD6	3.60129957	456
chr3.fa	58490863	58522929	ACOX2	9.4186213	1444
chr3.fa	58549845	58563491	FAM107A	3.304303804	760
chr3.fa	58619670	58652561	FAM3D	3.500393301	304
chr3.fa	58727737	59035715	C3orf67	0.897312765	152
chr3.fa	59735036	61237133	FHIT	11.11053328	796
chr3.fa	61547243	62280573	PTPRG	0.516579856	228
chr3.fa	62109159	62110392	ID2B	0	0
chr3.fa	62305396	62319320	C3orf14	31.33862573	1596
chr3.fa	62355347	62359190	FEZF2	0	0
chr3.fa	62384021	62861064	CADPS	0.206180123	76
chr3.fa	63088364	63110737	LOC285401	0	0
chr3.fa	63263914	63602597	SYNPR	0	0
chr3.fa	63638344	63650891	SNTN	0	0
chr3.fa	63805041	63834312	C3orf49	0	0
chr3.fa	63819546	63849597	THOC7	12.49084069	836
chr3.fa	63850233	63989136	ATXN7	10.64892475	5320

chr3.fa	63996231	64009120	PSMD6	35.00333058	3040
chr3.fa	64064037	64073039	LOC100287879	0	0
chr3.fa	64079526	64211131	PRICKLE2	4.676327855	2584
chr3.fa	64501331	64673365	ADAMTS9	2.0349933	988
chr3.fa	64705683	64941858	MIR548A2	0	0
chr3.fa	65339906	66024509	MAGI1	25.14373383	15301
chr3.fa	66119285	66429220	SLC25A26	8.995116182	1569
chr3.fa	66429352	66550845	LRIG1	7.167658462	2204
chr3.fa	67048727	67061632	KBTBD8	0.244434142	76
chr3.fa	67275888	67275951	MIR4272	0	0
chr3.fa	67410884	67705038	SUCLG2	38.25386791	6764
chr3.fa	68053454	68594771	FAM19A1	0	0
chr3.fa	68780917	68981711	FAM19A4	0.512814697	76
chr3.fa	69024368	69062774	C3orf64	3.042700536	836
chr3.fa	69068978	69101484	TMF1	14.1432938	6460
chr3.fa	69098109	69098186	MIR3136	0	0
chr3.fa	69103881	69129524	UBA3	27.61940137	3886
chr3.fa	69134090	69155239	ARL6IP5	6.45453729	912
chr3.fa	69156039	69171746	LMOD3	0	0
chr3.fa	69219146	69435430	FRMD4B	10.40012303	3633
chr3.fa	69788586	70017488	MITF	3.755821708	1520
chr3.fa	71004736	71633140	FOXP1	45.54381875	19378
chr3.fa	71591121	71591240	MIR1284	0	0
chr3.fa	71728440	71778269	EIF4E3	8.398714948	3496
chr3.fa	71803201	71804328	GPR27	0	0
chr3.fa	71820806	71834357	PROK2	0	0
chr3.fa	72423744	72495774	RYBP	22.89728918	6764
chr3.fa	72798428	72897598	SHQ1	4.74876952	898
chr3.fa	72937385	73024522	GXYLT2	0	0
chr3.fa	73046119	73115011	PPP4R2	16.41007031	1593
chr3.fa	73110810	73112471	EBLN2	0	0
chr3.fa	73431652	73674072	PDZRN3	5.197576509	1436
chr3.fa	74311722	74570343	CNTN3	3.435933774	1140
chr3.fa	75470703	75484266	FAM86DP	3.197222674	532
chr3.fa	75679914	75680009	MIR1324	0	0
chr3.fa	75713487	75716368	FRG2C	0	0
chr3.fa	75786029	75834255	ZNF717	6.096997763	1208
chr3.fa	77089294	77699114	ROBO2	0.112653566	65
chr3.fa	78646388	79817059	ROBO1	0.869149374	456
chr3.fa	81538850	81810950	GBE1	11.08041201	2280
chr3.fa	84687556	84918726	LOC440970	0	0
chr3.fa	85008133	86123579	CADM2	0.237355642	152
chr3.fa	86987123	87040257	VGLL3	52.73828515	36404

chr3.fa	87276413	87304698	CHMP2B	32.66576908	5700
chr3.fa	87308783	87325737	POU1F1	0	0
chr3.fa	88031726	88042919	HTR1F	0.360099836	76
chr3.fa	88101100	88198892	CGGBP1	19.4044262	5928
chr3.fa	88188262	88193814	ZNF654	6.33540765	2052
chr3.fa	88199017	88207115	C3orf38	6.394445348	1064
chr3.fa	89156674	89531284	EPHA3	0	0
chr3.fa	93591881	93692934	PROS1	0.319737328	76
chr3.fa	93698983	93774522	ARL13B	4.481593815	1140
chr3.fa	93733215	93747454	STX19	2.970409477	228
chr3.fa	93776766	93782067	DHFRL1	0.851076609	228
chr3.fa	93783281	93845630	NSUN3	7.006057825	608
chr3.fa	94657107	94710169	LOC255025	0	0
chr3.fa	96533425	97467786	EPHA6	0	0
chr3.fa	97483595	97517373	ARL6	2.363315191	228
chr3.fa	97660661	97691295	MINA	13.7130114	4864
chr3.fa	97705527	97753831	GABRR3	0	0
chr3.fa	97806017	97806946	OR5AC2	0	0
chr3.fa	97851542	97852483	OR5H1	0	0
chr3.fa	97868230	97869162	OR5H14	0	0
chr3.fa	97887544	97888485	OR5H15	0	0
chr3.fa	97983129	97984106	OR5H6	0	0
chr3.fa	98001732	98002676	OR5H2	0	0
chr3.fa	98072698	98073663	OR5K4	0	0
chr3.fa	98109510	98110475	OR5K3	0	0
chr3.fa	98188421	98189372	OR5K1	0	0
chr3.fa	98216525	98217475	OR5K2	0	0
chr3.fa	98234317	98241910	CLDND1	6.772768555	1140
chr3.fa	98250878	98251960	GPR15	0	0
chr3.fa	98298290	98312455	CPOX	2.535157062	456
chr3.fa	98451572	98513236	ST3GAL6	2.733053835	380
chr3.fa	98514814	98620533	DCBLD2	3.188638111	1290
chr3.fa	99273153	99717059	MIR548G	0	0
chr3.fa	99357454	99515158	COL8A1	0	0
chr3.fa	99536678	99897476	C3orf26	7.930329131	684
chr3.fa	99551988	99833349	FILIP1L	0	0
chr3.fa	99683158	99683242	MIR3921	0	0
chr3.fa	99904668	99913030	TMEM30C	0	0
chr3.fa	99979686	100044094	TBC1D23	13.87416021	3555
chr3.fa	100053562	100074478	NIT2	32.32524808	2728
chr3.fa	100082307	100120036	TOMM70A	38.96774212	10792
chr3.fa	100120226	100175170	LNP1	0.564472683	76
chr3.fa	100211463	100296285	TMEM45A	2.927336055	304

chr3.fa	100328433	100414323	GPR128	0	0
chr3.fa	100428134	100467810	TFG	48.75278872	6840
chr3.fa	100468179	100712334	ABI3BP	0	0
chr3.fa	100945288	101039419	IMPG2	0	0
chr3.fa	101043118	101232085	SENP7	4.176615912	1368
chr3.fa	101237716	101242724	FAM172B	0	0
chr3.fa	101280712	101285089	RG9MTD1	12.07095012	1292
chr3.fa	101293042	101313281	PCNP	34.412803	5116
chr3.fa	101368283	101391057	ZBTB11	11.44924702	3481
chr3.fa	101395274	101398057	LOC100009676	0.510103782	76
chr3.fa	101399934	101405563	RPL24	934.3223232	34865
chr3.fa	101431278	101432260	LOC285359	0	0
chr3.fa	101443494	101486181	CEP97	4.15176586	1216
chr3.fa	101498029	101547075	FAM55C	1.550643207	912
chr3.fa	101548457	101579869	NFKBIZ	8.580346233	2280
chr3.fa	101659703	101716770	LOC152225	0	0
chr3.fa	102153859	102198685	ZPLD1	0	0
chr3.fa	103903476	103946105	MIR548A3	0	0
chr3.fa	105085713	105295744	ALCAM	15.40371853	4849
chr3.fa	105377109	105587887	CBLB	11.80302138	3116
chr3.fa	106828637	106959485	LOC100302640	1.898242714	76
chr3.fa	106959539	107045811	LOC344595	2.566332581	684
chr3.fa	107096188	107097481	CCDC54	0	0
chr3.fa	107241783	107530049	BBX	22.23552478	13137
chr3.fa	107560509	107596915	LOC151658	0.650318315	76
chr3.fa	107602052	107647753	LOC285205	0	0
chr3.fa	107761941	107809935	CD47	1.873392663	665
chr3.fa	107879659	107941417	IFT57	46.45619215	9880
chr3.fa	108021332	108097126	HHLA2	0.430282405	76
chr3.fa	108099216	108248169	MYH15	0.647155581	304
chr3.fa	108268718	108308336	KIAA1524	0.835413546	228
chr3.fa	108308492	108413693	DZIP3	5.099381154	1824
chr3.fa	108474486	108476130	RETNLB	0	0
chr3.fa	108541631	108573714	TRAT1	0	0
chr3.fa	108626642	108672677	GUCA1C	0	0
chr3.fa	108677087	108836993	MORC1	0	0
chr3.fa	108855561	108868951	FLJ22763	0.592184255	76
chr3.fa	108897012	108904108	C3orf66	0	0
chr3.fa	109012635	109035364	DPPA2	0	0
chr3.fa	109044988	109056419	DPPA4	0	0
chr3.fa	109128837	109214014	FLJ25363	0	0
chr3.fa	110790865	110853062	PVRL3	6.937080107	760
chr3.fa	111260926	111371206	CD96	0.766285221	228

chr3.fa	111311747	111314166	ZBED2	0	0
chr3.fa	111393523	111565294	PLCXD2	0	0
chr3.fa	111451327	111695364	PHLDB2	10.80781448	4610
chr3.fa	111697828	111712210	ABHD10	16.0175901	2812
chr3.fa	111717586	111732735	TAGLN3	0.726223926	76
chr3.fa	111758465	111800116	TMPRSS7	0	0
chr3.fa	111805182	111837044	C3orf52	4.52210693	684
chr3.fa	111831648	111831745	MIR567	0	0
chr3.fa	111839688	111852152	GCET2	0	0
chr3.fa	111859752	112013074	SLC9A10	0.277567544	76
chr3.fa	112051916	112081658	CD200	0	0
chr3.fa	112182813	112218408	BTLA	0	0
chr3.fa	112251359	112280485	ATG3	24.14310509	1991
chr3.fa	112280895	112303003	SLC35A5	4.282492191	836
chr3.fa	112323409	112359977	CCDC80	0.253018705	76
chr3.fa	112534556	112564797	CD200R1L	0	0
chr3.fa	112641532	112693937	CD200R1	0	0
chr3.fa	112709800	112720221	GTPBP8	5.060976529	456
chr3.fa	112721292	112738555	C3orf17	12.69310504	4096
chr3.fa	112931375	113005776	BOC	0.91538553	228
chr3.fa	113006306	113160361	WDR52	2.858659549	1900
chr3.fa	113161565	113234034	SPICE1	5.672589006	2052
chr3.fa	113251218	113348422	SIDT1	5.678613261	1886
chr3.fa	113367233	113415493	KIAA2018	7.431972645	6764
chr3.fa	113435307	113465120	NAA50	18.84417049	7675
chr3.fa	113465866	113530905	ATP6V1A	40.37154411	12312
chr3.fa	113557681	113666021	GRAMD1C	6.972472604	1900
chr3.fa	113666748	113681827	ZDHHC23	2.425063804	608
chr3.fa	113682984	113775460	KIAA1407	5.469722223	1444
chr3.fa	113775611	113807268	QTRTD1	8.308802944	2204
chr3.fa	113847557	113897899	DRD3	0	0
chr3.fa	113953480	113956425	ZNF80	0	0
chr3.fa	114012833	114029135	TIGIT	0	0
chr3.fa	114035322	114035416	MIR568	12.04850977	76
chr3.fa	114056947	114866127	ZBTB20	16.12406881	5019
chr3.fa	115342151	115440334	GAP43	0	0
chr3.fa	115521210	116164385	LSAMP	1.328348202	836
chr3.fa	116428635	116435887	NCRNA00295	0	0
chr3.fa	118619479	118864898	IGSF11	1.185874574	304
chr3.fa	118864997	118870302	C3orf30	0	0
chr3.fa	118892425	118924000	UPK1B	26.14029619	3572
chr3.fa	118930589	118959752	B4GALT4	13.67279949	2128
chr3.fa	119013220	119138323	ARHGAP31	0.141720596	76

chr3.fa	119149701	119182471	TMEM39A	2.709559241	532
chr3.fa	119187785	119213554	POGLUT1	0	0
chr3.fa	119217368	119243125	C3orf1	66.05685845	6987
chr3.fa	119243140	119278481	CD80	0	0
chr3.fa	119298523	119308792	ADPRH	5.658130794	1292
chr3.fa	119316722	119348658	PLA1A	1.305907853	152
chr3.fa	119360908	119379404	POPDC2	0.696253258	76
chr3.fa	119388372	119396243	COX17	35.17713034	988
chr3.fa	119421869	119485949	C3orf15	0.258139322	76
chr3.fa	119499331	119537332	NR1I2	0.501971038	152
chr3.fa	119540802	119813264	GSK3B	22.51264051	10640
chr3.fa	119884328	119963142	GPR156	0.273049353	76
chr3.fa	120043576	120068186	LRRC58	7.150941155	3648
chr3.fa	120113061	120169918	FSTL1	17.26912905	4332
chr3.fa	120315128	120321258	NDUFB4	99.44960449	4028
chr3.fa	120347015	120401418	HGD	32.49648752	4337
chr3.fa	120405528	120461384	RABL3	13.17564786	3397
chr3.fa	120461558	120501916	GTF2E1	7.925057908	1596
chr3.fa	120627050	121143608	STXBP5L	0.244434142	152
chr3.fa	121150273	121264853	POLQ	0	0
chr3.fa	121286778	121309469	ARGFX	0.225909558	76
chr3.fa	121312170	121349139	FBXO40	0	0
chr3.fa	121350246	121379791	HCLS1	1.689050463	228
chr3.fa	121382048	121468602	GOLGB1	32.74694592	24320
chr3.fa	121488610	121553926	IQCB1	13.23754708	2280
chr3.fa	121554034	121605373	EAF2	28.05420197	1900
chr3.fa	121613171	121663034	SLC15A2	8.551580416	3136
chr3.fa	121706170	121741127	ILDR1	2.37792401	456
chr3.fa	121774221	121839983	CD86	0	0
chr3.fa	121902530	122005344	CASR	0.66793926	228
chr3.fa	122044011	122060815	CSTA	5.536139633	304
chr3.fa	122078436	122102074	CCDC58	9.565010694	456
chr3.fa	122103023	122128961	FAM162A	131.1245343	7296
chr3.fa	122130700	122134882	WDR5B	3.557171903	988
chr3.fa	122140748	122233786	KPNA1	13.87506385	6460
chr3.fa	122246760	122283523	PARP9	8.506699717	4400
chr3.fa	122283195	122294049	DTX3L	26.86200193	9956
chr3.fa	122296449	122355536	PARP15	0.409799939	76
chr3.fa	122399672	122449687	PARP14	19.09733981	9774
chr3.fa	122458846	122512650	HSPBAP1	5.912203744	760
chr3.fa	122513901	122599986	DIRC2	1.028942734	228
chr3.fa	122605360	122611263	LOC100129550	0.193830401	76
chr3.fa	122628043	122746576	SEMA5B	0	0

chr3.fa	122785856	122880953	PDIA5	6.147149685	760
chr3.fa	122920774	122992982	SEC22A	3.350690567	760
chr3.fa	123001143	123167392	ADCY5	0.54880962	228
chr3.fa	123213363	123303924	PTPLB	3.857179797	304
chr3.fa	123331143	123603149	MYLK	2.483197864	1292
chr3.fa	123632274	123680255	CCDC14	3.010621379	836
chr3.fa	123687879	123710199	ROPN1	0	0
chr3.fa	123813558	124440036	KALRN	8.687728576	8493
chr3.fa	124449213	124468119	UMPS	7.199888226	3268
chr3.fa	124481795	124606144	ITGB5	7.128350199	1596
chr3.fa	124624289	124653595	MUC13	1.989961995	380
chr3.fa	124684554	124774802	HEG1	0.500013155	304
chr3.fa	124801480	124931609	SLC12A8	0.978338993	228
chr3.fa	124944513	125094198	ZNF148	12.83377139	8132
chr3.fa	125165494	125239058	SNX4	31.67869492	5309
chr3.fa	125247702	125314381	OSBPL11	8.144039573	2508
chr3.fa	125509247	125509395	MIR548I1	0	0
chr3.fa	125635444	125648867	LOC100125556	0	0
chr3.fa	125649400	125655887	ALG1L	1.822638315	76
chr3.fa	125688028	125702296	ROPN1B	1.144608428	76
chr3.fa	125725200	125820391	SLC41A3	7.833037414	1216
chr3.fa	125822408	125899485	ALDH1L1	17.94746015	3724
chr3.fa	126061478	126076236	KLF15	7.252901669	1216
chr3.fa	126113782	126155398	CCDC37	0	0
chr3.fa	126156444	126194762	ZXDC	4.16547104	3800
chr3.fa	126200008	126236616	UROC1	0	0
chr3.fa	126243176	126262134	CHST13	0	0
chr3.fa	126268519	126277758	C3orf22	0	0
chr3.fa	126290622	126291485	TXNRD3IT1	0	0
chr3.fa	126325895	126373967	TXNRD3	4.508100537	760
chr3.fa	126380924	126390782	C3orf46	0	0
chr3.fa	126423118	126679244	CHCHD6	3.629764175	228
chr3.fa	126707437	126756235	PLXNA1	3.030049601	1824
chr3.fa	127291908	127309496	TPRA1	3.734435603	456
chr3.fa	127317253	127341278	MCM2	2.99977772	684
chr3.fa	127348039	127391652	PODXL2	2.636063331	380
chr3.fa	127391781	127399769	ABTB1	3.456265634	456
chr3.fa	127407909	127542051	MGLL	7.004401155	2204
chr3.fa	127641902	127706514	KBTBD12	0	0
chr3.fa	127771212	127790526	SEC61A1	17.62877707	4256
chr3.fa	127799800	127842671	RUVBL1	10.28174642	1207
chr3.fa	127872313	128127489	EEFSEC	7.231214351	1064
chr3.fa	128081008	128081101	MIR1280	0	0

chr3.fa	128181282	128186091	DNAJB8	0	0
chr3.fa	128198265	128212030	GATA2	79.67257813	19912
chr3.fa	128226678	128229429	LOC90246	3.743321379	684
chr3.fa	128290843	128294929	C3orf27	0	0
chr3.fa	128338813	128369719	RPN1	22.98012268	3572
chr3.fa	128444979	128533641	RAB7A	145.4309348	21553
chr3.fa	128580351	128590384	LOC653712	0	0
chr3.fa	128598333	128631957	ACAD9	23.71598542	4332
chr3.fa	128689782	128712986	KIAA1257	0.668541686	76
chr3.fa	128720472	128759585	CCDC48	0.425312395	76
chr3.fa	128779645	128781253	GP9	0	0
chr3.fa	128806418	128840619	RAB43	2.5738629	760
chr3.fa	128846259	128880073	ISY1	7.009220559	1789
chr3.fa	128886658	128902810	CNBP	79.48672987	17860
chr3.fa	128968453	128996616	COPG	108.0847714	22061
chr3.fa	128997684	129024135	C3orf37	17.24924901	2128
chr3.fa	129033614	129035113	H1FX	51.88886521	5168
chr3.fa	129035121	129043412	C3orf47	0.523658356	76
chr3.fa	129101677	129118282	RPL32P3	0.551671141	76
chr3.fa	129116053	129116191	SNORA7B	0	0
chr3.fa	129120164	129147494	C3orf25	2.12550773	304
chr3.fa	129149793	129158852	MBD4	9.731430735	1596
chr3.fa	129158968	129239191	IFT122	3.293008327	912
chr3.fa	129247482	129254187	RHO	0	0
chr3.fa	129262057	129270204	H1FOO	0	0
chr3.fa	129274056	129325582	PLXND1	0.328171285	152
chr3.fa	129366635	129612419	TMCC1	21.3189344	8908
chr3.fa	129693114	129696776	TRH	0	0
chr3.fa	129800674	129816093	ALG1L2	0	0
chr3.fa	129816625	129830276	LOC729375	0.587515458	76
chr3.fa	129931663	129992649	COL6A4P2	0	0
chr3.fa	130064359	130203688	COL6A5	0	0
chr3.fa	130279178	130395888	COL6A6	0	0
chr3.fa	130397778	130465696	PIK3R4	12.94747921	4332
chr3.fa	130569369	130735555	ATP2C1	18.17367092	7309
chr3.fa	130732721	130745646	ASTE1	1.387988326	228
chr3.fa	130745694	131069309	NEK11	3.787750259	836
chr3.fa	131080689	131083966	NUDT16P1	6.778491597	1064
chr3.fa	131100515	131107674	NUDT16	22.63207136	10184
chr3.fa	131181045	131221860	MRPL3	42.17656148	4940
chr3.fa	131197941	131198077	SNORA58	0	0
chr3.fa	131253577	131753844	CPNE4	61.24423183	10455
chr3.fa	132036211	132087146	ACPP	276.7086357	70148

chr3.fa	132136553	132257876	DNAJC13	35.40951597	17744
chr3.fa	132276982	132378975	ACAD11	9.978274579	2356
chr3.fa	132316094	132321382	CCRL1	0	0
chr3.fa	132373290	132396944	UBA5	16.87378733	3648
chr3.fa	132399454	132441276	NPHP3	5.581321545	1976
chr3.fa	132446337	132593050	NCRNA00119	0	0
chr3.fa	132757171	133116619	TMEM108	0	0
chr3.fa	133118790	133194056	BFSP2	0	0
chr3.fa	133292434	133309118	CDV3	33.02014588	9500
chr3.fa	133319449	133380737	TOPBP1	6.209048904	2204
chr3.fa	133464977	133497850	TF	0	0
chr3.fa	133502877	133540336	SRPRB	2.024450854	380
chr3.fa	133543080	133614691	RAB6B	10.07646993	3720
chr3.fa	133646990	133648656	C3orf36	0	0
chr3.fa	133651540	133748920	SLCO2A1	0	0
chr3.fa	133875978	133969586	RYK	16.01126463	3116
chr3.fa	134074190	134093406	AMOTL2	18.60214605	6156
chr3.fa	134196546	134204863	ANAPC13	45.89729191	4940
chr3.fa	134204866	134293855	CEP63	6.741291823	2888
chr3.fa	134318765	134369864	KY	0.401365982	152
chr3.fa	134514099	134979307	EPHB1	0	0
chr3.fa	135684515	135866752	PPP2R3A	5.345020147	2584
chr3.fa	135867760	135914688	MSL2	9.118462801	2888
chr3.fa	135969167	136049013	PCCB	27.45599346	3420
chr3.fa	136055999	136471245	STAG1	10.41352699	3572
chr3.fa	136537861	136574734	TMEM22	0	0
chr3.fa	136581050	136667968	NCK1	7.23377466	988
chr3.fa	136676707	136729926	IL20RB	0	0
chr3.fa	137483579	137484396	SOX14	0	0
chr3.fa	137717658	137752494	CLDN18	0	0
chr3.fa	137780827	137834451	DZIP1L	1.017195437	264
chr3.fa	137842560	137851229	A4GNT	0	0
chr3.fa	137879830	137893791	DBR1	8.510163663	1520
chr3.fa	137906148	138016219	ARMC8	9.623747179	3572
chr3.fa	137980279	138048728	TXNDC6	0.536610504	76
chr3.fa	138066625	138124377	MRAS	2.763476322	836
chr3.fa	138153415	138197256	ESYT3	0	0
chr3.fa	138213187	138313129	CEP70	21.06907843	3724
chr3.fa	138327542	138352213	FAIM	6.220645594	684
chr3.fa	138374231	138478185	PIK3CB	31.70565346	6764
chr3.fa	138663066	138665982	FOXL2	0	0
chr3.fa	138666076	138672830	C3orf72	0	0
chr3.fa	138722804	138725110	PRR23A	0	0

chr3.fa	138737873	138739768	PRR23B	0	0
chr3.fa	138760944	138763734	PRR23C	0	0
chr3.fa	138823027	138844005	BPESC1	0	0
chr3.fa	138951834	138952364	PISRT1	0	0
chr3.fa	139062861	139075887	MRPS22	16.3224174	1216
chr3.fa	139076433	139108522	COPB2	103.2750063	21957
chr3.fa	139171726	139195352	RBP2	0	0
chr3.fa	139236276	139258671	RBP1	2.343133938	304
chr3.fa	139279033	139396840	NMNAT3	3.071918172	380
chr3.fa	139654027	140286919	CLSTN2	4.454635275	1444
chr3.fa	140396866	140419992	TRIM42	0	0
chr3.fa	140660662	140698785	SLC25A36	12.76976369	3952
chr3.fa	140770743	140867453	SPSB4	0	0
chr3.fa	140950682	141013486	ACPL2	3.598287443	760
chr3.fa	141043055	141168632	ZBTB38	26.75853535	14896
chr3.fa	141205926	141331197	RASA2	10.15659252	1748
chr3.fa	141457051	141465243	RNF7	39.37046356	4180
chr3.fa	141497043	141535892	GRK7	0	0
chr3.fa	141595470	141645382	ATP1B3	5.469872829	673
chr3.fa	141663270	141868386	TFDP2	12.26929871	8259
chr3.fa	141876369	141944449	GK5	6.917953097	5016
chr3.fa	142025449	142166853	XRN1	10.8626352	7279
chr3.fa	142168077	142297668	ATR	5.271524237	2888
chr3.fa	142315229	142432505	PLS1	32.99619946	9044
chr3.fa	142443266	142526729	TRPC1	1.688146825	456
chr3.fa	142536702	142608045	PCOLCE2	0	0
chr3.fa	142680074	142682178	PAQR9	0	0
chr3.fa	142719687	142720309	LOC100289361	0	0
chr3.fa	142720372	142779567	SR140	11.26113966	5581
chr3.fa	142838668	142841812	CHST2	0	0
chr3.fa	142984064	143567373	SLC9A9	1.577902961	380
chr3.fa	143690640	143711210	C3orf58	2.242830094	684
chr3.fa	145787228	145879282	PLOD2	1.380307401	372
chr3.fa	145910124	145968966	PLSCR4	1.474888202	336
chr3.fa	146151082	146213722	PLSCR2	0	0
chr3.fa	146232967	146262628	PLSCR1	23.80393954	3493
chr3.fa	146303625	146324003	PLSCR5	0	0
chr3.fa	147103835	147124407	ZIC4	0	0
chr3.fa	147127181	147134506	ZIC1	0	0
chr3.fa	148415658	148460790	AGTR1	0.445192436	76
chr3.fa	148545588	148577972	CPB1	0	0
chr3.fa	148583043	148614872	CPA3	0	0
chr3.fa	148709195	148745456	GYG1	28.30390733	3800

chr3.fa	148747904	148804341	HLTF	11.89097551	3564
chr3.fa	148847371	148890289	HPS3	15.99454733	3648
chr3.fa	148891306	148939832	CP	0	0
chr3.fa	149036285	149051548	TM4SF18	0.297146372	76
chr3.fa	149086805	149095568	TM4SF1	7.445677825	836
chr3.fa	149192434	149221068	TM4SF4	0	0
chr3.fa	149235022	149421060	WWTR1	10.87664159	3876
chr3.fa	149456257	149470286	COMMD2	4.015015275	988
chr3.fa	149478890	149510610	C3orf16	0	0
chr3.fa	149530475	149679925	RNF13	12.35740344	2432
chr3.fa	149682691	149688741	PFN2	62.50239747	8740
chr3.fa	149689066	149691029	LOC646903	0.58284666	76
chr3.fa	150126788	150177615	TSC22D2	18.53407197	4781
chr3.fa	150259780	150264428	SERP1	136.0395732	28652
chr3.fa	150264574	150303803	EIF2A	39.24546027	10108
chr3.fa	150321066	150348234	SELT	11.80678654	2736
chr3.fa	150377675	150421742	FAM194A	0	0
chr3.fa	150458910	150481263	SIAH2	24.90185999	4332
chr3.fa	150643950	150690464	CLRN1	0	0
chr3.fa	150690787	150797617	CLRN1OS	0	0
chr3.fa	150804676	151153777	MED12L	1.540251368	988
chr3.fa	150915619	150920988	GPR171	0	0
chr3.fa	150929905	150996230	P2RY14	0	0
chr3.fa	151011876	151034636	GPR87	0	0
chr3.fa	151044096	151047337	P2RY13	0	0
chr3.fa	151055376	151102544	P2RY12	0	0
chr3.fa	151154466	151176497	IGSF10	0.141268777	76
chr3.fa	151347320	151542394	MIR548H2	0	0
chr3.fa	151451704	151475556	AADACL2	0	0
chr3.fa	151488244	151502682	LOC201651	0	0
chr3.fa	151531861	151546276	AADAC	0	0
chr3.fa	151591431	151599876	SUCNR1	2.08107885	228
chr3.fa	151980405	151987415	LOC401093	0	0
chr3.fa	151986862	152183569	MBNL1	21.41682854	7807
chr3.fa	152057487	152058779	TMEM14E	0	0
chr3.fa	152552736	152555843	P2RY1	3.682777618	760
chr3.fa	152880029	152886263	RAP2B	2.753686908	1140
chr3.fa	153202284	153220486	C3orf79	0	0
chr3.fa	153839149	153975616	ARHGEF26	14.05774938	4836
chr3.fa	153993457	154042286	DHX36	18.92173278	4484
chr3.fa	154055461	154147504	GPR149	0	0
chr3.fa	154797436	154901518	MME	12.96329287	5192
chr3.fa	155197671	155421997	PLCH1	4.027816816	1748

chr3.fa	155480401	155524055	C3orf33	0	0
chr3.fa	155544301	155572248	SLC33A1	5.610840394	912
chr3.fa	155588325	155655520	GMPS	16.31157374	2660
chr3.fa	155838337	156256927	KCNAB1	0	0
chr3.fa	156257929	156272973	SSR3	25.87387352	6384
chr3.fa	156390960	156393502	LOC100287227	0	0
chr3.fa	156394453	156424557	TIPARP	17.7146227	4332
chr3.fa	156527060	156529810	PA2G4P4	0	0
chr3.fa	156544096	156763918	LEKR1	0.562816013	152
chr3.fa	156799456	156840791	LOC339894	0	0
chr3.fa	156807670	156818924	LOC100498859	0	0
chr3.fa	156865586	156878482	CCNL1	8.287868658	1444
chr3.fa	156977532	157221415	VEPH1	5.906179489	1900
chr3.fa	157154580	157161417	PTX3	0	0
chr3.fa	157261159	157319021	C3orf55	0.844148716	152
chr3.fa	157813800	157823952	SHOX2	0	0
chr3.fa	157827892	158262576	RSRC1	16.73507886	1809
chr3.fa	158288953	158324248	MLF1	3.531418214	608
chr3.fa	158362317	158410360	GFM1	13.5358983	3116
chr3.fa	158384203	158390482	LXN	6.153776365	456
chr3.fa	158414897	158450275	RARRES1	2.061048203	228
chr3.fa	158519715	158547508	MFSD1	14.22537427	2231
chr3.fa	158983004	158984096	IQCJ	0	0
chr3.fa	158991036	159558003	SCHIP1	0	0
chr3.fa	159000435	159000523	MIR3919	0	0
chr3.fa	159706629	159713806	IL12A	0	0
chr3.fa	159943423	159946000	LOC401097	0	0
chr3.fa	159974774	160117320	IFT80	4.302974658	1368
chr3.fa	160117430	160152741	SMC4	2.124754698	760
chr3.fa	160122376	160122473	MIR15B	0	0
chr3.fa	160122533	160122613	MIR16-2	0	0
chr3.fa	160153291	160167626	TRIM59	0	0
chr3.fa	160217962	160283376	KPNA4	32.41877463	8184
chr3.fa	160232695	160233024	SCARNA7	0	0
chr3.fa	160394948	160396235	ARL14	7.109373796	608
chr3.fa	160473996	160788817	PPM1L	1.123674142	228
chr3.fa	160801671	160823160	B3GALNT1	1.571878706	380
chr3.fa	160939099	160969795	NMD3	25.59419749	4636
chr3.fa	161062580	161089871	C3orf57	47.15425269	7220
chr3.fa	161214596	161221730	OTOL1	0	0
chr3.fa	162895031	163021089	LOC647107	0	0
chr3.fa	164696686	164796283	SI	0.380883515	152
chr3.fa	164904508	164914469	SLITRK3	0	0

chr3.fa	165490692	165555253	BCHE	0	0
chr3.fa	166958077	167098085	ZBBX	0	0
chr3.fa	167159723	167191818	SERPINI2	0	0
chr3.fa	167196473	167371289	WDR49	0	0
chr3.fa	167401695	167452630	PDCD10	31.56122195	3244
chr3.fa	167453432	167543357	SERPINI1	1.150933896	152
chr3.fa	167613736	167641797	LOC646168	0	0
chr3.fa	167727654	167813417	GOLIM4	5.646383497	1064
chr3.fa	167967310	168548374	EGFEM1P	0.297899404	76
chr3.fa	168269642	168269737	MIR551B	0	0
chr3.fa	168801287	169381563	MECOM	47.3980844	21473
chr3.fa	169482398	169482848	TERC	0	0
chr3.fa	169484711	169487683	ARPM1	1.714352334	228
chr3.fa	169490853	169507504	MYNN	5.971542655	2128
chr3.fa	169511216	169530574	LRRC34	0	0
chr3.fa	169539710	169555560	LRRIQ4	0	0
chr3.fa	169557029	169587660	LRRC31	1.857428387	304
chr3.fa	169629482	169656948	SAMD7	0	0
chr3.fa	169661772	169684522	LOC100128164	0	0
chr3.fa	169684580	169716161	SEC62	12.05122068	5234
chr3.fa	169755735	169803183	GPR160	32.97933155	4408
chr3.fa	169805368	169899537	PHC3	16.89095645	14212
chr3.fa	169940220	170023770	PRKCI	21.32977806	6917
chr3.fa	170075473	170110950	SKIL	42.10939104	10032
chr3.fa	170136653	170152479	CLDN11	0	0
chr3.fa	170177342	170303863	SLC7A14	0	0
chr3.fa	170582665	170588045	RPL22L1	7.000635996	912
chr3.fa	170606204	170626426	EIF5A2	0.413414491	152
chr3.fa	170714137	170744768	SLC2A2	0	0
chr3.fa	170780292	171178197	TNIK	6.201367979	2508
chr3.fa	170824453	170824548	MIR569	0	0
chr3.fa	171318618	171528273	PLD1	4.094535439	1520
chr3.fa	171561139	171577108	TMEM212	0	0
chr3.fa	171757418	172118492	FNDC3B	25.0343936	11777
chr3.fa	172161081	172166246	GHSR	0	0
chr3.fa	172223298	172241297	TNFSF10	5.603460681	1140
chr3.fa	172348435	172429008	NCEH1	5.570477886	1596
chr3.fa	172472298	172539263	ECT2	5.560688472	1444
chr3.fa	172607147	172859058	SPATA16	0	0
chr3.fa	173116244	174001116	NLGN1	0	0
chr3.fa	174577111	175523428	NAALADL2	5.592617023	1824
chr3.fa	176738542	176915048	TBL1XR1	42.85730228	18639
chr3.fa	178254224	178562217	KCNMB2	0	0

chr3.fa	178735011	178789656	ZMAT3	5.901360085	3572
chr3.fa	178866311	178952497	PIK3CA	15.73881771	3876
chr3.fa	178957537	178984838	KCNMB3	0.654987112	152
chr3.fa	179041551	179053320	ZNF639	4.178724401	836
chr3.fa	179065480	179111008	MFN1	22.80752778	5320
chr3.fa	179113876	179169371	GNB4	1.410428675	602
chr3.fa	179280708	179306193	ACTL6A	20.9454306	2584
chr3.fa	179306255	179322434	MRPL47	11.60181127	909
chr3.fa	179322575	179342288	NDUFB5	34.38780234	2418
chr3.fa	179370933	179507189	USP13	5.173328883	2725
chr3.fa	179518050	179754517	PEX5L	0	0
chr3.fa	180319918	180328918	TTC14	0.956501069	304
chr3.fa	180331796	180397283	CCDC39	0	0
chr3.fa	180630234	180700539	FXR1	15.37510332	9095
chr3.fa	180701498	180707562	DNAJC19	10.60253799	1748
chr3.fa	181328151	181459005	SOX2OT	0.45874701	76
chr3.fa	181429722	181432223	SOX2	0.457542159	76
chr3.fa	182511291	182639421	ATP11B	17.73540638	8620
chr3.fa	182660559	182698326	DCUN1D1	9.078401506	1900
chr3.fa	182733006	182817365	MCCC1	36.57325141	6156
chr3.fa	182840003	182880667	LAMP3	0.342629497	76
chr3.fa	182895831	183145855	MCF2L2	0	0
chr3.fa	182971032	182991179	B3GNT5	1.665706476	456
chr3.fa	183205319	183273499	KLHL6	0	0
chr3.fa	183353411	183402304	KLHL24	14.19751209	6908
chr3.fa	183415606	183530413	YEATS2	12.44475514	5376
chr3.fa	183533664	183543393	MAP6D1	1.070660699	152
chr3.fa	183547173	183602693	PARL	29.16130941	2734
chr3.fa	183637724	183735727	ABCC5	4.377374206	2052
chr3.fa	183749332	183757157	HTR3D	0	0
chr3.fa	183770835	183778461	HTR3C	0	0
chr3.fa	183817967	183824783	HTR3E	0	0
chr3.fa	183852810	183863099	EIF2B5	20.93308087	4028
chr3.fa	183873284	183891314	DVL3	20.5767462	6916
chr3.fa	183892634	183901879	AP2M1	119.1195497	15352
chr3.fa	183903863	183911795	ABCF3	17.32681129	2959
chr3.fa	183948317	183960117	VWA5B2	0	0
chr3.fa	183959193	183959277	MIR1224	0	0
chr3.fa	183960117	183967313	ALG3	5.620027382	684
chr3.fa	183967445	184010819	ECE2	0	0
chr3.fa	183977003	183979251	CAMK2N2	0	0
chr3.fa	184017022	184026840	PSMD2	63.39699932	12300
chr3.fa	184032283	184053146	EIF4G1	68.04426014	27953

chr3.fa	184043484	184043559	SNORD66	0	0
chr3.fa	184053717	184063975	FAM131A	4.212761441	760
chr3.fa	184064064	184079391	CLCN2	0.738423043	152
chr3.fa	184081194	184086363	POLR2H	34.85407967	1900
chr3.fa	184089773	184095932	THPO	0	0
chr3.fa	184097861	184107617	CHRD	0	0
chr3.fa	184279587	184300196	EPHB3	1.356210381	380
chr3.fa	184428155	184429836	MAGEF1	21.77617535	2432
chr3.fa	184529931	184770402	VPS8	11.98465267	4013
chr3.fa	184795838	184870802	C3orf70	1.551697452	608
chr3.fa	184908412	184971886	EHHADH	21.89530499	5776
chr3.fa	185080970	185200628	MAP3K13	8.688331002	2052
chr3.fa	185207389	185216845	TMEM41A	3.643921174	684
chr3.fa	185225570	185270369	LIPH	9.253104897	1520
chr3.fa	185304031	185348885	SENP2	19.63922153	4165
chr3.fa	185361527	185542827	IGF2BP2	1.558926558	380
chr3.fa	185431040	185435955	C3orf65	0	0
chr3.fa	185632358	185655924	TRA2B	18.65726799	5312
chr3.fa	185686267	185697578	LOC344887	0	0
chr3.fa	185764106	185826901	ETV5	1.674291039	456
chr3.fa	185864990	186080023	DGKG	0	0
chr3.fa	186172770	186211450	LOC253573	0	0
chr3.fa	186256232	186262167	CRYGS	0	0
chr3.fa	186263856	186288332	TBCCD1	2.907004195	608
chr3.fa	186288467	186303589	DNAJB11	8.132141669	912
chr3.fa	186330850	186339107	AHSG	0	0
chr3.fa	186358149	186370797	FETUB	0	0
chr3.fa	186383798	186396023	HRG	1.17277182	152
chr3.fa	186435098	186462199	KNG1	5.875305183	836
chr3.fa	186501361	186507681	EIF4A2	249.7782585	31279
chr3.fa	186502585	186502654	SNORD2	0	0
chr3.fa	186504461	186504463	MIR1248	0	0
chr3.fa	186504567	186504641	SNORA81	0	0
chr3.fa	186505088	186505222	SNORA63	0	0
chr3.fa	186505402	186505538	SNORA4	0	0
chr3.fa	186507686	186524484	RFC4	2.171894492	228
chr3.fa	186560463	186576252	ADIPOQ	0	0
chr3.fa	186648315	186796341	ST6GAL1	6.289020887	1976
chr3.fa	186838741	186857263	RPL39L	3.256410978	152
chr3.fa	186915274	186919253	RTP1	0	0
chr3.fa	186933873	187009810	MASP1	0.343533135	228
chr3.fa	187086168	187089369	RTP4	1.132108099	76
chr3.fa	187386694	187388201	SST	0	0

chr3.fa	187416047	187420345	RTP2	0	0
chr3.fa	187433410	187450203	LOC100131635	0	0
chr3.fa	187439165	187463513	BCL6	22.32378012	5892
chr3.fa	187868994	187871876	LOC339929	2.9283903	532
chr3.fa	187896331	187898596	FLJ42393	0	0
chr3.fa	187930721	188608460	LPP	17.60452944	21432
chr3.fa	188889763	189041271	TPRG1	0	0
chr3.fa	189349216	189615068	TP63	1.929870052	836
chr3.fa	189547711	189547798	MIR944	0	0
chr3.fa	189674517	189840226	LEPREL1	17.33087767	4527
chr3.fa	190023490	190040235	CLDN1	1.326239713	304
chr3.fa	190105661	190129932	CLDN16	0	0
chr3.fa	190146444	190167665	TMEM207	2.371447936	228
chr3.fa	190231840	190374986	IL1RAP	0	0
chr3.fa	190570526	190580465	GEMC1	0.631643125	152
chr3.fa	190595719	190595839	SNAR-I	0	0
chr3.fa	190930322	190967910	OSTN	0	0
chr3.fa	190984944	191048325	UTS2D	0	0
chr3.fa	191046874	191116459	CCDC50	6.139318153	3648
chr3.fa	191178952	191179245	PYDC2	0	0
chr3.fa	191857182	192445388	FGF12	0.533748983	228
chr3.fa	192514605	192635950	C3orf59	10.1984611	2280
chr3.fa	192958918	192988644	HRASLS	0	0
chr3.fa	192959568	192961761	MGC2889	0	0
chr3.fa	192992831	193096514	ATP13A5	0	0
chr3.fa	193119866	193272696	ATP13A4	0	0
chr3.fa	193310933	193415600	OPA1	17.08328079	7365
chr3.fa	193675161	193721448	LOC647323	0	0
chr3.fa	193710883	193712027	LOC100128023	0	0
chr3.fa	193853934	193856396	HES1	74.83675813	7220
chr3.fa	194018989	194030593	LOC100131551	0	0
chr3.fa	194060494	194072057	CPN2	1.134065982	228
chr3.fa	194075976	194090472	LRRC15	0	0
chr3.fa	194115550	194119995	GP5	0	0
chr3.fa	194123403	194188968	ATP13A3	7.532427096	3665
chr3.fa	194207869	194209274	FLJ34208	2.038457247	152
chr3.fa	194308402	194354150	TMEM44	0.909813094	152
chr3.fa	194361517	194393206	LSG1	17.24473082	4090
chr3.fa	194406622	194409766	FAM43A	8.370702163	1748
chr3.fa	194789013	194991895	C3orf21	0	0
chr3.fa	194995465	195163817	ACAP2	15.54799943	7402
chr3.fa	195241221	195270224	PPP1R2	10.23445602	2356
chr3.fa	195295573	195311076	APOD	1.012978459	76

chr3.fa	195384910	195415735	SDHAP2	2.982006168	456
chr3.fa	195426272	195426368	MIR570	0	0
chr3.fa	195447753	195464540	MUC20	1.549739569	684
chr3.fa	195473638	195539148	MUC4	0	0
chr3.fa	195590236	195635880	TNK2	12.77834825	4104
chr3.fa	195686792	195717150	SDHAP1	8.271000745	1368
chr3.fa	195776155	195809032	TFRC	16.61655164	5842
chr3.fa	195869507	195887761	LOC401109	2.504583968	304
chr3.fa	195924323	195938300	ZDHHC19	0	0
chr3.fa	195943383	195960301	OSTalpha	2.360001851	228
chr3.fa	195965253	196014584	PCYT1A	20.24571339	2128
chr3.fa	196018090	196045159	TCTEX1D2	0	0
chr3.fa	196050419	196065258	TM4SF19	0	0
chr3.fa	196080369	196159345	UBXN7	15.48082899	4864
chr3.fa	196195657	196230639	RNF168	12.20860434	4332
chr3.fa	196233750	196242237	C3orf43	0	0
chr3.fa	196281059	196295413	WDR53	2.914383907	304
chr3.fa	196295725	196315930	FBXO45	4.591988286	1748
chr3.fa	196366656	196388874	LRRC33	0	0
chr3.fa	196433148	196439123	C3orf34	0.529983824	76
chr3.fa	196439245	196462876	PIGX	3.330509313	684
chr3.fa	196466728	196559518	PAK2	22.72213397	9262
chr3.fa	196594727	196661584	SENP5	11.40948693	4778
chr3.fa	196662273	196669464	NCBP2	29.01085364	4180
chr3.fa	196669494	196670884	LOC152217	11.52018262	1064
chr3.fa	196673214	196695704	PIGZ	0	0
chr3.fa	196728612	196756686	MFI2	0.475916136	152
chr3.fa	196769431	197025447	DLG1	37.56243406	12640
chr3.fa	197236654	197300194	BDH1	13.65216642	3344
chr3.fa	197340898	197354752	LOC220729	1.499135828	152
chr3.fa	197398259	197476568	KIAA0226	10.57045883	4932
chr3.fa	197476624	197511317	FYTTD1	18.01267271	5156
chr3.fa	197518145	197598456	LRCH3	19.45608418	2917
chr3.fa	197615946	197686886	IQCG	3.416806764	532
chr3.fa	197677052	197682721	RPL35A	1318.476105	42284
chr3.fa	197687071	197770591	LMLN	1.139186599	532
chr3.fa	197784404	197807542	LOC348840	0	0
chr3.fa	197879237	197907728	FAM157A	0	0
chr4.fa	53227	88099	ZNF595	4.527679365	760
chr4.fa	154702	156490	ZNF718	1.279551738	152
chr4.fa	206389	249773	ZNF876P	0.439017575	76
chr4.fa	264464	289944	ZNF732	0	0
chr4.fa	331596	367691	ZNF141	0.697156897	76

chr4.fa	419224	467998	ABCA11P	3.415903126	401
chr4.fa	433779	493442	ZNF721	5.870786992	1852
chr4.fa	494185	533320	PIGG	5.413696653	1064
chr4.fa	619363	664681	PDE6B	2.582296857	608
chr4.fa	666225	668127	ATP5I	119.3297962	2892
chr4.fa	671711	674929	MYL5	10.67151571	372
chr4.fa	675618	682973	MFSD7	0.661613793	76
chr4.fa	699573	764427	PCGF3	15.69333458	5903
chr4.fa	773937	775636	LOC100129917	0	0
chr4.fa	778745	819945	CPLX1	0	0
chr4.fa	843065	926174	GAK	25.00909173	7524
chr4.fa	926262	952443	TMEM175	1.810288593	213
chr4.fa	952675	967344	DGKQ	3.950405141	1216
chr4.fa	972863	987183	SLC26A1	0.878637575	228
chr4.fa	980785	998317	IDUA	0	0
chr4.fa	1005610	1020686	FGFRL1	1.767968202	380
chr4.fa	1065266	1107352	RNF212	1.058762796	214
chr4.fa	1108985	1116952	TMED11P	0	0
chr4.fa	1160721	1166999	SPON2	8.513928823	818
chr4.fa	1189571	1198904	LOC100130872	0.498356485	152
chr4.fa	1205228	1242908	CTBP1	51.16851493	8436
chr4.fa	1243228	1246795	C4orf42	7.031660908	1368
chr4.fa	1283672	1333925	MAEA	29.80485044	4332
chr4.fa	1341104	1381837	KIAA1530	4.171194082	1292
chr4.fa	1385340	1389782	CRIPAK	2.576272602	760
chr4.fa	1641608	1685988	FAM53A	0.807250155	152
chr4.fa	1694527	1714030	SLBP	17.25211053	1976
chr4.fa	1717679	1723084	TMEM129	3.227645161	608
chr4.fa	1723266	1746897	TACC3	0.823515643	152
chr4.fa	1795039	1810599	FGFR3	6.963586828	2052
chr4.fa	1813206	1857974	LETM1	8.801436387	3192
chr4.fa	1873123	1983934	WHSC1	3.418312828	3876
chr4.fa	1976363	1976487	SCARNA22	0	0
chr4.fa	1984443	2010959	WHSC2	9.835047919	1596
chr4.fa	1988130	1988204	MIR943	0	0
chr4.fa	2043720	2045697	C4orf48	0	0
chr4.fa	2061239	2070816	NAT8L	2.144484133	836
chr4.fa	2073645	2214853	POLN	1.655314636	304
chr4.fa	2230096	2243860	HAUS3	2.093729785	760
chr4.fa	2249160	2263739	MXD4	23.9661426	6004
chr4.fa	2271324	2420370	ZFYVE28	0.585557575	228
chr4.fa	2420701	2464690	LOC402160	0	0
chr4.fa	2470795	2517586	RNF4	19.12715987	4666

chr4.fa	2627159	2734302	FAM193A	11.12438907	3496
chr4.fa	2743387	2758103	TNIP2	17.40211448	2356
chr4.fa	2794750	2842823	SH3BP2	4.092125737	2660
chr4.fa	2845584	2931789	ADD1	65.84028649	17784
chr4.fa	2932288	2936586	MFSD10	1.116746249	152
chr4.fa	2937278	2952794	C4orf10	5.74216915	1368
chr4.fa	2939870	2965118	NOP14	32.65025663	5745
chr4.fa	2965343	3042474	GRK4	0.989333259	152
chr4.fa	3076408	3245687	HTT	17.87607273	15994
chr4.fa	3250767	3258342	C4orf44	0.6854096	76
chr4.fa	3315874	3441640	RGS12	5.540507218	2584
chr4.fa	3443726	3451213	HGFAC	0.561611162	76
chr4.fa	3465033	3496209	DOK7	0	0
chr4.fa	3514290	3534224	LRPAP1	11.66476473	1216
chr4.fa	3675320	3679582	LOC100133461	0	0
chr4.fa	3768296	3770253	ADRA2C	0	0
chr4.fa	3943669	3957148	LOC348926	0.670800781	152
chr4.fa	4190530	4228621	OTOP1	0	0
chr4.fa	4237269	4249934	TMEM128	3.304303804	380
chr4.fa	4269429	4291896	LYAR	4.995161544	532
chr4.fa	4291924	4323513	ZBTB49	1.944629477	380
chr4.fa	4387983	4420695	D4S234E	1.370819199	228
chr4.fa	4420786	4543775	STX18	16.53974239	2270
chr4.fa	4861392	4865660	MSX1	0.590979404	76
chr4.fa	5016314	5021197	CYTL1	0	0
chr4.fa	5053527	5502725	STK32B	2.485155746	532
chr4.fa	5526883	5529527	C4orf6	0	0
chr4.fa	5564146	5711275	EVC2	0.892493361	304
chr4.fa	5712924	5816031	EVC	4.45026769	1900
chr4.fa	5822491	5894785	CRMP1	0.652728017	152
chr4.fa	6027926	6202318	JAKMIP1	11.77154465	2584
chr4.fa	6271577	6304992	WFS1	4.716840969	1140
chr4.fa	6322305	6474326	PPP2R2C	4.040919571	1216
chr4.fa	6576902	6624188	MAN2B2	1.888754513	532
chr4.fa	6642445	6644449	MRFAP1	211.9091898	21584
chr4.fa	6675821	6677774	LOC93622	14.4104695	1596
chr4.fa	6695566	6698897	S100P	374.8030841	12692
chr4.fa	6709429	6711606	MRFAP1L1	51.7988026	5472
chr4.fa	6717842	6719387	CNO	8.144039573	836
chr4.fa	6784459	6885899	KIAA0232	26.80507272	13984
chr4.fa	6911171	7032280	TBC1D14	30.16600452	5396
chr4.fa	7034846	7047958	LOC100129931	0	0
chr4.fa	7042576	7044728	CCDC96	1.063280987	152

chr4.fa	7045156	7059677	TADA2B	14.34073875	4104
chr4.fa	7061780	7069800	GRPEL1	23.59233759	2356
chr4.fa	7099151	7105103	FLJ36777	0	0
chr4.fa	7194374	7744564	SORCS2	0.189161603	76
chr4.fa	7432021	7436700	PSAPL1	0	0
chr4.fa	7461755	7461845	MIR4274	0	0
chr4.fa	7755817	7780654	AFAP1-AS1	0	0
chr4.fa	7760440	7941653	AFAP1	5.802261093	2812
chr4.fa	7967037	8160559	ABLIM2	0	0
chr4.fa	8201060	8242830	SH3TC1	3.224181215	912
chr4.fa	8271489	8308838	HTRA3	0	0
chr4.fa	8368009	8442452	ACOX3	28.67500143	5472
chr4.fa	8442532	8478282	C4orf23	6.032688842	1140
chr4.fa	8582291	8589520	GPR78	0	0
chr4.fa	8594387	8621488	CPZ	0	0
chr4.fa	8868773	8873543	HMX1	0	0
chr4.fa	8951477	8952127	LOC650293	0	0
chr4.fa	9217131	9366447	USP17	0	0
chr4.fa	9369600	9370796	USP17L6P	0	0
chr4.fa	9446260	9452240	DEFB131	0	0
chr4.fa	9557789	9557937	MIR548I2	0	0
chr4.fa	9783258	9785633	DRD5	0	0
chr4.fa	9827848	10041872	SLC2A9	3.303400166	456
chr4.fa	10075963	10118573	WDR1	98.69190384	20596
chr4.fa	10080235	10080316	MIR3138	0	0
chr4.fa	10441504	10459032	ZNF518B	7.426098997	3420
chr4.fa	10491838	10686386	CLNK	0	0
chr4.fa	11370451	11370545	MIR572	0	0
chr4.fa	11399988	11430537	HS3ST1	0.582545447	76
chr4.fa	13335037	13339925	HSP90AB2P	0.468235211	152
chr4.fa	13369347	13485989	RAB28	9.720737683	1216
chr4.fa	13527943	13533041	LOC285547	0	0
chr4.fa	13542454	13546114	NKX3-2	0	0
chr4.fa	13547700	13549448	LOC285548	0	0
chr4.fa	13570366	13629328	BOD1L	12.75771518	8930
chr4.fa	14113592	14141676	LOC152742	0	0
chr4.fa	15004298	15071777	CPEB2	9.438350735	4316
chr4.fa	15341560	15447791	C1QTNF7	0.236602611	76
chr4.fa	15471489	15603180	CC2D2A	2.855647422	1216
chr4.fa	15606007	15657035	FBXL5	65.64856458	15884
chr4.fa	15683352	15692070	FAM200B	4.698466991	1368
chr4.fa	15704573	15733796	BST1	2.320091163	228
chr4.fa	15779931	15850706	CD38	3.070713321	304

chr4.fa	15937193	15939971	FGFBP1	0	0
chr4.fa	15961863	15964859	FGFBP2	0	0
chr4.fa	15969849	16085623	PROM1	7.497034598	2144
chr4.fa	16162128	16228161	TAPT1	3.243157617	988
chr4.fa	16228286	16259810	FLJ39653	1.348228243	228
chr4.fa	16503165	16900424	LDB2	0	0
chr4.fa	17488016	17513857	QDPR	62.31986254	6840
chr4.fa	17516788	17528727	CLRN2	0	0
chr4.fa	17578927	17609590	LAP3	31.55564951	4400
chr4.fa	17616273	17626160	MED28	21.44469072	1824
chr4.fa	17633709	17783135	FAM184B	1.191748223	304
chr4.fa	17802278	17812381	DCAF16	9.815318484	2964
chr4.fa	17812525	17844002	NCAPG	0	0
chr4.fa	17844839	18023483	LCORL	2.22761885	912
chr4.fa	20255235	20620788	SLIT2	1.156205119	380
chr4.fa	20529898	20530007	MIR218-1	0	0
chr4.fa	20702036	20729980	PACRGL	6.625776735	912
chr4.fa	20730239	21950374	KCNIP4	0	0
chr4.fa	21844964	21854811	NCRNA00099	0	0
chr4.fa	22388999	22517672	GPR125	4.016220126	1216
chr4.fa	22694548	22821192	GBA3	21.85795461	3116
chr4.fa	23793644	23891700	PPARGC1A	5.978470548	2508
chr4.fa	24521815	24521913	MIR573	0	0
chr4.fa	24529088	24586184	DHX15	45.17694163	9044
chr4.fa	24797085	24802467	SOD3	7.486040333	760
chr4.fa	24807739	24981826	CCDC149	16.12677972	4523
chr4.fa	25000471	25032414	LGI2	0	0
chr4.fa	25121627	25162204	SEPSECS	6.08299137	2280
chr4.fa	25235653	25280831	PI4K2B	6.357094968	1520
chr4.fa	25314396	25372005	ZCCHC4	4.501473857	836
chr4.fa	25378848	25420120	ANAPC4	5.568821216	988
chr4.fa	25657435	25680368	SLC34A2	0.269133587	76
chr4.fa	25749049	25864610	SEL1L3	5.284024566	1596
chr4.fa	25915814	25931501	C4orf52	41.52820105	2796
chr4.fa	26321332	26433278	RBPJ	22.94126624	4256
chr4.fa	26483018	26492042	CCKAR	0	0
chr4.fa	26585546	26756918	TBC1D19	4.080077227	596
chr4.fa	26862313	27027003	STIM2	2.400063146	836
chr4.fa	28821204	28821290	MIR4275	0	0
chr4.fa	30722037	31148423	PCDH7	0.24910294	152
chr4.fa	36067622	36245979	ARAP2	11.04170617	5396
chr4.fa	36283244	36347378	DTHD1	0	0
chr4.fa	37246690	37451087	KIAA1239	0	0

chr4.fa	37455552	37595132	C4orf19	5.750301894	1289
chr4.fa	37612256	37687999	RELL1	1.898242714	456
chr4.fa	37828282	37864559	PGM2	27.57241218	5928
chr4.fa	37892720	38140794	TBC1D1	66.20535634	25004
chr4.fa	37962056	37962631	PTTG2	0	0
chr4.fa	38614322	38666249	FLJ13197	0	0
chr4.fa	38665790	38703129	KLF3	23.32998129	8255
chr4.fa	38773860	38784611	TLR10	0	0
chr4.fa	38797876	38806412	TLR1	0	0
chr4.fa	38825329	38858437	TLR6	0	0
chr4.fa	38869354	38947365	FAM114A1	24.88032328	6836
chr4.fa	38869653	38869748	MIR574	0	0
chr4.fa	38968441	39034041	TMEM156	0	0
chr4.fa	39046451	39127853	KLHL5	3.297375911	1748
chr4.fa	39184024	39287430	WDR19	8.35669577	2508
chr4.fa	39289076	39367995	RFC1	21.79982055	7068
chr4.fa	39408473	39453153	KLB	0	0
chr4.fa	39455745	39460568	RPL9	26.59136228	1582
chr4.fa	39460665	39479271	LIAS	5.961452028	684
chr4.fa	39481875	39483523	LOC401127	0	0
chr4.fa	39500375	39529218	UGDH	39.03702105	8284
chr4.fa	39552546	39640481	C4orf34	72.17840506	7946
chr4.fa	39699664	39784410	UBE2K	22.01217553	7663
chr4.fa	39824483	39979576	PDS5A	29.96855956	15111
chr4.fa	40044537	40058819	LOC344967	0	0
chr4.fa	40058524	40157019	N4BP2	6.08630471	2725
chr4.fa	40198527	40246281	RHOH	2.290421707	304
chr4.fa	40337469	40356973	CHRNA9	0	0
chr4.fa	40425272	40631883	RBM47	73.28280158	25536
chr4.fa	40751914	40812002	NSUN7	6.499869808	1596
chr4.fa	40812044	41216635	APBB2	4.109746683	2505
chr4.fa	41258898	41270446	UCHL1	11.14803427	832
chr4.fa	41362804	41702061	LIMCH1	55.98611156	23650
chr4.fa	41746099	41750987	PHOX2B	0	0
chr4.fa	41937137	41962824	TMEM33	2.373104606	1216
chr4.fa	41983713	41988484	DCAF4L1	0	0
chr4.fa	41992523	42089551	SLC30A9	33.65434931	7287
chr4.fa	42112870	42154895	BEND4	8.406697086	4940
chr4.fa	42399856	42404504	SHISA3	1.161476342	152
chr4.fa	42410392	42659122	ATP8A1	3.422981626	1896
chr4.fa	42895283	43032673	GRXCR1	0	0
chr4.fa	44175920	44450824	KCTD8	0	0
chr4.fa	44624354	44653658	YIPF7	0	0

chr4.fa	44680433	44702697	GUF1	8.144792605	2280
chr4.fa	44704168	44728612	GNPDA2	5.370924443	684
chr4.fa	46037787	46126082	GABRG1	0	0
chr4.fa	46251581	46392056	GABRA2	0.371847133	76
chr4.fa	46736847	46911252	COX7B2	0	0
chr4.fa	46920917	46995580	GABRA4	0	0
chr4.fa	47033295	47428447	GABRB1	0	0
chr4.fa	47452811	47465676	COMMD8	14.6017396	1368
chr4.fa	47487410	47595503	ATP10D	2.446449909	1064
chr4.fa	47596018	47840059	CORIN	0	0
chr4.fa	47849258	47916633	NFXL1	17.92170646	4435
chr4.fa	47937994	48014961	CNGA1	9.09316093	1824
chr4.fa	48018791	48039080	NIPAL1	17.75649127	2582
chr4.fa	48068410	48136273	TXK	2.356839117	456
chr4.fa	48137800	48271814	TEC	4.289420085	1031
chr4.fa	48343613	48428215	SLAIN2	28.18206678	11248
chr4.fa	48485360	48491541	SLC10A4	0	0
chr4.fa	48492309	48496422	ZAR1	0.797008921	76
chr4.fa	48499380	48782316	FRYL	14.4624287	11241
chr4.fa	48833060	48863834	OCIAD1	13.96753616	2121
chr4.fa	48887405	48908815	OCIAD2	91.32981255	4633
chr4.fa	48988265	49064095	CWH43	17.14593304	2812
chr4.fa	52709276	52783003	DCUN1D4	9.619078381	2724
chr4.fa	52859866	52883786	LRRC66	0	0
chr4.fa	52886861	52904485	SGCB	2.942396692	836
chr4.fa	52917593	52963458	SPATA18	3.992876138	1140
chr4.fa	53457127	53525502	USP46	13.16118965	7220
chr4.fa	53578621	53580305	KIAA0114	124.5013178	7068
chr4.fa	53579416	53579537	SNORA26	0	0
chr4.fa	53728495	53733002	RASL11B	25.14975808	3268
chr4.fa	53739151	54232242	SCFD2	5.048626806	1064
chr4.fa	54243820	54326103	FIP1L1	29.06070435	4795
chr4.fa	54326437	54457724	LNX1	10.53717483	2432
chr4.fa	54851666	54853449	RPL21P44	0	0
chr4.fa	54875958	54930788	CHIC2	5.09621842	380
chr4.fa	54966248	54968122	GSX2	0	0
chr4.fa	55095264	55164412	PDGFRA	0	0
chr4.fa	55524095	55606881	KIT	0.664927133	228
chr4.fa	55944426	55991762	KDR	0	0
chr4.fa	56212409	56237866	SRD5A3	1.290847216	228
chr4.fa	56262090	56292342	TMEM165	8.155033838	1064
chr4.fa	56298660	56412997	CLOCK	11.4441264	4408
chr4.fa	56422692	56458379	PDCL2	0	0

chr4.fa	56461398	56502465	NMU	1.384223166	75
chr4.fa	56686237	56703430	LOC644145	0	0
chr4.fa	56719816	56771244	EXOC1	19.37746766	4408
chr4.fa	56815037	56899527	CEP135	3.431867402	1292
chr4.fa	57036361	57196890	KIAA1211	0.326364008	152
chr4.fa	57204457	57253638	AASDH	6.428632994	1520
chr4.fa	57259529	57301845	PPAT	1.222471923	304
chr4.fa	57301915	57327534	PAICS	10.08324722	2356
chr4.fa	57333762	57369847	SRP72	19.78937608	5093
chr4.fa	57371375	57390058	ARL9	0	0
chr4.fa	57514154	57547872	HOPX	22.33673226	2812
chr4.fa	57676034	57687893	SPINK2	0	0
chr4.fa	57774042	57802010	REST	20.71349678	10260
chr4.fa	57829516	57843826	C4orf14	21.15914104	3192
chr4.fa	57845109	57897243	POLR2B	35.36794861	8588
chr4.fa	57897335	57976539	IGFBP7	8.319947815	511
chr4.fa	57975928	58071465	LOC255130	0	0
chr4.fa	62362839	62938168	LPHN3	0.186902508	76
chr4.fa	65144177	65275178	TECRL	0	0
chr4.fa	65779999	65870218	LOC401134	0	0
chr4.fa	66185281	66535653	EPHA5	1.311028469	684
chr4.fa	66535679	66559104	LOC100144602	3.889861379	304
chr4.fa	68337989	68411256	CENPC1	9.848602493	2190
chr4.fa	68424446	68472616	STAP1	1.515100104	152
chr4.fa	68481479	68566889	UBA6	10.63612321	4553
chr4.fa	68566996	68588222	LOC550112	8.497060909	1292
chr4.fa	68603099	68621804	GNRHR	0	0
chr4.fa	68686594	68749716	TMPRSS11D	0	0
chr4.fa	68776019	68829232	TMPRSS11A	0	0
chr4.fa	68857405	68863301	LOC644759	0	0
chr4.fa	68918916	68995587	TMPRSS11F	0	0
chr4.fa	68926328	68929015	SYT14L	0	0
chr4.fa	69048010	69078079	FTLP10	0	0
chr4.fa	69054242	69083798	TMPRSS11BNL	0	0
chr4.fa	69092371	69111412	TMPRSS11B	0	0
chr4.fa	69176105	69215824	YTHDC1	26.59286834	11034
chr4.fa	69313167	69363322	TMPRSS11E	0.535857472	76
chr4.fa	69402903	69434245	UGT2B17	0	0
chr4.fa	69512315	69536374	UGT2B15	0	0
chr4.fa	69681713	69886113	UGT2B10	0.247898089	76
chr4.fa	69794177	69817509	UGT2A3	0.373353197	76
chr4.fa	69962193	69978705	UGT2B7	15.77089686	1976
chr4.fa	70066051	70080449	UGT2B11	0	0

chr4.fa	70146217	70160768	UGT2B28	0	0
chr4.fa	70345883	70361626	UGT2B4	0	0
chr4.fa	70454135	70513417	UGT2A1	0	0
chr4.fa	70504617	70505334	UGT2A2	0	0
chr4.fa	70592686	70626430	SULT1B1	0	0
chr4.fa	70706930	70725870	SULT1E1	0.643089209	76
chr4.fa	70796799	70812288	CSN1S1	0	0
chr4.fa	70820974	70826726	CSN2	0	0
chr4.fa	70861648	70868173	STATH	0	0
chr4.fa	70894130	70902255	HTN3	0	0
chr4.fa	70916159	70924559	HTN1	0	0
chr4.fa	70933103	70950988	CSN1S2AP	0	0
chr4.fa	70999321	71012421	CSN1S2BP	0	0
chr4.fa	71019904	71032326	C4orf40	0	0
chr4.fa	71062244	71070293	ODAM	0.736615766	63
chr4.fa	71091815	71100968	C4orf7	0	0
chr4.fa	71108333	71117145	CSN3	0	0
chr4.fa	71200671	71202833	C4orf35	0	0
chr4.fa	71226493	71232823	SMR3A	0	0
chr4.fa	71248795	71255961	SMR3B	0	0
chr4.fa	71263599	71275914	PROL1	0	0
chr4.fa	71296209	71348714	MUC7	0	0
chr4.fa	71384298	71398459	AMTN	2.298403845	152
chr4.fa	71458001	71473004	AMBN	0	0
chr4.fa	71494461	71512536	ENAM	0.201661932	76
chr4.fa	71521258	71532348	IGJ	0	0
chr4.fa	71554196	71556268	UTP3	10.49093867	1444
chr4.fa	71570654	71674336	RUFY3	5.720030013	2700
chr4.fa	71681499	71705627	GRSF1	21.48008322	9804
chr4.fa	71768064	71853891	MOBKL1A	5.922143765	2736
chr4.fa	71859265	71896629	DCK	6.590836057	1140
chr4.fa	72053003	72437804	SLC4A4	22.29200217	11856
chr4.fa	72607411	72649888	GC	4.50132325	532
chr4.fa	72897521	73013918	NPFFR2	0	0
chr4.fa	73146687	73434516	ADAMTS3	0.196691922	76
chr4.fa	73920416	73935472	COX18	3.289845593	988
chr4.fa	73940502	74124502	ANKRD17	52.74355637	32832
chr4.fa	74269972	74287129	ALB	67.24002211	10032
chr4.fa	74301933	74321492	AFP	0.563267832	76
chr4.fa	74347462	74369718	AFM	0.573207852	76
chr4.fa	74438862	74486340	RASSF6	7.197177311	2128
chr4.fa	74606275	74609433	IL8	2.76980179	304
chr4.fa	74702273	74704477	CXCL6	0.689927791	76

chr4.fa	74719013	74720198	PF4V1		1.544618953	76
chr4.fa	74735109	74736955	CXCL1		1.035870627	76
chr4.fa	74846796	74847715	PF4		0	0
chr4.fa	74852156	74853907	PPBP		0	0
chr4.fa	74861359	74864416	CXCL5		0	0
chr4.fa	74902312	74904490	CXCL3		0	0
chr4.fa	74919755	74921116	PPBPL2		0	0
chr4.fa	74962754	74964997	CXCL2		0.951531059	76
chr4.fa	75023829	75168814	MTHFD2L		2.911221173	456
chr4.fa	75174204	75179307	EPGN		0	0
chr4.fa	75230860	75254477	EREG		0.247295663	76
chr4.fa	75310853	75490485	AREG		0	0
chr4.fa	75671448	75719882	BTC		3.460633219	304
chr4.fa	75858285	75975325	PARM1		2.717691985	912
chr4.fa	76279286	76287776	LOC441025		0	0
chr4.fa	76404347	76439628	RCHY1		6.095341093	1748
chr4.fa	76439654	76455236	THAP6		5.438396098	1292
chr4.fa	76481258	76489927	C4orf26		0	0
chr4.fa	76501704	76555721	CDKL2		0.971109887	304
chr4.fa	76567953	76598667	G3BP2		65.39178071	19760
chr4.fa	76649829	76735366	USO1		22.40811968	5700
chr4.fa	76781026	76823681	PPEF2		0	0
chr4.fa	76831808	76862166	NAAA		1.787095212	304
chr4.fa	76871068	76912113	SDAD1		20.42011557	4092
chr4.fa	76922623	76928641	CXCL9		0	0
chr4.fa	76932337	77033955	ART3		0	0
chr4.fa	76942271	76944650	CXCL10		0	0
chr4.fa	76954840	76957350	CXCL11		0	0
chr4.fa	77035817	77069655	NUP54		15.55974673	2432
chr4.fa	77079894	77135035	SCARB2		20.25008098	6384
chr4.fa	77172853	77204929	FAM47E		2.959565819	304
chr4.fa	77227677	77232283	STBD1		9.332323849	1520
chr4.fa	77234192	77328458	CCDC158		0.315670956	76
chr4.fa	77356253	77704405	SHROOM3		18.90546729	13832
chr4.fa	77816082	77819002	ANKRD56		12.53933594	2432
chr4.fa	77870895	77959768		11-Sep	17.92637526	6612
chr4.fa	77969177	77997125	CCNI		307.3882079	38228
chr4.fa	78078357	78091213	CCNG2		33.66489176	12236
chr4.fa	78432907	78532988	CXCL13		0	0
chr4.fa	78634541	78740544	CNOT6L		8.330038442	4864
chr4.fa	78783805	78873944	MRPL1		15.16033863	1368
chr4.fa	78978724	79465423	FRAS1		1.937099158	2128
chr4.fa	79472742	79531605	ANXA3		92.22546864	9908

chr4.fa	79697532	79833341	BMP2K	4.81187359	1216
chr4.fa	79839094	79860582	PAQR3	1.224580412	304
chr4.fa	80238272	80247171	NAA11	0	0
chr4.fa	80327507	80329372	GK2	0	0
chr4.fa	80748625	80784401	GDEP	65.14026807	9044
chr4.fa	80822771	80994477	ANTXR2	6.050159181	3276
chr4.fa	81106424	81125482	PRDM8	18.71961902	5244
chr4.fa	81187742	81212171	FGF5	0.212354985	76
chr4.fa	81256874	81884902	C4orf22	1.290395396	76
chr4.fa	81952119	81978685	BMP3	1.197772478	456
chr4.fa	82009837	82126215	PRKG2	8.254434044	1824
chr4.fa	82348219	82393061	RASGEF1B	8.071296695	1216
chr4.fa	83274467	83295149	HNRNPD	49.19225812	7372
chr4.fa	83344347	83351378	HNRPDL	23.43043574	5624
chr4.fa	83351726	83382244	ENOPH1	27.2796334	3648
chr4.fa	83405604	83483126	TMEM150C	2.712119549	340
chr4.fa	83534266	83542590	C4orf11	0	0
chr4.fa	83550690	83720010	SCD5	1.332866393	380
chr4.fa	83674490	83674583	MIR575	0	0
chr4.fa	83739814	83812419	SEC31A	83.02462416	23462
chr4.fa	83814605	83822069	LOC100499177	5.970036591	836
chr4.fa	83825889	83841284	THAP9	2.683353732	684
chr4.fa	83845756	83934040	LIN54	3.678711246	1520
chr4.fa	83956239	83996971	COPS4	27.50207901	3192
chr4.fa	84011211	84035911	PLAC8	0	0
chr4.fa	84184977	84206067	COQ2	0.697458109	76
chr4.fa	84213614	84256306	HPSE	0	0
chr4.fa	84328499	84377025	HELQ	4.779794432	1140
chr4.fa	84377118	84380950	MRPS18C	28.59668612	769
chr4.fa	84382094	84406290	FAM175A	0.543237184	76
chr4.fa	84457653	84527026	AGPAT9	5.420473939	912
chr4.fa	85414436	85419387	NKX6-1	0	0
chr4.fa	85504057	85572493	CDS1	7.314650282	2204
chr4.fa	85590693	85887544	WDFY3	27.16532316	25824
chr4.fa	85887971	85928168	NCRNA00247	2.91799846	304
chr4.fa	86396284	86923823	ARHGAP24	7.453509357	2812
chr4.fa	86936276	87374283	MAPK10	10.86564732	3116
chr4.fa	87515468	87736328	PTPN13	26.58217529	15112
chr4.fa	87744621	87770416	SLC10A6	0	0
chr4.fa	87797358	87813575	C4orf36	1.260575335	76
chr4.fa	87856154	88062206	AFF1	43.17613597	27834
chr4.fa	88082214	88141674	KLHL8	6.253327177	1900
chr4.fa	88224941	88244056	HSD17B13	0	0

chr4.fa	88257691	88312455	HSD17B11	9.16665684	1140
chr4.fa	88343735	88379499	NUDT9	15.09377061	1368
chr4.fa	88394488	88450655	SPARCL1	0.380732909	76
chr4.fa	88529681	88538025	DSPP	0	0
chr4.fa	88571454	88585512	DMP1	0	0
chr4.fa	88720702	88733601	IBSP	0	0
chr4.fa	88742563	88767968	MEPE	0	0
chr4.fa	88812995	88815167	HSP90AB3P	0.637968592	76
chr4.fa	88896802	88904563	SPP1	9.712906151	1048
chr4.fa	88928820	88998929	PKD2	5.433275481	1824
chr4.fa	89011416	89080011	ABCG2	0.775020391	228
chr4.fa	89181532	89205888	PPM1K	14.138625	3496
chr4.fa	89299891	89364249	HERC6	7.938913694	2050
chr4.fa	89378268	89427314	HERC5	1.95487071	456
chr4.fa	89442135	89444955	PIGY	48.22069641	4332
chr4.fa	89513647	89629686	HERC3	19.10743043	6209
chr4.fa	89617066	89619023	NAP1L5	7.014943601	912
chr4.fa	89630940	89651254	FAM13AOS	0.479982508	76
chr4.fa	89647106	89978323	FAM13A	16.27196426	5736
chr4.fa	90033968	90036052	TIGD2	8.783514229	1216
chr4.fa	90165429	90229161	GPRIN3	0.900927318	380
chr4.fa	90645250	90759447	SNCA	2.961071882	684
chr4.fa	90816052	90875780	MMRN1	0	0
chr4.fa	91048684	92523370	FAM190A	9.774504158	5244
chr4.fa	91759642	91760269	TMSL3	3.645276631	152
chr4.fa	93225550	94693649	GRID2	0	0
chr4.fa	94750078	94751142	ATOH1	0	0
chr4.fa	95128759	95212443	SMARCAD1	10.94697476	3800
chr4.fa	95219707	95264027	HPGDS	1.417507174	152
chr4.fa	95373038	95589377	PDLIM5	61.73596164	30260
chr4.fa	95679128	96079601	BMPR1B	9.868030715	3643
chr4.fa	96083656	96470361	UNC5C	0.231783207	152
chr4.fa	96761239	96762625	PDHA2	0	0
chr4.fa	98480025	99064391	C4orf37	0.69203628	76
chr4.fa	99182527	99365012	RAP1GDS1	12.78211341	3192
chr4.fa	99391518	99579812	TSPAN5	1.01086997	228
chr4.fa	99799607	99851786	EIF4E	4.087908759	1368
chr4.fa	99916788	99983960	METAP1	32.59513469	5995
chr4.fa	99918538	99918611	MIR3684	0	0
chr4.fa	99992130	100009931	ADH5	62.36233354	10944
chr4.fa	100044833	100065449	ADH4	0	0
chr4.fa	100081751	100082804	PCNAP1	0	0
chr4.fa	100123795	100140403	ADH6	4.569246724	912

chr4.fa	100197523	100212185	ADH1A	0	0
chr4.fa	100227527	100242572	ADH1B	0.426818459	76
chr4.fa	100257649	100273917	ADH1C	6.993557496	684
chr4.fa	100333418	100356667	ADH7	0	0
chr4.fa	100432161	100463460	C4orf17	0	0
chr4.fa	100467864	100485189	RG9MTD2	2.388315849	608
chr4.fa	100485240	100545154	MTTP	0	0
chr4.fa	100737981	100791346	DAPP1	6.235555625	1216
chr4.fa	100799495	100815703	MAPKSP1	16.02828315	4555
chr4.fa	100820710	100867879	DNAJB14	5.379509006	988
chr4.fa	100869244	100871512	H2AFZ	24.17337697	1520
chr4.fa	100871636	100873620	LOC256880	0	0
chr4.fa	101107027	101111655	DDIT4L	0.869149374	152
chr4.fa	101316498	101439250	EMCN	0	0
chr4.fa	101944587	102268628	PPP3CA	86.65348469	26904
chr4.fa	102268934	102270040	FLJ20021	14.4339641	760
chr4.fa	102711764	102995969	BANK1	13.94735491	3295
chr4.fa	103172198	103266655	SLC39A8	1.975503783	760
chr4.fa	103422486	103538459	NFKB1	20.17417537	5472
chr4.fa	103552643	103682151	MANBA	3.457018666	760
chr4.fa	103717133	103790032	UBE2D3	66.54979311	12841
chr4.fa	103790135	103813963	CISD2	1.386632868	532
chr4.fa	103806205	103940896	NHEDC1	0.55920146	76
chr4.fa	103946652	103998170	NHEDC2	0	0
chr4.fa	103998782	104021024	BDH2	16.25720484	3152
chr4.fa	104026963	104119566	CENPE	0.13283482	76
chr4.fa	104510625	104640973	TACR3	0	0
chr4.fa	105393343	105412467	CXXC4	3.060472088	152
chr4.fa	106067943	106200958	TET2	10.75495164	10716
chr4.fa	106290234	106395227	PPA2	33.26307395	3695
chr4.fa	106405863	106407507	EEF1A1P9	12.52457651	1368
chr4.fa	106473777	106552837	ARHGEF38	3.186077802	304
chr4.fa	106603785	106629881	INTS12	20.90612233	2432
chr4.fa	106629941	106766910	GSTCD	8.165425678	1292
chr4.fa	106816597	106892828	NPNT	1.934689456	608
chr4.fa	106967233	107237861	TBCK	14.64270453	3223
chr4.fa	107236767	107270381	AIMP1	16.95571719	3239
chr4.fa	107279340	107288592	LOC100507096	0	0
chr4.fa	107842959	107957453	DKK2	0	0
chr4.fa	108534822	108641419	PAPSS1	43.22673972	7296
chr4.fa	108745721	108836204	SGMS2	3.323732027	1444
chr4.fa	108852717	108874613	CYP2U1	0.481789784	152
chr4.fa	108910870	108956331	HADH	37.15895959	5016

chr4.fa	108968701	109090112	LEF1	0.524260781	134
chr4.fa	109088681	109097586	LOC641518	0	0
chr4.fa	109459346	109541613	LOC285456	0.606793073	152
chr4.fa	109541722	109551639	RPL34	598.2667948	40280
chr4.fa	109571741	109588980	OSTC	18.0170403	1292
chr4.fa	109663202	109684235	AGXT2L1	0.534652621	76
chr4.fa	109734972	110223799	COL25A1	0	0
chr4.fa	110354971	110461615	SEC24B	29.07094559	8968
chr4.fa	110481355	110608872	CCDC109B	0	0
chr4.fa	110609785	110624629	CASP6	17.91673645	1976
chr4.fa	110631145	110651242	PLA2G12A	8.745862636	3040
chr4.fa	110661848	110723335	CFI	2.000203228	287
chr4.fa	110736666	110745893	GAR1	19.30397175	1292
chr4.fa	110749150	110765861	RRH	0	0
chr4.fa	110772486	110793471	LRIT3	0	0
chr4.fa	110834040	110934118	EGF	7.1756406	2660
chr4.fa	110970229	111119820	ELOVL6	10.79817567	2432
chr4.fa	111397229	111484493	ENPEP	2.749771142	912
chr4.fa	111538580	111558508	PITX2	1.114938973	228
chr4.fa	113066553	113110237	C4orf32	7.977770138	1444
chr4.fa	113152895	113191211	AP1AR	7.064191885	1364
chr4.fa	113196782	113207059	TIFA	10.0567405	2052
chr4.fa	113218499	113363764	ALPK1	6.556347198	2356
chr4.fa	113434672	113437328	NEUROG2	0	0
chr4.fa	113460489	113558119	C4orf21	0.511911059	228
chr4.fa	113558152	113578742	LARP7	24.77926641	3952
chr4.fa	113569030	113569097	MIR367	0	0
chr4.fa	113569160	113569227	MIR302D	0	0
chr4.fa	113569339	113569407	MIR302A	0	0
chr4.fa	113569519	113569586	MIR302C	0	0
chr4.fa	113569641	113569713	MIR302B	0	0
chr4.fa	113739239	114304896	ANK2	2.995259529	2888
chr4.fa	114028019	114028111	MIR1243	0	0
chr4.fa	114372188	114683083	CAMK2D	20.5223773	8304
chr4.fa	114821440	114900878	ARSJ	0.978790812	304
chr4.fa	115519611	115598202	UGT8	2.009239611	456
chr4.fa	115577915	115578010	MIR577	0	0
chr4.fa	115748927	116035032	NDST4	0	0
chr4.fa	117220881	117220924	MIR1973	0	0
chr4.fa	118004710	118006736	TRAM1L1	0	0
chr4.fa	118955500	119179789	NDST3	0	0
chr4.fa	119199917	119200978	SNHG8	221.8707471	9119
chr4.fa	119200345	119200475	SNORA24	0	0

chr4.fa	119201193	119273922	PRSS12	0	0
chr4.fa	119437495	119475359	CEP170P1	1.069757061	76
chr4.fa	119606574	119632077	METTL14	19.01345206	2660
chr4.fa	119643978	119757326	SEC24D	26.48126902	7144
chr4.fa	119809996	119982402	SYNPO2	0.131629969	76
chr4.fa	120056939	120108938	MYOZ2	0	0
chr4.fa	120133782	120216673	USP53	14.57884743	6384
chr4.fa	120217574	120225600	C4orf3	47.44100722	9576
chr4.fa	120238405	120243316	FABP2	0.508898931	76
chr4.fa	120326678	120331815	FLJ14186	0	0
chr4.fa	120375938	120420747	LOC645513	8.105484342	465
chr4.fa	120415550	120549981	PDE5A	0.976531717	456
chr4.fa	120980579	120988013	MAD2L1	0.797008921	76
chr4.fa	121615929	121844013	PRDM5	0.923066455	152
chr4.fa	121956782	121993673	C4orf31	0	0
chr4.fa	122052564	122137655	TNIP3	0	0
chr4.fa	122249797	122302181	QRFPR	0	0
chr4.fa	122589152	122618147	ANXA5	117.3957092	12464
chr4.fa	122680085	122685027	TMEM155	0	0
chr4.fa	122685740	122687963	LOC100192379	0	0
chr4.fa	122722472	122737598	EXOSC9	4.117277001	380
chr4.fa	122737603	122745088	CCNA2	0	0
chr4.fa	122745635	122791642	BBS7	2.778235747	760
chr4.fa	122800183	122872909	TRPC3	0	0
chr4.fa	123091758	123283914	KIAA1109	7.128500806	7372
chr4.fa	123300121	123350947	ADAD1	0	0
chr4.fa	123372626	123377650	IL2	0	0
chr4.fa	123533783	123542211	IL21	0	0
chr4.fa	123651344	123653613	CETN4P	0	0
chr4.fa	123653857	123666098	BBS12	4.002063127	912
chr4.fa	123747863	123819390	FGF2	0.186450689	76
chr4.fa	123833723	123844123	NUDT6	0	0
chr4.fa	123844225	124240604	SPATA5	1.828060145	988
chr4.fa	124317956	124324907	SPRY1	6.127872069	1064
chr4.fa	124573940	124851518	LOC285419	0	0
chr4.fa	125585204	125633887	ANKRD50	9.498292071	5548
chr4.fa	126237567	126414087	FAT4	0.213108017	228
chr4.fa	126428414	126428462	MIR2054	0	0
chr4.fa	128554087	128637934	INTU	3.145414082	684
chr4.fa	128651555	128695447	SLC25A31	0	0
chr4.fa	128703453	128754526	HSPA4L	2.800224277	608
chr4.fa	128802016	128820377	PLK4	0.573057246	152
chr4.fa	128838960	128887139	MFSD8	2.518439755	760

chr4.fa	128886461	128952455	C4orf29	5.233722038	760
chr4.fa	128982503	129132289	LARP1B	10.73386675	3952
chr4.fa	129190392	129209984	PGRMC2	8.758212358	2204
chr4.fa	129730779	129796379	PHF17	6.998075687	3800
chr4.fa	129805152	130014764	SCLT1	4.124054288	836
chr4.fa	130014829	130033843	C4orf33	5.299085203	684
chr4.fa	134070470	134112732	PCDH10	0	0
chr4.fa	135117489	135122903	PABPC4L	0.2316326	76
chr4.fa	138440074	138453629	PCDH18	0	0
chr4.fa	139085248	139163503	SLC7A11	0	0
chr4.fa	139936943	139967095	CCRN4L	2.925829991	380
chr4.fa	139978871	140060606	ELF2	17.7311894	4256
chr4.fa	140187317	140201492	C4orf49	0.854841768	76
chr4.fa	140211071	140223705	NDUFC1	28.35917987	2736
chr4.fa	140222676	140311935	NAA15	12.98347413	4788
chr4.fa	140374961	140397069	RAB33B	4.734612521	1216
chr4.fa	140427192	140477577	SETD7	42.27807018	19684
chr4.fa	140586922	140625406	MGST2	51.45090188	2733
chr4.fa	140637546	141075233	MAML3	13.08031403	6156
chr4.fa	141178440	141303710	SCOC	31.16588022	4863
chr4.fa	141204880	141294546	LOC100129858	0.56748481	76
chr4.fa	141309607	141348815	CLGN	1.21674888	228
chr4.fa	141445312	141474924	ELMOD2	0.676373217	198
chr4.fa	141481050	141489959	UCP1	0	0
chr4.fa	141541936	141677471	TBC1D9	47.95186403	17467
chr4.fa	141786725	142054616	RNF150	2.210148511	684
chr4.fa	142142049	142155850	ZNF330	12.96494954	1627
chr4.fa	142557754	142654612	IL15	0.588419096	76
chr4.fa	142949182	143767604	INPP4B	30.2601335	8284
chr4.fa	144106070	144143141	USP38	14.54164765	5016
chr4.fa	144257983	144395718	GAB1	5.643521976	2964
chr4.fa	144434616	144474567	SMARCA5	41.59175694	10635
chr4.fa	144480625	144482613	LOC441046	0	0
chr4.fa	144498561	144621828	FREM3	0	0
chr4.fa	144792019	144826716	GYPE	0	0
chr4.fa	144917257	144940496	GYPB	0	0
chr4.fa	145030456	145061904	GYPA	0.430282405	76
chr4.fa	145567173	145659881	HHIP	0	0
chr4.fa	145916313	146017263	ANAPC10	4.391079386	228
chr4.fa	146019156	146050676	ABCE1	14.09645522	3845
chr4.fa	146054802	146100832	OTUD4	11.51129684	5682
chr4.fa	146402951	146480325	SMAD1	6.60981246	1368
chr4.fa	146540540	146581187	MMAA	4.237159673	1672

chr4.fa	146601356	146653948	C4orf51	0	0
chr4.fa	146681888	146859607	ZNF827	61.29393194	17024
chr4.fa	147096835	147111213	LSM6	18.90712396	988
chr4.fa	147175137	147443123	SLC10A7	1.869476897	608
chr4.fa	147560045	147563623	POU4F2	0	0
chr4.fa	147628179	147867034	TTC29	0	0
chr4.fa	148402069	148466106	EDNRA	0	0
chr4.fa	148538539	148556672	TMEM184C	12.32005306	2432
chr4.fa	148559534	148605280	PRMT10	6.878193015	1292
chr4.fa	148653453	148993927	ARHGAP10	26.82916974	5444
chr4.fa	148999915	149363672	NR3C2	12.22622529	4788
chr4.fa	150999426	151178606	DCLK2	5.467914946	1596
chr4.fa	151185596	151936649	LRBA	53.2830284	36076
chr4.fa	151503077	151505845	MAB21L2	0	0
chr4.fa	152020754	152025804	RPS3A	135.3294642	8132
chr4.fa	152024979	152025043	SNORD73A	0	0
chr4.fa	152041433	152147660	SH3D19	13.51918099	4613
chr4.fa	152198325	152212605	PRSS48	0	0
chr4.fa	152330398	152584784	FAM160A1	10.50554749	3192
chr4.fa	152591809	152682175	PET112L	7.179405759	1064
chr4.fa	153242410	153456172	FBXW7	17.74399095	5548
chr4.fa	153410479	153410568	MIR3140	0	0
chr4.fa	153457416	153460415	DKFZP434I0714	1.526094369	304
chr4.fa	153547266	153601317	TMEM154	13.46375785	2875
chr4.fa	153690506	153700916	TIGD4	0.926078582	152
chr4.fa	153701112	153833063	ARFIP1	33.98447848	6668
chr4.fa	153864135	153900848	FHDC1	13.24778831	5700
chr4.fa	154074270	154260474	TRIM2	38.59529256	17708
chr4.fa	154228621	154229963	ANXA2P1	0	0
chr4.fa	154265801	154336243	MND1	0	0
chr4.fa	154387498	154557862	KIAA0922	0.228018047	76
chr4.fa	154605441	154627242	TLR2	0	0
chr4.fa	154631312	154681387	RNF175	0	0
chr4.fa	154701742	154710228	SFRP2	0	0
chr4.fa	155155527	155412877	DCHS2	0	0
chr4.fa	155457662	155471523	PLRG1	19.80775006	2345
chr4.fa	155484132	155493915	FGB	0.328321891	76
chr4.fa	155504280	155511897	FGA	0	0
chr4.fa	155525286	155533902	FGG	0	0
chr4.fa	155665163	155674270	LRAT	0	0
chr4.fa	155702427	155749965	RBM46	0	0
chr4.fa	156129781	156138228	NPY2R	0	0
chr4.fa	156263812	156298122	MAP9	5.308573405	2584

chr4.fa	156587862	156658214	GUCY1A3		22.12091333	14591
chr4.fa	156680126	156728783	GUCY1B3		4.932208081	1064
chr4.fa	156750881	156787425	ACCN5		0	0
chr4.fa	156824847	156841550	TDO2		0	0
chr4.fa	156845270	156875048	CTSO		2.731698378	532
chr4.fa	157682763	157892546	PDGFC		0.372901377	76
chr4.fa	157997277	158093242	GLRB		0.712217534	152
chr4.fa	158141736	158287226	GRIA2		0	0
chr4.fa	158493642	158497303	LOC340017		0	0
chr4.fa	159045732	159094202	FAM198B		0.446849106	152
chr4.fa	159131401	159176439	TMEM144		2.069783372	456
chr4.fa	159443047	159573263	RXFP1		0	0
chr4.fa	159587831	159593202	C4orf46		0.649414677	152
chr4.fa	159593277	159629841	ETFDH		18.52428256	2888
chr4.fa	159630279	159644552	PPID		16.22678235	1976
chr4.fa	159690182	159827954	FNIP2		15.62812202	6004
chr4.fa	159814684	159956333	C4orf45		0	0
chr4.fa	160049954	160050046	MIR3688		0	0
chr4.fa	160188998	160281301	RAPGEF2		16.11171908	7296
chr4.fa	162305049	163085186	FSTL5		0	0
chr4.fa	164047860	164088073	NAF1		4.948624175	760
chr4.fa	164245117	164253748	NPY1R		0	0
chr4.fa	164265091	164273086	NPY5R		0	0
chr4.fa	164392247	164395047	TKTL2		0	0
chr4.fa	164415673	164441691	C4orf43		5.951963827	758
chr4.fa	164445450	165304407		1-Mar	0.199704049	76
chr4.fa	165118159	165118863	ANP32C		0	0
chr4.fa	165875598	165898818	TRIM61		0	0
chr4.fa	165878100	165880273	C4orf39		0	0
chr4.fa	165953151	165962896	TRIM60		0	0
chr4.fa	165997230	166034024	TMEM192		6.111305368	1824
chr4.fa	166128770	166244308	KLHL2		11.44954823	2508
chr4.fa	166198944	166201175	GK3P		0	0
chr4.fa	166248818	166264225	SC4MOL		9.096926089	1292
chr4.fa	166300097	166419482	CPE		16.97107904	2736
chr4.fa	166307394	166307489	MIR578		0	0
chr4.fa	166794410	167024993	TLL1		1.376090422	608
chr4.fa	167654536	168155741	SPOCK3		1.915411841	380
chr4.fa	169013707	169108893	ANXA10		0	0
chr4.fa	169137442	169239958	DDX60		3.769526888	1520
chr4.fa	169277886	169401638	DDX60L		4.067275686	1824
chr4.fa	169418217	169849608	PALLD		7.867827486	3648
chr4.fa	169908742	169931468	CBR4		3.923295994	912

chr4.fa	170015407	170192249	SH3RF1	28.91416435	10108
chr4.fa	170314421	170533778	NEK1	8.417390138	3174
chr4.fa	170541722	170642157	CLCN3	19.49599487	5244
chr4.fa	170650619	170679093	C4orf27	10.35418808	836
chr4.fa	170907748	170947429	MFAP3L	6.174258832	2584
chr4.fa	170981373	171011372	AADAT	8.687728576	1216
chr4.fa	171502621	171526135	HSP90AA6P	0	0
chr4.fa	172734575	173961558	GALNTL6	0.401817801	76
chr4.fa	174089904	174245118	GALNT7	6.6438495	1900
chr4.fa	174252527	174255595	HMGB2	8.107291618	836
chr4.fa	174292093	174298683	SAP30	2.064210936	152
chr4.fa	174309299	174320617	SCRG1	0	0
chr4.fa	174447652	174451378	HAND2	0	0
chr4.fa	174451609	174462981	NBLA00301	0	0
chr4.fa	174537087	174537794	MORF4	0	0
chr4.fa	175157810	175205402	FBXO8	16.29154309	2356
chr4.fa	175205055	175254531	KIAA1712	4.1928814	1728
chr4.fa	175344946	175345015	MIR4276	0	0
chr4.fa	175411328	175444044	HPGD	150.7459842	30248
chr4.fa	175563198	175750465	GLRA3	0	0
chr4.fa	175839509	175899331	ADAM29	0	0
chr4.fa	176554088	176923648	GPM6A	0	0
chr4.fa	176986985	177103979	WDR17	1.065389476	532
chr4.fa	177105725	177116822	SPATA4	0.928337678	76
chr4.fa	177134826	177190373	ASB5	0	0
chr4.fa	177241090	177253396	SPCS3	3.984743394	1216
chr4.fa	177604691	177713895	VEGFC	0.550616897	76
chr4.fa	178230991	178284092	NEIL3	0	0
chr4.fa	178351929	178363657	AGA	1.089034677	152
chr4.fa	178649911	178911904	LOC285501	0	0
chr4.fa	181985243	182080302	NCRNA00290	0	0
chr4.fa	183060159	183065668	MGC45800	0	0
chr4.fa	183090446	183090531	MIR1305	0	0
chr4.fa	183245137	183724177	ODZ3	0.211752559	152
chr4.fa	183811244	183838630	DCTD	35.31357971	4788
chr4.fa	183958818	183961272	FAM92A3	0	0
chr4.fa	184018174	184020352	C4orf38	0.525315026	76
chr4.fa	184020463	184241929	WWC2	12.75952245	5168
chr4.fa	184242917	184243579	CLDN24	0	0
chr4.fa	184365789	184369049	CDKN2AIP	14.94647758	2356
chr4.fa	184415890	184425668	LOC389247	0	0
chr4.fa	184426220	184432249	ING2	11.28192334	836
chr4.fa	184560789	184580331	RWDD4	4.414272767	760

chr4.fa	184580446	184634745	C4orf41	17.40151205	5092
chr4.fa	184826509	184938875	STOX2	6.886325759	2128
chr4.fa	185009859	185139114	ENPP6	2.908058439	760
chr4.fa	185308876	185395726	IRF2	27.09724908	4113
chr4.fa	185548850	185570629	CASP3	10.27707762	1824
chr4.fa	185570767	185612866	CCDC111	5.544422984	684
chr4.fa	185615219	185655286	MLF1IP	1.625042755	228
chr4.fa	185676749	185747215	ACSL1	117.991508	29810
chr4.fa	185719451	185720200	SLED1	0	0
chr4.fa	185772167	185772264	MIR3945	0	0
chr4.fa	185940083	185941926	HELT	0	0
chr4.fa	186064417	186071538	SLC25A4	10.09951271	2964
chr4.fa	186080819	186125182	KIAA1430	11.02619372	3572
chr4.fa	186131284	186285031	SNX25	11.35813016	2356
chr4.fa	186285121	186300152	LRP2BP	0.45166851	152
chr4.fa	186317840	186320182	ANKRD37	27.87693827	944
chr4.fa	186320694	186347139	UFSP2	10.53853028	1368
chr4.fa	186350545	186370821	C4orf47	0	0
chr4.fa	186366338	186392913	CCDC110	0.790532847	152
chr4.fa	186421815	186456712	PDLIM3	0	0
chr4.fa	186506598	186877870	SORBS2	10.29605402	6001
chr4.fa	186990309	187006252	TLR3	1.505611902	304
chr4.fa	187065995	187093817	FAM149A	24.55154957	4408
chr4.fa	187112674	187134617	CYP4V2	4.136554617	1292
chr4.fa	187148672	187179625	KLKB1	0	0
chr4.fa	187187118	187210835	F11	2.026107524	367
chr4.fa	187207252	187422212	LOC285441	3.554611595	532
chr4.fa	187454809	187476537	MTNR1A	2.071741255	152
chr4.fa	187508937	187644987	FAT1	23.10301748	22648
chr4.fa	188916925	188926199	ZFP42	1.297775109	228
chr4.fa	189012427	189026408	TRIML2	0	0
chr4.fa	189060598	189068649	TRIML1	0	0
chr4.fa	189376732	189523062	LOC401164	0	0
chr4.fa	190394299	190396344	HSP90AA4P	0.910566126	76
chr4.fa	190861974	190884359	FRG1	12.95244922	897
chr4.fa	190903678	190906024	TUBB4Q	0	0
chr4.fa	190988867	191013476	DUX2	0	0
chr4.fa	191001979	191003583	DUX4L4	0	0
chr4.fa	191005267	191011865	DUX4	0	0
chr5.fa	140373	190087	PLEKHG4B	0.397600822	304
chr5.fa	191626	195468	LRRC14B	0	0
chr5.fa	204875	218297	CCDC127	4.030377125	380
chr5.fa	218356	256814	SDHA	22.51851415	3572

chr5.fa	271736	315089	PDCD6	39.90376072	2888
chr5.fa	304291	438405	AHRR	0	0
chr5.fa	403812	404662	LOC100310782	0	0
chr5.fa	441643	443258	C5orf55	3.541508841	380
chr5.fa	443334	467409	EXOC3	19.89178842	3648
chr5.fa	470625	473080	LOC25845	7.223533426	836
chr5.fa	473334	524549	SLC9A3	3.297375911	608
chr5.fa	612405	653666	CEP72	1.896586044	304
chr5.fa	659977	693510	TPPP	0.380130483	152
chr5.fa	795720	851101	ZDHHC11	0.439168181	76
chr5.fa	863850	892939	BRD9	6.232392891	1210
chr5.fa	892969	918164	TRIP13	0	0
chr5.fa	1009168	1038925	NKD2	0.636613135	76
chr5.fa	1050489	1112172	SLC12A7	7.7688791	2736
chr5.fa	1201710	1225230	SLC6A19	3.983538543	1368
chr5.fa	1225470	1246304	SLC6A18	0	0
chr5.fa	1253287	1295162	TERT	0	0
chr5.fa	1318000	1345002	CLPTM1L	27.09092361	3862
chr5.fa	1392905	1445543	SLC6A3	0	0
chr5.fa	1461542	1524076	LPCAT1	3.431264976	899
chr5.fa	1572073	1594646	SDHAP3	3.974351554	304
chr5.fa	1597672	1634120	LOC728613	1.777305797	304
chr5.fa	1708900	1708983	MIR4277	0	0
chr5.fa	1798499	1799956	MRPL36	41.01207301	1672
chr5.fa	1801496	1816167	NDUFS6	81.45937213	2964
chr5.fa	1877541	1882880	IRX4	1.030750011	152
chr5.fa	2746279	2751769	IRX2	2.982006168	456
chr5.fa	2752262	2755511	C5orf38	0	0
chr5.fa	3417266	3536208	LOC285577	0	0
chr5.fa	3596168	3601517	IRX1	1.23211073	152
chr5.fa	5034472	5070115	LOC340094	0	0
chr5.fa	5140443	5320412	ADAMTS16	1.150632683	380
chr5.fa	5422807	5490338	KIAA0947	12.93603312	6801
chr5.fa	6310554	6337405	FLJ33360	0	0
chr5.fa	6372039	6378639	MED10	27.83582273	1900
chr5.fa	6448736	6496834	UBE2QL1	1.120511409	456
chr5.fa	6582287	6588613	LOC255167	1.384524379	304
chr5.fa	6599352	6633473	NSUN2	29.46026306	6596
chr5.fa	6633500	6669675	SRD5A1	5.055705306	760
chr5.fa	6714718	6757161	PAPD7	8.12174983	2204
chr5.fa	6827966	6828034	MIR4278	0	0
chr5.fa	7299487	7306827	LOC442132	0	0
chr5.fa	7396343	7830194	ADCY2	1.746732704	760

chr5.fa	7831511	7851264	C5orf49	5.578008205	380
chr5.fa	7859272	7869150	FASTKD3	3.757478378	608
chr5.fa	7869217	7901235	MTRR	14.34254603	3116
chr5.fa	9035138	9546233	SEMA5A	2.320091163	1819
chr5.fa	9548939	9549026	SNORD123	0	0
chr5.fa	9629109	9630463	TAS2R1	0	0
chr5.fa	9641427	9903936	LOC285692	0	0
chr5.fa	10226438	10250014	FAM173B	2.317079035	284
chr5.fa	10250282	10266501	CCT5	34.30798096	7752
chr5.fa	10277707	10308168	CMBL	29.13133874	7828
chr5.fa	10353828	10435491	6-Mar	11.38614295	3486
chr5.fa	10442009	10465138	ROPN1L	0	0
chr5.fa	10564435	10657928	ANKRD33B	0.613269147	380
chr5.fa	10679342	10761387	DAP	156.7411721	24374
chr5.fa	10971952	11904110	CTNND2	3.579461646	1292
chr5.fa	12574969	12805295	TAG	0	0
chr5.fa	13690437	13944589	DNAH5	1.249430463	1292
chr5.fa	14143829	14509458	TRIO	18.6907026	12692
chr5.fa	14581891	14616287	FAM105A	0.076959856	36
chr5.fa	14664783	14699842	FAM105B	4.203875665	2204
chr5.fa	14704909	14871887	ANKH	16.17813649	8816
chr5.fa	15500305	15939900	FBXL7	4.516233281	1368
chr5.fa	16067474	16179897	11-Mar	0	0
chr5.fa	16451628	16465894	ZNF622	23.67682776	2660
chr5.fa	16473147	16617118	FAM134B	0.639474656	152
chr5.fa	16662016	16936385	MYO10	11.19336679	8495
chr5.fa	17130137	17217531	LOC285696	1.38075922	304
chr5.fa	17217750	17276943	BASP1	202.5517147	24168
chr5.fa	17379015	17387419	LOC401177	0	0
chr5.fa	19473155	19988353	CDH18	0	0
chr5.fa	21459589	21589481	GUSBP1	0.753784893	304
chr5.fa	21750973	22853731	CDH12	0	0
chr5.fa	22142461	22152379	PMCHL1	0	0
chr5.fa	23507724	23528706	PRDM9	0	0
chr5.fa	24487209	24645085	CDH10	0.948518932	228
chr5.fa	26880709	27038689	CDH9	0.372298952	76
chr5.fa	28926977	28927420	LOC729862	0	0
chr5.fa	31193796	31325237	CDH6	0.759507935	228
chr5.fa	31400602	31532282	DROSHA	16.80315294	6133
chr5.fa	31532373	31555165	C5orf22	11.40813148	2736
chr5.fa	31799031	32111038	PDZD2	3.932633589	3040
chr5.fa	31936208	31936265	MIR4279	0	0
chr5.fa	32124824	32174425	GOLPH3	105.2218949	18696

chr5.fa	32227111	32313114	MTMR12	16.30042887	5614
chr5.fa	32354456	32444844	ZFR	38.5986059	12084
chr5.fa	32585605	32604185	SUB1	25.4574469	5928
chr5.fa	32711665	32787254	NPR3	4.76202288	836
chr5.fa	32788945	32791819	C5orf23	10.35117596	1976
chr5.fa	32947549	32962573	LOC340113	0	0
chr5.fa	33440898	33468196	TARS	36.94871309	6992
chr5.fa	33527287	33892124	ADAMTS12	0	0
chr5.fa	33936491	33939023	RXFP3	0	0
chr5.fa	33944721	33984780	SLC45A2	0	0
chr5.fa	33987091	34008220	AMACR	15.06545662	3116
chr5.fa	34017963	34043317	C1QTNF3	1.517208593	380
chr5.fa	34656433	34832717	RAI14	15.89906289	6182
chr5.fa	34839269	34899564	TTC23L	0	0
chr5.fa	34905366	34915731	RAD1	5.435233364	1672
chr5.fa	34915820	34925787	BRIX1	17.98285265	1520
chr5.fa	34929698	34959069	DNAJC21	11.43132486	4712
chr5.fa	34998206	35048240	AGXT2	15.69905762	2508
chr5.fa	35055802	35230794	PRLR	0.873968777	684
chr5.fa	35617989	35814713	SPEF2	1.218104338	456
chr5.fa	35856991	35876923	IL7R	0	0
chr5.fa	35904398	35938881	CAPSL	0	0
chr5.fa	35953191	36001130	UGT3A1	0.438113937	152
chr5.fa	36035119	36067023	UGT3A2	0.479982508	76
chr5.fa	36103414	36152015	LMBRD2	3.536087011	760
chr5.fa	36147994	36148090	MIR580	0	0
chr5.fa	36152189	36184147	SKP2	7.630773057	912
chr5.fa	36192694	36242258	C5orf33	13.28408445	3477
chr5.fa	36249104	36302011	RANBP3L	14.42778924	2508
chr5.fa	36606457	36688436	SLC1A3	1.097920453	304
chr5.fa	36876861	37065921	NIPBL	28.1471261	18392
chr5.fa	37106330	37249530	C5orf42	6.643397681	4940
chr5.fa	37291941	37371197	NUP155	6.89250062	2048
chr5.fa	37379412	37752774	WDR70	12.94567193	1824
chr5.fa	37812779	37839782	GDNF	0	0
chr5.fa	38258533	38465581	EGFLAM	0	0
chr5.fa	38475065	38595507	LIFR	9.242261238	6384
chr5.fa	38557604	38557663	MIR3650	0	0
chr5.fa	38845960	38935743	OSMR	0.372449558	152
chr5.fa	38938022	39074501	RICTOR	10.19318987	6452
chr5.fa	39105357	39219667	FYB	0.711163289	228
chr5.fa	39284378	39364655	C9	0	0
chr5.fa	39371780	39425335	DAB2	71.34223848	21776

chr5.fa	40680032	40693837	PTGER4	0	0
chr5.fa	40711678	40756072	TTC33	4.157639509	1520
chr5.fa	40759481	40798297	PRKAA1	16.95722326	5776
chr5.fa	40825365	40829244	LOC100506548	15.04512476	3876
chr5.fa	40831430	40835387	RPL37	345.5696342	36024
chr5.fa	40832758	40832837	SNORD72	0	0
chr5.fa	40841410	40855456	CARD6	8.940144856	2432
chr5.fa	40909599	40983042	C7	0	0
chr5.fa	40998122	41071444	HEATR7B2	0	0
chr5.fa	41142336	41261540	C6	0	0
chr5.fa	41307048	41510730	PLCXD3	0.295790915	152
chr5.fa	41730167	41870791	OXCT1	10.55253668	2493
chr5.fa	41904470	41921738	C5orf51	11.39789024	3952
chr5.fa	41925356	41941672	FBXO4	6.826685636	684
chr5.fa	42424026	42721926	GHR	2.100205859	608
chr5.fa	42756920	42802539	CCDC152	0	0
chr5.fa	42804758	42812024	SEPP1	55.20898268	2753
chr5.fa	43014831	43018913	LOC648987	3.592564401	760
chr5.fa	43039182	43040447	C5orf39	0.904090052	76
chr5.fa	43042236	43045370	LOC153684	1.519317082	228
chr5.fa	43065289	43067073	LOC100132356	0.641281933	76
chr5.fa	43121642	43175823	ZNF131	9.530521835	1520
chr5.fa	43192327	43280952	MGC42105	0	0
chr5.fa	43289493	43313595	HMGCS1	95.16515442	22179
chr5.fa	43381600	43412488	CCL28	1.40274775	76
chr5.fa	43444354	43483992	C5orf28	2.0727955	380
chr5.fa	43486803	43515273	C5orf34	0	0
chr5.fa	43526370	43557521	PAIP1	14.73065865	2812
chr5.fa	43602791	43705668	NNT	11.80979867	3800
chr5.fa	44305097	44388784	FGF10	0	0
chr5.fa	44809027	44815618	MRPS30	14.37613125	1596
chr5.fa	45259352	45696220	HCN1	0	0
chr5.fa	49692031	49737234	EMB	2.390725551	684
chr5.fa	49961733	50142356	PARP8	5.140496693	2508
chr5.fa	50678958	50690563	ISL1	1.685134698	304
chr5.fa	52083774	52098452	PELO	7.744932687	1444
chr5.fa	52145199	52249485	ITGA1	0.244434142	76
chr5.fa	52285156	52390609	ITGA2	1.600042097	836
chr5.fa	52393895	52405598	MOCS2	39.24365299	4104
chr5.fa	52405687	52410952	LOC257396	1.460580597	228
chr5.fa	52776595	52781904	FST	0	0
chr5.fa	52856465	52979171	NDUFS4	42.33017998	1900
chr5.fa	53180614	53606403	ARL15	5.524994762	912

chr5.fa	53247334	53247429	MIR581	0	0
chr5.fa	53751431	53752213	HSPB3	0	0
chr5.fa	53813589	53842416	SNX18	9.580974969	3420
chr5.fa	54273695	54281414	ESM1	0	0
chr5.fa	54320107	54329960	GZMK	0	0
chr5.fa	54398474	54406080	GZMA	0	0
chr5.fa	54408799	54469005	CDC20B	0	0
chr5.fa	54455984	54463129	GPX8	0.610407626	152
chr5.fa	54466360	54466450	MIR449A	0	0
chr5.fa	54466474	54466570	MIR449B	0	0
chr5.fa	54468090	54468181	MIR449C	0	0
chr5.fa	54515425	54523143	LOC345643	0.543990216	76
chr5.fa	54526981	54529508	CCNO	12.7888907	1216
chr5.fa	54552073	54603521	DHX29	31.73065412	9424
chr5.fa	54603576	54721409	SKIV2L2	30.35185278	7505
chr5.fa	54721691	54830873	PPAP2A	47.35335431	4078
chr5.fa	54824670	54830370	RNF138P1	0.401516588	152
chr5.fa	54921676	55008163	SLC38A9	3.163185634	532
chr5.fa	55033845	55112974	DDX4	0	0
chr5.fa	55147334	55212981	IL31RA	0.478325838	76
chr5.fa	55230925	55290821	IL6ST	6.825932604	4104
chr5.fa	55395507	55529186	ANKRD55	0.457843371	76
chr5.fa	56110900	56191978	MAP3K1	12.49611191	6232
chr5.fa	56205087	56221359	C5orf35	0.595497595	76
chr5.fa	56215429	56247954	MIER3	8.802942451	3040
chr5.fa	56469775	56560506	GPBP1	30.05967642	9269
chr5.fa	56775843	56778636	ACTBL2	0	0
chr5.fa	57749810	57755913	PLK2	25.07038852	4636
chr5.fa	57787330	57792185	GAPT	0	0
chr5.fa	57878939	58147406	RAB3C	0	0
chr5.fa	58264866	59481514	PDE4D	6.840541422	4973
chr5.fa	59783540	59843484	PART1	9.924357498	6156
chr5.fa	59892739	59995993	DEPDC1B	1.371271018	228
chr5.fa	60047616	60140101	ELOVL7	8.552785267	2204
chr5.fa	60169659	60240905	ERCC8	3.359877556	456
chr5.fa	60240956	60448864	NDUFAF2	12.73557604	608
chr5.fa	60453536	60458302	C5orf43	22.57529276	4332
chr5.fa	60628100	60841999	ZSWIM6	11.64789682	4256
chr5.fa	60933636	61002362	FLJ37543	0	0
chr5.fa	61601989	61682210	KIF2A	16.65631173	3716
chr5.fa	61684351	61699728	DIMT1L	13.87084687	1434
chr5.fa	61708573	61924416	IPO11	9.820439101	3021
chr5.fa	61874562	61877275	LRRC70	0.508898931	76

chr5.fa	63256278	63257546	HTR1A	0	0
chr5.fa	63461671	63668696	RNF180	4.861874905	1596
chr5.fa	63802452	63908121	RGS7BP	2.099603434	532
chr5.fa	63986135	64013977	FAM159B	0	0
chr5.fa	64014018	64064496	SREK1IP1	2.520096425	1140
chr5.fa	64064755	64314590	CWC27	8.435462903	1165
chr5.fa	64444563	64777704	ADAMTS6	0	0
chr5.fa	64813593	64858995	CENPK	0	0
chr5.fa	64859131	64883370	PPWD1	12.01974395	1672
chr5.fa	64885507	64920187	TRIM23	21.32812139	3420
chr5.fa	64920558	64961754	C5orf44	8.01587355	2204
chr5.fa	64961955	65017941	SGTB	3.864860722	1348
chr5.fa	65018023	65125111	NLN	5.682679633	3268
chr5.fa	65222384	65376850	ERBB2IP	37.35760939	17155
chr5.fa	65240633	65241401	LOC100303749	0	0
chr5.fa	65440085	65476714	SREK1	17.35030589	5168
chr5.fa	65892176	66465423	MAST4	44.01938105	31996
chr5.fa	66478104	66492617	CD180	0.421547236	76
chr5.fa	67522462	67597649	PIK3R1	25.25970073	11628
chr5.fa	68389818	68425880	SLC30A5	6.200464341	1520
chr5.fa	68462913	68474070	CCNB1	3.284423763	456
chr5.fa	68485375	68506184	CENPH	0	0
chr5.fa	68513573	68525985	MRPS36	11.19637892	988
chr5.fa	68530622	68573257	CDK7	17.35392044	1748
chr5.fa	68576519	68616410	CCDC125	18.04279399	4332
chr5.fa	68647553	68665840	TAF9	21.60448408	3143
chr5.fa	68665124	68710630	RAD17	9.327353839	2128
chr5.fa	68710939	68737890	MARVELD2	14.83758918	2128
chr5.fa	68788119	68850133	OCLN	11.70618149	2052
chr5.fa	68856051	68888729	GTF2H2C	0	0
chr5.fa	68858500	68858597	GTF2H2D	0	0
chr5.fa	68926980	68929028	LOC100272216	0	0
chr5.fa	68935290	69006272	GUSBP3	0	0
chr5.fa	69321072	70203944	SERF1A	0	0
chr5.fa	69423289	69521622	LOC100170939	0	0
chr5.fa	69711197	69746189	GTF2H2B	0	0
chr5.fa	69776870	69881549	SMA5	0	0
chr5.fa	69812079	70555122	LOC100049076	0	0
chr5.fa	70264310	70320941	NAIP	0.524110175	228
chr5.fa	70330951	70363497	GTF2H2	1.528504071	198
chr5.fa	70370030	70388897	LOC647859	0	0
chr5.fa	70671612	70681820	PMCHL2	0	0
chr5.fa	70751442	70863649	BDP1	16.48040348	12119

chr5.fa	70883115	70954530	MCCC2	106.1871312	25897
chr5.fa	71014990	71016875	CARTPT	1.250936527	76
chr5.fa	71403118	71505397	MAP1B	0	0
chr5.fa	71515236	71616084	MRPS27	25.05773759	4627
chr5.fa	71616200	71655180	PTCD2	3.265597967	456
chr5.fa	71739234	71803249	ZNF366	0	0
chr5.fa	72112418	72210215	TNPO1	26.28171557	15196
chr5.fa	72251808	72386349	FCHO2	10.11095879	3344
chr5.fa	72416388	72427644	TMEM171	0	0
chr5.fa	72469023	72470970	TMEM174	22.13687761	2584
chr5.fa	72742085	72744352	FOXD1	0	0
chr5.fa	72794250	72801448	BTF3	238.729022	15978
chr5.fa	72848025	72861511	ANKRA2	9.894236223	1444
chr5.fa	72861598	72877794	UTP15	7.482877599	1816
chr5.fa	72921983	73237818	RGNEF	16.11096605	6795
chr5.fa	73923234	73937249	ENC1	78.00852833	28576
chr5.fa	73980969	74017030	HEXB	9.443923171	1140
chr5.fa	74017114	74063042	GFM2	11.31114097	2356
chr5.fa	74063103	74072734	NSA2	87.08979135	6384
chr5.fa	74073399	74162615	FAM169A	6.549419305	2584
chr5.fa	74323289	74326724	GCNT4	14.65731335	3344
chr5.fa	74364122	74532703	ANKRD31	0	0
chr5.fa	74632993	74657926	HMGCR	18.73543269	5700
chr5.fa	74666928	74807806	COL4A3BP	15.50688389	5699
chr5.fa	74842835	74895646	POLK	10.07903024	2964
chr5.fa	74970024	75013313	POC5	7.66134615	1140
chr5.fa	75379305	75621416	SV2C	0.484651306	76
chr5.fa	75699149	76003957	IQGAP2	14.86740924	5695
chr5.fa	75911307	75919240	F2RL2	0	0
chr5.fa	76007763	76008686	NCRUPAR	0	0
chr5.fa	76011868	76031595	F2R	0	0
chr5.fa	76114833	76131140	F2RL1	1.985142591	380
chr5.fa	76145826	76217056	S100Z	0	0
chr5.fa	76248680	76265299	CRHBP	0	0
chr5.fa	76326210	76361058	AGGF1	15.92255748	4766
chr5.fa	76372532	76383030	ZBED3	3.226590916	304
chr5.fa	76376259	76376396	SNORA47	0	0
chr5.fa	76382623	76444176	LOC728723	1.002285407	76
chr5.fa	76506706	76724080	PDE8B	24.90276363	7282
chr5.fa	76728069	76788332	WDR41	15.21365329	2584
chr5.fa	76924537	76934522	OTP	0	0
chr5.fa	76986995	77072185	TBCA	72.48624448	3268
chr5.fa	77298150	77590528	AP3B1	22.66279506	5998

chr5.fa	77656339	77776562	SCAMP1	2.566332581	1064
chr5.fa	77781038	77944648	LHFPL2	4.083842387	1368
chr5.fa	78073037	78282357	ARSB	2.229124914	988
chr5.fa	78293429	78365449	DMGDH	9.281569502	1900
chr5.fa	78365547	78385897	BHMT2	28.06474441	4940
chr5.fa	78407604	78428113	BHMT	123.1598669	20444
chr5.fa	78531925	78623038	JMY	9.152801053	5548
chr5.fa	78669786	78809700	HOMER1	8.420251659	2356
chr5.fa	78908243	78982471	PAPD4	13.69734833	2954
chr5.fa	78985659	79096049	CMYA5	17.31295551	14820
chr5.fa	79272539	79287088	MTX3	7.213292193	3800
chr5.fa	79331170	79379107	THBS4	0.710862076	152
chr5.fa	79407050	79551898	SERINC5	12.23224954	5700
chr5.fa	79594917	79596297	LOC644936	0	0
chr5.fa	79615790	79617660	SPZ1	0	0
chr5.fa	79646424	79647785	CRSP8P	0	0
chr5.fa	79703838	79775498	ZFYVE16	11.40813148	5472
chr5.fa	79783800	79838206	FAM151B	2.245842221	228
chr5.fa	79852574	79866304	ANKRD34B	0	0
chr5.fa	79922045	79950800	DHFR	1.001984194	228
chr5.fa	79945819	79946854	MTRNR2L2	0	0
chr5.fa	79952230	80172634	MSH3	18.29159571	5016
chr5.fa	80256558	80521953	RASGRF2	1.403350175	380
chr5.fa	80501462	80501507	RNU5E	0	0
chr5.fa	80529139	80562217	CKMT2	1.394163187	152
chr5.fa	80533384	80597388	LOC100131067	2.360001851	456
chr5.fa	80597402	80608965	ZCCHC9	11.18252313	1064
chr5.fa	80625947	80689988	ACOT12	1.150933896	152
chr5.fa	80715672	81047072	SSBP2	5.288241545	684
chr5.fa	81267844	81551216	ATG10	6.133444505	988
chr5.fa	81569139	81574235	RPS23	215.8660711	47500
chr5.fa	81601166	81614147	ATP6AP1L	1.509979487	76
chr5.fa	82348665	82373272	TMEM167A	13.60608087	4144
chr5.fa	82360023	82360156	SCARNA18	0	0
chr5.fa	82373317	82649579	XRCC4	2.015113259	228
chr5.fa	82767493	82878122	VCAN	0	0
chr5.fa	82934017	83016896	HAPLN1	0	0
chr5.fa	83238126	83680611	EDIL3	0.384799281	76
chr5.fa	85578262	85593362	NBPF22P	0	0
chr5.fa	85913784	85916583	COX7C	523.0889132	14136
chr5.fa	85916314	85916392	MIR3607	0	0
chr5.fa	86410696	86410771	MIR4280	0	0
chr5.fa	86564151	86687733	RASA1	29.63074947	8578

chr5.fa	86690079	86708850	CCNH	13.94810794	1517
chr5.fa	87491023	87564665	TMEM161B	1.323830011	228
chr5.fa	87836597	87980620	LOC645323	0	0
chr5.fa	88014058	88199922	MEF2C	0.500314368	228
chr5.fa	89312438	89312537	MIR3660	0	0
chr5.fa	89689529	89705603	CETN3	2.33349513	152
chr5.fa	89754020	89770585	MBLAC2	3.476597494	988
chr5.fa	89770681	89810369	POLR3G	3.378853959	737
chr5.fa	89811445	89825401	LYSMD3	6.179831268	1748
chr5.fa	89854617	90460033	GPR98	0.592033649	760
chr5.fa	90664541	90679149	ARRDC3	36.86075897	10184
chr5.fa	90676164	90716532	LOC100129716	0	0
chr5.fa	92745065	92916953	FLJ42709	0	0
chr5.fa	92919043	92929786	NR2F1	0	0
chr5.fa	92953431	93447404	FAM172A	9.15295166	2812
chr5.fa	93076015	93077309	POU5F2	0	0
chr5.fa	93486556	93954309	C5orf36	1.383018315	760
chr5.fa	93954391	94031573	ANKRD32	0.3001585	76
chr5.fa	94042289	94620279	MCTP1	0.793394368	228
chr5.fa	94727048	94786144	FAM81B	0	0
chr5.fa	94799599	94890709	TTC37	23.64715831	8956
chr5.fa	94890825	94940806	ARSK	1.026533032	228
chr5.fa	94955980	94957284	GPR150	0.877131511	76
chr5.fa	94982481	94992849	RFESD	0	0
chr5.fa	94994020	95018714	SPATA9	0.932102837	76
chr5.fa	95066850	95132071	RHOBTB3	27.5561467	10184
chr5.fa	95149553	95158577	GLRX	59.07203613	6460
chr5.fa	95187936	95195836	C5orf27	0	0
chr5.fa	95220802	95297775	ELL2	37.23064822	14946
chr5.fa	95414842	95414916	MIR583	0	0
chr5.fa	95726040	95768985	PCSK1	0	0
chr5.fa	95997741	96110187	CAST	59.71211321	19138
chr5.fa	96096514	96149848	ERAP1	3.451898049	1672
chr5.fa	96211644	96255406	ERAP2	0.603479733	228
chr5.fa	96271346	96365115	LNPEP	4.535510897	1444
chr5.fa	96427574	96478520	LIX1	0	0
chr5.fa	96496571	96519005	RIOK2	5.864762737	1824
chr5.fa	98104999	98132198	RGMB	5.091700229	1520
chr5.fa	98105330	98108788	FLJ35946	0.588117883	76
chr5.fa	98190908	98262238	CHD1	12.76313701	5472
chr5.fa	98264838	98266713	LOC100289230	0	0
chr5.fa	99715209	99723958	LOC100133050	0	0
chr5.fa	99871124	99922440	FAM174A	0	0

chr5.fa	100142639	100238970	ST8SIA4	0	0
chr5.fa	101569692	101632253	SLCO4C1	2.790736076	988
chr5.fa	101707652	101834720	SLCO6A1	0	0
chr5.fa	102201527	102366808	PAM	4.074806004	1444
chr5.fa	102421704	102455842	GIN1	2.580188367	608
chr5.fa	102465257	102538909	PPIP5K2	9.538353366	3344
chr5.fa	102594442	102614361	C5orf30	7.985601669	1596
chr5.fa	102884556	102898490	NUDT12	14.43893411	3344
chr5.fa	104435175	104435799	RAB9BP1	0	0
chr5.fa	106712590	107006596	EFNA5	3.21815696	1140
chr5.fa	107194734	107717799	FBXL17	8.144340786	2812
chr5.fa	108083523	108523373	FER	7.759993324	1520
chr5.fa	108670410	108745675	PJA2	43.96004214	14212
chr5.fa	109025156	109203429	MAN2A1	11.82997992	4028
chr5.fa	109218883	109221200	LOC100289673	0	0
chr5.fa	109755198	110062450	TMEM232	1.075781316	228
chr5.fa	110074754	110098484	SLC25A46	14.27070679	2204
chr5.fa	110407390	110413722	TSLP	0	0
chr5.fa	110427870	110466200	WDR36	6.598215769	2888
chr5.fa	110559947	110820748	CAMK4	0	0
chr5.fa	110834022	110848157	STARD4	7.077897065	1064
chr5.fa	111065000	111312628	C5orf13	7.014190569	1359
chr5.fa	111496223	111498198	NCRNA00219	26.84167007	2128
chr5.fa	111497182	111497314	SNORA13	0	0
chr5.fa	111498315	111755010	EPB41L4A	18.38180893	5745
chr5.fa	111755280	111756677	FLJ11235	0.818696239	76
chr5.fa	112043202	112181935	APC	7.960751618	5928
chr5.fa	112196993	112203604	SRP19	25.83772799	1520
chr5.fa	112212081	112258031	REEP5	9.91803203	2052
chr5.fa	112312433	112356667	DCP2	7.150790549	4248
chr5.fa	112357796	112824527	MCC	1.605915746	988
chr5.fa	112768251	112770728	TSSK1B	0	0
chr5.fa	112849391	112930984	YTHDC2	8.709717107	3648
chr5.fa	113698016	113832197	KCNN2	4.319240146	836
chr5.fa	114460459	114516243	TRIM36	8.657155483	2812
chr5.fa	114546527	114598569	PGGT1B	13.01163752	2356
chr5.fa	114602885	114632458	CCDC112	3.978417926	760
chr5.fa	114856608	114880591	FEM1C	8.860775298	3420
chr5.fa	114916895	114938176	TICAM2	0	0
chr5.fa	114948905	114952014	TMED7	2.576272602	532
chr5.fa	115140430	115152405	CDO1	1.418410813	152
chr5.fa	115163894	115177548	ATG12	7.27609505	2204
chr5.fa	115177619	115249778	AP3S1	19.53560435	1672

chr5.fa	115298151	115363299	AQPEP		0	0
chr5.fa	115387163	115394827	LOC644100		0	0
chr5.fa	115420727	115628978	COMMD10		21.54394032	2037
chr5.fa	115779251	115910551	SEMA6A		1.835439857	836
chr5.fa	118172569	118324240	DTWD2		4.142127053	1368
chr5.fa	118407084	118584822	DMXL1		43.11815252	31988
chr5.fa	118604418	118730294	TNFAIP8		7.317059983	1064
chr5.fa	118788138	118878030	HSD17B4		95.83851551	18696
chr5.fa	118965254	118971517	FAM170A		0	0
chr5.fa	119800019	120022964	PRR16		0	0
chr5.fa	121187650	121188523	FTMT		0	0
chr5.fa	121297656	121364295	SRFBP1		7.657731597	1442
chr5.fa	121398890	121414055	LOX		0	0
chr5.fa	121465215	121489266	ZNF474		0	0
chr5.fa	121495871	121518356	LOC728460		0	0
chr5.fa	121647820	121799794	SNCAIP		0	0
chr5.fa	122110750	122165802	SNX2		54.04494603	7421
chr5.fa	122181160	122344902	SNX24		4.728889478	671
chr5.fa	122359078	122372425	PPIC		0.926078582	76
chr5.fa	122424841	122523745	PRDM6		0	0
chr5.fa	122680579	122759286	CEP120		8.746766274	2736
chr5.fa	122847793	122952464	CSNK1G3		9.986407323	2958
chr5.fa	123972610	124080805	ZNF608		8.516187918	3192
chr5.fa	125695788	125829853	GRAMD3		11.39397448	2660
chr5.fa	125878918	125931082	ALDH7A1		43.90100444	10351
chr5.fa	125936607	125962944	PHAX		14.77523814	3572
chr5.fa	125967414	125971974	C5orf48		0	0
chr5.fa	126112315	126172712	LMNB1		0.336304029	76
chr5.fa	126203406	126366440		3-Mar	0.553478418	152
chr5.fa	126378250	126409184	FLJ44606		0.871559076	366
chr5.fa	126626456	126796910	MEGF10		0.297899404	152
chr5.fa	126853309	126890780	PRRC1		30.75065845	9576
chr5.fa	126984713	126994322	CTXN3		7.384531638	912
chr5.fa	127357244	127418766	FLJ33630		5.426498194	1064
chr5.fa	127419483	127525380	SLC12A2		4.788228389	2181
chr5.fa	127593601	127873735	FBN2		10.03294469	7144
chr5.fa	128301210	128369335	SLC27A6		0	0
chr5.fa	128430442	128449719	ISOC1		16.52016357	2128
chr5.fa	128796103	129074376	ADAMTS19		0.449861234	152
chr5.fa	129240523	129522327	CHSY3		0	0
chr5.fa	130494875	130501034	HINT1		319.716695	21356
chr5.fa	130506641	130541119	LYRM7		2.210750936	912
chr5.fa	130599702	130730382	CDC42SE2		36.63003001	8892

chr5.fa	130759614	130970929	RAPGEF6	6.467338832	4028
chr5.fa	130977407	131132756	FNIP1	8.825985226	3876
chr5.fa	131289152	131347349	ACSL6	0	0
chr5.fa	131396347	131398896	IL3	0	0
chr5.fa	131409485	131411859	CSF2	0	0
chr5.fa	131528304	131563556	P4HA2	4.654640537	836
chr5.fa	131593351	131609147	PDLIM4	9.476002328	1444
chr5.fa	131630145	131679899	SLC22A4	3.133064359	456
chr5.fa	131701182	131701291	MIR3936	10.40554486	76
chr5.fa	131705401	131731306	SLC22A5	4.187610177	912
chr5.fa	131746673	131798059	C5orf56	0	0
chr5.fa	131817301	131826465	IRF1	7.085427383	1672
chr5.fa	131877136	131879214	IL5	0	0
chr5.fa	131892616	131980313	RAD50	23.24955748	10184
chr5.fa	131993865	131996801	IL13	0	0
chr5.fa	132009373	132018368	IL4	0	0
chr5.fa	132028323	132073265	KIF3A	6.585564834	2736
chr5.fa	132083137	132089856	CCNI2	0	0
chr5.fa	132086509	132113561	8-Sep	61.50252176	22648
chr5.fa	132149033	132152489	ANKRD43	4.635362921	1064
chr5.fa	132157833	132166590	SHROOM1	33.23777208	9044
chr5.fa	132196878	132200477	GDF9	0	0
chr5.fa	132202319	132204536	UQCRQ	110.4890516	11628
chr5.fa	132209358	132210582	LEAP2	19.42761958	912
chr5.fa	132211071	132299354	AFF4	34.23388263	21776
chr5.fa	132332678	132362240	ZCCHC10	3.777659632	532
chr5.fa	132387662	132440709	HSPA4	60.86545681	13680
chr5.fa	132532152	132948223	FSTL4	0.425312395	152
chr5.fa	132763288	132763398	MIR1289-2	0	0
chr5.fa	133291198	133304406	C5orf15	3.512592417	532
chr5.fa	133307566	133340824	VDAC1	98.89221031	14715
chr5.fa	133450402	133483920	TCF7	1.280154163	356
chr5.fa	133492082	133512724	SKP1	91.17845314	16340
chr5.fa	133532148	133561950	PPP2CA	41.95125435	7103
chr5.fa	133561448	133561450	MIR3661	0	0
chr5.fa	133634115	133702765	CDKL3	0.50603741	76
chr5.fa	133706870	133727799	UBE2B	9.994690674	1746
chr5.fa	133737756	133747598	CDKN2AIPNL	9.127649789	760
chr5.fa	133861798	133918918	PHF15	11.68871115	5016
chr5.fa	133936839	133968533	SAR1B	16.17693164	7144
chr5.fa	133984479	134063601	SEC24A	19.69419286	8352
chr5.fa	134074206	134087843	CAMLG	11.4513555	1672
chr5.fa	134094461	134166812	DDX46	14.710628	5548

chr5.fa	134181370	134195425	C5orf24	12.46794852	4256
chr5.fa	134209460	134237323	TXNDC15	4.964437844	1216
chr5.fa	134240810	134298336	PCBD2	3.850703723	608
chr5.fa	134303596	134347397	CATSPER3	1.674592252	152
chr5.fa	134363424	134369964	PITX1	0.960718048	152
chr5.fa	134670071	134735577	H2AFY	55.873458	7980
chr5.fa	134779904	134783038	C5orf20	0	0
chr5.fa	134784558	134788089	TIFAB	0	0
chr5.fa	134869972	134871639	NEUROG1	0	0
chr5.fa	134906371	134914969	CXCL14	9.862307672	1292
chr5.fa	134984372	134989624	LOC340074	0	0
chr5.fa	135170365	135224326	SLC25A48	1.909236979	152
chr5.fa	135227935	135231516	IL9	0	0
chr5.fa	135266006	135277367	FBXL21	0.637968592	76
chr5.fa	135282600	135290723	LECT2	0	0
chr5.fa	135364584	135399507	TGFBI	0.692638705	129
chr5.fa	135416187	135416286	VTRNA2	0	0
chr5.fa	135465203	135470579	SMAD5OS	0	0
chr5.fa	135468536	135518422	SMAD5	11.29276699	5320
chr5.fa	135527156	135528851	LOC389332	8.098707055	912
chr5.fa	135548999	135701164	TRPC7	0	0
chr5.fa	136310987	136835018	SPOCK1	11.619131	3724
chr5.fa	136953189	137071779	KLHL3	4.709009437	2128
chr5.fa	136983261	136983338	MIR874	0	0
chr5.fa	137087073	137090039	HNRNPA0	38.57797283	7600
chr5.fa	137136882	137146439	NPY6R	0	0
chr5.fa	137203545	137223540	MYOT	0	0
chr5.fa	137225125	137276156	PKD2L2	0	0
chr5.fa	137273642	137368802	FAM13B	25.17415631	8961
chr5.fa	137419774	137427199	WNT8A	0	0
chr5.fa	137450861	137475132	NME5	1.867217802	152
chr5.fa	137475459	137514358	BRD8	17.29548517	5363
chr5.fa	137514417	137523336	KIF20A	0	0
chr5.fa	137523405	137549032	CDC23	15.2020566	3116
chr5.fa	137588069	137610253	GFRA3	0	0
chr5.fa	137620959	137667516	CDC25C	0	0
chr5.fa	137673224	137685418	FAM53C	13.36029127	4180
chr5.fa	137688285	137772716	KDM3B	39.98478695	18088
chr5.fa	137774776	137782658	REEP2	0	0
chr5.fa	137801181	137805004	EGR1	4.014864668	836
chr5.fa	137841784	137878932	ETF1	40.64609953	9956
chr5.fa	137890571	137911318	HSPA9	84.20808903	19603
chr5.fa	137896732	137896799	SNORD63	0	0

chr5.fa	138089107	138270723	CTNNA1	116.256372	29032
chr5.fa	138205079	138211057	LRRTM2	0	0
chr5.fa	138282410	138534065	SIL1	4.606145285	608
chr5.fa	138614469	138614668	SNORA74A	0	0
chr5.fa	138618428	138618873	SNHG4	0	0
chr5.fa	138629333	138667366	MATR3	43.80386333	15671
chr5.fa	138677519	138705409	PAIP2	73.31021194	8056
chr5.fa	138702885	138719039	SLC23A1	7.272028678	1140
chr5.fa	138723257	138725605	MZB1	1.38407256	76
chr5.fa	138727635	138730885	LOC389333	1.408320186	304
chr5.fa	138732456	138739776	SPATA24	5.064591082	228
chr5.fa	138745892	138775214	DNAJC18	1.12171626	380
chr5.fa	138784245	138842320	ECSCR	0	0
chr5.fa	138855113	138862343	TMEM173	0	0
chr5.fa	138940751	139008018	UBE2D2	22.80120231	4339
chr5.fa	139028301	139062680	CXXC5	16.61143103	1596
chr5.fa	139175406	139224048	PSD2	1.522781029	456
chr5.fa	139226364	139422884	NRG2	0	0
chr5.fa	139493708	139499001	PURA	14.26980315	5016
chr5.fa	139505521	139508391	C5orf53	6.777587959	1292
chr5.fa	139554653	139623374	C5orf32	67.92181716	4104
chr5.fa	139624635	139682689	PFDN1	44.95434541	3952
chr5.fa	139712428	139726188	HBEGF	0.970808675	152
chr5.fa	139739787	139754722	SLC4A9	0.699867811	152
chr5.fa	139851824	139919441	ANKHD1	3.106105819	152
chr5.fa	139921757	139921866	ANKHD1-EIF4EBP3	7.941022183	58
chr5.fa	139927251	139927425	EIF4EBP3	0	0
chr5.fa	139929653	139937678	SRA1	3.525544565	456
chr5.fa	139937853	139944189	APBB3	1.063732806	152
chr5.fa	139944420	139948683	SLC35A4	39.20193503	6916
chr5.fa	140011313	140013286	CD14	1.267503228	152
chr5.fa	140019012	140024947	TMCO6	2.478830279	304
chr5.fa	140024990	140027370	NDUFA2	62.63252137	3040
chr5.fa	140027384	140042065	IK	40.55166933	4992
chr5.fa	140027510	140027511	MIR3655	0	0
chr5.fa	140044384	140050380	WDR55	16.16819647	2584
chr5.fa	140050554	140053171	DND1	4.002063127	380
chr5.fa	140053490	140070971	HARS	15.15265771	1976
chr5.fa	140071018	140078890	HARS2	15.62917627	2584
chr5.fa	140080032	140086239	ZMAT2	49.2061139	4940
chr5.fa	140090861	140090958	VTRNA1-1	0	0
chr5.fa	140098511	140098598	VTRNA1-2	0	0
chr5.fa	140105744	140105831	VTRNA1-3	0	0

chr5.fa	140165876	140168269	PCDHA1	0	0
chr5.fa	140174444	140177062	PCDHA2	0	0
chr5.fa	140180783	140183176	PCDHA3	0	0
chr5.fa	140186672	140189169	PCDHA4	0	0
chr5.fa	140201361	140203712	PCDHA5	0	0
chr5.fa	140207650	140210070	PCDHA6	0	0
chr5.fa	140213969	140216323	PCDHA7	0	0
chr5.fa	140220907	140223300	PCDHA8	0	0
chr5.fa	140227357	140230474	PCDHA9	0	0
chr5.fa	140235634	140238021	PCDHA10	0	0
chr5.fa	140247831	140251079	PCDHA11	0	0
chr5.fa	140254931	140257436	PCDHA12	0	0
chr5.fa	140261854	140264280	PCDHA13	0	0
chr5.fa	140306302	140308910	PCDHAC1	0	0
chr5.fa	140345747	140349006	PCDHAC2	0	0
chr5.fa	140430979	140433512	PCDHB1	0	0
chr5.fa	140474237	140476964	PCDHB2	0	0
chr5.fa	140480234	140483406	PCDHB3	0	0
chr5.fa	140501581	140505201	PCDHB4	0	0
chr5.fa	140514800	140517704	PCDHB5	0	0
chr5.fa	140529839	140532868	PCDHB6	0	0
chr5.fa	140535580	140537990	PCDHB17	0	0
chr5.fa	140552243	140555957	PCDHB7	0	0
chr5.fa	140557430	140560021	PCDHB8	0	0
chr5.fa	140560980	140565796	PCDHB16	0	0
chr5.fa	140566893	140571111	PCDHB9	0	0
chr5.fa	140571952	140575213	PCDHB10	0	0
chr5.fa	140579348	140582618	PCDHB11	0	0
chr5.fa	140588291	140591698	PCDHB12	0	0
chr5.fa	140593509	140596993	PCDHB13	0	0
chr5.fa	140603078	140605860	PCDHB14	0	0
chr5.fa	140613938	140617101	PCDHB18	0	0
chr5.fa	140619689	140624312	PCDHB19P	0	0
chr5.fa	140625147	140627801	PCDHB15	0	0
chr5.fa	140682196	140683612	SLC25A2	0	0
chr5.fa	140698057	140700351	TAF7	36.90669392	5624
chr5.fa	140710252	140712672	PCDHGA1	0	0
chr5.fa	140718354	140720962	PCDHGA2	0	0
chr5.fa	140723601	140726024	PCDHGA3	0	0
chr5.fa	140729828	140732236	PCDHGB1	0.475163104	76
chr5.fa	140734768	140737296	PCDHGA4	0	0
chr5.fa	140739703	140742138	PCDHGB2	0	0
chr5.fa	140743898	140746318	PCDHGA5	0	0

chr5.fa	140749962	140752406	PCDHGB3	0	0
chr5.fa	140753651	140756107	PCDHGA6	0	0
chr5.fa	140762467	140765187	PCDHGA7	0	0
chr5.fa	140767452	140769863	PCDHGB4	0	0
chr5.fa	140771483	140774804	PCDHGA8	0	0
chr5.fa	140777695	140780151	PCDHGB5	0	0
chr5.fa	140782520	140784943	PCDHGA9	0	0
chr5.fa	140787770	140790187	PCDHGB6	0	0
chr5.fa	140792743	140795178	PCDHGA10	0	0
chr5.fa	140797282	140799841	PCDHGB7	0	0
chr5.fa	140800537	140803227	PCDHGA11	0	0
chr5.fa	140805853	140807825	PCDHGB8P	0	0
chr5.fa	140810158	140812750	PCDHGA12	0	0
chr5.fa	140855569	140855713	PCDHGC3	0	0
chr5.fa	140864741	140867182	PCDHGC4	0	0
chr5.fa	140868808	140871267	PCDHGC5	0	0
chr5.fa	140894588	140998622	DIAPH1	147.110497	56556
chr5.fa	141000443	141016423	HDAC3	22.85029999	2951
chr5.fa	141016517	141018588	RELL2	0	0
chr5.fa	141018869	141030986	FCHSD1	3.09360549	684
chr5.fa	141032968	141061800	ARAP3	0.435101809	152
chr5.fa	141232673	141257944	PCDH1	9.850710982	3496
chr5.fa	141275191	141276260	LOC729080	0	0
chr5.fa	141303385	141321612	KIAA0141	9.343769933	2584
chr5.fa	141324530	141338627	PCDH12	0	0
chr5.fa	141346437	141368754	RNF14	28.83765632	6156
chr5.fa	141380234	141392620	GNPDA1	20.9288639	3192
chr5.fa	141488324	141534008	NDFIP1	10.5010293	2508
chr5.fa	141689992	141704620	SPRY4	22.69954301	7524
chr5.fa	141971743	142077635	FGF1	2.159092951	608
chr5.fa	142150292	142608572	ARHGAP26	4.431140681	2660
chr5.fa	142657496	142815077	NR3C1	8.432300169	4408
chr5.fa	143191726	143200284	HMHB1	0	0
chr5.fa	143537729	143550278	YIPF5	8.301423232	1900
chr5.fa	143550437	143856944	KCTD16	0.220788942	76
chr5.fa	145138582	145214899	PRELID2	1.445821172	228
chr5.fa	145239296	145252531	GRXCR2	0	0
chr5.fa	145316126	145442879	SH3RF2	3.009115315	608
chr5.fa	145463876	145483946	PLAC8L1	0	0
chr5.fa	145492589	145562294	LARS	28.31053401	9115
chr5.fa	145583163	145668784	RBM27	18.20168371	7904
chr5.fa	145718587	145720083	POU4F3	0	0
chr5.fa	145826873	145891069	TCERG1	16.34771927	4560

chr5.fa	145894417	145895676	GPR151	0	0
chr5.fa	145969068	146461033	PPP2R2B	1.325486681	304
chr5.fa	146614579	146763768	STK32A	0.576671799	70
chr5.fa	146770371	146889619	DPYSL3	8.133647733	3040
chr5.fa	146970706	147162252	JAKMIP2	2.05743365	456
chr5.fa	147204143	147211260	SPINK1	174.5463092	5111
chr5.fa	147258274	147261756	SCGB3A2	0	0
chr5.fa	147272271	147286101	C5orf46	0	0
chr5.fa	147443535	147516925	SPINK5	1.841614718	456
chr5.fa	147549296	147554961	SPINK14	0	0
chr5.fa	147582357	147594700	SPINK6	0	0
chr5.fa	147648423	147665773	SPINK13	0	0
chr5.fa	147691990	147695481	SPINK7	0	0
chr5.fa	147715122	147719412	SPINK9	0	0
chr5.fa	147763546	147822399	FBXO38	25.37175187	7372
chr5.fa	147830595	148033739	HTR4	0	0
chr5.fa	148206156	148208197	ADRB2	2.242077062	304
chr5.fa	148361713	148442737	SH3TC2	5.568670609	9828
chr5.fa	148521054	148639999	ABLIM3	23.57245755	6785
chr5.fa	148651401	148721367	AFAP1L1	0.271693895	76
chr5.fa	148724977	148734146	GRPEL2	9.759142308	2660
chr5.fa	148737570	148749221	PCYOX1L	0.448656383	76
chr5.fa	148753830	148758838	IL17B	0	0
chr5.fa	148786440	148812399	LOC728264	0	0
chr5.fa	148874845	148931007	CSNK1A1	97.41355695	20368
chr5.fa	148961135	149014527	ARHGEF37	40.40527994	13052
chr5.fa	149109815	149234585	PPARGC1B	3.382920331	2432
chr5.fa	149237519	149324356	PDE6A	0	0
chr5.fa	149340300	149366963	SLC26A2	3.260176137	1748
chr5.fa	149372683	149380168	TIGD6	3.990466436	912
chr5.fa	149380218	149432706	HMGXB3	23.71688906	8436
chr5.fa	149432854	149492935	CSF1R	0	0
chr5.fa	149493402	149535422	PDGFRB	0	0
chr5.fa	149546344	149564121	CDX1	0	0
chr5.fa	149569520	149590635	SLC6A7	0	0
chr5.fa	149599054	149669403	CAMK2A	0.233439877	76
chr5.fa	149675909	149682525	ARSI	0	0
chr5.fa	149737202	149779871	TCOF1	6.658458318	2650
chr5.fa	149781200	149792323	CD74	10.6475693	1064
chr5.fa	149823792	149829319	RPS14	1227.614377	64068
chr5.fa	149887674	149937773	NDST1	7.127145348	3800
chr5.fa	149980642	150038792	SYNPO	3.606872006	1976
chr5.fa	150040404	150058925	MYOZ3	0	0

chr5.fa	150070352	150080669	RBM22	20.72283438	3228
chr5.fa	150088309	150138657	DCTN4	33.69004302	9648
chr5.fa	150157508	150176298	C5orf62	1.961647997	304
chr5.fa	150226085	150228231	IRGM	0	0
chr5.fa	150273954	150284545	ZNF300	1.033310319	228
chr5.fa	150309998	150326146	ZNF300P1	3.310779879	532
chr5.fa	150399999	150408554	GPX3	497.2319076	58140
chr5.fa	150409506	150460997	TNIP1	31.5222149	6840
chr5.fa	150480267	150537443	ANXA6	13.87129869	2869
chr5.fa	150560613	150603654	CCDC69	16.83749119	3800
chr5.fa	150632613	150649953	GM2A	5.296223682	1292
chr5.fa	150655926	150683334	SLC36A3	0	0
chr5.fa	150694539	150727151	SLC36A2	3.011223804	684
chr5.fa	150827163	150871940	SLC36A1	6.146396653	2356
chr5.fa	150883653	150948505	FAT2	1.338589435	1292
chr5.fa	151041009	151066517	SPARC	0	0
chr5.fa	151122383	151138210	ATOX1	56.17708044	1824
chr5.fa	151151476	151184915	G3BP1	36.74057509	6916
chr5.fa	151202074	151304397	GLRA1	0	0
chr5.fa	151771102	151784840	NMUR2	0	0
chr5.fa	152870084	153193429	GRIA1	0	0
chr5.fa	153371269	153418497	FAM114A2	8.346153324	1596
chr5.fa	153418519	153437014	MFAP3	2.141020186	760
chr5.fa	153570295	153800543	GALNT10	2.883208388	1140
chr5.fa	153825517	153840613	SAP30L	13.32851333	5548
chr5.fa	153854532	153857824	HAND1	0	0
chr5.fa	153975572	153975632	MIR3141	0	0
chr5.fa	154092462	154197163	LARP1	54.51619337	23858
chr5.fa	154198052	154230213	C5orf4	2.291475952	456
chr5.fa	154238198	154256352	CNOT8	17.97818385	2964
chr5.fa	154266976	154317776	GEMIN5	12.28481117	4408
chr5.fa	154320633	154348971	MRPL22	3.607323825	760
chr5.fa	154393260	154397685	KIF4B	0	0
chr5.fa	155753767	156194798	SGCD	0.233891696	152
chr5.fa	156277549	156279539	PPP1R2P3	0.574864522	76
chr5.fa	156346293	156390266	TIMD4	2.508198521	228
chr5.fa	156351100	156352013	LOC100286948	0	0
chr5.fa	156456531	156485970	HAVCR1	0.608901563	76
chr5.fa	156512843	156536138	HAVCR2	1.973395294	304
chr5.fa	156565451	156569921	MED7	6.984069295	684
chr5.fa	156589344	156593279	FAM71B	0	0
chr5.fa	156607907	156682109	ITK	0	0
chr5.fa	156693091	156822606	CYFIP2	28.77997408	13604

chr5.fa	156768607	156772729	C5orf40	0	0
chr5.fa	156887027	156901730	NIPAL4	4.887026169	1064
chr5.fa	156904312	157002783	ADAM19	0.176661275	76
chr5.fa	157052687	157079428	SOX30	0.350611634	76
chr5.fa	157098561	157107162	C5orf52	0	0
chr5.fa	157158323	157166772	THG1L	14.03350175	1216
chr5.fa	157170755	157183746	LSM11	4.022394987	684
chr5.fa	157212751	157286183	CLINT1	43.0207102	11386
chr5.fa	158122923	158526788	EBF1	0	0
chr5.fa	158584417	158637061	RNF145	2.389068881	684
chr5.fa	158690089	158713048	UBLCP1	7.076993426	1124
chr5.fa	158741791	158757481	IL12B	0	0
chr5.fa	158875564	158893284	LOC285627	0	0
chr5.fa	159343740	159400017	ADRA1B	0	0
chr5.fa	159436180	159492550	TTC1	26.57825952	2483
chr5.fa	159502892	159546452	PWWP2A	5.553459366	2128
chr5.fa	159614374	159665729	FABP6	3.960646374	228
chr5.fa	159678671	159739573	CCNJL	5.244113878	1140
chr5.fa	159774775	159797648	C1QTNF2	0	0
chr5.fa	159820155	159827060	C5orf54	2.446600515	456
chr5.fa	159828648	159846168	SLU7	25.07852127	5928
chr5.fa	159848865	159855746	PTTG1	1.607572416	76
chr5.fa	159901409	159901490	MIR3142	0	0
chr5.fa	159912359	159912457	MIR146A	0	0
chr5.fa	159990127	160279219	ATP10B	5.446227629	2736
chr5.fa	160358786	160365633	LOC285629	0	0
chr5.fa	160715436	160975130	GABRB2	0.154522138	76
chr5.fa	161112658	161129598	GABRA6	0	0
chr5.fa	161274197	161326965	GABRA1	0	0
chr5.fa	161494648	161582545	GABRG2	0	0
chr5.fa	162864577	162872022	CCNG1	85.07226839	13376
chr5.fa	162880586	162887143	NUDCD2	18.98468624	1195
chr5.fa	162887517	162918953	HMMR	0	0
chr5.fa	162930231	162946328	MAT2B	37.28034832	5624
chr5.fa	166711843	167691162	ODZ2	0.237355642	152
chr5.fa	167719065	167899308	WWC1	60.46604871	27056
chr5.fa	167913463	167946309	RARS	48.71814926	6916
chr5.fa	167956582	167957639	FBLL1	0	0
chr5.fa	167982628	168006614	PANK3	32.10445913	7220
chr5.fa	167987901	167987978	MIR103-1	0	0
chr5.fa	168093071	168728133	SLIT3	0.212806804	76
chr5.fa	168195151	168195260	MIR218-2	0	0
chr5.fa	168690605	168690698	MIR585	0	0

chr5.fa	169010638	169031781	CCDC99	1.716762036	304
chr5.fa	169064251	169510386	DOCK2	0	0
chr5.fa	169290719	169407744	FAM196B	0.322598849	76
chr5.fa	169532917	169536729	FOXI1	1.994028367	304
chr5.fa	169659950	169673235	C5orf58	0	0
chr5.fa	169675088	169724822	LCP2	0	0
chr5.fa	169758435	169762104	LOC257358	0	0
chr5.fa	169780881	170163636	KCNIP1	0.538116568	76
chr5.fa	169805167	169816638	KCNMB1	0	0
chr5.fa	170210723	170241050	GABRP	0.345942837	76
chr5.fa	170288896	170727019	RANBP17	2.741035973	831
chr5.fa	170736288	170739138	TLX3	0	0
chr5.fa	170813660	170813764	MIR3912	0	0
chr5.fa	170814708	170837888	NPM1	89.38232155	10564
chr5.fa	170846667	170884630	FGF18	0	0
chr5.fa	171288556	171433877	FBXW11	26.38698943	8126
chr5.fa	171469074	171615346	STK10	7.986957127	3192
chr5.fa	171621176	171630458	EFCAB9	0	0
chr5.fa	171636650	171710795	UBTD2	15.94484722	3192
chr5.fa	171760503	171881527	SH3PXD2B	10.44967252	5396
chr5.fa	172068276	172118533	NEURL1B	1.429254471	608
chr5.fa	172195093	172198203	DUSP1	32.80010997	4408
chr5.fa	172261223	172379688	ERGIC1	73.99004911	14424
chr5.fa	172381786	172386371	LOC100268168	0	0
chr5.fa	172386439	172396774	RPL26L1	12.89702607	608
chr5.fa	172410763	172461900	ATP6V0E1	94.34239181	5525
chr5.fa	172447729	172447932	SNORA74B	0	0
chr5.fa	172483355	172566291	C5orf41	7.489654886	4332
chr5.fa	172571445	172591390	BNIP1	7.432575071	684
chr5.fa	172659107	172662315	NKX2-5	0	0
chr5.fa	172741726	172756506	STC2	0.214162261	76
chr5.fa	173006646	173012075	LOC285593	0	0
chr5.fa	173034148	173043666	BOD1	21.89786529	2812
chr5.fa	173315331	173387313	CPEB4	27.66518571	14271
chr5.fa	173416162	173433143	C5orf47	0	0
chr5.fa	173472724	173536182	HMP19	0	0
chr5.fa	174151575	174157902	MSX2	13.47821606	1976
chr5.fa	174867675	174871163	DRD1	0	0
chr5.fa	174905514	174955621	SFXN1	17.94580348	3564
chr5.fa	175085040	175113245	HRH2	0	0
chr5.fa	175223610	175311023	CPLX2	0	0
chr5.fa	175386534	175395545	THOC3	1.252291984	152
chr5.fa	175511909	175543457	FAM153B	0.546399918	76

chr5.fa	175570088	175626298	LOC643201	1.066744934	304
chr5.fa	175665370	175772990	C5orf25	5.561742716	760
chr5.fa	175773066	175788809	KIAA1191	58.3033412	10944
chr5.fa	175792502	175800503	ARL10	0.475916136	76
chr5.fa	175811232	175815540	NOP16	19.18333604	684
chr5.fa	175815784	175816751	HIGD2A	86.17350219	3496
chr5.fa	175819456	175843540	CLTB	31.65911609	2491
chr5.fa	175875356	175937075	FAF2	9.126444938	2736
chr5.fa	175953700	175964421	RNF44	12.12682508	3344
chr5.fa	175969512	176022769	CDHR2	0.261603268	76
chr5.fa	176022803	176037131	GPRIN1	0.271995108	76
chr5.fa	176047210	176057557	SNCB	0	0
chr5.fa	176056440	176056501	MIR4281	0	0
chr5.fa	176057683	176073642	EIF4E1B	0	0
chr5.fa	176074388	176086059	TSPAN17	17.6909775	3040
chr5.fa	176237560	176307869	UNC5A	0	0
chr5.fa	176307900	176326333	HK3	0	0
chr5.fa	176332006	176433795	UIMC1	8.852040128	1748
chr5.fa	176449697	176493745	ZNF346	2.607297514	532
chr5.fa	176513921	176525126	FGFR4	1.820831039	380
chr5.fa	176560080	176727214	NSD1	26.99589099	23408
chr5.fa	176728199	176730744	RAB24	15.41561643	1650
chr5.fa	176730835	176731851	PRELID1	48.64269546	1518
chr5.fa	176732501	176739292	MXD3	0	0
chr5.fa	176758563	176778885	LMAN2	10.62136379	1292
chr5.fa	176784844	176799599	RGS14	1.427145982	228
chr5.fa	176811432	176825849	SLC34A1	3.637896919	836
chr5.fa	176827108	176827637	PFN3	0	0
chr5.fa	176829139	176836577	F12	0	0
chr5.fa	176853687	176869850	GRK6	8.600527487	1748
chr5.fa	176873796	176883287	PRR7	0	0
chr5.fa	176883614	176900694	DBN1	4.487316858	988
chr5.fa	176910395	176924602	PDLIM7	0	0
chr5.fa	176928906	176937427	DOK3	0	0
chr5.fa	176938578	176943967	DDX41	18.49657099	2584
chr5.fa	176946790	176981548	FAM193B	5.957686869	1824
chr5.fa	177019213	177023099	TMED9	9.860199183	912
chr5.fa	177027119	177037346	B4GALT7	7.290553262	836
chr5.fa	177045501	177099278	LOC202181	2.403828306	456
chr5.fa	177150365	177207505	FAM153A	0.621251285	74
chr5.fa	177302262	177311269	LOC728554	3.132461934	380
chr5.fa	177419236	177423243	PROP1	0	0
chr5.fa	177435689	177474656	FAM153C	0	0

chr5.fa	177540556	177553107	N4BP3	2.485306353	988
chr5.fa	177558028	177575479	RMND5B	17.09322081	2052
chr5.fa	177576465	177580961	NHP2	54.9518976	3116
chr5.fa	177611511	177614433	GMCL1L	0	0
chr5.fa	177631508	177638184	HNRNPAB	66.81079395	8056
chr5.fa	177635540	177659803	AGXT2L2	9.666368782	1292
chr5.fa	177664617	178017556	COL23A1	0	0
chr5.fa	178029665	178054054	CLK4	3.652505737	608
chr5.fa	178138522	178157703	ZNF354A	2.239968573	380
chr5.fa	178191864	178203277	AACSP1	0	0
chr5.fa	178286954	178311424	ZNF354B	1.56299293	228
chr5.fa	178322916	178360210	ZFP2	0.951832272	152
chr5.fa	178368194	178393218	ZNF454	1.624289723	228
chr5.fa	178405330	178422124	GRM6	0	0
chr5.fa	178450776	178461388	ZNF879	2.637418789	456
chr5.fa	178487607	178507691	ZNF354C	1.929418233	304
chr5.fa	178537852	178772431	ADAMTS2	0	0
chr5.fa	178977571	179037019	RUFY1	19.21315611	3484
chr5.fa	179041179	179050722	HNRNPH1	50.73341312	7576
chr5.fa	179068558	179072047	C5orf60	0	0
chr5.fa	179105559	179107975	CBY3	0	0
chr5.fa	179125930	179158639	CANX	52.2527302	17174
chr5.fa	179159851	179204287	MAML1	12.20001978	4636
chr5.fa	179220986	179223513	LTC4S	1.716009004	76
chr5.fa	179224598	179233952	MGAT4B	20.87283833	3268
chr5.fa	179225278	179225346	MIR1229	0	0
chr5.fa	179233388	179265077	SQSTM1	136.0532784	23587
chr5.fa	179267872	179285840	C5orf45	1.970081954	76
chr5.fa	179289071	179334856	TBC1D9B	23.04729313	7904
chr5.fa	179382474	179499109	RNF130	17.97170778	2204
chr5.fa	179442303	179442397	MIR340	0	0
chr5.fa	179527795	179636130	RASGEF1C	0	0
chr5.fa	179660595	179719071	MAPK9	15.13563919	5852
chr5.fa	179727700	179780315	GFPT2	0.760260967	152
chr5.fa	179921417	180005353	CNOT6	21.12781491	8664
chr5.fa	180017105	180018487	SCGB3A1	2.456239323	76
chr5.fa	180028506	180076624	FLT4	0	0
chr5.fa	180166123	180167058	OR2Y1	0	0
chr5.fa	180217541	180237137	MGAT1	7.557126541	1976
chr5.fa	180256959	180262726	LOC729678	17.81025775	3420
chr5.fa	180274611	180288286	ZFP62	10.24002845	2736
chr5.fa	180326077	180377906	BTNL8	0	0
chr5.fa	180415845	180433727	BTNL3	0	0

chr5.fa	180467225	180488523	BTNL9	0	0
chr5.fa	180581943	180582890	OR2V2	0	0
chr5.fa	180620924	180632177	TRIM7	0	0
chr5.fa	180650306	180662808	TRIM41	13.59614085	3268
chr5.fa	180663928	180670906	GNB2L1	1182.796481	87096
chr5.fa	180668818	180668889	SNORD96A	0	0
chr5.fa	180670314	180670376	SNORD95	0	0
chr5.fa	180683386	180688119	TRIM52	4.109897289	608
chr6.fa	292101	351355	DUSP22	13.91151059	1368
chr6.fa	391739	411443	IRF4	0	0
chr6.fa	485138	693109	EXOC2	12.05664251	3556
chr6.fa	655939	656964	HUS1B	0	0
chr6.fa	961241	1101567	LOC285768	0.978338993	152
chr6.fa	1312675	1314993	FOXQ1	36.52475616	5624
chr6.fa	1390069	1395832	FOXF2	0.523357143	76
chr6.fa	1610681	1614129	FOXC1	3.31861141	760
chr6.fa	1624041	2245846	GMDS	11.43057183	1269
chr6.fa	2622972	2635298	C6orf195	0	0
chr6.fa	2663863	2751154	MYLK4	0.993550237	380
chr6.fa	2765666	2785979	WRNIP1	22.87484883	4028
chr6.fa	2833734	2842081	SERPINB1	22.14802248	1900
chr6.fa	2854891	2876744	MGC39372	0.914180679	76
chr6.fa	2887504	2903545	SERPINB9	1.6568207	456
chr6.fa	2948393	2972399	SERPINB6	65.39027465	8892
chr6.fa	2988201	2991405	DKFZP686I15217	0.81493108	152
chr6.fa	3000067	3019994	NQO2	33.1624689	2508
chr6.fa	3020390	3025005	LOC401233	0.743844872	228
chr6.fa	3045618	3046034	FAM136BP	0	0
chr6.fa	3077058	3115421	RIPK1	22.51294172	5776
chr6.fa	3118926	3153432	BPHL	18.29490905	2240
chr6.fa	3153902	3157783	TUBB2A	4.948925388	532
chr6.fa	3224495	3227968	TUBB2B	0	0
chr6.fa	3259162	3268300	PSMG4	9.762154435	608
chr6.fa	3269207	3456793	SLC22A23	9.605373202	4180
chr6.fa	3722836	3752246	C6orf145	6.260405677	912
chr6.fa	3849632	3851551	FAM50B	5.659335645	608
chr6.fa	4021569	4065217	PRPF4B	13.15637024	6536
chr6.fa	4068601	4079391	C6orf146	0	0
chr6.fa	4079440	4130999	C6orf201	0	0
chr6.fa	4115927	4135831	PECI	60.9976892	5735
chr6.fa	4706393	4955778	CDYL	14.98759312	4104
chr6.fa	4995280	5004271	RPP40	3.950405141	304
chr6.fa	5085720	5087455	PPP1R3G	1.318709394	152

chr6.fa	5108653	5261172	LYRM4	9.76817869	1292
chr6.fa	5148467	5148556	MIR3691	0	0
chr6.fa	5261584	5771816	FARS2	6.839035358	836
chr6.fa	5998233	6007633	NRN1	0	0
chr6.fa	6144311	6320924	F13A1	1.481514883	380
chr6.fa	6346698	6623059	LY86-AS1	0	0
chr6.fa	6588934	6655216	LY86	0	0
chr6.fa	7107830	7252213	RREB1	27.89214951	16492
chr6.fa	7281288	7313541	SSR1	3.367106662	2204
chr6.fa	7326887	7389942	CAGE1	0	0
chr6.fa	7390062	7418270	RIOK1	7.103801361	1216
chr6.fa	7541870	7586946	DSP	71.02957965	45889
chr6.fa	7590432	7612200	SNRNP48	3.017248059	836
chr6.fa	7727011	7881482	BMP6	0	0
chr6.fa	7910046	7911047	TXNDC5	0	0
chr6.fa	7986335	7990577	PIP5K1P1	0	0
chr6.fa	8054503	8054605	MUTED	0	0
chr6.fa	8073593	8080263	EEF1E1	8.554140724	447
chr6.fa	8086641	8086766	SCARNA27	0	0
chr6.fa	8413301	8435794	SLC35B3	14.41468648	2120
chr6.fa	8652442	8654080	HULC	0	0
chr6.fa	10396916	10419797	TFAP2A	6.155282429	1672
chr6.fa	10414914	10416402	LOC100130275	0	0
chr6.fa	10428018	10435055	C6orf218	0	0
chr6.fa	10521568	10629601	GCNT2	7.993282594	3876
chr6.fa	10671651	10695030	C6orf52	7.795235215	456
chr6.fa	10695188	10709970	PAK1IP1	81.45846849	8086
chr6.fa	10723148	10731362	TMEM14C	62.67860692	4940
chr6.fa	10747995	10757214	TMEM14B	68.20676442	4402
chr6.fa	10762956	10831110	MAK	0	0
chr6.fa	10873456	10882098	GCM2	0	0
chr6.fa	10887064	10974541	SYCP2L	0.365822878	76
chr6.fa	10980993	11044624	ELOVL2	1.686188942	456
chr6.fa	11094266	11138969	LOC221710	2.195991512	684
chr6.fa	11102722	11112071	ERVFRDE1	0	0
chr6.fa	11183531	11382581	NEDD9	11.2739412	4180
chr6.fa	11538511	11583757	TMEM170B	0.821256547	456
chr6.fa	11713888	11779280	C6orf105	3.080201523	380
chr6.fa	12012724	12165232	HIVEP1	13.80126673	8132
chr6.fa	12290529	12297427	EDN1	2.725222304	380
chr6.fa	12717833	13287528	PHACTR1	6.899880333	985
chr6.fa	13305184	13328787	TBC1D7	15.86065826	1368
chr6.fa	13363819	13487787	GFOD1	0.764628551	152

chr6.fa	13469509	13486415	C6orf114	0	0
chr6.fa	13574792	13614785	SIRT5	5.892173097	2052
chr6.fa	13615559	13621127	NOL7	44.73385768	2596
chr6.fa	13621730	13711796	RANBP9	31.21467669	6452
chr6.fa	13791020	13814789	CCDC90A	23.33239099	1904
chr6.fa	13924677	13980240	RNF182	0.301513957	76
chr6.fa	14117865	14137148	CD83	2.772512705	456
chr6.fa	15246527	15522253	JARID2	11.13583516	4256
chr6.fa	15523032	15663289	DTNBP1	6.715538133	1121
chr6.fa	16129317	16148478	MYLIP	36.89886238	7524
chr6.fa	16238811	16295780	GMPR	66.12764345	6652
chr6.fa	16299343	16761721	ATXN1	6.672163498	4712
chr6.fa	17102489	17131603	FLJ23152	0.865836033	76
chr6.fa	17281809	17294099	RBM24	0	0
chr6.fa	17393736	17558023	CAP2	2.26873439	456
chr6.fa	17600518	17611950	FAM8A1	3.377197289	1064
chr6.fa	17615269	17706818	NUP153	27.29032645	10305
chr6.fa	17760585	17987799	KIF13A	16.61941316	7133
chr6.fa	18120718	18122851	NHLRC1	1.60907848	228
chr6.fa	18128545	18155374	TPMT	21.14152009	4558
chr6.fa	18155619	18224084	KDM1B	9.716520704	2500
chr6.fa	18224400	18264799	DEK	22.32603921	4256
chr6.fa	18387581	18469105	RNF144B	21.36486934	7144
chr6.fa	18572015	18572111	MIR548A1	0	0
chr6.fa	19837617	19840915	ID4	23.3941396	3641
chr6.fa	20100935	20212670	MBOAT1	3.131708902	681
chr6.fa	20402137	20493945	E2F3	3.65612029	1216
chr6.fa	20534688	21232634	CDKAL1	10.14785735	2204
chr6.fa	21593972	21598849	SOX4	28.626958	9272
chr6.fa	21666675	22194616	FLJ22536	0.605889435	76
chr6.fa	22134831	22147422	LOC729177	0	0
chr6.fa	22287477	22303082	PRL	0	0
chr6.fa	22569678	22570750	HDGFL1	0	0
chr6.fa	24126414	24147757	NRSN1	0	0
chr6.fa	24171983	24383520	DCDC2	7.507125225	2106
chr6.fa	24357131	24358512	KAAG1	1.153795417	76
chr6.fa	24403153	24425816	MRS2	10.20689505	2269
chr6.fa	24428405	24489799	GPLD1	1.361933423	358
chr6.fa	24495197	24537435	ALDH5A1	16.38311177	5624
chr6.fa	24544332	24646383	KIAA0319	0.490223741	228
chr6.fa	24650205	24667115	TDP2	101.6769221	12996
chr6.fa	24667263	24705089	ACOT13	16.67573995	4636
chr6.fa	24705296	24719403	C6orf62	107.5912344	16188

chr6.fa	24775164	24786278	GMNN	35.79838162	2888
chr6.fa	24804513	24911195	FAM65B	0.374708654	152
chr6.fa	25081295	25138620	CMAH	0	0
chr6.fa	25279656	25620758	LRRC16A	14.10398554	5086
chr6.fa	25652429	25702008	SCGN	3.095563373	304
chr6.fa	25726291	25726790	HIST1H2AA	0	0
chr6.fa	25727137	25727573	HIST1H2BA	0	0
chr6.fa	25754927	25781403	SLC17A4	0	0
chr6.fa	25783126	25832287	SLC17A1	3.105352787	380
chr6.fa	25845328	25874471	SLC17A3	4.4315925	532
chr6.fa	25912984	25930946	SLC17A2	0	0
chr6.fa	25963071	25985352	TRIM38	16.80164687	3648
chr6.fa	26017260	26018040	HIST1H1A	0	0
chr6.fa	26020718	26021186	HIST1H3A	2.44057626	76
chr6.fa	26021907	26022278	HIST1H4A	0	0
chr6.fa	26027124	26027480	HIST1H4B	118.6288742	2812
chr6.fa	26031817	26032288	HIST1H3B	0	0
chr6.fa	26033320	26033796	HIST1H2AB	0	0
chr6.fa	26043455	26043885	HIST1H2BB	5.311434926	152
chr6.fa	26045639	26046097	HIST1H3C	4.987330013	152
chr6.fa	26055968	26056699	HIST1H1C	118.8391207	5776
chr6.fa	26087509	26095469	HFE	0.515073793	76
chr6.fa	26104176	26104565	HIST1H4C	99.78636034	2584
chr6.fa	26107640	26108364	HIST1H1T	0	0
chr6.fa	26123695	26124132	HIST1H2BC	164.6355063	4788
chr6.fa	26124373	26124918	HIST1H2AC	123.6847301	4484
chr6.fa	26156559	26157343	HIST1H1E	46.65920954	2432
chr6.fa	26158349	26171576	HIST1H2BD	98.25514536	5852
chr6.fa	26184024	26184458	HIST1H2BE	5.262638461	152
chr6.fa	26188938	26189304	HIST1H4D	68.61415465	1672
chr6.fa	26197012	26197497	HIST1H3D	9.792576922	316
chr6.fa	26199012	26199471	HIST1H2AD	13.95865039	76
chr6.fa	26199787	26200216	HIST1H2BF	23.95695561	684
chr6.fa	26204873	26205249	HIST1H4E	224.671122	5624
chr6.fa	26216428	26216872	HIST1H2BG	51.44307035	1520
chr6.fa	26217148	26217711	HIST1H2AE	28.41219331	1064
chr6.fa	26225383	26225844	HIST1H3E	7.432575071	228
chr6.fa	26234440	26235216	HIST1H1D	5.89247431	304
chr6.fa	26240654	26241021	HIST1H4F	9.331118998	228
chr6.fa	26246839	26247205	HIST1H4G	0	0
chr6.fa	26250370	26250835	HIST1H3F	0	0
chr6.fa	26251879	26252303	HIST1H2BH	0	0
chr6.fa	26271146	26271612	HIST1H3G	0	0

chr6.fa	26273204	26273640	HIST1H2BI	7.857736859	228
chr6.fa	26285354	26285727	HIST1H4H	110.1762421	2736
chr6.fa	26365398	26378548	BTN3A2	3.584582263	978
chr6.fa	26383324	26395100	BTN2A2	1.308769374	337
chr6.fa	26402465	26415444	BTN3A1	3.004295911	836
chr6.fa	26421619	26430816	BTN2A3	1.382867709	228
chr6.fa	26440700	26453643	BTN3A3	3.812750917	760
chr6.fa	26458153	26476849	BTN2A1	2.822363414	684
chr6.fa	26501495	26510652	BTN1A1	0	0
chr6.fa	26521934	26527612	HCG11	2.821760988	1064
chr6.fa	26538572	26547164	HMGN4	36.05983429	4712
chr6.fa	26597180	26600277	ABT1	6.093684423	912
chr6.fa	26634611	26659963	ZNF322A	2.38455069	760
chr6.fa	26839266	26924333	GUSBL1	2.86152107	228
chr6.fa	26924772	26991753	NCRNA00240	0	0
chr6.fa	26987145	26988085	LOC100270746	0	0
chr6.fa	27100095	27100575	HIST1H2BJ	7.138892645	228
chr6.fa	27100817	27101314	HIST1H2AG	2.298403845	76
chr6.fa	27106072	27114637	HIST1H2BK	45.020311	2508
chr6.fa	27107088	27107457	HIST1H4I	0	0
chr6.fa	27114908	27115346	HIST1H2AH	2.607297514	76
chr6.fa	27115405	27115467	MIR3143	0	0
chr6.fa	27215502	27224399	PRSS16	5.593219448	1064
chr6.fa	27276842	27280011	POM121L2	0	0
chr6.fa	27292578	27293741	FKSG83	0	0
chr6.fa	27325602	27343153	ZNF204P	0.273199959	76
chr6.fa	27356524	27369227	ZNF391	0.717187544	76
chr6.fa	27418521	27440897	ZNF184	3.320870505	684
chr6.fa	27775257	27775709	HIST1H2BL	0	0
chr6.fa	27775977	27776445	HIST1H2AI	0	0
chr6.fa	27777842	27778314	HIST1H3H	2.419943187	76
chr6.fa	27782080	27782518	HIST1H2AJ	0	0
chr6.fa	27782822	27783267	HIST1H2BM	0	0
chr6.fa	27791903	27792258	HIST1H4J	3.215144832	76
chr6.fa	27798952	27799305	HIST1H4K	0	0
chr6.fa	27805658	27806117	HIST1H2AK	0	0
chr6.fa	27806440	27806888	HIST1H2BN	5.098477516	152
chr6.fa	27833107	27833576	HIST1H2AL	0	0
chr6.fa	27834570	27835359	HIST1H1B	2.897817206	152
chr6.fa	27839623	27840099	HIST1H3I	0	0
chr6.fa	27840926	27841289	HIST1H4L	3.144510444	76
chr6.fa	27858093	27858570	HIST1H3J	0	0
chr6.fa	27860477	27860963	HIST1H2AM	4.700575481	152

chr6.fa	27861203	27861669	HIST1H2BO	0	0
chr6.fa	27878963	27880174	OR2B2	0	0
chr6.fa	27925019	27925960	OR2B6	0	0
chr6.fa	28048482	28057340	ZNF165	3.852209786	608
chr6.fa	28058929	28063493	ZSCAN12P1	0.6168837	152
chr6.fa	28092387	28097856	ZSCAN16	5.386436899	456
chr6.fa	28109716	28125236	ZNF192	5.550146026	1976
chr6.fa	28129551	28137376	ZNF389	0	0
chr6.fa	28183116	28186707	LOC222699	0.955898644	228
chr6.fa	28193029	28201264	ZNF193	4.990191534	608
chr6.fa	28212490	28220002	ZKSCAN4	1.001381768	152
chr6.fa	28227098	28228736	NKAPL	0	0
chr6.fa	28234788	28245980	ZNF187	4.877537968	912
chr6.fa	28249314	28270326	PGBD1	2.920859981	608
chr6.fa	28292515	28324048	ZNF323	1.574137801	456
chr6.fa	28317691	28334524	ZKSCAN3	6.48360432	988
chr6.fa	28346598	28367544	ZSCAN12	3.440150752	1368
chr6.fa	28400432	28411279	ZSCAN23	0.724266044	152
chr6.fa	28471073	28483570	GPX6	0	0
chr6.fa	28493789	28502728	GPX5	0	0
chr6.fa	28539407	28555112	SCAND3	3.98986401	1292
chr6.fa	28827402	28831454	LOC401242	0	0
chr6.fa	28870779	28891768	TRIM27	25.25156799	4978
chr6.fa	28962594	28973037	ZNF311	1.272774451	228
chr6.fa	29004000	29044517	LOC100129636	0	0
chr6.fa	29011990	29012952	OR2W1	0	0
chr6.fa	29053985	29055090	OR2B3	0	0
chr6.fa	29079668	29080603	OR2J3	0	0
chr6.fa	29141311	29142351	OR2J2	0	0
chr6.fa	29274467	29275432	OR14J1	0	0
chr6.fa	29323007	29324054	OR5V1	0	0
chr6.fa	29341200	29343068	OR12D3	0	0
chr6.fa	29364416	29365448	OR12D2	0	0
chr6.fa	29393281	29395509	OR11A1	0	0
chr6.fa	29407793	29408731	OR10C1	0	0
chr6.fa	29426230	29432099	OR2H1	0	0
chr6.fa	29454543	29455679	MAS1L	0	0
chr6.fa	29497183	29501345	LOC100507362	0	0
chr6.fa	29523389	29527702	UBD	2.312410238	152
chr6.fa	29550029	29550105	SNORD32B	0	0
chr6.fa	29555683	29556745	OR2H2	0	0
chr6.fa	29570005	29600962	GABBR1	0.75679702	228
chr6.fa	29624758	29640149	MOG	0	0

chr6.fa	29640169	29644931	ZFP57	0	0
chr6.fa	29691117	29695073	HLA-F	1.588596013	152
chr6.fa	29694378	29716826	LOC285830	0	0
chr6.fa	29718584	29718925	IFITM4P	0	0
chr6.fa	29758808	29760850	HCG4	1.120511409	152
chr6.fa	29794756	29798899	HLA-G	0	0
chr6.fa	29855383	29858856	HLA-H	0.622456136	76
chr6.fa	29866808	29870431	HCG2P7	0	0
chr6.fa	29892369	29893428	HCG4P6	0	0
chr6.fa	29910309	29913661	HLA-A	7.389351042	760
chr6.fa	29942892	29946177	HCG9	0	0
chr6.fa	29968788	30028961	ZNRD1-AS1	0.942042858	152
chr6.fa	29973748	29977733	HLA-J	0.717639363	74
chr6.fa	30029036	30032686	ZNRD1	7.702612296	380
chr6.fa	30034932	30038042	PPP1R11	63.11129903	6688
chr6.fa	30038109	30043626	RNF39	0.601220638	76
chr6.fa	30070674	30080867	TRIM31	0.564623289	76
chr6.fa	30104510	30116512	TRIM40	0	0
chr6.fa	30119723	30128711	TRIM10	0	0
chr6.fa	30130983	30140473	TRIM15	0.866739672	128
chr6.fa	30152232	30181153	TRIM26	23.65739954	5479
chr6.fa	30227339	30234728	HLA-L	0.247596876	76
chr6.fa	30255174	30294927	HCG18	2.920859981	1292
chr6.fa	30295008	30308138	TRIM39	0	0
chr6.fa	30312906	30314341	RPP21	7.35787431	64
chr6.fa	30457183	30461982	HLA-E	20.02236414	3539
chr6.fa	30509155	30525371	GNL1	9.103251557	4332
chr6.fa	30525927	30532473	PRR3	1.991468059	352
chr6.fa	30539170	30559309	ABCF1	36.55156409	8407
chr6.fa	30552109	30552194	MIR877	0	0
chr6.fa	30568182	30585020	PPP1R10	31.86755531	9562
chr6.fa	30585486	30594174	MRPS18B	33.4201564	3404
chr6.fa	30594613	30614598	ATAT1	1.745829066	380
chr6.fa	30614816	30620895	C6orf136	7.664810097	684
chr6.fa	30620988	30640830	DHX16	11.21159016	2508
chr6.fa	30644166	30655672	KIAA1949	0.248952333	76
chr6.fa	30655826	30658769	NRM	0	0
chr6.fa	30667584	30685458	MDC1	2.951583681	1444
chr6.fa	30688157	30693195	TUBB	86.66387653	14288
chr6.fa	30695511	30710453	FLOT1	55.51862938	6746
chr6.fa	30710976	30712327	IER3	8.307598093	684
chr6.fa	30851861	30867933	DDR1	7.497938236	1968
chr6.fa	30875977	30881880	GTF2H4	7.679569521	875

chr6.fa	30881982	30894235	VARS2	3.06288179	760
chr6.fa	30899127	30899952	SFTA2	0	0
chr6.fa	30908777	30921998	DPCR1	0	0
chr6.fa	30951485	30957675	MUC21	0.313110648	76
chr6.fa	30973729	31003179	PBMUCL1	0	0
chr6.fa	31021984	31027653	HCG22	0	0
chr6.fa	31079000	31080332	C6orf15	0	0
chr6.fa	31082608	31107869	PSORS1C1	0	0
chr6.fa	31082865	31088252	CDSN	0	0
chr6.fa	31105311	31107127	PSORS1C2	0.993550237	76
chr6.fa	31110216	31126015	CCHCR1	7.442665698	1444
chr6.fa	31126303	31131992	TCF19	3.462289889	760
chr6.fa	31132114	31138451	POU5F1	0.670951388	76
chr6.fa	31141512	31145676	PSORS1C3	0	0
chr6.fa	31165537	31171745	HCG27	0	0
chr6.fa	31236529	31239855	HLA-C	31.70143648	3210
chr6.fa	31321649	31324989	HLA-B	12.1673382	1270
chr6.fa	31367561	31383036	MICA	2.965288861	456
chr6.fa	31430957	31433586	HCP5	9.016201074	1520
chr6.fa	31439006	31440185	HCG26	0	0
chr6.fa	31465855	31478901	MICB	0	0
chr6.fa	31496739	31497995	MCCD1	8.222806705	380
chr6.fa	31498009	31510225	BAT1	47.17985577	6234
chr6.fa	31504151	31504226	SNORD117	0	0
chr6.fa	31508878	31508955	SNORD84	0	0
chr6.fa	31512240	31514627	ATP6V1G2	0	0
chr6.fa	31514628	31526606	NFKBIL1	4.988986683	532
chr6.fa	31539876	31542098	LTA	0	0
chr6.fa	31543350	31546112	TNF	0	0
chr6.fa	31548336	31550202	LTB	1.281810833	76
chr6.fa	31553956	31556659	LST1	0	0
chr6.fa	31556687	31560762	NCR3	0	0
chr6.fa	31582994	31584798	AIF1	0	0
chr6.fa	31588450	31605554	PRRC2A	40.83330325	18846
chr6.fa	31590856	31590987	SNORA38	0	0
chr6.fa	31606805	31620477	BAG6	44.65373509	11750
chr6.fa	31623671	31625987	APOM	30.08166495	1520
chr6.fa	31626075	31628549	C6orf47	7.399441669	1216
chr6.fa	31629006	31634060	GPANK1	6.509659223	1368
chr6.fa	31634598	31637843	CSNK2B	133.2837778	7071
chr6.fa	31638728	31640227	LY6G5B	0	0
chr6.fa	31644461	31648150	LY6G5C	3.153245613	152
chr6.fa	31654726	31671137	ABHD16A	1.845379878	304

chr6.fa	31674684	31678372	LY6G6F	0	0
chr6.fa	31679753	31681842	LY6G6E	0	0
chr6.fa	31683133	31685581	LY6G6D	0	0
chr6.fa	31686425	31689510	LY6G6C	0	0
chr6.fa	31691161	31692851	C6orf25	0	0
chr6.fa	31694817	31698039	DDAH2	46.43796878	4104
chr6.fa	31698358	31704341	CLIC1	148.2227251	12312
chr6.fa	31707725	31730455	MSH5	2.656395192	484
chr6.fa	31730773	31732624	C6orf26	0	0
chr6.fa	31733371	31745108	C6orf27	0	0
chr6.fa	31745297	31763712	VARS	16.0010234	4560
chr6.fa	31765174	31774743	LSM2	18.52262589	1054
chr6.fa	31777396	31782835	HSPA1L	0.902282775	152
chr6.fa	31783291	31785719	HSPA1A	13.66557039	2204
chr6.fa	31795512	31798031	HSPA1B	17.71417088	2964
chr6.fa	31802693	31807541	C6orf48	111.3092539	6223
chr6.fa	31803040	31803103	SNORD48	0	0
chr6.fa	31804853	31804916	SNORD52	0	0
chr6.fa	31826829	31830709	NEU1	6.079527424	836
chr6.fa	31830970	31846823	SLC44A4	58.39159654	10631
chr6.fa	31847537	31865464	EHMT2	12.98392595	3420
chr6.fa	31867394	31869769	ZBTB12	1.327896383	152
chr6.fa	31895266	31913449	C2	2.016619323	380
chr6.fa	31913721	31919861	CFB	1.742816938	304
chr6.fa	31919864	31926231	RDBP	24.13015294	2187
chr6.fa	31924616	31924717	MIR1236	0	0
chr6.fa	31926581	31937532	SKIV2L	9.213043602	2431
chr6.fa	31937588	31939458	DOM3Z	6.343841607	532
chr6.fa	31938952	31981961	STK19	4.168784381	532
chr6.fa	31973348	32009419	CYP21A2	0	0
chr6.fa	31978762	32077151	TNXB	0.087201089	76
chr6.fa	32083045	32096017	ATF6B	7.000635996	1216
chr6.fa	32096484	32098067	FKBPL	4.258244566	380
chr6.fa	32116140	32119720	PRRT1	0	0
chr6.fa	32120579	32122142	LOC100507547	1.176386373	76
chr6.fa	32121622	32131452	PPT2	0	0
chr6.fa	32132405	32135982	EGFL8	0	0
chr6.fa	32136063	32145888	AGPAT1	19.01013872	2888
chr6.fa	32146162	32146231	RNF5	32.70311946	152
chr6.fa	32148746	32152023	AGER	0.793695581	76
chr6.fa	32152510	32157963	PBX2	14.24977251	3040
chr6.fa	32158543	32162606	GPSM3	5.05721137	456
chr6.fa	32162620	32191844	NOTCH4	0	0

chr6.fa	32260475	32339656	C6orf10	0	0
chr6.fa	32362513	32374900	BTNL2	0	0
chr6.fa	32407619	32412826	HLA-DRA	8.029277517	684
chr6.fa	32485154	32498006	HLA-DRB5	0.979995663	76
chr6.fa	32520490	32527779	HLA-DRB6	0	0
chr6.fa	32546547	32557562	HLA-DRB1	0.982555972	76
chr6.fa	32605183	32611429	HLA-DQA1	0	0
chr6.fa	32627657	32634466	HLA-DQB1	0.935114965	76
chr6.fa	32709163	32714664	HLA-DQA2	0	0
chr6.fa	32723875	32731330	HLA-DQB2	0	0
chr6.fa	32780540	32784825	HLA-DOB	0	0
chr6.fa	32789610	32806547	TAP2	0.556942364	228
chr6.fa	32808494	32812712	PSMB8	27.37587087	2812
chr6.fa	32811863	32814277	LOC100507463	0.858606928	76
chr6.fa	32812986	32821748	TAP1	2.751427812	456
chr6.fa	32821938	32827628	PSMB9	12.01402091	836
chr6.fa	32844255	32847851	PPP1R2P1	0	0
chr6.fa	32861953	32871535	LOC100294145	6.374866519	1520
chr6.fa	32902406	32908847	HLA-DMB	0	0
chr6.fa	32916391	32920899	HLA-DMA	5.100736611	380
chr6.fa	32936437	32949282	BRD2	86.35332619	30704
chr6.fa	32971960	32977389	HLA-DOA	0	0
chr6.fa	33032794	33041378	HLA-DPA1	2.967849169	228
chr6.fa	33043760	33054976	HLA-DPB1	8.889842328	886
chr6.fa	33080293	33096890	HLA-DPB2	0	0
chr6.fa	33130469	33160245	COL11A2	0	0
chr6.fa	33161365	33168432	RXRB	11.14743185	2128
chr6.fa	33168603	33172214	SLC39A7	8.334255421	1358
chr6.fa	33172419	33174607	HSD17B8	14.8169561	973
chr6.fa	33175612	33175721	MIR219-1	0	0
chr6.fa	33176286	33180499	RING1	22.39170359	2584
chr6.fa	33218049	33239662	VPS52	18.49024552	3623
chr6.fa	33239852	33244281	RPS18	1628.216032	58596
chr6.fa	33244917	33246602	B3GALT4	3.394517021	380
chr6.fa	33246880	33257304	WDR46	17.92230889	2812
chr6.fa	33257378	33258711	PFDN6	37.68261794	1824
chr6.fa	33259431	33267176	RGL2	27.00854193	6013
chr6.fa	33267472	33282164	TAPBP	7.294017209	1976
chr6.fa	33282182	33285719	ZBTB22	7.113590775	1289
chr6.fa	33286335	33290793	DAXX	21.57948342	3648
chr6.fa	33332514	33334139	LYPLA2P1	0	0
chr6.fa	33359313	33377699	KIFC1	0	0
chr6.fa	33378773	33384230	PHF1	21.94455327	3344

chr6.fa	33384319	33386065	CUTA	35.86073266	2443
chr6.fa	33387847	33421466	SYNGAP1	0.571400576	228
chr6.fa	33422356	33425320	ZBTB9	6.333148554	1140
chr6.fa	33540323	33548070	BAK1	4.175561667	608
chr6.fa	33551476	33556803	GGNBP1	0	0
chr6.fa	33553883	33561115	C6orf227	0	0
chr6.fa	33589156	33664348	ITPR3	3.675548512	2204
chr6.fa	33665346	33679504	C6orf125	38.45522863	1292
chr6.fa	33689443	33714762	IP6K3	0	0
chr6.fa	33738990	33756906	LEMD2	5.52424173	1140
chr6.fa	33762449	33771793	MLN	0	0
chr6.fa	33967749	33967828	MIR1275	0	0
chr6.fa	33989628	34101443	GRM4	0	0
chr6.fa	34204577	34214008	HMGA1	15.26139551	2432
chr6.fa	34214157	34216904	C6orf1	9.490912359	760
chr6.fa	34255997	34360441	NUDT3	46.61673855	4191
chr6.fa	34385231	34393876	RPS10	160.5006084	6586
chr6.fa	34433853	34504039	PACSIN1	2.901883578	1064
chr6.fa	34505580	34524091	SPDEF	117.6303539	14793
chr6.fa	34555066	34664625	C6orf106	47.41133777	13908
chr6.fa	34724871	34741634	SNRPC	25.27024318	2042
chr6.fa	34759794	34845291	UHRF1BP1	8.674023396	5548
chr6.fa	34845555	34855819	TAF11	9.706430077	988
chr6.fa	34857038	35059190	ANKS1A	34.2284608	14434
chr6.fa	35085849	35109187	TCP11	0	0
chr6.fa	35182190	35218609	SCUBE3	0.447602138	152
chr6.fa	35227510	35263760	ZNF76	12.51177497	2204
chr6.fa	35265595	35289548	DEF6	3.489700249	532
chr6.fa	35310335	35395968	PPARD	16.67920389	4256
chr6.fa	35420138	35434881	FANCE	1.344011265	228
chr6.fa	35436178	35438558	RPL10A	953.5750888	45524
chr6.fa	35441374	35464861	TEAD3	15.85117006	3148
chr6.fa	35465651	35480647	TULP1	0	0
chr6.fa	35541362	35688325	FKBP5	18.5488314	12996
chr6.fa	35694539	35704724	LOC285847	0	0
chr6.fa	35704859	35716685	C6orf81	0	0
chr6.fa	35744392	35747329	C6orf126	0	0
chr6.fa	35748831	35755841	C6orf127	0	0
chr6.fa	35762760	35765102	CLPS	0	0
chr6.fa	35773071	35791852	LHFPL5	0	0
chr6.fa	35800811	35888957	SRPK1	18.07487314	5235
chr6.fa	35911291	35992413	SLC26A8	0	0
chr6.fa	35995454	36079013	MAPK14	14.8499389	4371

chr6.fa	36098262	36107842	MAPK13	40.01279973	5016
chr6.fa	36164550	36200567	BRPF3	19.00396385	7600
chr6.fa	36210945	36276372	PNPLA1	1.321269703	228
chr6.fa	36283535	36304662	C6orf222	0	0
chr6.fa	36333971	36355467	ETV7	1.407868367	152
chr6.fa	36358329	36410543	PXT1	0	0
chr6.fa	36410667	36458315	KCTD20	24.10439925	7828
chr6.fa	36461669	36515247	STK38	34.56446362	8207
chr6.fa	36562090	36572244	SRSF3	36.1664636	8645
chr6.fa	36590213	36590289	MIR3925	0	0
chr6.fa	36644237	36655116	CDKN1A	36.89479601	6460
chr6.fa	36708555	36807220	CPNE5	0.342780103	76
chr6.fa	36822606	36842800	PPIL1	23.07681198	2660
chr6.fa	36853640	36896740	C6orf89	6.612372768	2964
chr6.fa	36916039	36932613	PI16	0	0
chr6.fa	36935917	36953949	MTCH1	130.7177464	16326
chr6.fa	36973423	36996845	FGD2	1.879416918	380
chr6.fa	37137922	37143204	PIM1	16.90089647	3040
chr6.fa	37179954	37225931	TMEM217	0	0
chr6.fa	37237370	37300746	TBC1D22B	13.22655281	2863
chr6.fa	37321748	37362514	RNF8	4.065317803	1520
chr6.fa	37400907	37449284	FTSJD2	10.23279935	2802
chr6.fa	37450697	37467700	C6orf129	35.99974234	1482
chr6.fa	37600284	37665766	MDGA1	0.128467235	76
chr6.fa	37787307	38122399	ZFAND3	42.21767702	9343
chr6.fa	38136227	38607924	BTBD9	8.706554373	5396
chr6.fa	38643702	38670952	GLO1	152.7263074	20748
chr6.fa	38690552	38998567	DNAH8	0.632697369	608
chr6.fa	39016557	39055520	GLP1R	0.36657591	76
chr6.fa	39071840	39082865	C6orf64	6.557702655	836
chr6.fa	39156747	39197251	KCNK5	2.723113814	684
chr6.fa	39266777	39282236	KCNK17	0	0
chr6.fa	39282474	39290330	KCNK16	0	0
chr6.fa	39302876	39693181	KIF6	0.28810999	76
chr6.fa	39760793	39872033	DAAM2	0.408293875	152
chr6.fa	39872642	39902290	MOCS1	6.086154104	1435
chr6.fa	40346163	40347631	TDRG1	0	0
chr6.fa	40359373	40555126	LRFN2	0	0
chr6.fa	40994640	41006938	UNC5CL	0.731796362	152
chr6.fa	41010237	41012076	TSPO2	0	0
chr6.fa	41020940	41032630	APOBEC2	0	0
chr6.fa	41034531	41040188	C6orf130	29.24896232	2280
chr6.fa	41040707	41070146	NFYA	12.27261205	4864

chr6.fa	41072849	41108573	LOC221442	0.265067215	76
chr6.fa	41117342	41122070	TREML1	0	0
chr6.fa	41126246	41130922	TREM2	0	0
chr6.fa	41157552	41168925	TREML2	0.304526084	76
chr6.fa	41176292	41185685	TREML3	0	0
chr6.fa	41196062	41206120	TREML4	0	0
chr6.fa	41217115	41217327	TREML2P1	0	0
chr6.fa	41243712	41254457	TREM1	0	0
chr6.fa	41303528	41318625	NCR2	0	0
chr6.fa	41514164	41570122	FOXP4	14.81499822	5852
chr6.fa	41606195	41621982	MDFI	4.247099694	456
chr6.fa	41651716	41703997	TFEB	3.246470958	532
chr6.fa	41704449	41715139	PGC	2.525367648	304
chr6.fa	41737914	41747630	FRS3	1.05695552	152
chr6.fa	41748500	41755110	PRICKLE4	1.390849847	152
chr6.fa	41755181	41757634	TOMM6	101.862168	5776
chr6.fa	41765383	41863099	USP49	0.360250442	76
chr6.fa	41873092	41888877	MED20	9.70010461	1596
chr6.fa	41888965	41900784	BYSL	4.026310752	532
chr6.fa	41902671	42016610	CCND3	19.84058225	3192
chr6.fa	42018251	42048644	TAF8	9.002194681	2584
chr6.fa	42068857	42110715	C6orf132	29.54053625	13224
chr6.fa	42123144	42147794	GUCA1A	0	0
chr6.fa	42151022	42162694	GUCA1B	0	0
chr6.fa	42174539	42185633	MRPS10	13.37158675	1900
chr6.fa	42192669	42419783	TRERF1	13.43935962	6764
chr6.fa	42531760	42661243	UBR2	13.76000058	8114
chr6.fa	42664333	42690358	PRPH2	0	0
chr6.fa	42695314	42695932	LOC442211	0	0
chr6.fa	42712234	42713884	TBCC	9.705978258	1064
chr6.fa	42788794	42836296	KIAA0240	14.40640313	6232
chr6.fa	42847671	42854731	RPL7L1	33.21608477	2812
chr6.fa	42858003	42858554	C6orf226	14.51499033	532
chr6.fa	42883727	42893575	PTCRA	0	0
chr6.fa	42896860	42907008	CNPY3	9.079003931	1064
chr6.fa	42928500	42931610	GNMT	51.83133357	3648
chr6.fa	42931619	42946981	PEX6	14.43065076	3344
chr6.fa	42952330	42979966	PPP2R5D	18.52608984	3400
chr6.fa	42980081	42981618	MEA1	66.97179216	3344
chr6.fa	42981977	42989031	KLHDC3	67.04302898	8360
chr6.fa	42989385	42997337	RRP36	26.61274838	2048
chr6.fa	43005355	43021683	CUL7	16.97680209	6308
chr6.fa	43021767	43027242	MRPL2	6.230435009	532

chr6.fa	43027372	43042833	KLC4	8.369949131	1595
chr6.fa	43044029	43129457	PTK7	0.538718993	152
chr6.fa	43138920	43149244	SRF	6.626981586	1900
chr6.fa	43149922	43192325	CUL9	6.186307342	3192
chr6.fa	43193367	43197211	C6orf108	42.52416099	1824
chr6.fa	43211222	43255997	TTBK1	0.481789784	228
chr6.fa	43265998	43273210	SLC22A7	2.754741153	456
chr6.fa	43273277	43276530	CRIP3	0	0
chr6.fa	43303808	43337181	ZNF318	10.00779343	5320
chr6.fa	43395292	43418089	ABCC10	3.185324771	1140
chr6.fa	43418164	43423786	DLK2	0	0
chr6.fa	43445261	43474294	TJAP1	8.382750673	1716
chr6.fa	43474707	43478424	C6orf154	1.079245263	152
chr6.fa	43479565	43484728	YIPF3	21.59710437	2260
chr6.fa	43484791	43497114	POLR1C	12.77111914	1216
chr6.fa	43490068	43543812	XPO5	13.65683522	4864
chr6.fa	43543878	43588217	POLH	2.735312931	1520
chr6.fa	43588261	43596936	GTPBP2	14.4246265	2812
chr6.fa	43597279	43608688	MAD2L1BP	9.837005802	1064
chr6.fa	43612767	43638748	RSPH9	1.080901933	76
chr6.fa	43638934	43655549	MRPS18A	24.06659705	1900
chr6.fa	43737946	43754223	VEGFA	16.73357279	3991
chr6.fa	43858765	43905944	LOC100132354	0	0
chr6.fa	43968337	43973694	C6orf223	0.315972169	76
chr6.fa	44081373	44095191	MRPL14	39.92815895	2052
chr6.fa	44095376	44123256	TMEM63B	7.156061772	1520
chr6.fa	44126548	44152139	CAPN11	0	0
chr6.fa	44187242	44201888	SLC29A1	2.022342365	380
chr6.fa	44214849	44221614	HSP90AB1	513.6757137	86564
chr6.fa	44221838	44225283	SLC35B2	1.683176815	228
chr6.fa	44225903	44233525	NFKBIE	3.991219468	684
chr6.fa	44238480	44247182	TMEM151B	0	0
chr6.fa	44247897	44265458	TCTE1	0	0
chr6.fa	44266463	44281063	AARS2	3.256561585	1034
chr6.fa	44310397	44344904	SPATS1	0	0
chr6.fa	44355302	44414780	CDC5L	24.79989948	4940
chr6.fa	44796470	45345575	SUPT3H	7.98680652	1216
chr6.fa	45165411	45165507	MIR586	0	0
chr6.fa	45296054	45518819	RUNX2	3.259874925	1292
chr6.fa	45866190	46048085	CLIC5	3.241952766	1368
chr6.fa	46097701	46114436	ENPP4	0.489169497	152
chr6.fa	46127762	46138717	ENPP5	0.777881912	152
chr6.fa	46188469	46293531	RCAN2	3.794678152	836

chr6.fa	46517445	46620523	CYP39A1	7.556825328	1140
chr6.fa	46620679	46645927	SLC25A27	1.160422097	228
chr6.fa	46655612	46672052	TDRD6	0.127563597	76
chr6.fa	46672057	46703430	PLA2G7	4.048600496	532
chr6.fa	46714699	46726942	LOC100287718	0	0
chr6.fa	46761094	46807519	MEP1A	0	0
chr6.fa	46820242	46922675	GPR116	1.14039145	456
chr6.fa	46967813	47010082	GPR110	5.869130322	1438
chr6.fa	47199268	47277680	TNFRSF21	28.03251465	6764
chr6.fa	47445525	47594996	CD2AP	50.5556976	18157
chr6.fa	47624326	47665533	GPR111	0	0
chr6.fa	47666289	47689757	GPR115	1.447929662	304
chr6.fa	47749775	47794116	OPN5	0	0
chr6.fa	47845764	48036425	C6orf138	0	0
chr6.fa	49398073	49431041	MUT	17.37831867	4484
chr6.fa	49431096	49460820	CENPQ	0.662366825	76
chr6.fa	49467671	49495777	GLYATL3	0	0
chr6.fa	49518113	49519808	C6orf141	0	0
chr6.fa	49572890	49604587	RHAG	0	0
chr6.fa	49660072	49681299	CRISP2	0.812973197	76
chr6.fa	49695092	49712150	CRISP3	1.541155006	228
chr6.fa	49753364	49755053	PGK2	0	0
chr6.fa	49801979	49834218	CRISP1	0	0
chr6.fa	49913814	49917157	DEFB133	0	0
chr6.fa	49928005	49931818	DEFB114	0	0
chr6.fa	49936390	49937338	DEFB113	0	0
chr6.fa	49976851	49989694	DEFB110	0	0
chr6.fa	50011288	50016364	DEFB112	0	0
chr6.fa	50681257	50740746	TFAP2D	0	0
chr6.fa	50786439	50815326	TFAP2B	1.190242159	456
chr6.fa	51480145	51952423	PKHD1	8.51362761	9840
chr6.fa	52009147	52009232	MIR206	0	0
chr6.fa	52013721	52013839	MIR133B	0	0
chr6.fa	52051185	52055436	IL17A	0	0
chr6.fa	52101484	52109298	IL17F	0	0
chr6.fa	52128812	52149582	MCM3	32.37720727	6688
chr6.fa	52226926	52272575	PAQR8	6.039466129	1900
chr6.fa	52284994	52360583	EFHC1	1.954117678	912
chr6.fa	52362200	52441862	TRAM2	1.459827565	684
chr6.fa	52529199	52533951	LOC730101	1.610283331	380
chr6.fa	52535884	52551385	TMEM14A	12.41689296	836
chr6.fa	52604261	52609957	GSTA7P	0	0
chr6.fa	52614885	52628361	GSTA2	22.7347849	1976

chr6.fa	52656178	52668664	GSTA1	43.63879875	3648
chr6.fa	52696541	52710893	GSTA5	0	0
chr6.fa	52761439	52774496	GSTA3	1.253647442	76
chr6.fa	52842746	52860178	GSTA4	16.21744476	1444
chr6.fa	52866098	52926600	ICK	12.66072467	5244
chr6.fa	52929796	52965670	FBXO9	23.79716225	7872
chr6.fa	52991760	53013624	GCM1	0.414167523	76
chr6.fa	53132196	53213942	ELOVL5	25.09252766	4940
chr6.fa	53362140	53409927	GCLC	38.99876703	9871
chr6.fa	53512699	53530506	KLHL31	0	0
chr6.fa	53659778	53788919	LRRC1	13.27986747	2804
chr6.fa	53883714	54131078	C6orf142	0	0
chr6.fa	54173203	54254950	TINAG	12.05513645	1444
chr6.fa	54711569	54806819	FAM83B	10.4844626	2204
chr6.fa	55039071	55147418	HCRTR2	0	0
chr6.fa	55192267	55267291	GFRAL	0	0
chr6.fa	55299171	55444012	HMGCLL1	0	0
chr6.fa	55620238	55740375	BMP5	0	0
chr6.fa	55921388	56112378	COL21A1	0	0
chr6.fa	56322785	56507694	DST	5.616412829	8284
chr6.fa	56819773	56892142	BEND6	0.848516301	152
chr6.fa	56911384	56920023	KIAA1586	3.952363024	760
chr6.fa	56954828	57035098	ZNF451	14.19570482	4940
chr6.fa	57037104	57050012	BAG2	6.693549603	912
chr6.fa	57053582	57087078	RAB23	18.88317754	3952
chr6.fa	57182422	57513376	PRIM2	57.13403333	8657
chr6.fa	58246159	58287724	GUSBP4	4.459454679	228
chr6.fa	62284008	62284534	MTRNR2L9	0	0
chr6.fa	62389865	62996100	KHDRBS2	0	0
chr6.fa	63985856	64029882	LGSN	0	0
chr6.fa	64281920	64293489	PTP4A1	29.05437889	9804
chr6.fa	64356431	64424405	PHF3	21.74876499	10032
chr6.fa	64429876	66417118	EYS	0	0
chr6.fa	66497772	66499376	MCART3P	0	0
chr6.fa	69345632	70099403	BAI3	0	0
chr6.fa	70385641	70507049	LMBRD1	10.41458124	1596
chr6.fa	70576448	70922157	COL19A1	0	0
chr6.fa	70925743	71012786	COL9A1	0.285399075	76
chr6.fa	71123107	71270877	FAM135A	23.37606684	9929
chr6.fa	71276625	71298606	C6orf57	10.90103982	380
chr6.fa	71377479	71571716	SMAP1	16.77227863	3116
chr6.fa	71603831	71666788	B3GAT2	0	0
chr6.fa	71998477	72011973	OGFRL1	3.289092561	380

chr6.fa	72086663	72086734	MIR30C2	0	0
chr6.fa	72113254	72113324	MIR30A	0	0
chr6.fa	72124149	72130448	C6orf155	1.170813937	228
chr6.fa	72596650	73112845	RIMS1	6.232694104	3648
chr6.fa	73331571	73908573	KCNQ5	0	0
chr6.fa	73677410	73677476	MIR4282	0	0
chr6.fa	73933268	73935175	KHDC1L	0	0
chr6.fa	73951038	73972907	KHDC1	0	0
chr6.fa	73983862	74020088	C6orf147	0	0
chr6.fa	74062785	74063999	DPPA5	0	0
chr6.fa	74072400	74073898	C6orf221	0	0
chr6.fa	74078280	74079515	OOEP	0	0
chr6.fa	74104285	74127289	DDX43	0.424559363	76
chr6.fa	74134856	74162043	C6orf150	1.270364749	152
chr6.fa	74171454	74211179	MTO1	11.74338126	2432
chr6.fa	74225473	74230755	EEF1A1	798.2824488	186152
chr6.fa	74303102	74363737	SLC17A5	3.799196343	836
chr6.fa	74405508	74538041	CD109	0.243530504	152
chr6.fa	75794042	75915623	COL12A1	1.285124173	988
chr6.fa	75947391	75953644	COX7A2	110.8175241	5548
chr6.fa	75962638	75994632	TMEM30A	7.053047013	2128
chr6.fa	76017800	76203496	FILIP1	1.485882467	456
chr6.fa	76311622	76427994	SENP6	21.5388197	9499
chr6.fa	76458909	76629254	MYO6	33.71519428	19391
chr6.fa	76631062	76782335	IMPG1	0	0
chr6.fa	78171948	78173120	HTR1B	0	0
chr6.fa	79577189	79608320	IRAK1BP1	4.946515686	380
chr6.fa	79644136	79788011	PHIP	8.564833777	6805
chr6.fa	79910962	79944455	HMGN3	36.25351408	2217
chr6.fa	80194708	80247147	LCA5	4.608554987	1444
chr6.fa	80341000	80413369	SH3BGRL2	57.64925773	17784
chr6.fa	80451636	80451669	RNY4	0	0
chr6.fa	80624529	80657315	ELOVL4	0	0
chr6.fa	80714322	80752244	TTK	1.136325078	228
chr6.fa	80816344	81055987	BCKDHB	28.79985412	2964
chr6.fa	82455447	82462428	FAM46A	5.506921997	2052
chr6.fa	82879956	82957448	IBTK	19.02730784	7296
chr6.fa	83072923	83077133	TPBG	0.329225529	76
chr6.fa	83602186	83775545	UBE2CBP	2.497806682	304
chr6.fa	83777385	83874438	DOPEY1	9.562299779	4706
chr6.fa	83874593	83903655	PGM3	32.04346355	12236
chr6.fa	83903032	83906256	RWDD2A	31.54751677	2964
chr6.fa	83920110	84140938	ME1	3.579913465	836

chr6.fa	84222194	84235421	PRSS35	0.439168181	76
chr6.fa	84262605	84419127	SNAP91	1.694622899	532
chr6.fa	84562985	84567234	RIPPLY2	0	0
chr6.fa	84569370	84670146	CYB5R4	4.584608574	684
chr6.fa	84743420	84800605	MRAP2	0.517182282	76
chr6.fa	84833960	84937335	KIAA1009	5.243963271	1789
chr6.fa	85444157	85473899	TBX18	0	0
chr6.fa	86159302	86205498	NT5E	0.846407811	228
chr6.fa	86215215	86303629	SNX14	7.972950734	1855
chr6.fa	86317502	86353043	SYNCRIP	20.60129504	11400
chr6.fa	86386725	86388451	SNHG5	6.772768555	228
chr6.fa	86387012	86387086	SNORD50A	0	0
chr6.fa	86387307	86387377	SNORD50B	0	0
chr6.fa	87647024	87726397	HTR1E	0	0
chr6.fa	87795222	87804824	CGA	0	0
chr6.fa	87865269	87973406	ZNF292	15.07253512	10108
chr6.fa	87992697	88038996	GJB7	0	0
chr6.fa	88032306	88052043	C6orf162	2.821610382	456
chr6.fa	88054571	88075181	C6orf163	0	0
chr6.fa	88106842	88109459	C6orf164	0	0
chr6.fa	88117690	88174191	C6orf165	0.516730463	76
chr6.fa	88182643	88222057	SLC35A1	6.605746088	836
chr6.fa	88224096	88299735	RARS2	24.31735666	2958
chr6.fa	88299785	88377172	ORC3	9.327956264	1593
chr6.fa	88384578	88411985	AKIRIN2	12.4414418	1596
chr6.fa	88410020	88410933	NCRNA00120	3.756875953	228
chr6.fa	88757507	88776550	SPACA1	0	0
chr6.fa	88849585	88875767	CNR1	0	0
chr6.fa	89319989	89673348	RNGTT	7.950510385	2356
chr6.fa	89790429	89794879	PNRC1	72.44362288	10260
chr6.fa	89805678	89827800	SRSF12	1.272172025	304
chr6.fa	89855769	89875284	PM20D2	14.34405209	4484
chr6.fa	89887223	89927496	GABRR1	0	0
chr6.fa	89967239	90024967	GABRR2	0	0
chr6.fa	90036344	90062619	UBE2J1	50.87739281	14668
chr6.fa	90074335	90121995	RRAGD	13.70924624	4484
chr6.fa	90142897	90341942	ANKRD6	4.604187402	1140
chr6.fa	90343404	90348474	LYRM2	17.70724299	4864
chr6.fa	90353231	90529442	MDN1	4.933714145	5700
chr6.fa	90539619	90584155	CASP8AP2	6.542039593	2863
chr6.fa	90604188	90605819	GJA10	0	0
chr6.fa	90636247	91006562	BACH2	0	0
chr6.fa	91225353	91296907	MAP3K7	20.09766733	3994

chr6.fa	93949740	94129300	EPHA7	0.17229369	76
chr6.fa	94416801	94486199	TSG1	0	0
chr6.fa	96025413	96057326	MANEA	1.501394924	456
chr6.fa	96463845	96663488	FUT9	0.894300638	760
chr6.fa	96969702	97003151	KIAA0776	10.61594196	2964
chr6.fa	97010424	97064512	FHL5	0	0
chr6.fa	97245888	97285353	GPR63	0.520796835	76
chr6.fa	97337187	97345767	NDUFAF4	6.171698523	988
chr6.fa	97372496	97588630	KLHL32	0.300610319	76
chr6.fa	97537843	97862283	MIR548H3	0	0
chr6.fa	97590037	97731052	MMS22L	0.264916609	152
chr6.fa	98472407	98472495	MIR2113	0	0
chr6.fa	99282580	99286666	POU3F2	0.280127852	76
chr6.fa	99321601	99395849	FBXL4	9.4800687	1748
chr6.fa	99720793	99797531	C6orf168	0.703632971	456
chr6.fa	99817348	99842082	COQ3	2.714529251	228
chr6.fa	99847841	99873207	SFRS18	11.01610309	2356
chr6.fa	99880184	99963252	USP45	2.688624955	1140
chr6.fa	99968870	99981059	LOC100130890	3.479760228	445
chr6.fa	99990263	100016690	CCNC	19.47370513	2983
chr6.fa	100054650	100063454	PRDM13	0	0
chr6.fa	100367786	100442114	MCHR2	0	0
chr6.fa	100836750	100911551	SIM1	1.719021131	456
chr6.fa	100956608	101329224	ASCC3	11.34578044	7904
chr6.fa	101846861	102517958	GRIK2	0	0
chr6.fa	105175968	105307794	HACE1	5.502855625	1672
chr6.fa	105404923	105531206	LIN28B	0	0
chr6.fa	105544699	105585049	BVES	0.593389106	228
chr6.fa	105585562	105617819	C6orf112	0	0
chr6.fa	105605775	105627858	POPDC3	2.99480771	380
chr6.fa	105725442	105850999	PREP	10.5010293	2052
chr6.fa	106534195	106557811	PRDM1	0.215216506	76
chr6.fa	106632352	106773695	ATG5	17.64187982	3800
chr6.fa	106959730	107018334	AIM1	91.48252741	45873
chr6.fa	107018903	107077373	RTN4IP1	5.644877434	988
chr6.fa	107077441	107116292	QRSL1	4.1692362	1140
chr6.fa	107165327	107235300	LOC100422737	2.475667545	228
chr6.fa	107349407	107372547	C6orf203	6.909067321	456
chr6.fa	107386385	107435636	BEND3	5.499692891	2432
chr6.fa	107473761	107780779	PDSS2	13.21194399	3116
chr6.fa	107811317	107982513	SOBP	3.489700249	1444
chr6.fa	108023364	108145521	SCML4	0	0
chr6.fa	108188960	108279482	SEC63	6.103022018	2634

chr6.fa	108362613	108395941	OSTM1	0.512513484	152
chr6.fa	108487215	108510013	NR2E1	0	0
chr6.fa	108532717	108582464	SNX3	68.80165959	6688
chr6.fa	108616098	108844251	LACE1	2.030324503	304
chr6.fa	108881026	109005971	FOXO3	16.55992365	8284
chr6.fa	109072857	109091145	NCRNA00222	0	0
chr6.fa	109169619	109295352	ARMC2	0.670800781	152
chr6.fa	109307640	109415708	SESN1	21.75102408	5244
chr6.fa	109416356	109485115	CEP57L1	0.740682138	152
chr6.fa	109615506	109629423	CCDC162	0	0
chr6.fa	109687717	109703762	CD164	9.848451886	2035
chr6.fa	109711418	109761930	PPIL6	1.521274965	380
chr6.fa	109762560	109765122	SMPD2	18.52111983	1520
chr6.fa	109765266	109777190	MICAL1	10.05146928	2432
chr6.fa	109783719	109804440	ZBTB24	7.200038832	2736
chr6.fa	109814059	110012415	AKD1	2.558651656	1283
chr6.fa	110012424	110146634	FIG4	17.16566248	3539
chr6.fa	110300298	110301924	GPR6	0	0
chr6.fa	110421022	110501207	WASF1	0.355431038	76
chr6.fa	110501624	110553422	CDC40	10.6972694	2736
chr6.fa	110567149	110679475	C6orf186	1.039635787	76
chr6.fa	110713383	110736753	DDO	2.009239611	228
chr6.fa	110745907	110797844	SLC22A16	0	0
chr6.fa	110931181	111136412	CDK19	28.0383883	11429
chr6.fa	111195987	111216913	AMD1	477.9958593	108576
chr6.fa	111279763	111289091	GTF3C6	96.70510458	6100
chr6.fa	111303291	111346794	RPF2	15.6796294	988
chr6.fa	111367622	111368757	GSTM2P1	0	0
chr6.fa	111408781	111544606	SLC16A10	0.395944152	76
chr6.fa	111580482	111590261	KIAA1919	1.168554841	304
chr6.fa	111620234	111804414	REV3L	7.915268494	5624
chr6.fa	111804675	111923497	LOC643749	0.981049908	456
chr6.fa	111880143	111927474	TRAF3IP2	14.76921388	2888
chr6.fa	111982485	112194627	FYN	0	0
chr6.fa	112375278	112390887	WISP3	0	0
chr6.fa	112391860	112408673	TUBE1	4.642441421	684
chr6.fa	112408752	112423993	C6orf225	3.089990937	532
chr6.fa	112429134	112575828	LAMA4	0.15316668	76
chr6.fa	112668532	112672498	RFPL4B	0	0
chr6.fa	114178527	114184652	MARCKS	38.94500056	11096
chr6.fa	114225551	114242806	FLJ34503	0	0
chr6.fa	114257320	114292359	HDAC2	9.418470694	4180
chr6.fa	114376750	114384041	HS3ST5	2.502776692	456

chr6.fa	116262693	116381921	FRK	16.87378733	3192
chr6.fa	116359894	116361107	TPI1P3	0	0
chr6.fa	116421999	116566853	NT5DC1	19.02188601	3952
chr6.fa	116440085	116447296	COL10A1	0	0
chr6.fa	116571131	116575261	TSPYL4	18.28707752	5016
chr6.fa	116596022	116601280	TSPYL1	52.23541047	18240
chr6.fa	116601283	116759442	DSE	1.095360144	304
chr6.fa	116782556	116784934	FAM26F	1.032105468	76
chr6.fa	116817651	116866773	BET3L	0	0
chr6.fa	116832808	116839709	FAM26E	0	0
chr6.fa	116850195	116880031	FAM26D	0	0
chr6.fa	116892583	116914436	RWDD1	34.10210205	2964
chr6.fa	116937642	116954148	RSPH4A	0	0
chr6.fa	116956781	116989973	ZUFSP	6.729996345	988
chr6.fa	117002367	117063030	KPNA5	5.304055214	760
chr6.fa	117073360	117086886	FAM162B	0	0
chr6.fa	117113248	117150198	GPRC6A	0	0
chr6.fa	117198376	117253326	RFX6	0	0
chr6.fa	117586721	117594725	VGLL2	0	0
chr6.fa	117609530	117747018	ROS1	0	0
chr6.fa	117803820	117891020	DCBLD1	1.230152848	152
chr6.fa	117881433	117923705	GOPC	13.67144404	4094
chr6.fa	117996617	118031886	NUS1	6.905302162	2204
chr6.fa	118228689	118638839	SLC35F1	0	0
chr6.fa	118781935	119031238	C6orf204	1.423230217	760
chr6.fa	118822536	118824996	BRD7P3	0	0
chr6.fa	118869442	118881587	PLN	0	0
chr6.fa	119103871	119104581	LOC100287632	0	0
chr6.fa	119134612	119256327	MCM9	6.703489623	2736
chr6.fa	119215241	119230335	ASF1A	14.14781199	2356
chr6.fa	119280996	119470358	FAM184A	1.100781974	304
chr6.fa	119390212	119390308	MIR548B	0	0
chr6.fa	119499267	119670926	MAN1A1	5.277247279	1444
chr6.fa	121400627	121655644	C6orf170	2.453377802	836
chr6.fa	121756745	121770873	GJA1	0.365672272	76
chr6.fa	122720696	122754264	HSF2	4.24393696	760
chr6.fa	122764493	122792952	SERINC1	11.34367195	2356
chr6.fa	122793062	123047518	PKIB	4.200411719	608
chr6.fa	123100646	123105218	FABP7	0	0
chr6.fa	123109971	123130864	SMPDL3A	3.651903311	532
chr6.fa	123317582	123385063	CLVS2	0	0
chr6.fa	123537483	123957942	TRDN	0	0
chr6.fa	124125069	125146786	NKAIN2	0	0

chr6.fa	125229392	125284173	STL	0	0
chr6.fa	125304514	125404661	RNF217	0	0
chr6.fa	125474879	125584644	TPD52L1	18.41674961	1992
chr6.fa	125596496	125623282	HDDC2	26.2232803	2812
chr6.fa	126070732	126082415	HEY2	1.285124173	228
chr6.fa	126102307	126253176	NCOA7	8.417390138	3952
chr6.fa	126277861	126301389	HINT3	10.83974303	2432
chr6.fa	126307576	126360420	TRMT11	3.520122736	456
chr6.fa	126661253	126669754	CENPW	0	0
chr6.fa	127440048	127518184	RSPO3	0	0
chr6.fa	127588020	127609504	RNF146	17.09969689	2432
chr6.fa	127609857	127664754	ECHDC1	18.19114126	2888
chr6.fa	127780320	127780535	KIAA0408	0	0
chr6.fa	127794225	127840500	C6orf174	0	0
chr6.fa	127898319	127912960	C6orf58	0	0
chr6.fa	128029339	128239776	THEMIS	0	0
chr6.fa	128289924	128841819	PTPRK	4.312161647	1748
chr6.fa	129204286	129837710	LAMA2	0.236150791	152
chr6.fa	129898240	130031370	ARHGAP18	21.25447487	4938
chr6.fa	130152389	130182416	C6orf191	0	0
chr6.fa	130339728	130462594	L3MBTL3	4.616386518	1292
chr6.fa	130465461	130544099	SAMD3	0	0
chr6.fa	130758262	130764210	TMEM200A	0.228018047	76
chr6.fa	131148545	131158276	LOC285733	0.455283063	76
chr6.fa	131160488	131384462	EPB41L2	0.422450874	131
chr6.fa	131466461	131604673	AKAP7	4.4008688	912
chr6.fa	131894365	131905468	ARG1	0	0
chr6.fa	131895106	131949363	MED23	9.238947898	3420
chr6.fa	131958442	132068550	ENPP3	1.084968305	228
chr6.fa	132021609	132022541	OR2A4	0	0
chr6.fa	132029581	132032157	CTAGE9	0	0
chr6.fa	132129156	132216295	ENPP1	0.461457924	228
chr6.fa	132269317	132272518	CTGF	0.976983536	152
chr6.fa	132617194	132722664	MOXD1	0	0
chr6.fa	132778663	132834337	STX7	14.6655967	4158
chr6.fa	132859427	132860475	TAAR9	0	0
chr6.fa	132873832	132874860	TAAR8	0	0
chr6.fa	132891461	132892498	TAAR6	0	0
chr6.fa	132909731	132910877	TAAR5	0	0
chr6.fa	132929364	132930441	TAAR3	0	0
chr6.fa	132938289	132945414	TAAR2	0	0
chr6.fa	132966123	132967142	TAAR1	0	0
chr6.fa	133001997	133035194	VNN1	0	0

chr6.fa	133043926	133055904	VNN3	0	0
chr6.fa	133065009	133084598	VNN2	0	0
chr6.fa	133090507	133119747	C6orf192	0.47200037	76
chr6.fa	133135708	133138703	RPS12	1408.693539	47048
chr6.fa	133136446	133136518	SNORD101	0	0
chr6.fa	133137941	133138016	SNORD100	0	0
chr6.fa	133138358	133138490	SNORA33	0	0
chr6.fa	133409219	133427717	LOC285735	0	0
chr6.fa	133562495	133853258	EYA4	1.975503783	760
chr6.fa	134142285	134175130	MGC34034	0	0
chr6.fa	134210259	134216675	TCF21	0	0
chr6.fa	134274301	134308629	TBPL1	16.27091002	1425
chr6.fa	134308719	134373789	SLC2A12	3.475392643	1292
chr6.fa	134490384	134639196	SGK1	4.330234411	1140
chr6.fa	135238528	135271260	ALDH8A1	6.71192358	1140
chr6.fa	135281517	135376036	HBS1L	5.383876591	3336
chr6.fa	135300476	135300570	MIR3662	0	0
chr6.fa	135502453	135540310	MYB	1.556516856	380
chr6.fa	135605110	135818903	AHI1	7.14988691	2800
chr6.fa	135818939	136011976	NCRNA00271	0	0
chr6.fa	136172834	136516709	PDE7B	3.173125654	1135
chr6.fa	136552168	136571449	FAM54A	0	0
chr6.fa	136578001	136610989	BCLAF1	29.15498394	14060
chr6.fa	136663419	136871957	MAP7	36.67400707	13281
chr6.fa	136878187	137113656	MAP3K5	40.38103231	13929
chr6.fa	137143702	137235072	PEX7	24.38964772	2400
chr6.fa	137243402	137246776	SLC35D3	0	0
chr6.fa	137303296	137314368	NHEG1	0	0
chr6.fa	137321108	137366298	IL20RA	8.393443725	2052
chr6.fa	137464957	137494785	IL22RA2	0	0
chr6.fa	137518621	137540567	IFNGR1	4.85916399	684
chr6.fa	137813336	137815531	OLIG3	0	0
chr6.fa	138188581	138204449	TNFAIP3	11.87998124	3496
chr6.fa	138409642	138428660	PERP	13.56933292	3876
chr6.fa	138483053	138665800	KIAA1244	46.36191256	45812
chr6.fa	138537127	138539627	PBOV1	0	0
chr6.fa	138725336	138734582	HEBP2	132.1388682	11248
chr6.fa	138743181	138893668	NHSL1	13.00696872	6764
chr6.fa	138756350	138756431	MIR3145	0	0
chr6.fa	139012805	139018425	FLJ46906	0	0
chr6.fa	139094657	139114456	CCDC28A	19.81241886	1968
chr6.fa	139117248	139225207	ECT2L	0.255277801	76
chr6.fa	139225620	139309398	REPS1	18.9837826	4476

chr6.fa	139349819	139364439	C6orf115	18.96390256	1064
chr6.fa	139456249	139501946	HECA	10.79245263	4028
chr6.fa	139561199	139613208	TXLNB	0.244434142	76
chr6.fa	139693397	139695785	CITED2	35.26041566	4940
chr6.fa	139790132	139795733	LOC645434	1.354553711	152
chr6.fa	140526389	140526463	MIR3668	0	0
chr6.fa	142396745	142409936	NMBR	0	0
chr6.fa	142468410	142542085	VTA1	11.99655057	2584
chr6.fa	142623056	142767403	GPR126	3.100382777	1433
chr6.fa	142847592	142959026	LOC153910	0	0
chr6.fa	143072604	143266338	HIVEP2	23.50167255	15174
chr6.fa	143382023	143661441	AIG1	9.917128392	912
chr6.fa	143743969	143771841	ADAT2	1.098221666	456
chr6.fa	143771918	143811751	PEX3	7.383628	1359
chr6.fa	143815949	143833020	FUCA2	7.187538503	1216
chr6.fa	143875467	143890476	LOC285740	0	0
chr6.fa	143929317	144152322	PHACTR2	20.11212554	13055
chr6.fa	144164508	144184943	LTV1	12.61975974	1520
chr6.fa	144185573	144259483	C6orf94	0	0
chr6.fa	144261437	144385735	PLAGL1	3.141648923	684
chr6.fa	144324034	144329867	HYMAI	0	0
chr6.fa	144416018	144416754	SF3B5	69.88783274	3420
chr6.fa	144471654	144513076	STX11	2.079572786	760
chr6.fa	144612873	145174170	UTRN	16.81971964	13883
chr6.fa	145946440	146056991	EPM2A	0.660710155	152
chr6.fa	146119272	146135921	FBXO30	5.194564381	1520
chr6.fa	146205945	146285233	SHPRH	8.832310693	4104
chr6.fa	146348782	146758731	GRM1	0.329978561	152
chr6.fa	146864828	146876086	RAB32	7.611646047	608
chr6.fa	146920136	147136597	C6orf103	0.224252888	76
chr6.fa	147122805	147124960	LOC729176	0	0
chr6.fa	147162525	147495084	LOC729178	0.398805673	76
chr6.fa	147525508	147708707	STXBP5	17.71944211	7331
chr6.fa	147829828	147891157	SAMD5	0.542935972	228
chr6.fa	148663729	148873184	SASH1	26.04993237	13334
chr6.fa	149068271	149398126	UST	1.721732046	502
chr6.fa	149639436	149732747	TAB2	50.52422087	14744
chr6.fa	149721495	149722182	SUMO4	0	0
chr6.fa	149768766	149806148	ZC3H12D	0.699717205	228
chr6.fa	149825631	149867171	PPIL4	10.1583998	1664
chr6.fa	149887528	149912067	C6orf72	3.096316405	228
chr6.fa	149916172	149959728	KATNA1	9.030508679	912
chr6.fa	149982051	150039392	LATS1	25.75670176	8132

chr6.fa	150045457	150067688	NUP43	13.38544254	3420
chr6.fa	150070831	150132557	PCMT1	19.40337195	2234
chr6.fa	150139894	150185480	LRP11	8.858214989	2128
chr6.fa	150209601	150212097	RAET1E	1.293407524	76
chr6.fa	150238014	150244214	RAET1G	0	0
chr6.fa	150263136	150270368	ULBP2	0	0
chr6.fa	150285143	150294846	ULBP1	0	0
chr6.fa	150319155	150326280	RAET1K	0	0
chr6.fa	150341266	150346668	RAET1L	0	0
chr6.fa	150385743	150390202	ULBP3	0	0
chr6.fa	150464188	150571528	PPP1R14C	0	0
chr6.fa	150690028	150725765	IYD	2.417684092	1216
chr6.fa	150920999	151164799	PLEKHG1	14.43215682	6935
chr6.fa	151186691	151423023	MTHFD1L	2.863177741	684
chr6.fa	151561134	151679694	AKAP12	63.55317812	37088
chr6.fa	151685250	151712677	ZBTB2	7.047173365	1444
chr6.fa	151725989	151773316	RMND1	13.72867446	1742
chr6.fa	151773422	151791232	C6orf211	12.46071941	2128
chr6.fa	151815175	151942328	C6orf97	0.650017102	228
chr6.fa	152011631	152424408	ESR1	0.657095602	304
chr6.fa	152442822	152958534	SYNE1	3.946790588	7353
chr6.fa	153019030	153045715	MYCT1	0	0
chr6.fa	153071933	153080902	VIP	0	0
chr6.fa	153291658	153304740	FBXO5	2.321747833	380
chr6.fa	153308400	153323925	MTRF1L	5.017601894	1292
chr6.fa	153332032	153452389	RGS17	0.780743433	76
chr6.fa	154331636	154568001	OPRM1	0	0
chr6.fa	154475618	154677900	IPCEF1	1.164187257	532
chr6.fa	154726433	154831753	CNKSR3	8.057591515	1727
chr6.fa	155054512	155155194	RBM16	31.73441928	10544
chr6.fa	155411423	155575707	TIAM2	0	0
chr6.fa	155577264	155635617	TFB1M	5.862202429	608
chr6.fa	155585147	155597682	CLDN20	0	0
chr6.fa	155716502	155777037	NOX3	0	0
chr6.fa	157099064	157531913	ARID1B	25.53335251	16340
chr6.fa	157710054	157745253	C6orf35	1.593867236	532
chr6.fa	157802557	158094977	ZDHHC14	1.23211073	228
chr6.fa	157950164	157950232	MIR3692	0	0
chr6.fa	158244294	158366109	SNX9	28.88780824	8056
chr6.fa	158402888	158520207	SYNJ2	9.07945575	4560
chr6.fa	158530536	158589312	SERAC1	6.632855235	1748
chr6.fa	158589379	158620376	GTF2H5	5.043656796	2507
chr6.fa	158733692	158932856	TULP4	25.52958735	18848

chr6.fa	158957468	159056467	TMEM181	5.49186136	1968
chr6.fa	159057507	159065804	DYNLT1	118.6571882	5972
chr6.fa	159071046	159185901	SYTL3	10.48175168	1520
chr6.fa	159186773	159240456	EZR	221.2052176	45752
chr6.fa	159262158	159278664	OSTCL	0	0
chr6.fa	159309619	159331385	C6orf99	1.794023105	76
chr6.fa	159398266	159421198	RSPH3	15.77315596	2280
chr6.fa	159456027	159466184	TAGAP	0	0
chr6.fa	159590429	159693140	FNDC1	0	0
chr6.fa	160100149	160114353	SOD2	58.03646671	6840
chr6.fa	160148152	160177351	WTAP	25.48259816	3785
chr6.fa	160181291	160183364	LOC100129518	0.729989086	76
chr6.fa	160182989	160199312	ACAT2	85.25374907	5689
chr6.fa	160199530	160210735	TCP1	108.5596333	14820
chr6.fa	160201282	160201413	SNORA20	0	0
chr6.fa	160206626	160206765	SNORA29	0	0
chr6.fa	160211492	160219461	MRPL18	43.34752603	2812
chr6.fa	160221301	160241735	PNLDC1	0.596100021	76
chr6.fa	160327974	160329107	MAS1	0	0
chr6.fa	160390131	160527583	IGF2R	8.813334291	5320
chr6.fa	160514114	160517244	LOC729603	0	0
chr6.fa	160542863	160579750	SLC22A1	0	0
chr6.fa	160637794	160679963	SLC22A2	6.379234104	1064
chr6.fa	160769425	160876014	SLC22A3	2.849321954	1064
chr6.fa	160887587	160932156	LPAL2	0	0
chr6.fa	160952515	161087407	LPA	0	0
chr6.fa	161123225	161175085	PLG	6.472158236	1520
chr6.fa	161412822	161538417	MAP3K4	24.62866003	8968
chr6.fa	161551057	161695107	AGPAT4	0.145335149	76
chr6.fa	161581164	161583014	NCRNA00241	0	0
chr6.fa	161768590	163148834	PARK2	1.405157452	380
chr6.fa	163148164	163736524	PACRG	4.42662249	532
chr6.fa	163731017	163745505	LOC285796	0	0
chr6.fa	163759374	163768065	DKFZp451B082	0	0
chr6.fa	163834097	163834982	LOC100526820	0	0
chr6.fa	163835675	163999628	QKI	4.009593445	4104
chr6.fa	165693153	165723111	C6orf118	0	0
chr6.fa	165740778	166075584	PDE10A	23.49730497	12976
chr6.fa	166337536	166401038	C6orf176	0	0
chr6.fa	166401528	166403103	LOC441177	0	0
chr6.fa	166571086	166582131	T	0	0
chr6.fa	166719168	166721871	PRR18	0	0
chr6.fa	166733517	166755991	SFT2D1	13.27083109	608

chr6.fa	166778408	166796486	BRP44L	39.34591472	2508
chr6.fa	166822854	167275771	RPS6KA2	18.90983487	7600
chr6.fa	166922842	166922921	MIR1913	0	0
chr6.fa	167343004	167370077	RNASET2	2.698866189	224
chr6.fa	167411295	167411400	MIR3939	0	0
chr6.fa	167412816	167454066	FGFR1OP	9.108221567	1140
chr6.fa	167525295	167552629	CCR6	0	0
chr6.fa	167570360	167571319	GPR31	0	0
chr6.fa	167584081	167596396	TCP10L2	0	0
chr6.fa	167704803	167729502	UNC93A	0	0
chr6.fa	167738574	167756177	TTLL2	0	0
chr6.fa	167786577	167797998	TCP10	0	0
chr6.fa	168185219	168197539	C6orf123	1.3873859	152
chr6.fa	168224570	168227476	C6orf124	1.485882467	228
chr6.fa	168227671	168365793	MLLT4	43.03004779	21797
chr6.fa	168376604	168377619	HGC6-3	0	0
chr6.fa	168418553	168445769	KIF25	0	0
chr6.fa	168456464	168479839	FRMD1	0.364316814	76
chr6.fa	168707584	168720402	DACT2	3.112431286	608
chr6.fa	168841831	169068674	SMOC2	0.726675746	152
chr6.fa	169615875	169654137	THBS2	0.196993135	76
chr6.fa	169857307	170102159	WDR27	1.651248264	380
chr6.fa	170102257	170106402	C6orf120	4.372253589	1064
chr6.fa	170110332	170124106	PHF10	27.41743823	2170
chr6.fa	170140215	170151638	TCTE3	0	0
chr6.fa	170151721	170181617	C6orf70	12.66885742	1748
chr6.fa	170188886	170198921	NCRNA00242	0	0
chr6.fa	170195280	170202969	C6orf208	0	0
chr6.fa	170563422	170571657	LOC154449	0	0
chr6.fa	170591294	170599697	DLL1	0	0
chr6.fa	170615844	170714237	FAM120B	31.42326651	6758
chr6.fa	170844204	170862417	PSMB1	107.3460472	6536
chr6.fa	170863421	170881958	TBP	4.809313281	608
chr6.fa	170884660	170893780	PDCD2	12.62337429	3724
chr7.fa	192969	300711	FAM20C	5.408877249	988
chr7.fa	330136	331454	LOC100288524	0	0
chr7.fa	419391	422845	LOC442497	0	0
chr7.fa	536897	559481	PDGFA	7.747643601	1444
chr7.fa	560028	564869	FLJ44511	0	0
chr7.fa	588834	767313	PRKAR1B	7.926413365	1511
chr7.fa	766338	826116	HEATR2	12.11040899	2660
chr7.fa	855194	914557	SUN1	9.516214229	2935
chr7.fa	916191	936071	GET4	17.54187719	2432

chr7.fa	937537	994289	ADAP1	3.943477248	608
chr7.fa	1004486	1015235	COX19	4.680394227	1520
chr7.fa	1022835	1029276	CYP2W1	0	0
chr7.fa	1036623	1177893	C7orf50	30.09793044	2584
chr7.fa	1062569	1062662	MIR339	0	0
chr7.fa	1097141	1098897	GPR146	0.651523166	76
chr7.fa	1126443	1133451	GPER	1.856976568	380
chr7.fa	1192543	1199855	ZFAND2A	15.24452759	912
chr7.fa	1272654	1276613	UNCX	0	0
chr7.fa	1473995	1499109	MICALL2	1.477448511	304
chr7.fa	1509913	1544018	INTS1	12.80169224	5928
chr7.fa	1570368	1581870	MAFK	11.26144087	1900
chr7.fa	1582680	1596066	TMEM184A	7.327903642	2660
chr7.fa	1606970	1609668	PSMG3	14.71213407	760
chr7.fa	1609709	1629261	KIAA1908	1.714051121	661
chr7.fa	1654106	1656328	TFAMP1	0	0
chr7.fa	1748798	1787590	ELFN1	0	0
chr7.fa	1855428	2272583	MAD1L1	5.016547649	912
chr7.fa	2273926	2281833	FTSJ2	12.15393423	1292
chr7.fa	2281857	2290780	NUDT1	1.365849189	76
chr7.fa	2291405	2354099	SNX8	3.632173877	1140
chr7.fa	2394474	2420377	EIF3B	44.8691022	9179
chr7.fa	2443259	2474216	CHST12	0	0
chr7.fa	2552163	2568810	LFNG	0.36281075	76
chr7.fa	2577444	2595392	C7orf27	6.837981114	1368
chr7.fa	2598632	2654368	IQCE	5.987657537	2721
chr7.fa	2671603	2704436	TTYH3	3.323280207	1064
chr7.fa	2719163	2755069	AMZ1	0	0
chr7.fa	2767741	2883959	GNA12	19.84269074	5776
chr7.fa	2945710	3083509	CARD11	21.47375775	6218
chr7.fa	3341080	4308631	SDK1	0.318381871	152
chr7.fa	4721930	4811074	FOXK1	12.47803914	9272
chr7.fa	4815264	4831399	KIAA0415	7.525047383	1444
chr7.fa	4838740	4923335	RADIL	0.311905797	76
chr7.fa	4897369	4901625	PAPOLB	0	0
chr7.fa	4945620	4998844	MMD2	0	0
chr7.fa	5013616	5037800	RNF216L	6.123353878	988
chr7.fa	5085553	5109119	RBAK	6.234802593	2712
chr7.fa	5111738	5112854	LOC389458	0	0
chr7.fa	5160941	5184177	ZNF890P	0	0
chr7.fa	5229835	5273486	WIPI2	24.64176279	7726
chr7.fa	5322561	5343704	SLC29A4	1.187832457	228
chr7.fa	5346423	5463177	TNRC18	10.93718535	7676

chr7.fa	5515428	5553399	FBXL18	3.894680783	2128
chr7.fa	5535450	5535548	MIR589	0	0
chr7.fa	5566779	5570232	ACTB	716.3277323	86184
chr7.fa	5632454	5646286	FSCN1	0.412209641	76
chr7.fa	5659678	5821292	RNF216	10.41668973	3876
chr7.fa	5862791	5894066	ZNF815	0.405733567	76
chr7.fa	5920429	5925994	OCM	1.644621584	76
chr7.fa	5938341	5965603	CCZ1	6.275315707	760
chr7.fa	6012870	6048737	PMS2	12.10799929	2280
chr7.fa	6048882	6057676	AIMP2	10.04047501	458
chr7.fa	6061878	6098860	EIF2AK1	41.46464516	10820
chr7.fa	6144550	6201195	USP42	5.365352007	1824
chr7.fa	6201412	6312242	CYTH3	3.580515891	1062
chr7.fa	6369040	6388590	C7orf70	5.169864936	760
chr7.fa	6414126	6443598	RAC1	73.50690386	11704
chr7.fa	6448747	6487643	DAGLB	0.786617082	152
chr7.fa	6500712	6523849	KDELR2	24.01283058	4560
chr7.fa	6536409	6591067	GRID2IP	0.247145057	76
chr7.fa	6617065	6628610	ZDHHC4	39.61565073	4256
chr7.fa	6629915	6648355	C7orf26	9.573294044	1216
chr7.fa	6655527	6663921	ZNF853	2.879142016	684
chr7.fa	6728064	6746566	ZNF12	10.40765335	3420
chr7.fa	6774936	6791232	PMS2CL	2.634256055	304
chr7.fa	6838566	6865926	C7orf28B	0	0
chr7.fa	7115401	7136417	LOC100131257	0	0
chr7.fa	7222246	7288251	C1GALT1	2.212558213	912
chr7.fa	7398244	7575460	COL28A1	1.829566209	427
chr7.fa	7606616	7647110	MIOS	38.92286142	8588
chr7.fa	7676575	7758238	RPA3	7.718124752	760
chr7.fa	7712940	7918851	LOC729852	16.96505479	2432
chr7.fa	8008423	8128709	GLCCI1	4.101613939	1292
chr7.fa	8152815	8302185	ICA1	34.24412386	5673
chr7.fa	8473585	8792593	NXPH1	0	0
chr7.fa	9673900	9675447	PER4	0	0
chr7.fa	10971580	10979813	NDUFA4	81.57473661	11147
chr7.fa	11013499	11209250	PHF14	9.792125103	5654
chr7.fa	11410062	11871824	THSD7A	3.784738132	2660
chr7.fa	12250848	12276890	TMEM106B	2.108639816	912
chr7.fa	12370509	12443852	VWDE	0.410101151	152
chr7.fa	12610203	12693228	SCIN	13.88666054	3040
chr7.fa	12726452	12730556	ARL4A	1.002285407	228
chr7.fa	13930856	14031050	ETV1	0.151058191	76
chr7.fa	14184674	14881075	DGKB	0	0

chr7.fa	15239943	15601640	TMEM195	0	0
chr7.fa	15650837	15726308	MEOX2	0.482693423	76
chr7.fa	16127152	16460947	ISPD	2.4509681	899
chr7.fa	16501106	16505474	SOSTDC1	1.837247134	228
chr7.fa	16566505	16621114	LOC100129335	0	0
chr7.fa	16639401	16685442	ANKMY2	17.47154402	2964
chr7.fa	16685759	16746148	BZW2	24.65501615	3040
chr7.fa	16793351	16824161	TSPAN13	4.814283291	608
chr7.fa	16832264	16844738	AGR2	273.7879263	17870
chr7.fa	16899030	16921613	AGR3	3.052339344	152
chr7.fa	17338276	17385775	AHR	46.17275096	19152
chr7.fa	17830385	17980131	SNX13	6.288117249	2660
chr7.fa	18066400	18067486	PRPS1L1	0	0
chr7.fa	18535369	19036992	HDAC9	0.963278356	304
chr7.fa	19155091	19157295	TWIST1	0.68706627	76
chr7.fa	19184405	19185044	FERD3L	0	0
chr7.fa	19735085	19748660	TWISTNB	6.764183991	1748
chr7.fa	19744981	19745059	MIR3146	0	0
chr7.fa	19758938	19812404	TMEM196	0	0
chr7.fa	20174279	20257013	MACC1	18.24776926	11096
chr7.fa	20370725	20455382	ITGB8	3.260176137	1900
chr7.fa	20655245	20796637	ABCB5	0	0
chr7.fa	20821894	20826508	SP8	5.524994762	1368
chr7.fa	20866917	20867439	RPL23P8	2.1886118	76
chr7.fa	21467689	21554151	SP4	3.157613198	1216
chr7.fa	21582833	21939738	DNAH11	0.171390051	152
chr7.fa	21940517	21985542	CDCA7L	2.688173136	380
chr7.fa	22157908	22396533	RAPGEF5	1.209971594	532
chr7.fa	22459063	22539901	MGC87042	0	0
chr7.fa	22766766	22771621	IL6	0	0
chr7.fa	22852252	22862421	TOMM7	246.3489514	11908
chr7.fa	22896232	22896305	SNORD93	0	0
chr7.fa	22980878	23053770	FAM126A	1.674742858	684
chr7.fa	23145353	23215038	KLHL7	8.389527959	2280
chr7.fa	23221446	23240630	NUPL2	9.554317641	1140
chr7.fa	23286316	23314729	GPNMB	0	0
chr7.fa	23338940	23349180	C7orf30	15.16033863	760
chr7.fa	23349828	23509995	IGF2BP3	0	0
chr7.fa	23530007	23531031	RPS2P32	0	0
chr7.fa	23544401	23571656	TRA2A	25.51874369	3116
chr7.fa	23624335	23626146	CLK2P	0	0
chr7.fa	23636998	23684327	CCDC126	1.851404133	304
chr7.fa	23719749	23742269	C7orf46	7.775806993	760

chr7.fa	23749838	23872127	STK31	0.315369743	76
chr7.fa	24323807	24331484	NPY	36.79102822	1368
chr7.fa	24613085	24727498	MPP6	25.1217453	3648
chr7.fa	24737974	24797639	DFNA5	0.446397287	76
chr7.fa	24836164	25019760	OSBPL3	3.583829231	1596
chr7.fa	25158270	25164980	CYCS	7.8539717	2888
chr7.fa	25174316	25219817	C7orf31	2.040565736	489
chr7.fa	25264191	25268105	NPVF	0	0
chr7.fa	25989539	25989606	MIR148A	0	0
chr7.fa	26191847	26226756	NFE2L3	1.234068613	304
chr7.fa	26229556	26240413	HNRNPA2B1	112.9873101	27773
chr7.fa	26241099	26252976	CBX3	27.8162439	3572
chr7.fa	26331515	26413949	SNX10	1.51856405	304
chr7.fa	26443107	26535986	LOC441204	0.701373875	76
chr7.fa	26572740	26578444	KIAA0087	0	0
chr7.fa	26677490	26686924	C7orf71	0	0
chr7.fa	26706688	26904341	SKAP2	14.31709355	3774
chr7.fa	27132614	27135625	HOXA1	0.898818829	152
chr7.fa	27139973	27142394	HOXA2	0	0
chr7.fa	27145809	27166639	HOXA3	3.951459386	912
chr7.fa	27168126	27170399	HOXA4	1.987100474	228
chr7.fa	27180996	27183287	HOXA5	8.593147775	760
chr7.fa	27185116	27187368	HOXA6	0	0
chr7.fa	27193338	27196296	HOXA7	1.704111101	228
chr7.fa	27202057	27205149	HOXA9	9.459586233	1292
chr7.fa	27209099	27209182	MIR196B	0	0
chr7.fa	27210210	27219876	HOXA10	15.10325882	3192
chr7.fa	27220776	27224835	HOXA11	4.745757392	836
chr7.fa	27225027	27228912	HOXA11-AS1	5.911450712	608
chr7.fa	27236499	27239725	HOXA13	16.84592515	2812
chr7.fa	27282164	27286192	EVX1	1.23211073	152
chr7.fa	27565059	27702620	HIBADH	50.98613061	6764
chr7.fa	27779738	27869386	TAX1BP1	90.06833357	20728
chr7.fa	27870193	28220437	JAZF1	2.199756671	456
chr7.fa	28220076	28280996	LOC100128081	0	0
chr7.fa	28338940	28865511	CREB5	0	0
chr7.fa	28992974	28998029	TRIL	0	0
chr7.fa	29035247	29186153	CPVL	3.86034253	456
chr7.fa	29234121	29553944	CHN2	4.119536097	1052
chr7.fa	29603427	29606911	PRR15	8.185456325	912
chr7.fa	29685538	29690955	LOC646762	5.704065738	2052
chr7.fa	29724388	29724769	LOC100271874	0	0
chr7.fa	29725438	29782019	DPY19L2P3	0.569743906	76

chr7.fa	29874341	29945791	WIPF3	0.353171943	76
chr7.fa	29959719	30029905	SCRN1	37.72749864	13908
chr7.fa	30052884	30066268	FKBP14	1.018852107	152
chr7.fa	30067977	30157961	PLEKHA8	3.900253219	2280
chr7.fa	30174552	30202381	C7orf41	25.00261566	9652
chr7.fa	30323923	30407308	ZNRF2	7.60863392	1748
chr7.fa	30409666	30412410	DKFZP586I1420	1.250936527	228
chr7.fa	30464143	30518393	NOD1	1.018400288	304
chr7.fa	30536237	30544457	GGCT	24.16976242	2128
chr7.fa	30634181	30673648	GARS	27.06426628	4940
chr7.fa	30692863	30722140	CRHR2	0	0
chr7.fa	30793347	30797218	INMT	0	0
chr7.fa	30805961	30806177	INMT-FAM188B	0	0
chr7.fa	30811033	30811172	FAM188B	0	0
chr7.fa	30951415	30965131	AQP1	13.3514055	2812
chr7.fa	31003636	31019146	GHRHR	0	0
chr7.fa	31092076	31151093	ADCYAP1R1	0	0
chr7.fa	31377080	31380538	NEUROD6	0	0
chr7.fa	31556978	31693303	CCDC129	0	0
chr7.fa	31726631	31748069	C7orf16	0	0
chr7.fa	31792632	32338383	PDE1C	0.875023022	228
chr7.fa	32524945	32534870	LSM5	7.168260888	1554
chr7.fa	32535176	32623779	AVL9	28.96913568	4526
chr7.fa	32620553	32758780	DPY19L1P1	2.408497103	304
chr7.fa	32767562	32769595	LOC441208	0	0
chr7.fa	32772593	32772689	MIR550A2	0	0
chr7.fa	32797898	32802536	LOC401321	0.246693238	76
chr7.fa	32907778	32931468	KBTBD2	10.18114136	2508
chr7.fa	32956427	32982782	RP9P	0.882553341	76
chr7.fa	32997005	33046543	FKBP9	2.635762119	608
chr7.fa	33053742	33102409	NT5C3	7.3529043	912
chr7.fa	33134410	33149002	RP9	5.1099236	380
chr7.fa	33169152	33645680	BBS9	6.662825903	1778
chr7.fa	33945112	34194112	BMPER	0	0
chr7.fa	34386124	34873943	AAA1	0	0
chr7.fa	34697897	34917944	NPSR1	0	0
chr7.fa	34968493	35077653	DPY19L1	1.092799836	353
chr7.fa	35129456	35147346	DPY19L2P1	0	0
chr7.fa	35242042	35293711	TBX20	0	0
chr7.fa	35672270	35734772	HERPUD2	8.260006479	1596
chr7.fa	35840627	35944917	7-Sep	23.54203506	3936
chr7.fa	36192836	36341152	EEPD1	1.465249394	456
chr7.fa	36363759	36429734	KIAA0895	5.421377578	1748

chr7.fa	36429432	36493400	ANLN	0.975778685	304
chr7.fa	36552549	36764154	AOAH	0.447602138	76
chr7.fa	36893961	37488511	ELMO1	0.568539055	152
chr7.fa	36958962	36959037	MIR1200	0	0
chr7.fa	37779996	37780913	GPR141	0	0
chr7.fa	37888199	37940002	TXNDC3	0	0
chr7.fa	37945535	37956525	SFRP4	0	0
chr7.fa	37960163	37991542	EPDR1	12.33601733	2128
chr7.fa	38217933	38270270	STARD3NL	16.28069943	1748
chr7.fa	38299244	38313248	TARP	400.9912731	27344
chr7.fa	38423297	38671167	AMPH	0	0
chr7.fa	38724946	38726689	FAM183B	0	0
chr7.fa	38763543	38948800	VPS41	21.64002719	7032
chr7.fa	39017609	39504390	POU6F2	0	0
chr7.fa	39606003	39612480	C7orf36	11.8271184	684
chr7.fa	39649086	39651687	LOC646999	0.439921213	76
chr7.fa	39663162	39747717	RALA	7.783638524	1444
chr7.fa	39773167	39834222	NCRNA00265	0.246542631	76
chr7.fa	39989959	40136733	CDK13	14.76921388	6840
chr7.fa	40172342	40174251	C7orf11	10.94742658	684
chr7.fa	40174575	40900366	C7orf10	3.335178111	380
chr7.fa	41728601	41742706	INHBA	0	0
chr7.fa	41733514	41818976	LOC285954	0	0
chr7.fa	42000548	42276618	GLI3	1.254099261	684
chr7.fa	42948872	42951689	C7orf25	19.44268022	2356
chr7.fa	42956462	42971805	PSMA2	80.49835287	7825
chr7.fa	42971939	42977453	MRPL32	29.51342711	1748
chr7.fa	43152198	43602938	HECW1	4.18414623	1900
chr7.fa	43190494	43190593	MIR3943	0	0
chr7.fa	43622692	43666978	STK17A	3.783382674	988
chr7.fa	43678859	43769083	C7orf44	26.71199798	1520
chr7.fa	43798272	43846941	BLVRA	29.50830649	2118
chr7.fa	43906157	43909145	MRPS24	59.03076998	2728
chr7.fa	43915501	43965996	URGCP	14.44028957	3724
chr7.fa	43966035	43995735	UBE2D4	10.68055209	2358
chr7.fa	43980494	44058748	POLR2J4	0.985266886	380
chr7.fa	44040489	44049723	SPDYE1	0	0
chr7.fa	44068486	44078575	RASA4P	0	0
chr7.fa	44078698	44082082	FLJ35390	0.085393813	9
chr7.fa	44084239	44101315	DBNL	29.93120918	4408
chr7.fa	44102326	44105186	PGAM2	1.335577308	76
chr7.fa	44111847	44122129	POLM	1.747485736	304
chr7.fa	44143960	44154159	AEBP1	0	0

chr7.fa	44154286	44163147	POLD2	27.08369451	3192
chr7.fa	44178463	44180916	MYL7	0	0
chr7.fa	44183870	44229022	GCK	0	0
chr7.fa	44240578	44253893	YKT6	42.62280816	7828
chr7.fa	44256749	44365230	CAMK2B	0.249404152	76
chr7.fa	44421965	44530385	NUDCD3	16.06216959	5244
chr7.fa	44552135	44580914	NPC1L1	0	0
chr7.fa	44605403	44614137	DDX56	16.12120729	2660
chr7.fa	44618762	44621827	TMED4	17.91944737	1216
chr7.fa	44646121	44748669	OGDH	51.84654482	15123
chr7.fa	44788530	44809479	ZMIZ2	11.35044924	3876
chr7.fa	44836241	44842716	PPIA	47.53106983	7183
chr7.fa	44866488	44887725	H2AFV	25.0627076	7828
chr7.fa	44915892	44924960	PURB	11.48524194	6916
chr7.fa	45002260	45018704	MYO1G	0.349105571	76
chr7.fa	45022627	45026259	C7orf40	2.850828018	152
chr7.fa	45024977	45025109	SNORA9	0	0
chr7.fa	45039345	45116069	CCM2	7.91210576	1140
chr7.fa	45120036	45128493	NACAD	0.961621686	304
chr7.fa	45139699	45151317	TBRG4	15.28187797	2280
chr7.fa	45143948	45144081	SNORA5A	0	0
chr7.fa	45144505	45144641	SNORA5C	0	0
chr7.fa	45145567	45145698	SNORA5B	0	0
chr7.fa	45197367	45223850	RAMP3	0	0
chr7.fa	45614125	45762714	ADCY1	2.839080721	2356
chr7.fa	45763386	45808617	SEPT7P2	4.093330588	608
chr7.fa	45927959	45933267	IGFBP1	0	0
chr7.fa	45951844	45960871	IGFBP3	6.960725307	1216
chr7.fa	47314752	47621742	TNS3	34.9352565	17671
chr7.fa	47694842	47701246	C7orf65	0	0
chr7.fa	47814250	47988071	PKD1L1	2.122646209	1279
chr7.fa	47834889	47859444	C7orf69	0	0
chr7.fa	48003783	48019246	HUS1	12.81871076	1824
chr7.fa	48026746	48068716	SUN3	0	0
chr7.fa	48075117	48100894	C7orf57	0.538568387	76
chr7.fa	48128355	48148330	UPP1	10.01457071	1292
chr7.fa	48211057	48687091	ABCA13	0.7327	836
chr7.fa	48964157	48967049	CDC14C	1.186928819	228
chr7.fa	49813257	49952138	VWC2	0	0
chr7.fa	49977024	50132860	ZPBP	0	0
chr7.fa	50135682	50199359	C7orf72	0	0
chr7.fa	50344378	50472798	IKZF1	0.184944625	76
chr7.fa	50511832	50518088	FIGNL1	2.593291122	608

chr7.fa	50526134	50633154	DDC	13.28092171	1776
chr7.fa	50599457	50611161	LOC100129427	0	0
chr7.fa	50657760	50861159	GRB10	10.84727335	3800
chr7.fa	51083909	51384515	COBL	26.7290165	9424
chr7.fa	53103349	53104618	POM121L12	0	0
chr7.fa	54268917	54270114	HPVC1	0	0
chr7.fa	54610019	54636948	VSTM2A	0	0
chr7.fa	54819940	54826939	SEC61G	81.05589766	2508
chr7.fa	55086725	55275031	EGFR	4.25764214	1824
chr7.fa	55433141	55501435	LANCL2	9.203103582	2660
chr7.fa	55538306	55640200	VOPP1	5.054349849	988
chr7.fa	55748767	55772260	FKBP9L	0	0
chr7.fa	55861237	55930482	14-Sep	0	0
chr7.fa	55980331	56008433	ZNF713	0.554231449	76
chr7.fa	56019611	56023033	MRPS17	27.39228696	1064
chr7.fa	56032296	56067871	GBAS	50.49967203	6619
chr7.fa	56078744	56119268	PSPH	8.646763643	1216
chr7.fa	56119378	56131682	CCT6A	46.83270808	8284
chr7.fa	56128163	56128295	SNORA15	0	0
chr7.fa	56131917	56148365	SUMF2	22.2903455	3040
chr7.fa	56148675	56160689	PHKG1	0	0
chr7.fa	56169266	56174187	CHCHD2	112.3541609	6080
chr7.fa	56182374	56184090	LOC389493	17.16912642	684
chr7.fa	56491397	56516068	LOC650226	0	0
chr7.fa	56563916	56564977	DKFZp434L192	0	0
chr7.fa	57187326	57207571	ZNF479	0	0
chr7.fa	57233377	57247863	LOC642006	0	0
chr7.fa	57472731	57472796	MIR3147	0	0
chr7.fa	57509883	57533265	ZNF716	0	0
chr7.fa	62751670	62764434	LOC643955	0	0
chr7.fa	62856718	62859419	LOC100287834	0	0
chr7.fa	63505821	63538927	ZNF727	3.40867402	380
chr7.fa	63667581	63680668	ZNF735	0	0
chr7.fa	63688852	63727309	ZNF679	0	0
chr7.fa	63773186	63810017	ZNF736	3.818925778	532
chr7.fa	63894071	63895895	LOC649395	0.627124934	76
chr7.fa	63980255	64023505	ZNF680	2.097193732	684
chr7.fa	64042988	64044129	LOC641746	0	0
chr7.fa	64126511	64171401	ZNF107	2.91091996	1064
chr7.fa	64254771	64294025	ZNF138	3.364094534	608
chr7.fa	64310071	64314178	LOC168474	0.557243577	152
chr7.fa	64363620	64391955	ZNF273	0.518688346	152
chr7.fa	64434830	64450732	ZNF117	3.400691883	1292

chr7.fa	64451415	64467124	ERV3	3.54361733	608
chr7.fa	64498732	64535091	CCT6P3	2.040264523	304
chr7.fa	64601603	64694600	INTS4L1	0	0
chr7.fa	64838768	64865998	ZNF92	1.837849559	380
chr7.fa	65112777	65183632	INTS4L2	0	0
chr7.fa	65216092	65228662	CCT6P1	0	0
chr7.fa	65220513	65220646	SNORA22	0	0
chr7.fa	65338257	65419800	VKORC1L1	8.989091927	684
chr7.fa	65425673	65447301	GUSB	2.49042697	380
chr7.fa	65540776	65558329	ASL	18.85064657	2651
chr7.fa	65579805	65619553	CRCP	19.18770363	3724
chr7.fa	65670259	65825438	TPST1	2.056078192	280
chr7.fa	65841031	65865395	NCRNA00174	1.552299878	456
chr7.fa	66018553	66043498	LOC493754	4.51999844	988
chr7.fa	66093868	66108038	KCTD7	6.10708839	1976
chr7.fa	66205643	66274979	RABGEF1	20.07221485	3116
chr7.fa	66276449	66309813	LOC729156	0	0
chr7.fa	66386203	66423538	C7orf42	12.69385807	3572
chr7.fa	66452690	66460588	SBDS	19.96829646	2128
chr7.fa	66461817	66704498	TYW1	2.749771142	608
chr7.fa	66757423	66767406	PMS2P4	18.1045426	684
chr7.fa	66767616	66786513	STAG3L4	4.824976344	684
chr7.fa	69063905	70257475	AUTS2	9.47841203	3941
chr7.fa	70597789	71178584	WBSCR17	1.408772005	304
chr7.fa	70772658	70772756	MIR3914-1	0	0
chr7.fa	71244476	71877360	CALN1	0.114611449	76
chr7.fa	72023729	72298813	TYW1B	1.039635787	228
chr7.fa	72299952	72307978	SBDSP1	1.911947894	228
chr7.fa	72333318	72339655	SPDYE7P	0	0
chr7.fa	72349936	72418831	POM121	4.968203004	1976
chr7.fa	72418839	72425302	NSUN5P2	1.30063663	152
chr7.fa	72440192	72443668	LOC541473	0	0
chr7.fa	72469027	72476455	STAG3L3	0	0
chr7.fa	72476680	74988215	PMS2L2	0	0
chr7.fa	72490260	74974606	SPDYE8P	0	0
chr7.fa	72569012	74653459	LOC100093631	0	0
chr7.fa	72569129	74653341	GTF2IP1	0	0
chr7.fa	72634674	72649979	NCF1B	0	0
chr7.fa	72656902	72685658	GTF2IRD2P1	0	0
chr7.fa	72716514	72722864	NSUN5	3.216199077	498
chr7.fa	72726535	72742085	TRIM50	0	0
chr7.fa	72742155	72772641	FKBP6	0	0
chr7.fa	72848109	72850450	FZD9	0	0

chr7.fa	72854728	72936615	BAZ1B	46.42079966	18848
chr7.fa	72950683	72972065	BCL7B	21.79650721	2812
chr7.fa	72983277	72993013	TBL2	1.507117966	304
chr7.fa	73007524	73038870	MLXIPL	0.349858602	76
chr7.fa	73082174	73086440	VPS37D	0.713121172	76
chr7.fa	73095248	73097781	DNAJC30	7.227147979	1216
chr7.fa	73097898	73112542	WBSCR22	13.86075625	1132
chr7.fa	73113535	73134017	STX1A	0.538568387	76
chr7.fa	73125647	73125727	MIR4284	0	0
chr7.fa	73149399	73150330	WBSCR26	2.419943187	76
chr7.fa	73150425	73153184	ABHD11	28.82952357	2812
chr7.fa	73183327	73184600	CLDN3	7.187538503	608
chr7.fa	73245193	73247015	CLDN4	20.71967165	2508
chr7.fa	73248921	73256855	WBSCR27	7.384531638	456
chr7.fa	73275489	73280223	WBSCR28	0	0
chr7.fa	73442427	73484236	ELN	0	0
chr7.fa	73498156	73536854	LIMK1	8.588027158	1900
chr7.fa	73588706	73611429	EIF4H	66.53653975	11248
chr7.fa	73605528	73605624	MIR590	0	0
chr7.fa	73624087	73644164	LAT2	2.557145592	372
chr7.fa	73645832	73668738	RFC2	9.192862348	1034
chr7.fa	73703805	73820273	CLIP2	9.193916593	3396
chr7.fa	73868120	74016920	GTF2IRD1	11.95799534	2802
chr7.fa	74072030	74175022	GTF2I	27.79350234	8358
chr7.fa	74188309	74203659	NCF1	0	0
chr7.fa	74210484	74267841	GTF2IRD2	0.644595273	152
chr7.fa	74299283	74306731	STAG3L2	1.018400288	76
chr7.fa	74306894	74336166	PMS2P5	0.56582814	76
chr7.fa	74379083	74438803	GATSL1	0	0
chr7.fa	74456283	74489699	WBSCR16	12.99597446	2052
chr7.fa	74508347	74565623	GTF2IRD2B	2.197196363	523
chr7.fa	74572384	74587816	NCF1C	0	0
chr7.fa	74807605	74867341	GATSL2	0	0
chr7.fa	74988447	74996990	STAG3L1	1.253647442	152
chr7.fa	75043758	75046064	NSUN5P1	2.201112129	228
chr7.fa	75046067	75115568	POM121C	3.319966867	1292
chr7.fa	75124299	75133628	SPDYE5	0	0
chr7.fa	75137069	75157453	PMS2P3	2.287710793	228
chr7.fa	75163409	75368279	HIP1	1.26494292	608
chr7.fa	75398842	75419064	CCL26	0	0
chr7.fa	75441114	75443033	CCL24	0	0
chr7.fa	75508317	75518244	RHBDD2	17.67501323	2204
chr7.fa	75544420	75616154	POR	5.945337146	979

chr7.fa	75573101	75573234	SNORA14A	0	0
chr7.fa	75616174	75623992	TMEM120A	15.65191783	1509
chr7.fa	75625655	75677321	STYXL1	3.233368203	304
chr7.fa	75677393	75695930	MDH2	105.2514138	9120
chr7.fa	75831216	75916605	SRRM3	0.316574594	76
chr7.fa	75931875	75933614	HSPB1	236.8566836	14107
chr7.fa	75956108	75988342	YWHAG	45.51550475	11324
chr7.fa	76018646	76039012	SRCRB4D	0.452120329	76
chr7.fa	76054272	76071388	ZP3	0.880444851	76
chr7.fa	76090972	76135312	DTX2	5.088989314	912
chr7.fa	76099337	76104317	FDPSL2A	0	0
chr7.fa	76139745	76157199	UPK3B	8.809870344	988
chr7.fa	76178658	76257299	LOC100133091	0.610708839	76
chr7.fa	76239303	76255000	POMZP3	7.387844978	363
chr7.fa	76610139	76653076	PMS2P11	0.650017102	76
chr7.fa	76668797	76682355	LOC100132832	0.490826167	76
chr7.fa	76751934	76924521	CCDC146	3.434126497	760
chr7.fa	76822688	76829150	FGL2	0.268229949	76
chr7.fa	76940068	77045717	PION	7.648394002	1651
chr7.fa	77166773	77269388	PTPN12	19.29508597	4224
chr7.fa	77325743	77409120	RSBN1L	8.531248556	1824
chr7.fa	77423045	77427747	TMEM60	3.933386621	228
chr7.fa	77428109	77586821	PHTF2	1.388891964	608
chr7.fa	77646374	79082890	MAGI2	6.322003683	2888
chr7.fa	77976559	77988770	RPL13AP17	0	0
chr7.fa	79400572	79400606	MIR548M	0	0
chr7.fa	79764140	79848725	GNAI1	3.449638954	760
chr7.fa	80087987	80141242	GNAT3	0	0
chr7.fa	80231504	80308593	CD36	0	0
chr7.fa	80371854	80548667	SEMA3C	19.37746766	6657
chr7.fa	81331444	81399452	HGF	0	0
chr7.fa	81579418	82073031	CACNA2D1	4.192730794	1064
chr7.fa	82383321	82792197	PCLO	8.006084136	11856
chr7.fa	82993222	83278479	SEMA3E	1.360879179	608
chr7.fa	83587659	83824217	SEMA3A	0	0
chr7.fa	84624872	84751247	SEMA3D	1.280304769	532
chr7.fa	86273230	86494192	GRM3	0	0
chr7.fa	86506223	86689014	KIAA1324L	3.207012088	1520
chr7.fa	86781677	86825477	DMTF1	11.52379717	3268
chr7.fa	86825649	86849031	C7orf23	3.937904812	228
chr7.fa	86954664	86974808	TP53TG1	50.04634685	2356
chr7.fa	86974951	87029112	CROT	20.68563461	4560
chr7.fa	87031361	87105019	ABCB4	0.288561809	76

chr7.fa	87132948	87342564	ABCB1	3.98158066	1288
chr7.fa	87257729	87461613	RUNDC3B	0.837371429	228
chr7.fa	87463814	87505543	SLC25A40	6.883765451	1368
chr7.fa	87505693	87538856	DBF4	1.218254944	304
chr7.fa	87563566	87832204	ADAM22	2.046138172	1292
chr7.fa	87834432	87856308	SRI	49.99845403	6955
chr7.fa	87905744	87936209	STEAP4	11.56431028	3420
chr7.fa	88388753	88966346	ZNF804B	0.249404152	76
chr7.fa	88423420	88425031	MGC26647	0	0
chr7.fa	89748714	89754914	DPY19L2P4	0	0
chr7.fa	89783689	89794141	STEAP1	19.17851664	1672
chr7.fa	89841000	89866992	STEAP2	33.97333361	6156
chr7.fa	89874488	89940377	C7orf63	8.285760169	2117
chr7.fa	89975979	90020769	GTPBP10	3.794075727	1900
chr7.fa	90032648	90045268	CLDN12	1.87640479	456
chr7.fa	90338712	90839904	CDK14	4.852989129	1596
chr7.fa	90893783	90898132	FZD1	2.105025263	608
chr7.fa	91502021	91510016	MTERF	4.233394514	608
chr7.fa	91570189	91739987	AKAP9	15.79649995	13072
chr7.fa	91741463	91764059	CYP51A1	12.53225744	2736
chr7.fa	91774198	91794590	LOC401387	0	0
chr7.fa	91828283	91875414	KRIT1	11.50933896	3876
chr7.fa	91875548	92030698	ANKIB1	25.78712425	10412
chr7.fa	92076765	92088742	GATAD1	11.28689335	2964
chr7.fa	92116337	92157845	PEX1	12.53798048	3648
chr7.fa	92158087	92166823	C7orf64	5.333122243	608
chr7.fa	92167789	92169079	MGC16142	0.886619713	76
chr7.fa	92190072	92214710	FAM133B	0	0
chr7.fa	92234235	92465941	CDK6	2.221594595	1735
chr7.fa	92728826	92747336	SAMD9	2.003667175	912
chr7.fa	92759368	92777680	SAMD9L	0.962826537	456
chr7.fa	92817899	92855782	HEPACAM2	0.381335334	53
chr7.fa	92861653	92988338	CCDC132	12.59701818	3022
chr7.fa	93053799	93204042	CALCR	0	0
chr7.fa	93112072	93112167	MIR653	0	0
chr7.fa	93113248	93113331	MIR489	0	0
chr7.fa	93515745	93520065	TFPI2	0	0
chr7.fa	93535820	93540485	GNGT1	0	0
chr7.fa	93551016	93555826	GNG11	5.998953015	380
chr7.fa	93621000	93633690	BET1	7.852616243	1596
chr7.fa	94023873	94060544	COL1A2	0	0
chr7.fa	94139170	94186328	CASD1	3.164390485	815
chr7.fa	94214536	94285521	SGCE	2.529584626	304

chr7.fa	94285637	94299006	PEG10	3.626601441	1596
chr7.fa	94536949	94925727	PPP1R9A	14.59134776	10104
chr7.fa	94927669	94953884	PON1	0.653330442	76
chr7.fa	94989184	95025687	PON3	0	0
chr7.fa	95034174	95064384	PON2	3.4601814	380
chr7.fa	95115213	95169543	ASB4	0	0
chr7.fa	95212809	95225925	PDK4	4.330987443	1064
chr7.fa	95401818	95727736	DYNC1I1	0	0
chr7.fa	95749532	95951459	SLC25A13	14.63577664	3100
chr7.fa	95848974	95849068	MIR591	0	0
chr7.fa	96318079	96339203	SHFM1	91.93645501	3040
chr7.fa	96597827	96643377	DLX6-AS1	0	0
chr7.fa	96635290	96640352	DLX6	0	0
chr7.fa	96649702	96654143	DLX5	0	0
chr7.fa	96745905	96811075	ACN9	8.34675575	1140
chr7.fa	97361271	97369784	TAC1	0.963428963	76
chr7.fa	97481429	97501854	ASNS	3.80266029	608
chr7.fa	97595908	97601638	MGC72080	4.919557146	228
chr7.fa	97614013	97619416	OCM2	0	0
chr7.fa	97736197	97838944	LMTK2	16.12301456	9576
chr7.fa	97841566	97842271	BHLHA15	9.727514969	456
chr7.fa	97846047	97881468	TECPR1	7.616766664	2204
chr7.fa	97910979	97920839	BRI3	140.3628797	7372
chr7.fa	97920962	98030427	BAIAP2L1	23.70981056	5266
chr7.fa	98246597	98259181	NPTX2	0	0
chr7.fa	98444111	98467673	TMEM130	0	0
chr7.fa	98476113	98610864	TRRAP	12.18435672	10184
chr7.fa	98479273	98479352	MIR3609	14.30760535	76
chr7.fa	98625058	98741743	SMURF1	21.45026316	8208
chr7.fa	98771197	98805089	KPNA7	0.679234738	76
chr7.fa	98870924	98895594	MYH16	0	0
chr7.fa	98923496	98963885	ARPC1A	158.8349019	16948
chr7.fa	98972329	98992297	ARPC1B	35.33255611	3268
chr7.fa	98992405	99006305	PDAP1	29.72427603	5092
chr7.fa	99006601	99013883	BUD31	47.84643957	2065
chr7.fa	99036315	99036462	PTCD1	6.309202141	62
chr7.fa	99036563	99054996	CPSF4	13.16359935	1596
chr7.fa	99055784	99063824	ATP5J2	77.66138064	4347
chr7.fa	99070515	99085217	ZNF789	3.888807135	456
chr7.fa	99090854	99097877	ZNF394	21.87828647	3116
chr7.fa	99102273	99131445	ZKSCAN5	6.203777681	1824
chr7.fa	99143923	99149757	FAM200A	4.600572849	760
chr7.fa	99156045	99174076	ZNF655	7.89132208	3192

chr7.fa	99195902	99208455	LOC100289187	3.090743969	228
chr7.fa	99214571	99230030	ZNF498	4.737474042	1368
chr7.fa	99245813	99277621	CYP3A5	2.195539693	456
chr7.fa	99302660	99332819	CYP3A7	1.101685612	152
chr7.fa	99354604	99381808	CYP3A4	0.826979589	152
chr7.fa	99425636	99464173	CYP3A43	0	0
chr7.fa	99473685	99474656	OR2AE1	0	0
chr7.fa	99488030	99517223	TRIM4	22.24682026	5108
chr7.fa	99520892	99527243	GJC3	2.051258788	152
chr7.fa	99564350	99573735	AZGP1	118.5326367	9956
chr7.fa	99578385	99581860	AZGP1P1	0	0
chr7.fa	99613219	99635403	ZKSCAN1	42.50096761	15504
chr7.fa	99647417	99662663	ZSCAN21	8.279284095	608
chr7.fa	99667594	99679371	ZNF3	15.80734361	2964
chr7.fa	99686583	99689822	COPS6	43.98760311	4162
chr7.fa	99690404	99699427	MCM7	14.39992706	2964
chr7.fa	99691183	99691266	MIR25	0	0
chr7.fa	99691391	99691470	MIR93	0	0
chr7.fa	99691616	99691697	MIR106B	0	0
chr7.fa	99699503	99704692	AP4M1	7.131362327	706
chr7.fa	99704804	99717021	TAF6	6.762376715	1202
chr7.fa	99717265	99723128	CNPY4	0	0
chr7.fa	99724320	99726121	MBLAC1	0	0
chr7.fa	99746530	99751833	C7orf59	110.3462767	4484
chr7.fa	99752043	99756302	C7orf43	3.186981441	532
chr7.fa	99756865	99766373	GAL3ST4	0.450614265	76
chr7.fa	99767229	99774990	GPC2	0	0
chr7.fa	99775538	99812010	STAG3	0	0
chr7.fa	99798278	99869855	GATS	5.236282346	1672
chr7.fa	99816871	99819111	PVRIG	0	0
chr7.fa	99905325	99919819	SPDYE3	0	0
chr7.fa	99918263	99933687	PMS2P1	6.353028595	456
chr7.fa	99933931	99965454	PILRB	3.480061441	760
chr7.fa	99971068	99997722	PILRA	0	0
chr7.fa	99998495	100026302	ZCWPW1	0.979543844	152
chr7.fa	100026413	100031749	MEPCE	18.85697204	3939
chr7.fa	100032912	100034094	C7orf47	7.384531638	456
chr7.fa	100054238	100061894	C7orf61	0	0
chr7.fa	100064142	100076902	TSC22D4	27.8587149	4408
chr7.fa	100081550	100092422	C7orf51	0	0
chr7.fa	100136834	100165843	AGFG2	9.516666048	3040
chr7.fa	100169853	100171270	SAP25	0	0
chr7.fa	100171634	100183776	LRCH4	15.01831682	3192

chr7.fa	100183956	100198740	FBXO24	0	0
chr7.fa	100199882	100205798	PCOLCE	0.706494492	76
chr7.fa	100209725	100213000	MOSPD3	8.999784979	836
chr7.fa	100218039	100239173	TFR2	0	0
chr7.fa	100240726	100254084	ACTL6B	0	0
chr7.fa	100271363	100276792	GNB2	107.6328017	11892
chr7.fa	100277130	100286870	GIGYF1	6.691441114	2812
chr7.fa	100303676	100305123	POP7	15.9656309	988
chr7.fa	100318423	100321323	EPO	0.86056481	76
chr7.fa	100331249	100395419	ZAN	0	0
chr7.fa	100400187	100425143	EPHB4	5.524392336	1596
chr7.fa	100450358	100464634	SLC12A9	0.347448901	76
chr7.fa	100464950	100471076	TRIP6	24.82595438	2888
chr7.fa	100472701	100486285	SRRT	16.71519882	3344
chr7.fa	100486344	100487339	UFSP1	0	0
chr7.fa	100487615	100493541	ACHE	0	0
chr7.fa	100612904	100662230	MUC12	0.631191306	684
chr7.fa	100663364	100702140	MUC17	0.079821377	76
chr7.fa	100728786	100733889	TRIM56	20.44526684	4788
chr7.fa	100770379	100782547	SERPINE1	0	0
chr7.fa	100797686	100804557	AP1S1	54.71529499	4712
chr7.fa	100805790	100808852	VGF	0	0
chr7.fa	100813774	100823557	C7orf52	0	0
chr7.fa	100839013	100844302	MOGAT3	0.813575622	76
chr7.fa	100849258	100860984	PLOD3	2.3280733	456
chr7.fa	100861012	100867471	ZNHIT1	47.28151507	3648
chr7.fa	100875373	100882101	CLDN15	0.510103782	76
chr7.fa	100882893	100888371	FIS1	123.9088324	6080
chr7.fa	100956648	100965093	RABL5	16.02828315	2584
chr7.fa	101006122	101202304	EMID2	0.380883515	76
chr7.fa	101256605	101272576	MYL10	0	0
chr7.fa	101459292	101927250	CUX1	20.55129372	9975
chr7.fa	101928405	101962178	SH2B2	0	0
chr7.fa	101936369	101936453	MIR4285	0	0
chr7.fa	101986192	101996889	SPDYE6	0	0
chr7.fa	102036804	102067129	PRKRIP1	7.347331864	1064
chr7.fa	102073996	102096666	ORAI2	0.900626105	608
chr7.fa	102097269	102105321	ALKBH4	3.767569005	380
chr7.fa	102105390	102113547	LRWD1	4.829645141	684
chr7.fa	102113613	102119381	POLR2J	15.07720391	912
chr7.fa	102178366	102213068	POLR2J3	0	0
chr7.fa	102220093	102257205	RASA4	0.204222241	76
chr7.fa	102283077	102283238	UPK3BL	0	0

chr7.fa	102306524	102312182	POLR2J2	2.13544775	76
chr7.fa	102389399	102449672	FAM185A	3.972393671	532
chr7.fa	102453308	102715288	FBXL13	0.714928448	152
chr7.fa	102553344	102585556	LRRC17	0	0
chr7.fa	102715328	102740022	ARMC10	10.76293378	1748
chr7.fa	102740206	102789569	NAPEPLD	12.81615045	4408
chr7.fa	102781717	102782850	RPL19P12	0	0
chr7.fa	102815462	102920759	DPY19L2P2	0	0
chr7.fa	102937873	102955133	PMPCB	22.29335763	5320
chr7.fa	102956211	102985320	DNAJC2	11.52500202	1444
chr7.fa	102988089	103008656	PSMC2	79.89924072	8101
chr7.fa	102993177	103086624	SLC26A5	0	0
chr7.fa	103112231	103629963	RELN	0	0
chr7.fa	103766788	103848495	ORC5	7.82866983	1824
chr7.fa	103969104	104549003	LHFPL3	0	0
chr7.fa	104436954	104444539	LOC645591	0	0
chr7.fa	104535075	104567092	LOC723809	4.061100824	494
chr7.fa	104650989	104654588	LOC100216545	0.953790155	228
chr7.fa	104654637	104754532	MLL5	33.98854485	15389
chr7.fa	104756823	105029341	SRPK2	21.94380024	5646
chr7.fa	105096960	105162685	PUS7	5.588249438	1292
chr7.fa	105172532	105208124	RINT1	10.21909417	1887
chr7.fa	105205580	105221976	EFCAB10	0	0
chr7.fa	105245221	105517031	ATXN7L1	3.712447073	1745
chr7.fa	105603657	105676877	CDHR3	0	0
chr7.fa	105730952	105753057	SYPL1	15.36034389	2204
chr7.fa	105888732	105925638	NAMPT	26.48518478	8056
chr7.fa	106297211	106301634	FLJ36031	3.104750361	912
chr7.fa	106505924	106547592	PIK3CG	1.48949702	532
chr7.fa	106685178	106802256	PRKAR2B	12.47306913	3032
chr7.fa	106809460	106842188	HBP1	52.10408171	7068
chr7.fa	106842975	107204959	COG5	13.39432831	5820
chr7.fa	107110502	107116125	GPR22	0	0
chr7.fa	107205033	107218968	DUS4L	5.646835317	760
chr7.fa	107220422	107263762	BCAP29	1.31388999	532
chr7.fa	107296961	107302083	LOC286002	0	0
chr7.fa	107301080	107358252	SLC26A4	0.240066557	76
chr7.fa	107384279	107402083	CBLL1	12.39836837	3572
chr7.fa	107405912	107443678	SLC26A3	0	0
chr7.fa	107531586	107561643	DLD	23.32952947	5544
chr7.fa	107564246	107643804	LAMB1	13.70548108	5320
chr7.fa	107663996	107770801	LAMB4	0	0
chr7.fa	107788071	108096841	NRCAM	1.027286064	456

chr7.fa	108112071	108166638	PNPLA8	14.2542907	3341
chr7.fa	108202671	108210167	THAP5	4.377072993	1064
chr7.fa	108210189	108215294	DNAJB9	7.66676798	1292
chr7.fa	108524038	108524637	C7orf66	0	0
chr7.fa	109599284	109600270	EIF3IP1	0	0
chr7.fa	110303110	111202347	IMMP2L	5.274686971	532
chr7.fa	110731062	110765509	LRRN3	0	0
chr7.fa	111366164	111846462	DOCK4	2.059692745	1140
chr7.fa	111846643	111983989	ZNF277	15.88460467	2834
chr7.fa	112063199	112117258	IFRD1	6.509960435	1748
chr7.fa	112120908	112130943	C7orf53	0	0
chr7.fa	112405787	112430478	TMEM168	6.083744402	1596
chr7.fa	112459202	112579932	C7orf60	9.582029214	2508
chr7.fa	112720468	112727833	GPR85	0.224403494	76
chr7.fa	112756773	112758637	LOC401397	24.3750389	1824
chr7.fa	113516882	113559082	PPP1R3A	0	0
chr7.fa	113726365	114333827	FOXP2	0.329677349	152
chr7.fa	114293400	114293510	MIR3666	0	0
chr7.fa	114562209	114659970	MDFIC	6.844005369	2508
chr7.fa	115575202	115670798	TFEC	0.517784707	228
chr7.fa	115850581	115898837	TES	17.20602498	3192
chr7.fa	116139444	116148595	CAV2	2.427172293	532
chr7.fa	116164839	116201239	CAV1	0	0
chr7.fa	116312459	116438440	MET	5.82937024	2584
chr7.fa	116502563	116559313	CAPZA2	55.00717014	8605
chr7.fa	116592501	116594388	ST7OT1	0	0
chr7.fa	116594515	116599867	ST7OT4	0	0
chr7.fa	116739816	116870075	ST7	5.468065553	867
chr7.fa	116752346	116785614	ST7OT2	0	0
chr7.fa	116822735	116839198	ST7OT3	0	0
chr7.fa	116916686	116963343	WNT2	0	0
chr7.fa	117003276	117067577	ASZ1	0	0
chr7.fa	117120017	117308718	CFTR	0.186751901	76
chr7.fa	117350706	117513561	CTTNBP2	11.69729571	4636
chr7.fa	117824086	117844093	NAA38	1.645675828	1368
chr7.fa	117864712	117882784	ANKRD7	0.84354629	76
chr7.fa	119913722	120390387	KCND2	1.073070401	380
chr7.fa	120427374	120498177	TSPAN12	8.928246953	1520
chr7.fa	120590817	120615711	ING3	3.027037473	608
chr7.fa	120628751	120937498	C7orf58	0	0
chr7.fa	120965421	120981158	WNT16	0	0
chr7.fa	120988905	121036422	FAM3C	0.770200987	130
chr7.fa	121513159	121702090	PTPRZ1	0	0

chr7.fa	121713598	121784344	AASS	3.899198974	1520
chr7.fa	121941448	121944565	FEZF1	0	0
chr7.fa	121943913	121950131	LOC154860	0	0
chr7.fa	121958478	122526813	CADPS2	18.72684813	7570
chr7.fa	122337766	122339208	RNF133	0.793243762	76
chr7.fa	122341720	122343021	RNF148	0	0
chr7.fa	122634759	122635754	TAS2R16	0	0
chr7.fa	122753588	122840025	SLC13A1	1.205754615	304
chr7.fa	123092236	123174718	IQUB	0.367630154	76
chr7.fa	123181083	123197958	NDUFA5	31.75369689	3268
chr7.fa	123249112	123277932	ASB15	0.423053299	76
chr7.fa	123295861	123304147	LMOD2	0	0
chr7.fa	123321997	123389116	WASL	13.39854529	3934
chr7.fa	123454193	123459484	HYALP1	0	0
chr7.fa	123485223	123517531	HYAL4	0	0
chr7.fa	123565286	123611461	SPAM1	0	0
chr7.fa	123670970	123673523	TMEM229A	0	0
chr7.fa	124386114	124405681	GPR37	0	0
chr7.fa	124417346	124430864	LOC154872	0	0
chr7.fa	124462440	124570037	POT1	6.389927157	1824
chr7.fa	126078652	126892428	GRM8	0.291122117	76
chr7.fa	126698142	126698238	MIR592	0	0
chr7.fa	127010354	127032767	ZNF800	11.38915507	3192
chr7.fa	127220682	127225654	GCC1	14.68156097	4028
chr7.fa	127228406	127231759	ARF5	63.6778802	4634
chr7.fa	127233689	127241851	FSCN3	0	0
chr7.fa	127250346	127255780	PAX4	0	0
chr7.fa	127292202	127732659	SND1	91.89654433	21405
chr7.fa	127637562	127640130	C7orf54	0	0
chr7.fa	127667124	127671002	LRRC4	0	0
chr7.fa	127721913	127722012	MIR593	0	0
chr7.fa	127847925	127847996	MIR129-1	0	0
chr7.fa	127881331	127897682	LEP	0	0
chr7.fa	127950436	127983962	RBM28	15.07675209	2812
chr7.fa	127990379	128001739	PRRT4	0	0
chr7.fa	128032331	128050036	IMPDH1	7.164345122	1292
chr7.fa	128095884	128098472	C7orf68	3.242555192	304
chr7.fa	128116783	128142978	METTL2B	4.721057947	684
chr7.fa	128281295	128301052	FLJ45340	1.891616034	1289
chr7.fa	128312346	128323309	FAM71F2	0	0
chr7.fa	128355443	128371797	FAM71F1	0	0
chr7.fa	128379346	128413477	CALU	4.233243908	1741
chr7.fa	128413703	128415844	OPN1SW	0	0

chr7.fa	128432099	128462183	CCDC136	0.823816856	228
chr7.fa	128470483	128499328	FLNC	0.125304502	76
chr7.fa	128502857	128505903	ATP6V1F	78.45537743	4256
chr7.fa	128506464	128512101	LOC100130705	0	0
chr7.fa	128516919	128550773	KCP	0.424709969	152
chr7.fa	128577994	128590088	IRF5	8.185456325	1668
chr7.fa	128594234	128695227	TNPO3	29.28480663	8892
chr7.fa	128695277	128697293	TPI1P2	0.56748481	76
chr7.fa	128766325	128768050	LOC407835	0	0
chr7.fa	128784712	128809534	TSPAN33	12.351831	2280
chr7.fa	128828713	128853385	SMO	0.918849476	228
chr7.fa	128864855	129070052	AHCYL2	6.537220189	2508
chr7.fa	129074274	129128239	FAM40B	0.223801069	76
chr7.fa	129142320	129152773	LOC100287482	0	0
chr7.fa	129251555	129396922	NRF1	6.000910897	1444
chr7.fa	129410223	129410332	MIR182	0	0
chr7.fa	129414533	129414609	MIR96	0	0
chr7.fa	129414745	129414854	MIR183	0	0
chr7.fa	129472995	129592789	UBE2H	51.79021803	9381
chr7.fa	129658126	129691233	ZC3HC1	5.382219921	684
chr7.fa	129710349	129775560	KLHDC10	19.2012582	8208
chr7.fa	129804555	129845338	TMEM209	1.937249765	456
chr7.fa	129847704	129856684	C7orf45	0	0
chr7.fa	129906703	129929637	CPA2	0	0
chr7.fa	129932974	129964020	CPA4	0	0
chr7.fa	129984630	130008571	CPA5	0	0
chr7.fa	130020290	130027949	CPA1	0	0
chr7.fa	130036375	130080854	TSGA14	0.64926407	152
chr7.fa	130126046	130146079	MEST	3.249483085	594
chr7.fa	130126898	130131013	MESTIT1	0	0
chr7.fa	130135952	130136045	MIR335	0	0
chr7.fa	130146132	130353485	COPG2	18.23782924	1900
chr7.fa	130353599	130371406	TSGA13	0	0
chr7.fa	130417396	130418888	KLF14	0	0
chr7.fa	130561506	130561569	MIR29A	0	0
chr7.fa	130562218	130562298	MIR29B1	0	0
chr7.fa	130565751	130598069	LOC646329	0	0
chr7.fa	130628919	130793562	FLJ43663	0.70739813	152
chr7.fa	130794855	131181398	MKLN1	13.73093355	10409
chr7.fa	131185021	131241376	PODXL	0.573810278	228
chr7.fa	131808091	132333447	PLXNA4	0.074248941	76
chr7.fa	132469623	132766828	CHCHD3	15.10792761	1595
chr7.fa	132937823	133750513	EXOC4	26.86998406	7752

chr7.fa	133812105	133948933	LRGUK	1.271720206	228
chr7.fa	133974090	134001827	SLC35B4	2.828688881	1280
chr7.fa	134127107	134143888	AKR1B1	13.09989285	1216
chr7.fa	134212344	134226166	AKR1B10	0	0
chr7.fa	134233849	134264592	AKR1B15	0	0
chr7.fa	134331531	134364567	BPGM	19.03152482	2660
chr7.fa	134464164	134655480	CALD1	19.71106077	6307
chr7.fa	134671259	134820530	AGBL3	0.971862919	228
chr7.fa	134832766	134850531	TMEM140	10.60479709	1140
chr7.fa	134850968	134855532	C7orf49	12.84265717	1216
chr7.fa	134868590	134896316	WDR91	5.203901976	1596
chr7.fa	134916731	134943244	STRA8	0	0
chr7.fa	135046547	135194875	CNOT4	12.95862408	4636
chr7.fa	135242662	135333499	NUP205	15.89484591	6612
chr7.fa	135347221	135361160	PL-5283	31.94812972	5299
chr7.fa	135365987	135412933	SLC13A4	0	0
chr7.fa	135414346	135433594	FAM180A	0	0
chr7.fa	136553399	136701771	CHRM2	0	0
chr7.fa	136587914	136588041	MIR490	0	0
chr7.fa	136912092	137028546	PTN	2.222498234	228
chr7.fa	137074385	137531609	DGKI	0.478928263	152
chr7.fa	137559725	137686846	CREB3L2	15.48082899	7663
chr7.fa	137761178	137803050	AKR1D1	0	0
chr7.fa	138145079	138270332	TRIM24	39.28040095	10177
chr7.fa	138279030	138363790	SVOPL	0	0
chr7.fa	138391039	138482941	ATP6V0A4	20.36288515	4332
chr7.fa	138482739	138490769	TMEM213	2.475215726	532
chr7.fa	138516127	138666064	KIAA1549	1.105300165	912
chr7.fa	138710452	138720775	ZC3HAV1L	3.264694329	380
chr7.fa	138728266	138794465	ZC3HAV1	13.52761495	7068
chr7.fa	138818490	138874550	TTC26	3.724947402	532
chr7.fa	138916231	138992982	UBN2	7.686648021	7372
chr7.fa	139025897	139030541	C7orf55	29.1729061	1292
chr7.fa	139044634	139108200	LUC7L2	58.52879894	9358
chr7.fa	139102209	139112272	LOC100129148	0	0
chr7.fa	139138088	139168457	KLRG2	0	0
chr7.fa	139208674	139229731	CLEC2L	0	0
chr7.fa	139246316	139477693	HIPK2	37.65656304	37865
chr7.fa	139478047	139720125	TBXAS1	0.429378767	76
chr7.fa	139723549	139763521	PARP12	10.26548093	2584
chr7.fa	139784546	139876741	JHDM1D	9.852217046	6004
chr7.fa	139877061	139879440	LOC100134229	4.328276529	684
chr7.fa	140033552	140098311	SLC37A3	2.765584811	608

chr7.fa	140103843	140126050	RAB19	1.202290669	76
chr7.fa	140152840	140179369	MKRN1	59.76000604	14364
chr7.fa	140218220	140302342	DENND2A	2.975530094	684
chr7.fa	140372953	140394908	ADCK2	8.231090056	1292
chr7.fa	140395136	140396877	LOC100134713	0	0
chr7.fa	140396621	140406446	NDUFB2	178.1822483	4200
chr7.fa	140433813	140624564	BRAF	36.42972354	7126
chr7.fa	140705961	140714781	MRPS33	61.64348932	3344
chr7.fa	140774032	141180179	LOC100131199	2.602176898	1824
chr7.fa	141251078	141354209	AGK	5.818225368	1130
chr7.fa	141356528	141401953	KIAA1147	13.49177063	6536
chr7.fa	141404138	141438030	FLJ40852	0.590979404	76
chr7.fa	141408153	141431071	WEE2	0	0
chr7.fa	141438176	141450257	SSBP1	42.95158187	1791
chr7.fa	141463897	141464997	TAS2R3	0	0
chr7.fa	141478289	141479188	TAS2R4	0	0
chr7.fa	141490017	141491166	TAS2R5	0	0
chr7.fa	141536078	141541221	PRSS37	0	0
chr7.fa	141618676	141619620	OR9A4	0	0
chr7.fa	141627157	141646783	CLEC5A	0	0
chr7.fa	141672431	141673573	TAS2R38	0	0
chr7.fa	141695679	141806547	MGAM	0.882703947	380
chr7.fa	141811549	141843783	LOC93432	0	0
chr7.fa	141870970	141921088	LOC100124692	0	0
chr7.fa	141940556	141946886	MOXD2P	0	0
chr7.fa	141951963	141957878	TRYX3	0	0
chr7.fa	141968101	141972068	LOC730441	0	0
chr7.fa	142374131	142375525	MTRNR2L6	0	0
chr7.fa	142457319	142460927	PRSS1	0	0
chr7.fa	142478757	142482399	TRY6	0	0
chr7.fa	142479908	142480066	PRSS2	0	0
chr7.fa	142552792	142568847	EPHB6	3.047218727	818
chr7.fa	142568960	142583477	TRPV6	27.29017584	5244
chr7.fa	142605648	142630820	TRPV5	0.46040368	76
chr7.fa	142636603	142637957	C7orf34	1.490400659	76
chr7.fa	142638201	142659503	KEL	0	0
chr7.fa	142723287	142724219	OR9A2	0	0
chr7.fa	142749438	142750379	OR6V1	0	0
chr7.fa	142759381	142760882	OR6W1P	0	0
chr7.fa	142829174	142836834	PIP	0	0
chr7.fa	142880512	142881528	TAS2R39	0	0
chr7.fa	142919172	142920143	TAS2R40	0	0
chr7.fa	142960522	142966222	GSTK1	73.63763019	5173

chr7.fa	142982063	142985141	TMEM139	12.59415666	1976
chr7.fa	142985402	143004785	CASP2	6.231489253	1672
chr7.fa	143013219	143049097	CLCN1	0	0
chr7.fa	143050493	143059840	FAM131B	0.515375005	152
chr7.fa	143078360	143088204	ZYX	10.92001622	1672
chr7.fa	143088207	143105985	EPHA1	0	0
chr7.fa	143104906	143220540	LOC285965	0	0
chr7.fa	143140546	143141502	TAS2R60	0	0
chr7.fa	143174966	143175889	TAS2R41	0	0
chr7.fa	143268890	143271244	CTAGE15P	0.486006763	76
chr7.fa	143318045	143427173	FAM115C	2.846309827	1140
chr7.fa	143452182	143454843	CTAGE6P	0	0
chr7.fa	143509061	143533810	LOC154761	0	0
chr7.fa	143550049	143599172	FAM115A	17.69142932	4788
chr7.fa	143632326	143633279	OR2F2	0	0
chr7.fa	143657020	143658108	OR2F1	0	0
chr7.fa	143701090	143702025	OR6B1	0	0
chr7.fa	143747495	143748430	OR2A5	0	0
chr7.fa	143771313	143772245	OR2A25	0	0
chr7.fa	143792201	143793133	OR2A12	0	0
chr7.fa	143806676	143807632	OR2A2	0	0
chr7.fa	143826206	143827138	OR2A14	0	0
chr7.fa	143880548	143966392	CTAGE4	0	0
chr7.fa	143883176	143892791	ARHGEF35	2.775976651	456
chr7.fa	143947564	143997598	OR2A9P	0	0
chr7.fa	143948549	143996613	OR2A20P	0	0
chr7.fa	143955789	143956088	OR2A7	0	0
chr7.fa	143956722	143983096	LOC728377	1.880922982	446
chr7.fa	144015218	144015235	OR2A1	0	0
chr7.fa	144052489	144077725	ARHGEF5	5.214143209	1900
chr7.fa	144094333	144100786	NOBOX	0	0
chr7.fa	144149034	144533146	TPK1	0.938578911	152
chr7.fa	145813453	148118088	CNTNAP2	0.694295376	456
chr7.fa	146991342	147254962	MIR548I4	0	0
chr7.fa	147075109	147075213	MIR548F4	0	0
chr7.fa	147592371	147797648	MIR548F3	0	0
chr7.fa	147626685	148043927	MIR548T	0	0
chr7.fa	148287657	148312952	C7orf33	0	0
chr7.fa	148395933	148498202	CUL1	40.53088565	8636
chr7.fa	148504475	148581414	EZH2	1.705165345	304
chr7.fa	148700154	148725782	PDIA4	6.591589089	1292
chr7.fa	148766733	148787869	ZNF786	5.954222922	1292
chr7.fa	148799878	148823438	ZNF425	4.979649088	1064

chr7.fa	148823508	148880118	ZNF398	22.53236994	8664
chr7.fa	148892577	148923339	ZNF282	14.76514751	3648
chr7.fa	148936742	148952700	ZNF212	5.702710281	1064
chr7.fa	148959262	148982085	ZNF783	3.339997515	988
chr7.fa	148982372	148994403	LOC155060	0	0
chr7.fa	149128454	149158053	ZNF777	7.451852687	1520
chr7.fa	149169884	149194898	ZNF746	5.727560332	1444
chr7.fa	149244245	149321881	ZNF767	5.78328469	912
chr7.fa	149412148	149431664	KRBA1	5.126339695	1368
chr7.fa	149461453	149470295	ZNF467	1.506666147	228
chr7.fa	149473131	149531053	SSPO	0.146690606	152
chr7.fa	149535509	149564568	ZNF862	2.489372725	1140
chr7.fa	149564783	149568885	LOC401431	0.471096732	76
chr7.fa	149570057	149577787	ATP6V0E2	31.51332912	4369
chr7.fa	149944301	150020758	ACTR3C	0.861920268	76
chr7.fa	150020296	150035245	LRRC61	4.894104669	608
chr7.fa	150026938	150029811	C7orf29	0.796557102	152
chr7.fa	150035407	150038763	RARRES2	1.544618953	76
chr7.fa	150065879	150071133	REPIN1	25.61784269	5700
chr7.fa	150076406	150095719	ZNF775	2.012552951	304
chr7.fa	150102840	150109558	LOC728743	1.019303927	228
chr7.fa	150130742	150145228	LOC285972	0	0
chr7.fa	150147962	150176483	GIMAP8	0	0
chr7.fa	150211945	150218161	GIMAP7	0	0
chr7.fa	150264458	150271041	GIMAP4	0	0
chr7.fa	150322464	150329680	GIMAP6	0	0
chr7.fa	150382794	150390728	GIMAP2	1.587541769	152
chr7.fa	150417495	150421368	GIMAP1	0	0
chr7.fa	150434436	150434592	GIMAP5	0	0
chr7.fa	150446824	150487837	LOC100128542	0	0
chr7.fa	150488376	150498448	TMEM176B	10.94667355	1216
chr7.fa	150497854	150502208	TMEM176A	20.95145485	1444
chr7.fa	150549573	150558379	ABP1	2.35714033	380
chr7.fa	150642049	150675014	KCNH2	0	0
chr7.fa	150688144	150711687	NOS3	0	0
chr7.fa	150709297	150721586	ATG9B	0	0
chr7.fa	150725510	150744869	ABCB8	0.733302426	228
chr7.fa	150745605	150749843	ACCN3	0	0
chr7.fa	150750899	150755052	CDK5	2.878238378	228
chr7.fa	150755299	150773614	SLC4A2	3.178396877	1216
chr7.fa	150773708	150777951	FASTK	27.19378776	3344
chr7.fa	150778172	150780620	TMUB1	6.030580353	760
chr7.fa	150783826	150841523	AGAP3	11.59157004	3191

chr7.fa	150845676	150864867	GBX1	0	0
chr7.fa	150872785	150884919	ASB10	0	0
chr7.fa	150904923	150924317	ABCF2	13.79042307	3496
chr7.fa	150929585	150935905	CHPF2	3.862902839	988
chr7.fa	150936059	150974231	SMARCD3	2.38455069	304
chr7.fa	151038858	151075532	NUB1	25.07520793	5168
chr7.fa	151078207	151107124	WDR86	0	0
chr7.fa	151106322	151110132	LOC100131176	0	0
chr7.fa	151127056	151137099	CRYGN	0	0
chr7.fa	151130575	151130725	MIR3907	0	0
chr7.fa	151163098	151217010	RHEB	19.80021974	2728
chr7.fa	151253201	151574316	PRKAG2	15.26335339	3915
chr7.fa	151653464	151717019	GALNTL5	0	0
chr7.fa	151722778	151819427	GALNT11	25.65067488	4636
chr7.fa	151832010	152133090	MLL3	25.82251675	28911
chr7.fa	152133980	152140100	FABP5P3	0	0
chr7.fa	152161209	152162630	LOC100128822	2.414822571	228
chr7.fa	152343587	152373250	XRCC2	0.372901377	76
chr7.fa	152456851	152552463	ACTR3B	4.684611205	684
chr7.fa	153584419	154685995	DPP6	0	0
chr7.fa	154720227	154741196	LOC100132707	6.864186623	608
chr7.fa	154735400	154794682	PAXIP1	13.06600642	2843
chr7.fa	154795143	154797413	LOC202781	2.520096425	380
chr7.fa	154862546	154877459	HTR5A	0	0
chr7.fa	155089486	155101945	INSIG1	19.68982527	4028
chr7.fa	155250824	155257526	EN2	0	0
chr7.fa	155293953	155326539	CNPY1	0	0
chr7.fa	155437203	155574179	RBM33	10.39153846	7124
chr7.fa	155595558	155604967	SHH	0	0
chr7.fa	156333185	156333795	NCRNA00244	0	0
chr7.fa	156431060	156433348	C7orf13	4.500419612	684
chr7.fa	156433401	156469820	RNF32	1.118854739	152
chr7.fa	156473570	156685902	LMBR1	6.440079079	2091
chr7.fa	156742417	156765876	NOM1	5.273783333	2128
chr7.fa	156797547	156803347	MNX1	0	0
chr7.fa	156931655	157062066	UBE3C	35.1123696	12079
chr7.fa	157129710	157210133	DNAJB6	18.45997364	3910
chr7.fa	157331750	158380482	PTPRN2	4.979649088	1596
chr7.fa	157367028	157367114	MIR153-2	0	0
chr7.fa	158325410	158325505	MIR595	0	0
chr7.fa	158424003	158497520	NCAPG2	1.164337863	304
chr7.fa	158523689	158622319	ESYT2	9.283677991	3672
chr7.fa	158649269	158738883	WDR60	23.852736	5974

chr7.fa	158801045	158818929	LOC154822	0	0
chr7.fa	158820866	158937649	VIPR2	0.290218479	76
chr8.fa	116086	117024	OR4F21	0	0
chr8.fa	158345	182199	RPL23AP53	0.605738829	228
chr8.fa	182319	197339	ZNF596	0.778634944	152
chr8.fa	356808	419875	FBXO25	32.35476692	5244
chr8.fa	441646	495331	C8orf42	6.679392604	836
chr8.fa	614200	681226	ERICH1	6.994762347	836
chr8.fa	1244294	1250827	LOC286083	0	0
chr8.fa	1449569	1656642	DLGAP2	0.226813196	152
chr8.fa	1711870	1734736	CLN8	2.235299775	1064
chr8.fa	1765397	1765473	MIR596	0	0
chr8.fa	1772149	1906807	ARHGEF10	3.480362654	1292
chr8.fa	1922044	1955109	KBTBD11	4.089716035	1824
chr8.fa	1993158	2093380	MYOM2	0.685258993	228
chr8.fa	2792875	4852328	CSMD1	0.479681295	456
chr8.fa	6264113	6501140	MCPH1	7.451250261	2448
chr8.fa	6357175	6420784	ANGPT2	0	0
chr8.fa	6565878	6619021	AGPAT5	2.696155274	988
chr8.fa	6666041	6693166	XKR5	0	0
chr8.fa	6728097	6735529	DEFB1	84.05326568	2584
chr8.fa	6782216	6783598	DEFA6	0	0
chr8.fa	6793345	6795786	DEFA4	0	0
chr8.fa	6825663	6826635	DEFA10P	0	0
chr8.fa	6912829	6914259	DEFA5	0	0
chr8.fa	7118141	7143880	LOC349196	0	0
chr8.fa	7144914	7147924	FAM90A5	0	0
chr8.fa	7152536	7155546	FAM90A20	0	0
chr8.fa	7159133	7212876	FAM66B	0.799268017	76
chr8.fa	7170368	7855043	DEFB109P1B	0	0
chr8.fa	7215498	7220490	ZNF705G	0	0
chr8.fa	7286416	7740105	DEFB103A	0	0
chr8.fa	7305276	7707686	SPAG11B	0	0
chr8.fa	7413660	7440175	FAM90A7	0	0
chr8.fa	7595909	7598918	FAM90A8	0	0
chr8.fa	7618852	7621862	FAM90A9	0	0
chr8.fa	7626499	7629510	FAM90A10	0	0
chr8.fa	7718168	7721319	SPAG11A	0	0
chr8.fa	7752199	7754237	DEFB4A	0	0
chr8.fa	7783859	7809935	LOC100132396	0	0
chr8.fa	7812535	7866277	FAM66E	0	0
chr8.fa	7946463	7946611	MIR548I3	0	0
chr8.fa	8086092	8102387	FLJ10661	0.93240405	152

chr8.fa	8175258	8239257	SGK223	10.38973119	3192
chr8.fa	8559666	8561617	CLDN23	3.518315459	456
chr8.fa	8641999	8751131	MFHAS1	6.752135482	2356
chr8.fa	8860314	8890849	ERI1	5.456468862	1672
chr8.fa	8993767	9008220	PPP1R3B	25.32129874	9348
chr8.fa	9413445	9639856	TNKS	19.67506585	12540
chr8.fa	9599182	9599278	MIR597	0	0
chr8.fa	9757574	9760839	LOC157627	0	0
chr8.fa	9760898	9760982	MIR124-1	0	0
chr8.fa	9911830	10286401	MSRA	16.77498955	2280
chr8.fa	10383056	10411676	PRSS55	0	0
chr8.fa	10463860	10512617	RP1L1	0	0
chr8.fa	10530147	10558103	C8orf74	0	0
chr8.fa	10581278	10588022	SOX7	0.355882857	76
chr8.fa	10622884	10697299	PINX1	5.46610767	380
chr8.fa	10682883	10682953	MIR1322	0	0
chr8.fa	10753657	11058875	XKR6	0.91372886	228
chr8.fa	10892716	10892812	MIR598	0	0
chr8.fa	11142000	11185654	MTMR9	5.719126375	2812
chr8.fa	11188495	11189695	AMAC1L2	0	0
chr8.fa	11197146	11225910	TDH	0	0
chr8.fa	11241322	11296166	C8orf12	0	0
chr8.fa	11278973	11324276	FAM167A	4.754040743	1292
chr8.fa	11351521	11422108	BLK	0	0
chr8.fa	11561717	11617509	GATA4	0	0
chr8.fa	11627172	11644854	NEIL2	17.87697637	2812
chr8.fa	11660190	11696818	FDFT1	155.4406861	21736
chr8.fa	11700034	11725646	CTSB	11.19065587	2964
chr8.fa	11831446	11832108	DEFB136	0	0
chr8.fa	11839830	11842099	DEFB135	0	0
chr8.fa	11851489	11853760	DEFB134	0	0
chr8.fa	11946847	11973025	ZNF705D	0	0
chr8.fa	11973291	12008698	FAM66D	0	0
chr8.fa	11985367	11986804	LOC392196	0	0
chr8.fa	11994677	11996269	USP17L2	0	0
chr8.fa	12039613	12051624	FAM86B1	0.469440062	76
chr8.fa	12219528	12268510	FAM66A	0	0
chr8.fa	12250793	12257874	DEFB109P1	0	0
chr8.fa	12272030	12277095	FAM90A25P	0	0
chr8.fa	12283124	12293852	FAM86B2	0	0
chr8.fa	12579406	12612992	LONRF1	9.580974969	2280
chr8.fa	12584741	12584813	MIR3926-1	0	0
chr8.fa	12623571	12668910	LOC340357	0	0

chr8.fa	12803183	12887284	C8orf79	0.343081316	228
chr8.fa	12940872	13372429	DLC1	2.130176527	1216
chr8.fa	13424352	13425797	C8orf48	0	0
chr8.fa	13947373	15095792	SGCZ	0	0
chr8.fa	14710947	14711019	MIR383	0	0
chr8.fa	15397730	15621995	TUSC3	7.933642471	836
chr8.fa	15965387	16050300	MSR1	0	0
chr8.fa	16850334	16859674	FGF20	0	0
chr8.fa	16884747	16980148	EFHA2	0.574111491	152
chr8.fa	17013836	17080241	ZDHHC2	21.13067644	5629
chr8.fa	17086740	17104387	CNOT7	33.43792795	7904
chr8.fa	17104401	17154305	VPS37A	11.56581635	2578
chr8.fa	17155534	17271040	MTMR7	0.281935129	76
chr8.fa	17354597	17428077	SLC7A2	6.266580538	3268
chr8.fa	17433942	17500642	PDGFRL	0.600919425	76
chr8.fa	17501303	17658426	MTUS1	40.03764979	20111
chr8.fa	17721900	17753047	FGL1	0	0
chr8.fa	17780366	17887457	PCM1	33.86971642	19752
chr8.fa	17913925	17942507	ASAH1	40.18885858	7904
chr8.fa	18027971	18081198	NAT1	5.157515214	1064
chr8.fa	18248755	18258723	NAT2	0.869149374	76
chr8.fa	18384813	18871196	PSD3	20.09525763	15782
chr8.fa	19171081	19253729	SH2D4A	36.26932775	8436
chr8.fa	19261672	19540261	CSGALNACT1	17.80031773	5776
chr8.fa	19674918	19709586	INTS10	43.36318909	7296
chr8.fa	19796582	19824770	LPL	1.221869497	304
chr8.fa	20002366	20040717	SLC18A1	0	0
chr8.fa	20054704	20079207	ATP6V1B2	46.86900422	9473
chr8.fa	20103676	20112803	LZTS1	0.20964407	76
chr8.fa	20831497	20852630	LOC286114	0	0
chr8.fa	21549530	21646346	GFRA2	0.343683741	76
chr8.fa	21766384	21771205	DOK2	0	0
chr8.fa	21777180	21864096	XPO7	28.23523083	9117
chr8.fa	21882354	21894408	NPM2	3.121618275	228
chr8.fa	21900428	21906319	FGF17	0	0
chr8.fa	21911081	21940036	EPB49	29.89205153	6520
chr8.fa	21946714	21961891	FAM160B2	12.26809386	3040
chr8.fa	21964383	21966932	NUDT18	3.762749601	380
chr8.fa	21971932	21988565	HR	1.659983434	608
chr8.fa	21995533	21999448	REEP4	3.37840214	380
chr8.fa	22004343	22014344	LGI3	0	0
chr8.fa	22019184	22021991	SFTPC	0	0
chr8.fa	22022653	22069840	BMP1	0	0

chr8.fa	22077216	22089851	PHYHIP	0	0
chr8.fa	22102475	22102556	MIR320A	0	0
chr8.fa	22102619	22108680	POLR3D	9.493924486	1216
chr8.fa	22132810	22213584	PIWIL2	0.558448428	152
chr8.fa	22224762	22291640	SLC39A14	1.079847688	380
chr8.fa	22298596	22398637	PPP3CC	9.395729132	1365
chr8.fa	22409251	22433008	SORBS3	33.61233013	7327
chr8.fa	22436254	22455538	PDLIM2	3.515905757	1368
chr8.fa	22457114	22461662	C8orf58	1.110721994	152
chr8.fa	22462145	22477946	KIAA1967	25.00818809	6916
chr8.fa	22477984	22526661	BIN3	5.524994762	664
chr8.fa	22497884	22499722	FLJ14107	0	0
chr8.fa	22545174	22550815	EGR3	1.270665962	380
chr8.fa	22570765	22785421	PEBP4	1.280304769	76
chr8.fa	22844930	22877647	RHOBTB2	9.14090315	3496
chr8.fa	22877711	22926700	TNFRSF10B	7.278052933	1976
chr8.fa	22960327	22974950	TNFRSF10C	0	0
chr8.fa	22993104	23021540	TNFRSF10D	2.268433177	532
chr8.fa	23048970	23082680	TNFRSF10A	2.590429601	301
chr8.fa	23082734	23088439	LOC389641	1.162078767	152
chr8.fa	23101150	23119512	CHMP7	16.11171908	3648
chr8.fa	23145612	23153792	R3HCC1	15.19392385	1596
chr8.fa	23154410	23261722	LOXL2	0.300459712	76
chr8.fa	23289572	23315244	ENTPD4	5.313995234	1055
chr8.fa	23386363	23430063	SLC25A37	18.41193021	2280
chr8.fa	23536206	23540450	NKX3-1	794.0044748	172976
chr8.fa	23559964	23563922	NKX2-6	0	0
chr8.fa	23699434	23712320	STC1	2.657148223	684
chr8.fa	24151580	24212726	ADAM28	1.421874759	304
chr8.fa	24241798	24263526	ADAMDEC1	0	0
chr8.fa	24298509	24366275	ADAM7	0	0
chr8.fa	24771274	24776606	NEFM	0	0
chr8.fa	24808469	24814131	NEFL	1.594620268	380
chr8.fa	25042287	25270619	DOCK5	16.79893596	8427
chr8.fa	25276774	25282556	GNRH1	0	0
chr8.fa	25285364	25315984	KCTD9	9.423440704	2128
chr8.fa	25316513	25365425	CDCA2	0.307688818	76
chr8.fa	25701573	25902392	EBF2	0	0
chr8.fa	26149007	26230195	PPP2R2A	17.42485604	4636
chr8.fa	26240523	26270644	BNIP3L	49.38172093	11476
chr8.fa	26362196	26371483	PNMA2	0.236150791	76
chr8.fa	26371709	26515693	DPYSL2	2.504583968	836
chr8.fa	26605667	26722922	ADRA1A	0	0

chr8.fa	27093814	27115903	STMN4	0	0
chr8.fa	27142404	27168834	TRIM35	9.947852092	2792
chr8.fa	27168999	27316903	PTK2B	11.53840599	3633
chr8.fa	27317278	27336813	CHRNA2	9.28744315	2508
chr8.fa	27348645	27402486	EPHX2	97.91477496	14277
chr8.fa	27454451	27472327	CLU	1.400488654	304
chr8.fa	27491577	27534286	SCARA3	0.597455478	152
chr8.fa	27559190	27559284	MIR3622B	0	0
chr8.fa	27590833	27630170	CCDC25	19.36782885	4681
chr8.fa	27632058	27662424	ESCO2	0	0
chr8.fa	27667138	27695349	PBK	0	0
chr8.fa	27727399	27850369	SCARA5	0	0
chr8.fa	27743556	27743633	MIR4287	0	0
chr8.fa	27879481	27941388	C8orf80	2.061349415	532
chr8.fa	27950584	28048669	ELP3	13.4839391	2882
chr8.fa	28174649	28200868	PNOC	0	0
chr8.fa	28203102	28243977	ZNF395	29.07998197	9272
chr8.fa	28285929	28347784	FBXO16	1.771883968	152
chr8.fa	28351722	28431785	FZD3	0.58118999	532
chr8.fa	28362633	28362699	MIR4288	0	0
chr8.fa	28559153	28611202	EXTL3	3.152643188	1292
chr8.fa	28625175	28747698	INTS9	6.55002173	1216
chr8.fa	28747911	28910242	HMBOX1	13.66496796	3192
chr8.fa	28924795	29120610	KIF13B	55.69137489	32415
chr8.fa	29193617	29208185	DUSP4	4.609910444	1292
chr8.fa	29578776	29605625	C8orf75	0.818696239	76
chr8.fa	29779029	29811123	LOC286135	0	0
chr8.fa	29814788	29814864	MIR3148	0	0
chr8.fa	29920631	29940649	TMEM66	66.00173652	8664
chr8.fa	29952922	29995222	LEPROTL1	1.818571943	380
chr8.fa	29989187	30002200	MBOAT4	0	0
chr8.fa	30013813	30041155	DCTN6	23.12590965	1729
chr8.fa	30241944	30429734	RBPMS	15.08669212	3467
chr8.fa	30436031	30515738	GTF2E2	14.15850504	1444
chr8.fa	30535580	30585486	GSR	65.45654145	13734
chr8.fa	30601690	30624520	UBXN8	0.795352251	76
chr8.fa	30643126	30670352	PPP2CB	56.93990172	7448
chr8.fa	30689060	30706533	TEX15	0	0
chr8.fa	30853321	30890777	PURG	0.345340411	76
chr8.fa	30891232	31031277	WRN	7.353807938	2584
chr8.fa	31497268	32622558	NRG1	0.186300082	76
chr8.fa	33228344	33330664	FUT10	3.549641585	836
chr8.fa	33342685	33358778	MAK16	7.216003108	1366

chr8.fa	33360947	33370703	C8orf41	7.04611912	684
chr8.fa	33405273	33424643	RNF122	1.22548405	152
chr8.fa	33448854	33457439	DUSP26	0.69534962	76
chr8.fa	35092975	35652181	UNC5D	0.314014286	152
chr8.fa	36641842	36793643	KCNU1	0	0
chr8.fa	37553301	37556396	ZNF703	5.265048163	760
chr8.fa	37594097	37615319	ERLIN2	4.479184113	1824
chr8.fa	37604074	37605564	LOC728024	0.767640679	76
chr8.fa	37620101	37637286	PROSC	36.86301807	6288
chr8.fa	37654401	37701397	GPR124	0	0
chr8.fa	37701505	37707431	BRF2	7.25576319	912
chr8.fa	37716465	37757015	RAB11FIP1	142.3475705	74252
chr8.fa	37791800	37797647	GOT1L1	0	0
chr8.fa	37820514	37824184	ADRB3	0	0
chr8.fa	37888020	37917883	EIF4EBP1	1.332414574	76
chr8.fa	37963011	37997228	ASH2L	22.55194877	4332
chr8.fa	38000218	38008600	STAR	0.424709969	76
chr8.fa	38020859	38034026	LSM1	31.13726501	1900
chr8.fa	38034106	38068537	BAG4	9.966828495	1444
chr8.fa	38089009	38120287	DDHD2	14.24630856	4636
chr8.fa	38120650	38126738	PPAPDC1B	10.46503437	1824
chr8.fa	38132561	38239790	WHSC1L1	27.43249886	12845
chr8.fa	38243959	38266062	LETM2	0.605889435	76
chr8.fa	38268656	38326352	FGFR1	0.36326257	152
chr8.fa	38368352	38386180	C8orf86	0	0
chr8.fa	38457693	38458775	RNF5P1	1.05695552	76
chr8.fa	38585704	38710546	TACC1	38.28398919	21068
chr8.fa	38758753	38831430	PLEKHA2	15.01530469	5624
chr8.fa	38831668	38846181	HTRA4	0	0
chr8.fa	38846327	38854041	TM2D2	4.715485511	1140
chr8.fa	38854505	38962779	ADAM9	5.027541914	1368
chr8.fa	38965050	39142436	ADAM32	0	0
chr8.fa	39172182	39260375	ADAM5P	0	0
chr8.fa	39308564	39380470	ADAM3A	0	0
chr8.fa	39442087	39587583	ADAM18	0	0
chr8.fa	39601255	39695779	ADAM2	0	0
chr8.fa	39771328	39785947	IDO1	0.729537267	76
chr8.fa	39792474	39873910	IDO2	0	0
chr8.fa	40010989	40012821	C8orf4	5.620027382	684
chr8.fa	40388111	40755343	ZMAT4	0	0
chr8.fa	41119476	41166990	SFRP1	1.281208408	380
chr8.fa	41348081	41368499	GOLGA7	24.12006232	3344
chr8.fa	41386725	41402565	GINS4	0	0

chr8.fa	41435707	41482520	AGPAT6	5.783134083	2423
chr8.fa	41503829	41504878	NKX6-3	0	0
chr8.fa	41510744	41754280	ANK1	0.123346619	76
chr8.fa	41517959	41518026	MIR486	0	0
chr8.fa	41786997	41909505	MYST3	24.77821216	15276
chr8.fa	42010464	42028701	AP3M2	11.85151664	2888
chr8.fa	42032236	42065194	PLAT	0.360702261	76
chr8.fa	42128820	42190171	IKBKB	8.572815914	2356
chr8.fa	42196030	42229313	POLB	24.2119322	2024
chr8.fa	42231586	42234674	DKK4	0	0
chr8.fa	42249346	42263415	VDAC3	49.21771059	4683
chr8.fa	42273993	42330172	SLC20A2	7.413749274	1748
chr8.fa	42396298	42408140	C8orf40	16.50871748	1596
chr8.fa	42552562	42592209	CHRNB3	0	0
chr8.fa	42607780	42623929	CHRNA6	0	0
chr8.fa	42691817	42698474	THAP1	6.866596324	988
chr8.fa	42704780	42751866	RNF170	2.297952026	836
chr8.fa	42752033	42885682	HOOK3	16.90044465	16204
chr8.fa	42911442	42940932	FNTA	29.03720976	3268
chr8.fa	42948657	42978323	SGK196	8.50489244	912
chr8.fa	42995592	43057970	HGSNAT	4.517588738	1564
chr8.fa	43147585	43218328	POTEA	0	0
chr8.fa	47752508	47767407	NCRNA00293	0	0
chr8.fa	48100930	48104439	LOC100287846	0.652125591	152
chr8.fa	48173542	48648475	KIAA0146	22.03898347	4712
chr8.fa	48649476	48650726	CEBPD	4.57481916	380
chr8.fa	48685669	48872743	PRKDC	10.49605929	9414
chr8.fa	48873494	48890068	MCM4	11.0542065	2508
chr8.fa	48920995	48974454	UBE2V2	19.5274716	1976
chr8.fa	49627474	49647870	EFCAB1	1.243104996	304
chr8.fa	49830239	49833988	SNAI2	1.091143166	152
chr8.fa	49984903	49988642	C8orf22	0	0
chr8.fa	50824597	51705427	SNTG1	0	0
chr8.fa	52232137	52722005	PXDNL	0	0
chr8.fa	52730140	52811735	PCMTD1	37.73427593	10488
chr8.fa	53023392	53322439	ST18	0	0
chr8.fa	53446597	53478021	FAM150A	0	0
chr8.fa	53535018	53627026	RB1CC1	19.513164	8638
chr8.fa	53852468	53853454	NPBWR1	0	0
chr8.fa	54138276	54164194	OPRK1	0.230879568	76
chr8.fa	54628115	54755850	ATP6V1H	40.33856132	6080
chr8.fa	54764368	54871863	RGS20	0	0
chr8.fa	54879116	54935008	TCEA1	16.38928663	3022

chr8.fa	54958938	55014577	LYPLA1	40.60633945	6940
chr8.fa	55047781	55061074	MRPL15	28.79518532	3344
chr8.fa	55370495	55373456	SOX17	0	0
chr8.fa	55528627	55543394	RP1	0	0
chr8.fa	56015017	56438710	XKR4	0	0
chr8.fa	56361757	56367881	SBF1P1	0	0
chr8.fa	56651320	56676539	TMEM68	1.013430278	152
chr8.fa	56685791	56738005	TGS1	20.78819754	5016
chr8.fa	56792386	56923940	LYN	6.945815276	1426
chr8.fa	56980739	56987140	RPS20	710.4607105	62316
chr8.fa	56986398	56986460	SNORD54	0	0
chr8.fa	57025501	57026541	MOS	0	0
chr8.fa	57073468	57123859	PLAG1	0.779990401	380
chr8.fa	57124315	57131176	CHCHD7	21.83355637	2508
chr8.fa	57212570	57233241	SDR16C5	0	0
chr8.fa	57353513	57359282	PENK	0	0
chr8.fa	57870488	57906427	IMPAD1	2.850828018	1368
chr8.fa	58192102	58197290	C8orf71	0	0
chr8.fa	58907113	59062277	FAM110B	2.358345181	684
chr8.fa	59323823	59364060	UBXN2B	10.84079727	3648
chr8.fa	59402737	59412720	CYP7A1	0	0
chr8.fa	59465728	59495419	SDCBP	36.44583842	5244
chr8.fa	59496064	59572404	NSMAF	9.843933695	2472
chr8.fa	59717977	60031767	TOX	1.108312292	304
chr8.fa	61101423	61193954	CA8	0	0
chr8.fa	61429559	61533629	RAB2A	135.5326322	10331
chr8.fa	61591339	61779465	CHD7	17.07228653	11839
chr8.fa	61878680	61880307	LOC100130298	0	0
chr8.fa	62200525	62413114	CLVS1	0.953037123	152
chr8.fa	62414205	62627199	ASPH	1.354252498	984
chr8.fa	63161501	63903628	NKAIN3	0	0
chr8.fa	63890420	63897460	LOC643763	0	0
chr8.fa	63927639	63951610	GGH	4.602982551	456
chr8.fa	63972048	63998612	TTPA	0.434951203	76
chr8.fa	64081121	64125346	YTHDF3	23.85168176	8164
chr8.fa	65285775	65291386	LOC100130155	0	0
chr8.fa	65291706	65291814	MIR124-2	0	0
chr8.fa	65486866	65489820	LOC401463	0	0
chr8.fa	65492814	65496186	BHLHE22	0	0
chr8.fa	65508529	65711348	CYP7B1	0	0
chr8.fa	66439243	66474901	LOC286186	0	0
chr8.fa	66514691	66546452	ARMC1	8.654143355	1748
chr8.fa	66556888	66622798	MTFR1	12.98317292	2356

chr8.fa	66629905	66753755	PDE7A	4.112306991	1368
chr8.fa	66933791	67012755	DNAJC5B	0	0
chr8.fa	67039278	67087718	TRIM55	0.80288257	152
chr8.fa	67088612	67090846	CRH	0	0
chr8.fa	67341263	67342968	RRS1	6.709363272	760
chr8.fa	67344718	67381044	ADHFE1	6.644150713	836
chr8.fa	67405491	67430759	C8orf46	0.651974985	150
chr8.fa	67474410	67525480	MYBL1	0	0
chr8.fa	67542488	67579452	VCPIP1	12.03962399	6536
chr8.fa	67588454	67593235	C8orf44	0.629986455	76
chr8.fa	67624891	67774257	SGK3	5.029499797	1444
chr8.fa	67679632	67680240	PTTG3P	0	0
chr8.fa	67782984	67834164	C8orf45	0	0
chr8.fa	67834284	67837777	SNHG6	0	0
chr8.fa	67834709	67834784	SNORD87	0	0
chr8.fa	67858736	67874825	TCF24	0	0
chr8.fa	67900367	67940786	LRRC67	0	0
chr8.fa	67955315	67974562	COPS5	41.51027889	4104
chr8.fa	67976603	68108849	CSPP1	16.01186706	5016
chr8.fa	68109897	68255912	ARFGEF1	35.99552536	17237
chr8.fa	68334405	68658620	CPA6	0	0
chr8.fa	68864603	69143897	PREX2	0.211451346	76
chr8.fa	69242957	69731258	C8orf34	0.426366639	76
chr8.fa	70378859	70573147	SULF1	0	0
chr8.fa	70584568	70747299	SLCO5A1	2.717993198	684
chr8.fa	70964023	70983562	PRDM14	0	0
chr8.fa	71024267	71316020	NCOA2	55.21335027	22572
chr8.fa	71485453	71520694	TRAM1	20.28502166	4174
chr8.fa	71549501	71581447	LACTB2	6.633307054	684
chr8.fa	71581600	71648177	XKR9	1.442507832	304
chr8.fa	72109668	72274467	EYA1	0	0
chr8.fa	72753777	72756731	MSC	0	0
chr8.fa	72755358	72968547	LOC100132891	0	0
chr8.fa	72933486	72987819	TRPA1	0	0
chr8.fa	73117534	73163869	LOC392232	0	0
chr8.fa	73449626	73850584	KCNB2	1.597783002	380
chr8.fa	73921097	73959987	TERF1	2.706848326	532
chr8.fa	73976778	74005507	C8orf84	0.904090052	228
chr8.fa	74153564	74171737	LOC100130301	0	0
chr8.fa	74202874	74205869	RPL7	51.96552385	2888
chr8.fa	74207265	74237516	RDH10	7.131512933	1672
chr8.fa	74332604	74659943	STAU2	9.889115607	3876
chr8.fa	74702840	74791110	UBE2W	10.20207565	2736

chr8.fa	74858634	74884346	TCEB1	48.53892767	2111
chr8.fa	74888377	74895018	TMEM70	5.255258749	760
chr8.fa	74903564	74941307	LY96	0	0
chr8.fa	75146939	75233562	JPH1	2.355634266	684
chr8.fa	75262618	75279335	GDAP1	0.88300516	228
chr8.fa	75512101	75670587	FLJ39080	0.538568387	76
chr8.fa	75617928	75617982	MIR2052	0	0
chr8.fa	75736772	75767264	PI15	2.380333711	1064
chr8.fa	75896708	75946793	CRISPLD1	0.793243762	228
chr8.fa	76452203	76479061	HNF4G	4.194688677	1140
chr8.fa	77523114	77595510	LOC100192378	0	0
chr8.fa	77593515	77779521	ZFHX4	0.737971223	684
chr8.fa	77892494	77913280	PEX2	10.75901801	3244
chr8.fa	79428336	79515485	PKIA	15.19859265	2204
chr8.fa	79578282	79631997	FAM164A	4.443038584	988
chr8.fa	79645007	79717758	IL7	0	0
chr8.fa	80523049	80578410	STMN2	0	0
chr8.fa	80676245	80680098	HEY1	1.986347442	304
chr8.fa	80831095	80942506	MRPS28	17.80860108	836
chr8.fa	80947105	81083836	TPD52	90.71609158	27039
chr8.fa	81398448	81434610	ZBTB10	14.91906722	6004
chr8.fa	81540686	81787016	ZNF704	10.26382426	9804
chr8.fa	81880046	82024303	PAG1	2.547205572	1817
chr8.fa	82192785	82197008	FABP5	0	0
chr8.fa	82352564	82359719	PMP2	0	0
chr8.fa	82370618	82373758	FABP9	0	0
chr8.fa	82390732	82395473	FABP4	0	0
chr8.fa	82437281	82443550	FABP12	0	0
chr8.fa	82569151	82598589	IMPA1	5.727861545	1368
chr8.fa	82605891	82607207	SLC10A5	0	0
chr8.fa	82613566	82633539	ZFAND1	17.98601539	2736
chr8.fa	82644688	82671748	CHMP4C	17.37078835	2128
chr8.fa	82711818	82754521	SNX16	3.311984729	760
chr8.fa	85095453	85834078	RALYL	1.421874759	304
chr8.fa	86019323	86058314	LRRCC1	4.430688862	1140
chr8.fa	86089619	86126287	E2F5	1.646880679	152
chr8.fa	86126754	86132643	C8orf59	23.76342643	912
chr8.fa	86157716	86196302	CA13	2.672208861	684
chr8.fa	86240458	86290342	CA1	0	0
chr8.fa	86351056	86361267	CA3	0	0
chr8.fa	86376131	86393721	CA2	39.14319854	4330
chr8.fa	86566828	86840171	REXO1L2P	0	0
chr8.fa	86568695	86789306	REXO1L1	0	0

chr8.fa	87060691	87081851	PSKH2	0	0
chr8.fa	87111139	87166454	ATP6V0D2	3.380661235	532
chr8.fa	87226288	87242604	SLC7A13	1.841162899	228
chr8.fa	87354994	87480178	WWP1	76.062694	20732
chr8.fa	87484578	87521009	FAM82B	10.69576334	2176
chr8.fa	87526656	87573726	CPNE3	35.56162841	11485
chr8.fa	87586163	87755903	CNGB3	0	0
chr8.fa	87878676	88394955	CNBD1	0	0
chr8.fa	88882971	88886296	DCAF4L2	0	0
chr8.fa	89049460	89339717	MMP16	0	0
chr8.fa	90769975	90803292	RIPK2	14.61198083	2508
chr8.fa	90914096	90940095	OSGIN2	8.642396058	2584
chr8.fa	90945564	90996899	NBN	24.27112051	7447
chr8.fa	91013580	91064227	DECR1	74.00300125	6147
chr8.fa	91070838	91095107	CALB1	21.64785872	3638
chr8.fa	91634223	91658133	TMEM64	11.87606547	3800
chr8.fa	91803921	91971630	NECAB1	0.249554759	76
chr8.fa	91972970	91997485	LOC100127983	8.012258997	266
chr8.fa	92006502	92053203	TMEM55A	8.097502204	1292
chr8.fa	92082424	92099323	OTUD6B	2.77642847	608
chr8.fa	92114847	92231464	LRRC69	0	0
chr8.fa	92261516	92410378	SLC26A7	2.727782612	1044
chr8.fa	92967195	93115454	RUNX1T1	0	0
chr8.fa	93895758	93978372	C8orf83	18.92369066	5168
chr8.fa	94358695	94712661	LOC642924	0.598057904	76
chr8.fa	94712773	94740671	FAM92A1	0	0
chr8.fa	94743731	94753224	RBM12B	4.204327484	1424
chr8.fa	94752339	94753047	C8orf39	0	0
chr8.fa	94767072	94831460	TMEM67	1.962702242	456
chr8.fa	94929083	94938296	PDP1	8.710921958	2660
chr8.fa	95139394	95229531	CDH17	0	0
chr8.fa	95261485	95274547	GEM	1.048672169	152
chr8.fa	95384188	95487310	RAD54B	3.387589128	684
chr8.fa	95500600	95565688	KIAA1429	21.18745504	8431
chr8.fa	95649513	95651695	LOC100288748	1.048672169	152
chr8.fa	95653364	95719694	ESRP1	42.66919493	10749
chr8.fa	95732103	95806076	DPY19L4	2.01963145	836
chr8.fa	95835534	95892452	INTS8	15.21591238	3136
chr8.fa	95892722	95907482	CCNE2	0.465825509	76
chr8.fa	95938200	95961615	TP53INP1	35.80771922	13438
chr8.fa	96037221	96070938	C8orf38	6.239471391	742
chr8.fa	96085139	96085224	MIR3150B	0	0
chr8.fa	96145949	96168913	PLEKHF2	9.265002801	1824

chr8.fa	96257141	96281462	C8orf37	0.339316156	76
chr8.fa	96959215	96960576	LOC100500773	0	0
chr8.fa	97154558	97173020	GDF6	0.309194882	76
chr8.fa	97239304	97247862	UQCRB	36.34086578	10938
chr8.fa	97251645	97273796	MTERFD1	11.3329789	1058
chr8.fa	97274167	97346774	PTDSS1	12.7510885	2120
chr8.fa	97505882	97624037	SDC2	3.941218152	912
chr8.fa	97657499	98155722	PGCP	4.829645141	608
chr8.fa	98285714	98290176	TSPYL5	20.77373933	6156
chr8.fa	98656407	98742488	MTDH	3.135022242	1596
chr8.fa	98787809	98864830	LAPTM4B	1.534377719	228
chr8.fa	98881311	99048946	MATN2	0.278621788	76
chr8.fa	99053942	99057773	RPL30	1370.309246	45948
chr8.fa	99054314	99054445	SNORA72	0	0
chr8.fa	99076750	99105838	C8orf47	10.33837441	1064
chr8.fa	99114567	99129418	HRSP12	37.2419437	2500
chr8.fa	99129521	99172069	POP1	1.611186969	504
chr8.fa	99204387	99306621	NIPAL2	3.045562057	456
chr8.fa	99439250	99443023	KCNS2	0	0
chr8.fa	99466861	99837909	STK3	13.36586371	2508
chr8.fa	99956631	99964326	OSR2	3.670578502	456
chr8.fa	100025494	100889808	VPS13B	6.378029253	6080
chr8.fa	100548864	100548958	MIR599	0	0
chr8.fa	100549014	100549089	MIR875	0	0
chr8.fa	100890223	100906242	COX6C	64.62504367	3952
chr8.fa	100973276	101118344	RGS22	0	0
chr8.fa	101145588	101158099	FBXO43	0	0
chr8.fa	101162839	101166230	POLR2K	34.10752388	2199
chr8.fa	101170263	101254130	SPAG1	6.106937783	1596
chr8.fa	101269288	101322327	RNF19A	33.36247416	10108
chr8.fa	101533000	101572012	ANKRD46	7.001991453	1292
chr8.fa	101585112	101661893	SNX31	124.270137	20257
chr8.fa	101715144	101734315	PABPC1	1000.131585	189924
chr8.fa	101930804	101965623	YWHAZ	106.5918105	25840
chr8.fa	102064282	102088479	FLJ42969	0	0
chr8.fa	102209266	102217960	ZNF706	16.00855372	3040
chr8.fa	102381121	102381823	NACAP1	0	0
chr8.fa	102504668	102681952	GRHL2	56.87273128	19746
chr8.fa	102698770	103137135	NCALD	3.316201708	988
chr8.fa	103216729	103251346	RRM2B	11.44608428	3952
chr8.fa	103265569	103424495	UBR5	33.22828388	20726
chr8.fa	103563848	103573245	ODF1	0	0
chr8.fa	103661005	103667983	KLF10	7.022172707	1596

chr8.fa	103838536	103876397	AZIN1	60.28411621	17404
chr8.fa	104033248	104085285	ATP6V1C1	15.65206844	5928
chr8.fa	104145191	104153570	C8orf56	0	0
chr8.fa	104152921	104242533	BAALC	0.401817801	76
chr8.fa	104166842	104166917	MIR3151	0	0
chr8.fa	104177737	104184368	LOC100499183	0	0
chr8.fa	104310661	104345094	FZD6	3.783382674	988
chr8.fa	104383786	104395217	CTHRC1	0	0
chr8.fa	104410866	104420012	SLC25A32	5.057361976	862
chr8.fa	104426942	104455680	DCAF13	13.62626212	1976
chr8.fa	104512976	105266656	RIMS2	1.723539323	836
chr8.fa	105352054	105368916	TM7SF4	0	0
chr8.fa	105391652	105479277	DPYS	19.4092456	2736
chr8.fa	105501459	105601252	LRP12	1.103492889	304
chr8.fa	106331147	106816767	ZFPM2	0	0
chr8.fa	107282406	107764921	OXR1	16.93207199	6080
chr8.fa	107771711	107782472	ABRA	0	0
chr8.fa	108261710	108510254	ANGPT1	1.31931182	380
chr8.fa	108911544	109095913	RSPO2	0	0
chr8.fa	109213972	109260959	EIF3E	221.0657561	22135
chr8.fa	109455853	109499136	TTC35	33.04092956	2716
chr8.fa	109795346	109799770	TMEM74	0	0
chr8.fa	110099676	110131812	TRHR	0	0
chr8.fa	110253148	110346350	NUDCD1	4.205984154	1140
chr8.fa	110346552	110358189	ENY2	7.592519038	1464
chr8.fa	110374706	110543500	PKHD1L1	0.081628654	76
chr8.fa	110551929	110577391	EBAG9	5.535386601	684
chr8.fa	110586405	110704020	SYBU	3.566660105	988
chr8.fa	110979233	110986959	KCNV1	0	0
chr8.fa	113235159	114449242	CSMD3	0	0
chr8.fa	113655722	113655812	MIR2053	0	0
chr8.fa	116420724	116681228	TRPS1	7.791168843	5168
chr8.fa	117657055	117768062	EIF3H	223.5352487	18924
chr8.fa	117778742	117786921	UTP23	5.89202249	1444
chr8.fa	117858173	117879000	RAD21	40.15602639	9316
chr8.fa	117886663	117889107	NCRNA00255	0	0
chr8.fa	117886967	117887039	MIR3610	0	0
chr8.fa	117950464	117956239	C8orf85	0	0
chr8.fa	117962512	118188953	SLC30A8	0.195788284	76
chr8.fa	118532965	118552501	MED30	1.177591224	76
chr8.fa	118811602	119124058	EXT1	3.743923805	836
chr8.fa	119201695	119634184	SAMD12	7.56420504	5244
chr8.fa	119935796	119964383	TNFRSF11B	2.439522016	380

chr8.fa	120079424	120119202	COLEC10	0.672156239	76
chr8.fa	120220610	120257914	MAL2	48.70670317	9120
chr8.fa	120428552	120436678	NOV	0	0
chr8.fa	120569319	120651106	ENPP2	0.341575252	76
chr8.fa	120743014	120845074	TAF2	15.24964821	5083
chr8.fa	120846181	120868170	DSCC1	0	0
chr8.fa	120885900	121063157	DEPTOR	14.35655242	2508
chr8.fa	121137352	121384273	COL14A1	0	0
chr8.fa	121408083	121457647	MRPL13	9.280665863	684
chr8.fa	121457666	121535875	MTBP	0	0
chr8.fa	121547985	121824309	SNTB1	10.58326038	3496
chr8.fa	122625271	122653429	HAS2	0	0
chr8.fa	122651586	122657564	HAS2-AS1	0	0
chr8.fa	123793901	123986755	ZHX2	19.95639855	5776
chr8.fa	124025568	124054648	DERL1	3.082611225	647
chr8.fa	124084920	124164392	WDR67	4.899827711	1140
chr8.fa	124194752	124222318	FAM83A	0	0
chr8.fa	124213412	124214983	LOC100131726	0	0
chr8.fa	124232231	124253617	C8orf76	7.120368061	608
chr8.fa	124260696	124286547	ZHX1	14.60716143	4864
chr8.fa	124332091	124408705	ATAD2	9.668627878	3572
chr8.fa	124428965	124454260	WDYHV1	14.20037362	1271
chr8.fa	124515358	124553446	FBXO32	15.86592948	1596
chr8.fa	124657915	124665190	KLHL38	0	0
chr8.fa	124693034	124749647	ANXA13	0	0
chr8.fa	124780882	124827690	FAM91A1	14.18937935	5016
chr8.fa	124864227	125132302	FER1L6	0.189161603	76
chr8.fa	125323159	125384940	TMEM65	1.765859713	505
chr8.fa	125463048	125465266	TRMT12	5.158268245	760
chr8.fa	125487008	125500734	RNF139	1.077437986	228
chr8.fa	125500860	125551329	TATDN1	9.657633613	656
chr8.fa	125551343	125562227	NDUFB9	41.39054683	1976
chr8.fa	125563028	125740730	MTSS1	11.88721035	3952
chr8.fa	125954250	125963337	LOC157381	0	0
chr8.fa	125985539	125991630	ZNF572	1.048521563	228
chr8.fa	126010720	126034525	SQLE	10.72227006	2128
chr8.fa	126036503	126104061	KIAA0196	21.16079771	5852
chr8.fa	126104083	126379367	NSMCE2	5.666414145	456
chr8.fa	126442563	126450644	TRIB1	27.1025203	6536
chr8.fa	127564683	127570711	FAM84B	24.22458314	8816
chr8.fa	128427857	128429441	POU5F1B	0	0
chr8.fa	128455595	128494384	LOC727677	0	0
chr8.fa	128748315	128753680	MYC	0.967495335	152

chr8.fa	128806779	129113499	PVT1	1.806372827	228
chr8.fa	128808208	128808274	MIR1204	0	0
chr8.fa	128972879	128972941	MIR1205	0	0
chr8.fa	129021144	129021202	MIR1206	0	0
chr8.fa	129061398	129061484	MIR1207	0	0
chr8.fa	129162362	129162434	MIR1208	0	0
chr8.fa	130228713	130253486	LOC728724	0	0
chr8.fa	130760442	130799134	GSDMC	0	0
chr8.fa	130853716	130952000	FAM49B	6.775931288	988
chr8.fa	131064353	131414217	ASAP1	14.69918192	5897
chr8.fa	131307601	131308779	ASAP1-IT	0	0
chr8.fa	131792547	132052835	ADCY8	0	0
chr8.fa	132916359	133025771	EFR3A	30.42836082	10700
chr8.fa	133036467	133071627	OC90	0	0
chr8.fa	133073733	133117512	HHLA1	0	0
chr8.fa	133141256	133493004	KCNQ3	1.108764112	228
chr8.fa	133572745	133573726	HPYR1	0	0
chr8.fa	133584447	133687813	LRRC6	9.405819759	988
chr8.fa	133722192	133772914	TMEM71	0	0
chr8.fa	133787604	133861052	PHF20L1	13.04703002	5624
chr8.fa	133879205	134147143	TG	0.135395129	76
chr8.fa	134048973	134115310	SLA	0	0
chr8.fa	134203312	134241571	WISP1	0	0
chr8.fa	134249414	134309547	NDRG1	417.623036	96443
chr8.fa	134467091	134584183	ST3GAL1	18.49145037	8559
chr8.fa	135490031	135725292	ZFAT	8.240427651	2553
chr8.fa	135610314	135612932	ZFAT-AS1	0	0
chr8.fa	135812763	135812850	MIR30B	0	0
chr8.fa	135817119	135817188	MIR30D	0	0
chr8.fa	136246374	136311962	LOC286094	0	0
chr8.fa	136469716	136659848	KHDRBS3	6.436916345	836
chr8.fa	139142266	139509065	FAM135B	0	0
chr8.fa	139600478	139926236	COL22A1	1.03948518	438
chr8.fa	140624804	140715299	KCNK9	0	0
chr8.fa	140742586	141468678	TRAPPC9	11.45105429	3496
chr8.fa	141521401	141527252	CHRAC1	7.32549394	1216
chr8.fa	141541264	141645646	EIF2C2	10.46503437	2432
chr8.fa	141668481	142011412	PTK2	56.94336566	17657
chr8.fa	142138720	142205900	DENND3	3.995436446	1444
chr8.fa	142220870	142238673	SLC45A4	2.373255212	532
chr8.fa	142350648	142354720	LOC731779	0	0
chr8.fa	142366587	142377365	GPR20	0	0
chr8.fa	142432007	142441620	PTP4A3	7.733787815	684

chr8.fa	142443929	142517330	FLJ43860	0	0
chr8.fa	143279717	143290364	NCRNA00051	0	0
chr8.fa	143293441	143484543	TSNARE1	2.925227566	370
chr8.fa	143545377	143626368	BAI1	0	0
chr8.fa	143692410	143695833	ARC	0	0
chr8.fa	143738874	143751401	JRK	5.523488698	3344
chr8.fa	143751726	143764145	PSCA	119.4528416	10184
chr8.fa	143781529	143785584	LY6K	0	0
chr8.fa	143808621	143818350	C8orf55	6.066876488	912
chr8.fa	143822362	143823829	SLURP1	0	0
chr8.fa	143831628	143833952	LYPD2	0	0
chr8.fa	143845756	143859639	LYNX1	0.195185858	76
chr8.fa	143866298	143868008	LY6D	0	0
chr8.fa	143916217	143928262	GML	0	0
chr8.fa	143953773	143961236	CYP11B1	0	0
chr8.fa	143991975	143999259	CYP11B2	0	0
chr8.fa	144063448	144099807	LOC100133669	0	0
chr8.fa	144099902	144103827	LY6E	10.70630578	836
chr8.fa	144120679	144135720	C8orf31	0	0
chr8.fa	144239331	144242053	LY6H	0	0
chr8.fa	144295068	144299044	GPIHBP1	0	0
chr8.fa	144329109	144344875	ZFP41	3.587293178	1140
chr8.fa	144349607	144359101	GLI4	2.55684438	228
chr8.fa	144373559	144382120	ZNF696	3.665457885	1140
chr8.fa	144391528	144417050	TOP1MT	4.199357474	532
chr8.fa	144448793	144450805	C8orf51	0	0
chr8.fa	144451025	144466390	RHPN1	2.772964524	684
chr8.fa	144510230	144512602	MAFA	0.964633813	152
chr8.fa	144519825	144623620	ZC3H3	4.191375336	912
chr8.fa	144635557	144645231	GSDMD	11.59096761	1824
chr8.fa	144648363	144654928	C8orf73	0.352117698	76
chr8.fa	144656955	144660513	NAPRT1	19.58319596	2204
chr8.fa	144661867	144679845	EEF1D	85.48342379	17936
chr8.fa	144680074	144682485	TIGD5	2.372803393	380
chr8.fa	144686083	144691784	PYCRL	3.846637351	684
chr8.fa	144694788	144699732	TSTA3	25.2304831	2275
chr8.fa	144718373	144735900	ZNF623	12.6566583	3496
chr8.fa	144766622	144777555	ZNF707	8.011204753	1216
chr8.fa	144779285	144780583	BREA2	0	0
chr8.fa	144788864	144790279	LOC100130274	0	0
chr8.fa	144798507	144804633	MAPK15	0	0
chr8.fa	144806103	144815914	FAM83H	14.8830723	5531
chr8.fa	144816310	144828507	LOC100128338	12.54596262	2280

chr8.fa	144873090	144897549	SCRIB	16.55510424	5716
chr8.fa	144895132	144895199	MIR937	0	0
chr8.fa	144898547	144911537	PUF60	38.40070913	4921
chr8.fa	144915755	144923146	NRBP2	7.556674722	1868
chr8.fa	144939912	144947434	EPPK1	8.216029419	4104
chr8.fa	144989321	145050913	PLEC	8.838033736	9648
chr8.fa	145019359	145019447	MIR661	0	0
chr8.fa	145051320	145060635	PARP10	9.435338607	2204
chr8.fa	145064226	145067583	GRINA	21.51954209	2812
chr8.fa	145086582	145102015	SPATC1	0	0
chr8.fa	145106167	145115584	OPLAH	2.853689539	760
chr8.fa	145133522	145135551	EXOSC4	10.23114268	608
chr8.fa	145137524	145141119	GPAA1	21.59635134	2964
chr8.fa	145149960	145152428	CYC1	46.35197254	3724
chr8.fa	145153536	145159140	SHARPIN	11.97546568	1444
chr8.fa	145159305	145162515	MAF1	42.56015591	5016
chr8.fa	145162629	145173218	KIAA1875	0	0
chr8.fa	145192672	145440828	C8orf30A	0.237958068	76
chr8.fa	145202919	145316843	HEATR7A	5.41505211	2098
chr8.fa	145486056	145515120	BOP1	1.929418233	304
chr8.fa	145515270	145538246	HSF1	23.31296277	3116
chr8.fa	145538386	145550567	DGAT1	1.837247134	435
chr8.fa	145554454	145559943	SCRT1	0	0
chr8.fa	145576886	145578505	C8ORFK29	0	0
chr8.fa	145579091	145582132	FBXL6	1.967822858	228
chr8.fa	145582225	145584946	GPR172A	1.771883968	228
chr8.fa	145597731	145618445	ADCK5	1.758178788	228
chr8.fa	145618454	145634733	CPSF1	8.186811783	2432
chr8.fa	145619376	145619445	MIR939	0	0
chr8.fa	145625476	145625559	MIR1234	0	0
chr8.fa	145637798	145642273	SLC39A4	5.262638461	912
chr8.fa	145649000	145653927	VPS28	68.69819301	4306
chr8.fa	145654163	145669812	TONSL	0.760562179	228
chr8.fa	145675315	145691031	CYHR1	9.813511208	1672
chr8.fa	145691738	145699114	KIFC2	2.798567607	532
chr8.fa	145699500	145701718	FOXH1	0	0
chr8.fa	145722109	145727504	PPP1R16A	13.5902672	2052
chr8.fa	145729465	145732555	GPT	3.641360865	456
chr8.fa	145734552	145736589	MFSD3	8.707307405	836
chr8.fa	145736667	145743210	RECQL4	4.198152623	1064
chr8.fa	145743363	145750559	LRRC14	7.831230138	1900
chr8.fa	145751603	145754458	C8orf82	31.33651724	3868
chr8.fa	145754563	145838888	ARHGAP39	1.780468531	608

chr8.fa	145946294	145980970	ZNF251	3.806425449	760
chr8.fa	145998501	146012725	ZNF34	1.183013053	152
chr8.fa	146015154	146017805	RPL8	1041.300138	67412
chr8.fa	146024261	146034529	ZNF517	1.470520617	228
chr8.fa	146052903	146068606	ZNF7	14.237724	2128
chr8.fa	146075551	146078932	COMMD5	6.300617578	684
chr8.fa	146102336	146126846	ZNF250	3.064839673	1292
chr8.fa	146155744	146176274	ZNF16	6.125612974	1064
chr8.fa	146198975	146225155	ZNF252	13.42429898	4676
chr8.fa	146220251	146224283	TMED10P1	1.746582098	456
chr8.fa	146228197	146231432	C8orf77	0.362961357	76
chr8.fa	146277824	146281416	C8orf33	15.74544439	2736
chr9.fa	14511	29739	WASH1	2.492384852	304
chr9.fa	34394	35864	FAM138C	0	0
chr9.fa	116234	118417	FOXD4	0.524110175	76
chr9.fa	121038	179075	CBWD1	14.90355477	1802
chr9.fa	168877	170508	LOC642313	0	0
chr9.fa	213108	215893	C9orf66	3.057008141	532
chr9.fa	271627	465259	DOCK8	29.94476376	14594
chr9.fa	504703	746103	KANK1	47.78017277	17176
chr9.fa	841690	969090	DMRT1	0	0
chr9.fa	976964	991732	DMRT3	0	0
chr9.fa	1050346	1057554	DMRT2	0.779990401	152
chr9.fa	2015342	2193623	SMARCA2	67.00884133	25610
chr9.fa	2535655	2622373	FLJ35024	1.607572416	76
chr9.fa	2621793	2654485	VLDLR	2.973873424	684
chr9.fa	2717526	2729757	KCNV2	0	0
chr9.fa	2804155	2844130	KIAA0020	18.05378825	2660
chr9.fa	3224647	3525983	RFX3	5.502855625	1444
chr9.fa	3824128	4300035	GLIS3	8.889842328	4499
chr9.fa	3898646	3901248	C9orf70	0	0
chr9.fa	4490427	4587469	SLC1A1	1.224580412	304
chr9.fa	4598316	4666674	C9orf68	0.305580329	76
chr9.fa	4662298	4665258	PPAPDC2	1.93288218	380
chr9.fa	4679566	4706594	CDC37L1	11.45963886	1292
chr9.fa	4709557	4742043	AK3	30.24944045	8968
chr9.fa	4792834	4861064	RCL1	13.19281699	1900
chr9.fa	4850297	4850375	MIR101-2	0	0
chr9.fa	4985245	5128183	JAK2	8.013313242	2812
chr9.fa	5163863	5185618	INSL6	0	0
chr9.fa	5231419	5233967	INSL4	0	0
chr9.fa	5299868	5304580	RLN2	0	0
chr9.fa	5334969	5339873	RLN1	2.367381563	152

chr9.fa	5357971	5437860	C9orf46	6.247905348	380
chr9.fa	5450503	5470567	CD274	0.310550339	76
chr9.fa	5510545	5571282	PDCD1LG2	0	0
chr9.fa	5629327	5776556	KIAA1432	6.450922737	2812
chr9.fa	5784572	5833081	ERMP1	13.0726331	4636
chr9.fa	5890909	5909822	MLANA	0	0
chr9.fa	5919008	6008003	KIAA2026	16.52407933	7904
chr9.fa	6011019	6015640	RANBP6	7.924606089	2432
chr9.fa	6215786	6257983	IL33	0	0
chr9.fa	6328349	6331900	TPD52L3	0	0
chr9.fa	6413151	6507051	UHRF2	15.80508451	3800
chr9.fa	6532464	6645692	GLDC	4.507498111	1140
chr9.fa	6720863	7175648	KDM4C	10.63913534	4709
chr9.fa	7796491	7799799	C9orf123	20.25851493	3344
chr9.fa	8314246	10612723	PTPRD	0.900927318	608
chr9.fa	12693386	12710266	TYRP1	0	0
chr9.fa	12775012	12823059	C9orf150	0.851076609	152
chr9.fa	13105703	13250365	MPDZ	11.80272017	5829
chr9.fa	13406379	13431328	FLJ41200	0	0
chr9.fa	14081842	14398982	NFIB	40.52531322	24072
chr9.fa	14611069	14693480	ZDHHC21	2.371297329	1444
chr9.fa	14719732	14722715	CER1	0	0
chr9.fa	14734664	14910993	FREM1	2.686667072	1824
chr9.fa	14993325	15019722	LOC389705	1.240092868	76
chr9.fa	15170842	15307358	TTC39B	2.889684462	684
chr9.fa	15422782	15461627	SNAPC3	11.83434751	2432
chr9.fa	15464065	15511003	PSIP1	2.552326188	665
chr9.fa	15553097	15971897	C9orf93	3.676150937	1064
chr9.fa	16409501	16870786	BNC2	1.151235109	988
chr9.fa	17135038	17503917	CNTLN	0.497001028	304
chr9.fa	17578953	17797122	SH3GL2	9.650253901	1748
chr9.fa	18474104	18910947	ADAMTSL1	0.732097575	380
chr9.fa	18573304	18573377	MIR3152	0	0
chr9.fa	18927891	19033256	FAM154A	0	0
chr9.fa	19049372	19051021	RRAGA	43.00956533	4712
chr9.fa	19053141	19102902	HAUS6	2.353977596	988
chr9.fa	19063654	19063784	SCARNA8	0	0
chr9.fa	19115759	19127573	PLIN2	39.14244551	5146
chr9.fa	19290749	19374139	DENND4C	21.45538377	10005
chr9.fa	19376254	19380235	RPS6	3367.551252	185364
chr9.fa	19408925	19452500	ACER2	52.97624322	10032
chr9.fa	19507450	19788591	SLC24A2	0	0
chr9.fa	20344968	20622514	MLLT3	6.323810959	1444

chr9.fa	20658309	20995954	KIAA1797	20.28185892	8208
chr9.fa	20716104	20716187	MIR491	0	0
chr9.fa	21006365	21031635	PTPLAD2	0	0
chr9.fa	21077104	21077943	IFNB1	0	0
chr9.fa	21140631	21142144	IFNW1	0	0
chr9.fa	21165636	21166659	IFNA21	0	0
chr9.fa	21186618	21187598	IFNA4	0	0
chr9.fa	21201468	21202204	IFNA7	0	0
chr9.fa	21206180	21207142	IFNA10	0	0
chr9.fa	21216372	21217310	IFNA16	0	0
chr9.fa	21227242	21228221	IFNA17	0	0
chr9.fa	21239201	21239978	IFNA14	0	0
chr9.fa	21277687	21278562	IFNA22P	0	0
chr9.fa	21304613	21305312	IFNA5	0	0
chr9.fa	21331018	21335429	KLHL9	21.79213962	6384
chr9.fa	21350317	21350886	IFNA6	0	0
chr9.fa	21367371	21368075	IFNA13	0	0
chr9.fa	21384254	21385396	IFNA2	0	0
chr9.fa	21409146	21410184	IFNA8	0	0
chr9.fa	21440440	21441315	IFNA1	0	0
chr9.fa	21454267	21559697	LOC554202	1.59868664	228
chr9.fa	21480839	21482312	IFNE	0	0
chr9.fa	21512114	21512184	MIR31	0	0
chr9.fa	21802635	21865969	MTAP	6.974882306	2279
chr9.fa	21967138	21967750	C9orf53	0	0
chr9.fa	21967754	21994490	CDKN2A	0.551671141	76
chr9.fa	21994790	22121093	CDKN2B-AS1	0	0
chr9.fa	22002902	22009312	CDKN2B	5.458727958	1444
chr9.fa	22446840	22452472	DMRTA1	4.474967135	684
chr9.fa	23690103	23826063	ELAVL2	0	0
chr9.fa	25676387	25678856	TUSC1	8.34133392	1368
chr9.fa	26840683	26892826	C9orf82	5.782832871	1140
chr9.fa	26903368	26947468	PLAA	8.290730179	2584
chr9.fa	26956371	27062931	IFT74	10.02451074	1672
chr9.fa	26993135	27005691	LRRC19	1.269009292	304
chr9.fa	27109147	27230172	TEK	0	0
chr9.fa	27245682	27282791	NCRNA00032	0	0
chr9.fa	27284654	27297137	C9orf11	0	0
chr9.fa	27325207	27529850	MOBKL2B	0.877131511	380
chr9.fa	27524312	27526496	IFNK	0	0
chr9.fa	27546544	27573842	C9orf72	6.017326992	1748
chr9.fa	27948528	28719303	LINGO2	0.409950545	76
chr9.fa	28863624	28863704	MIR876	0	0

chr9.fa	28888877	28888953	MIR873	0	0
chr9.fa	32384601	32450832	ACO1	26.1311092	6156
chr9.fa	32455300	32526322	DDX58	7.219918873	2280
chr9.fa	32540542	32552626	TOPORS	5.269566354	1444
chr9.fa	32551142	32553015	LOC100129250	4.028268635	268
chr9.fa	32553524	32573182	NDUFB6	13.29387386	760
chr9.fa	32629452	32635667	TAF1L	0	0
chr9.fa	32783497	32789199	TMEM215	0	0
chr9.fa	32972604	33001639	APTX	9.628415977	1519
chr9.fa	33025209	33039062	DNAJA1	141.4016119	14440
chr9.fa	33041850	33076714	SMU1	11.94489259	5624
chr9.fa	33110639	33167356	B4GALT1	19.35397306	5396
chr9.fa	33240196	33248565	SPINK4	0	0
chr9.fa	33252470	33264759	BAG1	37.24706432	9576
chr9.fa	33264877	33282067	CHMP5	51.23523355	7042
chr9.fa	33290418	33371155	NFX1	18.23331105	7448
chr9.fa	33384948	33402517	AQP7	2.733957473	228
chr9.fa	33441160	33447590	AQP3	15.70538309	1900
chr9.fa	33461351	33473941	NOL6	16.03822317	5168
chr9.fa	33504535	33511164	SUGT1P1	2.976885551	152
chr9.fa	33624223	33625532	ANXA2P2	0	0
chr9.fa	33673502	33677418	PTENP1	0.876679692	228
chr9.fa	33750464	33799229	PRSS3	0	0
chr9.fa	33817182	33920401	UBE2R2	25.16752963	7448
chr9.fa	33921691	34048947	UBAP2	25.83938466	7350
chr9.fa	33934286	33934378	SNORD121B	0	0
chr9.fa	33952762	33952852	SNORD121A	0	0
chr9.fa	34086381	34126771	DCAF12	21.78882628	5311
chr9.fa	34179003	34252377	UBAP1	21.79846509	4332
chr9.fa	34252522	34329198	KIF24	0	0
chr9.fa	34329504	34343696	NUDT2	20.51650365	1444
chr9.fa	34368907	34376894	KIAA1161	0.817190175	228
chr9.fa	34379017	34397849	C9orf24	0	0
chr9.fa	34398182	34458568	C9orf25	4.396350609	1064
chr9.fa	34458811	34520982	DNAI1	0	0
chr9.fa	34521040	34523037	ENHO	3.185324771	228
chr9.fa	34551431	34589722	CNTFR	0	0
chr9.fa	34568010	34583070	LOC415056	0	0
chr9.fa	34610492	34612101	C9orf23	13.73951812	988
chr9.fa	34613548	34620496	DCTN3	39.97349147	2195
chr9.fa	34621455	34628011	ARID3C	0	0
chr9.fa	34634719	34637768	SIGMAR1	2.764681173	304
chr9.fa	34646635	34650573	GALT	7.688304691	681

chr9.fa	34652182	34661884	IL11RA	1.29190146	152
chr9.fa	34661893	34662689	CCL27	0	0
chr9.fa	34689567	34691274	CCL19	0	0
chr9.fa	34709002	34710147	CCL21	0	0
chr9.fa	34723050	34729535	C9orf144B	0	0
chr9.fa	34830264	34838583	C9orf144	0	0
chr9.fa	34958192	34982541	KIAA1045	0	0
chr9.fa	34989725	34998428	DNAJB5	1.184067298	228
chr9.fa	35041102	35045988	C9orf131	0	0
chr9.fa	35056065	35072739	VCP	111.8210143	28652
chr9.fa	35073835	35080013	FANCG	1.305154821	228
chr9.fa	35088688	35096579	PIGO	2.519343393	684
chr9.fa	35099889	35103154	STOML2	41.03044699	3479
chr9.fa	35104118	35115893	KIAA1539	7.182116674	1444
chr9.fa	35161989	35405332	UNC13B	52.13058844	22049
chr9.fa	35406752	35483026	ATP8B5P	0	0
chr9.fa	35490007	35561826	RUSC2	3.366956055	1281
chr9.fa	35561896	35563896	FAM166B	0	0
chr9.fa	35605281	35609975	TESK1	9.332323849	1520
chr9.fa	35610039	35618424	CD72	0	0
chr9.fa	35649297	35650947	SIT1	0	0
chr9.fa	35657748	35658015	RMRP	0	0
chr9.fa	35658287	35661500	CCDC107	2.107886784	152
chr9.fa	35659341	35665278	C9orf100	0	0
chr9.fa	35673915	35681154	CA9	0	0
chr9.fa	35681990	35690053	TPM2	0.727127565	76
chr9.fa	35697334	35732316	TLN1	23.28103422	12920
chr9.fa	35732393	35736862	CREB3	41.37985377	4484
chr9.fa	35737006	35749225	GBA2	17.49262891	4028
chr9.fa	35749277	35752987	RGP1	24.64085915	2356
chr9.fa	35753265	35754274	MSMP	31.47673177	836
chr9.fa	35792406	35809728	NPR2	1.72323811	380
chr9.fa	35807782	35812259	SPAG8	0	0
chr9.fa	35812957	35815042	HINT2	43.88835351	1900
chr9.fa	35817014	35828744	C9orf128	0	0
chr9.fa	35829222	35854844	TMEM8B	9.142710426	2356
chr9.fa	35860271	35865515	LOC92973	1.309371799	456
chr9.fa	35869460	35870398	OR13J1	0	0
chr9.fa	35906189	35907138	HRCT1	0	0
chr9.fa	35909480	35911617	LOC158376	0	0
chr9.fa	35957105	35958151	OR2S2	0	0
chr9.fa	36036910	36124452	RECK	0.778333731	228
chr9.fa	36136742	36163903	GLIPR2	55.92345931	6992

chr9.fa	36169389	36171331	CCIN	0	0
chr9.fa	36190853	36212059	CLTA	133.3524543	10944
chr9.fa	36214439	36277053	GNE	20.90220657	7600
chr9.fa	36336399	36401195	RNF38	18.93950433	6992
chr9.fa	36572905	36677679	MELK	0	0
chr9.fa	36838531	37034476	PAX5	0	0
chr9.fa	37079893	37090398	LOC100506710	21.19167202	6156
chr9.fa	37120469	37358145	ZCCHC7	14.97087582	2669
chr9.fa	37422707	37436986	GRHPR	72.29105862	5928
chr9.fa	37438100	37465407	ZBTB5	11.57169	3572
chr9.fa	37485945	37503693	POLR1E	10.49530625	1292
chr9.fa	37510889	37576250	FBXO10	1.250936527	380
chr9.fa	37588412	37592636	TOMM5	53.11992169	3192
chr9.fa	37651052	37746901	FRMPD1	0.232837451	76
chr9.fa	37753802	37778969	RG9MTD3	2.495848799	380
chr9.fa	37780308	37785065	EXOSC3	3.752809581	304
chr9.fa	37800790	37867665	DCAF10	8.243891598	4332
chr9.fa	37877572	37904350	MCART1	2.425515623	760
chr9.fa	37915895	38069210	SHB	4.752534679	1900
chr9.fa	38392702	38398657	ALDH1B1	9.84122278	1976
chr9.fa	38408991	38424444	IGFBPL1	0	0
chr9.fa	38571361	38620360	ANKRD18A	0.306182755	76
chr9.fa	38621085	38623277	FAM201A	1.325486681	152
chr9.fa	39072764	39288300	CNTNAP3	0.437812724	152
chr9.fa	39443814	41609544	LOC653501	0	0
chr9.fa	39900200	39907240	FAM74A1	0	0
chr9.fa	40700291	40706537	FAM75A3	0	0
chr9.fa	40715524	40722679	FAM74A3	0	0
chr9.fa	40771402	40792112	ZNF658	0.284344831	76
chr9.fa	41321107	65503365	FAM75A5	0	0
chr9.fa	41952399	41955076	MGC21881	2.217377617	380
chr9.fa	41958802	42019584	KGFLP2	0	0
chr9.fa	42411411	67970293	ANKRD20A3	0	0
chr9.fa	42468589	43033312	FAM95B1	0	0
chr9.fa	42858152	42893137	AQP7P3	2.163761748	76
chr9.fa	43140537	43145484	LOC642929	0	0
chr9.fa	43624502	43630730	FAM75A6	0	0
chr9.fa	44990236	44991492	FAM27C	0	0
chr9.fa	45727029	45728283	FAM27A	0	0
chr9.fa	46687566	46748386	KGFLP1	0.316875807	76
chr9.fa	66494269	66503030	LOC442421	0	0
chr9.fa	67017400	69664949	LOC100133920	0	0
chr9.fa	67270215	67289492	AQP7P1	0	0

chr9.fa	67792929	67794189	FAM27B	0	0
chr9.fa	68427783	68454375	LOC642236	2.137405633	152
chr9.fa	68726541	68748372	LOC100132352	0.890083659	76
chr9.fa	69080244	69147854	PGM5P2	0	0
chr9.fa	69174214	69181041	LOC440896	0	0
chr9.fa	69199480	69202204	FOXD4L6	0	0
chr9.fa	69204538	69262593	CBWD6	0	0
chr9.fa	69381981	69425109	ANKRD20A4	0	0
chr9.fa	70175707	70178815	FOXD4L5	0	0
chr9.fa	70432000	70914932	CBWD3	0	0
chr9.fa	70917783	70920000	FOXD4L3	0	0
chr9.fa	70970109	70972757	LOC572558	0	0
chr9.fa	70971815	71145977	PGM5	2.05743365	456
chr9.fa	71151498	71155783	C9orf71	3.683380043	304
chr9.fa	71320616	71624091	PIP5K1B	1.293859343	228
chr9.fa	71394964	71398609	FAM122A	9.732033161	2356
chr9.fa	71627449	71629039	PRKACG	0	0
chr9.fa	71650479	71715094	FXN	1.383470134	684
chr9.fa	71736224	71870124	TJP2	64.59191027	22336
chr9.fa	71939488	72007370	FAM189A2	4.611567115	760
chr9.fa	72042449	72287275	APBA1	8.658059121	3785
chr9.fa	72324438	72374876	PTAR1	7.516011001	5016
chr9.fa	72435731	72521148	C9orf135	0	0
chr9.fa	72658497	72841888	MAMDC2	0	0
chr9.fa	72873878	72969789	SMC5	17.62832525	6982
chr9.fa	72999513	73029573	KLF9	11.86808334	4104
chr9.fa	73149966	73736514	TRPM3	5.29757914	2356
chr9.fa	73424891	73425000	MIR204	0	0
chr9.fa	74298282	74383800	TMEM2	32.7418253	14181
chr9.fa	74477368	74526148	FAM108B1	14.04434541	3040
chr9.fa	74526423	74588371	C9orf85	2.61121328	228
chr9.fa	74666297	74675521	C9orf57	0	0
chr9.fa	74764293	74867140	GDA	2.318735705	836
chr9.fa	74966341	74980163	ZFAND5	24.77248912	9652
chr9.fa	75136717	75451267	TMC1	1.072769189	228
chr9.fa	75515587	75567969	ALDH1A1	14.19841573	1976
chr9.fa	75766781	75785307	ANXA1	93.77641306	8711
chr9.fa	77112252	77302117	RORB	0.95273591	228
chr9.fa	77337411	77503010	TRPM6	0.795653464	456
chr9.fa	77561499	77567802	C9orf40	0	0
chr9.fa	77597873	77643310	C9orf41	6.785118277	988
chr9.fa	77676116	77703133	C9orf95	14.1362153	1361
chr9.fa	77703398	77762114	OSTF1	29.46749216	2569

chr9.fa	78118512	78338744	MIR548H3	0	0
chr9.fa	78505560	78977255	PCSK5	0.470494307	304
chr9.fa	79000433	79009444	RFK	11.83931752	2128
chr9.fa	79013515	79014954	RPSAP9	0	0
chr9.fa	79056582	79122332	GCNT1	1.362837061	608
chr9.fa	79226292	79521003	PRUNE2	7.275944444	6080
chr9.fa	79379354	79402465	PCA3	0	0
chr9.fa	79634571	79635869	FOXB2	0	0
chr9.fa	79791672	79792833	LOC100286938	0	0
chr9.fa	79792361	80032399	VPS13A	2.855195603	2354
chr9.fa	80037995	80263232	GNA14	9.649500869	1596
chr9.fa	80335191	80646219	GNAQ	31.27341317	4560
chr9.fa	80850991	80881983	CEP78	4.1621577	760
chr9.fa	80912059	80945009	PSAT1	23.54083021	3420
chr9.fa	82186878	82341656	TLE4	11.93555499	3762
chr9.fa	84198598	84303596	TLE1	20.83789765	4523
chr9.fa	84528352	84534842	FAM75D5	0	0
chr9.fa	84543343	84549913	FAM75D4	0	0
chr9.fa	84558415	84565009	FAM75D3	0	0
chr9.fa	84603687	84610171	FAM75D1	0	0
chr9.fa	85597317	85678043	RASEF	48.95384823	8968
chr9.fa	85862307	86153348	FRMD3	11.24472356	1883
chr9.fa	86238033	86259045	C9orf103	19.26150075	1238
chr9.fa	86274878	86323168	UBQLN1	49.70582584	13756
chr9.fa	86354336	86432752	GKAP1	4.888833446	608
chr9.fa	86451615	86536342	KIF27	3.691512787	1140
chr9.fa	86553227	86571663	C9orf64	12.95952772	2128
chr9.fa	86582998	86595569	HNRNPK	136.3680457	28024
chr9.fa	86584663	86584772	MIR7-1	0	0
chr9.fa	86595637	86618987	RMI1	4.586265244	1064
chr9.fa	86890765	86983413	SLC28A3	0.252265673	76
chr9.fa	87283466	87638505	NTRK2	0.271242076	304
chr9.fa	88161454	88356944	AGTPBP1	10.87890068	3192
chr9.fa	88420917	88457794	LOC389765	4.542137577	532
chr9.fa	88556057	88637217	NAA35	16.72709672	2951
chr9.fa	88641058	88715116	GOLM1	11.57590697	2508
chr9.fa	88842226	88874519	C9orf153	0	0
chr9.fa	88879463	88897490	ISCA1	9.147680437	1216
chr9.fa	88902648	88969402	ZCCHC6	27.86488976	10509
chr9.fa	89559277	89562104	GAS1	2.023697822	380
chr9.fa	89623366	89657041	LOC440173	0	0
chr9.fa	89698801	89700140	LOC494127	0	0
chr9.fa	89763559	89774641	C9orf170	0	0

chr9.fa	90112756	90323549	DAPK1	56.32648196	22208
chr9.fa	90340974	90346384	CTSL1	3.714706169	387
chr9.fa	90387830	90401799	CTSL3	0	0
chr9.fa	90459660	90462339	CTSL1P8	0	0
chr9.fa	90497772	90503814	C9orf79	0	0
chr9.fa	90532877	90538572	FAM75C1	0	0
chr9.fa	90581359	90589695	CDK20	4.839735768	760
chr9.fa	90744220	90749900	FAM75C2	0	0
chr9.fa	91003297	91093622	SPIN1	37.60174232	11320
chr9.fa	91150016	91190704	NXNL2	0	0
chr9.fa	91262094	91267075	LOC286238	0	0
chr9.fa	91360751	91360820	MIR4289	0	0
chr9.fa	91605778	91611057	C9orf47	0.248199301	76
chr9.fa	91606362	91619925	S1PR3	0.575165735	152
chr9.fa	91620686	91793682	SHC3	1.054545818	684
chr9.fa	91926113	91931618	CKS2	9.336089008	380
chr9.fa	91927140	91927221	MIR3153	0	0
chr9.fa	91933412	91974561	SECISBP2	25.75127993	5928
chr9.fa	91975706	92094611	SEMA4D	4.717895213	2128
chr9.fa	92219927	92221469	GADD45G	12.89702607	912
chr9.fa	92254698	92334674	LOC100129066	0	0
chr9.fa	92785723	92785817	MIR4290	0	0
chr9.fa	93224714	93345028	LOC340515	0	0
chr9.fa	93372114	93405108	DIRAS2	4.737775254	1292
chr9.fa	93564012	93660842	SYK	13.65698582	4816
chr9.fa	93825576	93837414	LOC100129316	0	0
chr9.fa	93976097	94124206	AUH	32.28368072	3404
chr9.fa	94171327	94186144	NFIL3	18.66509952	2584
chr9.fa	94398533	94398643	MIR3910-1	0	0
chr9.fa	94484878	94712444	ROR2	1.676248922	456
chr9.fa	94793427	94877690	SPTLC1	8.275820148	1804
chr9.fa	94895116	94900911	LOC100128076	0	0
chr9.fa	94903749	94921890	C9orf44	0	0
chr9.fa	94972625	95056038	IARS	36.51948493	11096
chr9.fa	95054740	95054742	MIR3651	0	0
chr9.fa	95054830	95054875	SNORA84	0	0
chr9.fa	95059640	95087740	NOL8	11.16851674	3188
chr9.fa	95087877	95375465	CENPP	0	0
chr9.fa	95146249	95166937	OGN	0	0
chr9.fa	95176527	95186836	OMD	0	0
chr9.fa	95218489	95244844	ASPN	0	0
chr9.fa	95255829	95298374	ECM2	0	0
chr9.fa	95377438	95432547	IPPK	7.578362039	1182

chr9.fa	95380332	95382817	LOC100128361	0	0
chr9.fa	95473645	95527083	BICD2	108.6450272	46284
chr9.fa	95571893	95600739	ANKRD19	0	0
chr9.fa	95608351	95640270	ZNF484	7.638755194	1444
chr9.fa	95709601	95798518	FGD3	35.12276144	8778
chr9.fa	95820989	95847415	SUSD3	1.923695191	152
chr9.fa	95858450	95875565	C9orf89	2.526723105	152
chr9.fa	95883771	95896570	NINJ1	17.8705003	1520
chr9.fa	95947212	96082854	WNK2	7.536945287	3420
chr9.fa	96080481	96108696	C9orf129	0.991893567	76
chr9.fa	96208782	96215874	FAM120AOS	36.69012195	6144
chr9.fa	96214173	96328397	FAM120A	56.93583534	17821
chr9.fa	96338909	96441869	PHF2	18.40274322	6536
chr9.fa	96581639	96581703	MIR4291	0	0
chr9.fa	96713909	96717608	BARX1	0	0
chr9.fa	96793076	96872136	PTPDC1	2.612116918	836
chr9.fa	96938239	96938318	MIRLET7A1	0	0
chr9.fa	96938629	96938715	MIRLET7F1	0	0
chr9.fa	96941116	96941202	MIRLET7D	0	0
chr9.fa	97021578	97065291	ZNF169	0.643691634	152
chr9.fa	97080478	97090926	FAM22F	0	0
chr9.fa	97094758	97123230	LOC100132077	0	0
chr9.fa	97136833	97223202	HIATL1	5.553007547	1216
chr9.fa	97321003	97356075	FBP2	0	0
chr9.fa	97365421	97402531	FBP1	71.37868522	8512
chr9.fa	97488951	97849500	C9orf3	2.210449724	760
chr9.fa	97572244	97572339	MIR2278	0	0
chr9.fa	97847490	97847586	MIR23B	0	0
chr9.fa	97847727	97847823	MIR27B	0	0
chr9.fa	97848296	97848376	MIR3074	0	0
chr9.fa	97861336	98079991	FANCC	3.98820734	1216
chr9.fa	98205264	98279247	PTCH1	4.070137207	2432
chr9.fa	98568370	98638259	C9orf130	2.565278336	1520
chr9.fa	98638260	98731122	C9orf102	5.33989953	1520
chr9.fa	98782014	98784037	NCRNA00092	2.660310957	304
chr9.fa	98828121	98864194	LOC158435	0	0
chr9.fa	98997589	99064434	HSD17B3	1.009363906	76
chr9.fa	99082988	99145992	SLC35D2	25.26783348	2728
chr9.fa	99148225	99180669	ZNF367	0.309194882	76
chr9.fa	99212414	99253618	HABP4	13.62626212	2432
chr9.fa	99262395	99382112	CDC14B	14.74858081	5624
chr9.fa	99403533	99417599	C9orf21	7.475046068	608
chr9.fa	99488103	99489749	LOC441455	0	0

chr9.fa	99518147	99540328	ZNF510	6.183144608	2116
chr9.fa	99579273	99616389	ZNF782	3.454910177	760
chr9.fa	99671357	99672737	LOC441454	0	0
chr9.fa	99691286	99704572	FAM22G	0	0
chr9.fa	99708327	99775862	HIATL2	2.912426024	152
chr9.fa	99794938	99801539	CTSL2	0	0
chr9.fa	99837953	99844227	LOC340508	0.50603741	76
chr9.fa	99959537	99961910	ZNF322B	0.482090997	76
chr9.fa	100054838	100059596	LOC100499484	0	0
chr9.fa	100069910	100070017	C9orf174	0	0
chr9.fa	100124534	100126332	LOC57653	0	0
chr9.fa	100153119	100158973	LOC286359	0	0
chr9.fa	100174302	100258405	TDRD7	15.51697452	3876
chr9.fa	100263462	100362361	TMOD1	0.649565283	76
chr9.fa	100364026	100395704	TSTD2	10.50524628	1748
chr9.fa	100395963	100436029	NCBP1	12.73768453	4332
chr9.fa	100437191	100459691	XPA	8.752941135	988
chr9.fa	100615537	100618997	FOXE1	0	0
chr9.fa	100666772	100684852	C9orf156	9.039996881	988
chr9.fa	100689073	100707134	HEMGN	0	0
chr9.fa	100745489	100778224	ANP32B	76.81301494	8242
chr9.fa	100818959	100845365	NANS	106.1532447	8740
chr9.fa	100831569	100881480	TRIM14	19.56798472	6308
chr9.fa	100883257	100954956	CORO2A	33.78778656	12920
chr9.fa	100961280	101018003	TBC1D2	12.7676552	2812
chr9.fa	101050364	101471479	GABBR2	2.570850772	988
chr9.fa	101494291	101558794	ANKS6	6.977141402	3311
chr9.fa	101569981	101612359	GALNT12	5.867624258	1064
chr9.fa	101706138	101833068	COL15A1	0	0
chr9.fa	101867412	101916473	TGFBR1	1.944780083	836
chr9.fa	101978707	101984246	ALG2	6.509960435	1292
chr9.fa	101984570	101992901	SEC61B	126.273503	4712
chr9.fa	102584137	102629173	NR4A3	1.002436013	380
chr9.fa	102668915	102736818	STX17	10.43867826	4788
chr9.fa	102741463	102861330	ERP44	4.629790486	1494
chr9.fa	102861511	103063426	INVS	7.080005554	1824
chr9.fa	103064357	103115259	TEX10	8.740139594	1900
chr9.fa	103189495	103214016	C9orf30	6.290526951	718
chr9.fa	103235520	103236022	TMEFF1	0	0
chr9.fa	103340336	103350173	MURC	0.436005447	76
chr9.fa	103791031	104087417	LPPR1	2.138610484	380
chr9.fa	104122699	104147287	BAAT	0	0
chr9.fa	104152249	104160919	MRPL50	26.90552717	1824

chr9.fa	104161163	104172942	ZNF189	9.70010461	2128
chr9.fa	104182842	104198062	ALDOB	1318.59418	212402
chr9.fa	104237608	104249475	C9orf125	0	0
chr9.fa	104296133	104325626	RNF20	28.76039525	7524
chr9.fa	104331634	104500862	GRIN3A	0	0
chr9.fa	104353897	104357283	PPP3R2	0	0
chr9.fa	105757593	105780770	CYLC2	0	0
chr9.fa	106856541	106903700	SMC2	3.830522469	1596
chr9.fa	107266544	107267503	OR13F1	0	0
chr9.fa	107288534	107289490	OR13C4	0	0
chr9.fa	107298051	107299094	OR13C3	0	0
chr9.fa	107331449	107332411	OR13C8	0	0
chr9.fa	107360738	107361694	OR13C5	0	0
chr9.fa	107366952	107367908	OR13C2	0	0
chr9.fa	107379529	107380485	OR13C9	0	0
chr9.fa	107456703	107457743	OR13D1	0	0
chr9.fa	107509969	107522403	NIPSNAP3A	19.62581756	2128
chr9.fa	107526451	107536291	NIPSNAP3B	0	0
chr9.fa	107536633	107540045	LOC286367	0	0
chr9.fa	107543284	107690527	ABCA1	5.09953176	3556
chr9.fa	108006929	108153682	SLC44A1	7.563452008	2280
chr9.fa	108210315	108309083	FSD1L	0.545345673	76
chr9.fa	108320411	108403399	FKTN	1.371271018	228
chr9.fa	108424738	108425385	TAL2	0	0
chr9.fa	108456825	108537444	TMEM38B	2.205479713	304
chr9.fa	109625378	109773796	ZNF462	22.77891257	12540
chr9.fa	109653505	109848716	MIR548Q	0	0
chr9.fa	110045544	110094470	RAD23B	55.75718988	15290
chr9.fa	110247133	110252047	KLF4	4.292733425	836
chr9.fa	111616869	111618275	ACTL7B	0	0
chr9.fa	111624603	111626035	ACTL7A	0	0
chr9.fa	111629800	111696608	IKBKAP	11.98148993	4864
chr9.fa	111696673	111703237	C9orf6	15.52555908	1970
chr9.fa	111704851	111775764	CTNNAL1	13.5761102	2204
chr9.fa	111777415	111882225	C9orf5	4.150711616	2204
chr9.fa	111808509	111808578	MIR32	0	0
chr9.fa	111899581	111929571	C9orf4	0	0
chr9.fa	111934254	112083021	EPB41L4B	22.01458523	11191
chr9.fa	112137974	112260593	PTPN3	21.98401214	10612
chr9.fa	112403068	112713756	PALM2	0	0
chr9.fa	112778234	112778319	PALM2-AKAP2	0	0
chr9.fa	112810878	112887876	AKAP2	0	0
chr9.fa	112961846	112970413	C9orf152	33.18325258	5852

chr9.fa	113006310	113018778	TXN	206.1054227	6952
chr9.fa	113065867	113100125	TXNDC8	0	0
chr9.fa	113127529	113342160	SVEP1	0	0
chr9.fa	113431051	113563278	MUSK	0	0
chr9.fa	113636054	113800365	LPAR1	0.361304687	76
chr9.fa	114089763	114090713	OR2K2	0	0
chr9.fa	114122973	114247025	KIAA0368	43.21755273	21209
chr9.fa	114287447	114340124	ZNF483	1.100330155	380
chr9.fa	114312002	114362135	PTGR1	141.9880731	14877
chr9.fa	114365111	114375833	C9orf29	0	0
chr9.fa	114405137	114416631	DNAJC25	1.624289723	228
chr9.fa	114423851	114431551	GNG10	72.65733332	714
chr9.fa	114448901	114545779	C9orf84	0.687216876	228
chr9.fa	114659206	114695433	UGCG	4.912478646	532
chr9.fa	114758832	115112545	MIR3134	0	0
chr9.fa	114803061	114937556	SUSD1	12.98949838	2584
chr9.fa	114980715	115095944	ROD1	39.27648518	18962
chr9.fa	115142189	115234685	HSDL2	39.36398748	8816
chr9.fa	115249248	115422706	KIAA1958	3.839558851	684
chr9.fa	115448791	115480387	C9orf80	5.274686971	532
chr9.fa	115513134	115637267	SNX30	16.70405395	8436
chr9.fa	115641200	115653193	SLC46A2	2.28740958	380
chr9.fa	115759400	115774472	ZNF883	0.962224112	152
chr9.fa	115804174	115818996	ZFP37	0.4159748	76
chr9.fa	115867003	115873957	NCRNA00256B	0	0
chr9.fa	115875176	115882126	NCRNA00256A	0	0
chr9.fa	115913238	115926422	SLC31A2	0.645197698	76
chr9.fa	115927800	115983641	FKBP15	16.08882692	4632
chr9.fa	115983808	116026772	SLC31A1	9.821945165	3116
chr9.fa	116029290	116037869	CDC26	9.209429049	532
chr9.fa	116037974	116055056	PRPF4	17.9461047	3259
chr9.fa	116059373	116061320	RNF183	1.891917247	152
chr9.fa	116077931	116102620	WDR31	7.770836983	1292
chr9.fa	116111812	116133513	BSPRY	21.26321004	3315
chr9.fa	116135698	116138341	HDHD3	18.62232731	2128
chr9.fa	116148592	116163618	ALAD	71.45172931	15101
chr9.fa	116169518	116173021	POLE3	21.80689905	3268
chr9.fa	116173030	116191879	C9orf43	0.561912374	76
chr9.fa	116207011	116360018	RGS3	1.967220433	906
chr9.fa	116638562	116818875	ZNF618	9.168915935	5548
chr9.fa	116822408	116840752	AMBP	0	0
chr9.fa	116853918	116861337	KIF12	9.143162246	1216
chr9.fa	116918231	117072975	COL27A1	1.583475397	587

chr9.fa	116971714	116971809	MIR455	0	0
chr9.fa	117085303	117088759	ORM1	0	0
chr9.fa	117092069	117095536	ORM2	4.068480537	228
chr9.fa	117096433	117156685	AKNA	3.565756467	1748
chr9.fa	117164360	117267736	DFNB31	0.550014471	152
chr9.fa	117349994	117361152	ATP6V1G1	149.2040762	15960
chr9.fa	117373706	117408703	C9orf91	7.88303873	2280
chr9.fa	117428714	117444369	LOC100505478	0	0
chr9.fa	117551611	117568408	TNFSF15	2.871611697	380
chr9.fa	117665124	117692770	TNFSF8	0	0
chr9.fa	117782805	117880486	TNC	0	0
chr9.fa	117904097	118164923	1-Dec	0	0
chr9.fa	118650544	118687377	C9orf27	0	0
chr9.fa	118916071	119164600	PAPPA	1.356360987	988
chr9.fa	119187504	120177317	ASTN2	2.379881892	1216
chr9.fa	119266562	119324572	LOC100128505	0	0
chr9.fa	119449581	119463579	TRIM32	13.54207316	3344
chr9.fa	119943345	119943480	SNORA70C	0	0
chr9.fa	120466460	120479766	TLR4	2.578381091	988
chr9.fa	121928908	122131739	DBC1	0	0
chr9.fa	123007257	123007328	MIR147	0	0
chr9.fa	123151147	123342437	CDK5RAP2	16.29109127	6739
chr9.fa	123363196	123476765	MEGF9	6.082388945	2508
chr9.fa	123519254	123555740	FBXW2	14.2047412	8664
chr9.fa	123555775	123561009	LOC100288842	0	0
chr9.fa	123578332	123605206	PSMD5	19.94450065	4484
chr9.fa	123605320	123616651	LOC253039	4.219237515	1064
chr9.fa	123617931	123639606	PHF19	1.004695108	304
chr9.fa	123664671	123691451	TRAF1	0.250157184	76
chr9.fa	123714614	123812554	C5	0.837973855	304
chr9.fa	123850574	123939886	CEP110	5.699095728	2812
chr9.fa	123940415	123964365	RAB14	36.15170417	10473
chr9.fa	124030380	124095120	GSN	27.93477112	5928
chr9.fa	124101353	124132545	STOM	1.861795972	380
chr9.fa	124217319	124262306	GGTA1	0	0
chr9.fa	124329399	124547809	DAB2IP	10.77603653	4180
chr9.fa	124584204	124855885	TTLL11	2.265270443	532
chr9.fa	124906338	124922098	NDUFA8	44.80675117	2508
chr9.fa	124922190	124962367	MORN5	1.623536692	76
chr9.fa	124964858	124991019	LHX6	0.340671614	76
chr9.fa	125001834	125023787	RBM18	16.1168397	3357
chr9.fa	125026882	125085742	MRRF	12.38089803	1520
chr9.fa	125133229	125157981	PTGS1	0.449409414	152

chr9.fa	125239237	125240205	OR1J1	0	0
chr9.fa	125273081	125274022	OR1J2	0	0
chr9.fa	125281420	125282361	OR1J4	0	0
chr9.fa	125288637	125289572	OR1N1	0	0
chr9.fa	125315449	125316441	OR1N2	0	0
chr9.fa	125329827	125330756	OR1L8	0	0
chr9.fa	125377017	125377961	OR1Q1	0	0
chr9.fa	125390858	125391814	OR1B1	0	0
chr9.fa	125423995	125424927	OR1L1	0	0
chr9.fa	125437409	125438383	OR1L3	0	0
chr9.fa	125486269	125487204	OR1L4	0	0
chr9.fa	125512127	125513062	OR1L6	0	0
chr9.fa	125551212	125552174	OR5C1	0	0
chr9.fa	125562402	125563352	OR1K1	0	0
chr9.fa	125580376	125590935	PDCL	4.823319674	988
chr9.fa	125611732	125667562	RC3H2	23.4483579	6536
chr9.fa	125642492	125642598	SNORD90	0	0
chr9.fa	125670335	125675609	ZBTB6	3.623890526	988
chr9.fa	125680378	125693779	ZBTB26	2.397051019	304
chr9.fa	125703288	125867147	RABGAP1	44.64861447	14820
chr9.fa	125796846	125797895	GPR21	0	0
chr9.fa	125871773	125877756	NCRNA00287	0.777881912	304
chr9.fa	125883908	126030843	STRBP	10.58702554	4636
chr9.fa	126118448	126141032	CRB2	0	0
chr9.fa	126141933	126692417	DENND1A	5.338544073	1901
chr9.fa	126164804	126164882	MIR601	0	0
chr9.fa	126773889	126795442	LHX2	0	0
chr9.fa	127019885	127114719	NEK6	18.24084137	3952
chr9.fa	127115744	127177721	PSMB7	99.4002056	3927
chr9.fa	127115989	127121463	LOC100129034	14.83713736	5244
chr9.fa	127213423	127239379	GPR144	0	0
chr9.fa	127243515	127269699	NR5A1	0	0
chr9.fa	127284703	127533576	NR6A1	2.426268655	304
chr9.fa	127454721	127454830	MIR181A2	0	0
chr9.fa	127455989	127456077	MIR181B2	0	0
chr9.fa	127539437	127577159	OLFML2A	0	0
chr9.fa	127615755	127620157	WDR38	0	0
chr9.fa	127620161	127624240	RPL35	581.0704086	17632
chr9.fa	127631484	127639696	ARPC5L	31.26738892	2128
chr9.fa	127640573	127703386	GOLGA1	13.36390582	4332
chr9.fa	127704888	127905838	SCAI	1.781070957	1444
chr9.fa	127908852	127952218	PPP6C	16.84020211	4864
chr9.fa	127962821	127996438	RABEPK	7.566464136	836

chr9.fa	127997127	128003666	HSPA5	19.02881391	5016
chr9.fa	128024111	128127290	GAPVD1	29.90696156	13680
chr9.fa	128199673	128469513	MAPKAP1	34.42198999	8436
chr9.fa	128509617	128729655	PBX3	16.22512568	3344
chr9.fa	129089128	129269319	FAM125B	2.542084955	1140
chr9.fa	129376722	129463311	LMX1B	0.197444954	76
chr9.fa	129567285	129600487	ZBTB43	7.091451638	2812
chr9.fa	129622944	129648156	ZBTB34	5.057663189	2204
chr9.fa	129677053	129985445	RALGPS1	5.122574535	2584
chr9.fa	129849628	129885044	ANGPTL2	0.32049036	76
chr9.fa	130026756	130155828	GARNL3	5.511741401	1368
chr9.fa	130159465	130170165	SLC2A8	4.868350979	684
chr9.fa	130186653	130207651	ZNF79	6.035851576	836
chr9.fa	130209955	130213684	RPL12	672.5732679	28179
chr9.fa	130210781	130210916	SNORA65	0	0
chr9.fa	130213765	130265780	LRSAM1	9.045117497	2072
chr9.fa	130267618	130341268	FAM129B	78.643786	20976
chr9.fa	130374486	130454995	STXBP1	1.450339364	380
chr9.fa	130452966	130453053	MIR3911	0	0
chr9.fa	130469271	130476226	C9orf117	0	0
chr9.fa	130476304	130477936	PTRH1	10.85676155	532
chr9.fa	130478358	130493802	TTC16	0	0
chr9.fa	130493880	130497604	TOR2A	1.574439014	152
chr9.fa	130500596	130541048	SH2D3C	0	0
chr9.fa	130548197	130548286	MIR2861	0	0
chr9.fa	130548305	130553052	CDK9	13.89087752	2280
chr9.fa	130565154	130576556	FPGS	12.87202541	2276
chr9.fa	130577291	130617047	ENG	0	0
chr9.fa	130628759	130640022	AK1	2.020535088	304
chr9.fa	130647601	130661871	ST6GALNAC6	12.41026628	1976
chr9.fa	130670165	130679305	ST6GALNAC4	0.676825036	76
chr9.fa	130683808	130693076	PIP5KL1	0	0
chr9.fa	130697374	130700763	DPM2	11.10556327	1140
chr9.fa	130702861	130742812	FAM102A	19.57958141	5637
chr9.fa	130823512	130829599	NAIF1	2.357893362	532
chr9.fa	130830479	130871524	SLC25A25	8.793454249	2280
chr9.fa	130873450	130881013	LOC100289019	3.277194658	836
chr9.fa	130882972	130890712	PTGES2	8.304887178	1292
chr9.fa	130890808	130892913	LOC389791	0	0
chr9.fa	130911732	130915734	LCN2	8.354737887	456
chr9.fa	130922539	130926207	C9orf16	38.52812212	1824
chr9.fa	130928344	130966662	CIZ1	23.32275218	4940
chr9.fa	130965663	131017527	DNM1	0.351967092	76

chr9.fa	131007000	131007109	MIR199B	0	0
chr9.fa	131007226	131007309	MIR3154	0	0
chr9.fa	131018108	131038268	GOLGA2	36.53800952	10335
chr9.fa	131038425	131051268	C9orf119	11.66777686	760
chr9.fa	131071396	131084697	TRUB2	18.29460784	1748
chr9.fa	131084791	131096351	COQ4	32.7654705	3420
chr9.fa	131102839	131123749	SLC27A4	1.790709765	380
chr9.fa	131133598	131153015	URM1	43.23607731	3933
chr9.fa	131154897	131154993	MIR219-2	0	0
chr9.fa	131182759	131199630	CERCAM	2.128369251	380
chr9.fa	131217434	131263571	ODF2	4.765185614	1520
chr9.fa	131266971	131304580	GLE1	14.69104918	3260
chr9.fa	131314837	131395939	SPTAN1	36.88922358	19355
chr9.fa	131395945	131419129	WDR34	19.57129806	2356
chr9.fa	131445934	131458675	SET	74.51144837	16188
chr9.fa	131464802	131483148	PKN3	0.688271121	152
chr9.fa	131483200	131486408	ZDHHC12	9.365005432	684
chr9.fa	131492067	131534198	ZER1	24.85381656	7068
chr9.fa	131549510	131572711	TBC1D13	23.60619337	6080
chr9.fa	131580779	131583127	ENDOG	12.43074874	657
chr9.fa	131584345	131592085	C9orf114	15.83008517	1584
chr9.fa	131595392	131644354	CCBL1	6.540684135	836
chr9.fa	131644391	131680317	LRRC8A	6.489779181	2052
chr9.fa	131683174	131704320	PHYHD1	11.77651466	1671
chr9.fa	131707809	131709976	DOLK	1.055901275	152
chr9.fa	131710013	131769374	NUP188	9.718930406	3648
chr9.fa	131770313	131790579	SH3GLB2	42.57822868	5609
chr9.fa	131799253	131834351	FAM73B	6.062810116	1444
chr9.fa	131843383	131852717	DOLPP1	3.156107134	456
chr9.fa	131857073	131873070	CRAT	60.184716	11449
chr9.fa	131873228	131911225	PPP2R4	58.47533368	11780
chr9.fa	131937831	131940540	IER5L	2.11180255	380
chr9.fa	132083295	132084882	C9orf106	0	0
chr9.fa	132374504	132383055	C9orf50	0	0
chr9.fa	132388435	132396585	METTL11A	6.058894351	245
chr9.fa	132396883	132404444	ASB6	5.147424587	1444
chr9.fa	132427920	132484951	PRRX2	0	0
chr9.fa	132500615	132515344	PTGES	1.921586702	228
chr9.fa	132565432	132573560	TOR1B	2.480185736	456
chr9.fa	132575221	132586441	TOR1A	8.696011927	1216
chr9.fa	132589564	132597572	C9orf78	38.83626276	4688
chr9.fa	132597696	132644107	USP20	6.680748061	1966
chr9.fa	132649466	132805473	FNBP1	12.6259346	4548

chr9.fa	132815985	132902448	GPR107	5.659938071	2660
chr9.fa	132934857	132999583	NCS1	8.660619429	2881
chr9.fa	133320094	133376661	ASS1	192.4387981	23613
chr9.fa	133452737	133454881	LOC100272217	0	0
chr9.fa	133454960	133513739	FUBP3	19.177613	4020
chr9.fa	133539981	133558384	PRDM12	0	0
chr9.fa	133569158	133580452	EXOSC2	4.695304257	684
chr9.fa	133589268	133763062	ABL1	31.48004512	12464
chr9.fa	133768815	133769225	QRFP	0	0
chr9.fa	133777825	133814455	FIBCD1	0	0
chr9.fa	133884504	133968446	LAMC3	0	0
chr9.fa	133971863	133998539	AIF1L	55.22660363	12970
chr9.fa	134000981	134109091	NUP214	16.81971964	7372
chr9.fa	134133465	134151906	FAM78A	0.290820905	76
chr9.fa	134165081	134184649	PPAPDC3	0	0
chr9.fa	134305477	134375575	PRRC2B	34.1551155	25080
chr9.fa	134378289	134399182	POMT1	1.8648081	380
chr9.fa	134399194	134406662	UCK1	25.34193181	3648
chr9.fa	134452157	134612925	RAPGEF1	18.50831828	7752
chr9.fa	134735499	134955253	MED27	8.270247713	760
chr9.fa	135037334	135118220	NTNG2	0.740682138	152
chr9.fa	135136827	135230372	SETX	22.23416932	16263
chr9.fa	135250937	135282221	TTF1	9.492719635	1976
chr9.fa	135285611	135448675	C9orf171	0	0
chr9.fa	135457993	135465640	BARHL1	0	0
chr9.fa	135469676	135545727	DDX31	5.838256016	1444
chr9.fa	135545789	135565470	GTF3C4	21.06275296	5320
chr9.fa	135600965	135754198	AK8	1.086926188	152
chr9.fa	135754290	135765418	C9orf9	6.36778802	304
chr9.fa	135766735	135820020	TSC1	13.79855581	7894
chr9.fa	135854098	135867084	GFI1B	0	0
chr9.fa	135906062	135933890	GTF3C5	13.59839995	2204
chr9.fa	135937365	135947248	CEL	0	0
chr9.fa	135957926	135962478	CELP	0	0
chr9.fa	135973107	136024588	RALGDS	9.076895442	2333
chr9.fa	136028340	136039301	GBGT1	0	0
chr9.fa	136080666	136084628	OBP2B	0	0
chr9.fa	136130563	136150630	ABO	1.449736938	152
chr9.fa	136197552	136203047	SURF6	14.82011884	2280
chr9.fa	136207755	136214972	MED22	6.503484361	1672
chr9.fa	136215069	136218280	RPL7A	545.2965758	32224
chr9.fa	136216251	136216325	SNORD24	0	0
chr9.fa	136216949	136217019	SNORD36B	0	0

chr9.fa	136217311	136217382	SNORD36A	0	0
chr9.fa	136217701	136217767	SNORD36C	0	0
chr9.fa	136218666	136223361	SURF1	22.07543021	1520
chr9.fa	136223421	136228040	SURF2	10.84938184	608
chr9.fa	136228340	136242970	SURF4	11.95106745	2356
chr9.fa	136243284	136271185	C9orf96	0	0
chr9.fa	136271221	136283164	REXO4	7.384531638	1140
chr9.fa	136279459	136324508	ADAMTS13	0.436909086	152
chr9.fa	136325087	136335909	C9orf7	4.985522737	912
chr9.fa	136336216	136344276	SLC2A6	5.844430877	988
chr9.fa	136379708	136390068	TMEM8C	0	0
chr9.fa	136397286	136440641	ADAMTSL2	0	0
chr9.fa	136443537	136445368	FAM163B	0	0
chr9.fa	136501485	136524466	DBH	0.414769949	76
chr9.fa	136528684	136605077	SARDH	4.60343437	1064
chr9.fa	136627016	136857446	VAV2	6.333449767	2040
chr9.fa	136890561	136895445	NCRNA00094	5.646082285	1672
chr9.fa	136896720	136933141	BRD3	19.68485526	5700
chr9.fa	137001210	137025094	WDR5	15.05114901	3181
chr9.fa	137029562	137029686	RNU6ATAC	0	0
chr9.fa	137218316	137332431	RXRA	36.89615147	13528
chr9.fa	137533652	137736688	COL5A1	0	0
chr9.fa	137741021	137742490	MIR3689A	0	0
chr9.fa	137741971	137742118	MIR3689B	0	0
chr9.fa	137772658	137779366	FCN2	0	0
chr9.fa	137801431	137809806	FCN1	0	0
chr9.fa	137967089	138013030	OLFM1	1.073070401	228
chr9.fa	138371648	138380739	KIAA0649	10.9076665	3572
chr9.fa	138387026	138391761	C9orf116	10.15930344	456
chr9.fa	138392483	138396519	MRPS2	17.4923277	1748
chr9.fa	138413286	138418378	LCN1	0	0
chr9.fa	138437985	138441815	OBP2A	0	0
chr9.fa	138453604	138458622	PAEP	0	0
chr9.fa	138466771	138478958	LOC100130954	0	0
chr9.fa	138515502	138531386	GLT6D1	0	0
chr9.fa	138555168	138557755	LCN9	0	0
chr9.fa	138585255	138591374	SOHLH1	0	0
chr9.fa	138594031	138684993	KCNT1	0	0
chr9.fa	138700333	138799005	CAMSAP1	11.54955086	5852
chr9.fa	138824815	138853226	UBAC1	10.42226216	1292
chr9.fa	138903203	138987131	NACC2	2.75278327	380
chr9.fa	139006427	139010731	C9orf69	7.241756797	1368
chr9.fa	139088096	139096955	LHX3	0	0

chr9.fa	139098182	139137687	QSOX2	0.505736198	152
chr9.fa	139216998	139221779	LOC26102	0	0
chr9.fa	139221932	139254057	GPSM1	0	0
chr9.fa	139256352	139258241	DNLZ	6.323810959	304
chr9.fa	139258408	139268133	CARD9	0	0
chr9.fa	139270029	139292889	SNAPC4	4.110047895	1269
chr9.fa	139296374	139305054	SDCCAG3	9.565613119	1511
chr9.fa	139305116	139318213	PMPCA	15.40823672	2128
chr9.fa	139323073	139334256	INPP5E	3.392408532	760
chr9.fa	139334548	139377507	SEC16A	33.41925276	20064
chr9.fa	139377947	139380519	C9orf163	0	0
chr9.fa	139388896	139440238	NOTCH1	0.862070874	532
chr9.fa	139557377	139567130	EGFL7	0	0
chr9.fa	139565054	139565138	MIR126	0	0
chr9.fa	139567595	139581911	AGPAT2	4.436411904	456
chr9.fa	139607024	139619045	FAM69B	3.10896734	456
chr9.fa	139619171	139622636	SNHG7	2.505638213	380
chr9.fa	139632619	139637411	LCN10	0	0
chr9.fa	139638469	139640366	LCN6	0	0
chr9.fa	139640613	139644363	LOC100128593	0	0
chr9.fa	139648840	139652731	LCN8	0	0
chr9.fa	139654086	139658965	LCN15	0	0
chr9.fa	139685777	139687769	TMEM141	225.2776139	12834
chr9.fa	139690790	139702193	KIAA1984	0.943548921	76
chr9.fa	139698379	139703300	LOC100131193	1.823541954	228
chr9.fa	139702374	139735639	C9orf86	30.99539381	7730
chr9.fa	139725409	139725475	MIR4292	0	0
chr9.fa	139738867	139741797	C9orf172	0	0
chr9.fa	139743256	139745490	PHPT1	28.36354746	2356
chr9.fa	139746819	139755251	MAMDC4	0	0
chr9.fa	139756571	139760738	EDF1	183.8185411	9337
chr9.fa	139780965	139821067	TRAF2	8.042982697	1216
chr9.fa	139834887	139839173	FBXW5	43.92946905	6840
chr9.fa	139839698	139841426	C8G	0	0
chr9.fa	139846768	139849949	LCN12	0	0
chr9.fa	139871956	139876194	PTGDS	0	0
chr9.fa	139877445	139880210	LCNL1	0	0
chr9.fa	139886870	139888428	C9orf142	12.68647836	684
chr9.fa	139889060	139891024	CLIC3	7.039341833	380
chr9.fa	139901686	139923374	ABCA2	2.052463639	1140
chr9.fa	139921916	139931234	C9orf139	0	0
chr9.fa	139924626	139926449	FUT7	0	0
chr9.fa	139933909	139940676	NPDC1	42.67838191	4330

chr9.fa	139942553	139948505	ENTPD2	0.563569045	76
chr9.fa	139956579	139965028	C9orf140	3.882029848	988
chr9.fa	139971953	139978990	UAP1L1	4.081884504	912
chr9.fa	139979398	139981269	LOC100289341	1.222923742	152
chr9.fa	139981417	140003639	MAN1B1	2.933812129	532
chr9.fa	140004992	140009195	DPP7	7.078650096	760
chr9.fa	140033609	140063211	GRIN1	1.030750011	304
chr9.fa	140063215	140064491	LRRC26	8.642245452	684
chr9.fa	140063638	140063722	MIR3621	0	0
chr9.fa	140069236	140083053	ANAPC2	10.61398408	1900
chr9.fa	140083058	140084822	SSNA1	28.09607054	1748
chr9.fa	140086069	140095163	TPRN	5.655570486	988
chr9.fa	140098535	140100090	TMEM203	13.97657255	1444
chr9.fa	140100119	140113813	NDOR1	6.10211838	1976
chr9.fa	140114707	140115775	RNF208	3.212132705	228
chr9.fa	140119644	140120763	C9orf169	3.09360549	152
chr9.fa	140122018	140124090	LOC643596	0	0
chr9.fa	140125209	140131006	SLC34A3	1.491003084	228
chr9.fa	140135711	140138036	TUBB2C	133.3297128	12996
chr9.fa	140138160	140142222	FAM166A	0	0
chr9.fa	140145730	140147934	C9orf173	0	0
chr9.fa	140149759	140168000	COBRA1	21.85735218	3724
chr9.fa	140172280	140177093	C9orf167	0.281332703	76
chr9.fa	140194083	140196703	NRARP	5.240499325	912
chr9.fa	140201348	140317714	EXD3	1.191898829	228
chr9.fa	140317847	140328815	NOXA1	4.319240146	456
chr9.fa	140328859	140335901	ENTPD8	0	0
chr9.fa	140342023	140353786	NELF	7.493269439	1818
chr9.fa	140354405	140444986	PNPLA7	2.381689169	760
chr9.fa	140446309	140447007	MRPL41	90.962333	3648
chr9.fa	140449361	140473387	WDR85	4.240171801	520
chr9.fa	140476531	140484937	ZMYND19	9.19030204	836
chr9.fa	140500096	140509783	ARRDC1	18.96736651	1976
chr9.fa	140509813	140513308	C9orf37	5.630569828	532
chr9.fa	140513444	140730578	EHMT1	17.1850907	6156
chr9.fa	140657474	140659224	FLJ40292	0	0
chr9.fa	140732871	140732968	MIR602	0	0
chr9.fa	140772241	141019076	CACNA1B	0	0
chr9.fa	141044565	141071885	TUBBP5	0.86056481	152
chr9.fa	141106637	141134172	FAM157B	0	0
chrX.fa	192991	220022	PLCXD1	0	0
chrX.fa	221426	230887	GTPBP6	0	0
chrX.fa	281385	282054	NCRNA00107	0	0

chrX.fa	294668	347690	PPP2R3B	0	0
chrX.fa	585079	620146	SHOX	0	0
chrX.fa	1314887	1331527	CRLF2	0	0
chrX.fa	1387693	1428828	CSF2RA	0	0
chrX.fa	1412811	1412885	MIR3690	0	0
chrX.fa	1455509	1501582	IL3RA	0	0
chrX.fa	1505045	1511039	SLC25A6	0	0
chrX.fa	1522032	1572655	ASMTL	0	0
chrX.fa	1532655	1534314	ASMTL-AS	0	0
chrX.fa	1581466	1656037	P2RY8	0	0
chrX.fa	1710486	1721407	AKAP17A	0	0
chrX.fa	1733941	1761974	ASMT	0	0
chrX.fa	2137555	2343345	DHRSX	0	0
chrX.fa	2404455	2418580	ZBED1	0	0
chrX.fa	2527306	2575270	CD99P1	0	0
chrX.fa	2609228	2659350	CD99	0	0
chrX.fa	2670093	2734541	XG	0	0
chrX.fa	2692781	2693037	XGPY2	0	0
chrX.fa	2746863	2800861	GYG2	2.697209518	608
chrX.fa	2822011	2847392	ARSD	4.166224072	1748
chrX.fa	2852673	2882494	ARSE	4.124656713	608
chrX.fa	2924654	2951426	ARSH	0	0
chrX.fa	2984853	3030747	ARSF	0.568840268	76
chrX.fa	3226609	3264684	MXRA5	1.40274775	912
chrX.fa	3522384	3631675	PRKX	1.854115047	749
chrX.fa	3735576	3761935	LOC389906	3.834588841	456
chrX.fa	5808083	6146706	NLGN4X	0	0
chrX.fa	6451659	6453159	VCX3A	0	0
chrX.fa	6966961	7066231	HDHD1	8.887432626	1444
chrX.fa	7137472	7272682	STS	11.30782763	4788
chrX.fa	7810303	7812184	VCX	0	0
chrX.fa	7866804	7895780	PNPLA4	6.312967301	1216
chrX.fa	8095006	8095102	MIR651	0	0
chrX.fa	8137985	8139308	VCX2	0	0
chrX.fa	8432871	8434551	VCX3B	0	0
chrX.fa	8496915	8700227	KAL1	0.362509538	152
chrX.fa	8758837	8769424	FAM9A	0	0
chrX.fa	8993037	9001116	FAM9B	0	0
chrX.fa	9431335	9687780	TBL1X	14.26483314	5700
chrX.fa	9693453	9734005	GPR143	0	0
chrX.fa	9754496	9917481	SHROOM2	9.378258792	4636
chrX.fa	9935398	9936042	LOC100288814	0	0
chrX.fa	9983795	10112518	WWC3	14.7199656	6308

chrX.fa	10124985	10205699	CLCN4	0	0
chrX.fa	10413350	10851809	MID1	7.072776448	4104
chrX.fa	11129406	11141204	HCCS	4.980853939	760
chrX.fa	11155663	11683821	ARHGAP6	19.09794223	7597
chrX.fa	11311533	11318881	AMELX	0	0
chrX.fa	11776278	11793872	MSL3	11.8436851	2128
chrX.fa	12156585	12742642	FRMPD4	0.811316527	456
chrX.fa	12809474	12842346	PRPS2	18.99387323	3192
chrX.fa	12885202	12908480	TLR7	0	0
chrX.fa	12920936	12961419	LOC349408	0	0
chrX.fa	12924739	12941288	TLR8	0	0
chrX.fa	12993225	12993287	TMSL3	0	0
chrX.fa	13053736	13062917	FAM9C	0	0
chrX.fa	13336768	13338518	ATXN3L	0	0
chrX.fa	13587694	13651694	EGFL6	0	0
chrX.fa	13671307	13682247	TCEANC	2.927336055	380
chrX.fa	13707240	13727944	RAB9A	17.64820529	1596
chrX.fa	13730361	13752754	TRAPPC2	5.202697125	988
chrX.fa	13752832	13787480	OFD1	9.858994332	2390
chrX.fa	13789062	13956831	GPM6B	0.28103149	76
chrX.fa	14024845	14048035	GEMIN8	2.065717	447
chrX.fa	14547420	14749933	GLRA2	0	0
chrX.fa	14861529	14891184	FANCB	1.522027997	304
chrX.fa	14891527	14939459	MOSPD2	2.712270156	608
chrX.fa	15262109	15288589	ASB9	0	0
chrX.fa	15301627	15333727	ASB11	0	0
chrX.fa	15337573	15353676	PIGA	2.209696692	532
chrX.fa	15363718	15402577	FIGF	0.54218294	76
chrX.fa	15402924	15511464	PIR	12.73211209	1110
chrX.fa	15518900	15574652	BMX	0	0
chrX.fa	15579156	15620192	ACE2	2.937426682	684
chrX.fa	15645441	15683154	TMEM27	10.88025614	1140
chrX.fa	15693039	15721474	CA5BP	4.606145285	304
chrX.fa	15756412	15805748	CA5B	3.578558008	988
chrX.fa	15808574	15841382	ZRSR2	14.39284856	1444
chrX.fa	15843929	15873100	AP1S2	0	0
chrX.fa	16141424	16171641	GRPR	0.426969065	76
chrX.fa	16606122	16731059	CTPS2	7.728817805	2492
chrX.fa	16668281	16672791	S100G	0	0
chrX.fa	16737707	16780807	SYAP1	24.35335159	5700
chrX.fa	16804555	16862642	TXLNG	7.550801073	2204
chrX.fa	16862775	16888534	RBBP7	33.17014982	5244
chrX.fa	16964814	17171403	REPS2	15.02840745	7936

chrX.fa	17393543	17754113	NHS	0.877432724	532
chrX.fa	17755592	17773105	SCML1	0.786466475	152
chrX.fa	17818169	17879457	RAI2	10.44726282	1900
chrX.fa	18181051	18239024	BEND2	0	0
chrX.fa	18257433	18372844	SCML2	0.272597534	76
chrX.fa	18443725	18671749	CDKL5	26.00716016	6075
chrX.fa	18657808	18690223	RS1	0	0
chrX.fa	18709045	18846034	PPEF1	0.398504461	76
chrX.fa	18908414	18913093	LOC100132163	1.190995191	76
chrX.fa	18910416	19002480	PHKA2	6.757707917	2239
chrX.fa	19007425	19140755	GPR64	0.235849579	76
chrX.fa	19362011	19379825	PDHA1	15.24347335	2584
chrX.fa	19380856	19533379	MAP3K15	1.244460453	304
chrX.fa	19552083	19905744	SH3KBP1	8.349918484	2812
chrX.fa	19930980	19988382	CXorf23	2.997217412	1292
chrX.fa	20004935	20007897	LOC729609	0	0
chrX.fa	20024831	20135114	MAP7D2	0.270338438	76
chrX.fa	20035206	20035305	MIR23C	0	0
chrX.fa	20142636	20159966	EIF1AX	10.49590868	3088
chrX.fa	20154184	20154531	SCARNA9L	0	0
chrX.fa	20168029	20284750	RPS6KA3	20.45595989	10472
chrX.fa	21392536	21672813	CNKSR2	0.322900062	152
chrX.fa	21673609	21676448	KLHL34	0	0
chrX.fa	21724091	21776230	SMPX	0	0
chrX.fa	21857656	21903541	MBTPS2	1.264792313	380
chrX.fa	21874105	21876845	YY2	0.41763147	76
chrX.fa	21958715	22012955	SMS	233.8340137	28180
chrX.fa	22050921	22266478	PHEX	0.800171655	152
chrX.fa	22291060	22292574	ZNF645	0	0
chrX.fa	23018087	23020206	DDX53	0	0
chrX.fa	23352985	23414918	PTCHD1	2.163309929	760
chrX.fa	23685645	23704514	PRDX4	3.728411348	228
chrX.fa	23721777	23761407	ACOT9	10.60434527	1216
chrX.fa	23801275	23804327	SAT1	303.5218412	23660
chrX.fa	23851465	23926057	APOO	22.30420129	1672
chrX.fa	23926123	23957624	CXorf58	0.739929106	76
chrX.fa	24001833	24045303	KLHL15	2.91799846	1216
chrX.fa	24073065	24096927	EIF2S3	73.65148598	16945
chrX.fa	24167762	24234372	ZFX	13.31285026	6916
chrX.fa	24328979	24331432	FAM48B2	0	0
chrX.fa	24380878	24383541	FAM48B1	0	0
chrX.fa	24483344	24568583	PDK3	7.667972831	6688
chrX.fa	24576204	24690979	PCYT1B	0	0

chrX.fa	24712064	25015102	POLA1	6.313268513	2280
chrX.fa	24762558	24762687	SCARNA23	0	0
chrX.fa	25021813	25034065	ARX	1.19476035	228
chrX.fa	26156460	26158853	MAGEB18	0	0
chrX.fa	26210557	26213763	MAGEB6	0	0
chrX.fa	26576454	26579169	VENTXP1	0	0
chrX.fa	27478328	27481458	SMEK3P	0	0
chrX.fa	27764926	27766938	DCAF8L2	0	0
chrX.fa	27826107	27841131	MAGEB10	0	0
chrX.fa	27996110	27999566	DCAF8L1	0	0
chrX.fa	28605681	29974017	IL1RAPL1	0	0
chrX.fa	30233675	30238206	MAGEB2	0	0
chrX.fa	30248553	30255610	MAGEB3	0	0
chrX.fa	30260057	30262308	MAGEB4	0	0
chrX.fa	30264406	30270155	MAGEB1	0	0
chrX.fa	30322539	30327495	NR0B1	0	0
chrX.fa	30576941	30596033	CXorf21	0	0
chrX.fa	30671476	30748725	GK	2.640129703	653
chrX.fa	30845559	30907511	TAB3	18.38256196	8284
chrX.fa	31089358	31090170	FTHL17	0	0
chrX.fa	31137345	33357726	DMD	0.510103782	532
chrX.fa	34147869	34150447	FAM47A	0	0
chrX.fa	34645181	34675405	TMEM47	0.564623289	152
chrX.fa	34960913	34963034	FAM47B	0	0
chrX.fa	35816459	35821852	MAGEB16	0	0
chrX.fa	35937851	36008269	CXorf22	0	0
chrX.fa	36065053	36163187	CXorf59	0	0
chrX.fa	36254051	36403434	CXorf30	0	0
chrX.fa	37026432	37029739	FAM47C	0	0
chrX.fa	37208528	37316548	PRRG1	1.668718603	675
chrX.fa	37430822	37536750	LANCL3	1.030599404	228
chrX.fa	37545133	37591383	XK	1.350637945	456
chrX.fa	37639270	37672714	CYBB	0.265067215	76
chrX.fa	37698089	37706889	DYNLT3	15.339259	2257
chrX.fa	37850070	37850570	CXorf27	0	0
chrX.fa	37865835	37988073	SYTL5	44.30282224	14364
chrX.fa	38008588	38080177	SRPX	1.202893094	152
chrX.fa	38128423	38186788	RPGR	1.201236424	456
chrX.fa	38211736	38280703	OTC	0	0
chrX.fa	38420731	38548172	TSPAN7	1.267503228	152
chrX.fa	38660685	38665783	MID1IP1	23.82698232	3952
chrX.fa	39910499	40036582	BCOR	21.70689641	9576
chrX.fa	40440216	40465888	ATP6AP2	6.146999078	831

chrX.fa	40482818	40483386	LOC347411	0	0
chrX.fa	40486173	40506819	CXorf38	7.271576859	2052
chrX.fa	40508795	40594804	MED14	16.23551752	7296
chrX.fa	40690470	40692449	LOC100132831	0	0
chrX.fa	40944888	41095832	USP9X	24.64718462	20216
chrX.fa	41192651	41209524	DDX3X	55.0958773	19740
chrX.fa	41306713	41334905	NYX	0.435403022	76
chrX.fa	41374189	41782287	CASK	3.305358049	1824
chrX.fa	41548226	41556530	GPR34	0	0
chrX.fa	41583408	41589388	GPR82	0	0
chrX.fa	42636617	42637486	PPP1R2P9	0	0
chrX.fa	43515409	43606068	MAOA	58.12005325	15718
chrX.fa	43625857	43741721	MAOB	8.276121361	1426
chrX.fa	43808024	43832921	NDP	0	0
chrX.fa	44007128	44202923	EFHC2	0.703934183	152
chrX.fa	44382885	44402221	FUNDC1	29.34896495	2280
chrX.fa	44703249	44704134	DUSP21	0	0
chrX.fa	44732423	44971845	KDM6A	15.21199662	5828
chrX.fa	45007618	45060146	CXorf36	0	0
chrX.fa	45605585	45605694	MIR221	0	0
chrX.fa	45606421	45606530	MIR222	0	0
chrX.fa	46306624	46334074	ZNF673	2.756397823	455
chrX.fa	46357160	46404892	ZNF674	1.119155951	304
chrX.fa	46404928	46407670	LOC401588	0.600919425	76
chrX.fa	46433192	46457838	CHST7	0	0
chrX.fa	46466373	46618472	SLC9A7	1.040539425	152
chrX.fa	46696347	46741791	RP2	5.081760208	1292
chrX.fa	46771736	46920641	PHF16	7.596886623	2508
chrX.fa	46937775	46952712	RGN	4.609157413	684
chrX.fa	47001615	47004609	NDUFB11	43.78835088	3268
chrX.fa	47004631	47046212	RBM10	13.85864776	3113
chrX.fa	47050199	47074527	UBA1	71.94165184	17913
chrX.fa	47064247	47065254	INE1	0	0
chrX.fa	47077528	47089394	CDK16	23.53811929	6225
chrX.fa	47092314	47107727	USP11	18.75019212	4096
chrX.fa	47229999	47273098	ZNF157	0	0
chrX.fa	47248049	47248175	SNORA11C	0	0
chrX.fa	47305561	47342345	ZNF41	7.32549394	2128
chrX.fa	47420516	47431299	ARAF	15.33022262	2502
chrX.fa	47431321	47479256	SYN1	0	0
chrX.fa	47441690	47446190	TIMP1	12.49566009	760
chrX.fa	47483612	47489704	CFP	0	0
chrX.fa	47494920	47510003	ELK1	11.77169526	2280

chrX.fa	47511195	47518231	UXT	149.6495698	4223
chrX.fa	47518252	47519776	LOC100133957	2.995861954	294
chrX.fa	47566590	47596027	CXXC1P1	0	0
chrX.fa	47696301	47781655	ZNF81	2.912877843	1520
chrX.fa	47834250	47863394	ZNF182	1.270364749	304
chrX.fa	47863734	47991997	SPACA5	0	0
chrX.fa	47917567	47931025	ZNF630	3.936097536	760
chrX.fa	47967367	47980068	SSX6	0	0
chrX.fa	48045656	48056199	SSX5	0	0
chrX.fa	48114797	48126879	SSX1	0	0
chrX.fa	48205863	48216142	SSX3	0	0
chrX.fa	48261524	48261527	SSX4	0	0
chrX.fa	48316927	48328644	SLC38A5	0	0
chrX.fa	48334549	48344752	FTSJ1	7.23377466	988
chrX.fa	48367371	48379202	PORCN	0.608148531	76
chrX.fa	48380164	48387104	EBP	11.87154728	912
chrX.fa	48398075	48420997	TBC1D25	3.603106847	912
chrX.fa	48432836	48436804	RBM3	61.98762489	6536
chrX.fa	48455880	48463582	WDR13	22.45134371	4180
chrX.fa	48542186	48549817	WAS	0	0
chrX.fa	48555131	48567406	SUV39H1	0.838877493	152
chrX.fa	48620154	48632064	GLOD5	3.106105819	152
chrX.fa	48644982	48652717	GATA1	0	0
chrX.fa	48660487	48683380	HDAC6	14.20459059	3866
chrX.fa	48687283	48688548	ERAS	0	0
chrX.fa	48689506	48693960	PCSK1N	3.529159118	228
chrX.fa	48750730	48755426	TIMM17B	20.06227483	1444
chrX.fa	48755195	48760420	PQBP1	9.041201732	760
chrX.fa	48760459	48768917	SLC35A2	2.21391367	380
chrX.fa	48770459	48776413	PIM2	0.523357143	76
chrX.fa	48779303	48815648	OTUD5	17.09472687	3496
chrX.fa	48818639	48828251	KCND1	0	0
chrX.fa	48830134	48858675	GRIPAP1	11.25451298	3300
chrX.fa	48886242	48900990	TFE3	15.49122083	3490
chrX.fa	48916516	48927510	CCDC120	6.203928287	1651
chrX.fa	48928818	48931662	PRAF2	3.625095377	304
chrX.fa	48932092	48958059	WDR45	21.24830001	2888
chrX.fa	48969022	48980079	GPKOW	4.616838338	912
chrX.fa	49019181	49023836	MAGIX	3.130504051	608
chrX.fa	49028184	49031468	PLP2	19.65925218	1368
chrX.fa	49031906	49042776	PRICKLE3	2.300662941	304
chrX.fa	49044265	49056661	SYP	0	0
chrX.fa	49061523	49089833	CACNA1F	0	0

chrX.fa	49091927	49106896	CCDC22	8.733512913	1292
chrX.fa	49106988	49121288	FOXP3	0.999273279	152
chrX.fa	49126306	49144555	PPP1R3F	0	0
chrX.fa	49160125	49176311	GAGE10	0	0
chrX.fa	49178516	49185866	GAGE12J	0	0
chrX.fa	49188094	49188103	GAGE13	0	0
chrX.fa	49197563	49226170	GAGE2D	0	0
chrX.fa	49197596	49223953	GAGE2C	0	0
chrX.fa	49204807	49301555	GAGE2E	0	0
chrX.fa	49216634	49361430	GAGE2A	0	0
chrX.fa	49216649	49216656	GAGE4	0	0
chrX.fa	49216678	49216722	GAGE7	0	0
chrX.fa	49235708	49242997	GAGE2B	0	0
chrX.fa	49296769	49351917	GAGE12D	0	0
chrX.fa	49296815	49315999	GAGE12B	0	0
chrX.fa	49325480	49335064	GAGE6	0	0
chrX.fa	49332808	49332811	GAGE12G	0	0
chrX.fa	49342357	49342360	GAGE12E	0	0
chrX.fa	49363616	49373139	GAGE1	0	0
chrX.fa	49452054	49460596	PAGE1	0	0
chrX.fa	49593906	49598570	PAGE4	0	0
chrX.fa	49641327	49643959	LOC158572	0.999725098	152
chrX.fa	49644470	49647168	USP27X	1.696279569	304
chrX.fa	49687225	49863892	CLCN5	1.44341147	988
chrX.fa	49767754	49767844	MIR532	0	0
chrX.fa	49768109	49768194	MIR188	0	0
chrX.fa	49773039	49773122	MIR500A	0	0
chrX.fa	49773572	49773635	MIR362	0	0
chrX.fa	49774330	49774413	MIR501	0	0
chrX.fa	49775280	49775358	MIR500B	0	0
chrX.fa	49777849	49777945	MIR660	0	0
chrX.fa	49779206	49779291	MIR502	0	0
chrX.fa	49955411	49965664	AKAP4	0	0
chrX.fa	50027540	50094911	CCNB3	0	0
chrX.fa	50111897	50213737	DGKK	0	0
chrX.fa	50334643	50557044	SHROOM4	1.90547182	760
chrX.fa	50648432	50648882	LOC347376	0	0
chrX.fa	50653735	50659641	BMP15	0	0
chrX.fa	51075083	51080377	NUDT10	0	0
chrX.fa	51232863	51239459	NUDT11	10.50976447	1672
chrX.fa	51486481	51489326	GSPT2	8.445854743	1596
chrX.fa	51546155	51645450	MAGED1	53.93650944	10640
chrX.fa	51811270	51934969	MAGED4B	0	0

chrX.fa	52112078	52387096	XAGE2	0	0
chrX.fa	52240600	52240615	XAGE1B	0	0
chrX.fa	52260211	52260363	XAGE1A	0	0
chrX.fa	52513551	52513566	XAGE1D	0	0
chrX.fa	52651985	52662998	SSX8	0	0
chrX.fa	52673111	52683950	SSX7	0	0
chrX.fa	52727836	52788723	SSX2	0	0
chrX.fa	52825186	52826388	SPANXN5	0	0
chrX.fa	52841228	52847322	XAGE5	0	0
chrX.fa	52891558	52897119	XAGE3	0	0
chrX.fa	52927862	52986176	FAM156A	0	0
chrX.fa	53078506	53109796	GPR173	0.269284193	76
chrX.fa	53111542	53117728	TSPYL2	7.285282039	1368
chrX.fa	53220503	53254604	KDM5C	16.27166305	6879
chrX.fa	53262058	53350522	IQSEC2	3.450090773	1596
chrX.fa	53401070	53449618	SMC1A	6.002567568	3876
chrX.fa	53449839	53458057	RIBC1	1.562541111	152
chrX.fa	53458206	53461323	HSD17B10	40.41190662	2584
chrX.fa	53559063	53713673	HUWE1	58.80335436	57528
chrX.fa	53583184	53583302	MIR98	0	0
chrX.fa	53584153	53584235	MIRLET7F2	0	0
chrX.fa	53963113	54071569	PHF8	15.98520973	7676
chrX.fa	54094757	54209714	FAM120C	1.762847586	988
chrX.fa	54219256	54384438	WNK3	5.953620497	4484
chrX.fa	54466853	54471731	TSR2	34.52816748	3040
chrX.fa	54471887	54522599	FGD1	2.141923824	608
chrX.fa	54556644	54593720	GNL3L	11.44698792	6516
chrX.fa	54775332	54824673	ITIH5L	0	0
chrX.fa	54834171	54842445	MAGED2	85.12648669	12938
chrX.fa	54840803	54840933	SNORA11	0	0
chrX.fa	54947249	54957865	TRO	1.478803968	228
chrX.fa	54959590	55020511	PFKFB1	0	0
chrX.fa	55026780	55034212	APEX2	11.00028942	1444
chrX.fa	55035488	55057497	ALAS2	0	0
chrX.fa	55101504	55105336	PAGE2B	0	0
chrX.fa	55115497	55119260	PAGE2	0	0
chrX.fa	55169535	55187628	FAM104B	7.540258627	1140
chrX.fa	55207824	55208944	MTRNR2L10	0	0
chrX.fa	55246791	55250541	PAGE5	0	0
chrX.fa	55284849	55291165	PAGE3	0	0
chrX.fa	55478538	55479999	MAGEH1	0	0
chrX.fa	55511049	55515631	USP51	1.039786393	304
chrX.fa	55649833	55652621	FOXR2	0	0

chrX.fa	55744110	55785207	RRAGB	7.779421546	1140
chrX.fa	56258822	56314322	KLF8	5.48975287	1976
chrX.fa	56590026	56593443	UBQLN2	27.12496065	6156
chrX.fa	56755718	56844004	LOC550643	61.0237441	2812
chrX.fa	56763221	56764017	LOC442454	0	0
chrX.fa	57002803	57021988	SPIN3	3.076888183	1140
chrX.fa	57146115	57147979	SPIN2B	4.54575213	380
chrX.fa	57162083	57164058	SPIN2A	0	0
chrX.fa	57313110	57515629	FAAH2	3.413342818	456
chrX.fa	57618269	57623910	ZXDB	2.231534616	836
chrX.fa	57931864	57937067	ZXDA	2.419491368	836
chrX.fa	62567107	62571218	SPIN4	2.78350697	760
chrX.fa	62646439	62780873	LOC92249	19.43379444	3724
chrX.fa	62854848	63005426	ARHGEF9	5.254957536	2052
chrX.fa	63005882	63005967	MIR1468	0	0
chrX.fa	63404997	63425624	FAM123B	3.660337268	2052
chrX.fa	63444076	63445503	ASB12	0	0
chrX.fa	63487961	63615333	MTMR8	3.441204997	608
chrX.fa	64136250	64254593	ZC4H2	2.94872216	456
chrX.fa	64708615	64727767	ZC3H12B	1.261930792	608
chrX.fa	64732462	64754686	LAS1L	11.03673616	1824
chrX.fa	64770502	64772301	FKSG43	0	0
chrX.fa	64887511	64961793	MSN	9.22599575	2432
chrX.fa	65238712	65238821	MIR223	0	0
chrX.fa	65241580	65259967	VSIG4	0	0
chrX.fa	65382433	65487230	HEPH	0	0
chrX.fa	65815482	65859108	EDA2R	0.655890751	152
chrX.fa	66763874	66944119	AR	19.09372525	5700
chrX.fa	67262186	67653299	OPHN1	13.90879968	6955
chrX.fa	67718624	67757127	YIPF6	2.915287545	1216
chrX.fa	67867511	67945684	STARD8	2.006980515	684
chrX.fa	68048840	68062006	EFNB1	0.687518089	152
chrX.fa	68380581	68385365	PJA1	6.358902244	1216
chrX.fa	68725078	68752351	FAM155B	1.708779898	456
chrX.fa	68835911	69259321	EDA	1.839958048	680
chrX.fa	69242707	69242773	MIR676	0	0
chrX.fa	69260392	69269788	AWAT2	0	0
chrX.fa	69282341	69284029	OTUD6A	0	0
chrX.fa	69353318	69386173	IGBP1	55.85131886	6156
chrX.fa	69397336	69425553	DGAT2L6	0	0
chrX.fa	69454505	69460511	AWAT1	0	0
chrX.fa	69478016	69479654	P2RY4	0	0
chrX.fa	69488185	69501690	ARR3	0	0

chrX.fa	69502022	69504852	RAB41	1.068702817	76
chrX.fa	69506211	69509798	PDZD11	40.96342716	3389
chrX.fa	69509879	69640774	KIF4A	0	0
chrX.fa	69642881	69653241	GDPD2	0.469289456	76
chrX.fa	69664705	69725339	DLG3	19.77732758	8906
chrX.fa	69748790	70128567	TEX11	0	0
chrX.fa	70145430	70150975	SLC7A3	0	0
chrX.fa	70280788	70288231	SNX12	35.65756467	1520
chrX.fa	70315999	70323384	FOXO4	17.97999113	3800
chrX.fa	70323739	70326638	CXorf65	0	0
chrX.fa	70327254	70331481	IL2RG	0	0
chrX.fa	70338406	70362304	MED12	10.30403616	4768
chrX.fa	70364681	70391051	NLGN3	0	0
chrX.fa	70430035	70948962	BCYRN1	0	0
chrX.fa	70435062	70445065	GJB1	4.623314412	532
chrX.fa	70459474	70475047	ZMYM3	7.839814701	3420
chrX.fa	70503042	70521018	NONO	97.74910795	20886
chrX.fa	70521628	70525204	ITGB1BP2	0	0
chrX.fa	70586114	70685855	TAF1	17.55904632	8968
chrX.fa	70711377	70712604	INGX	0	0
chrX.fa	70752912	70795747	OGT	20.46183354	7448
chrX.fa	70797874	70833433	ACRC	0	0
chrX.fa	70835766	70838367	CXCR3	0	0
chrX.fa	71130938	71363424	NHSL2	1.556065037	380
chrX.fa	71264259	71264811	RPS26P11	0	0
chrX.fa	71346961	71351751	RGAG4	3.430060125	988
chrX.fa	71364034	71381600	FLJ44635	1.513292827	228
chrX.fa	71401526	71483814	PIN4	7.092807095	1216
chrX.fa	71424507	71458858	ERCC6L	0	0
chrX.fa	71492453	71497141	RPS4X	1861.784677	118180
chrX.fa	71521488	71527037	CITED1	2.123549847	228
chrX.fa	71549366	71792953	HDAC8	5.815062634	1368
chrX.fa	71798664	71934029	PHKA1	4.64454991	1900
chrX.fa	72001689	72158798	LOC100129407	0	0
chrX.fa	72223352	72225551	PABPC1L2B	3.641963291	532
chrX.fa	72297177	72299351	PABPC1L2A	0.526218664	76
chrX.fa	72345876	72347919	NAP1L6	1.680014081	228
chrX.fa	72432137	72434710	NAP1L2	5.780874988	988
chrX.fa	72667090	72674421	CDX4	0	0
chrX.fa	72744111	72782921	LOC139201	0	0
chrX.fa	72782984	72906937	CHIC1	1.491003084	684
chrX.fa	73012040	73048902	TSIX	0	0
chrX.fa	73050901	73072588	XIST	0	0

chrX.fa	73164159	73290217	NCRNA00183	0	0
chrX.fa	73247971	73513409	NCRNA00182	1.94327402	304
chrX.fa	73438212	73438296	MIR421	0	0
chrX.fa	73438382	73438383	MIR374B	0	0
chrX.fa	73506939	73507044	MIR545	0	0
chrX.fa	73507121	73507192	MIR374A	0	0
chrX.fa	73524025	73524869	ZCCHC13	0	0
chrX.fa	73641085	73753752	SLC16A2	0.785412231	228
chrX.fa	73802811	73834461	RLIM	11.30722521	7980
chrX.fa	73952691	74145287	KIAA2022	1.172320001	912
chrX.fa	74273105	74376132	ABCB7	11.02890463	1748
chrX.fa	74493894	74524732	UPRT	2.672208861	456
chrX.fa	74588262	74743337	ZDHHC15	0	0
chrX.fa	74960373	74962914	TTC3P1	2.251414657	380
chrX.fa	75002823	75005079	MAGEE2	0	0
chrX.fa	75392771	75398033	CXorf26	20.11438464	1596
chrX.fa	75648046	75651746	MAGEE1	0.927735252	228
chrX.fa	76139698	76139785	MIR384	0	0
chrX.fa	76709647	76712013	FGF16	0	0
chrX.fa	76760356	77041719	ATRX	15.94800996	11843
chrX.fa	77081862	77151065	MAGT1	1.70787626	456
chrX.fa	77154961	77160881	COX7B	133.0355785	4028
chrX.fa	77166194	77305892	ATP7A	3.640909046	2052
chrX.fa	77223458	77225135	PGAM4	0	0
chrX.fa	77359666	77382324	PGK1	96.13822219	15467
chrX.fa	77385245	77395179	TAF9B	14.47251933	2584
chrX.fa	77528130	77583087	CYSLTR1	0	0
chrX.fa	77911566	77914825	ZCCHC5	0	0
chrX.fa	78003206	78012578	LPAR4	0	0
chrX.fa	78156691	78156746	MIR4328	0	0
chrX.fa	78200829	78217438	P2RY10	0	0
chrX.fa	78426469	78427726	GPR174	0	0
chrX.fa	78615881	78623049	ITM2A	0	0
chrX.fa	79270255	79287268	TBX22	0	0
chrX.fa	79591003	79700810	FAM46D	0	0
chrX.fa	79924987	80065233	BRWD3	4.892749211	4156
chrX.fa	80369200	80457302	HMGN5	2.304126887	304
chrX.fa	80457442	80554046	SH3BGRL	39.87981431	5092
chrX.fa	82763269	82764775	POU3F4	0.759507935	76
chrX.fa	83116170	83141626	CYLC1	0	0
chrX.fa	83313354	83442943	RPS6KA6	2.929595151	1596
chrX.fa	83572882	83757487	HDX	0.361304687	152
chrX.fa	84189157	84189896	UBE2DNL	0	0

chrX.fa	84258898	84348324	APOOL	7.936503992	3263
chrX.fa	84349134	84363974	SATL1	0	0
chrX.fa	84498997	84528368	ZNF711	2.197497576	608
chrX.fa	84532395	84634748	POF1B	27.89350497	7312
chrX.fa	85116185	85302566	CHM	4.039413507	2128
chrX.fa	85403455	86087605	DACH2	0.456337308	76
chrX.fa	86772715	86925050	KLHL4	0	0
chrX.fa	88002226	88009785	CPXCR1	0	0
chrX.fa	89176940	89177882	TGIF2LX	0	0
chrX.fa	90689597	90693583	PABPC5	0.996110545	228
chrX.fa	91034260	91878228	PCDH11X	0	0
chrX.fa	92925925	92928682	NAP1L3	0	0
chrX.fa	92929012	92967273	FAM133A	0	0
chrX.fa	95592085	95592901	LOC643486	0	0
chrX.fa	95939662	96855597	DIAPH2	4.240924833	1444
chrX.fa	96138907	96140450	RPA4	0	0
chrX.fa	98716600	99194841	LOC442459	0	0
chrX.fa	99546642	99665271	PCDH19	0.117322364	76
chrX.fa	99839790	99854882	TNMD	0	0
chrX.fa	99883795	99891794	TSPAN6	16.04334379	2204
chrX.fa	99899163	99926296	SRPX2	0.522604111	76
chrX.fa	99929489	99987135	SYTL4	66.93127905	18012
chrX.fa	100075348	100095923	CSTF2	8.516489131	1140
chrX.fa	100098313	100129334	NOX1	0	0
chrX.fa	100168431	100183898	XKRX	0	0
chrX.fa	100224697	100245820	ARL13A	0	0
chrX.fa	100264334	100307105	TRMT2B	9.246177004	2204
chrX.fa	100333836	100351355	TMEM35	0	0
chrX.fa	100354798	100417978	CENPI	0	0
chrX.fa	100474933	100519485	DRP2	0	0
chrX.fa	100523241	100548059	TAF7L	0.463566413	76
chrX.fa	100600644	100603957	TIMM8A	1.901405448	228
chrX.fa	100604435	100641212	BTK	0	0
chrX.fa	100650716	100651142	RPL36A	0	0
chrX.fa	100652779	100663001	GLA	0	0
chrX.fa	100663121	100663295	HNRNPH2	48.62432149	565
chrX.fa	100673266	100788446	ARMCX4	0.634956465	228
chrX.fa	100805514	100809675	ARMCX1	18.24505835	2584
chrX.fa	100870108	100872991	ARMCX6	2.956101872	380
chrX.fa	100878120	100882831	ARMCX3	25.43575958	5852
chrX.fa	100910268	100914863	ARMCX2	13.05636761	2495
chrX.fa	101087085	101112549	NXF5	0	0
chrX.fa	101137260	101187039	ZMAT1	0.164010339	76

chrX.fa	101380660	101382684	TCEAL2	28.06941321	2052
chrX.fa	101394933	101397388	TCEAL6	0	0
chrX.fa	101408679	101410986	BEX5	23.51658258	1588
chrX.fa	101485190	101711835	NXF2B	0	0
chrX.fa	101502170	101694853	NXF2	0	0
chrX.fa	101768610	101771699	TMSB15A	32.65402179	1444
chrX.fa	101804893	101826621	NXF4	0	0
chrX.fa	101854096	101859085	ARMCX5	5.267608472	965
chrX.fa	101854776	101972661	GPRASP2	7.981234085	2204
chrX.fa	101906294	101914010	GPRASP1	3.233820022	1292
chrX.fa	101975642	102007369	BHLHB9	5.614756159	1596
chrX.fa	102192200	102193228	RAB40AL	0	0
chrX.fa	102317581	102319168	BEX1	1.327896383	76
chrX.fa	102330750	102348022	NXF3	0	0
chrX.fa	102470020	102472095	BEX4	48.5702538	4028
chrX.fa	102507923	102510121	TCEAL8	47.21976646	3800
chrX.fa	102528618	102531797	TCEAL5	0	0
chrX.fa	102564274	102565974	BEX2	14.59963111	1140
chrX.fa	102585114	102587251	TCEAL7	0	0
chrX.fa	102611380	102613397	WBP5	58.85953054	4256
chrX.fa	102631268	102633001	NGFRAP1	113.9096235	9424
chrX.fa	102754681	102774417	RAB40A	0.850323577	76
chrX.fa	102840419	102842655	TCEAL4	73.87438341	6308
chrX.fa	102862834	102864855	TCEAL3	29.50544497	2204
chrX.fa	102883648	102885876	TCEAL1	21.66457602	1824
chrX.fa	102930426	102943086	MORF4L2	82.99073772	12145
chrX.fa	102962272	102983552	GLRA4	0	0
chrX.fa	102965837	102968960	TMEM31	0	0
chrX.fa	103031439	103047547	PLP1	0.748814882	152
chrX.fa	103077255	103087212	RAB9B	0.602576095	152
chrX.fa	103139055	103139097	MIR1256	0	0
chrX.fa	103217200	103220563	TMSB15B	3.437289231	152
chrX.fa	103230502	103317502	H2BFXP	0	0
chrX.fa	103265719	103268256	H2BFWT	0	0
chrX.fa	103294516	103297021	H2BFM	0	0
chrX.fa	103343898	103401708	MCART6	0	0
chrX.fa	103357107	103360533	ZCCHC18	0	0
chrX.fa	103411301	103436127	FAM199X	17.16340338	3420
chrX.fa	103494719	103499599	ESX1	0	0
chrX.fa	103810996	105011822	IL1RAPL2	0	0
chrX.fa	104463611	104465358	TEX13A	0	0
chrX.fa	105066536	105202602	NRK	0.42591482	228
chrX.fa	105277190	105282718	SERPINA7	1.434375088	152

chrX.fa	105412298	105452949	MUM1L1	1.593867236	456
chrX.fa	105855160	105922673	CXorf57	0.303321233	76
chrX.fa	105937068	106040246	RNF128	2.349007586	532
chrX.fa	106045919	106119377	TBC1D8B	4.361108718	1824
chrX.fa	106143293	106146561	RIPPLY1	0	0
chrX.fa	106161590	106174091	CLDN2	4.804644484	988
chrX.fa	106183964	106243474	MORC4	10.15041766	2584
chrX.fa	106305118	106362057	RBM41	3.044658419	1292
chrX.fa	106366657	106449670	NUP62CL	0	0
chrX.fa	106449862	106487473	CXorf41	0	0
chrX.fa	106871654	106894256	PRPS1	23.89023699	3420
chrX.fa	106956452	107019017	TSC22D3	90.79305144	16922
chrX.fa	107069084	107174867	MID2	13.25245711	6384
chrX.fa	107224094	107225600	TEX13B	0	0
chrX.fa	107288200	107322414	VSIG1	0	0
chrX.fa	107327437	107334848	PSMD10	18.50922192	1900
chrX.fa	107334899	107397901	ATG4A	5.935698338	912
chrX.fa	107398837	107682704	COL4A6	0.167926105	76
chrX.fa	107683154	107940775	COL4A5	0.845353567	361
chrX.fa	107975727	107979607	IRS4	0	0
chrX.fa	108616135	108725285	GUCY2F	0	0
chrX.fa	108779010	108787913	NXT2	2.976283126	532
chrX.fa	108866929	108868393	KCNE1L	0	0
chrX.fa	108884564	108976621	ACSL4	35.54415807	12591
chrX.fa	109245863	109421016	TMEM164	12.36674103	4687
chrX.fa	109437414	109683461	AMMECR1	3.294062571	1292
chrX.fa	109468217	109468288	SNORD96B	0	0
chrX.fa	109662285	109699562	RGAG1	0	0
chrX.fa	109763540	109766249	TDGF3	0	0
chrX.fa	109917084	110039286	CHRDL1	0.275609661	76
chrX.fa	110187513	110464173	PAK3	0.349105571	76
chrX.fa	110488327	110513774	CAPN6	0	0
chrX.fa	110537007	110655460	DCX	0	0
chrX.fa	110754890	110765627	DKFZp686D0853	0	0
chrX.fa	110924346	111003875	ALG13	2.590730813	1205
chrX.fa	111017542	111326004	TRPC5	0.19593889	76
chrX.fa	111119279	111147213	LOC100329135	0	0
chrX.fa	111326253	111700473	ZCCHC16	0	0
chrX.fa	111873879	111923375	LHFPL1	0.648511038	76
chrX.fa	112018105	112084043	AMOT	17.04668344	8360
chrX.fa	112023946	112024016	MIR4329	0	0
chrX.fa	113818551	114144624	HTR2C	0	0
chrX.fa	113865259	113865386	SNORA35	0	0

chrX.fa	113873918	113874002	MIR764		0	0
chrX.fa	113886019	113886098	MIR1912		0	0
chrX.fa	113887130	113887198	MIR1264		0	0
chrX.fa	113949650	113949761	MIR1298		0	0
chrX.fa	113997744	113997823	MIR1911		0	0
chrX.fa	114058017	114058127	MIR448		0	0
chrX.fa	114238538	114252207	IL13RA2		0	0
chrX.fa	114345183	114468635	LRCH2		1.3944644	456
chrX.fa	114423963	114427431	RBMXL3		0	0
chrX.fa	114524292	114542121	LUZP4		0	0
chrX.fa	114795177	114885179	PLS3		20.5270461	5092
chrX.fa	115301958	115306225	AGTR2		0	0
chrX.fa	115567747	115592625	SLC6A14		0	0
chrX.fa	115592852	115594137	CXorf61		0	0
chrX.fa	117031776	117251303	KLHL13		3.120714637	988
chrX.fa	117480036	117583923	WDR44		21.6900285	5971
chrX.fa	117520357	117520434	MIR1277		0	0
chrX.fa	117629872	117820123	DOCK11		2.241625243	988
chrX.fa	117861559	117928496	IL13RA1		2.571453198	684
chrX.fa	117957787	117960931	ZCCHC12		0	0
chrX.fa	118108713	118151949	LONRF3		0.87803515	152
chrX.fa	118212598	118284542	KIAA1210		0	0
chrX.fa	118370211	118378429	PGRMC1		7.168712707	912
chrX.fa	118533258	118588437	SLC25A43		15.29739043	2584
chrX.fa	118599996	118603083	LOC100303728		1.60034331	304
chrX.fa	118603624	118605359	SLC25A5		177.6994042	13262
chrX.fa	118672112	118699397	CXorf56		7.997650179	1292
chrX.fa	118708499	118718379	UBE2A		17.21716986	3393
chrX.fa	118722300	118739846	NKRF		6.140974824	1368
chrX.fa	118749688	118827333		6-Sep	5.179353137	1530
chrX.fa	118780701	118780811	MIR766		0	0
chrX.fa	118892576	118894165	ANKRD58		0	0
chrX.fa	118920469	118925606	RPL39		202.6613562	5396
chrX.fa	118921316	118921447	SNORA69		0	0
chrX.fa	118967989	118986991	UPF3B		8.094188864	1292
chrX.fa	119004495	119005733	RNF113A		12.00965333	988
chrX.fa	119005792	119010629	NDUFA1		157.7848742	4484
chrX.fa	119029936	119054679	AKAP14		0	0
chrX.fa	119059013	119077735	NKAP		10.83221271	1140
chrX.fa	119206229	119297945	RHOXF2B		0	0
chrX.fa	119243025	119249847	RHOXF1		0	0
chrX.fa	119370309	119379122	NKAPP1		0	0
chrX.fa	119384607	119392251	ZBTB33		8.620708741	3040

chrX.fa	119392505	119445391	FAM70A	0	0
chrX.fa	119495940	119517104	ATP1B4	0	0
chrX.fa	119560003	119603204	LAMP2	3.834137022	1668
chrX.fa	119658446	119709684	CUL4B	14.32853964	5089
chrX.fa	119737744	119755016	MCTS1	1.148976013	760
chrX.fa	119759529	119764005	C1GALT1C1	0.601220638	76
chrX.fa	120006452	120009779	CT47B1	0	0
chrX.fa	120012623	120012778	CT47A1	0	0
chrX.fa	120087137	120090454	CT47A7	0	0
chrX.fa	120094341	120095337	CT47A6	0	0
chrX.fa	120181462	120183796	GLUD2	0	0
chrX.fa	122318096	122624766	GRIA3	0	0
chrX.fa	122734412	122866904	THOC2	14.67403066	5466
chrX.fa	122994048	123047822	XIAP	13.29191598	7425
chrX.fa	123094475	123236505	STAG2	26.7431735	11400
chrX.fa	123480132	123507010	SH2D1A	0	0
chrX.fa	123509756	124097666	ODZ1	0.621401891	532
chrX.fa	124453969	124456950	LOC100129520	0	0
chrX.fa	125297482	125300080	DCAF12L2	0	0
chrX.fa	125683366	125686842	DCAF12L1	0	0
chrX.fa	125953747	125955768	CXorf64	0	0
chrX.fa	127184941	127186382	ACTRT1	0	0
chrX.fa	128580478	128657460	SMARCA1	20.16935596	5472
chrX.fa	128674252	128726530	OCRL	20.99844404	7186
chrX.fa	128779236	128788933	APLN	0.710109045	152
chrX.fa	128872946	128903525	XPNPEP2	3.090743969	684
chrX.fa	128913892	128929176	SASH3	0	0
chrX.fa	128937264	128977910	ZDHHC9	3.632324483	1114
chrX.fa	129040097	129063738	UTP14A	13.86045503	2356
chrX.fa	129139164	129192058	BCORL1	3.53322549	1672
chrX.fa	129198895	129244688	ELF4	10.44937131	2964
chrX.fa	129263338	129299861	AIFM1	20.6830743	3572
chrX.fa	129305773	129318844	RAB33A	0	0
chrX.fa	129336673	129402922	ZNF280C	3.900705038	1216
chrX.fa	129474047	129507335	SLC25A14	3.58804621	380
chrX.fa	129518319	129519511	GPR119	0	0
chrX.fa	129535943	129547317	RBMX2	8.989091927	912
chrX.fa	129628915	129631421	FAM45B	0	0
chrX.fa	129757357	130037208	ENOX2	7.116151083	1976
chrX.fa	130192216	130223857	ARHGAP36	0	0
chrX.fa	130407483	130423403	IGSF1	0	0
chrX.fa	130678048	130678974	OR13H1	0	0
chrX.fa	130836678	130964671	LOC286467	0	0

chrX.fa	131157245	131209971	MST4	2.058638501	456
chrX.fa	131211021	131262050	FRMD7	0.357539527	76
chrX.fa	131337053	131352189	RAP2C	4.656447813	1216
chrX.fa	131503343	131623996	MBNL3	2.60955661	2052
chrX.fa	131760038	132095423	HS6ST2	0	0
chrX.fa	132159507	132162300	USP26	0	0
chrX.fa	132350697	132352376	TFDP3	0	0
chrX.fa	132435064	132549205	GPC4	0.308140637	76
chrX.fa	132669776	133119673	GPC3	0	0
chrX.fa	133303408	133303482	MIR363	0	0
chrX.fa	133303568	133303642	MIR92A2	0	0
chrX.fa	133303701	133303796	MIR19B2	0	0
chrX.fa	133303839	133303907	MIR20B	0	0
chrX.fa	133304071	133304141	MIR18B	0	0
chrX.fa	133304228	133304308	MIR106A	0	0
chrX.fa	133371077	133379808	CCDC160	4.405688204	380
chrX.fa	133507342	133562822	PHF6	7.742522985	2280
chrX.fa	133594175	133634698	HPRT1	9.643024795	906
chrX.fa	133674215	133674292	MIR450B	0	0
chrX.fa	133674371	133674461	MIR450A1	0	0
chrX.fa	133674538	133674637	MIR450A2	0	0
chrX.fa	133675371	133675467	MIR542	0	0
chrX.fa	133677407	133680643	MGC16121	1.488442776	76
chrX.fa	133680358	133680428	MIR503	0	0
chrX.fa	133680661	133680741	MIR424	0	0
chrX.fa	133699873	133792513	PLAC1	0	0
chrX.fa	133903596	133931262	FAM122B	6.522611371	1576
chrX.fa	133930443	133988641	FAM122C	0.637366167	228
chrX.fa	134021662	134049297	MOSPD1	7.281215667	1140
chrX.fa	134124968	134126503	LOC644538	0.745200329	76
chrX.fa	134154534	134156566	FAM127C	6.756201854	912
chrX.fa	134166333	134167575	FAM127A	46.04217524	3800
chrX.fa	134184963	134186221	FAM127B	25.45594084	2128
chrX.fa	134229015	134232733	NCRNA00087	5.299085203	988
chrX.fa	134252882	134254405	LOC100129515	0	0
chrX.fa	134290461	134305751	CXorf48	0	0
chrX.fa	134419723	134429965	ZNF75D	10.50419203	2280
chrX.fa	134478696	134497338	ZNF449	1.409976856	380
chrX.fa	134555868	134560225	NCRNA00086	2.318434493	532
chrX.fa	134654555	134716460	DDX26B	2.37958068	608
chrX.fa	134847185	134856988	CT45A1	0	0
chrX.fa	134873998	134874268	CT45A2	0	0
chrX.fa	134883488	134890044	CT45A3	0	0

chrX.fa	134928697	134936735	CT45A4	0	0
chrX.fa	134945651	134953994	CT45A5	0	0
chrX.fa	134963215	134971244	CT45A6	0	0
chrX.fa	134975785	134995220	SAGE1	0	0
chrX.fa	135044231	135056134	MMGT1	2.823869477	684
chrX.fa	135067583	135129428	SLC9A6	0.48404888	152
chrX.fa	135228861	135293518	FHL1	0	0
chrX.fa	135295379	135338641	MAP7D3	0.525315026	228
chrX.fa	135383122	135499047	GPR112	0	0
chrX.fa	135570125	135574598	BRS3	0	0
chrX.fa	135579238	135594503	HTATSF1	25.93908608	5396
chrX.fa	135614311	135638966	VGLL1	51.59036338	4162
chrX.fa	135633037	135633119	MIR934	0	0
chrX.fa	135730336	135742549	CD40LG	0	0
chrX.fa	135747712	135863503	ARHGEF6	0.868998767	304
chrX.fa	135951353	135962939	RBMX	18.60877273	4180
chrX.fa	135961358	135961430	SNORD61	0	0
chrX.fa	136112307	136113833	GPR101	0	0
chrX.fa	136648346	136654259	ZIC3	0	0
chrX.fa	137696892	137699799	LOC158696	0	0
chrX.fa	137713734	138287185	FGF13	4.310504977	988
chrX.fa	137749872	137749954	MIR504	0	0
chrX.fa	138528978	138531132	SRD5A1P1	0	0
chrX.fa	138612895	138645617	F9	0	0
chrX.fa	138663930	138790381	MCF2	0	0
chrX.fa	138808505	138914447	ATP11C	1.481966702	602
chrX.fa	139006307	139006390	MIR505	0	0
chrX.fa	139037884	139047677	CXorf66	0	0
chrX.fa	139585152	139587225	SOX3	0	0
chrX.fa	139791924	139796996	RP1-177G6-2	0	0
chrX.fa	139865425	139866723	CDR1	85.47107407	7372
chrX.fa	140008337	140008384	MIR320D2	0	0
chrX.fa	140269931	140271310	LDOC1	25.71227288	2356
chrX.fa	140335596	140336646	SPANXC	0	0
chrX.fa	140590843	140738069	CXorf18	0	0
chrX.fa	140671796	140678899	SPANXA2	0	0
chrX.fa	140786599	140786655	SPANXE	0	0
chrX.fa	140926102	140985618	MAGEC3	0	0
chrX.fa	140991642	140997187	MAGEC1	0	0
chrX.fa	141290128	141293076	MAGEC2	0	0
chrX.fa	142113704	142122066	SPANXN4	0	0
chrX.fa	142596564	142605307	SPANXN3	0	0
chrX.fa	142710595	142723926	SLITRK4	0.371094101	228

chrX.fa	142795055	142804516	SPANXN2	0	0
chrX.fa	142967173	142968357	UBE2NL	0	0
chrX.fa	144329107	144337728	SPANXN1	0	0
chrX.fa	144899347	144907360	SLITRK2	0	0
chrX.fa	144908928	144911370	CXorf1	0	0
chrX.fa	145075793	145075869	MIR890	0	0
chrX.fa	145076302	145076378	MIR888	0	0
chrX.fa	145078187	145078261	MIR892A	0	0
chrX.fa	145078716	145078792	MIR892B	0	0
chrX.fa	145082571	145082649	MIR891B	0	0
chrX.fa	145109312	145109390	MIR891A	0	0
chrX.fa	145891302	145896249	CXorf51	0	0
chrX.fa	146312238	146312361	MIR506	0	0
chrX.fa	146312502	146312595	MIR507	0	0
chrX.fa	146318431	146318545	MIR508	0	0
chrX.fa	146331669	146331748	MIR514B	0	0
chrX.fa	146341170	146342133	MIR509-3	0	0
chrX.fa	146342050	146342052	MIR509-1	0	0
chrX.fa	146353853	146353926	MIR510	0	0
chrX.fa	146360765	146360862	MIR514-1	0	0
chrX.fa	146990949	147003676	FMR1-AS1	0	0
chrX.fa	147007058	147032647	FMR1	19.66602947	5325
chrX.fa	147062849	147108187	FMR1NB	0	0
chrX.fa	147582139	148082193	AFF2	0	0
chrX.fa	148560295	148586884	IDS	30.72520598	12312
chrX.fa	148609134	148621312	LOC100131434	0	0
chrX.fa	148622519	148632086	CXorf40A	2.68094403	591
chrX.fa	148674172	148674182	HSFX2	0	0
chrX.fa	148678216	148713487	TMEM185A	2.09478403	380
chrX.fa	148769903	148798928	MAGEA11	0	0
chrX.fa	148863593	148863599	MAGEA9B	0	0
chrX.fa	149009941	149014609	MAGEA8	0	0
chrX.fa	149100415	149106716	CXorf40B	18.71344416	1665
chrX.fa	149106766	149185018	LOC100272228	0	0
chrX.fa	149396239	149396318	MIR2114	0	0
chrX.fa	149531551	149682448	MAMLD1	0.235849579	76
chrX.fa	149737047	149841616	MTM1	8.99436315	2052
chrX.fa	149861869	149933575	MTMR1	18.01538363	3268
chrX.fa	149934809	150067289	CD99L2	4.621507135	1140
chrX.fa	150151763	150159248	HMGB3	10.94396264	2584
chrX.fa	150336694	150336798	MIR4330	0	0
chrX.fa	150345056	150349937	GPR50	0	0
chrX.fa	150565657	150577836	VMA21	10.3922915	3268

chrX.fa	150732007	150845211	PASD1	0	0
chrX.fa	150863730	150870063	PRRG3	0	0
chrX.fa	150884508	150891664	FATE1	1.070660699	76
chrX.fa	150903218	150914036	CNGA2	0	0
chrX.fa	151081361	151093642	MAGEA4	0	0
chrX.fa	151121596	151143151	GABRE	0	0
chrX.fa	151128100	151128184	MIR452	0	0
chrX.fa	151282527	151286444	MAGEA5	0	0
chrX.fa	151302907	151307025	MAGEA10	0	0
chrX.fa	151335634	151619831	GABRA3	0	0
chrX.fa	151560691	151560771	MIR105-1	0	0
chrX.fa	151561893	151562001	MIR767	0	0
chrX.fa	151562884	151562964	MIR105-2	0	0
chrX.fa	151806637	151821825	GABRQ	0	0
chrX.fa	151867245	151870814	MAGEA6	0	0
chrX.fa	151883119	151922364	MAGEA2	0	0
chrX.fa	151885384	151885450	MAGEA2B	0	0
chrX.fa	151899293	151903184	MAGEA12	0	0
chrX.fa	151903228	151909518	CSAG1	0	0
chrX.fa	151928150	151928200	CSAG3	0	0
chrX.fa	151934652	151938240	MAGEA3	0	0
chrX.fa	151995871	151999301	CETN2	41.33301519	2964
chrX.fa	151999511	152037907	NSDHL	10.53325906	1140
chrX.fa	152082986	152142025	ZNF185	16.74456706	5320
chrX.fa	152157368	152162671	PNMA5	0	0
chrX.fa	152224766	152228827	PNMA3	0	0
chrX.fa	152240819	152240838	LOC100287428	0	0
chrX.fa	152244152	152245958	PNMA6A	0	0
chrX.fa	152481522	152486116	MAGEA1	0	0
chrX.fa	152599613	152618384	ZNF275	7.244317106	3040
chrX.fa	152683781	152687086	ZFP92	0	0
chrX.fa	152710178	152711945	TREX2	0	0
chrX.fa	152713123	152736603	HAUS7	2.437865346	304
chrX.fa	152760347	152775004	BGN	0.468385817	76
chrX.fa	152801580	152848387	ATP2B3	0	0
chrX.fa	152853383	152864632	FAM58A	9.320877765	760
chrX.fa	152907897	152916781	DUSP9	5.290199427	836
chrX.fa	152935188	152939816	PNCK	0	0
chrX.fa	152953752	152962048	SLC6A8	11.90347583	2888
chrX.fa	152965947	152990201	BCAP31	17.19774163	2736
chrX.fa	152990323	153010216	ABCD1	0.937223454	228
chrX.fa	153029651	153044801	PLXNB3	0.53841778	228
chrX.fa	153046456	153051187	SRPK3	1.715255972	228

chrX.fa	153051221	153059967	IDH3G	18.50033615	2103
chrX.fa	153060094	153063954	SSR4	77.26558709	3268
chrX.fa	153067623	153096003	PDZD4	0.305580329	76
chrX.fa	153126971	153141399	L1CAM	2.048397267	684
chrX.fa	153167985	153172620	AVPR2	0	0
chrX.fa	153172830	153191714	ARHGAP4	1.018400288	228
chrX.fa	153195377	153200468	NAA10	32.1532556	1883
chrX.fa	153200722	153210232	RENBP	7.003045698	684
chrX.fa	153213008	153236819	HCFC1	16.0293374	8968
chrX.fa	153237991	153248646	TMEM187	0	0
chrX.fa	153246548	153246628	MIR3202-1	0	0
chrX.fa	153275957	153285342	IRAK1	38.14287102	9044
chrX.fa	153285371	153285440	MIR718	0	0
chrX.fa	153287264	153363188	MECP2	10.8413997	7372
chrX.fa	153409725	153424507	OPN1LW	0	0
chrX.fa	153461638	153462352	OPN1MW	0	0
chrX.fa	153500056	153523438	TEX28	0	0
chrX.fa	153524027	153558713	TKTL1	0	0
chrX.fa	153576900	153603006	FLNA	0.403474471	228
chrX.fa	153607597	153609883	EMD	43.17975053	3839
chrX.fa	153626571	153629578	RPL10	18.47895004	1492
chrX.fa	153628622	153628756	SNORA70	0	0
chrX.fa	153630681	153640422	DNASE1L1	3.758984442	608
chrX.fa	153639877	153650063	TAZ	3.002338028	366
chrX.fa	153656978	153664862	ATP6AP1	6.974279881	972
chrX.fa	153665259	153671814	GDI1	48.80595277	8040
chrX.fa	153672485	153679002	FAM50A	22.20886745	1976
chrX.fa	153686623	153701985	PLXNA3	1.187681851	532
chrX.fa	153705241	153707596	LAGE3	4.412013672	532
chrX.fa	153712056	153714932	UBL4A	16.88357674	2584
chrX.fa	153715650	153719002	SLC10A3	2.150508388	304
chrX.fa	153733327	153744566	FAM3A	6.849728411	1399
chrX.fa	153759606	153775233	G6PD	11.47003069	1824
chrX.fa	153770459	153793261	IKBKG	2.196142118	380
chrX.fa	153880246	153881853	CTAG2	0	0
chrX.fa	153903527	153979348	GAB3	0.241572621	76
chrX.fa	153991031	154005964	DKC1	18.21072009	3116
chrX.fa	153996803	153996932	SNORA36A	0	0
chrX.fa	154003273	154003401	SNORA56	0	0
chrX.fa	154006959	154033802	MPP1	13.02474027	1900
chrX.fa	154051623	154062937	LOC100132963	0	0
chrX.fa	154064064	154250998	F8	0.746103968	456
chrX.fa	154114635	154688276	F8A1	3.255356734	380

chrX.fa	154255064	154285191	FUNDC2	22.01172371	4332
chrX.fa	154289900	154292308	MTCP1NB	27.24770485	787
chrX.fa	154292309	154294332	MTCP1	0.657396814	76
chrX.fa	154299710	154351349	BRCC3	13.35502005	2584
chrX.fa	154444701	154468098	VBP1	21.79530236	2356
chrX.fa	154487526	154493852	RAB39B	1.314492416	304
chrX.fa	154505500	154563986	CLIC2	0.426065427	76
chrX.fa	154718673	154842622	TMLHE	4.993504874	1368
chrX.fa	154997451	155012117	SPRY3	0	0
chrX.fa	155110943	155173433	VAMP7	0	0
chrX.fa	155227246	155240482	IL9R	0	0
chrY.fa	142991	170022	PLCXD1	0	0
chrY.fa	171426	180887	GTPBP6	0	0
chrY.fa	231385	232054	NCRNA00107	0	0
chrY.fa	244668	297690	PPP2R3B	0	0
chrY.fa	535079	570146	SHOX	0	0
chrY.fa	1264887	1281527	CRLF2	0	0
chrY.fa	1337693	1378828	CSF2RA	0	0
chrY.fa	1362811	1362885	MIR3690	0	0
chrY.fa	1405509	1451582	IL3RA	0	0
chrY.fa	1455045	1461039	SLC25A6	0	0
chrY.fa	1472032	1522655	ASMTL	0	0
chrY.fa	1482655	1484314	ASMTL-AS	0	0
chrY.fa	1531466	1606037	P2RY8	0	0
chrY.fa	1660486	1671411	AKAP17A	0	0
chrY.fa	1683941	1711974	ASMT	0	0
chrY.fa	2087555	2293345	DHRSX	0	0
chrY.fa	2354455	2368580	ZBED1	0	0
chrY.fa	2477306	2525270	CD99P1	0	0
chrY.fa	2559228	2609350	CD99	0	0
chrY.fa	2620337	2643037	XGPY2	0	0
chrY.fa	2654896	2655792	SRY	0	0
chrY.fa	2709623	2734997	RPS4Y1	352.1872782	20976
chrY.fa	2803112	2850547	ZFY	9.579167693	3572
chrY.fa	3447126	3448082	TGIF2LY	0	0
chrY.fa	4868267	5610264	PCDH11Y	0.107081131	76
chrY.fa	6114264	6117053	TSPY2	0	0
chrY.fa	6258442	9611928	TTTY1B	0	0
chrY.fa	6325200	9545180	TTTY7	0	0
chrY.fa	6733959	6742068	AMELY	0	0
chrY.fa	6778727	6959724	TBL1Y	1.90215848	304
chrY.fa	7142013	7249588	PRKY	2.061801234	988
chrY.fa	7567398	7569288	TTTY16	0	0

chrY.fa	7672965	7678723	TTTY12	0	0
chrY.fa	8551411	8551919	TTTY18	0	0
chrY.fa	8572513	8573324	TTTY19	0	0
chrY.fa	8651359	8685423	TTTY11	0	0
chrY.fa	9154670	9160483	RBMY1A3P	0	0
chrY.fa	9167489	9172441	TTTY20	0	0
chrY.fa	9187189	9362877	FAM197Y2P	0	0
chrY.fa	9195452	9236312	TSPY4	0	0
chrY.fa	9236030	9365753	TSPY3	0	0
chrY.fa	9304564	9307358	TSPY1	0	0
chrY.fa	9448330	9452762	RBMY3AP	0	0
chrY.fa	9638762	9650854	TTTY22	0	0
chrY.fa	14517915	14533389	GYG2P1	2.778235747	152
chrY.fa	14774298	14804153	TTTY15	0	0
chrY.fa	14813160	14972768	USP9Y	8.892402636	5928
chrY.fa	15016019	15032390	DDX3Y	15.28986011	4868
chrY.fa	15360263	15592550	UTY	10.70796245	5016
chrY.fa	15815447	15817902	TMSB4Y	4.11983731	456
chrY.fa	16168098	16168101	VCY	0	0
chrY.fa	16634488	16955848	NLGN4Y	0.911921583	380
chrY.fa	19687041	20492736	NCRNA00230B	0	0
chrY.fa	19691333	20488439	NCRNA00230A	0	0
chrY.fa	19990140	19992099	CDY2B	0	0
chrY.fa	20137667	20139626	CDY2A	0	0
chrY.fa	20708557	20935621	HSFY2	0	0
chrY.fa	20933700	20934513	HSFY1	0	0
chrY.fa	21034387	21040114	NCRNA00185	0	0
chrY.fa	21094585	21239302	TTTY14	0	0
chrY.fa	21152526	21154705	CD24	114.4608428	16568
chrY.fa	21617317	21665039	BCORP1	0.624112806	152
chrY.fa	21729235	21752309	CYorf15A	14.55565405	836
chrY.fa	21758442	21767698	CYorf15B	0	0
chrY.fa	21867301	21906825	KDM5D	12.10031836	4484
chrY.fa	22627554	22681114	TTTY10	0	0
chrY.fa	22737611	22755040	EIF1AY	14.82222733	1368
chrY.fa	22917954	22942918	RPS4Y2	0	0
chrY.fa	23557034	23563448	RBMY2EP	0	0
chrY.fa	23673224	24064214	RBMY1B	0	0
chrY.fa	23696805	23696912	RBMY1D	0	0
chrY.fa	23745486	23756552	TTTY13	0	0
chrY.fa	24291112	24587606	TTTY6	0	0
chrY.fa	24442945	24445023	TTTY5	0	0
chrY.fa	24455006	24462352	RBMY2FP	0	0

chrY.fa	24549623	24549729	RBMY1J	0	0
chrY.fa	24585737	24585739	TTTY6B	0	0
chrY.fa	25275502	25345239	DAZ1	0	0
chrY.fa	25365622	27037136	DAZ2	0	0
chrY.fa	25382975	26980276	DAZ3	0	0
chrY.fa	26986878	26988731	DAZ4	0	0
chrY.fa	59100457	59115123	SPRY3	0	0
chrY.fa	59213949	59276439	VAMP7	0	0
chrY.fa	59330252	59343488	IL9R	0	0
